# No evidence for kin protection in the expression of sickness behaviors in house mice

**DOI:** 10.1038/s41598-018-35174-0

**Published:** 2018-11-12

**Authors:** Patricia C. Lopes, Per Block, Alice Pontiggia, Anna K. Lindholm, Barbara König

**Affiliations:** 1 Schmid College of Science and Technology, Chapman University, One University Drive, Orange, 92866 CA USA; 2 Chair of Social Networks, Department of Humanities, Social and Political Science, ETH Zurich, Clausiusstrasse 50, 8092 Zürich, Switzerland; 3 Agroscope, Vetsuisse Faculty, University of Bern, Bern, Switzerland; 4 Department of Evolutionary Biology and Environmental Studies, University of Zurich, Winterthurerstrasse 190, 8057 Zürich, Switzerland

## Abstract

When infected, animals change their behaviors in several ways, including by decreasing their activity, their food and water intake, and their interest in social interactions. These behavioral alterations are collectively called sickness behaviors and, for several decades, the main hypotheses put forward to explain this phenomenon were that engaging in sickness behaviors facilitated the fever response and improved the likelihood of host survival. However, a new hypothesis was recently proposed suggesting that engaging in sickness behaviors may serve to protect kin. We tested this kin protection hypothesis by combining a field and a laboratory experiment in house mice. In both experiments, we induced sickness behaviors by administration of a pro-inflammatory agent. In the field experiment, we then collected genetic data and assessed whether relatedness affected the intensity of sickness behaviors. In the lab experiment, we manipulated relatedness in small social groups and assessed whether having a closely related individual (a sibling) in the group altered social interactions or visits to common resources (such as food and water containers) once immune-challenged. Our results do not support the kinship protection hypothesis and therefore advance our understanding of why such an apparently costly set of behavioral changes would be evolutionarily maintained.

## Introduction

Understanding why animals show behavioral symptoms of sickness and the circumstances that ameliorate those symptoms is of widespread interest from an evolutionary, epidemiological, and clinical perspective. Most animals that have been tested experience extensive changes in behaviors when sick, the most obvious of those being reductions in activity, drinking and eating. Other symptoms associated with being sick include pain, fatigue and a decreased interest in social and sexual interactions^1^. The physiological pathways that are activated upon infection by different types of pathogens are relatively well understood. Once detecting an invasion by a pathogen, the host’s body responds by triggering fever and an inflammatory response, which crosstalks to the neuroendocrine system, eliciting changes in behavior^2–4^. So, discounting instances where pathogens directly alter host behavior, it is the host itself that induces the sickness behaviors. Why would such a system have evolved?

The prevailing view is that alteration of behavior when infected (or engaging in sickness behaviors) primarily serves the purpose of self-preservation^1,5,6^. Initially proposed by Hart in 1988^1^, the idea here is that most of the alterations in behavior observed during infection lead to increased host survival, either by reallocating energy into pathogen-combating mechanisms or by reducing pathogen access to essential nutrients. This idea is supported by research focusing on the study of fever, a primary symptom of infection that frequently coincides with and is facilitated by sickness behaviors^7^. Fever has been shown to increase survival in ectotherms^8–11^ (behavioral fever, in this case) and in mammals^12^. However, Shakhar and Shakhar^13^ recently proposed the novel hypothesis that kinship may explain why we alter our behaviors when infected. In other words, sickness behaviors would be an evolutionary mechanism to protect kin from infection. The authors argue that the costs associated with sickness behaviors (for example, reduced caloric intake due to anorexia, at a time when calories are needed to maintain the fever response) cannot be fully explained by a host survival focused hypothesis. What these authors are proposing is that, because in many species relatedness within social groups is higher than relatedness within the whole population, by reducing direct and indirect contacts and, therefore, disease transmission, the expression of sickness behaviors could be selected for via kin selection^14,15^. In populations with high viscosity, by displaying sickness behaviors, an infected animal is ultimately protecting its relatives from contracting the infection, thus selecting for the maintenance of those behaviors in the population. Specifically, their hypothesis predicts that “*Higher relatedness within social groups should promote SB* [sickness behaviors]” (p. 10)^13^. If true, this would fundamentally change our views regarding the causes of sickness behaviors.

Shakhar and Shakhar’s idea is particularly relevant given the array of studies that have demonstrated that the expression of sickness behaviors is plastic (reviewed in^16^). For instance, in the presence of potential mates, animals can forgo expressing sickness behaviors in order to attempt to mate^17,18^. The sickness behavior response is therefore not an “all or nothing” response. This plasticity could make it possible for animals to modulate the expression of sickness behaviors in accordance to relatedness to their social partners/groups. According to Shakhar and Shakhar’s kin protection hypothesis, the more related animals are to their social group, the strongest their sickness behavior symptoms should be.

We tested this innovative kin protection hypothesis by combining data from a field and a laboratory experiment in an animal species known to live in social groups, to distinguish kin, and to display sickness behaviors^19–22^: the house mouse (*Mus musculus domesticus*). In both experiments, we experimentally induced sickness behaviors by administering an immune challenge to a set of animals. In the field experiment, we calculated genetic relatedness for the entire population of wild mice and used remotely tracked changes in social interactions to test whether an association existed between natural variation in relatedness and changes in social contacts following the immune challenge. In the lab experiment, we manipulated genetic relatedness in small groups of mice (derived from the same population as the field experiment) to test whether changes in social contact patterns were greater when a sibling versus no closely related animal was present in the group. In neither experiment did we find evidence for an effect of kinship on the extent to which animals alter their social interactions when sick.

## Results

In this study, we used data from a field and a laboratory experiment to test whether kinship promotes the expression of sickness behaviors in mice.

### Field experiment

**Figure 1:**
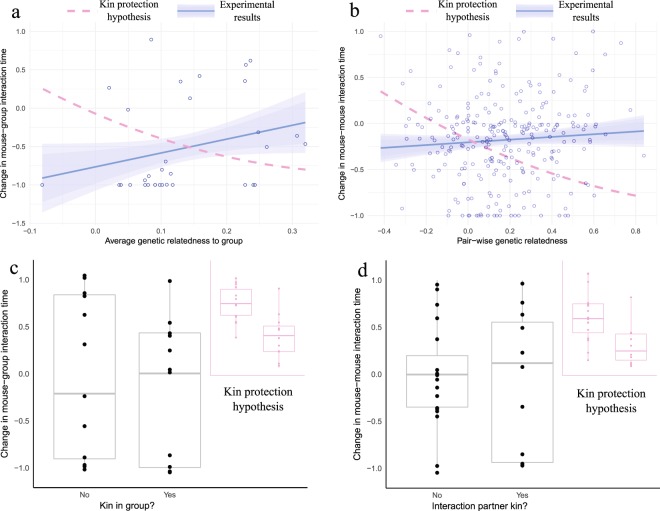
Relative change in interaction time of immune-challenged mice as a function of genetic relatedness. Results from the field experiment demonstrating (**a**) change in time socializing with other mice of the same group after receiving an immune challenge dependent on relatedness to the group and (**b**) change in pairwise interactions with other mice following an immune challenge depending on genetic relatedness to the interaction partner. (**b**) differs from (**a**) as it does not focus on average relatedness of an individual mouse to the social group, but on pairwise relatedness and pairwise interactions. Our findings do not support the prediction under the kin protection hypothesis (dotted line). Results from the laboratory experiment demonstrating changes in interaction time between (**c**) immune-challenged mice and other mice in the enclosure, depending on the presence or absence of kin in the group, or (**d**) immune-challenged mice that had kin in the group and an interaction partner that specifically was or was not a sibling. Our findings do not support the prediction under the kin protection hypothesis (inset). In all graphs, change in interaction time is normalized relative to interaction before treatment; loss of all social contact equals a change of score of −1; increase in contact is represented by positive numbers.

We first used data from a field experiment on wild mice^22^ in which adult mice were tagged with individualized RFIDs and their interactions within nest boxes were recorded with antennas placed at the entrances to those nests. We observed then that a large percentage of these animals (40%) dropped ties to other animals when injected with lipopolysaccharides (LPS; a substance that induces an inflammatory response). To understand whether these changes in social behavior were driven by genetic relatedness (as would be expected under the kin protection hypothesis), we used tissue samples that are routinely collected from the target population to genotype all of the animals that were alive when the experiment took place. Using the genotypic data, we were able to estimate genetic relatedness for all possible dyads in the entire population (Supplementary Data S1). Using the antenna data, we defined the social groups present in the population^22^. We then asked the question: following an LPS injection, does the change in the interaction time between a mouse and the members of its social group depend on the average relatedness between the injected mouse and all other members of its social group? Under the kin protection hypothesis, we predicted that as genetic relatedness to the social group increased, the time spent with the group when immune challenged would decrease (the negative relationship represented by the dotted line in Fig. 1a). Instead, we found no evidence for a relationship between these two variables (r = 0.27, *P* = 0.96, N = 33). We then hypothesized that it is not the average relatedness to the social group that matters, but instead relatedness to single social partners (pairwise genetic relatedness). As such, we asked: following an LPS injection, does the change in the interaction time between a mouse and any other given social partner depend on the genetic relatedness of the dyad? Again, under the kin protection hypothesis, we predicted this to be a negative relationship (represented by the dotted line in Fig. 1b). Instead, we found no evidence for a relationship between these two variables (r = 0.073, *P* = 0.87, N = 264). Finally, we tested whether important sources of variation, namely, sex or age of the injected mice could modulate the relationship between relatedness and social behavior, but found no significant role for any of these variables (Tables 1 and 2).

**Table 1:** Linear model predicting changes in time spent in group by genetic relatedness, sex, age and their interactions.

	*estimate*	*s.e*.	*p-val*
Intercept	−1.75	(0.68)	0.02
Relatedness	6.08	(4.84)	0.22
Sex	−0.69	(0.50)	0.18
Age	0.0055	(0.0036)	0.14
Sex * Relatedness	6.44	(3.36)	0.07
Age * Relatedness	−0.031	(0.025)	0.22

Standard errors are calculated using non-parametric bootstrap procedures.

**Table 2:** Linear model predicting changes in time spent between a pair of mice by genetic relatedness, sex, age and their interactions.

	*estimate*	*s.e*.	*p-val*
Intercept	−0.39	(0.08)	0.00
Relatedness	0.45	(0.30)	0.13
Sex	0.15	(0.08)	0.07
Age	0.0004	(0.0003)	0.24
Sex * Relatedness	0.33	(0.31)	0.29
Age * Relatedness	−0.0021	(0.0012)	0.08

Standard errors are calculated using non-parametric bootstrap procedures.

### Laboratory experiment

Testing for the kinship protection hypothesis in a natural wild population accounts for many of the variables that should be important in determining how infection impacts behavior. In the field experiment, we quantified social interactions happening inside nest boxes. Because the study was conducted during the relatively cold non-breeding season when animals spend a substantial amount of time in these nest boxes, we do believe that this is a good measure of social contact time. However, because there was a limited number of those boxes for a large population (40 boxes for over 250 mice), there is a possibility that an immune-challenged animal that wanted to spend time alone in a box would not have been able to do so.

To circumvent this problem and to also assess the interactions happening outside of nest boxes, we set up a laboratory experiment in which groups of four mice were provided with four nest boxes in large indoor enclosures. In this way, the boxes would not constitute a limiting resource and we would be able to observe social interactions both inside and outside of the boxes. We chose to only work with females in these groups since female house mice nest socially, forming important associations with other females with whom they can cooperate to communally rear offspring, a behavior that has strong fitness implications^23–26^.

We manipulated relatedness in these groups so that two of the animals were full sisters (same parents and litter) and two of the animals were not closely related to any of the other animals in the group (each an offspring of different sets of parents). Here too, we administered LPS injections and quantified changes in social interaction time. In addition to interaction in the nest boxes, we also quantified interactions outside the boxes using video cameras (explained in detail in Materials and Methods). Analogous to the field experiment, we then asked: 1) after an LPS injection, does the change in the interaction time between a mouse and its experimental group depend on having a sister (kin) in the group?; and 2) after an LPS injection, does the change in the interaction time between a mouse and any other given social partner depend on whether that partner is a sister (kin)? Under the kin protection hypothesis we predicted (top right of Fig. 1c,d) that following an LPS injection: a) having a kin in the group would reduce interaction time for the injected mouse, and/or b) time interacting with a social partner would be more greatly reduced if that partner was a kin. We found that, after an LPS injection, mice without kin decreased their social interaction time 0.13 units more than mice with kin. However, the difference between the groups was not significant (W = 68.5, *P* = 0.62). Also not significant was the reduction in interaction time to kin by on average 0.07 units more than to non-kin (W = 104, *P* = 1). Once again, we found no evidence for an effect of kinship on social interaction time during an immune challenge.

For the lab experiment, we were also able to investigate other factors that could influence contagion, such as the number of times mice visited a food source (food bowl) or a water source (water bottle). According to the kin protection hypothesis, we would expect LPS injected animals that shared a group with kin to reduce their visits to these sources more than LPS injected animals without close kin. We found that drinking behavior was significantly reduced (χ^2^ = 11.815, *p* = 0.00058, d.f. = 1) in LPS injected animals (parameter estimate ± standard error [SE] = 2.77 ± 0.7) compared to control animals (−0.27 ± 0.5), but not affected by kinship or an interaction between kinship and injection. A similar finding was observed for feeding behavior, where we found that the best fitting model included the factor injection (χ^2^ = 3.51, *p* = 0.061, d.f. = 1), but not kinship or an interaction of kinship and injection. While the effect of injection on decreasing eating behavior (−4.519 ± 1.49 for LPS and −1.731 ± 1.065 for control) was not significant, we found that body weight was significantly changed (χ^2^ = 58.64, *p* < 0.0001, d.f. = 1) in LPS treated animals (−0.77 ± 0.099 g) compared to control (0.214  ± 0.071 g). No effect of kinship or an interaction between kinship and injection was found for changes in body weight.

## Discussion

For a long time, we have mainly considered the importance of host survival when trying to explain the existence of sickness behaviors. The current study is a direct test of an innovative alternative hypothesis for the existence of sickness behaviors which states that the behavioral alterations experienced by infected animals may reduce disease spread to kin, thereby evolving through kin selection^13^. In order to test this hypothesis, we used experimental manipulations of sickness status (through LPS injections) of wild mice in both a laboratory and a field setting.

In both settings, we found that relatedness to the social group did not affect the extent to which immune challenged mice interacted socially. Also, after an immune challenge, social interactions were not more strongly disrupted amongst close kin than non-kin, as would be predicted under the kin protection hypothesis. Finally, in the laboratory experiment we were able to further test whether shared resources (like food and water) would be visited less when a kin was present in the social group, thereby reducing disease spread through direct as well as indirect contacts. While an LPS injection did decrease drinking behavior and led to increased mass loss, we found no evidence for an effect of kinship on numbers of visits to common resources when sick. In sum, these results confirm that our LPS injections were successful at inducing sickness behaviors, but that having a kin in the group did not affect the likelihood of environmental contamination of shared resources by immune-challenged animals, as also proposed by^13^. Importantly, because we quantified both changes to direct (social contacts) and indirect (contamination of shared resources) contacts, which together should account for some of the major routes of disease spread, our results strongly oppose the kin protection hypothesis. In sum, neither the field nor the laboratory experiments performed here suggest that kinship is an important factor influencing the expression of sickness behaviors.

While not directly addressing the kinship protection hypothesis, previous studies corroborate our findings. For instance, it was shown in female house sparrows (*Passer domesticus*) that, even though the probability of deserting a nest increased after an LPS injection, that probability was inversely related to brood size^27^. In other words, as the number of offspring (kin) in the nest increased, so did the likelihood that females overcame sickness behaviors and continued tending to the offspring. It has also been suggested that an LPS injection may have a stronger effect in de-stabilizing dominance hierarchies in dyads of mice that are not siblings than in dyads of mice that are siblings^28^; this would suggest that LPS has a stronger behavioral effect in animals that do not have a kin nearby than in those that do, which is also contrary to the kinship protection hypothesis. Finally, it has been demonstrated in the zebra finch (*Taeniopygia guttata*), a gregarious species of bird, that males express stronger symptoms of sickness behaviors when isolated than when in presence of conspecifics^29^. While relatedness to the social group was not determined in that experiment, the chances of having related individuals in a social group were higher than zero (which would be the chance in an isolation setting).

The instances where we may expect the kinship protection hypothesis to more likely be at play are in eusocial animals. In these organisms, germline (a few individuals that reproduce) and soma (the offspring of the reproducing individuals, which are themselves non-reproducing and perform all other tasks within the colony) are separated. The high level of genetic relatedness in these superorganisms would make it more likely for sickness behaviors to be selected for and evolve through kin selection. While the kinship protection hypothesis has not been directly tested in these organisms to our knowledge, there are examples in eusocial insects where infected individuals remove themselves from the colony^30–32^ or avoid visiting certain critical areas in the colony where disease spread would have important fitness consequences^33^. In these insects, because of the high levels of relatedness and division of labor within socially closed groups, survival of the colony (the superorganism) is tightly linked to survival of kin. Testing the kin protection hypothesis directly in eusocial insects would therefore be of interest. In the interim, we have found no evidence in support of the kinship protection hypothesis, leaving the survival of the host as the primary hypothesis for the evolution of sickness behaviors.

## Materials and Methods

### Ethics statement

Animal use and experimental design were approved by the Veterinary Office Zürich, Switzerland (Kantonales Veterinäramt Zürich, no. 88/2014 and no. 56/2013). All experiments were carried out in accordance with the Veterinary Office Zürich guidelines and are subject to the Swiss animal protection law (TschG).

### Field experiment

The field experiment was carried out over the winter in 2015 and is described in detail in^22^. In brief, for this experiment we used a free-living population of wild house mice living in a barn near Zurich, Switzerland. Mice living in this barn are implanted subcutaneously with a radio-frequency identification (RFID) tag (Trovan ID-100, Euro ID Identifikationssysteme GmbH & Co, Germany) once reaching 18 g in weight. Available in the barn are 40 artificial nest boxes that have a pair of antennas fitted around a tunnel constituting the only entrance to each box. Once a mouse goes through these entrance tunnels, the RFID number for that mouse is recorded, along with a timestamp and the identity of the box used. The use of two antennas allows us to determine the directionality of the movement (in or out of the box). This permits us to obtain information on which nest boxes are used by each mouse, how much time they spend in each box and, most importantly, which animals they meet in a nest box, measured as overlap in time once inside a box. All details of this logging system are provided in^34^. Over the winter, mice in this population show reduced levels of reproductive activity and therefore the majority (>90%) of the animals living in the barn during this experiment were over 18 g and tagged with an RFID^11^.

For both the purposes of our routine data collection from this population and for the purposes of this experiment, mice were caught by placing a glass jar near the entrance to their nest boxes. Once researchers enter the barn and approach the nest boxes, mice tend to hide inside these boxes. The glass jar is placed at the nest box entrance and, as mice leave the box, they run into the jar, which is quickly closed. Another jar is immediately used to replace the one containing a mouse and to allow for capturing additional mice. For the experiment, the jar was then scanned with an RFID reader to detect whether the mouse of interest had been caught.

In order to elicit and inflammatory response and accompanying sickness behaviors, we injected the animals with lipopolysaccharides (LPS). The LPS (*E. coli* Serotype 0111:B4, Sigma-Aldrich #L4391) was dissolved in saline (Sodium Chloride solution 0.9%, Sigma-Aldrich #S8776) and administered at a dose of 1.2 µg g^−1^ of body weight; as a control treatment, we used injections of the same relative amount of saline. To capture and inject the animals, we visited the barn every other day. During each visit, between 3 and 4 animals were injected, each belonging to a different social group. At that time, we also provided food, water and nest materials, as we would routinely do. We used a staggered design to, over the span of 2 months, inject 38 mice with LPS (20 males and 18 females) and 39 mice with saline control (20 males and 19 females). For each day that we injected mice, we used eight hours of antenna data beginning from sunset (~1–1.5 h post-injection) for our analysis because in a lab experiment mice from this population showed altered behaviors post an LPS injection for at least this entire period of time^10^. We then contrasted the data collected by the antennas over this 8 h period after the LPS or saline injection with antenna data collected over the same time of day two days prior to injection.

### Calculating pairwise genetic relatedness

As we do routine visits (every 4 to 6 weeks) to the population where we capture all mice, tag mice that lack RFIDs, and collect tissue samples (ear punches), we were able to determine genetic relatedness amongst all possible dyads living in the population during the field experiment. We used the Wang estimator^35^ as implemented in Coancestry 1.0^36^ to calculate relatedness using genotypes from 25 microsatellite loci (see^37^ for marker details). The Wang estimator was chosen as it correlates highly with pedigree *r* in this population^38^. By using the same set of genetic markers and matching samples collected from the mice when they were pups to samples collected from the same mice when they were first implanted with an RFID, we could determine the age of the mice during the experiment. When no match of the adult sample to the pup sample was found (either because the sequencing did not work, the sample was lost, or because a mouse was not initially found as a pup), we used the date of implantation as a proxy for age. This happened for 13 of the mice used. In these cases, we added 85 days to the difference between experimental date and implantation date, as this was the median difference found between biological age and implantation-determined age for the experimental mice with known age.

### Analysis of field experiment data

Data collected in interaction networks can violate assumptions of the independence of observations; further, parametric assumptions often do not hold. In consequence, we used non-parametric methods in the statistical tests to assess statistical significance. Statistical tests were carried out using R 3.3.3^39^.

#### Data preparation

In this context, we refer to communities as subsets of mice within the entire population having denser connections (meetings in nest boxes) amongst themselves than with the rest of the mice in the population. We determined the communities of mice existing in the barn during the experimental period in a similar way to what is described in^22^. Briefly, nest box encounter data for the entire network over two consecutive experimental nights was summed and then the communities were detected by: disconnected components in the network and a clustering algorithm based on betweenness^40^. The one difference is that we here used weighted communities (this accounts for the strength of the interactions, based on meeting durations), rather than binary communities (interactions are seen as either occurring or not). Due to technical and biological details (namely, reduced antenna data analysis time versus social group detection time, presence of communities that were too small or too unstable for meaningful analyses, and 2 focal mice being found dead; full details provided in^22^), the final sample size here was 33 communities in which one mouse had been injected with LPS.

#### Relation between average relatedness and change in total interaction time

We assessed whether the degree to which mice reduce contact to conspecifics when injected with LPS depended on the relatedness to their social group. To this end, we calculated the normalized change in group interaction time *t* before and after the injection by

1 $$t=\frac{{t}_{a}-{t}_{b}}{{t}_{a}+{t}_{b}}$$

where *t*_*a*_ is the total time in interaction after injection and *t*_*b*_ is the total time in interaction before injection. This metric ranges from −1 to +1, where −1 indicates complete loss of all ties. We then calculated the average genetic relatedness of each focal mouse (injected with LPS) to all members of their group. Finally, we calculated the Pearson correlation between *t* and average relatedness and assessed its significance using a standard bootstrap procedure. In the bootstrap procedure, the observed data was used to repeatedly draw a sample with replacement of the same size to assess the uncertainty of the correlation coefficient. 10000 draws were used in the bootstrap to assess significance. The sample size was N = 33.

To test for a role of sex and age we used a linear regression framework, where we estimated a model with the following regression equations:

2 $$\begin{array}{ccc}t & = & {\beta }_{1}+{\beta }_{2}\ast relatedness+{\beta }_{3}\ast sex+{\beta }_{4}\ast age+{\beta }_{5}\ast sex\ast relatedness\\  &  & +{\beta }_{6}\ast age\ast relatedness+{\epsilon }\end{array}$$

Given the data structure, standard errors where calculated using a non-parametric bootstrap procedure. In the bootstrap procedure, 10000 datasets were created with the same sample size that were sampled from the observed data with replacement. This allows to generate a distribution of regression coefficients and, from this distribution, standard errors.

#### Relation between dyad-wise relatedness and change in dyad-wise interaction time

Given that mice did not change their behavior as a function of average group relatedness after an immune challenge, we tested whether they reduced contact to individual conspecifics dependent on genetic relatedness. To that end we define change in normalized dyad-wise interaction time *d*_*ij*_ between the focal mice *i* and other mice in the group *j* as

3 $$\,{d}_{ij}=\frac{{d}_{a,ij}-{d}_{b,ij}}{{\rm{\max }}({d}_{a,i},\,{d}_{b,i})}$$

where *d*_*a*,*ij*_ is the time mice *i* and *j* spent together after injection and *d*_*b*,*ij*_ is the time spent together before injection. max(*d*_*a*,*I*,_*d*_*b*,*I*_) denotes the strongest tie mouse *i* has to any other mouse in their group. The metric ranges from −1 to +1 as above. In the sample, we correlated the interaction changes of N_i_ = 33 focal mice to a total of N_j_ = 264 other mice that interacted with the focal mice at any time-point with their genetic relatedness and assessed significance using a bootstrap method as outlined above.

In additional analysis on the role of sex and age in moderating the changes in social time spent in a dyads in a linear regression framework, we estimated a model with the following regression equations:

4 $$\begin{array}{ccc}{d}_{ij} & = & {\beta }_{1}+{\beta }_{2}\ast relatedness+\,{\beta }_{3}\ast sex+{\beta }_{4}\ast age+{\beta }_{5}\ast sex\ast relatedness\\  &  & +{\beta }_{6}\ast age\ast relatedness+{\epsilon }\end{array}$$

As in the previous analysis, standard errors were calculated using a non-parametric bootstrap procedure.

### Laboratory experiment

The laboratory experiment was carried out in an animal facility at the University of Zürich. Experimental animals were born in this facility and represented F1 to F3 descendants of wild house mice captured from the same population as the one in the field experiment. From birth and until being tested, animals were kept in cages under standardized laboratory conditions at a temperature of 22 ± 3 °C with a relative humidity of 50–60% and on a 14:10 h light:dark cycle.

The testing room contained six indoor 4 m^2^ enclosures (1.5 m × 2.7 m, with 0.8 m high walls) equipped with 4 nest boxes each. Similar to the field experiment, each nest box contained only one entrance tube fitted with two antennas capable of reading RFIDs. Attached to the ceiling at the top of each enclosure was a video camera (AXIS M1145-L Network camera). To provide some spatial structuring to each enclosure, PVC plates were placed vertically within each enclosure in a way that separated the space into four identical squared areas. Mice placed here could still reach any space within an enclosure, but had to go around the PVC plates or climb the plates (approximately 30 cm high). Each enclosure was covered with 1–2 cm of bedding material (Lignocel Hygienic Animal Bedding, JRS) and was provided with four food plates placed on each corner and two water bottles placed on each end of the enclosure (Supplementary Fig. S1). Sheets of paper towel were provided as nesting material. The testing room had a light cycle where the dark phase started at 1030 hours and lasted until 2030 hours (still a 14:10 h light:dark cycle with 1 h of sunrise and 1 h of dusk).

In total, we tested 29 social groups, each composed of four age-matched females where two individuals were same litter siblings and the other two were offspring of different breeding pairs, and thus unrelated to any of the other animals in the group. The minimum age at testing was 6 weeks, with the average age being 61 days. At least one week prior to testing, animals were transferred in cages with siblings into the test room to start acclimating to its light conditions.

At least twenty-four hours prior to being moved to the enclosures, animals were subcutaneously tagged with RFIDs (Trovan ID-100, Euro ID Identifikationssysteme GmbH & Co, Germany). The four animals in each social group were transferred to one enclosure simultaneously and allowed 6 days to acclimate before any injections were administered. On day 6, the four mice were captured and their ears were painted with different markings, so they could be distinguished during the video recordings. At 0930 hours the same day, the cameras started recording 20 min videos every hour, for a duration of 12 h. The next day (day 7), the focal animal was captured, weighed, and injected intraperitoneally with either LPS (0.6 µg g^−1^ of body weight^21^; *E. coli* Serotype 0111:B4, Sigma-Aldrich #L4391) or saline (Sodium Chloride solution 0.9%, Sigma-Aldrich #S8776) at approximately 0930 hours, then released again into the enclosure. Video was again recorded in the same way for the next 12 h. On day 8, the focal animal was captured and weighed again, to determine changes in body weight. After two days of rest, this process was repeated with a different mouse. So, per social group, two mice were injected. One of the injected mice would have kin in the group and the other not, and one would be injected with LPS and the other with control. There were therefore four possible treatment combinations:

First mouse: has kin + LPS; Second mouse: no kin + control

First mouse: has kin + control; Second mouse: no kin + LPS

First mouse: no kin + LPS; Second mouse: has kin + control

First mouse: no kin + control; Second mouse: has kin + LPS

The treatment combinations were balanced in a way that we had the same number of trials for each. Two days after the second mouse in a social group was injected, the experiment in that enclosure was considered completed and all the animals in the group were euthanized. The enclosure was then emptied of all bedding material, food plates and water bottles, and cleaned with ethanol before setting it up with new materials for a new social group.

### Analysis of laboratory experiment data

The video data was hand coded by a researcher made blind to the treatments in each video. We analyzed the videos starting 1 h after injection until 4 h after injection (span of 3 h, 60 min total of footage) both for the day of the injection and for the day prior to the injection treatment. The most easily identifiable behaviors and thus most representative were: number of times at a water bottle, number of times at a food bowl (Supplementary Data S2) and time spent within two body lengths of another mouse, which we used as a measure of interaction outside the nest. The antenna data was analyzed for the same period of time and we quantified the amount of time spent with other mice inside the nest. We then combined the interaction time measured outside and inside the nest (Supplementary Data S3). This was calculated for each interaction dyad. Due to equipment failure at certain time points, the sample sizes for different analysis vary and are indicated below for each treatment. The body mass data obtained during the experiment can be found as Supplementary Data S4. Statistical tests were carried out using R 3.3.3^39^.

#### Changes in individual behaviors and body weight

We first calculated the change in the behaviors by subtracting the behavior on the day prior to the injection to the behavior on injection day. To test whether kinship status (having a kin or not in the group) or injection treatment had an effect on the change in the number of times visiting the water bottle (drinking behavior) or visiting the food container (eating behavior), we used linear mixed effects models (package “lme4”^41^) including a main effect of injection and kinship, and the interaction between the two, and a random effect of social group. When the interaction term was not significant at *P* < 0.05, it was dropped from the model and no *P* values are reported for this term in such instances. We used likelihood ratio tests (χ^2^) to evaluate the significance of the omitted fixed terms. Visual inspection of residuals plots was used for assessment of fulfilment of model assumptions. Sample sizes for drinking and eating behavior were N = 27 for LPS injected (of which 13 had kin and 14 did not) and N = 26 control injected (of which half had kin). The same methodology was applied to test for changes in body weight and the sample sizes here were N = 29 for LPS injected (half with kin) and N = 28 for control injected (13 with kin).

#### Relation between having kin in the group and change in total interaction time

We used a combination of video-coded data and antenna recorded data to analyze whether, after an LPS injection, mice that have kin in their group were more likely to reduce contact to their group as opposed to mice that did not have kin. We compared the distribution of change in social interaction time as defined above (Equation 1) of mice with kin (N = 12) and mice without kin (N = 13) using a Wilcoxon rank sum test.

#### Relation between change in dyad-wise interaction time for sisters or unrelated mice

In the same logic as the analysis above, we analyzed whether mice that were injected with LPS that have kin in their group specifically reduce contact to their sister. Change in mouse-mouse interaction time was calculated as defined above (Equation 3), where mouse-mouse dyads that did not interact at either time-point were excluded. Interaction changes of N = 12 focal mice with siblings and with non-siblings were compared, using a Wilcoxon rank sum test.

## Electronic supplementary material

Supplementary Information

Dataset 1

Dataset 2

Dataset 3

Dataset 4

## Data Availability

All data used here can be found either in Dryad (10.5061/dryad.nk1b8) or within the Supplementary Information files.
