# Supplementary material for: No evidence for kin protection in the expression of sickness behaviors in house mice
Source: Sci Rep. 2018 Nov 12;8:16682. doi: 10.1038/s41598-018-35174-0 (PMC6232183; doi:10.1038/s41598-018-35174-0)
Supplement: Supplementary file 1 — Supplementary Information [file 41598_2018_35174_MOESM1_ESM.docx]

**Supplementary Information**

**Full title: No evidence for kin protection in the expression of sickness behaviors in house mice**

**Short title: Kinship and sickness behaviors**

**Author information**

Patricia C. Lopes¹^a^*, Per Block^2a^, Alice Pontiggia³, Anna K. Lindholm^4^, Barbara König^4^

1. Schmid College of Science and Technology, Chapman University, One University Drive, Orange, 92866 CA, USA
2. Chair of Social Networks, Department of Humanities, Social and Political Science, ETH Zurich, Clausiusstrasse 50, 8092 Zürich, Switzerland
3. Agroscope, Vetsuisse Faculty, University of Bern, Bern, Switzerland
4. Department of Evolutionary Biology and Environmental Studies, University of Zurich, Winterthurerstrasse 190, 8057 Zürich, Switzerland

a Authors contributed equally to this work

*Corresponding author email address: [lopes@chapman.edu](mailto:lopes@chapman.edu)


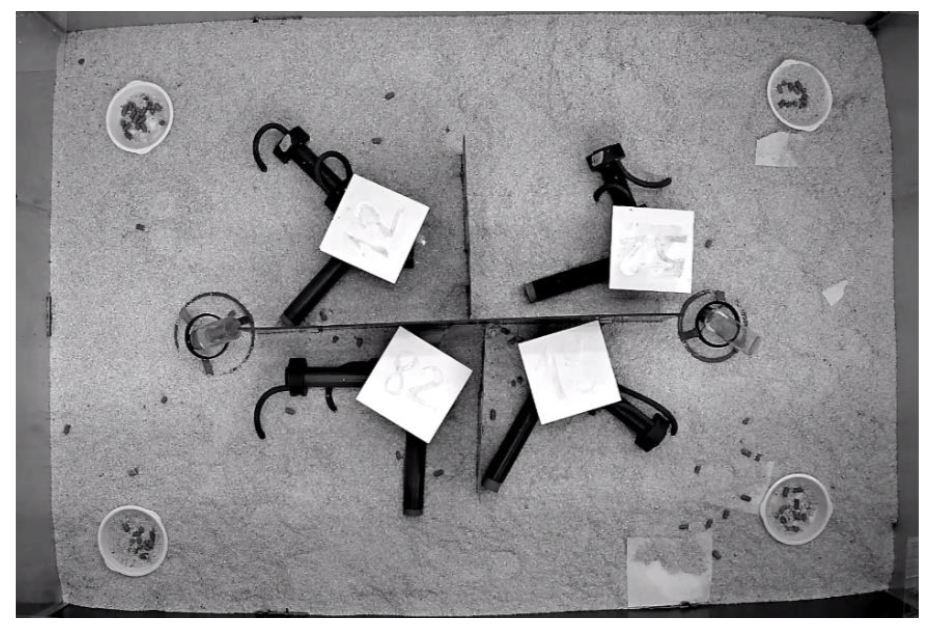


Fig. S1 Representative photograph of the laboratory experiment setup in each enclosure.

The following datasets were uploaded as separate files:

Dataset S1. Pairwise genetic relatedness of all animals in the field experiment during the study time, calculated as described in the text.

Dataset S2. Drinking and eating behavior of injected mice before and after injection (lab experiment).

Dataset S3. Social interactions of injected mice before and after injection, recorded both via antennas placed at the entrance of the nest boxes and via video recordings (lab experiment).

Dataset S4. Body mass (g) of injected mice before and after injection (lab experiment).
