# Supplementary material for: No evidence for kin protection in the expression of sickness behaviors in house mice
Source: Sci Rep. 2018 Nov 12;8:16682. doi: 10.1038/s41598-018-35174-0 (PMC6232183; doi:10.1038/s41598-018-35174-0)
Supplement: Supplementary file 2 — Dataset 1 [file 41598_2018_35174_MOESM2_ESM.pdf]

"idA"	"idB"	"relatedness"
"0007500319"	"0007501420"	0.3061
"0007500319"	"0007580382"	0.3081
"0007500319"	"00075e0043"	−0.0559
"0007500319"	"000757e069"	−0.1062
"0007500319"	"000757e146"	−0.2397
"0007500319"	"00074bd70a"	0.1932
"0007500319"	"00074bdb9c"	0.0965
"0007500319"	"00074be0ac"	−0.0466
"0007500319"	"00074bea69"	0.1717
"0007500319"	"00074bf097"	0.1381
"0007500319"	"00074bf36d"	0.1132
"0007500319"	"00074bfc0b"	0.1335
"0007500319"	"00074c0118"	0.0108
"0007500319"	"00074c10b5"	0.266
"0007500319"	"00074c1ca9"	−0.0684
"0007500319"	"00074c2119"	0.1538
"0007500319"	"00074c3272"	0.396
"0007500319"	"00074c33e4"	0.0962
"0007500319"	"00074c35b2"	0.3476
"0007500319"	"00074c38cd"	−0.0631
"0007500319"	"00074c3a40"	−0.4477
"0007500319"	"00074d84fc"	−0.0792
"0007500319"	"00074d8639"	0.0231
"0007500319"	"00074d8697"	0.2723
"0007500319"	"00074d87af"	−0.1336
"0007500319"	"00074d8813"	−0.0533
"0007500319"	"00074d8817"	0.3334
"0007500319"	"00074d8a39"	0.2822
"0007500319"	"00074d8ad8"	0.2375
"0007500319"	"00074d8c26"	0.0073
"0007500319"	"00074d8ca5"	−0.0684
"0007500319"	"00074d8e0a"	0.2014
"0007500319"	"00074d9179"	−0.0714
"0007500319"	"00074d929f"	0.1267
"0007500319"	"00074d93c4"	−0.1867
"0007500319"	"00074d93d0"	0.293
"0007500319"	"00074d945b"	−0.0265
"0007500319"	"00074d966c"	0.0695
"0007500319"	"00074d9953"	0.1224
"0007500319"	"00074d99b2"	0.1337
"0007500319"	"00074d99f3"	0.3533
"0007500319"	"00074d9afd"	−0.1187
"0007500319"	"00074d9c87"	0.2597
"0007500319"	"00074d9e9e"	−0.3813
"0007500319"	"00074d9f30"	−0.0797
"0007500319"	"00074da036"	0.3611
"0007500319"	"00074da082"	0.0042
"0007500319"	"00074da136"	0.1406
"0007500319"	"00074da3ed"	−0.1478

"0007500319"	"00074da4ac"	0.0952
"0007500319"	"00074da4b8"	-0.0514
"0007500319"	"00074da5e8"	0.3463
"0007500319"	"00074da6b4"	0.1241
"0007500319"	"00074daa3c"	0.1385
"0007500319"	"00074daaf6"	-0.0158
"0007500319"	"00074dad20"	-0.2021
"0007500319"	"00074db098"	-0.0591
"0007500319"	"00074db231"	0.1373
"0007500319"	"00074db3a3"	0.0492
"0007500319"	"00074db5d6"	0.252
"0007500319"	"00074db632"	0.1712
"0007500319"	"00074db688"	0.2751
"0007500319"	"00074db8a6"	-0.1692
"0007500319"	"00074dba19"	0.2189
"0007500319"	"00074dbc2e"	0.0519
"0007500319"	"00074dbe51"	0.0105
"0007500319"	"00074dbe5f"	-0.3103
"0007500319"	"00074dbf6d"	-0.0569
"0007500319"	"00074dc4a5"	0.0524
"0007500319"	"00074dc50c"	0.161
"0007500319"	"00074dcdfa"	0.2364
"0007500319"	"00074dcf5f"	-0.1745
"0007500319"	"00074dd007"	0.0181
"0007500319"	"00074dd163"	-0.0557
"0007500319"	"00074dd3df"	-0.1404
"0007500319"	"00074dd577"	-0.0269
"0007500319"	"00074dd62e"	0.2756
"0007500319"	"00074dd73c"	0.0495
"0007500319"	"00074dda10"	0.0662
"0007500319"	"00074ddab8"	0.3451
"0007500319"	"00074ddd3d"	-0.1
"0007500319"	"00074ddf16"	-0.0649
"0007500319"	"00074ddfc1"	-0.0647
"0007500319"	"00074de21a"	0.1458
"0007500319"	"00074de2a9"	0.0339
"0007500319"	"00074de544"	0.1558
"0007500319"	"00074de98a"	0.092
"0007500319"	"00074dea7e"	-0.1186
"0007500319"	"00074debd9"	-0.2488
"0007500319"	"00074deca3"	-0.1045
"0007500319"	"00074def43"	-0.0727
"0007500319"	"00074def99"	0.1595
"0007500319"	"00074ecdad"	-0.4629
"0007500319"	"00074ecf28"	0.0087
"0007500319"	"00074ed1e1"	-0.1076
"0007500319"	"00074ed83b"	0.0029
"0007500319"	"00074ee5e3"	0.1291
"0007500319"	"00074ee6e0"	0.0942
"0007500319"	"00074eea3a"	0.0751

"0007500319"	"00074eff82"	0.1351
"0007500319"	"00074f0477"	-0.4184
"0007500319"	"00074f08c3"	0.1855
"0007500319"	"00074f1859"	-0.1946
"0007500319"	"00074f2268"	-0.007
"0007500319"	"00074f28be"	0.1765
"0007500319"	"00074f294b"	-0.0175
"0007500319"	"00074f2ddd"	0.2578
"0007500319"	"00074f2e75"	-0.0087
"0007500319"	"00074f3088"	0.3438
"0007500319"	"00074f5a1c"	0.0848
"0007500319"	"00074f75b7"	0.0636
"0007500319"	"00074f8cd9"	-0.1275
"0007500319"	"00074f96dc"	0.1849
"0007500319"	"00074fabaa"	0.1299
"0007500319"	"00074facd9"	-0.1374
"0007500319"	"00074fae3c"	0.2027
"0007500319"	"00074fb0a8"	0.0237
"0007500319"	"00074fb4e4"	-0.121
"0007500319"	"00074fb7c2"	0.2225
"0007500319"	"00074fbd36"	0.1206
"0007500319"	"00074fc27f"	-0.0539
"0007500319"	"00074fc31d"	0.1098
"0007500319"	"00074fd569"	0.0046
"0007500319"	"00074fef15"	0.0132
"0007500319"	"00074ff562"	-0.2183
"0007500319"	"00075007ca"	-0.1505
"0007500319"	"0007500b86"	0.1397
"0007500319"	"0007500d05"	-0.1508
"0007500319"	"0007500ee4"	-0.0485
"0007500319"	"0007500eee"	-0.1369
"0007500319"	"00075013dc"	-0.0629
"0007500319"	"000757b515"	0.1274
"0007500319"	"000757bc5a"	0.2078
"0007500319"	"000757c320"	0.0693
"0007500319"	"000757c9aa"	0.3348
"0007500319"	"000757ccbe"	0.1662
"0007500319"	"000757cfa9"	-0.036
"0007500319"	"000757d390"	-0.2694
"0007500319"	"000757d393"	0.1154
"0007500319"	"000757d598"	0.0184
"0007500319"	"000757d5a2"	-0.0507
"0007500319"	"000757d790"	-0.0148
"0007500319"	"000757e30c"	0.0282
"0007500319"	"000757e4b0"	0.1669
"0007500319"	"000757e7a0"	-0.2483
"0007500319"	"000757e8b3"	0.3534
"0007500319"	"000757f627"	0.1106
"0007500319"	"000757f925"	0.1816
"0007500319"	"000757fa08"	-0.1859

"0007500319"	"000757fe52"	0.1312
"0007500319"	"000758024a"	-0.2081
"0007500319"	"00075804bb"	0.0436
"0007500319"	"00075a0c04"	0.0404
"0007500319"	"00075a3110"	0.2419
"0007500319"	"00075a341a"	-0.1777
"0007500319"	"00075a3dcf"	0.0559
"0007500319"	"00075a3e22"	0.16
"0007500319"	"00075a48d8"	0.2131
"0007500319"	"00075a5cfb"	0.0555
"0007500319"	"00075a6151"	0.102
"0007500319"	"00075a6708"	0.074
"0007500319"	"00075a7319"	-0.2605
"0007500319"	"00075a7723"	-0.0146
"0007500319"	"00075a778b"	-0.1945
"0007500319"	"00075a7b8e"	0.1101
"0007500319"	"00075a7c79"	0.1149
"0007500319"	"00075a81b6"	0.168
"0007500319"	"00075a82ac"	0.1007
"0007500319"	"00075a98e5"	-0.0258
"0007500319"	"00075b0d29"	0.2054
"0007500319"	"00075b102a"	0.1499
"0007500319"	"00075b1074"	0.0236
"0007500319"	"00075b135d"	0.2403
"0007500319"	"00075b138b"	0.2175
"0007500319"	"00075b13a0"	-0.0626
"0007500319"	"00075b13bd"	0.3974
"0007500319"	"00075b16a9"	-0.0842
"0007500319"	"00075b1a28"	-0.022
"0007500319"	"00075b1a97"	0.1853
"0007500319"	"00075b1c7b"	-0.2848
"0007500319"	"00075b1d24"	0.0398
"0007500319"	"00075b202b"	0.0938
"0007500319"	"00075b22cb"	0.0791
"0007500319"	"00075b22da"	-0.0065
"0007500319"	"00075b2556"	-0.1597
"0007500319"	"00075b25de"	0.0898
"0007500319"	"00075b260c"	0.0634
"0007500319"	"00075b26f1"	0.3053
"0007500319"	"00075b2920"	-0.062
"0007500319"	"00075b2a64"	-0.046
"0007500319"	"00075b2a9d"	0.0887
"0007500319"	"00075b2b37"	-0.3373
"0007500319"	"00075b2cdd"	0.1457
"0007500319"	"00075b3038"	0.0878
"0007500319"	"00075b30fe"	-0.0389
"0007500319"	"00075b3362"	0.0192
"0007500319"	"00075b350a"	0.1051
"0007500319"	"00075b350e"	0.144
"0007500319"	"00075b3651"	0.1385

"0007500319"	"00075b38ca"	−0.2128
"0007500319"	"00075b39cc"	0.0774
"0007500319"	"00075b3e1e"	−0.111
"0007500319"	"00075b3e57"	0.1016
"0007500319"	"00075b4079"	0.283
"0007500319"	"00075b4150"	−0.2177
"0007500319"	"00075b4194"	0.3467
"0007500319"	"00075b42d5"	−0.0827
"0007500319"	"00075b4424"	−0.2124
"0007500319"	"00075b4470"	0.2078
"0007500319"	"00075b47ed"	0.027
"0007500319"	"00075b4850"	0.2304
"0007500319"	"00075b4ca0"	0.3416
"0007500319"	"00075b4d7f"	0.2292
"0007500319"	"00075b520f"	0.1603
"0007500319"	"00075b525f"	0.2622
"0007500319"	"00075b58f8"	−0.152
"0007500319"	"00075b5bcc"	0.3026
"0007500319"	"00075b5bfa"	−0.1238
"0007500319"	"00075b6339"	0.2476
"0007500319"	"00075b6658"	0.1548
"0007500319"	"00075b679a"	0.1553
"0007500319"	"00075b6cb7"	0.0971
"0007500319"	"00075b6df8"	0.0317
"0007500319"	"00075b6ff6"	0.0332
"0007500319"	"00075b70ee"	−0.1095
"0007500319"	"00075b7157"	0.223
"0007500319"	"00075b7225"	0.1554
"0007500319"	"00075b7c89"	0.1501
"0007500319"	"00075b9048"	0.1713
"0007500319"	"00075d0801"	0.3043
"0007500319"	"00075d1820"	−0.0187
"0007500319"	"00075d1f3d"	0.2149
"0007500319"	"00075d2329"	−0.0419
"0007500319"	"00075d2b9b"	0.1333
"0007500319"	"00075d3941"	0.0589
"0007500319"	"00075d3e96"	0.1326
"0007500319"	"00075d4864"	−0.1426
"0007500319"	"00075d5961"	−0.0854
"0007500319"	"00075d5a63"	0.0861
"0007500319"	"00075d6150"	0.1135
"0007500319"	"00075d67d0"	0.056
"0007500319"	"00075d67e2"	0.0329
"0007500319"	"00075d73fc"	0.1175
"0007500319"	"00075d7729"	0.1932
"0007500319"	"00075d778c"	−0.1608
"0007500319"	"00075d7b9e"	0.1494
"0007500319"	"00075d7c8f"	0.1155
"0007500319"	"00075d804d"	0.0512
"0007500319"	"00075d819f"	0.2806

"0007500319"	"00075d8601"	0.1917
"0007500319"	"00075d8c6a"	0.0776
"0007500319"	"00075dfedc"	0.182
"0007500319"	"00075e05f2"	0.0926
"0007500319"	"00075e0837"	0.2113
"0007500319"	"00075e092e"	0.1508
"0007500319"	"00075e0965"	−0.3385
"0007500319"	"00075e0bc8"	0.218
"0007500319"	"00075e0fbb"	−0.0959
"0007501420"	"0007580382"	0.3314
"0007501420"	"00075e0043"	0.2539
"0007501420"	"000757e069"	0.2427
"0007501420"	"000757e146"	0.0075
"0007501420"	"00074bd70a"	−0.0494
"0007501420"	"00074bdb9c"	−0.1081
"0007501420"	"00074be0ac"	0.0828
"0007501420"	"00074bea69"	0.07
"0007501420"	"00074bf097"	0.3579
"0007501420"	"00074bf36d"	0.1386
"0007501420"	"00074bfc0b"	0.012
"0007501420"	"00074c0118"	−0.1491
"0007501420"	"00074c10b5"	0.1871
"0007501420"	"00074c1ca9"	−0.0427
"0007501420"	"00074c2119"	0.1879
"0007501420"	"00074c3272"	0.3722
"0007501420"	"00074c33e4"	0.2208
"0007501420"	"00074c35b2"	0.232
"0007501420"	"00074c38cd"	−0.2722
"0007501420"	"00074c3a40"	−0.3386
"0007501420"	"00074d84fc"	0.29
"0007501420"	"00074d8639"	−0.1295
"0007501420"	"00074d8697"	0.2549
"0007501420"	"00074d87af"	0.2262
"0007501420"	"00074d8813"	0.0092
"0007501420"	"00074d8817"	0.3294
"0007501420"	"00074d8a39"	0.032
"0007501420"	"00074d8ad8"	0.3209
"0007501420"	"00074d8c26"	0.3661
"0007501420"	"00074d8ca5"	0.2475
"0007501420"	"00074d8e0a"	0.3001
"0007501420"	"00074d9179"	0.1544
"0007501420"	"00074d929f"	−0.0206
"0007501420"	"00074d93c4"	0.0681
"0007501420"	"00074d93d0"	0.2264
"0007501420"	"00074d945b"	0.3797
"0007501420"	"00074d966c"	0.0867
"0007501420"	"00074d9953"	0.5032
"0007501420"	"00074d99b2"	−0.0332
"0007501420"	"00074d99f3"	0.3239
"0007501420"	"00074d9afd"	−0.1206

"0007501420"	"00074d9c87"	0.2546
"0007501420"	"00074d9e9e"	-0.3365
"0007501420"	"00074d9f30"	0.151
"0007501420"	"00074da036"	0.3817
"0007501420"	"00074da082"	0.0787
"0007501420"	"00074da136"	-0.0775
"0007501420"	"00074da3ed"	0.1813
"0007501420"	"00074da4ac"	0.3636
"0007501420"	"00074da4b8"	0.4527
"0007501420"	"00074da5e8"	0.3302
"0007501420"	"00074da6b4"	0.1647
"0007501420"	"00074daa3c"	0.1982
"0007501420"	"00074daaf6"	-0.085
"0007501420"	"00074dad20"	-0.1648
"0007501420"	"00074db098"	-0.0115
"0007501420"	"00074db231"	-0.0322
"0007501420"	"00074db3a3"	0.5727
"0007501420"	"00074db5d6"	0.0357
"0007501420"	"00074db632"	0.5297
"0007501420"	"00074db688"	-0.0184
"0007501420"	"00074db8a6"	-0.0399
"0007501420"	"00074dba19"	0.1211
"0007501420"	"00074dbc2e"	0.1368
"0007501420"	"00074dbe51"	0.3275
"0007501420"	"00074dbe5f"	-0.2208
"0007501420"	"00074dbf6d"	0.2882
"0007501420"	"00074dc4a5"	-0.1767
"0007501420"	"00074dc50c"	0.3496
"0007501420"	"00074dcdfa"	-0.0365
"0007501420"	"00074dcf5f"	0.1959
"0007501420"	"00074dd007"	0.2893
"0007501420"	"00074dd163"	-0.0038
"0007501420"	"00074dd3df"	-0.0586
"0007501420"	"00074dd577"	0.2125
"0007501420"	"00074dd62e"	0.0957
"0007501420"	"00074dd73c"	0.0777
"0007501420"	"00074dda10"	0.073
"0007501420"	"00074ddab8"	0.3004
"0007501420"	"00074ddd3d"	0.379
"0007501420"	"00074ddf16"	0.2002
"0007501420"	"00074ddfc1"	0.1235
"0007501420"	"00074de21a"	0.134
"0007501420"	"00074de2a9"	0.3756
"0007501420"	"00074de544"	0.1916
"0007501420"	"00074de98a"	0.3401
"0007501420"	"00074dea7e"	0.1541
"0007501420"	"00074debd9"	-0.0791
"0007501420"	"00074deca3"	0.0698
"0007501420"	"00074def43"	0.2075
"0007501420"	"00074def99"	0.0401

"0007501420"	"00074ecdad"	-0.0517
"0007501420"	"00074ecf28"	0.0975
"0007501420"	"00074ed1e1"	0.1589
"0007501420"	"00074ed83b"	0.3244
"0007501420"	"00074ee5e3"	-0.0852
"0007501420"	"00074ee6e0"	-0.1347
"0007501420"	"00074eea3a"	0.1781
"0007501420"	"00074eff82"	-0.0244
"0007501420"	"00074f0477"	-0.2694
"0007501420"	"00074f08c3"	0.0773
"0007501420"	"00074f1859"	0.1121
"0007501420"	"00074f2268"	-0.0701
"0007501420"	"00074f28be"	0.1845
"0007501420"	"00074f294b"	0.1826
"0007501420"	"00074f2ddd"	0.2954
"0007501420"	"00074f2e75"	-0.0372
"0007501420"	"00074f3088"	0.0334
"0007501420"	"00074f5a1c"	0.0237
"0007501420"	"00074f75b7"	0.0041
"0007501420"	"00074f8cd9"	0.0459
"0007501420"	"00074f96dc"	-0.1321
"0007501420"	"00074fabaa"	0.4131
"0007501420"	"00074facd9"	-0.0853
"0007501420"	"00074fae3c"	-0.18
"0007501420"	"00074fb0a8"	0.0384
"0007501420"	"00074fb4e4"	-0.0165
"0007501420"	"00074fb7c2"	0.4081
"0007501420"	"00074fbd36"	0.1571
"0007501420"	"00074fc27f"	0.2883
"0007501420"	"00074fc31d"	0.023
"0007501420"	"00074fd569"	0.2162
"0007501420"	"00074fef15"	-0.0058
"0007501420"	"00074ff562"	-0.1841
"0007501420"	"00075007ca"	-0.1353
"0007501420"	"0007500b86"	0.3071
"0007501420"	"0007500d05"	0.0207
"0007501420"	"0007500ee4"	0.0063
"0007501420"	"0007500eee"	-0.201
"0007501420"	"00075013dc"	0.078
"0007501420"	"000757b515"	0.1436
"0007501420"	"000757bc5a"	0.3172
"0007501420"	"000757c320"	0.2951
"0007501420"	"000757c9aa"	0.2251
"0007501420"	"000757ccbe"	0.1643
"0007501420"	"000757cfa9"	0.1294
"0007501420"	"000757d390"	-0.0937
"0007501420"	"000757d393"	0.3061
"0007501420"	"000757d598"	0.4434
"0007501420"	"000757d5a2"	0.118
"0007501420"	"000757d790"	0.1985

"0007501420"	"000757e30c"	0.401
"0007501420"	"000757e4b0"	0.3342
"0007501420"	"000757e7a0"	-0.0488
"0007501420"	"000757e8b3"	0.4093
"0007501420"	"000757f627"	0.1172
"0007501420"	"000757f925"	0.3244
"0007501420"	"000757fa08"	-0.0613
"0007501420"	"000757fe52"	0.2046
"0007501420"	"000758024a"	0.263
"0007501420"	"00075804bb"	0.3621
"0007501420"	"00075a0c04"	0.1164
"0007501420"	"00075a3110"	0.3025
"0007501420"	"00075a341a"	0.0707
"0007501420"	"00075a3dcf"	7e-04
"0007501420"	"00075a3e22"	0.1557
"0007501420"	"00075a48d8"	0.1057
"0007501420"	"00075a5cfb"	0.3723
"0007501420"	"00075a6151"	0.0222
"0007501420"	"00075a6708"	0.0418
"0007501420"	"00075a7319"	-0.1831
"0007501420"	"00075a7723"	0.196
"0007501420"	"00075a778b"	0.2748
"0007501420"	"00075a7b8e"	-0.0243
"0007501420"	"00075a7c79"	0.2858
"0007501420"	"00075a81b6"	-0.0696
"0007501420"	"00075a82ac"	0.1456
"0007501420"	"00075a98e5"	0.0562
"0007501420"	"00075b0d29"	0.1688
"0007501420"	"00075b102a"	0.2989
"0007501420"	"00075b1074"	-0.0614
"0007501420"	"00075b135d"	0.0911
"0007501420"	"00075b138b"	0.4126
"0007501420"	"00075b13a0"	-0.0591
"0007501420"	"00075b13bd"	-0.1177
"0007501420"	"00075b16a9"	0.1992
"0007501420"	"00075b1a28"	0.0084
"0007501420"	"00075b1a97"	0.107
"0007501420"	"00075b1c7b"	6e-04
"0007501420"	"00075b1d24"	0.0185
"0007501420"	"00075b202b"	0.2202
"0007501420"	"00075b22cb"	0.1641
"0007501420"	"00075b22da"	0.2001
"0007501420"	"00075b2556"	0.2022
"0007501420"	"00075b25de"	0.1666
"0007501420"	"00075b260c"	0.2352
"0007501420"	"00075b26f1"	-0.0587
"0007501420"	"00075b2920"	0.4208
"0007501420"	"00075b2a64"	0.2267
"0007501420"	"00075b2a9d"	0.024
"0007501420"	"00075b2b37"	-0.231

"0007501420"	"00075b2cdd"	0.0812
"0007501420"	"00075b3038"	0.2461
"0007501420"	"00075b30fe"	0.2003
"0007501420"	"00075b3362"	0.0893
"0007501420"	"00075b350a"	-0.0385
"0007501420"	"00075b350e"	0.2356
"0007501420"	"00075b3651"	-0.0256
"0007501420"	"00075b38ca"	-0.1374
"0007501420"	"00075b39cc"	-0.1009
"0007501420"	"00075b3e1e"	0.1926
"0007501420"	"00075b3e57"	0.1579
"0007501420"	"00075b4079"	-0.0083
"0007501420"	"00075b4150"	0.3803
"0007501420"	"00075b4194"	0.2615
"0007501420"	"00075b42d5"	0.1193
"0007501420"	"00075b4424"	0.2171
"0007501420"	"00075b4470"	0.3172
"0007501420"	"00075b47ed"	-0.057
"0007501420"	"00075b4850"	0.3537
"0007501420"	"00075b4ca0"	0.1168
"0007501420"	"00075b4d7f"	0.2508
"0007501420"	"00075b520f"	-0.0476
"0007501420"	"00075b525f"	0.2046
"0007501420"	"00075b58f8"	0.1322
"0007501420"	"00075b5bcc"	0.1492
"0007501420"	"00075b5bfa"	-0.1626
"0007501420"	"00075b6339"	0.2952
"0007501420"	"00075b6658"	-0.1937
"0007501420"	"00075b679a"	0.2815
"0007501420"	"00075b6cb7"	0.1576
"0007501420"	"00075b6df8"	0.0457
"0007501420"	"00075b6ff6"	0.3511
"0007501420"	"00075b70ee"	0.3071
"0007501420"	"00075b7157"	0.0411
"0007501420"	"00075b7225"	0.1784
"0007501420"	"00075b7c89"	0.1937
"0007501420"	"00075b9048"	0.3515
"0007501420"	"00075d0801"	0.2019
"0007501420"	"00075d1820"	0.005
"0007501420"	"00075d1f3d"	0.2783
"0007501420"	"00075d2329"	-0.0813
"0007501420"	"00075d2b9b"	0.3459
"0007501420"	"00075d3941"	-0.1399
"0007501420"	"00075d3e96"	0.1159
"0007501420"	"00075d4864"	0.064
"0007501420"	"00075d5961"	0.065
"0007501420"	"00075d5a63"	0.2744
"0007501420"	"00075d6150"	-0.1961
"0007501420"	"00075d67d0"	-0.2024
"0007501420"	"00075d67e2"	0.3158

"0007501420"	"00075d73fc"	0.2176
"0007501420"	"00075d7729"	-0.0494
"0007501420"	"00075d778c"	-0.1198
"0007501420"	"00075d7b9e"	-0.0656
"0007501420"	"00075d7c8f"	0.1868
"0007501420"	"00075d804d"	0.0775
"0007501420"	"00075d819f"	0.19
"0007501420"	"00075d8601"	-0.1088
"0007501420"	"00075d8c6a"	0.0727
"0007501420"	"00075dfedc"	0.2191
"0007501420"	"00075e05f2"	0.1836
"0007501420"	"00075e0837"	0.1658
"0007501420"	"00075e092e"	0.0579
"0007501420"	"00075e0965"	0.211
"0007501420"	"00075e0bc8"	0.2845
"0007501420"	"00075e0fbb"	0.095
"0007580382"	"00075e0043"	0.1305
"0007580382"	"000757e069"	-0.0141
"0007580382"	"000757e146"	0.0926
"0007580382"	"00074bd70a"	0.0218
"0007580382"	"00074bdb9c"	-0.2275
"0007580382"	"00074be0ac"	0.1203
"0007580382"	"00074bea69"	0.0182
"0007580382"	"00074bf097"	0.2937
"0007580382"	"00074bf36d"	0.1295
"0007580382"	"00074bfc0b"	0.1518
"0007580382"	"00074c0118"	-0.0856
"0007580382"	"00074c10b5"	0.2344
"0007580382"	"00074c1ca9"	-0.1333
"0007580382"	"00074c2119"	0.233
"0007580382"	"00074c3272"	0.1659
"0007580382"	"00074c33e4"	-0.145
"0007580382"	"00074c35b2"	0.0915
"0007580382"	"00074c38cd"	0.1869
"0007580382"	"00074c3a40"	-0.5506
"0007580382"	"00074d84fc"	0.1234
"0007580382"	"00074d8639"	0.2434
"0007580382"	"00074d8697"	0.1596
"0007580382"	"00074d87af"	-0.0723
"0007580382"	"00074d8813"	-0.1704
"0007580382"	"00074d8817"	0.208
"0007580382"	"00074d8a39"	0.073
"0007580382"	"00074d8ad8"	0.3632
"0007580382"	"00074d8c26"	0.0939
"0007580382"	"00074d8ca5"	0.0767
"0007580382"	"00074d8e0a"	0.1943
"0007580382"	"00074d9179"	0.0268
"0007580382"	"00074d929f"	0.003
"0007580382"	"00074d93c4"	0.0518
"0007580382"	"00074d93d0"	0.2981

"0007580382"	"00074d945b"	0.1728
"0007580382"	"00074d966c"	0.117
"0007580382"	"00074d9953"	0.1609
"0007580382"	"00074d99b2"	-0.0168
"0007580382"	"00074d99f3"	0.2931
"0007580382"	"00074d9afd"	0.0331
"0007580382"	"00074d9c87"	0.3456
"0007580382"	"00074d9e9e"	-0.3344
"0007580382"	"00074d9f30"	-0.057
"0007580382"	"00074da036"	0.4727
"0007580382"	"00074da082"	0.2049
"0007580382"	"00074da136"	0.161
"0007580382"	"00074da3ed"	-0.1412
"0007580382"	"00074da4ac"	0.469
"0007580382"	"00074da4b8"	0.1224
"0007580382"	"00074da5e8"	0.3807
"0007580382"	"00074da6b4"	-0.0208
"0007580382"	"00074daa3c"	-0.1225
"0007580382"	"00074daaf6"	-0.0695
"0007580382"	"00074dad20"	-0.176
"0007580382"	"00074db098"	0.0326
"0007580382"	"00074db231"	0.1924
"0007580382"	"00074db3a3"	0.2091
"0007580382"	"00074db5d6"	0.0688
"0007580382"	"00074db632"	0.2946
"0007580382"	"00074db688"	0.2259
"0007580382"	"00074db8a6"	-0.0839
"0007580382"	"00074dba19"	0.1739
"0007580382"	"00074dbc2e"	-0.1357
"0007580382"	"00074dbe51"	0.4663
"0007580382"	"00074dbe5f"	-0.2331
"0007580382"	"00074dbf6d"	-0.0073
"0007580382"	"00074dc4a5"	-0.0984
"0007580382"	"00074dc50c"	0.0804
"0007580382"	"00074dcdfa"	0.0724
"0007580382"	"00074dcf5f"	-0.1019
"0007580382"	"00074dd007"	-0.147
"0007580382"	"00074dd163"	-0.0059
"0007580382"	"00074dd3df"	-0.1159
"0007580382"	"00074dd577"	0.1592
"0007580382"	"00074dd62e"	0.0475
"0007580382"	"00074dd73c"	0.0802
"0007580382"	"00074dda10"	-0.0734
"0007580382"	"00074ddab8"	0.0831
"0007580382"	"00074ddd3d"	0.0717
"0007580382"	"00074ddf16"	0.0332
"0007580382"	"00074ddfc1"	0.1151
"0007580382"	"00074de21a"	-0.0359
"0007580382"	"00074de2a9"	0.268
"0007580382"	"00074de544"	0.1929

"0007580382"	"00074de98a"	0.0706
"0007580382"	"00074dea7e"	-0.0654
"0007580382"	"00074debd9"	-0.269
"0007580382"	"00074deca3"	0.0172
"0007580382"	"00074def43"	-0.0233
"0007580382"	"00074def99"	0.1499
"0007580382"	"00074ecdad"	-0.1684
"0007580382"	"00074ecf28"	-0.2731
"0007580382"	"00074ed1e1"	-0.0335
"0007580382"	"00074ed83b"	0.2769
"0007580382"	"00074ee5e3"	-0.2685
"0007580382"	"00074ee6e0"	-0.0852
"0007580382"	"00074eea3a"	0.2414
"0007580382"	"00074eff82"	0.0577
"0007580382"	"00074f0477"	-0.3438
"0007580382"	"00074f08c3"	-0.0942
"0007580382"	"00074f1859"	0.1155
"0007580382"	"00074f2268"	0.1395
"0007580382"	"00074f28be"	0.265
"0007580382"	"00074f294b"	0.0096
"0007580382"	"00074f2ddd"	0.2739
"0007580382"	"00074f2e75"	0.0858
"0007580382"	"00074f3088"	0.0638
"0007580382"	"00074f5a1c"	0.0226
"0007580382"	"00074f75b7"	-0.0688
"0007580382"	"00074f8cd9"	-0.0417
"0007580382"	"00074f96dc"	-0.0206
"0007580382"	"00074fabaa"	0.0289
"0007580382"	"00074facd9"	-0.1703
"0007580382"	"00074fae3c"	-0.0768
"0007580382"	"00074fb0a8"	0.0805
"0007580382"	"00074fb4e4"	-0.2746
"0007580382"	"00074fb7c2"	0.1076
"0007580382"	"00074fbd36"	0.2581
"0007580382"	"00074fc27f"	0.1427
"0007580382"	"00074fc31d"	0.0068
"0007580382"	"00074fd569"	-0.0968
"0007580382"	"00074fef15"	-0.0413
"0007580382"	"00074ff562"	-0.2616
"0007580382"	"00075007ca"	-0.1411
"0007580382"	"0007500b86"	0.0706
"0007580382"	"0007500d05"	-0.0675
"0007580382"	"0007500ee4"	-0.0446
"0007580382"	"0007500eee"	-0.3487
"0007580382"	"00075013dc"	-0.194
"0007580382"	"000757b515"	0.1651
"0007580382"	"000757bc5a"	0.136
"0007580382"	"000757c320"	-0.0094
"0007580382"	"000757c9aa"	0.1733
"0007580382"	"000757ccbe"	0.3763

"0007580382"	"000757cfa9"	0.0932
"0007580382"	"000757d390"	−0.3092
"0007580382"	"000757d393"	0.1257
"0007580382"	"000757d598"	0.0498
"0007580382"	"000757d5a2"	0.0865
"0007580382"	"000757d790"	−0.0098
"0007580382"	"000757e30c"	0.0325
"0007580382"	"000757e4b0"	0.0805
"0007580382"	"000757e7a0"	−0.011
"0007580382"	"000757e8b3"	0.7021
"0007580382"	"000757f627"	0.0287
"0007580382"	"000757f925"	0.3824
"0007580382"	"000757fa08"	0.0246
"0007580382"	"000757fe52"	0.1356
"0007580382"	"000758024a"	0.0404
"0007580382"	"00075804bb"	0.0175
"0007580382"	"00075a0c04"	0.1966
"0007580382"	"00075a3110"	0.2066
"0007580382"	"00075a341a"	0.01
"0007580382"	"00075a3dcf"	0.0091
"0007580382"	"00075a3e22"	0.2785
"0007580382"	"00075a48d8"	0.2998
"0007580382"	"00075a5cfb"	0.1043
"0007580382"	"00075a6151"	0.2096
"0007580382"	"00075a6708"	−0.0736
"0007580382"	"00075a7319"	−0.246
"0007580382"	"00075a7723"	0.0532
"0007580382"	"00075a778b"	−0.0659
"0007580382"	"00075a7b8e"	−0.0062
"0007580382"	"00075a7c79"	0.3053
"0007580382"	"00075a81b6"	0.1692
"0007580382"	"00075a82ac"	0.0513
"0007580382"	"00075a98e5"	−0.0469
"0007580382"	"00075b0d29"	0.2111
"0007580382"	"00075b102a"	0.1663
"0007580382"	"00075b1074"	0.0557
"0007580382"	"00075b135d"	0.2909
"0007580382"	"00075b138b"	0.1873
"0007580382"	"00075b13a0"	−0.036
"0007580382"	"00075b13bd"	0.0667
"0007580382"	"00075b16a9"	0.2548
"0007580382"	"00075b1a28"	−0.0705
"0007580382"	"00075b1a97"	0.068
"0007580382"	"00075b1c7b"	−0.1932
"0007580382"	"00075b1d24"	0.1051
"0007580382"	"00075b202b"	−0.0217
"0007580382"	"00075b22cb"	0.0552
"0007580382"	"00075b22da"	0.2003
"0007580382"	"00075b2556"	0.0658
"0007580382"	"00075b25de"	−0.1121

"0007580382"	"00075b260c"	0.1206
"0007580382"	"00075b26f1"	0.0335
"0007580382"	"00075b2920"	0.0061
"0007580382"	"00075b2a64"	-0.0804
"0007580382"	"00075b2a9d"	-0.029
"0007580382"	"00075b2b37"	-0.3914
"0007580382"	"00075b2cdd"	0.0795
"0007580382"	"00075b3038"	-0.0813
"0007580382"	"00075b30fe"	-0.0339
"0007580382"	"00075b3362"	-0.0258
"0007580382"	"00075b350a"	0.0132
"0007580382"	"00075b350e"	-0.0323
"0007580382"	"00075b3651"	-0.1098
"0007580382"	"00075b38ca"	-0.1567
"0007580382"	"00075b39cc"	0.1633
"0007580382"	"00075b3e1e"	0.1323
"0007580382"	"00075b3e57"	-0.0293
"0007580382"	"00075b4079"	0.0111
"0007580382"	"00075b4150"	0.0017
"0007580382"	"00075b4194"	0.2674
"0007580382"	"00075b42d5"	-0.0646
"0007580382"	"00075b4424"	-0.0867
"0007580382"	"00075b4470"	0.136
"0007580382"	"00075b47ed"	0.0915
"0007580382"	"00075b4850"	0.1167
"0007580382"	"00075b4ca0"	0.2495
"0007580382"	"00075b4d7f"	0.3954
"0007580382"	"00075b520f"	-0.1412
"0007580382"	"00075b525f"	0.0654
"0007580382"	"00075b58f8"	-0.0068
"0007580382"	"00075b5bcc"	0.0557
"0007580382"	"00075b5bfa"	-0.3236
"0007580382"	"00075b6339"	0.5801
"0007580382"	"00075b6658"	-0.1868
"0007580382"	"00075b679a"	0.1209
"0007580382"	"00075b6cb7"	0.0058
"0007580382"	"00075b6df8"	0.0781
"0007580382"	"00075b6ff6"	0.1571
"0007580382"	"00075b70ee"	-0.0232
"0007580382"	"00075b7157"	0.195
"0007580382"	"00075b7225"	0.2641
"0007580382"	"00075b7c89"	0.3814
"0007580382"	"00075b9048"	0.0701
"0007580382"	"00075d0801"	-0.0758
"0007580382"	"00075d1820"	0.0034
"0007580382"	"00075d1f3d"	0.0177
"0007580382"	"00075d2329"	-0.0225
"0007580382"	"00075d2b9b"	-0.0436
"0007580382"	"00075d3941"	0.0406
"0007580382"	"00075d3e96"	0.0557

"0007580382"	"00075d4864"	−0.1184
"0007580382"	"00075d5961"	0.015
"0007580382"	"00075d5a63"	0.0962
"0007580382"	"00075d6150"	−0.0992
"0007580382"	"00075d67d0"	−0.2349
"0007580382"	"00075d67e2"	0.3337
"0007580382"	"00075d73fc"	0.1858
"0007580382"	"00075d7729"	0.0218
"0007580382"	"00075d778c"	−0.0668
"0007580382"	"00075d7b9e"	0.0666
"0007580382"	"00075d7c8f"	0.561
"0007580382"	"00075d804d"	0.0955
"0007580382"	"00075d819f"	0.0044
"0007580382"	"00075d8601"	0.0232
"0007580382"	"00075d8c6a"	0.2249
"0007580382"	"00075dfedc"	0.4858
"0007580382"	"00075e05f2"	0.0573
"0007580382"	"00075e0837"	0.0249
"0007580382"	"00075e092e"	0.0123
"0007580382"	"00075e0965"	−0.1352
"0007580382"	"00075e0bc8"	0.0498
"0007580382"	"00075e0fbb"	0.0374
"00075e0043"	"000757e069"	0.2529
"00075e0043"	"000757e146"	−0.0462
"00075e0043"	"00074bd70a"	−0.2705
"00075e0043"	"00074bdb9c"	−0.0544
"00075e0043"	"00074be0ac"	0.1201
"00075e0043"	"00074bea69"	−0.0468
"00075e0043"	"00074bf097"	0.1611
"00075e0043"	"00074bf36d"	0.0276
"00075e0043"	"00074bfc0b"	−0.1284
"00075e0043"	"00074c0118"	−0.4316
"00075e0043"	"00074c10b5"	−0.1474
"00075e0043"	"00074c1ca9"	0.0603
"00075e0043"	"00074c2119"	−0.0378
"00075e0043"	"00074c3272"	0.1021
"00075e0043"	"00074c33e4"	−0.1055
"00075e0043"	"00074c35b2"	0.1382
"00075e0043"	"00074c38cd"	0.0556
"00075e0043"	"00074c3a40"	−0.1618
"00075e0043"	"00074d84fc"	0.1702
"00075e0043"	"00074d8639"	−0.0608
"00075e0043"	"00074d8697"	0.0262
"00075e0043"	"00074d87af"	−0.0719
"00075e0043"	"00074d8813"	0.0527
"00075e0043"	"00074d8817"	0.0329
"00075e0043"	"00074d8a39"	−0.3566
"00075e0043"	"00074d8ad8"	0.4139
"00075e0043"	"00074d8c26"	−0.1003
"00075e0043"	"00074d8ca5"	−0.1289

"00075e0043"	"00074d8e0a"	0.2294
"00075e0043"	"00074d9179"	−0.1603
"00075e0043"	"00074d929f"	−0.0634
"00075e0043"	"00074d93c4"	−0.1184
"00075e0043"	"00074d93d0"	−0.1803
"00075e0043"	"00074d945b"	−0.1467
"00075e0043"	"00074d966c"	0.0581
"00075e0043"	"00074d9953"	−0.0458
"00075e0043"	"00074d99b2"	−0.0119
"00075e0043"	"00074d99f3"	−0.185
"00075e0043"	"00074d9afd"	−0.4316
"00075e0043"	"00074d9c87"	0.2761
"00075e0043"	"00074d9e9e"	−0.2856
"00075e0043"	"00074d9f30"	−0.2459
"00075e0043"	"00074da036"	0.3128
"00075e0043"	"00074da082"	−0.4269
"00075e0043"	"00074da136"	0.0834
"00075e0043"	"00074da3ed"	−0.1104
"00075e0043"	"00074da4ac"	−0.023
"00075e0043"	"00074da4b8"	0.1822
"00075e0043"	"00074da5e8"	0.0841
"00075e0043"	"00074da6b4"	0.0329
"00075e0043"	"00074daa3c"	−0.304
"00075e0043"	"00074daaf6"	−0.3966
"00075e0043"	"00074dad20"	−0.1499
"00075e0043"	"00074db098"	0.0376
"00075e0043"	"00074db231"	0.0758
"00075e0043"	"00074db3a3"	0.0184
"00075e0043"	"00074db5d6"	−0.377
"00075e0043"	"00074db632"	0.1785
"00075e0043"	"00074db688"	0.2032
"00075e0043"	"00074db8a6"	−0.3338
"00075e0043"	"00074dba19"	0.0464
"00075e0043"	"00074dbc2e"	−0.0756
"00075e0043"	"00074dbe51"	0.2468
"00075e0043"	"00074dbe5f"	−0.4664
"00075e0043"	"00074dbf6d"	0.0956
"00075e0043"	"00074dc4a5"	−0.5471
"00075e0043"	"00074dc50c"	0.0668
"00075e0043"	"00074dcdfa"	−0.1407
"00075e0043"	"00074dcf5f"	0.0698
"00075e0043"	"00074dd007"	0.0722
"00075e0043"	"00074dd163"	−0.3608
"00075e0043"	"00074dd3df"	−0.5251
"00075e0043"	"00074dd577"	0.1233
"00075e0043"	"00074dd62e"	−0.2705
"00075e0043"	"00074dd73c"	−0.1341
"00075e0043"	"00074dda10"	−0.2279
"00075e0043"	"00074ddab8"	−0.0618
"00075e0043"	"00074ddd3d"	0.414

"00075e0043"	"00074ddf16"	-0.1269
"00075e0043"	"00074ddfc1"	0.0429
"00075e0043"	"00074de21a"	-0.0632
"00075e0043"	"00074de2a9"	0.0049
"00075e0043"	"00074de544"	-0.2018
"00075e0043"	"00074de98a"	-0.1054
"00075e0043"	"00074dea7e"	-0.0409
"00075e0043"	"00074debd9"	-0.2696
"00075e0043"	"00074deca3"	-0.3145
"00075e0043"	"00074def43"	-0.2948
"00075e0043"	"00074def99"	-0.2833
"00075e0043"	"00074ecdad"	-0.0693
"00075e0043"	"00074ecf28"	-0.3168
"00075e0043"	"00074ed1e1"	0.1332
"00075e0043"	"00074ed83b"	-0.0353
"00075e0043"	"00074ee5e3"	-0.3153
"00075e0043"	"00074ee6e0"	-0.2867
"00075e0043"	"00074eea3a"	0.0377
"00075e0043"	"00074eff82"	-0.3517
"00075e0043"	"00074f0477"	-0.1037
"00075e0043"	"00074f08c3"	-0.0461
"00075e0043"	"00074f1859"	-0.1923
"00075e0043"	"00074f2268"	-0.1653
"00075e0043"	"00074f28be"	0.22
"00075e0043"	"00074f294b"	0.1181
"00075e0043"	"00074f2ddd"	0.0924
"00075e0043"	"00074f2e75"	-0.1819
"00075e0043"	"00074f3088"	-0.1687
"00075e0043"	"00074f5a1c"	-0.1296
"00075e0043"	"00074f75b7"	-0.2061
"00075e0043"	"00074f8cd9"	-0.0728
"00075e0043"	"00074f96dc"	-0.5264
"00075e0043"	"00074fabaa"	-0.2546
"00075e0043"	"00074facd9"	-0.1027
"00075e0043"	"00074fae3c"	-0.1847
"00075e0043"	"00074fb0a8"	0.127
"00075e0043"	"00074fb4e4"	-0.0909
"00075e0043"	"00074fb7c2"	0.1704
"00075e0043"	"00074fbd36"	0.0494
"00075e0043"	"00074fc27f"	0.0565
"00075e0043"	"00074fc31d"	0.1296
"00075e0043"	"00074fd569"	-0.3538
"00075e0043"	"00074fef15"	-0.5736
"00075e0043"	"00074ff562"	-0.1077
"00075e0043"	"00075007ca"	0.1453
"00075e0043"	"0007500b86"	-0.0587
"00075e0043"	"0007500d05"	-0.2448
"00075e0043"	"0007500ee4"	0.1027
"00075e0043"	"0007500eee"	-0.271
"00075e0043"	"00075013dc"	0.0611

"00075e0043"	"000757b515"	0.1523
"00075e0043"	"000757bc5a"	0.2407
"00075e0043"	"000757c320"	-0.0047
"00075e0043"	"000757c9aa"	0.1553
"00075e0043"	"000757ccbe"	0.0569
"00075e0043"	"000757cfa9"	0.2216
"00075e0043"	"000757d390"	-0.0372
"00075e0043"	"000757d393"	0.0227
"00075e0043"	"000757d598"	0.1666
"00075e0043"	"000757d5a2"	0.3115
"00075e0043"	"000757d790"	-5e-04
"00075e0043"	"000757e30c"	0.4475
"00075e0043"	"000757e4b0"	0.123
"00075e0043"	"000757e7a0"	0.1919
"00075e0043"	"000757e8b3"	0.1183
"00075e0043"	"000757f627"	0.0131
"00075e0043"	"000757f925"	0.1923
"00075e0043"	"000757fa08"	-0.0778
"00075e0043"	"000757fe52"	-0.1409
"00075e0043"	"000758024a"	0.1614
"00075e0043"	"00075804bb"	0.4189
"00075e0043"	"00075a0c04"	-0.0889
"00075e0043"	"00075a3110"	-0.0872
"00075e0043"	"00075a341a"	-0.1862
"00075e0043"	"00075a3dcf"	-0.1036
"00075e0043"	"00075a3e22"	-0.1285
"00075e0043"	"00075a48d8"	-0.23
"00075e0043"	"00075a5cfb"	0.1811
"00075e0043"	"00075a6151"	0.2435
"00075e0043"	"00075a6708"	0.2045
"00075e0043"	"00075a7319"	-0.0171
"00075e0043"	"00075a7723"	0.1357
"00075e0043"	"00075a778b"	0.1341
"00075e0043"	"00075a7b8e"	0.177
"00075e0043"	"00075a7c79"	0.0488
"00075e0043"	"00075a81b6"	-0.1116
"00075e0043"	"00075a82ac"	-0.0121
"00075e0043"	"00075a98e5"	-0.2321
"00075e0043"	"00075b0d29"	-0.0378
"00075e0043"	"00075b102a"	-0.0951
"00075e0043"	"00075b1074"	-0.1755
"00075e0043"	"00075b135d"	0.015
"00075e0043"	"00075b138b"	-0.0195
"00075e0043"	"00075b13a0"	-0.204
"00075e0043"	"00075b13bd"	-0.4128
"00075e0043"	"00075b16a9"	0.1285
"00075e0043"	"00075b1a28"	-0.1791
"00075e0043"	"00075b1a97"	-0.3632
"00075e0043"	"00075b1c7b"	-0.0604
"00075e0043"	"00075b1d24"	-0.0848

"00075e0043"	"00075b202b"	0.0211
"00075e0043"	"00075b22cb"	-0.315
"00075e0043"	"00075b22da"	-0.1267
"00075e0043"	"00075b2556"	-0.0445
"00075e0043"	"00075b25de"	0.0868
"00075e0043"	"00075b260c"	-0.0799
"00075e0043"	"00075b26f1"	-0.0898
"00075e0043"	"00075b2920"	0.4529
"00075e0043"	"00075b2a64"	-0.0131
"00075e0043"	"00075b2a9d"	0.2243
"00075e0043"	"00075b2b37"	-0.0942
"00075e0043"	"00075b2cdd"	-0.2704
"00075e0043"	"00075b3038"	-0.0758
"00075e0043"	"00075b30fe"	-0.2191
"00075e0043"	"00075b3362"	-0.4193
"00075e0043"	"00075b350a"	-0.1095
"00075e0043"	"00075b350e"	-0.3186
"00075e0043"	"00075b3651"	-0.4198
"00075e0043"	"00075b38ca"	-0.2203
"00075e0043"	"00075b39cc"	0.0546
"00075e0043"	"00075b3e1e"	-0.0493
"00075e0043"	"00075b3e57"	-0.3079
"00075e0043"	"00075b4079"	-0.039
"00075e0043"	"00075b4150"	0.2704
"00075e0043"	"00075b4194"	-0.0138
"00075e0043"	"00075b42d5"	-0.1105
"00075e0043"	"00075b4424"	0.1055
"00075e0043"	"00075b4470"	0.2407
"00075e0043"	"00075b47ed"	-0.0271
"00075e0043"	"00075b4850"	0.3321
"00075e0043"	"00075b4ca0"	0.0372
"00075e0043"	"00075b4d7f"	-0.1009
"00075e0043"	"00075b520f"	0.0567
"00075e0043"	"00075b525f"	-0.2377
"00075e0043"	"00075b58f8"	0.2311
"00075e0043"	"00075b5bcc"	-0.2304
"00075e0043"	"00075b5bfa"	-0.4439
"00075e0043"	"00075b6339"	0.2659
"00075e0043"	"00075b6658"	-0.3394
"00075e0043"	"00075b679a"	-0.1782
"00075e0043"	"00075b6cb7"	0.0966
"00075e0043"	"00075b6df8"	-0.2347
"00075e0043"	"00075b6ff6"	0.0379
"00075e0043"	"00075b70ee"	0.3139
"00075e0043"	"00075b7157"	-0.2903
"00075e0043"	"00075b7225"	-0.1897
"00075e0043"	"00075b7c89"	0.0716
"00075e0043"	"00075b9048"	0.0827
"00075e0043"	"00075d0801"	0.1136
"00075e0043"	"00075d1820"	-0.2519

"00075e0043"	"00075d1f3d"	-0.0826
"00075e0043"	"00075d2329"	0.0286
"00075e0043"	"00075d2b9b"	-0.1421
"00075e0043"	"00075d3941"	-0.2271
"00075e0043"	"00075d3e96"	0.0401
"00075e0043"	"00075d4864"	0.0222
"00075e0043"	"00075d5961"	-0.0113
"00075e0043"	"00075d5a63"	-0.1599
"00075e0043"	"00075d6150"	-0.146
"00075e0043"	"00075d67d0"	-0.2932
"00075e0043"	"00075d67e2"	-0.2345
"00075e0043"	"00075d73fc"	-0.215
"00075e0043"	"00075d7729"	-0.2705
"00075e0043"	"00075d778c"	-0.2612
"00075e0043"	"00075d7b9e"	-0.2254
"00075e0043"	"00075d7c8f"	-0.085
"00075e0043"	"00075d804d"	0.0548
"00075e0043"	"00075d819f"	-0.0684
"00075e0043"	"00075d8601"	-0.224
"00075e0043"	"00075d8c6a"	4e-04
"00075e0043"	"00075dfedc"	0.0696
"00075e0043"	"00075e05f2"	-0.0289
"00075e0043"	"00075e0837"	0.2038
"00075e0043"	"00075e092e"	-0.1671
"00075e0043"	"00075e0965"	0.2702
"00075e0043"	"00075e0bc8"	0.2197
"00075e0043"	"00075e0fbb"	-0.1884
"000757e069"	"000757e146"	0.1351
"000757e069"	"00074bd70a"	-0.1666
"000757e069"	"00074bdb9c"	-0.0325
"000757e069"	"00074be0ac"	-0.0073
"000757e069"	"00074bea69"	-0.0659
"000757e069"	"00074bf097"	0.1243
"000757e069"	"00074bf36d"	-0.0637
"000757e069"	"00074bfc0b"	-0.0561
"000757e069"	"00074c0118"	-0.0423
"000757e069"	"00074c10b5"	-0.334
"000757e069"	"00074c1ca9"	-0.3917
"000757e069"	"00074c2119"	-0.0805
"000757e069"	"00074c3272"	0.0597
"000757e069"	"00074c33e4"	-0.1642
"000757e069"	"00074c35b2"	-0.2316
"000757e069"	"00074c38cd"	-0.0901
"000757e069"	"00074c3a40"	-0.1797
"000757e069"	"00074d84fc"	0.1256
"000757e069"	"00074d8639"	-0.2727
"000757e069"	"00074d8697"	-0.0637
"000757e069"	"00074d87af"	-0.152
"000757e069"	"00074d8813"	0.3568
"000757e069"	"00074d8817"	0.1723

"000757e069"	"00074d8a39"	−0.0175
"000757e069"	"00074d8ad8"	0.2395
"000757e069"	"00074d8c26"	0.1513
"000757e069"	"00074d8ca5"	−0.0793
"000757e069"	"00074d8e0a"	0.302
"000757e069"	"00074d9179"	−0.3046
"000757e069"	"00074d929f"	−0.2448
"000757e069"	"00074d93c4"	−0.0333
"000757e069"	"00074d93d0"	−0.2591
"000757e069"	"00074d945b"	−0.1306
"000757e069"	"00074d966c"	0.1179
"000757e069"	"00074d9953"	0.067
"000757e069"	"00074d99b2"	−0.2023
"000757e069"	"00074d99f3"	−0.0382
"000757e069"	"00074d9afd"	−0.5906
"000757e069"	"00074d9c87"	0.11
"000757e069"	"00074d9e9e"	−0.2206
"000757e069"	"00074d9f30"	0.0495
"000757e069"	"00074da036"	−0.0088
"000757e069"	"00074da082"	−0.49
"000757e069"	"00074da136"	−0.3724
"000757e069"	"00074da3ed"	−0.1068
"000757e069"	"00074da4ac"	0.1618
"000757e069"	"00074da4b8"	0.6577
"000757e069"	"00074da5e8"	0.1725
"000757e069"	"00074da6b4"	−0.0821
"000757e069"	"00074daa3c"	0.0902
"000757e069"	"00074daaf6"	−0.3076
"000757e069"	"00074dad20"	0.0394
"000757e069"	"00074db098"	0.2623
"000757e069"	"00074db231"	−0.1162
"000757e069"	"00074db3a3"	0.1801
"000757e069"	"00074db5d6"	−0.1469
"000757e069"	"00074db632"	0.4599
"000757e069"	"00074db688"	−0.0503
"000757e069"	"00074db8a6"	−0.1771
"000757e069"	"00074dba19"	0.0916
"000757e069"	"00074dbc2e"	0.0424
"000757e069"	"00074dbe51"	0.0939
"000757e069"	"00074dbe5f"	−0.0926
"000757e069"	"00074dbf6d"	0.4416
"000757e069"	"00074dc4a5"	−0.4871
"000757e069"	"00074dc50c"	0.1791
"000757e069"	"00074dcdfa"	−0.1753
"000757e069"	"00074dcf5f"	0.3288
"000757e069"	"00074dd007"	0.4191
"000757e069"	"00074dd163"	−0.2069
"000757e069"	"00074dd3df"	−0.37
"000757e069"	"00074dd577"	0.006
"000757e069"	"00074dd62e"	−0.1795

"000757e069"	"00074dd73c"	0.0892
"000757e069"	"00074dda10"	−0.2537
"000757e069"	"00074ddab8"	−0.0944
"000757e069"	"00074ddd3d"	0.0925
"000757e069"	"00074ddf16"	0.22
"000757e069"	"00074ddfc1"	0.3125
"000757e069"	"00074de21a"	−0.3371
"000757e069"	"00074de2a9"	0.2135
"000757e069"	"00074de544"	−0.3974
"000757e069"	"00074de98a"	−0.1243
"000757e069"	"00074dea7e"	−0.3115
"000757e069"	"00074debd9"	0.1521
"000757e069"	"00074deca3"	−0.1602
"000757e069"	"00074def43"	0.0218
"000757e069"	"00074def99"	−0.2505
"000757e069"	"00074ecdad"	−0.0452
"000757e069"	"00074ecf28"	−0.2066
"000757e069"	"00074ed1e1"	−0.1629
"000757e069"	"00074ed83b"	0.0222
"000757e069"	"00074ee5e3"	−0.0446
"000757e069"	"00074ee6e0"	−0.2212
"000757e069"	"00074eea3a"	−0.4606
"000757e069"	"00074eff82"	−0.2291
"000757e069"	"00074f0477"	−0.1331
"000757e069"	"00074f08c3"	−0.071
"000757e069"	"00074f1859"	−0.0167
"000757e069"	"00074f2268"	−0.1501
"000757e069"	"00074f28be"	0.0795
"000757e069"	"00074f294b"	−0.1168
"000757e069"	"00074f2ddd"	−0.1842
"000757e069"	"00074f2e75"	−0.2922
"000757e069"	"00074f3088"	−0.2518
"000757e069"	"00074f5a1c"	0.0359
"000757e069"	"00074f75b7"	0.1079
"000757e069"	"00074f8cd9"	0.1449
"000757e069"	"00074f96dc"	−0.5036
"000757e069"	"00074fabaa"	−0.1681
"000757e069"	"00074facd9"	0.0023
"000757e069"	"00074fae3c"	−0.2652
"000757e069"	"00074fb0a8"	0.0294
"000757e069"	"00074fb4e4"	−0.1354
"000757e069"	"00074fb7c2"	−0.2463
"000757e069"	"00074fbd36"	−0.1543
"000757e069"	"00074fc27f"	0.3051
"000757e069"	"00074fc31d"	−0.1777
"000757e069"	"00074fd569"	−0.3973
"000757e069"	"00074fef15"	−0.5538
"000757e069"	"00074ff562"	−0.0274
"000757e069"	"00075007ca"	0.4524
"000757e069"	"0007500b86"	0.1589

"000757e069"	"0007500d05"	0.0496
"000757e069"	"0007500ee4"	-0.1348
"000757e069"	"0007500eee"	-0.0705
"000757e069"	"00075013dc"	-0.0372
"000757e069"	"000757b515"	0.1607
"000757e069"	"000757bc5a"	-0.0295
"000757e069"	"000757c320"	0.0786
"000757e069"	"000757c9aa"	0.1193
"000757e069"	"000757ccbe"	-0.2343
"000757e069"	"000757cfa9"	0.0534
"000757e069"	"000757d390"	0.0571
"000757e069"	"000757d393"	0.2138
"000757e069"	"000757d598"	0.1463
"000757e069"	"000757d5a2"	0.0509
"000757e069"	"000757d790"	-0.075
"000757e069"	"000757e30c"	0.5431
"000757e069"	"000757e4b0"	-0.0872
"000757e069"	"000757e7a0"	0.0187
"000757e069"	"000757e8b3"	-0.0461
"000757e069"	"000757f627"	0.0703
"000757e069"	"000757f925"	0.1501
"000757e069"	"000757fa08"	-0.0138
"000757e069"	"000757fe52"	-0.0645
"000757e069"	"000758024a"	0.6553
"000757e069"	"00075804bb"	0.4164
"000757e069"	"00075a0c04"	-0.2814
"000757e069"	"00075a3110"	-0.0217
"000757e069"	"00075a341a"	-0.2741
"000757e069"	"00075a3dcf"	-0.2643
"000757e069"	"00075a3e22"	-0.1539
"000757e069"	"00075a48d8"	-0.2502
"000757e069"	"00075a5cfb"	0.3909
"000757e069"	"00075a6151"	0.2702
"000757e069"	"00075a6708"	0.0878
"000757e069"	"00075a7319"	-0.0227
"000757e069"	"00075a7723"	0.0897
"000757e069"	"00075a778b"	-0.0714
"000757e069"	"00075a7b8e"	0.1368
"000757e069"	"00075a7c79"	-0.0216
"000757e069"	"00075a81b6"	-0.2774
"000757e069"	"00075a82ac"	-0.3011
"000757e069"	"00075a98e5"	-0.2552
"000757e069"	"00075b0d29"	0.0072
"000757e069"	"00075b102a"	0.2002
"000757e069"	"00075b1074"	-0.3656
"000757e069"	"00075b135d"	-0.2553
"000757e069"	"00075b138b"	0.016
"000757e069"	"00075b13a0"	-0.3556
"000757e069"	"00075b13bd"	-0.4008
"000757e069"	"00075b16a9"	0.1037

"000757e069"	"00075b1a28"	−0.091
"000757e069"	"00075b1a97"	−0.3056
"000757e069"	"00075b1c7b"	−0.1337
"000757e069"	"00075b1d24"	−0.1584
"000757e069"	"00075b202b"	0.2585
"000757e069"	"00075b22cb"	−0.236
"000757e069"	"00075b22da"	−0.107
"000757e069"	"00075b2556"	−0.034
"000757e069"	"00075b25de"	0.1279
"000757e069"	"00075b260c"	0.3297
"000757e069"	"00075b26f1"	−0.2769
"000757e069"	"00075b2920"	0.5152
"000757e069"	"00075b2a64"	0.2808
"000757e069"	"00075b2a9d"	0.1738
"000757e069"	"00075b2b37"	−0.069
"000757e069"	"00075b2cdd"	−0.2202
"000757e069"	"00075b3038"	0.1425
"000757e069"	"00075b30fe"	−0.1396
"000757e069"	"00075b3362"	−0.5044
"000757e069"	"00075b350a"	−0.1456
"000757e069"	"00075b350e"	−0.2629
"000757e069"	"00075b3651"	−0.2933
"000757e069"	"00075b38ca"	−0.2563
"000757e069"	"00075b39cc"	−0.2169
"000757e069"	"00075b3e1e"	0.023
"000757e069"	"00075b3e57"	−0.0861
"000757e069"	"00075b4079"	−0.212
"000757e069"	"00075b4150"	0.5815
"000757e069"	"00075b4194"	−0.0413
"000757e069"	"00075b42d5"	0.0389
"000757e069"	"00075b4424"	−0.0406
"000757e069"	"00075b4470"	−0.0456
"000757e069"	"00075b47ed"	−0.3882
"000757e069"	"00075b4850"	−0.0114
"000757e069"	"00075b4ca0"	−0.2063
"000757e069"	"00075b4d7f"	0.0551
"000757e069"	"00075b520f"	−0.0092
"000757e069"	"00075b525f"	−0.2862
"000757e069"	"00075b58f8"	0.4299
"000757e069"	"00075b5bcc"	−0.043
"000757e069"	"00075b5bfa"	−0.1659
"000757e069"	"00075b6339"	0.2372
"000757e069"	"00075b6658"	−0.5857
"000757e069"	"00075b679a"	0.0819
"000757e069"	"00075b6cb7"	−0.0657
"000757e069"	"00075b6df8"	−0.2095
"000757e069"	"00075b6ff6"	0.1832
"000757e069"	"00075b70ee"	0.0218
"000757e069"	"00075b7157"	−0.0433
"000757e069"	"00075b7225"	−0.2456

"000757e069"	"00075b7c89"	-0.2443
"000757e069"	"00075b9048"	0.2423
"000757e069"	"00075d0801"	0.1633
"000757e069"	"00075d1820"	-0.4098
"000757e069"	"00075d1f3d"	0.1037
"000757e069"	"00075d2329"	-0.0783
"000757e069"	"00075d2b9b"	0.1495
"000757e069"	"00075d3941"	-0.3256
"000757e069"	"00075d3e96"	-0.1134
"000757e069"	"00075d4864"	-0.0049
"000757e069"	"00075d5961"	-0.0395
"000757e069"	"00075d5a63"	0.185
"000757e069"	"00075d6150"	-0.2865
"000757e069"	"00075d67d0"	-0.1985
"000757e069"	"00075d67e2"	-0.1801
"000757e069"	"00075d73fc"	-0.351
"000757e069"	"00075d7729"	-0.1666
"000757e069"	"00075d778c"	-0.365
"000757e069"	"00075d7b9e"	-0.1556
"000757e069"	"00075d7c8f"	-0.1379
"000757e069"	"00075d804d"	0.2416
"000757e069"	"00075d819f"	-0.1281
"000757e069"	"00075d8601"	-0.1441
"000757e069"	"00075d8c6a"	0.0454
"000757e069"	"00075dfedc"	-0.044
"000757e069"	"00075e05f2"	0.1426
"000757e069"	"00075e0837"	0.1788
"000757e069"	"00075e092e"	-0.2714
"000757e069"	"00075e0965"	0.5022
"000757e069"	"00075e0bc8"	0.1163
"000757e069"	"00075e0fbb"	-0.1824
"000757e146"	"00074bd70a"	-0.4346
"000757e146"	"00074bdb9c"	-0.0864
"000757e146"	"00074be0ac"	-0.3318
"000757e146"	"00074bea69"	-0.2468
"000757e146"	"00074bf097"	0.3325
"000757e146"	"00074bf36d"	-0.2418
"000757e146"	"00074bfc0b"	-0.2606
"000757e146"	"00074c0118"	-0.0211
"000757e146"	"00074c10b5"	-0.0999
"000757e146"	"00074c1ca9"	-0.6717
"000757e146"	"00074c2119"	-0.412
"000757e146"	"00074c3272"	-0.2014
"000757e146"	"00074c33e4"	-0.0768
"000757e146"	"00074c35b2"	-0.1207
"000757e146"	"00074c38cd"	0.3734
"000757e146"	"00074c3a40"	-0.3389
"000757e146"	"00074d84fc"	-0.0801
"000757e146"	"00074d8639"	0.1158
"000757e146"	"00074d8697"	-0.1331

"000757e146"	"00074d87af"	0.1452
"000757e146"	"00074d8813"	−0.0722
"000757e146"	"00074d8817"	−0.1438
"000757e146"	"00074d8a39"	−0.2288
"000757e146"	"00074d8ad8"	0.091
"000757e146"	"00074d8c26"	0.2243
"000757e146"	"00074d8ca5"	0.0637
"000757e146"	"00074d8e0a"	−0.0395
"000757e146"	"00074d9179"	−0.1821
"000757e146"	"00074d929f"	−0.1301
"000757e146"	"00074d93c4"	0.1801
"000757e146"	"00074d93d0"	−0.123
"000757e146"	"00074d945b"	−0.3067
"000757e146"	"00074d966c"	−0.2327
"000757e146"	"00074d9953"	0.1892
"000757e146"	"00074d99b2"	−0.1564
"000757e146"	"00074d99f3"	−0.2398
"000757e146"	"00074d9afd"	−0.3506
"000757e146"	"00074d9c87"	0.0198
"000757e146"	"00074d9e9e"	−0.11
"000757e146"	"00074d9f30"	−0.2254
"000757e146"	"00074da036"	−0.0326
"000757e146"	"00074da082"	−0.1987
"000757e146"	"00074da136"	0.0464
"000757e146"	"00074da3ed"	−0.0556
"000757e146"	"00074da4ac"	0.3016
"000757e146"	"00074da4b8"	0.0871
"000757e146"	"00074da5e8"	−0.058
"000757e146"	"00074da6b4"	−0.1194
"000757e146"	"00074daa3c"	−0.2774
"000757e146"	"00074daaf6"	−0.2052
"000757e146"	"00074dad20"	−0.1422
"000757e146"	"00074db098"	−0.1244
"000757e146"	"00074db231"	0.2133
"000757e146"	"00074db3a3"	0.0919
"000757e146"	"00074db5d6"	−0.1093
"000757e146"	"00074db632"	0.1825
"000757e146"	"00074db688"	−0.1032
"000757e146"	"00074db8a6"	−0.0671
"000757e146"	"00074dba19"	0.0675
"000757e146"	"00074dbc2e"	−0.1247
"000757e146"	"00074dbe51"	0.057
"000757e146"	"00074dbe5f"	−0.004
"000757e146"	"00074dbf6d"	−0.0483
"000757e146"	"00074dc4a5"	−0.1866
"000757e146"	"00074dc50c"	−0.1451
"000757e146"	"00074dcdfa"	−0.1556
"000757e146"	"00074dcf5f"	−0.3532
"000757e146"	"00074dd007"	−0.2226
"000757e146"	"00074dd163"	0.0695

"000757e146"	"00074dd3df"	-0.1136
"000757e146"	"00074dd577"	-0.2332
"000757e146"	"00074dd62e"	0.0328
"000757e146"	"00074dd73c"	-0.0057
"000757e146"	"00074dda10"	0.0519
"000757e146"	"00074ddab8"	-0.0869
"000757e146"	"00074ddd3d"	-0.2882
"000757e146"	"00074ddf16"	-0.0105
"000757e146"	"00074ddfc1"	0.3153
"000757e146"	"00074de21a"	-0.3942
"000757e146"	"00074de2a9"	0.1753
"000757e146"	"00074de544"	0.0336
"000757e146"	"00074de98a"	0.207
"000757e146"	"00074dea7e"	-0.2059
"000757e146"	"00074debd9"	0.0683
"000757e146"	"00074deca3"	0.0163
"000757e146"	"00074def43"	-0.0699
"000757e146"	"00074def99"	-0.3166
"000757e146"	"00074ecdad"	-0.2699
"000757e146"	"00074ecf28"	-0.4297
"000757e146"	"00074ed1e1"	-0.2256
"000757e146"	"00074ed83b"	-0.0309
"000757e146"	"00074ee5e3"	-0.0353
"000757e146"	"00074ee6e0"	-0.1497
"000757e146"	"00074eea3a"	-0.0561
"000757e146"	"00074eff82"	-0.0326
"000757e146"	"00074f0477"	-0.2829
"000757e146"	"00074f08c3"	-0.293
"000757e146"	"00074f1859"	0.252
"000757e146"	"00074f2268"	-0.1431
"000757e146"	"00074f28be"	0.1527
"000757e146"	"00074f294b"	-0.2029
"000757e146"	"00074f2ddd"	-0.3005
"000757e146"	"00074f2e75"	-0.0629
"000757e146"	"00074f3088"	-0.2521
"000757e146"	"00074f5a1c"	1e-04
"000757e146"	"00074f75b7"	0.0184
"000757e146"	"00074f8cd9"	0.2533
"000757e146"	"00074f96dc"	-0.0766
"000757e146"	"00074fabaa"	-0.0149
"000757e146"	"00074facd9"	-0.008
"000757e146"	"00074fae3c"	-0.0881
"000757e146"	"00074fb0a8"	-0.2228
"000757e146"	"00074fb4e4"	-0.4381
"000757e146"	"00074fb7c2"	0.0249
"000757e146"	"00074fbd36"	-0.0435
"000757e146"	"00074fc27f"	0.0226
"000757e146"	"00074fc31d"	-0.2158
"000757e146"	"00074fd569"	-0.357
"000757e146"	"00074fef15"	-0.2728

"000757e146"	"00074ff562"	-0.4062
"000757e146"	"00075007ca"	-0.3406
"000757e146"	"0007500b86"	0.2415
"000757e146"	"0007500d05"	-0.0526
"000757e146"	"0007500ee4"	-0.2348
"000757e146"	"0007500eee"	0.0111
"000757e146"	"00075013dc"	-0.2783
"000757e146"	"000757b515"	-0.2457
"000757e146"	"000757bc5a"	0.1346
"000757e146"	"000757c320"	-0.2047
"000757e146"	"000757c9aa"	-0.2583
"000757e146"	"000757ccbe"	-0.0405
"000757e146"	"000757cfa9"	-0.0275
"000757e146"	"000757d390"	-0.5135
"000757e146"	"000757d393"	0.2616
"000757e146"	"000757d598"	-0.4265
"000757e146"	"000757d5a2"	-0.262
"000757e146"	"000757d790"	-0.2596
"000757e146"	"000757e30c"	-0.0944
"000757e146"	"000757e4b0"	-0.0237
"000757e146"	"000757e7a0"	-0.1441
"000757e146"	"000757e8b3"	0.1515
"000757e146"	"000757f627"	-0.0389
"000757e146"	"000757f925"	0.1244
"000757e146"	"000757fa08"	0.2626
"000757e146"	"000757fe52"	0.0025
"000757e146"	"000758024a"	0.154
"000757e146"	"00075804bb"	0.0223
"000757e146"	"00075a0c04"	0.062
"000757e146"	"00075a3110"	-0.154
"000757e146"	"00075a341a"	-0.0463
"000757e146"	"00075a3dcf"	-0.0528
"000757e146"	"00075a3e22"	0.1
"000757e146"	"00075a48d8"	0.2525
"000757e146"	"00075a5cfb"	-0.0139
"000757e146"	"00075a6151"	-0.0347
"000757e146"	"00075a6708"	-0.2877
"000757e146"	"00075a7319"	-0.4662
"000757e146"	"00075a7723"	0.1647
"000757e146"	"00075a778b"	0.1933
"000757e146"	"00075a7b8e"	0.1157
"000757e146"	"00075a7c79"	-0.2111
"000757e146"	"00075a81b6"	0.0521
"000757e146"	"00075a82ac"	0.0019
"000757e146"	"00075a98e5"	0.1594
"000757e146"	"00075b0d29"	0.2536
"000757e146"	"00075b102a"	0.0219
"000757e146"	"00075b1074"	-0.1814
"000757e146"	"00075b135d"	-0.0426
"000757e146"	"00075b138b"	0.0993

"000757e146"	"00075b13a0"	-0.0955
"000757e146"	"00075b13bd"	-0.123
"000757e146"	"00075b16a9"	-0.0052
"000757e146"	"00075b1a28"	0.0875
"000757e146"	"00075b1a97"	-0.2364
"000757e146"	"00075b1c7b"	-0.0679
"000757e146"	"00075b1d24"	-0.2087
"000757e146"	"00075b202b"	-0.1073
"000757e146"	"00075b22cb"	0.0202
"000757e146"	"00075b22da"	-0.0837
"000757e146"	"00075b2556"	-0.0604
"000757e146"	"00075b25de"	-0.1345
"000757e146"	"00075b260c"	-0.2254
"000757e146"	"00075b26f1"	-0.1211
"000757e146"	"00075b2920"	-0.1007
"000757e146"	"00075b2a64"	0.0022
"000757e146"	"00075b2a9d"	-0.2155
"000757e146"	"00075b2b37"	-0.4706
"000757e146"	"00075b2cdd"	-0.1059
"000757e146"	"00075b3038"	-0.1405
"000757e146"	"00075b30fe"	-0.1793
"000757e146"	"00075b3362"	-0.4773
"000757e146"	"00075b350a"	-0.2806
"000757e146"	"00075b350e"	-0.256
"000757e146"	"00075b3651"	-0.2166
"000757e146"	"00075b38ca"	-0.0332
"000757e146"	"00075b39cc"	0.0162
"000757e146"	"00075b3e1e"	0.0652
"000757e146"	"00075b3e57"	0.0142
"000757e146"	"00075b4079"	-0.4275
"000757e146"	"00075b4150"	-0.0023
"000757e146"	"00075b4194"	-0.0333
"000757e146"	"00075b42d5"	0.1438
"000757e146"	"00075b4424"	-0.025
"000757e146"	"00075b4470"	0.0992
"000757e146"	"00075b47ed"	-0.1686
"000757e146"	"00075b4850"	-0.1045
"000757e146"	"00075b4ca0"	-0.4041
"000757e146"	"00075b4d7f"	0.3926
"000757e146"	"00075b520f"	-0.076
"000757e146"	"00075b525f"	-0.5022
"000757e146"	"00075b58f8"	0.1914
"000757e146"	"00075b5bcc"	-0.3161
"000757e146"	"00075b5bfa"	-0.5318
"000757e146"	"00075b6339"	0.1925
"000757e146"	"00075b6658"	-0.252
"000757e146"	"00075b679a"	-0.1792
"000757e146"	"00075b6cb7"	-0.2179
"000757e146"	"00075b6df8"	0.063
"000757e146"	"00075b6ff6"	-0.2013

"000757e146"	"00075b70ee"	0.0527
"000757e146"	"00075b7157"	−0.084
"000757e146"	"00075b7225"	−0.1547
"000757e146"	"00075b7c89"	−0.1372
"000757e146"	"00075b9048"	−0.1831
"000757e146"	"00075d0801"	−0.0839
"000757e146"	"00075d1820"	−0.3618
"000757e146"	"00075d1f3d"	−0.1973
"000757e146"	"00075d2329"	−0.0823
"000757e146"	"00075d2b9b"	−0.2473
"000757e146"	"00075d3941"	−0.1899
"000757e146"	"00075d3e96"	−0.3914
"000757e146"	"00075d4864"	0.1716
"000757e146"	"00075d5961"	0.1485
"000757e146"	"00075d5a63"	0.0449
"000757e146"	"00075d6150"	−0.2842
"000757e146"	"00075d67d0"	−0.356
"000757e146"	"00075d67e2"	0.192
"000757e146"	"00075d73fc"	−0.1369
"000757e146"	"00075d7729"	−0.4346
"000757e146"	"00075d778c"	−0.3563
"000757e146"	"00075d7b9e"	−0.0508
"000757e146"	"00075d7c8f"	0.0276
"000757e146"	"00075d804d"	0.1383
"000757e146"	"00075d819f"	−0.3145
"000757e146"	"00075d8601"	−0.0545
"000757e146"	"00075d8c6a"	0.249
"000757e146"	"00075dfedc"	0.0684
"000757e146"	"00075e05f2"	0.2033
"000757e146"	"00075e0837"	−0.2463
"000757e146"	"00075e092e"	−0.2258
"000757e146"	"00075e0965"	−0.01
"000757e146"	"00075e0bc8"	−0.0336
"000757e146"	"00075e0fbb"	−0.2013
"00074bd70a"	"00074bdb9c"	−0.6765
"00074bd70a"	"00074be0ac"	−0.0139
"00074bd70a"	"00074bea69"	0.0276
"00074bd70a"	"00074bf097"	−0.0971
"00074bd70a"	"00074bf36d"	0.0949
"00074bd70a"	"00074bfc0b"	−0.0212
"00074bd70a"	"00074c0118"	−0.4151
"00074bd70a"	"00074c10b5"	−0.0701
"00074bd70a"	"00074c1ca9"	−0.272
"00074bd70a"	"00074c2119"	0.1746
"00074bd70a"	"00074c3272"	0.0959
"00074bd70a"	"00074c33e4"	−0.2572
"00074bd70a"	"00074c35b2"	−0.2092
"00074bd70a"	"00074c38cd"	−0.0223
"00074bd70a"	"00074c3a40"	−0.268
"00074bd70a"	"00074d84fc"	−0.0952

"00074bd70a"	"00074d8639"	−0.0885
"00074bd70a"	"00074d8697"	0.1699
"00074bd70a"	"00074d87af"	−0.2719
"00074bd70a"	"00074d8813"	−0.3344
"00074bd70a"	"00074d8817"	0.1201
"00074bd70a"	"00074d8a39"	0.341
"00074bd70a"	"00074d8ad8"	−0.1227
"00074bd70a"	"00074d8c26"	−0.399
"00074bd70a"	"00074d8ca5"	−0.3121
"00074bd70a"	"00074d8e0a"	−0.1302
"00074bd70a"	"00074d9179"	−0.0937
"00074bd70a"	"00074d929f"	0.0327
"00074bd70a"	"00074d93c4"	−0.3439
"00074bd70a"	"00074d93d0"	0.0952
"00074bd70a"	"00074d945b"	−0.0707
"00074bd70a"	"00074d966c"	0.2075
"00074bd70a"	"00074d9953"	−0.1122
"00074bd70a"	"00074d99b2"	0.464
"00074bd70a"	"00074d99f3"	0.0669
"00074bd70a"	"00074d9afd"	−0.0785
"00074bd70a"	"00074d9c87"	0.0367
"00074bd70a"	"00074d9e9e"	−0.3849
"00074bd70a"	"00074d9f30"	0.1866
"00074bd70a"	"00074da036"	0.0675
"00074bd70a"	"00074da082"	−0.0841
"00074bd70a"	"00074da136"	−0.0176
"00074bd70a"	"00074da3ed"	−0.2515
"00074bd70a"	"00074da4ac"	−0.1113
"00074bd70a"	"00074da4b8"	0.0137
"00074bd70a"	"00074da5e8"	0.1017
"00074bd70a"	"00074da6b4"	0.1356
"00074bd70a"	"00074daa3c"	−0.2827
"00074bd70a"	"00074daaf6"	−0.0644
"00074bd70a"	"00074dad20"	−0.3474
"00074bd70a"	"00074db098"	−0.3081
"00074bd70a"	"00074db231"	−0.0764
"00074bd70a"	"00074db3a3"	−0.1771
"00074bd70a"	"00074db5d6"	−0.0347
"00074bd70a"	"00074db632"	0.0454
"00074bd70a"	"00074db688"	0.1695
"00074bd70a"	"00074db8a6"	−0.2769
"00074bd70a"	"00074dba19"	0.1381
"00074bd70a"	"00074dbc2e"	−0.3111
"00074bd70a"	"00074dbe51"	−0.1174
"00074bd70a"	"00074dbe5f"	−0.5615
"00074bd70a"	"00074dbf6d"	−0.3926
"00074bd70a"	"00074dc4a5"	−0.0521
"00074bd70a"	"00074dc50c"	−0.0589
"00074bd70a"	"00074dcdfa"	−0.2998
"00074bd70a"	"00074dcf5f"	0.0534

"00074bd70a"	"00074dd007"	-0.1819
"00074bd70a"	"00074dd163"	-0.3931
"00074bd70a"	"00074dd3df"	-0.3279
"00074bd70a"	"00074dd577"	0.1284
"00074bd70a"	"00074dd62e"	-0.196
"00074bd70a"	"00074dd73c"	-0.028
"00074bd70a"	"00074dda10"	-0.2123
"00074bd70a"	"00074ddab8"	-0.1813
"00074bd70a"	"00074ddd3d"	-0.1484
"00074bd70a"	"00074ddf16"	-0.3243
"00074bd70a"	"00074ddfc1"	-0.3188
"00074bd70a"	"00074de21a"	-0.0168
"00074bd70a"	"00074de2a9"	0.0229
"00074bd70a"	"00074de544"	-0.0955
"00074bd70a"	"00074de98a"	-0.2296
"00074bd70a"	"00074dea7e"	-0.4754
"00074bd70a"	"00074debd9"	-0.6684
"00074bd70a"	"00074deca3"	-0.4932
"00074bd70a"	"00074def43"	-0.3381
"00074bd70a"	"00074def99"	0.3211
"00074bd70a"	"00074ecdad"	-0.3767
"00074bd70a"	"00074ecf28"	0.1938
"00074bd70a"	"00074ed1e1"	-0.2475
"00074bd70a"	"00074ed83b"	-0.008
"00074bd70a"	"00074ee5e3"	-0.4546
"00074bd70a"	"00074ee6e0"	-0.2582
"00074bd70a"	"00074eea3a"	-0.3101
"00074bd70a"	"00074eff82"	0.0611
"00074bd70a"	"00074f0477"	-0.3811
"00074bd70a"	"00074f08c3"	-0.0067
"00074bd70a"	"00074f1859"	-0.2739
"00074bd70a"	"00074f2268"	-0.0121
"00074bd70a"	"00074f28be"	-0.0412
"00074bd70a"	"00074f294b"	-0.224
"00074bd70a"	"00074f2ddd"	-0.0872
"00074bd70a"	"00074f2e75"	-0.0473
"00074bd70a"	"00074f3088"	0.0154
"00074bd70a"	"00074f5a1c"	-0.0833
"00074bd70a"	"00074f75b7"	-0.07
"00074bd70a"	"00074f8cd9"	-0.3048
"00074bd70a"	"00074f96dc"	-0.2053
"00074bd70a"	"00074fabaa"	-0.4341
"00074bd70a"	"00074facd9"	-0.3014
"00074bd70a"	"00074fae3c"	-0.0693
"00074bd70a"	"00074fb0a8"	-0.2966
"00074bd70a"	"00074fb4e4"	-0.3217
"00074bd70a"	"00074fb7c2"	0.0025
"00074bd70a"	"00074fbd36"	-0.0671
"00074bd70a"	"00074fc27f"	-0.0877
"00074bd70a"	"00074fc31d"	-0.082

"00074bd70a"	"00074fd569"	−0.0274
"00074bd70a"	"00074fef15"	0.1898
"00074bd70a"	"00074ff562"	−0.1192
"00074bd70a"	"00075007ca"	−0.1007
"00074bd70a"	"0007500b86"	−0.3645
"00074bd70a"	"0007500d05"	−0.2039
"00074bd70a"	"0007500ee4"	−0.0932
"00074bd70a"	"0007500eee"	−0.4129
"00074bd70a"	"00075013dc"	−0.2481
"00074bd70a"	"000757b515"	0.1299
"00074bd70a"	"000757bc5a"	−0.2895
"00074bd70a"	"000757c320"	−0.3784
"00074bd70a"	"000757c9aa"	−0.0155
"00074bd70a"	"000757ccbe"	−0.2053
"00074bd70a"	"000757cfa9"	−0.1289
"00074bd70a"	"000757d390"	−0.1921
"00074bd70a"	"000757d393"	−0.3602
"00074bd70a"	"000757d598"	−0.1048
"00074bd70a"	"000757d5a2"	−0.1103
"00074bd70a"	"000757d790"	−0.3009
"00074bd70a"	"000757e30c"	−0.2366
"00074bd70a"	"000757e4b0"	0.0364
"00074bd70a"	"000757e7a0"	−0.0931
"00074bd70a"	"000757e8b3"	−0.0845
"00074bd70a"	"000757f627"	−0.0859
"00074bd70a"	"000757f925"	−0.3715
"00074bd70a"	"000757fa08"	−0.0279
"00074bd70a"	"000757fe52"	−0.209
"00074bd70a"	"000758024a"	−0.0816
"00074bd70a"	"00075804bb"	−0.3898
"00074bd70a"	"00075a0c04"	0.1772
"00074bd70a"	"00075a3110"	0.1296
"00074bd70a"	"00075a341a"	0.1165
"00074bd70a"	"00075a3dcf"	−0.2181
"00074bd70a"	"00075a3e22"	0.0174
"00074bd70a"	"00075a48d8"	−0.0522
"00074bd70a"	"00075a5cfb"	−0.1568
"00074bd70a"	"00075a6151"	−0.3478
"00074bd70a"	"00075a6708"	−0.1178
"00074bd70a"	"00075a7319"	−0.2921
"00074bd70a"	"00075a7723"	−0.2027
"00074bd70a"	"00075a778b"	−0.2829
"00074bd70a"	"00075a7b8e"	−0.1229
"00074bd70a"	"00075a7c79"	0.0015
"00074bd70a"	"00075a81b6"	0.2378
"00074bd70a"	"00075a82ac"	−0.0299
"00074bd70a"	"00075a98e5"	−0.3713
"00074bd70a"	"00075b0d29"	0.0818
"00074bd70a"	"00075b102a"	−0.3049
"00074bd70a"	"00075b1074"	−0.1601

"00074bd70a"	"00075b135d"	0.1178
"00074bd70a"	"00075b138b"	-0.1385
"00074bd70a"	"00075b13a0"	-0.4323
"00074bd70a"	"00075b13bd"	0.1401
"00074bd70a"	"00075b16a9"	-0.3575
"00074bd70a"	"00075b1a28"	-0.3347
"00074bd70a"	"00075b1a97"	0.0497
"00074bd70a"	"00075b1c7b"	-0.5912
"00074bd70a"	"00075b1d24"	-0.0671
"00074bd70a"	"00075b202b"	-0.0277
"00074bd70a"	"00075b22cb"	-0.4006
"00074bd70a"	"00075b22da"	0.0038
"00074bd70a"	"00075b2556"	-0.0764
"00074bd70a"	"00075b25de"	-0.1899
"00074bd70a"	"00075b260c"	-0.0198
"00074bd70a"	"00075b26f1"	-0.0392
"00074bd70a"	"00075b2920"	-0.2195
"00074bd70a"	"00075b2a64"	-0.2855
"00074bd70a"	"00075b2a9d"	-0.2023
"00074bd70a"	"00075b2b37"	0.0176
"00074bd70a"	"00075b2cdd"	-0.1694
"00074bd70a"	"00075b3038"	-0.0816
"00074bd70a"	"00075b30fe"	-0.084
"00074bd70a"	"00075b3362"	-0.1284
"00074bd70a"	"00075b350a"	-0.1616
"00074bd70a"	"00075b350e"	-0.3027
"00074bd70a"	"00075b3651"	-0.1615
"00074bd70a"	"00075b38ca"	-0.3109
"00074bd70a"	"00075b39cc"	-0.4151
"00074bd70a"	"00075b3e1e"	-0.2998
"00074bd70a"	"00075b3e57"	0.0117
"00074bd70a"	"00075b4079"	-0.1708
"00074bd70a"	"00075b4150"	-0.1536
"00074bd70a"	"00075b4194"	-0.2592
"00074bd70a"	"00075b42d5"	-0.3636
"00074bd70a"	"00075b4424"	-0.0367
"00074bd70a"	"00075b4470"	-0.2895
"00074bd70a"	"00075b47ed"	-0.2863
"00074bd70a"	"00075b4850"	-0.1475
"00074bd70a"	"00075b4ca0"	0.2283
"00074bd70a"	"00075b4d7f"	0.2172
"00074bd70a"	"00075b520f"	0.0403
"00074bd70a"	"00075b525f"	0.0516
"00074bd70a"	"00075b58f8"	-0.2272
"00074bd70a"	"00075b5bcc"	0.2166
"00074bd70a"	"00075b5bfa"	-7e-04
"00074bd70a"	"00075b6339"	0.0303
"00074bd70a"	"00075b6658"	0.0019
"00074bd70a"	"00075b679a"	0.1062
"00074bd70a"	"00075b6cb7"	-0.3947

"00074bd70a"	"00075b6df8"	0.2663
"00074bd70a"	"00075b6ff6"	0.0936
"00074bd70a"	"00075b70ee"	−0.238
"00074bd70a"	"00075b7157"	0.1157
"00074bd70a"	"00075b7225"	−0.189
"00074bd70a"	"00075b7c89"	−0.0845
"00074bd70a"	"00075b9048"	0.1172
"00074bd70a"	"00075d0801"	−0.2439
"00074bd70a"	"00075d1820"	−0.1141
"00074bd70a"	"00075d1f3d"	−0.0934
"00074bd70a"	"00075d2329"	−0.1383
"00074bd70a"	"00075d2b9b"	−0.1862
"00074bd70a"	"00075d3941"	−0.2605
"00074bd70a"	"00075d3e96"	−0.0785
"00074bd70a"	"00075d4864"	−0.3111
"00074bd70a"	"00075d5961"	−0.0767
"00074bd70a"	"00075d5a63"	0.1165
"00074bd70a"	"00075d6150"	−0.1028
"00074bd70a"	"00075d67d0"	−0.1211
"00074bd70a"	"00075d67e2"	−0.2031
"00074bd70a"	"00075d73fc"	−0.1126
"00074bd70a"	"00075d7729"	1
"00074bd70a"	"00075d778c"	−0.0146
"00074bd70a"	"00075d7b9e"	0.138
"00074bd70a"	"00075d7c8f"	−0.0891
"00074bd70a"	"00075d804d"	−0.06
"00074bd70a"	"00075d819f"	−0.3184
"00074bd70a"	"00075d8601"	0.025
"00074bd70a"	"00075d8c6a"	0.0309
"00074bd70a"	"00075dfedc"	−0.0579
"00074bd70a"	"00075e05f2"	−0.55
"00074bd70a"	"00075e0837"	−0.2089
"00074bd70a"	"00075e092e"	−0.1981
"00074bd70a"	"00075e0965"	−0.2844
"00074bd70a"	"00075e0bc8"	0.0916
"00074bd70a"	"00075e0fbb"	−0.2202
"00074bdb9c"	"00074be0ac"	−0.0575
"00074bdb9c"	"00074bea69"	−0.0471
"00074bdb9c"	"00074bf097"	0.135
"00074bdb9c"	"00074bf36d"	−0.2436
"00074bdb9c"	"00074bfc0b"	−0.3886
"00074bdb9c"	"00074c0118"	0.1313
"00074bdb9c"	"00074c10b5"	−0.2471
"00074bdb9c"	"00074c1ca9"	−0.5096
"00074bdb9c"	"00074c2119"	−0.1086
"00074bdb9c"	"00074c3272"	−0.0221
"00074bdb9c"	"00074c33e4"	8e−04
"00074bdb9c"	"00074c35b2"	−0.0077
"00074bdb9c"	"00074c38cd"	−0.1157
"00074bdb9c"	"00074c3a40"	−0.3176

"00074bdb9c"	"00074d84fc"	-0.2555
"00074bdb9c"	"00074d8639"	-0.2927
"00074bdb9c"	"00074d8697"	-0.2071
"00074bdb9c"	"00074d87af"	-0.3505
"00074bdb9c"	"00074d8813"	-0.0607
"00074bdb9c"	"00074d8817"	-0.0286
"00074bdb9c"	"00074d8a39"	-0.1751
"00074bdb9c"	"00074d8ad8"	0.1998
"00074bdb9c"	"00074d8c26"	0.0207
"00074bdb9c"	"00074d8ca5"	-0.1934
"00074bdb9c"	"00074d8e0a"	-0.0179
"00074bdb9c"	"00074d9179"	-0.4158
"00074bdb9c"	"00074d929f"	-0.1158
"00074bdb9c"	"00074d93c4"	0.0391
"00074bdb9c"	"00074d93d0"	-0.1068
"00074bdb9c"	"00074d945b"	-0.5765
"00074bdb9c"	"00074d966c"	-0.3719
"00074bdb9c"	"00074d9953"	-0.3275
"00074bdb9c"	"00074d99b2"	-0.5376
"00074bdb9c"	"00074d99f3"	0.0594
"00074bdb9c"	"00074d9afd"	-0.6226
"00074bdb9c"	"00074d9c87"	-0.0322
"00074bdb9c"	"00074d9e9e"	0.1031
"00074bdb9c"	"00074d9f30"	-0.2866
"00074bdb9c"	"00074da036"	0.0259
"00074bdb9c"	"00074da082"	-0.4232
"00074bdb9c"	"00074da136"	-0.3892
"00074bdb9c"	"00074da3ed"	-0.2804
"00074bdb9c"	"00074da4ac"	0.017
"00074bdb9c"	"00074da4b8"	-0.2913
"00074bdb9c"	"00074da5e8"	-0.0479
"00074bdb9c"	"00074da6b4"	-0.3882
"00074bdb9c"	"00074daa3c"	-0.0534
"00074bdb9c"	"00074daaf6"	-0.0455
"00074bdb9c"	"00074dad20"	0.3424
"00074bdb9c"	"00074db098"	0.1503
"00074bdb9c"	"00074db231"	-0.1339
"00074bdb9c"	"00074db3a3"	0.0268
"00074bdb9c"	"00074db5d6"	-0.2126
"00074bdb9c"	"00074db632"	-0.174
"00074bdb9c"	"00074db688"	-0.2889
"00074bdb9c"	"00074db8a6"	0.1448
"00074bdb9c"	"00074dba19"	-0.1878
"00074bdb9c"	"00074dbc2e"	0.3584
"00074bdb9c"	"00074dbe51"	-0.1806
"00074bdb9c"	"00074dbe5f"	0.0074
"00074bdb9c"	"00074dbf6d"	-0.1184
"00074bdb9c"	"00074dc4a5"	-0.5245
"00074bdb9c"	"00074dc50c"	-0.1014
"00074bdb9c"	"00074dcdfa"	-0.0323

"00074bdb9c"	"00074dcf5f"	-0.408
"00074bdb9c"	"00074dd007"	-0.0035
"00074bdb9c"	"00074dd163"	0.2455
"00074bdb9c"	"00074dd3df"	-0.5688
"00074bdb9c"	"00074dd577"	-0.3358
"00074bdb9c"	"00074dd62e"	-0.1034
"00074bdb9c"	"00074dd73c"	0.107
"00074bdb9c"	"00074dda10"	-0.0555
"00074bdb9c"	"00074ddab8"	0.0525
"00074bdb9c"	"00074ddd3d"	-0.1899
"00074bdb9c"	"00074ddf16"	0.1319
"00074bdb9c"	"00074ddfc1"	0.2714
"00074bdb9c"	"00074de21a"	-0.4105
"00074bdb9c"	"00074de2a9"	-0.3176
"00074bdb9c"	"00074de544"	-0.1841
"00074bdb9c"	"00074de98a"	-0.0483
"00074bdb9c"	"00074dea7e"	-0.1802
"00074bdb9c"	"00074debd9"	0.2433
"00074bdb9c"	"00074deca3"	0.0662
"00074bdb9c"	"00074def43"	0.1248
"00074bdb9c"	"00074def99"	-0.2905
"00074bdb9c"	"00074ecdad"	-0.4638
"00074bdb9c"	"00074ecf28"	-0.4917
"00074bdb9c"	"00074ed1e1"	-0.3005
"00074bdb9c"	"00074ed83b"	-0.1144
"00074bdb9c"	"00074ee5e3"	0.6733
"00074bdb9c"	"00074ee6e0"	0.0913
"00074bdb9c"	"00074eea3a"	-0.1476
"00074bdb9c"	"00074eff82"	-0.0535
"00074bdb9c"	"00074f0477"	0.1097
"00074bdb9c"	"00074f08c3"	-0.1668
"00074bdb9c"	"00074f1859"	-0.1297
"00074bdb9c"	"00074f2268"	-0.6788
"00074bdb9c"	"00074f28be"	-0.1952
"00074bdb9c"	"00074f294b"	-0.2077
"00074bdb9c"	"00074f2ddd"	-0.3667
"00074bdb9c"	"00074f2e75"	-0.6821
"00074bdb9c"	"00074f3088"	-0.0418
"00074bdb9c"	"00074f5a1c"	-0.1014
"00074bdb9c"	"00074f75b7"	0.3242
"00074bdb9c"	"00074f8cd9"	0.2698
"00074bdb9c"	"00074f96dc"	-0.3353
"00074bdb9c"	"00074fabaa"	-0.2237
"00074bdb9c"	"00074facd9"	0.1594
"00074bdb9c"	"00074fae3c"	0.2425
"00074bdb9c"	"00074fb0a8"	-0.0913
"00074bdb9c"	"00074fb4e4"	-0.0238
"00074bdb9c"	"00074fb7c2"	-0.207
"00074bdb9c"	"00074fbd36"	-0.2868
"00074bdb9c"	"00074fc27f"	-0.2089

"00074bdb9c"	"00074fc31d"	-0.0131
"00074bdb9c"	"00074fd569"	-0.1698
"00074bdb9c"	"00074fef15"	-0.2269
"00074bdb9c"	"00074ff562"	-0.4104
"00074bdb9c"	"00075007ca"	-0.1065
"00074bdb9c"	"0007500b86"	0.1723
"00074bdb9c"	"0007500d05"	0.0653
"00074bdb9c"	"0007500ee4"	-0.0798
"00074bdb9c"	"0007500eee"	0.1962
"00074bdb9c"	"00075013dc"	-0.4764
"00074bdb9c"	"000757b515"	-0.0972
"00074bdb9c"	"000757bc5a"	0.0228
"00074bdb9c"	"000757c320"	0.0816
"00074bdb9c"	"000757c9aa"	0.1408
"00074bdb9c"	"000757ccbe"	-0.3761
"00074bdb9c"	"000757cfa9"	-0.1275
"00074bdb9c"	"000757d390"	-0.269
"00074bdb9c"	"000757d393"	0.1481
"00074bdb9c"	"000757d598"	-0.3231
"00074bdb9c"	"000757d5a2"	-0.2575
"00074bdb9c"	"000757d790"	0.1987
"00074bdb9c"	"000757e30c"	-0.0979
"00074bdb9c"	"000757e4b0"	-0.0669
"00074bdb9c"	"000757e7a0"	-0.1862
"00074bdb9c"	"000757e8b3"	-0.1254
"00074bdb9c"	"000757f627"	-0.0538
"00074bdb9c"	"000757f925"	0.1488
"00074bdb9c"	"000757fa08"	-0.4706
"00074bdb9c"	"000757fe52"	-0.2005
"00074bdb9c"	"000758024a"	-0.3433
"00074bdb9c"	"00075804bb"	-0.0349
"00074bdb9c"	"00075a0c04"	-0.3729
"00074bdb9c"	"00075a3110"	-0.2917
"00074bdb9c"	"00075a341a"	-0.623
"00074bdb9c"	"00075a3dcf"	-0.006
"00074bdb9c"	"00075a3e22"	-0.3852
"00074bdb9c"	"00075a48d8"	-0.2998
"00074bdb9c"	"00075a5cfb"	-0.3988
"00074bdb9c"	"00075a6151"	0.1919
"00074bdb9c"	"00075a6708"	0.0958
"00074bdb9c"	"00075a7319"	-0.1001
"00074bdb9c"	"00075a7723"	-0.0706
"00074bdb9c"	"00075a778b"	-0.4756
"00074bdb9c"	"00075a7b8e"	0.0036
"00074bdb9c"	"00075a7c79"	-0.2891
"00074bdb9c"	"00075a81b6"	-0.3522
"00074bdb9c"	"00075a82ac"	-0.249
"00074bdb9c"	"00075a98e5"	-0.1674
"00074bdb9c"	"00075b0d29"	-0.1045
"00074bdb9c"	"00075b102a"	-0.0905

"00074bdb9c"	"00075b1074"	-0.5508
"00074bdb9c"	"00075b135d"	-0.3618
"00074bdb9c"	"00075b138b"	-0.0204
"00074bdb9c"	"00075b13a0"	-0.4476
"00074bdb9c"	"00075b13bd"	-0.3053
"00074bdb9c"	"00075b16a9"	-0.0352
"00074bdb9c"	"00075b1a28"	-0.086
"00074bdb9c"	"00075b1a97"	-0.2138
"00074bdb9c"	"00075b1c7b"	-0.1893
"00074bdb9c"	"00075b1d24"	-0.0779
"00074bdb9c"	"00075b202b"	-0.0566
"00074bdb9c"	"00075b22cb"	-0.0789
"00074bdb9c"	"00075b22da"	-0.4395
"00074bdb9c"	"00075b2556"	-0.4969
"00074bdb9c"	"00075b25de"	0.3924
"00074bdb9c"	"00075b260c"	-0.269
"00074bdb9c"	"00075b26f1"	0.0826
"00074bdb9c"	"00075b2920"	-0.1647
"00074bdb9c"	"00075b2a64"	-0.2479
"00074bdb9c"	"00075b2a9d"	0.073
"00074bdb9c"	"00075b2b37"	-0.4004
"00074bdb9c"	"00075b2cdd"	-0.1397
"00074bdb9c"	"00075b3038"	0.3523
"00074bdb9c"	"00075b30fe"	-0.4859
"00074bdb9c"	"00075b3362"	-0.2959
"00074bdb9c"	"00075b350a"	-0.3364
"00074bdb9c"	"00075b350e"	-0.1561
"00074bdb9c"	"00075b3651"	0.0371
"00074bdb9c"	"00075b38ca"	-0.1045
"00074bdb9c"	"00075b39cc"	0.195
"00074bdb9c"	"00075b3e1e"	0.067
"00074bdb9c"	"00075b3e57"	-0.3321
"00074bdb9c"	"00075b4079"	-0.0872
"00074bdb9c"	"00075b4150"	-0.293
"00074bdb9c"	"00075b4194"	0.0869
"00074bdb9c"	"00075b42d5"	0.0054
"00074bdb9c"	"00075b4424"	-0.443
"00074bdb9c"	"00075b4470"	0.0228
"00074bdb9c"	"00075b47ed"	0.0295
"00074bdb9c"	"00075b4850"	0.0295
"00074bdb9c"	"00075b4ca0"	-0.3464
"00074bdb9c"	"00075b4d7f"	-0.2834
"00074bdb9c"	"00075b520f"	0.0797
"00074bdb9c"	"00075b525f"	-0.3031
"00074bdb9c"	"00075b58f8"	-0.1675
"00074bdb9c"	"00075b5bcc"	-0.0599
"00074bdb9c"	"00075b5bfa"	0.1852
"00074bdb9c"	"00075b6339"	0.0134
"00074bdb9c"	"00075b6658"	-0.1725
"00074bdb9c"	"00075b679a"	-0.0146

"00074bdb9c"	"00075b6cb7"	-0.1073
"00074bdb9c"	"00075b6df8"	-0.2812
"00074bdb9c"	"00075b6ff6"	-0.3551
"00074bdb9c"	"00075b70ee"	-0.2022
"00074bdb9c"	"00075b7157"	0.0227
"00074bdb9c"	"00075b7225"	-0.1474
"00074bdb9c"	"00075b7c89"	-0.1842
"00074bdb9c"	"00075b9048"	-0.0317
"00074bdb9c"	"00075d0801"	0.2366
"00074bdb9c"	"00075d1820"	-0.0168
"00074bdb9c"	"00075d1f3d"	-0.1845
"00074bdb9c"	"00075d2329"	0.0239
"00074bdb9c"	"00075d2b9b"	-0.2746
"00074bdb9c"	"00075d3941"	-0.1545
"00074bdb9c"	"00075d3e96"	-0.0082
"00074bdb9c"	"00075d4864"	-0.25
"00074bdb9c"	"00075d5961"	-0.1572
"00074bdb9c"	"00075d5a63"	-0.2041
"00074bdb9c"	"00075d6150"	-0.1649
"00074bdb9c"	"00075d67d0"	-0.1057
"00074bdb9c"	"00075d67e2"	-0.2453
"00074bdb9c"	"00075d73fc"	-0.0857
"00074bdb9c"	"00075d7729"	-0.6765
"00074bdb9c"	"00075d778c"	-0.3025
"00074bdb9c"	"00075d7b9e"	-0.004
"00074bdb9c"	"00075d7c8f"	-0.1899
"00074bdb9c"	"00075d804d"	0.0375
"00074bdb9c"	"00075d819f"	0.0794
"00074bdb9c"	"00075d8601"	0.079
"00074bdb9c"	"00075d8c6a"	-0.0904
"00074bdb9c"	"00075dfedc"	-0.0481
"00074bdb9c"	"00075e05f2"	0.1859
"00074bdb9c"	"00075e0837"	0.1107
"00074bdb9c"	"00075e092e"	-0.1997
"00074bdb9c"	"00075e0965"	-0.392
"00074bdb9c"	"00075e0bc8"	0.0272
"00074bdb9c"	"00075e0fbb"	-0.4717
"00074be0ac"	"00074bea69"	0.1145
"00074be0ac"	"00074bf097"	-0.0052
"00074be0ac"	"00074bf36d"	-0.112
"00074be0ac"	"00074bfc0b"	-0.0539
"00074be0ac"	"00074c0118"	-0.15
"00074be0ac"	"00074c10b5"	-0.1019
"00074be0ac"	"00074c1ca9"	-0.156
"00074be0ac"	"00074c2119"	0.1845
"00074be0ac"	"00074c3272"	0.065
"00074be0ac"	"00074c33e4"	0.0172
"00074be0ac"	"00074c35b2"	0.0058
"00074be0ac"	"00074c38cd"	-0.367
"00074be0ac"	"00074c3a40"	-0.2577

"00074be0ac"	"00074d84fc"	0.1627
"00074be0ac"	"00074d8639"	-0.0595
"00074be0ac"	"00074d8697"	-0.064
"00074be0ac"	"00074d87af"	-0.1474
"00074be0ac"	"00074d8813"	-0.156
"00074be0ac"	"00074d8817"	0.1645
"00074be0ac"	"00074d8a39"	0.0275
"00074be0ac"	"00074d8ad8"	0.2439
"00074be0ac"	"00074d8c26"	4e-04
"00074be0ac"	"00074d8ca5"	0.0547
"00074be0ac"	"00074d8e0a"	0.2803
"00074be0ac"	"00074d9179"	-0.0403
"00074be0ac"	"00074d929f"	-0.186
"00074be0ac"	"00074d93c4"	-0.0477
"00074be0ac"	"00074d93d0"	0.1557
"00074be0ac"	"00074d945b"	0.0376
"00074be0ac"	"00074d966c"	0.1967
"00074be0ac"	"00074d9953"	-0.0974
"00074be0ac"	"00074d99b2"	-0.0021
"00074be0ac"	"00074d99f3"	0.0904
"00074be0ac"	"00074d9afd"	-0.171
"00074be0ac"	"00074d9c87"	0.3424
"00074be0ac"	"00074d9e9e"	-0.2514
"00074be0ac"	"00074d9f30"	0.0378
"00074be0ac"	"00074da036"	0.2661
"00074be0ac"	"00074da082"	0.0502
"00074be0ac"	"00074da136"	-0.1507
"00074be0ac"	"00074da3ed"	-0.1198
"00074be0ac"	"00074da4ac"	0.0582
"00074be0ac"	"00074da4b8"	-0.0409
"00074be0ac"	"00074da5e8"	0.3294
"00074be0ac"	"00074da6b4"	-0.0206
"00074be0ac"	"00074daa3c"	-0.0514
"00074be0ac"	"00074daaf6"	0.0326
"00074be0ac"	"00074dad20"	0.0085
"00074be0ac"	"00074db098"	0.4982
"00074be0ac"	"00074db231"	-0.0195
"00074be0ac"	"00074db3a3"	0.1648
"00074be0ac"	"00074db5d6"	0.0426
"00074be0ac"	"00074db632"	0.0078
"00074be0ac"	"00074db688"	0.0813
"00074be0ac"	"00074db8a6"	0.0938
"00074be0ac"	"00074dba19"	0.0233
"00074be0ac"	"00074dbc2e"	0.1613
"00074be0ac"	"00074dbe51"	0.2765
"00074be0ac"	"00074dbe5f"	-0.3044
"00074be0ac"	"00074dbf6d"	0.1392
"00074be0ac"	"00074dc4a5"	-0.2223
"00074be0ac"	"00074dc50c"	0.1282
"00074be0ac"	"00074dcdfa"	-0.0046

"00074be0ac"	"00074dcf5f"	0.0756
"00074be0ac"	"00074dd007"	−0.0519
"00074be0ac"	"00074dd163"	−0.1126
"00074be0ac"	"00074dd3df"	−0.3282
"00074be0ac"	"00074dd577"	0.4085
"00074be0ac"	"00074dd62e"	−0.0527
"00074be0ac"	"00074dd73c"	0.2954
"00074be0ac"	"00074dda10"	−0.0175
"00074be0ac"	"00074ddab8"	0.1389
"00074be0ac"	"00074ddd3d"	0.2679
"00074be0ac"	"00074ddf16"	0.0704
"00074be0ac"	"00074ddfc1"	0.0806
"00074be0ac"	"00074de21a"	−0.1789
"00074be0ac"	"00074de2a9"	−0.1251
"00074be0ac"	"00074de544"	0.0738
"00074be0ac"	"00074de98a"	−0.0303
"00074be0ac"	"00074dea7e"	−0.1169
"00074be0ac"	"00074debd9"	−0.0686
"00074be0ac"	"00074deca3"	−0.0132
"00074be0ac"	"00074def43"	0.177
"00074be0ac"	"00074def99"	0.1059
"00074be0ac"	"00074ecdad"	−0.0213
"00074be0ac"	"00074ecf28"	−0.1851
"00074be0ac"	"00074ed1e1"	−0.0768
"00074be0ac"	"00074ed83b"	0.2967
"00074be0ac"	"00074ee5e3"	−0.0527
"00074be0ac"	"00074ee6e0"	−0.1066
"00074be0ac"	"00074eea3a"	−0.1124
"00074be0ac"	"00074eff82"	−0.241
"00074be0ac"	"00074f0477"	−0.043
"00074be0ac"	"00074f08c3"	−0.2021
"00074be0ac"	"00074f1859"	−0.0621
"00074be0ac"	"00074f2268"	0.0665
"00074be0ac"	"00074f28be"	0.1821
"00074be0ac"	"00074f294b"	0.002
"00074be0ac"	"00074f2ddd"	0.3195
"00074be0ac"	"00074f2e75"	−0.2188
"00074be0ac"	"00074f3088"	−0.0307
"00074be0ac"	"00074f5a1c"	0.0361
"00074be0ac"	"00074f75b7"	0.1022
"00074be0ac"	"00074f8cd9"	0.0649
"00074be0ac"	"00074f96dc"	−0.2328
"00074be0ac"	"00074fabaa"	−0.1182
"00074be0ac"	"00074facd9"	0.0171
"00074be0ac"	"00074fae3c"	−0.1157
"00074be0ac"	"00074fb0a8"	−0.1259
"00074be0ac"	"00074fb4e4"	−0.0416
"00074be0ac"	"00074fb7c2"	0.0784
"00074be0ac"	"00074fbd36"	0.0401
"00074be0ac"	"00074fc27f"	0.1364

"00074be0ac"	"00074fc31d"	0.1031
"00074be0ac"	"00074fd569"	-0.0493
"00074be0ac"	"00074fef15"	0.0082
"00074be0ac"	"00074ff562"	-0.3438
"00074be0ac"	"00075007ca"	0.1258
"00074be0ac"	"0007500b86"	-0.0044
"00074be0ac"	"0007500d05"	-0.1949
"00074be0ac"	"0007500ee4"	0.1176
"00074be0ac"	"0007500eee"	-0.1772
"00074be0ac"	"00075013dc"	-0.0119
"00074be0ac"	"000757b515"	0.4422
"00074be0ac"	"000757bc5a"	0.074
"00074be0ac"	"000757c320"	0.0682
"00074be0ac"	"000757c9aa"	0.0981
"00074be0ac"	"000757ccbe"	0.0361
"00074be0ac"	"000757cfa9"	0.0762
"00074be0ac"	"000757d390"	-0.0088
"00074be0ac"	"000757d393"	0.0916
"00074be0ac"	"000757d598"	0.1562
"00074be0ac"	"000757d5a2"	0.3759
"00074be0ac"	"000757d790"	0.0713
"00074be0ac"	"000757e30c"	0.0735
"00074be0ac"	"000757e4b0"	0.0728
"00074be0ac"	"000757e7a0"	0.1341
"00074be0ac"	"000757e8b3"	0.0155
"00074be0ac"	"000757f627"	0.1829
"00074be0ac"	"000757f925"	0.1694
"00074be0ac"	"000757fa08"	-0.0649
"00074be0ac"	"000757fe52"	-0.1885
"00074be0ac"	"000758024a"	-0.1206
"00074be0ac"	"00075804bb"	0.2225
"00074be0ac"	"00075a0c04"	0.1327
"00074be0ac"	"00075a3110"	0.2307
"00074be0ac"	"00075a341a"	0.0835
"00074be0ac"	"00075a3dcf"	0.0058
"00074be0ac"	"00075a3e22"	0.2147
"00074be0ac"	"00075a48d8"	-0.1184
"00074be0ac"	"00075a5cfb"	-0.1605
"00074be0ac"	"00075a6151"	0.1285
"00074be0ac"	"00075a6708"	0.6102
"00074be0ac"	"00075a7319"	0.0202
"00074be0ac"	"00075a7723"	0.1161
"00074be0ac"	"00075a778b"	-0.178
"00074be0ac"	"00075a7b8e"	-0.171
"00074be0ac"	"00075a7c79"	0.5053
"00074be0ac"	"00075a81b6"	-0.1426
"00074be0ac"	"00075a82ac"	0.1096
"00074be0ac"	"00075a98e5"	-0.0485
"00074be0ac"	"00075b0d29"	-0.0252
"00074be0ac"	"00075b102a"	0.1782

"00074be0ac"	"00075b1074"	−0.0135
"00074be0ac"	"00075b135d"	0.0049
"00074be0ac"	"00075b138b"	0.1108
"00074be0ac"	"00075b13a0"	−0.3057
"00074be0ac"	"00075b13bd"	−0.0777
"00074be0ac"	"00075b16a9"	−0.015
"00074be0ac"	"00075b1a28"	0.0087
"00074be0ac"	"00075b1a97"	0.0532
"00074be0ac"	"00075b1c7b"	−0.39
"00074be0ac"	"00075b1d24"	0.1083
"00074be0ac"	"00075b202b"	0.0371
"00074be0ac"	"00075b22cb"	−0.205
"00074be0ac"	"00075b22da"	0.0555
"00074be0ac"	"00075b2556"	0.086
"00074be0ac"	"00075b25de"	0.1213
"00074be0ac"	"00075b260c"	0.2608
"00074be0ac"	"00075b26f1"	−0.1233
"00074be0ac"	"00075b2920"	0.1101
"00074be0ac"	"00075b2a64"	0.0208
"00074be0ac"	"00075b2a9d"	0.1549
"00074be0ac"	"00075b2b37"	−0.16
"00074be0ac"	"00075b2cdd"	−0.1499
"00074be0ac"	"00075b3038"	0.1441
"00074be0ac"	"00075b30fe"	0.0745
"00074be0ac"	"00075b3362"	−0.2287
"00074be0ac"	"00075b350a"	0.1561
"00074be0ac"	"00075b350e"	−0.0832
"00074be0ac"	"00075b3651"	−0.057
"00074be0ac"	"00075b38ca"	−0.1367
"00074be0ac"	"00075b39cc"	0.0084
"00074be0ac"	"00075b3e1e"	0.1131
"00074be0ac"	"00075b3e57"	0.0338
"00074be0ac"	"00075b4079"	−0.0678
"00074be0ac"	"00075b4150"	−0.0943
"00074be0ac"	"00075b4194"	0.0943
"00074be0ac"	"00075b42d5"	−0.0055
"00074be0ac"	"00075b4424"	−0.0897
"00074be0ac"	"00075b4470"	0.1434
"00074be0ac"	"00075b47ed"	−0.0932
"00074be0ac"	"00075b4850"	0.3044
"00074be0ac"	"00075b4ca0"	0.1159
"00074be0ac"	"00075b4d7f"	−0.0738
"00074be0ac"	"00075b520f"	−0.0023
"00074be0ac"	"00075b525f"	−0.1113
"00074be0ac"	"00075b58f8"	−0.1767
"00074be0ac"	"00075b5bcc"	0.0649
"00074be0ac"	"00075b5bfa"	−0.1845
"00074be0ac"	"00075b6339"	0.2126
"00074be0ac"	"00075b6658"	−0.0692
"00074be0ac"	"00075b679a"	0.2788

"00074be0ac"	"00075b6cb7"	0.118
"00074be0ac"	"00075b6df8"	-0.0019
"00074be0ac"	"00075b6ff6"	0.243
"00074be0ac"	"00075b70ee"	0.0697
"00074be0ac"	"00075b7157"	-0.0953
"00074be0ac"	"00075b7225"	-0.0452
"00074be0ac"	"00075b7c89"	0.4478
"00074be0ac"	"00075b9048"	0.348
"00074be0ac"	"00075d0801"	-0.0957
"00074be0ac"	"00075d1820"	-0.151
"00074be0ac"	"00075d1f3d"	0.0878
"00074be0ac"	"00075d2329"	-0.0021
"00074be0ac"	"00075d2b9b"	0.0121
"00074be0ac"	"00075d3941"	-0.1117
"00074be0ac"	"00075d3e96"	-0.0423
"00074be0ac"	"00075d4864"	-0.2598
"00074be0ac"	"00075d5961"	0.0403
"00074be0ac"	"00075d5a63"	0.0922
"00074be0ac"	"00075d6150"	0.0056
"00074be0ac"	"00075d67d0"	0.0719
"00074be0ac"	"00075d67e2"	-0.09
"00074be0ac"	"00075d73fc"	0.0439
"00074be0ac"	"00075d7729"	-0.0139
"00074be0ac"	"00075d778c"	-0.2066
"00074be0ac"	"00075d7b9e"	-0.0155
"00074be0ac"	"00075d7c8f"	0.2338
"00074be0ac"	"00075d804d"	0.1316
"00074be0ac"	"00075d819f"	0.2472
"00074be0ac"	"00075d8601"	-0.0262
"00074be0ac"	"00075d8c6a"	-0.1006
"00074be0ac"	"00075dfedc"	0.1551
"00074be0ac"	"00075e05f2"	-0.1251
"00074be0ac"	"00075e0837"	0.0894
"00074be0ac"	"00075e092e"	0.0853
"00074be0ac"	"00075e0965"	-0.3862
"00074be0ac"	"00075e0bc8"	0.1056
"00074be0ac"	"00075e0fbb"	-0.1629
"00074bea69"	"00074bf097"	0.1044
"00074bea69"	"00074bf36d"	0.2538
"00074bea69"	"00074bfc0b"	-0.0067
"00074bea69"	"00074c0118"	-0.0542
"00074bea69"	"00074c10b5"	0.0955
"00074bea69"	"00074c1ca9"	0.0188
"00074bea69"	"00074c2119"	0.2658
"00074bea69"	"00074c3272"	-0.1021
"00074bea69"	"00074c33e4"	0.0684
"00074bea69"	"00074c35b2"	0.0265
"00074bea69"	"00074c38cd"	-0.0294
"00074bea69"	"00074c3a40"	-0.1306
"00074bea69"	"00074d84fc"	-0.2751

"00074bea69"	"00074d8639"	0.2568
"00074bea69"	"00074d8697"	-0.033
"00074bea69"	"00074d87af"	-0.0319
"00074bea69"	"00074d8813"	-0.2792
"00074bea69"	"00074d8817"	0.036
"00074bea69"	"00074d8a39"	-0.0478
"00074bea69"	"00074d8ad8"	0.0446
"00074bea69"	"00074d8c26"	-0.1259
"00074bea69"	"00074d8ca5"	-0.3243
"00074bea69"	"00074d8e0a"	-0.2443
"00074bea69"	"00074d9179"	0.0493
"00074bea69"	"00074d929f"	0.3329
"00074bea69"	"00074d93c4"	-0.333
"00074bea69"	"00074d93d0"	0.345
"00074bea69"	"00074d945b"	-0.0946
"00074bea69"	"00074d966c"	0.0022
"00074bea69"	"00074d9953"	-0.098
"00074bea69"	"00074d99b2"	0.1077
"00074bea69"	"00074d99f3"	0.26
"00074bea69"	"00074d9afd"	0.0969
"00074bea69"	"00074d9c87"	0.2509
"00074bea69"	"00074d9e9e"	-0.2941
"00074bea69"	"00074d9f30"	-0.1025
"00074bea69"	"00074da036"	0.1257
"00074bea69"	"00074da082"	-0.0014
"00074bea69"	"00074da136"	0.0231
"00074bea69"	"00074da3ed"	-0.0742
"00074bea69"	"00074da4ac"	-0.1529
"00074bea69"	"00074da4b8"	0.0923
"00074bea69"	"00074da5e8"	0.2335
"00074bea69"	"00074da6b4"	0.0313
"00074bea69"	"00074daa3c"	-0.3409
"00074bea69"	"00074daaf6"	-0.009
"00074bea69"	"00074dad20"	-0.0366
"00074bea69"	"00074db098"	0.016
"00074bea69"	"00074db231"	-0.076
"00074bea69"	"00074db3a3"	0.0762
"00074bea69"	"00074db5d6"	0.2738
"00074bea69"	"00074db632"	0.0028
"00074bea69"	"00074db688"	0.2988
"00074bea69"	"00074db8a6"	-0.1485
"00074bea69"	"00074dba19"	-0.2247
"00074bea69"	"00074dbc2e"	-0.0464
"00074bea69"	"00074dbe51"	0.0903
"00074bea69"	"00074dbe5f"	-0.5111
"00074bea69"	"00074dbf6d"	-0.3092
"00074bea69"	"00074dc4a5"	0.1207
"00074bea69"	"00074dc50c"	-0.1559
"00074bea69"	"00074dcdfa"	-0.1475
"00074bea69"	"00074dcf5f"	0.0936

"00074bea69"	"00074dd007"	−0.2183
"00074bea69"	"00074dd163"	−0.1012
"00074bea69"	"00074dd3df"	−0.2539
"00074bea69"	"00074dd577"	−0.0604
"00074bea69"	"00074dd62e"	0.0944
"00074bea69"	"00074dd73c"	−0.1602
"00074bea69"	"00074dda10"	−0.0034
"00074bea69"	"00074ddab8"	−0.3783
"00074bea69"	"00074ddd3d"	0.0105
"00074bea69"	"00074ddf16"	−0.2907
"00074bea69"	"00074ddfc1"	−0.1897
"00074bea69"	"00074de21a"	0.096
"00074bea69"	"00074de2a9"	0.0343
"00074bea69"	"00074de544"	0.1745
"00074bea69"	"00074de98a"	−0.0363
"00074bea69"	"00074dea7e"	−0.2667
"00074bea69"	"00074debd9"	−0.47
"00074bea69"	"00074deca3"	−0.0727
"00074bea69"	"00074def43"	−0.1118
"00074bea69"	"00074def99"	0.0315
"00074bea69"	"00074ecdad"	−0.0994
"00074bea69"	"00074ecf28"	0.2293
"00074bea69"	"00074ed1e1"	−0.1677
"00074bea69"	"00074ed83b"	0.0207
"00074bea69"	"00074ee5e3"	−0.2522
"00074bea69"	"00074ee6e0"	−0.0232
"00074bea69"	"00074eea3a"	−0.1288
"00074bea69"	"00074eff82"	−0.1194
"00074bea69"	"00074f0477"	−0.3948
"00074bea69"	"00074f08c3"	0.0037
"00074bea69"	"00074f1859"	−0.1177
"00074bea69"	"00074f2268"	−0.4357
"00074bea69"	"00074f28be"	−0.0882
"00074bea69"	"00074f294b"	0.1776
"00074bea69"	"00074f2ddd"	−0.0474
"00074bea69"	"00074f2e75"	−0.2524
"00074bea69"	"00074f3088"	0.1567
"00074bea69"	"00074f5a1c"	−0.0875
"00074bea69"	"00074f75b7"	−0.1075
"00074bea69"	"00074f8cd9"	−0.0659
"00074bea69"	"00074f96dc"	0.0448
"00074bea69"	"00074fabaa"	−0.2689
"00074bea69"	"00074facd9"	−0.272
"00074bea69"	"00074fae3c"	0.1646
"00074bea69"	"00074fb0a8"	0.1763
"00074bea69"	"00074fb4e4"	0.1119
"00074bea69"	"00074fb7c2"	0.2867
"00074bea69"	"00074fbd36"	−0.0116
"00074bea69"	"00074fc27f"	−0.1677
"00074bea69"	"00074fc31d"	0.371

"00074bea69"	"00074fd569"	0.1752
"00074bea69"	"00074fef15"	-0.045
"00074bea69"	"00074ff562"	-0.2839
"00074bea69"	"00075007ca"	-0.1716
"00074bea69"	"0007500b86"	-0.1875
"00074bea69"	"0007500d05"	-0.2555
"00074bea69"	"0007500ee4"	0.1965
"00074bea69"	"0007500eee"	-0.6049
"00074bea69"	"00075013dc"	-0.2668
"00074bea69"	"000757b515"	-0.0536
"00074bea69"	"000757bc5a"	-0.1923
"00074bea69"	"000757c320"	0.0064
"00074bea69"	"000757c9aa"	-0.0368
"00074bea69"	"000757ccbe"	0.0888
"00074bea69"	"000757cfa9"	0.2737
"00074bea69"	"000757d390"	0.092
"00074bea69"	"000757d393"	0.0657
"00074bea69"	"000757d598"	-0.2091
"00074bea69"	"000757d5a2"	-0.1787
"00074bea69"	"000757d790"	0.042
"00074bea69"	"000757e30c"	-0.1781
"00074bea69"	"000757e4b0"	-0.1497
"00074bea69"	"000757e7a0"	-0.0032
"00074bea69"	"000757e8b3"	-0.0466
"00074bea69"	"000757f627"	-0.0571
"00074bea69"	"000757f925"	-0.0673
"00074bea69"	"000757fa08"	-0.0664
"00074bea69"	"000757fe52"	-0.0248
"00074bea69"	"000758024a"	-0.0015
"00074bea69"	"00075804bb"	-0.2548
"00074bea69"	"00075a0c04"	0.0176
"00074bea69"	"00075a3110"	-0.0493
"00074bea69"	"00075a341a"	0.0296
"00074bea69"	"00075a3dcf"	-0.0607
"00074bea69"	"00075a3e22"	-0.0881
"00074bea69"	"00075a48d8"	-0.2351
"00074bea69"	"00075a5cfb"	-0.2726
"00074bea69"	"00075a6151"	-0.2336
"00074bea69"	"00075a6708"	0.0527
"00074bea69"	"00075a7319"	0.192
"00074bea69"	"00075a7723"	0.0252
"00074bea69"	"00075a778b"	0.0565
"00074bea69"	"00075a7b8e"	-0.1059
"00074bea69"	"00075a7c79"	-0.0626
"00074bea69"	"00075a81b6"	-0.2131
"00074bea69"	"00075a82ac"	-0.0446
"00074bea69"	"00075a98e5"	0.0764
"00074bea69"	"00075b0d29"	0.2587
"00074bea69"	"00075b102a"	-0.4126
"00074bea69"	"00075b1074"	-0.14

"00074bea69"	"00075b135d"	0.2143
"00074bea69"	"00075b138b"	-0.15
"00074bea69"	"00075b13a0"	-0.343
"00074bea69"	"00075b13bd"	0.1131
"00074bea69"	"00075b16a9"	-0.0346
"00074bea69"	"00075b1a28"	0.1145
"00074bea69"	"00075b1a97"	0.1508
"00074bea69"	"00075b1c7b"	-0.0521
"00074bea69"	"00075b1d24"	0.0513
"00074bea69"	"00075b202b"	-0.02
"00074bea69"	"00075b22cb"	-0.0206
"00074bea69"	"00075b22da"	-0.3139
"00074bea69"	"00075b2556"	-0.1641
"00074bea69"	"00075b25de"	-0.0021
"00074bea69"	"00075b260c"	-0.234
"00074bea69"	"00075b26f1"	-0.1782
"00074bea69"	"00075b2920"	-0.1428
"00074bea69"	"00075b2a64"	-0.33
"00074bea69"	"00075b2a9d"	-0.2648
"00074bea69"	"00075b2b37"	-0.0583
"00074bea69"	"00075b2cdd"	0.0575
"00074bea69"	"00075b3038"	-0.0407
"00074bea69"	"00075b30fe"	-0.2115
"00074bea69"	"00075b3362"	0.0416
"00074bea69"	"00075b350a"	-0.1017
"00074bea69"	"00075b350e"	-0.0553
"00074bea69"	"00075b3651"	0.0308
"00074bea69"	"00075b38ca"	-0.1038
"00074bea69"	"00075b39cc"	0.1106
"00074bea69"	"00075b3e1e"	-0.0677
"00074bea69"	"00075b3e57"	0.1357
"00074bea69"	"00075b4079"	-0.0666
"00074bea69"	"00075b4150"	0.0348
"00074bea69"	"00075b4194"	-0.1539
"00074bea69"	"00075b42d5"	-0.1143
"00074bea69"	"00075b4424"	0.0598
"00074bea69"	"00075b4470"	-0.1923
"00074bea69"	"00075b47ed"	0.0468
"00074bea69"	"00075b4850"	-0.2454
"00074bea69"	"00075b4ca0"	-0.0086
"00074bea69"	"00075b4d7f"	0.0686
"00074bea69"	"00075b520f"	0.1857
"00074bea69"	"00075b525f"	0.1315
"00074bea69"	"00075b58f8"	-0.1811
"00074bea69"	"00075b5bcc"	-0.0851
"00074bea69"	"00075b5bfa"	-0.4189
"00074bea69"	"00075b6339"	0.1982
"00074bea69"	"00075b6658"	0.0864
"00074bea69"	"00075b679a"	0.2649
"00074bea69"	"00075b6cb7"	-0.5977

"00074bea69"	"00075b6df8"	-0.0028
"00074bea69"	"00075b6ff6"	-0.1984
"00074bea69"	"00075b70ee"	-0.1338
"00074bea69"	"00075b7157"	-0.0235
"00074bea69"	"00075b7225"	-0.0587
"00074bea69"	"00075b7c89"	0.192
"00074bea69"	"00075b9048"	0.0243
"00074bea69"	"00075d0801"	0.0433
"00074bea69"	"00075d1820"	0.1343
"00074bea69"	"00075d1f3d"	-0.2165
"00074bea69"	"00075d2329"	0.0241
"00074bea69"	"00075d2b9b"	-0.2877
"00074bea69"	"00075d3941"	-0.0411
"00074bea69"	"00075d3e96"	0.0483
"00074bea69"	"00075d4864"	-0.0803
"00074bea69"	"00075d5961"	0.2175
"00074bea69"	"00075d5a63"	0.0088
"00074bea69"	"00075d6150"	-0.0104
"00074bea69"	"00075d67d0"	-0.0715
"00074bea69"	"00075d67e2"	0.05
"00074bea69"	"00075d73fc"	0.0206
"00074bea69"	"00075d7729"	0.0276
"00074bea69"	"00075d778c"	-0.173
"00074bea69"	"00075d7b9e"	0.1259
"00074bea69"	"00075d7c8f"	0.1062
"00074bea69"	"00075d804d"	-0.2399
"00074bea69"	"00075d819f"	-0.2603
"00074bea69"	"00075d8601"	0.1926
"00074bea69"	"00075d8c6a"	0.0174
"00074bea69"	"00075dfedc"	0.0867
"00074bea69"	"00075e05f2"	-0.3411
"00074bea69"	"00075e0837"	-0.2909
"00074bea69"	"00075e092e"	-0.0819
"00074bea69"	"00075e0965"	-0.1812
"00074bea69"	"00075e0bc8"	-0.1006
"00074bea69"	"00075e0fbb"	-0.1338
"00074bf097"	"00074bf36d"	0.2505
"00074bf097"	"00074bfc0b"	-0.198
"00074bf097"	"00074c0118"	0.0844
"00074bf097"	"00074c10b5"	0.0249
"00074bf097"	"00074c1ca9"	-0.0549
"00074bf097"	"00074c2119"	0.2496
"00074bf097"	"00074c3272"	0.0974
"00074bf097"	"00074c33e4"	0.1476
"00074bf097"	"00074c35b2"	0.0569
"00074bf097"	"00074c38cd"	0.4239
"00074bf097"	"00074c3a40"	-0.0218
"00074bf097"	"00074d84fc"	0.2338
"00074bf097"	"00074d8639"	0.2892
"00074bf097"	"00074d8697"	0.1082

"00074bf097"	"00074d87af"	0.3038
"00074bf097"	"00074d8813"	-0.1144
"00074bf097"	"00074d8817"	0.0926
"00074bf097"	"00074d8a39"	-0.106
"00074bf097"	"00074d8ad8"	0.3191
"00074bf097"	"00074d8c26"	0.1977
"00074bf097"	"00074d8ca5"	0.1821
"00074bf097"	"00074d8e0a"	0.2232
"00074bf097"	"00074d9179"	0.0659
"00074bf097"	"00074d929f"	0.239
"00074bf097"	"00074d93c4"	0.3773
"00074bf097"	"00074d93d0"	0.1084
"00074bf097"	"00074d945b"	0.0612
"00074bf097"	"00074d966c"	-0.1525
"00074bf097"	"00074d9953"	0.2464
"00074bf097"	"00074d99b2"	0.0167
"00074bf097"	"00074d99f3"	0.3755
"00074bf097"	"00074d9afd"	-0.0203
"00074bf097"	"00074d9c87"	0.1714
"00074bf097"	"00074d9e9e"	0.0576
"00074bf097"	"00074d9f30"	-0.0625
"00074bf097"	"00074da036"	0.2883
"00074bf097"	"00074da082"	0.0574
"00074bf097"	"00074da136"	0.2046
"00074bf097"	"00074da3ed"	0.458
"00074bf097"	"00074da4ac"	0.5944
"00074bf097"	"00074da4b8"	0.0665
"00074bf097"	"00074da5e8"	0.1759
"00074bf097"	"00074da6b4"	0.166
"00074bf097"	"00074daa3c"	0.0257
"00074bf097"	"00074daaf6"	-0.0635
"00074bf097"	"00074dad20"	-0.0254
"00074bf097"	"00074db098"	-0.0142
"00074bf097"	"00074db231"	0.2212
"00074bf097"	"00074db3a3"	0.2637
"00074bf097"	"00074db5d6"	-0.1848
"00074bf097"	"00074db632"	0.3996
"00074bf097"	"00074db688"	0.1264
"00074bf097"	"00074db8a6"	0.06
"00074bf097"	"00074dba19"	0.0067
"00074bf097"	"00074dbc2e"	0.1252
"00074bf097"	"00074dbe51"	0.3227
"00074bf097"	"00074dbe5f"	-0.0806
"00074bf097"	"00074dbf6d"	-0.1788
"00074bf097"	"00074dc4a5"	-0.0631
"00074bf097"	"00074dc50c"	0.1325
"00074bf097"	"00074dcdfa"	0.0283
"00074bf097"	"00074dcf5f"	0.0224
"00074bf097"	"00074dd007"	-0.2019
"00074bf097"	"00074dd163"	0.0969

"00074bf097"	"00074dd3df"	-0.113
"00074bf097"	"00074dd577"	0.0651
"00074bf097"	"00074dd62e"	0.0571
"00074bf097"	"00074dd73c"	0.14
"00074bf097"	"00074dda10"	0.1363
"00074bf097"	"00074ddab8"	0.0278
"00074bf097"	"00074ddd3d"	0.0792
"00074bf097"	"00074ddf16"	0.3807
"00074bf097"	"00074ddfc1"	0.3844
"00074bf097"	"00074de21a"	-0.0233
"00074bf097"	"00074de2a9"	0.0588
"00074bf097"	"00074de544"	0.2468
"00074bf097"	"00074de98a"	0.2969
"00074bf097"	"00074dea7e"	0.348
"00074bf097"	"00074debd9"	-0.1527
"00074bf097"	"00074deca3"	0.0246
"00074bf097"	"00074def43"	0.2123
"00074bf097"	"00074def99"	0.1791
"00074bf097"	"00074ecdad"	-0.1445
"00074bf097"	"00074ecf28"	-0.2055
"00074bf097"	"00074ed1e1"	0.3477
"00074bf097"	"00074ed83b"	0.417
"00074bf097"	"00074ee5e3"	-0.0498
"00074bf097"	"00074ee6e0"	-0.1093
"00074bf097"	"00074eea3a"	0.3131
"00074bf097"	"00074eff82"	-0.0093
"00074bf097"	"00074f0477"	-0.2491
"00074bf097"	"00074f08c3"	-0.1756
"00074bf097"	"00074f1859"	0.1409
"00074bf097"	"00074f2268"	-0.2843
"00074bf097"	"00074f28be"	0.3331
"00074bf097"	"00074f294b"	0.2432
"00074bf097"	"00074f2ddd"	0.0761
"00074bf097"	"00074f2e75"	0.1908
"00074bf097"	"00074f3088"	0.2028
"00074bf097"	"00074f5a1c"	0.0681
"00074bf097"	"00074f75b7"	0.191
"00074bf097"	"00074f8cd9"	0.4702
"00074bf097"	"00074f96dc"	-0.1691
"00074bf097"	"00074fabaa"	0.1304
"00074bf097"	"00074facd9"	0.0658
"00074bf097"	"00074fae3c"	0.0523
"00074bf097"	"00074fb0a8"	0.1398
"00074bf097"	"00074fb4e4"	0.1018
"00074bf097"	"00074fb7c2"	0.4814
"00074bf097"	"00074fbd36"	-0.1133
"00074bf097"	"00074fc27f"	0.082
"00074bf097"	"00074fc31d"	0.2941
"00074bf097"	"00074fd569"	0.1646
"00074bf097"	"00074fef15"	-0.0638

"00074bf097"	"00074ff562"	-0.1173
"00074bf097"	"00075007ca"	0.0025
"00074bf097"	"0007500b86"	0.2456
"00074bf097"	"0007500d05"	0.2906
"00074bf097"	"0007500ee4"	0.1829
"00074bf097"	"0007500eee"	-0.0744
"00074bf097"	"00075013dc"	-0.2261
"00074bf097"	"000757b515"	0.2133
"00074bf097"	"000757bc5a"	0.2202
"00074bf097"	"000757c320"	-0.1022
"00074bf097"	"000757c9aa"	0.0257
"00074bf097"	"000757ccbe"	0.2203
"00074bf097"	"000757cfa9"	-0.1321
"00074bf097"	"000757d390"	-0.0178
"00074bf097"	"000757d393"	0.2781
"00074bf097"	"000757d598"	-0.1118
"00074bf097"	"000757d5a2"	0.0177
"00074bf097"	"000757d790"	0.1239
"00074bf097"	"000757e30c"	0.0535
"00074bf097"	"000757e4b0"	0.1731
"00074bf097"	"000757e7a0"	0.0384
"00074bf097"	"000757e8b3"	0.2986
"00074bf097"	"000757f627"	0.1696
"00074bf097"	"000757f925"	0.3797
"00074bf097"	"000757fa08"	0.2589
"00074bf097"	"000757fe52"	0.0662
"00074bf097"	"000758024a"	0.0557
"00074bf097"	"00075804bb"	0.0675
"00074bf097"	"00075a0c04"	0.3465
"00074bf097"	"00075a3110"	0.0657
"00074bf097"	"00075a341a"	0.2114
"00074bf097"	"00075a3dcf"	0.097
"00074bf097"	"00075a3e22"	0.2704
"00074bf097"	"00075a48d8"	0.2965
"00074bf097"	"00075a5cfb"	-0.0231
"00074bf097"	"00075a6151"	0.1627
"00074bf097"	"00075a6708"	-0.003
"00074bf097"	"00075a7319"	0.0142
"00074bf097"	"00075a7723"	0.5598
"00074bf097"	"00075a778b"	0.1085
"00074bf097"	"00075a7b8e"	0.3538
"00074bf097"	"00075a7c79"	0.1657
"00074bf097"	"00075a81b6"	0.1226
"00074bf097"	"00075a82ac"	0.3799
"00074bf097"	"00075a98e5"	0.1441
"00074bf097"	"00075b0d29"	0.1887
"00074bf097"	"00075b102a"	0.193
"00074bf097"	"00075b1074"	0.1436
"00074bf097"	"00075b135d"	0.2051
"00074bf097"	"00075b138b"	0.2736

"00074bf097"	"00075b13a0"	-0.2223
"00074bf097"	"00075b13bd"	0.0578
"00074bf097"	"00075b16a9"	0.3161
"00074bf097"	"00075b1a28"	0.0944
"00074bf097"	"00075b1a97"	0.0862
"00074bf097"	"00075b1c7b"	-0.0049
"00074bf097"	"00075b1d24"	0.0401
"00074bf097"	"00075b202b"	-0.0342
"00074bf097"	"00075b22cb"	0.1946
"00074bf097"	"00075b22da"	0.1724
"00074bf097"	"00075b2556"	0.2232
"00074bf097"	"00075b25de"	0.1206
"00074bf097"	"00075b260c"	0.0234
"00074bf097"	"00075b26f1"	0.0735
"00074bf097"	"00075b2920"	0.0831
"00074bf097"	"00075b2a64"	-0.0959
"00074bf097"	"00075b2a9d"	-0.0275
"00074bf097"	"00075b2b37"	-0.3617
"00074bf097"	"00075b2cdd"	0.1384
"00074bf097"	"00075b3038"	0.3256
"00074bf097"	"00075b30fe"	0.1159
"00074bf097"	"00075b3362"	-0.0507
"00074bf097"	"00075b350a"	-0.1303
"00074bf097"	"00075b350e"	0.1123
"00074bf097"	"00075b3651"	0.1649
"00074bf097"	"00075b38ca"	0.1104
"00074bf097"	"00075b39cc"	0.2093
"00074bf097"	"00075b3e1e"	0.2256
"00074bf097"	"00075b3e57"	-0.0751
"00074bf097"	"00075b4079"	0.0295
"00074bf097"	"00075b4150"	0.0097
"00074bf097"	"00075b4194"	0.2925
"00074bf097"	"00075b42d5"	0.2943
"00074bf097"	"00075b4424"	-0.1053
"00074bf097"	"00075b4470"	0.2202
"00074bf097"	"00075b47ed"	0.27
"00074bf097"	"00075b4850"	0.1251
"00074bf097"	"00075b4ca0"	0.0211
"00074bf097"	"00075b4d7f"	0.506
"00074bf097"	"00075b520f"	-0.084
"00074bf097"	"00075b525f"	0.1254
"00074bf097"	"00075b58f8"	-0.0141
"00074bf097"	"00075b5bcc"	0.1135
"00074bf097"	"00075b5bfa"	-0.0916
"00074bf097"	"00075b6339"	0.2279
"00074bf097"	"00075b6658"	-0.2643
"00074bf097"	"00075b679a"	0.1191
"00074bf097"	"00075b6cb7"	-0.0534
"00074bf097"	"00075b6df8"	0.4595
"00074bf097"	"00075b6ff6"	-0.0162

"00074bf097"	"00075b70ee"	0.2784
"00074bf097"	"00075b7157"	0.2039
"00074bf097"	"00075b7225"	0.1136
"00074bf097"	"00075b7c89"	0.1356
"00074bf097"	"00075b9048"	0.239
"00074bf097"	"00075d0801"	0.3329
"00074bf097"	"00075d1820"	0.0854
"00074bf097"	"00075d1f3d"	0.047
"00074bf097"	"00075d2329"	0.0574
"00074bf097"	"00075d2b9b"	-0.107
"00074bf097"	"00075d3941"	-0.107
"00074bf097"	"00075d3e96"	0.2168
"00074bf097"	"00075d4864"	-0.0529
"00074bf097"	"00075d5961"	0.264
"00074bf097"	"00075d5a63"	-0.0918
"00074bf097"	"00075d6150"	0.0993
"00074bf097"	"00075d67d0"	-0.1628
"00074bf097"	"00075d67e2"	0.1395
"00074bf097"	"00075d73fc"	0.3234
"00074bf097"	"00075d7729"	-0.0971
"00074bf097"	"00075d778c"	-0.0432
"00074bf097"	"00075d7b9e"	0.2144
"00074bf097"	"00075d7c8f"	0.2691
"00074bf097"	"00075d804d"	-0.0319
"00074bf097"	"00075d819f"	0.0734
"00074bf097"	"00075d8601"	0.1771
"00074bf097"	"00075d8c6a"	0.6685
"00074bf097"	"00075dfedc"	0.2322
"00074bf097"	"00075e05f2"	0.1861
"00074bf097"	"00075e0837"	0.1012
"00074bf097"	"00075e092e"	0.2759
"00074bf097"	"00075e0965"	0.0401
"00074bf097"	"00075e0bc8"	0.0334
"00074bf097"	"00075e0fbb"	0.2184
"00074bf36d"	"00074bfc0b"	0.1253
"00074bf36d"	"00074c0118"	0.1027
"00074bf36d"	"00074c10b5"	0.0853
"00074bf36d"	"00074c1ca9"	0.1856
"00074bf36d"	"00074c2119"	0.1438
"00074bf36d"	"00074c3272"	0.3202
"00074bf36d"	"00074c33e4"	-0.0138
"00074bf36d"	"00074c35b2"	0.0725
"00074bf36d"	"00074c38cd"	-0.2281
"00074bf36d"	"00074c3a40"	0.3436
"00074bf36d"	"00074d84fc"	-0.0249
"00074bf36d"	"00074d8639"	-0.104
"00074bf36d"	"00074d8697"	0.1881
"00074bf36d"	"00074d87af"	0.206
"00074bf36d"	"00074d8813"	-0.2126
"00074bf36d"	"00074d8817"	0.1449

"00074bf36d"	"00074d8a39"	0.1476
"00074bf36d"	"00074d8ad8"	0.0752
"00074bf36d"	"00074d8c26"	-0.0885
"00074bf36d"	"00074d8ca5"	-0.0767
"00074bf36d"	"00074d8e0a"	-0.1257
"00074bf36d"	"00074d9179"	-0.2474
"00074bf36d"	"00074d929f"	0.2199
"00074bf36d"	"00074d93c4"	-0.1289
"00074bf36d"	"00074d93d0"	-0.138
"00074bf36d"	"00074d945b"	0.002
"00074bf36d"	"00074d966c"	-0.1686
"00074bf36d"	"00074d9953"	0.024
"00074bf36d"	"00074d99b2"	-0.0172
"00074bf36d"	"00074d99f3"	0.225
"00074bf36d"	"00074d9afd"	-0.2559
"00074bf36d"	"00074d9c87"	0.0936
"00074bf36d"	"00074d9e9e"	-0.332
"00074bf36d"	"00074d9f30"	-0.0089
"00074bf36d"	"00074da036"	0.0914
"00074bf36d"	"00074da082"	-0.2763
"00074bf36d"	"00074da136"	-0.0252
"00074bf36d"	"00074da3ed"	0.1672
"00074bf36d"	"00074da4ac"	0.2838
"00074bf36d"	"00074da4b8"	0.0537
"00074bf36d"	"00074da5e8"	0.2227
"00074bf36d"	"00074da6b4"	0.143
"00074bf36d"	"00074daa3c"	-0.2782
"00074bf36d"	"00074daaf6"	0.1259
"00074bf36d"	"00074dad20"	-0.1539
"00074bf36d"	"00074db098"	0.0607
"00074bf36d"	"00074db231"	-0.0655
"00074bf36d"	"00074db3a3"	0.0329
"00074bf36d"	"00074db5d6"	-9e-04
"00074bf36d"	"00074db632"	0.2762
"00074bf36d"	"00074db688"	0.3538
"00074bf36d"	"00074db8a6"	-0.0113
"00074bf36d"	"00074dba19"	-0.143
"00074bf36d"	"00074dbc2e"	-0.2083
"00074bf36d"	"00074dbe51"	0.0738
"00074bf36d"	"00074dbe5f"	-0.5153
"00074bf36d"	"00074dbf6d"	-0.1603
"00074bf36d"	"00074dc4a5"	-0.0285
"00074bf36d"	"00074dc50c"	-0.0275
"00074bf36d"	"00074dcdfa"	0.1216
"00074bf36d"	"00074dcf5f"	-0.1317
"00074bf36d"	"00074dd007"	-0.0888
"00074bf36d"	"00074dd163"	-0.0427
"00074bf36d"	"00074dd3df"	0.0902
"00074bf36d"	"00074dd577"	0.006
"00074bf36d"	"00074dd62e"	0.1123

"00074bf36d"	"00074dd73c"	−0.1405
"00074bf36d"	"00074dda10"	0.0401
"00074bf36d"	"00074ddab8"	0.1137
"00074bf36d"	"00074ddd3d"	−0.1088
"00074bf36d"	"00074ddf16"	−0.0336
"00074bf36d"	"00074ddfc1"	−0.0281
"00074bf36d"	"00074de21a"	0.5456
"00074bf36d"	"00074de2a9"	0.2637
"00074bf36d"	"00074de544"	0.1092
"00074bf36d"	"00074de98a"	−0.0121
"00074bf36d"	"00074dea7e"	0.2554
"00074bf36d"	"00074debd9"	−0.5743
"00074bf36d"	"00074deca3"	−0.1028
"00074bf36d"	"00074def43"	−0.0328
"00074bf36d"	"00074def99"	0.1667
"00074bf36d"	"00074ecdad"	0.0909
"00074bf36d"	"00074ecf28"	−0.1069
"00074bf36d"	"00074ed1e1"	0.1304
"00074bf36d"	"00074ed83b"	0.2993
"00074bf36d"	"00074ee5e3"	−0.2989
"00074bf36d"	"00074ee6e0"	0.1425
"00074bf36d"	"00074eea3a"	0.2911
"00074bf36d"	"00074eff82"	0.0773
"00074bf36d"	"00074f0477"	−0.4397
"00074bf36d"	"00074f08c3"	0.3451
"00074bf36d"	"00074f1859"	−0.0346
"00074bf36d"	"00074f2268"	−0.0733
"00074bf36d"	"00074f28be"	0.2482
"00074bf36d"	"00074f294b"	0.3093
"00074bf36d"	"00074f2ddd"	−0.1661
"00074bf36d"	"00074f2e75"	−0.2011
"00074bf36d"	"00074f3088"	0.0323
"00074bf36d"	"00074f5a1c"	0.0033
"00074bf36d"	"00074f75b7"	−0.2705
"00074bf36d"	"00074f8cd9"	−0.0255
"00074bf36d"	"00074f96dc"	−0.2077
"00074bf36d"	"00074fabaa"	0.0767
"00074bf36d"	"00074facd9"	0.0027
"00074bf36d"	"00074fae3c"	0.1008
"00074bf36d"	"00074fb0a8"	0.3968
"00074bf36d"	"00074fb4e4"	0.0612
"00074bf36d"	"00074fb7c2"	0.3151
"00074bf36d"	"00074fbd36"	−0.0866
"00074bf36d"	"00074fc27f"	0.0388
"00074bf36d"	"00074fc31d"	0.3142
"00074bf36d"	"00074fd569"	−0.0327
"00074bf36d"	"00074fef15"	0.078
"00074bf36d"	"00074ff562"	−0.1725
"00074bf36d"	"00075007ca"	−0.1386
"00074bf36d"	"0007500b86"	−0.0441

"00074bf36d"	"0007500d05"	-0.1188
"00074bf36d"	"0007500ee4"	0.1068
"00074bf36d"	"0007500eee"	-0.4114
"00074bf36d"	"00075013dc"	-0.0981
"00074bf36d"	"000757b515"	-0.0584
"00074bf36d"	"000757bc5a"	0.2044
"00074bf36d"	"000757c320"	-0.0016
"00074bf36d"	"000757c9aa"	0.1988
"00074bf36d"	"000757ccbe"	0.2214
"00074bf36d"	"000757cfa9"	-0.14
"00074bf36d"	"000757d390"	0.3704
"00074bf36d"	"000757d393"	-0.0589
"00074bf36d"	"000757d598"	-0.2085
"00074bf36d"	"000757d5a2"	-0.1833
"00074bf36d"	"000757d790"	0.0445
"00074bf36d"	"000757e30c"	-0.0237
"00074bf36d"	"000757e4b0"	0.3273
"00074bf36d"	"000757e7a0"	0.0494
"00074bf36d"	"000757e8b3"	0.0748
"00074bf36d"	"000757f627"	0.1952
"00074bf36d"	"000757f925"	-0.0457
"00074bf36d"	"000757fa08"	-0.2076
"00074bf36d"	"000757fe52"	-0.1158
"00074bf36d"	"000758024a"	-0.107
"00074bf36d"	"00075804bb"	-0.1479
"00074bf36d"	"00075a0c04"	0.0481
"00074bf36d"	"00075a3110"	0.056
"00074bf36d"	"00075a341a"	0.0617
"00074bf36d"	"00075a3dcf"	0.018
"00074bf36d"	"00075a3e22"	0.1515
"00074bf36d"	"00075a48d8"	0.026
"00074bf36d"	"00075a5cfb"	0.0102
"00074bf36d"	"00075a6151"	-0.1474
"00074bf36d"	"00075a6708"	-0.0831
"00074bf36d"	"00075a7319"	0.432
"00074bf36d"	"00075a7723"	0.1496
"00074bf36d"	"00075a778b"	0.0828
"00074bf36d"	"00075a7b8e"	0.4669
"00074bf36d"	"00075a7c79"	0.102
"00074bf36d"	"00075a81b6"	0.2086
"00074bf36d"	"00075a82ac"	0.1079
"00074bf36d"	"00075a98e5"	-0.0078
"00074bf36d"	"00075b0d29"	-0.1441
"00074bf36d"	"00075b102a"	-0.1976
"00074bf36d"	"00075b1074"	0.1412
"00074bf36d"	"00075b135d"	0.114
"00074bf36d"	"00075b138b"	0.3058
"00074bf36d"	"00075b13a0"	-0.176
"00074bf36d"	"00075b13bd"	0.2206
"00074bf36d"	"00075b16a9"	0.0275

"00074bf36d"	"00075b1a28"	−0.0094
"00074bf36d"	"00075b1a97"	0.4071
"00074bf36d"	"00075b1c7b"	−0.2606
"00074bf36d"	"00075b1d24"	0.1252
"00074bf36d"	"00075b202b"	0.0274
"00074bf36d"	"00075b22cb"	0.0444
"00074bf36d"	"00075b22da"	0.2269
"00074bf36d"	"00075b2556"	0.1582
"00074bf36d"	"00075b25de"	−0.2269
"00074bf36d"	"00075b260c"	−0.082
"00074bf36d"	"00075b26f1"	−0.1129
"00074bf36d"	"00075b2920"	−0.0361
"00074bf36d"	"00075b2a64"	−0.1296
"00074bf36d"	"00075b2a9d"	−0.0726
"00074bf36d"	"00075b2b37"	−0.1706
"00074bf36d"	"00075b2cdd"	0.1535
"00074bf36d"	"00075b3038"	−0.0577
"00074bf36d"	"00075b30fe"	0.0461
"00074bf36d"	"00075b3362"	−0.0734
"00074bf36d"	"00075b350a"	0.0953
"00074bf36d"	"00075b350e"	−0.0717
"00074bf36d"	"00075b3651"	−0.0815
"00074bf36d"	"00075b38ca"	0.0263
"00074bf36d"	"00075b39cc"	−0.0185
"00074bf36d"	"00075b3e1e"	0.0679
"00074bf36d"	"00075b3e57"	−0.1169
"00074bf36d"	"00075b4079"	−0.0505
"00074bf36d"	"00075b4150"	−0.1009
"00074bf36d"	"00075b4194"	0.2104
"00074bf36d"	"00075b42d5"	0.2125
"00074bf36d"	"00075b4424"	−0.1171
"00074bf36d"	"00075b4470"	0.1889
"00074bf36d"	"00075b47ed"	0.2006
"00074bf36d"	"00075b4850"	0.2521
"00074bf36d"	"00075b4ca0"	0.0315
"00074bf36d"	"00075b4d7f"	0.1493
"00074bf36d"	"00075b520f"	0.011
"00074bf36d"	"00075b525f"	0.3032
"00074bf36d"	"00075b58f8"	−0.1846
"00074bf36d"	"00075b5bcc"	0.2028
"00074bf36d"	"00075b5bfa"	−0.2568
"00074bf36d"	"00075b6339"	0.0794
"00074bf36d"	"00075b6658"	−0.2964
"00074bf36d"	"00075b679a"	0.135
"00074bf36d"	"00075b6cb7"	−0.1634
"00074bf36d"	"00075b6df8"	0.0366
"00074bf36d"	"00075b6ff6"	0.1617
"00074bf36d"	"00075b70ee"	−0.1534
"00074bf36d"	"00075b7157"	0.152
"00074bf36d"	"00075b7225"	0.0697

"00074bf36d"	"00075b7c89"	−0.1987
"00074bf36d"	"00075b9048"	0.0729
"00074bf36d"	"00075d0801"	−0.0204
"00074bf36d"	"00075d1820"	0.1005
"00074bf36d"	"00075d1f3d"	0.2387
"00074bf36d"	"00075d2329"	0.0173
"00074bf36d"	"00075d2b9b"	−0.1372
"00074bf36d"	"00075d3941"	−0.1475
"00074bf36d"	"00075d3e96"	0.1469
"00074bf36d"	"00075d4864"	0.1456
"00074bf36d"	"00075d5961"	−0.1372
"00074bf36d"	"00075d5a63"	−0.0805
"00074bf36d"	"00075d6150"	0.2401
"00074bf36d"	"00075d67d0"	−0.1124
"00074bf36d"	"00075d67e2"	0.1336
"00074bf36d"	"00075d73fc"	−0.0147
"00074bf36d"	"00075d7729"	0.0949
"00074bf36d"	"00075d778c"	−0.1026
"00074bf36d"	"00075d7b9e"	0.2265
"00074bf36d"	"00075d7c8f"	0.1479
"00074bf36d"	"00075d804d"	0.0834
"00074bf36d"	"00075d819f"	0.0547
"00074bf36d"	"00075d8601"	0.0231
"00074bf36d"	"00075d8c6a"	0.0034
"00074bf36d"	"00075dfedc"	−0.1248
"00074bf36d"	"00075e05f2"	−0.1243
"00074bf36d"	"00075e0837"	−0.0121
"00074bf36d"	"00075e092e"	0.239
"00074bf36d"	"00075e0965"	−0.0864
"00074bf36d"	"00075e0bc8"	0.052
"00074bf36d"	"00075e0fbb"	0.1634
"00074bfc0b"	"00074c0118"	−0.1308
"00074bfc0b"	"00074c10b5"	−0.2479
"00074bfc0b"	"00074c1ca9"	−0.3064
"00074bfc0b"	"00074c2119"	−0.1861
"00074bfc0b"	"00074c3272"	0.0926
"00074bfc0b"	"00074c33e4"	−0.098
"00074bfc0b"	"00074c35b2"	−0.0313
"00074bfc0b"	"00074c38cd"	−0.2447
"00074bfc0b"	"00074c3a40"	−0.2608
"00074bfc0b"	"00074d84fc"	−0.0099
"00074bfc0b"	"00074d8639"	−0.1661
"00074bfc0b"	"00074d8697"	0.0457
"00074bfc0b"	"00074d87af"	−0.1927
"00074bfc0b"	"00074d8813"	−0.1
"00074bfc0b"	"00074d8817"	−0.039
"00074bfc0b"	"00074d8a39"	−0.0631
"00074bfc0b"	"00074d8ad8"	0.1103
"00074bfc0b"	"00074d8c26"	−0.1256
"00074bfc0b"	"00074d8ca5"	−0.3277

"00074bfc0b"	"00074d8e0a"	-0.0978
"00074bfc0b"	"00074d9179"	-0.218
"00074bfc0b"	"00074d929f"	-0.2923
"00074bfc0b"	"00074d93c4"	-0.1421
"00074bfc0b"	"00074d93d0"	-0.1288
"00074bfc0b"	"00074d945b"	-0.1201
"00074bfc0b"	"00074d966c"	0.5648
"00074bfc0b"	"00074d9953"	-3e-04
"00074bfc0b"	"00074d99b2"	-0.1736
"00074bfc0b"	"00074d99f3"	-0.1883
"00074bfc0b"	"00074d9afd"	-0.2489
"00074bfc0b"	"00074d9c87"	0.0157
"00074bfc0b"	"00074d9e9e"	-0.5577
"00074bfc0b"	"00074d9f30"	-0.0075
"00074bfc0b"	"00074da036"	-0.0702
"00074bfc0b"	"00074da082"	-0.1703
"00074bfc0b"	"00074da136"	-0.398
"00074bfc0b"	"00074da3ed"	-0.3084
"00074bfc0b"	"00074da4ac"	-0.0172
"00074bfc0b"	"00074da4b8"	0.1027
"00074bfc0b"	"00074da5e8"	0.1384
"00074bfc0b"	"00074da6b4"	-0.2124
"00074bfc0b"	"00074daa3c"	-0.2445
"00074bfc0b"	"00074daaf6"	-0.0347
"00074bfc0b"	"00074dad20"	-0.3249
"00074bfc0b"	"00074db098"	0.1757
"00074bfc0b"	"00074db231"	-0.0843
"00074bfc0b"	"00074db3a3"	0.0503
"00074bfc0b"	"00074db5d6"	0.3193
"00074bfc0b"	"00074db632"	0.1313
"00074bfc0b"	"00074db688"	-0.0274
"00074bfc0b"	"00074db8a6"	-0.3571
"00074bfc0b"	"00074dba19"	0.0305
"00074bfc0b"	"00074dbc2e"	-0.2242
"00074bfc0b"	"00074dbe51"	-0.0588
"00074bfc0b"	"00074dbe5f"	-0.2668
"00074bfc0b"	"00074dbf6d"	0.2214
"00074bfc0b"	"00074dc4a5"	-0.2637
"00074bfc0b"	"00074dc50c"	-0.1471
"00074bfc0b"	"00074dcdfa"	-0.1
"00074bfc0b"	"00074dcf5f"	-0.3813
"00074bfc0b"	"00074dd007"	-0.2015
"00074bfc0b"	"00074dd163"	-0.2742
"00074bfc0b"	"00074dd3df"	-0.1856
"00074bfc0b"	"00074dd577"	-0.1673
"00074bfc0b"	"00074dd62e"	0.0883
"00074bfc0b"	"00074dd73c"	-0.1578
"00074bfc0b"	"00074dda10"	-0.0095
"00074bfc0b"	"00074ddab8"	0.1019
"00074bfc0b"	"00074ddd3d"	-0.2137

"00074bfc0b"	"00074ddf16"	-0.3898
"00074bfc0b"	"00074ddfc1"	-0.0033
"00074bfc0b"	"00074de21a"	-0.0451
"00074bfc0b"	"00074de2a9"	0.1378
"00074bfc0b"	"00074de544"	0.0289
"00074bfc0b"	"00074de98a"	-0.1161
"00074bfc0b"	"00074dea7e"	-0.3124
"00074bfc0b"	"00074debd9"	-0.3165
"00074bfc0b"	"00074deca3"	-0.419
"00074bfc0b"	"00074def43"	-0.1106
"00074bfc0b"	"00074def99"	-0.2939
"00074bfc0b"	"00074ecdad"	-0.3309
"00074bfc0b"	"00074ecf28"	-0.1202
"00074bfc0b"	"00074ed1e1"	-0.3203
"00074bfc0b"	"00074ed83b"	-0.225
"00074bfc0b"	"00074ee5e3"	-0.1994
"00074bfc0b"	"00074ee6e0"	0.0759
"00074bfc0b"	"00074eea3a"	-0.1545
"00074bfc0b"	"00074eff82"	-0.224
"00074bfc0b"	"00074f0477"	-0.3729
"00074bfc0b"	"00074f08c3"	-0.0429
"00074bfc0b"	"00074f1859"	-0.0552
"00074bfc0b"	"00074f2268"	0.4978
"00074bfc0b"	"00074f28be"	0.0834
"00074bfc0b"	"00074f294b"	-0.117
"00074bfc0b"	"00074f2ddd"	0.0603
"00074bfc0b"	"00074f2e75"	-0.1639
"00074bfc0b"	"00074f3088"	-0.0499
"00074bfc0b"	"00074f5a1c"	-0.1851
"00074bfc0b"	"00074f75b7"	-0.1495
"00074bfc0b"	"00074f8cd9"	-0.0871
"00074bfc0b"	"00074f96dc"	0.0764
"00074bfc0b"	"00074fabaa"	-0.0406
"00074bfc0b"	"00074facd9"	-0.0028
"00074bfc0b"	"00074fae3c"	-0.0546
"00074bfc0b"	"00074fb0a8"	-0.1243
"00074bfc0b"	"00074fb4e4"	-0.4339
"00074bfc0b"	"00074fb7c2"	-0.1612
"00074bfc0b"	"00074fbd36"	0.0558
"00074bfc0b"	"00074fc27f"	0.0292
"00074bfc0b"	"00074fc31d"	-0.2039
"00074bfc0b"	"00074fd569"	-0.3406
"00074bfc0b"	"00074fef15"	-0.2823
"00074bfc0b"	"00074ff562"	-0.3266
"00074bfc0b"	"00075007ca"	-0.0857
"00074bfc0b"	"0007500b86"	-0.0634
"00074bfc0b"	"0007500d05"	-0.1301
"00074bfc0b"	"0007500ee4"	-0.203
"00074bfc0b"	"0007500eee"	-0.1724
"00074bfc0b"	"00075013dc"	-0.1591

"00074bfc0b"	"000757b515"	−0.1555
"00074bfc0b"	"000757bc5a"	0.2476
"00074bfc0b"	"000757c320"	−0.0014
"00074bfc0b"	"000757c9aa"	−0.0112
"00074bfc0b"	"000757ccbe"	0.0629
"00074bfc0b"	"000757cfa9"	−0.0226
"00074bfc0b"	"000757d390"	−0.173
"00074bfc0b"	"000757d393"	−0.1127
"00074bfc0b"	"000757d598"	−0.0211
"00074bfc0b"	"000757d5a2"	−0.2175
"00074bfc0b"	"000757d790"	−0.3399
"00074bfc0b"	"000757e30c"	0.0226
"00074bfc0b"	"000757e4b0"	−0.0035
"00074bfc0b"	"000757e7a0"	−0.2403
"00074bfc0b"	"000757e8b3"	0.1387
"00074bfc0b"	"000757f627"	−0.1586
"00074bfc0b"	"000757f925"	−0.0505
"00074bfc0b"	"000757fa08"	−0.2512
"00074bfc0b"	"000757fe52"	−0.1743
"00074bfc0b"	"000758024a"	−0.0741
"00074bfc0b"	"00075804bb"	0.2111
"00074bfc0b"	"00075a0c04"	−0.0153
"00074bfc0b"	"00075a3110"	−0.0011
"00074bfc0b"	"00075a341a"	−0.0717
"00074bfc0b"	"00075a3dcf"	−0.4682
"00074bfc0b"	"00075a3e22"	0.1861
"00074bfc0b"	"00075a48d8"	0.0494
"00074bfc0b"	"00075a5cfb"	0.2541
"00074bfc0b"	"00075a6151"	−0.2748
"00074bfc0b"	"00075a6708"	−0.0761
"00074bfc0b"	"00075a7319"	−0.258
"00074bfc0b"	"00075a7723"	−0.1277
"00074bfc0b"	"00075a778b"	−0.3606
"00074bfc0b"	"00075a7b8e"	0.0105
"00074bfc0b"	"00075a7c79"	0.1793
"00074bfc0b"	"00075a81b6"	−0.1025
"00074bfc0b"	"00075a82ac"	−0.2184
"00074bfc0b"	"00075a98e5"	−0.099
"00074bfc0b"	"00075b0d29"	−0.0685
"00074bfc0b"	"00075b102a"	−0.0306
"00074bfc0b"	"00075b1074"	0.2558
"00074bfc0b"	"00075b135d"	0.0019
"00074bfc0b"	"00075b138b"	0.0744
"00074bfc0b"	"00075b13a0"	−0.4787
"00074bfc0b"	"00075b13bd"	0.0449
"00074bfc0b"	"00075b16a9"	−0.2683
"00074bfc0b"	"00075b1a28"	0.0199
"00074bfc0b"	"00075b1a97"	0.1105
"00074bfc0b"	"00075b1c7b"	−0.4247
"00074bfc0b"	"00075b1d24"	0.1538

"00074bfc0b"	"00075b202b"	0.2098
"00074bfc0b"	"00075b22cb"	-0.1379
"00074bfc0b"	"00075b22da"	0.1425
"00074bfc0b"	"00075b2556"	0.0644
"00074bfc0b"	"00075b25de"	-0.2076
"00074bfc0b"	"00075b260c"	-0.2486
"00074bfc0b"	"00075b26f1"	-0.0857
"00074bfc0b"	"00075b2920"	0.174
"00074bfc0b"	"00075b2a64"	0.1301
"00074bfc0b"	"00075b2a9d"	-0.087
"00074bfc0b"	"00075b2b37"	0.0917
"00074bfc0b"	"00075b2cdd"	-0.1537
"00074bfc0b"	"00075b3038"	-0.1325
"00074bfc0b"	"00075b30fe"	0.0107
"00074bfc0b"	"00075b3362"	-0.1075
"00074bfc0b"	"00075b350a"	0.6591
"00074bfc0b"	"00075b350e"	-0.151
"00074bfc0b"	"00075b3651"	-0.2043
"00074bfc0b"	"00075b38ca"	-0.0681
"00074bfc0b"	"00075b39cc"	-0.2717
"00074bfc0b"	"00075b3e1e"	0.1248
"00074bfc0b"	"00075b3e57"	0.0694
"00074bfc0b"	"00075b4079"	0.5439
"00074bfc0b"	"00075b4150"	-0.0467
"00074bfc0b"	"00075b4194"	-0.0111
"00074bfc0b"	"00075b42d5"	-0.1279
"00074bfc0b"	"00075b4424"	-0.1465
"00074bfc0b"	"00075b4470"	0.2321
"00074bfc0b"	"00075b47ed"	-0.3345
"00074bfc0b"	"00075b4850"	0.1712
"00074bfc0b"	"00075b4ca0"	0.005
"00074bfc0b"	"00075b4d7f"	0.2446
"00074bfc0b"	"00075b520f"	-0.1547
"00074bfc0b"	"00075b525f"	0.0095
"00074bfc0b"	"00075b58f8"	-0.113
"00074bfc0b"	"00075b5bcc"	0.4915
"00074bfc0b"	"00075b5bfa"	-0.4555
"00074bfc0b"	"00075b6339"	0.1511
"00074bfc0b"	"00075b6658"	-0.0919
"00074bfc0b"	"00075b679a"	-0.1266
"00074bfc0b"	"00075b6cb7"	0.0031
"00074bfc0b"	"00075b6df8"	-0.1093
"00074bfc0b"	"00075b6ff6"	0.1655
"00074bfc0b"	"00075b70ee"	-0.163
"00074bfc0b"	"00075b7157"	0.0892
"00074bfc0b"	"00075b7225"	-0.0831
"00074bfc0b"	"00075b7c89"	0.0032
"00074bfc0b"	"00075b9048"	-0.1543
"00074bfc0b"	"00075d0801"	-0.0869
"00074bfc0b"	"00075d1820"	-0.2614

"00074bfc0b"	"00075d1f3d"	0.1151
"00074bfc0b"	"00075d2329"	-0.2815
"00074bfc0b"	"00075d2b9b"	-0.0449
"00074bfc0b"	"00075d3941"	-0.0771
"00074bfc0b"	"00075d3e96"	-0.0688
"00074bfc0b"	"00075d4864"	-0.1328
"00074bfc0b"	"00075d5961"	-0.064
"00074bfc0b"	"00075d5a63"	0.1135
"00074bfc0b"	"00075d6150"	-0.1626
"00074bfc0b"	"00075d67d0"	-0.3798
"00074bfc0b"	"00075d67e2"	0.0539
"00074bfc0b"	"00075d73fc"	-0.3548
"00074bfc0b"	"00075d7729"	-0.0212
"00074bfc0b"	"00075d778c"	-0.2455
"00074bfc0b"	"00075d7b9e"	0.053
"00074bfc0b"	"00075d7c8f"	0.1103
"00074bfc0b"	"00075d804d"	0.056
"00074bfc0b"	"00075d819f"	-0.1057
"00074bfc0b"	"00075d8601"	-0.1243
"00074bfc0b"	"00075d8c6a"	-0.2007
"00074bfc0b"	"00075dfedc"	0.0836
"00074bfc0b"	"00075e05f2"	-0.2138
"00074bfc0b"	"00075e0837"	-0.0549
"00074bfc0b"	"00075e092e"	0.271
"00074bfc0b"	"00075e0965"	-0.0866
"00074bfc0b"	"00075e0bc8"	0.0463
"00074bfc0b"	"00075e0fbb"	0.1695
"00074c0118"	"00074c10b5"	-0.134
"00074c0118"	"00074c1ca9"	-0.2775
"00074c0118"	"00074c2119"	-0.3182
"00074c0118"	"00074c3272"	-0.0764
"00074c0118"	"00074c33e4"	-0.3247
"00074c0118"	"00074c35b2"	-0.1538
"00074c0118"	"00074c38cd"	-0.3368
"00074c0118"	"00074c3a40"	-0.0514
"00074c0118"	"00074d84fc"	-0.1587
"00074c0118"	"00074d8639"	-0.0698
"00074c0118"	"00074d8697"	-0.1541
"00074c0118"	"00074d87af"	-0.2896
"00074c0118"	"00074d8813"	-0.1393
"00074c0118"	"00074d8817"	-0.0669
"00074c0118"	"00074d8a39"	0.1377
"00074c0118"	"00074d8ad8"	-0.1906
"00074c0118"	"00074d8c26"	0.0924
"00074c0118"	"00074d8ca5"	-0.0757
"00074c0118"	"00074d8e0a"	-0.0342
"00074c0118"	"00074d9179"	-0.2324
"00074c0118"	"00074d929f"	-0.0228
"00074c0118"	"00074d93c4"	0.2326
"00074c0118"	"00074d93d0"	-0.1658

"00074c0118"	"00074d945b"	-0.0858
"00074c0118"	"00074d966c"	-0.3296
"00074c0118"	"00074d9953"	-0.0633
"00074c0118"	"00074d99b2"	-0.1783
"00074c0118"	"00074d99f3"	-0.0905
"00074c0118"	"00074d9afd"	-0.1272
"00074c0118"	"00074d9c87"	-0.1264
"00074c0118"	"00074d9e9e"	0.064
"00074c0118"	"00074d9f30"	-0.0795
"00074c0118"	"00074da036"	-0.1805
"00074c0118"	"00074da082"	-0.1762
"00074c0118"	"00074da136"	-0.19
"00074c0118"	"00074da3ed"	0.0083
"00074c0118"	"00074da4ac"	0.1522
"00074c0118"	"00074da4b8"	-0.103
"00074c0118"	"00074da5e8"	0.1349
"00074c0118"	"00074da6b4"	-0.2301
"00074c0118"	"00074daa3c"	0.0327
"00074c0118"	"00074daaf6"	0.1385
"00074c0118"	"00074dad20"	0.3313
"00074c0118"	"00074db098"	-6e-04
"00074c0118"	"00074db231"	-0.067
"00074c0118"	"00074db3a3"	-0.0056
"00074c0118"	"00074db5d6"	-0.101
"00074c0118"	"00074db632"	-0.0599
"00074c0118"	"00074db688"	-0.188
"00074c0118"	"00074db8a6"	0.3041
"00074c0118"	"00074dba19"	-0.2759
"00074c0118"	"00074dbc2e"	0.3137
"00074c0118"	"00074dbe51"	0.0519
"00074c0118"	"00074dbe5f"	-0.0855
"00074c0118"	"00074dbf6d"	-0.2743
"00074c0118"	"00074dc4a5"	0.0048
"00074c0118"	"00074dc50c"	-0.1781
"00074c0118"	"00074dcdfa"	0.1032
"00074c0118"	"00074dcf5f"	-0.4014
"00074c0118"	"00074dd007"	-0.0097
"00074c0118"	"00074dd163"	0.4547
"00074c0118"	"00074dd3df"	-0.0728
"00074c0118"	"00074dd577"	-0.1639
"00074c0118"	"00074dd62e"	0.0836
"00074c0118"	"00074dd73c"	0.1754
"00074c0118"	"00074dda10"	-0.3151
"00074c0118"	"00074ddab8"	-0.0759
"00074c0118"	"00074ddd3d"	-0.3241
"00074c0118"	"00074ddf16"	0.0491
"00074c0118"	"00074ddfc1"	0.1226
"00074c0118"	"00074de21a"	-0.3409
"00074c0118"	"00074de2a9"	-0.1686
"00074c0118"	"00074de544"	-0.1539

"00074c0118"	"00074de98a"	-0.2212
"00074c0118"	"00074dea7e"	0.0368
"00074c0118"	"00074debd9"	0.0752
"00074c0118"	"00074deca3"	0.4862
"00074c0118"	"00074def43"	0.3662
"00074c0118"	"00074def99"	-0.2791
"00074c0118"	"00074ecdad"	0.0543
"00074c0118"	"00074ecf28"	-0.1091
"00074c0118"	"00074ed1e1"	-0.0842
"00074c0118"	"00074ed83b"	-0.0533
"00074c0118"	"00074ee5e3"	0.082
"00074c0118"	"00074ee6e0"	0.1284
"00074c0118"	"00074eea3a"	0.0677
"00074c0118"	"00074eff82"	-0.0341
"00074c0118"	"00074f0477"	-0.1445
"00074c0118"	"00074f08c3"	-0.2065
"00074c0118"	"00074f1859"	0.0073
"00074c0118"	"00074f2268"	-0.2634
"00074c0118"	"00074f28be"	-0.0087
"00074c0118"	"00074f294b"	-0.0961
"00074c0118"	"00074f2ddd"	-0.043
"00074c0118"	"00074f2e75"	-0.3409
"00074c0118"	"00074f3088"	-0.3295
"00074c0118"	"00074f5a1c"	0.1988
"00074c0118"	"00074f75b7"	0.1484
"00074c0118"	"00074f8cd9"	0.2443
"00074c0118"	"00074f96dc"	-0.1763
"00074c0118"	"00074fabaa"	-0.036
"00074c0118"	"00074facd9"	0.0139
"00074c0118"	"00074fae3c"	-0.0642
"00074c0118"	"00074fb0a8"	0.0093
"00074c0118"	"00074fb4e4"	-0.2369
"00074c0118"	"00074fb7c2"	-0.1689
"00074c0118"	"00074fbd36"	-0.4691
"00074c0118"	"00074fc27f"	-0.0877
"00074c0118"	"00074fc31d"	-0.2661
"00074c0118"	"00074fd569"	-0.1086
"00074c0118"	"00074fef15"	-0.1811
"00074c0118"	"00074ff562"	-0.0753
"00074c0118"	"00075007ca"	-0.1443
"00074c0118"	"0007500b86"	-0.0223
"00074c0118"	"0007500d05"	0.2093
"00074c0118"	"0007500ee4"	-0.1455
"00074c0118"	"0007500eee"	-0.1792
"00074c0118"	"00075013dc"	-0.4012
"00074c0118"	"000757b515"	-0.2465
"00074c0118"	"000757bc5a"	-0.0842
"00074c0118"	"000757c320"	-0.1469
"00074c0118"	"000757c9aa"	-0.1087
"00074c0118"	"000757ccbe"	-0.0825

"00074c0118"	"000757cfa9"	−0.3496
"00074c0118"	"000757d390"	0.0763
"00074c0118"	"000757d393"	0.1685
"00074c0118"	"000757d598"	−0.1766
"00074c0118"	"000757d5a2"	−0.2723
"00074c0118"	"000757d790"	0.1016
"00074c0118"	"000757e30c"	−0.2951
"00074c0118"	"000757e4b0"	−0.2752
"00074c0118"	"000757e7a0"	−0.2201
"00074c0118"	"000757e8b3"	−0.0943
"00074c0118"	"000757f627"	−0.1317
"00074c0118"	"000757f925"	−0.0402
"00074c0118"	"000757fa08"	−0.0275
"00074c0118"	"000757fe52"	0.2314
"00074c0118"	"000758024a"	−0.1798
"00074c0118"	"00075804bb"	−0.3122
"00074c0118"	"00075a0c04"	0.0334
"00074c0118"	"00075a3110"	0.0466
"00074c0118"	"00075a341a"	−0.1683
"00074c0118"	"00075a3dcf"	0.3209
"00074c0118"	"00075a3e22"	−0.06
"00074c0118"	"00075a48d8"	0.1102
"00074c0118"	"00075a5cfb"	−0.0677
"00074c0118"	"00075a6151"	0.0213
"00074c0118"	"00075a6708"	−0.1179
"00074c0118"	"00075a7319"	0.0556
"00074c0118"	"00075a7723"	−0.0939
"00074c0118"	"00075a778b"	−0.2092
"00074c0118"	"00075a7b8e"	0.1293
"00074c0118"	"00075a7c79"	−0.0381
"00074c0118"	"00075a81b6"	−0.3718
"00074c0118"	"00075a82ac"	−0.2105
"00074c0118"	"00075a98e5"	−0.2969
"00074c0118"	"00075b0d29"	−0.3737
"00074c0118"	"00075b102a"	−0.1975
"00074c0118"	"00075b1074"	−0.0926
"00074c0118"	"00075b135d"	−0.2936
"00074c0118"	"00075b138b"	0.0277
"00074c0118"	"00075b13a0"	0.0602
"00074c0118"	"00075b13bd"	−0.1974
"00074c0118"	"00075b16a9"	−0.0965
"00074c0118"	"00075b1a28"	0.0355
"00074c0118"	"00075b1a97"	0.0415
"00074c0118"	"00075b1c7b"	−0.2797
"00074c0118"	"00075b1d24"	0.0287
"00074c0118"	"00075b202b"	0.0519
"00074c0118"	"00075b22cb"	0.089
"00074c0118"	"00075b22da"	0.033
"00074c0118"	"00075b2556"	−0.0917
"00074c0118"	"00075b25de"	0.0423

"00074c0118"	"00075b260c"	−0.0115
"00074c0118"	"00075b26f1"	−0.1911
"00074c0118"	"00075b2920"	−0.2446
"00074c0118"	"00075b2a64"	−0.2677
"00074c0118"	"00075b2a9d"	−0.1883
"00074c0118"	"00075b2b37"	−0.2917
"00074c0118"	"00075b2cdd"	0.1837
"00074c0118"	"00075b3038"	0.1593
"00074c0118"	"00075b30fe"	−0.2107
"00074c0118"	"00075b3362"	−0.0888
"00074c0118"	"00075b350a"	−0.0263
"00074c0118"	"00075b350e"	0.1113
"00074c0118"	"00075b3651"	−0.2112
"00074c0118"	"00075b38ca"	0.0991
"00074c0118"	"00075b39cc"	0.1282
"00074c0118"	"00075b3e1e"	0.0282
"00074c0118"	"00075b3e57"	−0.0416
"00074c0118"	"00075b4079"	−0.1891
"00074c0118"	"00075b4150"	−0.1036
"00074c0118"	"00075b4194"	0.1749
"00074c0118"	"00075b42d5"	0.1589
"00074c0118"	"00075b4424"	−0.473
"00074c0118"	"00075b4470"	−0.0997
"00074c0118"	"00075b47ed"	0.1872
"00074c0118"	"00075b4850"	−0.1929
"00074c0118"	"00075b4ca0"	−0.4082
"00074c0118"	"00075b4d7f"	0.0645
"00074c0118"	"00075b520f"	−0.0646
"00074c0118"	"00075b525f"	−0.0766
"00074c0118"	"00075b58f8"	−0.2561
"00074c0118"	"00075b5bcc"	0.0336
"00074c0118"	"00075b5bfa"	−0.0035
"00074c0118"	"00075b6339"	0.0442
"00074c0118"	"00075b6658"	−0.1852
"00074c0118"	"00075b679a"	0.1368
"00074c0118"	"00075b6cb7"	−0.515
"00074c0118"	"00075b6df8"	0.0668
"00074c0118"	"00075b6ff6"	−0.044
"00074c0118"	"00075b70ee"	−0.2279
"00074c0118"	"00075b7157"	0.1508
"00074c0118"	"00075b7225"	0.1787
"00074c0118"	"00075b7c89"	−0.135
"00074c0118"	"00075b9048"	0.0964
"00074c0118"	"00075d0801"	0.059
"00074c0118"	"00075d1820"	−0.1746
"00074c0118"	"00075d1f3d"	−0.1329
"00074c0118"	"00075d2329"	0.0689
"00074c0118"	"00075d2b9b"	−0.2182
"00074c0118"	"00075d3941"	−0.2395
"00074c0118"	"00075d3e96"	−0.2193

"00074c0118"	"00075d4864"	0.0135
"00074c0118"	"00075d5961"	-0.0459
"00074c0118"	"00075d5a63"	0.0209
"00074c0118"	"00075d6150"	0.2021
"00074c0118"	"00075d67d0"	-0.1927
"00074c0118"	"00075d67e2"	-0.1898
"00074c0118"	"00075d73fc"	-0.0532
"00074c0118"	"00075d7729"	-0.4151
"00074c0118"	"00075d778c"	-0.0661
"00074c0118"	"00075d7b9e"	-0.0065
"00074c0118"	"00075d7c8f"	0.0153
"00074c0118"	"00075d804d"	-0.0736
"00074c0118"	"00075d819f"	-0.1521
"00074c0118"	"00075d8601"	-0.0251
"00074c0118"	"00075d8c6a"	0.1028
"00074c0118"	"00075dfedc"	0.0412
"00074c0118"	"00075e05f2"	0.0917
"00074c0118"	"00075e0837"	-0.1098
"00074c0118"	"00075e092e"	0.1968
"00074c0118"	"00075e0965"	-0.2522
"00074c0118"	"00075e0bc8"	0.0421
"00074c0118"	"00075e0fbb"	-0.0458
"00074c10b5"	"00074c1ca9"	-0.2026
"00074c10b5"	"00074c2119"	-0.1128
"00074c10b5"	"00074c3272"	0.0112
"00074c10b5"	"00074c33e4"	0.021
"00074c10b5"	"00074c35b2"	0.0618
"00074c10b5"	"00074c38cd"	-0.1936
"00074c10b5"	"00074c3a40"	-0.3071
"00074c10b5"	"00074d84fc"	-0.3082
"00074c10b5"	"00074d8639"	0.0519
"00074c10b5"	"00074d8697"	-0.1851
"00074c10b5"	"00074d87af"	-0.0657
"00074c10b5"	"00074d8813"	-0.5321
"00074c10b5"	"00074d8817"	0.2568
"00074c10b5"	"00074d8a39"	0.049
"00074c10b5"	"00074d8ad8"	-0.1762
"00074c10b5"	"00074d8c26"	-0.1823
"00074c10b5"	"00074d8ca5"	-0.2068
"00074c10b5"	"00074d8e0a"	-0.0147
"00074c10b5"	"00074d9179"	0.2906
"00074c10b5"	"00074d929f"	0.4472
"00074c10b5"	"00074d93c4"	-0.17
"00074c10b5"	"00074d93d0"	0.4794
"00074c10b5"	"00074d945b"	0.0904
"00074c10b5"	"00074d966c"	-0.1514
"00074c10b5"	"00074d9953"	-0.0107
"00074c10b5"	"00074d99b2"	-0.1198
"00074c10b5"	"00074d99f3"	0.1005
"00074c10b5"	"00074d9afd"	-5e-04

"00074c10b5"	"00074d9c87"	0.0012
"00074c10b5"	"00074d9e9e"	−0.3895
"00074c10b5"	"00074d9f30"	−0.2414
"00074c10b5"	"00074da036"	−0.0166
"00074c10b5"	"00074da082"	0.3249
"00074c10b5"	"00074da136"	−0.0836
"00074c10b5"	"00074da3ed"	−0.5334
"00074c10b5"	"00074da4ac"	0.1432
"00074c10b5"	"00074da4b8"	−0.0167
"00074c10b5"	"00074da5e8"	−0.1141
"00074c10b5"	"00074da6b4"	−0.1432
"00074c10b5"	"00074daa3c"	−0.3546
"00074c10b5"	"00074daaf6"	−0.0423
"00074c10b5"	"00074dad20"	0.0154
"00074c10b5"	"00074db098"	−0.2305
"00074c10b5"	"00074db231"	−0.1298
"00074c10b5"	"00074db3a3"	−0.1177
"00074c10b5"	"00074db5d6"	0.1664
"00074c10b5"	"00074db632"	0.0564
"00074c10b5"	"00074db688"	−0.0995
"00074c10b5"	"00074db8a6"	−0.1599
"00074c10b5"	"00074dba19"	−0.0552
"00074c10b5"	"00074dbc2e"	−0.2952
"00074c10b5"	"00074dbe51"	−0.0583
"00074c10b5"	"00074dbe5f"	−0.2987
"00074c10b5"	"00074dbf6d"	−0.3163
"00074c10b5"	"00074dc4a5"	0.3403
"00074c10b5"	"00074dc50c"	−0.2074
"00074c10b5"	"00074dcdfa"	−0.0982
"00074c10b5"	"00074dcf5f"	−0.0997
"00074c10b5"	"00074dd007"	−0.2051
"00074c10b5"	"00074dd163"	−0.1477
"00074c10b5"	"00074dd3df"	−0.0128
"00074c10b5"	"00074dd577"	−0.2244
"00074c10b5"	"00074dd62e"	0.0433
"00074c10b5"	"00074dd73c"	−0.2
"00074c10b5"	"00074dda10"	−0.147
"00074c10b5"	"00074ddab8"	−0.031
"00074c10b5"	"00074ddd3d"	−0.0781
"00074c10b5"	"00074ddf16"	−0.114
"00074c10b5"	"00074ddfc1"	−0.1767
"00074c10b5"	"00074de21a"	0.0845
"00074c10b5"	"00074de2a9"	−0.1082
"00074c10b5"	"00074de544"	−0.0705
"00074c10b5"	"00074de98a"	−0.012
"00074c10b5"	"00074dea7e"	−0.117
"00074c10b5"	"00074debd9"	−0.5301
"00074c10b5"	"00074deca3"	−0.1553
"00074c10b5"	"00074def43"	−0.1119
"00074c10b5"	"00074def99"	−0.0934

"00074c10b5"	"00074ecdad"	−0.2811
"00074c10b5"	"00074ecf28"	0.1641
"00074c10b5"	"00074ed1e1"	−0.2085
"00074c10b5"	"00074ed83b"	0.0241
"00074c10b5"	"00074ee5e3"	−0.2242
"00074c10b5"	"00074ee6e0"	−0.2885
"00074c10b5"	"00074eea3a"	−0.0533
"00074c10b5"	"00074eff82"	−0.0705
"00074c10b5"	"00074f0477"	−0.2725
"00074c10b5"	"00074f08c3"	−0.0111
"00074c10b5"	"00074f1859"	−0.0465
"00074c10b5"	"00074f2268"	−0.3085
"00074c10b5"	"00074f28be"	−0.1159
"00074c10b5"	"00074f294b"	−0.1692
"00074c10b5"	"00074f2ddd"	−0.1694
"00074c10b5"	"00074f2e75"	−0.1165
"00074c10b5"	"00074f3088"	0.1906
"00074c10b5"	"00074f5a1c"	−0.0491
"00074c10b5"	"00074f75b7"	−0.1396
"00074c10b5"	"00074f8cd9"	−0.0233
"00074c10b5"	"00074f96dc"	0.2964
"00074c10b5"	"00074fabaa"	−0.1026
"00074c10b5"	"00074facd9"	−0.1544
"00074c10b5"	"00074fae3c"	0.1038
"00074c10b5"	"00074fb0a8"	−0.088
"00074c10b5"	"00074fb4e4"	−0.0754
"00074c10b5"	"00074fb7c2"	0.0545
"00074c10b5"	"00074fbd36"	0.102
"00074c10b5"	"00074fc27f"	−0.3161
"00074c10b5"	"00074fc31d"	−0.0789
"00074c10b5"	"00074fd569"	0.2297
"00074c10b5"	"00074fef15"	0.1493
"00074c10b5"	"00074ff562"	−0.0798
"00074c10b5"	"00075007ca"	−0.4707
"00074c10b5"	"0007500b86"	−0.0452
"00074c10b5"	"0007500d05"	−0.0512
"00074c10b5"	"0007500ee4"	−0.0214
"00074c10b5"	"0007500eee"	−0.3929
"00074c10b5"	"00075013dc"	−0.2372
"00074c10b5"	"000757b515"	−0.3015
"00074c10b5"	"000757bc5a"	−0.1321
"00074c10b5"	"000757c320"	−0.0102
"00074c10b5"	"000757c9aa"	0.0839
"00074c10b5"	"000757ccbe"	0.1102
"00074c10b5"	"000757cfa9"	−0.0595
"00074c10b5"	"000757d390"	−0.2782
"00074c10b5"	"000757d393"	0.106
"00074c10b5"	"000757d598"	−0.2329
"00074c10b5"	"000757d5a2"	−0.2317
"00074c10b5"	"000757d790"	−0.191

"00074c10b5"	"000757e30c"	-0.2951
"00074c10b5"	"000757e4b0"	-0.0491
"00074c10b5"	"000757e7a0"	-0.0949
"00074c10b5"	"000757e8b3"	0.057
"00074c10b5"	"000757f627"	-0.0815
"00074c10b5"	"000757f925"	0.1917
"00074c10b5"	"000757fa08"	-0.2122
"00074c10b5"	"000757fe52"	-0.0412
"00074c10b5"	"000758024a"	-0.1989
"00074c10b5"	"00075804bb"	-0.3976
"00074c10b5"	"00075a0c04"	0.3256
"00074c10b5"	"00075a3110"	0.0036
"00074c10b5"	"00075a341a"	0.1388
"00074c10b5"	"00075a3dcf"	-0.0086
"00074c10b5"	"00075a3e22"	0.1539
"00074c10b5"	"00075a48d8"	0.124
"00074c10b5"	"00075a5cfb"	-0.2779
"00074c10b5"	"00075a6151"	-0.3026
"00074c10b5"	"00075a6708"	-0.1902
"00074c10b5"	"00075a7319"	-0.2583
"00074c10b5"	"00075a7723"	-0.0952
"00074c10b5"	"00075a778b"	0.1549
"00074c10b5"	"00075a7b8e"	-0.121
"00074c10b5"	"00075a7c79"	-0.2331
"00074c10b5"	"00075a81b6"	-0.0833
"00074c10b5"	"00075a82ac"	-6e-04
"00074c10b5"	"00075a98e5"	0.1822
"00074c10b5"	"00075b0d29"	0.2481
"00074c10b5"	"00075b102a"	-0.1046
"00074c10b5"	"00075b1074"	-0.3061
"00074c10b5"	"00075b135d"	-0.0461
"00074c10b5"	"00075b138b"	-0.0853
"00074c10b5"	"00075b13a0"	-0.0514
"00074c10b5"	"00075b13bd"	0.3711
"00074c10b5"	"00075b16a9"	0.1835
"00074c10b5"	"00075b1a28"	-0.1871
"00074c10b5"	"00075b1a97"	0.137
"00074c10b5"	"00075b1c7b"	-0.3452
"00074c10b5"	"00075b1d24"	-0.225
"00074c10b5"	"00075b202b"	-0.1096
"00074c10b5"	"00075b22cb"	0.0347
"00074c10b5"	"00075b22da"	-0.2764
"00074c10b5"	"00075b2556"	-0.2396
"00074c10b5"	"00075b25de"	-0.1317
"00074c10b5"	"00075b260c"	-0.2724
"00074c10b5"	"00075b26f1"	0.0439
"00074c10b5"	"00075b2920"	-0.3293
"00074c10b5"	"00075b2a64"	-0.2404
"00074c10b5"	"00075b2a9d"	-0.3294
"00074c10b5"	"00075b2b37"	-0.4876

"00074c10b5"	"00075b2cdd"	0.0659
"00074c10b5"	"00075b3038"	-0.0887
"00074c10b5"	"00075b30fe"	-0.5152
"00074c10b5"	"00075b3362"	-0.0693
"00074c10b5"	"00075b350a"	-0.1811
"00074c10b5"	"00075b350e"	-0.0872
"00074c10b5"	"00075b3651"	0.0103
"00074c10b5"	"00075b38ca"	-0.0557
"00074c10b5"	"00075b39cc"	0.0293
"00074c10b5"	"00075b3e1e"	0.0715
"00074c10b5"	"00075b3e57"	0.3092
"00074c10b5"	"00075b4079"	-0.2123
"00074c10b5"	"00075b4150"	-0.149
"00074c10b5"	"00075b4194"	0.0153
"00074c10b5"	"00075b42d5"	-0.1349
"00074c10b5"	"00075b4424"	0.0339
"00074c10b5"	"00075b4470"	-0.1321
"00074c10b5"	"00075b47ed"	0.1322
"00074c10b5"	"00075b4850"	-0.1056
"00074c10b5"	"00075b4ca0"	-0.2178
"00074c10b5"	"00075b4d7f"	0.079
"00074c10b5"	"00075b520f"	0.0216
"00074c10b5"	"00075b525f"	-0.1211
"00074c10b5"	"00075b58f8"	-0.3702
"00074c10b5"	"00075b5bcc"	-0.1198
"00074c10b5"	"00075b5bfa"	-0.4303
"00074c10b5"	"00075b6339"	0.0568
"00074c10b5"	"00075b6658"	0.0589
"00074c10b5"	"00075b679a"	-0.0892
"00074c10b5"	"00075b6cb7"	-0.3776
"00074c10b5"	"00075b6df8"	0.1306
"00074c10b5"	"00075b6ff6"	-0.2379
"00074c10b5"	"00075b70ee"	-0.0873
"00074c10b5"	"00075b7157"	-0.1176
"00074c10b5"	"00075b7225"	0.0202
"00074c10b5"	"00075b7c89"	0.1906
"00074c10b5"	"00075b9048"	-0.0717
"00074c10b5"	"00075d0801"	-0.1192
"00074c10b5"	"00075d1820"	0.2925
"00074c10b5"	"00075d1f3d"	-0.3
"00074c10b5"	"00075d2329"	-0.018
"00074c10b5"	"00075d2b9b"	-0.2346
"00074c10b5"	"00075d3941"	0.0507
"00074c10b5"	"00075d3e96"	-0.0891
"00074c10b5"	"00075d4864"	-0.2503
"00074c10b5"	"00075d5961"	-0.0484
"00074c10b5"	"00075d5a63"	-0.0201
"00074c10b5"	"00075d6150"	-0.127
"00074c10b5"	"00075d67d0"	-0.3319
"00074c10b5"	"00075d67e2"	0.0126

"00074c10b5"	"00075d73fc"	0.1272
"00074c10b5"	"00075d7729"	-0.0701
"00074c10b5"	"00075d778c"	-0.1772
"00074c10b5"	"00075d7b9e"	-0.2139
"00074c10b5"	"00075d7c8f"	-0.0911
"00074c10b5"	"00075d804d"	-0.2386
"00074c10b5"	"00075d819f"	-0.2203
"00074c10b5"	"00075d8601"	0.2844
"00074c10b5"	"00075d8c6a"	0.2421
"00074c10b5"	"00075dfedc"	0.1655
"00074c10b5"	"00075e05f2"	-0.2922
"00074c10b5"	"00075e0837"	-0.1462
"00074c10b5"	"00075e092e"	-0.3583
"00074c10b5"	"00075e0965"	-0.4764
"00074c10b5"	"00075e0bc8"	-0.1237
"00074c10b5"	"00075e0fbb"	-0.5164
"00074c1ca9"	"00074c2119"	-0.0633
"00074c1ca9"	"00074c3272"	0.2205
"00074c1ca9"	"00074c33e4"	0.0347
"00074c1ca9"	"00074c35b2"	0.1314
"00074c1ca9"	"00074c38cd"	-0.4238
"00074c1ca9"	"00074c3a40"	-0.0767
"00074c1ca9"	"00074d84fc"	-0.3562
"00074c1ca9"	"00074d8639"	-0.3473
"00074c1ca9"	"00074d8697"	0.0434
"00074c1ca9"	"00074d87af"	-0.2499
"00074c1ca9"	"00074d8813"	-0.4753
"00074c1ca9"	"00074d8817"	0.1057
"00074c1ca9"	"00074d8a39"	-0.1989
"00074c1ca9"	"00074d8ad8"	-0.1209
"00074c1ca9"	"00074d8c26"	-0.2071
"00074c1ca9"	"00074d8ca5"	-0.4879
"00074c1ca9"	"00074d8e0a"	0.0323
"00074c1ca9"	"00074d9179"	-0.3875
"00074c1ca9"	"00074d929f"	-0.0612
"00074c1ca9"	"00074d93c4"	-0.4461
"00074c1ca9"	"00074d93d0"	-0.3431
"00074c1ca9"	"00074d945b"	-0.2905
"00074c1ca9"	"00074d966c"	-0.3569
"00074c1ca9"	"00074d9953"	-0.2302
"00074c1ca9"	"00074d99b2"	0.0195
"00074c1ca9"	"00074d99f3"	-0.1531
"00074c1ca9"	"00074d9afd"	-0.5926
"00074c1ca9"	"00074d9c87"	-0.1279
"00074c1ca9"	"00074d9e9e"	-0.5756
"00074c1ca9"	"00074d9f30"	-0.326
"00074c1ca9"	"00074da036"	-0.1237
"00074c1ca9"	"00074da082"	-0.5026
"00074c1ca9"	"00074da136"	-0.3363
"00074c1ca9"	"00074da3ed"	-0.4142

"00074c1ca9"	"00074da4ac"	-0.101
"00074c1ca9"	"00074da4b8"	-0.1314
"00074c1ca9"	"00074da5e8"	0.0513
"00074c1ca9"	"00074da6b4"	-0.2249
"00074c1ca9"	"00074daa3c"	-0.35
"00074c1ca9"	"00074daaf6"	0.0211
"00074c1ca9"	"00074dad20"	-0.5861
"00074c1ca9"	"00074db098"	-0.3114
"00074c1ca9"	"00074db231"	-0.0161
"00074c1ca9"	"00074db3a3"	-0.139
"00074c1ca9"	"00074db5d6"	-0.1978
"00074c1ca9"	"00074db632"	-0.0428
"00074c1ca9"	"00074db688"	0.1057
"00074c1ca9"	"00074db8a6"	-0.2413
"00074c1ca9"	"00074dba19"	-0.2671
"00074c1ca9"	"00074dbc2e"	-0.3606
"00074c1ca9"	"00074dbe51"	-0.2291
"00074c1ca9"	"00074dbe5f"	-0.7694
"00074c1ca9"	"00074dbf6d"	-0.3434
"00074c1ca9"	"00074dc4a5"	-0.6405
"00074c1ca9"	"00074dc50c"	-0.1468
"00074c1ca9"	"00074dcdfa"	0.0365
"00074c1ca9"	"00074dcf5f"	-0.3037
"00074c1ca9"	"00074dd007"	-0.1602
"00074c1ca9"	"00074dd163"	-0.219
"00074c1ca9"	"00074dd3df"	-0.6787
"00074c1ca9"	"00074dd577"	-0.3299
"00074c1ca9"	"00074dd62e"	-0.0313
"00074c1ca9"	"00074dd73c"	-0.4374
"00074c1ca9"	"00074dda10"	-0.3986
"00074c1ca9"	"00074ddab8"	-0.2517
"00074c1ca9"	"00074ddd3d"	-0.2127
"00074c1ca9"	"00074ddf16"	-0.2506
"00074c1ca9"	"00074ddfc1"	-0.4963
"00074c1ca9"	"00074de21a"	0.2305
"00074c1ca9"	"00074de2a9"	-0.0891
"00074c1ca9"	"00074de544"	-0.1763
"00074c1ca9"	"00074de98a"	-0.3283
"00074c1ca9"	"00074dea7e"	-0.2585
"00074c1ca9"	"00074debd9"	-0.6149
"00074c1ca9"	"00074deca3"	-0.2872
"00074c1ca9"	"00074def43"	-0.4604
"00074c1ca9"	"00074def99"	-0.1754
"00074c1ca9"	"00074ecdad"	-0.3502
"00074c1ca9"	"00074ecf28"	-0.2938
"00074c1ca9"	"00074ed1e1"	-0.162
"00074c1ca9"	"00074ed83b"	0.089
"00074c1ca9"	"00074ee5e3"	-0.5212
"00074c1ca9"	"00074ee6e0"	-0.0857
"00074c1ca9"	"00074eea3a"	-0.3384

"00074c1ca9"	"00074eff82"	-0.261
"00074c1ca9"	"00074f0477"	-0.6572
"00074c1ca9"	"00074f08c3"	0.0659
"00074c1ca9"	"00074f1859"	-0.5141
"00074c1ca9"	"00074f2268"	-0.4784
"00074c1ca9"	"00074f28be"	0.2239
"00074c1ca9"	"00074f294b"	-0.2604
"00074c1ca9"	"00074f2ddd"	-0.152
"00074c1ca9"	"00074f2e75"	-0.5662
"00074c1ca9"	"00074f3088"	0.0162
"00074c1ca9"	"00074f5a1c"	-0.267
"00074c1ca9"	"00074f75b7"	-0.4138
"00074c1ca9"	"00074f8cd9"	-0.4098
"00074c1ca9"	"00074f96dc"	-0.2917
"00074c1ca9"	"00074fabaa"	-0.0925
"00074c1ca9"	"00074facd9"	-0.5894
"00074c1ca9"	"00074fae3c"	-0.3288
"00074c1ca9"	"00074fb0a8"	0.5611
"00074c1ca9"	"00074fb4e4"	0.3369
"00074c1ca9"	"00074fb7c2"	0.0202
"00074c1ca9"	"00074fbd36"	-0.3093
"00074c1ca9"	"00074fc27f"	-0.3759
"00074c1ca9"	"00074fc31d"	0.1433
"00074c1ca9"	"00074fd569"	-0.0822
"00074c1ca9"	"00074fef15"	-0.2907
"00074c1ca9"	"00074ff562"	-0.3878
"00074c1ca9"	"00075007ca"	-0.1814
"00074c1ca9"	"0007500b86"	-0.1288
"00074c1ca9"	"0007500d05"	-0.5107
"00074c1ca9"	"0007500ee4"	-0.1541
"00074c1ca9"	"0007500eee"	-0.7679
"00074c1ca9"	"00075013dc"	0.4385
"00074c1ca9"	"000757b515"	-0.1222
"00074c1ca9"	"000757bc5a"	-0.0757
"00074c1ca9"	"000757c320"	-0.3134
"00074c1ca9"	"000757c9aa"	0.049
"00074c1ca9"	"000757ccbe"	0.1251
"00074c1ca9"	"000757cfa9"	-0.265
"00074c1ca9"	"000757d390"	0.1958
"00074c1ca9"	"000757d393"	-0.2638
"00074c1ca9"	"000757d598"	-0.0975
"00074c1ca9"	"000757d5a2"	-0.1748
"00074c1ca9"	"000757d790"	-0.1935
"00074c1ca9"	"000757e30c"	-0.1265
"00074c1ca9"	"000757e4b0"	0.0772
"00074c1ca9"	"000757e7a0"	-0.2214
"00074c1ca9"	"000757e8b3"	-0.042
"00074c1ca9"	"000757f627"	0.0634
"00074c1ca9"	"000757f925"	-0.0748
"00074c1ca9"	"000757fa08"	-0.4413

"00074c1ca9"	"000757fe52"	-0.4546
"00074c1ca9"	"000758024a"	-0.3814
"00074c1ca9"	"00075804bb"	-0.137
"00074c1ca9"	"00075a0c04"	-0.3073
"00074c1ca9"	"00075a3110"	-0.1514
"00074c1ca9"	"00075a341a"	-0.3984
"00074c1ca9"	"00075a3dcf"	-0.3682
"00074c1ca9"	"00075a3e22"	-0.1363
"00074c1ca9"	"00075a48d8"	-0.4237
"00074c1ca9"	"00075a5cfb"	-0.0645
"00074c1ca9"	"00075a6151"	-0.2076
"00074c1ca9"	"00075a6708"	-0.395
"00074c1ca9"	"00075a7319"	0.2123
"00074c1ca9"	"00075a7723"	-0.1558
"00074c1ca9"	"00075a778b"	-0.4842
"00074c1ca9"	"00075a7b8e"	-0.1532
"00074c1ca9"	"00075a7c79"	-0.0981
"00074c1ca9"	"00075a81b6"	-0.4278
"00074c1ca9"	"00075a82ac"	-0.1203
"00074c1ca9"	"00075a98e5"	-0.4048
"00074c1ca9"	"00075b0d29"	-0.3973
"00074c1ca9"	"00075b102a"	-0.4055
"00074c1ca9"	"00075b1074"	-0.3691
"00074c1ca9"	"00075b135d"	0.0576
"00074c1ca9"	"00075b138b"	-0.2647
"00074c1ca9"	"00075b13a0"	-0.4432
"00074c1ca9"	"00075b13bd"	-0.2717
"00074c1ca9"	"00075b16a9"	-0.4661
"00074c1ca9"	"00075b1a28"	-0.5007
"00074c1ca9"	"00075b1a97"	-0.2
"00074c1ca9"	"00075b1c7b"	-0.6379
"00074c1ca9"	"00075b1d24"	-0.1161
"00074c1ca9"	"00075b202b"	-0.2364
"00074c1ca9"	"00075b22cb"	-0.6828
"00074c1ca9"	"00075b22da"	-0.3657
"00074c1ca9"	"00075b2556"	-0.3111
"00074c1ca9"	"00075b25de"	-0.133
"00074c1ca9"	"00075b260c"	-0.2976
"00074c1ca9"	"00075b26f1"	-0.3677
"00074c1ca9"	"00075b2920"	0.0138
"00074c1ca9"	"00075b2a64"	-0.3044
"00074c1ca9"	"00075b2a9d"	-0.0634
"00074c1ca9"	"00075b2b37"	-0.3607
"00074c1ca9"	"00075b2cdd"	-0.3353
"00074c1ca9"	"00075b3038"	-0.2321
"00074c1ca9"	"00075b30fe"	-0.3227
"00074c1ca9"	"00075b3362"	-0.1657
"00074c1ca9"	"00075b350a"	-0.2489
"00074c1ca9"	"00075b350e"	0.0737
"00074c1ca9"	"00075b3651"	-0.3514

"00074c1ca9"	"00075b38ca"	-0.4355
"00074c1ca9"	"00075b39cc"	-0.2052
"00074c1ca9"	"00075b3e1e"	-0.39
"00074c1ca9"	"00075b3e57"	-0.4909
"00074c1ca9"	"00075b4079"	-0.0876
"00074c1ca9"	"00075b4150"	-0.2646
"00074c1ca9"	"00075b4194"	0.1542
"00074c1ca9"	"00075b42d5"	-0.382
"00074c1ca9"	"00075b4424"	-0.3708
"00074c1ca9"	"00075b4470"	-0.0757
"00074c1ca9"	"00075b47ed"	0.0158
"00074c1ca9"	"00075b4850"	0.0965
"00074c1ca9"	"00075b4ca0"	-0.0763
"00074c1ca9"	"00075b4d7f"	-0.2133
"00074c1ca9"	"00075b520f"	-0.1107
"00074c1ca9"	"00075b525f"	-0.0378
"00074c1ca9"	"00075b58f8"	-0.4641
"00074c1ca9"	"00075b5bcc"	-0.2002
"00074c1ca9"	"00075b5bfa"	-0.4455
"00074c1ca9"	"00075b6339"	-0.2051
"00074c1ca9"	"00075b6658"	-0.4756
"00074c1ca9"	"00075b679a"	-0.1625
"00074c1ca9"	"00075b6cb7"	-0.4879
"00074c1ca9"	"00075b6df8"	-0.4686
"00074c1ca9"	"00075b6ff6"	-0.1319
"00074c1ca9"	"00075b70ee"	-0.2216
"00074c1ca9"	"00075b7157"	-0.2729
"00074c1ca9"	"00075b7225"	-0.1874
"00074c1ca9"	"00075b7c89"	-0.168
"00074c1ca9"	"00075b9048"	-0.1164
"00074c1ca9"	"00075d0801"	0.049
"00074c1ca9"	"00075d1820"	-0.331
"00074c1ca9"	"00075d1f3d"	-0.1263
"00074c1ca9"	"00075d2329"	-0.3929
"00074c1ca9"	"00075d2b9b"	-0.0569
"00074c1ca9"	"00075d3941"	-0.392
"00074c1ca9"	"00075d3e96"	-0.0199
"00074c1ca9"	"00075d4864"	-0.2312
"00074c1ca9"	"00075d5961"	-0.3355
"00074c1ca9"	"00075d5a63"	-0.3007
"00074c1ca9"	"00075d6150"	0.127
"00074c1ca9"	"00075d67d0"	-0.3025
"00074c1ca9"	"00075d67e2"	-0.5092
"00074c1ca9"	"00075d73fc"	-0.4354
"00074c1ca9"	"00075d7729"	-0.272
"00074c1ca9"	"00075d778c"	-0.3493
"00074c1ca9"	"00075d7b9e"	-0.1518
"00074c1ca9"	"00075d7c8f"	-0.1629
"00074c1ca9"	"00075d804d"	-0.2885
"00074c1ca9"	"00075d819f"	-0.1471

"00074c1ca9"	"00075d8601"	-0.2778
"00074c1ca9"	"00075d8c6a"	-0.2001
"00074c1ca9"	"00075dfedc"	-0.3217
"00074c1ca9"	"00075e05f2"	-0.3827
"00074c1ca9"	"00075e0837"	-0.2638
"00074c1ca9"	"00075e092e"	-0.3261
"00074c1ca9"	"00075e0965"	-0.4017
"00074c1ca9"	"00075e0bc8"	-0.2138
"00074c1ca9"	"00075e0fbb"	-0.3648
"00074c2119"	"00074c3272"	0.0835
"00074c2119"	"00074c33e4"	-0.1084
"00074c2119"	"00074c35b2"	-0.0875
"00074c2119"	"00074c38cd"	0.0158
"00074c2119"	"00074c3a40"	-0.2345
"00074c2119"	"00074d84fc"	-0.198
"00074c2119"	"00074d8639"	0.16
"00074c2119"	"00074d8697"	-0.05
"00074c2119"	"00074d87af"	-0.0784
"00074c2119"	"00074d8813"	-0.1058
"00074c2119"	"00074d8817"	0.1311
"00074c2119"	"00074d8a39"	0.092
"00074c2119"	"00074d8ad8"	0.2612
"00074c2119"	"00074d8c26"	-0.3479
"00074c2119"	"00074d8ca5"	-0.0969
"00074c2119"	"00074d8e0a"	-0.1863
"00074c2119"	"00074d9179"	-0.05
"00074c2119"	"00074d929f"	0.0167
"00074c2119"	"00074d93c4"	-0.2093
"00074c2119"	"00074d93d0"	0.2222
"00074c2119"	"00074d945b"	-0.1025
"00074c2119"	"00074d966c"	0.0215
"00074c2119"	"00074d9953"	0.0253
"00074c2119"	"00074d99b2"	0.0163
"00074c2119"	"00074d99f3"	0.4295
"00074c2119"	"00074d9afd"	-0.1353
"00074c2119"	"00074d9c87"	0.0937
"00074c2119"	"00074d9e9e"	-0.4692
"00074c2119"	"00074d9f30"	0.2403
"00074c2119"	"00074da036"	0.2559
"00074c2119"	"00074da082"	0.0384
"00074c2119"	"00074da136"	-0.1268
"00074c2119"	"00074da3ed"	0.0022
"00074c2119"	"00074da4ac"	0.0479
"00074c2119"	"00074da4b8"	0.0954
"00074c2119"	"00074da5e8"	0.2554
"00074c2119"	"00074da6b4"	0.1099
"00074c2119"	"00074daa3c"	-0.2128
"00074c2119"	"00074daaf6"	0.1454
"00074c2119"	"00074dad20"	-0.2229
"00074c2119"	"00074db098"	0.0108

"00074c2119"	"00074db231"	-0.1602
"00074c2119"	"00074db3a3"	0.16
"00074c2119"	"00074db5d6"	-0.0897
"00074c2119"	"00074db632"	0.1395
"00074c2119"	"00074db688"	0.2896
"00074c2119"	"00074db8a6"	-0.1353
"00074c2119"	"00074dba19"	-0.117
"00074c2119"	"00074dbc2e"	-0.179
"00074c2119"	"00074dbe51"	0.2304
"00074c2119"	"00074dbe5f"	-0.5819
"00074c2119"	"00074dbf6d"	-0.1677
"00074c2119"	"00074dc4a5"	-0.0799
"00074c2119"	"00074dc50c"	-0.2308
"00074c2119"	"00074dcdfa"	-0.1287
"00074c2119"	"00074dcf5f"	0.1956
"00074c2119"	"00074dd007"	-0.1814
"00074c2119"	"00074dd163"	-0.3277
"00074c2119"	"00074dd3df"	-0.2327
"00074c2119"	"00074dd577"	0.0415
"00074c2119"	"00074dd62e"	-0.2821
"00074c2119"	"00074dd73c"	0.0016
"00074c2119"	"00074dda10"	0.1172
"00074c2119"	"00074ddab8"	-0.159
"00074c2119"	"00074ddd3d"	0.2308
"00074c2119"	"00074ddf16"	-0.1948
"00074c2119"	"00074ddfc1"	-0.2381
"00074c2119"	"00074de21a"	0.0646
"00074c2119"	"00074de2a9"	-0.2129
"00074c2119"	"00074de544"	0.1095
"00074c2119"	"00074de98a"	-0.1323
"00074c2119"	"00074dea7e"	-0.0468
"00074c2119"	"00074debd9"	-0.4951
"00074c2119"	"00074deca3"	-0.1852
"00074c2119"	"00074def43"	-0.1689
"00074c2119"	"00074def99"	0.5729
"00074c2119"	"00074ecdad"	-0.4458
"00074c2119"	"00074ecf28"	-0.1733
"00074c2119"	"00074ed1e1"	-0.0784
"00074c2119"	"00074ed83b"	0.1395
"00074c2119"	"00074ee5e3"	-0.2135
"00074c2119"	"00074ee6e0"	-0.0361
"00074c2119"	"00074eea3a"	-0.0655
"00074c2119"	"00074eff82"	0.0967
"00074c2119"	"00074f0477"	-0.3142
"00074c2119"	"00074f08c3"	0.0605
"00074c2119"	"00074f1859"	-0.3766
"00074c2119"	"00074f2268"	-0.2095
"00074c2119"	"00074f28be"	-0.0364
"00074c2119"	"00074f294b"	0.1784
"00074c2119"	"00074f2ddd"	0.1326

"00074c2119"	"00074f2e75"	-0.2229
"00074c2119"	"00074f3088"	0.0749
"00074c2119"	"00074f5a1c"	-0.1313
"00074c2119"	"00074f75b7"	0.0074
"00074c2119"	"00074f8cd9"	-0.175
"00074c2119"	"00074f96dc"	-0.129
"00074c2119"	"00074fabaa"	-0.2177
"00074c2119"	"00074facd9"	-0.4014
"00074c2119"	"00074fae3c"	-0.0682
"00074c2119"	"00074fb0a8"	-0.015
"00074c2119"	"00074fb4e4"	0.0759
"00074c2119"	"00074fb7c2"	0.0531
"00074c2119"	"00074fbd36"	0.0806
"00074c2119"	"00074fc27f"	-0.1279
"00074c2119"	"00074fc31d"	0.3183
"00074c2119"	"00074fd569"	0.0142
"00074c2119"	"00074fef15"	0.1681
"00074c2119"	"00074ff562"	-0.4513
"00074c2119"	"00075007ca"	-0.093
"00074c2119"	"0007500b86"	9e-04
"00074c2119"	"0007500d05"	-0.2262
"00074c2119"	"0007500ee4"	0.1389
"00074c2119"	"0007500eee"	-0.4554
"00074c2119"	"00075013dc"	-0.3122
"00074c2119"	"000757b515"	0.2442
"00074c2119"	"000757bc5a"	-0.0274
"00074c2119"	"000757c320"	-0.01
"00074c2119"	"000757c9aa"	0.0813
"00074c2119"	"000757ccbe"	-0.2174
"00074c2119"	"000757cfa9"	0.0081
"00074c2119"	"000757d390"	0.0345
"00074c2119"	"000757d393"	-0.1138
"00074c2119"	"000757d598"	-0.0727
"00074c2119"	"000757d5a2"	-0.0731
"00074c2119"	"000757d790"	-0.0152
"00074c2119"	"000757e30c"	0.1026
"00074c2119"	"000757e4b0"	-0.0918
"00074c2119"	"000757e7a0"	0.2081
"00074c2119"	"000757e8b3"	0.1274
"00074c2119"	"000757f627"	0.0313
"00074c2119"	"000757f925"	0.0637
"00074c2119"	"000757fa08"	-0.1435
"00074c2119"	"000757fe52"	-0.0388
"00074c2119"	"000758024a"	0.0152
"00074c2119"	"00075804bb"	-0.1613
"00074c2119"	"00075a0c04"	-0.2016
"00074c2119"	"00075a3110"	-0.1317
"00074c2119"	"00075a341a"	0.051
"00074c2119"	"00075a3dcf"	0.0219
"00074c2119"	"00075a3e22"	-0.2011

"00074c2119"	"00075a48d8"	-0.1709
"00074c2119"	"00075a5cfb"	-0.108
"00074c2119"	"00075a6151"	-0.0041
"00074c2119"	"00075a6708"	0.0636
"00074c2119"	"00075a7319"	0.0706
"00074c2119"	"00075a7723"	0.2371
"00074c2119"	"00075a778b"	-0.1199
"00074c2119"	"00075a7b8e"	-0.1887
"00074c2119"	"00075a7c79"	0.1467
"00074c2119"	"00075a81b6"	-0.0191
"00074c2119"	"00075a82ac"	0.0762
"00074c2119"	"00075a98e5"	-0.0792
"00074c2119"	"00075b0d29"	0.1324
"00074c2119"	"00075b102a"	-0.1535
"00074c2119"	"00075b1074"	-0.1096
"00074c2119"	"00075b135d"	0.0214
"00074c2119"	"00075b138b"	-0.016
"00074c2119"	"00075b13a0"	-0.4243
"00074c2119"	"00075b13bd"	0.0965
"00074c2119"	"00075b16a9"	0.1185
"00074c2119"	"00075b1a28"	-0.0222
"00074c2119"	"00075b1a97"	0.1876
"00074c2119"	"00075b1c7b"	-0.2862
"00074c2119"	"00075b1d24"	0.4104
"00074c2119"	"00075b202b"	-0.2532
"00074c2119"	"00075b22cb"	-0.1433
"00074c2119"	"00075b22da"	-0.1062
"00074c2119"	"00075b2556"	0.0316
"00074c2119"	"00075b25de"	0.1998
"00074c2119"	"00075b260c"	-0.1054
"00074c2119"	"00075b26f1"	-0.0501
"00074c2119"	"00075b2920"	-0.0723
"00074c2119"	"00075b2a64"	-0.1714
"00074c2119"	"00075b2a9d"	-0.1978
"00074c2119"	"00075b2b37"	-0.2383
"00074c2119"	"00075b2cdd"	-0.1021
"00074c2119"	"00075b3038"	0.0267
"00074c2119"	"00075b30fe"	-0.0802
"00074c2119"	"00075b3362"	0.2278
"00074c2119"	"00075b350a"	-0.0977
"00074c2119"	"00075b350e"	-0.046
"00074c2119"	"00075b3651"	0.2975
"00074c2119"	"00075b38ca"	-0.2117
"00074c2119"	"00075b39cc"	0.0398
"00074c2119"	"00075b3e1e"	-0.3239
"00074c2119"	"00075b3e57"	-0.1232
"00074c2119"	"00075b4079"	0.0687
"00074c2119"	"00075b4150"	-0.0179
"00074c2119"	"00075b4194"	-0.0551
"00074c2119"	"00075b42d5"	-0.1231

"00074c2119"	"00075b4424"	-0.0583
"00074c2119"	"00075b4470"	-0.0274
"00074c2119"	"00075b47ed"	0.0288
"00074c2119"	"00075b4850"	-0.0215
"00074c2119"	"00075b4ca0"	0.0679
"00074c2119"	"00075b4d7f"	0.1098
"00074c2119"	"00075b520f"	0.001
"00074c2119"	"00075b525f"	0.413
"00074c2119"	"00075b58f8"	-0.2099
"00074c2119"	"00075b5bcc"	0.2773
"00074c2119"	"00075b5bfa"	-0.1277
"00074c2119"	"00075b6339"	0.2603
"00074c2119"	"00075b6658"	0.0777
"00074c2119"	"00075b679a"	0.3231
"00074c2119"	"00075b6cb7"	-0.111
"00074c2119"	"00075b6df8"	0.1046
"00074c2119"	"00075b6ff6"	0.1266
"00074c2119"	"00075b70ee"	-0.0237
"00074c2119"	"00075b7157"	0.1752
"00074c2119"	"00075b7225"	0.0331
"00074c2119"	"00075b7c89"	0.0508
"00074c2119"	"00075b9048"	0.0126
"00074c2119"	"00075d0801"	0.062
"00074c2119"	"00075d1820"	0.2139
"00074c2119"	"00075d1f3d"	-0.1179
"00074c2119"	"00075d2329"	-0.0387
"00074c2119"	"00075d2b9b"	-0.185
"00074c2119"	"00075d3941"	-0.3222
"00074c2119"	"00075d3e96"	0.1693
"00074c2119"	"00075d4864"	-0.1399
"00074c2119"	"00075d5961"	-0.079
"00074c2119"	"00075d5a63"	-0.0271
"00074c2119"	"00075d6150"	-0.1477
"00074c2119"	"00075d67d0"	-0.028
"00074c2119"	"00075d67e2"	0.0065
"00074c2119"	"00075d73fc"	0.1201
"00074c2119"	"00075d7729"	0.1746
"00074c2119"	"00075d778c"	0.1471
"00074c2119"	"00075d7b9e"	0.1207
"00074c2119"	"00075d7c8f"	0.1955
"00074c2119"	"00075d804d"	-0.2171
"00074c2119"	"00075d819f"	-0.1148
"00074c2119"	"00075d8601"	0.0374
"00074c2119"	"00075d8c6a"	0.0707
"00074c2119"	"00075dfedc"	0.121
"00074c2119"	"00075e05f2"	-0.2439
"00074c2119"	"00075e0837"	-0.1773
"00074c2119"	"00075e092e"	-0.0978
"00074c2119"	"00075e0965"	-0.1501
"00074c2119"	"00075e0bc8"	-0.0729

"00074c2119"	"00075e0fbb"	-0.0474
"00074c3272"	"00074c33e4"	-0.0599
"00074c3272"	"00074c35b2"	0.2265
"00074c3272"	"00074c38cd"	-0.3002
"00074c3272"	"00074c3a40"	-0.249
"00074c3272"	"00074d84fc"	0.2631
"00074c3272"	"00074d8639"	-0.2879
"00074c3272"	"00074d8697"	0.3354
"00074c3272"	"00074d87af"	0.0343
"00074c3272"	"00074d8813"	-0.0839
"00074c3272"	"00074d8817"	0.6716
"00074c3272"	"00074d8a39"	0.3574
"00074c3272"	"00074d8ad8"	0.3034
"00074c3272"	"00074d8c26"	0.1553
"00074c3272"	"00074d8ca5"	-0.0721
"00074c3272"	"00074d8e0a"	0.3243
"00074c3272"	"00074d9179"	-0.294
"00074c3272"	"00074d929f"	-0.0869
"00074c3272"	"00074d93c4"	-0.0785
"00074c3272"	"00074d93d0"	-0.0595
"00074c3272"	"00074d945b"	-0.0149
"00074c3272"	"00074d966c"	0.1046
"00074c3272"	"00074d9953"	0.2209
"00074c3272"	"00074d99b2"	-0.0509
"00074c3272"	"00074d99f3"	0.2891
"00074c3272"	"00074d9afd"	-0.2722
"00074c3272"	"00074d9c87"	0.0525
"00074c3272"	"00074d9e9e"	-0.5169
"00074c3272"	"00074d9f30"	0.1062
"00074c3272"	"00074da036"	0.1873
"00074c3272"	"00074da082"	-0.1816
"00074c3272"	"00074da136"	-0.1971
"00074c3272"	"00074da3ed"	-0.0681
"00074c3272"	"00074da4ac"	0.1767
"00074c3272"	"00074da4b8"	0.2881
"00074c3272"	"00074da5e8"	0.1717
"00074c3272"	"00074da6b4"	0.0418
"00074c3272"	"00074daa3c"	0.1615
"00074c3272"	"00074daaf6"	0.0845
"00074c3272"	"00074dad20"	-0.1704
"00074c3272"	"00074db098"	0.0963
"00074c3272"	"00074db231"	0.1347
"00074c3272"	"00074db3a3"	0.3069
"00074c3272"	"00074db5d6"	-0.0807
"00074c3272"	"00074db632"	0.4185
"00074c3272"	"00074db688"	-0.0301
"00074c3272"	"00074db8a6"	-0.1097
"00074c3272"	"00074dba19"	0.1761
"00074c3272"	"00074dbc2e"	0.0366
"00074c3272"	"00074dbe51"	0.0531

"00074c3272"	"00074dbe5f"	-0.4136
"00074c3272"	"00074dbf6d"	0.1703
"00074c3272"	"00074dc4a5"	-0.2796
"00074c3272"	"00074dc50c"	0.4412
"00074c3272"	"00074dcdfa"	0.2369
"00074c3272"	"00074dcf5f"	-0.1948
"00074c3272"	"00074dd007"	0.2407
"00074c3272"	"00074dd163"	-0.1153
"00074c3272"	"00074dd3df"	-0.3192
"00074c3272"	"00074dd577"	0.0567
"00074c3272"	"00074dd62e"	0.1598
"00074c3272"	"00074dd73c"	0.1313
"00074c3272"	"00074dda10"	-0.0279
"00074c3272"	"00074ddab8"	0.5162
"00074c3272"	"00074ddd3d"	-0.1353
"00074c3272"	"00074ddf16"	0.0298
"00074c3272"	"00074ddfc1"	0.0609
"00074c3272"	"00074de21a"	0.4144
"00074c3272"	"00074de2a9"	0.4074
"00074c3272"	"00074de544"	-0.0555
"00074c3272"	"00074de98a"	-0.0016
"00074c3272"	"00074dea7e"	0.079
"00074c3272"	"00074debd9"	-0.0858
"00074c3272"	"00074deca3"	-0.0844
"00074c3272"	"00074def43"	0.0386
"00074c3272"	"00074def99"	0.0477
"00074c3272"	"00074ecdad"	-0.2966
"00074c3272"	"00074ecf28"	-0.2439
"00074c3272"	"00074ed1e1"	-0.1299
"00074c3272"	"00074ed83b"	0.319
"00074c3272"	"00074ee5e3"	0.0558
"00074c3272"	"00074ee6e0"	0.0318
"00074c3272"	"00074eea3a"	0.1088
"00074c3272"	"00074eff82"	-0.0512
"00074c3272"	"00074f0477"	-0.1498
"00074c3272"	"00074f08c3"	0.5955
"00074c3272"	"00074f1859"	-0.0811
"00074c3272"	"00074f2268"	0.0332
"00074c3272"	"00074f28be"	0.3442
"00074c3272"	"00074f294b"	-0.0395
"00074c3272"	"00074f2ddd"	-0.0763
"00074c3272"	"00074f2e75"	-0.4306
"00074c3272"	"00074f3088"	-0.052
"00074c3272"	"00074f5a1c"	-0.0159
"00074c3272"	"00074f75b7"	-0.0341
"00074c3272"	"00074f8cd9"	-0.2102
"00074c3272"	"00074f96dc"	0.0015
"00074c3272"	"00074fabaa"	0.1989
"00074c3272"	"00074facd9"	0.0071
"00074c3272"	"00074fae3c"	0.0936

"00074c3272"	"00074fb0a8"	0.1651
"00074c3272"	"00074fb4e4"	0.1405
"00074c3272"	"00074fb7c2"	0.1173
"00074c3272"	"00074fbd36"	-0.0851
"00074c3272"	"00074fc27f"	0.1343
"00074c3272"	"00074fc31d"	0.01
"00074c3272"	"00074fd569"	0.0667
"00074c3272"	"00074fef15"	0.014
"00074c3272"	"00074ff562"	-0.2489
"00074c3272"	"00075007ca"	0.0012
"00074c3272"	"0007500b86"	0.0855
"00074c3272"	"0007500d05"	-0.2472
"00074c3272"	"0007500ee4"	-0.1847
"00074c3272"	"0007500eee"	-0.1182
"00074c3272"	"00075013dc"	0.1825
"00074c3272"	"000757b515"	0.2524
"00074c3272"	"000757bc5a"	0.3813
"00074c3272"	"000757c320"	0.163
"00074c3272"	"000757c9aa"	0.6908
"00074c3272"	"000757ccbe"	-0.0142
"00074c3272"	"000757cfa9"	-0.1147
"00074c3272"	"000757d390"	0.007
"00074c3272"	"000757d393"	-0.0242
"00074c3272"	"000757d598"	0.1928
"00074c3272"	"000757d5a2"	0.025
"00074c3272"	"000757d790"	-0.0722
"00074c3272"	"000757e30c"	0.1471
"00074c3272"	"000757e4b0"	0.6218
"00074c3272"	"000757e7a0"	-0.0437
"00074c3272"	"000757e8b3"	0.3043
"00074c3272"	"000757f627"	0.2082
"00074c3272"	"000757f925"	0.0257
"00074c3272"	"000757fa08"	-0.273
"00074c3272"	"000757fe52"	-0.0958
"00074c3272"	"000758024a"	0.068
"00074c3272"	"00075804bb"	0.205
"00074c3272"	"00075a0c04"	0.0275
"00074c3272"	"00075a3110"	0.1491
"00074c3272"	"00075a341a"	-0.25
"00074c3272"	"00075a3dcf"	-0.1116
"00074c3272"	"00075a3e22"	0.1878
"00074c3272"	"00075a48d8"	-0.1043
"00074c3272"	"00075a5cfb"	0.1818
"00074c3272"	"00075a6151"	0.0379
"00074c3272"	"00075a6708"	0.0417
"00074c3272"	"00075a7319"	0.0716
"00074c3272"	"00075a7723"	0.0774
"00074c3272"	"00075a778b"	-0.27
"00074c3272"	"00075a7b8e"	0.2625
"00074c3272"	"00075a7c79"	0.2127

"00074c3272"	"00075a81b6"	0.1326
"00074c3272"	"00075a82ac"	0.0253
"00074c3272"	"00075a98e5"	-0.2032
"00074c3272"	"00075b0d29"	-0.1287
"00074c3272"	"00075b102a"	0.1717
"00074c3272"	"00075b1074"	-0.0574
"00074c3272"	"00075b135d"	0.0272
"00074c3272"	"00075b138b"	0.4846
"00074c3272"	"00075b13a0"	-0.3093
"00074c3272"	"00075b13bd"	0.0618
"00074c3272"	"00075b16a9"	-0.2839
"00074c3272"	"00075b1a28"	-0.2594
"00074c3272"	"00075b1a97"	-0.0022
"00074c3272"	"00075b1c7b"	-0.4371
"00074c3272"	"00075b1d24"	0.1425
"00074c3272"	"00075b202b"	0.3661
"00074c3272"	"00075b22cb"	-0.255
"00074c3272"	"00075b22da"	0.115
"00074c3272"	"00075b2556"	0.0856
"00074c3272"	"00075b25de"	0.1646
"00074c3272"	"00075b260c"	0.3094
"00074c3272"	"00075b26f1"	0.2286
"00074c3272"	"00075b2920"	0.3271
"00074c3272"	"00075b2a64"	0.1804
"00074c3272"	"00075b2a9d"	0.3058
"00074c3272"	"00075b2b37"	-0.2811
"00074c3272"	"00075b2cdd"	-0.1997
"00074c3272"	"00075b3038"	0.1546
"00074c3272"	"00075b30fe"	-0.0276
"00074c3272"	"00075b3362"	0.0407
"00074c3272"	"00075b350a"	0.2062
"00074c3272"	"00075b350e"	0.0354
"00074c3272"	"00075b3651"	-0.0764
"00074c3272"	"00075b38ca"	-0.2089
"00074c3272"	"00075b39cc"	-0.016
"00074c3272"	"00075b3e1e"	-0.0195
"00074c3272"	"00075b3e57"	-0.1234
"00074c3272"	"00075b4079"	0.0259
"00074c3272"	"00075b4150"	0.0279
"00074c3272"	"00075b4194"	0.4263
"00074c3272"	"00075b42d5"	-0.0918
"00074c3272"	"00075b4424"	-0.0669
"00074c3272"	"00075b4470"	0.3813
"00074c3272"	"00075b47ed"	-0.0103
"00074c3272"	"00075b4850"	0.5284
"00074c3272"	"00075b4ca0"	0.2954
"00074c3272"	"00075b4d7f"	-0.0601
"00074c3272"	"00075b520f"	0.0324
"00074c3272"	"00075b525f"	0.168
"00074c3272"	"00075b58f8"	-0.1551

"00074c3272"	"00075b5bcc"	0.351
"00074c3272"	"00075b5bfa"	-0.0773
"00074c3272"	"00075b6339"	0.3017
"00074c3272"	"00075b6658"	-0.0904
"00074c3272"	"00075b679a"	0.1168
"00074c3272"	"00075b6cb7"	0.1568
"00074c3272"	"00075b6df8"	-0.1908
"00074c3272"	"00075b6ff6"	0.2142
"00074c3272"	"00075b70ee"	0.0139
"00074c3272"	"00075b7157"	-0.0159
"00074c3272"	"00075b7225"	0.1304
"00074c3272"	"00075b7c89"	-0.0051
"00074c3272"	"00075b9048"	0.1828
"00074c3272"	"00075d0801"	0.0641
"00074c3272"	"00075d1820"	-0.0753
"00074c3272"	"00075d1f3d"	0.5077
"00074c3272"	"00075d2329"	-0.1162
"00074c3272"	"00075d2b9b"	0.4051
"00074c3272"	"00075d3941"	0.0953
"00074c3272"	"00075d3e96"	0.2758
"00074c3272"	"00075d4864"	-0.0936
"00074c3272"	"00075d5961"	-0.2692
"00074c3272"	"00075d5a63"	0.323
"00074c3272"	"00075d6150"	-0.0448
"00074c3272"	"00075d67d0"	0.0656
"00074c3272"	"00075d67e2"	0.0446
"00074c3272"	"00075d73fc"	-0.0803
"00074c3272"	"00075d7729"	0.0959
"00074c3272"	"00075d778c"	-0.0558
"00074c3272"	"00075d7b9e"	0.0148
"00074c3272"	"00075d7c8f"	0.1616
"00074c3272"	"00075d804d"	0.3406
"00074c3272"	"00075d819f"	0.3195
"00074c3272"	"00075d8601"	0.0261
"00074c3272"	"00075d8c6a"	0.0626
"00074c3272"	"00075dfedc"	0.0542
"00074c3272"	"00075e05f2"	-0.003
"00074c3272"	"00075e0837"	0.3216
"00074c3272"	"00075e092e"	0.0143
"00074c3272"	"00075e0965"	-0.0331
"00074c3272"	"00075e0bc8"	0.4854
"00074c3272"	"00075e0fbb"	-0.1725
"00074c33e4"	"00074c35b2"	0.3757
"00074c33e4"	"00074c38cd"	-0.3114
"00074c33e4"	"00074c3a40"	-0.0503
"00074c33e4"	"00074d84fc"	-0.2193
"00074c33e4"	"00074d8639"	-0.2935
"00074c33e4"	"00074d8697"	0.0654
"00074c33e4"	"00074d87af"	0.1016
"00074c33e4"	"00074d8813"	-0.1427

"00074c33e4"	"00074d8817"	0.1894
"00074c33e4"	"00074d8a39"	-0.0898
"00074c33e4"	"00074d8ad8"	-0.0241
"00074c33e4"	"00074d8c26"	0.1967
"00074c33e4"	"00074d8ca5"	0.011
"00074c33e4"	"00074d8e0a"	-0.1312
"00074c33e4"	"00074d9179"	-0.0015
"00074c33e4"	"00074d929f"	-0.0836
"00074c33e4"	"00074d93c4"	-0.056
"00074c33e4"	"00074d93d0"	-0.0072
"00074c33e4"	"00074d945b"	-0.0297
"00074c33e4"	"00074d966c"	-0.1048
"00074c33e4"	"00074d9953"	-0.0546
"00074c33e4"	"00074d99b2"	-0.0684
"00074c33e4"	"00074d99f3"	0.0545
"00074c33e4"	"00074d9afd"	-0.3276
"00074c33e4"	"00074d9c87"	0.2227
"00074c33e4"	"00074d9e9e"	-0.1373
"00074c33e4"	"00074d9f30"	-0.2723
"00074c33e4"	"00074da036"	0.1625
"00074c33e4"	"00074da082"	-0.0033
"00074c33e4"	"00074da136"	-0.2069
"00074c33e4"	"00074da3ed"	-0.1274
"00074c33e4"	"00074da4ac"	0.1487
"00074c33e4"	"00074da4b8"	-0.1576
"00074c33e4"	"00074da5e8"	-0.0693
"00074c33e4"	"00074da6b4"	-0.0997
"00074c33e4"	"00074daa3c"	-0.3278
"00074c33e4"	"00074daaf6"	0.0164
"00074c33e4"	"00074dad20"	-0.3885
"00074c33e4"	"00074db098"	-0.157
"00074c33e4"	"00074db231"	0.0853
"00074c33e4"	"00074db3a3"	0.1071
"00074c33e4"	"00074db5d6"	0.0231
"00074c33e4"	"00074db632"	-0.0252
"00074c33e4"	"00074db688"	-0.0722
"00074c33e4"	"00074db8a6"	0.0355
"00074c33e4"	"00074dba19"	0.4601
"00074c33e4"	"00074dbc2e"	-0.1479
"00074c33e4"	"00074dbe51"	-0.1968
"00074c33e4"	"00074dbe5f"	-0.184
"00074c33e4"	"00074dbf6d"	-0.0704
"00074c33e4"	"00074dc4a5"	-0.2328
"00074c33e4"	"00074dc50c"	-0.219
"00074c33e4"	"00074dcdfa"	-0.301
"00074c33e4"	"00074dcf5f"	-0.225
"00074c33e4"	"00074dd007"	0.0307
"00074c33e4"	"00074dd163"	0.0814
"00074c33e4"	"00074dd3df"	-0.102
"00074c33e4"	"00074dd577"	-0.2066

"00074c33e4"	"00074dd62e"	-0.1496
"00074c33e4"	"00074dd73c"	-0.1991
"00074c33e4"	"00074dda10"	0.1237
"00074c33e4"	"00074ddab8"	-0.0328
"00074c33e4"	"00074ddd3d"	-0.1678
"00074c33e4"	"00074ddf16"	0.0139
"00074c33e4"	"00074ddfc1"	0.0681
"00074c33e4"	"00074de21a"	0.0221
"00074c33e4"	"00074de2a9"	0.0686
"00074c33e4"	"00074de544"	0.1891
"00074c33e4"	"00074de98a"	0.4239
"00074c33e4"	"00074dea7e"	-0.0949
"00074c33e4"	"00074debd9"	-0.1653
"00074c33e4"	"00074deca3"	-0.0631
"00074c33e4"	"00074def43"	-0.2614
"00074c33e4"	"00074def99"	0.0449
"00074c33e4"	"00074ecdad"	-0.4567
"00074c33e4"	"00074ecf28"	0.0782
"00074c33e4"	"00074ed1e1"	-0.2147
"00074c33e4"	"00074ed83b"	0.0099
"00074c33e4"	"00074ee5e3"	-0.061
"00074c33e4"	"00074ee6e0"	-0.1947
"00074c33e4"	"00074eea3a"	-0.1175
"00074c33e4"	"00074eff82"	-0.1739
"00074c33e4"	"00074f0477"	-0.3531
"00074c33e4"	"00074f08c3"	-0.1623
"00074c33e4"	"00074f1859"	0.1294
"00074c33e4"	"00074f2268"	-0.2133
"00074c33e4"	"00074f28be"	-0.0372
"00074c33e4"	"00074f294b"	-0.1413
"00074c33e4"	"00074f2ddd"	-0.0331
"00074c33e4"	"00074f2e75"	-0.3573
"00074c33e4"	"00074f3088"	0.243
"00074c33e4"	"00074f5a1c"	-0.0604
"00074c33e4"	"00074f75b7"	-0.2143
"00074c33e4"	"00074f8cd9"	0.0292
"00074c33e4"	"00074f96dc"	-0.2217
"00074c33e4"	"00074fabaa"	-0.1533
"00074c33e4"	"00074facd9"	-0.011
"00074c33e4"	"00074fae3c"	0.0207
"00074c33e4"	"00074fb0a8"	0.1406
"00074c33e4"	"00074fb4e4"	0.3164
"00074c33e4"	"00074fb7c2"	0.0984
"00074c33e4"	"00074fbd36"	0.3081
"00074c33e4"	"00074fc27f"	-0.0994
"00074c33e4"	"00074fc31d"	0.2561
"00074c33e4"	"00074fd569"	-0.0421
"00074c33e4"	"00074fef15"	0.1844
"00074c33e4"	"00074ff562"	-0.4619
"00074c33e4"	"00075007ca"	-0.3095

"00074c33e4"	"0007500b86"	0.1774
"00074c33e4"	"0007500d05"	-0.1355
"00074c33e4"	"0007500ee4"	-0.0025
"00074c33e4"	"0007500eee"	-0.1017
"00074c33e4"	"00075013dc"	-0.0456
"00074c33e4"	"000757b515"	-0.1174
"00074c33e4"	"000757bc5a"	0.131
"00074c33e4"	"000757c320"	0.2683
"00074c33e4"	"000757c9aa"	-0.0855
"00074c33e4"	"000757ccbe"	0.1141
"00074c33e4"	"000757cfa9"	0.112
"00074c33e4"	"000757d390"	-0.1298
"00074c33e4"	"000757d393"	0.3568
"00074c33e4"	"000757d598"	-0.2329
"00074c33e4"	"000757d5a2"	-0.2927
"00074c33e4"	"000757d790"	0.0888
"00074c33e4"	"000757e30c"	-0.1512
"00074c33e4"	"000757e4b0"	0.0018
"00074c33e4"	"000757e7a0"	-0.3127
"00074c33e4"	"000757e8b3"	-0.0342
"00074c33e4"	"000757f627"	0.4759
"00074c33e4"	"000757f925"	0.1096
"00074c33e4"	"000757fa08"	-0.306
"00074c33e4"	"000757fe52"	-0.1377
"00074c33e4"	"000758024a"	-0.2491
"00074c33e4"	"00075804bb"	-0.0356
"00074c33e4"	"00075a0c04"	-0.1907
"00074c33e4"	"00075a3110"	-0.0897
"00074c33e4"	"00075a341a"	-0.0119
"00074c33e4"	"00075a3dcf"	-0.0095
"00074c33e4"	"00075a3e22"	0.1436
"00074c33e4"	"00075a48d8"	-0.166
"00074c33e4"	"00075a5cfb"	-0.2469
"00074c33e4"	"00075a6151"	-0.0626
"00074c33e4"	"00075a6708"	-0.0513
"00074c33e4"	"00075a7319"	-0.0381
"00074c33e4"	"00075a7723"	-0.0414
"00074c33e4"	"00075a778b"	-0.0641
"00074c33e4"	"00075a7b8e"	-0.1292
"00074c33e4"	"00075a7c79"	-0.1517
"00074c33e4"	"00075a81b6"	-0.2324
"00074c33e4"	"00075a82ac"	0.3001
"00074c33e4"	"00075a98e5"	0.4558
"00074c33e4"	"00075b0d29"	0.3699
"00074c33e4"	"00075b102a"	0.01
"00074c33e4"	"00075b1074"	-0.3613
"00074c33e4"	"00075b135d"	0.009
"00074c33e4"	"00075b138b"	-0.03
"00074c33e4"	"00075b13a0"	-0.1266
"00074c33e4"	"00075b13bd"	0.2125

"00074c33e4"	"00075b16a9"	-0.1065
"00074c33e4"	"00075b1a28"	0.1655
"00074c33e4"	"00075b1a97"	0.1217
"00074c33e4"	"00075b1c7b"	-0.1251
"00074c33e4"	"00075b1d24"	-0.1824
"00074c33e4"	"00075b202b"	-0.1877
"00074c33e4"	"00075b22cb"	0.0269
"00074c33e4"	"00075b22da"	-0.1571
"00074c33e4"	"00075b2556"	-0.0717
"00074c33e4"	"00075b25de"	0.0902
"00074c33e4"	"00075b260c"	-0.3551
"00074c33e4"	"00075b26f1"	-0.2202
"00074c33e4"	"00075b2920"	0.0156
"00074c33e4"	"00075b2a64"	-0.4322
"00074c33e4"	"00075b2a9d"	-0.1534
"00074c33e4"	"00075b2b37"	-0.1
"00074c33e4"	"00075b2cdd"	-0.102
"00074c33e4"	"00075b3038"	0.0059
"00074c33e4"	"00075b30fe"	-0.0793
"00074c33e4"	"00075b3362"	-0.3194
"00074c33e4"	"00075b350a"	-0.158
"00074c33e4"	"00075b350e"	0.2437
"00074c33e4"	"00075b3651"	-0.1715
"00074c33e4"	"00075b38ca"	-0.1522
"00074c33e4"	"00075b39cc"	-0.1151
"00074c33e4"	"00075b3e1e"	0.1346
"00074c33e4"	"00075b3e57"	0.1146
"00074c33e4"	"00075b4079"	-0.0386
"00074c33e4"	"00075b4150"	-0.1326
"00074c33e4"	"00075b4194"	0.0288
"00074c33e4"	"00075b42d5"	-0.0583
"00074c33e4"	"00075b4424"	-0.1928
"00074c33e4"	"00075b4470"	0.131
"00074c33e4"	"00075b47ed"	-0.1778
"00074c33e4"	"00075b4850"	7e-04
"00074c33e4"	"00075b4ca0"	-0.1979
"00074c33e4"	"00075b4d7f"	0.0361
"00074c33e4"	"00075b520f"	0.1545
"00074c33e4"	"00075b525f"	-0.1607
"00074c33e4"	"00075b58f8"	-0.1698
"00074c33e4"	"00075b5bcc"	-0.1474
"00074c33e4"	"00075b5bfa"	-0.3937
"00074c33e4"	"00075b6339"	-0.198
"00074c33e4"	"00075b6658"	-0.1596
"00074c33e4"	"00075b679a"	-0.0512
"00074c33e4"	"00075b6cb7"	-0.1997
"00074c33e4"	"00075b6df8"	-0.0407
"00074c33e4"	"00075b6ff6"	-0.1922
"00074c33e4"	"00075b70ee"	-0.2212
"00074c33e4"	"00075b7157"	-0.0381

"00074c33e4"	"00075b7225"	0.0658
"00074c33e4"	"00075b7c89"	0.1373
"00074c33e4"	"00075b9048"	0.0367
"00074c33e4"	"00075d0801"	0.126
"00074c33e4"	"00075d1820"	0.0354
"00074c33e4"	"00075d1f3d"	−0.3331
"00074c33e4"	"00075d2329"	−0.0975
"00074c33e4"	"00075d2b9b"	−0.3546
"00074c33e4"	"00075d3941"	−0.2645
"00074c33e4"	"00075d3e96"	−0.1474
"00074c33e4"	"00075d4864"	0.1751
"00074c33e4"	"00075d5961"	0.2554
"00074c33e4"	"00075d5a63"	−0.2114
"00074c33e4"	"00075d6150"	−0.2495
"00074c33e4"	"00075d67d0"	−0.4515
"00074c33e4"	"00075d67e2"	0.1038
"00074c33e4"	"00075d73fc"	0.1444
"00074c33e4"	"00075d7729"	−0.2572
"00074c33e4"	"00075d778c"	−0.4487
"00074c33e4"	"00075d7b9e"	−0.0134
"00074c33e4"	"00075d7c8f"	−0.0341
"00074c33e4"	"00075d804d"	−0.0204
"00074c33e4"	"00075d819f"	0.0642
"00074c33e4"	"00075d8601"	0.1175
"00074c33e4"	"00075d8c6a"	0.0289
"00074c33e4"	"00075dfedc"	−0.1739
"00074c33e4"	"00075e05f2"	−0.0816
"00074c33e4"	"00075e0837"	−0.1729
"00074c33e4"	"00075e092e"	−0.049
"00074c33e4"	"00075e0965"	−0.4039
"00074c33e4"	"00075e0bc8"	0.0777
"00074c33e4"	"00075e0fbb"	−0.1199
"00074c35b2"	"00074c38cd"	−0.1322
"00074c35b2"	"00074c3a40"	−0.4918
"00074c35b2"	"00074d84fc"	−0.1002
"00074c35b2"	"00074d8639"	−0.2049
"00074c35b2"	"00074d8697"	0.3445
"00074c35b2"	"00074d87af"	−0.1172
"00074c35b2"	"00074d8813"	−0.0828
"00074c35b2"	"00074d8817"	0.0092
"00074c35b2"	"00074d8a39"	−0.1503
"00074c35b2"	"00074d8ad8"	0.1274
"00074c35b2"	"00074d8c26"	0.2897
"00074c35b2"	"00074d8ca5"	−0.2025
"00074c35b2"	"00074d8e0a"	0.1719
"00074c35b2"	"00074d9179"	−0.1821
"00074c35b2"	"00074d929f"	−0.1265
"00074c35b2"	"00074d93c4"	−0.0873
"00074c35b2"	"00074d93d0"	−0.054
"00074c35b2"	"00074d945b"	−0.0462

"00074c35b2"	"00074d966c"	0.1274
"00074c35b2"	"00074d9953"	0.1331
"00074c35b2"	"00074d99b2"	0.1171
"00074c35b2"	"00074d99f3"	-0.0213
"00074c35b2"	"00074d9afd"	-0.4798
"00074c35b2"	"00074d9c87"	0.3598
"00074c35b2"	"00074d9e9e"	-0.3534
"00074c35b2"	"00074d9f30"	-0.3438
"00074c35b2"	"00074da036"	0.3694
"00074c35b2"	"00074da082"	-0.2165
"00074c35b2"	"00074da136"	-0.1384
"00074c35b2"	"00074da3ed"	-0.0325
"00074c35b2"	"00074da4ac"	-0.0345
"00074c35b2"	"00074da4b8"	-0.2395
"00074c35b2"	"00074da5e8"	0.241
"00074c35b2"	"00074da6b4"	0.016
"00074c35b2"	"00074daa3c"	0.0835
"00074c35b2"	"00074daaf6"	-0.1649
"00074c35b2"	"00074dad20"	-0.2844
"00074c35b2"	"00074db098"	-0.1642
"00074c35b2"	"00074db231"	0.312
"00074c35b2"	"00074db3a3"	0.2461
"00074c35b2"	"00074db5d6"	-0.0271
"00074c35b2"	"00074db632"	0.0898
"00074c35b2"	"00074db688"	0.0962
"00074c35b2"	"00074db8a6"	-0.0771
"00074c35b2"	"00074dba19"	0.5536
"00074c35b2"	"00074dbc2e"	-0.1351
"00074c35b2"	"00074dbe51"	0.0077
"00074c35b2"	"00074dbe5f"	-0.3117
"00074c35b2"	"00074dbf6d"	0.0552
"00074c35b2"	"00074dc4a5"	-0.3997
"00074c35b2"	"00074dc50c"	0.0463
"00074c35b2"	"00074dcdfa"	-0.054
"00074c35b2"	"00074dcf5f"	-0.386
"00074c35b2"	"00074dd007"	-0.0505
"00074c35b2"	"00074dd163"	0.064
"00074c35b2"	"00074dd3df"	-0.1361
"00074c35b2"	"00074dd577"	-0.0242
"00074c35b2"	"00074dd62e"	-0.0495
"00074c35b2"	"00074dd73c"	-0.0567
"00074c35b2"	"00074dda10"	0.0168
"00074c35b2"	"00074ddab8"	0.2035
"00074c35b2"	"00074ddd3d"	-0.1427
"00074c35b2"	"00074ddf16"	-0.1509
"00074c35b2"	"00074ddfc1"	-0.0349
"00074c35b2"	"00074de21a"	-0.0626
"00074c35b2"	"00074de2a9"	0.1032
"00074c35b2"	"00074de544"	0.165
"00074c35b2"	"00074de98a"	0.0583

"00074c35b2"	"00074dea7e"	-0.0097
"00074c35b2"	"00074debd9"	-0.1118
"00074c35b2"	"00074deca3"	0.0147
"00074c35b2"	"00074def43"	-0.2824
"00074c35b2"	"00074def99"	-0.1546
"00074c35b2"	"00074ecdad"	-0.5221
"00074c35b2"	"00074ecf28"	-0.1411
"00074c35b2"	"00074ed1e1"	-0.1311
"00074c35b2"	"00074ed83b"	0.0232
"00074c35b2"	"00074ee5e3"	-0.0748
"00074c35b2"	"00074ee6e0"	0.0774
"00074c35b2"	"00074eea3a"	0.1899
"00074c35b2"	"00074eff82"	-0.136
"00074c35b2"	"00074f0477"	-0.0897
"00074c35b2"	"00074f08c3"	0.1072
"00074c35b2"	"00074f1859"	0.0286
"00074c35b2"	"00074f2268"	0.1042
"00074c35b2"	"00074f28be"	0.0897
"00074c35b2"	"00074f294b"	-0.084
"00074c35b2"	"00074f2ddd"	0.1972
"00074c35b2"	"00074f2e75"	-0.3419
"00074c35b2"	"00074f3088"	0.1337
"00074c35b2"	"00074f5a1c"	0.0893
"00074c35b2"	"00074f75b7"	-0.1044
"00074c35b2"	"00074f8cd9"	-0.1005
"00074c35b2"	"00074f96dc"	-0.1564
"00074c35b2"	"00074fabaa"	0.2697
"00074c35b2"	"00074facd9"	0.0154
"00074c35b2"	"00074fae3c"	-0.0749
"00074c35b2"	"00074fb0a8"	0.1257
"00074c35b2"	"00074fb4e4"	-0.1141
"00074c35b2"	"00074fb7c2"	0.2857
"00074c35b2"	"00074fbd36"	0.2059
"00074c35b2"	"00074fc27f"	-0.003
"00074c35b2"	"00074fc31d"	0.1056
"00074c35b2"	"00074fd569"	-0.0584
"00074c35b2"	"00074fef15"	-0.0035
"00074c35b2"	"00074ff562"	-0.2275
"00074c35b2"	"00075007ca"	-0.2599
"00074c35b2"	"0007500b86"	0.1319
"00074c35b2"	"0007500d05"	-0.0915
"00074c35b2"	"0007500ee4"	-0.1221
"00074c35b2"	"0007500eee"	-0.131
"00074c35b2"	"00075013dc"	0.037
"00074c35b2"	"000757b515"	0.0854
"00074c35b2"	"000757bc5a"	0.155
"00074c35b2"	"000757c320"	0.1303
"00074c35b2"	"000757c9aa"	0.1682
"00074c35b2"	"000757ccbe"	0.1751
"00074c35b2"	"000757cfa9"	0.117

"00074c35b2"	"000757d390"	-0.2771
"00074c35b2"	"000757d393"	0.3106
"00074c35b2"	"000757d598"	-0.1771
"00074c35b2"	"000757d5a2"	-0.1308
"00074c35b2"	"000757d790"	0.1454
"00074c35b2"	"000757e30c"	-0.0694
"00074c35b2"	"000757e4b0"	0.2348
"00074c35b2"	"000757e7a0"	-0.2469
"00074c35b2"	"000757e8b3"	0.1558
"00074c35b2"	"000757f627"	-0.115
"00074c35b2"	"000757f925"	0.176
"00074c35b2"	"000757fa08"	-0.2191
"00074c35b2"	"000757fe52"	0.0426
"00074c35b2"	"000758024a"	-0.3492
"00074c35b2"	"00075804bb"	0.051
"00074c35b2"	"00075a0c04"	0.0121
"00074c35b2"	"00075a3110"	0.3114
"00074c35b2"	"00075a341a"	-0.0358
"00074c35b2"	"00075a3dcf"	0.0898
"00074c35b2"	"00075a3e22"	0.1963
"00074c35b2"	"00075a48d8"	-0.0211
"00074c35b2"	"00075a5cfb"	-0.1207
"00074c35b2"	"00075a6151"	-0.1034
"00074c35b2"	"00075a6708"	-0.1723
"00074c35b2"	"00075a7319"	-0.0772
"00074c35b2"	"00075a7723"	0.0608
"00074c35b2"	"00075a778b"	-0.1876
"00074c35b2"	"00075a7b8e"	-0.1218
"00074c35b2"	"00075a7c79"	-0.0291
"00074c35b2"	"00075a81b6"	-0.2462
"00074c35b2"	"00075a82ac"	0.1975
"00074c35b2"	"00075a98e5"	0.0132
"00074c35b2"	"00075b0d29"	0.1606
"00074c35b2"	"00075b102a"	-0.0988
"00074c35b2"	"00075b1074"	-0.1009
"00074c35b2"	"00075b135d"	0.2175
"00074c35b2"	"00075b138b"	0.1426
"00074c35b2"	"00075b13a0"	0.0064
"00074c35b2"	"00075b13bd"	0.1407
"00074c35b2"	"00075b16a9"	-0.2669
"00074c35b2"	"00075b1a28"	-0.0499
"00074c35b2"	"00075b1a97"	-0.0824
"00074c35b2"	"00075b1c7b"	-0.4276
"00074c35b2"	"00075b1d24"	0.0382
"00074c35b2"	"00075b202b"	-0.0114
"00074c35b2"	"00075b22cb"	-0.2746
"00074c35b2"	"00075b22da"	-0.049
"00074c35b2"	"00075b2556"	0.0877
"00074c35b2"	"00075b25de"	0.1304
"00074c35b2"	"00075b260c"	-0.0371

"00074c35b2"	"00075b26f1"	-0.1327
"00074c35b2"	"00075b2920"	-0.0903
"00074c35b2"	"00075b2a64"	-0.0468
"00074c35b2"	"00075b2a9d"	0.1137
"00074c35b2"	"00075b2b37"	-0.2491
"00074c35b2"	"00075b2cdd"	-0.0555
"00074c35b2"	"00075b3038"	0.111
"00074c35b2"	"00075b30fe"	0.0053
"00074c35b2"	"00075b3362"	-0.1588
"00074c35b2"	"00075b350a"	0.0669
"00074c35b2"	"00075b350e"	0.1284
"00074c35b2"	"00075b3651"	-0.144
"00074c35b2"	"00075b38ca"	0.0148
"00074c35b2"	"00075b39cc"	0.0183
"00074c35b2"	"00075b3e1e"	0.0987
"00074c35b2"	"00075b3e57"	-0.0366
"00074c35b2"	"00075b4079"	0.1078
"00074c35b2"	"00075b4150"	-0.2089
"00074c35b2"	"00075b4194"	0.214
"00074c35b2"	"00075b42d5"	-0.031
"00074c35b2"	"00075b4424"	-0.1847
"00074c35b2"	"00075b4470"	0.155
"00074c35b2"	"00075b47ed"	0.0566
"00074c35b2"	"00075b4850"	0.2083
"00074c35b2"	"00075b4ca0"	0.0499
"00074c35b2"	"00075b4d7f"	-0.0229
"00074c35b2"	"00075b520f"	0.0652
"00074c35b2"	"00075b525f"	-0.0599
"00074c35b2"	"00075b58f8"	-0.0145
"00074c35b2"	"00075b5bcc"	-0.016
"00074c35b2"	"00075b5bfa"	-0.2044
"00074c35b2"	"00075b6339"	0.0049
"00074c35b2"	"00075b6658"	-0.0655
"00074c35b2"	"00075b679a"	-0.2291
"00074c35b2"	"00075b6cb7"	-0.0064
"00074c35b2"	"00075b6df8"	-0.0777
"00074c35b2"	"00075b6ff6"	3e-04
"00074c35b2"	"00075b70ee"	-0.2595
"00074c35b2"	"00075b7157"	0.0879
"00074c35b2"	"00075b7225"	0.0188
"00074c35b2"	"00075b7c89"	0.1414
"00074c35b2"	"00075b9048"	0.1858
"00074c35b2"	"00075d0801"	0.2965
"00074c35b2"	"00075d1820"	-0.1806
"00074c35b2"	"00075d1f3d"	0.0453
"00074c35b2"	"00075d2329"	0.1328
"00074c35b2"	"00075d2b9b"	0.08
"00074c35b2"	"00075d3941"	-0.0854
"00074c35b2"	"00075d3e96"	-0.1085
"00074c35b2"	"00075d4864"	0.0747

"00074c35b2"	"00075d5961"	0.0666
"00074c35b2"	"00075d5a63"	0.0156
"00074c35b2"	"00075d6150"	−0.3159
"00074c35b2"	"00075d67d0"	−0.141
"00074c35b2"	"00075d67e2"	−0.0962
"00074c35b2"	"00075d73fc"	−0.1144
"00074c35b2"	"00075d7729"	−0.2092
"00074c35b2"	"00075d778c"	−0.2412
"00074c35b2"	"00075d7b9e"	0.073
"00074c35b2"	"00075d7c8f"	0.1973
"00074c35b2"	"00075d804d"	0.1418
"00074c35b2"	"00075d819f"	0.3614
"00074c35b2"	"00075d8601"	0.0753
"00074c35b2"	"00075d8c6a"	0.0959
"00074c35b2"	"00075dfedc"	−0.0619
"00074c35b2"	"00075e05f2"	−0.0822
"00074c35b2"	"00075e0837"	0.0977
"00074c35b2"	"00075e092e"	0.1143
"00074c35b2"	"00075e0965"	−0.0833
"00074c35b2"	"00075e0bc8"	0.2579
"00074c35b2"	"00075e0fbb"	0.0867
"00074c38cd"	"00074c3a40"	−0.3921
"00074c38cd"	"00074d84fc"	−0.1627
"00074c38cd"	"00074d8639"	0.4208
"00074c38cd"	"00074d8697"	0.0026
"00074c38cd"	"00074d87af"	−0.0629
"00074c38cd"	"00074d8813"	−0.2218
"00074c38cd"	"00074d8817"	−0.2185
"00074c38cd"	"00074d8a39"	−0.3479
"00074c38cd"	"00074d8ad8"	0.074
"00074c38cd"	"00074d8c26"	−0.2212
"00074c38cd"	"00074d8ca5"	−0.1399
"00074c38cd"	"00074d8e0a"	−0.1105
"00074c38cd"	"00074d9179"	−0.3523
"00074c38cd"	"00074d929f"	−0.0949
"00074c38cd"	"00074d93c4"	−0.1087
"00074c38cd"	"00074d93d0"	0.0896
"00074c38cd"	"00074d945b"	−0.3371
"00074c38cd"	"00074d966c"	−0.1064
"00074c38cd"	"00074d9953"	−0.0279
"00074c38cd"	"00074d99b2"	−0.0314
"00074c38cd"	"00074d99f3"	−0.148
"00074c38cd"	"00074d9afd"	−0.3093
"00074c38cd"	"00074d9c87"	0.0321
"00074c38cd"	"00074d9e9e"	0.041
"00074c38cd"	"00074d9f30"	−0.4068
"00074c38cd"	"00074da036"	0.204
"00074c38cd"	"00074da082"	−0.2256
"00074c38cd"	"00074da136"	0.3104
"00074c38cd"	"00074da3ed"	−0.074

"00074c38cd"	"00074da4ac"	0.1075
"00074c38cd"	"00074da4b8"	−0.2891
"00074c38cd"	"00074da5e8"	0.0857
"00074c38cd"	"00074da6b4"	0.0142
"00074c38cd"	"00074daa3c"	−0.5175
"00074c38cd"	"00074daaf6"	−0.4131
"00074c38cd"	"00074dad20"	−0.151
"00074c38cd"	"00074db098"	−0.2236
"00074c38cd"	"00074db231"	0.1637
"00074c38cd"	"00074db3a3"	−0.5336
"00074c38cd"	"00074db5d6"	−0.2945
"00074c38cd"	"00074db632"	0.1198
"00074c38cd"	"00074db688"	0.1312
"00074c38cd"	"00074db8a6"	−0.3205
"00074c38cd"	"00074dba19"	0.0062
"00074c38cd"	"00074dbc2e"	−0.1657
"00074c38cd"	"00074dbe51"	0.1463
"00074c38cd"	"00074dbe5f"	−0.1596
"00074c38cd"	"00074dbf6d"	−0.6287
"00074c38cd"	"00074dc4a5"	−0.3168
"00074c38cd"	"00074dc50c"	−0.1906
"00074c38cd"	"00074dcdfa"	−0.1429
"00074c38cd"	"00074dcf5f"	−0.2867
"00074c38cd"	"00074dd007"	−0.344
"00074c38cd"	"00074dd163"	−0.2335
"00074c38cd"	"00074dd3df"	−0.4634
"00074c38cd"	"00074dd577"	−0.0401
"00074c38cd"	"00074dd62e"	−0.3382
"00074c38cd"	"00074dd73c"	−0.0633
"00074c38cd"	"00074dda10"	−0.0597
"00074c38cd"	"00074ddab8"	−0.2399
"00074c38cd"	"00074ddd3d"	−0.3287
"00074c38cd"	"00074ddf16"	−0.1289
"00074c38cd"	"00074ddfc1"	0.0698
"00074c38cd"	"00074de21a"	−0.3114
"00074c38cd"	"00074de2a9"	−0.3443
"00074c38cd"	"00074de544"	0.0824
"00074c38cd"	"00074de98a"	−0.2164
"00074c38cd"	"00074dea7e"	−0.1701
"00074c38cd"	"00074debd9"	−0.3511
"00074c38cd"	"00074deca3"	−0.6155
"00074c38cd"	"00074def43"	−0.3476
"00074c38cd"	"00074def99"	−0.1699
"00074c38cd"	"00074ecdad"	−0.538
"00074c38cd"	"00074ecf28"	−0.7567
"00074c38cd"	"00074ed1e1"	−0.0844
"00074c38cd"	"00074ed83b"	−0.1652
"00074c38cd"	"00074ee5e3"	−0.2742
"00074c38cd"	"00074ee6e0"	−0.3413
"00074c38cd"	"00074eea3a"	−9e−04

"00074c38cd"	"00074eff82"	0.0023
"00074c38cd"	"00074f0477"	-0.1483
"00074c38cd"	"00074f08c3"	-0.3719
"00074c38cd"	"00074f1859"	-0.1145
"00074c38cd"	"00074f2268"	-0.2526
"00074c38cd"	"00074f28be"	-0.0087
"00074c38cd"	"00074f294b"	-0.1235
"00074c38cd"	"00074f2ddd"	-0.2523
"00074c38cd"	"00074f2e75"	0.1868
"00074c38cd"	"00074f3088"	0.1645
"00074c38cd"	"00074f5a1c"	-0.0182
"00074c38cd"	"00074f75b7"	0.0623
"00074c38cd"	"00074f8cd9"	0.1814
"00074c38cd"	"00074f96dc"	-0.0964
"00074c38cd"	"00074fabaa"	-0.34
"00074c38cd"	"00074facd9"	-0.0829
"00074c38cd"	"00074fae3c"	-0.1768
"00074c38cd"	"00074fb0a8"	-0.2042
"00074c38cd"	"00074fb4e4"	-0.3392
"00074c38cd"	"00074fb7c2"	0.0706
"00074c38cd"	"00074fbd36"	-0.1272
"00074c38cd"	"00074fc27f"	-0.4049
"00074c38cd"	"00074fc31d"	-0.2508
"00074c38cd"	"00074fd569"	-0.3823
"00074c38cd"	"00074fef15"	-0.3165
"00074c38cd"	"00074ff562"	-0.3549
"00074c38cd"	"00075007ca"	-0.3939
"00074c38cd"	"0007500b86"	-0.021
"00074c38cd"	"0007500d05"	0.0037
"00074c38cd"	"0007500ee4"	-0.1524
"00074c38cd"	"0007500eee"	-0.2231
"00074c38cd"	"00075013dc"	-0.4589
"00074c38cd"	"000757b515"	0.0303
"00074c38cd"	"000757bc5a"	-0.0338
"00074c38cd"	"000757c320"	-0.7251
"00074c38cd"	"000757c9aa"	-0.356
"00074c38cd"	"000757ccbe"	-0.0964
"00074c38cd"	"000757cfa9"	-0.2199
"00074c38cd"	"000757d390"	-0.38
"00074c38cd"	"000757d393"	-0.1516
"00074c38cd"	"000757d598"	-0.5932
"00074c38cd"	"000757d5a2"	-0.4166
"00074c38cd"	"000757d790"	-0.2163
"00074c38cd"	"000757e30c"	-0.1478
"00074c38cd"	"000757e4b0"	-0.1304
"00074c38cd"	"000757e7a0"	-0.069
"00074c38cd"	"000757e8b3"	-0.0352
"00074c38cd"	"000757f627"	-0.3014
"00074c38cd"	"000757f925"	-0.1266
"00074c38cd"	"000757fa08"	0.3238

"00074c38cd"	"000757fe52"	−0.022
"00074c38cd"	"000758024a"	−0.2021
"00074c38cd"	"00075804bb"	−0.2452
"00074c38cd"	"00075a0c04"	0.09
"00074c38cd"	"00075a3110"	−0.1525
"00074c38cd"	"00075a341a"	−0.1489
"00074c38cd"	"00075a3dcf"	0.0753
"00074c38cd"	"00075a3e22"	−0.0829
"00074c38cd"	"00075a48d8"	0.1831
"00074c38cd"	"00075a5cfb"	−0.2323
"00074c38cd"	"00075a6151"	−0.0144
"00074c38cd"	"00075a6708"	−0.1086
"00074c38cd"	"00075a7319"	−0.3065
"00074c38cd"	"00075a7723"	0.1549
"00074c38cd"	"00075a778b"	−0.1777
"00074c38cd"	"00075a7b8e"	0.2
"00074c38cd"	"00075a7c79"	−0.0794
"00074c38cd"	"00075a81b6"	0.2207
"00074c38cd"	"00075a82ac"	0.0116
"00074c38cd"	"00075a98e5"	−0.2525
"00074c38cd"	"00075b0d29"	0.2003
"00074c38cd"	"00075b102a"	−0.2458
"00074c38cd"	"00075b1074"	−0.0174
"00074c38cd"	"00075b135d"	0.1359
"00074c38cd"	"00075b138b"	−0.3375
"00074c38cd"	"00075b13a0"	−0.3017
"00074c38cd"	"00075b13bd"	0.0526
"00074c38cd"	"00075b16a9"	−0.1113
"00074c38cd"	"00075b1a28"	−0.3333
"00074c38cd"	"00075b1a97"	−0.3956
"00074c38cd"	"00075b1c7b"	−0.4495
"00074c38cd"	"00075b1d24"	−0.2086
"00074c38cd"	"00075b202b"	−0.2719
"00074c38cd"	"00075b22cb"	−0.4728
"00074c38cd"	"00075b22da"	−0.1278
"00074c38cd"	"00075b2556"	−0.2234
"00074c38cd"	"00075b25de"	0.0967
"00074c38cd"	"00075b260c"	−0.4711
"00074c38cd"	"00075b26f1"	−0.1621
"00074c38cd"	"00075b2920"	−0.2786
"00074c38cd"	"00075b2a64"	−0.3977
"00074c38cd"	"00075b2a9d"	−0.3472
"00074c38cd"	"00075b2b37"	−0.5067
"00074c38cd"	"00075b2cdd"	−0.3076
"00074c38cd"	"00075b3038"	0.0639
"00074c38cd"	"00075b30fe"	−0.2751
"00074c38cd"	"00075b3362"	−0.2761
"00074c38cd"	"00075b350a"	−0.3818
"00074c38cd"	"00075b350e"	−0.3579
"00074c38cd"	"00075b3651"	−0.0887

"00074c38cd"	"00075b38ca"	0.188
"00074c38cd"	"00075b39cc"	0.0055
"00074c38cd"	"00075b3e1e"	−0.1297
"00074c38cd"	"00075b3e57"	−0.3495
"00074c38cd"	"00075b4079"	−0.1482
"00074c38cd"	"00075b4150"	−0.2643
"00074c38cd"	"00075b4194"	−0.1513
"00074c38cd"	"00075b42d5"	0.1719
"00074c38cd"	"00075b4424"	−0.2354
"00074c38cd"	"00075b4470"	−0.0338
"00074c38cd"	"00075b47ed"	0.0611
"00074c38cd"	"00075b4850"	−0.205
"00074c38cd"	"00075b4ca0"	0.0072
"00074c38cd"	"00075b4d7f"	0.5258
"00074c38cd"	"00075b520f"	−0.1508
"00074c38cd"	"00075b525f"	−0.296
"00074c38cd"	"00075b58f8"	0.0403
"00074c38cd"	"00075b5bcc"	−0.1747
"00074c38cd"	"00075b5bfa"	−0.1892
"00074c38cd"	"00075b6339"	0.1547
"00074c38cd"	"00075b6658"	−0.2631
"00074c38cd"	"00075b679a"	−0.3555
"00074c38cd"	"00075b6cb7"	−0.2259
"00074c38cd"	"00075b6df8"	0.3908
"00074c38cd"	"00075b6ff6"	−0.3859
"00074c38cd"	"00075b70ee"	0.0446
"00074c38cd"	"00075b7157"	0.1055
"00074c38cd"	"00075b7225"	−0.2013
"00074c38cd"	"00075b7c89"	0.1174
"00074c38cd"	"00075b9048"	−0.2357
"00074c38cd"	"00075d0801"	0.0225
"00074c38cd"	"00075d1820"	−0.1457
"00074c38cd"	"00075d1f3d"	−0.3006
"00074c38cd"	"00075d2329"	0.1883
"00074c38cd"	"00075d2b9b"	−0.5085
"00074c38cd"	"00075d3941"	−0.3516
"00074c38cd"	"00075d3e96"	−0.1051
"00074c38cd"	"00075d4864"	−0.0996
"00074c38cd"	"00075d5961"	0.1532
"00074c38cd"	"00075d5a63"	−0.1397
"00074c38cd"	"00075d6150"	−0.3294
"00074c38cd"	"00075d67d0"	−0.5901
"00074c38cd"	"00075d67e2"	−0.1756
"00074c38cd"	"00075d73fc"	−0.1052
"00074c38cd"	"00075d7729"	−0.0223
"00074c38cd"	"00075d778c"	−0.2336
"00074c38cd"	"00075d7b9e"	−0.1537
"00074c38cd"	"00075d7c8f"	−0.0165
"00074c38cd"	"00075d804d"	−0.2115
"00074c38cd"	"00075d819f"	−0.397

"00074c38cd"	"00075d8601"	0.0242
"00074c38cd"	"00075d8c6a"	0.5179
"00074c38cd"	"00075dfedc"	0.1146
"00074c38cd"	"00075e05f2"	−0.1447
"00074c38cd"	"00075e0837"	−0.1288
"00074c38cd"	"00075e092e"	−0.157
"00074c38cd"	"00075e0965"	−0.0111
"00074c38cd"	"00075e0bc8"	−0.0244
"00074c38cd"	"00075e0fbb"	−0.0985
"00074c3a40"	"00074d84fc"	−0.518
"00074c3a40"	"00074d8639"	−0.3491
"00074c3a40"	"00074d8697"	−0.4636
"00074c3a40"	"00074d87af"	−0.0447
"00074c3a40"	"00074d8813"	−0.1823
"00074c3a40"	"00074d8817"	−0.0254
"00074c3a40"	"00074d8a39"	−0.0025
"00074c3a40"	"00074d8ad8"	−0.49
"00074c3a40"	"00074d8c26"	−0.3039
"00074c3a40"	"00074d8ca5"	−0.3999
"00074c3a40"	"00074d8e0a"	−0.3564
"00074c3a40"	"00074d9179"	−0.4093
"00074c3a40"	"00074d929f"	−0.1547
"00074c3a40"	"00074d93c4"	−0.1651
"00074c3a40"	"00074d93d0"	−0.5073
"00074c3a40"	"00074d945b"	−0.4014
"00074c3a40"	"00074d966c"	−0.471
"00074c3a40"	"00074d9953"	−0.3969
"00074c3a40"	"00074d99b2"	−0.3935
"00074c3a40"	"00074d99f3"	−0.3351
"00074c3a40"	"00074d9afd"	−0.429
"00074c3a40"	"00074d9c87"	−0.4814
"00074c3a40"	"00074d9e9e"	−0.3189
"00074c3a40"	"00074d9f30"	−0.1993
"00074c3a40"	"00074da036"	−0.4827
"00074c3a40"	"00074da082"	−0.5821
"00074c3a40"	"00074da136"	−0.3686
"00074c3a40"	"00074da3ed"	−0.2991
"00074c3a40"	"00074da4ac"	0.0877
"00074c3a40"	"00074da4b8"	−0.1483
"00074c3a40"	"00074da5e8"	−0.455
"00074c3a40"	"00074da6b4"	−0.1587
"00074c3a40"	"00074daa3c"	−0.2514
"00074c3a40"	"00074daaf6"	0.149
"00074c3a40"	"00074dad20"	−0.1674
"00074c3a40"	"00074db098"	−0.1583
"00074c3a40"	"00074db231"	−0.2711
"00074c3a40"	"00074db3a3"	−0.27
"00074c3a40"	"00074db5d6"	−0.0069
"00074c3a40"	"00074db632"	−0.1809
"00074c3a40"	"00074db688"	−0.2038

"00074c3a40"	"00074db8a6"	−0.1553
"00074c3a40"	"00074dba19"	−0.5696
"00074c3a40"	"00074dbc2e"	−0.2313
"00074c3a40"	"00074dbe51"	−0.4083
"00074c3a40"	"00074dbe5f"	−0.3277
"00074c3a40"	"00074dbf6d"	−0.3313
"00074c3a40"	"00074dc4a5"	−0.3639
"00074c3a40"	"00074dc50c"	−0.5103
"00074c3a40"	"00074dcdfa"	−0.4119
"00074c3a40"	"00074dcf5f"	−0.2285
"00074c3a40"	"00074dd007"	−0.147
"00074c3a40"	"00074dd163"	−0.1701
"00074c3a40"	"00074dd3df"	−0.2917
"00074c3a40"	"00074dd577"	−0.442
"00074c3a40"	"00074dd62e"	−0.1089
"00074c3a40"	"00074dd73c"	−0.6652
"00074c3a40"	"00074dda10"	−0.1404
"00074c3a40"	"00074ddab8"	−0.2798
"00074c3a40"	"00074ddd3d"	−0.2806
"00074c3a40"	"00074ddf16"	−0.2227
"00074c3a40"	"00074ddfc1"	−0.3072
"00074c3a40"	"00074de21a"	0.0542
"00074c3a40"	"00074de2a9"	−0.2739
"00074c3a40"	"00074de544"	−0.2376
"00074c3a40"	"00074de98a"	−0.3148
"00074c3a40"	"00074dea7e"	−0.1757
"00074c3a40"	"00074debd9"	−0.4137
"00074c3a40"	"00074deca3"	−0.2717
"00074c3a40"	"00074def43"	−0.2853
"00074c3a40"	"00074def99"	−0.1929
"00074c3a40"	"00074ecdad"	−0.0814
"00074c3a40"	"00074ecf28"	−0.2022
"00074c3a40"	"00074ed1e1"	−0.221
"00074c3a40"	"00074ed83b"	−0.1626
"00074c3a40"	"00074ee5e3"	−0.2906
"00074c3a40"	"00074ee6e0"	0.0761
"00074c3a40"	"00074eea3a"	−0.2613
"00074c3a40"	"00074eff82"	−0.2179
"00074c3a40"	"00074f0477"	−0.4037
"00074c3a40"	"00074f08c3"	0.0085
"00074c3a40"	"00074f1859"	−0.1906
"00074c3a40"	"00074f2268"	−0.5835
"00074c3a40"	"00074f28be"	−0.0667
"00074c3a40"	"00074f294b"	−0.1931
"00074c3a40"	"00074f2ddd"	−0.6473
"00074c3a40"	"00074f2e75"	−0.4809
"00074c3a40"	"00074f3088"	−0.1395
"00074c3a40"	"00074f5a1c"	−0.3373
"00074c3a40"	"00074f75b7"	−0.2415
"00074c3a40"	"00074f8cd9"	−0.0806

"00074c3a40"	"00074f96dc"	−0.4271
"00074c3a40"	"00074fabaa"	−0.3181
"00074c3a40"	"00074facd9"	0.0172
"00074c3a40"	"00074fae3c"	−0.0224
"00074c3a40"	"00074fb0a8"	0.3682
"00074c3a40"	"00074fb4e4"	0.338
"00074c3a40"	"00074fb7c2"	−0.1441
"00074c3a40"	"00074fbd36"	−0.5596
"00074c3a40"	"00074fc27f"	−0.2472
"00074c3a40"	"00074fc31d"	0.226
"00074c3a40"	"00074fd569"	−0.2432
"00074c3a40"	"00074fef15"	−0.1923
"00074c3a40"	"00074ff562"	−0.5429
"00074c3a40"	"00075007ca"	−0.1656
"00074c3a40"	"0007500b86"	−0.3137
"00074c3a40"	"0007500d05"	−0.105
"00074c3a40"	"0007500ee4"	−0.0366
"00074c3a40"	"0007500eee"	−0.2696
"00074c3a40"	"00075013dc"	−0.1748
"00074c3a40"	"000757b515"	−0.3794
"00074c3a40"	"000757bc5a"	−0.0979
"00074c3a40"	"000757c320"	−0.0219
"00074c3a40"	"000757c9aa"	−0.2151
"00074c3a40"	"000757ccbe"	−0.2775
"00074c3a40"	"000757cfa9"	−0.4428
"00074c3a40"	"000757d390"	0.5152
"00074c3a40"	"000757d393"	−0.1838
"00074c3a40"	"000757d598"	−0.3818
"00074c3a40"	"000757d5a2"	−0.3519
"00074c3a40"	"000757d790"	−0.1253
"00074c3a40"	"000757e30c"	−0.1615
"00074c3a40"	"000757e4b0"	−0.3077
"00074c3a40"	"000757e7a0"	−0.1063
"00074c3a40"	"000757e8b3"	−0.6041
"00074c3a40"	"000757f627"	0.1633
"00074c3a40"	"000757f925"	−0.2647
"00074c3a40"	"000757fa08"	−0.4752
"00074c3a40"	"000757fe52"	−0.3787
"00074c3a40"	"000758024a"	−0.255
"00074c3a40"	"00075804bb"	−0.2876
"00074c3a40"	"00075a0c04"	−0.3022
"00074c3a40"	"00075a3110"	−0.4934
"00074c3a40"	"00075a341a"	−0.2735
"00074c3a40"	"00075a3dcf"	−0.4126
"00074c3a40"	"00075a3e22"	−0.3189
"00074c3a40"	"00075a48d8"	−0.3746
"00074c3a40"	"00075a5cfb"	−0.3096
"00074c3a40"	"00075a6151"	−0.3482
"00074c3a40"	"00075a6708"	−0.2619
"00074c3a40"	"00075a7319"	0.5179

"00074c3a40"	"00075a7723"	−0.1994
"00074c3a40"	"00075a778b"	−0.1135
"00074c3a40"	"00075a7b8e"	0.3722
"00074c3a40"	"00075a7c79"	−0.1855
"00074c3a40"	"00075a81b6"	−0.336
"00074c3a40"	"00075a82ac"	−0.035
"00074c3a40"	"00075a98e5"	−0.1985
"00074c3a40"	"00075b0d29"	−0.4683
"00074c3a40"	"00075b102a"	−0.2724
"00074c3a40"	"00075b1074"	−0.4025
"00074c3a40"	"00075b135d"	−0.3925
"00074c3a40"	"00075b138b"	−0.2177
"00074c3a40"	"00075b13a0"	−0.4583
"00074c3a40"	"00075b13bd"	−0.1869
"00074c3a40"	"00075b16a9"	−0.1183
"00074c3a40"	"00075b1a28"	−0.1727
"00074c3a40"	"00075b1a97"	0.0015
"00074c3a40"	"00075b1c7b"	−0.1805
"00074c3a40"	"00075b1d24"	−0.1731
"00074c3a40"	"00075b202b"	−0.2355
"00074c3a40"	"00075b22cb"	−0.173
"00074c3a40"	"00075b22da"	−0.3822
"00074c3a40"	"00075b2556"	−0.1849
"00074c3a40"	"00075b25de"	−0.2243
"00074c3a40"	"00075b260c"	−0.4645
"00074c3a40"	"00075b26f1"	−0.394
"00074c3a40"	"00075b2920"	0.0151
"00074c3a40"	"00075b2a64"	−0.3377
"00074c3a40"	"00075b2a9d"	−0.3111
"00074c3a40"	"00075b2b37"	−0.1282
"00074c3a40"	"00075b2cdd"	−0.1351
"00074c3a40"	"00075b3038"	−0.2683
"00074c3a40"	"00075b30fe"	−0.4267
"00074c3a40"	"00075b3362"	−0.2387
"00074c3a40"	"00075b350a"	−0.3152
"00074c3a40"	"00075b350e"	−0.2884
"00074c3a40"	"00075b3651"	−0.0963
"00074c3a40"	"00075b38ca"	−0.1871
"00074c3a40"	"00075b39cc"	−0.2878
"00074c3a40"	"00075b3e1e"	−0.1459
"00074c3a40"	"00075b3e57"	−0.1691
"00074c3a40"	"00075b4079"	−0.4341
"00074c3a40"	"00075b4150"	−0.2631
"00074c3a40"	"00075b4194"	−0.3858
"00074c3a40"	"00075b42d5"	−0.1996
"00074c3a40"	"00075b4424"	−0.2323
"00074c3a40"	"00075b4470"	−0.1321
"00074c3a40"	"00075b47ed"	−0.2462
"00074c3a40"	"00075b4850"	−0.1081
"00074c3a40"	"00075b4ca0"	−0.5708

"00074c3a40"	"00075b4d7f"	−0.1372
"00074c3a40"	"00075b520f"	−0.1571
"00074c3a40"	"00075b525f"	−0.372
"00074c3a40"	"00075b58f8"	−0.1869
"00074c3a40"	"00075b5bcc"	−0.1008
"00074c3a40"	"00075b5bfa"	−0.3528
"00074c3a40"	"00075b6339"	−0.4711
"00074c3a40"	"00075b6658"	−0.4037
"00074c3a40"	"00075b679a"	−0.0781
"00074c3a40"	"00075b6cb7"	−0.2555
"00074c3a40"	"00075b6df8"	−0.2603
"00074c3a40"	"00075b6ff6"	−0.2926
"00074c3a40"	"00075b70ee"	−0.4122
"00074c3a40"	"00075b7157"	−0.0954
"00074c3a40"	"00075b7225"	−0.3659
"00074c3a40"	"00075b7c89"	−0.4041
"00074c3a40"	"00075b9048"	0.0065
"00074c3a40"	"00075d0801"	−0.3936
"00074c3a40"	"00075d1820"	−0.1037
"00074c3a40"	"00075d1f3d"	−0.3473
"00074c3a40"	"00075d2329"	−0.3894
"00074c3a40"	"00075d2b9b"	−0.4797
"00074c3a40"	"00075d3941"	−0.4071
"00074c3a40"	"00075d3e96"	−0.0481
"00074c3a40"	"00075d4864"	−0.2519
"00074c3a40"	"00075d5961"	0.0353
"00074c3a40"	"00075d5a63"	−0.4062
"00074c3a40"	"00075d6150"	0.273
"00074c3a40"	"00075d67d0"	−0.5101
"00074c3a40"	"00075d67e2"	−0.4013
"00074c3a40"	"00075d73fc"	−0.2807
"00074c3a40"	"00075d7729"	−0.268
"00074c3a40"	"00075d778c"	−0.3994
"00074c3a40"	"00075d7b9e"	0.021
"00074c3a40"	"00075d7c8f"	−0.5968
"00074c3a40"	"00075d804d"	−0.1329
"00074c3a40"	"00075d819f"	−0.1765
"00074c3a40"	"00075d8601"	−0.0947
"00074c3a40"	"00075d8c6a"	−0.3608
"00074c3a40"	"00075dfedc"	−0.3504
"00074c3a40"	"00075e05f2"	−0.2659
"00074c3a40"	"00075e0837"	−0.2486
"00074c3a40"	"00075e092e"	−0.2045
"00074c3a40"	"00075e0965"	−0.319
"00074c3a40"	"00075e0bc8"	−0.2341
"00074c3a40"	"00075e0fbb"	−0.464
"00074d84fc"	"00074d8639"	−0.0712
"00074d84fc"	"00074d8697"	0.2145
"00074d84fc"	"00074d87af"	0.0268
"00074d84fc"	"00074d8813"	−0.2073

"00074d84fc"	"00074d8817"	0.1304
"00074d84fc"	"00074d8a39"	−0.2767
"00074d84fc"	"00074d8ad8"	0.4246
"00074d84fc"	"00074d8c26"	0.1396
"00074d84fc"	"00074d8ca5"	0.142
"00074d84fc"	"00074d8e0a"	0.4139
"00074d84fc"	"00074d9179"	−0.168
"00074d84fc"	"00074d929f"	−0.0243
"00074d84fc"	"00074d93c4"	0.0746
"00074d84fc"	"00074d93d0"	−0.1037
"00074d84fc"	"00074d945b"	0.3887
"00074d84fc"	"00074d966c"	0.0935
"00074d84fc"	"00074d9953"	0.1464
"00074d84fc"	"00074d99b2"	−0.2677
"00074d84fc"	"00074d99f3"	−0.0392
"00074d84fc"	"00074d9afd"	−0.0731
"00074d84fc"	"00074d9c87"	0.0957
"00074d84fc"	"00074d9e9e"	−0.378
"00074d84fc"	"00074d9f30"	−0.3168
"00074d84fc"	"00074da036"	0.2697
"00074d84fc"	"00074da082"	−0.0951
"00074d84fc"	"00074da136"	−0.1431
"00074d84fc"	"00074da3ed"	0.1171
"00074d84fc"	"00074da4ac"	0.1479
"00074d84fc"	"00074da4b8"	0.0897
"00074d84fc"	"00074da5e8"	0.2661
"00074d84fc"	"00074da6b4"	0.0191
"00074d84fc"	"00074daa3c"	0.0965
"00074d84fc"	"00074daaf6"	−0.2658
"00074d84fc"	"00074dad20"	−0.0418
"00074d84fc"	"00074db098"	−0.0104
"00074d84fc"	"00074db231"	0.1029
"00074d84fc"	"00074db3a3"	0.2721
"00074d84fc"	"00074db5d6"	−0.3083
"00074d84fc"	"00074db632"	0.3465
"00074d84fc"	"00074db688"	−0.0971
"00074d84fc"	"00074db8a6"	−0.2574
"00074d84fc"	"00074dba19"	0.2026
"00074d84fc"	"00074dbc2e"	0.1115
"00074d84fc"	"00074dbe51"	0.2516
"00074d84fc"	"00074dbe5f"	−0.2029
"00074d84fc"	"00074dbf6d"	0.153
"00074d84fc"	"00074dc4a5"	−0.2199
"00074d84fc"	"00074dc50c"	0.6081
"00074d84fc"	"00074dcdfa"	0.2762
"00074d84fc"	"00074dcf5f"	−0.1989
"00074d84fc"	"00074dd007"	−0.0882
"00074d84fc"	"00074dd163"	−0.2369
"00074d84fc"	"00074dd3df"	−0.1935
"00074d84fc"	"00074dd577"	0.3113

"00074d84fc"	"00074dd62e"	−0.2659
"00074d84fc"	"00074dd73c"	0.3357
"00074d84fc"	"00074dda10"	−0.0078
"00074d84fc"	"00074ddab8"	0.3224
"00074d84fc"	"00074ddd3d"	−0.1145
"00074d84fc"	"00074ddf16"	0.2969
"00074d84fc"	"00074ddfc1"	0.309
"00074d84fc"	"00074de21a"	−0.2002
"00074d84fc"	"00074de2a9"	0.2569
"00074d84fc"	"00074de544"	0.0056
"00074d84fc"	"00074de98a"	0.0189
"00074d84fc"	"00074dea7e"	−0.0576
"00074d84fc"	"00074debd9"	−0.1398
"00074d84fc"	"00074deca3"	−0.0686
"00074d84fc"	"00074def43"	0.2079
"00074d84fc"	"00074def99"	−0.1797
"00074d84fc"	"00074ecdad"	−0.0576
"00074d84fc"	"00074ecf28"	−0.1932
"00074d84fc"	"00074ed1e1"	0.292
"00074d84fc"	"00074ed83b"	0.2876
"00074d84fc"	"00074ee5e3"	−0.2877
"00074d84fc"	"00074ee6e0"	−0.4232
"00074d84fc"	"00074eea3a"	0.1833
"00074d84fc"	"00074eff82"	−0.3316
"00074d84fc"	"00074f0477"	0.0828
"00074d84fc"	"00074f08c3"	−0.0869
"00074d84fc"	"00074f1859"	0.0963
"00074d84fc"	"00074f2268"	−0.0175
"00074d84fc"	"00074f28be"	0.3994
"00074d84fc"	"00074f294b"	−0.0543
"00074d84fc"	"00074f2ddd"	0.0885
"00074d84fc"	"00074f2e75"	−0.1961
"00074d84fc"	"00074f3088"	−0.301
"00074d84fc"	"00074f5a1c"	−0.0682
"00074d84fc"	"00074f75b7"	−5e−04
"00074d84fc"	"00074f8cd9"	0.1765
"00074d84fc"	"00074f96dc"	−0.332
"00074d84fc"	"00074fabaa"	0.0577
"00074d84fc"	"00074facd9"	−0.0286
"00074d84fc"	"00074fae3c"	−0.1875
"00074d84fc"	"00074fb0a8"	−0.2243
"00074d84fc"	"00074fb4e4"	−0.4358
"00074d84fc"	"00074fb7c2"	0.1093
"00074d84fc"	"00074fbd36"	−0.31
"00074d84fc"	"00074fc27f"	0.2941
"00074d84fc"	"00074fc31d"	−0.2993
"00074d84fc"	"00074fd569"	−0.1129
"00074d84fc"	"00074fef15"	−0.3074
"00074d84fc"	"00074ff562"	0.0131
"00074d84fc"	"00075007ca"	0.218

"00074d84fc"	"0007500b86"	-0.0164
"00074d84fc"	"0007500d05"	0.0257
"00074d84fc"	"0007500ee4"	-0.2622
"00074d84fc"	"0007500eee"	-0.095
"00074d84fc"	"00075013dc"	0.0203
"00074d84fc"	"000757b515"	0.1885
"00074d84fc"	"000757bc5a"	0.0915
"00074d84fc"	"000757c320"	-0.197
"00074d84fc"	"000757c9aa"	-5e-04
"00074d84fc"	"000757ccbe"	-0.0534
"00074d84fc"	"000757cfa9"	-0.2141
"00074d84fc"	"000757d390"	-0.358
"00074d84fc"	"000757d393"	-0.1325
"00074d84fc"	"000757d598"	-0.0131
"00074d84fc"	"000757d5a2"	0.1326
"00074d84fc"	"000757d790"	-0.0956
"00074d84fc"	"000757e30c"	-0.0132
"00074d84fc"	"000757e4b0"	0.3768
"00074d84fc"	"000757e7a0"	-0.0557
"00074d84fc"	"000757e8b3"	0.1516
"00074d84fc"	"000757f627"	0.1345
"00074d84fc"	"000757f925"	-0.0147
"00074d84fc"	"000757fa08"	-0.0248
"00074d84fc"	"000757fe52"	0.0222
"00074d84fc"	"000758024a"	0.0804
"00074d84fc"	"00075804bb"	0.1667
"00074d84fc"	"00075a0c04"	0.2855
"00074d84fc"	"00075a3110"	0.1945
"00074d84fc"	"00075a341a"	-0.193
"00074d84fc"	"00075a3dcf"	-0.0098
"00074d84fc"	"00075a3e22"	0.1468
"00074d84fc"	"00075a48d8"	0.0245
"00074d84fc"	"00075a5cfb"	0.1987
"00074d84fc"	"00075a6151"	0.0471
"00074d84fc"	"00075a6708"	0.0462
"00074d84fc"	"00075a7319"	-0.3645
"00074d84fc"	"00075a7723"	0.0709
"00074d84fc"	"00075a778b"	-0.0676
"00074d84fc"	"00075a7b8e"	0.1453
"00074d84fc"	"00075a7c79"	0.3921
"00074d84fc"	"00075a81b6"	0.0458
"00074d84fc"	"00075a82ac"	0.087
"00074d84fc"	"00075a98e5"	-0.1423
"00074d84fc"	"00075b0d29"	-0.1799
"00074d84fc"	"00075b102a"	0.1134
"00074d84fc"	"00075b1074"	0.0489
"00074d84fc"	"00075b135d"	-0.0405
"00074d84fc"	"00075b138b"	0.5512
"00074d84fc"	"00075b13a0"	-0.3257
"00074d84fc"	"00075b13bd"	-0.281

"00074d84fc"	"00075b16a9"	-0.1556
"00074d84fc"	"00075b1a28"	-0.2166
"00074d84fc"	"00075b1a97"	-0.0111
"00074d84fc"	"00075b1c7b"	-0.4419
"00074d84fc"	"00075b1d24"	-0.1023
"00074d84fc"	"00075b202b"	0.4097
"00074d84fc"	"00075b22cb"	-0.1059
"00074d84fc"	"00075b22da"	0.1876
"00074d84fc"	"00075b2556"	0.0189
"00074d84fc"	"00075b25de"	-0.0823
"00074d84fc"	"00075b260c"	0.347
"00074d84fc"	"00075b26f1"	0.009
"00074d84fc"	"00075b2920"	0.1696
"00074d84fc"	"00075b2a64"	0.1605
"00074d84fc"	"00075b2a9d"	0.317
"00074d84fc"	"00075b2b37"	-0.2997
"00074d84fc"	"00075b2cdd"	-0.1613
"00074d84fc"	"00075b3038"	0.2978
"00074d84fc"	"00075b30fe"	0.1261
"00074d84fc"	"00075b3362"	-0.5912
"00074d84fc"	"00075b350a"	0.1015
"00074d84fc"	"00075b350e"	0.0653
"00074d84fc"	"00075b3651"	-0.3617
"00074d84fc"	"00075b38ca"	-0.136
"00074d84fc"	"00075b39cc"	0.0158
"00074d84fc"	"00075b3e1e"	0.1812
"00074d84fc"	"00075b3e57"	-0.2506
"00074d84fc"	"00075b4079"	-0.118
"00074d84fc"	"00075b4150"	0.1008
"00074d84fc"	"00075b4194"	0.3127
"00074d84fc"	"00075b42d5"	0.1599
"00074d84fc"	"00075b4424"	-0.2083
"00074d84fc"	"00075b4470"	0.0915
"00074d84fc"	"00075b47ed"	0.003
"00074d84fc"	"00075b4850"	0.3008
"00074d84fc"	"00075b4ca0"	0.2734
"00074d84fc"	"00075b4d7f"	0.1205
"00074d84fc"	"00075b520f"	-0.0759
"00074d84fc"	"00075b525f"	-0.2013
"00074d84fc"	"00075b58f8"	-0.1371
"00074d84fc"	"00075b5bcc"	0.1082
"00074d84fc"	"00075b5bfa"	-0.147
"00074d84fc"	"00075b6339"	0.234
"00074d84fc"	"00075b6658"	-0.4893
"00074d84fc"	"00075b679a"	0.085
"00074d84fc"	"00075b6cb7"	0.0544
"00074d84fc"	"00075b6df8"	-0.0516
"00074d84fc"	"00075b6ff6"	0.3528
"00074d84fc"	"00075b70ee"	0.1833
"00074d84fc"	"00075b7157"	-0.3157

"00074d84fc"	"00075b7225"	−0.0266
"00074d84fc"	"00075b7c89"	0.102
"00074d84fc"	"00075b9048"	0.2957
"00074d84fc"	"00075d0801"	−0.1133
"00074d84fc"	"00075d1820"	−0.3067
"00074d84fc"	"00075d1f3d"	0.4739
"00074d84fc"	"00075d2329"	0.1614
"00074d84fc"	"00075d2b9b"	0.2294
"00074d84fc"	"00075d3941"	0.0564
"00074d84fc"	"00075d3e96"	−0.1408
"00074d84fc"	"00075d4864"	−0.2776
"00074d84fc"	"00075d5961"	0.1469
"00074d84fc"	"00075d5a63"	0.2679
"00074d84fc"	"00075d6150"	−0.1793
"00074d84fc"	"00075d67d0"	−0.1116
"00074d84fc"	"00075d67e2"	0.006
"00074d84fc"	"00075d73fc"	−0.0145
"00074d84fc"	"00075d7729"	−0.0952
"00074d84fc"	"00075d778c"	−0.4711
"00074d84fc"	"00075d7b9e"	−0.361
"00074d84fc"	"00075d7c8f"	0.3039
"00074d84fc"	"00075d804d"	0.2928
"00074d84fc"	"00075d819f"	0.0246
"00074d84fc"	"00075d8601"	−0.1537
"00074d84fc"	"00075d8c6a"	0.0445
"00074d84fc"	"00075dfedc"	0.1757
"00074d84fc"	"00075e05f2"	0.0094
"00074d84fc"	"00075e0837"	0.3737
"00074d84fc"	"00075e092e"	0.0467
"00074d84fc"	"00075e0965"	0.0575
"00074d84fc"	"00075e0bc8"	0.406
"00074d84fc"	"00075e0fbb"	0.1447
"00074d8639"	"00074d8697"	−0.2232
"00074d8639"	"00074d87af"	−0.2148
"00074d8639"	"00074d8813"	−0.35
"00074d8639"	"00074d8817"	−0.1635
"00074d8639"	"00074d8a39"	−0.236
"00074d8639"	"00074d8ad8"	0.1144
"00074d8639"	"00074d8c26"	−0.1767
"00074d8639"	"00074d8ca5"	−0.1843
"00074d8639"	"00074d8e0a"	−0.1984
"00074d8639"	"00074d9179"	0.2654
"00074d8639"	"00074d929f"	0.254
"00074d8639"	"00074d93c4"	0.0032
"00074d8639"	"00074d93d0"	0.3296
"00074d8639"	"00074d945b"	0.0296
"00074d8639"	"00074d966c"	−0.1714
"00074d8639"	"00074d9953"	−0.0389
"00074d8639"	"00074d99b2"	0.0549
"00074d8639"	"00074d99f3"	0.1263

"00074d8639"	"00074d9afd"	0.2649
"00074d8639"	"00074d9c87"	0.1581
"00074d8639"	"00074d9e9e"	−0.2412
"00074d8639"	"00074d9f30"	−0.2852
"00074d8639"	"00074da036"	0.1941
"00074d8639"	"00074da082"	0.404
"00074d8639"	"00074da136"	0.6085
"00074d8639"	"00074da3ed"	0.0066
"00074d8639"	"00074da4ac"	0.1474
"00074d8639"	"00074da4b8"	−0.0927
"00074d8639"	"00074da5e8"	0.2379
"00074d8639"	"00074da6b4"	0.281
"00074d8639"	"00074daa3c"	−0.4762
"00074d8639"	"00074daaf6"	−0.0552
"00074d8639"	"00074dad20"	0.1127
"00074d8639"	"00074db098"	−0.1625
"00074d8639"	"00074db231"	0.1817
"00074d8639"	"00074db3a3"	−0.1623
"00074d8639"	"00074db5d6"	−0.0064
"00074d8639"	"00074db632"	0.0497
"00074d8639"	"00074db688"	0.1475
"00074d8639"	"00074db8a6"	−0.2734
"00074d8639"	"00074dba19"	−0.2138
"00074d8639"	"00074dbc2e"	−0.0322
"00074d8639"	"00074dbe51"	0.3366
"00074d8639"	"00074dbe5f"	−0.1312
"00074d8639"	"00074dbf6d"	−0.5625
"00074d8639"	"00074dc4a5"	0.2191
"00074d8639"	"00074dc50c"	−0.1516
"00074d8639"	"00074dcdfa"	−0.2081
"00074d8639"	"00074dcf5f"	−0.0753
"00074d8639"	"00074dd007"	−0.5258
"00074d8639"	"00074dd163"	−0.1883
"00074d8639"	"00074dd3df"	−0.096
"00074d8639"	"00074dd577"	−0.0208
"00074d8639"	"00074dd62e"	0.0274
"00074d8639"	"00074dd73c"	0.0569
"00074d8639"	"00074dda10"	−0.0506
"00074d8639"	"00074ddab8"	−0.2053
"00074d8639"	"00074ddd3d"	−0.0927
"00074d8639"	"00074ddf16"	−0.2045
"00074d8639"	"00074ddfc1"	−0.1293
"00074d8639"	"00074de21a"	−0.455
"00074d8639"	"00074de2a9"	−0.1822
"00074d8639"	"00074de544"	0.1233
"00074d8639"	"00074de98a"	−0.1003
"00074d8639"	"00074dea7e"	−0.0169
"00074d8639"	"00074debd9"	−0.2989
"00074d8639"	"00074deca3"	−0.2101
"00074d8639"	"00074def43"	−0.0778

"00074d8639"	"00074def99"	0.0856
"00074d8639"	"00074ecdad"	0.0895
"00074d8639"	"00074ecf28"	−0.0775
"00074d8639"	"00074ed1e1"	−0.0614
"00074d8639"	"00074ed83b"	−0.0367
"00074d8639"	"00074ee5e3"	−0.389
"00074d8639"	"00074ee6e0"	−0.0868
"00074d8639"	"00074eea3a"	0.1835
"00074d8639"	"00074eff82"	0.0263
"00074d8639"	"00074f0477"	−0.191
"00074d8639"	"00074f08c3"	−0.381
"00074d8639"	"00074f1859"	0.0193
"00074d8639"	"00074f2268"	−0.2449
"00074d8639"	"00074f28be"	0.0273
"00074d8639"	"00074f294b"	0.0875
"00074d8639"	"00074f2ddd"	−0.0594
"00074d8639"	"00074f2e75"	0.4459
"00074d8639"	"00074f3088"	0.0318
"00074d8639"	"00074f5a1c"	0.0578
"00074d8639"	"00074f75b7"	0.0809
"00074d8639"	"00074f8cd9"	0.1545
"00074d8639"	"00074f96dc"	0.1515
"00074d8639"	"00074fabaa"	−0.2918
"00074d8639"	"00074facd9"	−0.2805
"00074d8639"	"00074fae3c"	−0.0104
"00074d8639"	"00074fb0a8"	−0.1032
"00074d8639"	"00074fb4e4"	−0.3998
"00074d8639"	"00074fb7c2"	0.3706
"00074d8639"	"00074fbd36"	−0.2419
"00074d8639"	"00074fc27f"	−0.2237
"00074d8639"	"00074fc31d"	−0.0049
"00074d8639"	"00074fd569"	0.0971
"00074d8639"	"00074fef15"	−0.033
"00074d8639"	"00074ff562"	−0.4288
"00074d8639"	"00075007ca"	−0.1042
"00074d8639"	"0007500b86"	−0.2656
"00074d8639"	"0007500d05"	−0.0058
"00074d8639"	"0007500ee4"	−0.0391
"00074d8639"	"0007500eee"	−0.1982
"00074d8639"	"00075013dc"	−0.468
"00074d8639"	"000757b515"	−0.1199
"00074d8639"	"000757bc5a"	−0.1173
"00074d8639"	"000757c320"	−0.5117
"00074d8639"	"000757c9aa"	−0.4051
"00074d8639"	"000757ccbe"	0.1331
"00074d8639"	"000757cfa9"	−0.1247
"00074d8639"	"000757d390"	−0.2844
"00074d8639"	"000757d393"	−0.0469
"00074d8639"	"000757d598"	−0.447
"00074d8639"	"000757d5a2"	−0.2218

"00074d8639"	"000757d790"	-0.0498
"00074d8639"	"000757e30c"	-0.2332
"00074d8639"	"000757e4b0"	-0.1279
"00074d8639"	"000757e7a0"	0.2072
"00074d8639"	"000757e8b3"	0.0713
"00074d8639"	"000757f627"	-0.3119
"00074d8639"	"000757f925"	0.0249
"00074d8639"	"000757fa08"	0.5826
"00074d8639"	"000757fe52"	0.0684
"00074d8639"	"000758024a"	-0.1034
"00074d8639"	"00075804bb"	-0.3436
"00074d8639"	"00075a0c04"	0.4042
"00074d8639"	"00075a3110"	0.0507
"00074d8639"	"00075a341a"	0.1868
"00074d8639"	"00075a3dcf"	0.165
"00074d8639"	"00075a3e22"	-0.0209
"00074d8639"	"00075a48d8"	0.3068
"00074d8639"	"00075a5cfb"	-0.2452
"00074d8639"	"00075a6151"	-0.269
"00074d8639"	"00075a6708"	-0.1001
"00074d8639"	"00075a7319"	-0.2828
"00074d8639"	"00075a7723"	0.1644
"00074d8639"	"00075a778b"	0.0505
"00074d8639"	"00075a7b8e"	-0.0647
"00074d8639"	"00075a7c79"	-0.0252
"00074d8639"	"00075a81b6"	-0.0672
"00074d8639"	"00075a82ac"	0.1396
"00074d8639"	"00075a98e5"	-0.2277
"00074d8639"	"00075b0d29"	-0.0087
"00074d8639"	"00075b102a"	-0.061
"00074d8639"	"00075b1074"	0.1946
"00074d8639"	"00075b135d"	0.0866
"00074d8639"	"00075b138b"	-0.0857
"00074d8639"	"00075b13a0"	-0.2594
"00074d8639"	"00075b13bd"	0.0847
"00074d8639"	"00075b16a9"	0.2549
"00074d8639"	"00075b1a28"	0.0531
"00074d8639"	"00075b1a97"	0.164
"00074d8639"	"00075b1c7b"	0.0719
"00074d8639"	"00075b1d24"	0.3017
"00074d8639"	"00075b202b"	-0.0932
"00074d8639"	"00075b22cb"	0.1172
"00074d8639"	"00075b22da"	-0.0903
"00074d8639"	"00075b2556"	-0.1371
"00074d8639"	"00075b25de"	-0.0641
"00074d8639"	"00075b260c"	-0.2548
"00074d8639"	"00075b26f1"	0.0523
"00074d8639"	"00075b2920"	-0.2885
"00074d8639"	"00075b2a64"	-0.3916
"00074d8639"	"00075b2a9d"	-0.2948

"00074d8639"	"00075b2b37"	-0.2652
"00074d8639"	"00075b2cdd"	0.0891
"00074d8639"	"00075b3038"	-0.0972
"00074d8639"	"00075b30fe"	-0.1107
"00074d8639"	"00075b3362"	0.0974
"00074d8639"	"00075b350a"	-0.2063
"00074d8639"	"00075b350e"	-0.0402
"00074d8639"	"00075b3651"	0.103
"00074d8639"	"00075b38ca"	0.1861
"00074d8639"	"00075b39cc"	0.0889
"00074d8639"	"00075b3e1e"	0.0746
"00074d8639"	"00075b3e57"	0.0543
"00074d8639"	"00075b4079"	-0.1759
"00074d8639"	"00075b4150"	-0.0959
"00074d8639"	"00075b4194"	-0.1978
"00074d8639"	"00075b42d5"	0.1979
"00074d8639"	"00075b4424"	0.0735
"00074d8639"	"00075b4470"	-0.0479
"00074d8639"	"00075b47ed"	0.1084
"00074d8639"	"00075b4850"	-0.1909
"00074d8639"	"00075b4ca0"	-0.2068
"00074d8639"	"00075b4d7f"	0.5234
"00074d8639"	"00075b520f"	-0.0694
"00074d8639"	"00075b525f"	-9e-04
"00074d8639"	"00075b58f8"	-0.2358
"00074d8639"	"00075b5bcc"	0.0013
"00074d8639"	"00075b5bfa"	-0.3336
"00074d8639"	"00075b6339"	0.2288
"00074d8639"	"00075b6658"	0.1279
"00074d8639"	"00075b679a"	0.0508
"00074d8639"	"00075b6cb7"	-0.2939
"00074d8639"	"00075b6df8"	0.4145
"00074d8639"	"00075b6ff6"	-0.0859
"00074d8639"	"00075b70ee"	0.0404
"00074d8639"	"00075b7157"	0.0065
"00074d8639"	"00075b7225"	0.1979
"00074d8639"	"00075b7c89"	0.2175
"00074d8639"	"00075b9048"	-0.1585
"00074d8639"	"00075d0801"	-0.1644
"00074d8639"	"00075d1820"	0.1035
"00074d8639"	"00075d1f3d"	-0.1937
"00074d8639"	"00075d2329"	0.0871
"00074d8639"	"00075d2b9b"	-0.383
"00074d8639"	"00075d3941"	-0.0304
"00074d8639"	"00075d3e96"	0.1026
"00074d8639"	"00075d4864"	-0.0101
"00074d8639"	"00075d5961"	0.3281
"00074d8639"	"00075d5a63"	0.0449
"00074d8639"	"00075d6150"	0.0368
"00074d8639"	"00075d67d0"	-0.2039

"00074d8639"	"00075d67e2"	-0.1135
"00074d8639"	"00075d73fc"	0.2212
"00074d8639"	"00075d7729"	-0.0885
"00074d8639"	"00075d778c"	0.0742
"00074d8639"	"00075d7b9e"	0.0646
"00074d8639"	"00075d7c8f"	0.31
"00074d8639"	"00075d804d"	-0.3593
"00074d8639"	"00075d819f"	-0.3421
"00074d8639"	"00075d8601"	0.0985
"00074d8639"	"00075d8c6a"	0.3157
"00074d8639"	"00075dfedc"	0.371
"00074d8639"	"00075e05f2"	-0.2217
"00074d8639"	"00075e0837"	-0.1059
"00074d8639"	"00075e092e"	-0.1174
"00074d8639"	"00075e0965"	-0.1568
"00074d8639"	"00075e0bc8"	-0.1175
"00074d8639"	"00075e0fbb"	0.0321
"00074d8697"	"00074d87af"	0.0999
"00074d8697"	"00074d8813"	-0.0846
"00074d8697"	"00074d8817"	0.1635
"00074d8697"	"00074d8a39"	0.0693
"00074d8697"	"00074d8ad8"	0.0684
"00074d8697"	"00074d8c26"	0.2382
"00074d8697"	"00074d8ca5"	-0.051
"00074d8697"	"00074d8e0a"	0.222
"00074d8697"	"00074d9179"	0.0337
"00074d8697"	"00074d929f"	-0.1929
"00074d8697"	"00074d93c4"	-0.0359
"00074d8697"	"00074d93d0"	-0.087
"00074d8697"	"00074d945b"	0.0237
"00074d8697"	"00074d966c"	0.1135
"00074d8697"	"00074d9953"	0.1318
"00074d8697"	"00074d99b2"	0.2606
"00074d8697"	"00074d99f3"	0.04
"00074d8697"	"00074d9afd"	-0.1606
"00074d8697"	"00074d9c87"	0.2914
"00074d8697"	"00074d9e9e"	-0.1143
"00074d8697"	"00074d9f30"	-0.0279
"00074d8697"	"00074da036"	0.3823
"00074d8697"	"00074da082"	-0.1937
"00074d8697"	"00074da136"	-0.1027
"00074d8697"	"00074da3ed"	0.1261
"00074d8697"	"00074da4ac"	0.0277
"00074d8697"	"00074da4b8"	0.0081
"00074d8697"	"00074da5e8"	0.2302
"00074d8697"	"00074da6b4"	0.1256
"00074d8697"	"00074daa3c"	0.0268
"00074d8697"	"00074daaf6"	-0.119
"00074d8697"	"00074dad20"	-0.2159
"00074d8697"	"00074db098"	-0.1812

"00074d8697"	"00074db231"	0.2125
"00074d8697"	"00074db3a3"	0.0802
"00074d8697"	"00074db5d6"	-0.0147
"00074d8697"	"00074db632"	0.2977
"00074d8697"	"00074db688"	0.1694
"00074d8697"	"00074db8a6"	-0.0156
"00074d8697"	"00074dba19"	0.3966
"00074d8697"	"00074dbc2e"	-0.2397
"00074d8697"	"00074dbe51"	0.1057
"00074d8697"	"00074dbe5f"	-0.1599
"00074d8697"	"00074dbf6d"	-0.1645
"00074d8697"	"00074dc4a5"	-0.3554
"00074d8697"	"00074dc50c"	0.2737
"00074d8697"	"00074dcdfa"	0.125
"00074d8697"	"00074dcf5f"	-0.4033
"00074d8697"	"00074dd007"	0.0651
"00074d8697"	"00074dd163"	0.031
"00074d8697"	"00074dd3df"	-0.0453
"00074d8697"	"00074dd577"	0.2553
"00074d8697"	"00074dd62e"	0.0688
"00074d8697"	"00074dd73c"	0.2588
"00074d8697"	"00074dda10"	0.1108
"00074d8697"	"00074ddab8"	0.3001
"00074d8697"	"00074ddd3d"	-0.3816
"00074d8697"	"00074ddf16"	-0.0708
"00074d8697"	"00074ddfc1"	0.1183
"00074d8697"	"00074de21a"	0.2006
"00074d8697"	"00074de2a9"	0.2964
"00074d8697"	"00074de544"	0.1669
"00074d8697"	"00074de98a"	0.0904
"00074d8697"	"00074dea7e"	-0.0886
"00074d8697"	"00074debd9"	-0.2339
"00074d8697"	"00074deca3"	-0.1115
"00074d8697"	"00074def43"	-0.1588
"00074d8697"	"00074def99"	-0.1108
"00074d8697"	"00074ecdad"	-0.3461
"00074d8697"	"00074ecf28"	0.0023
"00074d8697"	"00074ed1e1"	0.0656
"00074d8697"	"00074ed83b"	0.0997
"00074d8697"	"00074ee5e3"	-0.1364
"00074d8697"	"00074ee6e0"	-0.131
"00074d8697"	"00074eea3a"	0.1836
"00074d8697"	"00074eff82"	-0.0517
"00074d8697"	"00074f0477"	-0.0461
"00074d8697"	"00074f08c3"	0.075
"00074d8697"	"00074f1859"	-0.084
"00074d8697"	"00074f2268"	-0.0486
"00074d8697"	"00074f28be"	0.2326
"00074d8697"	"00074f294b"	-0.0683
"00074d8697"	"00074f2ddd"	0.0734

"00074d8697"	"00074f2e75"	-0.3331
"00074d8697"	"00074f3088"	0.0507
"00074d8697"	"00074f5a1c"	0.0869
"00074d8697"	"00074f75b7"	-0.0609
"00074d8697"	"00074f8cd9"	-0.0235
"00074d8697"	"00074f96dc"	-0.0308
"00074d8697"	"00074fabaa"	0.1957
"00074d8697"	"00074facd9"	0.0233
"00074d8697"	"00074fae3c"	-0.1608
"00074d8697"	"00074fb0a8"	0.2321
"00074d8697"	"00074fb4e4"	-0.13
"00074d8697"	"00074fb7c2"	0.2561
"00074d8697"	"00074fbd36"	0.0724
"00074d8697"	"00074fc27f"	0.0484
"00074d8697"	"00074fc31d"	0.0796
"00074d8697"	"00074fd569"	0.114
"00074d8697"	"00074fef15"	-0.0273
"00074d8697"	"00074ff562"	-0.1004
"00074d8697"	"00075007ca"	-0.2052
"00074d8697"	"0007500b86"	0.2175
"00074d8697"	"0007500d05"	0.0072
"00074d8697"	"0007500ee4"	0.0428
"00074d8697"	"0007500eee"	-0.1056
"00074d8697"	"00075013dc"	0.0567
"00074d8697"	"000757b515"	0.2103
"00074d8697"	"000757bc5a"	0.355
"00074d8697"	"000757c320"	-0.2352
"00074d8697"	"000757c9aa"	0.0402
"00074d8697"	"000757ccbe"	-0.0189
"00074d8697"	"000757cfa9"	0.0417
"00074d8697"	"000757d390"	-0.0963
"00074d8697"	"000757d393"	0.1324
"00074d8697"	"000757d598"	-0.0691
"00074d8697"	"000757d5a2"	-0.1625
"00074d8697"	"000757d790"	-0.0474
"00074d8697"	"000757e30c"	-0.144
"00074d8697"	"000757e4b0"	0.5749
"00074d8697"	"000757e7a0"	0.0244
"00074d8697"	"000757e8b3"	0.2926
"00074d8697"	"000757f627"	0.0182
"00074d8697"	"000757f925"	-0.0683
"00074d8697"	"000757fa08"	-0.1894
"00074d8697"	"000757fe52"	0.1078
"00074d8697"	"000758024a"	-0.1259
"00074d8697"	"00075804bb"	0.0827
"00074d8697"	"00075a0c04"	0.1152
"00074d8697"	"00075a3110"	0.1707
"00074d8697"	"00075a341a"	-0.2334
"00074d8697"	"00075a3dcf"	0.0938
"00074d8697"	"00075a3e22"	0.2255

"00074d8697"	"00075a48d8"	−0.1512
"00074d8697"	"00075a5cfb"	0.1128
"00074d8697"	"00075a6151"	−0.1248
"00074d8697"	"00075a6708"	−0.1989
"00074d8697"	"00075a7319"	−0.1356
"00074d8697"	"00075a7723"	−0.0103
"00074d8697"	"00075a778b"	−0.0685
"00074d8697"	"00075a7b8e"	−0.0391
"00074d8697"	"00075a7c79"	0.2516
"00074d8697"	"00075a81b6"	−0.1005
"00074d8697"	"00075a82ac"	0.2247
"00074d8697"	"00075a98e5"	−0.0415
"00074d8697"	"00075b0d29"	0.0864
"00074d8697"	"00075b102a"	−0.0377
"00074d8697"	"00075b1074"	0.002
"00074d8697"	"00075b135d"	0.3782
"00074d8697"	"00075b138b"	0.3268
"00074d8697"	"00075b13a0"	−0.0597
"00074d8697"	"00075b13bd"	0.1987
"00074d8697"	"00075b16a9"	−0.2157
"00074d8697"	"00075b1a28"	−0.0573
"00074d8697"	"00075b1a97"	−0.0148
"00074d8697"	"00075b1c7b"	−0.4875
"00074d8697"	"00075b1d24"	−0.1272
"00074d8697"	"00075b202b"	0.209
"00074d8697"	"00075b22cb"	−0.1193
"00074d8697"	"00075b22da"	0.1577
"00074d8697"	"00075b2556"	−0.0451
"00074d8697"	"00075b25de"	0.2529
"00074d8697"	"00075b260c"	−0.096
"00074d8697"	"00075b26f1"	−0.2046
"00074d8697"	"00075b2920"	−0.0024
"00074d8697"	"00075b2a64"	−0.0013
"00074d8697"	"00075b2a9d"	0.2273
"00074d8697"	"00075b2b37"	−0.019
"00074d8697"	"00075b2cdd"	−0.0025
"00074d8697"	"00075b3038"	0.3065
"00074d8697"	"00075b30fe"	0.2433
"00074d8697"	"00075b3362"	−0.3621
"00074d8697"	"00075b350a"	−0.0682
"00074d8697"	"00075b350e"	0.24
"00074d8697"	"00075b3651"	−0.3346
"00074d8697"	"00075b38ca"	−0.0924
"00074d8697"	"00075b39cc"	−0.0735
"00074d8697"	"00075b3e1e"	−0.0294
"00074d8697"	"00075b3e57"	−0.1406
"00074d8697"	"00075b4079"	0.0145
"00074d8697"	"00075b4150"	−0.0709
"00074d8697"	"00075b4194"	0.2513
"00074d8697"	"00075b42d5"	0.1349

"00074d8697"	"00075b4424"	-0.2243
"00074d8697"	"00075b4470"	0.355
"00074d8697"	"00075b47ed"	0.0328
"00074d8697"	"00075b4850"	0.3873
"00074d8697"	"00075b4ca0"	0.3518
"00074d8697"	"00075b4d7f"	0.271
"00074d8697"	"00075b520f"	0.3005
"00074d8697"	"00075b525f"	-0.077
"00074d8697"	"00075b58f8"	0.2105
"00074d8697"	"00075b5bcc"	0.1452
"00074d8697"	"00075b5bfa"	-0.0759
"00074d8697"	"00075b6339"	0.1076
"00074d8697"	"00075b6658"	-0.1956
"00074d8697"	"00075b679a"	0.0994
"00074d8697"	"00075b6cb7"	-0.0974
"00074d8697"	"00075b6df8"	-0.0573
"00074d8697"	"00075b6ff6"	0.1673
"00074d8697"	"00075b70ee"	-0.0735
"00074d8697"	"00075b7157"	0.1904
"00074d8697"	"00075b7225"	0.1497
"00074d8697"	"00075b7c89"	0.1291
"00074d8697"	"00075b9048"	0.1248
"00074d8697"	"00075d0801"	0.3334
"00074d8697"	"00075d1820"	-0.1187
"00074d8697"	"00075d1f3d"	0.2615
"00074d8697"	"00075d2329"	0.1877
"00074d8697"	"00075d2b9b"	0.0777
"00074d8697"	"00075d3941"	0.1532
"00074d8697"	"00075d3e96"	0.1129
"00074d8697"	"00075d4864"	0.1691
"00074d8697"	"00075d5961"	0.0081
"00074d8697"	"00075d5a63"	0.1083
"00074d8697"	"00075d6150"	-0.0931
"00074d8697"	"00075d67d0"	-0.182
"00074d8697"	"00075d67e2"	0.115
"00074d8697"	"00075d73fc"	0.0061
"00074d8697"	"00075d7729"	0.1699
"00074d8697"	"00075d778c"	-0.3594
"00074d8697"	"00075d7b9e"	-0.0561
"00074d8697"	"00075d7c8f"	0.2143
"00074d8697"	"00075d804d"	0.4601
"00074d8697"	"00075d819f"	0.1155
"00074d8697"	"00075d8601"	0.0602
"00074d8697"	"00075d8c6a"	0.0432
"00074d8697"	"00075dfedc"	0.0296
"00074d8697"	"00075e05f2"	-0.1195
"00074d8697"	"00075e0837"	0.0609
"00074d8697"	"00075e092e"	0.0172
"00074d8697"	"00075e0965"	-0.0673
"00074d8697"	"00075e0bc8"	0.4447

"00074d8697"	"00075e0fbb"	-0.0082
"00074d87af"	"00074d8813"	-0.3265
"00074d87af"	"00074d8817"	-0.0396
"00074d87af"	"00074d8a39"	-0.1756
"00074d87af"	"00074d8ad8"	-0.2231
"00074d87af"	"00074d8c26"	0.0986
"00074d87af"	"00074d8ca5"	0.386
"00074d87af"	"00074d8e0a"	-0.0543
"00074d87af"	"00074d9179"	-0.1695
"00074d87af"	"00074d929f"	-0.0057
"00074d87af"	"00074d93c4"	0.0798
"00074d87af"	"00074d93d0"	-0.1312
"00074d87af"	"00074d945b"	-0.2191
"00074d87af"	"00074d966c"	-0.2362
"00074d87af"	"00074d9953"	0.2795
"00074d87af"	"00074d99b2"	-0.3151
"00074d87af"	"00074d99f3"	-0.0279
"00074d87af"	"00074d9afd"	0.0354
"00074d87af"	"00074d9c87"	-0.2525
"00074d87af"	"00074d9e9e"	-0.1636
"00074d87af"	"00074d9f30"	-0.1989
"00074d87af"	"00074da036"	-0.0591
"00074d87af"	"00074da082"	-0.2412
"00074d87af"	"00074da136"	-0.1174
"00074d87af"	"00074da3ed"	-0.0284
"00074d87af"	"00074da4ac"	0.0703
"00074d87af"	"00074da4b8"	0.0335
"00074d87af"	"00074da5e8"	-0.124
"00074d87af"	"00074da6b4"	0.1459
"00074d87af"	"00074daa3c"	-0.2623
"00074d87af"	"00074daaf6"	-0.3378
"00074d87af"	"00074dad20"	-0.3335
"00074d87af"	"00074db098"	-0.2665
"00074d87af"	"00074db231"	-0.1836
"00074d87af"	"00074db3a3"	-0.1069
"00074d87af"	"00074db5d6"	-0.0087
"00074d87af"	"00074db632"	0.2841
"00074d87af"	"00074db688"	-0.1936
"00074d87af"	"00074db8a6"	-0.1753
"00074d87af"	"00074dba19"	-0.1247
"00074d87af"	"00074dbc2e"	-0.157
"00074d87af"	"00074dbe51"	-0.0072
"00074d87af"	"00074dbe5f"	-0.2481
"00074d87af"	"00074dbf6d"	-0.2421
"00074d87af"	"00074dc4a5"	-0.2071
"00074d87af"	"00074dc50c"	0.0346
"00074d87af"	"00074dcdfa"	-0.043
"00074d87af"	"00074dcf5f"	-0.2781
"00074d87af"	"00074dd007"	-0.2668
"00074d87af"	"00074dd163"	-0.2361

"00074d87af"	"00074dd3df"	-0.2778
"00074d87af"	"00074dd577"	0
"00074d87af"	"00074dd62e"	-0.1636
"00074d87af"	"00074dd73c"	-0.1338
"00074d87af"	"00074dda10"	0.4129
"00074d87af"	"00074ddab8"	0.131
"00074d87af"	"00074ddd3d"	-0.339
"00074d87af"	"00074ddf16"	-0.0873
"00074d87af"	"00074ddfc1"	0.018
"00074d87af"	"00074de21a"	0.3329
"00074d87af"	"00074de2a9"	0.091
"00074d87af"	"00074de544"	0.38
"00074d87af"	"00074de98a"	0.4067
"00074d87af"	"00074dea7e"	-0.0178
"00074d87af"	"00074debd9"	-0.3953
"00074d87af"	"00074deca3"	-0.2446
"00074d87af"	"00074def43"	-0.2174
"00074d87af"	"00074def99"	-0.1038
"00074d87af"	"00074ecdad"	-0.1667
"00074d87af"	"00074ecf28"	-0.152
"00074d87af"	"00074ed1e1"	-0.0075
"00074d87af"	"00074ed83b"	0.0641
"00074d87af"	"00074ee5e3"	-0.3518
"00074d87af"	"00074ee6e0"	-0.3466
"00074d87af"	"00074eea3a"	-0.0132
"00074d87af"	"00074eff82"	-0.219
"00074d87af"	"00074f0477"	-0.4389
"00074d87af"	"00074f08c3"	0.0527
"00074d87af"	"00074f1859"	0.0147
"00074d87af"	"00074f2268"	-0.2481
"00074d87af"	"00074f28be"	0.1187
"00074d87af"	"00074f294b"	-0.0568
"00074d87af"	"00074f2ddd"	-0.2383
"00074d87af"	"00074f2e75"	-0.0686
"00074d87af"	"00074f3088"	-0.0176
"00074d87af"	"00074f5a1c"	-0.1287
"00074d87af"	"00074f75b7"	-0.2619
"00074d87af"	"00074f8cd9"	0.0743
"00074d87af"	"00074f96dc"	-0.0235
"00074d87af"	"00074fabaa"	0.1429
"00074d87af"	"00074facd9"	-0.1701
"00074d87af"	"00074fae3c"	-0.2068
"00074d87af"	"00074fb0a8"	-0.0336
"00074d87af"	"00074fb4e4"	-0.1093
"00074d87af"	"00074fb7c2"	0.3125
"00074d87af"	"00074fbd36"	-0.1671
"00074d87af"	"00074fc27f"	-0.1209
"00074d87af"	"00074fc31d"	0.0568
"00074d87af"	"00074fd569"	-0.0364
"00074d87af"	"00074fef15"	-0.2256

"00074d87af"	"00074ff562"	-0.2697
"00074d87af"	"00075007ca"	-0.3496
"00074d87af"	"0007500b86"	0.2435
"00074d87af"	"0007500d05"	0.0064
"00074d87af"	"0007500ee4"	-0.0747
"00074d87af"	"0007500eee"	-0.3278
"00074d87af"	"00075013dc"	-0.046
"00074d87af"	"000757b515"	-0.2413
"00074d87af"	"000757bc5a"	0.3019
"00074d87af"	"000757c320"	-0.1145
"00074d87af"	"000757c9aa"	-0.2027
"00074d87af"	"000757ccbe"	0.2507
"00074d87af"	"000757cfa9"	-0.2779
"00074d87af"	"000757d390"	-0.1514
"00074d87af"	"000757d393"	0.1449
"00074d87af"	"000757d598"	-0.3702
"00074d87af"	"000757d5a2"	-0.5324
"00074d87af"	"000757d790"	0.0154
"00074d87af"	"000757e30c"	-0.0677
"00074d87af"	"000757e4b0"	0.1038
"00074d87af"	"000757e7a0"	-0.2245
"00074d87af"	"000757e8b3"	0.0437
"00074d87af"	"000757f627"	0.2397
"00074d87af"	"000757f925"	0.0127
"00074d87af"	"000757fa08"	-0.1402
"00074d87af"	"000757fe52"	0.0032
"00074d87af"	"000758024a"	-0.0317
"00074d87af"	"00075804bb"	-0.0766
"00074d87af"	"00075a0c04"	-0.0437
"00074d87af"	"00075a3110"	-0.1134
"00074d87af"	"00075a341a"	0.0361
"00074d87af"	"00075a3dcf"	-0.0479
"00074d87af"	"00075a3e22"	0.1714
"00074d87af"	"00075a48d8"	0.1207
"00074d87af"	"00075a5cfb"	0.0055
"00074d87af"	"00075a6151"	-0.0029
"00074d87af"	"00075a6708"	-0.0012
"00074d87af"	"00075a7319"	-0.2028
"00074d87af"	"00075a7723"	0.2289
"00074d87af"	"00075a778b"	0.4002
"00074d87af"	"00075a7b8e"	0.1321
"00074d87af"	"00075a7c79"	0.1482
"00074d87af"	"00075a81b6"	0.1962
"00074d87af"	"00075a82ac"	0.3279
"00074d87af"	"00075a98e5"	0.5579
"00074d87af"	"00075b0d29"	0.0727
"00074d87af"	"00075b102a"	-0.0427
"00074d87af"	"00075b1074"	-0.1707
"00074d87af"	"00075b135d"	0.0568
"00074d87af"	"00075b138b"	0.2843

"00074d87af"	"00075b13a0"	-0.0502
"00074d87af"	"00075b13bd"	0.0048
"00074d87af"	"00075b16a9"	-0.191
"00074d87af"	"00075b1a28"	-0.0922
"00074d87af"	"00075b1a97"	-0.0507
"00074d87af"	"00075b1c7b"	-0.1571
"00074d87af"	"00075b1d24"	-0.2861
"00074d87af"	"00075b202b"	-0.0216
"00074d87af"	"00075b22cb"	-0.0738
"00074d87af"	"00075b22da"	-0.1564
"00074d87af"	"00075b2556"	-0.1118
"00074d87af"	"00075b25de"	-0.1867
"00074d87af"	"00075b260c"	-0.2441
"00074d87af"	"00075b26f1"	-0.2777
"00074d87af"	"00075b2920"	0.0256
"00074d87af"	"00075b2a64"	-0.1138
"00074d87af"	"00075b2a9d"	-0.2084
"00074d87af"	"00075b2b37"	-0.4673
"00074d87af"	"00075b2cdd"	0.0355
"00074d87af"	"00075b3038"	0.0577
"00074d87af"	"00075b30fe"	-0.1144
"00074d87af"	"00075b3362"	-0.3806
"00074d87af"	"00075b350a"	-0.0756
"00074d87af"	"00075b350e"	0.0289
"00074d87af"	"00075b3651"	-0.2453
"00074d87af"	"00075b38ca"	-0.1354
"00074d87af"	"00075b39cc"	-0.1564
"00074d87af"	"00075b3e1e"	0.0014
"00074d87af"	"00075b3e57"	0.0922
"00074d87af"	"00075b4079"	-0.2715
"00074d87af"	"00075b4150"	-0.119
"00074d87af"	"00075b4194"	0.0513
"00074d87af"	"00075b42d5"	0.1541
"00074d87af"	"00075b4424"	-0.064
"00074d87af"	"00075b4470"	0.2678
"00074d87af"	"00075b47ed"	-0.0634
"00074d87af"	"00075b4850"	0.153
"00074d87af"	"00075b4ca0"	-0.1335
"00074d87af"	"00075b4d7f"	0.1491
"00074d87af"	"00075b520f"	-0.1351
"00074d87af"	"00075b525f"	-0.2056
"00074d87af"	"00075b58f8"	-0.1098
"00074d87af"	"00075b5bcc"	-0.2171
"00074d87af"	"00075b5bfa"	-0.3997
"00074d87af"	"00075b6339"	-0.0042
"00074d87af"	"00075b6658"	-0.3604
"00074d87af"	"00075b679a"	-0.1632
"00074d87af"	"00075b6cb7"	-0.1114
"00074d87af"	"00075b6df8"	0.0383
"00074d87af"	"00075b6ff6"	-0.1861

"00074d87af"	"00075b70ee"	-0.0389
"00074d87af"	"00075b7157"	-0.214
"00074d87af"	"00075b7225"	-0.1886
"00074d87af"	"00075b7c89"	-0.0046
"00074d87af"	"00075b9048"	0.1376
"00074d87af"	"00075d0801"	-0.0753
"00074d87af"	"00075d1820"	-0.2946
"00074d87af"	"00075d1f3d"	0.0633
"00074d87af"	"00075d2329"	-0.0952
"00074d87af"	"00075d2b9b"	-0.0674
"00074d87af"	"00075d3941"	-0.1655
"00074d87af"	"00075d3e96"	-0.289
"00074d87af"	"00075d4864"	-0.0848
"00074d87af"	"00075d5961"	-0.0963
"00074d87af"	"00075d5a63"	0.0494
"00074d87af"	"00075d6150"	-0.2923
"00074d87af"	"00075d67d0"	-0.2534
"00074d87af"	"00075d67e2"	0.642
"00074d87af"	"00075d73fc"	0.0015
"00074d87af"	"00075d7729"	-0.2719
"00074d87af"	"00075d778c"	-0.3607
"00074d87af"	"00075d7b9e"	-0.1803
"00074d87af"	"00075d7c8f"	-0.0143
"00074d87af"	"00075d804d"	0.1288
"00074d87af"	"00075d819f"	-0.1068
"00074d87af"	"00075d8601"	-0.1228
"00074d87af"	"00075d8c6a"	0.1288
"00074d87af"	"00075dfedc"	-0.3016
"00074d87af"	"00075e05f2"	0.1261
"00074d87af"	"00075e0837"	-0.1454
"00074d87af"	"00075e092e"	-0.0306
"00074d87af"	"00075e0965"	-0.1484
"00074d87af"	"00075e0bc8"	0.0056
"00074d87af"	"00075e0fbb"	-0.2327
"00074d8813"	"00074d8817"	-0.0494
"00074d8813"	"00074d8a39"	-0.2073
"00074d8813"	"00074d8ad8"	0.0937
"00074d8813"	"00074d8c26"	0.1152
"00074d8813"	"00074d8ca5"	-0.214
"00074d8813"	"00074d8e0a"	0.052
"00074d8813"	"00074d9179"	-0.4165
"00074d8813"	"00074d929f"	-0.585
"00074d8813"	"00074d93c4"	-0.1054
"00074d8813"	"00074d93d0"	-0.4642
"00074d8813"	"00074d945b"	-0.2531
"00074d8813"	"00074d966c"	-0.0479
"00074d8813"	"00074d9953"	0.1001
"00074d8813"	"00074d99b2"	-0.1271
"00074d8813"	"00074d99f3"	-0.1545
"00074d8813"	"00074d9afd"	-0.6053

"00074d8813"	"00074d9c87"	−0.0487
"00074d8813"	"00074d9e9e"	−0.2864
"00074d8813"	"00074d9f30"	0.0563
"00074d8813"	"00074da036"	−0.0091
"00074d8813"	"00074da082"	−0.4765
"00074d8813"	"00074da136"	−0.2144
"00074d8813"	"00074da3ed"	−0.1624
"00074d8813"	"00074da4ac"	0.0768
"00074d8813"	"00074da4b8"	0.2171
"00074d8813"	"00074da5e8"	0.0098
"00074d8813"	"00074da6b4"	0.0202
"00074d8813"	"00074daa3c"	0.0035
"00074d8813"	"00074daaf6"	−0.4252
"00074d8813"	"00074dad20"	−0.2783
"00074d8813"	"00074db098"	0.1873
"00074d8813"	"00074db231"	0.0085
"00074d8813"	"00074db3a3"	−0.0397
"00074d8813"	"00074db5d6"	−0.127
"00074d8813"	"00074db632"	0.0549
"00074d8813"	"00074db688"	−0.1367
"00074d8813"	"00074db8a6"	−0.1438
"00074d8813"	"00074dba19"	−0.0164
"00074d8813"	"00074dbc2e"	−0.1464
"00074d8813"	"00074dbe51"	−0.0073
"00074d8813"	"00074dbe5f"	−0.0458
"00074d8813"	"00074dbf6d"	−0.0264
"00074d8813"	"00074dc4a5"	−0.4911
"00074d8813"	"00074dc50c"	−0.1593
"00074d8813"	"00074dcdfa"	−0.1867
"00074d8813"	"00074dcf5f"	−0.166
"00074d8813"	"00074dd007"	0.3097
"00074d8813"	"00074dd163"	−0.0818
"00074d8813"	"00074dd3df"	−0.2313
"00074d8813"	"00074dd577"	−0.2318
"00074d8813"	"00074dd62e"	−0.1143
"00074d8813"	"00074dd73c"	−0.0982
"00074d8813"	"00074dda10"	−0.1432
"00074d8813"	"00074ddab8"	−0.1246
"00074d8813"	"00074ddd3d"	−0.1619
"00074d8813"	"00074ddf16"	0.0175
"00074d8813"	"00074ddfc1"	−0.0665
"00074d8813"	"00074de21a"	−0.4398
"00074d8813"	"00074de2a9"	0.1705
"00074d8813"	"00074de544"	−0.1808
"00074d8813"	"00074de98a"	−0.1077
"00074d8813"	"00074dea7e"	−0.3677
"00074d8813"	"00074debd9"	0.2162
"00074d8813"	"00074deca3"	−0.1698
"00074d8813"	"00074def43"	−0.179
"00074d8813"	"00074def99"	−0.3764

"00074d8813"	"00074ecdad"	-0.3542
"00074d8813"	"00074ecf28"	-0.4202
"00074d8813"	"00074ed1e1"	-0.2979
"00074d8813"	"00074ed83b"	0.0601
"00074d8813"	"00074ee5e3"	-0.0483
"00074d8813"	"00074ee6e0"	-0.0364
"00074d8813"	"00074eea3a"	-0.1975
"00074d8813"	"00074eff82"	-0.1817
"00074d8813"	"00074f0477"	-0.0598
"00074d8813"	"00074f08c3"	-0.1583
"00074d8813"	"00074f1859"	-0.1318
"00074d8813"	"00074f2268"	-0.2275
"00074d8813"	"00074f28be"	-0.087
"00074d8813"	"00074f294b"	-0.0618
"00074d8813"	"00074f2ddd"	-0.2734
"00074d8813"	"00074f2e75"	-0.3748
"00074d8813"	"00074f3088"	-0.2271
"00074d8813"	"00074f5a1c"	-0.0699
"00074d8813"	"00074f75b7"	0.0638
"00074d8813"	"00074f8cd9"	-0.0273
"00074d8813"	"00074f96dc"	-0.5737
"00074d8813"	"00074fabaa"	-0.1382
"00074d8813"	"00074facd9"	0.032
"00074d8813"	"00074fae3c"	-0.3394
"00074d8813"	"00074fb0a8"	0.0732
"00074d8813"	"00074fb4e4"	-0.2818
"00074d8813"	"00074fb7c2"	-0.1792
"00074d8813"	"00074fbd36"	-0.0719
"00074d8813"	"00074fc27f"	0.4983
"00074d8813"	"00074fc31d"	-0.1173
"00074d8813"	"00074fd569"	-0.3662
"00074d8813"	"00074fef15"	-0.3979
"00074d8813"	"00074ff562"	-0.5199
"00074d8813"	"00075007ca"	0.0615
"00074d8813"	"0007500b86"	0.2685
"00074d8813"	"0007500d05"	0.0265
"00074d8813"	"0007500ee4"	-0.0321
"00074d8813"	"0007500eee"	0.0104
"00074d8813"	"00075013dc"	-0.3993
"00074d8813"	"000757b515"	0.0523
"00074d8813"	"000757bc5a"	0.1649
"00074d8813"	"000757c320"	-0.1439
"00074d8813"	"000757c9aa"	0.1311
"00074d8813"	"000757ccbe"	-0.1187
"00074d8813"	"000757cfa9"	-0.0754
"00074d8813"	"000757d390"	-0.1429
"00074d8813"	"000757d393"	0.0851
"00074d8813"	"000757d598"	0.0359
"00074d8813"	"000757d5a2"	-0.0644
"00074d8813"	"000757d790"	0.0483

"00074d8813"	"000757e30c"	0.297
"00074d8813"	"000757e4b0"	-0.209
"00074d8813"	"000757e7a0"	-0.0431
"00074d8813"	"000757e8b3"	-0.0014
"00074d8813"	"000757f627"	-0.0343
"00074d8813"	"000757f925"	0.0625
"00074d8813"	"000757fa08"	-0.2431
"00074d8813"	"000757fe52"	0.0415
"00074d8813"	"000758024a"	0.2588
"00074d8813"	"00075804bb"	0.0287
"00074d8813"	"00075a0c04"	-0.4559
"00074d8813"	"00075a3110"	-0.2018
"00074d8813"	"00075a341a"	-0.3634
"00074d8813"	"00075a3dcf"	-0.0836
"00074d8813"	"00075a3e22"	-0.188
"00074d8813"	"00075a48d8"	-0.2247
"00074d8813"	"00075a5cfb"	0.3154
"00074d8813"	"00075a6151"	0.0879
"00074d8813"	"00075a6708"	-0.0618
"00074d8813"	"00075a7319"	-0.1318
"00074d8813"	"00075a7723"	0.003
"00074d8813"	"00075a778b"	-0.1999
"00074d8813"	"00075a7b8e"	0.1093
"00074d8813"	"00075a7c79"	-0.1274
"00074d8813"	"00075a81b6"	-0.4346
"00074d8813"	"00075a82ac"	-0.0847
"00074d8813"	"00075a98e5"	-0.3145
"00074d8813"	"00075b0d29"	-0.1322
"00074d8813"	"00075b102a"	-0.0282
"00074d8813"	"00075b1074"	-0.3394
"00074d8813"	"00075b135d"	-0.2152
"00074d8813"	"00075b138b"	-0.057
"00074d8813"	"00075b13a0"	-0.0782
"00074d8813"	"00075b13bd"	-0.2941
"00074d8813"	"00075b16a9"	0.0205
"00074d8813"	"00075b1a28"	-0.2852
"00074d8813"	"00075b1a97"	-0.3557
"00074d8813"	"00075b1c7b"	-0.1057
"00074d8813"	"00075b1d24"	-0.0847
"00074d8813"	"00075b202b"	-0.0053
"00074d8813"	"00075b22cb"	-0.3023
"00074d8813"	"00075b22da"	-0.2914
"00074d8813"	"00075b2556"	-0.2504
"00074d8813"	"00075b25de"	0.0721
"00074d8813"	"00075b260c"	-0.0718
"00074d8813"	"00075b26f1"	-0.2864
"00074d8813"	"00075b2920"	0.2582
"00074d8813"	"00075b2a64"	0.3082
"00074d8813"	"00075b2a9d"	-0.1031
"00074d8813"	"00075b2b37"	-0.0988

"00074d8813"	"00075b2cdd"	-0.2943
"00074d8813"	"00075b3038"	-0.1364
"00074d8813"	"00075b30fe"	-0.3104
"00074d8813"	"00075b3362"	-0.2433
"00074d8813"	"00075b350a"	-0.2295
"00074d8813"	"00075b350e"	-0.1801
"00074d8813"	"00075b3651"	-0.3253
"00074d8813"	"00075b38ca"	-0.2031
"00074d8813"	"00075b39cc"	-0.2096
"00074d8813"	"00075b3e1e"	-0.1549
"00074d8813"	"00075b3e57"	0.0484
"00074d8813"	"00075b4079"	-0.0898
"00074d8813"	"00075b4150"	0.3499
"00074d8813"	"00075b4194"	0.0082
"00074d8813"	"00075b42d5"	-0.147
"00074d8813"	"00075b4424"	-0.1789
"00074d8813"	"00075b4470"	0.1308
"00074d8813"	"00075b47ed"	-0.3977
"00074d8813"	"00075b4850"	0.1031
"00074d8813"	"00075b4ca0"	-0.2544
"00074d8813"	"00075b4d7f"	-0.0567
"00074d8813"	"00075b520f"	-0.2995
"00074d8813"	"00075b525f"	-0.4585
"00074d8813"	"00075b58f8"	0.4765
"00074d8813"	"00075b5bcc"	-0.073
"00074d8813"	"00075b5bfa"	-0.2371
"00074d8813"	"00075b6339"	0.04
"00074d8813"	"00075b6658"	-0.3647
"00074d8813"	"00075b679a"	-0.2025
"00074d8813"	"00075b6cb7"	0.4023
"00074d8813"	"00075b6df8"	-0.3206
"00074d8813"	"00075b6ff6"	0.0967
"00074d8813"	"00075b70ee"	-0.2676
"00074d8813"	"00075b7157"	0.001
"00074d8813"	"00075b7225"	-0.3556
"00074d8813"	"00075b7c89"	-0.3098
"00074d8813"	"00075b9048"	0.1301
"00074d8813"	"00075d0801"	0.0125
"00074d8813"	"00075d1820"	-0.3046
"00074d8813"	"00075d1f3d"	-0.0817
"00074d8813"	"00075d2329"	-0.0847
"00074d8813"	"00075d2b9b"	-0.0378
"00074d8813"	"00075d3941"	-0.5696
"00074d8813"	"00075d3e96"	-0.086
"00074d8813"	"00075d4864"	-0.1587
"00074d8813"	"00075d5961"	0.0923
"00074d8813"	"00075d5a63"	-0.0222
"00074d8813"	"00075d6150"	-0.4318
"00074d8813"	"00075d67d0"	-0.3235
"00074d8813"	"00075d67e2"	-0.263

"00074d8813"	"00075d73fc"	-0.3485
"00074d8813"	"00075d7729"	-0.3344
"00074d8813"	"00075d778c"	-0.4003
"00074d8813"	"00075d7b9e"	-0.2609
"00074d8813"	"00075d7c8f"	-0.2856
"00074d8813"	"00075d804d"	0.2307
"00074d8813"	"00075d819f"	-0.1364
"00074d8813"	"00075d8601"	-0.3058
"00074d8813"	"00075d8c6a"	-0.2292
"00074d8813"	"00075dfedc"	-0.2776
"00074d8813"	"00075e05f2"	0.1629
"00074d8813"	"00075e0837"	-0.0495
"00074d8813"	"00075e092e"	-0.1753
"00074d8813"	"00075e0965"	0.3154
"00074d8813"	"00075e0bc8"	0.3673
"00074d8813"	"00075e0fbb"	-0.176
"00074d8817"	"00074d8a39"	0.3513
"00074d8817"	"00074d8ad8"	0.1672
"00074d8817"	"00074d8c26"	0.1505
"00074d8817"	"00074d8ca5"	0.0487
"00074d8817"	"00074d8e0a"	0.3287
"00074d8817"	"00074d9179"	-0.12
"00074d8817"	"00074d929f"	0.0482
"00074d8817"	"00074d93c4"	-0.1162
"00074d8817"	"00074d93d0"	0.1214
"00074d8817"	"00074d945b"	0.1434
"00074d8817"	"00074d966c"	0.0081
"00074d8817"	"00074d9953"	0.1266
"00074d8817"	"00074d99b2"	-0.0528
"00074d8817"	"00074d99f3"	0.3033
"00074d8817"	"00074d9afd"	-0.2689
"00074d8817"	"00074d9c87"	0.083
"00074d8817"	"00074d9e9e"	-0.4179
"00074d8817"	"00074d9f30"	0.0216
"00074d8817"	"00074da036"	0.0523
"00074d8817"	"00074da082"	-0.0358
"00074d8817"	"00074da136"	-0.2308
"00074d8817"	"00074da3ed"	-0.2398
"00074d8817"	"00074da4ac"	0.3658
"00074d8817"	"00074da4b8"	0.382
"00074d8817"	"00074da5e8"	0.1006
"00074d8817"	"00074da6b4"	0.1212
"00074d8817"	"00074daa3c"	0.1466
"00074d8817"	"00074daaf6"	0.1001
"00074d8817"	"00074dad20"	-0.1499
"00074d8817"	"00074db098"	0.1225
"00074d8817"	"00074db231"	0.2971
"00074d8817"	"00074db3a3"	0.2869
"00074d8817"	"00074db5d6"	0.1429
"00074d8817"	"00074db632"	0.3175

"00074d8817"	"00074db688"	−0.1036
"00074d8817"	"00074db8a6"	−0.0493
"00074d8817"	"00074dba19"	0.1363
"00074d8817"	"00074dbc2e"	0.0509
"00074d8817"	"00074dbe51"	0.0052
"00074d8817"	"00074dbe5f"	−0.2025
"00074d8817"	"00074dbf6d"	0.0595
"00074d8817"	"00074dc4a5"	0.0855
"00074d8817"	"00074dc50c"	0.254
"00074d8817"	"00074dcdfa"	0.2112
"00074d8817"	"00074dcf5f"	0.0787
"00074d8817"	"00074dd007"	0.2798
"00074d8817"	"00074dd163"	−0.0498
"00074d8817"	"00074dd3df"	−0.2484
"00074d8817"	"00074dd577"	−0.1563
"00074d8817"	"00074dd62e"	0.0527
"00074d8817"	"00074dd73c"	−0.05
"00074d8817"	"00074dda10"	0.096
"00074d8817"	"00074ddab8"	0.321
"00074d8817"	"00074ddd3d"	−0.1149
"00074d8817"	"00074ddf16"	0.0394
"00074d8817"	"00074ddfc1"	0.068
"00074d8817"	"00074de21a"	0.4418
"00074d8817"	"00074de2a9"	0.2817
"00074d8817"	"00074de544"	0.0684
"00074d8817"	"00074de98a"	0.0865
"00074d8817"	"00074dea7e"	−0.1962
"00074d8817"	"00074debd9"	−0.0888
"00074d8817"	"00074deca3"	−0.0744
"00074d8817"	"00074def43"	0.0054
"00074d8817"	"00074def99"	0.0909
"00074d8817"	"00074ecdad"	−0.329
"00074d8817"	"00074ecf28"	−0.0602
"00074d8817"	"00074ed1e1"	−0.1752
"00074d8817"	"00074ed83b"	0.3542
"00074d8817"	"00074ee5e3"	0.0554
"00074d8817"	"00074ee6e0"	−0.0074
"00074d8817"	"00074eea3a"	−0.0324
"00074d8817"	"00074eff82"	−0.0536
"00074d8817"	"00074f0477"	−0.2364
"00074d8817"	"00074f08c3"	0.5227
"00074d8817"	"00074f1859"	−2e−04
"00074d8817"	"00074f2268"	−0.1243
"00074d8817"	"00074f28be"	0.4066
"00074d8817"	"00074f294b"	−0.172
"00074d8817"	"00074f2ddd"	−0.071
"00074d8817"	"00074f2e75"	−0.4215
"00074d8817"	"00074f3088"	5e−04
"00074d8817"	"00074f5a1c"	−0.1172
"00074d8817"	"00074f75b7"	−0.0482

"00074d8817"	"00074f8cd9"	-0.1328
"00074d8817"	"00074f96dc"	0.0776
"00074d8817"	"00074fabaa"	0.0532
"00074d8817"	"00074facd9"	0.104
"00074d8817"	"00074fae3c"	0.2453
"00074d8817"	"00074fb0a8"	0.3775
"00074d8817"	"00074fb4e4"	0.3834
"00074d8817"	"00074fb7c2"	-0.0296
"00074d8817"	"00074fbd36"	0.0699
"00074d8817"	"00074fc27f"	0.0403
"00074d8817"	"00074fc31d"	-0.0037
"00074d8817"	"00074fd569"	0.0449
"00074d8817"	"00074fef15"	0.0014
"00074d8817"	"00074ff562"	-0.3001
"00074d8817"	"00075007ca"	0.0726
"00074d8817"	"0007500b86"	-0.0161
"00074d8817"	"0007500d05"	-0.1786
"00074d8817"	"0007500ee4"	-0.0642
"00074d8817"	"0007500eee"	-0.0743
"00074d8817"	"00075013dc"	0.0711
"00074d8817"	"000757b515"	0.2334
"00074d8817"	"000757bc5a"	0.3087
"00074d8817"	"000757c320"	0.2834
"00074d8817"	"000757c9aa"	0.5987
"00074d8817"	"000757ccbe"	-0.1704
"00074d8817"	"000757cfa9"	-0.0164
"00074d8817"	"000757d390"	0.2222
"00074d8817"	"000757d393"	0.0627
"00074d8817"	"000757d598"	0.0947
"00074d8817"	"000757d5a2"	0.0301
"00074d8817"	"000757d790"	-0.0293
"00074d8817"	"000757e30c"	0.3092
"00074d8817"	"000757e4b0"	0.3795
"00074d8817"	"000757e7a0"	0.0527
"00074d8817"	"000757e8b3"	0.2005
"00074d8817"	"000757f627"	0.6189
"00074d8817"	"000757f925"	0.0874
"00074d8817"	"000757fa08"	-0.3231
"00074d8817"	"000757fe52"	-0.2159
"00074d8817"	"000758024a"	0.208
"00074d8817"	"00075804bb"	0.1868
"00074d8817"	"00075a0c04"	0.0976
"00074d8817"	"00075a3110"	0.0441
"00074d8817"	"00075a341a"	-0.2649
"00074d8817"	"00075a3dcf"	-0.1189
"00074d8817"	"00075a3e22"	0.3128
"00074d8817"	"00075a48d8"	-0.0672
"00074d8817"	"00075a5cfb"	0.1665
"00074d8817"	"00075a6151"	0.2143
"00074d8817"	"00075a6708"	0.0868

"00074d8817"	"00075a7319"	0.296
"00074d8817"	"00075a7723"	-0.0837
"00074d8817"	"00075a778b"	-0.1842
"00074d8817"	"00075a7b8e"	0.3335
"00074d8817"	"00075a7c79"	0.1316
"00074d8817"	"00075a81b6"	0.0497
"00074d8817"	"00075a82ac"	0.0513
"00074d8817"	"00075a98e5"	-0.0255
"00074d8817"	"00075b0d29"	0.0726
"00074d8817"	"00075b102a"	0.2912
"00074d8817"	"00075b1074"	-0.2015
"00074d8817"	"00075b135d"	0.0924
"00074d8817"	"00075b138b"	0.3272
"00074d8817"	"00075b13a0"	-0.3109
"00074d8817"	"00075b13bd"	0.2599
"00074d8817"	"00075b16a9"	0.0252
"00074d8817"	"00075b1a28"	-0.1658
"00074d8817"	"00075b1a97"	0.103
"00074d8817"	"00075b1c7b"	-0.2587
"00074d8817"	"00075b1d24"	0.0338
"00074d8817"	"00075b202b"	0.3665
"00074d8817"	"00075b22cb"	-0.1155
"00074d8817"	"00075b22da"	-0.0553
"00074d8817"	"00075b2556"	-0.0432
"00074d8817"	"00075b25de"	0.1979
"00074d8817"	"00075b260c"	0.2043
"00074d8817"	"00075b26f1"	0.2188
"00074d8817"	"00075b2920"	0.3277
"00074d8817"	"00075b2a64"	0.1099
"00074d8817"	"00075b2a9d"	0.2703
"00074d8817"	"00075b2b37"	-0.1241
"00074d8817"	"00075b2cdd"	-0.0573
"00074d8817"	"00075b3038"	0.0659
"00074d8817"	"00075b30fe"	-0.1973
"00074d8817"	"00075b3362"	0.0232
"00074d8817"	"00075b350a"	-0.048
"00074d8817"	"00075b350e"	0.0264
"00074d8817"	"00075b3651"	0.0539
"00074d8817"	"00075b38ca"	-0.2705
"00074d8817"	"00075b39cc"	0.1282
"00074d8817"	"00075b3e1e"	0.0397
"00074d8817"	"00075b3e57"	0.0197
"00074d8817"	"00075b4079"	-0.1359
"00074d8817"	"00075b4150"	0.0947
"00074d8817"	"00075b4194"	0.3929
"00074d8817"	"00075b42d5"	-0.1879
"00074d8817"	"00075b4424"	-0.159
"00074d8817"	"00075b4470"	0.3087
"00074d8817"	"00075b47ed"	0.0533
"00074d8817"	"00075b4850"	0.3484

"00074d8817"	"00075b4ca0"	0.2123
"00074d8817"	"00075b4d7f"	0.089
"00074d8817"	"00075b520f"	0.1872
"00074d8817"	"00075b525f"	0.0178
"00074d8817"	"00075b58f8"	−0.0466
"00074d8817"	"00075b5bcc"	0.1359
"00074d8817"	"00075b5bfa"	−0.1961
"00074d8817"	"00075b6339"	0.2194
"00074d8817"	"00075b6658"	−0.0773
"00074d8817"	"00075b679a"	0.1789
"00074d8817"	"00075b6cb7"	0.0208
"00074d8817"	"00075b6df8"	−0.1817
"00074d8817"	"00075b6ff6"	−0.0981
"00074d8817"	"00075b70ee"	−0.0089
"00074d8817"	"00075b7157"	0.0271
"00074d8817"	"00075b7225"	0.0446
"00074d8817"	"00075b7c89"	0.0759
"00074d8817"	"00075b9048"	0.1541
"00074d8817"	"00075d0801"	0.0781
"00074d8817"	"00075d1820"	0.1721
"00074d8817"	"00075d1f3d"	0.2307
"00074d8817"	"00075d2329"	−0.1232
"00074d8817"	"00075d2b9b"	0.1761
"00074d8817"	"00075d3941"	−0.0283
"00074d8817"	"00075d3e96"	0.1686
"00074d8817"	"00075d4864"	−0.1917
"00074d8817"	"00075d5961"	0.0772
"00074d8817"	"00075d5a63"	0.1564
"00074d8817"	"00075d6150"	−0.0264
"00074d8817"	"00075d67d0"	−0.0559
"00074d8817"	"00075d67e2"	−0.0392
"00074d8817"	"00075d73fc"	−0.0436
"00074d8817"	"00075d7729"	0.1201
"00074d8817"	"00075d778c"	−0.2297
"00074d8817"	"00075d7b9e"	−0.0471
"00074d8817"	"00075d7c8f"	0.1134
"00074d8817"	"00075d804d"	0.3075
"00074d8817"	"00075d819f"	0.2385
"00074d8817"	"00075d8601"	0.2799
"00074d8817"	"00075d8c6a"	0.0465
"00074d8817"	"00075dfedc"	0.092
"00074d8817"	"00075e05f2"	−0.097
"00074d8817"	"00075e0837"	0.2328
"00074d8817"	"00075e092e"	−0.1445
"00074d8817"	"00075e0965"	−0.2198
"00074d8817"	"00075e0bc8"	0.349
"00074d8817"	"00075e0fbb"	−0.274
"00074d8a39"	"00074d8ad8"	−0.1624
"00074d8a39"	"00074d8c26"	−0.1292
"00074d8a39"	"00074d8ca5"	−0.2344

"00074d8a39"	"00074d8e0a"	-0.0788
"00074d8a39"	"00074d9179"	-0.2097
"00074d8a39"	"00074d929f"	-0.0089
"00074d8a39"	"00074d93c4"	-0.0299
"00074d8a39"	"00074d93d0"	-0.0385
"00074d8a39"	"00074d945b"	-0.1215
"00074d8a39"	"00074d966c"	0.0096
"00074d8a39"	"00074d9953"	0.0474
"00074d8a39"	"00074d99b2"	0.0583
"00074d8a39"	"00074d99f3"	0.403
"00074d8a39"	"00074d9afd"	0.0286
"00074d8a39"	"00074d9c87"	0.0277
"00074d8a39"	"00074d9e9e"	-0.239
"00074d8a39"	"00074d9f30"	0.3498
"00074d8a39"	"00074da036"	-0.0072
"00074d8a39"	"00074da082"	-0.0221
"00074d8a39"	"00074da136"	-0.1548
"00074d8a39"	"00074da3ed"	-0.2585
"00074d8a39"	"00074da4ac"	0.2157
"00074d8a39"	"00074da4b8"	0.1424
"00074d8a39"	"00074da5e8"	0.0015
"00074d8a39"	"00074da6b4"	-0.0271
"00074d8a39"	"00074daa3c"	0.0656
"00074d8a39"	"00074daaf6"	0.4504
"00074d8a39"	"00074dad20"	-0.0922
"00074d8a39"	"00074db098"	-0.1589
"00074d8a39"	"00074db231"	-0.1389
"00074d8a39"	"00074db3a3"	-0.0507
"00074d8a39"	"00074db5d6"	0.2356
"00074d8a39"	"00074db632"	0.1646
"00074d8a39"	"00074db688"	-3e-04
"00074d8a39"	"00074db8a6"	-0.0268
"00074d8a39"	"00074dba19"	0.0186
"00074d8a39"	"00074dbc2e"	-0.1001
"00074d8a39"	"00074dbe51"	0.0187
"00074d8a39"	"00074dbe5f"	-0.2481
"00074d8a39"	"00074dbf6d"	-0.1463
"00074d8a39"	"00074dc4a5"	-0.0969
"00074d8a39"	"00074dc50c"	-0.1672
"00074d8a39"	"00074dcdfa"	0.029
"00074d8a39"	"00074dcf5f"	-0.0278
"00074d8a39"	"00074dd007"	0.1593
"00074d8a39"	"00074dd163"	-0.1357
"00074d8a39"	"00074dd3df"	-0.1068
"00074d8a39"	"00074dd577"	0.0638
"00074d8a39"	"00074dd62e"	0.1373
"00074d8a39"	"00074dd73c"	0.0831
"00074d8a39"	"00074dda10"	0.0399
"00074d8a39"	"00074ddab8"	0.0878
"00074d8a39"	"00074ddd3d"	-0.0659

"00074d8a39"	"00074ddf16"	-0.1324
"00074d8a39"	"00074ddfc1"	-0.1218
"00074d8a39"	"00074de21a"	0.1037
"00074d8a39"	"00074de2a9"	0.1514
"00074d8a39"	"00074de544"	0.0431
"00074d8a39"	"00074de98a"	-0.1375
"00074d8a39"	"00074dea7e"	-0.3561
"00074d8a39"	"00074debd9"	-0.1966
"00074d8a39"	"00074deca3"	-0.1031
"00074d8a39"	"00074def43"	-0.127
"00074d8a39"	"00074def99"	0.1904
"00074d8a39"	"00074ecdad"	-0.3482
"00074d8a39"	"00074ecf28"	0.1237
"00074d8a39"	"00074ed1e1"	-0.3566
"00074d8a39"	"00074ed83b"	0.0684
"00074d8a39"	"00074ee5e3"	0.0028
"00074d8a39"	"00074ee6e0"	0.2121
"00074d8a39"	"00074eea3a"	-0.1178
"00074d8a39"	"00074eff82"	0.423
"00074d8a39"	"00074f0477"	-0.2855
"00074d8a39"	"00074f08c3"	0.1036
"00074d8a39"	"00074f1859"	-0.0255
"00074d8a39"	"00074f2268"	0.0364
"00074d8a39"	"00074f28be"	0.029
"00074d8a39"	"00074f294b"	-0.1675
"00074d8a39"	"00074f2ddd"	-0.0827
"00074d8a39"	"00074f2e75"	-0.3671
"00074d8a39"	"00074f3088"	0.1938
"00074d8a39"	"00074f5a1c"	0.0531
"00074d8a39"	"00074f75b7"	0.1573
"00074d8a39"	"00074f8cd9"	-0.132
"00074d8a39"	"00074f96dc"	0.0381
"00074d8a39"	"00074fabaa"	-0.219
"00074d8a39"	"00074facd9"	0.0169
"00074d8a39"	"00074fae3c"	0.1626
"00074d8a39"	"00074fb0a8"	-0.0892
"00074d8a39"	"00074fb4e4"	-0.0835
"00074d8a39"	"00074fb7c2"	-0.1101
"00074d8a39"	"00074fbd36"	-0.1205
"00074d8a39"	"00074fc27f"	0.0022
"00074d8a39"	"00074fc31d"	-0.0634
"00074d8a39"	"00074fd569"	-0.0044
"00074d8a39"	"00074fef15"	0.3598
"00074d8a39"	"00074ff562"	-0.2463
"00074d8a39"	"00075007ca"	-0.1312
"00074d8a39"	"0007500b86"	0.0097
"00074d8a39"	"0007500d05"	0.0391
"00074d8a39"	"0007500ee4"	0.073
"00074d8a39"	"0007500eee"	-0.0654
"00074d8a39"	"00075013dc"	-0.0354

"00074d8a39"	"000757b515"	0.06
"00074d8a39"	"000757bc5a"	-0.0058
"00074d8a39"	"000757c320"	0.0418
"00074d8a39"	"000757c9aa"	0.2579
"00074d8a39"	"000757ccbe"	-0.0748
"00074d8a39"	"000757cfa9"	-0.2044
"00074d8a39"	"000757d390"	-0.0683
"00074d8a39"	"000757d393"	-0.0033
"00074d8a39"	"000757d598"	-0.0316
"00074d8a39"	"000757d5a2"	-0.0983
"00074d8a39"	"000757d790"	-0.0623
"00074d8a39"	"000757e30c"	-0.0729
"00074d8a39"	"000757e4b0"	0.0951
"00074d8a39"	"000757e7a0"	-0.0072
"00074d8a39"	"000757e8b3"	0.0616
"00074d8a39"	"000757f627"	0.1035
"00074d8a39"	"000757f925"	-0.0818
"00074d8a39"	"000757fa08"	-0.2484
"00074d8a39"	"000757fe52"	0.0479
"00074d8a39"	"000758024a"	-0.1747
"00074d8a39"	"00075804bb"	-0.1578
"00074d8a39"	"00075a0c04"	-0.0637
"00074d8a39"	"00075a3110"	0.0259
"00074d8a39"	"00075a341a"	-0.0515
"00074d8a39"	"00075a3dcf"	0.0017
"00074d8a39"	"00075a3e22"	0.0507
"00074d8a39"	"00075a48d8"	-0.0598
"00074d8a39"	"00075a5cfb"	-0.0983
"00074d8a39"	"00075a6151"	-0.1974
"00074d8a39"	"00075a6708"	-0.0581
"00074d8a39"	"00075a7319"	-0.0778
"00074d8a39"	"00075a7723"	-0.1466
"00074d8a39"	"00075a778b"	-0.2302
"00074d8a39"	"00075a7b8e"	0.0945
"00074d8a39"	"00075a7c79"	0.0112
"00074d8a39"	"00075a81b6"	0.1706
"00074d8a39"	"00075a82ac"	0.0983
"00074d8a39"	"00075a98e5"	-0.0386
"00074d8a39"	"00075b0d29"	-0.0466
"00074d8a39"	"00075b102a"	-0.0443
"00074d8a39"	"00075b1074"	-0.2974
"00074d8a39"	"00075b135d"	-0.1739
"00074d8a39"	"00075b138b"	0.0964
"00074d8a39"	"00075b13a0"	-0.1733
"00074d8a39"	"00075b13bd"	0.2207
"00074d8a39"	"00075b16a9"	-0.0908
"00074d8a39"	"00075b1a28"	-0.0617
"00074d8a39"	"00075b1a97"	0.2281
"00074d8a39"	"00075b1c7b"	-0.257
"00074d8a39"	"00075b1d24"	0.1823

"00074d8a39"	"00075b202b"	0.0091
"00074d8a39"	"00075b22cb"	-0.0426
"00074d8a39"	"00075b22da"	-0.0128
"00074d8a39"	"00075b2556"	0.0014
"00074d8a39"	"00075b25de"	0.0538
"00074d8a39"	"00075b260c"	0.1171
"00074d8a39"	"00075b26f1"	0.1265
"00074d8a39"	"00075b2920"	-0.0282
"00074d8a39"	"00075b2a64"	-0.1637
"00074d8a39"	"00075b2a9d"	-0.1514
"00074d8a39"	"00075b2b37"	-0.0162
"00074d8a39"	"00075b2cdd"	0.0582
"00074d8a39"	"00075b3038"	0.0431
"00074d8a39"	"00075b30fe"	-0.0619
"00074d8a39"	"00075b3362"	0.188
"00074d8a39"	"00075b350a"	-0.0492
"00074d8a39"	"00075b350e"	-0.1402
"00074d8a39"	"00075b3651"	0.1755
"00074d8a39"	"00075b38ca"	-0.2412
"00074d8a39"	"00075b39cc"	-0.1877
"00074d8a39"	"00075b3e1e"	-0.1379
"00074d8a39"	"00075b3e57"	0.2578
"00074d8a39"	"00075b4079"	-0.2745
"00074d8a39"	"00075b4150"	-0.1238
"00074d8a39"	"00075b4194"	0.0676
"00074d8a39"	"00075b42d5"	-0.1074
"00074d8a39"	"00075b4424"	0.0118
"00074d8a39"	"00075b4470"	-0.0214
"00074d8a39"	"00075b47ed"	-0.2212
"00074d8a39"	"00075b4850"	-0.0062
"00074d8a39"	"00075b4ca0"	-0.0062
"00074d8a39"	"00075b4d7f"	0.0848
"00074d8a39"	"00075b520f"	0.2333
"00074d8a39"	"00075b525f"	0.1815
"00074d8a39"	"00075b58f8"	-0.1973
"00074d8a39"	"00075b5bcc"	0.2754
"00074d8a39"	"00075b5bfa"	0.1268
"00074d8a39"	"00075b6339"	0.2049
"00074d8a39"	"00075b6658"	0.0878
"00074d8a39"	"00075b679a"	0.2878
"00074d8a39"	"00075b6cb7"	-0.2568
"00074d8a39"	"00075b6df8"	-0.0296
"00074d8a39"	"00075b6ff6"	0.126
"00074d8a39"	"00075b70ee"	-0.3229
"00074d8a39"	"00075b7157"	0.4335
"00074d8a39"	"00075b7225"	0.1948
"00074d8a39"	"00075b7c89"	-0.1227
"00074d8a39"	"00075b9048"	0.1826
"00074d8a39"	"00075d0801"	-0.1096
"00074d8a39"	"00075d1820"	0.0842

"00074d8a39"	"00075d1f3d"	0.0178
"00074d8a39"	"00075d2329"	-0.1015
"00074d8a39"	"00075d2b9b"	-0.1402
"00074d8a39"	"00075d3941"	0.0427
"00074d8a39"	"00075d3e96"	0.199
"00074d8a39"	"00075d4864"	-0.1612
"00074d8a39"	"00075d5961"	-0.0593
"00074d8a39"	"00075d5a63"	0.2435
"00074d8a39"	"00075d6150"	0.2598
"00074d8a39"	"00075d67d0"	-0.0409
"00074d8a39"	"00075d67e2"	0.0422
"00074d8a39"	"00075d73fc"	0.0138
"00074d8a39"	"00075d7729"	0.341
"00074d8a39"	"00075d778c"	0.1896
"00074d8a39"	"00075d7b9e"	0.4269
"00074d8a39"	"00075d7c8f"	-0.0597
"00074d8a39"	"00075d804d"	0.2647
"00074d8a39"	"00075d819f"	0.0539
"00074d8a39"	"00075d8601"	0.1621
"00074d8a39"	"00075d8c6a"	-0.1056
"00074d8a39"	"00075dfedc"	0.0062
"00074d8a39"	"00075e05f2"	-0.1199
"00074d8a39"	"00075e0837"	-0.0635
"00074d8a39"	"00075e092e"	-0.0454
"00074d8a39"	"00075e0965"	-0.3808
"00074d8a39"	"00075e0bc8"	0.0052
"00074d8a39"	"00075e0fbb"	-0.3477
"00074d8ad8"	"00074d8c26"	0.0733
"00074d8ad8"	"00074d8ca5"	0.123
"00074d8ad8"	"00074d8e0a"	0.2998
"00074d8ad8"	"00074d9179"	-0.1274
"00074d8ad8"	"00074d929f"	-0.1438
"00074d8ad8"	"00074d93c4"	0.0051
"00074d8ad8"	"00074d93d0"	0.0801
"00074d8ad8"	"00074d945b"	0.0035
"00074d8ad8"	"00074d966c"	0.2018
"00074d8ad8"	"00074d9953"	0.0882
"00074d8ad8"	"00074d99b2"	-0.1195
"00074d8ad8"	"00074d99f3"	0.14
"00074d8ad8"	"00074d9afd"	-0.3949
"00074d8ad8"	"00074d9c87"	0.3686
"00074d8ad8"	"00074d9e9e"	-0.3625
"00074d8ad8"	"00074d9f30"	-0.2183
"00074d8ad8"	"00074da036"	0.5452
"00074d8ad8"	"00074da082"	-0.1031
"00074d8ad8"	"00074da136"	-0.0462
"00074d8ad8"	"00074da3ed"	-0.1008
"00074d8ad8"	"00074da4ac"	0.2798
"00074d8ad8"	"00074da4b8"	0.2327
"00074d8ad8"	"00074da5e8"	0.4574

"00074d8ad8"	"00074da6b4"	-0.0308
"00074d8ad8"	"00074daa3c"	0.0113
"00074d8ad8"	"00074daaf6"	-0.1251
"00074d8ad8"	"00074dad20"	-0.0545
"00074d8ad8"	"00074db098"	0.3819
"00074d8ad8"	"00074db231"	0.1783
"00074d8ad8"	"00074db3a3"	0.4201
"00074d8ad8"	"00074db5d6"	-0.0954
"00074d8ad8"	"00074db632"	0.3057
"00074d8ad8"	"00074db688"	0.3608
"00074d8ad8"	"00074db8a6"	-0.0173
"00074d8ad8"	"00074dba19"	0.1985
"00074d8ad8"	"00074dbc2e"	0.1627
"00074d8ad8"	"00074dbe51"	0.3612
"00074d8ad8"	"00074dbe5f"	-0.298
"00074d8ad8"	"00074dbf6d"	0.3357
"00074d8ad8"	"00074dc4a5"	-0.4193
"00074d8ad8"	"00074dc50c"	0.4232
"00074d8ad8"	"00074dcdfa"	0.0934
"00074d8ad8"	"00074dcf5f"	0.0253
"00074d8ad8"	"00074dd007"	-0.0136
"00074d8ad8"	"00074dd163"	-0.0135
"00074d8ad8"	"00074dd3df"	-0.3604
"00074d8ad8"	"00074dd577"	0.2767
"00074d8ad8"	"00074dd62e"	-0.084
"00074d8ad8"	"00074dd73c"	0.1883
"00074d8ad8"	"00074dda10"	-0.1185
"00074d8ad8"	"00074ddab8"	0.151
"00074d8ad8"	"00074ddd3d"	0.3087
"00074d8ad8"	"00074ddf16"	0.1414
"00074d8ad8"	"00074ddfc1"	0.3079
"00074d8ad8"	"00074de21a"	-0.1857
"00074d8ad8"	"00074de2a9"	0.228
"00074d8ad8"	"00074de544"	0.0081
"00074d8ad8"	"00074de98a"	0.0717
"00074d8ad8"	"00074dea7e"	-0.0974
"00074d8ad8"	"00074debd9"	0.0696
"00074d8ad8"	"00074deca3"	0.0279
"00074d8ad8"	"00074def43"	0.0941
"00074d8ad8"	"00074def99"	0.0937
"00074d8ad8"	"00074ecdad"	-0.1403
"00074d8ad8"	"00074ecf28"	-0.378
"00074d8ad8"	"00074ed1e1"	-0.0093
"00074d8ad8"	"00074ed83b"	0.2096
"00074d8ad8"	"00074ee5e3"	0.0633
"00074d8ad8"	"00074ee6e0"	-0.064
"00074d8ad8"	"00074eea3a"	0.0361
"00074d8ad8"	"00074eff82"	-0.0778
"00074d8ad8"	"00074f0477"	-0.1814
"00074d8ad8"	"00074f08c3"	0.1135

"00074d8ad8"	"00074f1859"	0.0137
"00074d8ad8"	"00074f2268"	-0.0982
"00074d8ad8"	"00074f28be"	0.3723
"00074d8ad8"	"00074f294b"	0.0505
"00074d8ad8"	"00074f2ddd"	0.0782
"00074d8ad8"	"00074f2e75"	-0.1659
"00074d8ad8"	"00074f3088"	-0.1001
"00074d8ad8"	"00074f5a1c"	0.0369
"00074d8ad8"	"00074f75b7"	0.2266
"00074d8ad8"	"00074f8cd9"	0.1309
"00074d8ad8"	"00074f96dc"	-0.234
"00074d8ad8"	"00074fabaa"	0.0148
"00074d8ad8"	"00074facd9"	-0.0714
"00074d8ad8"	"00074fae3c"	-0.0551
"00074d8ad8"	"00074fb0a8"	0.052
"00074d8ad8"	"00074fb4e4"	-0.1425
"00074d8ad8"	"00074fb7c2"	0.0942
"00074d8ad8"	"00074fbd36"	0.1133
"00074d8ad8"	"00074fc27f"	0.3716
"00074d8ad8"	"00074fc31d"	0.0904
"00074d8ad8"	"00074fd569"	-0.1126
"00074d8ad8"	"00074fef15"	-0.2353
"00074d8ad8"	"00074ff562"	-0.2419
"00074d8ad8"	"00075007ca"	0.1944
"00074d8ad8"	"0007500b86"	0.2331
"00074d8ad8"	"0007500d05"	-0.1789
"00074d8ad8"	"0007500ee4"	-0.1247
"00074d8ad8"	"0007500eee"	-0.1552
"00074d8ad8"	"00075013dc"	-0.0831
"00074d8ad8"	"000757b515"	0.3437
"00074d8ad8"	"000757bc5a"	0.2167
"00074d8ad8"	"000757c320"	0.122
"00074d8ad8"	"000757c9aa"	0.3276
"00074d8ad8"	"000757ccbe"	-0.0785
"00074d8ad8"	"000757cfa9"	0.3166
"00074d8ad8"	"000757d390"	-0.162
"00074d8ad8"	"000757d393"	-0.026
"00074d8ad8"	"000757d598"	0.1902
"00074d8ad8"	"000757d5a2"	0.228
"00074d8ad8"	"000757d790"	-0.1042
"00074d8ad8"	"000757e30c"	0.2749
"00074d8ad8"	"000757e4b0"	0.2585
"00074d8ad8"	"000757e7a0"	0.1198
"00074d8ad8"	"000757e8b3"	0.4346
"00074d8ad8"	"000757f627"	0.0884
"00074d8ad8"	"000757f925"	0.1045
"00074d8ad8"	"000757fa08"	-0.0308
"00074d8ad8"	"000757fe52"	0.0997
"00074d8ad8"	"000758024a"	0.1621
"00074d8ad8"	"00075804bb"	0.324

"00074d8ad8"	"00075a0c04"	0.0431
"00074d8ad8"	"00075a3110"	0.1072
"00074d8ad8"	"00075a341a"	-0.1079
"00074d8ad8"	"00075a3dcf"	0.0032
"00074d8ad8"	"00075a3e22"	0.0955
"00074d8ad8"	"00075a48d8"	-0.0488
"00074d8ad8"	"00075a5cfb"	0.266
"00074d8ad8"	"00075a6151"	0.286
"00074d8ad8"	"00075a6708"	0.1678
"00074d8ad8"	"00075a7319"	-0.0967
"00074d8ad8"	"00075a7723"	0.1271
"00074d8ad8"	"00075a778b"	-0.1847
"00074d8ad8"	"00075a7b8e"	0.1721
"00074d8ad8"	"00075a7c79"	0.3028
"00074d8ad8"	"00075a81b6"	0.106
"00074d8ad8"	"00075a82ac"	0.0038
"00074d8ad8"	"00075a98e5"	-0.1838
"00074d8ad8"	"00075b0d29"	0.2668
"00074d8ad8"	"00075b102a"	0.1184
"00074d8ad8"	"00075b1074"	-0.1695
"00074d8ad8"	"00075b135d"	0.1127
"00074d8ad8"	"00075b138b"	0.309
"00074d8ad8"	"00075b13a0"	-0.4084
"00074d8ad8"	"00075b13bd"	-0.1267
"00074d8ad8"	"00075b16a9"	0.174
"00074d8ad8"	"00075b1a28"	-0.0454
"00074d8ad8"	"00075b1a97"	0.0578
"00074d8ad8"	"00075b1c7b"	-0.1212
"00074d8ad8"	"00075b1d24"	0.1788
"00074d8ad8"	"00075b202b"	0.1206
"00074d8ad8"	"00075b22cb"	0.005
"00074d8ad8"	"00075b22da"	-0.0404
"00074d8ad8"	"00075b2556"	-0.1764
"00074d8ad8"	"00075b25de"	0.1784
"00074d8ad8"	"00075b260c"	0.2454
"00074d8ad8"	"00075b26f1"	0.0513
"00074d8ad8"	"00075b2920"	0.2553
"00074d8ad8"	"00075b2a64"	0.2158
"00074d8ad8"	"00075b2a9d"	0.1455
"00074d8ad8"	"00075b2b37"	-0.2921
"00074d8ad8"	"00075b2cdd"	-0.1117
"00074d8ad8"	"00075b3038"	0.2222
"00074d8ad8"	"00075b30fe"	-0.1453
"00074d8ad8"	"00075b3362"	-0.3402
"00074d8ad8"	"00075b350a"	0.0894
"00074d8ad8"	"00075b350e"	0.0679
"00074d8ad8"	"00075b3651"	-0.0785
"00074d8ad8"	"00075b38ca"	-0.0729
"00074d8ad8"	"00075b39cc"	0.1723
"00074d8ad8"	"00075b3e1e"	0.0817

"00074d8ad8"	"00075b3e57"	-0.2438
"00074d8ad8"	"00075b4079"	0.2335
"00074d8ad8"	"00075b4150"	0.155
"00074d8ad8"	"00075b4194"	0.282
"00074d8ad8"	"00075b42d5"	0.1368
"00074d8ad8"	"00075b4424"	0.0378
"00074d8ad8"	"00075b4470"	0.2167
"00074d8ad8"	"00075b47ed"	-0.01
"00074d8ad8"	"00075b4850"	0.3219
"00074d8ad8"	"00075b4ca0"	0.302
"00074d8ad8"	"00075b4d7f"	0.2101
"00074d8ad8"	"00075b520f"	0.0506
"00074d8ad8"	"00075b525f"	0.0375
"00074d8ad8"	"00075b58f8"	-0.0341
"00074d8ad8"	"00075b5bcc"	0.2951
"00074d8ad8"	"00075b5bfa"	-0.187
"00074d8ad8"	"00075b6339"	0.5233
"00074d8ad8"	"00075b6658"	-0.1727
"00074d8ad8"	"00075b679a"	0.1967
"00074d8ad8"	"00075b6cb7"	0.2242
"00074d8ad8"	"00075b6df8"	0.04
"00074d8ad8"	"00075b6ff6"	0.4005
"00074d8ad8"	"00075b70ee"	0.2688
"00074d8ad8"	"00075b7157"	-0.0262
"00074d8ad8"	"00075b7225"	0.144
"00074d8ad8"	"00075b7c89"	0.2612
"00074d8ad8"	"00075b9048"	0.2631
"00074d8ad8"	"00075d0801"	0.1472
"00074d8ad8"	"00075d1820"	-0.0644
"00074d8ad8"	"00075d1f3d"	0.2667
"00074d8ad8"	"00075d2329"	-0.0202
"00074d8ad8"	"00075d2b9b"	0.1578
"00074d8ad8"	"00075d3941"	-0.1142
"00074d8ad8"	"00075d3e96"	0.0889
"00074d8ad8"	"00075d4864"	-0.0867
"00074d8ad8"	"00075d5961"	-0.0144
"00074d8ad8"	"00075d5a63"	0.1074
"00074d8ad8"	"00075d6150"	-0.0848
"00074d8ad8"	"00075d67d0"	-0.0869
"00074d8ad8"	"00075d67e2"	0.0399
"00074d8ad8"	"00075d73fc"	0.1029
"00074d8ad8"	"00075d7729"	-0.1227
"00074d8ad8"	"00075d778c"	-0.2638
"00074d8ad8"	"00075d7b9e"	-0.0662
"00074d8ad8"	"00075d7c8f"	0.3815
"00074d8ad8"	"00075d804d"	0.0822
"00074d8ad8"	"00075d819f"	0.0463
"00074d8ad8"	"00075d8601"	0.0521
"00074d8ad8"	"00075d8c6a"	0.2023
"00074d8ad8"	"00075dfedc"	0.5031

"00074d8ad8"	"00075e05f2"	0.2011
"00074d8ad8"	"00075e0837"	0.1996
"00074d8ad8"	"00075e092e"	-0.1599
"00074d8ad8"	"00075e0965"	0.0729
"00074d8ad8"	"00075e0bc8"	0.2755
"00074d8ad8"	"00075e0fbb"	-0.0866
"00074d8c26"	"00074d8ca5"	0.0201
"00074d8c26"	"00074d8e0a"	0.273
"00074d8c26"	"00074d9179"	-0.2631
"00074d8c26"	"00074d929f"	-0.2685
"00074d8c26"	"00074d93c4"	0.1575
"00074d8c26"	"00074d93d0"	-0.164
"00074d8c26"	"00074d945b"	0.0154
"00074d8c26"	"00074d966c"	-0.0027
"00074d8c26"	"00074d9953"	0.2274
"00074d8c26"	"00074d99b2"	-0.1493
"00074d8c26"	"00074d99f3"	-3e-04
"00074d8c26"	"00074d9afd"	-0.3293
"00074d8c26"	"00074d9c87"	0.1892
"00074d8c26"	"00074d9e9e"	-0.0157
"00074d8c26"	"00074d9f30"	-0.1241
"00074d8c26"	"00074da036"	0.1591
"00074d8c26"	"00074da082"	-0.2429
"00074d8c26"	"00074da136"	-0.1161
"00074d8c26"	"00074da3ed"	0.1921
"00074d8c26"	"00074da4ac"	0.3454
"00074d8c26"	"00074da4b8"	0.1198
"00074d8c26"	"00074da5e8"	0.1673
"00074d8c26"	"00074da6b4"	0.0357
"00074d8c26"	"00074daa3c"	0.2318
"00074d8c26"	"00074daaf6"	-0.1549
"00074d8c26"	"00074dad20"	-0.0529
"00074d8c26"	"00074db098"	0.0438
"00074d8c26"	"00074db231"	0.3136
"00074d8c26"	"00074db3a3"	0.4644
"00074d8c26"	"00074db5d6"	0.0415
"00074d8c26"	"00074db632"	0.1366
"00074d8c26"	"00074db688"	-0.339
"00074d8c26"	"00074db8a6"	0.2774
"00074d8c26"	"00074dba19"	0.1782
"00074d8c26"	"00074dbc2e"	0.2826
"00074d8c26"	"00074dbe51"	0.0903
"00074d8c26"	"00074dbe5f"	0.1651
"00074d8c26"	"00074dbf6d"	0.0611
"00074d8c26"	"00074dc4a5"	-0.362
"00074d8c26"	"00074dc50c"	0.3638
"00074d8c26"	"00074dcdfa"	0.0527
"00074d8c26"	"00074dcf5f"	-0.4489
"00074d8c26"	"00074dd007"	0.1503
"00074d8c26"	"00074dd163"	0.4313

"00074d8c26"	"00074dd3df"	0.1119
"00074d8c26"	"00074dd577"	0.025
"00074d8c26"	"00074dd62e"	0.0495
"00074d8c26"	"00074dd73c"	0.0065
"00074d8c26"	"00074dda10"	0.1277
"00074d8c26"	"00074ddab8"	0.3425
"00074d8c26"	"00074ddd3d"	−0.3054
"00074d8c26"	"00074ddf16"	0.1794
"00074d8c26"	"00074ddfc1"	0.4683
"00074d8c26"	"00074de21a"	−0.1574
"00074d8c26"	"00074de2a9"	0.3653
"00074d8c26"	"00074de544"	0.2046
"00074d8c26"	"00074de98a"	0.2655
"00074d8c26"	"00074dea7e"	0.064
"00074d8c26"	"00074debd9"	0.4478
"00074d8c26"	"00074deca3"	0.3413
"00074d8c26"	"00074def43"	0.0997
"00074d8c26"	"00074def99"	−0.297
"00074d8c26"	"00074ecdad"	0.0085
"00074d8c26"	"00074ecf28"	−0.2465
"00074d8c26"	"00074ed1e1"	−0.0911
"00074d8c26"	"00074ed83b"	0.1746
"00074d8c26"	"00074ee5e3"	0.1221
"00074d8c26"	"00074ee6e0"	−0.1225
"00074d8c26"	"00074eea3a"	0.3005
"00074d8c26"	"00074eff82"	−0.1785
"00074d8c26"	"00074f0477"	−0.0998
"00074d8c26"	"00074f08c3"	−0.1294
"00074d8c26"	"00074f1859"	0.4973
"00074d8c26"	"00074f2268"	−0.0301
"00074d8c26"	"00074f28be"	0.0647
"00074d8c26"	"00074f294b"	−0.0455
"00074d8c26"	"00074f2ddd"	−0.0256
"00074d8c26"	"00074f2e75"	−0.1106
"00074d8c26"	"00074f3088"	−0.1043
"00074d8c26"	"00074f5a1c"	0.2175
"00074d8c26"	"00074f75b7"	0.0518
"00074d8c26"	"00074f8cd9"	0.1151
"00074d8c26"	"00074f96dc"	−0.1949
"00074d8c26"	"00074fabaa"	0.4843
"00074d8c26"	"00074facd9"	0.1943
"00074d8c26"	"00074fae3c"	−0.1087
"00074d8c26"	"00074fb0a8"	0.0177
"00074d8c26"	"00074fb4e4"	−0.1411
"00074d8c26"	"00074fb7c2"	0.2865
"00074d8c26"	"00074fbd36"	0.0382
"00074d8c26"	"00074fc27f"	0.2602
"00074d8c26"	"00074fc31d"	−0.133
"00074d8c26"	"00074fd569"	−0.1432
"00074d8c26"	"00074fef15"	−0.1058

"00074d8c26"	"00074ff562"	-0.3266
"00074d8c26"	"00075007ca"	0.1724
"00074d8c26"	"0007500b86"	0.3568
"00074d8c26"	"0007500d05"	0.1126
"00074d8c26"	"0007500ee4"	-0.1038
"00074d8c26"	"0007500eee"	0.1553
"00074d8c26"	"00075013dc"	-0.1784
"00074d8c26"	"000757b515"	-0.0175
"00074d8c26"	"000757bc5a"	0.2184
"00074d8c26"	"000757c320"	0.1206
"00074d8c26"	"000757c9aa"	0.0352
"00074d8c26"	"000757ccbe"	-0.0264
"00074d8c26"	"000757cfa9"	0.0745
"00074d8c26"	"000757d390"	-0.234
"00074d8c26"	"000757d393"	0.51
"00074d8c26"	"000757d598"	-0.0973
"00074d8c26"	"000757d5a2"	-0.1917
"00074d8c26"	"000757d790"	0.2477
"00074d8c26"	"000757e30c"	-0.0419
"00074d8c26"	"000757e4b0"	0.3618
"00074d8c26"	"000757e7a0"	-0.2557
"00074d8c26"	"000757e8b3"	0.2809
"00074d8c26"	"000757f627"	0.1441
"00074d8c26"	"000757f925"	0.2926
"00074d8c26"	"000757fa08"	0.0087
"00074d8c26"	"000757fe52"	0.1928
"00074d8c26"	"000758024a"	0.046
"00074d8c26"	"00075804bb"	0.1943
"00074d8c26"	"00075a0c04"	0.0646
"00074d8c26"	"00075a3110"	0.1029
"00074d8c26"	"00075a341a"	-0.1211
"00074d8c26"	"00075a3dcf"	0.1691
"00074d8c26"	"00075a3e22"	0.1165
"00074d8c26"	"00075a48d8"	0.0409
"00074d8c26"	"00075a5cfb"	0.1652
"00074d8c26"	"00075a6151"	0.0356
"00074d8c26"	"00075a6708"	-0.2525
"00074d8c26"	"00075a7319"	-0.2156
"00074d8c26"	"00075a7723"	-0.0323
"00074d8c26"	"00075a778b"	0.0922
"00074d8c26"	"00075a7b8e"	-0.0644
"00074d8c26"	"00075a7c79"	0.081
"00074d8c26"	"00075a81b6"	-0.2627
"00074d8c26"	"00075a82ac"	0.1455
"00074d8c26"	"00075a98e5"	0.1098
"00074d8c26"	"00075b0d29"	0.0168
"00074d8c26"	"00075b102a"	0.412
"00074d8c26"	"00075b1074"	-0.0143
"00074d8c26"	"00075b135d"	-0.0438
"00074d8c26"	"00075b138b"	0.4525

"00074d8c26"	"00075b13a0"	-0.064
"00074d8c26"	"00075b13bd"	-0.248
"00074d8c26"	"00075b16a9"	-0.0358
"00074d8c26"	"00075b1a28"	-0.0568
"00074d8c26"	"00075b1a97"	-0.0691
"00074d8c26"	"00075b1c7b"	-0.1406
"00074d8c26"	"00075b1d24"	-0.2512
"00074d8c26"	"00075b202b"	0.2969
"00074d8c26"	"00075b22cb"	0.0344
"00074d8c26"	"00075b22da"	0.2276
"00074d8c26"	"00075b2556"	0.1608
"00074d8c26"	"00075b25de"	0.1539
"00074d8c26"	"00075b260c"	0.2377
"00074d8c26"	"00075b26f1"	-0.1556
"00074d8c26"	"00075b2920"	0.0544
"00074d8c26"	"00075b2a64"	0.006
"00074d8c26"	"00075b2a9d"	0.2857
"00074d8c26"	"00075b2b37"	-0.2518
"00074d8c26"	"00075b2cdd"	0.1513
"00074d8c26"	"00075b3038"	0.1453
"00074d8c26"	"00075b30fe"	0.0971
"00074d8c26"	"00075b3362"	-0.2758
"00074d8c26"	"00075b350a"	-0.0148
"00074d8c26"	"00075b350e"	0.3191
"00074d8c26"	"00075b3651"	-0.2852
"00074d8c26"	"00075b38ca"	0.1739
"00074d8c26"	"00075b39cc"	-0.0456
"00074d8c26"	"00075b3e1e"	0.4871
"00074d8c26"	"00075b3e57"	0.0341
"00074d8c26"	"00075b4079"	-0.1944
"00074d8c26"	"00075b4150"	0.0396
"00074d8c26"	"00075b4194"	0.2028
"00074d8c26"	"00075b42d5"	0.208
"00074d8c26"	"00075b4424"	-0.073
"00074d8c26"	"00075b4470"	0.3034
"00074d8c26"	"00075b47ed"	-0.0383
"00074d8c26"	"00075b4850"	0.1019
"00074d8c26"	"00075b4ca0"	-0.1901
"00074d8c26"	"00075b4d7f"	0.1112
"00074d8c26"	"00075b520f"	-0.1245
"00074d8c26"	"00075b525f"	-0.1663
"00074d8c26"	"00075b58f8"	0.1974
"00074d8c26"	"00075b5bcc"	-0.1467
"00074d8c26"	"00075b5bfa"	-0.1957
"00074d8c26"	"00075b6339"	0.1066
"00074d8c26"	"00075b6658"	-0.2672
"00074d8c26"	"00075b679a"	-0.0564
"00074d8c26"	"00075b6cb7"	0.0594
"00074d8c26"	"00075b6df8"	-0.1864
"00074d8c26"	"00075b6ff6"	-0.026

"00074d8c26"	"00075b70ee"	−0.1988
"00074d8c26"	"00075b7157"	−0.1078
"00074d8c26"	"00075b7225"	−0.0754
"00074d8c26"	"00075b7c89"	−0.0231
"00074d8c26"	"00075b9048"	0.1154
"00074d8c26"	"00075d0801"	0.138
"00074d8c26"	"00075d1820"	−0.2864
"00074d8c26"	"00075d1f3d"	0.1078
"00074d8c26"	"00075d2329"	0.1418
"00074d8c26"	"00075d2b9b"	0.4454
"00074d8c26"	"00075d3941"	−0.1293
"00074d8c26"	"00075d3e96"	−0.2553
"00074d8c26"	"00075d4864"	0.1866
"00074d8c26"	"00075d5961"	0.2483
"00074d8c26"	"00075d5a63"	0.2301
"00074d8c26"	"00075d6150"	−0.2553
"00074d8c26"	"00075d67d0"	−0.1016
"00074d8c26"	"00075d67e2"	0.1696
"00074d8c26"	"00075d73fc"	−0.042
"00074d8c26"	"00075d7729"	−0.399
"00074d8c26"	"00075d778c"	−0.2619
"00074d8c26"	"00075d7b9e"	0.0193
"00074d8c26"	"00075d7c8f"	0.1585
"00074d8c26"	"00075d804d"	0.3188
"00074d8c26"	"00075d819f"	0.3531
"00074d8c26"	"00075d8601"	−0.0415
"00074d8c26"	"00075d8c6a"	−0.0361
"00074d8c26"	"00075dfedc"	0.0056
"00074d8c26"	"00075e05f2"	0.3599
"00074d8c26"	"00075e0837"	0.2895
"00074d8c26"	"00075e092e"	0.0528
"00074d8c26"	"00075e0965"	−0.0103
"00074d8c26"	"00075e0bc8"	0.3442
"00074d8c26"	"00075e0fbb"	0.1657
"00074d8ca5"	"00074d8e0a"	0.146
"00074d8ca5"	"00074d9179"	−0.2546
"00074d8ca5"	"00074d929f"	−0.2979
"00074d8ca5"	"00074d93c4"	−0.1616
"00074d8ca5"	"00074d93d0"	−0.1801
"00074d8ca5"	"00074d945b"	0.0208
"00074d8ca5"	"00074d966c"	−0.1484
"00074d8ca5"	"00074d9953"	0.2859
"00074d8ca5"	"00074d99b2"	−0.2719
"00074d8ca5"	"00074d99f3"	−0.0727
"00074d8ca5"	"00074d9afd"	−0.277
"00074d8ca5"	"00074d9c87"	−0.133
"00074d8ca5"	"00074d9e9e"	−0.2753
"00074d8ca5"	"00074d9f30"	−0.2917
"00074d8ca5"	"00074da036"	0.1761
"00074d8ca5"	"00074da082"	−0.173

"00074d8ca5"	"00074da136"	-0.0491
"00074d8ca5"	"00074da3ed"	-0.1048
"00074d8ca5"	"00074da4ac"	0.1869
"00074d8ca5"	"00074da4b8"	-0.0549
"00074d8ca5"	"00074da5e8"	0.0687
"00074d8ca5"	"00074da6b4"	-0.0064
"00074d8ca5"	"00074daa3c"	-0.0843
"00074d8ca5"	"00074daaf6"	-0.4652
"00074d8ca5"	"00074dad20"	-0.3251
"00074d8ca5"	"00074db098"	-0.0842
"00074d8ca5"	"00074db231"	-0.0188
"00074d8ca5"	"00074db3a3"	0.132
"00074d8ca5"	"00074db5d6"	-0.3279
"00074d8ca5"	"00074db632"	0.1675
"00074d8ca5"	"00074db688"	-0.1407
"00074d8ca5"	"00074db8a6"	0.0885
"00074d8ca5"	"00074dba19"	0.0097
"00074d8ca5"	"00074dbc2e"	0.0091
"00074d8ca5"	"00074dbe51"	0.2151
"00074d8ca5"	"00074dbe5f"	-0.2598
"00074d8ca5"	"00074dbf6d"	-0.1645
"00074d8ca5"	"00074dc4a5"	-0.1938
"00074d8ca5"	"00074dc50c"	0.1256
"00074d8ca5"	"00074dcdfa"	-0.1453
"00074d8ca5"	"00074dcf5f"	-0.1833
"00074d8ca5"	"00074dd007"	-0.1436
"00074d8ca5"	"00074dd163"	-0.0432
"00074d8ca5"	"00074dd3df"	-0.2418
"00074d8ca5"	"00074dd577"	0.1805
"00074d8ca5"	"00074dd62e"	-0.2007
"00074d8ca5"	"00074dd73c"	0.0187
"00074d8ca5"	"00074dda10"	0.0365
"00074d8ca5"	"00074ddab8"	0.0709
"00074d8ca5"	"00074ddd3d"	-0.2131
"00074d8ca5"	"00074ddf16"	-0.028
"00074d8ca5"	"00074ddfc1"	0.0221
"00074d8ca5"	"00074de21a"	0.0196
"00074d8ca5"	"00074de2a9"	-0.0137
"00074d8ca5"	"00074de544"	0.2755
"00074d8ca5"	"00074de98a"	0.4156
"00074d8ca5"	"00074dea7e"	-0.1289
"00074d8ca5"	"00074debd9"	-0.2742
"00074d8ca5"	"00074deca3"	0.0121
"00074d8ca5"	"00074def43"	0.0715
"00074d8ca5"	"00074def99"	0.024
"00074d8ca5"	"00074ecdad"	-0.1599
"00074d8ca5"	"00074ecf28"	-0.4797
"00074d8ca5"	"00074ed1e1"	0.0208
"00074d8ca5"	"00074ed83b"	0.2431
"00074d8ca5"	"00074ee5e3"	-0.2868

"00074d8ca5"	"00074ee6e0"	-0.3837
"00074d8ca5"	"00074eea3a"	0.0947
"00074d8ca5"	"00074eff82"	-0.2115
"00074d8ca5"	"00074f0477"	-0.3123
"00074d8ca5"	"00074f08c3"	-0.1878
"00074d8ca5"	"00074f1859"	-0.1088
"00074d8ca5"	"00074f2268"	-0.0291
"00074d8ca5"	"00074f28be"	0.2312
"00074d8ca5"	"00074f294b"	-0.0652
"00074d8ca5"	"00074f2ddd"	-0.0617
"00074d8ca5"	"00074f2e75"	-0.0263
"00074d8ca5"	"00074f3088"	-0.4244
"00074d8ca5"	"00074f5a1c"	0.121
"00074d8ca5"	"00074f75b7"	-0.022
"00074d8ca5"	"00074f8cd9"	-0.0771
"00074d8ca5"	"00074f96dc"	-0.2842
"00074d8ca5"	"00074fabaa"	0.1723
"00074d8ca5"	"00074facd9"	-0.3464
"00074d8ca5"	"00074fae3c"	-0.3656
"00074d8ca5"	"00074fb0a8"	-0.1655
"00074d8ca5"	"00074fb4e4"	-0.2844
"00074d8ca5"	"00074fb7c2"	0.0817
"00074d8ca5"	"00074fbd36"	0.1463
"00074d8ca5"	"00074fc27f"	0.194
"00074d8ca5"	"00074fc31d"	-0.1096
"00074d8ca5"	"00074fd569"	-0.2803
"00074d8ca5"	"00074fef15"	-0.2053
"00074d8ca5"	"00074ff562"	-0.2412
"00074d8ca5"	"00075007ca"	-0.3028
"00074d8ca5"	"0007500b86"	0.0901
"00074d8ca5"	"0007500d05"	-0.1335
"00074d8ca5"	"0007500ee4"	-0.3813
"00074d8ca5"	"0007500eee"	-0.2782
"00074d8ca5"	"00075013dc"	-0.1203
"00074d8ca5"	"000757b515"	0.1203
"00074d8ca5"	"000757bc5a"	0.0718
"00074d8ca5"	"000757c320"	-0.0992
"00074d8ca5"	"000757c9aa"	-0.125
"00074d8ca5"	"000757ccbe"	0.1157
"00074d8ca5"	"000757cfa9"	-0.2598
"00074d8ca5"	"000757d390"	-0.2669
"00074d8ca5"	"000757d393"	0.0668
"00074d8ca5"	"000757d598"	-0.2031
"00074d8ca5"	"000757d5a2"	-0.3337
"00074d8ca5"	"000757d790"	0.2855
"00074d8ca5"	"000757e30c"	-0.0136
"00074d8ca5"	"000757e4b0"	-0.0045
"00074d8ca5"	"000757e7a0"	-0.1966
"00074d8ca5"	"000757e8b3"	0.0945
"00074d8ca5"	"000757f627"	0.1392

"00074d8ca5"	"000757f925"	-0.025
"00074d8ca5"	"000757fa08"	-0.166
"00074d8ca5"	"000757fe52"	0.091
"00074d8ca5"	"000758024a"	-0.1048
"00074d8ca5"	"00075804bb"	-0.1158
"00074d8ca5"	"00075a0c04"	-0.1737
"00074d8ca5"	"00075a3110"	0.0276
"00074d8ca5"	"00075a341a"	-0.0574
"00074d8ca5"	"00075a3dcf"	0.1431
"00074d8ca5"	"00075a3e22"	0.1592
"00074d8ca5"	"00075a48d8"	0.0449
"00074d8ca5"	"00075a5cfb"	-0.0136
"00074d8ca5"	"00075a6151"	0.5254
"00074d8ca5"	"00075a6708"	0.196
"00074d8ca5"	"00075a7319"	-0.3847
"00074d8ca5"	"00075a7723"	0.1371
"00074d8ca5"	"00075a778b"	0.0786
"00074d8ca5"	"00075a7b8e"	0.0451
"00074d8ca5"	"00075a7c79"	0.2779
"00074d8ca5"	"00075a81b6"	0.0484
"00074d8ca5"	"00075a82ac"	0.2435
"00074d8ca5"	"00075a98e5"	0.143
"00074d8ca5"	"00075b0d29"	0.0153
"00074d8ca5"	"00075b102a"	0.1252
"00074d8ca5"	"00075b1074"	-0.18
"00074d8ca5"	"00075b135d"	-0.002
"00074d8ca5"	"00075b138b"	0.2668
"00074d8ca5"	"00075b13a0"	0.1188
"00074d8ca5"	"00075b13bd"	-0.1141
"00074d8ca5"	"00075b16a9"	-0.1959
"00074d8ca5"	"00075b1a28"	-0.1214
"00074d8ca5"	"00075b1a97"	-0.087
"00074d8ca5"	"00075b1c7b"	-0.2725
"00074d8ca5"	"00075b1d24"	-0.2345
"00074d8ca5"	"00075b202b"	-0.0896
"00074d8ca5"	"00075b22cb"	0.0194
"00074d8ca5"	"00075b22da"	-0.0174
"00074d8ca5"	"00075b2556"	-0.0857
"00074d8ca5"	"00075b25de"	-0.2105
"00074d8ca5"	"00075b260c"	0.0626
"00074d8ca5"	"00075b26f1"	-0.1869
"00074d8ca5"	"00075b2920"	-0.0978
"00074d8ca5"	"00075b2a64"	-0.0267
"00074d8ca5"	"00075b2a9d"	-0.2721
"00074d8ca5"	"00075b2b37"	-0.6675
"00074d8ca5"	"00075b2cdd"	0.0195
"00074d8ca5"	"00075b3038"	0.149
"00074d8ca5"	"00075b30fe"	-0.0076
"00074d8ca5"	"00075b3362"	-0.4111
"00074d8ca5"	"00075b350a"	-0.2664

"00074d8ca5"	"00075b350e"	0.1734
"00074d8ca5"	"00075b3651"	−0.2595
"00074d8ca5"	"00075b38ca"	−0.1978
"00074d8ca5"	"00075b39cc"	−0.1256
"00074d8ca5"	"00075b3e1e"	−0.154
"00074d8ca5"	"00075b3e57"	−0.2651
"00074d8ca5"	"00075b4079"	−0.3531
"00074d8ca5"	"00075b4150"	−0.0176
"00074d8ca5"	"00075b4194"	0.0735
"00074d8ca5"	"00075b42d5"	0.2
"00074d8ca5"	"00075b4424"	−0.1896
"00074d8ca5"	"00075b4470"	0.0562
"00074d8ca5"	"00075b47ed"	−0.1862
"00074d8ca5"	"00075b4850"	0.1082
"00074d8ca5"	"00075b4ca0"	0.1507
"00074d8ca5"	"00075b4d7f"	0.1275
"00074d8ca5"	"00075b520f"	−0.1134
"00074d8ca5"	"00075b525f"	−0.2139
"00074d8ca5"	"00075b58f8"	−0.1876
"00074d8ca5"	"00075b5bcc"	−0.139
"00074d8ca5"	"00075b5bfa"	−0.1054
"00074d8ca5"	"00075b6339"	0.1764
"00074d8ca5"	"00075b6658"	−0.3439
"00074d8ca5"	"00075b679a"	−0.1444
"00074d8ca5"	"00075b6cb7"	0.0759
"00074d8ca5"	"00075b6df8"	0.0032
"00074d8ca5"	"00075b6ff6"	−0.0284
"00074d8ca5"	"00075b70ee"	−0.0145
"00074d8ca5"	"00075b7157"	−0.1832
"00074d8ca5"	"00075b7225"	0.0263
"00074d8ca5"	"00075b7c89"	0.1838
"00074d8ca5"	"00075b9048"	0.2892
"00074d8ca5"	"00075d0801"	−0.1425
"00074d8ca5"	"00075d1820"	−0.3331
"00074d8ca5"	"00075d1f3d"	0.1502
"00074d8ca5"	"00075d2329"	−0.1169
"00074d8ca5"	"00075d2b9b"	0.0322
"00074d8ca5"	"00075d3941"	−0.4306
"00074d8ca5"	"00075d3e96"	−0.4406
"00074d8ca5"	"00075d4864"	−0.1424
"00074d8ca5"	"00075d5961"	−0.0583
"00074d8ca5"	"00075d5a63"	−0.1596
"00074d8ca5"	"00075d6150"	−0.2523
"00074d8ca5"	"00075d67d0"	−0.2681
"00074d8ca5"	"00075d67e2"	0.3211
"00074d8ca5"	"00075d73fc"	−0.0441
"00074d8ca5"	"00075d7729"	−0.3121
"00074d8ca5"	"00075d778c"	−0.3153
"00074d8ca5"	"00075d7b9e"	−0.2904
"00074d8ca5"	"00075d7c8f"	0.0436

"00074d8ca5"	"00075d804d"	-0.0619
"00074d8ca5"	"00075d819f"	-0.1196
"00074d8ca5"	"00075d8601"	-0.2837
"00074d8ca5"	"00075d8c6a"	0.0896
"00074d8ca5"	"00075dfedc"	0.0311
"00074d8ca5"	"00075e05f2"	0.4297
"00074d8ca5"	"00075e0837"	-0.0964
"00074d8ca5"	"00075e092e"	-0.09
"00074d8ca5"	"00075e0965"	-0.2486
"00074d8ca5"	"00075e0bc8"	0.0464
"00074d8ca5"	"00075e0fbb"	-0.163
"00074d8e0a"	"00074d9179"	-0.1387
"00074d8e0a"	"00074d929f"	-0.1475
"00074d8e0a"	"00074d93c4"	-0.0285
"00074d8e0a"	"00074d93d0"	-0.0948
"00074d8e0a"	"00074d945b"	0.1339
"00074d8e0a"	"00074d966c"	0.1666
"00074d8e0a"	"00074d9953"	0.271
"00074d8e0a"	"00074d99b2"	-0.1365
"00074d8e0a"	"00074d99f3"	0.088
"00074d8e0a"	"00074d9afd"	-0.4724
"00074d8e0a"	"00074d9c87"	0.1408
"00074d8e0a"	"00074d9e9e"	-0.3059
"00074d8e0a"	"00074d9f30"	-0.1081
"00074d8e0a"	"00074da036"	0.2427
"00074d8e0a"	"00074da082"	-0.2422
"00074d8e0a"	"00074da136"	-0.15
"00074d8e0a"	"00074da3ed"	-0.1852
"00074d8e0a"	"00074da4ac"	0.2098
"00074d8e0a"	"00074da4b8"	0.2061
"00074d8e0a"	"00074da5e8"	0.3291
"00074d8e0a"	"00074da6b4"	0.0528
"00074d8e0a"	"00074daa3c"	0.4537
"00074d8e0a"	"00074daaf6"	-0.1684
"00074d8e0a"	"00074dad20"	-0.051
"00074d8e0a"	"00074db098"	0.2132
"00074d8e0a"	"00074db231"	0.2728
"00074d8e0a"	"00074db3a3"	0.3309
"00074d8e0a"	"00074db5d6"	-0.0784
"00074d8e0a"	"00074db632"	0.4081
"00074d8e0a"	"00074db688"	-0.002
"00074d8e0a"	"00074db8a6"	-0.0571
"00074d8e0a"	"00074dba19"	0.3297
"00074d8e0a"	"00074dbc2e"	0.1758
"00074d8e0a"	"00074dbe51"	0.1851
"00074d8e0a"	"00074dbe5f"	-0.0666
"00074d8e0a"	"00074dbf6d"	0.2913
"00074d8e0a"	"00074dc4a5"	-0.4126
"00074d8e0a"	"00074dc50c"	0.4331
"00074d8e0a"	"00074dcdfa"	0.1408

"00074d8e0a"	"00074dcf5f"	−0.0447
"00074d8e0a"	"00074dd007"	0.0589
"00074d8e0a"	"00074dd163"	−0.0605
"00074d8e0a"	"00074dd3df"	−0.3687
"00074d8e0a"	"00074dd577"	0.2164
"00074d8e0a"	"00074dd62e"	0.2371
"00074d8e0a"	"00074dd73c"	0.102
"00074d8e0a"	"00074dda10"	−0.1749
"00074d8e0a"	"00074ddab8"	0.2464
"00074d8e0a"	"00074ddd3d"	0.0257
"00074d8e0a"	"00074ddf16"	0.2604
"00074d8e0a"	"00074ddfc1"	0.1821
"00074d8e0a"	"00074de21a"	−0.0629
"00074d8e0a"	"00074de2a9"	0.1671
"00074d8e0a"	"00074de544"	−0.0234
"00074d8e0a"	"00074de98a"	0.0144
"00074d8e0a"	"00074dea7e"	−0.1814
"00074d8e0a"	"00074debd9"	0.0886
"00074d8e0a"	"00074deca3"	0.035
"00074d8e0a"	"00074def43"	0.0897
"00074d8e0a"	"00074def99"	−0.2436
"00074d8e0a"	"00074ecdad"	−0.2093
"00074d8e0a"	"00074ecf28"	−0.3705
"00074d8e0a"	"00074ed1e1"	0.1734
"00074d8e0a"	"00074ed83b"	0.3094
"00074d8e0a"	"00074ee5e3"	0.068
"00074d8e0a"	"00074ee6e0"	−0.0998
"00074d8e0a"	"00074eea3a"	0.0138
"00074d8e0a"	"00074eff82"	−0.1661
"00074d8e0a"	"00074f0477"	0.2518
"00074d8e0a"	"00074f08c3"	0.0944
"00074d8e0a"	"00074f1859"	−0.0443
"00074d8e0a"	"00074f2268"	−0.0956
"00074d8e0a"	"00074f28be"	0.4092
"00074d8e0a"	"00074f294b"	−0.186
"00074d8e0a"	"00074f2ddd"	−0.0332
"00074d8e0a"	"00074f2e75"	−0.1044
"00074d8e0a"	"00074f3088"	−0.0743
"00074d8e0a"	"00074f5a1c"	0.0591
"00074d8e0a"	"00074f75b7"	0.1527
"00074d8e0a"	"00074f8cd9"	0.1005
"00074d8e0a"	"00074f96dc"	−0.2815
"00074d8e0a"	"00074fabaa"	0.2396
"00074d8e0a"	"00074facd9"	−0.0139
"00074d8e0a"	"00074fae3c"	−0.2592
"00074d8e0a"	"00074fb0a8"	0.1559
"00074d8e0a"	"00074fb4e4"	−0.0558
"00074d8e0a"	"00074fb7c2"	0.134
"00074d8e0a"	"00074fbd36"	−0.0619
"00074d8e0a"	"00074fc27f"	0.4277

"00074d8e0a"	"00074fc31d"	-0.1842
"00074d8e0a"	"00074fd569"	-0.0289
"00074d8e0a"	"00074fef15"	-0.2904
"00074d8e0a"	"00074ff562"	0.0705
"00074d8e0a"	"00075007ca"	0.2843
"00074d8e0a"	"0007500b86"	0.1854
"00074d8e0a"	"0007500d05"	0.1404
"00074d8e0a"	"0007500ee4"	-0.1108
"00074d8e0a"	"0007500eee"	-0.0182
"00074d8e0a"	"00075013dc"	0.258
"00074d8e0a"	"000757b515"	0.3939
"00074d8e0a"	"000757bc5a"	0.062
"00074d8e0a"	"000757c320"	0.0086
"00074d8e0a"	"000757c9aa"	0.288
"00074d8e0a"	"000757ccbe"	-0.0327
"00074d8e0a"	"000757cfa9"	-0.1438
"00074d8e0a"	"000757d390"	0.0167
"00074d8e0a"	"000757d393"	0.1699
"00074d8e0a"	"000757d598"	0.3648
"00074d8e0a"	"000757d5a2"	0.3228
"00074d8e0a"	"000757d790"	0.2777
"00074d8e0a"	"000757e30c"	0.1828
"00074d8e0a"	"000757e4b0"	0.275
"00074d8e0a"	"000757e7a0"	-0.0616
"00074d8e0a"	"000757e8b3"	0.2854
"00074d8e0a"	"000757f627"	0.1631
"00074d8e0a"	"000757f925"	0.3423
"00074d8e0a"	"000757fa08"	-0.2079
"00074d8e0a"	"000757fe52"	0.1278
"00074d8e0a"	"000758024a"	0.0643
"00074d8e0a"	"00075804bb"	0.3693
"00074d8e0a"	"00075a0c04"	0.1843
"00074d8e0a"	"00075a3110"	0.3487
"00074d8e0a"	"00075a341a"	-0.0754
"00074d8e0a"	"00075a3dcf"	-0.119
"00074d8e0a"	"00075a3e22"	0.3064
"00074d8e0a"	"00075a48d8"	0.0499
"00074d8e0a"	"00075a5cfb"	0.3605
"00074d8e0a"	"00075a6151"	0.4137
"00074d8e0a"	"00075a6708"	0.1469
"00074d8e0a"	"00075a7319"	-0.0393
"00074d8e0a"	"00075a7723"	-0.0414
"00074d8e0a"	"00075a778b"	-0.0436
"00074d8e0a"	"00075a7b8e"	-0.0253
"00074d8e0a"	"00075a7c79"	0.4187
"00074d8e0a"	"00075a81b6"	-0.0259
"00074d8e0a"	"00075a82ac"	0.0787
"00074d8e0a"	"00075a98e5"	-0.1533
"00074d8e0a"	"00075b0d29"	-0.036
"00074d8e0a"	"00075b102a"	0.2536

"00074d8e0a"	"00075b1074"	-0.2702
"00074d8e0a"	"00075b135d"	0.0712
"00074d8e0a"	"00075b138b"	0.3228
"00074d8e0a"	"00075b13a0"	-0.1341
"00074d8e0a"	"00075b13bd"	-0.2538
"00074d8e0a"	"00075b16a9"	0.0069
"00074d8e0a"	"00075b1a28"	-0.2994
"00074d8e0a"	"00075b1a97"	-0.1944
"00074d8e0a"	"00075b1c7b"	-0.5454
"00074d8e0a"	"00075b1d24"	-0.2063
"00074d8e0a"	"00075b202b"	0.3737
"00074d8e0a"	"00075b22cb"	-0.2717
"00074d8e0a"	"00075b22da"	-0.0867
"00074d8e0a"	"00075b2556"	-0.1054
"00074d8e0a"	"00075b25de"	0.2246
"00074d8e0a"	"00075b260c"	0.544
"00074d8e0a"	"00075b26f1"	0.0189
"00074d8e0a"	"00075b2920"	0.3796
"00074d8e0a"	"00075b2a64"	0.5054
"00074d8e0a"	"00075b2a9d"	0.3338
"00074d8e0a"	"00075b2b37"	-0.3778
"00074d8e0a"	"00075b2cdd"	-0.1509
"00074d8e0a"	"00075b3038"	0.4181
"00074d8e0a"	"00075b30fe"	-0.1486
"00074d8e0a"	"00075b3362"	-0.3764
"00074d8e0a"	"00075b350a"	-0.065
"00074d8e0a"	"00075b350e"	0.0879
"00074d8e0a"	"00075b3651"	-0.0754
"00074d8e0a"	"00075b38ca"	-0.2118
"00074d8e0a"	"00075b39cc"	0.0811
"00074d8e0a"	"00075b3e1e"	0.1415
"00074d8e0a"	"00075b3e57"	-0.1952
"00074d8e0a"	"00075b4079"	-0.0414
"00074d8e0a"	"00075b4150"	0.2587
"00074d8e0a"	"00075b4194"	0.2781
"00074d8e0a"	"00075b42d5"	-0.0314
"00074d8e0a"	"00075b4424"	-0.0927
"00074d8e0a"	"00075b4470"	0.062
"00074d8e0a"	"00075b47ed"	-0.0458
"00074d8e0a"	"00075b4850"	0.3802
"00074d8e0a"	"00075b4ca0"	0.2418
"00074d8e0a"	"00075b4d7f"	0.145
"00074d8e0a"	"00075b520f"	-0.0472
"00074d8e0a"	"00075b525f"	-0.1351
"00074d8e0a"	"00075b58f8"	7e-04
"00074d8e0a"	"00075b5bcc"	0.0822
"00074d8e0a"	"00075b5bfa"	0.1425
"00074d8e0a"	"00075b6339"	0.1912
"00074d8e0a"	"00075b6658"	-0.3504
"00074d8e0a"	"00075b679a"	-0.0127

"00074d8e0a"	"00075b6cb7"	0.0683
"00074d8e0a"	"00075b6df8"	-0.0453
"00074d8e0a"	"00075b6ff6"	0.1909
"00074d8e0a"	"00075b70ee"	0.2079
"00074d8e0a"	"00075b7157"	-0.0567
"00074d8e0a"	"00075b7225"	-0.0388
"00074d8e0a"	"00075b7c89"	0.2178
"00074d8e0a"	"00075b9048"	0.5777
"00074d8e0a"	"00075d0801"	0.1845
"00074d8e0a"	"00075d1820"	-0.2469
"00074d8e0a"	"00075d1f3d"	0.3239
"00074d8e0a"	"00075d2329"	0.0043
"00074d8e0a"	"00075d2b9b"	0.4685
"00074d8e0a"	"00075d3941"	-0.0033
"00074d8e0a"	"00075d3e96"	-0.0072
"00074d8e0a"	"00075d4864"	-0.3078
"00074d8e0a"	"00075d5961"	-0.0679
"00074d8e0a"	"00075d5a63"	0.118
"00074d8e0a"	"00075d6150"	-0.0677
"00074d8e0a"	"00075d67d0"	-0.0102
"00074d8e0a"	"00075d67e2"	-0.0575
"00074d8e0a"	"00075d73fc"	-0.1616
"00074d8e0a"	"00075d7729"	-0.1302
"00074d8e0a"	"00075d778c"	-0.3582
"00074d8e0a"	"00075d7b9e"	-0.1647
"00074d8e0a"	"00075d7c8f"	0.1146
"00074d8e0a"	"00075d804d"	0.419
"00074d8e0a"	"00075d819f"	0.2033
"00074d8e0a"	"00075d8601"	-0.0441
"00074d8e0a"	"00075d8c6a"	0.1409
"00074d8e0a"	"00075dfedc"	0.253
"00074d8e0a"	"00075e05f2"	0.3013
"00074d8e0a"	"00075e0837"	0.3913
"00074d8e0a"	"00075e092e"	-0.2342
"00074d8e0a"	"00075e0965"	0.0143
"00074d8e0a"	"00075e0bc8"	0.3088
"00074d8e0a"	"00075e0fbb"	-0.291
"00074d9179"	"00074d929f"	0.2735
"00074d9179"	"00074d93c4"	0.0451
"00074d9179"	"00074d93d0"	0.3166
"00074d9179"	"00074d945b"	0.1608
"00074d9179"	"00074d966c"	-0.1834
"00074d9179"	"00074d9953"	-0.0408
"00074d9179"	"00074d99b2"	-0.161
"00074d9179"	"00074d99f3"	0.0982
"00074d9179"	"00074d9afd"	0.2576
"00074d9179"	"00074d9c87"	-0.0054
"00074d9179"	"00074d9e9e"	-0.2128
"00074d9179"	"00074d9f30"	-0.0926
"00074d9179"	"00074da036"	-0.0121

"00074d9179"	"00074da082"	0.4894
"00074d9179"	"00074da136"	0.0505
"00074d9179"	"00074da3ed"	−0.1692
"00074d9179"	"00074da4ac"	0.0519
"00074d9179"	"00074da4b8"	0.0302
"00074d9179"	"00074da5e8"	−0.1122
"00074d9179"	"00074da6b4"	−0.0663
"00074d9179"	"00074daa3c"	−0.393
"00074d9179"	"00074daaf6"	−0.2022
"00074d9179"	"00074dad20"	−0.09
"00074d9179"	"00074db098"	−0.242
"00074d9179"	"00074db231"	−0.1181
"00074d9179"	"00074db3a3"	−0.0238
"00074d9179"	"00074db5d6"	0.1055
"00074d9179"	"00074db632"	0.1116
"00074d9179"	"00074db688"	−0.0272
"00074d9179"	"00074db8a6"	−0.1796
"00074d9179"	"00074dba19"	−0.1468
"00074d9179"	"00074dbc2e"	−0.3047
"00074d9179"	"00074dbe51"	0.0517
"00074d9179"	"00074dbe5f"	−0.2185
"00074d9179"	"00074dbf6d"	−0.1736
"00074d9179"	"00074dc4a5"	0.1561
"00074d9179"	"00074dc50c"	−0.3376
"00074d9179"	"00074dcdfa"	−0.5788
"00074d9179"	"00074dcf5f"	0.1671
"00074d9179"	"00074dd007"	−0.3909
"00074d9179"	"00074dd163"	−0.3021
"00074d9179"	"00074dd3df"	−0.1218
"00074d9179"	"00074dd577"	−0.1871
"00074d9179"	"00074dd62e"	0.0343
"00074d9179"	"00074dd73c"	−0.0364
"00074d9179"	"00074dda10"	−0.255
"00074d9179"	"00074ddab8"	−0.2762
"00074d9179"	"00074ddd3d"	0.1241
"00074d9179"	"00074ddf16"	−0.2919
"00074d9179"	"00074ddfc1"	−0.2959
"00074d9179"	"00074de21a"	−0.4154
"00074d9179"	"00074de2a9"	−0.071
"00074d9179"	"00074de544"	−0.1704
"00074d9179"	"00074de98a"	−0.2258
"00074d9179"	"00074dea7e"	−0.1793
"00074d9179"	"00074debd9"	−0.517
"00074d9179"	"00074deca3"	−0.1937
"00074d9179"	"00074def43"	−0.0805
"00074d9179"	"00074def99"	−0.0992
"00074d9179"	"00074ecdad"	0.0296
"00074d9179"	"00074ecf28"	0.5644
"00074d9179"	"00074ed1e1"	−0.0214
"00074d9179"	"00074ed83b"	0.0182

"00074d9179"	"00074ee5e3"	-0.3473
"00074d9179"	"00074ee6e0"	-0.3143
"00074d9179"	"00074eea3a"	-0.0434
"00074d9179"	"00074eff82"	-0.2401
"00074d9179"	"00074f0477"	-0.1732
"00074d9179"	"00074f08c3"	-0.4007
"00074d9179"	"00074f1859"	-0.1709
"00074d9179"	"00074f2268"	-0.389
"00074d9179"	"00074f28be"	-0.3057
"00074d9179"	"00074f294b"	-0.208
"00074d9179"	"00074f2ddd"	-0.0252
"00074d9179"	"00074f2e75"	-0.0227
"00074d9179"	"00074f3088"	0.0996
"00074d9179"	"00074f5a1c"	-0.2199
"00074d9179"	"00074f75b7"	-0.1796
"00074d9179"	"00074f8cd9"	0.0607
"00074d9179"	"00074f96dc"	0.3428
"00074d9179"	"00074fabaa"	-0.3331
"00074d9179"	"00074facd9"	-0.2689
"00074d9179"	"00074fae3c"	0.0974
"00074d9179"	"00074fb0a8"	-0.2693
"00074d9179"	"00074fb4e4"	-0.201
"00074d9179"	"00074fb7c2"	0.1541
"00074d9179"	"00074fbd36"	-0.1841
"00074d9179"	"00074fc27f"	-0.1948
"00074d9179"	"00074fc31d"	0.0027
"00074d9179"	"00074fd569"	0.5959
"00074d9179"	"00074fef15"	0.011
"00074d9179"	"00074ff562"	-0.2398
"00074d9179"	"00075007ca"	-0.1576
"00074d9179"	"0007500b86"	-0.2654
"00074d9179"	"0007500d05"	0.1047
"00074d9179"	"0007500ee4"	0.0584
"00074d9179"	"0007500eee"	-0.2626
"00074d9179"	"00075013dc"	-0.3933
"00074d9179"	"000757b515"	-0.2292
"00074d9179"	"000757bc5a"	-0.1264
"00074d9179"	"000757c320"	-0.2571
"00074d9179"	"000757c9aa"	-0.3476
"00074d9179"	"000757ccbe"	-0.1034
"00074d9179"	"000757cfa9"	-0.2201
"00074d9179"	"000757d390"	-0.295
"00074d9179"	"000757d393"	-0.0689
"00074d9179"	"000757d598"	-0.0703
"00074d9179"	"000757d5a2"	-0.0649
"00074d9179"	"000757d790"	-0.1197
"00074d9179"	"000757e30c"	-0.2258
"00074d9179"	"000757e4b0"	-0.106
"00074d9179"	"000757e7a0"	0.2045
"00074d9179"	"000757e8b3"	0.0461

"00074d9179"	"000757f627"	-0.3541
"00074d9179"	"000757f925"	0.0633
"00074d9179"	"000757fa08"	-0.0928
"00074d9179"	"000757fe52"	0.0376
"00074d9179"	"000758024a"	-0.1205
"00074d9179"	"00075804bb"	-0.3238
"00074d9179"	"00075a0c04"	0.1904
"00074d9179"	"00075a3110"	-0.083
"00074d9179"	"00075a341a"	0.2584
"00074d9179"	"00075a3dcf"	-0.1227
"00074d9179"	"00075a3e22"	-0.0882
"00074d9179"	"00075a48d8"	0.0621
"00074d9179"	"00075a5cfb"	-0.1946
"00074d9179"	"00075a6151"	-0.3822
"00074d9179"	"00075a6708"	-0.3101
"00074d9179"	"00075a7319"	-0.3596
"00074d9179"	"00075a7723"	-0.202
"00074d9179"	"00075a778b"	0.0396
"00074d9179"	"00075a7b8e"	-0.371
"00074d9179"	"00075a7c79"	-0.1189
"00074d9179"	"00075a81b6"	-0.2847
"00074d9179"	"00075a82ac"	0.0968
"00074d9179"	"00075a98e5"	-0.0978
"00074d9179"	"00075b0d29"	0.0476
"00074d9179"	"00075b102a"	-0.0655
"00074d9179"	"00075b1074"	-0.0884
"00074d9179"	"00075b135d"	-0.0887
"00074d9179"	"00075b138b"	-0.1171
"00074d9179"	"00075b13a0"	-0.233
"00074d9179"	"00075b13bd"	0.2649
"00074d9179"	"00075b16a9"	0.2632
"00074d9179"	"00075b1a28"	0.3353
"00074d9179"	"00075b1a97"	0.1447
"00074d9179"	"00075b1c7b"	0.2319
"00074d9179"	"00075b1d24"	-0.1417
"00074d9179"	"00075b202b"	-0.0684
"00074d9179"	"00075b22cb"	0.3464
"00074d9179"	"00075b22da"	-0.1779
"00074d9179"	"00075b2556"	-0.1603
"00074d9179"	"00075b25de"	-0.1352
"00074d9179"	"00075b260c"	-0.2882
"00074d9179"	"00075b26f1"	-0.0684
"00074d9179"	"00075b2920"	-0.1206
"00074d9179"	"00075b2a64"	-0.3412
"00074d9179"	"00075b2a9d"	-0.2948
"00074d9179"	"00075b2b37"	0.089
"00074d9179"	"00075b2cdd"	0.1811
"00074d9179"	"00075b3038"	-0.0105
"00074d9179"	"00075b30fe"	-0.1017
"00074d9179"	"00075b3362"	-0.2462

"00074d9179"	"00075b350a"	-0.4262
"00074d9179"	"00075b350e"	-0.1008
"00074d9179"	"00075b3651"	-0.1181
"00074d9179"	"00075b38ca"	-0.3512
"00074d9179"	"00075b39cc"	-0.163
"00074d9179"	"00075b3e1e"	0.0112
"00074d9179"	"00075b3e57"	0.4084
"00074d9179"	"00075b4079"	-0.1467
"00074d9179"	"00075b4150"	0.0258
"00074d9179"	"00075b4194"	-0.4127
"00074d9179"	"00075b42d5"	-0.1425
"00074d9179"	"00075b4424"	0.1948
"00074d9179"	"00075b4470"	-0.1264
"00074d9179"	"00075b47ed"	-0.1418
"00074d9179"	"00075b4850"	-0.125
"00074d9179"	"00075b4ca0"	-0.2772
"00074d9179"	"00075b4d7f"	0.1552
"00074d9179"	"00075b520f"	0.0816
"00074d9179"	"00075b525f"	-0.1157
"00074d9179"	"00075b58f8"	-0.1527
"00074d9179"	"00075b5bcc"	-0.1473
"00074d9179"	"00075b5bfa"	-0.3064
"00074d9179"	"00075b6339"	-0.0697
"00074d9179"	"00075b6658"	-0.0289
"00074d9179"	"00075b679a"	0.12
"00074d9179"	"00075b6cb7"	-0.3484
"00074d9179"	"00075b6df8"	0.2157
"00074d9179"	"00075b6ff6"	-0.098
"00074d9179"	"00075b70ee"	0.0462
"00074d9179"	"00075b7157"	-0.1142
"00074d9179"	"00075b7225"	0.0489
"00074d9179"	"00075b7c89"	-0.0018
"00074d9179"	"00075b9048"	-0.0979
"00074d9179"	"00075d0801"	-0.1404
"00074d9179"	"00075d1820"	0.2477
"00074d9179"	"00075d1f3d"	-0.3582
"00074d9179"	"00075d2329"	-0.1942
"00074d9179"	"00075d2b9b"	-0.4881
"00074d9179"	"00075d3941"	0.1103
"00074d9179"	"00075d3e96"	0.2103
"00074d9179"	"00075d4864"	-0.2272
"00074d9179"	"00075d5961"	0.0079
"00074d9179"	"00075d5a63"	-0.1297
"00074d9179"	"00075d6150"	-0.1888
"00074d9179"	"00075d67d0"	-0.2624
"00074d9179"	"00075d67e2"	-0.0824
"00074d9179"	"00075d73fc"	0.2344
"00074d9179"	"00075d7729"	-0.0937
"00074d9179"	"00075d778c"	-0.2756
"00074d9179"	"00075d7b9e"	-0.2672

"00074d9179"	"00075d7c8f"	0.017
"00074d9179"	"00075d804d"	-0.2572
"00074d9179"	"00075d819f"	-0.4107
"00074d9179"	"00075d8601"	0.2842
"00074d9179"	"00075d8c6a"	0.1011
"00074d9179"	"00075dfedc"	0.1047
"00074d9179"	"00075e05f2"	-0.235
"00074d9179"	"00075e0837"	-0.1697
"00074d9179"	"00075e092e"	-0.2913
"00074d9179"	"00075e0965"	-0.3168
"00074d9179"	"00075e0bc8"	-0.1647
"00074d9179"	"00075e0fbb"	-0.3874
"00074d929f"	"00074d93c4"	-0.0687
"00074d929f"	"00074d93d0"	0.2396
"00074d929f"	"00074d945b"	0.1252
"00074d929f"	"00074d966c"	-0.2143
"00074d929f"	"00074d9953"	-0.0482
"00074d929f"	"00074d99b2"	-0.0128
"00074d929f"	"00074d99f3"	0.2424
"00074d929f"	"00074d9afd"	0.313
"00074d929f"	"00074d9c87"	-0.0621
"00074d929f"	"00074d9e9e"	-0.2982
"00074d929f"	"00074d9f30"	-0.2337
"00074d929f"	"00074da036"	-0.1477
"00074d929f"	"00074da082"	0.1521
"00074d929f"	"00074da136"	0.0821
"00074d929f"	"00074da3ed"	-0.2006
"00074d929f"	"00074da4ac"	0.1713
"00074d929f"	"00074da4b8"	-0.0856
"00074d929f"	"00074da5e8"	0.0244
"00074d929f"	"00074da6b4"	0.0357
"00074d929f"	"00074daa3c"	-0.4065
"00074d929f"	"00074daaf6"	0.0017
"00074d929f"	"00074dad20"	0.2138
"00074d929f"	"00074db098"	-0.2942
"00074d929f"	"00074db231"	-0.0392
"00074d929f"	"00074db3a3"	-0.1533
"00074d929f"	"00074db5d6"	0.0216
"00074d929f"	"00074db632"	0.0824
"00074d929f"	"00074db688"	0.0472
"00074d929f"	"00074db8a6"	-0.1153
"00074d929f"	"00074dba19"	-0.238
"00074d929f"	"00074dbc2e"	-0.0223
"00074d929f"	"00074dbe51"	-0.1715
"00074d929f"	"00074dbe5f"	-0.2055
"00074d929f"	"00074dbf6d"	-0.4192
"00074d929f"	"00074dc4a5"	0.3423
"00074d929f"	"00074dc50c"	-0.1499
"00074d929f"	"00074dcdfa"	0.0382
"00074d929f"	"00074dcf5f"	-0.1116

"00074d929f"	"00074dd007"	-0.4445
"00074d929f"	"00074dd163"	-0.1559
"00074d929f"	"00074dd3df"	-0.1992
"00074d929f"	"00074dd577"	-0.2982
"00074d929f"	"00074dd62e"	-0.0491
"00074d929f"	"00074dd73c"	-0.1113
"00074d929f"	"00074dda10"	0.0653
"00074d929f"	"00074ddab8"	-0.2331
"00074d929f"	"00074ddd3d"	-0.2125
"00074d929f"	"00074ddf16"	-0.0515
"00074d929f"	"00074ddfc1"	0.0273
"00074d929f"	"00074de21a"	6e-04
"00074d929f"	"00074de2a9"	-0.118
"00074d929f"	"00074de544"	-0.0321
"00074d929f"	"00074de98a"	-0.0583
"00074d929f"	"00074dea7e"	-0.1858
"00074d929f"	"00074debd9"	-0.5989
"00074d929f"	"00074deca3"	-0.1419
"00074d929f"	"00074def43"	-0.0535
"00074d929f"	"00074def99"	-0.0177
"00074d929f"	"00074ecdad"	-0.0237
"00074d929f"	"00074ecf28"	0.3884
"00074d929f"	"00074ed1e1"	-0.015
"00074d929f"	"00074ed83b"	0.1003
"00074d929f"	"00074ee5e3"	-0.2273
"00074d929f"	"00074ee6e0"	-0.1506
"00074d929f"	"00074eea3a"	-0.05
"00074d929f"	"00074eff82"	0.1608
"00074d929f"	"00074f0477"	-0.2701
"00074d929f"	"00074f08c3"	-0.1254
"00074d929f"	"00074f1859"	0.0165
"00074d929f"	"00074f2268"	-0.6159
"00074d929f"	"00074f28be"	0.022
"00074d929f"	"00074f294b"	-0.0141
"00074d929f"	"00074f2ddd"	-0.1967
"00074d929f"	"00074f2e75"	-0.1403
"00074d929f"	"00074f3088"	0.2571
"00074d929f"	"00074f5a1c"	0.09
"00074d929f"	"00074f75b7"	-0.1192
"00074d929f"	"00074f8cd9"	0.2514
"00074d929f"	"00074f96dc"	0.1837
"00074d929f"	"00074fabaa"	-0.1337
"00074d929f"	"00074facd9"	-0.2235
"00074d929f"	"00074fae3c"	0.5395
"00074d929f"	"00074fb0a8"	-0.0729
"00074d929f"	"00074fb4e4"	-0.0219
"00074d929f"	"00074fb7c2"	0.2315
"00074d929f"	"00074fbd36"	-0.3067
"00074d929f"	"00074fc27f"	-0.4654
"00074d929f"	"00074fc31d"	0.0638

"00074d929f"	"00074fd569"	0.2606
"00074d929f"	"00074fef15"	0.0217
"00074d929f"	"00074ff562"	0.2285
"00074d929f"	"00075007ca"	−0.1017
"00074d929f"	"0007500b86"	−0.0694
"00074d929f"	"0007500d05"	0.1704
"00074d929f"	"0007500ee4"	0.1949
"00074d929f"	"0007500eee"	−0.3397
"00074d929f"	"00075013dc"	−0.0672
"00074d929f"	"000757b515"	−0.3605
"00074d929f"	"000757bc5a"	−0.2485
"00074d929f"	"000757c320"	−0.0951
"00074d929f"	"000757c9aa"	−0.1465
"00074d929f"	"000757ccbe"	−0.0576
"00074d929f"	"000757cfa9"	−0.0969
"00074d929f"	"000757d390"	−0.2025
"00074d929f"	"000757d393"	−0.0945
"00074d929f"	"000757d598"	−0.5077
"00074d929f"	"000757d5a2"	−0.3227
"00074d929f"	"000757d790"	−0.0844
"00074d929f"	"000757e30c"	−0.3155
"00074d929f"	"000757e4b0"	−0.0745
"00074d929f"	"000757e7a0"	0.0872
"00074d929f"	"000757e8b3"	−0.1121
"00074d929f"	"000757f627"	0.038
"00074d929f"	"000757f925"	0.0493
"00074d929f"	"000757fa08"	0.0267
"00074d929f"	"000757fe52"	−0.1302
"00074d929f"	"000758024a"	−0.232
"00074d929f"	"00075804bb"	−0.3487
"00074d929f"	"00075a0c04"	0.2639
"00074d929f"	"00075a3110"	−0.1137
"00074d929f"	"00075a341a"	0.0268
"00074d929f"	"00075a3dcf"	0.0757
"00074d929f"	"00075a3e22"	−0.1442
"00074d929f"	"00075a48d8"	0.091
"00074d929f"	"00075a5cfb"	−0.296
"00074d929f"	"00075a6151"	−0.2466
"00074d929f"	"00075a6708"	−0.2712
"00074d929f"	"00075a7319"	−0.1305
"00074d929f"	"00075a7723"	0.1544
"00074d929f"	"00075a778b"	0.0731
"00074d929f"	"00075a7b8e"	0.0844
"00074d929f"	"00075a7c79"	−0.2729
"00074d929f"	"00075a81b6"	0.0294
"00074d929f"	"00075a82ac"	0.0867
"00074d929f"	"00075a98e5"	0.138
"00074d929f"	"00075b0d29"	0.1187
"00074d929f"	"00075b102a"	−0.3338
"00074d929f"	"00075b1074"	−0.218

"00074d929f"	"00075b135d"	−0.0278
"00074d929f"	"00075b138b"	−0.1031
"00074d929f"	"00075b13a0"	−0.449
"00074d929f"	"00075b13bd"	0.1782
"00074d929f"	"00075b16a9"	0.0863
"00074d929f"	"00075b1a28"	−0.0062
"00074d929f"	"00075b1a97"	0.1877
"00074d929f"	"00075b1c7b"	−0.1373
"00074d929f"	"00075b1d24"	−0.1433
"00074d929f"	"00075b202b"	−0.0581
"00074d929f"	"00075b22cb"	0.0756
"00074d929f"	"00075b22da"	−0.3589
"00074d929f"	"00075b2556"	−0.2946
"00074d929f"	"00075b25de"	−0.155
"00074d929f"	"00075b260c"	−0.2899
"00074d929f"	"00075b26f1"	0.3462
"00074d929f"	"00075b2920"	−0.1746
"00074d929f"	"00075b2a64"	−0.4501
"00074d929f"	"00075b2a9d"	−0.1746
"00074d929f"	"00075b2b37"	−0.084
"00074d929f"	"00075b2cdd"	0.2754
"00074d929f"	"00075b3038"	0.0058
"00074d929f"	"00075b30fe"	−0.3866
"00074d929f"	"00075b3362"	−0.0431
"00074d929f"	"00075b350a"	−0.1805
"00074d929f"	"00075b350e"	−0.0553
"00074d929f"	"00075b3651"	0.0669
"00074d929f"	"00075b38ca"	0.0519
"00074d929f"	"00075b39cc"	0.1582
"00074d929f"	"00075b3e1e"	0.1475
"00074d929f"	"00075b3e57"	0.1535
"00074d929f"	"00075b4079"	−0.1776
"00074d929f"	"00075b4150"	−0.1715
"00074d929f"	"00075b4194"	0.0718
"00074d929f"	"00075b42d5"	0.0276
"00074d929f"	"00075b4424"	0.1001
"00074d929f"	"00075b4470"	−0.2485
"00074d929f"	"00075b47ed"	0.3124
"00074d929f"	"00075b4850"	−0.2151
"00074d929f"	"00075b4ca0"	−0.1917
"00074d929f"	"00075b4d7f"	0.0601
"00074d929f"	"00075b520f"	0.1304
"00074d929f"	"00075b525f"	0.1202
"00074d929f"	"00075b58f8"	−0.4436
"00074d929f"	"00075b5bcc"	−0.0817
"00074d929f"	"00075b5bfa"	−0.2333
"00074d929f"	"00075b6339"	−0.0363
"00074d929f"	"00075b6658"	−0.0275
"00074d929f"	"00075b679a"	−0.0034
"00074d929f"	"00075b6cb7"	−0.5921

"00074d929f"	"00075b6df8"	0.1564
"00074d929f"	"00075b6ff6"	−0.3255
"00074d929f"	"00075b70ee"	−0.1092
"00074d929f"	"00075b7157"	−0.0338
"00074d929f"	"00075b7225"	−0.1532
"00074d929f"	"00075b7c89"	−0.0769
"00074d929f"	"00075b9048"	−0.1685
"00074d929f"	"00075d0801"	0.0204
"00074d929f"	"00075d1820"	0.2397
"00074d929f"	"00075d1f3d"	−0.1891
"00074d929f"	"00075d2329"	0.2539
"00074d929f"	"00075d2b9b"	−0.2773
"00074d929f"	"00075d3941"	0.321
"00074d929f"	"00075d3e96"	0.1353
"00074d929f"	"00075d4864"	−0.0486
"00074d929f"	"00075d5961"	0.1449
"00074d929f"	"00075d5a63"	0.1398
"00074d929f"	"00075d6150"	0.134
"00074d929f"	"00075d67d0"	−0.2513
"00074d929f"	"00075d67e2"	−0.1737
"00074d929f"	"00075d73fc"	0.0241
"00074d929f"	"00075d7729"	0.0327
"00074d929f"	"00075d778c"	−0.025
"00074d929f"	"00075d7b9e"	0.147
"00074d929f"	"00075d7c8f"	−0.1084
"00074d929f"	"00075d804d"	−0.2536
"00074d929f"	"00075d819f"	−0.3528
"00074d929f"	"00075d8601"	0.4476
"00074d929f"	"00075d8c6a"	0.2379
"00074d929f"	"00075dfedc"	0.1469
"00074d929f"	"00075e05f2"	−0.4045
"00074d929f"	"00075e0837"	−0.0733
"00074d929f"	"00075e092e"	−0.2906
"00074d929f"	"00075e0965"	−0.2746
"00074d929f"	"00075e0bc8"	−0.2473
"00074d929f"	"00075e0fbb"	−0.3611
"00074d93c4"	"00074d93d0"	−0.1789
"00074d93c4"	"00074d945b"	−0.1664
"00074d93c4"	"00074d966c"	−0.1629
"00074d93c4"	"00074d9953"	−0.0989
"00074d93c4"	"00074d99b2"	−0.3086
"00074d93c4"	"00074d99f3"	0.1133
"00074d93c4"	"00074d9afd"	−0.0768
"00074d93c4"	"00074d9c87"	0.0553
"00074d93c4"	"00074d9e9e"	0.5809
"00074d93c4"	"00074d9f30"	0.0504
"00074d93c4"	"00074da036"	0.138
"00074d93c4"	"00074da082"	−0.0195
"00074d93c4"	"00074da136"	−0.1207
"00074d93c4"	"00074da3ed"	0.1323

"00074d93c4"	"00074da4ac"	0.4486
"00074d93c4"	"00074da4b8"	-0.0317
"00074d93c4"	"00074da5e8"	-0.118
"00074d93c4"	"00074da6b4"	-0.0331
"00074d93c4"	"00074daa3c"	-0.1019
"00074d93c4"	"00074daaf6"	0.0033
"00074d93c4"	"00074dad20"	0.1318
"00074d93c4"	"00074db098"	-0.0526
"00074d93c4"	"00074db231"	0.0461
"00074d93c4"	"00074db3a3"	0.082
"00074d93c4"	"00074db5d6"	-0.0635
"00074d93c4"	"00074db632"	-0.037
"00074d93c4"	"00074db688"	-0.4677
"00074d93c4"	"00074db8a6"	0.0648
"00074d93c4"	"00074dba19"	-0.1447
"00074d93c4"	"00074dbc2e"	0.1067
"00074d93c4"	"00074dbe51"	0.3025
"00074d93c4"	"00074dbe5f"	0.329
"00074d93c4"	"00074dbf6d"	-0.0663
"00074d93c4"	"00074dc4a5"	-0.2915
"00074d93c4"	"00074dc50c"	-0.0969
"00074d93c4"	"00074dcdfa"	0.0411
"00074d93c4"	"00074dcf5f"	-0.2758
"00074d93c4"	"00074dd007"	-0.2182
"00074d93c4"	"00074dd163"	0.0832
"00074d93c4"	"00074dd3df"	-0.1033
"00074d93c4"	"00074dd577"	0.0232
"00074d93c4"	"00074dd62e"	-0.0638
"00074d93c4"	"00074dd73c"	0.2115
"00074d93c4"	"00074dda10"	0.0862
"00074d93c4"	"00074ddab8"	0.0962
"00074d93c4"	"00074ddd3d"	-0.1706
"00074d93c4"	"00074ddf16"	0.3835
"00074d93c4"	"00074ddfc1"	0.3263
"00074d93c4"	"00074de21a"	-0.4224
"00074d93c4"	"00074de2a9"	-0.0082
"00074d93c4"	"00074de544"	0.0574
"00074d93c4"	"00074de98a"	-0.0581
"00074d93c4"	"00074dea7e"	0.1824
"00074d93c4"	"00074debd9"	-1e-04
"00074d93c4"	"00074deca3"	0.0295
"00074d93c4"	"00074def43"	0.2862
"00074d93c4"	"00074def99"	-0.294
"00074d93c4"	"00074ecdad"	-0.1018
"00074d93c4"	"00074ecf28"	-0.0417
"00074d93c4"	"00074ed1e1"	0.2154
"00074d93c4"	"00074ed83b"	-0.0226
"00074d93c4"	"00074ee5e3"	0.2739
"00074d93c4"	"00074ee6e0"	0.1022
"00074d93c4"	"00074eea3a"	0.0346

"00074d93c4"	"00074eff82"	0.0368
"00074d93c4"	"00074f0477"	0.0721
"00074d93c4"	"00074f08c3"	−0.3084
"00074d93c4"	"00074f1859"	0.1893
"00074d93c4"	"00074f2268"	−0.1912
"00074d93c4"	"00074f28be"	−0.0503
"00074d93c4"	"00074f294b"	−0.142
"00074d93c4"	"00074f2ddd"	−0.0617
"00074d93c4"	"00074f2e75"	−0.0993
"00074d93c4"	"00074f3088"	0.027
"00074d93c4"	"00074f5a1c"	−0.1958
"00074d93c4"	"00074f75b7"	0.148
"00074d93c4"	"00074f8cd9"	0.5248
"00074d93c4"	"00074f96dc"	−0.1885
"00074d93c4"	"00074fabaa"	−0.0934
"00074d93c4"	"00074facd9"	0.3127
"00074d93c4"	"00074fae3c"	−0.1385
"00074d93c4"	"00074fb0a8"	−0.1463
"00074d93c4"	"00074fb4e4"	−0.2212
"00074d93c4"	"00074fb7c2"	0.1637
"00074d93c4"	"00074fbd36"	−0.4683
"00074d93c4"	"00074fc27f"	0.0298
"00074d93c4"	"00074fc31d"	−0.2219
"00074d93c4"	"00074fd569"	0.1223
"00074d93c4"	"00074fef15"	−0.061
"00074d93c4"	"00074ff562"	6e−04
"00074d93c4"	"00075007ca"	−0.162
"00074d93c4"	"0007500b86"	0.3289
"00074d93c4"	"0007500d05"	0.618
"00074d93c4"	"0007500ee4"	0.1543
"00074d93c4"	"0007500eee"	0.2926
"00074d93c4"	"00075013dc"	−0.3615
"00074d93c4"	"000757b515"	0.0367
"00074d93c4"	"000757bc5a"	0.1343
"00074d93c4"	"000757c320"	−0.0831
"00074d93c4"	"000757c9aa"	−0.0425
"00074d93c4"	"000757ccbe"	0.1056
"00074d93c4"	"000757cfa9"	−0.2193
"00074d93c4"	"000757d390"	−0.279
"00074d93c4"	"000757d393"	0.3525
"00074d93c4"	"000757d598"	−0.1683
"00074d93c4"	"000757d5a2"	−0.235
"00074d93c4"	"000757d790"	0.062
"00074d93c4"	"000757e30c"	−0.1544
"00074d93c4"	"000757e4b0"	0.0068
"00074d93c4"	"000757e7a0"	0.0084
"00074d93c4"	"000757e8b3"	0.2575
"00074d93c4"	"000757f627"	0.0387
"00074d93c4"	"000757f925"	0.2913
"00074d93c4"	"000757fa08"	0.1059

"00074d93c4"	"000757fe52"	0.2718
"00074d93c4"	"000758024a"	-0.138
"00074d93c4"	"00075804bb"	-0.2453
"00074d93c4"	"00075a0c04"	0.3445
"00074d93c4"	"00075a3110"	0.1152
"00074d93c4"	"00075a341a"	0.2472
"00074d93c4"	"00075a3dcf"	0.1243
"00074d93c4"	"00075a3e22"	0.2163
"00074d93c4"	"00075a48d8"	0.4897
"00074d93c4"	"00075a5cfb"	-0.1873
"00074d93c4"	"00075a6151"	-0.0821
"00074d93c4"	"00075a6708"	-0.155
"00074d93c4"	"00075a7319"	-0.1969
"00074d93c4"	"00075a7723"	0.1417
"00074d93c4"	"00075a778b"	0.0357
"00074d93c4"	"00075a7b8e"	0.1455
"00074d93c4"	"00075a7c79"	0.1163
"00074d93c4"	"00075a81b6"	0.0178
"00074d93c4"	"00075a82ac"	0.2358
"00074d93c4"	"00075a98e5"	0.0873
"00074d93c4"	"00075b0d29"	-0.06
"00074d93c4"	"00075b102a"	0.1013
"00074d93c4"	"00075b1074"	-0.1772
"00074d93c4"	"00075b135d"	-0.3659
"00074d93c4"	"00075b138b"	0.2802
"00074d93c4"	"00075b13a0"	0.1363
"00074d93c4"	"00075b13bd"	-0.0487
"00074d93c4"	"00075b16a9"	0.2815
"00074d93c4"	"00075b1a28"	-0.1166
"00074d93c4"	"00075b1a97"	0.0551
"00074d93c4"	"00075b1c7b"	0.032
"00074d93c4"	"00075b1d24"	0.1355
"00074d93c4"	"00075b202b"	-0.0219
"00074d93c4"	"00075b22cb"	0.2204
"00074d93c4"	"00075b22da"	0.0348
"00074d93c4"	"00075b2556"	0.0722
"00074d93c4"	"00075b25de"	0.2813
"00074d93c4"	"00075b260c"	-0.1037
"00074d93c4"	"00075b26f1"	-0.168
"00074d93c4"	"00075b2920"	-0.0549
"00074d93c4"	"00075b2a64"	-0.2011
"00074d93c4"	"00075b2a9d"	-0.0813
"00074d93c4"	"00075b2b37"	-0.1125
"00074d93c4"	"00075b2cdd"	0.1668
"00074d93c4"	"00075b3038"	0.2567
"00074d93c4"	"00075b30fe"	-0.0196
"00074d93c4"	"00075b3362"	-0.155
"00074d93c4"	"00075b350a"	0.068
"00074d93c4"	"00075b350e"	-0.2667
"00074d93c4"	"00075b3651"	-0.1614

"00074d93c4"	"00075b38ca"	-0.1731
"00074d93c4"	"00075b39cc"	0.2264
"00074d93c4"	"00075b3e1e"	0.2662
"00074d93c4"	"00075b3e57"	0.227
"00074d93c4"	"00075b4079"	0.0095
"00074d93c4"	"00075b4150"	-0.2144
"00074d93c4"	"00075b4194"	0.1289
"00074d93c4"	"00075b42d5"	0.0877
"00074d93c4"	"00075b4424"	-0.2057
"00074d93c4"	"00075b4470"	0.1187
"00074d93c4"	"00075b47ed"	0.2646
"00074d93c4"	"00075b4850"	0.0723
"00074d93c4"	"00075b4ca0"	-0.4791
"00074d93c4"	"00075b4d7f"	0.4013
"00074d93c4"	"00075b520f"	-0.0585
"00074d93c4"	"00075b525f"	-0.1554
"00074d93c4"	"00075b58f8"	-0.1798
"00074d93c4"	"00075b5bcc"	-0.0012
"00074d93c4"	"00075b5bfa"	0.0169
"00074d93c4"	"00075b6339"	0.1993
"00074d93c4"	"00075b6658"	-0.0692
"00074d93c4"	"00075b679a"	0.0975
"00074d93c4"	"00075b6cb7"	-0.0817
"00074d93c4"	"00075b6df8"	0.3757
"00074d93c4"	"00075b6ff6"	0.1267
"00074d93c4"	"00075b70ee"	-0.1016
"00074d93c4"	"00075b7157"	0.1769
"00074d93c4"	"00075b7225"	-0.0301
"00074d93c4"	"00075b7c89"	-0.0445
"00074d93c4"	"00075b9048"	0.0754
"00074d93c4"	"00075d0801"	0.2674
"00074d93c4"	"00075d1820"	-0.1211
"00074d93c4"	"00075d1f3d"	-0.1497
"00074d93c4"	"00075d2329"	0.0937
"00074d93c4"	"00075d2b9b"	-0.2298
"00074d93c4"	"00075d3941"	-0.178
"00074d93c4"	"00075d3e96"	-0.1507
"00074d93c4"	"00075d4864"	-0.1319
"00074d93c4"	"00075d5961"	0.1079
"00074d93c4"	"00075d5a63"	0.2471
"00074d93c4"	"00075d6150"	-0.156
"00074d93c4"	"00075d67d0"	-0.0803
"00074d93c4"	"00075d67e2"	0.1634
"00074d93c4"	"00075d73fc"	0.1466
"00074d93c4"	"00075d7729"	-0.3439
"00074d93c4"	"00075d778c"	0.0852
"00074d93c4"	"00075d7b9e"	0.2396
"00074d93c4"	"00075d7c8f"	0.1893
"00074d93c4"	"00075d804d"	0.2542
"00074d93c4"	"00075d819f"	-0.0502

"00074d93c4"	"00075d8601"	0.2009
"00074d93c4"	"00075d8c6a"	0.338
"00074d93c4"	"00075dfedc"	0.2352
"00074d93c4"	"00075e05f2"	0.1086
"00074d93c4"	"00075e0837"	0.0102
"00074d93c4"	"00075e092e"	0.1035
"00074d93c4"	"00075e0965"	−0.2486
"00074d93c4"	"00075e0bc8"	0.1809
"00074d93c4"	"00075e0fbb"	−0.0238
"00074d93d0"	"00074d945b"	0.2001
"00074d93d0"	"00074d966c"	−0.1033
"00074d93d0"	"00074d9953"	0.07
"00074d93d0"	"00074d99b2"	0.0301
"00074d93d0"	"00074d99f3"	0.1386
"00074d93d0"	"00074d9afd"	0.2599
"00074d93d0"	"00074d9c87"	0.1469
"00074d93d0"	"00074d9e9e"	−0.401
"00074d93d0"	"00074d9f30"	−0.0262
"00074d93d0"	"00074da036"	0.2251
"00074d93d0"	"00074da082"	0.5115
"00074d93d0"	"00074da136"	0.0316
"00074d93d0"	"00074da3ed"	−0.2591
"00074d93d0"	"00074da4ac"	−0.0508
"00074d93d0"	"00074da4b8"	−0.0492
"00074d93d0"	"00074da5e8"	0.2855
"00074d93d0"	"00074da6b4"	0.004
"00074d93d0"	"00074daa3c"	−0.3292
"00074d93d0"	"00074daaf6"	−0.0315
"00074d93d0"	"00074dad20"	−0.0428
"00074d93d0"	"00074db098"	−0.0391
"00074d93d0"	"00074db231"	−0.1022
"00074d93d0"	"00074db3a3"	−0.091
"00074d93d0"	"00074db5d6"	0.1576
"00074d93d0"	"00074db632"	−0.0044
"00074d93d0"	"00074db688"	0.0233
"00074d93d0"	"00074db8a6"	−0.1772
"00074d93d0"	"00074dba19"	−0.0656
"00074d93d0"	"00074dbc2e"	−0.1242
"00074d93d0"	"00074dbe51"	0.0343
"00074d93d0"	"00074dbe5f"	−0.3452
"00074d93d0"	"00074dbf6d"	−0.2458
"00074d93d0"	"00074dc4a5"	0.2112
"00074d93d0"	"00074dc50c"	−0.0649
"00074d93d0"	"00074dcdfa"	−0.1164
"00074d93d0"	"00074dcf5f"	−0.0108
"00074d93d0"	"00074dd007"	−0.1929
"00074d93d0"	"00074dd163"	−0.1281
"00074d93d0"	"00074dd3df"	−0.2494
"00074d93d0"	"00074dd577"	−0.0809
"00074d93d0"	"00074dd62e"	−0.0506

"00074d93d0"	"00074dd73c"	0.0887
"00074d93d0"	"00074dda10"	−0.0236
"00074d93d0"	"00074ddab8"	0.0292
"00074d93d0"	"00074ddd3d"	0.0829
"00074d93d0"	"00074ddf16"	−0.2347
"00074d93d0"	"00074ddfc1"	−0.1942
"00074d93d0"	"00074de21a"	−0.1295
"00074d93d0"	"00074de2a9"	−0.1187
"00074d93d0"	"00074de544"	0.0892
"00074d93d0"	"00074de98a"	−0.0413
"00074d93d0"	"00074dea7e"	−0.0087
"00074d93d0"	"00074debd9"	−0.4026
"00074d93d0"	"00074deca3"	−0.1878
"00074d93d0"	"00074def43"	−0.0408
"00074d93d0"	"00074def99"	0.1592
"00074d93d0"	"00074ecdad"	−0.2896
"00074d93d0"	"00074ecf28"	0.0692
"00074d93d0"	"00074ed1e1"	−0.2139
"00074d93d0"	"00074ed83b"	−0.0555
"00074d93d0"	"00074ee5e3"	−0.0839
"00074d93d0"	"00074ee6e0"	−0.1651
"00074d93d0"	"00074eea3a"	0.0298
"00074d93d0"	"00074eff82"	−0.0789
"00074d93d0"	"00074f0477"	−0.2287
"00074d93d0"	"00074f08c3"	−0.1615
"00074d93d0"	"00074f1859"	−0.0893
"00074d93d0"	"00074f2268"	−0.2498
"00074d93d0"	"00074f28be"	−0.1031
"00074d93d0"	"00074f294b"	−0.1138
"00074d93d0"	"00074f2ddd"	0.1392
"00074d93d0"	"00074f2e75"	−0.0583
"00074d93d0"	"00074f3088"	0.2123
"00074d93d0"	"00074f5a1c"	−0.1415
"00074d93d0"	"00074f75b7"	−0.0174
"00074d93d0"	"00074f8cd9"	0.1094
"00074d93d0"	"00074f96dc"	0.3325
"00074d93d0"	"00074fabaa"	−0.1779
"00074d93d0"	"00074facd9"	−0.1678
"00074d93d0"	"00074fae3c"	0.0573
"00074d93d0"	"00074fb0a8"	−0.3348
"00074d93d0"	"00074fb4e4"	−0.2658
"00074d93d0"	"00074fb7c2"	0.0722
"00074d93d0"	"00074fbd36"	0.1442
"00074d93d0"	"00074fc27f"	−0.3106
"00074d93d0"	"00074fc31d"	−0.0808
"00074d93d0"	"00074fd569"	0.123
"00074d93d0"	"00074fef15"	0.1432
"00074d93d0"	"00074ff562"	−0.2869
"00074d93d0"	"00075007ca"	−0.3218
"00074d93d0"	"0007500b86"	−0.0303

"00074d93d0"	"0007500d05"	−0.0329
"00074d93d0"	"0007500ee4"	−0.1038
"00074d93d0"	"0007500eee"	−0.3481
"00074d93d0"	"00075013dc"	−0.2553
"00074d93d0"	"000757b515"	−0.19
"00074d93d0"	"000757bc5a"	0.1012
"00074d93d0"	"000757c320"	−0.2033
"00074d93d0"	"000757c9aa"	−0.2292
"00074d93d0"	"000757ccbe"	−0.0047
"00074d93d0"	"000757cfa9"	0.1102
"00074d93d0"	"000757d390"	−0.331
"00074d93d0"	"000757d393"	−0.0866
"00074d93d0"	"000757d598"	−0.2434
"00074d93d0"	"000757d5a2"	−0.18
"00074d93d0"	"000757d790"	−0.1403
"00074d93d0"	"000757e30c"	−0.2397
"00074d93d0"	"000757e4b0"	−0.1277
"00074d93d0"	"000757e7a0"	−0.1123
"00074d93d0"	"000757e8b3"	0.1777
"00074d93d0"	"000757f627"	−0.1093
"00074d93d0"	"000757f925"	0.1073
"00074d93d0"	"000757fa08"	0.0715
"00074d93d0"	"000757fe52"	0.1376
"00074d93d0"	"000758024a"	−0.0936
"00074d93d0"	"00075804bb"	−0.2804
"00074d93d0"	"00075a0c04"	0.1368
"00074d93d0"	"00075a3110"	0.1379
"00074d93d0"	"00075a341a"	0.0768
"00074d93d0"	"00075a3dcf"	−0.0062
"00074d93d0"	"00075a3e22"	−0.0887
"00074d93d0"	"00075a48d8"	0.0027
"00074d93d0"	"00075a5cfb"	−0.2035
"00074d93d0"	"00075a6151"	−0.3063
"00074d93d0"	"00075a6708"	0.1621
"00074d93d0"	"00075a7319"	−0.3788
"00074d93d0"	"00075a7723"	−0.172
"00074d93d0"	"00075a778b"	0.0422
"00074d93d0"	"00075a7b8e"	−0.1596
"00074d93d0"	"00075a7c79"	0.0101
"00074d93d0"	"00075a81b6"	−0.0897
"00074d93d0"	"00075a82ac"	−0.033
"00074d93d0"	"00075a98e5"	0.0198
"00074d93d0"	"00075b0d29"	0.3698
"00074d93d0"	"00075b102a"	−0.0467
"00074d93d0"	"00075b1074"	−0.1161
"00074d93d0"	"00075b135d"	−0.0058
"00074d93d0"	"00075b138b"	−0.0251
"00074d93d0"	"00075b13a0"	−0.1911
"00074d93d0"	"00075b13bd"	0.2823
"00074d93d0"	"00075b16a9"	0.0535

"00074d93d0"	"00075b1a28"	0.1353
"00074d93d0"	"00075b1a97"	0.1373
"00074d93d0"	"00075b1c7b"	−0.2249
"00074d93d0"	"00075b1d24"	0.1137
"00074d93d0"	"00075b202b"	0.0077
"00074d93d0"	"00075b22cb"	0.1057
"00074d93d0"	"00075b22da"	−0.043
"00074d93d0"	"00075b2556"	−0.1543
"00074d93d0"	"00075b25de"	0.0708
"00074d93d0"	"00075b260c"	−0.1578
"00074d93d0"	"00075b26f1"	−0.0804
"00074d93d0"	"00075b2920"	−0.2503
"00074d93d0"	"00075b2a64"	−0.3831
"00074d93d0"	"00075b2a9d"	−0.2726
"00074d93d0"	"00075b2b37"	−0.4535
"00074d93d0"	"00075b2cdd"	−0.0529
"00074d93d0"	"00075b3038"	−0.0673
"00074d93d0"	"00075b30fe"	−0.2407
"00074d93d0"	"00075b3362"	0.0369
"00074d93d0"	"00075b350a"	−0.0986
"00074d93d0"	"00075b350e"	−0.1284
"00074d93d0"	"00075b3651"	0.1582
"00074d93d0"	"00075b38ca"	−0.1711
"00074d93d0"	"00075b39cc"	−0.0507
"00074d93d0"	"00075b3e1e"	−0.0544
"00074d93d0"	"00075b3e57"	0.2226
"00074d93d0"	"00075b4079"	−0.0925
"00074d93d0"	"00075b4150"	−0.0994
"00074d93d0"	"00075b4194"	−0.1617
"00074d93d0"	"00075b42d5"	−0.0785
"00074d93d0"	"00075b4424"	0.0147
"00074d93d0"	"00075b4470"	0.1012
"00074d93d0"	"00075b47ed"	0.0015
"00074d93d0"	"00075b4850"	0.0071
"00074d93d0"	"00075b4ca0"	−0.1626
"00074d93d0"	"00075b4d7f"	0.2783
"00074d93d0"	"00075b520f"	0.0315
"00074d93d0"	"00075b525f"	−0.0424
"00074d93d0"	"00075b58f8"	−0.1529
"00074d93d0"	"00075b5bcc"	−1e−04
"00074d93d0"	"00075b5bfa"	−0.3443
"00074d93d0"	"00075b6339"	0.1393
"00074d93d0"	"00075b6658"	0.1729
"00074d93d0"	"00075b679a"	0.3581
"00074d93d0"	"00075b6cb7"	−0.2997
"00074d93d0"	"00075b6df8"	0.1851
"00074d93d0"	"00075b6ff6"	−0.0717
"00074d93d0"	"00075b70ee"	0.0389
"00074d93d0"	"00075b7157"	−0.1294
"00074d93d0"	"00075b7225"	0.0661

"00074d93d0"	"00075b7c89"	0.3164
"00074d93d0"	"00075b9048"	−0.0823
"00074d93d0"	"00075d0801"	−0.0833
"00074d93d0"	"00075d1820"	0.1971
"00074d93d0"	"00075d1f3d"	−0.1076
"00074d93d0"	"00075d2329"	−0.1022
"00074d93d0"	"00075d2b9b"	−0.3081
"00074d93d0"	"00075d3941"	0.0645
"00074d93d0"	"00075d3e96"	−0.0033
"00074d93d0"	"00075d4864"	−0.1694
"00074d93d0"	"00075d5961"	−0.0082
"00074d93d0"	"00075d5a63"	0.0978
"00074d93d0"	"00075d6150"	−0.2017
"00074d93d0"	"00075d67d0"	−0.3052
"00074d93d0"	"00075d67e2"	0.0109
"00074d93d0"	"00075d73fc"	0.448
"00074d93d0"	"00075d7729"	0.0952
"00074d93d0"	"00075d778c"	−0.1952
"00074d93d0"	"00075d7b9e"	−0.1213
"00074d93d0"	"00075d7c8f"	0.081
"00074d93d0"	"00075d804d"	−0.312
"00074d93d0"	"00075d819f"	−0.263
"00074d93d0"	"00075d8601"	0.1859
"00074d93d0"	"00075d8c6a"	0.1257
"00074d93d0"	"00075dfedc"	0.1725
"00074d93d0"	"00075e05f2"	−0.2852
"00074d93d0"	"00075e0837"	−0.0097
"00074d93d0"	"00075e092e"	−0.1409
"00074d93d0"	"00075e0965"	−0.2815
"00074d93d0"	"00075e0bc8"	0.0741
"00074d93d0"	"00075e0fbb"	−0.2628
"00074d945b"	"00074d966c"	0.0266
"00074d945b"	"00074d9953"	0.329
"00074d945b"	"00074d99b2"	0.0572
"00074d945b"	"00074d99f3"	0.0322
"00074d945b"	"00074d9afd"	−0.007
"00074d945b"	"00074d9c87"	0.2191
"00074d945b"	"00074d9e9e"	−0.5333
"00074d945b"	"00074d9f30"	−0.1577
"00074d945b"	"00074da036"	0.217
"00074d945b"	"00074da082"	0.3053
"00074d945b"	"00074da136"	4e−04
"00074d945b"	"00074da3ed"	0.1187
"00074d945b"	"00074da4ac"	0.2039
"00074d945b"	"00074da4b8"	0.0658
"00074d945b"	"00074da5e8"	0.2879
"00074d945b"	"00074da6b4"	0.1988
"00074d945b"	"00074daa3c"	0.096
"00074d945b"	"00074daaf6"	−0.2676
"00074d945b"	"00074dad20"	−0.1654

"00074d945b"	"00074db098"	−0.1824
"00074d945b"	"00074db231"	0.1476
"00074d945b"	"00074db3a3"	0.1005
"00074d945b"	"00074db5d6"	−0.0575
"00074d945b"	"00074db632"	0.238
"00074d945b"	"00074db688"	−0.1141
"00074d945b"	"00074db8a6"	−0.4036
"00074d945b"	"00074dba19"	0.1108
"00074d945b"	"00074dbc2e"	−0.2409
"00074d945b"	"00074dbe51"	0.1666
"00074d945b"	"00074dbe5f"	−0.277
"00074d945b"	"00074dbf6d"	−0.0916
"00074d945b"	"00074dc4a5"	0.15
"00074d945b"	"00074dc50c"	0.0999
"00074d945b"	"00074dcdfa"	−0.0326
"00074d945b"	"00074dcf5f"	0.169
"00074d945b"	"00074dd007"	−0.1611
"00074d945b"	"00074dd163"	−0.2691
"00074d945b"	"00074dd3df"	0.0143
"00074d945b"	"00074dd577"	0.1347
"00074d945b"	"00074dd62e"	−0.0371
"00074d945b"	"00074dd73c"	−0.0655
"00074d945b"	"00074dda10"	−0.0024
"00074d945b"	"00074ddab8"	−0.0259
"00074d945b"	"00074ddd3d"	0.0938
"00074d945b"	"00074ddf16"	−0.0947
"00074d945b"	"00074ddfc1"	−0.1426
"00074d945b"	"00074de21a"	−0.1241
"00074d945b"	"00074de2a9"	0.2029
"00074d945b"	"00074de544"	0.1124
"00074d945b"	"00074de98a"	0.0273
"00074d945b"	"00074dea7e"	0.0046
"00074d945b"	"00074debd9"	−0.4676
"00074d945b"	"00074deca3"	−0.1988
"00074d945b"	"00074def43"	−0.0844
"00074d945b"	"00074def99"	−0.0649
"00074d945b"	"00074ecdad"	−0.1504
"00074d945b"	"00074ecf28"	0.1003
"00074d945b"	"00074ed1e1"	0.0791
"00074d945b"	"00074ed83b"	0.2372
"00074d945b"	"00074ee5e3"	−0.4947
"00074d945b"	"00074ee6e0"	−0.3549
"00074d945b"	"00074eea3a"	0.203
"00074d945b"	"00074eff82"	−0.2143
"00074d945b"	"00074f0477"	−0.1227
"00074d945b"	"00074f08c3"	−0.3478
"00074d945b"	"00074f1859"	−0.0073
"00074d945b"	"00074f2268"	−0.1147
"00074d945b"	"00074f28be"	0.1676
"00074d945b"	"00074f294b"	0.0326

"00074d945b"	"00074f2ddd"	0.2634
"00074d945b"	"00074f2e75"	-0.0561
"00074d945b"	"00074f3088"	0.0111
"00074d945b"	"00074f5a1c"	-0.1431
"00074d945b"	"00074f75b7"	-0.231
"00074d945b"	"00074f8cd9"	-0.0167
"00074d945b"	"00074f96dc"	-0.1965
"00074d945b"	"00074fabaa"	0.1177
"00074d945b"	"00074facd9"	-0.216
"00074d945b"	"00074fae3c"	-0.0836
"00074d945b"	"00074fb0a8"	-0.1966
"00074d945b"	"00074fb4e4"	-0.2882
"00074d945b"	"00074fb7c2"	0.1982
"00074d945b"	"00074fbd36"	-0.0498
"00074d945b"	"00074fc27f"	0.1032
"00074d945b"	"00074fc31d"	-0.3118
"00074d945b"	"00074fd569"	0.1293
"00074d945b"	"00074fef15"	-0.107
"00074d945b"	"00074ff562"	-0.2909
"00074d945b"	"00075007ca"	-0.1414
"00074d945b"	"0007500b86"	-0.2663
"00074d945b"	"0007500d05"	0.0072
"00074d945b"	"0007500ee4"	-0.1634
"00074d945b"	"0007500eee"	-0.3422
"00074d945b"	"00075013dc"	-0.0558
"00074d945b"	"000757b515"	0.0096
"00074d945b"	"000757bc5a"	0.0088
"00074d945b"	"000757c320"	-0.1603
"00074d945b"	"000757c9aa"	-0.223
"00074d945b"	"000757ccbe"	-0.0103
"00074d945b"	"000757cfa9"	-0.1173
"00074d945b"	"000757d390"	-0.1524
"00074d945b"	"000757d393"	-0.0999
"00074d945b"	"000757d598"	0.0585
"00074d945b"	"000757d5a2"	0.1307
"00074d945b"	"000757d790"	-0.0783
"00074d945b"	"000757e30c"	-0.0677
"00074d945b"	"000757e4b0"	0.0133
"00074d945b"	"000757e7a0"	-0.0167
"00074d945b"	"000757e8b3"	0.1135
"00074d945b"	"000757f627"	0.0469
"00074d945b"	"000757f925"	0.0237
"00074d945b"	"000757fa08"	0.0342
"00074d945b"	"000757fe52"	0.1919
"00074d945b"	"000758024a"	-0.075
"00074d945b"	"00075804bb"	-0.0798
"00074d945b"	"00075a0c04"	0.165
"00074d945b"	"00075a3110"	0.2852
"00074d945b"	"00075a341a"	-0.0018
"00074d945b"	"00075a3dcf"	-0.0524

"00074d945b"	"00075a3e22"	0.1118
"00074d945b"	"00075a48d8"	0.0353
"00074d945b"	"00075a5cfb"	0.1424
"00074d945b"	"00075a6151"	−0.1605
"00074d945b"	"00075a6708"	−0.0861
"00074d945b"	"00075a7319"	−0.2754
"00074d945b"	"00075a7723"	−0.0362
"00074d945b"	"00075a778b"	0.0473
"00074d945b"	"00075a7b8e"	0.0416
"00074d945b"	"00075a7c79"	0.2229
"00074d945b"	"00075a81b6"	−0.2809
"00074d945b"	"00075a82ac"	0.1323
"00074d945b"	"00075a98e5"	−0.1847
"00074d945b"	"00075b0d29"	−0.0549
"00074d945b"	"00075b102a"	−0.0115
"00074d945b"	"00075b1074"	0.0675
"00074d945b"	"00075b135d"	0.0567
"00074d945b"	"00075b138b"	0.2852
"00074d945b"	"00075b13a0"	−0.0367
"00074d945b"	"00075b13bd"	−0.0675
"00074d945b"	"00075b16a9"	0.0165
"00074d945b"	"00075b1a28"	−0.0285
"00074d945b"	"00075b1a97"	0.0225
"00074d945b"	"00075b1c7b"	−0.1805
"00074d945b"	"00075b1d24"	−0.1202
"00074d945b"	"00075b202b"	0.0691
"00074d945b"	"00075b22cb"	0.0648
"00074d945b"	"00075b22da"	0.1596
"00074d945b"	"00075b2556"	0.0966
"00074d945b"	"00075b25de"	−0.1519
"00074d945b"	"00075b260c"	0.1952
"00074d945b"	"00075b26f1"	−0.1387
"00074d945b"	"00075b2920"	0.0709
"00074d945b"	"00075b2a64"	−0.1622
"00074d945b"	"00075b2a9d"	−0.2107
"00074d945b"	"00075b2b37"	−0.2235
"00074d945b"	"00075b2cdd"	−0.0559
"00074d945b"	"00075b3038"	0.0463
"00074d945b"	"00075b30fe"	0.074
"00074d945b"	"00075b3362"	−0.2171
"00074d945b"	"00075b350a"	−0.0677
"00074d945b"	"00075b350e"	0.1346
"00074d945b"	"00075b3651"	−0.3928
"00074d945b"	"00075b38ca"	−0.2127
"00074d945b"	"00075b39cc"	−0.1797
"00074d945b"	"00075b3e1e"	0.0142
"00074d945b"	"00075b3e57"	0.1651
"00074d945b"	"00075b4079"	−0.2124
"00074d945b"	"00075b4150"	0.1916
"00074d945b"	"00075b4194"	0.1965

"00074d945b"	"00075b42d5"	−0.0555
"00074d945b"	"00075b4424"	−0.1428
"00074d945b"	"00075b4470"	0.0088
"00074d945b"	"00075b47ed"	−0.1015
"00074d945b"	"00075b4850"	−0.0578
"00074d945b"	"00075b4ca0"	−0.0671
"00074d945b"	"00075b4d7f"	0.2309
"00074d945b"	"00075b520f"	−0.3556
"00074d945b"	"00075b525f"	−0.1391
"00074d945b"	"00075b58f8"	−0.1231
"00074d945b"	"00075b5bcc"	0.0238
"00074d945b"	"00075b5bfa"	−0.2449
"00074d945b"	"00075b6339"	−0.0136
"00074d945b"	"00075b6658"	−0.4851
"00074d945b"	"00075b679a"	0.1916
"00074d945b"	"00075b6cb7"	−0.1728
"00074d945b"	"00075b6df8"	0.0659
"00074d945b"	"00075b6ff6"	0.2026
"00074d945b"	"00075b70ee"	0.1237
"00074d945b"	"00075b7157"	−0.1769
"00074d945b"	"00075b7225"	0.158
"00074d945b"	"00075b7c89"	0.2115
"00074d945b"	"00075b9048"	0.1416
"00074d945b"	"00075d0801"	−0.2097
"00074d945b"	"00075d1820"	0.0648
"00074d945b"	"00075d1f3d"	0.1346
"00074d945b"	"00075d2329"	0.0337
"00074d945b"	"00075d2b9b"	−0.0063
"00074d945b"	"00075d3941"	−0.0502
"00074d945b"	"00075d3e96"	0.1112
"00074d945b"	"00075d4864"	−0.1529
"00074d945b"	"00075d5961"	0.2265
"00074d945b"	"00075d5a63"	0.1201
"00074d945b"	"00075d6150"	−0.24
"00074d945b"	"00075d67d0"	−0.4247
"00074d945b"	"00075d67e2"	−0.108
"00074d945b"	"00075d73fc"	0.1919
"00074d945b"	"00075d7729"	−0.0707
"00074d945b"	"00075d778c"	−0.4368
"00074d945b"	"00075d7b9e"	−0.2778
"00074d945b"	"00075d7c8f"	0.2045
"00074d945b"	"00075d804d"	−0.1241
"00074d945b"	"00075d819f"	−0.1346
"00074d945b"	"00075d8601"	−0.0993
"00074d945b"	"00075d8c6a"	−0.0868
"00074d945b"	"00075dfedc"	0.0616
"00074d945b"	"00075e05f2"	−0.2798
"00074d945b"	"00075e0837"	0.0393
"00074d945b"	"00075e092e"	0.0171
"00074d945b"	"00075e0965"	−0.0456

"00074d945b"	"00075e0bc8"	0.0846
"00074d945b"	"00075e0fbb"	0.118
"00074d966c"	"00074d9953"	0.119
"00074d966c"	"00074d99b2"	−0.0508
"00074d966c"	"00074d99f3"	−0.0743
"00074d966c"	"00074d9afd"	−0.2926
"00074d966c"	"00074d9c87"	0.2245
"00074d966c"	"00074d9e9e"	−0.2851
"00074d966c"	"00074d9f30"	0.0415
"00074d966c"	"00074da036"	0.091
"00074d966c"	"00074da082"	−0.169
"00074d966c"	"00074da136"	−0.316
"00074d966c"	"00074da3ed"	−0.2369
"00074d966c"	"00074da4ac"	−0.059
"00074d966c"	"00074da4b8"	0.2283
"00074d966c"	"00074da5e8"	0.0633
"00074d966c"	"00074da6b4"	−0.007
"00074d966c"	"00074daa3c"	−0.0334
"00074d966c"	"00074daaf6"	−0.3136
"00074d966c"	"00074dad20"	−0.2834
"00074d966c"	"00074db098"	0.26
"00074d966c"	"00074db231"	0.1916
"00074d966c"	"00074db3a3"	0.2298
"00074d966c"	"00074db5d6"	0.1681
"00074d966c"	"00074db632"	0.1553
"00074d966c"	"00074db688"	−0.0354
"00074d966c"	"00074db8a6"	−0.2172
"00074d966c"	"00074dba19"	0.3422
"00074d966c"	"00074dbc2e"	−0.1965
"00074d966c"	"00074dbe51"	0.0751
"00074d966c"	"00074dbe5f"	−0.0798
"00074d966c"	"00074dbf6d"	0.3984
"00074d966c"	"00074dc4a5"	−0.3128
"00074d966c"	"00074dc50c"	0.0445
"00074d966c"	"00074dcdfa"	−0.14
"00074d966c"	"00074dcf5f"	−0.1001
"00074d966c"	"00074dd007"	−0.1484
"00074d966c"	"00074dd163"	−0.3061
"00074d966c"	"00074dd3df"	−0.0967
"00074d966c"	"00074dd577"	0.1219
"00074d966c"	"00074dd62e"	−0.0966
"00074d966c"	"00074dd73c"	−0.0885
"00074d966c"	"00074dda10"	0.0144
"00074d966c"	"00074ddab8"	0.0436
"00074d966c"	"00074ddd3d"	−0.047
"00074d966c"	"00074ddf16"	−0.281
"00074d966c"	"00074ddfc1"	0.1129
"00074d966c"	"00074de21a"	−0.061
"00074d966c"	"00074de2a9"	0.1416
"00074d966c"	"00074de544"	−0.0079

"00074d966c"	"00074de98a"	−0.1101
"00074d966c"	"00074dea7e"	−0.4171
"00074d966c"	"00074debd9"	−0.1056
"00074d966c"	"00074deca3"	−0.3536
"00074d966c"	"00074def43"	−0.1613
"00074d966c"	"00074def99"	−0.1999
"00074d966c"	"00074ecdad"	−0.4083
"00074d966c"	"00074ecf28"	−0.0683
"00074d966c"	"00074ed1e1"	−0.2597
"00074d966c"	"00074ed83b"	−0.0925
"00074d966c"	"00074ee5e3"	−0.166
"00074d966c"	"00074ee6e0"	−0.1505
"00074d966c"	"00074eea3a"	−0.2188
"00074d966c"	"00074eff82"	−0.1791
"00074d966c"	"00074f0477"	−0.0234
"00074d966c"	"00074f08c3"	−0.0193
"00074d966c"	"00074f1859"	−0.1748
"00074d966c"	"00074f2268"	0.4531
"00074d966c"	"00074f28be"	0.0893
"00074d966c"	"00074f294b"	−0.0541
"00074d966c"	"00074f2ddd"	−0.0234
"00074d966c"	"00074f2e75"	−0.0857
"00074d966c"	"00074f3088"	0.0579
"00074d966c"	"00074f5a1c"	0.0534
"00074d966c"	"00074f75b7"	−0.0292
"00074d966c"	"00074f8cd9"	−0.1214
"00074d966c"	"00074f96dc"	−0.0581
"00074d966c"	"00074fabaa"	0.0479
"00074d966c"	"00074facd9"	−0.1784
"00074d966c"	"00074fae3c"	−0.1199
"00074d966c"	"00074fb0a8"	−0.263
"00074d966c"	"00074fb4e4"	−0.329
"00074d966c"	"00074fb7c2"	−0.0202
"00074d966c"	"00074fbd36"	0.3522
"00074d966c"	"00074fc27f"	0.1752
"00074d966c"	"00074fc31d"	−0.2378
"00074d966c"	"00074fd569"	−0.352
"00074d966c"	"00074fef15"	−0.2579
"00074d966c"	"00074ff562"	−0.0722
"00074d966c"	"00075007ca"	0.0877
"00074d966c"	"0007500b86"	0.0121
"00074d966c"	"0007500d05"	−0.0715
"00074d966c"	"0007500ee4"	−0.2488
"00074d966c"	"0007500eee"	−0.0323
"00074d966c"	"00075013dc"	0.0147
"00074d966c"	"000757b515"	0.2513
"00074d966c"	"000757bc5a"	0.0194
"00074d966c"	"000757c320"	0.1892
"00074d966c"	"000757c9aa"	0.1717
"00074d966c"	"000757ccbe"	−0.1073

"00074d966c"	"000757cfa9"	0.1665
"00074d966c"	"000757d390"	-0.2753
"00074d966c"	"000757d393"	-0.0692
"00074d966c"	"000757d598"	0.0967
"00074d966c"	"000757d5a2"	-0.0898
"00074d966c"	"000757d790"	-0.2311
"00074d966c"	"000757e30c"	0.0387
"00074d966c"	"000757e4b0"	0.133
"00074d966c"	"000757e7a0"	-0.2221
"00074d966c"	"000757e8b3"	0.1663
"00074d966c"	"000757f627"	-0.0854
"00074d966c"	"000757f925"	0.0269
"00074d966c"	"000757fa08"	-0.0803
"00074d966c"	"000757fe52"	-0.031
"00074d966c"	"000758024a"	0.0522
"00074d966c"	"00075804bb"	0.3416
"00074d966c"	"00075a0c04"	0.033
"00074d966c"	"00075a3110"	0.0671
"00074d966c"	"00075a341a"	0.0391
"00074d966c"	"00075a3dcf"	-0.2001
"00074d966c"	"00075a3e22"	0.2028
"00074d966c"	"00075a48d8"	-0.0831
"00074d966c"	"00075a5cfb"	0.1153
"00074d966c"	"00075a6151"	0.026
"00074d966c"	"00075a6708"	-0.0962
"00074d966c"	"00075a7319"	-0.3604
"00074d966c"	"00075a7723"	-0.0054
"00074d966c"	"00075a778b"	-0.256
"00074d966c"	"00075a7b8e"	-0.2479
"00074d966c"	"00075a7c79"	0.1433
"00074d966c"	"00075a81b6"	0.0398
"00074d966c"	"00075a82ac"	-0.0933
"00074d966c"	"00075a98e5"	-0.0709
"00074d966c"	"00075b0d29"	0.1922
"00074d966c"	"00075b102a"	0.0957
"00074d966c"	"00075b1074"	0.067
"00074d966c"	"00075b135d"	0.0137
"00074d966c"	"00075b138b"	0.046
"00074d966c"	"00075b13a0"	-0.2716
"00074d966c"	"00075b13bd"	0.0564
"00074d966c"	"00075b16a9"	-0.234
"00074d966c"	"00075b1a28"	-0.2716
"00074d966c"	"00075b1a97"	-0.2332
"00074d966c"	"00075b1c7b"	-0.6533
"00074d966c"	"00075b1d24"	-0.0478
"00074d966c"	"00075b202b"	0.1661
"00074d966c"	"00075b22cb"	-0.4057
"00074d966c"	"00075b22da"	0.0369
"00074d966c"	"00075b2556"	0.2564
"00074d966c"	"00075b25de"	0.0424

"00074d966c"	"00075b260c"	0.1671
"00074d966c"	"00075b26f1"	-0.1368
"00074d966c"	"00075b2920"	0.2094
"00074d966c"	"00075b2a64"	0.2745
"00074d966c"	"00075b2a9d"	-0.0013
"00074d966c"	"00075b2b37"	0.0404
"00074d966c"	"00075b2cdd"	-0.3864
"00074d966c"	"00075b3038"	0.0282
"00074d966c"	"00075b30fe"	-0.036
"00074d966c"	"00075b3362"	-0.1993
"00074d966c"	"00075b350a"	0.5977
"00074d966c"	"00075b350e"	-0.1984
"00074d966c"	"00075b3651"	-0.3399
"00074d966c"	"00075b38ca"	-0.0831
"00074d966c"	"00075b39cc"	-0.1245
"00074d966c"	"00075b3e1e"	-0.0228
"00074d966c"	"00075b3e57"	0.0197
"00074d966c"	"00075b4079"	0.442
"00074d966c"	"00075b4150"	0.1591
"00074d966c"	"00075b4194"	-0.0608
"00074d966c"	"00075b42d5"	-0.1992
"00074d966c"	"00075b4424"	-0.0346
"00074d966c"	"00075b4470"	0.095
"00074d966c"	"00075b47ed"	-0.2885
"00074d966c"	"00075b4850"	0.0508
"00074d966c"	"00075b4ca0"	0.1505
"00074d966c"	"00075b4d7f"	0.2037
"00074d966c"	"00075b520f"	-0.001
"00074d966c"	"00075b525f"	-0.0206
"00074d966c"	"00075b58f8"	-0.0954
"00074d966c"	"00075b5bcc"	0.3414
"00074d966c"	"00075b5bfa"	-0.1853
"00074d966c"	"00075b6339"	0.3287
"00074d966c"	"00075b6658"	-0.0031
"00074d966c"	"00075b679a"	-0.1176
"00074d966c"	"00075b6cb7"	0.0024
"00074d966c"	"00075b6df8"	-0.0554
"00074d966c"	"00075b6ff6"	0.1943
"00074d966c"	"00075b70ee"	-0.0022
"00074d966c"	"00075b7157"	-0.0146
"00074d966c"	"00075b7225"	-0.3048
"00074d966c"	"00075b7c89"	0.076
"00074d966c"	"00075b9048"	0.0179
"00074d966c"	"00075d0801"	0.0732
"00074d966c"	"00075d1820"	-0.4221
"00074d966c"	"00075d1f3d"	0.0496
"00074d966c"	"00075d2329"	-0.0226
"00074d966c"	"00075d2b9b"	0.301
"00074d966c"	"00075d3941"	-0.1576
"00074d966c"	"00075d3e96"	-0.2892

"00074d966c"	"00075d4864"	−0.1514
"00074d966c"	"00075d5961"	0.0313
"00074d966c"	"00075d5a63"	0.2756
"00074d966c"	"00075d6150"	−0.3566
"00074d966c"	"00075d67d0"	−0.046
"00074d966c"	"00075d67e2"	0.0174
"00074d966c"	"00075d73fc"	−0.4451
"00074d966c"	"00075d7729"	0.2075
"00074d966c"	"00075d778c"	−0.1023
"00074d966c"	"00075d7b9e"	0.0732
"00074d966c"	"00075d7c8f"	0.0883
"00074d966c"	"00075d804d"	0.1023
"00074d966c"	"00075d819f"	−0.0044
"00074d966c"	"00075d8601"	−0.006
"00074d966c"	"00075d8c6a"	−0.045
"00074d966c"	"00075dfedc"	0.2479
"00074d966c"	"00075e05f2"	−0.1356
"00074d966c"	"00075e0837"	0.0621
"00074d966c"	"00075e092e"	−0.2446
"00074d966c"	"00075e0965"	−0.0054
"00074d966c"	"00075e0bc8"	0.1385
"00074d966c"	"00075e0fbb"	0.0294
"00074d9953"	"00074d99b2"	0.0998
"00074d9953"	"00074d99f3"	0.0892
"00074d9953"	"00074d9afd"	−0.2068
"00074d9953"	"00074d9c87"	0.1416
"00074d9953"	"00074d9e9e"	−0.5435
"00074d9953"	"00074d9f30"	0.0153
"00074d9953"	"00074da036"	0.0913
"00074d9953"	"00074da082"	−0.0281
"00074d9953"	"00074da136"	0.0109
"00074d9953"	"00074da3ed"	−0.0213
"00074d9953"	"00074da4ac"	0.2581
"00074d9953"	"00074da4b8"	0.4026
"00074d9953"	"00074da5e8"	0.3057
"00074d9953"	"00074da6b4"	0.3147
"00074d9953"	"00074daa3c"	0.276
"00074d9953"	"00074daaf6"	−0.1694
"00074d9953"	"00074dad20"	−0.2412
"00074d9953"	"00074db098"	−0.0374
"00074d9953"	"00074db231"	0.1206
"00074d9953"	"00074db3a3"	0.3361
"00074d9953"	"00074db5d6"	0.1012
"00074d9953"	"00074db632"	0.5596
"00074d9953"	"00074db688"	0.0028
"00074d9953"	"00074db8a6"	−0.1863
"00074d9953"	"00074dba19"	0.2462
"00074d9953"	"00074dbc2e"	−0.1826
"00074d9953"	"00074dbe51"	0.1489
"00074d9953"	"00074dbe5f"	−0.2505

"00074d9953"	"00074dbf6d"	0.0597
"00074d9953"	"00074dc4a5"	-0.134
"00074d9953"	"00074dc50c"	0.0652
"00074d9953"	"00074dcdfa"	-0.0081
"00074d9953"	"00074dcf5f"	0.0294
"00074d9953"	"00074dd007"	0.1726
"00074d9953"	"00074dd163"	-0.1532
"00074d9953"	"00074dd3df"	-0.1084
"00074d9953"	"00074dd577"	0.0742
"00074d9953"	"00074dd62e"	0.202
"00074d9953"	"00074dd73c"	-0.1412
"00074d9953"	"00074dda10"	0.1071
"00074d9953"	"00074ddab8"	0.1873
"00074d9953"	"00074ddd3d"	-0.0242
"00074d9953"	"00074ddf16"	-0.228
"00074d9953"	"00074ddfc1"	-0.0696
"00074d9953"	"00074de21a"	-0.0052
"00074d9953"	"00074de2a9"	0.38
"00074d9953"	"00074de544"	0.2824
"00074d9953"	"00074de98a"	0.1994
"00074d9953"	"00074dea7e"	-0.0574
"00074d9953"	"00074debd9"	-0.1943
"00074d9953"	"00074deca3"	-0.0226
"00074d9953"	"00074def43"	-0.1774
"00074d9953"	"00074def99"	-0.0782
"00074d9953"	"00074ecdad"	-0.2977
"00074d9953"	"00074ecf28"	-0.2693
"00074d9953"	"00074ed1e1"	-0.1429
"00074d9953"	"00074ed83b"	0.2833
"00074d9953"	"00074ee5e3"	-0.2196
"00074d9953"	"00074ee6e0"	-0.0075
"00074d9953"	"00074eea3a"	0.1118
"00074d9953"	"00074eff82"	-0.0802
"00074d9953"	"00074f0477"	-0.1832
"00074d9953"	"00074f08c3"	-0.053
"00074d9953"	"00074f1859"	0
"00074d9953"	"00074f2268"	0.0759
"00074d9953"	"00074f28be"	0.2618
"00074d9953"	"00074f294b"	-0.0757
"00074d9953"	"00074f2ddd"	0.0245
"00074d9953"	"00074f2e75"	0.0194
"00074d9953"	"00074f3088"	0.0173
"00074d9953"	"00074f5a1c"	0.1922
"00074d9953"	"00074f75b7"	0.0323
"00074d9953"	"00074f8cd9"	0.1141
"00074d9953"	"00074f96dc"	-0.051
"00074d9953"	"00074fabaa"	0.5531
"00074d9953"	"00074facd9"	-0.1587
"00074d9953"	"00074fae3c"	-0.1213
"00074d9953"	"00074fb0a8"	-0.0854

"00074d9953"	"00074fb4e4"	−0.2289
"00074d9953"	"00074fb7c2"	0.2904
"00074d9953"	"00074fbd36"	0.0991
"00074d9953"	"00074fc27f"	0.2775
"00074d9953"	"00074fc31d"	−0.173
"00074d9953"	"00074fd569"	0.0959
"00074d9953"	"00074fef15"	−0.2176
"00074d9953"	"00074ff562"	−0.1625
"00074d9953"	"00075007ca"	−0.0889
"00074d9953"	"0007500b86"	0.3098
"00074d9953"	"0007500d05"	0.1855
"00074d9953"	"0007500ee4"	−0.0867
"00074d9953"	"0007500eee"	−0.2536
"00074d9953"	"00075013dc"	0.1505
"00074d9953"	"000757b515"	0.0193
"00074d9953"	"000757bc5a"	0.3266
"00074d9953"	"000757c320"	−0.1105
"00074d9953"	"000757c9aa"	−0.0078
"00074d9953"	"000757ccbe"	0.126
"00074d9953"	"000757cfa9"	−0.0532
"00074d9953"	"000757d390"	−0.1479
"00074d9953"	"000757d393"	0.0622
"00074d9953"	"000757d598"	0.115
"00074d9953"	"000757d5a2"	−0.1132
"00074d9953"	"000757d790"	0.0785
"00074d9953"	"000757e30c"	0.1749
"00074d9953"	"000757e4b0"	0.1106
"00074d9953"	"000757e7a0"	−0.1132
"00074d9953"	"000757e8b3"	0.2402
"00074d9953"	"000757f627"	−0.0167
"00074d9953"	"000757f925"	0.1731
"00074d9953"	"000757fa08"	0.1743
"00074d9953"	"000757fe52"	0.2244
"00074d9953"	"000758024a"	0.2055
"00074d9953"	"00075804bb"	0.1923
"00074d9953"	"00075a0c04"	0.025
"00074d9953"	"00075a3110"	0.2797
"00074d9953"	"00075a341a"	0.1296
"00074d9953"	"00075a3dcf"	−0.1171
"00074d9953"	"00075a3e22"	0.2966
"00074d9953"	"00075a48d8"	0.0554
"00074d9953"	"00075a5cfb"	0.5654
"00074d9953"	"00075a6151"	−0.0163
"00074d9953"	"00075a6708"	−0.0533
"00074d9953"	"00075a7319"	−0.3131
"00074d9953"	"00075a7723"	0.1985
"00074d9953"	"00075a778b"	0.3305
"00074d9953"	"00075a7b8e"	0.0192
"00074d9953"	"00075a7c79"	0.1213
"00074d9953"	"00075a81b6"	−0.0496

"00074d9953"	"00075a82ac"	0.2886
"00074d9953"	"00075a98e5"	0.0867
"00074d9953"	"00075b0d29"	0.2673
"00074d9953"	"00075b102a"	0.1446
"00074d9953"	"00075b1074"	−0.0435
"00074d9953"	"00075b135d"	0.0945
"00074d9953"	"00075b138b"	0.2752
"00074d9953"	"00075b13a0"	−0.0907
"00074d9953"	"00075b13bd"	−0.0227
"00074d9953"	"00075b16a9"	−0.0427
"00074d9953"	"00075b1a28"	0.0736
"00074d9953"	"00075b1a97"	−0.0166
"00074d9953"	"00075b1c7b"	−0.2883
"00074d9953"	"00075b1d24"	−0.0347
"00074d9953"	"00075b202b"	0.2857
"00074d9953"	"00075b22cb"	−0.0585
"00074d9953"	"00075b22da"	−0.023
"00074d9953"	"00075b2556"	0.1365
"00074d9953"	"00075b25de"	−0.0013
"00074d9953"	"00075b260c"	0.3158
"00074d9953"	"00075b26f1"	−0.1784
"00074d9953"	"00075b2920"	0.375
"00074d9953"	"00075b2a64"	0.3678
"00074d9953"	"00075b2a9d"	−0.0734
"00074d9953"	"00075b2b37"	−0.263
"00074d9953"	"00075b2cdd"	0.1162
"00074d9953"	"00075b3038"	0
"00074d9953"	"00075b30fe"	−0.0488
"00074d9953"	"00075b3362"	−0.0284
"00074d9953"	"00075b350a"	0.1226
"00074d9953"	"00075b350e"	0.2967
"00074d9953"	"00075b3651"	−0.1759
"00074d9953"	"00075b38ca"	0.0164
"00074d9953"	"00075b39cc"	−0.2524
"00074d9953"	"00075b3e1e"	−0.0081
"00074d9953"	"00075b3e57"	0.1589
"00074d9953"	"00075b4079"	−0.1497
"00074d9953"	"00075b4150"	0.5011
"00074d9953"	"00075b4194"	0.2324
"00074d9953"	"00075b42d5"	0.0833
"00074d9953"	"00075b4424"	0.2043
"00074d9953"	"00075b4470"	0.2924
"00074d9953"	"00075b47ed"	−0.167
"00074d9953"	"00075b4850"	0.1503
"00074d9953"	"00075b4ca0"	0.0237
"00074d9953"	"00075b4d7f"	0.2571
"00074d9953"	"00075b520f"	−0.2402
"00074d9953"	"00075b525f"	−0.1097
"00074d9953"	"00075b58f8"	0.2507
"00074d9953"	"00075b5bcc"	0.0205

"00074d9953"	"00075b5bfa"	−0.1876
"00074d9953"	"00075b6339"	0.1312
"00074d9953"	"00075b6658"	−0.3221
"00074d9953"	"00075b679a"	0.0129
"00074d9953"	"00075b6cb7"	−0.059
"00074d9953"	"00075b6df8"	0.0185
"00074d9953"	"00075b6ff6"	0.1371
"00074d9953"	"00075b70ee"	0.1616
"00074d9953"	"00075b7157"	0.0409
"00074d9953"	"00075b7225"	0.1088
"00074d9953"	"00075b7c89"	0.1295
"00074d9953"	"00075b9048"	0.3211
"00074d9953"	"00075d0801"	0.0781
"00074d9953"	"00075d1820"	−0.1393
"00074d9953"	"00075d1f3d"	0.3021
"00074d9953"	"00075d2329"	−0.0582
"00074d9953"	"00075d2b9b"	0.2626
"00074d9953"	"00075d3941"	−0.0376
"00074d9953"	"00075d3e96"	0.0989
"00074d9953"	"00075d4864"	−0.017
"00074d9953"	"00075d5961"	0.0146
"00074d9953"	"00075d5a63"	0.2405
"00074d9953"	"00075d6150"	−0.2649
"00074d9953"	"00075d67d0"	−0.5066
"00074d9953"	"00075d67e2"	0.2087
"00074d9953"	"00075d73fc"	0.0241
"00074d9953"	"00075d7729"	−0.1122
"00074d9953"	"00075d778c"	−0.3258
"00074d9953"	"00075d7b9e"	−0.0805
"00074d9953"	"00075d7c8f"	0.0323
"00074d9953"	"00075d804d"	−0.0053
"00074d9953"	"00075d819f"	−0.0524
"00074d9953"	"00075d8601"	−0.1021
"00074d9953"	"00075d8c6a"	0.14
"00074d9953"	"00075dfedc"	0.0204
"00074d9953"	"00075e05f2"	0.1889
"00074d9953"	"00075e0837"	−0.0308
"00074d9953"	"00075e092e"	−0.0966
"00074d9953"	"00075e0965"	0.3469
"00074d9953"	"00075e0bc8"	0.2454
"00074d9953"	"00075e0fbb"	−0.1495
"00074d99b2"	"00074d99f3"	−0.1849
"00074d99b2"	"00074d9afd"	−0.2292
"00074d99b2"	"00074d9c87"	0.277
"00074d99b2"	"00074d9e9e"	−0.2934
"00074d99b2"	"00074d9f30"	−0.0659
"00074d99b2"	"00074da036"	0.1647
"00074d99b2"	"00074da082"	−0.1098
"00074d99b2"	"00074da136"	0.1824
"00074d99b2"	"00074da3ed"	−0.1116

"00074d99b2"	"00074da4ac"	−0.164
"00074d99b2"	"00074da4b8"	−0.1014
"00074d99b2"	"00074da5e8"	0.3129
"00074d99b2"	"00074da6b4"	0.1574
"00074d99b2"	"00074daa3c"	−0.3087
"00074d99b2"	"00074daaf6"	−0.1341
"00074d99b2"	"00074dad20"	−0.3133
"00074d99b2"	"00074db098"	−0.1901
"00074d99b2"	"00074db231"	0.1254
"00074d99b2"	"00074db3a3"	−0.1626
"00074d99b2"	"00074db5d6"	−0.0956
"00074d99b2"	"00074db632"	−0.0894
"00074d99b2"	"00074db688"	0.3405
"00074d99b2"	"00074db8a6"	−0.213
"00074d99b2"	"00074dba19"	0.1042
"00074d99b2"	"00074dbc2e"	−0.3096
"00074d99b2"	"00074dbe51"	0.0534
"00074d99b2"	"00074dbe5f"	−0.5717
"00074d99b2"	"00074dbf6d"	−0.59
"00074d99b2"	"00074dc4a5"	−0.06
"00074d99b2"	"00074dc50c"	−0.1576
"00074d99b2"	"00074dcdfa"	−0.2207
"00074d99b2"	"00074dcf5f"	−0.128
"00074d99b2"	"00074dd007"	−0.1602
"00074d99b2"	"00074dd163"	−0.1305
"00074d99b2"	"00074dd3df"	−0.2622
"00074d99b2"	"00074dd577"	0.1436
"00074d99b2"	"00074dd62e"	0.0135
"00074d99b2"	"00074dd73c"	−0.0907
"00074d99b2"	"00074dda10"	−0.2345
"00074d99b2"	"00074ddab8"	−0.282
"00074d99b2"	"00074ddd3d"	−0.1325
"00074d99b2"	"00074ddf16"	−0.3266
"00074d99b2"	"00074ddfc1"	−0.3095
"00074d99b2"	"00074de21a"	−0.0577
"00074d99b2"	"00074de2a9"	−0.0911
"00074d99b2"	"00074de544"	0.0301
"00074d99b2"	"00074de98a"	−0.0475
"00074d99b2"	"00074dea7e"	−0.2222
"00074d99b2"	"00074debd9"	−0.5997
"00074d99b2"	"00074deca3"	−0.2685
"00074d99b2"	"00074def43"	−0.4866
"00074d99b2"	"00074def99"	0.199
"00074d99b2"	"00074ecdad"	−0.3171
"00074d99b2"	"00074ecf28"	−0.0604
"00074d99b2"	"00074ed1e1"	−0.2622
"00074d99b2"	"00074ed83b"	−0.0397
"00074d99b2"	"00074ee5e3"	−0.4621
"00074d99b2"	"00074ee6e0"	−0.134
"00074d99b2"	"00074eea3a"	−0.1725

"00074d99b2"	"00074eff82"	−0.1567
"00074d99b2"	"00074f0477"	−0.3299
"00074d99b2"	"00074f08c3"	−0.3164
"00074d99b2"	"00074f1859"	−0.2164
"00074d99b2"	"00074f2268"	−0.0698
"00074d99b2"	"00074f28be"	0.0885
"00074d99b2"	"00074f294b"	−0.0136
"00074d99b2"	"00074f2ddd"	0.165
"00074d99b2"	"00074f2e75"	−0.0765
"00074d99b2"	"00074f3088"	−0.0085
"00074d99b2"	"00074f5a1c"	0.0558
"00074d99b2"	"00074f75b7"	−0.1108
"00074d99b2"	"00074f8cd9"	−0.1903
"00074d99b2"	"00074f96dc"	−0.4059
"00074d99b2"	"00074fabaa"	−0.2081
"00074d99b2"	"00074facd9"	−0.2917
"00074d99b2"	"00074fae3c"	−0.1379
"00074d99b2"	"00074fb0a8"	−0.0824
"00074d99b2"	"00074fb4e4"	−0.3357
"00074d99b2"	"00074fb7c2"	0.2625
"00074d99b2"	"00074fbd36"	0.0853
"00074d99b2"	"00074fc27f"	−0.184
"00074d99b2"	"00074fc31d"	0.1253
"00074d99b2"	"00074fd569"	−0.137
"00074d99b2"	"00074fef15"	0.0129
"00074d99b2"	"00074ff562"	−0.199
"00074d99b2"	"00075007ca"	−0.2808
"00074d99b2"	"0007500b86"	−0.1294
"00074d99b2"	"0007500d05"	−0.2497
"00074d99b2"	"0007500ee4"	−0.0523
"00074d99b2"	"0007500eee"	−0.4932
"00074d99b2"	"00075013dc"	−0.1226
"00074d99b2"	"000757b515"	0.0765
"00074d99b2"	"000757bc5a"	−0.0068
"00074d99b2"	"000757c320"	−0.4911
"00074d99b2"	"000757c9aa"	−0.2319
"00074d99b2"	"000757ccbe"	0.0012
"00074d99b2"	"000757cfa9"	0.2627
"00074d99b2"	"000757d390"	−0.1574
"00074d99b2"	"000757d393"	0.0414
"00074d99b2"	"000757d598"	−0.1364
"00074d99b2"	"000757d5a2"	−0.0864
"00074d99b2"	"000757d790"	−0.1674
"00074d99b2"	"000757e30c"	−0.2209
"00074d99b2"	"000757e4b0"	−0.0276
"00074d99b2"	"000757e7a0"	−0.1373
"00074d99b2"	"000757e8b3"	−0.0013
"00074d99b2"	"000757f627"	−0.1323
"00074d99b2"	"000757f925"	−0.278
"00074d99b2"	"000757fa08"	0.3401

"00074d99b2"	"000757fe52"	-0.1123
"00074d99b2"	"000758024a"	-0.0959
"00074d99b2"	"00075804bb"	-0.2107
"00074d99b2"	"00075a0c04"	0.1738
"00074d99b2"	"00075a3110"	0.2164
"00074d99b2"	"00075a341a"	0.1055
"00074d99b2"	"00075a3dcf"	-0.0629
"00074d99b2"	"00075a3e22"	0.1132
"00074d99b2"	"00075a48d8"	-0.1084
"00074d99b2"	"00075a5cfb"	-0.1412
"00074d99b2"	"00075a6151"	-0.2934
"00074d99b2"	"00075a6708"	-0.0484
"00074d99b2"	"00075a7319"	-0.289
"00074d99b2"	"00075a7723"	0.1053
"00074d99b2"	"00075a778b"	-0.0872
"00074d99b2"	"00075a7b8e"	-0.2166
"00074d99b2"	"00075a7c79"	-0.0022
"00074d99b2"	"00075a81b6"	-0.2581
"00074d99b2"	"00075a82ac"	0.1337
"00074d99b2"	"00075a98e5"	-0.3177
"00074d99b2"	"00075b0d29"	0.2176
"00074d99b2"	"00075b102a"	-0.2248
"00074d99b2"	"00075b1074"	-0.1073
"00074d99b2"	"00075b135d"	0.4024
"00074d99b2"	"00075b138b"	-0.2343
"00074d99b2"	"00075b13a0"	-0.2509
"00074d99b2"	"00075b13bd"	0.0286
"00074d99b2"	"00075b16a9"	-0.292
"00074d99b2"	"00075b1a28"	-0.1141
"00074d99b2"	"00075b1a97"	-0.0377
"00074d99b2"	"00075b1c7b"	-0.4493
"00074d99b2"	"00075b1d24"	-0.0417
"00074d99b2"	"00075b202b"	-0.1073
"00074d99b2"	"00075b22cb"	-0.347
"00074d99b2"	"00075b22da"	-0.0627
"00074d99b2"	"00075b2556"	0.0407
"00074d99b2"	"00075b25de"	0.0067
"00074d99b2"	"00075b260c"	-0.1398
"00074d99b2"	"00075b26f1"	-0.232
"00074d99b2"	"00075b2920"	-0.1698
"00074d99b2"	"00075b2a64"	-0.3557
"00074d99b2"	"00075b2a9d"	-0.1933
"00074d99b2"	"00075b2b37"	-0.0606
"00074d99b2"	"00075b2cdd"	-0.0733
"00074d99b2"	"00075b3038"	-0.0945
"00074d99b2"	"00075b30fe"	-0.0273
"00074d99b2"	"00075b3362"	-0.1361
"00074d99b2"	"00075b350a"	-0.2282
"00074d99b2"	"00075b350e"	-0.0161
"00074d99b2"	"00075b3651"	-0.3811

"00074d99b2"	"00075b38ca"	-0.0732
"00074d99b2"	"00075b39cc"	-0.3049
"00074d99b2"	"00075b3e1e"	-0.3189
"00074d99b2"	"00075b3e57"	-0.0966
"00074d99b2"	"00075b4079"	-0.3064
"00074d99b2"	"00075b4150"	-0.0638
"00074d99b2"	"00075b4194"	-0.0866
"00074d99b2"	"00075b42d5"	-0.1109
"00074d99b2"	"00075b4424"	-0.1044
"00074d99b2"	"00075b4470"	-0.0068
"00074d99b2"	"00075b47ed"	-0.2226
"00074d99b2"	"00075b4850"	-0.0896
"00074d99b2"	"00075b4ca0"	0.07
"00074d99b2"	"00075b4d7f"	0.2213
"00074d99b2"	"00075b520f"	-0.0489
"00074d99b2"	"00075b525f"	-0.187
"00074d99b2"	"00075b58f8"	0.0215
"00074d99b2"	"00075b5bcc"	-0.1002
"00074d99b2"	"00075b5bfa"	-0.221
"00074d99b2"	"00075b6339"	-0.0775
"00074d99b2"	"00075b6658"	-0.0054
"00074d99b2"	"00075b679a"	0.0414
"00074d99b2"	"00075b6cb7"	-0.3549
"00074d99b2"	"00075b6df8"	0.1524
"00074d99b2"	"00075b6ff6"	-0.036
"00074d99b2"	"00075b70ee"	-0.039
"00074d99b2"	"00075b7157"	-0.0291
"00074d99b2"	"00075b7225"	-0.0093
"00074d99b2"	"00075b7c89"	0.0902
"00074d99b2"	"00075b9048"	0.0207
"00074d99b2"	"00075d0801"	0.0417
"00074d99b2"	"00075d1820"	-0.3153
"00074d99b2"	"00075d1f3d"	-0.166
"00074d99b2"	"00075d2329"	-0.0464
"00074d99b2"	"00075d2b9b"	-0.2854
"00074d99b2"	"00075d3941"	-0.2673
"00074d99b2"	"00075d3e96"	-0.073
"00074d99b2"	"00075d4864"	0.0694
"00074d99b2"	"00075d5961"	0.2104
"00074d99b2"	"00075d5a63"	-0.1412
"00074d99b2"	"00075d6150"	0.0314
"00074d99b2"	"00075d67d0"	-0.4092
"00074d99b2"	"00075d67e2"	-0.3527
"00074d99b2"	"00075d73fc"	-0.0214
"00074d99b2"	"00075d7729"	0.464
"00074d99b2"	"00075d778c"	-0.2065
"00074d99b2"	"00075d7b9e"	0.0672
"00074d99b2"	"00075d7c8f"	-0.0114
"00074d99b2"	"00075d804d"	-0.2466
"00074d99b2"	"00075d819f"	-0.264

"00074d99b2"	"00075d8601"	−0.0413
"00074d99b2"	"00075d8c6a"	0.1085
"00074d99b2"	"00075dfedc"	−0.0651
"00074d99b2"	"00075e05f2"	−0.5457
"00074d99b2"	"00075e0837"	−0.3813
"00074d99b2"	"00075e092e"	−0.1269
"00074d99b2"	"00075e0965"	−0.108
"00074d99b2"	"00075e0bc8"	0.1567
"00074d99b2"	"00075e0fbb"	−0.1659
"00074d99f3"	"00074d9afd"	0.0457
"00074d99f3"	"00074d9c87"	0.2198
"00074d99f3"	"00074d9e9e"	−0.1438
"00074d99f3"	"00074d9f30"	0.3566
"00074d99f3"	"00074da036"	0.3073
"00074d99f3"	"00074da082"	0.2369
"00074d99f3"	"00074da136"	0.0605
"00074d99f3"	"00074da3ed"	0.1062
"00074d99f3"	"00074da4ac"	0.4106
"00074d99f3"	"00074da4b8"	0.1568
"00074d99f3"	"00074da5e8"	0.2038
"00074d99f3"	"00074da6b4"	0.1752
"00074d99f3"	"00074daa3c"	−0.0296
"00074d99f3"	"00074daaf6"	0.2479
"00074d99f3"	"00074dad20"	−0.0499
"00074d99f3"	"00074db098"	−0.082
"00074d99f3"	"00074db231"	−0.0953
"00074d99f3"	"00074db3a3"	0.1837
"00074d99f3"	"00074db5d6"	0.0691
"00074d99f3"	"00074db632"	0.208
"00074d99f3"	"00074db688"	0.0899
"00074d99f3"	"00074db8a6"	−0.208
"00074d99f3"	"00074dba19"	−0.055
"00074d99f3"	"00074dbc2e"	−0.068
"00074d99f3"	"00074dbe51"	0.3446
"00074d99f3"	"00074dbe5f"	−0.2421
"00074d99f3"	"00074dbf6d"	−0.1281
"00074d99f3"	"00074dc4a5"	−0.0383
"00074d99f3"	"00074dc50c"	−0.0771
"00074d99f3"	"00074dcdfa"	0.0189
"00074d99f3"	"00074dcf5f"	0.1244
"00074d99f3"	"00074dd007"	−0.0535
"00074d99f3"	"00074dd163"	−0.2318
"00074d99f3"	"00074dd3df"	−0.0044
"00074d99f3"	"00074dd577"	0.0158
"00074d99f3"	"00074dd62e"	0.2401
"00074d99f3"	"00074dd73c"	0.146
"00074d99f3"	"00074dda10"	0.0467
"00074d99f3"	"00074ddab8"	0.0516
"00074d99f3"	"00074ddd3d"	0.1368
"00074d99f3"	"00074ddf16"	0.1464

"00074d99f3"	"00074ddfc1"	0.0161
"00074d99f3"	"00074de21a"	0.007
"00074d99f3"	"00074de2a9"	0.1485
"00074d99f3"	"00074de544"	0.101
"00074d99f3"	"00074de98a"	0.0362
"00074d99f3"	"00074dea7e"	-0.0104
"00074d99f3"	"00074debd9"	-0.2768
"00074d99f3"	"00074deca3"	-0.1575
"00074d99f3"	"00074def43"	0.145
"00074d99f3"	"00074def99"	0.2514
"00074d99f3"	"00074ecdad"	-0.3355
"00074d99f3"	"00074ecf28"	0.1247
"00074d99f3"	"00074ed1e1"	-0.0948
"00074d99f3"	"00074ed83b"	0.4101
"00074d99f3"	"00074ee5e3"	0.0335
"00074d99f3"	"00074ee6e0"	0.0937
"00074d99f3"	"00074eea3a"	0.1207
"00074d99f3"	"00074eff82"	0.1764
"00074d99f3"	"00074f0477"	-0.1573
"00074d99f3"	"00074f08c3"	-6e-04
"00074d99f3"	"00074f1859"	-0.0406
"00074d99f3"	"00074f2268"	-0.1822
"00074d99f3"	"00074f28be"	-0.0821
"00074d99f3"	"00074f294b"	0.2281
"00074d99f3"	"00074f2ddd"	-0.0233
"00074d99f3"	"00074f2e75"	-0.0764
"00074d99f3"	"00074f3088"	0.3458
"00074d99f3"	"00074f5a1c"	-0.146
"00074d99f3"	"00074f75b7"	0.0425
"00074d99f3"	"00074f8cd9"	-0.0138
"00074d99f3"	"00074f96dc"	0.0226
"00074d99f3"	"00074fabaa"	-0.1243
"00074d99f3"	"00074facd9"	-0.1026
"00074d99f3"	"00074fae3c"	0.1811
"00074d99f3"	"00074fb0a8"	-0.0103
"00074d99f3"	"00074fb4e4"	0.0125
"00074d99f3"	"00074fb7c2"	0.2377
"00074d99f3"	"00074fbd36"	-0.1519
"00074d99f3"	"00074fc27f"	0.0809
"00074d99f3"	"00074fc31d"	0.0558
"00074d99f3"	"00074fd569"	0.2697
"00074d99f3"	"00074fef15"	0.2822
"00074d99f3"	"00074ff562"	-0.2767
"00074d99f3"	"00075007ca"	0.0083
"00074d99f3"	"0007500b86"	0.0749
"00074d99f3"	"0007500d05"	0.0919
"00074d99f3"	"0007500ee4"	0.2566
"00074d99f3"	"0007500eee"	-0.1674
"00074d99f3"	"00075013dc"	-0.3083
"00074d99f3"	"000757b515"	0.2551

"00074d99f3"	"000757bc5a"	-0.0415
"00074d99f3"	"000757c320"	0.0879
"00074d99f3"	"000757c9aa"	0.3124
"00074d99f3"	"000757ccbe"	0.1129
"00074d99f3"	"000757cfa9"	-0.2408
"00074d99f3"	"000757d390"	-0.0637
"00074d99f3"	"000757d393"	0.0556
"00074d99f3"	"000757d598"	0.051
"00074d99f3"	"000757d5a2"	0.0067
"00074d99f3"	"000757d790"	0.2791
"00074d99f3"	"000757e30c"	0.0939
"00074d99f3"	"000757e4b0"	0.1263
"00074d99f3"	"000757e7a0"	0.1571
"00074d99f3"	"000757e8b3"	0.3654
"00074d99f3"	"000757f627"	0.1201
"00074d99f3"	"000757f925"	0.2657
"00074d99f3"	"000757fa08"	-0.2115
"00074d99f3"	"000757fe52"	0.0709
"00074d99f3"	"000758024a"	-0.1257
"00074d99f3"	"00075804bb"	-0.1247
"00074d99f3"	"00075a0c04"	0.0784
"00074d99f3"	"00075a3110"	0.077
"00074d99f3"	"00075a341a"	0.1112
"00074d99f3"	"00075a3dcf"	0.062
"00074d99f3"	"00075a3e22"	0.1364
"00074d99f3"	"00075a48d8"	0.1436
"00074d99f3"	"00075a5cfb"	-0.1205
"00074d99f3"	"00075a6151"	0.016
"00074d99f3"	"00075a6708"	0.0195
"00074d99f3"	"00075a7319"	0.0122
"00074d99f3"	"00075a7723"	0.1592
"00074d99f3"	"00075a778b"	-0.0667
"00074d99f3"	"00075a7b8e"	0.0031
"00074d99f3"	"00075a7c79"	0.1196
"00074d99f3"	"00075a81b6"	0.2099
"00074d99f3"	"00075a82ac"	0.2357
"00074d99f3"	"00075a98e5"	0.0626
"00074d99f3"	"00075b0d29"	-0.0722
"00074d99f3"	"00075b102a"	0.0449
"00074d99f3"	"00075b1074"	-0.0411
"00074d99f3"	"00075b135d"	-0.1786
"00074d99f3"	"00075b138b"	0.1621
"00074d99f3"	"00075b13a0"	-0.1391
"00074d99f3"	"00075b13bd"	0.2101
"00074d99f3"	"00075b16a9"	0.2193
"00074d99f3"	"00075b1a28"	-0.1663
"00074d99f3"	"00075b1a97"	0.2362
"00074d99f3"	"00075b1c7b"	-0.0435
"00074d99f3"	"00075b1d24"	0.2846
"00074d99f3"	"00075b202b"	0.0062

"00074d99f3"	"00075b22cb"	0.1534
"00074d99f3"	"00075b22da"	0.0627
"00074d99f3"	"00075b2556"	0.0834
"00074d99f3"	"00075b25de"	0.1145
"00074d99f3"	"00075b260c"	0.1247
"00074d99f3"	"00075b26f1"	0.2626
"00074d99f3"	"00075b2920"	-0.0029
"00074d99f3"	"00075b2a64"	-0.1651
"00074d99f3"	"00075b2a9d"	-0.0783
"00074d99f3"	"00075b2b37"	-0.1403
"00074d99f3"	"00075b2cdd"	0.191
"00074d99f3"	"00075b3038"	0.1937
"00074d99f3"	"00075b30fe"	0.0553
"00074d99f3"	"00075b3362"	0.3106
"00074d99f3"	"00075b350a"	-0.1229
"00074d99f3"	"00075b350e"	-0.0996
"00074d99f3"	"00075b3651"	0.4572
"00074d99f3"	"00075b38ca"	-0.3352
"00074d99f3"	"00075b39cc"	0.205
"00074d99f3"	"00075b3e1e"	0.001
"00074d99f3"	"00075b3e57"	0.2681
"00074d99f3"	"00075b4079"	-0.0641
"00074d99f3"	"00075b4150"	-0.049
"00074d99f3"	"00075b4194"	0.1838
"00074d99f3"	"00075b42d5"	-0.0305
"00074d99f3"	"00075b4424"	0.0202
"00074d99f3"	"00075b4470"	-0.0415
"00074d99f3"	"00075b47ed"	0.1044
"00074d99f3"	"00075b4850"	-0.0035
"00074d99f3"	"00075b4ca0"	0.1033
"00074d99f3"	"00075b4d7f"	0.1211
"00074d99f3"	"00075b520f"	-0.0347
"00074d99f3"	"00075b525f"	0.6542
"00074d99f3"	"00075b58f8"	-0.3021
"00074d99f3"	"00075b5bcc"	0.1632
"00074d99f3"	"00075b5bfa"	0.0668
"00074d99f3"	"00075b6339"	0.3827
"00074d99f3"	"00075b6658"	0.106
"00074d99f3"	"00075b679a"	0.2564
"00074d99f3"	"00075b6cb7"	-0.1618
"00074d99f3"	"00075b6df8"	0.1178
"00074d99f3"	"00075b6ff6"	0.1471
"00074d99f3"	"00075b70ee"	-0.1864
"00074d99f3"	"00075b7157"	0.3299
"00074d99f3"	"00075b7225"	0.0262
"00074d99f3"	"00075b7c89"	0.0361
"00074d99f3"	"00075b9048"	0.1866
"00074d99f3"	"00075d0801"	0.157
"00074d99f3"	"00075d1820"	0.2725
"00074d99f3"	"00075d1f3d"	-0.0264

"00074d99f3"	"00075d2329"	0.0139
"00074d99f3"	"00075d2b9b"	-0.0687
"00074d99f3"	"00075d3941"	-0.0372
"00074d99f3"	"00075d3e96"	0.3136
"00074d99f3"	"00075d4864"	-0.2202
"00074d99f3"	"00075d5961"	-0.1198
"00074d99f3"	"00075d5a63"	0.13
"00074d99f3"	"00075d6150"	-0.1503
"00074d99f3"	"00075d67d0"	0.2782
"00074d99f3"	"00075d67e2"	0.1576
"00074d99f3"	"00075d73fc"	0.077
"00074d99f3"	"00075d7729"	0.0669
"00074d99f3"	"00075d778c"	0.3136
"00074d99f3"	"00075d7b9e"	0.4413
"00074d99f3"	"00075d7c8f"	0.3368
"00074d99f3"	"00075d804d"	0.1818
"00074d99f3"	"00075d819f"	0.1283
"00074d99f3"	"00075d8601"	0.1153
"00074d99f3"	"00075d8c6a"	0.1062
"00074d99f3"	"00075dfedc"	0.1847
"00074d99f3"	"00075e05f2"	0.0442
"00074d99f3"	"00075e0837"	0.1336
"00074d99f3"	"00075e092e"	0.0615
"00074d99f3"	"00075e0965"	-0.3561
"00074d99f3"	"00075e0bc8"	-0.0022
"00074d99f3"	"00075e0fbb"	-0.0178
"00074d9afd"	"00074d9c87"	-0.3186
"00074d9afd"	"00074d9e9e"	-0.4105
"00074d9afd"	"00074d9f30"	-0.0377
"00074d9afd"	"00074da036"	-0.2301
"00074d9afd"	"00074da082"	0.5374
"00074d9afd"	"00074da136"	0.136
"00074d9afd"	"00074da3ed"	-0.0891
"00074d9afd"	"00074da4ac"	-0.1475
"00074d9afd"	"00074da4b8"	-0.2984
"00074d9afd"	"00074da5e8"	-0.1598
"00074d9afd"	"00074da6b4"	-0.1968
"00074d9afd"	"00074daa3c"	-0.5064
"00074d9afd"	"00074daaf6"	-0.2711
"00074d9afd"	"00074dad20"	-0.198
"00074d9afd"	"00074db098"	-0.5172
"00074d9afd"	"00074db231"	-0.3302
"00074d9afd"	"00074db3a3"	-0.3331
"00074d9afd"	"00074db5d6"	-0.0519
"00074d9afd"	"00074db632"	-0.1524
"00074d9afd"	"00074db688"	-0.2283
"00074d9afd"	"00074db8a6"	-0.2752
"00074d9afd"	"00074dba19"	-0.5331
"00074d9afd"	"00074dbc2e"	-0.1912
"00074d9afd"	"00074dbe51"	0.0236

"00074d9afd"	"00074dbe5f"	-0.2973
"00074d9afd"	"00074dbf6d"	-0.6325
"00074d9afd"	"00074dc4a5"	0.3211
"00074d9afd"	"00074dc50c"	-0.228
"00074d9afd"	"00074dcdfa"	-0.0857
"00074d9afd"	"00074dcf5f"	-0.534
"00074d9afd"	"00074dd007"	-0.6415
"00074d9afd"	"00074dd163"	-0.2299
"00074d9afd"	"00074dd3df"	-0.178
"00074d9afd"	"00074dd577"	-0.1866
"00074d9afd"	"00074dd62e"	-0.274
"00074d9afd"	"00074dd73c"	0.1579
"00074d9afd"	"00074dda10"	-0.0729
"00074d9afd"	"00074ddab8"	-0.2686
"00074d9afd"	"00074ddd3d"	-0.359
"00074d9afd"	"00074ddf16"	-0.2452
"00074d9afd"	"00074ddfc1"	-0.2863
"00074d9afd"	"00074de21a"	-0.2935
"00074d9afd"	"00074de2a9"	-0.2296
"00074d9afd"	"00074de544"	-0.0427
"00074d9afd"	"00074de98a"	-0.1924
"00074d9afd"	"00074dea7e"	-0.3027
"00074d9afd"	"00074debd9"	-0.4383
"00074d9afd"	"00074deca3"	-0.1628
"00074d9afd"	"00074def43"	0.1078
"00074d9afd"	"00074def99"	-0.2161
"00074d9afd"	"00074ecdad"	-0.0318
"00074d9afd"	"00074ecf28"	0.287
"00074d9afd"	"00074ed1e1"	-0.2497
"00074d9afd"	"00074ed83b"	-0.1138
"00074d9afd"	"00074ee5e3"	-0.543
"00074d9afd"	"00074ee6e0"	-0.5765
"00074d9afd"	"00074eea3a"	0.0129
"00074d9afd"	"00074eff82"	-0.1425
"00074d9afd"	"00074f0477"	-0.4675
"00074d9afd"	"00074f08c3"	-0.5058
"00074d9afd"	"00074f1859"	-0.1221
"00074d9afd"	"00074f2268"	-0.4712
"00074d9afd"	"00074f28be"	-0.1809
"00074d9afd"	"00074f294b"	-0.1241
"00074d9afd"	"00074f2ddd"	-0.0382
"00074d9afd"	"00074f2e75"	0.0098
"00074d9afd"	"00074f3088"	0.1081
"00074d9afd"	"00074f5a1c"	-0.1347
"00074d9afd"	"00074f75b7"	-0.1858
"00074d9afd"	"00074f8cd9"	-0.1042
"00074d9afd"	"00074f96dc"	0.3216
"00074d9afd"	"00074fabaa"	-0.3465
"00074d9afd"	"00074facd9"	-0.3821
"00074d9afd"	"00074fae3c"	0.0012

"00074d9afd"	"00074fb0a8"	-0.47
"00074d9afd"	"00074fb4e4"	-0.447
"00074d9afd"	"00074fb7c2"	0.0314
"00074d9afd"	"00074fbd36"	-0.5041
"00074d9afd"	"00074fc27f"	-0.4128
"00074d9afd"	"00074fc31d"	-0.2504
"00074d9afd"	"00074fd569"	0.1429
"00074d9afd"	"00074fef15"	-0.0342
"00074d9afd"	"00074ff562"	-0.3736
"00074d9afd"	"00075007ca"	-0.453
"00074d9afd"	"0007500b86"	-0.2726
"00074d9afd"	"0007500d05"	-0.0594
"00074d9afd"	"0007500ee4"	-0.0921
"00074d9afd"	"0007500eee"	-0.4267
"00074d9afd"	"00075013dc"	-0.4684
"00074d9afd"	"000757b515"	-0.485
"00074d9afd"	"000757bc5a"	-0.1667
"00074d9afd"	"000757c320"	-0.3717
"00074d9afd"	"000757c9aa"	-0.55
"00074d9afd"	"000757ccbe"	0.1624
"00074d9afd"	"000757cfa9"	-0.3984
"00074d9afd"	"000757d390"	-0.5803
"00074d9afd"	"000757d393"	-0.165
"00074d9afd"	"000757d598"	-0.5889
"00074d9afd"	"000757d5a2"	-0.4583
"00074d9afd"	"000757d790"	-0.2544
"00074d9afd"	"000757e30c"	-0.6514
"00074d9afd"	"000757e4b0"	-0.2644
"00074d9afd"	"000757e7a0"	-0.0929
"00074d9afd"	"000757e8b3"	0.0446
"00074d9afd"	"000757f627"	-0.2647
"00074d9afd"	"000757f925"	-0.1971
"00074d9afd"	"000757fa08"	-0.0622
"00074d9afd"	"000757fe52"	0.0321
"00074d9afd"	"000758024a"	-0.3703
"00074d9afd"	"00075804bb"	-0.5655
"00074d9afd"	"00075a0c04"	0.1341
"00074d9afd"	"00075a3110"	-0.2957
"00074d9afd"	"00075a341a"	0.0535
"00074d9afd"	"00075a3dcf"	0.1285
"00074d9afd"	"00075a3e22"	-0.228
"00074d9afd"	"00075a48d8"	0.1118
"00074d9afd"	"00075a5cfb"	-0.5105
"00074d9afd"	"00075a6151"	-0.6053
"00074d9afd"	"00075a6708"	-0.3719
"00074d9afd"	"00075a7319"	-0.5965
"00074d9afd"	"00075a7723"	-0.1046
"00074d9afd"	"00075a778b"	-0.1153
"00074d9afd"	"00075a7b8e"	-0.3938
"00074d9afd"	"00075a7c79"	-0.0366

"00074d9afd"	"00075a81b6"	-0.1677
"00074d9afd"	"00075a82ac"	0.0573
"00074d9afd"	"00075a98e5"	-0.0326
"00074d9afd"	"00075b0d29"	-0.218
"00074d9afd"	"00075b102a"	-0.3831
"00074d9afd"	"00075b1074"	-0.078
"00074d9afd"	"00075b135d"	-0.2837
"00074d9afd"	"00075b138b"	-0.1369
"00074d9afd"	"00075b13a0"	-0.2529
"00074d9afd"	"00075b13bd"	0.078
"00074d9afd"	"00075b16a9"	-0.0623
"00074d9afd"	"00075b1a28"	-0.0046
"00074d9afd"	"00075b1a97"	0.0237
"00074d9afd"	"00075b1c7b"	-0.0225
"00074d9afd"	"00075b1d24"	-0.1387
"00074d9afd"	"00075b202b"	-0.1926
"00074d9afd"	"00075b22cb"	0.1755
"00074d9afd"	"00075b22da"	-0.1593
"00074d9afd"	"00075b2556"	-0.1912
"00074d9afd"	"00075b25de"	-0.3395
"00074d9afd"	"00075b260c"	-0.4247
"00074d9afd"	"00075b26f1"	-0.0825
"00074d9afd"	"00075b2920"	-0.3918
"00074d9afd"	"00075b2a64"	-0.6814
"00074d9afd"	"00075b2a9d"	-0.6211
"00074d9afd"	"00075b2b37"	-0.3201
"00074d9afd"	"00075b2cdd"	-0.0373
"00074d9afd"	"00075b3038"	-0.2256
"00074d9afd"	"00075b30fe"	-0.1428
"00074d9afd"	"00075b3362"	0.0463
"00074d9afd"	"00075b350a"	-0.2186
"00074d9afd"	"00075b350e"	-0.2001
"00074d9afd"	"00075b3651"	-0.1424
"00074d9afd"	"00075b38ca"	-0.1416
"00074d9afd"	"00075b39cc"	-0.1649
"00074d9afd"	"00075b3e1e"	-0.0793
"00074d9afd"	"00075b3e57"	0.2907
"00074d9afd"	"00075b4079"	-0.3017
"00074d9afd"	"00075b4150"	-0.4418
"00074d9afd"	"00075b4194"	-0.2081
"00074d9afd"	"00075b42d5"	-0.1364
"00074d9afd"	"00075b4424"	0.1282
"00074d9afd"	"00075b4470"	-0.1667
"00074d9afd"	"00075b47ed"	-0.082
"00074d9afd"	"00075b4850"	-0.1901
"00074d9afd"	"00075b4ca0"	-0.2768
"00074d9afd"	"00075b4d7f"	0.1096
"00074d9afd"	"00075b520f"	-0.0942
"00074d9afd"	"00075b525f"	-0.0945
"00074d9afd"	"00075b58f8"	-0.4979

"00074d9afd"	"00075b5bcc"	-0.1359
"00074d9afd"	"00075b5bfa"	-0.4739
"00074d9afd"	"00075b6339"	0.0828
"00074d9afd"	"00075b6658"	0.1059
"00074d9afd"	"00075b679a"	0.0825
"00074d9afd"	"00075b6cb7"	-0.5653
"00074d9afd"	"00075b6df8"	0.0782
"00074d9afd"	"00075b6ff6"	-0.2575
"00074d9afd"	"00075b70ee"	-0.3576
"00074d9afd"	"00075b7157"	-0.1388
"00074d9afd"	"00075b7225"	-0.1517
"00074d9afd"	"00075b7c89"	-0.0579
"00074d9afd"	"00075b9048"	-0.2804
"00074d9afd"	"00075d0801"	-0.3137
"00074d9afd"	"00075d1820"	-0.1961
"00074d9afd"	"00075d1f3d"	-0.2047
"00074d9afd"	"00075d2329"	-0.0994
"00074d9afd"	"00075d2b9b"	-0.4845
"00074d9afd"	"00075d3941"	0.2094
"00074d9afd"	"00075d3e96"	-0.1208
"00074d9afd"	"00075d4864"	-0.1652
"00074d9afd"	"00075d5961"	0.0973
"00074d9afd"	"00075d5a63"	0.1168
"00074d9afd"	"00075d6150"	0.0039
"00074d9afd"	"00075d67d0"	-0.2815
"00074d9afd"	"00075d67e2"	0.0772
"00074d9afd"	"00075d73fc"	0.2484
"00074d9afd"	"00075d7729"	-0.0785
"00074d9afd"	"00075d778c"	0.0144
"00074d9afd"	"00075d7b9e"	-0.0892
"00074d9afd"	"00075d7c8f"	0.0103
"00074d9afd"	"00075d804d"	-0.3363
"00074d9afd"	"00075d819f"	-0.5262
"00074d9afd"	"00075d8601"	0.0163
"00074d9afd"	"00075d8c6a"	-0.1054
"00074d9afd"	"00075dfedc"	0.0492
"00074d9afd"	"00075e05f2"	-0.3003
"00074d9afd"	"00075e0837"	-0.2976
"00074d9afd"	"00075e092e"	-0.0809
"00074d9afd"	"00075e0965"	-0.4724
"00074d9afd"	"00075e0bc8"	-0.152
"00074d9afd"	"00075e0fbb"	-0.222
"00074d9c87"	"00074d9e9e"	-0.0262
"00074d9c87"	"00074d9f30"	-0.1451
"00074d9c87"	"00074da036"	0.6452
"00074d9c87"	"00074da082"	0.0765
"00074d9c87"	"00074da136"	0.2327
"00074d9c87"	"00074da3ed"	0.256
"00074d9c87"	"00074da4ac"	0.1832
"00074d9c87"	"00074da4b8"	0.1005

"00074d9c87"	"00074da5e8"	0.3858
"00074d9c87"	"00074da6b4"	0.3156
"00074d9c87"	"00074daa3c"	−0.1383
"00074d9c87"	"00074daaf6"	−0.1001
"00074d9c87"	"00074dad20"	0.0064
"00074d9c87"	"00074db098"	0.0877
"00074d9c87"	"00074db231"	0.4295
"00074d9c87"	"00074db3a3"	0.1887
"00074d9c87"	"00074db5d6"	−0.0188
"00074d9c87"	"00074db632"	0.0572
"00074d9c87"	"00074db688"	0.3069
"00074d9c87"	"00074db8a6"	−0.1658
"00074d9c87"	"00074dba19"	0.4139
"00074d9c87"	"00074dbc2e"	−0.1444
"00074d9c87"	"00074dbe51"	0.3277
"00074d9c87"	"00074dbe5f"	−0.2528
"00074d9c87"	"00074dbf6d"	0.1055
"00074d9c87"	"00074dc4a5"	−0.1483
"00074d9c87"	"00074dc50c"	0.0747
"00074d9c87"	"00074dcdfa"	−0.0316
"00074d9c87"	"00074dcf5f"	0.1389
"00074d9c87"	"00074dd007"	0.1614
"00074d9c87"	"00074dd163"	−0.1528
"00074d9c87"	"00074dd3df"	0.1743
"00074d9c87"	"00074dd577"	0.4163
"00074d9c87"	"00074dd62e"	0.0192
"00074d9c87"	"00074dd73c"	0.0368
"00074d9c87"	"00074dda10"	−0.0818
"00074d9c87"	"00074ddab8"	0.1421
"00074d9c87"	"00074ddd3d"	0.3113
"00074d9c87"	"00074ddf16"	−0.0659
"00074d9c87"	"00074ddfc1"	0.1413
"00074d9c87"	"00074de21a"	−0.225
"00074d9c87"	"00074de2a9"	0.1921
"00074d9c87"	"00074de544"	0.125
"00074d9c87"	"00074de98a"	0.0272
"00074d9c87"	"00074dea7e"	0.1024
"00074d9c87"	"00074debd9"	−0.0744
"00074d9c87"	"00074deca3"	−0.1561
"00074d9c87"	"00074def43"	−0.0942
"00074d9c87"	"00074def99"	0.0359
"00074d9c87"	"00074ecdad"	−0.1417
"00074d9c87"	"00074ecf28"	0.0648
"00074d9c87"	"00074ed1e1"	−0.0176
"00074d9c87"	"00074ed83b"	0.0264
"00074d9c87"	"00074ee5e3"	−0.0894
"00074d9c87"	"00074ee6e0"	0.0493
"00074d9c87"	"00074eea3a"	0.1808
"00074d9c87"	"00074eff82"	−0.1623
"00074d9c87"	"00074f0477"	0.0196

"00074d9c87"	"00074f08c3"	-0.2408
"00074d9c87"	"00074f1859"	0.1065
"00074d9c87"	"00074f2268"	0.2279
"00074d9c87"	"00074f28be"	0.1218
"00074d9c87"	"00074f294b"	0.2257
"00074d9c87"	"00074f2ddd"	0.3588
"00074d9c87"	"00074f2e75"	0.1184
"00074d9c87"	"00074f3088"	0.2234
"00074d9c87"	"00074f5a1c"	0.0796
"00074d9c87"	"00074f75b7"	-0.043
"00074d9c87"	"00074f8cd9"	0.0434
"00074d9c87"	"00074f96dc"	-0.3433
"00074d9c87"	"00074fabaa"	-0.0362
"00074d9c87"	"00074facd9"	0.0748
"00074d9c87"	"00074fae3c"	-0.0236
"00074d9c87"	"00074fb0a8"	0.1165
"00074d9c87"	"00074fb4e4"	-0.2003
"00074d9c87"	"00074fb7c2"	0.3194
"00074d9c87"	"00074fbd36"	0.3174
"00074d9c87"	"00074fc27f"	0.1685
"00074d9c87"	"00074fc31d"	0.1921
"00074d9c87"	"00074fd569"	-0.0452
"00074d9c87"	"00074fef15"	-0.132
"00074d9c87"	"00074ff562"	-0.2738
"00074d9c87"	"00075007ca"	-0.0128
"00074d9c87"	"0007500b86"	0.047
"00074d9c87"	"0007500d05"	-0.0683
"00074d9c87"	"0007500ee4"	0.2013
"00074d9c87"	"0007500eee"	-0.1385
"00074d9c87"	"00075013dc"	-0.1118
"00074d9c87"	"000757b515"	0.5121
"00074d9c87"	"000757bc5a"	0.1747
"00074d9c87"	"000757c320"	-0.0166
"00074d9c87"	"000757c9aa"	0.0927
"00074d9c87"	"000757ccbe"	0.2663
"00074d9c87"	"000757cfa9"	0.2598
"00074d9c87"	"000757d390"	-0.1087
"00074d9c87"	"000757d393"	0.2544
"00074d9c87"	"000757d598"	0.1239
"00074d9c87"	"000757d5a2"	0.33
"00074d9c87"	"000757d790"	0.0792
"00074d9c87"	"000757e30c"	0.1477
"00074d9c87"	"000757e4b0"	0.2553
"00074d9c87"	"000757e7a0"	0.0999
"00074d9c87"	"000757e8b3"	0.258
"00074d9c87"	"000757f627"	0.0489
"00074d9c87"	"000757f925"	0.3424
"00074d9c87"	"000757fa08"	0.2637
"00074d9c87"	"000757fe52"	0.0865
"00074d9c87"	"000758024a"	-0.0368

"00074d9c87"	"00075804bb"	0.1586
"00074d9c87"	"00075a0c04"	0.1511
"00074d9c87"	"00075a3110"	0.4038
"00074d9c87"	"00075a341a"	0.1546
"00074d9c87"	"00075a3dcf"	0.1914
"00074d9c87"	"00075a3e22"	0.2724
"00074d9c87"	"00075a48d8"	0.1339
"00074d9c87"	"00075a5cfb"	-0.0297
"00074d9c87"	"00075a6151"	0.0763
"00074d9c87"	"00075a6708"	0.3025
"00074d9c87"	"00075a7319"	-0.0589
"00074d9c87"	"00075a7723"	0.0507
"00074d9c87"	"00075a778b"	0.0802
"00074d9c87"	"00075a7b8e"	0.0917
"00074d9c87"	"00075a7c79"	0.1026
"00074d9c87"	"00075a81b6"	-0.0392
"00074d9c87"	"00075a82ac"	0.211
"00074d9c87"	"00075a98e5"	-0.085
"00074d9c87"	"00075b0d29"	0.2797
"00074d9c87"	"00075b102a"	0.0867
"00074d9c87"	"00075b1074"	0.2595
"00074d9c87"	"00075b135d"	0.1774
"00074d9c87"	"00075b138b"	0.0766
"00074d9c87"	"00075b13a0"	-0.0187
"00074d9c87"	"00075b13bd"	0.0897
"00074d9c87"	"00075b16a9"	0.2019
"00074d9c87"	"00075b1a28"	0.0484
"00074d9c87"	"00075b1a97"	-0.0404
"00074d9c87"	"00075b1c7b"	-0.2307
"00074d9c87"	"00075b1d24"	0.0874
"00074d9c87"	"00075b202b"	0.0596
"00074d9c87"	"00075b22cb"	0.0379
"00074d9c87"	"00075b22da"	0.2575
"00074d9c87"	"00075b2556"	0.2677
"00074d9c87"	"00075b25de"	0.2036
"00074d9c87"	"00075b260c"	0.1893
"00074d9c87"	"00075b26f1"	-0.1758
"00074d9c87"	"00075b2920"	0.0589
"00074d9c87"	"00075b2a64"	-0.0888
"00074d9c87"	"00075b2a9d"	0.2534
"00074d9c87"	"00075b2b37"	-0.0887
"00074d9c87"	"00075b2cdd"	-0.0087
"00074d9c87"	"00075b3038"	0.1588
"00074d9c87"	"00075b30fe"	0.2855
"00074d9c87"	"00075b3362"	-0.288
"00074d9c87"	"00075b350a"	0.1664
"00074d9c87"	"00075b350e"	-0.0189
"00074d9c87"	"00075b3651"	-0.1741
"00074d9c87"	"00075b38ca"	-0.1149
"00074d9c87"	"00075b39cc"	0.2358

"00074d9c87"	"00075b3e1e"	0.026
"00074d9c87"	"00075b3e57"	0.1824
"00074d9c87"	"00075b4079"	-0.017
"00074d9c87"	"00075b4150"	0.1119
"00074d9c87"	"00075b4194"	0.1393
"00074d9c87"	"00075b42d5"	0.0095
"00074d9c87"	"00075b4424"	-0.0267
"00074d9c87"	"00075b4470"	0.2441
"00074d9c87"	"00075b47ed"	0.2161
"00074d9c87"	"00075b4850"	0.1151
"00074d9c87"	"00075b4ca0"	0.2717
"00074d9c87"	"00075b4d7f"	0.2917
"00074d9c87"	"00075b520f"	0.2026
"00074d9c87"	"00075b525f"	-0.0905
"00074d9c87"	"00075b58f8"	0.1421
"00074d9c87"	"00075b5bcc"	-0.1022
"00074d9c87"	"00075b5bfa"	-0.202
"00074d9c87"	"00075b6339"	0.273
"00074d9c87"	"00075b6658"	-0.2042
"00074d9c87"	"00075b679a"	0.2558
"00074d9c87"	"00075b6cb7"	-0.0791
"00074d9c87"	"00075b6df8"	0.1775
"00074d9c87"	"00075b6ff6"	0.2551
"00074d9c87"	"00075b70ee"	0.0265
"00074d9c87"	"00075b7157"	0.1849
"00074d9c87"	"00075b7225"	0.1266
"00074d9c87"	"00075b7c89"	0.4908
"00074d9c87"	"00075b9048"	0.1989
"00074d9c87"	"00075d0801"	0.2715
"00074d9c87"	"00075d1820"	0.0164
"00074d9c87"	"00075d1f3d"	-0.0096
"00074d9c87"	"00075d2329"	0.2444
"00074d9c87"	"00075d2b9b"	-0.0998
"00074d9c87"	"00075d3941"	-0.0592
"00074d9c87"	"00075d3e96"	0.1029
"00074d9c87"	"00075d4864"	0.1122
"00074d9c87"	"00075d5961"	0.1201
"00074d9c87"	"00075d5a63"	0.145
"00074d9c87"	"00075d6150"	-0.2489
"00074d9c87"	"00075d67d0"	-0.0493
"00074d9c87"	"00075d67e2"	-0.0465
"00074d9c87"	"00075d73fc"	0.0924
"00074d9c87"	"00075d7729"	0.0367
"00074d9c87"	"00075d778c"	-0.376
"00074d9c87"	"00075d7b9e"	0.0887
"00074d9c87"	"00075d7c8f"	0.3649
"00074d9c87"	"00075d804d"	0.0859
"00074d9c87"	"00075d819f"	0.2451
"00074d9c87"	"00075d8601"	-0.004
"00074d9c87"	"00075d8c6a"	0.0871

"00074d9c87"	"00075dfedc"	0.1612
"00074d9c87"	"00075e05f2"	-0.097
"00074d9c87"	"00075e0837"	0.1295
"00074d9c87"	"00075e092e"	0.2065
"00074d9c87"	"00075e0965"	-0.1448
"00074d9c87"	"00075e0bc8"	0.283
"00074d9c87"	"00075e0fbb"	0.2323
"00074d9e9e"	"00074d9f30"	-0.2266
"00074d9e9e"	"00074da036"	-0.0345
"00074d9e9e"	"00074da082"	-0.3572
"00074d9e9e"	"00074da136"	-0.2724
"00074d9e9e"	"00074da3ed"	-0.1022
"00074d9e9e"	"00074da4ac"	-0.0105
"00074d9e9e"	"00074da4b8"	-0.4498
"00074d9e9e"	"00074da5e8"	-0.3189
"00074d9e9e"	"00074da6b4"	-0.259
"00074d9e9e"	"00074daa3c"	-0.4749
"00074d9e9e"	"00074daaf6"	-0.15
"00074d9e9e"	"00074dad20"	0.1557
"00074d9e9e"	"00074db098"	-0.2384
"00074d9e9e"	"00074db231"	-0.0689
"00074d9e9e"	"00074db3a3"	-0.3897
"00074d9e9e"	"00074db5d6"	-0.2672
"00074d9e9e"	"00074db632"	-0.5008
"00074d9e9e"	"00074db688"	-0.549
"00074d9e9e"	"00074db8a6"	0.0845
"00074d9e9e"	"00074dba19"	-0.2876
"00074d9e9e"	"00074dbc2e"	-0.0504
"00074d9e9e"	"00074dbe51"	0.0866
"00074d9e9e"	"00074dbe5f"	0.2526
"00074d9e9e"	"00074dbf6d"	-0.4931
"00074d9e9e"	"00074dc4a5"	-0.5838
"00074d9e9e"	"00074dc50c"	-0.4133
"00074d9e9e"	"00074dcdfa"	-0.278
"00074d9e9e"	"00074dcf5f"	-0.3462
"00074d9e9e"	"00074dd007"	-0.2045
"00074d9e9e"	"00074dd163"	0.0628
"00074d9e9e"	"00074dd3df"	-0.267
"00074d9e9e"	"00074dd577"	-0.0136
"00074d9e9e"	"00074dd62e"	-0.3123
"00074d9e9e"	"00074dd73c"	-0.07
"00074d9e9e"	"00074dda10"	-0.2876
"00074d9e9e"	"00074ddab8"	-0.2581
"00074d9e9e"	"00074ddd3d"	-0.3702
"00074d9e9e"	"00074ddf16"	0.145
"00074d9e9e"	"00074ddfc1"	0.1644
"00074d9e9e"	"00074de21a"	-0.4966
"00074d9e9e"	"00074de2a9"	-0.3962
"00074d9e9e"	"00074de544"	-0.1583
"00074d9e9e"	"00074de98a"	-0.2221

"00074d9e9e"	"00074dea7e"	−0.1156
"00074d9e9e"	"00074debd9"	−0.0869
"00074d9e9e"	"00074deca3"	−0.178
"00074d9e9e"	"00074def43"	−0.1025
"00074d9e9e"	"00074def99"	−0.3865
"00074d9e9e"	"00074ecdad"	−0.2962
"00074d9e9e"	"00074ecf28"	−0.176
"00074d9e9e"	"00074ed1e1"	−0.0401
"00074d9e9e"	"00074ed83b"	−0.3943
"00074d9e9e"	"00074ee5e3"	0.1478
"00074d9e9e"	"00074ee6e0"	−0.1558
"00074d9e9e"	"00074eea3a"	−0.2025
"00074d9e9e"	"00074eff82"	−0.1278
"00074d9e9e"	"00074f0477"	−0.0076
"00074d9e9e"	"00074f08c3"	−0.6866
"00074d9e9e"	"00074f1859"	−0.2391
"00074d9e9e"	"00074f2268"	−0.4914
"00074d9e9e"	"00074f28be"	−0.4033
"00074d9e9e"	"00074f294b"	−0.1629
"00074d9e9e"	"00074f2ddd"	−0.4332
"00074d9e9e"	"00074f2e75"	−0.2791
"00074d9e9e"	"00074f3088"	−0.0367
"00074d9e9e"	"00074f5a1c"	−0.1752
"00074d9e9e"	"00074f75b7"	−0.0362
"00074d9e9e"	"00074f8cd9"	0.2306
"00074d9e9e"	"00074f96dc"	−0.5526
"00074d9e9e"	"00074fabaa"	−0.5455
"00074d9e9e"	"00074facd9"	−0.0254
"00074d9e9e"	"00074fae3c"	−0.3275
"00074d9e9e"	"00074fb0a8"	−0.297
"00074d9e9e"	"00074fb4e4"	−0.2961
"00074d9e9e"	"00074fb7c2"	−0.0971
"00074d9e9e"	"00074fbd36"	−0.3773
"00074d9e9e"	"00074fc27f"	−0.2905
"00074d9e9e"	"00074fc31d"	−0.255
"00074d9e9e"	"00074fd569"	−0.187
"00074d9e9e"	"00074fef15"	−0.1655
"00074d9e9e"	"00074ff562"	−0.2139
"00074d9e9e"	"00075007ca"	−0.2837
"00074d9e9e"	"0007500b86"	0.0313
"00074d9e9e"	"0007500d05"	0.3358
"00074d9e9e"	"0007500ee4"	−0.0243
"00074d9e9e"	"0007500eee"	0.1735
"00074d9e9e"	"00075013dc"	−0.6594
"00074d9e9e"	"000757b515"	−0.0821
"00074d9e9e"	"000757bc5a"	−0.2627
"00074d9e9e"	"000757c320"	−0.4277
"00074d9e9e"	"000757c9aa"	−0.3162
"00074d9e9e"	"000757ccbe"	−0.2096
"00074d9e9e"	"000757cfa9"	−0.2398

"00074d9e9e"	"000757d390"	−0.3089
"00074d9e9e"	"000757d393"	0.1836
"00074d9e9e"	"000757d598"	−0.3775
"00074d9e9e"	"000757d5a2"	−0.5188
"00074d9e9e"	"000757d790"	0.1205
"00074d9e9e"	"000757e30c"	−0.4275
"00074d9e9e"	"000757e4b0"	−0.257
"00074d9e9e"	"000757e7a0"	−0.2467
"00074d9e9e"	"000757e8b3"	−0.1434
"00074d9e9e"	"000757f627"	−0.2072
"00074d9e9e"	"000757f925"	−0.0371
"00074d9e9e"	"000757fa08"	−0.0978
"00074d9e9e"	"000757fe52"	−0.0118
"00074d9e9e"	"000758024a"	−0.5211
"00074d9e9e"	"00075804bb"	−0.4541
"00074d9e9e"	"00075a0c04"	−0.0401
"00074d9e9e"	"00075a3110"	−0.1926
"00074d9e9e"	"00075a341a"	−0.166
"00074d9e9e"	"00075a3dcf"	0.1028
"00074d9e9e"	"00075a3e22"	−0.174
"00074d9e9e"	"00075a48d8"	0.0369
"00074d9e9e"	"00075a5cfb"	−0.6505
"00074d9e9e"	"00075a6151"	−0.001
"00074d9e9e"	"00075a6708"	−0.2484
"00074d9e9e"	"00075a7319"	−0.3225
"00074d9e9e"	"00075a7723"	−0.2023
"00074d9e9e"	"00075a778b"	−0.2796
"00074d9e9e"	"00075a7b8e"	−0.293
"00074d9e9e"	"00075a7c79"	−0.2199
"00074d9e9e"	"00075a81b6"	−0.1438
"00074d9e9e"	"00075a82ac"	−0.0342
"00074d9e9e"	"00075a98e5"	−0.1673
"00074d9e9e"	"00075b0d29"	−0.2354
"00074d9e9e"	"00075b102a"	−0.2013
"00074d9e9e"	"00075b1074"	−0.3963
"00074d9e9e"	"00075b135d"	−0.4161
"00074d9e9e"	"00075b138b"	−0.2444
"00074d9e9e"	"00075b13a0"	−0.0307
"00074d9e9e"	"00075b13bd"	−0.2873
"00074d9e9e"	"00075b16a9"	−0.0081
"00074d9e9e"	"00075b1a28"	−0.5236
"00074d9e9e"	"00075b1a97"	−0.3338
"00074d9e9e"	"00075b1c7b"	−0.2238
"00074d9e9e"	"00075b1d24"	−0.3078
"00074d9e9e"	"00075b202b"	−0.4597
"00074d9e9e"	"00075b22cb"	−0.253
"00074d9e9e"	"00075b22da"	−0.2466
"00074d9e9e"	"00075b2556"	−0.2279
"00074d9e9e"	"00075b25de"	0.2068
"00074d9e9e"	"00075b260c"	−0.3494

"00074d9e9e"	"00075b26f1"	-0.4819
"00074d9e9e"	"00075b2920"	-0.4529
"00074d9e9e"	"00075b2a64"	-0.6476
"00074d9e9e"	"00075b2a9d"	-0.2568
"00074d9e9e"	"00075b2b37"	-0.1568
"00074d9e9e"	"00075b2cdd"	-0.2345
"00074d9e9e"	"00075b3038"	0.215
"00074d9e9e"	"00075b30fe"	-0.2523
"00074d9e9e"	"00075b3362"	-0.4841
"00074d9e9e"	"00075b350a"	-0.3182
"00074d9e9e"	"00075b350e"	-0.4257
"00074d9e9e"	"00075b3651"	-0.4663
"00074d9e9e"	"00075b38ca"	-0.2336
"00074d9e9e"	"00075b39cc"	0.1534
"00074d9e9e"	"00075b3e1e"	-0.0779
"00074d9e9e"	"00075b3e57"	-0.1375
"00074d9e9e"	"00075b4079"	-0.3018
"00074d9e9e"	"00075b4150"	-0.4766
"00074d9e9e"	"00075b4194"	-0.2857
"00074d9e9e"	"00075b42d5"	-0.0529
"00074d9e9e"	"00075b4424"	-0.6155
"00074d9e9e"	"00075b4470"	-0.1872
"00074d9e9e"	"00075b47ed"	0.1233
"00074d9e9e"	"00075b4850"	-0.315
"00074d9e9e"	"00075b4ca0"	-0.6187
"00074d9e9e"	"00075b4d7f"	0.1213
"00074d9e9e"	"00075b520f"	-0.0605
"00074d9e9e"	"00075b525f"	-0.3889
"00074d9e9e"	"00075b58f8"	-0.2541
"00074d9e9e"	"00075b5bcc"	-0.3949
"00074d9e9e"	"00075b5bfa"	0.0712
"00074d9e9e"	"00075b6339"	-0.0213
"00074d9e9e"	"00075b6658"	-0.3323
"00074d9e9e"	"00075b679a"	-0.1278
"00074d9e9e"	"00075b6cb7"	-0.4408
"00074d9e9e"	"00075b6df8"	0.1644
"00074d9e9e"	"00075b6ff6"	-0.2555
"00074d9e9e"	"00075b70ee"	-0.2382
"00074d9e9e"	"00075b7157"	-0.0391
"00074d9e9e"	"00075b7225"	-0.2924
"00074d9e9e"	"00075b7c89"	-0.2477
"00074d9e9e"	"00075b9048"	-0.177
"00074d9e9e"	"00075d0801"	0.1781
"00074d9e9e"	"00075d1820"	-0.3034
"00074d9e9e"	"00075d1f3d"	-0.6003
"00074d9e9e"	"00075d2329"	0.0997
"00074d9e9e"	"00075d2b9b"	-0.5946
"00074d9e9e"	"00075d3941"	-0.5958
"00074d9e9e"	"00075d3e96"	-0.4001
"00074d9e9e"	"00075d4864"	-0.2655

"00074d9e9e"	"00075d5961"	−0.0514
"00074d9e9e"	"00075d5a63"	−0.1881
"00074d9e9e"	"00075d6150"	−0.3371
"00074d9e9e"	"00075d67d0"	−0.1442
"00074d9e9e"	"00075d67e2"	−0.0365
"00074d9e9e"	"00075d73fc"	−0.2365
"00074d9e9e"	"00075d7729"	−0.3849
"00074d9e9e"	"00075d778c"	−0.1867
"00074d9e9e"	"00075d7b9e"	−0.0421
"00074d9e9e"	"00075d7c8f"	−0.1616
"00074d9e9e"	"00075d804d"	−0.0864
"00074d9e9e"	"00075d819f"	−0.3029
"00074d9e9e"	"00075d8601"	−0.0677
"00074d9e9e"	"00075d8c6a"	0.1861
"00074d9e9e"	"00075dfedc"	−0.0966
"00074d9e9e"	"00075e05f2"	−0.1405
"00074d9e9e"	"00075e0837"	−0.2129
"00074d9e9e"	"00075e092e"	−0.2607
"00074d9e9e"	"00075e0965"	−0.6449
"00074d9e9e"	"00075e0bc8"	−0.0964
"00074d9e9e"	"00075e0fbb"	−0.2782
"00074d9f30"	"00074da036"	−0.1053
"00074d9f30"	"00074da082"	−0.0802
"00074d9f30"	"00074da136"	−0.3437
"00074d9f30"	"00074da3ed"	0.0455
"00074d9f30"	"00074da4ac"	0.0494
"00074d9f30"	"00074da4b8"	0.1683
"00074d9f30"	"00074da5e8"	−0.0512
"00074d9f30"	"00074da6b4"	−0.2067
"00074d9f30"	"00074daa3c"	−0.1549
"00074d9f30"	"00074daaf6"	0.1103
"00074d9f30"	"00074dad20"	−0.3289
"00074d9f30"	"00074db098"	−0.0412
"00074d9f30"	"00074db231"	−0.2906
"00074d9f30"	"00074db3a3"	−0.0076
"00074d9f30"	"00074db5d6"	−0.0414
"00074d9f30"	"00074db632"	0.0903
"00074d9f30"	"00074db688"	−0.1651
"00074d9f30"	"00074db8a6"	−0.0118
"00074d9f30"	"00074dba19"	−0.2938
"00074d9f30"	"00074dbc2e"	−0.1906
"00074d9f30"	"00074dbe51"	0.0196
"00074d9f30"	"00074dbe5f"	−0.4294
"00074d9f30"	"00074dbf6d"	−0.1236
"00074d9f30"	"00074dc4a5"	−0.173
"00074d9f30"	"00074dc50c"	−0.3733
"00074d9f30"	"00074dcdfa"	−0.2312
"00074d9f30"	"00074dcf5f"	−0.0221
"00074d9f30"	"00074dd007"	0.0071
"00074d9f30"	"00074dd163"	−0.099

"00074d9f30"	"00074dd3df"	-0.2415
"00074d9f30"	"00074dd577"	-0.1089
"00074d9f30"	"00074dd62e"	0.194
"00074d9f30"	"00074dd73c"	0.1812
"00074d9f30"	"00074dda10"	-0.2757
"00074d9f30"	"00074ddab8"	-0.2327
"00074d9f30"	"00074ddd3d"	0.1428
"00074d9f30"	"00074ddf16"	-0.0701
"00074d9f30"	"00074ddfc1"	-0.1345
"00074d9f30"	"00074de21a"	-0.125
"00074d9f30"	"00074de2a9"	0.1332
"00074d9f30"	"00074de544"	-0.3525
"00074d9f30"	"00074de98a"	-0.3511
"00074d9f30"	"00074dea7e"	-0.1744
"00074d9f30"	"00074debd9"	-0.1668
"00074d9f30"	"00074deca3"	-0.2183
"00074d9f30"	"00074def43"	0.1267
"00074d9f30"	"00074def99"	-0.1372
"00074d9f30"	"00074ecdad"	-0.2369
"00074d9f30"	"00074ecf28"	0.059
"00074d9f30"	"00074ed1e1"	-0.3926
"00074d9f30"	"00074ed83b"	0.1419
"00074d9f30"	"00074ee5e3"	0.0028
"00074d9f30"	"00074ee6e0"	-0.1329
"00074d9f30"	"00074eea3a"	-0.2078
"00074d9f30"	"00074eff82"	0.0723
"00074d9f30"	"00074f0477"	-0.2992
"00074d9f30"	"00074f08c3"	-0.1517
"00074d9f30"	"00074f1859"	-0.2068
"00074d9f30"	"00074f2268"	0.0452
"00074d9f30"	"00074f28be"	-0.2292
"00074d9f30"	"00074f294b"	0.0389
"00074d9f30"	"00074f2ddd"	-0.0565
"00074d9f30"	"00074f2e75"	-0.2299
"00074d9f30"	"00074f3088"	0.0182
"00074d9f30"	"00074f5a1c"	-0.1989
"00074d9f30"	"00074f75b7"	0.0316
"00074d9f30"	"00074f8cd9"	-0.2333
"00074d9f30"	"00074f96dc"	-0.1158
"00074d9f30"	"00074fabaa"	-0.2249
"00074d9f30"	"00074facd9"	-0.1186
"00074d9f30"	"00074fae3c"	-0.1485
"00074d9f30"	"00074fb0a8"	-0.3051
"00074d9f30"	"00074fb4e4"	-0.1657
"00074d9f30"	"00074fb7c2"	-0.2618
"00074d9f30"	"00074fbd36"	-0.1051
"00074d9f30"	"00074fc27f"	0.1496
"00074d9f30"	"00074fc31d"	-0.2567
"00074d9f30"	"00074fd569"	-0.02
"00074d9f30"	"00074fef15"	0.0621

"00074d9f30"	"00074ff562"	-0.3666
"00074d9f30"	"00075007ca"	-0.1634
"00074d9f30"	"0007500b86"	0.0474
"00074d9f30"	"0007500d05"	-0.0084
"00074d9f30"	"0007500ee4"	0.2234
"00074d9f30"	"0007500eee"	-0.295
"00074d9f30"	"00075013dc"	-0.3034
"00074d9f30"	"000757b515"	-0.0076
"00074d9f30"	"000757bc5a"	-0.1453
"00074d9f30"	"000757c320"	-0.0886
"00074d9f30"	"000757c9aa"	0.0775
"00074d9f30"	"000757ccbe"	-0.168
"00074d9f30"	"000757cfa9"	-0.221
"00074d9f30"	"000757d390"	-0.0584
"00074d9f30"	"000757d393"	-0.0582
"00074d9f30"	"000757d598"	0.1373
"00074d9f30"	"000757d5a2"	-0.0253
"00074d9f30"	"000757d790"	-0.0312
"00074d9f30"	"000757e30c"	0.0724
"00074d9f30"	"000757e4b0"	-0.0097
"00074d9f30"	"000757e7a0"	0.2935
"00074d9f30"	"000757e8b3"	0.0909
"00074d9f30"	"000757f627"	-0.0941
"00074d9f30"	"000757f925"	-0.0039
"00074d9f30"	"000757fa08"	-0.229
"00074d9f30"	"000757fe52"	0.0917
"00074d9f30"	"000758024a"	-0.0448
"00074d9f30"	"00075804bb"	-0.1045
"00074d9f30"	"00075a0c04"	-0.347
"00074d9f30"	"00075a3110"	-0.0926
"00074d9f30"	"00075a341a"	0.1896
"00074d9f30"	"00075a3dcf"	-0.1339
"00074d9f30"	"00075a3e22"	-0.2853
"00074d9f30"	"00075a48d8"	-0.0825
"00074d9f30"	"00075a5cfb"	0.1482
"00074d9f30"	"00075a6151"	-0.1826
"00074d9f30"	"00075a6708"	-0.0799
"00074d9f30"	"00075a7319"	-0.1144
"00074d9f30"	"00075a7723"	-0.1422
"00074d9f30"	"00075a778b"	-0.1899
"00074d9f30"	"00075a7b8e"	-0.2985
"00074d9f30"	"00075a7c79"	0.0018
"00074d9f30"	"00075a81b6"	-0.1634
"00074d9f30"	"00075a82ac"	-0.1567
"00074d9f30"	"00075a98e5"	-0.3035
"00074d9f30"	"00075b0d29"	-0.2373
"00074d9f30"	"00075b102a"	-0.2442
"00074d9f30"	"00075b1074"	-0.1792
"00074d9f30"	"00075b135d"	-0.4286
"00074d9f30"	"00075b138b"	-0.1346

"00074d9f30"	"00075b13a0"	-0.1032
"00074d9f30"	"00075b13bd"	-0.1799
"00074d9f30"	"00075b16a9"	-0.1177
"00074d9f30"	"00075b1a28"	-0.1096
"00074d9f30"	"00075b1a97"	-0.0866
"00074d9f30"	"00075b1c7b"	-0.152
"00074d9f30"	"00075b1d24"	0.1855
"00074d9f30"	"00075b202b"	-0.1747
"00074d9f30"	"00075b22cb"	-0.1516
"00074d9f30"	"00075b22da"	0.1571
"00074d9f30"	"00075b2556"	0.1544
"00074d9f30"	"00075b25de"	0.1102
"00074d9f30"	"00075b260c"	-0.076
"00074d9f30"	"00075b26f1"	-0.0202
"00074d9f30"	"00075b2920"	0.0874
"00074d9f30"	"00075b2a64"	-0.1215
"00074d9f30"	"00075b2a9d"	-0.3398
"00074d9f30"	"00075b2b37"	0.2198
"00074d9f30"	"00075b2cdd"	-0.2869
"00074d9f30"	"00075b3038"	-0.0589
"00074d9f30"	"00075b30fe"	0.0822
"00074d9f30"	"00075b3362"	0.2997
"00074d9f30"	"00075b350a"	-0.0592
"00074d9f30"	"00075b350e"	-0.3249
"00074d9f30"	"00075b3651"	0.1092
"00074d9f30"	"00075b38ca"	-0.3152
"00074d9f30"	"00075b39cc"	-0.2369
"00074d9f30"	"00075b3e1e"	-0.2366
"00074d9f30"	"00075b3e57"	0.3215
"00074d9f30"	"00075b4079"	-0.0701
"00074d9f30"	"00075b4150"	0.0433
"00074d9f30"	"00075b4194"	-0.1489
"00074d9f30"	"00075b42d5"	-0.185
"00074d9f30"	"00075b4424"	0.2097
"00074d9f30"	"00075b4470"	-0.1609
"00074d9f30"	"00075b47ed"	-0.3018
"00074d9f30"	"00075b4850"	-0.0728
"00074d9f30"	"00075b4ca0"	-0.3048
"00074d9f30"	"00075b4d7f"	0.0532
"00074d9f30"	"00075b520f"	-0.1983
"00074d9f30"	"00075b525f"	0.3881
"00074d9f30"	"00075b58f8"	0.0226
"00074d9f30"	"00075b5bcc"	0.1233
"00074d9f30"	"00075b5bfa"	0.0493
"00074d9f30"	"00075b6339"	0.147
"00074d9f30"	"00075b6658"	0.1307
"00074d9f30"	"00075b679a"	0.2032
"00074d9f30"	"00075b6cb7"	-0.1841
"00074d9f30"	"00075b6df8"	-0.0374
"00074d9f30"	"00075b6ff6"	0.2577

"00074d9f30"	"00075b70ee"	-0.1607
"00074d9f30"	"00075b7157"	0.1763
"00074d9f30"	"00075b7225"	-0.2758
"00074d9f30"	"00075b7c89"	-0.4029
"00074d9f30"	"00075b9048"	-0.0457
"00074d9f30"	"00075d0801"	-0.0059
"00074d9f30"	"00075d1820"	-0.0457
"00074d9f30"	"00075d1f3d"	-0.2106
"00074d9f30"	"00075d2329"	-0.3315
"00074d9f30"	"00075d2b9b"	-0.133
"00074d9f30"	"00075d3941"	-0.415
"00074d9f30"	"00075d3e96"	0.1332
"00074d9f30"	"00075d4864"	-0.078
"00074d9f30"	"00075d5961"	-0.2692
"00074d9f30"	"00075d5a63"	0.2763
"00074d9f30"	"00075d6150"	-0.1851
"00074d9f30"	"00075d67d0"	0.0687
"00074d9f30"	"00075d67e2"	-0.0779
"00074d9f30"	"00075d73fc"	-0.1803
"00074d9f30"	"00075d7729"	0.1866
"00074d9f30"	"00075d778c"	0.4053
"00074d9f30"	"00075d7b9e"	0.2514
"00074d9f30"	"00075d7c8f"	-0.2094
"00074d9f30"	"00075d804d"	0.1453
"00074d9f30"	"00075d819f"	-0.3144
"00074d9f30"	"00075d8601"	-0.1793
"00074d9f30"	"00075d8c6a"	-0.1736
"00074d9f30"	"00075dfedc"	-0.1978
"00074d9f30"	"00075e05f2"	-0.0812
"00074d9f30"	"00075e0837"	-0.2304
"00074d9f30"	"00075e092e"	0.0019
"00074d9f30"	"00075e0965"	-0.0501
"00074d9f30"	"00075e0bc8"	-0.0205
"00074d9f30"	"00075e0fbb"	-0.0783
"00074da036"	"00074da082"	0.0985
"00074da036"	"00074da136"	0.2483
"00074da036"	"00074da3ed"	0.2124
"00074da036"	"00074da4ac"	0.2208
"00074da036"	"00074da4b8"	-0.0088
"00074da036"	"00074da5e8"	0.4578
"00074da036"	"00074da6b4"	0.2432
"00074da036"	"00074daa3c"	-0.037
"00074da036"	"00074daaf6"	-0.0773
"00074da036"	"00074dad20"	-0.0726
"00074da036"	"00074db098"	-0.0029
"00074da036"	"00074db231"	0.2637
"00074da036"	"00074db3a3"	0.0988
"00074da036"	"00074db5d6"	-0.1112
"00074da036"	"00074db632"	0.1379
"00074da036"	"00074db688"	0.3278

"00074da036"	"00074db8a6"	−0.1461
"00074da036"	"00074dba19"	0.3552
"00074da036"	"00074dbc2e"	−0.036
"00074da036"	"00074dbe51"	0.4894
"00074da036"	"00074dbe5f"	−0.261
"00074da036"	"00074dbf6d"	−0.045
"00074da036"	"00074dc4a5"	−0.3917
"00074da036"	"00074dc50c"	0.328
"00074da036"	"00074dcdfa"	0.0236
"00074da036"	"00074dcf5f"	−0.0134
"00074da036"	"00074dd007"	0.0835
"00074da036"	"00074dd163"	−0.0837
"00074da036"	"00074dd3df"	−0.0336
"00074da036"	"00074dd577"	0.6072
"00074da036"	"00074dd62e"	−0.0257
"00074da036"	"00074dd73c"	0.185
"00074da036"	"00074dda10"	0.0734
"00074da036"	"00074ddab8"	0.3188
"00074da036"	"00074ddd3d"	0.2006
"00074da036"	"00074ddf16"	0.1394
"00074da036"	"00074ddfc1"	0.1693
"00074da036"	"00074de21a"	−0.1699
"00074da036"	"00074de2a9"	0.2224
"00074da036"	"00074de544"	0.2944
"00074da036"	"00074de98a"	0.1469
"00074da036"	"00074dea7e"	0.2152
"00074da036"	"00074debd9"	−0.1524
"00074da036"	"00074deca3"	−0.1415
"00074da036"	"00074def43"	−0.0522
"00074da036"	"00074def99"	0.1872
"00074da036"	"00074ecdad"	−0.1754
"00074da036"	"00074ecf28"	−0.1217
"00074da036"	"00074ed1e1"	0.2295
"00074da036"	"00074ed83b"	0.0913
"00074da036"	"00074ee5e3"	−0.0259
"00074da036"	"00074ee6e0"	−0.0609
"00074da036"	"00074eea3a"	0.3733
"00074da036"	"00074eff82"	0.0453
"00074da036"	"00074f0477"	0.0682
"00074da036"	"00074f08c3"	−0.0647
"00074da036"	"00074f1859"	0.0821
"00074da036"	"00074f2268"	0.0925
"00074da036"	"00074f28be"	0.1895
"00074da036"	"00074f294b"	0.1959
"00074da036"	"00074f2ddd"	0.2342
"00074da036"	"00074f2e75"	0.0696
"00074da036"	"00074f3088"	0.24
"00074da036"	"00074f5a1c"	0.0444
"00074da036"	"00074f75b7"	0.1121
"00074da036"	"00074f8cd9"	0.026

"00074da036"	"00074f96dc"	-0.249
"00074da036"	"00074fabaa"	-0.0018
"00074da036"	"00074facd9"	0.0431
"00074da036"	"00074fae3c"	-0.1771
"00074da036"	"00074fb0a8"	0.044
"00074da036"	"00074fb4e4"	-0.2737
"00074da036"	"00074fb7c2"	0.3757
"00074da036"	"00074fbd36"	0.2067
"00074da036"	"00074fc27f"	0.3204
"00074da036"	"00074fc31d"	0.1862
"00074da036"	"00074fd569"	0.0453
"00074da036"	"00074fef15"	0.088
"00074da036"	"00074ff562"	-0.2105
"00074da036"	"00075007ca"	-0.1507
"00074da036"	"0007500b86"	0.1276
"00074da036"	"0007500d05"	-0.0021
"00074da036"	"0007500ee4"	0.1846
"00074da036"	"0007500eee"	-0.1317
"00074da036"	"00075013dc"	-0.0961
"00074da036"	"000757b515"	0.5928
"00074da036"	"000757bc5a"	0.2316
"00074da036"	"000757c320"	-0.1295
"00074da036"	"000757c9aa"	0.1327
"00074da036"	"000757ccbe"	0.305
"00074da036"	"000757cfa9"	0.1698
"00074da036"	"000757d390"	-0.1829
"00074da036"	"000757d393"	0.2721
"00074da036"	"000757d598"	0.1392
"00074da036"	"000757d5a2"	0.2656
"00074da036"	"000757d790"	0.2018
"00074da036"	"000757e30c"	0.0979
"00074da036"	"000757e4b0"	0.3866
"00074da036"	"000757e7a0"	0.2175
"00074da036"	"000757e8b3"	0.432
"00074da036"	"000757f627"	-0.0581
"00074da036"	"000757f925"	0.2881
"00074da036"	"000757fa08"	0.0955
"00074da036"	"000757fe52"	0.3358
"00074da036"	"000758024a"	-0.1387
"00074da036"	"00075804bb"	-0.0433
"00074da036"	"00075a0c04"	0.1677
"00074da036"	"00075a3110"	0.4527
"00074da036"	"00075a341a"	0.0724
"00074da036"	"00075a3dcf"	0.2836
"00074da036"	"00075a3e22"	0.1647
"00074da036"	"00075a48d8"	0.1299
"00074da036"	"00075a5cfb"	-0.1039
"00074da036"	"00075a6151"	0.2414
"00074da036"	"00075a6708"	0.3508
"00074da036"	"00075a7319"	-0.1684

"00074da036"	"00075a7723"	0.073
"00074da036"	"00075a778b"	0.041
"00074da036"	"00075a7b8e"	0.1737
"00074da036"	"00075a7c79"	0.478
"00074da036"	"00075a81b6"	0.0933
"00074da036"	"00075a82ac"	0.4081
"00074da036"	"00075a98e5"	−0.0446
"00074da036"	"00075b0d29"	0.1811
"00074da036"	"00075b102a"	0.1346
"00074da036"	"00075b1074"	0.1268
"00074da036"	"00075b135d"	0.1571
"00074da036"	"00075b138b"	0.3604
"00074da036"	"00075b13a0"	0.148
"00074da036"	"00075b13bd"	0.0771
"00074da036"	"00075b16a9"	0.1062
"00074da036"	"00075b1a28"	−0.1956
"00074da036"	"00075b1a97"	0.0394
"00074da036"	"00075b1c7b"	−0.1896
"00074da036"	"00075b1d24"	0.1818
"00074da036"	"00075b202b"	0.0929
"00074da036"	"00075b22cb"	0.0344
"00074da036"	"00075b22da"	0.246
"00074da036"	"00075b2556"	0.0818
"00074da036"	"00075b25de"	0.2587
"00074da036"	"00075b260c"	0.1085
"00074da036"	"00075b26f1"	−0.1017
"00074da036"	"00075b2920"	0.0413
"00074da036"	"00075b2a64"	−0.0917
"00074da036"	"00075b2a9d"	0.1873
"00074da036"	"00075b2b37"	−0.3448
"00074da036"	"00075b2cdd"	0.0542
"00074da036"	"00075b3038"	0.2987
"00074da036"	"00075b30fe"	0.2988
"00074da036"	"00075b3362"	−0.1866
"00074da036"	"00075b350a"	−0.0049
"00074da036"	"00075b350e"	0.0301
"00074da036"	"00075b3651"	−0.0169
"00074da036"	"00075b38ca"	−0.1101
"00074da036"	"00075b39cc"	0.2533
"00074da036"	"00075b3e1e"	−0.0049
"00074da036"	"00075b3e57"	0.0224
"00074da036"	"00075b4079"	0.0405
"00074da036"	"00075b4150"	0.0026
"00074da036"	"00075b4194"	0.2186
"00074da036"	"00075b42d5"	0.1956
"00074da036"	"00075b4424"	0.043
"00074da036"	"00075b4470"	0.2316
"00074da036"	"00075b47ed"	0.1566
"00074da036"	"00075b4850"	0.2607
"00074da036"	"00075b4ca0"	0.3768

"00074da036"	"00075b4d7f"	0.3863
"00074da036"	"00075b520f"	0.0852
"00074da036"	"00075b525f"	0.0711
"00074da036"	"00075b58f8"	0.0718
"00074da036"	"00075b5bcc"	0.1058
"00074da036"	"00075b5bfa"	-0.0315
"00074da036"	"00075b6339"	0.3861
"00074da036"	"00075b6658"	-0.0308
"00074da036"	"00075b679a"	0.1871
"00074da036"	"00075b6cb7"	0.254
"00074da036"	"00075b6df8"	0.2354
"00074da036"	"00075b6ff6"	0.428
"00074da036"	"00075b70ee"	0.09
"00074da036"	"00075b7157"	0.1462
"00074da036"	"00075b7225"	0.1754
"00074da036"	"00075b7c89"	0.5964
"00074da036"	"00075b9048"	0.3241
"00074da036"	"00075d0801"	0.1955
"00074da036"	"00075d1820"	-0.0054
"00074da036"	"00075d1f3d"	0.1893
"00074da036"	"00075d2329"	0.2428
"00074da036"	"00075d2b9b"	0.012
"00074da036"	"00075d3941"	-0.1695
"00074da036"	"00075d3e96"	0.0943
"00074da036"	"00075d4864"	0.0013
"00074da036"	"00075d5961"	0.1049
"00074da036"	"00075d5a63"	0.14
"00074da036"	"00075d6150"	-0.2159
"00074da036"	"00075d67d0"	0.0248
"00074da036"	"00075d67e2"	0.1484
"00074da036"	"00075d73fc"	0.247
"00074da036"	"00075d7729"	0.0675
"00074da036"	"00075d778c"	-0.1231
"00074da036"	"00075d7b9e"	0.0482
"00074da036"	"00075d7c8f"	0.4605
"00074da036"	"00075d804d"	0.217
"00074da036"	"00075d819f"	0.2542
"00074da036"	"00075d8601"	-0.0723
"00074da036"	"00075d8c6a"	0.1708
"00074da036"	"00075dfedc"	0.3325
"00074da036"	"00075e05f2"	0.1043
"00074da036"	"00075e0837"	0.2643
"00074da036"	"00075e092e"	0.2001
"00074da036"	"00075e0965"	-0.1354
"00074da036"	"00075e0bc8"	0.4335
"00074da036"	"00075e0fbb"	0.2443
"00074da082"	"00074da136"	0.1132
"00074da082"	"00074da3ed"	-0.1018
"00074da082"	"00074da4ac"	0.1141
"00074da082"	"00074da4b8"	-0.1791

"00074da082"	"00074da5e8"	0.06
"00074da082"	"00074da6b4"	0.0084
"00074da082"	"00074daa3c"	-0.4441
"00074da082"	"00074daaf6"	-0.054
"00074da082"	"00074dad20"	-0.0813
"00074da082"	"00074db098"	-0.2028
"00074da082"	"00074db231"	-0.0937
"00074da082"	"00074db3a3"	-0.1027
"00074da082"	"00074db5d6"	-0.034
"00074da082"	"00074db632"	-0.058
"00074da082"	"00074db688"	-0.1797
"00074da082"	"00074db8a6"	-0.3292
"00074da082"	"00074dba19"	-0.1502
"00074da082"	"00074dbc2e"	-0.3197
"00074da082"	"00074dbe51"	0.1606
"00074da082"	"00074dbe5f"	-0.2537
"00074da082"	"00074dbf6d"	-0.3856
"00074da082"	"00074dc4a5"	0.3845
"00074da082"	"00074dc50c"	-0.2883
"00074da082"	"00074dcdfa"	-0.3108
"00074da082"	"00074dcf5f"	-0.076
"00074da082"	"00074dd007"	-0.4005
"00074da082"	"00074dd163"	-0.3057
"00074da082"	"00074dd3df"	0.1252
"00074da082"	"00074dd577"	-0.1411
"00074da082"	"00074dd62e"	-0.0041
"00074da082"	"00074dd73c"	0.0899
"00074da082"	"00074dda10"	-0.1595
"00074da082"	"00074ddab8"	-0.0286
"00074da082"	"00074ddd3d"	-0.0586
"00074da082"	"00074ddf16"	-0.1777
"00074da082"	"00074ddfc1"	-0.2391
"00074da082"	"00074de21a"	-0.4455
"00074da082"	"00074de2a9"	-0.1712
"00074da082"	"00074de544"	-0.005
"00074da082"	"00074de98a"	-0.0752
"00074da082"	"00074dea7e"	0.0133
"00074da082"	"00074debd9"	-0.4485
"00074da082"	"00074deca3"	-0.2266
"00074da082"	"00074def43"	0.013
"00074da082"	"00074def99"	0.1157
"00074da082"	"00074ecdad"	-0.305
"00074da082"	"00074ecf28"	0.1989
"00074da082"	"00074ed1e1"	-0.2197
"00074da082"	"00074ed83b"	0.021
"00074da082"	"00074ee5e3"	-0.3155
"00074da082"	"00074ee6e0"	-0.3029
"00074da082"	"00074eea3a"	0.0962
"00074da082"	"00074eff82"	-0.1569
"00074da082"	"00074f0477"	-0.0977

"00074da082"	"00074f08c3"	-0.4783
"00074da082"	"00074f1859"	-0.0681
"00074da082"	"00074f2268"	-0.1273
"00074da082"	"00074f28be"	-0.1645
"00074da082"	"00074f294b"	0.0386
"00074da082"	"00074f2ddd"	0.0889
"00074da082"	"00074f2e75"	0.1186
"00074da082"	"00074f3088"	0.2107
"00074da082"	"00074f5a1c"	-0.2982
"00074da082"	"00074f75b7"	-0.1185
"00074da082"	"00074f8cd9"	-0.0322
"00074da082"	"00074f96dc"	0.3823
"00074da082"	"00074fabaa"	-0.3041
"00074da082"	"00074facd9"	-0.2115
"00074da082"	"00074fae3c"	0.0647
"00074da082"	"00074fb0a8"	-0.4383
"00074da082"	"00074fb4e4"	-0.4014
"00074da082"	"00074fb7c2"	0.1055
"00074da082"	"00074fbd36"	-0.0162
"00074da082"	"00074fc27f"	-0.2273
"00074da082"	"00074fc31d"	-0.0937
"00074da082"	"00074fd569"	0.3231
"00074da082"	"00074fef15"	0.2372
"00074da082"	"00074ff562"	-0.5089
"00074da082"	"00075007ca"	-0.4291
"00074da082"	"0007500b86"	-0.1661
"00074da082"	"0007500d05"	-0.0279
"00074da082"	"0007500ee4"	-0.0637
"00074da082"	"0007500eee"	-0.2382
"00074da082"	"00075013dc"	-0.39
"00074da082"	"000757b515"	-0.192
"00074da082"	"000757bc5a"	0.0063
"00074da082"	"000757c320"	-0.332
"00074da082"	"000757c9aa"	-0.3344
"00074da082"	"000757ccbe"	0.062
"00074da082"	"000757cfa9"	-0.243
"00074da082"	"000757d390"	-0.5794
"00074da082"	"000757d393"	-0.1144
"00074da082"	"000757d598"	-0.3658
"00074da082"	"000757d5a2"	-0.1875
"00074da082"	"000757d790"	-0.1572
"00074da082"	"000757e30c"	-0.3534
"00074da082"	"000757e4b0"	-0.0723
"00074da082"	"000757e7a0"	0.0486
"00074da082"	"000757e8b3"	0.1829
"00074da082"	"000757f627"	-0.2766
"00074da082"	"000757f925"	0.0245
"00074da082"	"000757fa08"	0.057
"00074da082"	"000757fe52"	0.0436
"00074da082"	"000758024a"	-0.2939

"00074da082"	"00075804bb"	-0.5423
"00074da082"	"00075a0c04"	0.2449
"00074da082"	"00075a3110"	0.0106
"00074da082"	"00075a341a"	0.3267
"00074da082"	"00075a3dcf"	0.0208
"00074da082"	"00075a3e22"	0.0456
"00074da082"	"00075a48d8"	0.1742
"00074da082"	"00075a5cfb"	-0.3164
"00074da082"	"00075a6151"	-0.4605
"00074da082"	"00075a6708"	-0.1158
"00074da082"	"00075a7319"	-0.5957
"00074da082"	"00075a7723"	-0.1079
"00074da082"	"00075a778b"	-0.0523
"00074da082"	"00075a7b8e"	-0.3943
"00074da082"	"00075a7c79"	0.0164
"00074da082"	"00075a81b6"	-0.2436
"00074da082"	"00075a82ac"	0.1887
"00074da082"	"00075a98e5"	-0.0256
"00074da082"	"00075b0d29"	0.0515
"00074da082"	"00075b102a"	-0.0421
"00074da082"	"00075b1074"	0.1065
"00074da082"	"00075b135d"	-0.1514
"00074da082"	"00075b138b"	1e-04
"00074da082"	"00075b13a0"	-0.2219
"00074da082"	"00075b13bd"	0.2828
"00074da082"	"00075b16a9"	0.1173
"00074da082"	"00075b1a28"	0.15
"00074da082"	"00075b1a97"	0.2026
"00074da082"	"00075b1c7b"	0.0056
"00074da082"	"00075b1d24"	0.143
"00074da082"	"00075b202b"	-0.1617
"00074da082"	"00075b22cb"	0.345
"00074da082"	"00075b22da"	0.0719
"00074da082"	"00075b2556"	0.0184
"00074da082"	"00075b25de"	-0.2152
"00074da082"	"00075b260c"	-0.3043
"00074da082"	"00075b26f1"	0.0717
"00074da082"	"00075b2920"	-0.3826
"00074da082"	"00075b2a64"	-0.5078
"00074da082"	"00075b2a9d"	-0.4644
"00074da082"	"00075b2b37"	-0.2704
"00074da082"	"00075b2cdd"	-5e-04
"00074da082"	"00075b3038"	-0.0685
"00074da082"	"00075b30fe"	-0.094
"00074da082"	"00075b3362"	0.1808
"00074da082"	"00075b350a"	-0.1688
"00074da082"	"00075b350e"	-0.1177
"00074da082"	"00075b3651"	0.1181
"00074da082"	"00075b38ca"	-0.2848
"00074da082"	"00075b39cc"	-0.1191

"00074da082"	"00075b3e1e"	-0.0724
"00074da082"	"00075b3e57"	0.4391
"00074da082"	"00075b4079"	-0.2824
"00074da082"	"00075b4150"	-0.2169
"00074da082"	"00075b4194"	-0.1706
"00074da082"	"00075b42d5"	-0.1393
"00074da082"	"00075b4424"	0.2066
"00074da082"	"00075b4470"	0.0063
"00074da082"	"00075b47ed"	-0.0637
"00074da082"	"00075b4850"	-0.0396
"00074da082"	"00075b4ca0"	-0.2688
"00074da082"	"00075b4d7f"	0.269
"00074da082"	"00075b520f"	-0.1003
"00074da082"	"00075b525f"	-0.0699
"00074da082"	"00075b58f8"	-0.3692
"00074da082"	"00075b5bcc"	-0.0515
"00074da082"	"00075b5bfa"	-0.3927
"00074da082"	"00075b6339"	0.042
"00074da082"	"00075b6658"	0.1472
"00074da082"	"00075b679a"	0.2069
"00074da082"	"00075b6cb7"	-0.2235
"00074da082"	"00075b6df8"	0.2689
"00074da082"	"00075b6ff6"	-0.0417
"00074da082"	"00075b70ee"	-0.2224
"00074da082"	"00075b7157"	-0.1437
"00074da082"	"00075b7225"	0.1106
"00074da082"	"00075b7c89"	0.2082
"00074da082"	"00075b9048"	-0.1254
"00074da082"	"00075d0801"	-0.2525
"00074da082"	"00075d1820"	0.1876
"00074da082"	"00075d1f3d"	-0.2186
"00074da082"	"00075d2329"	-0.0962
"00074da082"	"00075d2b9b"	-0.4615
"00074da082"	"00075d3941"	0.2736
"00074da082"	"00075d3e96"	0.1039
"00074da082"	"00075d4864"	-0.3551
"00074da082"	"00075d5961"	0.0472
"00074da082"	"00075d5a63"	0.0924
"00074da082"	"00075d6150"	-0.2489
"00074da082"	"00075d67d0"	-0.2698
"00074da082"	"00075d67e2"	-0.0519
"00074da082"	"00075d73fc"	0.4669
"00074da082"	"00075d7729"	-0.0841
"00074da082"	"00075d778c"	-0.1134
"00074da082"	"00075d7b9e"	-0.1708
"00074da082"	"00075d7c8f"	0.2563
"00074da082"	"00075d804d"	-0.3699
"00074da082"	"00075d819f"	-0.262
"00074da082"	"00075d8601"	-0.0176
"00074da082"	"00075d8c6a"	0.0801

"00074da082"	"00075dfedc"	0.2016
"00074da082"	"00075e05f2"	−0.2561
"00074da082"	"00075e0837"	−0.1377
"00074da082"	"00075e092e"	−0.0276
"00074da082"	"00075e0965"	−0.5135
"00074da082"	"00075e0bc8"	−0.0035
"00074da082"	"00075e0fbb"	−0.0916
"00074da136"	"00074da3ed"	0.1319
"00074da136"	"00074da4ac"	0.1073
"00074da136"	"00074da4b8"	−0.15
"00074da136"	"00074da5e8"	0.1049
"00074da136"	"00074da6b4"	0.4328
"00074da136"	"00074daa3c"	−0.5514
"00074da136"	"00074daaf6"	−0.3676
"00074da136"	"00074dad20"	−0.1263
"00074da136"	"00074db098"	−0.3904
"00074da136"	"00074db231"	0.2244
"00074da136"	"00074db3a3"	−0.3134
"00074da136"	"00074db5d6"	−0.2754
"00074da136"	"00074db632"	0.0012
"00074da136"	"00074db688"	0.2048
"00074da136"	"00074db8a6"	−0.3593
"00074da136"	"00074dba19"	−0.0688
"00074da136"	"00074dbc2e"	−0.1019
"00074da136"	"00074dbe51"	0.251
"00074da136"	"00074dbe5f"	−0.3008
"00074da136"	"00074dbf6d"	−0.7105
"00074da136"	"00074dc4a5"	0.0536
"00074da136"	"00074dc50c"	−0.0937
"00074da136"	"00074dcdfa"	−0.3208
"00074da136"	"00074dcf5f"	−0.149
"00074da136"	"00074dd007"	−0.4121
"00074da136"	"00074dd163"	−0.2741
"00074da136"	"00074dd3df"	−0.1123
"00074da136"	"00074dd577"	0.0547
"00074da136"	"00074dd62e"	−0.0386
"00074da136"	"00074dd73c"	0.0133
"00074da136"	"00074dda10"	−0.0727
"00074da136"	"00074ddab8"	−0.1222
"00074da136"	"00074ddd3d"	−0.0888
"00074da136"	"00074ddf16"	−0.3263
"00074da136"	"00074ddfc1"	−0.2415
"00074da136"	"00074de21a"	−0.375
"00074da136"	"00074de2a9"	−0.0615
"00074da136"	"00074de544"	0.1374
"00074da136"	"00074de98a"	0.0594
"00074da136"	"00074dea7e"	−0.0217
"00074da136"	"00074debd9"	−0.4411
"00074da136"	"00074deca3"	−0.3149
"00074da136"	"00074def43"	−0.1599

"00074da136"	"00074def99"	0.1013
"00074da136"	"00074ecdad"	0.1179
"00074da136"	"00074ecf28"	−0.1151
"00074da136"	"00074ed1e1"	0.0082
"00074da136"	"00074ed83b"	0.0769
"00074da136"	"00074ee5e3"	−0.5045
"00074da136"	"00074ee6e0"	−0.1345
"00074da136"	"00074eea3a"	0.2647
"00074da136"	"00074eff82"	−0.085
"00074da136"	"00074f0477"	−0.2431
"00074da136"	"00074f08c3"	−0.4194
"00074da136"	"00074f1859"	0.0854
"00074da136"	"00074f2268"	−0.2407
"00074da136"	"00074f28be"	−0.1148
"00074da136"	"00074f294b"	0.1294
"00074da136"	"00074f2ddd"	0.0197
"00074da136"	"00074f2e75"	0.6024
"00074da136"	"00074f3088"	0.0221
"00074da136"	"00074f5a1c"	0.0612
"00074da136"	"00074f75b7"	−0.1554
"00074da136"	"00074f8cd9"	0.0071
"00074da136"	"00074f96dc"	−0.277
"00074da136"	"00074fabaa"	−0.3669
"00074da136"	"00074facd9"	−0.2247
"00074da136"	"00074fae3c"	−0.0655
"00074da136"	"00074fb0a8"	−0.1534
"00074da136"	"00074fb4e4"	−0.5711
"00074da136"	"00074fb7c2"	0.5469
"00074da136"	"00074fbd36"	−0.1629
"00074da136"	"00074fc27f"	−0.1222
"00074da136"	"00074fc31d"	0.1434
"00074da136"	"00074fd569"	0.0163
"00074da136"	"00074fef15"	−0.1157
"00074da136"	"00074ff562"	−0.4683
"00074da136"	"00075007ca"	−0.2183
"00074da136"	"0007500b86"	−0.3048
"00074da136"	"0007500d05"	−0.1449
"00074da136"	"0007500ee4"	−0.1802
"00074da136"	"0007500eee"	−0.1684
"00074da136"	"00075013dc"	−0.5006
"00074da136"	"000757b515"	−0.0541
"00074da136"	"000757bc5a"	−0.0986
"00074da136"	"000757c320"	−0.608
"00074da136"	"000757c9aa"	−0.2179
"00074da136"	"000757ccbe"	0.3749
"00074da136"	"000757cfa9"	−0.3023
"00074da136"	"000757d390"	−0.4587
"00074da136"	"000757d393"	−0.0075
"00074da136"	"000757d598"	−0.4031
"00074da136"	"000757d5a2"	−0.1523

"00074da136"	"000757d790"	0.0782
"00074da136"	"000757e30c"	−0.2545
"00074da136"	"000757e4b0"	−0.0384
"00074da136"	"000757e7a0"	−0.0712
"00074da136"	"000757e8b3"	0.1027
"00074da136"	"000757f627"	−0.2739
"00074da136"	"000757f925"	0.0676
"00074da136"	"000757fa08"	0.465
"00074da136"	"000757fe52"	0.0449
"00074da136"	"000758024a"	−0.1788
"00074da136"	"00075804bb"	−0.3581
"00074da136"	"00075a0c04"	0.2743
"00074da136"	"00075a3110"	0.0656
"00074da136"	"00075a341a"	0.248
"00074da136"	"00075a3dcf"	0.2515
"00074da136"	"00075a3e22"	0.0717
"00074da136"	"00075a48d8"	0.4102
"00074da136"	"00075a5cfb"	−0.2811
"00074da136"	"00075a6151"	−0.1433
"00074da136"	"00075a6708"	−0.0913
"00074da136"	"00075a7319"	−0.4973
"00074da136"	"00075a7723"	0.1763
"00074da136"	"00075a778b"	0.1465
"00074da136"	"00075a7b8e"	0.0904
"00074da136"	"00075a7c79"	−0.1244
"00074da136"	"00075a81b6"	0.177
"00074da136"	"00075a82ac"	0.1652
"00074da136"	"00075a98e5"	−0.2424
"00074da136"	"00075b0d29"	−0.1134
"00074da136"	"00075b102a"	−0.0879
"00074da136"	"00075b1074"	0.1966
"00074da136"	"00075b135d"	0.0558
"00074da136"	"00075b138b"	−0.1235
"00074da136"	"00075b13a0"	0.0485
"00074da136"	"00075b13bd"	−0.0523
"00074da136"	"00075b16a9"	0.1174
"00074da136"	"00075b1a28"	−0.0143
"00074da136"	"00075b1a97"	−0.0701
"00074da136"	"00075b1c7b"	0.0365
"00074da136"	"00075b1d24"	0.0656
"00074da136"	"00075b202b"	−0.1588
"00074da136"	"00075b22cb"	0.0241
"00074da136"	"00075b22da"	0.0358
"00074da136"	"00075b2556"	−0.0959
"00074da136"	"00075b25de"	−0.2253
"00074da136"	"00075b260c"	−0.2189
"00074da136"	"00075b26f1"	0.066
"00074da136"	"00075b2920"	−0.2824
"00074da136"	"00075b2a64"	−0.4493
"00074da136"	"00075b2a9d"	−0.197

"00074da136"	"00075b2b37"	−0.3968
"00074da136"	"00075b2cdd"	0.0305
"00074da136"	"00075b3038"	−0.1996
"00074da136"	"00075b30fe"	−0.0264
"00074da136"	"00075b3362"	−0.0528
"00074da136"	"00075b350a"	−0.4278
"00074da136"	"00075b350e"	−0.1465
"00074da136"	"00075b3651"	−0.2289
"00074da136"	"00075b38ca"	−0.0901
"00074da136"	"00075b39cc"	−0.1574
"00074da136"	"00075b3e1e"	0.087
"00074da136"	"00075b3e57"	0.0508
"00074da136"	"00075b4079"	−0.3111
"00074da136"	"00075b4150"	−0.0655
"00074da136"	"00075b4194"	−0.2485
"00074da136"	"00075b42d5"	0.0232
"00074da136"	"00075b4424"	0.0358
"00074da136"	"00075b4470"	−0.0292
"00074da136"	"00075b47ed"	1e−04
"00074da136"	"00075b4850"	−0.1592
"00074da136"	"00075b4ca0"	−0.0244
"00074da136"	"00075b4d7f"	0.3177
"00074da136"	"00075b520f"	−0.0865
"00074da136"	"00075b525f"	−0.1348
"00074da136"	"00075b58f8"	−0.2559
"00074da136"	"00075b5bcc"	−0.2396
"00074da136"	"00075b5bfa"	−0.3556
"00074da136"	"00075b6339"	0.1118
"00074da136"	"00075b6658"	−0.0858
"00074da136"	"00075b679a"	−0.0899
"00074da136"	"00075b6cb7"	−0.1223
"00074da136"	"00075b6df8"	0.362
"00074da136"	"00075b6ff6"	−0.1377
"00074da136"	"00075b70ee"	−0.1227
"00074da136"	"00075b7157"	0.0152
"00074da136"	"00075b7225"	0.1906
"00074da136"	"00075b7c89"	0.1017
"00074da136"	"00075b9048"	−0.0184
"00074da136"	"00075d0801"	−0.2087
"00074da136"	"00075d1820"	−0.0878
"00074da136"	"00075d1f3d"	−0.1988
"00074da136"	"00075d2329"	0.1397
"00074da136"	"00075d2b9b"	−0.3381
"00074da136"	"00075d3941"	−0.1327
"00074da136"	"00075d3e96"	0.0708
"00074da136"	"00075d4864"	0.1254
"00074da136"	"00075d5961"	0.148
"00074da136"	"00075d5a63"	−0.0743
"00074da136"	"00075d6150"	−0.0547
"00074da136"	"00075d67d0"	−0.0854

"00074da136"	"00075d67e2"	−0.0708
"00074da136"	"00075d73fc"	0.2313
"00074da136"	"00075d7729"	−0.0176
"00074da136"	"00075d778c"	−0.0537
"00074da136"	"00075d7b9e"	0.0188
"00074da136"	"00075d7c8f"	0.0853
"00074da136"	"00075d804d"	−0.3173
"00074da136"	"00075d819f"	−0.1778
"00074da136"	"00075d8601"	−0.2075
"00074da136"	"00075d8c6a"	0.0683
"00074da136"	"00075dfedc"	0.0155
"00074da136"	"00075e05f2"	−0.0292
"00074da136"	"00075e0837"	−0.0735
"00074da136"	"00075e092e"	0.0515
"00074da136"	"00075e0965"	−0.1899
"00074da136"	"00075e0bc8"	0.0225
"00074da136"	"00075e0fbb"	−0.0712
"00074da3ed"	"00074da4ac"	0.1602
"00074da3ed"	"00074da4b8"	−0.2927
"00074da3ed"	"00074da5e8"	−0.0114
"00074da3ed"	"00074da6b4"	0.186
"00074da3ed"	"00074daa3c"	−0.2694
"00074da3ed"	"00074daaf6"	−0.2865
"00074da3ed"	"00074dad20"	−0.1335
"00074da3ed"	"00074db098"	−0.347
"00074da3ed"	"00074db231"	0.1316
"00074da3ed"	"00074db3a3"	0.0546
"00074da3ed"	"00074db5d6"	−0.5699
"00074da3ed"	"00074db632"	−0.0011
"00074da3ed"	"00074db688"	−0.136
"00074da3ed"	"00074db8a6"	−0.1145
"00074da3ed"	"00074dba19"	−0.1845
"00074da3ed"	"00074dbc2e"	0.092
"00074da3ed"	"00074dbe51"	0.1433
"00074da3ed"	"00074dbe5f"	−0.3044
"00074da3ed"	"00074dbf6d"	−0.309
"00074da3ed"	"00074dc4a5"	−0.1579
"00074da3ed"	"00074dc50c"	−0.0597
"00074da3ed"	"00074dcdfa"	−0.2283
"00074da3ed"	"00074dcf5f"	−0.2224
"00074da3ed"	"00074dd007"	−0.2255
"00074da3ed"	"00074dd163"	−0.0995
"00074da3ed"	"00074dd3df"	0.3618
"00074da3ed"	"00074dd577"	0.1609
"00074da3ed"	"00074dd62e"	−0.2262
"00074da3ed"	"00074dd73c"	0.1586
"00074da3ed"	"00074dda10"	−0.0966
"00074da3ed"	"00074ddab8"	−0.0198
"00074da3ed"	"00074ddd3d"	−0.0819
"00074da3ed"	"00074ddf16"	0.1605

"00074da3ed"	"00074ddfc1"	0.2587
"00074da3ed"	"00074de21a"	-0.2415
"00074da3ed"	"00074de2a9"	0.0083
"00074da3ed"	"00074de544"	-0.044
"00074da3ed"	"00074de98a"	-0.0242
"00074da3ed"	"00074dea7e"	0.4791
"00074da3ed"	"00074debd9"	-0.2414
"00074da3ed"	"00074deca3"	-0.1125
"00074da3ed"	"00074def43"	0.2844
"00074da3ed"	"00074def99"	-0.0947
"00074da3ed"	"00074ecdad"	-0.0122
"00074da3ed"	"00074ecf28"	-0.1699
"00074da3ed"	"00074ed1e1"	0.3021
"00074da3ed"	"00074ed83b"	0.067
"00074da3ed"	"00074ee5e3"	-0.2816
"00074da3ed"	"00074ee6e0"	-0.3171
"00074da3ed"	"00074eea3a"	0.5238
"00074da3ed"	"00074eff82"	-0.1916
"00074da3ed"	"00074f0477"	-0.1234
"00074da3ed"	"00074f08c3"	-0.4027
"00074da3ed"	"00074f1859"	0.0498
"00074da3ed"	"00074f2268"	-0.0433
"00074da3ed"	"00074f28be"	-0.0874
"00074da3ed"	"00074f294b"	0.5325
"00074da3ed"	"00074f2ddd"	0.1191
"00074da3ed"	"00074f2e75"	0.0427
"00074da3ed"	"00074f3088"	-0.146
"00074da3ed"	"00074f5a1c"	0.0878
"00074da3ed"	"00074f75b7"	-0.1394
"00074da3ed"	"00074f8cd9"	-0.0483
"00074da3ed"	"00074f96dc"	-0.5318
"00074da3ed"	"00074fabaa"	0.0191
"00074da3ed"	"00074facd9"	-0.0403
"00074da3ed"	"00074fae3c"	-0.2432
"00074da3ed"	"00074fb0a8"	-0.185
"00074da3ed"	"00074fb4e4"	-0.3128
"00074da3ed"	"00074fb7c2"	0.3388
"00074da3ed"	"00074fbd36"	-0.3035
"00074da3ed"	"00074fc27f"	0.0759
"00074da3ed"	"00074fc31d"	-0.0445
"00074da3ed"	"00074fd569"	-0.1272
"00074da3ed"	"00074fef15"	-0.1323
"00074da3ed"	"00074ff562"	-0.4086
"00074da3ed"	"00075007ca"	-0.2899
"00074da3ed"	"0007500b86"	-0.182
"00074da3ed"	"0007500d05"	0.0317
"00074da3ed"	"0007500ee4"	0.0328
"00074da3ed"	"0007500eee"	-0.1877
"00074da3ed"	"00075013dc"	-0.5426
"00074da3ed"	"000757b515"	0.1643

"00074da3ed"	"000757bc5a"	0.0623
"00074da3ed"	"000757c320"	-0.3023
"00074da3ed"	"000757c9aa"	-0.2718
"00074da3ed"	"000757ccbe"	-0.1118
"00074da3ed"	"000757cfa9"	-0.4215
"00074da3ed"	"000757d390"	-0.1654
"00074da3ed"	"000757d393"	0.057
"00074da3ed"	"000757d598"	-0.2852
"00074da3ed"	"000757d5a2"	-0.0887
"00074da3ed"	"000757d790"	0.1401
"00074da3ed"	"000757e30c"	-0.0941
"00074da3ed"	"000757e4b0"	0.25
"00074da3ed"	"000757e7a0"	0.0261
"00074da3ed"	"000757e8b3"	-0.1024
"00074da3ed"	"000757f627"	-0.1816
"00074da3ed"	"000757f925"	-0.0022
"00074da3ed"	"000757fa08"	0.157
"00074da3ed"	"000757fe52"	0.0552
"00074da3ed"	"000758024a"	-0.33
"00074da3ed"	"00075804bb"	-0.1675
"00074da3ed"	"00075a0c04"	0.0145
"00074da3ed"	"00075a3110"	0.001
"00074da3ed"	"00075a341a"	0.1388
"00074da3ed"	"00075a3dcf"	0.2823
"00074da3ed"	"00075a3e22"	-0.0551
"00074da3ed"	"00075a48d8"	0.0551
"00074da3ed"	"00075a5cfb"	-0.26
"00074da3ed"	"00075a6151"	-0.2477
"00074da3ed"	"00075a6708"	-0.1402
"00074da3ed"	"00075a7319"	-0.1777
"00074da3ed"	"00075a7723"	0.249
"00074da3ed"	"00075a778b"	0.067
"00074da3ed"	"00075a7b8e"	0.0423
"00074da3ed"	"00075a7c79"	0.0848
"00074da3ed"	"00075a81b6"	-0.2166
"00074da3ed"	"00075a82ac"	0.2278
"00074da3ed"	"00075a98e5"	-0.2523
"00074da3ed"	"00075b0d29"	-0.3752
"00074da3ed"	"00075b102a"	-0.1258
"00074da3ed"	"00075b1074"	0.5566
"00074da3ed"	"00075b135d"	-0.2208
"00074da3ed"	"00075b138b"	0.1568
"00074da3ed"	"00075b13a0"	-0.0702
"00074da3ed"	"00075b13bd"	-0.2175
"00074da3ed"	"00075b16a9"	-0.0329
"00074da3ed"	"00075b1a28"	-0.0142
"00074da3ed"	"00075b1a97"	-0.1771
"00074da3ed"	"00075b1c7b"	-0.1525
"00074da3ed"	"00075b1d24"	-0.029
"00074da3ed"	"00075b202b"	-0.2393

"00074da3ed"	"00075b22cb"	0.1413
"00074da3ed"	"00075b22da"	0.5719
"00074da3ed"	"00075b2556"	0.5131
"00074da3ed"	"00075b25de"	−0.0176
"00074da3ed"	"00075b260c"	−0.1074
"00074da3ed"	"00075b26f1"	−0.1638
"00074da3ed"	"00075b2920"	−0.1158
"00074da3ed"	"00075b2a64"	−0.3866
"00074da3ed"	"00075b2a9d"	−0.1715
"00074da3ed"	"00075b2b37"	−0.3843
"00074da3ed"	"00075b2cdd"	−0.0642
"00074da3ed"	"00075b3038"	0.149
"00074da3ed"	"00075b30fe"	0.6148
"00074da3ed"	"00075b3362"	−0.1125
"00074da3ed"	"00075b350a"	−0.1614
"00074da3ed"	"00075b350e"	−0.1252
"00074da3ed"	"00075b3651"	−0.0933
"00074da3ed"	"00075b38ca"	0.0545
"00074da3ed"	"00075b39cc"	−0.0314
"00074da3ed"	"00075b3e1e"	−0.0095
"00074da3ed"	"00075b3e57"	−0.0592
"00074da3ed"	"00075b4079"	−0.2605
"00074da3ed"	"00075b4150"	−0.2477
"00074da3ed"	"00075b4194"	−0.0591
"00074da3ed"	"00075b42d5"	0.2783
"00074da3ed"	"00075b4424"	−0.1465
"00074da3ed"	"00075b4470"	0.1317
"00074da3ed"	"00075b47ed"	0.1142
"00074da3ed"	"00075b4850"	0.0074
"00074da3ed"	"00075b4ca0"	−0.1074
"00074da3ed"	"00075b4d7f"	0.1572
"00074da3ed"	"00075b520f"	−0.3549
"00074da3ed"	"00075b525f"	0.1312
"00074da3ed"	"00075b58f8"	−0.0663
"00074da3ed"	"00075b5bcc"	−0.1044
"00074da3ed"	"00075b5bfa"	−0.1662
"00074da3ed"	"00075b6339"	−0.134
"00074da3ed"	"00075b6658"	−0.3418
"00074da3ed"	"00075b679a"	0.0375
"00074da3ed"	"00075b6cb7"	−0.105
"00074da3ed"	"00075b6df8"	0.1721
"00074da3ed"	"00075b6ff6"	0.1553
"00074da3ed"	"00075b70ee"	−0.102
"00074da3ed"	"00075b7157"	0.1287
"00074da3ed"	"00075b7225"	−0.0172
"00074da3ed"	"00075b7c89"	−0.1371
"00074da3ed"	"00075b9048"	−0.0304
"00074da3ed"	"00075d0801"	0.0702
"00074da3ed"	"00075d1820"	−0.1417
"00074da3ed"	"00075d1f3d"	−0.0425

"00074da3ed"	"00075d2329"	0.1784
"00074da3ed"	"00075d2b9b"	-0.1805
"00074da3ed"	"00075d3941"	-0.2726
"00074da3ed"	"00075d3e96"	0.1234
"00074da3ed"	"00075d4864"	0.2177
"00074da3ed"	"00075d5961"	0.0404
"00074da3ed"	"00075d5a63"	0.0526
"00074da3ed"	"00075d6150"	-0.3307
"00074da3ed"	"00075d67d0"	-0.1297
"00074da3ed"	"00075d67e2"	-0.1694
"00074da3ed"	"00075d73fc"	-0.0155
"00074da3ed"	"00075d7729"	-0.2515
"00074da3ed"	"00075d778c"	-0.0305
"00074da3ed"	"00075d7b9e"	-0.0047
"00074da3ed"	"00075d7c8f"	0.126
"00074da3ed"	"00075d804d"	-0.039
"00074da3ed"	"00075d819f"	-0.0162
"00074da3ed"	"00075d8601"	-0.3467
"00074da3ed"	"00075d8c6a"	0.0365
"00074da3ed"	"00075dfedc"	-0.2498
"00074da3ed"	"00075e05f2"	-0.0705
"00074da3ed"	"00075e0837"	0.0312
"00074da3ed"	"00075e092e"	0.5545
"00074da3ed"	"00075e0965"	-0.0684
"00074da3ed"	"00075e0bc8"	0.0267
"00074da3ed"	"00075e0fbb"	0.6445
"00074da4ac"	"00074da4b8"	0.2938
"00074da4ac"	"00074da5e8"	0.1609
"00074da4ac"	"00074da6b4"	0.1613
"00074da4ac"	"00074daa3c"	0.0618
"00074da4ac"	"00074daaf6"	0.159
"00074da4ac"	"00074dad20"	0.0275
"00074da4ac"	"00074db098"	0.0735
"00074da4ac"	"00074db231"	0.3586
"00074da4ac"	"00074db3a3"	0.3785
"00074da4ac"	"00074db5d6"	0.0905
"00074da4ac"	"00074db632"	0.3443
"00074da4ac"	"00074db688"	-5e-04
"00074da4ac"	"00074db8a6"	0.1583
"00074da4ac"	"00074dba19"	0.1335
"00074da4ac"	"00074dbc2e"	0.0296
"00074da4ac"	"00074dbe51"	0.406
"00074da4ac"	"00074dbe5f"	0.2061
"00074da4ac"	"00074dbf6d"	0.1387
"00074da4ac"	"00074dc4a5"	-0.0178
"00074da4ac"	"00074dc50c"	0.1183
"00074da4ac"	"00074dcdfa"	0.1054
"00074da4ac"	"00074dcf5f"	0.073
"00074da4ac"	"00074dd007"	0.0857
"00074da4ac"	"00074dd163"	0.224

"00074da4ac"	"00074dd3df"	0.0411
"00074da4ac"	"00074dd577"	0.0202
"00074da4ac"	"00074dd62e"	0.1879
"00074da4ac"	"00074dd73c"	0.1148
"00074da4ac"	"00074dda10"	0.2534
"00074da4ac"	"00074ddab8"	0.1773
"00074da4ac"	"00074ddd3d"	0.0482
"00074da4ac"	"00074ddf16"	0.4056
"00074da4ac"	"00074ddfc1"	0.4232
"00074da4ac"	"00074de21a"	-0.0785
"00074da4ac"	"00074de2a9"	0.2706
"00074da4ac"	"00074de544"	0.319
"00074da4ac"	"00074de98a"	0.3176
"00074da4ac"	"00074dea7e"	0.1514
"00074da4ac"	"00074debd9"	0.0186
"00074da4ac"	"00074deca3"	0.1824
"00074da4ac"	"00074def43"	0.3672
"00074da4ac"	"00074def99"	0.1258
"00074da4ac"	"00074ecdad"	-0.1222
"00074da4ac"	"00074ecf28"	-0.1075
"00074da4ac"	"00074ed1e1"	0.0997
"00074da4ac"	"00074ed83b"	0.463
"00074da4ac"	"00074ee5e3"	0.0154
"00074da4ac"	"00074ee6e0"	0.1443
"00074da4ac"	"00074eea3a"	0.2637
"00074da4ac"	"00074eff82"	0.2104
"00074da4ac"	"00074f0477"	-0.193
"00074da4ac"	"00074f08c3"	-0.1164
"00074da4ac"	"00074f1859"	0.3404
"00074da4ac"	"00074f2268"	-0.0613
"00074da4ac"	"00074f28be"	0.2898
"00074da4ac"	"00074f294b"	0.1212
"00074da4ac"	"00074f2ddd"	0.06
"00074da4ac"	"00074f2e75"	0.113
"00074da4ac"	"00074f3088"	0.0667
"00074da4ac"	"00074f5a1c"	0.0506
"00074da4ac"	"00074f75b7"	0.1842
"00074da4ac"	"00074f8cd9"	0.4075
"00074da4ac"	"00074f96dc"	-0.0628
"00074da4ac"	"00074fabaa"	0.2119
"00074da4ac"	"00074facd9"	0.2467
"00074da4ac"	"00074fae3c"	0.245
"00074da4ac"	"00074fb0a8"	0.2447
"00074da4ac"	"00074fb4e4"	0.0551
"00074da4ac"	"00074fb7c2"	0.2985
"00074da4ac"	"00074fbd36"	-0.0359
"00074da4ac"	"00074fc27f"	0.2355
"00074da4ac"	"00074fc31d"	0.0556
"00074da4ac"	"00074fd569"	0.1046
"00074da4ac"	"00074fef15"	0.137

"00074da4ac"	"00074ff562"	−0.2584
"00074da4ac"	"00075007ca"	0.0742
"00074da4ac"	"0007500b86"	0.3065
"00074da4ac"	"0007500d05"	0.2958
"00074da4ac"	"0007500ee4"	0.1608
"00074da4ac"	"0007500eee"	0.1097
"00074da4ac"	"00075013dc"	−0.1063
"00074da4ac"	"000757b515"	0.2087
"00074da4ac"	"000757bc5a"	0.2104
"00074da4ac"	"000757c320"	0.1335
"00074da4ac"	"000757c9aa"	0.2164
"00074da4ac"	"000757ccbe"	0.1359
"00074da4ac"	"000757cfa9"	−0.1221
"00074da4ac"	"000757d390"	−0.0034
"00074da4ac"	"000757d393"	0.3382
"00074da4ac"	"000757d598"	0.0086
"00074da4ac"	"000757d5a2"	0.0688
"00074da4ac"	"000757d790"	0.1735
"00074da4ac"	"000757e30c"	0.1626
"00074da4ac"	"000757e4b0"	0.1515
"00074da4ac"	"000757e7a0"	0.1185
"00074da4ac"	"000757e8b3"	0.4552
"00074da4ac"	"000757f627"	0.3853
"00074da4ac"	"000757f925"	0.4614
"00074da4ac"	"000757fa08"	0.0278
"00074da4ac"	"000757fe52"	0.1188
"00074da4ac"	"000758024a"	0.1518
"00074da4ac"	"00075804bb"	0.0182
"00074da4ac"	"00075a0c04"	0.3512
"00074da4ac"	"00075a3110"	0.0689
"00074da4ac"	"00075a341a"	0.2253
"00074da4ac"	"00075a3dcf"	0.0549
"00074da4ac"	"00075a3e22"	0.4074
"00074da4ac"	"00075a48d8"	0.431
"00074da4ac"	"00075a5cfb"	0.1493
"00074da4ac"	"00075a6151"	0.2281
"00074da4ac"	"00075a6708"	−0.1254
"00074da4ac"	"00075a7319"	0.0619
"00074da4ac"	"00075a7723"	0.3502
"00074da4ac"	"00075a778b"	0.0992
"00074da4ac"	"00075a7b8e"	0.4482
"00074da4ac"	"00075a7c79"	0.1809
"00074da4ac"	"00075a81b6"	0.2727
"00074da4ac"	"00075a82ac"	0.2908
"00074da4ac"	"00075a98e5"	0.1691
"00074da4ac"	"00075b0d29"	0.1389
"00074da4ac"	"00075b102a"	0.3663
"00074da4ac"	"00075b1074"	0.0082
"00074da4ac"	"00075b135d"	0.0675
"00074da4ac"	"00075b138b"	0.3721

"00074da4ac"	"00075b13a0"	−0.0721
"00074da4ac"	"00075b13bd"	0.0801
"00074da4ac"	"00075b16a9"	0.4506
"00074da4ac"	"00075b1a28"	0.0908
"00074da4ac"	"00075b1a97"	0.2491
"00074da4ac"	"00075b1c7b"	0.14
"00074da4ac"	"00075b1d24"	0.1944
"00074da4ac"	"00075b202b"	0.0826
"00074da4ac"	"00075b22cb"	0.3324
"00074da4ac"	"00075b22da"	0.2055
"00074da4ac"	"00075b2556"	0.1622
"00074da4ac"	"00075b25de"	0.0873
"00074da4ac"	"00075b260c"	0.1528
"00074da4ac"	"00075b26f1"	0.2263
"00074da4ac"	"00075b2920"	0.155
"00074da4ac"	"00075b2a64"	−0.0111
"00074da4ac"	"00075b2a9d"	0.0836
"00074da4ac"	"00075b2b37"	−0.21
"00074da4ac"	"00075b2cdd"	0.2805
"00074da4ac"	"00075b3038"	0.0916
"00074da4ac"	"00075b30fe"	−0.0542
"00074da4ac"	"00075b3362"	0.0717
"00074da4ac"	"00075b350a"	−0.08
"00074da4ac"	"00075b350e"	0.0889
"00074da4ac"	"00075b3651"	0.1334
"00074da4ac"	"00075b38ca"	−0.0382
"00074da4ac"	"00075b39cc"	0.0795
"00074da4ac"	"00075b3e1e"	0.4224
"00074da4ac"	"00075b3e57"	0.1881
"00074da4ac"	"00075b4079"	−0.0839
"00074da4ac"	"00075b4150"	0.0997
"00074da4ac"	"00075b4194"	0.3602
"00074da4ac"	"00075b42d5"	0.1616
"00074da4ac"	"00075b4424"	−0.0071
"00074da4ac"	"00075b4470"	0.1948
"00074da4ac"	"00075b47ed"	0.1355
"00074da4ac"	"00075b4850"	0.1372
"00074da4ac"	"00075b4ca0"	0.0426
"00074da4ac"	"00075b4d7f"	0.4823
"00074da4ac"	"00075b520f"	−0.0866
"00074da4ac"	"00075b525f"	0.1486
"00074da4ac"	"00075b58f8"	−0.0321
"00074da4ac"	"00075b5bcc"	0.1672
"00074da4ac"	"00075b5bfa"	−0.2075
"00074da4ac"	"00075b6339"	0.3893
"00074da4ac"	"00075b6658"	−0.2277
"00074da4ac"	"00075b679a"	0.1903
"00074da4ac"	"00075b6cb7"	0.1572
"00074da4ac"	"00075b6df8"	0.2479
"00074da4ac"	"00075b6ff6"	0.1444

"00074da4ac"	"00075b70ee"	−0.0585
"00074da4ac"	"00075b7157"	0.2814
"00074da4ac"	"00075b7225"	0.2124
"00074da4ac"	"00075b7c89"	0.1575
"00074da4ac"	"00075b9048"	0.2209
"00074da4ac"	"00075d0801"	0.075
"00074da4ac"	"00075d1820"	0.2025
"00074da4ac"	"00075d1f3d"	0.027
"00074da4ac"	"00075d2329"	−0.0279
"00074da4ac"	"00075d2b9b"	0.0314
"00074da4ac"	"00075d3941"	−0.0401
"00074da4ac"	"00075d3e96"	0.2103
"00074da4ac"	"00075d4864"	−0.0783
"00074da4ac"	"00075d5961"	0.2432
"00074da4ac"	"00075d5a63"	0.1083
"00074da4ac"	"00075d6150"	0.016
"00074da4ac"	"00075d67d0"	−0.1391
"00074da4ac"	"00075d67e2"	0.2748
"00074da4ac"	"00075d73fc"	0.2019
"00074da4ac"	"00075d7729"	−0.1113
"00074da4ac"	"00075d778c"	0.0349
"00074da4ac"	"00075d7b9e"	0.3305
"00074da4ac"	"00075d7c8f"	0.3423
"00074da4ac"	"00075d804d"	0.1909
"00074da4ac"	"00075d819f"	0.1329
"00074da4ac"	"00075d8601"	0.175
"00074da4ac"	"00075d8c6a"	0.2954
"00074da4ac"	"00075dfedc"	0.385
"00074da4ac"	"00075e05f2"	0.3533
"00074da4ac"	"00075e0837"	0.1329
"00074da4ac"	"00075e092e"	0.191
"00074da4ac"	"00075e0965"	−0.1536
"00074da4ac"	"00075e0bc8"	0.1098
"00074da4ac"	"00075e0fbb"	0.0401
"00074da4b8"	"00074da5e8"	0.175
"00074da4b8"	"00074da6b4"	0.2652
"00074da4b8"	"00074daa3c"	0.0863
"00074da4b8"	"00074daaf6"	−0.1498
"00074da4b8"	"00074dad20"	−0.0705
"00074da4b8"	"00074db098"	0.1616
"00074da4b8"	"00074db231"	−0.0844
"00074da4b8"	"00074db3a3"	0.3281
"00074da4b8"	"00074db5d6"	0.1734
"00074da4b8"	"00074db632"	0.5947
"00074da4b8"	"00074db688"	0.0174
"00074da4b8"	"00074db8a6"	−0.2787
"00074da4b8"	"00074dba19"	0.0466
"00074da4b8"	"00074dbc2e"	−0.1245
"00074da4b8"	"00074dbe51"	0.1371
"00074da4b8"	"00074dbe5f"	−0.2399

"00074da4b8"	"00074dbf6d"	0.306
"00074da4b8"	"00074dc4a5"	−0.2716
"00074da4b8"	"00074dc50c"	0.2091
"00074da4b8"	"00074dcdfa"	−0.1415
"00074da4b8"	"00074dcf5f"	0.4681
"00074da4b8"	"00074dd007"	0.3495
"00074da4b8"	"00074dd163"	−0.2874
"00074da4b8"	"00074dd3df"	−0.3324
"00074da4b8"	"00074dd577"	−0.0194
"00074da4b8"	"00074dd62e"	0.0943
"00074da4b8"	"00074dd73c"	−0.1052
"00074da4b8"	"00074dda10"	−0.0443
"00074da4b8"	"00074ddab8"	0.0304
"00074da4b8"	"00074ddd3d"	0.1871
"00074da4b8"	"00074ddf16"	−0.1467
"00074da4b8"	"00074ddfc1"	−0.0389
"00074da4b8"	"00074de21a"	−0.1062
"00074da4b8"	"00074de2a9"	0.4632
"00074da4b8"	"00074de544"	−0.1533
"00074da4b8"	"00074de98a"	−0.0586
"00074da4b8"	"00074dea7e"	−0.3776
"00074da4b8"	"00074debd9"	−0.0429
"00074da4b8"	"00074deca3"	−0.2513
"00074da4b8"	"00074def43"	−0.072
"00074da4b8"	"00074def99"	−0.1464
"00074da4b8"	"00074ecdad"	−0.0163
"00074da4b8"	"00074ecf28"	0.0638
"00074da4b8"	"00074ed1e1"	−0.2469
"00074da4b8"	"00074ed83b"	0.1977
"00074da4b8"	"00074ee5e3"	−0.2075
"00074da4b8"	"00074ee6e0"	−0.0023
"00074da4b8"	"00074eea3a"	−0.3453
"00074da4b8"	"00074eff82"	−0.139
"00074da4b8"	"00074f0477"	−0.2063
"00074da4b8"	"00074f08c3"	0.2003
"00074da4b8"	"00074f1859"	0.0643
"00074da4b8"	"00074f2268"	−0.1339
"00074da4b8"	"00074f28be"	0.057
"00074da4b8"	"00074f294b"	−0.1832
"00074da4b8"	"00074f2ddd"	−0.1652
"00074da4b8"	"00074f2e75"	−0.139
"00074da4b8"	"00074f3088"	−0.1081
"00074da4b8"	"00074f5a1c"	−0.075
"00074da4b8"	"00074f75b7"	0.0268
"00074da4b8"	"00074f8cd9"	0.0934
"00074da4b8"	"00074f96dc"	−0.1036
"00074da4b8"	"00074fabaa"	0.0438
"00074da4b8"	"00074facd9"	−0.0501
"00074da4b8"	"00074fae3c"	−0.1423
"00074da4b8"	"00074fb0a8"	0.1982

"00074da4b8"	"00074fb4e4"	-0.0973
"00074da4b8"	"00074fb7c2"	0.0669
"00074da4b8"	"00074fbd36"	-0.081
"00074da4b8"	"00074fc27f"	0.2664
"00074da4b8"	"00074fc31d"	-0.1065
"00074da4b8"	"00074fd569"	0.0769
"00074da4b8"	"00074fef15"	-0.368
"00074da4b8"	"00074ff562"	-0.122
"00074da4b8"	"00075007ca"	0.3207
"00074da4b8"	"0007500b86"	0.1269
"00074da4b8"	"0007500d05"	0.016
"00074da4b8"	"0007500ee4"	-0.0467
"00074da4b8"	"0007500eee"	-0.2769
"00074da4b8"	"00075013dc"	0.0275
"00074da4b8"	"000757b515"	0.1215
"00074da4b8"	"000757bc5a"	0.1718
"00074da4b8"	"000757c320"	0.0547
"00074da4b8"	"000757c9aa"	0.284
"00074da4b8"	"000757ccbe"	-0.0441
"00074da4b8"	"000757cfa9"	0.0602
"00074da4b8"	"000757d390"	0.0754
"00074da4b8"	"000757d393"	0.0283
"00074da4b8"	"000757d598"	0.3147
"00074da4b8"	"000757d5a2"	0.0583
"00074da4b8"	"000757d790"	-0.1573
"00074da4b8"	"000757e30c"	0.5501
"00074da4b8"	"000757e4b0"	0.0518
"00074da4b8"	"000757e7a0"	0.097
"00074da4b8"	"000757e8b3"	0.1743
"00074da4b8"	"000757f627"	0.0794
"00074da4b8"	"000757f925"	0.1549
"00074da4b8"	"000757fa08"	0.038
"00074da4b8"	"000757fe52"	0.0384
"00074da4b8"	"000758024a"	0.8388
"00074da4b8"	"00075804bb"	0.2663
"00074da4b8"	"00075a0c04"	0.0073
"00074da4b8"	"00075a3110"	0.0541
"00074da4b8"	"00075a341a"	-0.0633
"00074da4b8"	"00075a3dcf"	-0.3363
"00074da4b8"	"00075a3e22"	0.1139
"00074da4b8"	"00075a48d8"	-0.0861
"00074da4b8"	"00075a5cfb"	0.5279
"00074da4b8"	"00075a6151"	0.0388
"00074da4b8"	"00075a6708"	0.028
"00074da4b8"	"00075a7319"	-0.0431
"00074da4b8"	"00075a7723"	0.0624
"00074da4b8"	"00075a778b"	0.2352
"00074da4b8"	"00075a7b8e"	0.2397
"00074da4b8"	"00075a7c79"	0.0169
"00074da4b8"	"00075a81b6"	-0.0996

"00074da4b8"	"00075a82ac"	-0.1729
"00074da4b8"	"00075a98e5"	-0.1234
"00074da4b8"	"00075b0d29"	0.1405
"00074da4b8"	"00075b102a"	0.2078
"00074da4b8"	"00075b1074"	-0.3378
"00074da4b8"	"00075b135d"	-0.0819
"00074da4b8"	"00075b138b"	0.1569
"00074da4b8"	"00075b13a0"	-0.2945
"00074da4b8"	"00075b13bd"	-0.2085
"00074da4b8"	"00075b16a9"	0.1478
"00074da4b8"	"00075b1a28"	-0.0688
"00074da4b8"	"00075b1a97"	-0.0905
"00074da4b8"	"00075b1c7b"	-0.044
"00074da4b8"	"00075b1d24"	0.0651
"00074da4b8"	"00075b202b"	0.4629
"00074da4b8"	"00075b22cb"	-0.1224
"00074da4b8"	"00075b22da"	-0.228
"00074da4b8"	"00075b2556"	-0.1083
"00074da4b8"	"00075b25de"	0.0467
"00074da4b8"	"00075b260c"	0.2003
"00074da4b8"	"00075b26f1"	-0.2419
"00074da4b8"	"00075b2920"	0.5324
"00074da4b8"	"00075b2a64"	0.4615
"00074da4b8"	"00075b2a9d"	0.1265
"00074da4b8"	"00075b2b37"	0.0194
"00074da4b8"	"00075b2cdd"	0.0293
"00074da4b8"	"00075b3038"	0.027
"00074da4b8"	"00075b30fe"	-0.2671
"00074da4b8"	"00075b3362"	-0.1829
"00074da4b8"	"00075b350a"	0.0218
"00074da4b8"	"00075b350e"	-0.0448
"00074da4b8"	"00075b3651"	-0.243
"00074da4b8"	"00075b38ca"	-0.3198
"00074da4b8"	"00075b39cc"	-0.2924
"00074da4b8"	"00075b3e1e"	0.0445
"00074da4b8"	"00075b3e57"	0.1792
"00074da4b8"	"00075b4079"	-0.0953
"00074da4b8"	"00075b4150"	0.7746
"00074da4b8"	"00075b4194"	0.0287
"00074da4b8"	"00075b42d5"	-0.0817
"00074da4b8"	"00075b4424"	0.2296
"00074da4b8"	"00075b4470"	0.1377
"00074da4b8"	"00075b47ed"	-0.3243
"00074da4b8"	"00075b4850"	0.0851
"00074da4b8"	"00075b4ca0"	-0.0324
"00074da4b8"	"00075b4d7f"	0.1677
"00074da4b8"	"00075b520f"	-0.0291
"00074da4b8"	"00075b525f"	-0.0402
"00074da4b8"	"00075b58f8"	0.2542
"00074da4b8"	"00075b5bcc"	0.086

"00074da4b8"	"00075b5bfa"	-0.295
"00074da4b8"	"00075b6339"	0.3206
"00074da4b8"	"00075b6658"	-0.3915
"00074da4b8"	"00075b679a"	0.1012
"00074da4b8"	"00075b6cb7"	-0.0617
"00074da4b8"	"00075b6df8"	-0.1529
"00074da4b8"	"00075b6ff6"	0.2257
"00074da4b8"	"00075b70ee"	0.2001
"00074da4b8"	"00075b7157"	0.0257
"00074da4b8"	"00075b7225"	-0.0313
"00074da4b8"	"00075b7c89"	-0.0284
"00074da4b8"	"00075b9048"	0.2528
"00074da4b8"	"00075d0801"	0.0134
"00074da4b8"	"00075d1820"	-0.0687
"00074da4b8"	"00075d1f3d"	0.1498
"00074da4b8"	"00075d2329"	-0.2322
"00074da4b8"	"00075d2b9b"	0.2708
"00074da4b8"	"00075d3941"	-0.2174
"00074da4b8"	"00075d3e96"	-0.0057
"00074da4b8"	"00075d4864"	-0.0538
"00074da4b8"	"00075d5961"	-0.1086
"00074da4b8"	"00075d5a63"	0.329
"00074da4b8"	"00075d6150"	-0.2924
"00074da4b8"	"00075d67d0"	-0.1298
"00074da4b8"	"00075d67e2"	0.0761
"00074da4b8"	"00075d73fc"	-0.1912
"00074da4b8"	"00075d7729"	0.0137
"00074da4b8"	"00075d778c"	-0.2379
"00074da4b8"	"00075d7b9e"	-0.0373
"00074da4b8"	"00075d7c8f"	-0.0683
"00074da4b8"	"00075d804d"	0.1568
"00074da4b8"	"00075d819f"	-0.1013
"00074da4b8"	"00075d8601"	-0.1077
"00074da4b8"	"00075d8c6a"	-0.017
"00074da4b8"	"00075dfedc"	0.07
"00074da4b8"	"00075e05f2"	0.0831
"00074da4b8"	"00075e0837"	0.0417
"00074da4b8"	"00075e092e"	-0.333
"00074da4b8"	"00075e0965"	0.3973
"00074da4b8"	"00075e0bc8"	0.2316
"00074da4b8"	"00075e0fbb"	-0.3516
"00074da5e8"	"00074da6b4"	0.2421
"00074da5e8"	"00074daa3c"	0.1689
"00074da5e8"	"00074daaf6"	0.1152
"00074da5e8"	"00074dad20"	-0.1115
"00074da5e8"	"00074db098"	0.3539
"00074da5e8"	"00074db231"	0.1525
"00074da5e8"	"00074db3a3"	0.2198
"00074da5e8"	"00074db5d6"	0.1512
"00074da5e8"	"00074db632"	0.3296

"00074da5e8"	"00074db688"	0.5431
"00074da5e8"	"00074db8a6"	-0.0423
"00074da5e8"	"00074dba19"	0.1258
"00074da5e8"	"00074dbc2e"	0.1899
"00074da5e8"	"00074dbe51"	0.4067
"00074da5e8"	"00074dbe5f"	-0.3668
"00074da5e8"	"00074dbf6d"	0.0673
"00074da5e8"	"00074dc4a5"	-0.2277
"00074da5e8"	"00074dc50c"	0.3189
"00074da5e8"	"00074dcdfa"	0.273
"00074da5e8"	"00074dcf5f"	0.098
"00074da5e8"	"00074dd007"	-0.0317
"00074da5e8"	"00074dd163"	0.0818
"00074da5e8"	"00074dd3df"	-0.329
"00074da5e8"	"00074dd577"	0.2912
"00074da5e8"	"00074dd62e"	0.1599
"00074da5e8"	"00074dd73c"	0.1519
"00074da5e8"	"00074dda10"	3e-04
"00074da5e8"	"00074ddab8"	0.201
"00074da5e8"	"00074ddd3d"	0.0499
"00074da5e8"	"00074ddf16"	0.0461
"00074da5e8"	"00074ddfc1"	-0.0307
"00074da5e8"	"00074de21a"	-0.1248
"00074da5e8"	"00074de2a9"	0.1633
"00074da5e8"	"00074de544"	0.2307
"00074da5e8"	"00074de98a"	-0.031
"00074da5e8"	"00074dea7e"	-0.1249
"00074da5e8"	"00074debd9"	-0.2044
"00074da5e8"	"00074deca3"	0.0462
"00074da5e8"	"00074def43"	-0.0342
"00074da5e8"	"00074def99"	0.2821
"00074da5e8"	"00074ecdad"	-0.0994
"00074da5e8"	"00074ecf28"	-0.2464
"00074da5e8"	"00074ed1e1"	0.016
"00074da5e8"	"00074ed83b"	0.2326
"00074da5e8"	"00074ee5e3"	-0.0323
"00074da5e8"	"00074ee6e0"	0.17
"00074da5e8"	"00074eea3a"	0.1277
"00074da5e8"	"00074eff82"	0.0607
"00074da5e8"	"00074f0477"	-0.185
"00074da5e8"	"00074f08c3"	0.0057
"00074da5e8"	"00074f1859"	0.0843
"00074da5e8"	"00074f2268"	0.0286
"00074da5e8"	"00074f28be"	0.4032
"00074da5e8"	"00074f294b"	0.0897
"00074da5e8"	"00074f2ddd"	0.393
"00074da5e8"	"00074f2e75"	-0.0774
"00074da5e8"	"00074f3088"	0.0937
"00074da5e8"	"00074f5a1c"	0.0639
"00074da5e8"	"00074f75b7"	0.0891

"00074da5e8"	"00074f8cd9"	0.1953
"00074da5e8"	"00074f96dc"	−0.1093
"00074da5e8"	"00074fabaa"	0.1915
"00074da5e8"	"00074facd9"	−0.0429
"00074da5e8"	"00074fae3c"	−0.1236
"00074da5e8"	"00074fb0a8"	0.1455
"00074da5e8"	"00074fb4e4"	−0.2823
"00074da5e8"	"00074fb7c2"	0.2508
"00074da5e8"	"00074fbd36"	0.0987
"00074da5e8"	"00074fc27f"	0.1269
"00074da5e8"	"00074fc31d"	0.0523
"00074da5e8"	"00074fd569"	−0.0802
"00074da5e8"	"00074fef15"	0.0049
"00074da5e8"	"00074ff562"	−0.2272
"00074da5e8"	"00075007ca"	0.0498
"00074da5e8"	"0007500b86"	0.2663
"00074da5e8"	"0007500d05"	−0.0339
"00074da5e8"	"0007500ee4"	−0.0563
"00074da5e8"	"0007500eee"	−0.3755
"00074da5e8"	"00075013dc"	0.0726
"00074da5e8"	"000757b515"	0.26
"00074da5e8"	"000757bc5a"	0.1966
"00074da5e8"	"000757c320"	−0.1114
"00074da5e8"	"000757c9aa"	0.032
"00074da5e8"	"000757ccbe"	0.1723
"00074da5e8"	"000757cfa9"	0.3323
"00074da5e8"	"000757d390"	−0.0035
"00074da5e8"	"000757d393"	0.1059
"00074da5e8"	"000757d598"	0.099
"00074da5e8"	"000757d5a2"	0.1222
"00074da5e8"	"000757d790"	0.1932
"00074da5e8"	"000757e30c"	0.2428
"00074da5e8"	"000757e4b0"	0.1098
"00074da5e8"	"000757e7a0"	0.0346
"00074da5e8"	"000757e8b3"	0.378
"00074da5e8"	"000757f627"	0.0849
"00074da5e8"	"000757f925"	0.091
"00074da5e8"	"000757fa08"	0.1153
"00074da5e8"	"000757fe52"	0.2422
"00074da5e8"	"000758024a"	0.1532
"00074da5e8"	"00075804bb"	0.1759
"00074da5e8"	"00075a0c04"	0.1768
"00074da5e8"	"00075a3110"	0.5545
"00074da5e8"	"00075a341a"	0.0023
"00074da5e8"	"00075a3dcf"	0.0674
"00074da5e8"	"00075a3e22"	0.2775
"00074da5e8"	"00075a48d8"	0.1165
"00074da5e8"	"00075a5cfb"	0.291
"00074da5e8"	"00075a6151"	0.0766
"00074da5e8"	"00075a6708"	0.1851

"00074da5e8"	"00075a7319"	-0.011
"00074da5e8"	"00075a7723"	-0.0246
"00074da5e8"	"00075a778b"	-0.0113
"00074da5e8"	"00075a7b8e"	0.0982
"00074da5e8"	"00075a7c79"	0.5511
"00074da5e8"	"00075a81b6"	0.0544
"00074da5e8"	"00075a82ac"	0.032
"00074da5e8"	"00075a98e5"	-0.1426
"00074da5e8"	"00075b0d29"	0.2758
"00074da5e8"	"00075b102a"	0.1324
"00074da5e8"	"00075b1074"	0.0431
"00074da5e8"	"00075b135d"	0.4258
"00074da5e8"	"00075b138b"	0.276
"00074da5e8"	"00075b13a0"	-0.2455
"00074da5e8"	"00075b13bd"	-0.0665
"00074da5e8"	"00075b16a9"	0.0369
"00074da5e8"	"00075b1a28"	0.121
"00074da5e8"	"00075b1a97"	0.1951
"00074da5e8"	"00075b1c7b"	-0.216
"00074da5e8"	"00075b1d24"	0.2758
"00074da5e8"	"00075b202b"	0.232
"00074da5e8"	"00075b22cb"	-0.0833
"00074da5e8"	"00075b22da"	0.1394
"00074da5e8"	"00075b2556"	-0.0571
"00074da5e8"	"00075b25de"	0.0615
"00074da5e8"	"00075b260c"	0.3273
"00074da5e8"	"00075b26f1"	-0.1358
"00074da5e8"	"00075b2920"	0.2105
"00074da5e8"	"00075b2a64"	0.1699
"00074da5e8"	"00075b2a9d"	0.189
"00074da5e8"	"00075b2b37"	-0.2731
"00074da5e8"	"00075b2cdd"	0.1809
"00074da5e8"	"00075b3038"	0.1896
"00074da5e8"	"00075b30fe"	0.0184
"00074da5e8"	"00075b3362"	-0.0861
"00074da5e8"	"00075b350a"	0.0961
"00074da5e8"	"00075b350e"	0.1626
"00074da5e8"	"00075b3651"	-0.0328
"00074da5e8"	"00075b38ca"	-0.0319
"00074da5e8"	"00075b39cc"	0.0733
"00074da5e8"	"00075b3e1e"	0.1023
"00074da5e8"	"00075b3e57"	-0.091
"00074da5e8"	"00075b4079"	0.0673
"00074da5e8"	"00075b4150"	0.1274
"00074da5e8"	"00075b4194"	0.4021
"00074da5e8"	"00075b42d5"	0.2457
"00074da5e8"	"00075b4424"	-0.1002
"00074da5e8"	"00075b4470"	0.1809
"00074da5e8"	"00075b47ed"	0.0229
"00074da5e8"	"00075b4850"	0.22

"00074da5e8"	"00075b4ca0"	0.2291
"00074da5e8"	"00075b4d7f"	0.3188
"00074da5e8"	"00075b520f"	-0.0718
"00074da5e8"	"00075b525f"	0.2173
"00074da5e8"	"00075b58f8"	0.0734
"00074da5e8"	"00075b5bcc"	0.2132
"00074da5e8"	"00075b5bfa"	-0.1121
"00074da5e8"	"00075b6339"	0.3115
"00074da5e8"	"00075b6658"	-0.2159
"00074da5e8"	"00075b679a"	0.3067
"00074da5e8"	"00075b6cb7"	0.0503
"00074da5e8"	"00075b6df8"	0.0619
"00074da5e8"	"00075b6ff6"	0.34
"00074da5e8"	"00075b70ee"	0.1234
"00074da5e8"	"00075b7157"	0.1746
"00074da5e8"	"00075b7225"	0.2374
"00074da5e8"	"00075b7c89"	0.269
"00074da5e8"	"00075b9048"	0.3375
"00074da5e8"	"00075d0801"	0.1694
"00074da5e8"	"00075d1820"	-0.0905
"00074da5e8"	"00075d1f3d"	0.3663
"00074da5e8"	"00075d2329"	0.0677
"00074da5e8"	"00075d2b9b"	0.158
"00074da5e8"	"00075d3941"	-0.1566
"00074da5e8"	"00075d3e96"	-0.019
"00074da5e8"	"00075d4864"	-0.0607
"00074da5e8"	"00075d5961"	0.0742
"00074da5e8"	"00075d5a63"	0.2579
"00074da5e8"	"00075d6150"	0.0168
"00074da5e8"	"00075d67d0"	-0.0687
"00074da5e8"	"00075d67e2"	0.0222
"00074da5e8"	"00075d73fc"	0.1635
"00074da5e8"	"00075d7729"	0.1017
"00074da5e8"	"00075d778c"	-0.1692
"00074da5e8"	"00075d7b9e"	0.0797
"00074da5e8"	"00075d7c8f"	0.5333
"00074da5e8"	"00075d804d"	0.0955
"00074da5e8"	"00075d819f"	0.1818
"00074da5e8"	"00075d8601"	-0.0532
"00074da5e8"	"00075d8c6a"	0.1145
"00074da5e8"	"00075dfedc"	0.3125
"00074da5e8"	"00075e05f2"	0.0352
"00074da5e8"	"00075e0837"	0.1096
"00074da5e8"	"00075e092e"	0.1265
"00074da5e8"	"00075e0965"	0.0338
"00074da5e8"	"00075e0bc8"	0.255
"00074da5e8"	"00075e0fbb"	0.0747
"00074da6b4"	"00074daa3c"	-0.0908
"00074da6b4"	"00074daaf6"	-0.2347
"00074da6b4"	"00074dad20"	0.0106

"00074da6b4"	"00074db098"	-0.1454
"00074da6b4"	"00074db231"	0.1985
"00074da6b4"	"00074db3a3"	-0.0569
"00074da6b4"	"00074db5d6"	-0.0564
"00074da6b4"	"00074db632"	0.2195
"00074da6b4"	"00074db688"	0.0693
"00074da6b4"	"00074db8a6"	-0.5588
"00074da6b4"	"00074dba19"	0.1202
"00074da6b4"	"00074dbc2e"	-0.138
"00074da6b4"	"00074dbe51"	0.2345
"00074da6b4"	"00074dbe5f"	-0.2412
"00074da6b4"	"00074dbf6d"	-0.237
"00074da6b4"	"00074dc4a5"	-0.0017
"00074da6b4"	"00074dc50c"	0.1001
"00074da6b4"	"00074dcdfa"	-0.052
"00074da6b4"	"00074dcf5f"	0.1319
"00074da6b4"	"00074dd007"	-0.1049
"00074da6b4"	"00074dd163"	-0.5321
"00074da6b4"	"00074dd3df"	-0.0153
"00074da6b4"	"00074dd577"	0.1271
"00074da6b4"	"00074dd62e"	-0.0401
"00074da6b4"	"00074dd73c"	-0.1299
"00074da6b4"	"00074dda10"	0.2117
"00074da6b4"	"00074ddab8"	0.2116
"00074da6b4"	"00074ddd3d"	-0.1252
"00074da6b4"	"00074ddf16"	-0.2813
"00074da6b4"	"00074ddfc1"	-0.2085
"00074da6b4"	"00074de21a"	-0.0785
"00074da6b4"	"00074de2a9"	0.0443
"00074da6b4"	"00074de544"	0.204
"00074da6b4"	"00074de98a"	0.1662
"00074da6b4"	"00074dea7e"	0.0447
"00074da6b4"	"00074debd9"	-0.4212
"00074da6b4"	"00074deca3"	-0.5209
"00074da6b4"	"00074def43"	-0.3279
"00074da6b4"	"00074def99"	0.1455
"00074da6b4"	"00074ecdad"	-0.2466
"00074da6b4"	"00074ecf28"	-9e-04
"00074da6b4"	"00074ed1e1"	0.0338
"00074da6b4"	"00074ed83b"	-0.0207
"00074da6b4"	"00074ee5e3"	-0.4047
"00074da6b4"	"00074ee6e0"	0.2067
"00074da6b4"	"00074eea3a"	0.2115
"00074da6b4"	"00074eff82"	-0.0262
"00074da6b4"	"00074f0477"	0.0181
"00074da6b4"	"00074f08c3"	0.0158
"00074da6b4"	"00074f1859"	0.0031
"00074da6b4"	"00074f2268"	-0.1491
"00074da6b4"	"00074f28be"	0.1257
"00074da6b4"	"00074f294b"	0.1457

"00074da6b4"	"00074f2ddd"	−0.0424
"00074da6b4"	"00074f2e75"	0.2645
"00074da6b4"	"00074f3088"	0.0117
"00074da6b4"	"00074f5a1c"	−0.1045
"00074da6b4"	"00074f75b7"	−0.0917
"00074da6b4"	"00074f8cd9"	0.1794
"00074da6b4"	"00074f96dc"	−0.1985
"00074da6b4"	"00074fabaa"	0.0463
"00074da6b4"	"00074facd9"	−0.0152
"00074da6b4"	"00074fae3c"	−0.0826
"00074da6b4"	"00074fb0a8"	0.105
"00074da6b4"	"00074fb4e4"	−0.3047
"00074da6b4"	"00074fb7c2"	0.6013
"00074da6b4"	"00074fbd36"	−0.1765
"00074da6b4"	"00074fc27f"	−0.0514
"00074da6b4"	"00074fc31d"	0.0186
"00074da6b4"	"00074fd569"	0.0859
"00074da6b4"	"00074fef15"	−0.063
"00074da6b4"	"00074ff562"	−0.405
"00074da6b4"	"00075007ca"	−0.0532
"00074da6b4"	"0007500b86"	−0.086
"00074da6b4"	"0007500d05"	0.0682
"00074da6b4"	"0007500ee4"	−0.0667
"00074da6b4"	"0007500eee"	−0.1405
"00074da6b4"	"00075013dc"	−0.1307
"00074da6b4"	"000757b515"	0.1633
"00074da6b4"	"000757bc5a"	0.2728
"00074da6b4"	"000757c320"	−0.3268
"00074da6b4"	"000757c9aa"	0.0088
"00074da6b4"	"000757ccbe"	0.0554
"00074da6b4"	"000757cfa9"	−0.2312
"00074da6b4"	"000757d390"	−0.0177
"00074da6b4"	"000757d393"	−0.1237
"00074da6b4"	"000757d598"	−0.1933
"00074da6b4"	"000757d5a2"	−0.1965
"00074da6b4"	"000757d790"	0.1106
"00074da6b4"	"000757e30c"	0.1719
"00074da6b4"	"000757e4b0"	0.0852
"00074da6b4"	"000757e7a0"	−0.1088
"00074da6b4"	"000757e8b3"	0.0162
"00074da6b4"	"000757f627"	0.0583
"00074da6b4"	"000757f925"	0.0147
"00074da6b4"	"000757fa08"	0.3802
"00074da6b4"	"000757fe52"	0.097
"00074da6b4"	"000758024a"	0.2013
"00074da6b4"	"00075804bb"	−0.12
"00074da6b4"	"00075a0c04"	0.2324
"00074da6b4"	"00075a3110"	0.2504
"00074da6b4"	"00075a341a"	0.0843
"00074da6b4"	"00075a3dcf"	0.0457

"00074da6b4"	"00075a3e22"	0.2957
"00074da6b4"	"00075a48d8"	0.1528
"00074da6b4"	"00075a5cfb"	0.1408
"00074da6b4"	"00075a6151"	−0.2045
"00074da6b4"	"00075a6708"	0.0749
"00074da6b4"	"00075a7319"	−0.1424
"00074da6b4"	"00075a7723"	0.1715
"00074da6b4"	"00075a778b"	0.503
"00074da6b4"	"00075a7b8e"	0.4915
"00074da6b4"	"00075a7c79"	0.134
"00074da6b4"	"00075a81b6"	0.0755
"00074da6b4"	"00075a82ac"	0.3397
"00074da6b4"	"00075a98e5"	−0.0087
"00074da6b4"	"00075b0d29"	0.0388
"00074da6b4"	"00075b102a"	−0.04
"00074da6b4"	"00075b1074"	0.1562
"00074da6b4"	"00075b135d"	0.0955
"00074da6b4"	"00075b138b"	0.2052
"00074da6b4"	"00075b13a0"	0.1267
"00074da6b4"	"00075b13bd"	0.0364
"00074da6b4"	"00075b16a9"	−0.0697
"00074da6b4"	"00075b1a28"	−0.1707
"00074da6b4"	"00075b1a97"	−0.0467
"00074da6b4"	"00075b1c7b"	−0.3288
"00074da6b4"	"00075b1d24"	0.2219
"00074da6b4"	"00075b202b"	0.1566
"00074da6b4"	"00075b22cb"	−0.0936
"00074da6b4"	"00075b22da"	−0.0073
"00074da6b4"	"00075b2556"	−0.0715
"00074da6b4"	"00075b25de"	−0.0314
"00074da6b4"	"00075b260c"	0.0062
"00074da6b4"	"00075b26f1"	−0.1518
"00074da6b4"	"00075b2920"	0.0781
"00074da6b4"	"00075b2a64"	0.0336
"00074da6b4"	"00075b2a9d"	0.0792
"00074da6b4"	"00075b2b37"	−0.2383
"00074da6b4"	"00075b2cdd"	0.1425
"00074da6b4"	"00075b3038"	0.0381
"00074da6b4"	"00075b30fe"	−0.0671
"00074da6b4"	"00075b3362"	−0.043
"00074da6b4"	"00075b350a"	−0.0658
"00074da6b4"	"00075b350e"	−0.0525
"00074da6b4"	"00075b3651"	−0.142
"00074da6b4"	"00075b38ca"	−0.2166
"00074da6b4"	"00075b39cc"	−0.1529
"00074da6b4"	"00075b3e1e"	−0.0298
"00074da6b4"	"00075b3e57"	0.1327
"00074da6b4"	"00075b4079"	−0.1598
"00074da6b4"	"00075b4150"	0.2475
"00074da6b4"	"00075b4194"	0.0538

"00074da6b4"	"00075b42d5"	-0.0052
"00074da6b4"	"00075b4424"	-0.1349
"00074da6b4"	"00075b4470"	0.3069
"00074da6b4"	"00075b47ed"	0.0586
"00074da6b4"	"00075b4850"	0.1023
"00074da6b4"	"00075b4ca0"	0.0441
"00074da6b4"	"00075b4d7f"	0.3247
"00074da6b4"	"00075b520f"	-0.1017
"00074da6b4"	"00075b525f"	-0.0587
"00074da6b4"	"00075b58f8"	-0.0705
"00074da6b4"	"00075b5bcc"	0.0602
"00074da6b4"	"00075b5bfa"	-0.2337
"00074da6b4"	"00075b6339"	0.1109
"00074da6b4"	"00075b6658"	-0.2859
"00074da6b4"	"00075b679a"	0.0173
"00074da6b4"	"00075b6cb7"	0.0025
"00074da6b4"	"00075b6df8"	0.1933
"00074da6b4"	"00075b6ff6"	0.1513
"00074da6b4"	"00075b70ee"	-0.0753
"00074da6b4"	"00075b7157"	0.0883
"00074da6b4"	"00075b7225"	0.0462
"00074da6b4"	"00075b7c89"	0.1798
"00074da6b4"	"00075b9048"	0.2984
"00074da6b4"	"00075d0801"	0.0262
"00074da6b4"	"00075d1820"	0.0395
"00074da6b4"	"00075d1f3d"	0.0305
"00074da6b4"	"00075d2329"	0.2011
"00074da6b4"	"00075d2b9b"	0.0148
"00074da6b4"	"00075d3941"	-0.1827
"00074da6b4"	"00075d3e96"	8e-04
"00074da6b4"	"00075d4864"	-0.1098
"00074da6b4"	"00075d5961"	0.1061
"00074da6b4"	"00075d5a63"	0.2179
"00074da6b4"	"00075d6150"	-0.3751
"00074da6b4"	"00075d67d0"	0.0704
"00074da6b4"	"00075d67e2"	-0.0521
"00074da6b4"	"00075d73fc"	0.0066
"00074da6b4"	"00075d7729"	0.1356
"00074da6b4"	"00075d778c"	-0.1908
"00074da6b4"	"00075d7b9e"	0.1686
"00074da6b4"	"00075d7c8f"	0.1583
"00074da6b4"	"00075d804d"	0.1593
"00074da6b4"	"00075d819f"	0.0578
"00074da6b4"	"00075d8601"	-0.2092
"00074da6b4"	"00075d8c6a"	3e-04
"00074da6b4"	"00075dfedc"	0.0069
"00074da6b4"	"00075e05f2"	-0.1906
"00074da6b4"	"00075e0837"	0.0539
"00074da6b4"	"00075e092e"	0.0468
"00074da6b4"	"00075e0965"	0.0443

"00074da6b4"	"00075e0bc8"	0.2866
"00074da6b4"	"00075e0fbb"	0.0124
"00074daa3c"	"00074daaf6"	−0.1696
"00074daa3c"	"00074dad20"	−0.3075
"00074daa3c"	"00074db098"	0.005
"00074daa3c"	"00074db231"	0.0374
"00074daa3c"	"00074db3a3"	0.4081
"00074daa3c"	"00074db5d6"	0.0882
"00074daa3c"	"00074db632"	0.1244
"00074daa3c"	"00074db688"	−0.387
"00074daa3c"	"00074db8a6"	−0.2244
"00074daa3c"	"00074dba19"	−0.1174
"00074daa3c"	"00074dbc2e"	0.0163
"00074daa3c"	"00074dbe51"	−0.1181
"00074daa3c"	"00074dbe5f"	−0.1098
"00074daa3c"	"00074dbf6d"	0.1506
"00074daa3c"	"00074dc4a5"	−0.5889
"00074daa3c"	"00074dc50c"	0.2629
"00074daa3c"	"00074dcdfa"	0.0915
"00074daa3c"	"00074dcf5f"	−0.1091
"00074daa3c"	"00074dd007"	0.1336
"00074daa3c"	"00074dd163"	−0.0886
"00074daa3c"	"00074dd3df"	−0.5191
"00074daa3c"	"00074dd577"	−0.0822
"00074daa3c"	"00074dd62e"	0.0401
"00074daa3c"	"00074dd73c"	−0.2607
"00074daa3c"	"00074dda10"	−0.1002
"00074daa3c"	"00074ddab8"	0.2072
"00074daa3c"	"00074ddd3d"	−0.2186
"00074daa3c"	"00074ddf16"	−0.074
"00074daa3c"	"00074ddfc1"	−0.1384
"00074daa3c"	"00074de21a"	−0.2396
"00074daa3c"	"00074de2a9"	0.1141
"00074daa3c"	"00074de544"	−0.0266
"00074daa3c"	"00074de98a"	−0.2215
"00074daa3c"	"00074dea7e"	−0.4695
"00074daa3c"	"00074debd9"	0.126
"00074daa3c"	"00074deca3"	0.0102
"00074daa3c"	"00074def43"	−0.1452
"00074daa3c"	"00074def99"	−0.3411
"00074daa3c"	"00074ecdad"	−0.5132
"00074daa3c"	"00074ecf28"	−0.3923
"00074daa3c"	"00074ed1e1"	−0.2165
"00074daa3c"	"00074ed83b"	0.0756
"00074daa3c"	"00074ee5e3"	0.1959
"00074daa3c"	"00074ee6e0"	−0.0199
"00074daa3c"	"00074eea3a"	−0.1582
"00074daa3c"	"00074eff82"	−0.1589
"00074daa3c"	"00074f0477"	−0.21
"00074daa3c"	"00074f08c3"	−0.0223

"00074daa3c"	"00074f1859"	−0.0322
"00074daa3c"	"00074f2268"	−0.2976
"00074daa3c"	"00074f28be"	0.1451
"00074daa3c"	"00074f294b"	−0.529
"00074daa3c"	"00074f2ddd"	−0.1363
"00074daa3c"	"00074f2e75"	−0.546
"00074daa3c"	"00074f3088"	−0.1302
"00074daa3c"	"00074f5a1c"	−0.1627
"00074daa3c"	"00074f75b7"	0.1658
"00074daa3c"	"00074f8cd9"	−0.108
"00074daa3c"	"00074f96dc"	−0.322
"00074daa3c"	"00074fabaa"	0.4756
"00074daa3c"	"00074facd9"	0.0836
"00074daa3c"	"00074fae3c"	−0.3293
"00074daa3c"	"00074fb0a8"	0.0235
"00074daa3c"	"00074fb4e4"	−0.0656
"00074daa3c"	"00074fb7c2"	−0.0952
"00074daa3c"	"00074fbd36"	−0.3963
"00074daa3c"	"00074fc27f"	0.1774
"00074daa3c"	"00074fc31d"	−0.3251
"00074daa3c"	"00074fd569"	−0.0638
"00074daa3c"	"00074fef15"	−0.4451
"00074daa3c"	"00074ff562"	−0.3418
"00074daa3c"	"00075007ca"	0.0883
"00074daa3c"	"0007500b86"	0.0298
"00074daa3c"	"0007500d05"	0.0819
"00074daa3c"	"0007500ee4"	−0.2361
"00074daa3c"	"0007500eee"	−0.0639
"00074daa3c"	"00075013dc"	−0.0533
"00074daa3c"	"000757b515"	0.1287
"00074daa3c"	"000757bc5a"	0.0101
"00074daa3c"	"000757c320"	0.093
"00074daa3c"	"000757c9aa"	0.0885
"00074daa3c"	"000757ccbe"	−0.323
"00074daa3c"	"000757cfa9"	−0.2184
"00074daa3c"	"000757d390"	0.1058
"00074daa3c"	"000757d393"	−0.0097
"00074daa3c"	"000757d598"	0.1508
"00074daa3c"	"000757d5a2"	0.0088
"00074daa3c"	"000757d790"	−0.0923
"00074daa3c"	"000757e30c"	0.0503
"00074daa3c"	"000757e4b0"	0.0011
"00074daa3c"	"000757e7a0"	−0.2828
"00074daa3c"	"000757e8b3"	0.0508
"00074daa3c"	"000757f627"	−0.0434
"00074daa3c"	"000757f925"	−0.0287
"00074daa3c"	"000757fa08"	−0.4925
"00074daa3c"	"000757fe52"	0.103
"00074daa3c"	"000758024a"	−0.0897
"00074daa3c"	"00075804bb"	0.1529

"00074daa3c"	"00075a0c04"	−0.1396
"00074daa3c"	"00075a3110"	0.1295
"00074daa3c"	"00075a341a"	−0.4722
"00074daa3c"	"00075a3dcf"	−0.2966
"00074daa3c"	"00075a3e22"	0.0064
"00074daa3c"	"00075a48d8"	−0.2228
"00074daa3c"	"00075a5cfb"	0.1185
"00074daa3c"	"00075a6151"	−0.0215
"00074daa3c"	"00075a6708"	−0.2132
"00074daa3c"	"00075a7319"	−0.0574
"00074daa3c"	"00075a7723"	−0.3162
"00074daa3c"	"00075a778b"	−0.3528
"00074daa3c"	"00075a7b8e"	−0.0192
"00074daa3c"	"00075a7c79"	0.222
"00074daa3c"	"00075a81b6"	−0.3541
"00074daa3c"	"00075a82ac"	0.0192
"00074daa3c"	"00075a98e5"	−0.4195
"00074daa3c"	"00075b0d29"	−0.1897
"00074daa3c"	"00075b102a"	0.1598
"00074daa3c"	"00075b1074"	−0.4947
"00074daa3c"	"00075b135d"	−0.1124
"00074daa3c"	"00075b138b"	0.3528
"00074daa3c"	"00075b13a0"	−0.4186
"00074daa3c"	"00075b13bd"	−0.3552
"00074daa3c"	"00075b16a9"	−0.0885
"00074daa3c"	"00075b1a28"	−0.2892
"00074daa3c"	"00075b1a97"	−0.2476
"00074daa3c"	"00075b1c7b"	−0.3893
"00074daa3c"	"00075b1d24"	−0.1942
"00074daa3c"	"00075b202b"	0.2763
"00074daa3c"	"00075b22cb"	−0.2156
"00074daa3c"	"00075b22da"	−0.3401
"00074daa3c"	"00075b2556"	−0.1797
"00074daa3c"	"00075b25de"	0.0667
"00074daa3c"	"00075b260c"	0.4065
"00074daa3c"	"00075b26f1"	−0.3565
"00074daa3c"	"00075b2920"	0.181
"00074daa3c"	"00075b2a64"	0.385
"00074daa3c"	"00075b2a9d"	0.0629
"00074daa3c"	"00075b2b37"	−0.496
"00074daa3c"	"00075b2cdd"	0.0042
"00074daa3c"	"00075b3038"	0.2329
"00074daa3c"	"00075b30fe"	−0.2045
"00074daa3c"	"00075b3362"	−0.3511
"00074daa3c"	"00075b350a"	−0.0545
"00074daa3c"	"00075b350e"	0.1775
"00074daa3c"	"00075b3651"	−0.1898
"00074daa3c"	"00075b38ca"	−0.2825
"00074daa3c"	"00075b39cc"	−0.118
"00074daa3c"	"00075b3e1e"	−0.0857

"00074daa3c"	"00075b3e57"	−0.1996
"00074daa3c"	"00075b4079"	−0.1234
"00074daa3c"	"00075b4150"	−0.0201
"00074daa3c"	"00075b4194"	0.2829
"00074daa3c"	"00075b42d5"	−0.2459
"00074daa3c"	"00075b4424"	−0.3643
"00074daa3c"	"00075b4470"	−0.0055
"00074daa3c"	"00075b47ed"	−0.2502
"00074daa3c"	"00075b4850"	0.082
"00074daa3c"	"00075b4ca0"	−0.0539
"00074daa3c"	"00075b4d7f"	−0.0926
"00074daa3c"	"00075b520f"	−0.3354
"00074daa3c"	"00075b525f"	−0.3105
"00074daa3c"	"00075b58f8"	0.0729
"00074daa3c"	"00075b5bcc"	0.1293
"00074daa3c"	"00075b5bfa"	0.0657
"00074daa3c"	"00075b6339"	−0.1035
"00074daa3c"	"00075b6658"	−0.5138
"00074daa3c"	"00075b679a"	−0.039
"00074daa3c"	"00075b6cb7"	−0.0126
"00074daa3c"	"00075b6df8"	−0.3484
"00074daa3c"	"00075b6ff6"	0.026
"00074daa3c"	"00075b70ee"	−0.1216
"00074daa3c"	"00075b7157"	0.1165
"00074daa3c"	"00075b7225"	−0.1577
"00074daa3c"	"00075b7c89"	−0.1183
"00074daa3c"	"00075b9048"	0.4771
"00074daa3c"	"00075d0801"	0.0993
"00074daa3c"	"00075d1820"	−0.3559
"00074daa3c"	"00075d1f3d"	0.3561
"00074daa3c"	"00075d2329"	−0.2398
"00074daa3c"	"00075d2b9b"	0.4231
"00074daa3c"	"00075d3941"	−0.2486
"00074daa3c"	"00075d3e96"	−0.1472
"00074daa3c"	"00075d4864"	−0.5896
"00074daa3c"	"00075d5961"	−0.1089
"00074daa3c"	"00075d5a63"	0.1122
"00074daa3c"	"00075d6150"	−0.1407
"00074daa3c"	"00075d67d0"	−0.1346
"00074daa3c"	"00075d67e2"	−0.2406
"00074daa3c"	"00075d73fc"	−0.1989
"00074daa3c"	"00075d7729"	−0.2827
"00074daa3c"	"00075d778c"	−0.52
"00074daa3c"	"00075d7b9e"	−0.0448
"00074daa3c"	"00075d7c8f"	−0.0744
"00074daa3c"	"00075d804d"	0.1591
"00074daa3c"	"00075d819f"	0.3242
"00074daa3c"	"00075d8601"	−0.0861
"00074daa3c"	"00075d8c6a"	−0.2662
"00074daa3c"	"00075dfedc"	0.0134

"00074daa3c"	"00075e05f2"	0.0425
"00074daa3c"	"00075e0837"	0.1467
"00074daa3c"	"00075e092e"	-0.2534
"00074daa3c"	"00075e0965"	-0.1289
"00074daa3c"	"00075e0bc8"	0.1039
"00074daa3c"	"00075e0fbb"	-0.3447
"00074daaf6"	"00074dad20"	0.0101
"00074daaf6"	"00074db098"	-0.0114
"00074daaf6"	"00074db231"	-0.1213
"00074daaf6"	"00074db3a3"	-0.0104
"00074daaf6"	"00074db5d6"	0.1943
"00074daaf6"	"00074db632"	-0.0431
"00074daaf6"	"00074db688"	-0.0018
"00074daaf6"	"00074db8a6"	-0.0483
"00074daaf6"	"00074dba19"	-0.1909
"00074daaf6"	"00074dbc2e"	-0.098
"00074daaf6"	"00074dbe51"	0.0669
"00074daaf6"	"00074dbe5f"	-0.3576
"00074daaf6"	"00074dbf6d"	-0.2217
"00074daaf6"	"00074dc4a5"	-0.1813
"00074daaf6"	"00074dc50c"	-0.362
"00074daaf6"	"00074dcdfa"	0.1055
"00074daaf6"	"00074dcf5f"	-0.2119
"00074daaf6"	"00074dd007"	-0.0778
"00074daaf6"	"00074dd163"	-0.1033
"00074daaf6"	"00074dd3df"	-0.0921
"00074daaf6"	"00074dd577"	-0.0942
"00074daaf6"	"00074dd62e"	0.2181
"00074daaf6"	"00074dd73c"	-0.0901
"00074daaf6"	"00074dda10"	-0.0484
"00074daaf6"	"00074ddab8"	0.0305
"00074daaf6"	"00074ddd3d"	-0.092
"00074daaf6"	"00074ddf16"	-1e-04
"00074daaf6"	"00074ddfc1"	-0.1589
"00074daaf6"	"00074de21a"	-0.0635
"00074daaf6"	"00074de2a9"	-0.1431
"00074daaf6"	"00074de544"	0.0446
"00074daaf6"	"00074de98a"	-0.312
"00074daaf6"	"00074dea7e"	-0.1208
"00074daaf6"	"00074debd9"	-0.3612
"00074daaf6"	"00074deca3"	0.0383
"00074daaf6"	"00074def43"	-0.1221
"00074daaf6"	"00074def99"	0.1464
"00074daaf6"	"00074ecdad"	-0.3705
"00074daaf6"	"00074ecf28"	-0.0932
"00074daaf6"	"00074ed1e1"	-0.1518
"00074daaf6"	"00074ed83b"	-0.037
"00074daaf6"	"00074ee5e3"	0.0775
"00074daaf6"	"00074ee6e0"	0.378
"00074daaf6"	"00074eea3a"	0.0219

"00074daaf6"	"00074eff82"	0.2715
"00074daaf6"	"00074f0477"	-0.1966
"00074daaf6"	"00074f08c3"	-0.0155
"00074daaf6"	"00074f1859"	-0.0864
"00074daaf6"	"00074f2268"	-0.0756
"00074daaf6"	"00074f28be"	0.0708
"00074daaf6"	"00074f294b"	-0.0364
"00074daaf6"	"00074f2ddd"	-0.09
"00074daaf6"	"00074f2e75"	-0.5491
"00074daaf6"	"00074f3088"	0.0798
"00074daaf6"	"00074f5a1c"	-0.1723
"00074daaf6"	"00074f75b7"	0.0736
"00074daaf6"	"00074f8cd9"	0.0175
"00074daaf6"	"00074f96dc"	0.0497
"00074daaf6"	"00074fabaa"	-0.2493
"00074daaf6"	"00074facd9"	0.0134
"00074daaf6"	"00074fae3c"	0.0533
"00074daaf6"	"00074fb0a8"	0.1174
"00074daaf6"	"00074fb4e4"	0.0435
"00074daaf6"	"00074fb7c2"	-0.1612
"00074daaf6"	"00074fbd36"	-0.1541
"00074daaf6"	"00074fc27f"	-0.1751
"00074daaf6"	"00074fc31d"	0.0258
"00074daaf6"	"00074fd569"	-7e-04
"00074daaf6"	"00074fef15"	0.4908
"00074daaf6"	"00074ff562"	-0.4848
"00074daaf6"	"00075007ca"	-0.2069
"00074daaf6"	"0007500b86"	0.0939
"00074daaf6"	"0007500d05"	-0.0219
"00074daaf6"	"0007500ee4"	0.132
"00074daaf6"	"0007500eee"	-0.1072
"00074daaf6"	"00075013dc"	-0.0797
"00074daaf6"	"000757b515"	-0.1464
"00074daaf6"	"000757bc5a"	-0.0571
"00074daaf6"	"000757c320"	-0.1359
"00074daaf6"	"000757c9aa"	-0.0026
"00074daaf6"	"000757ccbe"	-0.179
"00074daaf6"	"000757cfa9"	-0.2468
"00074daaf6"	"000757d390"	0.0244
"00074daaf6"	"000757d393"	-0.0668
"00074daaf6"	"000757d598"	-0.1442
"00074daaf6"	"000757d5a2"	-0.1146
"00074daaf6"	"000757d790"	0.0135
"00074daaf6"	"000757e30c"	-0.2307
"00074daaf6"	"000757e4b0"	0.0519
"00074daaf6"	"000757e7a0"	0.0264
"00074daaf6"	"000757e8b3"	-0.0685
"00074daaf6"	"000757f627"	0.1151
"00074daaf6"	"000757f925"	-0.0372
"00074daaf6"	"000757fa08"	-0.2968

"00074daaf6"	"000757fe52"	-0.0763
"00074daaf6"	"000758024a"	-0.4359
"00074daaf6"	"00075804bb"	-0.3001
"00074daaf6"	"00075a0c04"	-0.0504
"00074daaf6"	"00075a3110"	-0.1104
"00074daaf6"	"00075a341a"	-0.1031
"00074daaf6"	"00075a3dcf"	-0.1263
"00074daaf6"	"00075a3e22"	-0.106
"00074daaf6"	"00075a48d8"	-0.1303
"00074daaf6"	"00075a5cfb"	-0.2124
"00074daaf6"	"00075a6151"	-0.3197
"00074daaf6"	"00075a6708"	-0.1705
"00074daaf6"	"00075a7319"	0.0541
"00074daaf6"	"00075a7723"	-0.2401
"00074daaf6"	"00075a778b"	-0.2645
"00074daaf6"	"00075a7b8e"	-0.0372
"00074daaf6"	"00075a7c79"	0.0878
"00074daaf6"	"00075a81b6"	-0.1195
"00074daaf6"	"00075a82ac"	0.0144
"00074daaf6"	"00075a98e5"	-0.008
"00074daaf6"	"00075b0d29"	-0.2506
"00074daaf6"	"00075b102a"	-0.186
"00074daaf6"	"00075b1074"	-0.2115
"00074daaf6"	"00075b135d"	-0.2328
"00074daaf6"	"00075b138b"	0.0536
"00074daaf6"	"00075b13a0"	-0.4115
"00074daaf6"	"00075b13bd"	0.0533
"00074daaf6"	"00075b16a9"	-0.0991
"00074daaf6"	"00075b1a28"	0.0275
"00074daaf6"	"00075b1a97"	0.562
"00074daaf6"	"00075b1c7b"	-0.3128
"00074daaf6"	"00075b1d24"	0.4279
"00074daaf6"	"00075b202b"	-0.1073
"00074daaf6"	"00075b22cb"	-0.0238
"00074daaf6"	"00075b22da"	-0.0866
"00074daaf6"	"00075b2556"	-0.1108
"00074daaf6"	"00075b25de"	0.0651
"00074daaf6"	"00075b260c"	-0.1774
"00074daaf6"	"00075b26f1"	0.0259
"00074daaf6"	"00075b2920"	-0.1833
"00074daaf6"	"00075b2a64"	-0.2606
"00074daaf6"	"00075b2a9d"	-0.0725
"00074daaf6"	"00075b2b37"	-0.1414
"00074daaf6"	"00075b2cdd"	-0.0097
"00074daaf6"	"00075b3038"	-0.005
"00074daaf6"	"00075b30fe"	-0.0665
"00074daaf6"	"00075b3362"	0.122
"00074daaf6"	"00075b350a"	0.016
"00074daaf6"	"00075b350e"	0.009
"00074daaf6"	"00075b3651"	0.3646

"00074daaf6"	"00075b38ca"	−0.1796
"00074daaf6"	"00075b39cc"	0.0994
"00074daaf6"	"00075b3e1e"	−0.0376
"00074daaf6"	"00075b3e57"	−0.0013
"00074daaf6"	"00075b4079"	−0.1845
"00074daaf6"	"00075b4150"	−0.3247
"00074daaf6"	"00075b4194"	0.0886
"00074daaf6"	"00075b42d5"	−0.0219
"00074daaf6"	"00075b4424"	−0.1869
"00074daaf6"	"00075b4470"	−0.0728
"00074daaf6"	"00075b47ed"	0.0301
"00074daaf6"	"00075b4850"	−0.0162
"00074daaf6"	"00075b4ca0"	−0.3191
"00074daaf6"	"00075b4d7f"	0.0743
"00074daaf6"	"00075b520f"	0.1318
"00074daaf6"	"00075b525f"	0.2446
"00074daaf6"	"00075b58f8"	−0.4165
"00074daaf6"	"00075b5bcc"	0.2709
"00074daaf6"	"00075b5bfa"	−0.0824
"00074daaf6"	"00075b6339"	0.0651
"00074daaf6"	"00075b6658"	0.0428
"00074daaf6"	"00075b679a"	0.3677
"00074daaf6"	"00075b6cb7"	−0.3792
"00074daaf6"	"00075b6df8"	−0.1657
"00074daaf6"	"00075b6ff6"	0.1123
"00074daaf6"	"00075b70ee"	−0.2674
"00074daaf6"	"00075b7157"	0.2031
"00074daaf6"	"00075b7225"	0.2367
"00074daaf6"	"00075b7c89"	−0.1624
"00074daaf6"	"00075b9048"	−0.0249
"00074daaf6"	"00075d0801"	−0.1741
"00074daaf6"	"00075d1820"	0.2141
"00074daaf6"	"00075d1f3d"	−0.1706
"00074daaf6"	"00075d2329"	−0.1526
"00074daaf6"	"00075d2b9b"	−0.3615
"00074daaf6"	"00075d3941"	−0.1962
"00074daaf6"	"00075d3e96"	0.0842
"00074daaf6"	"00075d4864"	−0.2922
"00074daaf6"	"00075d5961"	0.047
"00074daaf6"	"00075d5a63"	0.0032
"00074daaf6"	"00075d6150"	0.3824
"00074daaf6"	"00075d67d0"	−0.1311
"00074daaf6"	"00075d67e2"	−0.1297
"00074daaf6"	"00075d73fc"	−0.0457
"00074daaf6"	"00075d7729"	−0.0644
"00074daaf6"	"00075d778c"	0.1042
"00074daaf6"	"00075d7b9e"	0.2715
"00074daaf6"	"00075d7c8f"	0.0741
"00074daaf6"	"00075d804d"	0.0844
"00074daaf6"	"00075d819f"	−0.0747

"00074daaf6"	"00075d8601"	0.0336
"00074daaf6"	"00075d8c6a"	-0.1659
"00074daaf6"	"00075dfedc"	0.1259
"00074daaf6"	"00075e05f2"	-0.2193
"00074daaf6"	"00075e0837"	-0.2106
"00074daaf6"	"00075e092e"	0.02
"00074daaf6"	"00075e0965"	-0.642
"00074daaf6"	"00075e0bc8"	-0.1328
"00074daaf6"	"00075e0fbb"	-0.1987
"00074dad20"	"00074db098"	0.0799
"00074dad20"	"00074db231"	-0.164
"00074dad20"	"00074db3a3"	-0.1693
"00074dad20"	"00074db5d6"	-0.1848
"00074dad20"	"00074db632"	0.0362
"00074dad20"	"00074db688"	-0.3091
"00074dad20"	"00074db8a6"	0.0748
"00074dad20"	"00074dba19"	-0.2691
"00074dad20"	"00074dbc2e"	0.3618
"00074dad20"	"00074dbe51"	0.0286
"00074dad20"	"00074dbe5f"	0.0363
"00074dad20"	"00074dbf6d"	-0.2639
"00074dad20"	"00074dc4a5"	-0.1215
"00074dad20"	"00074dc50c"	-0.0531
"00074dad20"	"00074dcdfa"	-0.0321
"00074dad20"	"00074dcf5f"	-0.2273
"00074dad20"	"00074dd007"	-0.1428
"00074dad20"	"00074dd163"	0.0244
"00074dad20"	"00074dd3df"	-0.0707
"00074dad20"	"00074dd577"	0.0552
"00074dad20"	"00074dd62e"	-0.1096
"00074dad20"	"00074dd73c"	0.2812
"00074dad20"	"00074dda10"	-0.2154
"00074dad20"	"00074ddab8"	0.0081
"00074dad20"	"00074ddd3d"	-0.1345
"00074dad20"	"00074ddf16"	0.1782
"00074dad20"	"00074ddfc1"	0.3572
"00074dad20"	"00074de21a"	-0.5365
"00074dad20"	"00074de2a9"	-0.3979
"00074dad20"	"00074de544"	-0.3006
"00074dad20"	"00074de98a"	-0.1736
"00074dad20"	"00074dea7e"	0.0755
"00074dad20"	"00074debd9"	0.0453
"00074dad20"	"00074deca3"	0.0544
"00074dad20"	"00074def43"	0.2057
"00074dad20"	"00074def99"	-0.2765
"00074dad20"	"00074ecdad"	0.1573
"00074dad20"	"00074ecf28"	-0.0416
"00074dad20"	"00074ed1e1"	-0.0616
"00074dad20"	"00074ed83b"	-0.1917
"00074dad20"	"00074ee5e3"	0.051

"00074dad20"	"00074ee6e0"	-0.0504
"00074dad20"	"00074eea3a"	-0.0426
"00074dad20"	"00074eff82"	-0.0997
"00074dad20"	"00074f0477"	0.3105
"00074dad20"	"00074f08c3"	-0.2645
"00074dad20"	"00074f1859"	0.0149
"00074dad20"	"00074f2268"	-0.4621
"00074dad20"	"00074f28be"	-0.1033
"00074dad20"	"00074f294b"	0.1028
"00074dad20"	"00074f2ddd"	-0.2918
"00074dad20"	"00074f2e75"	-0.2148
"00074dad20"	"00074f3088"	-0.1792
"00074dad20"	"00074f5a1c"	0.2143
"00074dad20"	"00074f75b7"	0.3748
"00074dad20"	"00074f8cd9"	0.3843
"00074dad20"	"00074f96dc"	-0.2674
"00074dad20"	"00074fabaa"	-0.2204
"00074dad20"	"00074facd9"	0.0027
"00074dad20"	"00074fae3c"	0.1487
"00074dad20"	"00074fb0a8"	-0.1395
"00074dad20"	"00074fb4e4"	-0.28
"00074dad20"	"00074fb7c2"	0.0382
"00074dad20"	"00074fbd36"	-0.4479
"00074dad20"	"00074fc27f"	-0.206
"00074dad20"	"00074fc31d"	-0.1922
"00074dad20"	"00074fd569"	0.108
"00074dad20"	"00074fef15"	-0.168
"00074dad20"	"00074ff562"	0.0809
"00074dad20"	"00075007ca"	0.0319
"00074dad20"	"0007500b86"	3e-04
"00074dad20"	"0007500d05"	0.326
"00074dad20"	"0007500ee4"	-0.042
"00074dad20"	"0007500eee"	0.0629
"00074dad20"	"00075013dc"	-0.3212
"00074dad20"	"000757b515"	-0.1821
"00074dad20"	"000757bc5a"	-0.1283
"00074dad20"	"000757c320"	-0.1757
"00074dad20"	"000757c9aa"	-0.0717
"00074dad20"	"000757ccbe"	-0.3132
"00074dad20"	"000757cfa9"	-0.4477
"00074dad20"	"000757d390"	-0.2035
"00074dad20"	"000757d393"	-0.0262
"00074dad20"	"000757d598"	-0.3135
"00074dad20"	"000757d5a2"	-0.3272
"00074dad20"	"000757d790"	0.069
"00074dad20"	"000757e30c"	-0.2485
"00074dad20"	"000757e4b0"	-0.0819
"00074dad20"	"000757e7a0"	0.0106
"00074dad20"	"000757e8b3"	-0.2889
"00074dad20"	"000757f627"	-0.3271

"00074dad20"	"000757f925"	0.004
"00074dad20"	"000757fa08"	0.2
"00074dad20"	"000757fe52"	−0.0512
"00074dad20"	"000758024a"	−0.1829
"00074dad20"	"00075804bb"	−0.296
"00074dad20"	"00075a0c04"	0.1051
"00074dad20"	"00075a3110"	−0.1347
"00074dad20"	"00075a341a"	−0.2885
"00074dad20"	"00075a3dcf"	0.1746
"00074dad20"	"00075a3e22"	−0.32
"00074dad20"	"00075a48d8"	−0.1258
"00074dad20"	"00075a5cfb"	−0.2799
"00074dad20"	"00075a6151"	−0.0955
"00074dad20"	"00075a6708"	0.0352
"00074dad20"	"00075a7319"	−0.2387
"00074dad20"	"00075a7723"	0.0017
"00074dad20"	"00075a778b"	−0.07
"00074dad20"	"00075a7b8e"	0.1504
"00074dad20"	"00075a7c79"	−0.1325
"00074dad20"	"00075a81b6"	−0.4492
"00074dad20"	"00075a82ac"	−0.2184
"00074dad20"	"00075a98e5"	−0.1935
"00074dad20"	"00075b0d29"	−0.3617
"00074dad20"	"00075b102a"	−0.1694
"00074dad20"	"00075b1074"	−0.2354
"00074dad20"	"00075b135d"	−0.4131
"00074dad20"	"00075b138b"	−0.1086
"00074dad20"	"00075b13a0"	−0.2905
"00074dad20"	"00075b13bd"	−0.3255
"00074dad20"	"00075b16a9"	−0.0022
"00074dad20"	"00075b1a28"	−0.1546
"00074dad20"	"00075b1a97"	−0.0459
"00074dad20"	"00075b1c7b"	−0.3783
"00074dad20"	"00075b1d24"	−0.0272
"00074dad20"	"00075b202b"	0.1107
"00074dad20"	"00075b22cb"	−0.0166
"00074dad20"	"00075b22da"	−0.277
"00074dad20"	"00075b2556"	−0.3549
"00074dad20"	"00075b25de"	0.2589
"00074dad20"	"00075b260c"	−0.1335
"00074dad20"	"00075b26f1"	0.0933
"00074dad20"	"00075b2920"	−0.1972
"00074dad20"	"00075b2a64"	−0.3491
"00074dad20"	"00075b2a9d"	−0.0202
"00074dad20"	"00075b2b37"	−0.2673
"00074dad20"	"00075b2cdd"	0.0291
"00074dad20"	"00075b3038"	0.4818
"00074dad20"	"00075b30fe"	−0.2995
"00074dad20"	"00075b3362"	−0.2954
"00074dad20"	"00075b350a"	−0.2222

"00074dad20"	"00075b350e"	−0.0783
"00074dad20"	"00075b3651"	−0.068
"00074dad20"	"00075b38ca"	0.1548
"00074dad20"	"00075b39cc"	0.179
"00074dad20"	"00075b3e1e"	0.1902
"00074dad20"	"00075b3e57"	0.0204
"00074dad20"	"00075b4079"	−0.3603
"00074dad20"	"00075b4150"	0.0214
"00074dad20"	"00075b4194"	−0.0315
"00074dad20"	"00075b42d5"	0.2582
"00074dad20"	"00075b4424"	−0.1196
"00074dad20"	"00075b4470"	−0.0589
"00074dad20"	"00075b47ed"	0.2786
"00074dad20"	"00075b4850"	−0.1365
"00074dad20"	"00075b4ca0"	−0.4388
"00074dad20"	"00075b4d7f"	−0.0373
"00074dad20"	"00075b520f"	0.0452
"00074dad20"	"00075b525f"	−0.3669
"00074dad20"	"00075b58f8"	−0.2983
"00074dad20"	"00075b5bcc"	0.0725
"00074dad20"	"00075b5bfa"	0.223
"00074dad20"	"00075b6339"	0.0229
"00074dad20"	"00075b6658"	−0.2123
"00074dad20"	"00075b679a"	0.1577
"00074dad20"	"00075b6cb7"	−0.3676
"00074dad20"	"00075b6df8"	0.1036
"00074dad20"	"00075b6ff6"	−0.0538
"00074dad20"	"00075b70ee"	−0.2288
"00074dad20"	"00075b7157"	−0.1362
"00074dad20"	"00075b7225"	−0.0147
"00074dad20"	"00075b7c89"	−0.0255
"00074dad20"	"00075b9048"	0.0903
"00074dad20"	"00075d0801"	−0.0342
"00074dad20"	"00075d1820"	−0.0032
"00074dad20"	"00075d1f3d"	−0.2619
"00074dad20"	"00075d2329"	0.4084
"00074dad20"	"00075d2b9b"	−0.2227
"00074dad20"	"00075d3941"	0.1973
"00074dad20"	"00075d3e96"	0.0777
"00074dad20"	"00075d4864"	−0.1526
"00074dad20"	"00075d5961"	−0.0443
"00074dad20"	"00075d5a63"	0.1576
"00074dad20"	"00075d6150"	0.0358
"00074dad20"	"00075d67d0"	−0.2368
"00074dad20"	"00075d67e2"	−0.3725
"00074dad20"	"00075d73fc"	−0.0253
"00074dad20"	"00075d7729"	−0.3474
"00074dad20"	"00075d778c"	−0.2276
"00074dad20"	"00075d7b9e"	−0.1563
"00074dad20"	"00075d7c8f"	−0.198

"00074dad20"	"00075d804d"	-0.03
"00074dad20"	"00075d819f"	-0.1917
"00074dad20"	"00075d8601"	0.0588
"00074dad20"	"00075d8c6a"	0.0453
"00074dad20"	"00075dfedc"	0.1875
"00074dad20"	"00075e05f2"	-0.0805
"00074dad20"	"00075e0837"	0.1026
"00074dad20"	"00075e092e"	-0.2293
"00074dad20"	"00075e0965"	-0.2314
"00074dad20"	"00075e0bc8"	0.0118
"00074dad20"	"00075e0fbb"	-0.3382
"00074db098"	"00074db231"	0.0402
"00074db098"	"00074db3a3"	0.2204
"00074db098"	"00074db5d6"	0.0379
"00074db098"	"00074db632"	0.1289
"00074db098"	"00074db688"	0.1476
"00074db098"	"00074db8a6"	0.0872
"00074db098"	"00074dba19"	-0.0949
"00074db098"	"00074dbc2e"	0.099
"00074db098"	"00074dbe51"	0.0296
"00074db098"	"00074dbe5f"	-0.1331
"00074db098"	"00074dbf6d"	0.3198
"00074db098"	"00074dc4a5"	-0.3458
"00074db098"	"00074dc50c"	0.0085
"00074db098"	"00074dcdfa"	0.034
"00074db098"	"00074dcf5f"	-0.021
"00074db098"	"00074dd007"	-0.0791
"00074db098"	"00074dd163"	-0.0208
"00074db098"	"00074dd3df"	-0.3296
"00074db098"	"00074dd577"	-0.0487
"00074db098"	"00074dd62e"	0.0733
"00074db098"	"00074dd73c"	0.0143
"00074db098"	"00074dda10"	-0.2288
"00074db098"	"00074ddab8"	0.0994
"00074db098"	"00074ddd3d"	-0.0145
"00074db098"	"00074ddf16"	0.0875
"00074db098"	"00074ddfc1"	0.2528
"00074db098"	"00074de21a"	-0.1512
"00074db098"	"00074de2a9"	-0.0596
"00074db098"	"00074de544"	-0.2111
"00074db098"	"00074de98a"	-0.2733
"00074db098"	"00074dea7e"	-0.0969
"00074db098"	"00074debd9"	0.0135
"00074db098"	"00074deca3"	-0.0051
"00074db098"	"00074def43"	0.0505
"00074db098"	"00074def99"	-0.1431
"00074db098"	"00074ecdad"	0.0169
"00074db098"	"00074ecf28"	-0.4722
"00074db098"	"00074ed1e1"	-0.255
"00074db098"	"00074ed83b"	0.1281

"00074db098"	"00074ee5e3"	0.2363
"00074db098"	"00074ee6e0"	0.0852
"00074db098"	"00074eea3a"	−0.1423
"00074db098"	"00074eff82"	−0.2635
"00074db098"	"00074f0477"	−0.0587
"00074db098"	"00074f08c3"	0.0987
"00074db098"	"00074f1859"	0.0187
"00074db098"	"00074f2268"	−0.0441
"00074db098"	"00074f28be"	0.2909
"00074db098"	"00074f294b"	0.1123
"00074db098"	"00074f2ddd"	−0.0573
"00074db098"	"00074f2e75"	−0.3768
"00074db098"	"00074f3088"	−0.1654
"00074db098"	"00074f5a1c"	−0.0176
"00074db098"	"00074f75b7"	0.1174
"00074db098"	"00074f8cd9"	0.2018
"00074db098"	"00074f96dc"	−0.1908
"00074db098"	"00074fabaa"	0.0294
"00074db098"	"00074facd9"	0.1931
"00074db098"	"00074fae3c"	−0.0372
"00074db098"	"00074fb0a8"	0.0469
"00074db098"	"00074fb4e4"	−0.0074
"00074db098"	"00074fb7c2"	−0.111
"00074db098"	"00074fbd36"	0.2228
"00074db098"	"00074fc27f"	0.2271
"00074db098"	"00074fc31d"	0.0431
"00074db098"	"00074fd569"	−0.2868
"00074db098"	"00074fef15"	−0.2008
"00074db098"	"00074ff562"	−0.2495
"00074db098"	"00075007ca"	0.2066
"00074db098"	"0007500b86"	0.2856
"00074db098"	"0007500d05"	−0.1477
"00074db098"	"0007500ee4"	−0.0799
"00074db098"	"0007500eee"	−0.0309
"00074db098"	"00075013dc"	−0.0943
"00074db098"	"000757b515"	0.0996
"00074db098"	"000757bc5a"	0.1597
"00074db098"	"000757c320"	0.115
"00074db098"	"000757c9aa"	0.2755
"00074db098"	"000757ccbe"	−0.1539
"00074db098"	"000757cfa9"	0.1796
"00074db098"	"000757d390"	0.0617
"00074db098"	"000757d393"	−0.1083
"00074db098"	"000757d598"	0.1156
"00074db098"	"000757d5a2"	0.0239
"00074db098"	"000757d790"	−0.2434
"00074db098"	"000757e30c"	0.1655
"00074db098"	"000757e4b0"	0.0183
"00074db098"	"000757e7a0"	0.0306
"00074db098"	"000757e8b3"	0.0667

"00074db098"	"000757f627"	0.1893
"00074db098"	"000757f925"	-0.0468
"00074db098"	"000757fa08"	-0.0962
"00074db098"	"000757fe52"	-0.0921
"00074db098"	"000758024a"	0.1739
"00074db098"	"00075804bb"	0.2574
"00074db098"	"00075a0c04"	-0.2356
"00074db098"	"00075a3110"	0.0453
"00074db098"	"00075a341a"	-0.2062
"00074db098"	"00075a3dcf"	-0.1933
"00074db098"	"00075a3e22"	-0.0579
"00074db098"	"00075a48d8"	-0.2607
"00074db098"	"00075a5cfb"	0.1372
"00074db098"	"00075a6151"	0.0964
"00074db098"	"00075a6708"	0.3726
"00074db098"	"00075a7319"	0.0798
"00074db098"	"00075a7723"	-0.1342
"00074db098"	"00075a778b"	-0.3112
"00074db098"	"00075a7b8e"	0.193
"00074db098"	"00075a7c79"	0.2272
"00074db098"	"00075a81b6"	-0.2912
"00074db098"	"00075a82ac"	-0.2835
"00074db098"	"00075a98e5"	-0.2493
"00074db098"	"00075b0d29"	0.1279
"00074db098"	"00075b102a"	0.1532
"00074db098"	"00075b1074"	-0.2128
"00074db098"	"00075b135d"	-0.0559
"00074db098"	"00075b138b"	0.0575
"00074db098"	"00075b13a0"	-0.3993
"00074db098"	"00075b13bd"	-0.0778
"00074db098"	"00075b16a9"	-0.0051
"00074db098"	"00075b1a28"	0.0672
"00074db098"	"00075b1a97"	0.001
"00074db098"	"00075b1c7b"	-0.3824
"00074db098"	"00075b1d24"	0.1728
"00074db098"	"00075b202b"	0.1311
"00074db098"	"00075b22cb"	-0.0976
"00074db098"	"00075b22da"	-0.1472
"00074db098"	"00075b2556"	-0.1421
"00074db098"	"00075b25de"	0.102
"00074db098"	"00075b260c"	0.1348
"00074db098"	"00075b26f1"	-0.2522
"00074db098"	"00075b2920"	0.2623
"00074db098"	"00075b2a64"	0.242
"00074db098"	"00075b2a9d"	0.132
"00074db098"	"00075b2b37"	-0.1605
"00074db098"	"00075b2cdd"	-0.153
"00074db098"	"00075b3038"	0.0331
"00074db098"	"00075b30fe"	-0.2592
"00074db098"	"00075b3362"	-0.3434

"00074db098"	"00075b350a"	0.1997
"00074db098"	"00075b350e"	-0.1345
"00074db098"	"00075b3651"	-0.101
"00074db098"	"00075b38ca"	-0.156
"00074db098"	"00075b39cc"	0.1049
"00074db098"	"00075b3e1e"	0.1219
"00074db098"	"00075b3e57"	-0.1659
"00074db098"	"00075b4079"	0.1059
"00074db098"	"00075b4150"	0.1269
"00074db098"	"00075b4194"	0.2136
"00074db098"	"00075b42d5"	0.1024
"00074db098"	"00075b4424"	-0.1254
"00074db098"	"00075b4470"	0.1938
"00074db098"	"00075b47ed"	-0.1689
"00074db098"	"00075b4850"	0.2838
"00074db098"	"00075b4ca0"	-0.1668
"00074db098"	"00075b4d7f"	-0.0333
"00074db098"	"00075b520f"	1e-04
"00074db098"	"00075b525f"	-0.1663
"00074db098"	"00075b58f8"	0.0389
"00074db098"	"00075b5bcc"	0.112
"00074db098"	"00075b5bfa"	-0.3074
"00074db098"	"00075b6339"	0.0926
"00074db098"	"00075b6658"	-0.1627
"00074db098"	"00075b679a"	0.1464
"00074db098"	"00075b6cb7"	0.2246
"00074db098"	"00075b6df8"	-0.2473
"00074db098"	"00075b6ff6"	0.1376
"00074db098"	"00075b70ee"	0.0484
"00074db098"	"00075b7157"	-0.2029
"00074db098"	"00075b7225"	-0.1738
"00074db098"	"00075b7c89"	-0.127
"00074db098"	"00075b9048"	0.1037
"00074db098"	"00075d0801"	0.0305
"00074db098"	"00075d1820"	-0.1317
"00074db098"	"00075d1f3d"	0.0207
"00074db098"	"00075d2329"	-0.1141
"00074db098"	"00075d2b9b"	0.0579
"00074db098"	"00075d3941"	-0.2842
"00074db098"	"00075d3e96"	-0.1196
"00074db098"	"00075d4864"	-0.121
"00074db098"	"00075d5961"	-0.181
"00074db098"	"00075d5a63"	0.1193
"00074db098"	"00075d6150"	-0.0216
"00074db098"	"00075d67d0"	-0.1334
"00074db098"	"00075d67e2"	-0.2164
"00074db098"	"00075d73fc"	-0.1399
"00074db098"	"00075d7729"	-0.3081
"00074db098"	"00075d778c"	-0.3949
"00074db098"	"00075d7b9e"	-0.1428

"00074db098"	"00075d7c8f"	0.0492
"00074db098"	"00075d804d"	0.091
"00074db098"	"00075d819f"	0.1155
"00074db098"	"00075d8601"	−0.0394
"00074db098"	"00075d8c6a"	−0.0833
"00074db098"	"00075dfedc"	0.2931
"00074db098"	"00075e05f2"	0.0255
"00074db098"	"00075e0837"	0.0755
"00074db098"	"00075e092e"	−0.2039
"00074db098"	"00075e0965"	−0.0201
"00074db098"	"00075e0bc8"	0.187
"00074db098"	"00075e0fbb"	−0.4008
"00074db231"	"00074db3a3"	0.1979
"00074db231"	"00074db5d6"	−0.0514
"00074db231"	"00074db632"	−0.0217
"00074db231"	"00074db688"	0.0496
"00074db231"	"00074db8a6"	0.0171
"00074db231"	"00074dba19"	0.4093
"00074db231"	"00074dbc2e"	−0.0884
"00074db231"	"00074dbe51"	0.0981
"00074db231"	"00074dbe5f"	0.2021
"00074db231"	"00074dbf6d"	−0.0369
"00074db231"	"00074dc4a5"	−0.0524
"00074db231"	"00074dc50c"	0.0975
"00074db231"	"00074dcdfa"	0.1312
"00074db231"	"00074dcf5f"	−0.1977
"00074db231"	"00074dd007"	−0.0272
"00074db231"	"00074dd163"	0.1465
"00074db231"	"00074dd3df"	0.0406
"00074db231"	"00074dd577"	−0.0074
"00074db231"	"00074dd62e"	0.0379
"00074db231"	"00074dd73c"	−0.1166
"00074db231"	"00074dda10"	0.0625
"00074db231"	"00074ddab8"	0.1372
"00074db231"	"00074ddd3d"	−0.2267
"00074db231"	"00074ddf16"	0.0482
"00074db231"	"00074ddfc1"	0.1924
"00074db231"	"00074de21a"	−0.0626
"00074db231"	"00074de2a9"	0.1779
"00074db231"	"00074de544"	0.2483
"00074db231"	"00074de98a"	0.0155
"00074db231"	"00074dea7e"	−0.1004
"00074db231"	"00074debd9"	0.1745
"00074db231"	"00074deca3"	0.0518
"00074db231"	"00074def43"	−0.034
"00074db231"	"00074def99"	−0.112
"00074db231"	"00074ecdad"	−0.1852
"00074db231"	"00074ecf28"	−0.2276
"00074db231"	"00074ed1e1"	−0.038
"00074db231"	"00074ed83b"	0.1567

"00074db231"	"00074ee5e3"	0.008
"00074db231"	"00074ee6e0"	0.0956
"00074db231"	"00074eea3a"	0.3846
"00074db231"	"00074eff82"	-0.0201
"00074db231"	"00074f0477"	0.025
"00074db231"	"00074f08c3"	-0.0757
"00074db231"	"00074f1859"	0.2187
"00074db231"	"00074f2268"	0.0355
"00074db231"	"00074f28be"	0.3829
"00074db231"	"00074f294b"	0.0412
"00074db231"	"00074f2ddd"	0.0293
"00074db231"	"00074f2e75"	0.051
"00074db231"	"00074f3088"	0.0195
"00074db231"	"00074f5a1c"	0.2061
"00074db231"	"00074f75b7"	0.0611
"00074db231"	"00074f8cd9"	0.0023
"00074db231"	"00074f96dc"	-0.1444
"00074db231"	"00074fabaa"	0.1751
"00074db231"	"00074facd9"	0.2034
"00074db231"	"00074fae3c"	0.1135
"00074db231"	"00074fb0a8"	0.2792
"00074db231"	"00074fb4e4"	0.0087
"00074db231"	"00074fb7c2"	0.2831
"00074db231"	"00074fbd36"	0.1986
"00074db231"	"00074fc27f"	0.1215
"00074db231"	"00074fc31d"	0.0201
"00074db231"	"00074fd569"	-0.1364
"00074db231"	"00074fef15"	-0.1087
"00074db231"	"00074ff562"	-0.3545
"00074db231"	"00075007ca"	0.0449
"00074db231"	"0007500b86"	-0.0407
"00074db231"	"0007500d05"	-0.0505
"00074db231"	"0007500ee4"	-0.1446
"00074db231"	"0007500eee"	0.3057
"00074db231"	"00075013dc"	-0.0758
"00074db231"	"000757b515"	0.3693
"00074db231"	"000757bc5a"	0.1489
"00074db231"	"000757c320"	-0.1665
"00074db231"	"000757c9aa"	0.1499
"00074db231"	"000757ccbe"	-8e-04
"00074db231"	"000757cfa9"	-0.0184
"00074db231"	"000757d390"	-0.0859
"00074db231"	"000757d393"	0.1604
"00074db231"	"000757d598"	-0.1106
"00074db231"	"000757d5a2"	0.0397
"00074db231"	"000757d790"	-0.0164
"00074db231"	"000757e30c"	-0.0768
"00074db231"	"000757e4b0"	0.294
"00074db231"	"000757e7a0"	0.0509
"00074db231"	"000757e8b3"	0.2401

"00074db231"	"000757f627"	0.2568
"00074db231"	"000757f925"	0.1756
"00074db231"	"000757fa08"	0.1582
"00074db231"	"000757fe52"	-4e-04
"00074db231"	"000758024a"	-0.1418
"00074db231"	"00075804bb"	0.0519
"00074db231"	"00075a0c04"	0.187
"00074db231"	"00075a3110"	0.1446
"00074db231"	"00075a341a"	-0.0806
"00074db231"	"00075a3dcf"	0.1911
"00074db231"	"00075a3e22"	0.2188
"00074db231"	"00075a48d8"	0.2117
"00074db231"	"00075a5cfb"	-0.103
"00074db231"	"00075a6151"	0.2285
"00074db231"	"00075a6708"	-0.1645
"00074db231"	"00075a7319"	-0.0043
"00074db231"	"00075a7723"	0.0252
"00074db231"	"00075a778b"	-0.2268
"00074db231"	"00075a7b8e"	0.3371
"00074db231"	"00075a7c79"	0.0143
"00074db231"	"00075a81b6"	0.0835
"00074db231"	"00075a82ac"	0.2333
"00074db231"	"00075a98e5"	-0.2856
"00074db231"	"00075b0d29"	0.1066
"00074db231"	"00075b102a"	0.1731
"00074db231"	"00075b1074"	0.0999
"00074db231"	"00075b135d"	0.2279
"00074db231"	"00075b138b"	0.1852
"00074db231"	"00075b13a0"	-0.1117
"00074db231"	"00075b13bd"	0.1187
"00074db231"	"00075b16a9"	0.0942
"00074db231"	"00075b1a28"	-0.0938
"00074db231"	"00075b1a97"	-0.1181
"00074db231"	"00075b1c7b"	-0.2628
"00074db231"	"00075b1d24"	0.1008
"00074db231"	"00075b202b"	0.1094
"00074db231"	"00075b22cb"	-0.0591
"00074db231"	"00075b22da"	0.0143
"00074db231"	"00075b2556"	0.1071
"00074db231"	"00075b25de"	0.1983
"00074db231"	"00075b260c"	0.1243
"00074db231"	"00075b26f1"	-0.0119
"00074db231"	"00075b2920"	0.0329
"00074db231"	"00075b2a64"	-0.0113
"00074db231"	"00075b2a9d"	0.2352
"00074db231"	"00075b2b37"	-0.3094
"00074db231"	"00075b2cdd"	-0.1209
"00074db231"	"00075b3038"	0.0405
"00074db231"	"00075b30fe"	0.1111
"00074db231"	"00075b3362"	-0.201

"00074db231"	"00075b350a"	0.0132
"00074db231"	"00075b350e"	0.0562
"00074db231"	"00075b3651"	-0.0708
"00074db231"	"00075b38ca"	0.073
"00074db231"	"00075b39cc"	0.3106
"00074db231"	"00075b3e1e"	0.1462
"00074db231"	"00075b3e57"	-0.0711
"00074db231"	"00075b4079"	-0.0149
"00074db231"	"00075b4150"	-0.0998
"00074db231"	"00075b4194"	0.2463
"00074db231"	"00075b42d5"	0.0672
"00074db231"	"00075b4424"	-0.2356
"00074db231"	"00075b4470"	0.2183
"00074db231"	"00075b47ed"	0.237
"00074db231"	"00075b4850"	0.1201
"00074db231"	"00075b4ca0"	0.1982
"00074db231"	"00075b4d7f"	0.3876
"00074db231"	"00075b520f"	0.0692
"00074db231"	"00075b525f"	-0.2281
"00074db231"	"00075b58f8"	0.0948
"00074db231"	"00075b5bcc"	-0.045
"00074db231"	"00075b5bfa"	-0.1806
"00074db231"	"00075b6339"	0.1199
"00074db231"	"00075b6658"	-0.117
"00074db231"	"00075b679a"	0.0118
"00074db231"	"00075b6cb7"	0.0626
"00074db231"	"00075b6df8"	0.0389
"00074db231"	"00075b6ff6"	-0.0966
"00074db231"	"00075b70ee"	-0.003
"00074db231"	"00075b7157"	0.079
"00074db231"	"00075b7225"	0.0067
"00074db231"	"00075b7c89"	0.128
"00074db231"	"00075b9048"	-0.0024
"00074db231"	"00075d0801"	0.1599
"00074db231"	"00075d1820"	-0.0341
"00074db231"	"00075d1f3d"	0.0212
"00074db231"	"00075d2329"	0.1409
"00074db231"	"00075d2b9b"	0.0859
"00074db231"	"00075d3941"	-0.1297
"00074db231"	"00075d3e96"	0.0168
"00074db231"	"00075d4864"	0.1271
"00074db231"	"00075d5961"	0.2631
"00074db231"	"00075d5a63"	0.14
"00074db231"	"00075d6150"	-0.1053
"00074db231"	"00075d67d0"	-0.0221
"00074db231"	"00075d67e2"	-0.0984
"00074db231"	"00075d73fc"	-0.1189
"00074db231"	"00075d7729"	-0.0764
"00074db231"	"00075d778c"	-0.2667
"00074db231"	"00075d7b9e"	0.0759

"00074db231"	"00075d7c8f"	0.2394
"00074db231"	"00075d804d"	0.044
"00074db231"	"00075d819f"	0.1296
"00074db231"	"00075d8601"	0.1501
"00074db231"	"00075d8c6a"	0.0918
"00074db231"	"00075dfedc"	0.2138
"00074db231"	"00075e05f2"	0.0765
"00074db231"	"00075e0837"	0.1688
"00074db231"	"00075e092e"	−0.0176
"00074db231"	"00075e0965"	−0.2044
"00074db231"	"00075e0bc8"	0.319
"00074db231"	"00075e0fbb"	0.0866
"00074db3a3"	"00074db5d6"	0.1018
"00074db3a3"	"00074db632"	0.3121
"00074db3a3"	"00074db688"	−0.1651
"00074db3a3"	"00074db8a6"	0.1489
"00074db3a3"	"00074dba19"	0.0827
"00074db3a3"	"00074dbc2e"	0.2142
"00074db3a3"	"00074dbe51"	0.2152
"00074db3a3"	"00074dbe5f"	−0.094
"00074db3a3"	"00074dbf6d"	0.4289
"00074db3a3"	"00074dc4a5"	−0.2123
"00074db3a3"	"00074dc50c"	0.1801
"00074db3a3"	"00074dcdfa"	−0.0881
"00074db3a3"	"00074dcf5f"	−0.013
"00074db3a3"	"00074dd007"	−0.0066
"00074db3a3"	"00074dd163"	0.1995
"00074db3a3"	"00074dd3df"	−0.0411
"00074db3a3"	"00074dd577"	−0.0733
"00074db3a3"	"00074dd62e"	0.1515
"00074db3a3"	"00074dd73c"	−0.0802
"00074db3a3"	"00074dda10"	−0.0019
"00074db3a3"	"00074ddab8"	0.1302
"00074db3a3"	"00074ddd3d"	0.1014
"00074db3a3"	"00074ddf16"	0.073
"00074db3a3"	"00074ddfc1"	0.1808
"00074db3a3"	"00074de21a"	−0.0402
"00074db3a3"	"00074de2a9"	0.3656
"00074db3a3"	"00074de544"	0.1566
"00074db3a3"	"00074de98a"	0.1775
"00074db3a3"	"00074dea7e"	−0.1843
"00074db3a3"	"00074debd9"	0.1029
"00074db3a3"	"00074deca3"	0.3999
"00074db3a3"	"00074def43"	0.2325
"00074db3a3"	"00074def99"	−0.012
"00074db3a3"	"00074ecdad"	−0.1733
"00074db3a3"	"00074ecf28"	−0.1231
"00074db3a3"	"00074ed1e1"	−0.0643
"00074db3a3"	"00074ed83b"	0.3931
"00074db3a3"	"00074ee5e3"	0.1539

"00074db3a3"	"00074ee6e0"	0.0885
"00074db3a3"	"00074eea3a"	0.0882
"00074db3a3"	"00074eff82"	-0.0855
"00074db3a3"	"00074f0477"	-0.1841
"00074db3a3"	"00074f08c3"	0.0781
"00074db3a3"	"00074f1859"	0.1427
"00074db3a3"	"00074f2268"	0.0661
"00074db3a3"	"00074f28be"	0.2135
"00074db3a3"	"00074f294b"	-0.0553
"00074db3a3"	"00074f2ddd"	0.1844
"00074db3a3"	"00074f2e75"	-0.3656
"00074db3a3"	"00074f3088"	-0.2502
"00074db3a3"	"00074f5a1c"	-0.0769
"00074db3a3"	"00074f75b7"	0.1179
"00074db3a3"	"00074f8cd9"	0.0316
"00074db3a3"	"00074f96dc"	-0.1936
"00074db3a3"	"00074fabaa"	0.4671
"00074db3a3"	"00074facd9"	-0.1076
"00074db3a3"	"00074fae3c"	-0.0467
"00074db3a3"	"00074fb0a8"	0.1327
"00074db3a3"	"00074fb4e4"	0.0564
"00074db3a3"	"00074fb7c2"	0.2452
"00074db3a3"	"00074fbd36"	0.0985
"00074db3a3"	"00074fc27f"	0.3812
"00074db3a3"	"00074fc31d"	0.0871
"00074db3a3"	"00074fd569"	0.0798
"00074db3a3"	"00074fef15"	-0.0798
"00074db3a3"	"00074ff562"	-0.2642
"00074db3a3"	"00075007ca"	0.1752
"00074db3a3"	"0007500b86"	0.1905
"00074db3a3"	"0007500d05"	-0.0173
"00074db3a3"	"0007500ee4"	-0.104
"00074db3a3"	"0007500eee"	0.0145
"00074db3a3"	"00075013dc"	-0.158
"00074db3a3"	"000757b515"	0.2786
"00074db3a3"	"000757bc5a"	0.1581
"00074db3a3"	"000757c320"	0.469
"00074db3a3"	"000757c9aa"	0.3022
"00074db3a3"	"000757ccbe"	-0.0342
"00074db3a3"	"000757cfa9"	0.1535
"00074db3a3"	"000757d390"	-0.0889
"00074db3a3"	"000757d393"	0.2711
"00074db3a3"	"000757d598"	0.1669
"00074db3a3"	"000757d5a2"	-0.0127
"00074db3a3"	"000757d790"	0.1658
"00074db3a3"	"000757e30c"	0.1469
"00074db3a3"	"000757e4b0"	0.2637
"00074db3a3"	"000757e7a0"	-0.1401
"00074db3a3"	"000757e8b3"	0.3516
"00074db3a3"	"000757f627"	0.1468

"00074db3a3"	"000757f925"	0.3879
"00074db3a3"	"000757fa08"	-0.1253
"00074db3a3"	"000757fe52"	0.0233
"00074db3a3"	"000758024a"	0.1591
"00074db3a3"	"00075804bb"	0.2077
"00074db3a3"	"00075a0c04"	0.1446
"00074db3a3"	"00075a3110"	0.2009
"00074db3a3"	"00075a341a"	0.0173
"00074db3a3"	"00075a3dcf"	-0.1566
"00074db3a3"	"00075a3e22"	0.2403
"00074db3a3"	"00075a48d8"	0.0443
"00074db3a3"	"00075a5cfb"	0.1814
"00074db3a3"	"00075a6151"	0.1244
"00074db3a3"	"00075a6708"	-0.1218
"00074db3a3"	"00075a7319"	-0.0935
"00074db3a3"	"00075a7723"	0.129
"00074db3a3"	"00075a778b"	-0.0184
"00074db3a3"	"00075a7b8e"	-0.1283
"00074db3a3"	"00075a7c79"	0.214
"00074db3a3"	"00075a81b6"	-0.1402
"00074db3a3"	"00075a82ac"	0.0575
"00074db3a3"	"00075a98e5"	-0.0813
"00074db3a3"	"00075b0d29"	0.1188
"00074db3a3"	"00075b102a"	0.3254
"00074db3a3"	"00075b1074"	-0.0683
"00074db3a3"	"00075b135d"	0.0945
"00074db3a3"	"00075b138b"	0.469
"00074db3a3"	"00075b13a0"	-0.372
"00074db3a3"	"00075b13bd"	-0.162
"00074db3a3"	"00075b16a9"	0.1539
"00074db3a3"	"00075b1a28"	0.1525
"00074db3a3"	"00075b1a97"	0.0737
"00074db3a3"	"00075b1c7b"	-0.2383
"00074db3a3"	"00075b1d24"	0.1101
"00074db3a3"	"00075b202b"	0.2756
"00074db3a3"	"00075b22cb"	0.1462
"00074db3a3"	"00075b22da"	0.1076
"00074db3a3"	"00075b2556"	0.3103
"00074db3a3"	"00075b25de"	0.131
"00074db3a3"	"00075b260c"	0.3387
"00074db3a3"	"00075b26f1"	-0.0044
"00074db3a3"	"00075b2920"	0.1874
"00074db3a3"	"00075b2a64"	0.3192
"00074db3a3"	"00075b2a9d"	0.152
"00074db3a3"	"00075b2b37"	-0.1702
"00074db3a3"	"00075b2cdd"	0.0538
"00074db3a3"	"00075b3038"	0.2149
"00074db3a3"	"00075b30fe"	0.1719
"00074db3a3"	"00075b3362"	-0.0117
"00074db3a3"	"00075b350a"	0.0477

"00074db3a3"	"00075b350e"	0.2281
"00074db3a3"	"00075b3651"	-0.0032
"00074db3a3"	"00075b38ca"	-0.1953
"00074db3a3"	"00075b39cc"	0.0223
"00074db3a3"	"00075b3e1e"	0.1891
"00074db3a3"	"00075b3e57"	-0.0589
"00074db3a3"	"00075b4079"	0.0651
"00074db3a3"	"00075b4150"	0.1875
"00074db3a3"	"00075b4194"	0.2073
"00074db3a3"	"00075b42d5"	-0.1059
"00074db3a3"	"00075b4424"	-0.0065
"00074db3a3"	"00075b4470"	0.1581
"00074db3a3"	"00075b47ed"	-0.0675
"00074db3a3"	"00075b4850"	0.2175
"00074db3a3"	"00075b4ca0"	0.0944
"00074db3a3"	"00075b4d7f"	0.1479
"00074db3a3"	"00075b520f"	0.005
"00074db3a3"	"00075b525f"	0.1451
"00074db3a3"	"00075b58f8"	0.0135
"00074db3a3"	"00075b5bcc"	0.2121
"00074db3a3"	"00075b5bfa"	-0.1059
"00074db3a3"	"00075b6339"	0.2743
"00074db3a3"	"00075b6658"	-0.0351
"00074db3a3"	"00075b679a"	0.1667
"00074db3a3"	"00075b6cb7"	-0.0387
"00074db3a3"	"00075b6df8"	-0.1096
"00074db3a3"	"00075b6ff6"	0.1323
"00074db3a3"	"00075b70ee"	0.0643
"00074db3a3"	"00075b7157"	0.0092
"00074db3a3"	"00075b7225"	0.0395
"00074db3a3"	"00075b7c89"	0.0385
"00074db3a3"	"00075b9048"	0.2332
"00074db3a3"	"00075d0801"	0.1335
"00074db3a3"	"00075d1820"	-0.0277
"00074db3a3"	"00075d1f3d"	0.2094
"00074db3a3"	"00075d2329"	-0.178
"00074db3a3"	"00075d2b9b"	0.3911
"00074db3a3"	"00075d3941"	-0.0684
"00074db3a3"	"00075d3e96"	-0.0598
"00074db3a3"	"00075d4864"	-0.0528
"00074db3a3"	"00075d5961"	0.1285
"00074db3a3"	"00075d5a63"	0.1684
"00074db3a3"	"00075d6150"	-0.0762
"00074db3a3"	"00075d67d0"	-0.1017
"00074db3a3"	"00075d67e2"	0.146
"00074db3a3"	"00075d73fc"	-0.0719
"00074db3a3"	"00075d7729"	-0.1771
"00074db3a3"	"00075d778c"	-0.1128
"00074db3a3"	"00075d7b9e"	0.1899
"00074db3a3"	"00075d7c8f"	0.2601

"00074db3a3"	"00075d804d"	0.0905
"00074db3a3"	"00075d819f"	0.2442
"00074db3a3"	"00075d8601"	0.066
"00074db3a3"	"00075d8c6a"	0.0016
"00074db3a3"	"00075dfedc"	0.3524
"00074db3a3"	"00075e05f2"	0.2092
"00074db3a3"	"00075e0837"	0.0659
"00074db3a3"	"00075e092e"	−0.0369
"00074db3a3"	"00075e0965"	−0.0234
"00074db3a3"	"00075e0bc8"	0.1555
"00074db3a3"	"00075e0fbb"	0.0265
"00074db5d6"	"00074db632"	0.1082
"00074db5d6"	"00074db688"	−0.0825
"00074db5d6"	"00074db8a6"	−0.0498
"00074db5d6"	"00074dba19"	−0.0681
"00074db5d6"	"00074dbc2e"	−0.0788
"00074db5d6"	"00074dbe51"	−0.0498
"00074db5d6"	"00074dbe5f"	−0.0932
"00074db5d6"	"00074dbf6d"	0.1163
"00074db5d6"	"00074dc4a5"	−0.0447
"00074db5d6"	"00074dc50c"	−0.1881
"00074db5d6"	"00074dcdfa"	0.1285
"00074db5d6"	"00074dcf5f"	−0.1129
"00074db5d6"	"00074dd007"	−0.275
"00074db5d6"	"00074dd163"	0.0114
"00074db5d6"	"00074dd3df"	−0.2569
"00074db5d6"	"00074dd577"	−0.1664
"00074db5d6"	"00074dd62e"	0.3662
"00074db5d6"	"00074dd73c"	−0.2569
"00074db5d6"	"00074dda10"	0.293
"00074db5d6"	"00074ddab8"	0.0593
"00074db5d6"	"00074ddd3d"	−0.1734
"00074db5d6"	"00074ddf16"	−0.301
"00074db5d6"	"00074ddfc1"	−0.192
"00074db5d6"	"00074de21a"	0.0212
"00074db5d6"	"00074de2a9"	0.0831
"00074db5d6"	"00074de544"	0.3203
"00074db5d6"	"00074de98a"	−0.1036
"00074db5d6"	"00074dea7e"	−0.6337
"00074db5d6"	"00074debd9"	−0.1717
"00074db5d6"	"00074deca3"	−0.0916
"00074db5d6"	"00074def43"	−0.2725
"00074db5d6"	"00074def99"	−0.2479
"00074db5d6"	"00074ecdad"	−0.3325
"00074db5d6"	"00074ecf28"	0.149
"00074db5d6"	"00074ed1e1"	−0.3652
"00074db5d6"	"00074ed83b"	−0.1376
"00074db5d6"	"00074ee5e3"	0.0316
"00074db5d6"	"00074ee6e0"	0.3223
"00074db5d6"	"00074eea3a"	−0.3205

"00074db5d6"	"00074eff82"	0.0494
"00074db5d6"	"00074f0477"	-0.4378
"00074db5d6"	"00074f08c3"	0.0132
"00074db5d6"	"00074f1859"	-0.0213
"00074db5d6"	"00074f2268"	-0.0885
"00074db5d6"	"00074f28be"	0.047
"00074db5d6"	"00074f294b"	-0.4716
"00074db5d6"	"00074f2ddd"	-0.0666
"00074db5d6"	"00074f2e75"	-0.3042
"00074db5d6"	"00074f3088"	0.2585
"00074db5d6"	"00074f5a1c"	-0.2162
"00074db5d6"	"00074f75b7"	0.1017
"00074db5d6"	"00074f8cd9"	0.0124
"00074db5d6"	"00074f96dc"	0.4139
"00074db5d6"	"00074fabaa"	0.0799
"00074db5d6"	"00074facd9"	-0.1253
"00074db5d6"	"00074fae3c"	0.2013
"00074db5d6"	"00074fb0a8"	0.1419
"00074db5d6"	"00074fb4e4"	-0.0541
"00074db5d6"	"00074fb7c2"	0.0929
"00074db5d6"	"00074fbd36"	-0.1673
"00074db5d6"	"00074fc27f"	-0.0729
"00074db5d6"	"00074fc31d"	-0.0753
"00074db5d6"	"00074fd569"	0.1827
"00074db5d6"	"00074fef15"	-0.078
"00074db5d6"	"00074ff562"	-0.3677
"00074db5d6"	"00075007ca"	-0.0774
"00074db5d6"	"0007500b86"	0.1295
"00074db5d6"	"0007500d05"	0.0636
"00074db5d6"	"0007500ee4"	0.0642
"00074db5d6"	"0007500eee"	-0.1781
"00074db5d6"	"00075013dc"	-0.0475
"00074db5d6"	"000757b515"	-0.1519
"00074db5d6"	"000757bc5a"	0.1749
"00074db5d6"	"000757c320"	0.2007
"00074db5d6"	"000757c9aa"	-0.0543
"00074db5d6"	"000757ccbe"	0.0693
"00074db5d6"	"000757cfa9"	0.0409
"00074db5d6"	"000757d390"	-0.0036
"00074db5d6"	"000757d393"	0.0892
"00074db5d6"	"000757d598"	-0.0672
"00074db5d6"	"000757d5a2"	-0.1962
"00074db5d6"	"000757d790"	-0.1173
"00074db5d6"	"000757e30c"	-0.1307
"00074db5d6"	"000757e4b0"	-0.1781
"00074db5d6"	"000757e7a0"	-0.1623
"00074db5d6"	"000757e8b3"	0.1707
"00074db5d6"	"000757f627"	0.0759
"00074db5d6"	"000757f925"	0.0964
"00074db5d6"	"000757fa08"	-0.3479

"00074db5d6"	"000757fe52"	0.0494
"00074db5d6"	"000758024a"	-0.0937
"00074db5d6"	"00075804bb"	-0.0615
"00074db5d6"	"00075a0c04"	0.1383
"00074db5d6"	"00075a3110"	0.1202
"00074db5d6"	"00075a341a"	-0.0083
"00074db5d6"	"00075a3dcf"	-0.3285
"00074db5d6"	"00075a3e22"	0.2795
"00074db5d6"	"00075a48d8"	0.0286
"00074db5d6"	"00075a5cfb"	0.0221
"00074db5d6"	"00075a6151"	-0.3093
"00074db5d6"	"00075a6708"	-0.2613
"00074db5d6"	"00075a7319"	0.035
"00074db5d6"	"00075a7723"	-0.286
"00074db5d6"	"00075a778b"	0.0052
"00074db5d6"	"00075a7b8e"	-0.0096
"00074db5d6"	"00075a7c79"	0.1867
"00074db5d6"	"00075a81b6"	0.0022
"00074db5d6"	"00075a82ac"	0.0291
"00074db5d6"	"00075a98e5"	0.2132
"00074db5d6"	"00075b0d29"	0.1593
"00074db5d6"	"00075b102a"	0.1127
"00074db5d6"	"00075b1074"	-0.2582
"00074db5d6"	"00075b135d"	0.0838
"00074db5d6"	"00075b138b"	0.1086
"00074db5d6"	"00075b13a0"	-0.3696
"00074db5d6"	"00075b13bd"	0.344
"00074db5d6"	"00075b16a9"	-0.0025
"00074db5d6"	"00075b1a28"	0.0457
"00074db5d6"	"00075b1a97"	0.3507
"00074db5d6"	"00075b1c7b"	-0.0606
"00074db5d6"	"00075b1d24"	-0.0487
"00074db5d6"	"00075b202b"	0.1795
"00074db5d6"	"00075b22cb"	0.0624
"00074db5d6"	"00075b22da"	-0.3342
"00074db5d6"	"00075b2556"	-0.287
"00074db5d6"	"00075b25de"	0.1019
"00074db5d6"	"00075b260c"	-0.0786
"00074db5d6"	"00075b26f1"	5e-04
"00074db5d6"	"00075b2920"	-0.011
"00074db5d6"	"00075b2a64"	0.065
"00074db5d6"	"00075b2a9d"	-0.1748
"00074db5d6"	"00075b2b37"	0.0452
"00074db5d6"	"00075b2cdd"	0.2978
"00074db5d6"	"00075b3038"	0.0072
"00074db5d6"	"00075b30fe"	-0.2778
"00074db5d6"	"00075b3362"	-0.0875
"00074db5d6"	"00075b350a"	0.1116
"00074db5d6"	"00075b350e"	-0.0038
"00074db5d6"	"00075b3651"	0.058

"00074db5d6"	"00075b38ca"	−0.1065
"00074db5d6"	"00075b39cc"	−0.0139
"00074db5d6"	"00075b3e1e"	0.0395
"00074db5d6"	"00075b3e57"	0.5209
"00074db5d6"	"00075b4079"	0.0922
"00074db5d6"	"00075b4150"	−0.1184
"00074db5d6"	"00075b4194"	0.0871
"00074db5d6"	"00075b42d5"	−0.1204
"00074db5d6"	"00075b4424"	−0.0168
"00074db5d6"	"00075b4470"	0.138
"00074db5d6"	"00075b47ed"	−0.1467
"00074db5d6"	"00075b4850"	−0.0215
"00074db5d6"	"00075b4ca0"	−0.1342
"00074db5d6"	"00075b4d7f"	0.2369
"00074db5d6"	"00075b520f"	−0.0471
"00074db5d6"	"00075b525f"	−0.0474
"00074db5d6"	"00075b58f8"	−0.0713
"00074db5d6"	"00075b5bcc"	0.147
"00074db5d6"	"00075b5bfa"	−0.1744
"00074db5d6"	"00075b6339"	0.1255
"00074db5d6"	"00075b6658"	0.0967
"00074db5d6"	"00075b679a"	0.0593
"00074db5d6"	"00075b6cb7"	−0.198
"00074db5d6"	"00075b6df8"	−0.1612
"00074db5d6"	"00075b6ff6"	−0.0312
"00074db5d6"	"00075b70ee"	−0.2145
"00074db5d6"	"00075b7157"	0.1286
"00074db5d6"	"00075b7225"	−0.1054
"00074db5d6"	"00075b7c89"	0.0807
"00074db5d6"	"00075b9048"	−0.0289
"00074db5d6"	"00075d0801"	−0.1464
"00074db5d6"	"00075d1820"	0.0047
"00074db5d6"	"00075d1f3d"	−0.0925
"00074db5d6"	"00075d2329"	−0.2731
"00074db5d6"	"00075d2b9b"	−0.0628
"00074db5d6"	"00075d3941"	0.1109
"00074db5d6"	"00075d3e96"	0.0606
"00074db5d6"	"00075d4864"	−0.3181
"00074db5d6"	"00075d5961"	0.1401
"00074db5d6"	"00075d5a63"	0.0917
"00074db5d6"	"00075d6150"	0.1714
"00074db5d6"	"00075d67d0"	−0.227
"00074db5d6"	"00075d67e2"	0.2182
"00074db5d6"	"00075d73fc"	−0.1249
"00074db5d6"	"00075d7729"	−0.0347
"00074db5d6"	"00075d778c"	−0.3527
"00074db5d6"	"00075d7b9e"	0.2632
"00074db5d6"	"00075d7c8f"	−0.0064
"00074db5d6"	"00075d804d"	0.1522
"00074db5d6"	"00075d819f"	0.0128

"00074db5d6"	"00075d8601"	0.4487
"00074db5d6"	"00075d8c6a"	−0.1701
"00074db5d6"	"00075dfedc"	0.1922
"00074db5d6"	"00075e05f2"	0.0262
"00074db5d6"	"00075e0837"	−0.1224
"00074db5d6"	"00075e092e"	−0.2007
"00074db5d6"	"00075e0965"	−0.4697
"00074db5d6"	"00075e0bc8"	−0.0796
"00074db5d6"	"00075e0fbb"	−0.4386
"00074db632"	"00074db688"	0.2278
"00074db632"	"00074db8a6"	−0.1244
"00074db632"	"00074dba19"	0.356
"00074db632"	"00074dbc2e"	0.0867
"00074db632"	"00074dbe51"	0.19
"00074db632"	"00074dbe5f"	−0.3027
"00074db632"	"00074dbf6d"	0.2505
"00074db632"	"00074dc4a5"	−0.1965
"00074db632"	"00074dc50c"	0.2416
"00074db632"	"00074dcdfa"	0.0276
"00074db632"	"00074dcf5f"	0.2194
"00074db632"	"00074dd007"	0.092
"00074db632"	"00074dd163"	−0.2191
"00074db632"	"00074dd3df"	−0.1797
"00074db632"	"00074dd577"	0.1342
"00074db632"	"00074dd62e"	0.1962
"00074db632"	"00074dd73c"	0.2441
"00074db632"	"00074dda10"	0.0648
"00074db632"	"00074ddab8"	0.229
"00074db632"	"00074ddd3d"	0.0949
"00074db632"	"00074ddf16"	−0.0127
"00074db632"	"00074ddfc1"	0.2016
"00074db632"	"00074de21a"	0.0598
"00074db632"	"00074de2a9"	0.4011
"00074db632"	"00074de544"	0.0274
"00074db632"	"00074de98a"	0.1755
"00074db632"	"00074dea7e"	−0.0884
"00074db632"	"00074debd9"	−0.1488
"00074db632"	"00074deca3"	−0.1067
"00074db632"	"00074def43"	−0.0187
"00074db632"	"00074def99"	−0.0268
"00074db632"	"00074ecdad"	−0.0602
"00074db632"	"00074ecf28"	−0.0831
"00074db632"	"00074ed1e1"	0.0836
"00074db632"	"00074ed83b"	0.3817
"00074db632"	"00074ee5e3"	−0.1752
"00074db632"	"00074ee6e0"	−0.0443
"00074db632"	"00074eea3a"	−0.0451
"00074db632"	"00074eff82"	0.0025
"00074db632"	"00074f0477"	−0.2182
"00074db632"	"00074f08c3"	0.3172

"00074db632"	"00074f1859"	0.0809
"00074db632"	"00074f2268"	-0.0573
"00074db632"	"00074f28be"	0.3938
"00074db632"	"00074f294b"	-0.0506
"00074db632"	"00074f2ddd"	-0.0487
"00074db632"	"00074f2e75"	0.0218
"00074db632"	"00074f3088"	0.001
"00074db632"	"00074f5a1c"	0.1535
"00074db632"	"00074f75b7"	0.1596
"00074db632"	"00074f8cd9"	0.2295
"00074db632"	"00074f96dc"	0.0462
"00074db632"	"00074fabaa"	0.2793
"00074db632"	"00074facd9"	-0.1542
"00074db632"	"00074fae3c"	-0.0102
"00074db632"	"00074fb0a8"	0.2045
"00074db632"	"00074fb4e4"	-0.0215
"00074db632"	"00074fb7c2"	0.2183
"00074db632"	"00074fbd36"	-0.0742
"00074db632"	"00074fc27f"	0.3135
"00074db632"	"00074fc31d"	-0.0343
"00074db632"	"00074fd569"	0.2483
"00074db632"	"00074fef15"	-0.1865
"00074db632"	"00074ff562"	-0.0325
"00074db632"	"00075007ca"	0.0465
"00074db632"	"0007500b86"	0.2799
"00074db632"	"0007500d05"	0.1641
"00074db632"	"0007500ee4"	-0.1206
"00074db632"	"0007500eee"	-0.1959
"00074db632"	"00075013dc"	0.1093
"00074db632"	"000757b515"	0.1127
"00074db632"	"000757bc5a"	0.2669
"00074db632"	"000757c320"	0.0932
"00074db632"	"000757c9aa"	0.2497
"00074db632"	"000757ccbe"	0.1751
"00074db632"	"000757cfa9"	-0.2761
"00074db632"	"000757d390"	-0.016
"00074db632"	"000757d393"	0.0628
"00074db632"	"000757d598"	0.1764
"00074db632"	"000757d5a2"	-0.0319
"00074db632"	"000757d790"	-0.0081
"00074db632"	"000757e30c"	0.3417
"00074db632"	"000757e4b0"	0.2762
"00074db632"	"000757e7a0"	0.0625
"00074db632"	"000757e8b3"	0.2487
"00074db632"	"000757f627"	0.0857
"00074db632"	"000757f925"	0.2068
"00074db632"	"000757fa08"	0.0988
"00074db632"	"000757fe52"	0.1627
"00074db632"	"000758024a"	0.4395
"00074db632"	"00075804bb"	0.3262

"00074db632"	"00075a0c04"	0.0961
"00074db632"	"00075a3110"	0.2601
"00074db632"	"00075a341a"	0.0466
"00074db632"	"00075a3dcf"	-0.1393
"00074db632"	"00075a3e22"	0.283
"00074db632"	"00075a48d8"	0.1502
"00074db632"	"00075a5cfb"	0.5373
"00074db632"	"00075a6151"	0.1388
"00074db632"	"00075a6708"	-0.0113
"00074db632"	"00075a7319"	-0.0573
"00074db632"	"00075a7723"	0.3497
"00074db632"	"00075a778b"	0.191
"00074db632"	"00075a7b8e"	0.1515
"00074db632"	"00075a7c79"	0.3129
"00074db632"	"00075a81b6"	0.0842
"00074db632"	"00075a82ac"	0.0706
"00074db632"	"00075a98e5"	0.0621
"00074db632"	"00075b0d29"	0.0588
"00074db632"	"00075b102a"	0.1529
"00074db632"	"00075b1074"	-0.0907
"00074db632"	"00075b135d"	0.1792
"00074db632"	"00075b138b"	0.2682
"00074db632"	"00075b13a0"	-0.2134
"00074db632"	"00075b13bd"	0.0188
"00074db632"	"00075b16a9"	0.0282
"00074db632"	"00075b1a28"	0.1205
"00074db632"	"00075b1a97"	0.0881
"00074db632"	"00075b1c7b"	-0.1478
"00074db632"	"00075b1d24"	0.0232
"00074db632"	"00075b202b"	0.2946
"00074db632"	"00075b22cb"	-0.0984
"00074db632"	"00075b22da"	0.0213
"00074db632"	"00075b2556"	0.0017
"00074db632"	"00075b25de"	0.1163
"00074db632"	"00075b260c"	0.2155
"00074db632"	"00075b26f1"	0.1435
"00074db632"	"00075b2920"	0.4956
"00074db632"	"00075b2a64"	0.3826
"00074db632"	"00075b2a9d"	0.0364
"00074db632"	"00075b2b37"	-0.1649
"00074db632"	"00075b2cdd"	0.0045
"00074db632"	"00075b3038"	0.3117
"00074db632"	"00075b30fe"	-0.0669
"00074db632"	"00075b3362"	-0.1677
"00074db632"	"00075b350a"	-0.05
"00074db632"	"00075b350e"	0.1056
"00074db632"	"00075b3651"	-0.0592
"00074db632"	"00075b38ca"	-0.0353
"00074db632"	"00075b39cc"	-0.1274
"00074db632"	"00075b3e1e"	0.144

"00074db632"	"00075b3e57"	0.1306
"00074db632"	"00075b4079"	-0.0516
"00074db632"	"00075b4150"	0.5564
"00074db632"	"00075b4194"	0.3362
"00074db632"	"00075b42d5"	0.167
"00074db632"	"00075b4424"	0.2211
"00074db632"	"00075b4470"	0.2513
"00074db632"	"00075b47ed"	-0.0743
"00074db632"	"00075b4850"	0.2084
"00074db632"	"00075b4ca0"	0.1183
"00074db632"	"00075b4d7f"	0.3321
"00074db632"	"00075b520f"	0.0498
"00074db632"	"00075b525f"	0.0202
"00074db632"	"00075b58f8"	0.1362
"00074db632"	"00075b5bcc"	0.3071
"00074db632"	"00075b5bfa"	0.0403
"00074db632"	"00075b6339"	0.2811
"00074db632"	"00075b6658"	-0.4007
"00074db632"	"00075b679a"	0.1181
"00074db632"	"00075b6cb7"	0.0343
"00074db632"	"00075b6df8"	0.1324
"00074db632"	"00075b6ff6"	0.3273
"00074db632"	"00075b70ee"	0.1789
"00074db632"	"00075b7157"	0.1181
"00074db632"	"00075b7225"	0.2009
"00074db632"	"00075b7c89"	0.0525
"00074db632"	"00075b9048"	0.4296
"00074db632"	"00075d0801"	0.0057
"00074db632"	"00075d1820"	-0.0481
"00074db632"	"00075d1f3d"	0.2402
"00074db632"	"00075d2329"	0.0488
"00074db632"	"00075d2b9b"	0.2922
"00074db632"	"00075d3941"	0.1921
"00074db632"	"00075d3e96"	0.3545
"00074db632"	"00075d4864"	0.0757
"00074db632"	"00075d5961"	-0.0281
"00074db632"	"00075d5a63"	0.3724
"00074db632"	"00075d6150"	-0.0584
"00074db632"	"00075d67d0"	-0.2598
"00074db632"	"00075d67e2"	0.2025
"00074db632"	"00075d73fc"	0.06
"00074db632"	"00075d7729"	0.0454
"00074db632"	"00075d778c"	-0.2575
"00074db632"	"00075d7b9e"	-0.1448
"00074db632"	"00075d7c8f"	0.0999
"00074db632"	"00075d804d"	0.277
"00074db632"	"00075d819f"	0.0219
"00074db632"	"00075d8601"	0.108
"00074db632"	"00075d8c6a"	0.375
"00074db632"	"00075dfedc"	0.1853

"00074db632"	"00075e05f2"	0.2176
"00074db632"	"00075e0837"	0.0512
"00074db632"	"00075e092e"	−0.1445
"00074db632"	"00075e0965"	0.4677
"00074db632"	"00075e0bc8"	0.1777
"00074db632"	"00075e0fbb"	−0.1599
"00074db688"	"00074db8a6"	−0.1697
"00074db688"	"00074dba19"	0.1471
"00074db688"	"00074dbc2e"	−0.2322
"00074db688"	"00074dbe51"	0.1457
"00074db688"	"00074dbe5f"	−0.7672
"00074db688"	"00074dbf6d"	−0.1706
"00074db688"	"00074dc4a5"	−0.2599
"00074db688"	"00074dc50c"	−0.1119
"00074db688"	"00074dcdfa"	−0.1174
"00074db688"	"00074dcf5f"	0.0532
"00074db688"	"00074dd007"	−0.218
"00074db688"	"00074dd163"	−0.2741
"00074db688"	"00074dd3df"	−0.2615
"00074db688"	"00074dd577"	0.1584
"00074db688"	"00074dd62e"	0.0326
"00074db688"	"00074dd73c"	−0.0593
"00074db688"	"00074dda10"	−0.1778
"00074db688"	"00074ddab8"	−0.1876
"00074db688"	"00074ddd3d"	0.1502
"00074db688"	"00074ddf16"	−0.3021
"00074db688"	"00074ddfc1"	−0.2565
"00074db688"	"00074de21a"	−0.0675
"00074db688"	"00074de2a9"	0.0091
"00074db688"	"00074de544"	0.1074
"00074db688"	"00074de98a"	−0.1608
"00074db688"	"00074dea7e"	−0.221
"00074db688"	"00074debd9"	−0.62
"00074db688"	"00074deca3"	−0.2261
"00074db688"	"00074def43"	−0.3932
"00074db688"	"00074def99"	0.2975
"00074db688"	"00074ecdad"	−0.1862
"00074db688"	"00074ecf28"	−0.2149
"00074db688"	"00074ed1e1"	−0.0281
"00074db688"	"00074ed83b"	0.0611
"00074db688"	"00074ee5e3"	−0.4153
"00074db688"	"00074ee6e0"	−0.038
"00074db688"	"00074eea3a"	−0.0372
"00074db688"	"00074eff82"	−0.1099
"00074db688"	"00074f0477"	−0.49
"00074db688"	"00074f08c3"	−0.048
"00074db688"	"00074f1859"	−0.2912
"00074db688"	"00074f2268"	−0.1302
"00074db688"	"00074f28be"	0.2843
"00074db688"	"00074f294b"	0.1382

"00074db688"	"00074f2ddd"	0.1244
"00074db688"	"00074f2e75"	-0.0452
"00074db688"	"00074f3088"	0.0608
"00074db688"	"00074f5a1c"	0.0462
"00074db688"	"00074f75b7"	-0.1342
"00074db688"	"00074f8cd9"	-0.144
"00074db688"	"00074f96dc"	-0.1899
"00074db688"	"00074fabaa"	-0.2809
"00074db688"	"00074facd9"	-0.3862
"00074db688"	"00074fae3c"	-0.1203
"00074db688"	"00074fb0a8"	0.1417
"00074db688"	"00074fb4e4"	-0.1978
"00074db688"	"00074fb7c2"	0.1779
"00074db688"	"00074fbd36"	0.1738
"00074db688"	"00074fc27f"	-0.0013
"00074db688"	"00074fc31d"	0.3121
"00074db688"	"00074fd569"	-0.1953
"00074db688"	"00074fef15"	-0.0883
"00074db688"	"00074ff562"	-0.2607
"00074db688"	"00075007ca"	-0.2285
"00074db688"	"0007500b86"	-0.0366
"00074db688"	"0007500d05"	-0.4371
"00074db688"	"0007500ee4"	-0.0526
"00074db688"	"0007500eee"	-0.6345
"00074db688"	"00075013dc"	-0.0074
"00074db688"	"000757b515"	0.0732
"00074db688"	"000757bc5a"	-0.0258
"00074db688"	"000757c320"	-0.3573
"00074db688"	"000757c9aa"	-0.0514
"00074db688"	"000757ccbe"	0.1068
"00074db688"	"000757cfa9"	0.1295
"00074db688"	"000757d390"	-0.0702
"00074db688"	"000757d393"	-0.2132
"00074db688"	"000757d598"	-0.0493
"00074db688"	"000757d5a2"	0.097
"00074db688"	"000757d790"	-0.1847
"00074db688"	"000757e30c"	0.0498
"00074db688"	"000757e4b0"	-0.0435
"00074db688"	"000757e7a0"	0.034
"00074db688"	"000757e8b3"	0.1566
"00074db688"	"000757f627"	0.0334
"00074db688"	"000757f925"	-0.2352
"00074db688"	"000757fa08"	-0.0367
"00074db688"	"000757fe52"	0.0075
"00074db688"	"000758024a"	0.0295
"00074db688"	"00075804bb"	-0.0238
"00074db688"	"00075a0c04"	-0.0357
"00074db688"	"00075a3110"	0.0715
"00074db688"	"00075a341a"	-0.0319
"00074db688"	"00075a3dcf"	-0.0543

"00074db688"	"00075a3e22"	−0.0113
"00074db688"	"00075a48d8"	−0.0241
"00074db688"	"00075a5cfb"	0.0464
"00074db688"	"00075a6151"	−0.0082
"00074db688"	"00075a6708"	0.0558
"00074db688"	"00075a7319"	0.0357
"00074db688"	"00075a7723"	0.0401
"00074db688"	"00075a778b"	−0.1218
"00074db688"	"00075a7b8e"	0.1705
"00074db688"	"00075a7c79"	0.225
"00074db688"	"00075a81b6"	0.1091
"00074db688"	"00075a82ac"	−0.0527
"00074db688"	"00075a98e5"	−0.1877
"00074db688"	"00075b0d29"	0.219
"00074db688"	"00075b102a"	−0.3514
"00074db688"	"00075b1074"	−0.1455
"00074db688"	"00075b135d"	0.5258
"00074db688"	"00075b138b"	−0.1586
"00074db688"	"00075b13a0"	−0.2881
"00074db688"	"00075b13bd"	0.0801
"00074db688"	"00075b16a9"	−0.1202
"00074db688"	"00075b1a28"	0.0756
"00074db688"	"00075b1a97"	0.0494
"00074db688"	"00075b1c7b"	−0.2604
"00074db688"	"00075b1d24"	0.0741
"00074db688"	"00075b202b"	−0.173
"00074db688"	"00075b22cb"	−0.2215
"00074db688"	"00075b22da"	−0.1657
"00074db688"	"00075b2556"	−0.197
"00074db688"	"00075b25de"	−0.1778
"00074db688"	"00075b260c"	−0.1381
"00074db688"	"00075b26f1"	−0.1118
"00074db688"	"00075b2920"	−0.0126
"00074db688"	"00075b2a64"	−0.1437
"00074db688"	"00075b2a9d"	−0.1391
"00074db688"	"00075b2b37"	−0.3746
"00074db688"	"00075b2cdd"	−0.0179
"00074db688"	"00075b3038"	−0.1263
"00074db688"	"00075b30fe"	−0.2146
"00074db688"	"00075b3362"	−0.3229
"00074db688"	"00075b350a"	−0.1418
"00074db688"	"00075b350e"	−0.1655
"00074db688"	"00075b3651"	−0.0696
"00074db688"	"00075b38ca"	−0.2451
"00074db688"	"00075b39cc"	3e−04
"00074db688"	"00075b3e1e"	−0.3338
"00074db688"	"00075b3e57"	−0.2684
"00074db688"	"00075b4079"	−0.1483
"00074db688"	"00075b4150"	0.0265
"00074db688"	"00075b4194"	0.0435

"00074db688"	"00075b42d5"	0.0336
"00074db688"	"00075b4424"	−0.0638
"00074db688"	"00075b4470"	−0.0439
"00074db688"	"00075b47ed"	−0.1433
"00074db688"	"00075b4850"	−0.0136
"00074db688"	"00075b4ca0"	0.3489
"00074db688"	"00075b4d7f"	0.1779
"00074db688"	"00075b520f"	0.1684
"00074db688"	"00075b525f"	0.1497
"00074db688"	"00075b58f8"	−0.0989
"00074db688"	"00075b5bcc"	−0.0481
"00074db688"	"00075b5bfa"	−0.4096
"00074db688"	"00075b6339"	0.1555
"00074db688"	"00075b6658"	−0.1606
"00074db688"	"00075b679a"	0.2005
"00074db688"	"00075b6cb7"	−0.1748
"00074db688"	"00075b6df8"	0.0088
"00074db688"	"00075b6ff6"	0.1336
"00074db688"	"00075b70ee"	0.0199
"00074db688"	"00075b7157"	0.0243
"00074db688"	"00075b7225"	0.0509
"00074db688"	"00075b7c89"	0.0621
"00074db688"	"00075b9048"	0.1808
"00074db688"	"00075d0801"	−0.0605
"00074db688"	"00075d1820"	−0.1786
"00074db688"	"00075d1f3d"	−0.1299
"00074db688"	"00075d2329"	−0.1058
"00074db688"	"00075d2b9b"	−0.3049
"00074db688"	"00075d3941"	−0.2693
"00074db688"	"00075d3e96"	0.0264
"00074db688"	"00075d4864"	−0.0141
"00074db688"	"00075d5961"	−0.094
"00074db688"	"00075d5a63"	−0.212
"00074db688"	"00075d6150"	0.1077
"00074db688"	"00075d67d0"	−0.273
"00074db688"	"00075d67e2"	−0.0928
"00074db688"	"00075d73fc"	−0.0148
"00074db688"	"00075d7729"	0.1695
"00074db688"	"00075d778c"	−0.248
"00074db688"	"00075d7b9e"	−0.0771
"00074db688"	"00075d7c8f"	0.1768
"00074db688"	"00075d804d"	−0.2012
"00074db688"	"00075d819f"	−0.1598
"00074db688"	"00075d8601"	−0.1627
"00074db688"	"00075d8c6a"	0.1132
"00074db688"	"00075dfedc"	0.1287
"00074db688"	"00075e05f2"	−0.0603
"00074db688"	"00075e0837"	−0.2198
"00074db688"	"00075e092e"	−0.1193
"00074db688"	"00075e0965"	−0.0759

"00074db688"	"00075e0bc8"	0.0447
"00074db688"	"00075e0fbb"	−0.2024
"00074db8a6"	"00074dba19"	−0.1378
"00074db8a6"	"00074dbc2e"	0.2989
"00074db8a6"	"00074dbe51"	−0.0669
"00074db8a6"	"00074dbe5f"	−0.035
"00074db8a6"	"00074dbf6d"	−0.1905
"00074db8a6"	"00074dc4a5"	−0.3088
"00074db8a6"	"00074dc50c"	−0.0419
"00074db8a6"	"00074dcdfa"	−0.0245
"00074db8a6"	"00074dcf5f"	−0.4291
"00074db8a6"	"00074dd007"	−0.1079
"00074db8a6"	"00074dd163"	0.7401
"00074db8a6"	"00074dd3df"	−0.2095
"00074db8a6"	"00074dd577"	−0.1097
"00074db8a6"	"00074dd62e"	−0.1937
"00074db8a6"	"00074dd73c"	0.2633
"00074db8a6"	"00074dda10"	−0.1724
"00074db8a6"	"00074ddab8"	−0.1606
"00074db8a6"	"00074ddd3d"	−0.2134
"00074db8a6"	"00074ddf16"	0.1422
"00074db8a6"	"00074ddfc1"	0.3619
"00074db8a6"	"00074de21a"	−0.1922
"00074db8a6"	"00074de2a9"	−0.2292
"00074db8a6"	"00074de544"	−0.0095
"00074db8a6"	"00074de98a"	−0.1067
"00074db8a6"	"00074dea7e"	−0.1159
"00074db8a6"	"00074debd9"	0.265
"00074db8a6"	"00074deca3"	0.667
"00074db8a6"	"00074def43"	0.2653
"00074db8a6"	"00074def99"	−0.1053
"00074db8a6"	"00074ecdad"	0.1242
"00074db8a6"	"00074ecf28"	−0.2787
"00074db8a6"	"00074ed1e1"	−0.1313
"00074db8a6"	"00074ed83b"	0.2133
"00074db8a6"	"00074ee5e3"	0.0659
"00074db8a6"	"00074ee6e0"	−0.2487
"00074db8a6"	"00074eea3a"	−0.0761
"00074db8a6"	"00074eff82"	−0.015
"00074db8a6"	"00074f0477"	−0.4032
"00074db8a6"	"00074f08c3"	−0.2856
"00074db8a6"	"00074f1859"	−0.0904
"00074db8a6"	"00074f2268"	−0.2502
"00074db8a6"	"00074f28be"	−0.0349
"00074db8a6"	"00074f294b"	−0.2582
"00074db8a6"	"00074f2ddd"	−0.0667
"00074db8a6"	"00074f2e75"	−0.3541
"00074db8a6"	"00074f3088"	−0.3112
"00074db8a6"	"00074f5a1c"	0.3761
"00074db8a6"	"00074f75b7"	0.2136

"00074db8a6"	"00074f8cd9"	0.0718
"00074db8a6"	"00074f96dc"	-0.2681
"00074db8a6"	"00074fabaa"	-8e-04
"00074db8a6"	"00074facd9"	-0.1987
"00074db8a6"	"00074fae3c"	-0.0481
"00074db8a6"	"00074fb0a8"	-0.0852
"00074db8a6"	"00074fb4e4"	-0.0579
"00074db8a6"	"00074fb7c2"	-0.0863
"00074db8a6"	"00074fbd36"	0.007
"00074db8a6"	"00074fc27f"	-0.0324
"00074db8a6"	"00074fc31d"	-0.0242
"00074db8a6"	"00074fd569"	-0.0924
"00074db8a6"	"00074fef15"	0.0475
"00074db8a6"	"00074ff562"	-0.0932
"00074db8a6"	"00075007ca"	-0.0197
"00074db8a6"	"0007500b86"	0.2597
"00074db8a6"	"0007500d05"	0.0059
"00074db8a6"	"0007500ee4"	-0.0782
"00074db8a6"	"0007500eee"	0.0089
"00074db8a6"	"00075013dc"	-0.3179
"00074db8a6"	"000757b515"	-0.1359
"00074db8a6"	"000757bc5a"	-0.0524
"00074db8a6"	"000757c320"	0.0474
"00074db8a6"	"000757c9aa"	-0.0698
"00074db8a6"	"000757ccbe"	-0.1954
"00074db8a6"	"000757cfa9"	-0.0373
"00074db8a6"	"000757d390"	-0.1404
"00074db8a6"	"000757d393"	0.27
"00074db8a6"	"000757d598"	-0.3522
"00074db8a6"	"000757d5a2"	-0.3275
"00074db8a6"	"000757d790"	0.1491
"00074db8a6"	"000757e30c"	-0.4285
"00074db8a6"	"000757e4b0"	0.064
"00074db8a6"	"000757e7a0"	-0.1188
"00074db8a6"	"000757e8b3"	-0.032
"00074db8a6"	"000757f627"	-0.0249
"00074db8a6"	"000757f925"	0.0147
"00074db8a6"	"000757fa08"	-0.2381
"00074db8a6"	"000757fe52"	-0.183
"00074db8a6"	"000758024a"	-0.3496
"00074db8a6"	"00075804bb"	-0.2209
"00074db8a6"	"00075a0c04"	-0.1666
"00074db8a6"	"00075a3110"	-0.1711
"00074db8a6"	"00075a341a"	-0.1178
"00074db8a6"	"00075a3dcf"	0.362
"00074db8a6"	"00075a3e22"	-0.149
"00074db8a6"	"00075a48d8"	-0.1584
"00074db8a6"	"00075a5cfb"	-0.3734
"00074db8a6"	"00075a6151"	0.0872
"00074db8a6"	"00075a6708"	-0.3277

"00074db8a6"	"00075a7319"	−0.0069
"00074db8a6"	"00075a7723"	−0.1937
"00074db8a6"	"00075a778b"	−0.3733
"00074db8a6"	"00075a7b8e"	−0.2767
"00074db8a6"	"00075a7c79"	−0.0186
"00074db8a6"	"00075a81b6"	−0.2057
"00074db8a6"	"00075a82ac"	−0.166
"00074db8a6"	"00075a98e5"	−0.131
"00074db8a6"	"00075b0d29"	−0.0661
"00074db8a6"	"00075b102a"	−0.0715
"00074db8a6"	"00075b1074"	−0.345
"00074db8a6"	"00075b135d"	−0.2127
"00074db8a6"	"00075b138b"	−0.0017
"00074db8a6"	"00075b13a0"	−0.2566
"00074db8a6"	"00075b13bd"	−0.2145
"00074db8a6"	"00075b16a9"	−0.1754
"00074db8a6"	"00075b1a28"	−0.008
"00074db8a6"	"00075b1a97"	0.0298
"00074db8a6"	"00075b1c7b"	−0.3809
"00074db8a6"	"00075b1d24"	−0.3094
"00074db8a6"	"00075b202b"	−0.1303
"00074db8a6"	"00075b22cb"	−0.1409
"00074db8a6"	"00075b22da"	−0.1063
"00074db8a6"	"00075b2556"	−0.184
"00074db8a6"	"00075b25de"	0.1815
"00074db8a6"	"00075b260c"	−0.0051
"00074db8a6"	"00075b26f1"	−0.0221
"00074db8a6"	"00075b2920"	−0.312
"00074db8a6"	"00075b2a64"	−0.3247
"00074db8a6"	"00075b2a9d"	−0.2141
"00074db8a6"	"00075b2b37"	−0.2651
"00074db8a6"	"00075b2cdd"	−0.1033
"00074db8a6"	"00075b3038"	0.1892
"00074db8a6"	"00075b30fe"	−0.1083
"00074db8a6"	"00075b3362"	−0.2763
"00074db8a6"	"00075b350a"	−0.2792
"00074db8a6"	"00075b350e"	0.1566
"00074db8a6"	"00075b3651"	−0.1438
"00074db8a6"	"00075b38ca"	0.2639
"00074db8a6"	"00075b39cc"	0.113
"00074db8a6"	"00075b3e1e"	0.1172
"00074db8a6"	"00075b3e57"	−0.1903
"00074db8a6"	"00075b4079"	−0.2421
"00074db8a6"	"00075b4150"	−0.3133
"00074db8a6"	"00075b4194"	0.085
"00074db8a6"	"00075b42d5"	0.2293
"00074db8a6"	"00075b4424"	−0.2454
"00074db8a6"	"00075b4470"	0.0232
"00074db8a6"	"00075b47ed"	0.1546
"00074db8a6"	"00075b4850"	−0.0098

"00074db8a6"	"00075b4ca0"	−0.2875
"00074db8a6"	"00075b4d7f"	−0.0015
"00074db8a6"	"00075b520f"	0.1479
"00074db8a6"	"00075b525f"	−0.081
"00074db8a6"	"00075b58f8"	−0.204
"00074db8a6"	"00075b5bcc"	−0.153
"00074db8a6"	"00075b5bfa"	0.0279
"00074db8a6"	"00075b6339"	0.0093
"00074db8a6"	"00075b6658"	−0.0626
"00074db8a6"	"00075b679a"	0.044
"00074db8a6"	"00075b6cb7"	−0.3259
"00074db8a6"	"00075b6df8"	−0.0225
"00074db8a6"	"00075b6ff6"	−0.2485
"00074db8a6"	"00075b70ee"	−0.2795
"00074db8a6"	"00075b7157"	−0.0819
"00074db8a6"	"00075b7225"	−0.0845
"00074db8a6"	"00075b7c89"	−0.2537
"00074db8a6"	"00075b9048"	−0.0986
"00074db8a6"	"00075d0801"	0.0658
"00074db8a6"	"00075d1820"	−0.2515
"00074db8a6"	"00075d1f3d"	−0.2501
"00074db8a6"	"00075d2329"	0.0663
"00074db8a6"	"00075d2b9b"	−0.0665
"00074db8a6"	"00075d3941"	−0.2027
"00074db8a6"	"00075d3e96"	−0.201
"00074db8a6"	"00075d4864"	0.1501
"00074db8a6"	"00075d5961"	−0.0016
"00074db8a6"	"00075d5a63"	−0.1227
"00074db8a6"	"00075d6150"	0.1779
"00074db8a6"	"00075d67d0"	−0.2742
"00074db8a6"	"00075d67e2"	−0.0879
"00074db8a6"	"00075d73fc"	−0.0787
"00074db8a6"	"00075d7729"	−0.2769
"00074db8a6"	"00075d778c"	−0.0479
"00074db8a6"	"00075d7b9e"	0.0339
"00074db8a6"	"00075d7c8f"	−0.231
"00074db8a6"	"00075d804d"	−0.1249
"00074db8a6"	"00075d819f"	−0.1582
"00074db8a6"	"00075d8601"	0.1664
"00074db8a6"	"00075d8c6a"	0.0114
"00074db8a6"	"00075dfedc"	−0.0675
"00074db8a6"	"00075e05f2"	0.2714
"00074db8a6"	"00075e0837"	−0.0845
"00074db8a6"	"00075e092e"	−0.1307
"00074db8a6"	"00075e0965"	−0.5654
"00074db8a6"	"00075e0bc8"	0.0427
"00074db8a6"	"00075e0fbb"	−0.1615
"00074dba19"	"00074dbc2e"	−0.2986
"00074dba19"	"00074dbe51"	0.0249
"00074dba19"	"00074dbe5f"	−0.1452

"00074dba19"	"00074dbf6d"	0.1945
"00074dba19"	"00074dc4a5"	-0.2737
"00074dba19"	"00074dc50c"	0.0826
"00074dba19"	"00074dcdfa"	-0.131
"00074dba19"	"00074dcf5f"	-0.2431
"00074dba19"	"00074dd007"	0.0706
"00074dba19"	"00074dd163"	-0.1532
"00074dba19"	"00074dd3df"	-0.0067
"00074dba19"	"00074dd577"	0.1733
"00074dba19"	"00074dd62e"	-0.1293
"00074dba19"	"00074dd73c"	0.087
"00074dba19"	"00074dda10"	0.063
"00074dba19"	"00074ddab8"	0.2046
"00074dba19"	"00074ddd3d"	-0.1728
"00074dba19"	"00074ddf16"	-0.072
"00074dba19"	"00074ddfc1"	0.1438
"00074dba19"	"00074de21a"	-0.2281
"00074dba19"	"00074de2a9"	0.1992
"00074dba19"	"00074de544"	0.1297
"00074dba19"	"00074de98a"	0.2191
"00074dba19"	"00074dea7e"	-0.1774
"00074dba19"	"00074debd9"	-0.078
"00074dba19"	"00074deca3"	-0.1517
"00074dba19"	"00074def43"	-0.3124
"00074dba19"	"00074def99"	-0.0382
"00074dba19"	"00074ecdad"	-0.5349
"00074dba19"	"00074ecf28"	-0.1771
"00074dba19"	"00074ed1e1"	-0.0998
"00074dba19"	"00074ed83b"	0.0271
"00074dba19"	"00074ee5e3"	-0.2113
"00074dba19"	"00074ee6e0"	-0.0466
"00074dba19"	"00074eea3a"	0.0667
"00074dba19"	"00074eff82"	-0.0938
"00074dba19"	"00074f0477"	0.0574
"00074dba19"	"00074f08c3"	-0.0629
"00074dba19"	"00074f1859"	0.0412
"00074dba19"	"00074f2268"	0.2688
"00074dba19"	"00074f28be"	0.1964
"00074dba19"	"00074f294b"	-0.1838
"00074dba19"	"00074f2ddd"	0.0473
"00074dba19"	"00074f2e75"	-0.1457
"00074dba19"	"00074f3088"	0.0415
"00074dba19"	"00074f5a1c"	0.2476
"00074dba19"	"00074f75b7"	-0.0559
"00074dba19"	"00074f8cd9"	0.0347
"00074dba19"	"00074f96dc"	-0.2483
"00074dba19"	"00074fabaa"	-0.0077
"00074dba19"	"00074facd9"	-0.0091
"00074dba19"	"00074fae3c"	-0.0683
"00074dba19"	"00074fb0a8"	-0.1229

"00074dba19"	"00074fb4e4"	-0.351
"00074dba19"	"00074fb7c2"	0.0972
"00074dba19"	"00074fbd36"	0.369
"00074dba19"	"00074fc27f"	0.2271
"00074dba19"	"00074fc31d"	-0.1583
"00074dba19"	"00074fd569"	-0.2426
"00074dba19"	"00074fef15"	-0.0021
"00074dba19"	"00074ff562"	-0.1643
"00074dba19"	"00075007ca"	-0.0976
"00074dba19"	"0007500b86"	0.1258
"00074dba19"	"0007500d05"	-0.0511
"00074dba19"	"0007500ee4"	-0.3036
"00074dba19"	"0007500eee"	0.0732
"00074dba19"	"00075013dc"	0.0078
"00074dba19"	"000757b515"	0.2814
"00074dba19"	"000757bc5a"	0.1065
"00074dba19"	"000757c320"	-0.0392
"00074dba19"	"000757c9aa"	0.0901
"00074dba19"	"000757ccbe"	0.0308
"00074dba19"	"000757cfa9"	-0.0332
"00074dba19"	"000757d390"	-0.4849
"00074dba19"	"000757d393"	0.1827
"00074dba19"	"000757d598"	-0.0748
"00074dba19"	"000757d5a2"	-0.02
"00074dba19"	"000757d790"	-0.0208
"00074dba19"	"000757e30c"	-0.116
"00074dba19"	"000757e4b0"	0.2213
"00074dba19"	"000757e7a0"	-0.336
"00074dba19"	"000757e8b3"	0.1208
"00074dba19"	"000757f627"	0.1482
"00074dba19"	"000757f925"	0.1674
"00074dba19"	"000757fa08"	1e-04
"00074dba19"	"000757fe52"	-0.0205
"00074dba19"	"000758024a"	-0.0168
"00074dba19"	"00075804bb"	0.1143
"00074dba19"	"00075a0c04"	0.0382
"00074dba19"	"00075a3110"	0.3189
"00074dba19"	"00075a341a"	-0.0184
"00074dba19"	"00075a3dcf"	0.0035
"00074dba19"	"00075a3e22"	0.3171
"00074dba19"	"00075a48d8"	-0.0013
"00074dba19"	"00075a5cfb"	0.0283
"00074dba19"	"00075a6151"	0.1446
"00074dba19"	"00075a6708"	-0.069
"00074dba19"	"00075a7319"	-0.4317
"00074dba19"	"00075a7723"	0.0161
"00074dba19"	"00075a778b"	-0.0718
"00074dba19"	"00075a7b8e"	-0.0396
"00074dba19"	"00075a7c79"	-0.0135
"00074dba19"	"00075a81b6"	0.0624

"00074dba19"	"00075a82ac"	0.2142
"00074dba19"	"00075a98e5"	0.112
"00074dba19"	"00075b0d29"	0.3146
"00074dba19"	"00075b102a"	0.0714
"00074dba19"	"00075b1074"	−0.171
"00074dba19"	"00075b135d"	0.1628
"00074dba19"	"00075b138b"	0.1733
"00074dba19"	"00075b13a0"	0.0224
"00074dba19"	"00075b13bd"	0.2302
"00074dba19"	"00075b16a9"	−0.2777
"00074dba19"	"00075b1a28"	−0.0171
"00074dba19"	"00075b1a97"	0.0163
"00074dba19"	"00075b1c7b"	−0.6526
"00074dba19"	"00075b1d24"	−0.0617
"00074dba19"	"00075b202b"	0.1216
"00074dba19"	"00075b22cb"	−0.2817
"00074dba19"	"00075b22da"	0.0098
"00074dba19"	"00075b2556"	−0.0224
"00074dba19"	"00075b25de"	0.1484
"00074dba19"	"00075b260c"	0.1414
"00074dba19"	"00075b26f1"	−0.0194
"00074dba19"	"00075b2920"	0.0219
"00074dba19"	"00075b2a64"	0.0403
"00074dba19"	"00075b2a9d"	0.159
"00074dba19"	"00075b2b37"	−0.1643
"00074dba19"	"00075b2cdd"	−0.2692
"00074dba19"	"00075b3038"	0.1061
"00074dba19"	"00075b30fe"	0.0167
"00074dba19"	"00075b3362"	−0.5412
"00074dba19"	"00075b350a"	0.0309
"00074dba19"	"00075b350e"	0.0447
"00074dba19"	"00075b3651"	−0.2932
"00074dba19"	"00075b38ca"	−0.128
"00074dba19"	"00075b39cc"	−0.176
"00074dba19"	"00075b3e1e"	0.0704
"00074dba19"	"00075b3e57"	−0.028
"00074dba19"	"00075b4079"	−0.076
"00074dba19"	"00075b4150"	0.1594
"00074dba19"	"00075b4194"	0.1032
"00074dba19"	"00075b42d5"	0.0051
"00074dba19"	"00075b4424"	−0.2077
"00074dba19"	"00075b4470"	0.1065
"00074dba19"	"00075b47ed"	−0.2283
"00074dba19"	"00075b4850"	0.0338
"00074dba19"	"00075b4ca0"	0.2308
"00074dba19"	"00075b4d7f"	0.2727
"00074dba19"	"00075b520f"	0.2388
"00074dba19"	"00075b525f"	−0.2301
"00074dba19"	"00075b58f8"	−2e−04
"00074dba19"	"00075b5bcc"	0.0754

"00074dba19"	"00075b5bfa"	-0.0777
"00074dba19"	"00075b6339"	0.1285
"00074dba19"	"00075b6658"	-0.2903
"00074dba19"	"00075b679a"	-0.1279
"00074dba19"	"00075b6cb7"	-0.0635
"00074dba19"	"00075b6df8"	0.0653
"00074dba19"	"00075b6ff6"	0.1098
"00074dba19"	"00075b70ee"	-0.1382
"00074dba19"	"00075b7157"	0.0265
"00074dba19"	"00075b7225"	0.097
"00074dba19"	"00075b7c89"	0.211
"00074dba19"	"00075b9048"	0.1992
"00074dba19"	"00075d0801"	0.0243
"00074dba19"	"00075d1820"	-0.2542
"00074dba19"	"00075d1f3d"	-0.0607
"00074dba19"	"00075d2329"	0.1117
"00074dba19"	"00075d2b9b"	0.0509
"00074dba19"	"00075d3941"	-0.0499
"00074dba19"	"00075d3e96"	-0.2198
"00074dba19"	"00075d4864"	0.1286
"00074dba19"	"00075d5961"	0.168
"00074dba19"	"00075d5a63"	0.0081
"00074dba19"	"00075d6150"	-0.3621
"00074dba19"	"00075d67d0"	-0.2994
"00074dba19"	"00075d67e2"	0.0759
"00074dba19"	"00075d73fc"	-0.0545
"00074dba19"	"00075d7729"	0.1381
"00074dba19"	"00075d778c"	-0.4493
"00074dba19"	"00075d7b9e"	-0.0364
"00074dba19"	"00075d7c8f"	0.1502
"00074dba19"	"00075d804d"	0.2429
"00074dba19"	"00075d819f"	0.1092
"00074dba19"	"00075d8601"	0.0951
"00074dba19"	"00075d8c6a"	0.1571
"00074dba19"	"00075dfedc"	0.0061
"00074dba19"	"00075e05f2"	0.0316
"00074dba19"	"00075e0837"	0.0822
"00074dba19"	"00075e092e"	-0.0887
"00074dba19"	"00075e0965"	-0.0707
"00074dba19"	"00075e0bc8"	0.3491
"00074dba19"	"00075e0fbb"	-0.0262
"00074dbc2e"	"00074dbe51"	-0.0323
"00074dbc2e"	"00074dbe5f"	-0.058
"00074dbc2e"	"00074dbf6d"	-0.0969
"00074dbc2e"	"00074dc4a5"	-0.2663
"00074dbc2e"	"00074dc50c"	0.2509
"00074dbc2e"	"00074dcdfa"	0.0163
"00074dbc2e"	"00074dcf5f"	-0.261
"00074dbc2e"	"00074dd007"	-0.2182
"00074dbc2e"	"00074dd163"	0.3077

"00074dbc2e"	"00074dd3df"	-0.3275
"00074dbc2e"	"00074dd577"	-0.0191
"00074dbc2e"	"00074dd62e"	-0.1274
"00074dbc2e"	"00074dd73c"	0.2888
"00074dbc2e"	"00074dda10"	-0.1869
"00074dbc2e"	"00074ddab8"	0.1136
"00074dbc2e"	"00074ddd3d"	-0.1698
"00074dbc2e"	"00074ddf16"	0.2339
"00074dbc2e"	"00074ddfc1"	0.3293
"00074dbc2e"	"00074de21a"	-0.3398
"00074dbc2e"	"00074de2a9"	-0.1336
"00074dbc2e"	"00074de544"	-0.1317
"00074dbc2e"	"00074de98a"	-0.0342
"00074dbc2e"	"00074dea7e"	-0.1609
"00074dbc2e"	"00074debd9"	0.2084
"00074dbc2e"	"00074deca3"	0.2933
"00074dbc2e"	"00074def43"	0.3726
"00074dbc2e"	"00074def99"	-0.2287
"00074dbc2e"	"00074ecdad"	0.0545
"00074dbc2e"	"00074ecf28"	-0.251
"00074dbc2e"	"00074ed1e1"	-0.0396
"00074dbc2e"	"00074ed83b"	0.0235
"00074dbc2e"	"00074ee5e3"	0.3473
"00074dbc2e"	"00074ee6e0"	-0.0932
"00074dbc2e"	"00074eea3a"	-0.0012
"00074dbc2e"	"00074eff82"	0.0613
"00074dbc2e"	"00074f0477"	-0.1887
"00074dbc2e"	"00074f08c3"	0.0326
"00074dbc2e"	"00074f1859"	0.1958
"00074dbc2e"	"00074f2268"	-0.4865
"00074dbc2e"	"00074f28be"	0.0485
"00074dbc2e"	"00074f294b"	-0.1429
"00074dbc2e"	"00074f2ddd"	-0.0537
"00074dbc2e"	"00074f2e75"	-0.3268
"00074dbc2e"	"00074f3088"	-0.249
"00074dbc2e"	"00074f5a1c"	0.2361
"00074dbc2e"	"00074f75b7"	0.353
"00074dbc2e"	"00074f8cd9"	0.1926
"00074dbc2e"	"00074f96dc"	-0.3314
"00074dbc2e"	"00074fabaa"	0.0074
"00074dbc2e"	"00074facd9"	-0.097
"00074dbc2e"	"00074fae3c"	0.023
"00074dbc2e"	"00074fb0a8"	-0.1328
"00074dbc2e"	"00074fb4e4"	-0.0899
"00074dbc2e"	"00074fb7c2"	0.027
"00074dbc2e"	"00074fbd36"	-0.4853
"00074dbc2e"	"00074fc27f"	0.0392
"00074dbc2e"	"00074fc31d"	-0.2318
"00074dbc2e"	"00074fd569"	-0.0416
"00074dbc2e"	"00074fef15"	-0.1829

"00074dbc2e"	"00074ff562"	-0.2169
"00074dbc2e"	"00075007ca"	0.0203
"00074dbc2e"	"0007500b86"	0.0746
"00074dbc2e"	"0007500d05"	0.1183
"00074dbc2e"	"0007500ee4"	-0.3141
"00074dbc2e"	"0007500eee"	0.035
"00074dbc2e"	"00075013dc"	-0.4402
"00074dbc2e"	"000757b515"	-0.049
"00074dbc2e"	"000757bc5a"	0.0165
"00074dbc2e"	"000757c320"	0.1944
"00074dbc2e"	"000757c9aa"	0.0634
"00074dbc2e"	"000757ccbe"	-0.2372
"00074dbc2e"	"000757cfa9"	-0.2135
"00074dbc2e"	"000757d390"	-0.1516
"00074dbc2e"	"000757d393"	0.1351
"00074dbc2e"	"000757d598"	-0.1231
"00074dbc2e"	"000757d5a2"	-0.218
"00074dbc2e"	"000757d790"	0.3074
"00074dbc2e"	"000757e30c"	0.0468
"00074dbc2e"	"000757e4b0"	0.0288
"00074dbc2e"	"000757e7a0"	-0.2169
"00074dbc2e"	"000757e8b3"	-0.1267
"00074dbc2e"	"000757f627"	-0.045
"00074dbc2e"	"000757f925"	0.0857
"00074dbc2e"	"000757fa08"	-0.0412
"00074dbc2e"	"000757fe52"	-0.0117
"00074dbc2e"	"000758024a"	-0.1686
"00074dbc2e"	"00075804bb"	0.041
"00074dbc2e"	"00075a0c04"	0.0076
"00074dbc2e"	"00075a3110"	0.165
"00074dbc2e"	"00075a341a"	-0.2805
"00074dbc2e"	"00075a3dcf"	0.1012
"00074dbc2e"	"00075a3e22"	-0.1185
"00074dbc2e"	"00075a48d8"	-0.0123
"00074dbc2e"	"00075a5cfb"	-0.1119
"00074dbc2e"	"00075a6151"	0.1243
"00074dbc2e"	"00075a6708"	0.0592
"00074dbc2e"	"00075a7319"	-0.1435
"00074dbc2e"	"00075a7723"	-0.0692
"00074dbc2e"	"00075a778b"	-0.2566
"00074dbc2e"	"00075a7b8e"	-0.0668
"00074dbc2e"	"00075a7c79"	0.2384
"00074dbc2e"	"00075a81b6"	-0.2623
"00074dbc2e"	"00075a82ac"	-0.1553
"00074dbc2e"	"00075a98e5"	-0.2789
"00074dbc2e"	"00075b0d29"	-0.2518
"00074dbc2e"	"00075b102a"	0.0849
"00074dbc2e"	"00075b1074"	-0.081
"00074dbc2e"	"00075b135d"	-0.3117
"00074dbc2e"	"00075b138b"	0.1179

"00074dbc2e"	"00075b13a0"	−0.3276
"00074dbc2e"	"00075b13bd"	−0.4254
"00074dbc2e"	"00075b16a9"	−0.1336
"00074dbc2e"	"00075b1a28"	−0.1168
"00074dbc2e"	"00075b1a97"	−0.0773
"00074dbc2e"	"00075b1c7b"	−0.2538
"00074dbc2e"	"00075b1d24"	−0.1509
"00074dbc2e"	"00075b202b"	0.1136
"00074dbc2e"	"00075b22cb"	−0.111
"00074dbc2e"	"00075b22da"	−0.0102
"00074dbc2e"	"00075b2556"	−0.1998
"00074dbc2e"	"00075b25de"	0.2127
"00074dbc2e"	"00075b260c"	0.0832
"00074dbc2e"	"00075b26f1"	0.1774
"00074dbc2e"	"00075b2920"	0.0585
"00074dbc2e"	"00075b2a64"	−0.1085
"00074dbc2e"	"00075b2a9d"	−0.0279
"00074dbc2e"	"00075b2b37"	−0.4757
"00074dbc2e"	"00075b2cdd"	0.0346
"00074dbc2e"	"00075b3038"	0.4245
"00074dbc2e"	"00075b30fe"	0.0116
"00074dbc2e"	"00075b3362"	−0.2271
"00074dbc2e"	"00075b350a"	−0.245
"00074dbc2e"	"00075b350e"	−0.042
"00074dbc2e"	"00075b3651"	−0.0409
"00074dbc2e"	"00075b38ca"	0.2006
"00074dbc2e"	"00075b39cc"	0.1055
"00074dbc2e"	"00075b3e1e"	0.2752
"00074dbc2e"	"00075b3e57"	−0.1412
"00074dbc2e"	"00075b4079"	−0.1086
"00074dbc2e"	"00075b4150"	−0.2204
"00074dbc2e"	"00075b4194"	0.1204
"00074dbc2e"	"00075b42d5"	0.277
"00074dbc2e"	"00075b4424"	−0.1824
"00074dbc2e"	"00075b4470"	0.0921
"00074dbc2e"	"00075b47ed"	0.0414
"00074dbc2e"	"00075b4850"	0.1065
"00074dbc2e"	"00075b4ca0"	−0.2184
"00074dbc2e"	"00075b4d7f"	−0.0865
"00074dbc2e"	"00075b520f"	−0.1757
"00074dbc2e"	"00075b525f"	−0.0465
"00074dbc2e"	"00075b58f8"	−0.2898
"00074dbc2e"	"00075b5bcc"	0.0678
"00074dbc2e"	"00075b5bfa"	0.2269
"00074dbc2e"	"00075b6339"	0.0505
"00074dbc2e"	"00075b6658"	−0.1359
"00074dbc2e"	"00075b679a"	−0.034
"00074dbc2e"	"00075b6cb7"	−0.0799
"00074dbc2e"	"00075b6df8"	0.0083
"00074dbc2e"	"00075b6ff6"	−0.0392

"00074dbc2e"	"00075b70ee"	−0.0603
"00074dbc2e"	"00075b7157"	−0.0169
"00074dbc2e"	"00075b7225"	−0.0287
"00074dbc2e"	"00075b7c89"	−0.1102
"00074dbc2e"	"00075b9048"	0.2058
"00074dbc2e"	"00075d0801"	−0.0643
"00074dbc2e"	"00075d1820"	−0.2592
"00074dbc2e"	"00075d1f3d"	0.0045
"00074dbc2e"	"00075d2329"	0.1024
"00074dbc2e"	"00075d2b9b"	0.1552
"00074dbc2e"	"00075d3941"	−0.2329
"00074dbc2e"	"00075d3e96"	−0.0957
"00074dbc2e"	"00075d4864"	−0.0765
"00074dbc2e"	"00075d5961"	0.0233
"00074dbc2e"	"00075d5a63"	0.2864
"00074dbc2e"	"00075d6150"	0.0315
"00074dbc2e"	"00075d67d0"	−0.106
"00074dbc2e"	"00075d67e2"	−0.1992
"00074dbc2e"	"00075d73fc"	−0.1072
"00074dbc2e"	"00075d7729"	−0.3111
"00074dbc2e"	"00075d778c"	−0.0384
"00074dbc2e"	"00075d7b9e"	−0.0889
"00074dbc2e"	"00075d7c8f"	−0.1032
"00074dbc2e"	"00075d804d"	0.0504
"00074dbc2e"	"00075d819f"	0.0537
"00074dbc2e"	"00075d8601"	0.0766
"00074dbc2e"	"00075d8c6a"	0.0975
"00074dbc2e"	"00075dfedc"	0.0913
"00074dbc2e"	"00075e05f2"	0.1789
"00074dbc2e"	"00075e0837"	0.1494
"00074dbc2e"	"00075e092e"	0.0631
"00074dbc2e"	"00075e0965"	−0.1619
"00074dbc2e"	"00075e0bc8"	0.066
"00074dbc2e"	"00075e0fbb"	−0.0532
"00074dbe51"	"00074dbe5f"	−0.1479
"00074dbe51"	"00074dbf6d"	0.0038
"00074dbe51"	"00074dc4a5"	−0.116
"00074dbe51"	"00074dc50c"	0.1157
"00074dbe51"	"00074dcdfa"	2e−04
"00074dbe51"	"00074dcf5f"	0.0959
"00074dbe51"	"00074dd007"	−0.0655
"00074dbe51"	"00074dd163"	−0.0512
"00074dbe51"	"00074dd3df"	−0.024
"00074dbe51"	"00074dd577"	0.4877
"00074dbe51"	"00074dd62e"	0.0207
"00074dbe51"	"00074dd73c"	0.2471
"00074dbe51"	"00074dda10"	−0.0147
"00074dbe51"	"00074ddab8"	0.0109
"00074dbe51"	"00074ddd3d"	0.2704
"00074dbe51"	"00074ddf16"	0.2214

"00074dbe51"	"00074ddfc1"	0.0909
"00074dbe51"	"00074de21a"	-0.2113
"00074dbe51"	"00074de2a9"	0.0022
"00074dbe51"	"00074de544"	0.2747
"00074dbe51"	"00074de98a"	0.0661
"00074dbe51"	"00074dea7e"	0.1106
"00074dbe51"	"00074debd9"	-0.2031
"00074dbe51"	"00074deca3"	0.1352
"00074dbe51"	"00074def43"	0.1821
"00074dbe51"	"00074def99"	0.1495
"00074dbe51"	"00074ecdad"	-0.0022
"00074dbe51"	"00074ecf28"	-0.2152
"00074dbe51"	"00074ed1e1"	0.2275
"00074dbe51"	"00074ed83b"	0.2573
"00074dbe51"	"00074ee5e3"	-0.3456
"00074dbe51"	"00074ee6e0"	-0.0163
"00074dbe51"	"00074eea3a"	0.3073
"00074dbe51"	"00074eff82"	0.0047
"00074dbe51"	"00074f0477"	0.0638
"00074dbe51"	"00074f08c3"	-0.3343
"00074dbe51"	"00074f1859"	-0.0795
"00074dbe51"	"00074f2268"	0.159
"00074dbe51"	"00074f28be"	0.0928
"00074dbe51"	"00074f294b"	0.3773
"00074dbe51"	"00074f2ddd"	0.3714
"00074dbe51"	"00074f2e75"	0.124
"00074dbe51"	"00074f3088"	-0.0866
"00074dbe51"	"00074f5a1c"	-0.0252
"00074dbe51"	"00074f75b7"	0.0254
"00074dbe51"	"00074f8cd9"	0.1399
"00074dbe51"	"00074f96dc"	-0.2201
"00074dbe51"	"00074fabaa"	-0.1158
"00074dbe51"	"00074facd9"	-0.1516
"00074dbe51"	"00074fae3c"	-0.2999
"00074dbe51"	"00074fb0a8"	-0.0628
"00074dbe51"	"00074fb4e4"	-0.4597
"00074dbe51"	"00074fb7c2"	0.3685
"00074dbe51"	"00074fbd36"	0.0183
"00074dbe51"	"00074fc27f"	0.2438
"00074dbe51"	"00074fc31d"	-0.0673
"00074dbe51"	"00074fd569"	-0.006
"00074dbe51"	"00074fef15"	0.1048
"00074dbe51"	"00074ff562"	-0.3276
"00074dbe51"	"00075007ca"	0.0536
"00074dbe51"	"0007500b86"	0.108
"00074dbe51"	"0007500d05"	0.0693
"00074dbe51"	"0007500ee4"	0.0319
"00074dbe51"	"0007500eee"	-0.1768
"00074dbe51"	"00075013dc"	-0.2392
"00074dbe51"	"000757b515"	0.4204

"00074dbe51"	"000757bc5a"	0.076
"00074dbe51"	"000757c320"	-0.1811
"00074dbe51"	"000757c9aa"	0.0676
"00074dbe51"	"000757ccbe"	0.2697
"00074dbe51"	"000757cfa9"	0.1405
"00074dbe51"	"000757d390"	-0.1912
"00074dbe51"	"000757d393"	0.2849
"00074dbe51"	"000757d598"	0.1325
"00074dbe51"	"000757d5a2"	0.1579
"00074dbe51"	"000757d790"	0.372
"00074dbe51"	"000757e30c"	0.1067
"00074dbe51"	"000757e4b0"	0.0697
"00074dbe51"	"000757e7a0"	0.1811
"00074dbe51"	"000757e8b3"	0.4712
"00074dbe51"	"000757f627"	-0.1038
"00074dbe51"	"000757f925"	0.258
"00074dbe51"	"000757fa08"	0.2634
"00074dbe51"	"000757fe52"	0.3389
"00074dbe51"	"000758024a"	0.0374
"00074dbe51"	"00075804bb"	-0.0166
"00074dbe51"	"00075a0c04"	0.3802
"00074dbe51"	"00075a3110"	0.2602
"00074dbe51"	"00075a341a"	0.2897
"00074dbe51"	"00075a3dcf"	0.2856
"00074dbe51"	"00075a3e22"	0.326
"00074dbe51"	"00075a48d8"	0.2947
"00074dbe51"	"00075a5cfb"	0.0461
"00074dbe51"	"00075a6151"	0.2236
"00074dbe51"	"00075a6708"	0.0696
"00074dbe51"	"00075a7319"	-0.2099
"00074dbe51"	"00075a7723"	0.3106
"00074dbe51"	"00075a778b"	0.2616
"00074dbe51"	"00075a7b8e"	-0.0716
"00074dbe51"	"00075a7c79"	0.4965
"00074dbe51"	"00075a81b6"	0.1795
"00074dbe51"	"00075a82ac"	0.1832
"00074dbe51"	"00075a98e5"	0.0077
"00074dbe51"	"00075b0d29"	0.0428
"00074dbe51"	"00075b102a"	0.1495
"00074dbe51"	"00075b1074"	0.1577
"00074dbe51"	"00075b135d"	0.1825
"00074dbe51"	"00075b138b"	0.2641
"00074dbe51"	"00075b13a0"	0.1748
"00074dbe51"	"00075b13bd"	-0.1188
"00074dbe51"	"00075b16a9"	0.3659
"00074dbe51"	"00075b1a28"	-0.0808
"00074dbe51"	"00075b1a97"	0.1661
"00074dbe51"	"00075b1c7b"	-0.061
"00074dbe51"	"00075b1d24"	0.3697
"00074dbe51"	"00075b202b"	-0.0218

"00074dbe51"	"00075b22cb"	0.0637
"00074dbe51"	"00075b22da"	0.2296
"00074dbe51"	"00075b2556"	0.1719
"00074dbe51"	"00075b25de"	0.0738
"00074dbe51"	"00075b260c"	0.2533
"00074dbe51"	"00075b26f1"	−0.0983
"00074dbe51"	"00075b2920"	0.0094
"00074dbe51"	"00075b2a64"	−0.0438
"00074dbe51"	"00075b2a9d"	0.0301
"00074dbe51"	"00075b2b37"	−0.1138
"00074dbe51"	"00075b2cdd"	0.0356
"00074dbe51"	"00075b3038"	0.1698
"00074dbe51"	"00075b30fe"	0.1543
"00074dbe51"	"00075b3362"	0.1061
"00074dbe51"	"00075b350a"	−0.0805
"00074dbe51"	"00075b350e"	0.003
"00074dbe51"	"00075b3651"	−0.097
"00074dbe51"	"00075b38ca"	−0.1581
"00074dbe51"	"00075b39cc"	0.144
"00074dbe51"	"00075b3e1e"	0.0794
"00074dbe51"	"00075b3e57"	0.033
"00074dbe51"	"00075b4079"	−0.1388
"00074dbe51"	"00075b4150"	0.1258
"00074dbe51"	"00075b4194"	0.0772
"00074dbe51"	"00075b42d5"	0.1825
"00074dbe51"	"00075b4424"	−0.089
"00074dbe51"	"00075b4470"	0.076
"00074dbe51"	"00075b47ed"	0.0828
"00074dbe51"	"00075b4850"	0.1332
"00074dbe51"	"00075b4ca0"	0.2047
"00074dbe51"	"00075b4d7f"	0.3962
"00074dbe51"	"00075b520f"	−0.0894
"00074dbe51"	"00075b525f"	0.2051
"00074dbe51"	"00075b58f8"	−0.0811
"00074dbe51"	"00075b5bcc"	0.121
"00074dbe51"	"00075b5bfa"	−0.1909
"00074dbe51"	"00075b6339"	0.7616
"00074dbe51"	"00075b6658"	−0.0703
"00074dbe51"	"00075b679a"	0.2693
"00074dbe51"	"00075b6cb7"	0.0563
"00074dbe51"	"00075b6df8"	0.2252
"00074dbe51"	"00075b6ff6"	0.4146
"00074dbe51"	"00075b70ee"	0.13
"00074dbe51"	"00075b7157"	0.1249
"00074dbe51"	"00075b7225"	0.3086
"00074dbe51"	"00075b7c89"	0.4321
"00074dbe51"	"00075b9048"	0.2639
"00074dbe51"	"00075d0801"	0.0561
"00074dbe51"	"00075d1820"	0.0459
"00074dbe51"	"00075d1f3d"	0.0277

"00074dbe51"	"00075d2329"	0.1528
"00074dbe51"	"00075d2b9b"	-0.0892
"00074dbe51"	"00075d3941"	-0.2227
"00074dbe51"	"00075d3e96"	0.0123
"00074dbe51"	"00075d4864"	-0.136
"00074dbe51"	"00075d5961"	0.2454
"00074dbe51"	"00075d5a63"	0.0918
"00074dbe51"	"00075d6150"	-0.0948
"00074dbe51"	"00075d67d0"	-0.0044
"00074dbe51"	"00075d67e2"	0.311
"00074dbe51"	"00075d73fc"	0.1731
"00074dbe51"	"00075d7729"	-0.1174
"00074dbe51"	"00075d778c"	0.247
"00074dbe51"	"00075d7b9e"	0.1126
"00074dbe51"	"00075d7c8f"	0.6458
"00074dbe51"	"00075d804d"	0.1371
"00074dbe51"	"00075d819f"	-0.0162
"00074dbe51"	"00075d8601"	-0.1763
"00074dbe51"	"00075d8c6a"	0.2061
"00074dbe51"	"00075dfedc"	0.4672
"00074dbe51"	"00075e05f2"	0.1538
"00074dbe51"	"00075e0837"	0.0769
"00074dbe51"	"00075e092e"	0.1529
"00074dbe51"	"00075e0965"	-0.115
"00074dbe51"	"00075e0bc8"	0.1595
"00074dbe51"	"00075e0fbb"	0.1666
"00074dbe5f"	"00074dbf6d"	-0.1342
"00074dbe5f"	"00074dc4a5"	-0.4375
"00074dbe5f"	"00074dc50c"	-0.1635
"00074dbe5f"	"00074dcdfa"	-0.2314
"00074dbe5f"	"00074dcf5f"	-0.5583
"00074dbe5f"	"00074dd007"	-0.195
"00074dbe5f"	"00074dd163"	0.0273
"00074dbe5f"	"00074dd3df"	-0.1059
"00074dbe5f"	"00074dd577"	-0.34
"00074dbe5f"	"00074dd62e"	-0.2336
"00074dbe5f"	"00074dd73c"	-0.0488
"00074dbe5f"	"00074dda10"	0.1144
"00074dbe5f"	"00074ddab8"	-0.0287
"00074dbe5f"	"00074ddd3d"	-0.5466
"00074dbe5f"	"00074ddf16"	0.2421
"00074dbe5f"	"00074ddfc1"	0.263
"00074dbe5f"	"00074de21a"	-0.5575
"00074dbe5f"	"00074de2a9"	-0.2428
"00074dbe5f"	"00074de544"	0.0151
"00074dbe5f"	"00074de98a"	-0.0284
"00074dbe5f"	"00074dea7e"	-0.336
"00074dbe5f"	"00074debd9"	0.3756
"00074dbe5f"	"00074deca3"	-0.0809
"00074dbe5f"	"00074def43"	0.1244

"00074dbe5f"	"00074def99"	−0.548
"00074dbe5f"	"00074ecdad"	−0.2173
"00074dbe5f"	"00074ecf28"	−0.2459
"00074dbe5f"	"00074ed1e1"	−0.3772
"00074dbe5f"	"00074ed83b"	−0.2155
"00074dbe5f"	"00074ee5e3"	0.1944
"00074dbe5f"	"00074ee6e0"	−0.1098
"00074dbe5f"	"00074eea3a"	−0.1156
"00074dbe5f"	"00074eff82"	−0.0708
"00074dbe5f"	"00074f0477"	0.0997
"00074dbe5f"	"00074f08c3"	−0.6251
"00074dbe5f"	"00074f1859"	0.0413
"00074dbe5f"	"00074f2268"	−0.4893
"00074dbe5f"	"00074f28be"	−0.2635
"00074dbe5f"	"00074f294b"	−0.3701
"00074dbe5f"	"00074f2ddd"	−0.5723
"00074dbe5f"	"00074f2e75"	−0.3186
"00074dbe5f"	"00074f3088"	−0.0863
"00074dbe5f"	"00074f5a1c"	−0.0709
"00074dbe5f"	"00074f75b7"	0.1296
"00074dbe5f"	"00074f8cd9"	0.2704
"00074dbe5f"	"00074f96dc"	−0.2718
"00074dbe5f"	"00074fabaa"	−0.0776
"00074dbe5f"	"00074facd9"	0.1513
"00074dbe5f"	"00074fae3c"	−0.1062
"00074dbe5f"	"00074fb0a8"	−0.2499
"00074dbe5f"	"00074fb4e4"	−0.3487
"00074dbe5f"	"00074fb7c2"	−0.0793
"00074dbe5f"	"00074fbd36"	−0.3723
"00074dbe5f"	"00074fc27f"	−0.1876
"00074dbe5f"	"00074fc31d"	−0.4102
"00074dbe5f"	"00074fd569"	−0.2663
"00074dbe5f"	"00074fef15"	−0.2424
"00074dbe5f"	"00074ff562"	−0.2746
"00074dbe5f"	"00075007ca"	1e−04
"00074dbe5f"	"0007500b86"	0.0582
"00074dbe5f"	"0007500d05"	0.4723
"00074dbe5f"	"0007500ee4"	−0.3244
"00074dbe5f"	"0007500eee"	0.7224
"00074dbe5f"	"00075013dc"	−0.4961
"00074dbe5f"	"000757b515"	−0.2131
"00074dbe5f"	"000757bc5a"	−0.0746
"00074dbe5f"	"000757c320"	−0.2017
"00074dbe5f"	"000757c9aa"	−0.315
"00074dbe5f"	"000757ccbe"	−0.4235
"00074dbe5f"	"000757cfa9"	−0.3556
"00074dbe5f"	"000757d390"	−0.4566
"00074dbe5f"	"000757d393"	0.1818
"00074dbe5f"	"000757d598"	−0.4917
"00074dbe5f"	"000757d5a2"	−0.5417

"00074dbe5f"	"000757d790"	-0.0059
"00074dbe5f"	"000757e30c"	-0.3348
"00074dbe5f"	"000757e4b0"	-0.1655
"00074dbe5f"	"000757e7a0"	-0.2576
"00074dbe5f"	"000757e8b3"	-0.055
"00074dbe5f"	"000757f627"	-0.1379
"00074dbe5f"	"000757f925"	0.0937
"00074dbe5f"	"000757fa08"	-0.1321
"00074dbe5f"	"000757fe52"	-0.0684
"00074dbe5f"	"000758024a"	-0.3543
"00074dbe5f"	"00075804bb"	-0.135
"00074dbe5f"	"00075a0c04"	-0.0094
"00074dbe5f"	"00075a3110"	-0.3602
"00074dbe5f"	"00075a341a"	-0.4439
"00074dbe5f"	"00075a3dcf"	0.05
"00074dbe5f"	"00075a3e22"	-0.2045
"00074dbe5f"	"00075a48d8"	-0.0735
"00074dbe5f"	"00075a5cfb"	-0.3323
"00074dbe5f"	"00075a6151"	-0.0621
"00074dbe5f"	"00075a6708"	-0.516
"00074dbe5f"	"00075a7319"	-0.4806
"00074dbe5f"	"00075a7723"	-0.1118
"00074dbe5f"	"00075a778b"	-0.4611
"00074dbe5f"	"00075a7b8e"	-0.0666
"00074dbe5f"	"00075a7c79"	-0.2693
"00074dbe5f"	"00075a81b6"	-0.3004
"00074dbe5f"	"00075a82ac"	-0.074
"00074dbe5f"	"00075a98e5"	-0.1584
"00074dbe5f"	"00075b0d29"	-0.2757
"00074dbe5f"	"00075b102a"	0.1781
"00074dbe5f"	"00075b1074"	-0.4438
"00074dbe5f"	"00075b135d"	-0.4172
"00074dbe5f"	"00075b138b"	-0.0253
"00074dbe5f"	"00075b13a0"	-0.2856
"00074dbe5f"	"00075b13bd"	-0.2302
"00074dbe5f"	"00075b16a9"	0.2094
"00074dbe5f"	"00075b1a28"	-0.326
"00074dbe5f"	"00075b1a97"	-0.3119
"00074dbe5f"	"00075b1c7b"	-0.165
"00074dbe5f"	"00075b1d24"	-0.2323
"00074dbe5f"	"00075b202b"	-0.0052
"00074dbe5f"	"00075b22cb"	0.0139
"00074dbe5f"	"00075b22da"	-0.4128
"00074dbe5f"	"00075b2556"	-0.3022
"00074dbe5f"	"00075b25de"	0.1289
"00074dbe5f"	"00075b260c"	-0.229
"00074dbe5f"	"00075b26f1"	-0.2837
"00074dbe5f"	"00075b2920"	-0.138
"00074dbe5f"	"00075b2a64"	-0.172
"00074dbe5f"	"00075b2a9d"	-0.1218

"00074dbe5f"	"00075b2b37"	-0.2292
"00074dbe5f"	"00075b2cdd"	-0.1877
"00074dbe5f"	"00075b3038"	-0.0051
"00074dbe5f"	"00075b30fe"	-0.3303
"00074dbe5f"	"00075b3362"	-0.3095
"00074dbe5f"	"00075b350a"	-0.3213
"00074dbe5f"	"00075b350e"	-0.3035
"00074dbe5f"	"00075b3651"	-0.2388
"00074dbe5f"	"00075b38ca"	-0.0241
"00074dbe5f"	"00075b39cc"	-0.058
"00074dbe5f"	"00075b3e1e"	0.2425
"00074dbe5f"	"00075b3e57"	-0.0615
"00074dbe5f"	"00075b4079"	-0.2068
"00074dbe5f"	"00075b4150"	-0.2869
"00074dbe5f"	"00075b4194"	-0.1881
"00074dbe5f"	"00075b42d5"	0.0228
"00074dbe5f"	"00075b4424"	-0.5053
"00074dbe5f"	"00075b4470"	-0.0052
"00074dbe5f"	"00075b47ed"	-0.1605
"00074dbe5f"	"00075b4850"	-0.1573
"00074dbe5f"	"00075b4ca0"	-0.5581
"00074dbe5f"	"00075b4d7f"	0.1273
"00074dbe5f"	"00075b520f"	-0.1956
"00074dbe5f"	"00075b525f"	-0.6254
"00074dbe5f"	"00075b58f8"	-0.1011
"00074dbe5f"	"00075b5bcc"	-0.1589
"00074dbe5f"	"00075b5bfa"	-0.1189
"00074dbe5f"	"00075b6339"	-0.1356
"00074dbe5f"	"00075b6658"	-0.1913
"00074dbe5f"	"00075b679a"	-0.2183
"00074dbe5f"	"00075b6cb7"	-0.177
"00074dbe5f"	"00075b6df8"	-0.1293
"00074dbe5f"	"00075b6ff6"	-0.3623
"00074dbe5f"	"00075b70ee"	-0.3743
"00074dbe5f"	"00075b7157"	-0.0278
"00074dbe5f"	"00075b7225"	-0.3127
"00074dbe5f"	"00075b7c89"	-0.2977
"00074dbe5f"	"00075b9048"	-0.2032
"00074dbe5f"	"00075d0801"	0.0658
"00074dbe5f"	"00075d1820"	-0.2011
"00074dbe5f"	"00075d1f3d"	-0.197
"00074dbe5f"	"00075d2329"	0.0461
"00074dbe5f"	"00075d2b9b"	-0.1446
"00074dbe5f"	"00075d3941"	-0.2155
"00074dbe5f"	"00075d3e96"	-0.3012
"00074dbe5f"	"00075d4864"	-0.1008
"00074dbe5f"	"00075d5961"	0.1056
"00074dbe5f"	"00075d5a63"	0.0612
"00074dbe5f"	"00075d6150"	-0.3324
"00074dbe5f"	"00075d67d0"	-0.2641

"00074dbe5f"	"00075d67e2"	-0.1029
"00074dbe5f"	"00075d73fc"	-0.2176
"00074dbe5f"	"00075d7729"	-0.5615
"00074dbe5f"	"00075d778c"	-0.2929
"00074dbe5f"	"00075d7b9e"	-0.0254
"00074dbe5f"	"00075d7c8f"	-0.1688
"00074dbe5f"	"00075d804d"	0.0156
"00074dbe5f"	"00075d819f"	-0.1373
"00074dbe5f"	"00075d8601"	0.0123
"00074dbe5f"	"00075d8c6a"	-0.256
"00074dbe5f"	"00075dfedc"	0.0694
"00074dbe5f"	"00075e05f2"	-0.012
"00074dbe5f"	"00075e0837"	0.0873
"00074dbe5f"	"00075e092e"	-0.5025
"00074dbe5f"	"00075e0965"	-0.399
"00074dbe5f"	"00075e0bc8"	-0.0941
"00074dbe5f"	"00075e0fbb"	-0.366
"00074dbf6d"	"00074dc4a5"	-0.569
"00074dbf6d"	"00074dc50c"	0.0722
"00074dbf6d"	"00074dcdfa"	-0.1146
"00074dbf6d"	"00074dcf5f"	0.0393
"00074dbf6d"	"00074dd007"	0.0852
"00074dbf6d"	"00074dd163"	-0.2716
"00074dbf6d"	"00074dd3df"	-0.4012
"00074dbf6d"	"00074dd577"	-0.1151
"00074dbf6d"	"00074dd62e"	-0.1005
"00074dbf6d"	"00074dd73c"	-0.1501
"00074dbf6d"	"00074dda10"	-0.1775
"00074dbf6d"	"00074ddab8"	0.1622
"00074dbf6d"	"00074ddd3d"	0.1505
"00074dbf6d"	"00074ddf16"	-3e-04
"00074dbf6d"	"00074ddfc1"	0.1228
"00074dbf6d"	"00074de21a"	-0.3062
"00074dbf6d"	"00074de2a9"	0.1313
"00074dbf6d"	"00074de544"	-0.3209
"00074dbf6d"	"00074de98a"	-0.2141
"00074dbf6d"	"00074dea7e"	-0.328
"00074dbf6d"	"00074debd9"	0.1075
"00074dbf6d"	"00074deca3"	-0.0569
"00074dbf6d"	"00074def43"	0.0681
"00074dbf6d"	"00074def99"	-0.3592
"00074dbf6d"	"00074ecdad"	-0.304
"00074dbf6d"	"00074ecf28"	-0.2308
"00074dbf6d"	"00074ed1e1"	-0.3016
"00074dbf6d"	"00074ed83b"	-0.1022
"00074dbf6d"	"00074ee5e3"	-0.0261
"00074dbf6d"	"00074ee6e0"	-0.0015
"00074dbf6d"	"00074eea3a"	-0.3862
"00074dbf6d"	"00074eff82"	-0.3197
"00074dbf6d"	"00074f0477"	-0.2026

"00074dbf6d"	"00074f08c3"	-0.021
"00074dbf6d"	"00074f1859"	-0.251
"00074dbf6d"	"00074f2268"	0.0882
"00074dbf6d"	"00074f28be"	0.1349
"00074dbf6d"	"00074f294b"	-0.2638
"00074dbf6d"	"00074f2ddd"	0.0028
"00074dbf6d"	"00074f2e75"	-0.531
"00074dbf6d"	"00074f3088"	-0.2578
"00074dbf6d"	"00074f5a1c"	-0.2402
"00074dbf6d"	"00074f75b7"	-0.1376
"00074dbf6d"	"00074f8cd9"	0.002
"00074dbf6d"	"00074f96dc"	-0.2862
"00074dbf6d"	"00074fabaa"	0.0844
"00074dbf6d"	"00074facd9"	-0.1811
"00074dbf6d"	"00074fae3c"	-0.1902
"00074dbf6d"	"00074fb0a8"	-0.1644
"00074dbf6d"	"00074fb4e4"	-0.172
"00074dbf6d"	"00074fb7c2"	-0.2972
"00074dbf6d"	"00074fbd36"	-0.1667
"00074dbf6d"	"00074fc27f"	0.229
"00074dbf6d"	"00074fc31d"	-0.346
"00074dbf6d"	"00074fd569"	-0.4104
"00074dbf6d"	"00074fef15"	-0.4554
"00074dbf6d"	"00074ff562"	-0.4013
"00074dbf6d"	"00075007ca"	0.2153
"00074dbf6d"	"0007500b86"	0.1273
"00074dbf6d"	"0007500d05"	-0.1845
"00074dbf6d"	"0007500ee4"	-0.4427
"00074dbf6d"	"0007500eee"	-0.0887
"00074dbf6d"	"00075013dc"	0.1703
"00074dbf6d"	"000757b515"	0.2017
"00074dbf6d"	"000757bc5a"	0.1028
"00074dbf6d"	"000757c320"	0.4757
"00074dbf6d"	"000757c9aa"	0.2012
"00074dbf6d"	"000757ccbe"	-0.1818
"00074dbf6d"	"000757cfa9"	-0.0157
"00074dbf6d"	"000757d390"	-0.1575
"00074dbf6d"	"000757d393"	0.0625
"00074dbf6d"	"000757d598"	0.3284
"00074dbf6d"	"000757d5a2"	0.1222
"00074dbf6d"	"000757d790"	-0.1175
"00074dbf6d"	"000757e30c"	0.3006
"00074dbf6d"	"000757e4b0"	-0.0883
"00074dbf6d"	"000757e7a0"	-0.2588
"00074dbf6d"	"000757e8b3"	0.0699
"00074dbf6d"	"000757f627"	0.0792
"00074dbf6d"	"000757f925"	0.3108
"00074dbf6d"	"000757fa08"	-0.4005
"00074dbf6d"	"000757fe52"	-0.1584
"00074dbf6d"	"000758024a"	0.1371

"00074dbf6d"	"00075804bb"	0.5083
"00074dbf6d"	"00075a0c04"	−0.2933
"00074dbf6d"	"00075a3110"	0.138
"00074dbf6d"	"00075a341a"	−0.246
"00074dbf6d"	"00075a3dcf"	−0.4884
"00074dbf6d"	"00075a3e22"	0.0209
"00074dbf6d"	"00075a48d8"	−0.1521
"00074dbf6d"	"00075a5cfb"	0.2056
"00074dbf6d"	"00075a6151"	0.1073
"00074dbf6d"	"00075a6708"	−0.0846
"00074dbf6d"	"00075a7319"	−0.0875
"00074dbf6d"	"00075a7723"	−0.1488
"00074dbf6d"	"00075a778b"	−0.2068
"00074dbf6d"	"00075a7b8e"	−0.0664
"00074dbf6d"	"00075a7c79"	0.1074
"00074dbf6d"	"00075a81b6"	−0.0725
"00074dbf6d"	"00075a82ac"	−0.4097
"00074dbf6d"	"00075a98e5"	−0.1775
"00074dbf6d"	"00075b0d29"	−0.0397
"00074dbf6d"	"00075b102a"	0.2413
"00074dbf6d"	"00075b1074"	−0.2244
"00074dbf6d"	"00075b135d"	−0.3421
"00074dbf6d"	"00075b138b"	0.1427
"00074dbf6d"	"00075b13a0"	−0.3966
"00074dbf6d"	"00075b13bd"	−0.2728
"00074dbf6d"	"00075b16a9"	−0.1169
"00074dbf6d"	"00075b1a28"	−0.0879
"00074dbf6d"	"00075b1a97"	−0.2368
"00074dbf6d"	"00075b1c7b"	−0.426
"00074dbf6d"	"00075b1d24"	−0.1442
"00074dbf6d"	"00075b202b"	0.1052
"00074dbf6d"	"00075b22cb"	−0.2075
"00074dbf6d"	"00075b22da"	−0.141
"00074dbf6d"	"00075b2556"	−0.056
"00074dbf6d"	"00075b25de"	−0.0283
"00074dbf6d"	"00075b260c"	0.3688
"00074dbf6d"	"00075b26f1"	−0.2753
"00074dbf6d"	"00075b2920"	0.4326
"00074dbf6d"	"00075b2a64"	0.3724
"00074dbf6d"	"00075b2a9d"	0.1792
"00074dbf6d"	"00075b2b37"	−0.244
"00074dbf6d"	"00075b2cdd"	−0.3882
"00074dbf6d"	"00075b3038"	0.0248
"00074dbf6d"	"00075b30fe"	−0.1028
"00074dbf6d"	"00075b3362"	−0.5561
"00074dbf6d"	"00075b350a"	0.2147
"00074dbf6d"	"00075b350e"	−0.3656
"00074dbf6d"	"00075b3651"	−0.2784
"00074dbf6d"	"00075b38ca"	−0.5786
"00074dbf6d"	"00075b39cc"	−0.1693

"00074dbf6d"	"00075b3e1e"	−0.0683
"00074dbf6d"	"00075b3e57"	−0.0257
"00074dbf6d"	"00075b4079"	0.1258
"00074dbf6d"	"00075b4150"	0.1409
"00074dbf6d"	"00075b4194"	0.0222
"00074dbf6d"	"00075b42d5"	−0.2328
"00074dbf6d"	"00075b4424"	−0.2228
"00074dbf6d"	"00075b4470"	0.0872
"00074dbf6d"	"00075b47ed"	−0.4485
"00074dbf6d"	"00075b4850"	0.2314
"00074dbf6d"	"00075b4ca0"	−0.0864
"00074dbf6d"	"00075b4d7f"	−0.1032
"00074dbf6d"	"00075b520f"	−0.1759
"00074dbf6d"	"00075b525f"	−0.1599
"00074dbf6d"	"00075b58f8"	−0.056
"00074dbf6d"	"00075b5bcc"	0.0964
"00074dbf6d"	"00075b5bfa"	−0.3639
"00074dbf6d"	"00075b6339"	0.2209
"00074dbf6d"	"00075b6658"	−0.5302
"00074dbf6d"	"00075b679a"	0.0375
"00074dbf6d"	"00075b6cb7"	0.0813
"00074dbf6d"	"00075b6df8"	−0.4356
"00074dbf6d"	"00075b6ff6"	0.3078
"00074dbf6d"	"00075b70ee"	0.0096
"00074dbf6d"	"00075b7157"	−0.2369
"00074dbf6d"	"00075b7225"	−0.324
"00074dbf6d"	"00075b7c89"	−0.1293
"00074dbf6d"	"00075b9048"	0.0744
"00074dbf6d"	"00075d0801"	−0.0969
"00074dbf6d"	"00075d1820"	−0.4731
"00074dbf6d"	"00075d1f3d"	0.1105
"00074dbf6d"	"00075d2329"	−0.4426
"00074dbf6d"	"00075d2b9b"	0.181
"00074dbf6d"	"00075d3941"	−0.2715
"00074dbf6d"	"00075d3e96"	−0.2643
"00074dbf6d"	"00075d4864"	−0.3077
"00074dbf6d"	"00075d5961"	−0.3417
"00074dbf6d"	"00075d5a63"	0.1409
"00074dbf6d"	"00075d6150"	−0.3598
"00074dbf6d"	"00075d67d0"	−0.1041
"00074dbf6d"	"00075d67e2"	0.0152
"00074dbf6d"	"00075d73fc"	−0.5515
"00074dbf6d"	"00075d7729"	−0.3926
"00074dbf6d"	"00075d778c"	−0.4917
"00074dbf6d"	"00075d7b9e"	−0.1457
"00074dbf6d"	"00075d7c8f"	−0.0143
"00074dbf6d"	"00075d804d"	0.1787
"00074dbf6d"	"00075d819f"	0.1157
"00074dbf6d"	"00075d8601"	−0.115
"00074dbf6d"	"00075d8c6a"	−0.3089

"00074dbf6d"	"00075dfedc"	0.1355
"00074dbf6d"	"00075e05f2"	0.2001
"00074dbf6d"	"00075e0837"	0.206
"00074dbf6d"	"00075e092e"	−0.239
"00074dbf6d"	"00075e0965"	−0.0641
"00074dbf6d"	"00075e0bc8"	0.0377
"00074dbf6d"	"00075e0fbb"	−0.3003
"00074dc4a5"	"00074dc50c"	−0.4496
"00074dc4a5"	"00074dcdfa"	−0.0658
"00074dc4a5"	"00074dcf5f"	−0.3245
"00074dc4a5"	"00074dd007"	−0.5331
"00074dc4a5"	"00074dd163"	−0.2422
"00074dc4a5"	"00074dd3df"	0.1678
"00074dc4a5"	"00074dd577"	−0.3835
"00074dc4a5"	"00074dd62e"	−0.0836
"00074dc4a5"	"00074dd73c"	−0.0677
"00074dc4a5"	"00074dda10"	−0.0762
"00074dc4a5"	"00074ddab8"	−0.3434
"00074dc4a5"	"00074ddd3d"	−0.3691
"00074dc4a5"	"00074ddf16"	−0.1845
"00074dc4a5"	"00074ddfc1"	−0.3006
"00074dc4a5"	"00074de21a"	−0.1083
"00074dc4a5"	"00074de2a9"	−0.2543
"00074dc4a5"	"00074de544"	−0.1134
"00074dc4a5"	"00074de98a"	−0.0064
"00074dc4a5"	"00074dea7e"	−0.1524
"00074dc4a5"	"00074debd9"	−0.5676
"00074dc4a5"	"00074deca3"	−0.1766
"00074dc4a5"	"00074def43"	0.0603
"00074dc4a5"	"00074def99"	−0.1059
"00074dc4a5"	"00074ecdad"	−0.2064
"00074dc4a5"	"00074ecf28"	0.2123
"00074dc4a5"	"00074ed1e1"	−0.1941
"00074dc4a5"	"00074ed83b"	−0.0586
"00074dc4a5"	"00074ee5e3"	−0.5257
"00074dc4a5"	"00074ee6e0"	−0.3135
"00074dc4a5"	"00074eea3a"	0.0781
"00074dc4a5"	"00074eff82"	−0.3314
"00074dc4a5"	"00074f0477"	−0.4081
"00074dc4a5"	"00074f08c3"	−0.2867
"00074dc4a5"	"00074f1859"	−0.1419
"00074dc4a5"	"00074f2268"	−0.3139
"00074dc4a5"	"00074f28be"	−0.0649
"00074dc4a5"	"00074f294b"	0.0832
"00074dc4a5"	"00074f2ddd"	−0.0517
"00074dc4a5"	"00074f2e75"	−0.039
"00074dc4a5"	"00074f3088"	−0.1686
"00074dc4a5"	"00074f5a1c"	−0.0523
"00074dc4a5"	"00074f75b7"	−0.3613
"00074dc4a5"	"00074f8cd9"	−0.1606

"00074dc4a5"	"00074f96dc"	0.3023
"00074dc4a5"	"00074fabaa"	−0.2924
"00074dc4a5"	"00074facd9"	−0.3325
"00074dc4a5"	"00074fae3c"	0.1982
"00074dc4a5"	"00074fb0a8"	−0.3521
"00074dc4a5"	"00074fb4e4"	−0.3286
"00074dc4a5"	"00074fb7c2"	0.0187
"00074dc4a5"	"00074fbd36"	−0.1593
"00074dc4a5"	"00074fc27f"	−0.3467
"00074dc4a5"	"00074fc31d"	−0.2652
"00074dc4a5"	"00074fd569"	−0.0059
"00074dc4a5"	"00074fef15"	0.0026
"00074dc4a5"	"00074ff562"	−0.472
"00074dc4a5"	"00075007ca"	−0.4841
"00074dc4a5"	"0007500b86"	−0.3827
"00074dc4a5"	"0007500d05"	−0.2957
"00074dc4a5"	"0007500ee4"	−0.196
"00074dc4a5"	"0007500eee"	−0.4923
"00074dc4a5"	"00075013dc"	−0.6184
"00074dc4a5"	"000757b515"	−0.421
"00074dc4a5"	"000757bc5a"	−0.3425
"00074dc4a5"	"000757c320"	−0.2523
"00074dc4a5"	"000757c9aa"	−0.2628
"00074dc4a5"	"000757ccbe"	−0.0233
"00074dc4a5"	"000757cfa9"	−0.3075
"00074dc4a5"	"000757d390"	−0.4196
"00074dc4a5"	"000757d393"	−0.0771
"00074dc4a5"	"000757d598"	−0.5544
"00074dc4a5"	"000757d5a2"	−0.3709
"00074dc4a5"	"000757d790"	−0.3221
"00074dc4a5"	"000757e30c"	−0.6088
"00074dc4a5"	"000757e4b0"	−0.321
"00074dc4a5"	"000757e7a0"	−0.2444
"00074dc4a5"	"000757e8b3"	−0.2487
"00074dc4a5"	"000757f627"	0.0273
"00074dc4a5"	"000757f925"	−0.0613
"00074dc4a5"	"000757fa08"	0.0654
"00074dc4a5"	"000757fe52"	−0.2012
"00074dc4a5"	"000758024a"	−0.2321
"00074dc4a5"	"00075804bb"	−0.639
"00074dc4a5"	"00075a0c04"	0.1429
"00074dc4a5"	"00075a3110"	−0.1447
"00074dc4a5"	"00075a341a"	0.149
"00074dc4a5"	"00075a3dcf"	0.0176
"00074dc4a5"	"00075a3e22"	−0.0174
"00074dc4a5"	"00075a48d8"	0.1436
"00074dc4a5"	"00075a5cfb"	−0.4031
"00074dc4a5"	"00075a6151"	−0.5329
"00074dc4a5"	"00075a6708"	−0.2226
"00074dc4a5"	"00075a7319"	−0.4041

"00074dc4a5"	"00075a7723"	0.0319
"00074dc4a5"	"00075a778b"	0.074
"00074dc4a5"	"00075a7b8e"	-0.1183
"00074dc4a5"	"00075a7c79"	-0.4229
"00074dc4a5"	"00075a81b6"	-0.3392
"00074dc4a5"	"00075a82ac"	-0.0355
"00074dc4a5"	"00075a98e5"	-0.0101
"00074dc4a5"	"00075b0d29"	-0.0178
"00074dc4a5"	"00075b102a"	-0.364
"00074dc4a5"	"00075b1074"	0.056
"00074dc4a5"	"00075b135d"	-0.1307
"00074dc4a5"	"00075b138b"	-0.1219
"00074dc4a5"	"00075b13a0"	-0.1129
"00074dc4a5"	"00075b13bd"	0.4151
"00074dc4a5"	"00075b16a9"	-0.0581
"00074dc4a5"	"00075b1a28"	0.0581
"00074dc4a5"	"00075b1a97"	0.2468
"00074dc4a5"	"00075b1c7b"	-0.3627
"00074dc4a5"	"00075b1d24"	-0.0294
"00074dc4a5"	"00075b202b"	-0.2728
"00074dc4a5"	"00075b22cb"	0.236
"00074dc4a5"	"00075b22da"	-0.0454
"00074dc4a5"	"00075b2556"	-0.1761
"00074dc4a5"	"00075b25de"	-0.3951
"00074dc4a5"	"00075b260c"	-0.399
"00074dc4a5"	"00075b26f1"	0.0848
"00074dc4a5"	"00075b2920"	-0.5605
"00074dc4a5"	"00075b2a64"	-0.5187
"00074dc4a5"	"00075b2a9d"	-0.5117
"00074dc4a5"	"00075b2b37"	-0.3047
"00074dc4a5"	"00075b2cdd"	-0.0233
"00074dc4a5"	"00075b3038"	-0.3552
"00074dc4a5"	"00075b30fe"	-0.2894
"00074dc4a5"	"00075b3362"	0.0363
"00074dc4a5"	"00075b350a"	-0.2683
"00074dc4a5"	"00075b350e"	-0.1999
"00074dc4a5"	"00075b3651"	-0.0356
"00074dc4a5"	"00075b38ca"	-0.157
"00074dc4a5"	"00075b39cc"	-0.1585
"00074dc4a5"	"00075b3e1e"	-0.11
"00074dc4a5"	"00075b3e57"	0.2907
"00074dc4a5"	"00075b4079"	-0.3215
"00074dc4a5"	"00075b4150"	-0.2764
"00074dc4a5"	"00075b4194"	-0.1097
"00074dc4a5"	"00075b42d5"	-0.1706
"00074dc4a5"	"00075b4424"	-0.1431
"00074dc4a5"	"00075b4470"	-0.3425
"00074dc4a5"	"00075b47ed"	-0.0419
"00074dc4a5"	"00075b4850"	-0.438
"00074dc4a5"	"00075b4ca0"	-0.285

"00074dc4a5"	"00075b4d7f"	0.1359
"00074dc4a5"	"00075b520f"	-0.0854
"00074dc4a5"	"00075b525f"	-0.1619
"00074dc4a5"	"00075b58f8"	-0.5721
"00074dc4a5"	"00075b5bcc"	-0.1207
"00074dc4a5"	"00075b5bfa"	-0.5817
"00074dc4a5"	"00075b6339"	-0.0569
"00074dc4a5"	"00075b6658"	0.1404
"00074dc4a5"	"00075b679a"	8e-04
"00074dc4a5"	"00075b6cb7"	-0.6046
"00074dc4a5"	"00075b6df8"	0.128
"00074dc4a5"	"00075b6ff6"	-0.3225
"00074dc4a5"	"00075b70ee"	-0.3649
"00074dc4a5"	"00075b7157"	-0.2381
"00074dc4a5"	"00075b7225"	-0.1082
"00074dc4a5"	"00075b7c89"	-0.1287
"00074dc4a5"	"00075b9048"	-0.2977
"00074dc4a5"	"00075d0801"	-0.3684
"00074dc4a5"	"00075d1820"	0.1212
"00074dc4a5"	"00075d1f3d"	-0.4015
"00074dc4a5"	"00075d2329"	-0.1717
"00074dc4a5"	"00075d2b9b"	-0.5
"00074dc4a5"	"00075d3941"	-0.1462
"00074dc4a5"	"00075d3e96"	-0.2208
"00074dc4a5"	"00075d4864"	-0.1334
"00074dc4a5"	"00075d5961"	0.248
"00074dc4a5"	"00075d5a63"	-0.0946
"00074dc4a5"	"00075d6150"	-0.0482
"00074dc4a5"	"00075d67d0"	-0.2772
"00074dc4a5"	"00075d67e2"	-0.1295
"00074dc4a5"	"00075d73fc"	-0.0042
"00074dc4a5"	"00075d7729"	-0.0521
"00074dc4a5"	"00075d778c"	-0.2186
"00074dc4a5"	"00075d7b9e"	-0.1994
"00074dc4a5"	"00075d7c8f"	-0.0532
"00074dc4a5"	"00075d804d"	-0.4079
"00074dc4a5"	"00075d819f"	-0.5425
"00074dc4a5"	"00075d8601"	0.1358
"00074dc4a5"	"00075d8c6a"	0.0044
"00074dc4a5"	"00075dfedc"	-0.1309
"00074dc4a5"	"00075e05f2"	-0.4984
"00074dc4a5"	"00075e0837"	-0.5228
"00074dc4a5"	"00075e092e"	-0.0026
"00074dc4a5"	"00075e0965"	-0.4687
"00074dc4a5"	"00075e0bc8"	-0.2284
"00074dc4a5"	"00075e0fbb"	-0.2555
"00074dc50c"	"00074dcdfa"	0.2696
"00074dc50c"	"00074dcf5f"	-0.2093
"00074dc50c"	"00074dd007"	0.0505
"00074dc50c"	"00074dd163"	-0.0593

"00074dc50c"	"00074dd3df"	−0.4462
"00074dc50c"	"00074dd577"	0.3677
"00074dc50c"	"00074dd62e"	−0.2117
"00074dc50c"	"00074dd73c"	0.2634
"00074dc50c"	"00074dda10"	0.0163
"00074dc50c"	"00074ddab8"	0.4838
"00074dc50c"	"00074ddd3d"	−0.2531
"00074dc50c"	"00074ddf16"	0.2245
"00074dc50c"	"00074ddfc1"	0.3506
"00074dc50c"	"00074de21a"	−0.0789
"00074dc50c"	"00074de2a9"	0.2725
"00074dc50c"	"00074de544"	0.033
"00074dc50c"	"00074de98a"	0.0834
"00074dc50c"	"00074dea7e"	−0.0711
"00074dc50c"	"00074debd9"	0.1379
"00074dc50c"	"00074deca3"	−0.0445
"00074dc50c"	"00074def43"	0.1843
"00074dc50c"	"00074def99"	−0.1369
"00074dc50c"	"00074ecdad"	0.0225
"00074dc50c"	"00074ecf28"	−0.3271
"00074dc50c"	"00074ed1e1"	0.1064
"00074dc50c"	"00074ed83b"	0.1749
"00074dc50c"	"00074ee5e3"	−0.1291
"00074dc50c"	"00074ee6e0"	−0.3487
"00074dc50c"	"00074eea3a"	0.0504
"00074dc50c"	"00074eff82"	−0.1806
"00074dc50c"	"00074f0477"	−0.1621
"00074dc50c"	"00074f08c3"	0.113
"00074dc50c"	"00074f1859"	0.1658
"00074dc50c"	"00074f2268"	−0.2076
"00074dc50c"	"00074f28be"	0.2995
"00074dc50c"	"00074f294b"	−0.1634
"00074dc50c"	"00074f2ddd"	−0.0698
"00074dc50c"	"00074f2e75"	−0.1153
"00074dc50c"	"00074f3088"	−0.2482
"00074dc50c"	"00074f5a1c"	0.1818
"00074dc50c"	"00074f75b7"	0.0553
"00074dc50c"	"00074f8cd9"	0.0481
"00074dc50c"	"00074f96dc"	−0.2665
"00074dc50c"	"00074fabaa"	0.2835
"00074dc50c"	"00074facd9"	0.0418
"00074dc50c"	"00074fae3c"	−0.2838
"00074dc50c"	"00074fb0a8"	−0.0612
"00074dc50c"	"00074fb4e4"	−0.3045
"00074dc50c"	"00074fb7c2"	0.2358
"00074dc50c"	"00074fbd36"	−0.2521
"00074dc50c"	"00074fc27f"	0.1757
"00074dc50c"	"00074fc31d"	−0.1929
"00074dc50c"	"00074fd569"	−0.0829
"00074dc50c"	"00074fef15"	−0.2967

"00074dc50c"	"00074ff562"	-0.0943
"00074dc50c"	"00075007ca"	0.2072
"00074dc50c"	"0007500b86"	0.1027
"00074dc50c"	"0007500d05"	-0.1804
"00074dc50c"	"0007500ee4"	-0.3612
"00074dc50c"	"0007500eee"	-0.1155
"00074dc50c"	"00075013dc"	0.1426
"00074dc50c"	"000757b515"	0.1827
"00074dc50c"	"000757bc5a"	0.1808
"00074dc50c"	"000757c320"	-0.1809
"00074dc50c"	"000757c9aa"	0.1816
"00074dc50c"	"000757ccbe"	-0.0762
"00074dc50c"	"000757cfa9"	0.0162
"00074dc50c"	"000757d390"	-0.2338
"00074dc50c"	"000757d393"	0.0464
"00074dc50c"	"000757d598"	0.0121
"00074dc50c"	"000757d5a2"	0.0742
"00074dc50c"	"000757d790"	-0.1185
"00074dc50c"	"000757e30c"	0.0207
"00074dc50c"	"000757e4b0"	0.5368
"00074dc50c"	"000757e7a0"	-0.2073
"00074dc50c"	"000757e8b3"	0.1882
"00074dc50c"	"000757f627"	0.0779
"00074dc50c"	"000757f925"	-0.0907
"00074dc50c"	"000757fa08"	-0.0803
"00074dc50c"	"000757fe52"	-0.0963
"00074dc50c"	"000758024a"	0.1522
"00074dc50c"	"00075804bb"	0.1777
"00074dc50c"	"00075a0c04"	0.2211
"00074dc50c"	"00075a3110"	0.2155
"00074dc50c"	"00075a341a"	-0.3162
"00074dc50c"	"00075a3dcf"	0.0145
"00074dc50c"	"00075a3e22"	0.1454
"00074dc50c"	"00075a48d8"	-0.1129
"00074dc50c"	"00075a5cfb"	0.085
"00074dc50c"	"00075a6151"	0.0095
"00074dc50c"	"00075a6708"	0.0184
"00074dc50c"	"00075a7319"	-0.2659
"00074dc50c"	"00075a7723"	-0.0608
"00074dc50c"	"00075a778b"	-0.1293
"00074dc50c"	"00075a7b8e"	0.1322
"00074dc50c"	"00075a7c79"	0.3294
"00074dc50c"	"00075a81b6"	-0.005
"00074dc50c"	"00075a82ac"	-0.0394
"00074dc50c"	"00075a98e5"	-0.2266
"00074dc50c"	"00075b0d29"	-0.0152
"00074dc50c"	"00075b102a"	0.2603
"00074dc50c"	"00075b1074"	-0.1206
"00074dc50c"	"00075b135d"	0.0191
"00074dc50c"	"00075b138b"	0.5487

"00074dc50c"	"00075b13a0"	-0.3851
"00074dc50c"	"00075b13bd"	-0.3061
"00074dc50c"	"00075b16a9"	-0.2821
"00074dc50c"	"00075b1a28"	-0.4141
"00074dc50c"	"00075b1a97"	-0.1125
"00074dc50c"	"00075b1c7b"	-0.4723
"00074dc50c"	"00075b1d24"	-0.2123
"00074dc50c"	"00075b202b"	0.4366
"00074dc50c"	"00075b22cb"	-0.2451
"00074dc50c"	"00075b22da"	-0.0017
"00074dc50c"	"00075b2556"	-0.268
"00074dc50c"	"00075b25de"	-0.0064
"00074dc50c"	"00075b260c"	0.3857
"00074dc50c"	"00075b26f1"	-0.1105
"00074dc50c"	"00075b2920"	0.1027
"00074dc50c"	"00075b2a64"	0.1767
"00074dc50c"	"00075b2a9d"	0.3628
"00074dc50c"	"00075b2b37"	-0.611
"00074dc50c"	"00075b2cdd"	-0.0728
"00074dc50c"	"00075b3038"	0.292
"00074dc50c"	"00075b30fe"	-0.0432
"00074dc50c"	"00075b3362"	-0.5341
"00074dc50c"	"00075b350a"	0.0439
"00074dc50c"	"00075b350e"	0.217
"00074dc50c"	"00075b3651"	-0.3207
"00074dc50c"	"00075b38ca"	0.0371
"00074dc50c"	"00075b39cc"	-0.0803
"00074dc50c"	"00075b3e1e"	0.268
"00074dc50c"	"00075b3e57"	-0.3313
"00074dc50c"	"00075b4079"	-0.1257
"00074dc50c"	"00075b4150"	-0.0155
"00074dc50c"	"00075b4194"	0.3387
"00074dc50c"	"00075b42d5"	0.2508
"00074dc50c"	"00075b4424"	-0.1678
"00074dc50c"	"00075b4470"	0.2502
"00074dc50c"	"00075b47ed"	-0.0447
"00074dc50c"	"00075b4850"	0.4008
"00074dc50c"	"00075b4ca0"	0.2906
"00074dc50c"	"00075b4d7f"	0.0651
"00074dc50c"	"00075b520f"	-0.1261
"00074dc50c"	"00075b525f"	-0.2139
"00074dc50c"	"00075b58f8"	-0.1567
"00074dc50c"	"00075b5bcc"	0.1111
"00074dc50c"	"00075b5bfa"	-0.2596
"00074dc50c"	"00075b6339"	0.216
"00074dc50c"	"00075b6658"	-0.4502
"00074dc50c"	"00075b679a"	0.0044
"00074dc50c"	"00075b6cb7"	0.1558
"00074dc50c"	"00075b6df8"	-0.1346
"00074dc50c"	"00075b6ff6"	0.1937

"00074dc50c"	"00075b70ee"	0.0341
"00074dc50c"	"00075b7157"	-0.2495
"00074dc50c"	"00075b7225"	-0.0361
"00074dc50c"	"00075b7c89"	0.1479
"00074dc50c"	"00075b9048"	0.3717
"00074dc50c"	"00075d0801"	-0.0062
"00074dc50c"	"00075d1820"	-0.443
"00074dc50c"	"00075d1f3d"	0.6012
"00074dc50c"	"00075d2329"	0.0839
"00074dc50c"	"00075d2b9b"	0.6131
"00074dc50c"	"00075d3941"	-0.0702
"00074dc50c"	"00075d3e96"	-0.2406
"00074dc50c"	"00075d4864"	-0.1718
"00074dc50c"	"00075d5961"	-0.0519
"00074dc50c"	"00075d5a63"	0.3599
"00074dc50c"	"00075d6150"	-0.2093
"00074dc50c"	"00075d67d0"	0.0791
"00074dc50c"	"00075d67e2"	0.0232
"00074dc50c"	"00075d73fc"	-0.0252
"00074dc50c"	"00075d7729"	-0.0589
"00074dc50c"	"00075d778c"	-0.4287
"00074dc50c"	"00075d7b9e"	-0.2472
"00074dc50c"	"00075d7c8f"	0.1886
"00074dc50c"	"00075d804d"	0.2335
"00074dc50c"	"00075d819f"	0.2634
"00074dc50c"	"00075d8601"	-0.1617
"00074dc50c"	"00075d8c6a"	-0.0663
"00074dc50c"	"00075dfedc"	0.0869
"00074dc50c"	"00075e05f2"	0.0879
"00074dc50c"	"00075e0837"	0.4863
"00074dc50c"	"00075e092e"	-0.0565
"00074dc50c"	"00075e0965"	-0.1178
"00074dc50c"	"00075e0bc8"	0.4574
"00074dc50c"	"00075e0fbb"	-0.0704
"00074dcdfa"	"00074dcf5f"	-0.5309
"00074dcdfa"	"00074dd007"	-0.1324
"00074dcdfa"	"00074dd163"	0.108
"00074dcdfa"	"00074dd3df"	-0.4202
"00074dcdfa"	"00074dd577"	-0.0188
"00074dcdfa"	"00074dd62e"	-0.0338
"00074dcdfa"	"00074dd73c"	0.2106
"00074dcdfa"	"00074dda10"	0.196
"00074dcdfa"	"00074ddab8"	0.2627
"00074dcdfa"	"00074ddd3d"	-0.4282
"00074dcdfa"	"00074ddf16"	0.2533
"00074dcdfa"	"00074ddfc1"	0.193
"00074dcdfa"	"00074de21a"	0.0609
"00074dcdfa"	"00074de2a9"	0.0118
"00074dcdfa"	"00074de544"	0.067
"00074dcdfa"	"00074de98a"	-0.1064

"00074dcdfa"	"00074dea7e"	-0.2299
"00074dcdfa"	"00074debd9"	-0.1146
"00074dcdfa"	"00074deca3"	0.0661
"00074dcdfa"	"00074def43"	0.1251
"00074dcdfa"	"00074def99"	-0.3022
"00074dcdfa"	"00074ecdad"	-0.3056
"00074dcdfa"	"00074ecf28"	-0.3917
"00074dcdfa"	"00074ed1e1"	0.0138
"00074dcdfa"	"00074ed83b"	0.0324
"00074dcdfa"	"00074ee5e3"	-0.0212
"00074dcdfa"	"00074ee6e0"	-0.0517
"00074dcdfa"	"00074eea3a"	0.0332
"00074dcdfa"	"00074eff82"	0.0304
"00074dcdfa"	"00074f0477"	-0.3687
"00074dcdfa"	"00074f08c3"	0.0752
"00074dcdfa"	"00074f1859"	-0.0862
"00074dcdfa"	"00074f2268"	-0.3491
"00074dcdfa"	"00074f28be"	0.5607
"00074dcdfa"	"00074f294b"	-0.1105
"00074dcdfa"	"00074f2ddd"	-0.0672
"00074dcdfa"	"00074f2e75"	-0.4677
"00074dcdfa"	"00074f3088"	-0.0471
"00074dcdfa"	"00074f5a1c"	-0.0054
"00074dcdfa"	"00074f75b7"	0.0789
"00074dcdfa"	"00074f8cd9"	0.152
"00074dcdfa"	"00074f96dc"	-0.0438
"00074dcdfa"	"00074fabaa"	0.2002
"00074dcdfa"	"00074facd9"	-0.003
"00074dcdfa"	"00074fae3c"	-0.1036
"00074dcdfa"	"00074fb0a8"	0.2191
"00074dcdfa"	"00074fb4e4"	-0.0879
"00074dcdfa"	"00074fb7c2"	-0.0834
"00074dcdfa"	"00074fbd36"	-0.3899
"00074dcdfa"	"00074fc27f"	-0.1176
"00074dcdfa"	"00074fc31d"	-0.3341
"00074dcdfa"	"00074fd569"	-0.2334
"00074dcdfa"	"00074fef15"	-0.2501
"00074dcdfa"	"00074ff562"	-0.2288
"00074dcdfa"	"00075007ca"	-0.1103
"00074dcdfa"	"0007500b86"	0.3339
"00074dcdfa"	"0007500d05"	-0.0045
"00074dcdfa"	"0007500ee4"	-0.1039
"00074dcdfa"	"0007500eee"	-0.2717
"00074dcdfa"	"00075013dc"	0.2644
"00074dcdfa"	"000757b515"	-0.0022
"00074dcdfa"	"000757bc5a"	0.142
"00074dcdfa"	"000757c320"	-0.1193
"00074dcdfa"	"000757c9aa"	0.1914
"00074dcdfa"	"000757ccbe"	0.0578
"00074dcdfa"	"000757cfa9"	-0.2302

"00074dcdfa"	"000757d390"	-0.1218
"00074dcdfa"	"000757d393"	-0.0343
"00074dcdfa"	"000757d598"	-0.2211
"00074dcdfa"	"000757d5a2"	-0.1607
"00074dcdfa"	"000757d790"	-0.1438
"00074dcdfa"	"000757e30c"	-0.2217
"00074dcdfa"	"000757e4b0"	0.1509
"00074dcdfa"	"000757e7a0"	-0.1509
"00074dcdfa"	"000757e8b3"	0.1132
"00074dcdfa"	"000757f627"	0.3472
"00074dcdfa"	"000757f925"	0.0467
"00074dcdfa"	"000757fa08"	-0.2598
"00074dcdfa"	"000757fe52"	-0.0495
"00074dcdfa"	"000758024a"	-0.2214
"00074dcdfa"	"00075804bb"	-0.0567
"00074dcdfa"	"00075a0c04"	0.0641
"00074dcdfa"	"00075a3110"	0.0714
"00074dcdfa"	"00075a341a"	-0.4544
"00074dcdfa"	"00075a3dcf"	0.124
"00074dcdfa"	"00075a3e22"	0.1177
"00074dcdfa"	"00075a48d8"	0.0308
"00074dcdfa"	"00075a5cfb"	-0.0524
"00074dcdfa"	"00075a6151"	-0.0594
"00074dcdfa"	"00075a6708"	-0.1371
"00074dcdfa"	"00075a7319"	0.0122
"00074dcdfa"	"00075a7723"	-0.0778
"00074dcdfa"	"00075a778b"	-0.1952
"00074dcdfa"	"00075a7b8e"	0.2687
"00074dcdfa"	"00075a7c79"	0.197
"00074dcdfa"	"00075a81b6"	0.0101
"00074dcdfa"	"00075a82ac"	-0.0699
"00074dcdfa"	"00075a98e5"	-0.0081
"00074dcdfa"	"00075b0d29"	-0.2002
"00074dcdfa"	"00075b102a"	-0.2025
"00074dcdfa"	"00075b1074"	-0.2615
"00074dcdfa"	"00075b135d"	-0.0771
"00074dcdfa"	"00075b138b"	0.3034
"00074dcdfa"	"00075b13a0"	-0.2336
"00074dcdfa"	"00075b13bd"	-0.0796
"00074dcdfa"	"00075b16a9"	-0.2641
"00074dcdfa"	"00075b1a28"	-0.5172
"00074dcdfa"	"00075b1a97"	0.0461
"00074dcdfa"	"00075b1c7b"	-0.683
"00074dcdfa"	"00075b1d24"	-0.0472
"00074dcdfa"	"00075b202b"	0.1536
"00074dcdfa"	"00075b22cb"	-0.3305
"00074dcdfa"	"00075b22da"	-0.2759
"00074dcdfa"	"00075b2556"	-0.5367
"00074dcdfa"	"00075b25de"	0.0808
"00074dcdfa"	"00075b260c"	0.0429

"00074dcdfa"	"00075b26f1"	-0.0863
"00074dcdfa"	"00075b2920"	-0.0509
"00074dcdfa"	"00075b2a64"	0.0329
"00074dcdfa"	"00075b2a9d"	0.143
"00074dcdfa"	"00075b2b37"	-0.551
"00074dcdfa"	"00075b2cdd"	-0.0981
"00074dcdfa"	"00075b3038"	0.1094
"00074dcdfa"	"00075b30fe"	-0.3046
"00074dcdfa"	"00075b3362"	-0.2246
"00074dcdfa"	"00075b350a"	0.1814
"00074dcdfa"	"00075b350e"	0.0664
"00074dcdfa"	"00075b3651"	-0.1574
"00074dcdfa"	"00075b38ca"	-0.0248
"00074dcdfa"	"00075b39cc"	0.2485
"00074dcdfa"	"00075b3e1e"	0.0272
"00074dcdfa"	"00075b3e57"	-0.1628
"00074dcdfa"	"00075b4079"	0.1267
"00074dcdfa"	"00075b4150"	-0.3036
"00074dcdfa"	"00075b4194"	0.7394
"00074dcdfa"	"00075b42d5"	0.099
"00074dcdfa"	"00075b4424"	-0.3767
"00074dcdfa"	"00075b4470"	0.1264
"00074dcdfa"	"00075b47ed"	0.3244
"00074dcdfa"	"00075b4850"	0.1936
"00074dcdfa"	"00075b4ca0"	6e-04
"00074dcdfa"	"00075b4d7f"	0.0443
"00074dcdfa"	"00075b520f"	-0.0797
"00074dcdfa"	"00075b525f"	-0.0546
"00074dcdfa"	"00075b58f8"	-0.3379
"00074dcdfa"	"00075b5bcc"	0.129
"00074dcdfa"	"00075b5bfa"	-0.2063
"00074dcdfa"	"00075b6339"	0.2133
"00074dcdfa"	"00075b6658"	-0.3522
"00074dcdfa"	"00075b679a"	0.0543
"00074dcdfa"	"00075b6cb7"	-0.1672
"00074dcdfa"	"00075b6df8"	-0.2936
"00074dcdfa"	"00075b6ff6"	0.1637
"00074dcdfa"	"00075b70ee"	-0.1047
"00074dcdfa"	"00075b7157"	-0.0588
"00074dcdfa"	"00075b7225"	-0.1153
"00074dcdfa"	"00075b7c89"	-0.0668
"00074dcdfa"	"00075b9048"	-0.0363
"00074dcdfa"	"00075d0801"	0.1185
"00074dcdfa"	"00075d1820"	-0.247
"00074dcdfa"	"00075d1f3d"	0.2999
"00074dcdfa"	"00075d2329"	0.1344
"00074dcdfa"	"00075d2b9b"	0.1131
"00074dcdfa"	"00075d3941"	-0.0714
"00074dcdfa"	"00075d3e96"	-0.2123
"00074dcdfa"	"00075d4864"	-0.2978

"00074dcdfa"	"00075d5961"	−0.0083
"00074dcdfa"	"00075d5a63"	0.2608
"00074dcdfa"	"00075d6150"	0.0787
"00074dcdfa"	"00075d67d0"	0.0562
"00074dcdfa"	"00075d67e2"	−0.0607
"00074dcdfa"	"00075d73fc"	−0.2753
"00074dcdfa"	"00075d7729"	−0.2998
"00074dcdfa"	"00075d778c"	−0.3021
"00074dcdfa"	"00075d7b9e"	−0.0803
"00074dcdfa"	"00075d7c8f"	0.1325
"00074dcdfa"	"00075d804d"	0.2758
"00074dcdfa"	"00075d819f"	−0.0163
"00074dcdfa"	"00075d8601"	0.0656
"00074dcdfa"	"00075d8c6a"	−0.09
"00074dcdfa"	"00075dfedc"	0.1078
"00074dcdfa"	"00075e05f2"	−0.0382
"00074dcdfa"	"00075e0837"	0.0477
"00074dcdfa"	"00075e092e"	−0.0656
"00074dcdfa"	"00075e0965"	−0.4105
"00074dcdfa"	"00075e0bc8"	0.1895
"00074dcdfa"	"00075e0fbb"	−0.2354
"00074dcf5f"	"00074dd007"	−0.0018
"00074dcf5f"	"00074dd163"	−0.644
"00074dcf5f"	"00074dd3df"	−0.5722
"00074dcf5f"	"00074dd577"	0.0118
"00074dcf5f"	"00074dd62e"	−0.1586
"00074dcf5f"	"00074dd73c"	−0.3793
"00074dcf5f"	"00074dda10"	−0.3324
"00074dcf5f"	"00074ddab8"	−0.4022
"00074dcf5f"	"00074ddd3d"	0.5938
"00074dcf5f"	"00074ddf16"	−0.3251
"00074dcf5f"	"00074ddfc1"	−0.4335
"00074dcf5f"	"00074de21a"	−0.4513
"00074dcf5f"	"00074de2a9"	−0.0755
"00074dcf5f"	"00074de544"	−0.2147
"00074dcf5f"	"00074de98a"	−0.3807
"00074dcf5f"	"00074dea7e"	−0.4196
"00074dcf5f"	"00074debd9"	−0.597
"00074dcf5f"	"00074deca3"	−0.4662
"00074dcf5f"	"00074def43"	−0.3995
"00074dcf5f"	"00074def99"	0.1319
"00074dcf5f"	"00074ecdad"	−0.244
"00074dcf5f"	"00074ecf28"	0.0512
"00074dcf5f"	"00074ed1e1"	−0.1781
"00074dcf5f"	"00074ed83b"	0.1298
"00074dcf5f"	"00074ee5e3"	−0.449
"00074dcf5f"	"00074ee6e0"	−0.1777
"00074dcf5f"	"00074eea3a"	−0.3879
"00074dcf5f"	"00074eff82"	−0.1362
"00074dcf5f"	"00074f0477"	−0.3998

"00074dcf5f"	"00074f08c3"	−0.3362
"00074dcf5f"	"00074f1859"	−0.4075
"00074dcf5f"	"00074f2268"	−0.3075
"00074dcf5f"	"00074f28be"	−0.1998
"00074dcf5f"	"00074f294b"	−0.106
"00074dcf5f"	"00074f2ddd"	−0.0088
"00074dcf5f"	"00074f2e75"	−0.1487
"00074dcf5f"	"00074f3088"	−0.1015
"00074dcf5f"	"00074f5a1c"	−0.3921
"00074dcf5f"	"00074f75b7"	−0.157
"00074dcf5f"	"00074f8cd9"	−0.1826
"00074dcf5f"	"00074f96dc"	−0.3597
"00074dcf5f"	"00074fabaa"	−0.4859
"00074dcf5f"	"00074facd9"	−0.3673
"00074dcf5f"	"00074fae3c"	−0.2449
"00074dcf5f"	"00074fb0a8"	−0.1828
"00074dcf5f"	"00074fb4e4"	−0.1562
"00074dcf5f"	"00074fb7c2"	−0.0065
"00074dcf5f"	"00074fbd36"	−0.1796
"00074dcf5f"	"00074fc27f"	0.0172
"00074dcf5f"	"00074fc31d"	−0.0308
"00074dcf5f"	"00074fd569"	0.0064
"00074dcf5f"	"00074fef15"	−0.2259
"00074dcf5f"	"00074ff562"	−0.4367
"00074dcf5f"	"00075007ca"	0.1668
"00074dcf5f"	"0007500b86"	−0.3999
"00074dcf5f"	"0007500d05"	−0.2167
"00074dcf5f"	"0007500ee4"	−0.0323
"00074dcf5f"	"0007500eee"	−0.4909
"00074dcf5f"	"00075013dc"	−0.2321
"00074dcf5f"	"000757b515"	0.203
"00074dcf5f"	"000757bc5a"	−0.3036
"00074dcf5f"	"000757c320"	−0.1923
"00074dcf5f"	"000757c9aa"	0.0596
"00074dcf5f"	"000757ccbe"	−0.2489
"00074dcf5f"	"000757cfa9"	−0.1183
"00074dcf5f"	"000757d390"	−0.0237
"00074dcf5f"	"000757d393"	−0.3039
"00074dcf5f"	"000757d598"	0.3248
"00074dcf5f"	"000757d5a2"	0.266
"00074dcf5f"	"000757d790"	−0.095
"00074dcf5f"	"000757e30c"	0.534
"00074dcf5f"	"000757e4b0"	−0.2379
"00074dcf5f"	"000757e7a0"	0.2154
"00074dcf5f"	"000757e8b3"	−0.2591
"00074dcf5f"	"000757f627"	−0.2052
"00074dcf5f"	"000757f925"	−0.0232
"00074dcf5f"	"000757fa08"	−0.1199
"00074dcf5f"	"000757fe52"	−0.3185
"00074dcf5f"	"000758024a"	0.2909

"00074dcf5f"	"00075804bb"	-0.1557
"00074dcf5f"	"00075a0c04"	-0.1831
"00074dcf5f"	"00075a3110"	0.1295
"00074dcf5f"	"00075a341a"	-0.0482
"00074dcf5f"	"00075a3dcf"	-0.4882
"00074dcf5f"	"00075a3e22"	-0.2022
"00074dcf5f"	"00075a48d8"	-0.2176
"00074dcf5f"	"00075a5cfb"	0.0148
"00074dcf5f"	"00075a6151"	-0.0581
"00074dcf5f"	"00075a6708"	0.1254
"00074dcf5f"	"00075a7319"	-0.0591
"00074dcf5f"	"00075a7723"	-0.1664
"00074dcf5f"	"00075a778b"	-0.0709
"00074dcf5f"	"00075a7b8e"	0.0318
"00074dcf5f"	"00075a7c79"	-0.1159
"00074dcf5f"	"00075a81b6"	-0.185
"00074dcf5f"	"00075a82ac"	-0.2816
"00074dcf5f"	"00075a98e5"	-0.4635
"00074dcf5f"	"00075b0d29"	-0.0177
"00074dcf5f"	"00075b102a"	-0.1089
"00074dcf5f"	"00075b1074"	-0.3031
"00074dcf5f"	"00075b135d"	-0.1096
"00074dcf5f"	"00075b138b"	-0.151
"00074dcf5f"	"00075b13a0"	-0.5003
"00074dcf5f"	"00075b13bd"	-0.2717
"00074dcf5f"	"00075b16a9"	0.1028
"00074dcf5f"	"00075b1a28"	-0.1614
"00074dcf5f"	"00075b1a97"	-0.1443
"00074dcf5f"	"00075b1c7b"	0.0252
"00074dcf5f"	"00075b1d24"	-0.0566
"00074dcf5f"	"00075b202b"	-0.0355
"00074dcf5f"	"00075b22cb"	-0.2272
"00074dcf5f"	"00075b22da"	-0.3386
"00074dcf5f"	"00075b2556"	-0.1748
"00074dcf5f"	"00075b25de"	-0.1906
"00074dcf5f"	"00075b260c"	0.1367
"00074dcf5f"	"00075b26f1"	-0.2868
"00074dcf5f"	"00075b2920"	0.1666
"00074dcf5f"	"00075b2a64"	-0.0877
"00074dcf5f"	"00075b2a9d"	-0.2214
"00074dcf5f"	"00075b2b37"	-0.1644
"00074dcf5f"	"00075b2cdd"	-0.1272
"00074dcf5f"	"00075b3038"	-0.0621
"00074dcf5f"	"00075b30fe"	-0.2262
"00074dcf5f"	"00075b3362"	-0.3421
"00074dcf5f"	"00075b350a"	-0.3843
"00074dcf5f"	"00075b350e"	-0.3453
"00074dcf5f"	"00075b3651"	-0.1505
"00074dcf5f"	"00075b38ca"	-0.6699
"00074dcf5f"	"00075b39cc"	-0.3742

"00074dcf5f"	"00075b3e1e"	-0.3954
"00074dcf5f"	"00075b3e57"	0.0612
"00074dcf5f"	"00075b4079"	-0.3331
"00074dcf5f"	"00075b4150"	0.3857
"00074dcf5f"	"00075b4194"	-0.2895
"00074dcf5f"	"00075b42d5"	-0.3276
"00074dcf5f"	"00075b4424"	0.0195
"00074dcf5f"	"00075b4470"	-0.3192
"00074dcf5f"	"00075b47ed"	-0.4681
"00074dcf5f"	"00075b4850"	-0.2405
"00074dcf5f"	"00075b4ca0"	-0.1433
"00074dcf5f"	"00075b4d7f"	0.0136
"00074dcf5f"	"00075b520f"	-0.191
"00074dcf5f"	"00075b525f"	-0.0579
"00074dcf5f"	"00075b58f8"	-0.0449
"00074dcf5f"	"00075b5bcc"	-0.2234
"00074dcf5f"	"00075b5bfa"	-0.249
"00074dcf5f"	"00075b6339"	0.0166
"00074dcf5f"	"00075b6658"	-0.5628
"00074dcf5f"	"00075b679a"	0.0994
"00074dcf5f"	"00075b6cb7"	-0.2716
"00074dcf5f"	"00075b6df8"	0.0149
"00074dcf5f"	"00075b6ff6"	-0.0372
"00074dcf5f"	"00075b70ee"	0.2637
"00074dcf5f"	"00075b7157"	-0.1362
"00074dcf5f"	"00075b7225"	-0.1356
"00074dcf5f"	"00075b7c89"	-0.1956
"00074dcf5f"	"00075b9048"	0.1674
"00074dcf5f"	"00075d0801"	-0.2048
"00074dcf5f"	"00075d1820"	0.107
"00074dcf5f"	"00075d1f3d"	-0.233
"00074dcf5f"	"00075d2329"	-0.4254
"00074dcf5f"	"00075d2b9b"	-0.3286
"00074dcf5f"	"00075d3941"	-0.5934
"00074dcf5f"	"00075d3e96"	0.076
"00074dcf5f"	"00075d4864"	-0.6132
"00074dcf5f"	"00075d5961"	-0.1718
"00074dcf5f"	"00075d5a63"	-0.1736
"00074dcf5f"	"00075d6150"	-0.3151
"00074dcf5f"	"00075d67d0"	-0.2035
"00074dcf5f"	"00075d67e2"	-0.2999
"00074dcf5f"	"00075d73fc"	-0.224
"00074dcf5f"	"00075d7729"	0.0534
"00074dcf5f"	"00075d778c"	-0.2544
"00074dcf5f"	"00075d7b9e"	-0.1138
"00074dcf5f"	"00075d7c8f"	-0.2033
"00074dcf5f"	"00075d804d"	-0.3836
"00074dcf5f"	"00075d819f"	-0.2904
"00074dcf5f"	"00075d8601"	-0.2271
"00074dcf5f"	"00075d8c6a"	-0.0735

"00074dcf5f"	"00075dfedc"	-0.165
"00074dcf5f"	"00075e05f2"	-0.2404
"00074dcf5f"	"00075e0837"	-0.089
"00074dcf5f"	"00075e092e"	-0.2805
"00074dcf5f"	"00075e0965"	-0.1015
"00074dcf5f"	"00075e0bc8"	-0.2168
"00074dcf5f"	"00075e0fbb"	-0.3616
"00074dd007"	"00074dd163"	-0.0759
"00074dd007"	"00074dd3df"	-0.4335
"00074dd007"	"00074dd577"	-0.0082
"00074dd007"	"00074dd62e"	-0.2326
"00074dd007"	"00074dd73c"	-0.1677
"00074dd007"	"00074dda10"	-0.2875
"00074dd007"	"00074ddab8"	0.1549
"00074dd007"	"00074ddd3d"	0.0436
"00074dd007"	"00074ddf16"	-0.0207
"00074dd007"	"00074ddfc1"	-0.0782
"00074dd007"	"00074de21a"	-0.1948
"00074dd007"	"00074de2a9"	0.2114
"00074dd007"	"00074de544"	-0.2543
"00074dd007"	"00074de98a"	-0.1573
"00074dd007"	"00074dea7e"	-0.1514
"00074dd007"	"00074debd9"	0.166
"00074dd007"	"00074deca3"	-0.0945
"00074dd007"	"00074def43"	-0.1674
"00074dd007"	"00074def99"	-0.2195
"00074dd007"	"00074ecdad"	-0.297
"00074dd007"	"00074ecf28"	-0.163
"00074dd007"	"00074ed1e1"	-0.422
"00074dd007"	"00074ed83b"	-0.0377
"00074dd007"	"00074ee5e3"	-0.04
"00074dd007"	"00074ee6e0"	-0.1612
"00074dd007"	"00074eea3a"	-0.2226
"00074dd007"	"00074eff82"	-0.2464
"00074dd007"	"00074f0477"	-0.156
"00074dd007"	"00074f08c3"	-0.132
"00074dd007"	"00074f1859"	-0.1795
"00074dd007"	"00074f2268"	-0.1151
"00074dd007"	"00074f28be"	-0.1079
"00074dd007"	"00074f294b"	-0.1871
"00074dd007"	"00074f2ddd"	-0.1558
"00074dd007"	"00074f2e75"	-0.5735
"00074dd007"	"00074f3088"	-0.1645
"00074dd007"	"00074f5a1c"	-0.01
"00074dd007"	"00074f75b7"	0.0468
"00074dd007"	"00074f8cd9"	-0.1605
"00074dd007"	"00074f96dc"	-0.4374
"00074dd007"	"00074fabaa"	-0.0893
"00074dd007"	"00074facd9"	0.1079
"00074dd007"	"00074fae3c"	-0.3224

"00074dd007"	"00074fb0a8"	0.0695
"00074dd007"	"00074fb4e4"	-6e-04
"00074dd007"	"00074fb7c2"	-0.2762
"00074dd007"	"00074fbd36"	-0.0486
"00074dd007"	"00074fc27f"	0.1811
"00074dd007"	"00074fc31d"	-0.1148
"00074dd007"	"00074fd569"	-0.2462
"00074dd007"	"00074fef15"	-0.3082
"00074dd007"	"00074ff562"	-0.4935
"00074dd007"	"00075007ca"	0.0561
"00074dd007"	"0007500b86"	0.0466
"00074dd007"	"0007500d05"	-0.1294
"00074dd007"	"0007500ee4"	0.0117
"00074dd007"	"0007500eee"	-0.0972
"00074dd007"	"00075013dc"	-0.0762
"00074dd007"	"000757b515"	0.1553
"00074dd007"	"000757bc5a"	0.1243
"00074dd007"	"000757c320"	-0.1375
"00074dd007"	"000757c9aa"	0.173
"00074dd007"	"000757ccbe"	-0.2352
"00074dd007"	"000757cfa9"	-0.0978
"00074dd007"	"000757d390"	0.1263
"00074dd007"	"000757d393"	0.1674
"00074dd007"	"000757d598"	0.2537
"00074dd007"	"000757d5a2"	0.0577
"00074dd007"	"000757d790"	0
"00074dd007"	"000757e30c"	0.3172
"00074dd007"	"000757e4b0"	0.0548
"00074dd007"	"000757e7a0"	0.0073
"00074dd007"	"000757e8b3"	-0.1943
"00074dd007"	"000757f627"	0.0033
"00074dd007"	"000757f925"	0.0511
"00074dd007"	"000757fa08"	-0.2999
"00074dd007"	"000757fe52"	-0.1356
"00074dd007"	"000758024a"	0.2069
"00074dd007"	"00075804bb"	0.1748
"00074dd007"	"00075a0c04"	-0.4703
"00074dd007"	"00075a3110"	-0.0984
"00074dd007"	"00075a341a"	-0.5234
"00074dd007"	"00075a3dcf"	-0.0757
"00074dd007"	"00075a3e22"	-0.3462
"00074dd007"	"00075a48d8"	-0.5066
"00074dd007"	"00075a5cfb"	0.2345
"00074dd007"	"00075a6151"	0.1148
"00074dd007"	"00075a6708"	0.1836
"00074dd007"	"00075a7319"	-0.0326
"00074dd007"	"00075a7723"	-0.2243
"00074dd007"	"00075a778b"	-0.2375
"00074dd007"	"00075a7b8e"	0.2022
"00074dd007"	"00075a7c79"	-0.2091

"00074dd007"	"00075a81b6"	-0.3968
"00074dd007"	"00075a82ac"	-0.0772
"00074dd007"	"00075a98e5"	-0.4349
"00074dd007"	"00075b0d29"	-0.1591
"00074dd007"	"00075b102a"	0.0576
"00074dd007"	"00075b1074"	-0.4396
"00074dd007"	"00075b135d"	-0.3619
"00074dd007"	"00075b138b"	0.0258
"00074dd007"	"00075b13a0"	-0.2109
"00074dd007"	"00075b13bd"	-0.363
"00074dd007"	"00075b16a9"	-0.0802
"00074dd007"	"00075b1a28"	-0.2638
"00074dd007"	"00075b1a97"	-0.2505
"00074dd007"	"00075b1c7b"	-0.2413
"00074dd007"	"00075b1d24"	-0.1443
"00074dd007"	"00075b202b"	0.2803
"00074dd007"	"00075b22cb"	-0.2742
"00074dd007"	"00075b22da"	-0.1436
"00074dd007"	"00075b2556"	-0.1044
"00074dd007"	"00075b25de"	0.171
"00074dd007"	"00075b260c"	0.0872
"00074dd007"	"00075b26f1"	-0.4061
"00074dd007"	"00075b2920"	0.3324
"00074dd007"	"00075b2a64"	0.0922
"00074dd007"	"00075b2a9d"	0.1838
"00074dd007"	"00075b2b37"	-0.1556
"00074dd007"	"00075b2cdd"	-0.2786
"00074dd007"	"00075b3038"	-0.0142
"00074dd007"	"00075b30fe"	-0.1426
"00074dd007"	"00075b3362"	-0.283
"00074dd007"	"00075b350a"	-0.1151
"00074dd007"	"00075b350e"	0.0359
"00074dd007"	"00075b3651"	-0.3572
"00074dd007"	"00075b38ca"	-0.2786
"00074dd007"	"00075b39cc"	-0.3791
"00074dd007"	"00075b3e1e"	-0.1985
"00074dd007"	"00075b3e57"	-0.0372
"00074dd007"	"00075b4079"	-0.3717
"00074dd007"	"00075b4150"	0.388
"00074dd007"	"00075b4194"	-0.0027
"00074dd007"	"00075b42d5"	-0.1703
"00074dd007"	"00075b4424"	-0.1912
"00074dd007"	"00075b4470"	0.1087
"00074dd007"	"00075b47ed"	-0.3353
"00074dd007"	"00075b4850"	0.1421
"00074dd007"	"00075b4ca0"	-0.0751
"00074dd007"	"00075b4d7f"	-0.1801
"00074dd007"	"00075b520f"	8e-04
"00074dd007"	"00075b525f"	-0.4065
"00074dd007"	"00075b58f8"	0.4232

"00074dd007"	"00075b5bcc"	-0.0892
"00074dd007"	"00075b5bfa"	-0.1864
"00074dd007"	"00075b6339"	0.0724
"00074dd007"	"00075b6658"	-0.5744
"00074dd007"	"00075b679a"	0.1061
"00074dd007"	"00075b6cb7"	-0.0788
"00074dd007"	"00075b6df8"	-0.4009
"00074dd007"	"00075b6ff6"	0.134
"00074dd007"	"00075b70ee"	0.0191
"00074dd007"	"00075b7157"	-0.0518
"00074dd007"	"00075b7225"	-0.1052
"00074dd007"	"00075b7c89"	-0.1054
"00074dd007"	"00075b9048"	0.1598
"00074dd007"	"00075d0801"	0.1037
"00074dd007"	"00075d1820"	-0.2329
"00074dd007"	"00075d1f3d"	0.1104
"00074dd007"	"00075d2329"	-0.1485
"00074dd007"	"00075d2b9b"	-0.0235
"00074dd007"	"00075d3941"	-0.5007
"00074dd007"	"00075d3e96"	-0.0509
"00074dd007"	"00075d4864"	-0.1646
"00074dd007"	"00075d5961"	-0.0808
"00074dd007"	"00075d5a63"	0.007
"00074dd007"	"00075d6150"	-0.2723
"00074dd007"	"00075d67d0"	-0.3249
"00074dd007"	"00075d67e2"	-0.2351
"00074dd007"	"00075d73fc"	-0.2562
"00074dd007"	"00075d7729"	-0.1819
"00074dd007"	"00075d778c"	-0.4674
"00074dd007"	"00075d7b9e"	-0.2195
"00074dd007"	"00075d7c8f"	-0.2958
"00074dd007"	"00075d804d"	0.0685
"00074dd007"	"00075d819f"	-0.0053
"00074dd007"	"00075d8601"	-0.3668
"00074dd007"	"00075d8c6a"	-0.3053
"00074dd007"	"00075dfedc"	-0.3266
"00074dd007"	"00075e05f2"	-0.0265
"00074dd007"	"00075e0837"	0.1169
"00074dd007"	"00075e092e"	-0.1035
"00074dd007"	"00075e0965"	0.1403
"00074dd007"	"00075e0bc8"	0.3609
"00074dd007"	"00075e0fbb"	-0.2145
"00074dd163"	"00074dd3df"	-0.2658
"00074dd163"	"00074dd577"	-0.2925
"00074dd163"	"00074dd62e"	-0.048
"00074dd163"	"00074dd73c"	0.2112
"00074dd163"	"00074dda10"	-0.1596
"00074dd163"	"00074ddab8"	-0.1592
"00074dd163"	"00074ddd3d"	-0.4069
"00074dd163"	"00074ddf16"	0.197

"00074dd163"	"00074ddfc1"	0.2832
"00074dd163"	"00074de21a"	−0.2397
"00074dd163"	"00074de2a9"	−0.0595
"00074dd163"	"00074de544"	−0.0194
"00074dd163"	"00074de98a"	0.0063
"00074dd163"	"00074dea7e"	−0.1379
"00074dd163"	"00074debd9"	0.35
"00074dd163"	"00074deca3"	0.6835
"00074dd163"	"00074def43"	0.3686
"00074dd163"	"00074def99"	−0.2946
"00074dd163"	"00074ecdad"	−0.0605
"00074dd163"	"00074ecf28"	−0.3175
"00074dd163"	"00074ed1e1"	−0.2535
"00074dd163"	"00074ed83b"	0.0972
"00074dd163"	"00074ee5e3"	0.1793
"00074dd163"	"00074ee6e0"	−0.2321
"00074dd163"	"00074eea3a"	0.0182
"00074dd163"	"00074eff82"	−0.1024
"00074dd163"	"00074f0477"	−0.2963
"00074dd163"	"00074f08c3"	−0.2793
"00074dd163"	"00074f1859"	0.0516
"00074dd163"	"00074f2268"	−0.4127
"00074dd163"	"00074f28be"	−0.0115
"00074dd163"	"00074f294b"	−0.3326
"00074dd163"	"00074f2ddd"	−0.0552
"00074dd163"	"00074f2e75"	−0.4191
"00074dd163"	"00074f3088"	−0.2387
"00074dd163"	"00074f5a1c"	0.1807
"00074dd163"	"00074f75b7"	0.2763
"00074dd163"	"00074f8cd9"	0.0809
"00074dd163"	"00074f96dc"	−0.2212
"00074dd163"	"00074fabaa"	0.09
"00074dd163"	"00074facd9"	−0.0541
"00074dd163"	"00074fae3c"	−0.0373
"00074dd163"	"00074fb0a8"	0.0235
"00074dd163"	"00074fb4e4"	−0.0496
"00074dd163"	"00074fb7c2"	−0.0909
"00074dd163"	"00074fbd36"	−0.1297
"00074dd163"	"00074fc27f"	−0.0501
"00074dd163"	"00074fc31d"	−0.1132
"00074dd163"	"00074fd569"	−0.142
"00074dd163"	"00074fef15"	−0.0414
"00074dd163"	"00074ff562"	−0.2755
"00074dd163"	"00075007ca"	−0.1704
"00074dd163"	"0007500b86"	0.2378
"00074dd163"	"0007500d05"	−0.037
"00074dd163"	"0007500ee4"	−0.1425
"00074dd163"	"0007500eee"	−0.035
"00074dd163"	"00075013dc"	−0.4449
"00074dd163"	"000757b515"	−0.2335

"00074dd163"	"000757bc5a"	−0.0196
"00074dd163"	"000757c320"	0.0912
"00074dd163"	"000757c9aa"	−0.0917
"00074dd163"	"000757ccbe"	−0.111
"00074dd163"	"000757cfa9"	0.071
"00074dd163"	"000757d390"	−0.1109
"00074dd163"	"000757d393"	0.4093
"00074dd163"	"000757d598"	−0.3758
"00074dd163"	"000757d5a2"	−0.4236
"00074dd163"	"000757d790"	0.1113
"00074dd163"	"000757e30c"	−0.4727
"00074dd163"	"000757e4b0"	−0.0609
"00074dd163"	"000757e7a0"	−0.2549
"00074dd163"	"000757e8b3"	0.0977
"00074dd163"	"000757f627"	0.0037
"00074dd163"	"000757f925"	0.11
"00074dd163"	"000757fa08"	−0.2009
"00074dd163"	"000757fe52"	0.0681
"00074dd163"	"000758024a"	−0.3126
"00074dd163"	"00075804bb"	−0.2622
"00074dd163"	"00075a0c04"	−0.0655
"00074dd163"	"00075a3110"	−0.1122
"00074dd163"	"00075a341a"	−0.228
"00074dd163"	"00075a3dcf"	0.2553
"00074dd163"	"00075a3e22"	−0.1248
"00074dd163"	"00075a48d8"	−0.0913
"00074dd163"	"00075a5cfb"	−0.2498
"00074dd163"	"00075a6151"	−0.003
"00074dd163"	"00075a6708"	−0.3787
"00074dd163"	"00075a7319"	−0.0566
"00074dd163"	"00075a7723"	−0.1759
"00074dd163"	"00075a778b"	−0.3372
"00074dd163"	"00075a7b8e"	−0.1908
"00074dd163"	"00075a7c79"	−0.0797
"00074dd163"	"00075a81b6"	−0.3101
"00074dd163"	"00075a82ac"	−0.1216
"00074dd163"	"00075a98e5"	−0.1721
"00074dd163"	"00075b0d29"	−0.0455
"00074dd163"	"00075b102a"	−0.1239
"00074dd163"	"00075b1074"	−0.3885
"00074dd163"	"00075b135d"	−0.137
"00074dd163"	"00075b138b"	0.0659
"00074dd163"	"00075b13a0"	−0.0662
"00074dd163"	"00075b13bd"	−0.2545
"00074dd163"	"00075b16a9"	−0.0889
"00074dd163"	"00075b1a28"	−0.0197
"00074dd163"	"00075b1a97"	−0.0757
"00074dd163"	"00075b1c7b"	−0.2744
"00074dd163"	"00075b1d24"	−0.2886
"00074dd163"	"00075b202b"	−0.0717

"00074dd163"	"00075b22cb"	-0.0452
"00074dd163"	"00075b22da"	-0.0775
"00074dd163"	"00075b2556"	-0.2225
"00074dd163"	"00075b25de"	0.223
"00074dd163"	"00075b260c"	-0.1232
"00074dd163"	"00075b26f1"	-0.1705
"00074dd163"	"00075b2920"	-0.3399
"00074dd163"	"00075b2a64"	-0.3884
"00074dd163"	"00075b2a9d"	-0.2092
"00074dd163"	"00075b2b37"	-0.3152
"00074dd163"	"00075b2cdd"	-0.056
"00074dd163"	"00075b3038"	0.1326
"00074dd163"	"00075b30fe"	-0.2411
"00074dd163"	"00075b3362"	-0.2281
"00074dd163"	"00075b350a"	-0.2858
"00074dd163"	"00075b350e"	0.2634
"00074dd163"	"00075b3651"	-0.3337
"00074dd163"	"00075b38ca"	0.3021
"00074dd163"	"00075b39cc"	-0.0066
"00074dd163"	"00075b3e1e"	0.2001
"00074dd163"	"00075b3e57"	-0.1775
"00074dd163"	"00075b4079"	-0.1053
"00074dd163"	"00075b4150"	-0.3219
"00074dd163"	"00075b4194"	0.1877
"00074dd163"	"00075b42d5"	0.1809
"00074dd163"	"00075b4424"	-0.397
"00074dd163"	"00075b4470"	-0.0196
"00074dd163"	"00075b47ed"	0.0448
"00074dd163"	"00075b4850"	-0.095
"00074dd163"	"00075b4ca0"	-0.3965
"00074dd163"	"00075b4d7f"	0.0751
"00074dd163"	"00075b520f"	-0.0387
"00074dd163"	"00075b525f"	-0.0956
"00074dd163"	"00075b58f8"	-0.1113
"00074dd163"	"00075b5bcc"	-0.125
"00074dd163"	"00075b5bfa"	-0.0426
"00074dd163"	"00075b6339"	0.0633
"00074dd163"	"00075b6658"	-0.0842
"00074dd163"	"00075b679a"	-0.0187
"00074dd163"	"00075b6cb7"	-0.3894
"00074dd163"	"00075b6df8"	-0.0984
"00074dd163"	"00075b6ff6"	-0.261
"00074dd163"	"00075b70ee"	-0.28
"00074dd163"	"00075b7157"	-0.1245
"00074dd163"	"00075b7225"	-0.0048
"00074dd163"	"00075b7c89"	-0.0998
"00074dd163"	"00075b9048"	-0.169
"00074dd163"	"00075d0801"	0.1678
"00074dd163"	"00075d1820"	-0.2435
"00074dd163"	"00075d1f3d"	-0.2334

"00074dd163"	"00075d2329"	-0.0851
"00074dd163"	"00075d2b9b"	-0.1141
"00074dd163"	"00075d3941"	-0.2157
"00074dd163"	"00075d3e96"	-0.2841
"00074dd163"	"00075d4864"	0.159
"00074dd163"	"00075d5961"	0.1393
"00074dd163"	"00075d5a63"	-0.1088
"00074dd163"	"00075d6150"	0.0731
"00074dd163"	"00075d67d0"	-0.3115
"00074dd163"	"00075d67e2"	-0.0539
"00074dd163"	"00075d73fc"	-0.0405
"00074dd163"	"00075d7729"	-0.3931
"00074dd163"	"00075d778c"	-0.0608
"00074dd163"	"00075d7b9e"	0.0576
"00074dd163"	"00075d7c8f"	-0.049
"00074dd163"	"00075d804d"	-0.0729
"00074dd163"	"00075d819f"	-0.1927
"00074dd163"	"00075d8601"	0.1045
"00074dd163"	"00075d8c6a"	-0.019
"00074dd163"	"00075dfedc"	0.0341
"00074dd163"	"00075e05f2"	0.2975
"00074dd163"	"00075e0837"	-0.1786
"00074dd163"	"00075e092e"	-0.0374
"00074dd163"	"00075e0965"	-0.4274
"00074dd163"	"00075e0bc8"	0.1057
"00074dd163"	"00075e0fbb"	-0.1054
"00074dd3df"	"00074dd577"	0.005
"00074dd3df"	"00074dd62e"	-0.0836
"00074dd3df"	"00074dd73c"	-0.0521
"00074dd3df"	"00074dda10"	-0.1591
"00074dd3df"	"00074ddab8"	-0.0585
"00074dd3df"	"00074ddd3d"	-0.4159
"00074dd3df"	"00074ddf16"	-0.0214
"00074dd3df"	"00074ddfc1"	0.0244
"00074dd3df"	"00074de21a"	-0.2361
"00074dd3df"	"00074de2a9"	-0.0247
"00074dd3df"	"00074de544"	-0.0246
"00074dd3df"	"00074de98a"	-0.0169
"00074dd3df"	"00074dea7e"	0.1295
"00074dd3df"	"00074debd9"	-0.3046
"00074dd3df"	"00074deca3"	-0.1319
"00074dd3df"	"00074def43"	0.0021
"00074dd3df"	"00074def99"	-0.1828
"00074dd3df"	"00074ecdad"	-0.203
"00074dd3df"	"00074ecf28"	-0.0444
"00074dd3df"	"00074ed1e1"	-0.0514
"00074dd3df"	"00074ed83b"	-0.1921
"00074dd3df"	"00074ee5e3"	-0.4146
"00074dd3df"	"00074ee6e0"	-0.1827
"00074dd3df"	"00074eea3a"	0.4345

"00074dd3df"	"00074eff82"	-0.1075
"00074dd3df"	"00074f0477"	-0.1394
"00074dd3df"	"00074f08c3"	-0.4671
"00074dd3df"	"00074f1859"	0.0363
"00074dd3df"	"00074f2268"	0.0935
"00074dd3df"	"00074f28be"	-0.2677
"00074dd3df"	"00074f294b"	0.374
"00074dd3df"	"00074f2ddd"	-0.2398
"00074dd3df"	"00074f2e75"	-0.106
"00074dd3df"	"00074f3088"	-0.1141
"00074dd3df"	"00074f5a1c"	0.1704
"00074dd3df"	"00074f75b7"	-0.2616
"00074dd3df"	"00074f8cd9"	-0.2594
"00074dd3df"	"00074f96dc"	-0.3057
"00074dd3df"	"00074fabaa"	-0.0255
"00074dd3df"	"00074facd9"	-0.208
"00074dd3df"	"00074fae3c"	-0.1985
"00074dd3df"	"00074fb0a8"	-0.2462
"00074dd3df"	"00074fb4e4"	-0.5311
"00074dd3df"	"00074fb7c2"	0.125
"00074dd3df"	"00074fbd36"	0.0529
"00074dd3df"	"00074fc27f"	0.0722
"00074dd3df"	"00074fc31d"	-0.1979
"00074dd3df"	"00074fd569"	-0.2837
"00074dd3df"	"00074fef15"	0.0862
"00074dd3df"	"00074ff562"	-0.3192
"00074dd3df"	"00075007ca"	-0.4608
"00074dd3df"	"0007500b86"	-0.1417
"00074dd3df"	"0007500d05"	-0.0227
"00074dd3df"	"0007500ee4"	-0.163
"00074dd3df"	"0007500eee"	-0.1018
"00074dd3df"	"00075013dc"	-0.6982
"00074dd3df"	"000757b515"	-0.198
"00074dd3df"	"000757bc5a"	-0.0466
"00074dd3df"	"000757c320"	-0.2889
"00074dd3df"	"000757c9aa"	-0.3844
"00074dd3df"	"000757ccbe"	-0.1979
"00074dd3df"	"000757cfa9"	-0.3972
"00074dd3df"	"000757d390"	-0.4964
"00074dd3df"	"000757d393"	0.0094
"00074dd3df"	"000757d598"	-0.5555
"00074dd3df"	"000757d5a2"	-0.4516
"00074dd3df"	"000757d790"	-0.086
"00074dd3df"	"000757e30c"	-0.4489
"00074dd3df"	"000757e4b0"	0.0972
"00074dd3df"	"000757e7a0"	-0.2998
"00074dd3df"	"000757e8b3"	-0.0783
"00074dd3df"	"000757f627"	-0.2148
"00074dd3df"	"000757f925"	-0.1044
"00074dd3df"	"000757fa08"	-0.029

"00074dd3df"	"000757fe52"	-5e-04
"00074dd3df"	"000758024a"	-0.495
"00074dd3df"	"00075804bb"	-0.4848
"00074dd3df"	"00075a0c04"	0.0885
"00074dd3df"	"00075a3110"	-0.2116
"00074dd3df"	"00075a341a"	0.1288
"00074dd3df"	"00075a3dcf"	0.2382
"00074dd3df"	"00075a3e22"	0.0759
"00074dd3df"	"00075a48d8"	0.0075
"00074dd3df"	"00075a5cfb"	-0.3449
"00074dd3df"	"00075a6151"	-0.5113
"00074dd3df"	"00075a6708"	-0.5333
"00074dd3df"	"00075a7319"	-0.5577
"00074dd3df"	"00075a7723"	-0.061
"00074dd3df"	"00075a778b"	0.0575
"00074dd3df"	"00075a7b8e"	-0.1984
"00074dd3df"	"00075a7c79"	-0.1554
"00074dd3df"	"00075a81b6"	-0.4201
"00074dd3df"	"00075a82ac"	0.2876
"00074dd3df"	"00075a98e5"	0.0798
"00074dd3df"	"00075b0d29"	-0.2584
"00074dd3df"	"00075b102a"	-0.1252
"00074dd3df"	"00075b1074"	0.383
"00074dd3df"	"00075b135d"	-0.4003
"00074dd3df"	"00075b138b"	0.1379
"00074dd3df"	"00075b13a0"	-0.0025
"00074dd3df"	"00075b13bd"	0.1533
"00074dd3df"	"00075b16a9"	-7e-04
"00074dd3df"	"00075b1a28"	-0.0073
"00074dd3df"	"00075b1a97"	0.1581
"00074dd3df"	"00075b1c7b"	-0.4909
"00074dd3df"	"00075b1d24"	-0.0547
"00074dd3df"	"00075b202b"	-0.2722
"00074dd3df"	"00075b22cb"	0.29
"00074dd3df"	"00075b22da"	0.2704
"00074dd3df"	"00075b2556"	0.3238
"00074dd3df"	"00075b25de"	-0.2502
"00074dd3df"	"00075b260c"	-0.3048
"00074dd3df"	"00075b26f1"	-0.2009
"00074dd3df"	"00075b2920"	-0.4573
"00074dd3df"	"00075b2a64"	-0.3741
"00074dd3df"	"00075b2a9d"	-0.4257
"00074dd3df"	"00075b2b37"	-0.3895
"00074dd3df"	"00075b2cdd"	-0.0222
"00074dd3df"	"00075b3038"	-0.1463
"00074dd3df"	"00075b30fe"	0.244
"00074dd3df"	"00075b3362"	-0.2365
"00074dd3df"	"00075b350a"	-0.0648
"00074dd3df"	"00075b350e"	-0.1473
"00074dd3df"	"00075b3651"	-0.1164

"00074dd3df"	"00075b38ca"	0.0314
"00074dd3df"	"00075b39cc"	−0.0356
"00074dd3df"	"00075b3e1e"	−0.0743
"00074dd3df"	"00075b3e57"	0.1319
"00074dd3df"	"00075b4079"	−0.5215
"00074dd3df"	"00075b4150"	−0.3469
"00074dd3df"	"00075b4194"	−0.2458
"00074dd3df"	"00075b42d5"	0.1272
"00074dd3df"	"00075b4424"	−0.1417
"00074dd3df"	"00075b4470"	0.0445
"00074dd3df"	"00075b47ed"	−0.0252
"00074dd3df"	"00075b4850"	−0.2051
"00074dd3df"	"00075b4ca0"	−0.3397
"00074dd3df"	"00075b4d7f"	0.1677
"00074dd3df"	"00075b520f"	−0.2893
"00074dd3df"	"00075b525f"	−0.1348
"00074dd3df"	"00075b58f8"	−0.3531
"00074dd3df"	"00075b5bcc"	−0.1898
"00074dd3df"	"00075b5bfa"	−0.3929
"00074dd3df"	"00075b6339"	−0.2088
"00074dd3df"	"00075b6658"	−0.1075
"00074dd3df"	"00075b679a"	−0.0466
"00074dd3df"	"00075b6cb7"	−0.2568
"00074dd3df"	"00075b6df8"	0.0217
"00074dd3df"	"00075b6ff6"	−4e−04
"00074dd3df"	"00075b70ee"	−0.5895
"00074dd3df"	"00075b7157"	0.0327
"00074dd3df"	"00075b7225"	0.0148
"00074dd3df"	"00075b7c89"	−0.1804
"00074dd3df"	"00075b9048"	−0.1979
"00074dd3df"	"00075d0801"	−0.2905
"00074dd3df"	"00075d1820"	−0.1006
"00074dd3df"	"00075d1f3d"	−0.2826
"00074dd3df"	"00075d2329"	0.1857
"00074dd3df"	"00075d2b9b"	−0.2774
"00074dd3df"	"00075d3941"	−0.1133
"00074dd3df"	"00075d3e96"	−0.2256
"00074dd3df"	"00075d4864"	0.1587
"00074dd3df"	"00075d5961"	0.0726
"00074dd3df"	"00075d5a63"	−0.0978
"00074dd3df"	"00075d6150"	−0.3062
"00074dd3df"	"00075d67d0"	−0.3594
"00074dd3df"	"00075d67e2"	−0.079
"00074dd3df"	"00075d73fc"	−0.0093
"00074dd3df"	"00075d7729"	−0.3279
"00074dd3df"	"00075d778c"	−0.2635
"00074dd3df"	"00075d7b9e"	−0.1864
"00074dd3df"	"00075d7c8f"	−0.0048
"00074dd3df"	"00075d804d"	−0.121
"00074dd3df"	"00075d819f"	−0.1382

"00074dd3df"	"00075d8601"	-0.2804
"00074dd3df"	"00075d8c6a"	-0.1126
"00074dd3df"	"00075dfedc"	-0.1383
"00074dd3df"	"00075e05f2"	-0.2169
"00074dd3df"	"00075e0837"	-0.1728
"00074dd3df"	"00075e092e"	0.0327
"00074dd3df"	"00075e0965"	-0.4751
"00074dd3df"	"00075e0bc8"	-0.0952
"00074dd3df"	"00075e0fbb"	0.3258
"00074dd577"	"00074dd62e"	-0.1185
"00074dd577"	"00074dd73c"	0.2299
"00074dd577"	"00074dda10"	-0.0617
"00074dd577"	"00074ddab8"	0.1617
"00074dd577"	"00074ddd3d"	0.2581
"00074dd577"	"00074ddf16"	0.1075
"00074dd577"	"00074ddfc1"	0.1561
"00074dd577"	"00074de21a"	-0.2127
"00074dd577"	"00074de2a9"	0.0521
"00074dd577"	"00074de544"	0.2053
"00074dd577"	"00074de98a"	0.0312
"00074dd577"	"00074dea7e"	0.0624
"00074dd577"	"00074debd9"	-0.2574
"00074dd577"	"00074deca3"	-0.1299
"00074dd577"	"00074def43"	-0.053
"00074dd577"	"00074def99"	0.0356
"00074dd577"	"00074ecdad"	0.0314
"00074dd577"	"00074ecf28"	-0.2471
"00074dd577"	"00074ed1e1"	0.2675
"00074dd577"	"00074ed83b"	-0.0132
"00074dd577"	"00074ee5e3"	-0.3314
"00074dd577"	"00074ee6e0"	-0.2123
"00074dd577"	"00074eea3a"	0.179
"00074dd577"	"00074eff82"	-0.0532
"00074dd577"	"00074f0477"	0.0457
"00074dd577"	"00074f08c3"	-0.3182
"00074dd577"	"00074f1859"	-0.0838
"00074dd577"	"00074f2268"	0.1227
"00074dd577"	"00074f28be"	0.149
"00074dd577"	"00074f294b"	0.2188
"00074dd577"	"00074f2ddd"	0.1374
"00074dd577"	"00074f2e75"	0.0613
"00074dd577"	"00074f3088"	-0.0806
"00074dd577"	"00074f5a1c"	0.2476
"00074dd577"	"00074f75b7"	0.0409
"00074dd577"	"00074f8cd9"	-0.087
"00074dd577"	"00074f96dc"	-0.5252
"00074dd577"	"00074fabaa"	-0.0851
"00074dd577"	"00074facd9"	-0.1373
"00074dd577"	"00074fae3c"	-0.4736
"00074dd577"	"00074fb0a8"	-0.2241

"00074dd577"	"00074fb4e4"	-0.5104
"00074dd577"	"00074fb7c2"	0.3079
"00074dd577"	"00074fbd36"	-0.0135
"00074dd577"	"00074fc27f"	0.2919
"00074dd577"	"00074fc31d"	-0.0204
"00074dd577"	"00074fd569"	-0.069
"00074dd577"	"00074fef15"	-0.017
"00074dd577"	"00074ff562"	-0.1105
"00074dd577"	"00075007ca"	-0.059
"00074dd577"	"0007500b86"	-0.0447
"00074dd577"	"0007500d05"	-0.0053
"00074dd577"	"0007500ee4"	0.0606
"00074dd577"	"0007500eee"	-0.2633
"00074dd577"	"00075013dc"	-0.0427
"00074dd577"	"000757b515"	0.525
"00074dd577"	"000757bc5a"	-0.0252
"00074dd577"	"000757c320"	-0.3827
"00074dd577"	"000757c9aa"	-0.0803
"00074dd577"	"000757ccbe"	0.0484
"00074dd577"	"000757cfa9"	0.0076
"00074dd577"	"000757d390"	-0.2197
"00074dd577"	"000757d393"	0.1356
"00074dd577"	"000757d598"	0.238
"00074dd577"	"000757d5a2"	0.3212
"00074dd577"	"000757d790"	0.105
"00074dd577"	"000757e30c"	-0.0848
"00074dd577"	"000757e4b0"	0.3405
"00074dd577"	"000757e7a0"	0.1016
"00074dd577"	"000757e8b3"	0.1458
"00074dd577"	"000757f627"	-0.1804
"00074dd577"	"000757f925"	-0.0324
"00074dd577"	"000757fa08"	0.1197
"00074dd577"	"000757fe52"	0.1534
"00074dd577"	"000758024a"	-0.2021
"00074dd577"	"00075804bb"	-0.0168
"00074dd577"	"00075a0c04"	0.2097
"00074dd577"	"00075a3110"	0.412
"00074dd577"	"00075a341a"	0.1708
"00074dd577"	"00075a3dcf"	0.226
"00074dd577"	"00075a3e22"	0.1675
"00074dd577"	"00075a48d8"	-0.0369
"00074dd577"	"00075a5cfb"	-0.078
"00074dd577"	"00075a6151"	0.0955
"00074dd577"	"00075a6708"	0.2986
"00074dd577"	"00075a7319"	-0.3432
"00074dd577"	"00075a7723"	0.0494
"00074dd577"	"00075a778b"	0.146
"00074dd577"	"00075a7b8e"	-0.123
"00074dd577"	"00075a7c79"	0.5731
"00074dd577"	"00075a81b6"	-0.0047

"00074dd577"	"00075a82ac"	0.2691
"00074dd577"	"00075a98e5"	-0.1078
"00074dd577"	"00075b0d29"	-0.0534
"00074dd577"	"00075b102a"	0.0492
"00074dd577"	"00075b1074"	0.0175
"00074dd577"	"00075b135d"	0.0413
"00074dd577"	"00075b138b"	0.2691
"00074dd577"	"00075b13a0"	-0.0844
"00074dd577"	"00075b13bd"	-0.1855
"00074dd577"	"00075b16a9"	-0.0879
"00074dd577"	"00075b1a28"	-0.2828
"00074dd577"	"00075b1a97"	0.0845
"00074dd577"	"00075b1c7b"	-0.3657
"00074dd577"	"00075b1d24"	-0.0294
"00074dd577"	"00075b202b"	0.0364
"00074dd577"	"00075b22cb"	-0.1358
"00074dd577"	"00075b22da"	0.1809
"00074dd577"	"00075b2556"	0.084
"00074dd577"	"00075b25de"	0.1209
"00074dd577"	"00075b260c"	0.3057
"00074dd577"	"00075b26f1"	-0.2553
"00074dd577"	"00075b2920"	0.0227
"00074dd577"	"00075b2a64"	-0.0833
"00074dd577"	"00075b2a9d"	0.0962
"00074dd577"	"00075b2b37"	-0.342
"00074dd577"	"00075b2cdd"	-0.0517
"00074dd577"	"00075b3038"	0.304
"00074dd577"	"00075b30fe"	0.2646
"00074dd577"	"00075b3362"	-0.4359
"00074dd577"	"00075b350a"	0.0047
"00074dd577"	"00075b350e"	-0.0033
"00074dd577"	"00075b3651"	-0.2683
"00074dd577"	"00075b38ca"	-0.0225
"00074dd577"	"00075b39cc"	-0.0085
"00074dd577"	"00075b3e1e"	-0.0517
"00074dd577"	"00075b3e57"	-0.1116
"00074dd577"	"00075b4079"	-0.3238
"00074dd577"	"00075b4150"	0.0251
"00074dd577"	"00075b4194"	0.0459
"00074dd577"	"00075b42d5"	0.2196
"00074dd577"	"00075b4424"	0.0378
"00074dd577"	"00075b4470"	0.0441
"00074dd577"	"00075b47ed"	-0.0496
"00074dd577"	"00075b4850"	0.1185
"00074dd577"	"00075b4ca0"	0.2564
"00074dd577"	"00075b4d7f"	0.1979
"00074dd577"	"00075b520f"	-0.0358
"00074dd577"	"00075b525f"	-0.1044
"00074dd577"	"00075b58f8"	-0.1626
"00074dd577"	"00075b5bcc"	0.0679

"00074dd577"	"00075b5bfa"	0.0409
"00074dd577"	"00075b6339"	0.2986
"00074dd577"	"00075b6658"	−0.2512
"00074dd577"	"00075b679a"	0.2077
"00074dd577"	"00075b6cb7"	0.0126
"00074dd577"	"00075b6df8"	0.2047
"00074dd577"	"00075b6ff6"	0.5208
"00074dd577"	"00075b70ee"	0.1278
"00074dd577"	"00075b7157"	0.0805
"00074dd577"	"00075b7225"	0.1879
"00074dd577"	"00075b7c89"	0.4257
"00074dd577"	"00075b9048"	0.5567
"00074dd577"	"00075d0801"	−0.1588
"00074dd577"	"00075d1820"	−0.3246
"00074dd577"	"00075d1f3d"	0.1982
"00074dd577"	"00075d2329"	0.2544
"00074dd577"	"00075d2b9b"	0.0631
"00074dd577"	"00075d3941"	−0.1312
"00074dd577"	"00075d3e96"	−0.046
"00074dd577"	"00075d4864"	−0.0688
"00074dd577"	"00075d5961"	0.0505
"00074dd577"	"00075d5a63"	0.1565
"00074dd577"	"00075d6150"	−0.0203
"00074dd577"	"00075d67d0"	−0.0589
"00074dd577"	"00075d67e2"	0.1621
"00074dd577"	"00075d73fc"	0.1132
"00074dd577"	"00075d7729"	0.1284
"00074dd577"	"00075d778c"	−0.2012
"00074dd577"	"00075d7b9e"	−0.0922
"00074dd577"	"00075d7c8f"	0.2444
"00074dd577"	"00075d804d"	0.1209
"00074dd577"	"00075d819f"	0.081
"00074dd577"	"00075d8601"	−0.2385
"00074dd577"	"00075d8c6a"	0.0287
"00074dd577"	"00075dfedc"	0.1995
"00074dd577"	"00075e05f2"	−0.0399
"00074dd577"	"00075e0837"	0.1138
"00074dd577"	"00075e092e"	0.0279
"00074dd577"	"00075e0965"	−0.2224
"00074dd577"	"00075e0bc8"	0.2776
"00074dd577"	"00075e0fbb"	0.0663
"00074dd62e"	"00074dd73c"	−0.0336
"00074dd62e"	"00074dda10"	−0.1624
"00074dd62e"	"00074ddab8"	0.0219
"00074dd62e"	"00074ddd3d"	−0.1206
"00074dd62e"	"00074ddf16"	−0.0814
"00074dd62e"	"00074ddfc1"	−0.2093
"00074dd62e"	"00074de21a"	−0.1065
"00074dd62e"	"00074de2a9"	0.2358
"00074dd62e"	"00074de544"	−0.001

"00074dd62e"	"00074de98a"	-0.009
"00074dd62e"	"00074dea7e"	-0.2489
"00074dd62e"	"00074debd9"	-0.2558
"00074dd62e"	"00074deca3"	-0.1219
"00074dd62e"	"00074def43"	0.027
"00074dd62e"	"00074def99"	-0.18
"00074dd62e"	"00074ecdad"	-0.3744
"00074dd62e"	"00074ecf28"	-0.0623
"00074dd62e"	"00074ed1e1"	-0.3046
"00074dd62e"	"00074ed83b"	0.189
"00074dd62e"	"00074ee5e3"	0.1299
"00074dd62e"	"00074ee6e0"	0.3623
"00074dd62e"	"00074eea3a"	-0.0455
"00074dd62e"	"00074eff82"	-0.0286
"00074dd62e"	"00074f0477"	-0.2699
"00074dd62e"	"00074f08c3"	-0.0447
"00074dd62e"	"00074f1859"	-0.1774
"00074dd62e"	"00074f2268"	-0.1615
"00074dd62e"	"00074f28be"	0.1749
"00074dd62e"	"00074f294b"	-0.0148
"00074dd62e"	"00074f2ddd"	-0.2493
"00074dd62e"	"00074f2e75"	-0.1048
"00074dd62e"	"00074f3088"	0.0367
"00074dd62e"	"00074f5a1c"	-0.1538
"00074dd62e"	"00074f75b7"	0.0589
"00074dd62e"	"00074f8cd9"	0.0156
"00074dd62e"	"00074f96dc"	0.0375
"00074dd62e"	"00074fabaa"	0.1293
"00074dd62e"	"00074facd9"	-0.1719
"00074dd62e"	"00074fae3c"	0.0552
"00074dd62e"	"00074fb0a8"	0.1811
"00074dd62e"	"00074fb4e4"	-0.0999
"00074dd62e"	"00074fb7c2"	0.2436
"00074dd62e"	"00074fbd36"	-0.1511
"00074dd62e"	"00074fc27f"	0.1927
"00074dd62e"	"00074fc31d"	0.1344
"00074dd62e"	"00074fd569"	0.2093
"00074dd62e"	"00074fef15"	-6e-04
"00074dd62e"	"00074ff562"	-0.3822
"00074dd62e"	"00075007ca"	-0.2105
"00074dd62e"	"0007500b86"	0.1219
"00074dd62e"	"0007500d05"	0.0085
"00074dd62e"	"0007500ee4"	-0.0055
"00074dd62e"	"0007500eee"	-0.1822
"00074dd62e"	"00075013dc"	0.0593
"00074dd62e"	"000757b515"	-0.0176
"00074dd62e"	"000757bc5a"	0.029
"00074dd62e"	"000757c320"	-0.0779
"00074dd62e"	"000757c9aa"	0.1463
"00074dd62e"	"000757ccbe"	0.1592

"00074dd62e"	"000757cfa9"	-0.2177
"00074dd62e"	"000757d390"	-0.1148
"00074dd62e"	"000757d393"	0.0686
"00074dd62e"	"000757d598"	0.1332
"00074dd62e"	"000757d5a2"	-0.0488
"00074dd62e"	"000757d790"	0.0735
"00074dd62e"	"000757e30c"	-0.1717
"00074dd62e"	"000757e4b0"	0.1433
"00074dd62e"	"000757e7a0"	-0.039
"00074dd62e"	"000757e8b3"	0.2327
"00074dd62e"	"000757f627"	-0.1639
"00074dd62e"	"000757f925"	0.2112
"00074dd62e"	"000757fa08"	-0.1574
"00074dd62e"	"000757fe52"	0.0713
"00074dd62e"	"000758024a"	-0.2035
"00074dd62e"	"00075804bb"	-0.0617
"00074dd62e"	"00075a0c04"	0.1398
"00074dd62e"	"00075a3110"	0.1954
"00074dd62e"	"00075a341a"	0.0627
"00074dd62e"	"00075a3dcf"	-0.4426
"00074dd62e"	"00075a3e22"	0.2654
"00074dd62e"	"00075a48d8"	0.0958
"00074dd62e"	"00075a5cfb"	0.1421
"00074dd62e"	"00075a6151"	-0.2378
"00074dd62e"	"00075a6708"	-0.1659
"00074dd62e"	"00075a7319"	-0.108
"00074dd62e"	"00075a7723"	-0.0122
"00074dd62e"	"00075a778b"	-0.074
"00074dd62e"	"00075a7b8e"	0.0311
"00074dd62e"	"00075a7c79"	0.1135
"00074dd62e"	"00075a81b6"	-0.2825
"00074dd62e"	"00075a82ac"	0.0039
"00074dd62e"	"00075a98e5"	-0.1124
"00074dd62e"	"00075b0d29"	-0.176
"00074dd62e"	"00075b102a"	0.0102
"00074dd62e"	"00075b1074"	-0.1029
"00074dd62e"	"00075b135d"	0.0236
"00074dd62e"	"00075b138b"	0.0726
"00074dd62e"	"00075b13a0"	-0.185
"00074dd62e"	"00075b13bd"	0.053
"00074dd62e"	"00075b16a9"	-0.116
"00074dd62e"	"00075b1a28"	-0.0297
"00074dd62e"	"00075b1a97"	0.0689
"00074dd62e"	"00075b1c7b"	-0.223
"00074dd62e"	"00075b1d24"	0.058
"00074dd62e"	"00075b202b"	0.0774
"00074dd62e"	"00075b22cb"	-0.0156
"00074dd62e"	"00075b22da"	-0.0865
"00074dd62e"	"00075b2556"	-0.1098
"00074dd62e"	"00075b25de"	-1e-04

"00074dd62e"	"00075b260c"	-0.0208
"00074dd62e"	"00075b26f1"	0.13
"00074dd62e"	"00075b2920"	0.0695
"00074dd62e"	"00075b2a64"	0.108
"00074dd62e"	"00075b2a9d"	-0.1542
"00074dd62e"	"00075b2b37"	-0.1348
"00074dd62e"	"00075b2cdd"	-0.0412
"00074dd62e"	"00075b3038"	0.0129
"00074dd62e"	"00075b30fe"	-0.1786
"00074dd62e"	"00075b3362"	0.1145
"00074dd62e"	"00075b350a"	-0.085
"00074dd62e"	"00075b350e"	0.0644
"00074dd62e"	"00075b3651"	0.1466
"00074dd62e"	"00075b38ca"	-0.3067
"00074dd62e"	"00075b39cc"	-0.0823
"00074dd62e"	"00075b3e1e"	-0.1274
"00074dd62e"	"00075b3e57"	0.2058
"00074dd62e"	"00075b4079"	-0.0668
"00074dd62e"	"00075b4150"	3e-04
"00074dd62e"	"00075b4194"	0.1237
"00074dd62e"	"00075b42d5"	-0.1163
"00074dd62e"	"00075b4424"	0.1274
"00074dd62e"	"00075b4470"	0.0134
"00074dd62e"	"00075b47ed"	-0.2292
"00074dd62e"	"00075b4850"	0.0614
"00074dd62e"	"00075b4ca0"	-0.2265
"00074dd62e"	"00075b4d7f"	0.2253
"00074dd62e"	"00075b520f"	-0.0946
"00074dd62e"	"00075b525f"	0.1317
"00074dd62e"	"00075b58f8"	-0.1852
"00074dd62e"	"00075b5bcc"	0.1122
"00074dd62e"	"00075b5bfa"	-0.126
"00074dd62e"	"00075b6339"	0.0432
"00074dd62e"	"00075b6658"	0.0877
"00074dd62e"	"00075b679a"	0.0781
"00074dd62e"	"00075b6cb7"	-0.1604
"00074dd62e"	"00075b6df8"	-0.0638
"00074dd62e"	"00075b6ff6"	0.1099
"00074dd62e"	"00075b70ee"	-0.1564
"00074dd62e"	"00075b7157"	0.023
"00074dd62e"	"00075b7225"	0.1451
"00074dd62e"	"00075b7c89"	-0.0324
"00074dd62e"	"00075b9048"	0.1171
"00074dd62e"	"00075d0801"	-0.039
"00074dd62e"	"00075d1820"	-0.0212
"00074dd62e"	"00075d1f3d"	0.0617
"00074dd62e"	"00075d2329"	-0.455
"00074dd62e"	"00075d2b9b"	0.0147
"00074dd62e"	"00075d3941"	0.044
"00074dd62e"	"00075d3e96"	0.2991

"00074dd62e"	"00075d4864"	-0.266
"00074dd62e"	"00075d5961"	-0.081
"00074dd62e"	"00075d5a63"	-0.0395
"00074dd62e"	"00075d6150"	0.2039
"00074dd62e"	"00075d67d0"	-0.0572
"00074dd62e"	"00075d67e2"	0.0087
"00074dd62e"	"00075d73fc"	-0.1598
"00074dd62e"	"00075d7729"	-0.196
"00074dd62e"	"00075d778c"	-0.1673
"00074dd62e"	"00075d7b9e"	0.188
"00074dd62e"	"00075d7c8f"	-0.0041
"00074dd62e"	"00075d804d"	0.0425
"00074dd62e"	"00075d819f"	-0.0198
"00074dd62e"	"00075d8601"	-0.0395
"00074dd62e"	"00075d8c6a"	0.0157
"00074dd62e"	"00075dfedc"	0.1853
"00074dd62e"	"00075e05f2"	0.0952
"00074dd62e"	"00075e0837"	-0.1644
"00074dd62e"	"00075e092e"	-0.1577
"00074dd62e"	"00075e0965"	-0.2912
"00074dd62e"	"00075e0bc8"	0.046
"00074dd62e"	"00075e0fbb"	-0.3984
"00074dd73c"	"00074dda10"	-0.0113
"00074dd73c"	"00074ddab8"	0.1021
"00074dd73c"	"00074ddd3d"	-0.2215
"00074dd73c"	"00074ddf16"	0.381
"00074dd73c"	"00074ddfc1"	0.4068
"00074dd73c"	"00074de21a"	-0.4125
"00074dd73c"	"00074de2a9"	6e-04
"00074dd73c"	"00074de544"	-0.1196
"00074dd73c"	"00074de98a"	0.0794
"00074dd73c"	"00074dea7e"	-0.0411
"00074dd73c"	"00074debd9"	0.0961
"00074dd73c"	"00074deca3"	0.1474
"00074dd73c"	"00074def43"	0.5915
"00074dd73c"	"00074def99"	-0.0653
"00074dd73c"	"00074ecdad"	-0.0314
"00074dd73c"	"00074ecf28"	-0.107
"00074dd73c"	"00074ed1e1"	0.0932
"00074dd73c"	"00074ed83b"	0.2313
"00074dd73c"	"00074ee5e3"	0.0356
"00074dd73c"	"00074ee6e0"	-0.2132
"00074dd73c"	"00074eea3a"	0.067
"00074dd73c"	"00074eff82"	0.0449
"00074dd73c"	"00074f0477"	0.0287
"00074dd73c"	"00074f08c3"	-0.2211
"00074dd73c"	"00074f1859"	-0.048
"00074dd73c"	"00074f2268"	-0.267
"00074dd73c"	"00074f28be"	0.1519
"00074dd73c"	"00074f294b"	0.0451

"00074dd73c"	"00074f2ddd"	0.0471
"00074dd73c"	"00074f2e75"	-0.102
"00074dd73c"	"00074f3088"	-0.1398
"00074dd73c"	"00074f5a1c"	0.3169
"00074dd73c"	"00074f75b7"	0.4623
"00074dd73c"	"00074f8cd9"	0.3272
"00074dd73c"	"00074f96dc"	-0.1069
"00074dd73c"	"00074fabaa"	-0.1953
"00074dd73c"	"00074facd9"	0.0203
"00074dd73c"	"00074fae3c"	-0.1371
"00074dd73c"	"00074fb0a8"	-0.3053
"00074dd73c"	"00074fb4e4"	-0.4281
"00074dd73c"	"00074fb7c2"	0.0205
"00074dd73c"	"00074fbd36"	-0.3228
"00074dd73c"	"00074fc27f"	0.1476
"00074dd73c"	"00074fc31d"	-0.2201
"00074dd73c"	"00074fd569"	0.0025
"00074dd73c"	"00074fef15"	0.0506
"00074dd73c"	"00074ff562"	-0.0634
"00074dd73c"	"00075007ca"	-0.1455
"00074dd73c"	"0007500b86"	0.2411
"00074dd73c"	"0007500d05"	0.1255
"00074dd73c"	"0007500ee4"	-0.1879
"00074dd73c"	"0007500eee"	0.0967
"00074dd73c"	"00075013dc"	-0.2118
"00074dd73c"	"000757b515"	0.1227
"00074dd73c"	"000757bc5a"	0.066
"00074dd73c"	"000757c320"	-0.2415
"00074dd73c"	"000757c9aa"	0.0231
"00074dd73c"	"000757ccbe"	0.0452
"00074dd73c"	"000757cfa9"	-0.2995
"00074dd73c"	"000757d390"	-0.5149
"00074dd73c"	"000757d393"	0.1625
"00074dd73c"	"000757d598"	-0.2655
"00074dd73c"	"000757d5a2"	-0.1366
"00074dd73c"	"000757d790"	-0.037
"00074dd73c"	"000757e30c"	-0.228
"00074dd73c"	"000757e4b0"	0.2675
"00074dd73c"	"000757e7a0"	0.0148
"00074dd73c"	"000757e8b3"	0.1553
"00074dd73c"	"000757f627"	-0.0997
"00074dd73c"	"000757f925"	0.0385
"00074dd73c"	"000757fa08"	0.0905
"00074dd73c"	"000757fe52"	0.0897
"00074dd73c"	"000758024a"	-0.161
"00074dd73c"	"00075804bb"	-0.1135
"00074dd73c"	"00075a0c04"	0.2577
"00074dd73c"	"00075a3110"	0.1097
"00074dd73c"	"00075a341a"	0.0098
"00074dd73c"	"00075a3dcf"	0.3923

"00074dd73c"	"00075a3e22"	0.1102
"00074dd73c"	"00075a48d8"	0.1561
"00074dd73c"	"00075a5cfb"	-0.1597
"00074dd73c"	"00075a6151"	-0.1102
"00074dd73c"	"00075a6708"	0.0554
"00074dd73c"	"00075a7319"	-0.4502
"00074dd73c"	"00075a7723"	0.299
"00074dd73c"	"00075a778b"	-0.2104
"00074dd73c"	"00075a7b8e"	-0.1901
"00074dd73c"	"00075a7c79"	0.2486
"00074dd73c"	"00075a81b6"	-0.0848
"00074dd73c"	"00075a82ac"	0.0371
"00074dd73c"	"00075a98e5"	-0.0608
"00074dd73c"	"00075b0d29"	-0.2203
"00074dd73c"	"00075b102a"	-0.1651
"00074dd73c"	"00075b1074"	-0.0536
"00074dd73c"	"00075b135d"	-0.2749
"00074dd73c"	"00075b138b"	0.2178
"00074dd73c"	"00075b13a0"	0.0247
"00074dd73c"	"00075b13bd"	-0.0308
"00074dd73c"	"00075b16a9"	-0.1093
"00074dd73c"	"00075b1a28"	-0.0452
"00074dd73c"	"00075b1a97"	0.0257
"00074dd73c"	"00075b1c7b"	-0.4422
"00074dd73c"	"00075b1d24"	0.1508
"00074dd73c"	"00075b202b"	0.0704
"00074dd73c"	"00075b22cb"	-0.0582
"00074dd73c"	"00075b22da"	0.1093
"00074dd73c"	"00075b2556"	-0.2018
"00074dd73c"	"00075b25de"	0.3119
"00074dd73c"	"00075b260c"	-7e-04
"00074dd73c"	"00075b26f1"	0.1313
"00074dd73c"	"00075b2920"	-0.1121
"00074dd73c"	"00075b2a64"	-0.1742
"00074dd73c"	"00075b2a9d"	-0.1592
"00074dd73c"	"00075b2b37"	-0.3364
"00074dd73c"	"00075b2cdd"	-0.1756
"00074dd73c"	"00075b3038"	0.4056
"00074dd73c"	"00075b30fe"	0.0749
"00074dd73c"	"00075b3362"	-0.196
"00074dd73c"	"00075b350a"	-0.1088
"00074dd73c"	"00075b350e"	-0.0836
"00074dd73c"	"00075b3651"	-0.0527
"00074dd73c"	"00075b38ca"	0.1177
"00074dd73c"	"00075b39cc"	-0.024
"00074dd73c"	"00075b3e1e"	0.1503
"00074dd73c"	"00075b3e57"	0.0203
"00074dd73c"	"00075b4079"	-0.0604
"00074dd73c"	"00075b4150"	-0.139
"00074dd73c"	"00075b4194"	0.2671

"00074dd73c"	"00075b42d5"	0.3967
"00074dd73c"	"00075b4424"	-0.1366
"00074dd73c"	"00075b4470"	0.066
"00074dd73c"	"00075b47ed"	0.0265
"00074dd73c"	"00075b4850"	0.1918
"00074dd73c"	"00075b4ca0"	-0.0879
"00074dd73c"	"00075b4d7f"	0.2624
"00074dd73c"	"00075b520f"	0.0604
"00074dd73c"	"00075b525f"	-0.0196
"00074dd73c"	"00075b58f8"	-0.3523
"00074dd73c"	"00075b5bcc"	0.1927
"00074dd73c"	"00075b5bfa"	0.082
"00074dd73c"	"00075b6339"	0.2939
"00074dd73c"	"00075b6658"	0.0273
"00074dd73c"	"00075b679a"	0.2996
"00074dd73c"	"00075b6cb7"	-0.1825
"00074dd73c"	"00075b6df8"	0.2179
"00074dd73c"	"00075b6ff6"	0.3258
"00074dd73c"	"00075b70ee"	-0.1879
"00074dd73c"	"00075b7157"	0.0142
"00074dd73c"	"00075b7225"	0.1472
"00074dd73c"	"00075b7c89"	-0.0056
"00074dd73c"	"00075b9048"	0.1603
"00074dd73c"	"00075d0801"	0.0898
"00074dd73c"	"00075d1820"	-0.3337
"00074dd73c"	"00075d1f3d"	0.1558
"00074dd73c"	"00075d2329"	0.2555
"00074dd73c"	"00075d2b9b"	-0.0474
"00074dd73c"	"00075d3941"	0.0545
"00074dd73c"	"00075d3e96"	-0.0577
"00074dd73c"	"00075d4864"	0.1023
"00074dd73c"	"00075d5961"	0.0561
"00074dd73c"	"00075d5a63"	0.2302
"00074dd73c"	"00075d6150"	-0.05
"00074dd73c"	"00075d67d0"	0.1287
"00074dd73c"	"00075d67e2"	-0.0256
"00074dd73c"	"00075d73fc"	0.2313
"00074dd73c"	"00075d7729"	-0.028
"00074dd73c"	"00075d778c"	0.1356
"00074dd73c"	"00075d7b9e"	-0.1014
"00074dd73c"	"00075d7c8f"	0.2332
"00074dd73c"	"00075d804d"	0.2357
"00074dd73c"	"00075d819f"	-0.1507
"00074dd73c"	"00075d8601"	-0.0411
"00074dd73c"	"00075d8c6a"	0.1188
"00074dd73c"	"00075dfedc"	0.1242
"00074dd73c"	"00075e05f2"	0.2129
"00074dd73c"	"00075e0837"	0.1283
"00074dd73c"	"00075e092e"	0.0964
"00074dd73c"	"00075e0965"	-0.176

"00074dd73c"	"00075e0bc8"	0.2936
"00074dd73c"	"00075e0fbb"	0.0265
"00074dda10"	"00074ddab8"	0.2721
"00074dda10"	"00074ddd3d"	−0.3722
"00074dda10"	"00074ddf16"	−0.1591
"00074dda10"	"00074ddfc1"	0.1054
"00074dda10"	"00074de21a"	0.0874
"00074dda10"	"00074de2a9"	0.0018
"00074dda10"	"00074de544"	0.711
"00074dda10"	"00074de98a"	0.5011
"00074dda10"	"00074dea7e"	−0.2557
"00074dda10"	"00074debd9"	−0.2103
"00074dda10"	"00074deca3"	−0.2203
"00074dda10"	"00074def43"	−0.198
"00074dda10"	"00074def99"	0.0395
"00074dda10"	"00074ecdad"	−0.5052
"00074dda10"	"00074ecf28"	−0.1358
"00074dda10"	"00074ed1e1"	−0.0548
"00074dda10"	"00074ed83b"	0.0121
"00074dda10"	"00074ee5e3"	−0.1414
"00074dda10"	"00074ee6e0"	0.1118
"00074dda10"	"00074eea3a"	0.0387
"00074dda10"	"00074eff82"	0.0454
"00074dda10"	"00074f0477"	−0.2653
"00074dda10"	"00074f08c3"	−0.1068
"00074dda10"	"00074f1859"	0.1077
"00074dda10"	"00074f2268"	−0.3186
"00074dda10"	"00074f28be"	0.0535
"00074dda10"	"00074f294b"	−0.1639
"00074dda10"	"00074f2ddd"	−0.1148
"00074dda10"	"00074f2e75"	−0.2222
"00074dda10"	"00074f3088"	0.0686
"00074dda10"	"00074f5a1c"	−0.1902
"00074dda10"	"00074f75b7"	−0.0739
"00074dda10"	"00074f8cd9"	0.2616
"00074dda10"	"00074f96dc"	0.0533
"00074dda10"	"00074fabaa"	0.0741
"00074dda10"	"00074facd9"	0.0764
"00074dda10"	"00074fae3c"	0.1666
"00074dda10"	"00074fb0a8"	−0.0786
"00074dda10"	"00074fb4e4"	−0.2256
"00074dda10"	"00074fb7c2"	0.3232
"00074dda10"	"00074fbd36"	−0.2168
"00074dda10"	"00074fc27f"	−0.2315
"00074dda10"	"00074fc31d"	0.0669
"00074dda10"	"00074fd569"	−0.0922
"00074dda10"	"00074fef15"	−0.01
"00074dda10"	"00074ff562"	−0.4683
"00074dda10"	"00075007ca"	−0.252
"00074dda10"	"0007500b86"	0.1995

"00074dda10"	"0007500d05"	0.0351
"00074dda10"	"0007500ee4"	-0.001
"00074dda10"	"0007500eee"	0.0711
"00074dda10"	"00075013dc"	-0.1637
"00074dda10"	"000757b515"	-0.0746
"00074dda10"	"000757bc5a"	0.295
"00074dda10"	"000757c320"	0.0165
"00074dda10"	"000757c9aa"	-0.0802
"00074dda10"	"000757ccbe"	-0.052
"00074dda10"	"000757cfa9"	-0.0579
"00074dda10"	"000757d390"	-0.335
"00074dda10"	"000757d393"	0.3135
"00074dda10"	"000757d598"	-0.5026
"00074dda10"	"000757d5a2"	-0.3438
"00074dda10"	"000757d790"	-0.0207
"00074dda10"	"000757e30c"	-0.2011
"00074dda10"	"000757e4b0"	0.0301
"00074dda10"	"000757e7a0"	-0.1846
"00074dda10"	"000757e8b3"	0.1034
"00074dda10"	"000757f627"	0.26
"00074dda10"	"000757f925"	0.1601
"00074dda10"	"000757fa08"	-0.1883
"00074dda10"	"000757fe52"	-0.1228
"00074dda10"	"000758024a"	-0.1235
"00074dda10"	"00075804bb"	-0.076
"00074dda10"	"00075a0c04"	0.1643
"00074dda10"	"00075a3110"	-0.1543
"00074dda10"	"00075a341a"	-0.1336
"00074dda10"	"00075a3dcf"	0.0151
"00074dda10"	"00075a3e22"	0.2541
"00074dda10"	"00075a48d8"	0.1313
"00074dda10"	"00075a5cfb"	-0.2619
"00074dda10"	"00075a6151"	-0.1593
"00074dda10"	"00075a6708"	-0.1372
"00074dda10"	"00075a7319"	-0.1518
"00074dda10"	"00075a7723"	0.3928
"00074dda10"	"00075a778b"	0.1251
"00074dda10"	"00075a7b8e"	0.1586
"00074dda10"	"00075a7c79"	0.1301
"00074dda10"	"00075a81b6"	0.2117
"00074dda10"	"00075a82ac"	0.2445
"00074dda10"	"00075a98e5"	0.5729
"00074dda10"	"00075b0d29"	0.1437
"00074dda10"	"00075b102a"	0.1014
"00074dda10"	"00075b1074"	-0.1675
"00074dda10"	"00075b135d"	0.1119
"00074dda10"	"00075b138b"	0.4305
"00074dda10"	"00075b13a0"	-0.3428
"00074dda10"	"00075b13bd"	0.1692
"00074dda10"	"00075b16a9"	-0.0513

"00074dda10"	"00075b1a28"	-0.0874
"00074dda10"	"00075b1a97"	0.136
"00074dda10"	"00075b1c7b"	-0.2102
"00074dda10"	"00075b1d24"	0.0912
"00074dda10"	"00075b202b"	0.0546
"00074dda10"	"00075b22cb"	-0.0518
"00074dda10"	"00075b22da"	-0.2646
"00074dda10"	"00075b2556"	-0.2287
"00074dda10"	"00075b25de"	0.1015
"00074dda10"	"00075b260c"	-0.3224
"00074dda10"	"00075b26f1"	0.063
"00074dda10"	"00075b2920"	-0.1339
"00074dda10"	"00075b2a64"	-0.0868
"00074dda10"	"00075b2a9d"	-7e-04
"00074dda10"	"00075b2b37"	-0.337
"00074dda10"	"00075b2cdd"	0.0915
"00074dda10"	"00075b3038"	0.0532
"00074dda10"	"00075b30fe"	-0.2616
"00074dda10"	"00075b3362"	-0.023
"00074dda10"	"00075b350a"	0.0232
"00074dda10"	"00075b350e"	-0.0962
"00074dda10"	"00075b3651"	0.034
"00074dda10"	"00075b38ca"	-0.0922
"00074dda10"	"00075b39cc"	-0.0545
"00074dda10"	"00075b3e1e"	0.1791
"00074dda10"	"00075b3e57"	0.102
"00074dda10"	"00075b4079"	-0.014
"00074dda10"	"00075b4150"	-0.2064
"00074dda10"	"00075b4194"	0.2097
"00074dda10"	"00075b42d5"	0.0087
"00074dda10"	"00075b4424"	-0.2991
"00074dda10"	"00075b4470"	0.2794
"00074dda10"	"00075b47ed"	-0.0172
"00074dda10"	"00075b4850"	0.0384
"00074dda10"	"00075b4ca0"	-0.0881
"00074dda10"	"00075b4d7f"	0.2979
"00074dda10"	"00075b520f"	-0.0378
"00074dda10"	"00075b525f"	-0.1931
"00074dda10"	"00075b58f8"	-0.2202
"00074dda10"	"00075b5bcc"	0.0827
"00074dda10"	"00075b5bfa"	-0.3973
"00074dda10"	"00075b6339"	0.0292
"00074dda10"	"00075b6658"	-0.046
"00074dda10"	"00075b679a"	0.0074
"00074dda10"	"00075b6cb7"	0.0805
"00074dda10"	"00075b6df8"	-0.0176
"00074dda10"	"00075b6ff6"	-0.18
"00074dda10"	"00075b70ee"	-0.1978
"00074dda10"	"00075b7157"	0.042
"00074dda10"	"00075b7225"	-0.158

"00074dda10"	"00075b7c89"	0.0916
"00074dda10"	"00075b9048"	−0.0696
"00074dda10"	"00075d0801"	−0.0126
"00074dda10"	"00075d1820"	−0.084
"00074dda10"	"00075d1f3d"	0.0808
"00074dda10"	"00075d2329"	0.1363
"00074dda10"	"00075d2b9b"	−0.1239
"00074dda10"	"00075d3941"	0.0249
"00074dda10"	"00075d3e96"	−0.1309
"00074dda10"	"00075d4864"	−0.1274
"00074dda10"	"00075d5961"	0.3083
"00074dda10"	"00075d5a63"	0.0903
"00074dda10"	"00075d6150"	−0.2269
"00074dda10"	"00075d67d0"	−0.1365
"00074dda10"	"00075d67e2"	0.4327
"00074dda10"	"00075d73fc"	0.0728
"00074dda10"	"00075d7729"	−0.2123
"00074dda10"	"00075d778c"	−0.2284
"00074dda10"	"00075d7b9e"	0.1909
"00074dda10"	"00075d7c8f"	0.0842
"00074dda10"	"00075d804d"	0.1945
"00074dda10"	"00075d819f"	0.1899
"00074dda10"	"00075d8601"	0.1709
"00074dda10"	"00075d8c6a"	−0.1665
"00074dda10"	"00075dfedc"	−0.0929
"00074dda10"	"00075e05f2"	−0.0496
"00074dda10"	"00075e0837"	−0.0229
"00074dda10"	"00075e092e"	0.0587
"00074dda10"	"00075e0965"	−0.3642
"00074dda10"	"00075e0bc8"	0.0606
"00074dda10"	"00075e0fbb"	−0.1766
"00074ddab8"	"00074ddd3d"	−0.3415
"00074ddab8"	"00074ddf16"	0.1006
"00074ddab8"	"00074ddfc1"	0.2826
"00074ddab8"	"00074de21a"	0.0158
"00074ddab8"	"00074de2a9"	0.1783
"00074ddab8"	"00074de544"	0.1715
"00074ddab8"	"00074de98a"	0.0634
"00074ddab8"	"00074dea7e"	0.1309
"00074ddab8"	"00074debd9"	0.0814
"00074ddab8"	"00074deca3"	−0.1918
"00074ddab8"	"00074def43"	0.0116
"00074ddab8"	"00074def99"	−0.0383
"00074ddab8"	"00074ecdad"	−0.2518
"00074ddab8"	"00074ecf28"	−0.3192
"00074ddab8"	"00074ed1e1"	−0.0551
"00074ddab8"	"00074ed83b"	0.0165
"00074ddab8"	"00074ee5e3"	0.2134
"00074ddab8"	"00074ee6e0"	0.11
"00074ddab8"	"00074eea3a"	0.2868

"00074ddab8"	"00074eff82"	−0.0561
"00074ddab8"	"00074f0477"	0.0258
"00074ddab8"	"00074f08c3"	0.2176
"00074ddab8"	"00074f1859"	0.1852
"00074ddab8"	"00074f2268"	0.1146
"00074ddab8"	"00074f28be"	0.2415
"00074ddab8"	"00074f294b"	−0.076
"00074ddab8"	"00074f2ddd"	−0.0791
"00074ddab8"	"00074f2e75"	−0.2415
"00074ddab8"	"00074f3088"	0.0062
"00074ddab8"	"00074f5a1c"	0.0066
"00074ddab8"	"00074f75b7"	0.023
"00074ddab8"	"00074f8cd9"	0.1004
"00074ddab8"	"00074f96dc"	0.073
"00074ddab8"	"00074fabaa"	0.3742
"00074ddab8"	"00074facd9"	0.3487
"00074ddab8"	"00074fae3c"	−0.0151
"00074ddab8"	"00074fb0a8"	0.0339
"00074ddab8"	"00074fb4e4"	−0.2565
"00074ddab8"	"00074fb7c2"	0.1797
"00074ddab8"	"00074fbd36"	−0.1259
"00074ddab8"	"00074fc27f"	0.0452
"00074ddab8"	"00074fc31d"	−0.0879
"00074ddab8"	"00074fd569"	−0.0822
"00074ddab8"	"00074fef15"	−0.094
"00074ddab8"	"00074ff562"	−0.2596
"00074ddab8"	"00075007ca"	−0.091
"00074ddab8"	"0007500b86"	0.2448
"00074ddab8"	"0007500d05"	0.0652
"00074ddab8"	"0007500ee4"	−0.1081
"00074ddab8"	"0007500eee"	0.2312
"00074ddab8"	"00075013dc"	0.087
"00074ddab8"	"000757b515"	0.1592
"00074ddab8"	"000757bc5a"	0.5638
"00074ddab8"	"000757c320"	−0.056
"00074ddab8"	"000757c9aa"	0.2368
"00074ddab8"	"000757ccbe"	3e−04
"00074ddab8"	"000757cfa9"	−0.2671
"00074ddab8"	"000757d390"	−0.278
"00074ddab8"	"000757d393"	0.0721
"00074ddab8"	"000757d598"	−0.1629
"00074ddab8"	"000757d5a2"	−0.1473
"00074ddab8"	"000757d790"	−0.0263
"00074ddab8"	"000757e30c"	0.0998
"00074ddab8"	"000757e4b0"	0.6018
"00074ddab8"	"000757e7a0"	−0.0421
"00074ddab8"	"000757e8b3"	0.2073
"00074ddab8"	"000757f627"	0.1796
"00074ddab8"	"000757f925"	0.1168
"00074ddab8"	"000757fa08"	−0.1889

"00074ddab8"	"000757fe52"	-0.0602
"00074ddab8"	"000758024a"	-0.1256
"00074ddab8"	"00075804bb"	0.1792
"00074ddab8"	"00075a0c04"	0.1183
"00074ddab8"	"00075a3110"	0.2703
"00074ddab8"	"00075a341a"	-0.2987
"00074ddab8"	"00075a3dcf"	0.0083
"00074ddab8"	"00075a3e22"	0.2244
"00074ddab8"	"00075a48d8"	-0.0014
"00074ddab8"	"00075a5cfb"	0.0674
"00074ddab8"	"00075a6151"	-0.0812
"00074ddab8"	"00075a6708"	0.131
"00074ddab8"	"00075a7319"	-0.2081
"00074ddab8"	"00075a7723"	-0.1179
"00074ddab8"	"00075a778b"	-0.0818
"00074ddab8"	"00075a7b8e"	0.3101
"00074ddab8"	"00075a7c79"	0.3347
"00074ddab8"	"00075a81b6"	0.0839
"00074ddab8"	"00075a82ac"	0.2073
"00074ddab8"	"00075a98e5"	-0.0073
"00074ddab8"	"00075b0d29"	-0.0923
"00074ddab8"	"00075b102a"	0.531
"00074ddab8"	"00075b1074"	0.1244
"00074ddab8"	"00075b135d"	-0.1434
"00074ddab8"	"00075b138b"	0.6702
"00074ddab8"	"00075b13a0"	-0.2217
"00074ddab8"	"00075b13bd"	0.0817
"00074ddab8"	"00075b16a9"	-0.2185
"00074ddab8"	"00075b1a28"	-0.2104
"00074ddab8"	"00075b1a97"	0.0822
"00074ddab8"	"00075b1c7b"	-0.4423
"00074ddab8"	"00075b1d24"	0.0685
"00074ddab8"	"00075b202b"	0.4645
"00074ddab8"	"00075b22cb"	-0.0282
"00074ddab8"	"00075b22da"	0.194
"00074ddab8"	"00075b2556"	-0.0053
"00074ddab8"	"00075b25de"	0.0783
"00074ddab8"	"00075b260c"	0.113
"00074ddab8"	"00075b26f1"	0.0476
"00074ddab8"	"00075b2920"	0.0946
"00074ddab8"	"00075b2a64"	0.1876
"00074ddab8"	"00075b2a9d"	0.5322
"00074ddab8"	"00075b2b37"	-0.4235
"00074ddab8"	"00075b2cdd"	0.0643
"00074ddab8"	"00075b3038"	0.1797
"00074ddab8"	"00075b30fe"	0.0742
"00074ddab8"	"00075b3362"	-0.2374
"00074ddab8"	"00075b350a"	0.2157
"00074ddab8"	"00075b350e"	0.0571
"00074ddab8"	"00075b3651"	-0.0095

"00074ddab8"	"00075b38ca"	-0.0729
"00074ddab8"	"00075b39cc"	0.0569
"00074ddab8"	"00075b3e1e"	0.2273
"00074ddab8"	"00075b3e57"	-0.0173
"00074ddab8"	"00075b4079"	-0.1413
"00074ddab8"	"00075b4150"	-0.1847
"00074ddab8"	"00075b4194"	0.3953
"00074ddab8"	"00075b42d5"	0.1869
"00074ddab8"	"00075b4424"	-0.1588
"00074ddab8"	"00075b4470"	0.5979
"00074ddab8"	"00075b47ed"	0.0972
"00074ddab8"	"00075b4850"	0.6503
"00074ddab8"	"00075b4ca0"	0.1397
"00074ddab8"	"00075b4d7f"	0.1026
"00074ddab8"	"00075b520f"	-0.0506
"00074ddab8"	"00075b525f"	-0.1635
"00074ddab8"	"00075b58f8"	-0.016
"00074ddab8"	"00075b5bcc"	0.1384
"00074ddab8"	"00075b5bfa"	-0.154
"00074ddab8"	"00075b6339"	0.0765
"00074ddab8"	"00075b6658"	-0.2034
"00074ddab8"	"00075b679a"	0.0033
"00074ddab8"	"00075b6cb7"	0.5093
"00074ddab8"	"00075b6df8"	-0.2189
"00074ddab8"	"00075b6ff6"	0.2189
"00074ddab8"	"00075b70ee"	-0.1324
"00074ddab8"	"00075b7157"	0.0263
"00074ddab8"	"00075b7225"	0.036
"00074ddab8"	"00075b7c89"	0.0723
"00074ddab8"	"00075b9048"	0.2093
"00074ddab8"	"00075d0801"	0.0586
"00074ddab8"	"00075d1820"	-0.1966
"00074ddab8"	"00075d1f3d"	0.591
"00074ddab8"	"00075d2329"	0.1051
"00074ddab8"	"00075d2b9b"	0.3447
"00074ddab8"	"00075d3941"	0.1404
"00074ddab8"	"00075d3e96"	-0.0305
"00074ddab8"	"00075d4864"	-0.1589
"00074ddab8"	"00075d5961"	-0.1722
"00074ddab8"	"00075d5a63"	0.3983
"00074ddab8"	"00075d6150"	-0.1642
"00074ddab8"	"00075d67d0"	0.0896
"00074ddab8"	"00075d67e2"	0.1165
"00074ddab8"	"00075d73fc"	0.0728
"00074ddab8"	"00075d7729"	-0.1813
"00074ddab8"	"00075d778c"	-0.4005
"00074ddab8"	"00075d7b9e"	-0.059
"00074ddab8"	"00075d7c8f"	0.1697
"00074ddab8"	"00075d804d"	0.4241
"00074ddab8"	"00075d819f"	0.6945

"00074ddab8"	"00075d8601"	-0.1158
"00074ddab8"	"00075d8c6a"	-0.1812
"00074ddab8"	"00075dfedc"	0.055
"00074ddab8"	"00075e05f2"	0.1548
"00074ddab8"	"00075e0837"	0.5892
"00074ddab8"	"00075e092e"	0.164
"00074ddab8"	"00075e0965"	-0.2355
"00074ddab8"	"00075e0bc8"	0.564
"00074ddab8"	"00075e0fbb"	-0.046
"00074ddd3d"	"00074ddf16"	-0.2017
"00074ddd3d"	"00074ddfc1"	-0.2516
"00074ddd3d"	"00074de21a"	-0.4147
"00074ddd3d"	"00074de2a9"	-0.1327
"00074ddd3d"	"00074de544"	-0.1809
"00074ddd3d"	"00074de98a"	-0.2729
"00074ddd3d"	"00074dea7e"	-0.1049
"00074ddd3d"	"00074debd9"	-0.2744
"00074ddd3d"	"00074deca3"	-0.1978
"00074ddd3d"	"00074def43"	-0.1734
"00074ddd3d"	"00074def99"	-0.076
"00074ddd3d"	"00074ecdad"	0.0079
"00074ddd3d"	"00074ecf28"	-0.078
"00074ddd3d"	"00074ed1e1"	-0.1499
"00074ddd3d"	"00074ed83b"	0.1249
"00074ddd3d"	"00074ee5e3"	-0.3259
"00074ddd3d"	"00074ee6e0"	-0.1375
"00074ddd3d"	"00074eea3a"	-0.1581
"00074ddd3d"	"00074eff82"	-0.193
"00074ddd3d"	"00074f0477"	-0.2711
"00074ddd3d"	"00074f08c3"	-0.5229
"00074ddd3d"	"00074f1859"	-0.354
"00074ddd3d"	"00074f2268"	-0.1312
"00074ddd3d"	"00074f28be"	-0.1669
"00074ddd3d"	"00074f294b"	0.1452
"00074ddd3d"	"00074f2ddd"	0.3251
"00074ddd3d"	"00074f2e75"	-0.099
"00074ddd3d"	"00074f3088"	-0.0337
"00074ddd3d"	"00074f5a1c"	-0.2538
"00074ddd3d"	"00074f75b7"	-0.1155
"00074ddd3d"	"00074f8cd9"	-0.1915
"00074ddd3d"	"00074f96dc"	-0.4817
"00074ddd3d"	"00074fabaa"	-0.4292
"00074ddd3d"	"00074facd9"	-0.3473
"00074ddd3d"	"00074fae3c"	-0.2995
"00074ddd3d"	"00074fb0a8"	-0.2758
"00074ddd3d"	"00074fb4e4"	-0.2705
"00074ddd3d"	"00074fb7c2"	0.0613
"00074ddd3d"	"00074fbd36"	-0.0174
"00074ddd3d"	"00074fc27f"	0.0891
"00074ddd3d"	"00074fc31d"	0.0343

"00074ddd3d"	"00074fd569"	-0.0563
"00074ddd3d"	"00074fef15"	-0.2582
"00074ddd3d"	"00074ff562"	-0.427
"00074ddd3d"	"00075007ca"	0.0293
"00074ddd3d"	"0007500b86"	-0.2567
"00074ddd3d"	"0007500d05"	-0.2123
"00074ddd3d"	"0007500ee4"	0.0846
"00074ddd3d"	"0007500eee"	-0.4446
"00074ddd3d"	"00075013dc"	-0.2347
"00074ddd3d"	"000757b515"	0.1815
"00074ddd3d"	"000757bc5a"	-0.1435
"00074ddd3d"	"000757c320"	-0.045
"00074ddd3d"	"000757c9aa"	0.0176
"00074ddd3d"	"000757ccbe"	-0.1032
"00074ddd3d"	"000757cfa9"	0.0146
"00074ddd3d"	"000757d390"	-0.1579
"00074ddd3d"	"000757d393"	-0.0734
"00074ddd3d"	"000757d598"	0.5408
"00074ddd3d"	"000757d5a2"	0.5921
"00074ddd3d"	"000757d790"	0.0406
"00074ddd3d"	"000757e30c"	0.4271
"00074ddd3d"	"000757e4b0"	-0.1724
"00074ddd3d"	"000757e7a0"	0.2757
"00074ddd3d"	"000757e8b3"	-0.0147
"00074ddd3d"	"000757f627"	-0.238
"00074ddd3d"	"000757f925"	0.1702
"00074ddd3d"	"000757fa08"	-0.1064
"00074ddd3d"	"000757fe52"	-0.1742
"00074ddd3d"	"000758024a"	-0.0471
"00074ddd3d"	"00075804bb"	0.1862
"00074ddd3d"	"00075a0c04"	-0.197
"00074ddd3d"	"00075a3110"	0.0472
"00074ddd3d"	"00075a341a"	0.0695
"00074ddd3d"	"00075a3dcf"	-0.1375
"00074ddd3d"	"00075a3e22"	-0.3257
"00074ddd3d"	"00075a48d8"	-0.1419
"00074ddd3d"	"00075a5cfb"	0.0536
"00074ddd3d"	"00075a6151"	0.0095
"00074ddd3d"	"00075a6708"	0.2213
"00074ddd3d"	"00075a7319"	-0.1336
"00074ddd3d"	"00075a7723"	-0.0554
"00074ddd3d"	"00075a778b"	-0.0021
"00074ddd3d"	"00075a7b8e"	-0.2504
"00074ddd3d"	"00075a7c79"	-0.0224
"00074ddd3d"	"00075a81b6"	-0.2407
"00074ddd3d"	"00075a82ac"	-0.2803
"00074ddd3d"	"00075a98e5"	-0.4666
"00074ddd3d"	"00075b0d29"	-0.1216
"00074ddd3d"	"00075b102a"	-0.1191
"00074ddd3d"	"00075b1074"	-0.2571

"00074ddd3d"	"00075b135d"	−0.1619
"00074ddd3d"	"00075b138b"	−0.2017
"00074ddd3d"	"00075b13a0"	−0.4243
"00074ddd3d"	"00075b13bd"	−0.3996
"00074ddd3d"	"00075b16a9"	0.2779
"00074ddd3d"	"00075b1a28"	−0.0772
"00074ddd3d"	"00075b1a97"	−0.0913
"00074ddd3d"	"00075b1c7b"	0.0969
"00074ddd3d"	"00075b1d24"	0.0279
"00074ddd3d"	"00075b202b"	−0.2194
"00074ddd3d"	"00075b22cb"	−0.0576
"00074ddd3d"	"00075b22da"	−0.1374
"00074ddd3d"	"00075b2556"	0.0086
"00074ddd3d"	"00075b25de"	0.0095
"00074ddd3d"	"00075b260c"	0.0497
"00074ddd3d"	"00075b26f1"	−0.1531
"00074ddd3d"	"00075b2920"	0.1862
"00074ddd3d"	"00075b2a64"	−0.1139
"00074ddd3d"	"00075b2a9d"	−0.2327
"00074ddd3d"	"00075b2b37"	−0.1333
"00074ddd3d"	"00075b2cdd"	−0.2465
"00074ddd3d"	"00075b3038"	0.0217
"00074ddd3d"	"00075b30fe"	−0.0851
"00074ddd3d"	"00075b3362"	−0.1583
"00074ddd3d"	"00075b350a"	−0.2408
"00074ddd3d"	"00075b350e"	−0.3512
"00074ddd3d"	"00075b3651"	−0.0423
"00074ddd3d"	"00075b38ca"	−0.4527
"00074ddd3d"	"00075b39cc"	−0.1639
"00074ddd3d"	"00075b3e1e"	−0.2876
"00074ddd3d"	"00075b3e57"	0.0403
"00074ddd3d"	"00075b4079"	−0.2098
"00074ddd3d"	"00075b4150"	0.2798
"00074ddd3d"	"00075b4194"	−0.2277
"00074ddd3d"	"00075b42d5"	−0.2873
"00074ddd3d"	"00075b4424"	0.161
"00074ddd3d"	"00075b4470"	−0.1435
"00074ddd3d"	"00075b47ed"	−0.3348
"00074ddd3d"	"00075b4850"	−0.0424
"00074ddd3d"	"00075b4ca0"	−0.1032
"00074ddd3d"	"00075b4d7f"	−0.0331
"00074ddd3d"	"00075b520f"	−0.1441
"00074ddd3d"	"00075b525f"	0.0469
"00074ddd3d"	"00075b58f8"	−0.0604
"00074ddd3d"	"00075b5bcc"	−0.1399
"00074ddd3d"	"00075b5bfa"	−0.1586
"00074ddd3d"	"00075b6339"	0.1751
"00074ddd3d"	"00075b6658"	−0.2602
"00074ddd3d"	"00075b679a"	0.2338
"00074ddd3d"	"00075b6cb7"	−0.2019

"00074ddd3d"	"00075b6df8"	0.0611
"00074ddd3d"	"00075b6ff6"	0.2384
"00074ddd3d"	"00075b70ee"	0.4906
"00074ddd3d"	"00075b7157"	−0.0223
"00074ddd3d"	"00075b7225"	−0.0625
"00074ddd3d"	"00075b7c89"	0.0038
"00074ddd3d"	"00075b9048"	0.2087
"00074ddd3d"	"00075d0801"	−0.1844
"00074ddd3d"	"00075d1820"	0.0267
"00074ddd3d"	"00075d1f3d"	−0.2872
"00074ddd3d"	"00075d2329"	−0.2631
"00074ddd3d"	"00075d2b9b"	−0.3947
"00074ddd3d"	"00075d3941"	−0.4199
"00074ddd3d"	"00075d3e96"	0.1881
"00074ddd3d"	"00075d4864"	−0.2308
"00074ddd3d"	"00075d5961"	−0.1322
"00074ddd3d"	"00075d5a63"	−0.1909
"00074ddd3d"	"00075d6150"	−0.0171
"00074ddd3d"	"00075d67d0"	−0.2442
"00074ddd3d"	"00075d67e2"	−0.1623
"00074ddd3d"	"00075d73fc"	−0.0374
"00074ddd3d"	"00075d7729"	−0.1484
"00074ddd3d"	"00075d778c"	−0.0988
"00074ddd3d"	"00075d7b9e"	−0.0084
"00074ddd3d"	"00075d7c8f"	−0.1497
"00074ddd3d"	"00075d804d"	−0.331
"00074ddd3d"	"00075d819f"	−0.1179
"00074ddd3d"	"00075d8601"	−0.2469
"00074ddd3d"	"00075d8c6a"	−0.1708
"00074ddd3d"	"00075dfedc"	0.0405
"00074ddd3d"	"00075e05f2"	−0.0977
"00074ddd3d"	"00075e0837"	−0.0476
"00074ddd3d"	"00075e092e"	−0.0975
"00074ddd3d"	"00075e0965"	−0.0828
"00074ddd3d"	"00075e0bc8"	−0.142
"00074ddd3d"	"00075e0fbb"	−0.2152
"00074ddf16"	"00074ddfc1"	0.4508
"00074ddf16"	"00074de21a"	−0.2664
"00074ddf16"	"00074de2a9"	−0.0526
"00074ddf16"	"00074de544"	−0.1522
"00074ddf16"	"00074de98a"	0.0128
"00074ddf16"	"00074dea7e"	0.2024
"00074ddf16"	"00074debd9"	0.1405
"00074ddf16"	"00074deca3"	0.1461
"00074ddf16"	"00074def43"	0.5571
"00074ddf16"	"00074def99"	−0.2182
"00074ddf16"	"00074ecdad"	−0.0503
"00074ddf16"	"00074ecf28"	−0.2866
"00074ddf16"	"00074ed1e1"	0.1603
"00074ddf16"	"00074ed83b"	0.2466

"00074ddf16"	"00074ee5e3"	0.1753
"00074ddf16"	"00074ee6e0"	-0.2751
"00074ddf16"	"00074eea3a"	0.1089
"00074ddf16"	"00074eff82"	-0.0546
"00074ddf16"	"00074f0477"	0.0801
"00074ddf16"	"00074f08c3"	-0.2591
"00074ddf16"	"00074f1859"	0.0462
"00074ddf16"	"00074f2268"	-0.3742
"00074ddf16"	"00074f28be"	0.2299
"00074ddf16"	"00074f294b"	0.2155
"00074ddf16"	"00074f2ddd"	-0.1207
"00074ddf16"	"00074f2e75"	-0.3481
"00074ddf16"	"00074f3088"	0.0207
"00074ddf16"	"00074f5a1c"	0.2014
"00074ddf16"	"00074f75b7"	0.2698
"00074ddf16"	"00074f8cd9"	0.3382
"00074ddf16"	"00074f96dc"	-0.4886
"00074ddf16"	"00074fabaa"	-0.0046
"00074ddf16"	"00074facd9"	0.1556
"00074ddf16"	"00074fae3c"	-0.2445
"00074ddf16"	"00074fb0a8"	-0.0618
"00074ddf16"	"00074fb4e4"	-0.0259
"00074ddf16"	"00074fb7c2"	0.0031
"00074ddf16"	"00074fbd36"	-0.2542
"00074ddf16"	"00074fc27f"	0.235
"00074ddf16"	"00074fc31d"	-0.1429
"00074ddf16"	"00074fd569"	-0.0938
"00074ddf16"	"00074fef15"	0.0133
"00074ddf16"	"00074ff562"	-0.1264
"00074ddf16"	"00075007ca"	-0.0181
"00074ddf16"	"0007500b86"	0.414
"00074ddf16"	"0007500d05"	0.2009
"00074ddf16"	"0007500ee4"	-0.0352
"00074ddf16"	"0007500eee"	0.1324
"00074ddf16"	"00075013dc"	-0.1162
"00074ddf16"	"000757b515"	0.1879
"00074ddf16"	"000757bc5a"	-0.1098
"00074ddf16"	"000757c320"	-0.0506
"00074ddf16"	"000757c9aa"	0.0921
"00074ddf16"	"000757ccbe"	-0.0593
"00074ddf16"	"000757cfa9"	-0.2314
"00074ddf16"	"000757d390"	-0.2319
"00074ddf16"	"000757d393"	0.3209
"00074ddf16"	"000757d598"	-0.0969
"00074ddf16"	"000757d5a2"	-0.0388
"00074ddf16"	"000757d790"	0.1207
"00074ddf16"	"000757e30c"	-0.1558
"00074ddf16"	"000757e4b0"	0.1733
"00074ddf16"	"000757e7a0"	-0.0041
"00074ddf16"	"000757e8b3"	0.0615

"00074ddf16"	"000757f627"	0.2597
"00074ddf16"	"000757f925"	0.2965
"00074ddf16"	"000757fa08"	−0.1006
"00074ddf16"	"000757fe52"	−0.0072
"00074ddf16"	"000758024a"	−0.1501
"00074ddf16"	"00075804bb"	−0.0656
"00074ddf16"	"00075a0c04"	0.1347
"00074ddf16"	"00075a3110"	0.0624
"00074ddf16"	"00075a341a"	−0.0587
"00074ddf16"	"00075a3dcf"	0.1777
"00074ddf16"	"00075a3e22"	0.0042
"00074ddf16"	"00075a48d8"	0.0015
"00074ddf16"	"00075a5cfb"	−0.1462
"00074ddf16"	"00075a6151"	0.0283
"00074ddf16"	"00075a6708"	−0.1172
"00074ddf16"	"00075a7319"	−0.1624
"00074ddf16"	"00075a7723"	0.1848
"00074ddf16"	"00075a778b"	−0.2067
"00074ddf16"	"00075a7b8e"	0.0899
"00074ddf16"	"00075a7c79"	0.1796
"00074ddf16"	"00075a81b6"	−0.2416
"00074ddf16"	"00075a82ac"	0.148
"00074ddf16"	"00075a98e5"	−0.0189
"00074ddf16"	"00075b0d29"	−0.1392
"00074ddf16"	"00075b102a"	0.1136
"00074ddf16"	"00075b1074"	−0.2154
"00074ddf16"	"00075b135d"	−0.3569
"00074ddf16"	"00075b138b"	0.2817
"00074ddf16"	"00075b13a0"	−0.0769
"00074ddf16"	"00075b13bd"	−0.1334
"00074ddf16"	"00075b16a9"	0.1753
"00074ddf16"	"00075b1a28"	−0.3846
"00074ddf16"	"00075b1a97"	0.097
"00074ddf16"	"00075b1c7b"	−0.3815
"00074ddf16"	"00075b1d24"	−0.0802
"00074ddf16"	"00075b202b"	−0.0889
"00074ddf16"	"00075b22cb"	0.0561
"00074ddf16"	"00075b22da"	0.0454
"00074ddf16"	"00075b2556"	−0.0684
"00074ddf16"	"00075b25de"	0.1751
"00074ddf16"	"00075b260c"	0.1379
"00074ddf16"	"00075b26f1"	−0.0705
"00074ddf16"	"00075b2920"	0.0545
"00074ddf16"	"00075b2a64"	−0.0612
"00074ddf16"	"00075b2a9d"	0.008
"00074ddf16"	"00075b2b37"	−0.4372
"00074ddf16"	"00075b2cdd"	−0.168
"00074ddf16"	"00075b3038"	0.3238
"00074ddf16"	"00075b30fe"	0.0603
"00074ddf16"	"00075b3362"	−0.3122

"00074ddf16"	"00075b350a"	−0.2233
"00074ddf16"	"00075b350e"	−0.13
"00074ddf16"	"00075b3651"	0.0281
"00074ddf16"	"00075b38ca"	0.0654
"00074ddf16"	"00075b39cc"	0.0853
"00074ddf16"	"00075b3e1e"	0.2599
"00074ddf16"	"00075b3e57"	−0.1208
"00074ddf16"	"00075b4079"	−0.1543
"00074ddf16"	"00075b4150"	−0.2192
"00074ddf16"	"00075b4194"	0.3137
"00074ddf16"	"00075b42d5"	0.4291
"00074ddf16"	"00075b4424"	−0.2386
"00074ddf16"	"00075b4470"	−0.1098
"00074ddf16"	"00075b47ed"	−0.0081
"00074ddf16"	"00075b4850"	0.1716
"00074ddf16"	"00075b4ca0"	−0.2628
"00074ddf16"	"00075b4d7f"	0.137
"00074ddf16"	"00075b520f"	−0.1614
"00074ddf16"	"00075b525f"	−0.12
"00074ddf16"	"00075b58f8"	−0.249
"00074ddf16"	"00075b5bcc"	−0.0063
"00074ddf16"	"00075b5bfa"	−0.0157
"00074ddf16"	"00075b6339"	0.141
"00074ddf16"	"00075b6658"	−0.2538
"00074ddf16"	"00075b679a"	0.0764
"00074ddf16"	"00075b6cb7"	−0.0534
"00074ddf16"	"00075b6df8"	0.0727
"00074ddf16"	"00075b6ff6"	0.2288
"00074ddf16"	"00075b70ee"	−0.1347
"00074ddf16"	"00075b7157"	−0.0674
"00074ddf16"	"00075b7225"	−0.1235
"00074ddf16"	"00075b7c89"	−0.12
"00074ddf16"	"00075b9048"	0.186
"00074ddf16"	"00075d0801"	0.1219
"00074ddf16"	"00075d1820"	−0.1815
"00074ddf16"	"00075d1f3d"	0.1445
"00074ddf16"	"00075d2329"	0.0651
"00074ddf16"	"00075d2b9b"	0.0336
"00074ddf16"	"00075d3941"	−0.3596
"00074ddf16"	"00075d3e96"	−0.164
"00074ddf16"	"00075d4864"	−0.1764
"00074ddf16"	"00075d5961"	0.1512
"00074ddf16"	"00075d5a63"	0.0538
"00074ddf16"	"00075d6150"	−0.0047
"00074ddf16"	"00075d67d0"	−0.1124
"00074ddf16"	"00075d67e2"	−0.0758
"00074ddf16"	"00075d73fc"	−0.0349
"00074ddf16"	"00075d7729"	−0.3243
"00074ddf16"	"00075d778c"	−0.115
"00074ddf16"	"00075d7b9e"	−0.0935

"00074ddf16"	"00075d7c8f"	0.1489
"00074ddf16"	"00075d804d"	0.1659
"00074ddf16"	"00075d819f"	−0.035
"00074ddf16"	"00075d8601"	−0.0432
"00074ddf16"	"00075d8c6a"	0.2129
"00074ddf16"	"00075dfedc"	0.2303
"00074ddf16"	"00075e05f2"	0.197
"00074ddf16"	"00075e0837"	0.1753
"00074ddf16"	"00075e092e"	−0.0112
"00074ddf16"	"00075e0965"	−0.2472
"00074ddf16"	"00075e0bc8"	0.1661
"00074ddf16"	"00075e0fbb"	−0.0223
"00074ddfc1"	"00074de21a"	−0.2449
"00074ddfc1"	"00074de2a9"	0.0233
"00074ddfc1"	"00074de544"	−0.0242
"00074ddfc1"	"00074de98a"	0.193
"00074ddfc1"	"00074dea7e"	0.1845
"00074ddfc1"	"00074debd9"	0.2363
"00074ddfc1"	"00074deca3"	0.2746
"00074ddfc1"	"00074def43"	0.4107
"00074ddfc1"	"00074def99"	−0.2515
"00074ddfc1"	"00074ecdad"	0.0158
"00074ddfc1"	"00074ecf28"	−0.3313
"00074ddfc1"	"00074ed1e1"	0.114
"00074ddfc1"	"00074ed83b"	0.1341
"00074ddfc1"	"00074ee5e3"	0.1346
"00074ddfc1"	"00074ee6e0"	−0.2917
"00074ddfc1"	"00074eea3a"	0.1233
"00074ddfc1"	"00074eff82"	−0.1093
"00074ddfc1"	"00074f0477"	−0.0496
"00074ddfc1"	"00074f08c3"	−0.1904
"00074ddfc1"	"00074f1859"	0.3955
"00074ddfc1"	"00074f2268"	−0.0903
"00074ddfc1"	"00074f28be"	0.2233
"00074ddfc1"	"00074f294b"	0.1128
"00074ddfc1"	"00074f2ddd"	−0.1506
"00074ddfc1"	"00074f2e75"	−0.12
"00074ddfc1"	"00074f3088"	−0.1502
"00074ddfc1"	"00074f5a1c"	0.3177
"00074ddfc1"	"00074f75b7"	0.2584
"00074ddfc1"	"00074f8cd9"	0.4388
"00074ddfc1"	"00074f96dc"	−0.2846
"00074ddfc1"	"00074fabaa"	0.1296
"00074ddfc1"	"00074facd9"	0.2916
"00074ddfc1"	"00074fae3c"	0.1235
"00074ddfc1"	"00074fb0a8"	−0.2112
"00074ddfc1"	"00074fb4e4"	−0.1831
"00074ddfc1"	"00074fb7c2"	0.0638
"00074ddfc1"	"00074fbd36"	−0.1035
"00074ddfc1"	"00074fc27f"	0.145

"00074ddfc1"	"00074fc31d"	−0.1881
"00074ddfc1"	"00074fd569"	−0.2886
"00074ddfc1"	"00074fef15"	−0.1687
"00074ddfc1"	"00074ff562"	0.1114
"00074ddfc1"	"00075007ca"	0.1026
"00074ddfc1"	"0007500b86"	0.421
"00074ddfc1"	"0007500d05"	0.2383
"00074ddfc1"	"0007500ee4"	−0.0196
"00074ddfc1"	"0007500eee"	0.301
"00074ddfc1"	"00075013dc"	−0.1374
"00074ddfc1"	"000757b515"	0.0406
"00074ddfc1"	"000757bc5a"	0.1868
"00074ddfc1"	"000757c320"	0.1405
"00074ddfc1"	"000757c9aa"	0.105
"00074ddfc1"	"000757ccbe"	−0.2108
"00074ddfc1"	"000757cfa9"	−0.0836
"00074ddfc1"	"000757d390"	−0.3113
"00074ddfc1"	"000757d393"	0.3528
"00074ddfc1"	"000757d598"	−0.2764
"00074ddfc1"	"000757d5a2"	−0.1795
"00074ddfc1"	"000757d790"	−0.0227
"00074ddfc1"	"000757e30c"	−0.1008
"00074ddfc1"	"000757e4b0"	0.3497
"00074ddfc1"	"000757e7a0"	−0.044
"00074ddfc1"	"000757e8b3"	0.1542
"00074ddfc1"	"000757f627"	0.1458
"00074ddfc1"	"000757f925"	0.1396
"00074ddfc1"	"000757fa08"	0.0974
"00074ddfc1"	"000757fe52"	−0.0639
"00074ddfc1"	"000758024a"	−0.1005
"00074ddfc1"	"00075804bb"	0.1982
"00074ddfc1"	"00075a0c04"	0.1505
"00074ddfc1"	"00075a3110"	−0.0931
"00074ddfc1"	"00075a341a"	−0.0875
"00074ddfc1"	"00075a3dcf"	0.2028
"00074ddfc1"	"00075a3e22"	0.0842
"00074ddfc1"	"00075a48d8"	−0.0202
"00074ddfc1"	"00075a5cfb"	−0.0839
"00074ddfc1"	"00075a6151"	0.2117
"00074ddfc1"	"00075a6708"	−0.0954
"00074ddfc1"	"00075a7319"	−0.2267
"00074ddfc1"	"00075a7723"	0.2019
"00074ddfc1"	"00075a778b"	−0.0969
"00074ddfc1"	"00075a7b8e"	0.0985
"00074ddfc1"	"00075a7c79"	0.0942
"00074ddfc1"	"00075a81b6"	−0.0088
"00074ddfc1"	"00075a82ac"	0.0023
"00074ddfc1"	"00075a98e5"	0.1777
"00074ddfc1"	"00075b0d29"	0.0137
"00074ddfc1"	"00075b102a"	0.2331

"00074ddfc1"	"00075b1074"	-0.0062
"00074ddfc1"	"00075b135d"	-0.3171
"00074ddfc1"	"00075b138b"	0.289
"00074ddfc1"	"00075b13a0"	-0.2417
"00074ddfc1"	"00075b13bd"	-0.1617
"00074ddfc1"	"00075b16a9"	-0.0067
"00074ddfc1"	"00075b1a28"	-0.0477
"00074ddfc1"	"00075b1a97"	0.0172
"00074ddfc1"	"00075b1c7b"	-0.3121
"00074ddfc1"	"00075b1d24"	-0.2353
"00074ddfc1"	"00075b202b"	0.1614
"00074ddfc1"	"00075b22cb"	0.1153
"00074ddfc1"	"00075b22da"	0.1582
"00074ddfc1"	"00075b2556"	0.0587
"00074ddfc1"	"00075b25de"	0.2383
"00074ddfc1"	"00075b260c"	0.0807
"00074ddfc1"	"00075b26f1"	0.1217
"00074ddfc1"	"00075b2920"	0.0143
"00074ddfc1"	"00075b2a64"	-0.1468
"00074ddfc1"	"00075b2a9d"	0.1623
"00074ddfc1"	"00075b2b37"	-0.2827
"00074ddfc1"	"00075b2cdd"	0.022
"00074ddfc1"	"00075b3038"	0.3476
"00074ddfc1"	"00075b30fe"	0.105
"00074ddfc1"	"00075b3362"	-0.4731
"00074ddfc1"	"00075b350a"	0.068
"00074ddfc1"	"00075b350e"	-0.0462
"00074ddfc1"	"00075b3651"	-0.1446
"00074ddfc1"	"00075b38ca"	0.2835
"00074ddfc1"	"00075b39cc"	0.2874
"00074ddfc1"	"00075b3e1e"	0.5422
"00074ddfc1"	"00075b3e57"	-0.0769
"00074ddfc1"	"00075b4079"	-0.0845
"00074ddfc1"	"00075b4150"	-0.1022
"00074ddfc1"	"00075b4194"	0.3082
"00074ddfc1"	"00075b42d5"	0.4467
"00074ddfc1"	"00075b4424"	-0.0976
"00074ddfc1"	"00075b4470"	0.2623
"00074ddfc1"	"00075b47ed"	0.2321
"00074ddfc1"	"00075b4850"	0.1707
"00074ddfc1"	"00075b4ca0"	-0.1803
"00074ddfc1"	"00075b4d7f"	0.2113
"00074ddfc1"	"00075b520f"	0.0456
"00074ddfc1"	"00075b525f"	-0.1856
"00074ddfc1"	"00075b58f8"	-0.0569
"00074ddfc1"	"00075b5bcc"	0.0263
"00074ddfc1"	"00075b5bfa"	-0.1453
"00074ddfc1"	"00075b6339"	0.2129
"00074ddfc1"	"00075b6658"	-0.2414
"00074ddfc1"	"00075b679a"	0.0511

"00074ddfc1"	"00075b6cb7"	-0.0013
"00074ddfc1"	"00075b6df8"	0.0342
"00074ddfc1"	"00075b6ff6"	0.0055
"00074ddfc1"	"00075b70ee"	-0.0882
"00074ddfc1"	"00075b7157"	-0.1223
"00074ddfc1"	"00075b7225"	-0.1314
"00074ddfc1"	"00075b7c89"	-0.1111
"00074ddfc1"	"00075b9048"	0.0369
"00074ddfc1"	"00075d0801"	0.2337
"00074ddfc1"	"00075d1820"	-0.3487
"00074ddfc1"	"00075d1f3d"	0.0267
"00074ddfc1"	"00075d2329"	0.3821
"00074ddfc1"	"00075d2b9b"	0.0889
"00074ddfc1"	"00075d3941"	0.1014
"00074ddfc1"	"00075d3e96"	-0.1579
"00074ddfc1"	"00075d4864"	0.1194
"00074ddfc1"	"00075d5961"	0.111
"00074ddfc1"	"00075d5a63"	0.2435
"00074ddfc1"	"00075d6150"	-0.0278
"00074ddfc1"	"00075d67d0"	-0.1427
"00074ddfc1"	"00075d67e2"	0.0449
"00074ddfc1"	"00075d73fc"	-0.0416
"00074ddfc1"	"00075d7729"	-0.3188
"00074ddfc1"	"00075d778c"	-0.1673
"00074ddfc1"	"00075d7b9e"	0.0534
"00074ddfc1"	"00075d7c8f"	0.0408
"00074ddfc1"	"00075d804d"	0.3432
"00074ddfc1"	"00075d819f"	0.1165
"00074ddfc1"	"00075d8601"	0.1887
"00074ddfc1"	"00075d8c6a"	0.1803
"00074ddfc1"	"00075dfedc"	0.2786
"00074ddfc1"	"00075e05f2"	0.3082
"00074ddfc1"	"00075e0837"	0.3514
"00074ddfc1"	"00075e092e"	0.0801
"00074ddfc1"	"00075e0965"	-0.1444
"00074ddfc1"	"00075e0bc8"	0.2079
"00074ddfc1"	"00075e0fbb"	0.1473
"00074de21a"	"00074de2a9"	0.1114
"00074de21a"	"00074de544"	0.0839
"00074de21a"	"00074de98a"	0.1042
"00074de21a"	"00074dea7e"	-0.0764
"00074de21a"	"00074debd9"	-0.7418
"00074de21a"	"00074deca3"	-0.3177
"00074de21a"	"00074def43"	-0.3506
"00074de21a"	"00074def99"	-0.0248
"00074de21a"	"00074ecdad"	-0.3395
"00074de21a"	"00074ecf28"	-0.2561
"00074de21a"	"00074ed1e1"	-0.2093
"00074de21a"	"00074ed83b"	0.122
"00074de21a"	"00074ee5e3"	-0.3537

"00074de21a"	"00074ee6e0"	-0.176
"00074de21a"	"00074eea3a"	-2e-04
"00074de21a"	"00074eff82"	-0.2217
"00074de21a"	"00074f0477"	-0.5825
"00074de21a"	"00074f08c3"	0.5343
"00074de21a"	"00074f1859"	-0.2431
"00074de21a"	"00074f2268"	-0.2546
"00074de21a"	"00074f28be"	0.2668
"00074de21a"	"00074f294b"	0.0489
"00074de21a"	"00074f2ddd"	-0.3063
"00074de21a"	"00074f2e75"	-0.5288
"00074de21a"	"00074f3088"	-0.0275
"00074de21a"	"00074f5a1c"	-0.1777
"00074de21a"	"00074f75b7"	-0.4618
"00074de21a"	"00074f8cd9"	-0.4378
"00074de21a"	"00074f96dc"	-0.0896
"00074de21a"	"00074fabaa"	0.1031
"00074de21a"	"00074facd9"	-0.2477
"00074de21a"	"00074fae3c"	0.0224
"00074de21a"	"00074fb0a8"	0.2482
"00074de21a"	"00074fb4e4"	0.3066
"00074de21a"	"00074fb7c2"	0.0738
"00074de21a"	"00074fbd36"	0.0935
"00074de21a"	"00074fc27f"	-0.2427
"00074de21a"	"00074fc31d"	0.0885
"00074de21a"	"00074fd569"	-0.1565
"00074de21a"	"00074fef15"	-0.0781
"00074de21a"	"00074ff562"	-0.3522
"00074de21a"	"00075007ca"	-0.5209
"00074de21a"	"0007500b86"	-0.0782
"00074de21a"	"0007500d05"	-0.5153
"00074de21a"	"0007500ee4"	-0.2661
"00074de21a"	"0007500eee"	-0.5133
"00074de21a"	"00075013dc"	0.0623
"00074de21a"	"000757b515"	-0.2307
"00074de21a"	"000757bc5a"	0.1909
"00074de21a"	"000757c320"	0.192
"00074de21a"	"000757c9aa"	0.2312
"00074de21a"	"000757ccbe"	-0.0919
"00074de21a"	"000757cfa9"	-0.0712
"00074de21a"	"000757d390"	0.2478
"00074de21a"	"000757d393"	-0.1506
"00074de21a"	"000757d598"	-0.2193
"00074de21a"	"000757d5a2"	-0.4687
"00074de21a"	"000757d790"	-0.1725
"00074de21a"	"000757e30c"	-0.1815
"00074de21a"	"000757e4b0"	0.2267
"00074de21a"	"000757e7a0"	-0.294
"00074de21a"	"000757e8b3"	0.0606
"00074de21a"	"000757f627"	0.3927

"00074de21a"	"000757f925"	−0.2899
"00074de21a"	"000757fa08"	−0.5547
"00074de21a"	"000757fe52"	−0.2146
"00074de21a"	"000758024a"	−0.2547
"00074de21a"	"00075804bb"	0.059
"00074de21a"	"00075a0c04"	−0.2303
"00074de21a"	"00075a3110"	−0.271
"00074de21a"	"00075a341a"	−0.2714
"00074de21a"	"00075a3dcf"	−0.2317
"00074de21a"	"00075a3e22"	0.0679
"00074de21a"	"00075a48d8"	−0.2885
"00074de21a"	"00075a5cfb"	−0.0779
"00074de21a"	"00075a6151"	−0.0974
"00074de21a"	"00075a6708"	−0.2016
"00074de21a"	"00075a7319"	0.2332
"00074de21a"	"00075a7723"	0.0321
"00074de21a"	"00075a778b"	−0.0449
"00074de21a"	"00075a7b8e"	0.0303
"00074de21a"	"00075a7c79"	0.0124
"00074de21a"	"00075a81b6"	0.0877
"00074de21a"	"00075a82ac"	0.0526
"00074de21a"	"00075a98e5"	0.1281
"00074de21a"	"00075b0d29"	−0.0625
"00074de21a"	"00075b102a"	−0.2258
"00074de21a"	"00075b1074"	−0.2417
"00074de21a"	"00075b135d"	0.1149
"00074de21a"	"00075b138b"	0.1391
"00074de21a"	"00075b13a0"	−0.2473
"00074de21a"	"00075b13bd"	0.2461
"00074de21a"	"00075b16a9"	−0.2574
"00074de21a"	"00075b1a28"	−0.3233
"00074de21a"	"00075b1a97"	0.1106
"00074de21a"	"00075b1c7b"	−0.4998
"00074de21a"	"00075b1d24"	−0.1972
"00074de21a"	"00075b202b"	−0.1707
"00074de21a"	"00075b22cb"	−0.2492
"00074de21a"	"00075b22da"	−0.1588
"00074de21a"	"00075b2556"	0.0158
"00074de21a"	"00075b25de"	−0.1423
"00074de21a"	"00075b260c"	−0.1899
"00074de21a"	"00075b26f1"	−0.1768
"00074de21a"	"00075b2920"	−0.0379
"00074de21a"	"00075b2a64"	−0.1547
"00074de21a"	"00075b2a9d"	−0.2683
"00074de21a"	"00075b2b37"	−0.3941
"00074de21a"	"00075b2cdd"	−0.1939
"00074de21a"	"00075b3038"	−0.1491
"00074de21a"	"00075b30fe"	−0.1851
"00074de21a"	"00075b3362"	−0.1194
"00074de21a"	"00075b350a"	0.0165

"00074de21a"	"00075b350e"	-0.1454
"00074de21a"	"00075b3651"	-0.2062
"00074de21a"	"00075b38ca"	-0.2143
"00074de21a"	"00075b39cc"	-0.1317
"00074de21a"	"00075b3e1e"	-0.1778
"00074de21a"	"00075b3e57"	-0.2967
"00074de21a"	"00075b4079"	-0.1097
"00074de21a"	"00075b4150"	-0.3264
"00074de21a"	"00075b4194"	0.0547
"00074de21a"	"00075b42d5"	-0.169
"00074de21a"	"00075b4424"	-0.2901
"00074de21a"	"00075b4470"	0.1909
"00074de21a"	"00075b47ed"	-0.0901
"00074de21a"	"00075b4850"	0.2433
"00074de21a"	"00075b4ca0"	0.0241
"00074de21a"	"00075b4d7f"	-0.1059
"00074de21a"	"00075b520f"	-0.0045
"00074de21a"	"00075b525f"	-3e-04
"00074de21a"	"00075b58f8"	-0.312
"00074de21a"	"00075b5bcc"	0.0622
"00074de21a"	"00075b5bfa"	-0.3796
"00074de21a"	"00075b6339"	-0.0286
"00074de21a"	"00075b6658"	-0.219
"00074de21a"	"00075b679a"	0.0027
"00074de21a"	"00075b6cb7"	-0.2106
"00074de21a"	"00075b6df8"	-0.3149
"00074de21a"	"00075b6ff6"	-0.1747
"00074de21a"	"00075b70ee"	-0.1135
"00074de21a"	"00075b7157"	-0.1813
"00074de21a"	"00075b7225"	-0.2568
"00074de21a"	"00075b7c89"	-0.1498
"00074de21a"	"00075b9048"	-0.0869
"00074de21a"	"00075d0801"	-0.1148
"00074de21a"	"00075d1820"	-0.0917
"00074de21a"	"00075d1f3d"	0.1152
"00074de21a"	"00075d2329"	-0.2263
"00074de21a"	"00075d2b9b"	-0.0182
"00074de21a"	"00075d3941"	-0.1844
"00074de21a"	"00075d3e96"	-0.0636
"00074de21a"	"00075d4864"	-0.0969
"00074de21a"	"00075d5961"	-0.2092
"00074de21a"	"00075d5a63"	-0.1934
"00074de21a"	"00075d6150"	0.0383
"00074de21a"	"00075d67d0"	-0.3031
"00074de21a"	"00075d67e2"	0.1701
"00074de21a"	"00075d73fc"	-0.1931
"00074de21a"	"00075d7729"	-0.0168
"00074de21a"	"00075d778c"	-0.3605
"00074de21a"	"00075d7b9e"	-0.1158
"00074de21a"	"00075d7c8f"	-0.1541

"00074de21a"	"00075d804d"	0.0537
"00074de21a"	"00075d819f"	-0.0779
"00074de21a"	"00075d8601"	0.0904
"00074de21a"	"00075d8c6a"	-0.1571
"00074de21a"	"00075dfedc"	-0.1738
"00074de21a"	"00075e05f2"	-0.354
"00074de21a"	"00075e0837"	-0.1986
"00074de21a"	"00075e092e"	-0.2043
"00074de21a"	"00075e0965"	-0.2777
"00074de21a"	"00075e0bc8"	-0.0461
"00074de21a"	"00075e0fbb"	-0.3352
"00074de2a9"	"00074de544"	0.0774
"00074de2a9"	"00074de98a"	0.1901
"00074de2a9"	"00074dea7e"	-0.2893
"00074de2a9"	"00074debd9"	-0.0276
"00074de2a9"	"00074deca3"	-0.1373
"00074de2a9"	"00074def43"	-0.0397
"00074de2a9"	"00074def99"	-0.1636
"00074de2a9"	"00074ecdad"	-0.1517
"00074de2a9"	"00074ecf28"	0.0177
"00074de2a9"	"00074ed1e1"	-0.1426
"00074de2a9"	"00074ed83b"	0.3805
"00074de2a9"	"00074ee5e3"	-0.1053
"00074de2a9"	"00074ee6e0"	-0.0677
"00074de2a9"	"00074eea3a"	0.1282
"00074de2a9"	"00074eff82"	-0.0832
"00074de2a9"	"00074f0477"	-0.2715
"00074de2a9"	"00074f08c3"	0.2749
"00074de2a9"	"00074f1859"	0.3117
"00074de2a9"	"00074f2268"	-0.0163
"00074de2a9"	"00074f28be"	0.236
"00074de2a9"	"00074f294b"	-0.1357
"00074de2a9"	"00074f2ddd"	-0.3243
"00074de2a9"	"00074f2e75"	-0.2304
"00074de2a9"	"00074f3088"	-0.0033
"00074de2a9"	"00074f5a1c"	0.0316
"00074de2a9"	"00074f75b7"	-0.1124
"00074de2a9"	"00074f8cd9"	-0.1166
"00074de2a9"	"00074f96dc"	-0.1156
"00074de2a9"	"00074fabaa"	0.1762
"00074de2a9"	"00074facd9"	0.0624
"00074de2a9"	"00074fae3c"	-0.0944
"00074de2a9"	"00074fb0a8"	0.1031
"00074de2a9"	"00074fb4e4"	-0.0835
"00074de2a9"	"00074fb7c2"	0.1099
"00074de2a9"	"00074fbd36"	-0.003
"00074de2a9"	"00074fc27f"	0.57
"00074de2a9"	"00074fc31d"	-0.0455
"00074de2a9"	"00074fd569"	0.0695
"00074de2a9"	"00074fef15"	-0.0755

"00074de2a9"	"00074ff562"	−0.3605
"00074de2a9"	"00075007ca"	−0.006
"00074de2a9"	"0007500b86"	0.0648
"00074de2a9"	"0007500d05"	−0.0834
"00074de2a9"	"0007500ee4"	−0.1209
"00074de2a9"	"0007500eee"	−0.1906
"00074de2a9"	"00075013dc"	0.015
"00074de2a9"	"000757b515"	0.0101
"00074de2a9"	"000757bc5a"	0.1539
"00074de2a9"	"000757c320"	0.0683
"00074de2a9"	"000757c9aa"	0.2962
"00074de2a9"	"000757ccbe"	0.195
"00074de2a9"	"000757cfa9"	0.0748
"00074de2a9"	"000757d390"	−0.1359
"00074de2a9"	"000757d393"	0.1051
"00074de2a9"	"000757d598"	0.1345
"00074de2a9"	"000757d5a2"	0.0232
"00074de2a9"	"000757d790"	−0.1072
"00074de2a9"	"000757e30c"	0.1082
"00074de2a9"	"000757e4b0"	0.4367
"00074de2a9"	"000757e7a0"	−0.0192
"00074de2a9"	"000757e8b3"	0.3965
"00074de2a9"	"000757f627"	0.1402
"00074de2a9"	"000757f925"	0.1206
"00074de2a9"	"000757fa08"	−0.1605
"00074de2a9"	"000757fe52"	0.1702
"00074de2a9"	"000758024a"	0.3065
"00074de2a9"	"00075804bb"	0.155
"00074de2a9"	"00075a0c04"	0.0075
"00074de2a9"	"00075a3110"	0.1241
"00074de2a9"	"00075a341a"	−0.0356
"00074de2a9"	"00075a3dcf"	−0.2542
"00074de2a9"	"00075a3e22"	0.2332
"00074de2a9"	"00075a48d8"	0.0499
"00074de2a9"	"00075a5cfb"	0.4328
"00074de2a9"	"00075a6151"	−0.0919
"00074de2a9"	"00075a6708"	−0.2046
"00074de2a9"	"00075a7319"	−0.1224
"00074de2a9"	"00075a7723"	−0.0378
"00074de2a9"	"00075a778b"	0.033
"00074de2a9"	"00075a7b8e"	0.0885
"00074de2a9"	"00075a7c79"	0.0577
"00074de2a9"	"00075a81b6"	0.013
"00074de2a9"	"00075a82ac"	0.1562
"00074de2a9"	"00075a98e5"	0.0097
"00074de2a9"	"00075b0d29"	0.0643
"00074de2a9"	"00075b102a"	0.0195
"00074de2a9"	"00075b1074"	−0.1231
"00074de2a9"	"00075b135d"	−0.0241
"00074de2a9"	"00075b138b"	0.4776

"00074de2a9"	"00075b13a0"	-0.0575
"00074de2a9"	"00075b13bd"	-0.0166
"00074de2a9"	"00075b16a9"	-0.061
"00074de2a9"	"00075b1a28"	-0.1421
"00074de2a9"	"00075b1a97"	-0.0304
"00074de2a9"	"00075b1c7b"	-0.0521
"00074de2a9"	"00075b1d24"	-0.1262
"00074de2a9"	"00075b202b"	0.2954
"00074de2a9"	"00075b22cb"	-0.053
"00074de2a9"	"00075b22da"	0.1159
"00074de2a9"	"00075b2556"	0.1198
"00074de2a9"	"00075b25de"	-0.1838
"00074de2a9"	"00075b260c"	0.2281
"00074de2a9"	"00075b26f1"	-0.18
"00074de2a9"	"00075b2920"	0.3225
"00074de2a9"	"00075b2a64"	0.2774
"00074de2a9"	"00075b2a9d"	0.0195
"00074de2a9"	"00075b2b37"	-0.1193
"00074de2a9"	"00075b2cdd"	-0.0481
"00074de2a9"	"00075b3038"	-0.0742
"00074de2a9"	"00075b30fe"	0.0086
"00074de2a9"	"00075b3362"	-0.2717
"00074de2a9"	"00075b350a"	0.1059
"00074de2a9"	"00075b350e"	-0.0091
"00074de2a9"	"00075b3651"	-0.3956
"00074de2a9"	"00075b38ca"	-0.1483
"00074de2a9"	"00075b39cc"	-0.2517
"00074de2a9"	"00075b3e1e"	0.1983
"00074de2a9"	"00075b3e57"	0.1447
"00074de2a9"	"00075b4079"	-0.0644
"00074de2a9"	"00075b4150"	0.3136
"00074de2a9"	"00075b4194"	0.1346
"00074de2a9"	"00075b42d5"	-0.0282
"00074de2a9"	"00075b4424"	0.1663
"00074de2a9"	"00075b4470"	0.1383
"00074de2a9"	"00075b47ed"	-0.3174
"00074de2a9"	"00075b4850"	0.1752
"00074de2a9"	"00075b4ca0"	-0.0682
"00074de2a9"	"00075b4d7f"	0.1695
"00074de2a9"	"00075b520f"	-0.0742
"00074de2a9"	"00075b525f"	-0.0962
"00074de2a9"	"00075b58f8"	0.2133
"00074de2a9"	"00075b5bcc"	0.0088
"00074de2a9"	"00075b5bfa"	-0.2968
"00074de2a9"	"00075b6339"	0.263
"00074de2a9"	"00075b6658"	-0.3528
"00074de2a9"	"00075b679a"	0.0326
"00074de2a9"	"00075b6cb7"	-0.0506
"00074de2a9"	"00075b6df8"	-0.1876
"00074de2a9"	"00075b6ff6"	0.3304

"00074de2a9"	"00075b70ee"	−0.0026
"00074de2a9"	"00075b7157"	0.0098
"00074de2a9"	"00075b7225"	0.041
"00074de2a9"	"00075b7c89"	−0.0813
"00074de2a9"	"00075b9048"	0.2265
"00074de2a9"	"00075d0801"	−0.1412
"00074de2a9"	"00075d1820"	−0.1957
"00074de2a9"	"00075d1f3d"	0.4309
"00074de2a9"	"00075d2329"	−0.2149
"00074de2a9"	"00075d2b9b"	0.2318
"00074de2a9"	"00075d3941"	−0.1706
"00074de2a9"	"00075d3e96"	−0.0278
"00074de2a9"	"00075d4864"	−0.0149
"00074de2a9"	"00075d5961"	0.0677
"00074de2a9"	"00075d5a63"	0.3288
"00074de2a9"	"00075d6150"	−0.2941
"00074de2a9"	"00075d67d0"	−0.0184
"00074de2a9"	"00075d67e2"	0.3344
"00074de2a9"	"00075d73fc"	−0.1468
"00074de2a9"	"00075d7729"	0.0229
"00074de2a9"	"00075d778c"	−0.3072
"00074de2a9"	"00075d7b9e"	−0.0112
"00074de2a9"	"00075d7c8f"	0.2104
"00074de2a9"	"00075d804d"	0.3757
"00074de2a9"	"00075d819f"	−0.0187
"00074de2a9"	"00075d8601"	−0.0632
"00074de2a9"	"00075d8c6a"	−0.0394
"00074de2a9"	"00075dfedc"	−0.0602
"00074de2a9"	"00075e05f2"	0.1189
"00074de2a9"	"00075e0837"	0.002
"00074de2a9"	"00075e092e"	−0.1186
"00074de2a9"	"00075e0965"	0.2599
"00074de2a9"	"00075e0bc8"	0.3492
"00074de2a9"	"00075e0fbb"	0.0109
"00074de544"	"00074de98a"	0.5081
"00074de544"	"00074dea7e"	−0.168
"00074de544"	"00074debd9"	−0.3494
"00074de544"	"00074deca3"	0.0261
"00074de544"	"00074def43"	−0.2314
"00074de544"	"00074def99"	0.1829
"00074de544"	"00074ecdad"	−0.4268
"00074de544"	"00074ecf28"	−0.2423
"00074de544"	"00074ed1e1"	0.0771
"00074de544"	"00074ed83b"	0.1739
"00074de544"	"00074ee5e3"	−0.2202
"00074de544"	"00074ee6e0"	0.1683
"00074de544"	"00074eea3a"	0.2623
"00074de544"	"00074eff82"	0.1082
"00074de544"	"00074f0477"	−0.2588
"00074de544"	"00074f08c3"	−0.259

"00074de544"	"00074f1859"	0.1346
"00074de544"	"00074f2268"	-0.0227
"00074de544"	"00074f28be"	0.142
"00074de544"	"00074f294b"	-0.0112
"00074de544"	"00074f2ddd"	0.099
"00074de544"	"00074f2e75"	-0.066
"00074de544"	"00074f3088"	0.0817
"00074de544"	"00074f5a1c"	-0.0563
"00074de544"	"00074f75b7"	-0.0193
"00074de544"	"00074f8cd9"	0.132
"00074de544"	"00074f96dc"	-0.0021
"00074de544"	"00074fabaa"	0.1548
"00074de544"	"00074facd9"	-0.0397
"00074de544"	"00074fae3c"	-0.0298
"00074de544"	"00074fb0a8"	0.0193
"00074de544"	"00074fb4e4"	-0.2975
"00074de544"	"00074fb7c2"	0.5388
"00074de544"	"00074fbd36"	0.1246
"00074de544"	"00074fc27f"	-0.0146
"00074de544"	"00074fc31d"	0.2206
"00074de544"	"00074fd569"	-0.028
"00074de544"	"00074fef15"	0.1276
"00074de544"	"00074ff562"	-0.4191
"00074de544"	"00075007ca"	-0.2829
"00074de544"	"0007500b86"	0.1493
"00074de544"	"0007500d05"	0.0761
"00074de544"	"0007500ee4"	0.0487
"00074de544"	"0007500eee"	-0.0919
"00074de544"	"00075013dc"	-0.1202
"00074de544"	"000757b515"	0.0844
"00074de544"	"000757bc5a"	0.2443
"00074de544"	"000757c320"	-0.2056
"00074de544"	"000757c9aa"	-0.1479
"00074de544"	"000757ccbe"	0.178
"00074de544"	"000757cfa9"	0.0785
"00074de544"	"000757d390"	-0.3349
"00074de544"	"000757d393"	0.2922
"00074de544"	"000757d598"	-0.3148
"00074de544"	"000757d5a2"	-0.2042
"00074de544"	"000757d790"	0.2145
"00074de544"	"000757e30c"	-0.2128
"00074de544"	"000757e4b0"	0.0914
"00074de544"	"000757e7a0"	-0.183
"00074de544"	"000757e8b3"	0.3088
"00074de544"	"000757f627"	0.1769
"00074de544"	"000757f925"	0.0945
"00074de544"	"000757fa08"	-0.0898
"00074de544"	"000757fe52"	0.0439
"00074de544"	"000758024a"	-0.2683
"00074de544"	"00075804bb"	-0.257

"00074de544"	"00075a0c04"	0.3196
"00074de544"	"00075a3110"	0.1064
"00074de544"	"00075a341a"	0.0993
"00074de544"	"00075a3dcf"	0.1521
"00074de544"	"00075a3e22"	0.4171
"00074de544"	"00075a48d8"	0.2465
"00074de544"	"00075a5cfb"	-0.1847
"00074de544"	"00075a6151"	-0.01
"00074de544"	"00075a6708"	-0.0661
"00074de544"	"00075a7319"	-0.2147
"00074de544"	"00075a7723"	0.2295
"00074de544"	"00075a778b"	0.1671
"00074de544"	"00075a7b8e"	0.0252
"00074de544"	"00075a7c79"	0.3444
"00074de544"	"00075a81b6"	0.2548
"00074de544"	"00075a82ac"	0.4194
"00074de544"	"00075a98e5"	0.4466
"00074de544"	"00075b0d29"	0.3009
"00074de544"	"00075b102a"	0.1193
"00074de544"	"00075b1074"	-0.063
"00074de544"	"00075b135d"	0.4624
"00074de544"	"00075b138b"	0.4365
"00074de544"	"00075b13a0"	-0.1786
"00074de544"	"00075b13bd"	0.2118
"00074de544"	"00075b16a9"	0.0018
"00074de544"	"00075b1a28"	-0.0261
"00074de544"	"00075b1a97"	0.26
"00074de544"	"00075b1c7b"	-0.1699
"00074de544"	"00075b1d24"	0.1358
"00074de544"	"00075b202b"	0.0613
"00074de544"	"00075b22cb"	0.0647
"00074de544"	"00075b22da"	-0.0873
"00074de544"	"00075b2556"	-0.0118
"00074de544"	"00075b25de"	0.0394
"00074de544"	"00075b260c"	-0.1203
"00074de544"	"00075b26f1"	-0.0026
"00074de544"	"00075b2920"	-0.22
"00074de544"	"00075b2a64"	-0.1273
"00074de544"	"00075b2a9d"	-0.1049
"00074de544"	"00075b2b37"	-0.4315
"00074de544"	"00075b2cdd"	0.2685
"00074de544"	"00075b3038"	0.0698
"00074de544"	"00075b30fe"	-0.0635
"00074de544"	"00075b3362"	0.0506
"00074de544"	"00075b350a"	-0.0374
"00074de544"	"00075b350e"	0.1476
"00074de544"	"00075b3651"	0.0827
"00074de544"	"00075b38ca"	-0.0648
"00074de544"	"00075b39cc"	0.0522
"00074de544"	"00075b3e1e"	0.1011

"00074de544"	"00075b3e57"	0.0173
"00074de544"	"00075b4079"	-0.1717
"00074de544"	"00075b4150"	-0.1784
"00074de544"	"00075b4194"	0.1754
"00074de544"	"00075b42d5"	0.0349
"00074de544"	"00075b4424"	-0.1942
"00074de544"	"00075b4470"	0.2287
"00074de544"	"00075b47ed"	0.05
"00074de544"	"00075b4850"	0.0528
"00074de544"	"00075b4ca0"	0.1876
"00074de544"	"00075b4d7f"	0.4753
"00074de544"	"00075b520f"	0.0042
"00074de544"	"00075b525f"	-0.0848
"00074de544"	"00075b58f8"	-0.1495
"00074de544"	"00075b5bcc"	-0.0281
"00074de544"	"00075b5bfa"	-0.2781
"00074de544"	"00075b6339"	0.1335
"00074de544"	"00075b6658"	0.0689
"00074de544"	"00075b679a"	0.082
"00074de544"	"00075b6cb7"	0.0275
"00074de544"	"00075b6df8"	0.1552
"00074de544"	"00075b6ff6"	-0.118
"00074de544"	"00075b70ee"	-0.1161
"00074de544"	"00075b7157"	0.1605
"00074de544"	"00075b7225"	0.0905
"00074de544"	"00075b7c89"	0.3269
"00074de544"	"00075b9048"	0.2217
"00074de544"	"00075d0801"	-0.0631
"00074de544"	"00075d1820"	-0.0463
"00074de544"	"00075d1f3d"	0.0864
"00074de544"	"00075d2329"	0.1451
"00074de544"	"00075d2b9b"	-0.1225
"00074de544"	"00075d3941"	-0.0217
"00074de544"	"00075d3e96"	-0.096
"00074de544"	"00075d4864"	-0.1621
"00074de544"	"00075d5961"	0.4302
"00074de544"	"00075d5a63"	-0.0116
"00074de544"	"00075d6150"	-0.0506
"00074de544"	"00075d67d0"	-0.2886
"00074de544"	"00075d67e2"	0.5322
"00074de544"	"00075d73fc"	0.2388
"00074de544"	"00075d7729"	-0.0955
"00074de544"	"00075d778c"	-0.199
"00074de544"	"00075d7b9e"	0.2239
"00074de544"	"00075d7c8f"	0.3242
"00074de544"	"00075d804d"	0.0338
"00074de544"	"00075d819f"	0.1693
"00074de544"	"00075d8601"	0.0444
"00074de544"	"00075d8c6a"	-0.0091
"00074de544"	"00075dfedc"	0.1454

"00074de544"	"00075e05f2"	0.0726
"00074de544"	"00075e0837"	-0.0684
"00074de544"	"00075e092e"	0.1418
"00074de544"	"00075e0965"	-0.4429
"00074de544"	"00075e0bc8"	0.1449
"00074de544"	"00075e0fbb"	0.0095
"00074de98a"	"00074dea7e"	-0.091
"00074de98a"	"00074debd9"	-0.1907
"00074de98a"	"00074deca3"	-0.0791
"00074de98a"	"00074def43"	-0.0051
"00074de98a"	"00074def99"	0.0371
"00074de98a"	"00074ecdad"	-0.4007
"00074de98a"	"00074ecf28"	-0.1756
"00074de98a"	"00074ed1e1"	-0.0789
"00074de98a"	"00074ed83b"	0.1888
"00074de98a"	"00074ee5e3"	-0.2289
"00074de98a"	"00074ee6e0"	-0.1734
"00074de98a"	"00074eea3a"	0.0335
"00074de98a"	"00074eff82"	-0.1529
"00074de98a"	"00074f0477"	-0.4022
"00074de98a"	"00074f08c3"	-0.1603
"00074de98a"	"00074f1859"	0.111
"00074de98a"	"00074f2268"	-0.3047
"00074de98a"	"00074f28be"	0.1674
"00074de98a"	"00074f294b"	0.0637
"00074de98a"	"00074f2ddd"	-0.1401
"00074de98a"	"00074f2e75"	0.0054
"00074de98a"	"00074f3088"	-0.1581
"00074de98a"	"00074f5a1c"	0.008
"00074de98a"	"00074f75b7"	-0.1007
"00074de98a"	"00074f8cd9"	0.1956
"00074de98a"	"00074f96dc"	-0.325
"00074de98a"	"00074fabaa"	0.1339
"00074de98a"	"00074facd9"	-0.2047
"00074de98a"	"00074fae3c"	-0.1042
"00074de98a"	"00074fb0a8"	-0.0631
"00074de98a"	"00074fb4e4"	-0.1976
"00074de98a"	"00074fb7c2"	0.3877
"00074de98a"	"00074fbd36"	0.1375
"00074de98a"	"00074fc27f"	0.1046
"00074de98a"	"00074fc31d"	0.1557
"00074de98a"	"00074fd569"	-0.1553
"00074de98a"	"00074fef15"	-0.0353
"00074de98a"	"00074ff562"	-0.4357
"00074de98a"	"00075007ca"	-0.4377
"00074de98a"	"0007500b86"	0.2529
"00074de98a"	"0007500d05"	-0.1073
"00074de98a"	"0007500ee4"	-0.2692
"00074de98a"	"0007500eee"	-0.0791
"00074de98a"	"00075013dc"	-0.1508

"00074de98a"	"000757b515"	−0.1076
"00074de98a"	"000757bc5a"	0.2286
"00074de98a"	"000757c320"	0.0753
"00074de98a"	"000757c9aa"	−0.0697
"00074de98a"	"000757ccbe"	0.2134
"00074de98a"	"000757cfa9"	−0.0507
"00074de98a"	"000757d390"	−0.3981
"00074de98a"	"000757d393"	0.4338
"00074de98a"	"000757d598"	−0.3128
"00074de98a"	"000757d5a2"	−0.4177
"00074de98a"	"000757d790"	0.151
"00074de98a"	"000757e30c"	−0.1969
"00074de98a"	"000757e4b0"	0.0579
"00074de98a"	"000757e7a0"	−0.556
"00074de98a"	"000757e8b3"	0.2482
"00074de98a"	"000757f627"	0.3105
"00074de98a"	"000757f925"	0.2604
"00074de98a"	"000757fa08"	0.0052
"00074de98a"	"000757fe52"	−0.0646
"00074de98a"	"000758024a"	−0.0694
"00074de98a"	"00075804bb"	−0.0104
"00074de98a"	"00075a0c04"	0.129
"00074de98a"	"00075a3110"	−0.0819
"00074de98a"	"00075a341a"	0.0179
"00074de98a"	"00075a3dcf"	−0.0525
"00074de98a"	"00075a3e22"	0.3687
"00074de98a"	"00075a48d8"	0.2022
"00074de98a"	"00075a5cfb"	−0.092
"00074de98a"	"00075a6151"	0.1215
"00074de98a"	"00075a6708"	0.007
"00074de98a"	"00075a7319"	−0.4828
"00074de98a"	"00075a7723"	0.5289
"00074de98a"	"00075a778b"	0.2265
"00074de98a"	"00075a7b8e"	−0.0812
"00074de98a"	"00075a7c79"	0.0379
"00074de98a"	"00075a81b6"	−0.0407
"00074de98a"	"00075a82ac"	0.3287
"00074de98a"	"00075a98e5"	0.6086
"00074de98a"	"00075b0d29"	0.2251
"00074de98a"	"00075b102a"	0.0467
"00074de98a"	"00075b1074"	−0.2192
"00074de98a"	"00075b135d"	0.179
"00074de98a"	"00075b138b"	0.2841
"00074de98a"	"00075b13a0"	0.0081
"00074de98a"	"00075b13bd"	−0.025
"00074de98a"	"00075b16a9"	−0.0848
"00074de98a"	"00075b1a28"	−0.0509
"00074de98a"	"00075b1a97"	−0.0053
"00074de98a"	"00075b1c7b"	−0.2282
"00074de98a"	"00075b1d24"	−0.1642

"00074de98a"	"00075b202b"	−0.097
"00074de98a"	"00075b22cb"	0.0054
"00074de98a"	"00075b22da"	−0.0903
"00074de98a"	"00075b2556"	−0.1509
"00074de98a"	"00075b25de"	−0.0759
"00074de98a"	"00075b260c"	−0.2317
"00074de98a"	"00075b26f1"	−0.0571
"00074de98a"	"00075b2920"	−0.0917
"00074de98a"	"00075b2a64"	−0.1789
"00074de98a"	"00075b2a9d"	−0.2493
"00074de98a"	"00075b2b37"	−0.5247
"00074de98a"	"00075b2cdd"	−0.1014
"00074de98a"	"00075b3038"	0.0619
"00074de98a"	"00075b30fe"	−0.1949
"00074de98a"	"00075b3362"	−0.1779
"00074de98a"	"00075b350a"	−0.2333
"00074de98a"	"00075b350e"	0.1315
"00074de98a"	"00075b3651"	−0.2061
"00074de98a"	"00075b38ca"	−0.1481
"00074de98a"	"00075b39cc"	−0.2635
"00074de98a"	"00075b3e1e"	0.2163
"00074de98a"	"00075b3e57"	−0.0185
"00074de98a"	"00075b4079"	−0.1646
"00074de98a"	"00075b4150"	0.0014
"00074de98a"	"00075b4194"	0.1271
"00074de98a"	"00075b42d5"	−0.0211
"00074de98a"	"00075b4424"	−0.197
"00074de98a"	"00075b4470"	0.2286
"00074de98a"	"00075b47ed"	−0.2121
"00074de98a"	"00075b4850"	0.0494
"00074de98a"	"00075b4ca0"	−0.1234
"00074de98a"	"00075b4d7f"	0.3085
"00074de98a"	"00075b520f"	−0.0613
"00074de98a"	"00075b525f"	−0.2679
"00074de98a"	"00075b58f8"	−0.2635
"00074de98a"	"00075b5bcc"	0.0306
"00074de98a"	"00075b5bfa"	−0.3676
"00074de98a"	"00075b6339"	0.1261
"00074de98a"	"00075b6658"	−0.1826
"00074de98a"	"00075b679a"	−0.0326
"00074de98a"	"00075b6cb7"	−0.0333
"00074de98a"	"00075b6df8"	0.1283
"00074de98a"	"00075b6ff6"	−0.0693
"00074de98a"	"00075b70ee"	−0.1599
"00074de98a"	"00075b7157"	−0.1326
"00074de98a"	"00075b7225"	0.0509
"00074de98a"	"00075b7c89"	0.2493
"00074de98a"	"00075b9048"	0.2091
"00074de98a"	"00075d0801"	−0.0371
"00074de98a"	"00075d1820"	−0.3118

"00074de98a"	"00075d1f3d"	−0.0072
"00074de98a"	"00075d2329"	−0.0874
"00074de98a"	"00075d2b9b"	−0.0282
"00074de98a"	"00075d3941"	−0.0857
"00074de98a"	"00075d3e96"	−0.2694
"00074de98a"	"00075d4864"	0.0565
"00074de98a"	"00075d5961"	0.3149
"00074de98a"	"00075d5a63"	−0.1372
"00074de98a"	"00075d6150"	−0.2756
"00074de98a"	"00075d67d0"	−0.2561
"00074de98a"	"00075d67e2"	0.505
"00074de98a"	"00075d73fc"	0.149
"00074de98a"	"00075d7729"	−0.2296
"00074de98a"	"00075d778c"	−0.423
"00074de98a"	"00075d7b9e"	−0.1006
"00074de98a"	"00075d7c8f"	0.0644
"00074de98a"	"00075d804d"	0.055
"00074de98a"	"00075d819f"	−0.0395
"00074de98a"	"00075d8601"	−0.1348
"00074de98a"	"00075d8c6a"	0.1126
"00074de98a"	"00075dfedc"	−0.06
"00074de98a"	"00075e05f2"	0.0693
"00074de98a"	"00075e0837"	−0.2501
"00074de98a"	"00075e092e"	−0.0477
"00074de98a"	"00075e0965"	−0.2118
"00074de98a"	"00075e0bc8"	0.0454
"00074de98a"	"00075e0fbb"	−0.1491
"00074dea7e"	"00074debd9"	−0.3593
"00074dea7e"	"00074deca3"	−0.0953
"00074dea7e"	"00074def43"	0.1153
"00074dea7e"	"00074def99"	−0.0077
"00074dea7e"	"00074ecdad"	0.0922
"00074dea7e"	"00074ecf28"	−0.4122
"00074dea7e"	"00074ed1e1"	0.1967
"00074dea7e"	"00074ed83b"	−0.0566
"00074dea7e"	"00074ee5e3"	−0.2664
"00074dea7e"	"00074ee6e0"	−0.3645
"00074dea7e"	"00074eea3a"	0.4573
"00074dea7e"	"00074eff82"	−0.3995
"00074dea7e"	"00074f0477"	−0.0743
"00074dea7e"	"00074f08c3"	−0.2499
"00074dea7e"	"00074f1859"	−0.0751
"00074dea7e"	"00074f2268"	−0.017
"00074dea7e"	"00074f28be"	−0.0515
"00074dea7e"	"00074f294b"	0.5285
"00074dea7e"	"00074f2ddd"	0.0518
"00074dea7e"	"00074f2e75"	0.0104
"00074dea7e"	"00074f3088"	−0.1711
"00074dea7e"	"00074f5a1c"	0.0198
"00074dea7e"	"00074f75b7"	−0.2192

"00074dea7e"	"00074f8cd9"	0.0624
"00074dea7e"	"00074f96dc"	−0.3903
"00074dea7e"	"00074fabaa"	−0.0047
"00074dea7e"	"00074facd9"	−0.0062
"00074dea7e"	"00074fae3c"	−0.2527
"00074dea7e"	"00074fb0a8"	−0.2495
"00074dea7e"	"00074fb4e4"	−0.2047
"00074dea7e"	"00074fb7c2"	0.2603
"00074dea7e"	"00074fbd36"	−0.1188
"00074dea7e"	"00074fc27f"	−0.1812
"00074dea7e"	"00074fc31d"	−0.0646
"00074dea7e"	"00074fd569"	−0.1221
"00074dea7e"	"00074fef15"	−0.04
"00074dea7e"	"00074ff562"	−0.3036
"00074dea7e"	"00075007ca"	−0.4036
"00074dea7e"	"0007500b86"	−0.0613
"00074dea7e"	"0007500d05"	−0.0457
"00074dea7e"	"0007500ee4"	−0.0755
"00074dea7e"	"0007500eee"	−0.238
"00074dea7e"	"00075013dc"	−0.3436
"00074dea7e"	"000757b515"	−0.1405
"00074dea7e"	"000757bc5a"	0.1586
"00074dea7e"	"000757c320"	−0.3005
"00074dea7e"	"000757c9aa"	−0.2317
"00074dea7e"	"000757ccbe"	−0.0159
"00074dea7e"	"000757cfa9"	−0.4065
"00074dea7e"	"000757d390"	−0.1204
"00074dea7e"	"000757d393"	0.0482
"00074dea7e"	"000757d598"	−0.2512
"00074dea7e"	"000757d5a2"	−0.2174
"00074dea7e"	"000757d790"	0.0471
"00074dea7e"	"000757e30c"	−0.2802
"00074dea7e"	"000757e4b0"	0.1578
"00074dea7e"	"000757e7a0"	−0.116
"00074dea7e"	"000757e8b3"	−0.166
"00074dea7e"	"000757f627"	−0.1047
"00074dea7e"	"000757f925"	−0.0171
"00074dea7e"	"000757fa08"	0.16
"00074dea7e"	"000757fe52"	0.0029
"00074dea7e"	"000758024a"	−0.39
"00074dea7e"	"00075804bb"	−0.2129
"00074dea7e"	"00075a0c04"	−0.0529
"00074dea7e"	"00075a3110"	−0.0505
"00074dea7e"	"00075a341a"	0.1176
"00074dea7e"	"00075a3dcf"	0.2245
"00074dea7e"	"00075a3e22"	−0.1611
"00074dea7e"	"00075a48d8"	−0.0151
"00074dea7e"	"00075a5cfb"	−0.3096
"00074dea7e"	"00075a6151"	−0.267
"00074dea7e"	"00075a6708"	0.0584

"00074dea7e"	"00075a7319"	-0.1613
"00074dea7e"	"00075a7723"	0.1754
"00074dea7e"	"00075a778b"	0.0993
"00074dea7e"	"00075a7b8e"	0.0377
"00074dea7e"	"00075a7c79"	-0.0374
"00074dea7e"	"00075a81b6"	-0.3375
"00074dea7e"	"00075a82ac"	0.1388
"00074dea7e"	"00075a98e5"	-0.1624
"00074dea7e"	"00075b0d29"	-0.291
"00074dea7e"	"00075b102a"	-0.0433
"00074dea7e"	"00075b1074"	0.3088
"00074dea7e"	"00075b135d"	-0.3602
"00074dea7e"	"00075b138b"	0.1492
"00074dea7e"	"00075b13a0"	0.054
"00074dea7e"	"00075b13bd"	-0.0828
"00074dea7e"	"00075b16a9"	-0.1109
"00074dea7e"	"00075b1a28"	-0.0919
"00074dea7e"	"00075b1a97"	0.0663
"00074dea7e"	"00075b1c7b"	-0.3233
"00074dea7e"	"00075b1d24"	0.0423
"00074dea7e"	"00075b202b"	-0.2837
"00074dea7e"	"00075b22cb"	0.1723
"00074dea7e"	"00075b22da"	0.3931
"00074dea7e"	"00075b2556"	0.3279
"00074dea7e"	"00075b25de"	-0.0374
"00074dea7e"	"00075b260c"	-0.1489
"00074dea7e"	"00075b26f1"	-0.2759
"00074dea7e"	"00075b2920"	-0.2375
"00074dea7e"	"00075b2a64"	-0.5723
"00074dea7e"	"00075b2a9d"	-0.2093
"00074dea7e"	"00075b2b37"	-0.6158
"00074dea7e"	"00075b2cdd"	-0.1714
"00074dea7e"	"00075b3038"	-0.0199
"00074dea7e"	"00075b30fe"	0.2339
"00074dea7e"	"00075b3362"	-0.1506
"00074dea7e"	"00075b350a"	0.0024
"00074dea7e"	"00075b350e"	-0.1071
"00074dea7e"	"00075b3651"	-0.1181
"00074dea7e"	"00075b38ca"	0.0173
"00074dea7e"	"00075b39cc"	-0.0515
"00074dea7e"	"00075b3e1e"	0.0752
"00074dea7e"	"00075b3e57"	-0.1952
"00074dea7e"	"00075b4079"	-0.2589
"00074dea7e"	"00075b4150"	-0.2828
"00074dea7e"	"00075b4194"	-0.0907
"00074dea7e"	"00075b42d5"	0.2499
"00074dea7e"	"00075b4424"	-0.2269
"00074dea7e"	"00075b4470"	0.2279
"00074dea7e"	"00075b47ed"	0.177
"00074dea7e"	"00075b4850"	0.14

"00074dea7e"	"00075b4ca0"	-0.3916
"00074dea7e"	"00075b4d7f"	0.0622
"00074dea7e"	"00075b520f"	-0.3453
"00074dea7e"	"00075b525f"	-0.0809
"00074dea7e"	"00075b58f8"	-0.257
"00074dea7e"	"00075b5bcc"	-0.0367
"00074dea7e"	"00075b5bfa"	-0.3721
"00074dea7e"	"00075b6339"	-0.1847
"00074dea7e"	"00075b6658"	-0.3037
"00074dea7e"	"00075b679a"	0.0721
"00074dea7e"	"00075b6cb7"	-0.0749
"00074dea7e"	"00075b6df8"	0.1911
"00074dea7e"	"00075b6ff6"	0.058
"00074dea7e"	"00075b70ee"	-0.1017
"00074dea7e"	"00075b7157"	-0.2511
"00074dea7e"	"00075b7225"	-0.0145
"00074dea7e"	"00075b7c89"	0.0362
"00074dea7e"	"00075b9048"	-0.0698
"00074dea7e"	"00075d0801"	-0.024
"00074dea7e"	"00075d1820"	-0.0678
"00074dea7e"	"00075d1f3d"	-0.1026
"00074dea7e"	"00075d2329"	0.1447
"00074dea7e"	"00075d2b9b"	-0.1934
"00074dea7e"	"00075d3941"	-0.2783
"00074dea7e"	"00075d3e96"	0.0083
"00074dea7e"	"00075d4864"	0.1391
"00074dea7e"	"00075d5961"	-0.181
"00074dea7e"	"00075d5a63"	-0.1151
"00074dea7e"	"00075d6150"	-0.1842
"00074dea7e"	"00075d67d0"	-0.3619
"00074dea7e"	"00075d67e2"	-0.1729
"00074dea7e"	"00075d73fc"	0.1908
"00074dea7e"	"00075d7729"	-0.4754
"00074dea7e"	"00075d778c"	-0.177
"00074dea7e"	"00075d7b9e"	-0.1514
"00074dea7e"	"00075d7c8f"	-0.0102
"00074dea7e"	"00075d804d"	-0.2173
"00074dea7e"	"00075d819f"	-0.0624
"00074dea7e"	"00075d8601"	-0.3113
"00074dea7e"	"00075d8c6a"	0.0703
"00074dea7e"	"00075dfedc"	-0.0877
"00074dea7e"	"00075e05f2"	-0.1714
"00074dea7e"	"00075e0837"	0.0776
"00074dea7e"	"00075e092e"	0.4178
"00074dea7e"	"00075e0965"	-0.1995
"00074dea7e"	"00075e0bc8"	0.0798
"00074dea7e"	"00075e0fbb"	0.2832
"00074debd9"	"00074deca3"	0.1661
"00074debd9"	"00074def43"	0.2541
"00074debd9"	"00074def99"	-0.7199

"00074debd9"	"00074ecdad"	-0.0682
"00074debd9"	"00074ecf28"	-0.472
"00074debd9"	"00074ed1e1"	-0.6315
"00074debd9"	"00074ed83b"	-0.1472
"00074debd9"	"00074ee5e3"	0.2469
"00074debd9"	"00074ee6e0"	-0.2136
"00074debd9"	"00074eea3a"	-0.1911
"00074debd9"	"00074eff82"	-0.2996
"00074debd9"	"00074f0477"	-0.0439
"00074debd9"	"00074f08c3"	-0.4143
"00074debd9"	"00074f1859"	0.0113
"00074debd9"	"00074f2268"	-0.3806
"00074debd9"	"00074f28be"	-0.1583
"00074debd9"	"00074f294b"	-0.4088
"00074debd9"	"00074f2ddd"	-0.4133
"00074debd9"	"00074f2e75"	-0.3773
"00074debd9"	"00074f3088"	-0.2035
"00074debd9"	"00074f5a1c"	0.1085
"00074debd9"	"00074f75b7"	0.2577
"00074debd9"	"00074f8cd9"	0.0137
"00074debd9"	"00074f96dc"	-0.3566
"00074debd9"	"00074fabaa"	0.0581
"00074debd9"	"00074facd9"	0.0837
"00074debd9"	"00074fae3c"	-0.2833
"00074debd9"	"00074fb0a8"	-0.2095
"00074debd9"	"00074fb4e4"	-0.2595
"00074debd9"	"00074fb7c2"	-0.3145
"00074debd9"	"00074fbd36"	-0.3431
"00074debd9"	"00074fc27f"	0.0907
"00074debd9"	"00074fc31d"	-0.4654
"00074debd9"	"00074fd569"	-0.2796
"00074debd9"	"00074fef15"	-0.4635
"00074debd9"	"00074ff562"	-0.6104
"00074debd9"	"00075007ca"	0.1274
"00074debd9"	"0007500b86"	0.1061
"00074debd9"	"0007500d05"	-0.0668
"00074debd9"	"0007500ee4"	-0.3889
"00074debd9"	"0007500eee"	0.3925
"00074debd9"	"00075013dc"	-0.432
"00074debd9"	"000757b515"	-0.0553
"00074debd9"	"000757bc5a"	-0.0335
"00074debd9"	"000757c320"	-0.0295
"00074debd9"	"000757c9aa"	-0.0678
"00074debd9"	"000757ccbe"	-0.3106
"00074debd9"	"000757cfa9"	-0.2283
"00074debd9"	"000757d390"	-0.3787
"00074debd9"	"000757d393"	0.2758
"00074debd9"	"000757d598"	-0.1963
"00074debd9"	"000757d5a2"	-0.1386
"00074debd9"	"000757d790"	-0.0839

"00074debd9"	"000757e30c"	-0.1269
"00074debd9"	"000757e4b0"	-0.0169
"00074debd9"	"000757e7a0"	-0.187
"00074debd9"	"000757e8b3"	-0.0922
"00074debd9"	"000757f627"	-0.1802
"00074debd9"	"000757f925"	0.1511
"00074debd9"	"000757fa08"	-0.1668
"00074debd9"	"000757fe52"	-0.2102
"00074debd9"	"000758024a"	-0.1202
"00074debd9"	"00075804bb"	0.1923
"00074debd9"	"00075a0c04"	-0.3967
"00074debd9"	"00075a3110"	-0.2574
"00074debd9"	"00075a341a"	-0.5929
"00074debd9"	"00075a3dcf"	0.191
"00074debd9"	"00075a3e22"	-0.3766
"00074debd9"	"00075a48d8"	-0.2276
"00074debd9"	"00075a5cfb"	-0.1151
"00074debd9"	"00075a6151"	-0.0264
"00074debd9"	"00075a6708"	-0.2941
"00074debd9"	"00075a7319"	-0.3039
"00074debd9"	"00075a7723"	-0.294
"00074debd9"	"00075a778b"	-0.5896
"00074debd9"	"00075a7b8e"	-0.2763
"00074debd9"	"00075a7c79"	-0.2731
"00074debd9"	"00075a81b6"	-0.5702
"00074debd9"	"00075a82ac"	-0.2993
"00074debd9"	"00075a98e5"	-0.406
"00074debd9"	"00075b0d29"	-0.3599
"00074debd9"	"00075b102a"	0.1526
"00074debd9"	"00075b1074"	-0.4754
"00074debd9"	"00075b135d"	-0.5659
"00074debd9"	"00075b138b"	-0.0031
"00074debd9"	"00075b13a0"	-0.4367
"00074debd9"	"00075b13bd"	-0.5446
"00074debd9"	"00075b16a9"	-0.0681
"00074debd9"	"00075b1a28"	-0.3316
"00074debd9"	"00075b1a97"	-0.4867
"00074debd9"	"00075b1c7b"	-0.2708
"00074debd9"	"00075b1d24"	-0.2863
"00074debd9"	"00075b202b"	0.0833
"00074debd9"	"00075b22cb"	-0.2779
"00074debd9"	"00075b22da"	-0.3459
"00074debd9"	"00075b2556"	-0.359
"00074debd9"	"00075b25de"	0.2078
"00074debd9"	"00075b260c"	0.0505
"00074debd9"	"00075b26f1"	-0.3163
"00074debd9"	"00075b2920"	0.0573
"00074debd9"	"00075b2a64"	0.0608
"00074debd9"	"00075b2a9d"	0.0529
"00074debd9"	"00075b2b37"	-0.2583

"00074debd9"	"00075b2cdd"	-0.4673
"00074debd9"	"00075b3038"	0.1244
"00074debd9"	"00075b30fe"	-0.3125
"00074debd9"	"00075b3362"	-0.3492
"00074debd9"	"00075b350a"	-0.1865
"00074debd9"	"00075b350e"	-0.0667
"00074debd9"	"00075b3651"	-0.2446
"00074debd9"	"00075b38ca"	0.1259
"00074debd9"	"00075b39cc"	-0.0713
"00074debd9"	"00075b3e1e"	0.1037
"00074debd9"	"00075b3e57"	-0.1114
"00074debd9"	"00075b4079"	-0.244
"00074debd9"	"00075b4150"	-0.0972
"00074debd9"	"00075b4194"	0.005
"00074debd9"	"00075b42d5"	0.0505
"00074debd9"	"00075b4424"	-0.3862
"00074debd9"	"00075b4470"	0.0576
"00074debd9"	"00075b47ed"	-0.2551
"00074debd9"	"00075b4850"	-0.0618
"00074debd9"	"00075b4ca0"	-0.4633
"00074debd9"	"00075b4d7f"	-0.1233
"00074debd9"	"00075b520f"	-0.2351
"00074debd9"	"00075b525f"	-0.5729
"00074debd9"	"00075b58f8"	0.0545
"00074debd9"	"00075b5bcc"	-0.1818
"00074debd9"	"00075b5bfa"	-0.0931
"00074debd9"	"00075b6339"	0.0306
"00074debd9"	"00075b6658"	-0.253
"00074debd9"	"00075b679a"	-0.1811
"00074debd9"	"00075b6cb7"	-0.0887
"00074debd9"	"00075b6df8"	-0.383
"00074debd9"	"00075b6ff6"	-0.1653
"00074debd9"	"00075b70ee"	-0.2802
"00074debd9"	"00075b7157"	-0.2023
"00074debd9"	"00075b7225"	-0.4106
"00074debd9"	"00075b7c89"	-0.2205
"00074debd9"	"00075b9048"	-0.0691
"00074debd9"	"00075d0801"	0.0307
"00074debd9"	"00075d1820"	-0.5021
"00074debd9"	"00075d1f3d"	-0.0608
"00074debd9"	"00075d2329"	-0.1369
"00074debd9"	"00075d2b9b"	0.1385
"00074debd9"	"00075d3941"	-0.37
"00074debd9"	"00075d3e96"	-0.3276
"00074debd9"	"00075d4864"	-0.0462
"00074debd9"	"00075d5961"	-0.0806
"00074debd9"	"00075d5a63"	0.1445
"00074debd9"	"00075d6150"	-0.3638
"00074debd9"	"00075d67d0"	-0.054
"00074debd9"	"00075d67e2"	-0.2399

"00074debd9"	"00075d73fc"	-0.3417
"00074debd9"	"00075d7729"	-0.6684
"00074debd9"	"00075d778c"	-0.401
"00074debd9"	"00075d7b9e"	-0.2075
"00074debd9"	"00075d7c8f"	-0.1876
"00074debd9"	"00075d804d"	0.103
"00074debd9"	"00075d819f"	0.0916
"00074debd9"	"00075d8601"	-0.1901
"00074debd9"	"00075d8c6a"	-0.3444
"00074debd9"	"00075dfedc"	-0.1589
"00074debd9"	"00075e05f2"	0.3079
"00074debd9"	"00075e0837"	0.128
"00074debd9"	"00075e092e"	-0.3819
"00074debd9"	"00075e0965"	-0.1747
"00074debd9"	"00075e0bc8"	0.1369
"00074debd9"	"00075e0fbb"	-0.2411
"00074deca3"	"00074def43"	0.3279
"00074deca3"	"00074def99"	-0.1642
"00074deca3"	"00074ecdad"	0.0791
"00074deca3"	"00074ecf28"	-0.2778
"00074deca3"	"00074ed1e1"	-0.0975
"00074deca3"	"00074ed83b"	0.1762
"00074deca3"	"00074ee5e3"	-0.0874
"00074deca3"	"00074ee6e0"	-0.2276
"00074deca3"	"00074eea3a"	0.0761
"00074deca3"	"00074eff82"	-0.1156
"00074deca3"	"00074f0477"	-0.3766
"00074deca3"	"00074f08c3"	-0.2797
"00074deca3"	"00074f1859"	-0.0222
"00074deca3"	"00074f2268"	-0.3252
"00074deca3"	"00074f28be"	0.0034
"00074deca3"	"00074f294b"	-0.1765
"00074deca3"	"00074f2ddd"	0.1328
"00074deca3"	"00074f2e75"	-0.5336
"00074deca3"	"00074f3088"	-0.5205
"00074deca3"	"00074f5a1c"	0.1725
"00074deca3"	"00074f75b7"	0.1317
"00074deca3"	"00074f8cd9"	0.0234
"00074deca3"	"00074f96dc"	-0.3168
"00074deca3"	"00074fabaa"	0.06
"00074deca3"	"00074facd9"	-0.2872
"00074deca3"	"00074fae3c"	-0.1134
"00074deca3"	"00074fb0a8"	-0.0637
"00074deca3"	"00074fb4e4"	-0.1368
"00074deca3"	"00074fb7c2"	-0.0839
"00074deca3"	"00074fbd36"	-0.1908
"00074deca3"	"00074fc27f"	0.0549
"00074deca3"	"00074fc31d"	-0.1499
"00074deca3"	"00074fd569"	-0.1193
"00074deca3"	"00074fef15"	-0.0088

"00074deca3"	"00074ff562"	-0.2351
"00074deca3"	"00075007ca"	0.002
"00074deca3"	"0007500b86"	0.1747
"00074deca3"	"0007500d05"	-0.0605
"00074deca3"	"0007500ee4"	-0.1953
"00074deca3"	"0007500eee"	-0.1246
"00074deca3"	"00075013dc"	-0.3865
"00074deca3"	"000757b515"	-0.1919
"00074deca3"	"000757bc5a"	-0.0993
"00074deca3"	"000757c320"	0.1867
"00074deca3"	"000757c9aa"	-0.0445
"00074deca3"	"000757ccbe"	-0.1236
"00074deca3"	"000757cfa9"	-0.0372
"00074deca3"	"000757d390"	-0.202
"00074deca3"	"000757d393"	0.3537
"00074deca3"	"000757d598"	-0.1911
"00074deca3"	"000757d5a2"	-0.2242
"00074deca3"	"000757d790"	0.2371
"00074deca3"	"000757e30c"	-0.4559
"00074deca3"	"000757e4b0"	-0.1223
"00074deca3"	"000757e7a0"	-0.2502
"00074deca3"	"000757e8b3"	0.0699
"00074deca3"	"000757f627"	-0.0401
"00074deca3"	"000757f925"	0.1185
"00074deca3"	"000757fa08"	-0.188
"00074deca3"	"000757fe52"	0.0629
"00074deca3"	"000758024a"	-0.3147
"00074deca3"	"00075804bb"	-0.2892
"00074deca3"	"00075a0c04"	-0.0658
"00074deca3"	"00075a3110"	-0.1127
"00074deca3"	"00075a341a"	-0.2318
"00074deca3"	"00075a3dcf"	0.2949
"00074deca3"	"00075a3e22"	-0.1142
"00074deca3"	"00075a48d8"	-0.1446
"00074deca3"	"00075a5cfb"	-0.2518
"00074deca3"	"00075a6151"	0.1221
"00074deca3"	"00075a6708"	-0.368
"00074deca3"	"00075a7319"	-0.1414
"00074deca3"	"00075a7723"	-0.1722
"00074deca3"	"00075a778b"	-0.175
"00074deca3"	"00075a7b8e"	-0.3235
"00074deca3"	"00075a7c79"	0.025
"00074deca3"	"00075a81b6"	-0.2619
"00074deca3"	"00075a82ac"	-0.1598
"00074deca3"	"00075a98e5"	-0.1043
"00074deca3"	"00075b0d29"	-0.1335
"00074deca3"	"00075b102a"	-0.1012
"00074deca3"	"00075b1074"	-0.3364
"00074deca3"	"00075b135d"	-0.1805
"00074deca3"	"00075b138b"	0.1455

"00074deca3"	"00075b13a0"	-0.0935
"00074deca3"	"00075b13bd"	-0.3588
"00074deca3"	"00075b16a9"	-0.0213
"00074deca3"	"00075b1a28"	0.1363
"00074deca3"	"00075b1a97"	0.0929
"00074deca3"	"00075b1c7b"	-0.2685
"00074deca3"	"00075b1d24"	-0.1911
"00074deca3"	"00075b202b"	-0.0614
"00074deca3"	"00075b22cb"	0.1108
"00074deca3"	"00075b22da"	-0.138
"00074deca3"	"00075b2556"	-0.1679
"00074deca3"	"00075b25de"	0.0187
"00074deca3"	"00075b260c"	0.1767
"00074deca3"	"00075b26f1"	-0.1511
"00074deca3"	"00075b2920"	-0.3204
"00074deca3"	"00075b2a64"	-0.2985
"00074deca3"	"00075b2a9d"	-0.1592
"00074deca3"	"00075b2b37"	-0.4639
"00074deca3"	"00075b2cdd"	0.0263
"00074deca3"	"00075b3038"	0.023
"00074deca3"	"00075b30fe"	-0.0776
"00074deca3"	"00075b3362"	-0.3114
"00074deca3"	"00075b350a"	-0.3496
"00074deca3"	"00075b350e"	0.2512
"00074deca3"	"00075b3651"	-0.2894
"00074deca3"	"00075b38ca"	0.0049
"00074deca3"	"00075b39cc"	0.1557
"00074deca3"	"00075b3e1e"	0.1044
"00074deca3"	"00075b3e57"	-0.1378
"00074deca3"	"00075b4079"	-0.3606
"00074deca3"	"00075b4150"	-0.195
"00074deca3"	"00075b4194"	0.1629
"00074deca3"	"00075b42d5"	0.0209
"00074deca3"	"00075b4424"	-0.3765
"00074deca3"	"00075b4470"	-0.0993
"00074deca3"	"00075b47ed"	0.1124
"00074deca3"	"00075b4850"	-0.1035
"00074deca3"	"00075b4ca0"	-0.264
"00074deca3"	"00075b4d7f"	-0.104
"00074deca3"	"00075b520f"	-0.0566
"00074deca3"	"00075b525f"	-0.1269
"00074deca3"	"00075b58f8"	-0.2105
"00074deca3"	"00075b5bcc"	-0.17
"00074deca3"	"00075b5bfa"	-0.2012
"00074deca3"	"00075b6339"	0.1222
"00074deca3"	"00075b6658"	-0.2102
"00074deca3"	"00075b679a"	0.1843
"00074deca3"	"00075b6cb7"	-0.4583
"00074deca3"	"00075b6df8"	-0.1909
"00074deca3"	"00075b6ff6"	-0.2009

"00074deca3"	"00075b70ee"	-0.2702
"00074deca3"	"00075b7157"	-0.2232
"00074deca3"	"00075b7225"	0.084
"00074deca3"	"00075b7c89"	-0.1211
"00074deca3"	"00075b9048"	0.0162
"00074deca3"	"00075d0801"	-0.0713
"00074deca3"	"00075d1820"	-0.2049
"00074deca3"	"00075d1f3d"	-0.2177
"00074deca3"	"00075d2329"	0.0391
"00074deca3"	"00075d2b9b"	-0.0879
"00074deca3"	"00075d3941"	-0.1405
"00074deca3"	"00075d3e96"	-0.3065
"00074deca3"	"00075d4864"	-0.0038
"00074deca3"	"00075d5961"	0.0579
"00074deca3"	"00075d5a63"	-0.081
"00074deca3"	"00075d6150"	0.1906
"00074deca3"	"00075d67d0"	-0.295
"00074deca3"	"00075d67e2"	-0.005
"00074deca3"	"00075d73fc"	0.017
"00074deca3"	"00075d7729"	-0.4932
"00074deca3"	"00075d778c"	-0.1107
"00074deca3"	"00075d7b9e"	-0.0705
"00074deca3"	"00075d7c8f"	-0.0205
"00074deca3"	"00075d804d"	-0.1006
"00074deca3"	"00075d819f"	-0.1732
"00074deca3"	"00075d8601"	0.0473
"00074deca3"	"00075d8c6a"	-0.1554
"00074deca3"	"00075dfedc"	0.1242
"00074deca3"	"00075e05f2"	0.2726
"00074deca3"	"00075e0837"	-0.1168
"00074deca3"	"00075e092e"	-0.0329
"00074deca3"	"00075e0965"	-0.4987
"00074deca3"	"00075e0bc8"	0.0111
"00074deca3"	"00075e0fbb"	-0.1465
"00074def43"	"00074def99"	-0.2732
"00074def43"	"00074ecdad"	0.1904
"00074def43"	"00074ecf28"	-0.1076
"00074def43"	"00074ed1e1"	0.0389
"00074def43"	"00074ed83b"	0.2233
"00074def43"	"00074ee5e3"	0.1195
"00074def43"	"00074ee6e0"	-0.2279
"00074def43"	"00074eea3a"	0.1536
"00074def43"	"00074eff82"	-0.0692
"00074def43"	"00074f0477"	-0.0806
"00074def43"	"00074f08c3"	-0.3183
"00074def43"	"00074f1859"	0.0093
"00074def43"	"00074f2268"	-0.3552
"00074def43"	"00074f28be"	0.0855
"00074def43"	"00074f294b"	0.1706
"00074def43"	"00074f2ddd"	-0.0365

"00074def43"	"00074f2e75"	-0.111
"00074def43"	"00074f3088"	-0.2831
"00074def43"	"00074f5a1c"	0.2339
"00074def43"	"00074f75b7"	0.323
"00074def43"	"00074f8cd9"	0.3045
"00074def43"	"00074f96dc"	-0.1881
"00074def43"	"00074fabaa"	0.0222
"00074def43"	"00074facd9"	0.1161
"00074def43"	"00074fae3c"	-0.1186
"00074def43"	"00074fb0a8"	-0.1671
"00074def43"	"00074fb4e4"	-0.1395
"00074def43"	"00074fb7c2"	-0.0731
"00074def43"	"00074fbd36"	-0.4397
"00074def43"	"00074fc27f"	0.155
"00074def43"	"00074fc31d"	-0.268
"00074def43"	"00074fd569"	-0.0314
"00074def43"	"00074fef15"	-0.1252
"00074def43"	"00074ff562"	-0.1987
"00074def43"	"00075007ca"	-0.1098
"00074def43"	"0007500b86"	0.0947
"00074def43"	"0007500d05"	0.0966
"00074def43"	"0007500ee4"	-0.1424
"00074def43"	"0007500eee"	0.0576
"00074def43"	"00075013dc"	-0.3393
"00074def43"	"000757b515"	0.0928
"00074def43"	"000757bc5a"	-0.1173
"00074def43"	"000757c320"	0.0338
"00074def43"	"000757c9aa"	0.0402
"00074def43"	"000757ccbe"	-0.0146
"00074def43"	"000757cfa9"	-0.4013
"00074def43"	"000757d390"	-0.2055
"00074def43"	"000757d393"	0.1975
"00074def43"	"000757d598"	-0.1232
"00074def43"	"000757d5a2"	-0.1038
"00074def43"	"000757d790"	0.0925
"00074def43"	"000757e30c"	-0.2171
"00074def43"	"000757e4b0"	0.0865
"00074def43"	"000757e7a0"	-0.006
"00074def43"	"000757e8b3"	0.0555
"00074def43"	"000757f627"	0.0423
"00074def43"	"000757f925"	0.2696
"00074def43"	"000757fa08"	-0.1595
"00074def43"	"000757fe52"	-0.0517
"00074def43"	"000758024a"	-0.2196
"00074def43"	"00075804bb"	-0.1348
"00074def43"	"00075a0c04"	0.2463
"00074def43"	"00075a3110"	-0.0413
"00074def43"	"00075a341a"	0.0378
"00074def43"	"00075a3dcf"	0.2724
"00074def43"	"00075a3e22"	0.0454

"00074def43"	"00075a48d8"	0.198
"00074def43"	"00075a5cfb"	-0.2174
"00074def43"	"00075a6151"	-0.0752
"00074def43"	"00075a6708"	-0.0557
"00074def43"	"00075a7319"	-0.1834
"00074def43"	"00075a7723"	0.1369
"00074def43"	"00075a778b"	-0.2633
"00074def43"	"00075a7b8e"	-0.0722
"00074def43"	"00075a7c79"	0.0711
"00074def43"	"00075a81b6"	-0.1787
"00074def43"	"00075a82ac"	-0.1069
"00074def43"	"00075a98e5"	-0.2049
"00074def43"	"00075b0d29"	-0.4116
"00074def43"	"00075b102a"	-0.0366
"00074def43"	"00075b1074"	0.1139
"00074def43"	"00075b135d"	-0.5425
"00074def43"	"00075b138b"	0.1899
"00074def43"	"00075b13a0"	-0.1153
"00074def43"	"00075b13bd"	-0.2401
"00074def43"	"00075b16a9"	0.1452
"00074def43"	"00075b1a28"	-0.0961
"00074def43"	"00075b1a97"	-0.012
"00074def43"	"00075b1c7b"	-0.3233
"00074def43"	"00075b1d24"	0.0452
"00074def43"	"00075b202b"	0.0156
"00074def43"	"00075b22cb"	0.1838
"00074def43"	"00075b22da"	0.1751
"00074def43"	"00075b2556"	-0.0326
"00074def43"	"00075b25de"	0.1168
"00074def43"	"00075b260c"	0.0619
"00074def43"	"00075b26f1"	-0.0174
"00074def43"	"00075b2920"	-0.037
"00074def43"	"00075b2a64"	-0.0965
"00074def43"	"00075b2a9d"	-0.1837
"00074def43"	"00075b2b37"	-0.4112
"00074def43"	"00075b2cdd"	-0.1333
"00074def43"	"00075b3038"	0.2377
"00074def43"	"00075b30fe"	0.1182
"00074def43"	"00075b3362"	-0.0467
"00074def43"	"00075b350a"	-0.0655
"00074def43"	"00075b350e"	-0.0624
"00074def43"	"00075b3651"	0.0444
"00074def43"	"00075b38ca"	0.1354
"00074def43"	"00075b39cc"	0.068
"00074def43"	"00075b3e1e"	0.3211
"00074def43"	"00075b3e57"	0.0472
"00074def43"	"00075b4079"	4e-04
"00074def43"	"00075b4150"	-0.223
"00074def43"	"00075b4194"	0.1892
"00074def43"	"00075b42d5"	0.3306

"00074def43"	"00075b4424"	-0.1286
"00074def43"	"00075b4470"	-0.1173
"00074def43"	"00075b47ed"	0.118
"00074def43"	"00075b4850"	0.1124
"00074def43"	"00075b4ca0"	-0.1455
"00074def43"	"00075b4d7f"	0.146
"00074def43"	"00075b520f"	-0.2039
"00074def43"	"00075b525f"	0.0844
"00074def43"	"00075b58f8"	-0.4944
"00074def43"	"00075b5bcc"	0.1186
"00074def43"	"00075b5bfa"	-0.1695
"00074def43"	"00075b6339"	0.2303
"00074def43"	"00075b6658"	-0.0619
"00074def43"	"00075b679a"	0.2369
"00074def43"	"00075b6cb7"	-0.2283
"00074def43"	"00075b6df8"	0.1141
"00074def43"	"00075b6ff6"	0.163
"00074def43"	"00075b70ee"	-0.2072
"00074def43"	"00075b7157"	-0.0147
"00074def43"	"00075b7225"	-0.0136
"00074def43"	"00075b7c89"	-0.0325
"00074def43"	"00075b9048"	0.0571
"00074def43"	"00075d0801"	0.0737
"00074def43"	"00075d1820"	-0.2129
"00074def43"	"00075d1f3d"	0.1395
"00074def43"	"00075d2329"	-0.0471
"00074def43"	"00075d2b9b"	0.0498
"00074def43"	"00075d3941"	-0.2204
"00074def43"	"00075d3e96"	-0.1818
"00074def43"	"00075d4864"	-0.0265
"00074def43"	"00075d5961"	-0.0686
"00074def43"	"00075d5a63"	0.1614
"00074def43"	"00075d6150"	-0.0442
"00074def43"	"00075d67d0"	0.1539
"00074def43"	"00075d67e2"	-0.0395
"00074def43"	"00075d73fc"	0.0138
"00074def43"	"00075d7729"	-0.3381
"00074def43"	"00075d778c"	0.097
"00074def43"	"00075d7b9e"	-0.0915
"00074def43"	"00075d7c8f"	0.1585
"00074def43"	"00075d804d"	-0.0391
"00074def43"	"00075d819f"	-0.1686
"00074def43"	"00075d8601"	-0.1487
"00074def43"	"00075d8c6a"	-0.0422
"00074def43"	"00075dfedc"	0.2022
"00074def43"	"00075e05f2"	0.3084
"00074def43"	"00075e0837"	0.0966
"00074def43"	"00075e092e"	0.2334
"00074def43"	"00075e0965"	-0.3898
"00074def43"	"00075e0bc8"	0.0417

"00074def43"	"00075e0fbb"	0.0939
"00074def99"	"00074ecdad"	-0.4421
"00074def99"	"00074ecf28"	-0.1926
"00074def99"	"00074ed1e1"	-0.0192
"00074def99"	"00074ed83b"	0.2066
"00074def99"	"00074ee5e3"	-0.3226
"00074def99"	"00074ee6e0"	-0.0973
"00074def99"	"00074eea3a"	-0.0587
"00074def99"	"00074eff82"	0.0471
"00074def99"	"00074f0477"	-0.4031
"00074def99"	"00074f08c3"	-0.1103
"00074def99"	"00074f1859"	-0.2136
"00074def99"	"00074f2268"	-0.1451
"00074def99"	"00074f28be"	-0.0833
"00074def99"	"00074f294b"	0.0883
"00074def99"	"00074f2ddd"	0.079
"00074def99"	"00074f2e75"	-0.0349
"00074def99"	"00074f3088"	-0.0183
"00074def99"	"00074f5a1c"	-0.1433
"00074def99"	"00074f75b7"	-0.1068
"00074def99"	"00074f8cd9"	-0.1916
"00074def99"	"00074f96dc"	-0.3008
"00074def99"	"00074fabaa"	-0.3773
"00074def99"	"00074facd9"	-0.3401
"00074def99"	"00074fae3c"	0.0092
"00074def99"	"00074fb0a8"	-0.2017
"00074def99"	"00074fb4e4"	-0.174
"00074def99"	"00074fb7c2"	0.1673
"00074def99"	"00074fbd36"	0.0825
"00074def99"	"00074fc27f"	-0.1871
"00074def99"	"00074fc31d"	0.4495
"00074def99"	"00074fd569"	-0.1284
"00074def99"	"00074fef15"	0.4588
"00074def99"	"00074ff562"	-0.4888
"00074def99"	"00075007ca"	-0.1485
"00074def99"	"0007500b86"	-0.1258
"00074def99"	"0007500d05"	-0.3243
"00074def99"	"0007500ee4"	-0.2303
"00074def99"	"0007500eee"	-0.2969
"00074def99"	"00075013dc"	-0.2671
"00074def99"	"000757b515"	0.0959
"00074def99"	"000757bc5a"	-0.0832
"00074def99"	"000757c320"	-0.2286
"00074def99"	"000757c9aa"	-0.0307
"00074def99"	"000757ccbe"	-0.124
"00074def99"	"000757cfa9"	-0.0737
"00074def99"	"000757d390"	-0.2234
"00074def99"	"000757d393"	-0.1203
"00074def99"	"000757d598"	-0.2806
"00074def99"	"000757d5a2"	-0.2036

"00074def99"	"000757d790"	0.0054
"00074def99"	"000757e30c"	-0.2005
"00074def99"	"000757e4b0"	-0.0419
"00074def99"	"000757e7a0"	-0.1319
"00074def99"	"000757e8b3"	0.0459
"00074def99"	"000757f627"	6e-04
"00074def99"	"000757f925"	-0.1408
"00074def99"	"000757fa08"	-0.039
"00074def99"	"000757fe52"	-0.2143
"00074def99"	"000758024a"	-0.1627
"00074def99"	"00075804bb"	-0.4048
"00074def99"	"00075a0c04"	0.0027
"00074def99"	"00075a3110"	-0.0515
"00074def99"	"00075a341a"	0.0988
"00074def99"	"00075a3dcf"	-0.1274
"00074def99"	"00075a3e22"	-0.0054
"00074def99"	"00075a48d8"	-0.0696
"00074def99"	"00075a5cfb"	-0.3406
"00074def99"	"00075a6151"	-0.091
"00074def99"	"00075a6708"	-0.0133
"00074def99"	"00075a7319"	-0.2089
"00074def99"	"00075a7723"	0.1393
"00074def99"	"00075a778b"	-0.167
"00074def99"	"00075a7b8e"	-0.0052
"00074def99"	"00075a7c79"	0.0952
"00074def99"	"00075a81b6"	0.1983
"00074def99"	"00075a82ac"	0.1373
"00074def99"	"00075a98e5"	-0.0314
"00074def99"	"00075b0d29"	0.1644
"00074def99"	"00075b102a"	0.0302
"00074def99"	"00075b1074"	-0.0338
"00074def99"	"00075b135d"	0.1054
"00074def99"	"00075b138b"	0.103
"00074def99"	"00075b13a0"	-0.415
"00074def99"	"00075b13bd"	0.0941
"00074def99"	"00075b16a9"	-0.1182
"00074def99"	"00075b1a28"	0.0712
"00074def99"	"00075b1a97"	0.2936
"00074def99"	"00075b1c7b"	-0.293
"00074def99"	"00075b1d24"	0.1825
"00074def99"	"00075b202b"	-0.2098
"00074def99"	"00075b22cb"	-0.0705
"00074def99"	"00075b22da"	0.0035
"00074def99"	"00075b2556"	3e-04
"00074def99"	"00075b25de"	-0.2281
"00074def99"	"00075b260c"	-0.0563
"00074def99"	"00075b26f1"	-0.0294
"00074def99"	"00075b2920"	-0.3515
"00074def99"	"00075b2a64"	-0.4266
"00074def99"	"00075b2a9d"	-0.1439

"00074def99"	"00075b2b37"	−0.5048
"00074def99"	"00075b2cdd"	0.0059
"00074def99"	"00075b3038"	−0.0548
"00074def99"	"00075b30fe"	−0.0504
"00074def99"	"00075b3362"	−0.0902
"00074def99"	"00075b350a"	−0.2567
"00074def99"	"00075b350e"	−0.0114
"00074def99"	"00075b3651"	0.0367
"00074def99"	"00075b38ca"	−0.3119
"00074def99"	"00075b39cc"	−0.2398
"00074def99"	"00075b3e1e"	−0.2085
"00074def99"	"00075b3e57"	−0.197
"00074def99"	"00075b4079"	−0.2774
"00074def99"	"00075b4150"	−0.2742
"00074def99"	"00075b4194"	−0.2045
"00074def99"	"00075b42d5"	−0.0923
"00074def99"	"00075b4424"	−0.2181
"00074def99"	"00075b4470"	−0.0832
"00074def99"	"00075b47ed"	−0.1853
"00074def99"	"00075b4850"	−0.0013
"00074def99"	"00075b4ca0"	0.0261
"00074def99"	"00075b4d7f"	0.0889
"00074def99"	"00075b520f"	−0.0849
"00074def99"	"00075b525f"	0.1359
"00074def99"	"00075b58f8"	−0.4704
"00074def99"	"00075b5bcc"	0.143
"00074def99"	"00075b5bfa"	−0.2825
"00074def99"	"00075b6339"	0.093
"00074def99"	"00075b6658"	−0.1792
"00074def99"	"00075b679a"	0.3709
"00074def99"	"00075b6cb7"	−0.0462
"00074def99"	"00075b6df8"	0.1141
"00074def99"	"00075b6ff6"	−0.0221
"00074def99"	"00075b70ee"	−0.2177
"00074def99"	"00075b7157"	−0.0915
"00074def99"	"00075b7225"	0.2327
"00074def99"	"00075b7c89"	−0.0695
"00074def99"	"00075b9048"	0.0491
"00074def99"	"00075d0801"	−0.2092
"00074def99"	"00075d1820"	0.0248
"00074def99"	"00075d1f3d"	0.0113
"00074def99"	"00075d2329"	−0.1095
"00074def99"	"00075d2b9b"	−0.259
"00074def99"	"00075d3941"	−0.2915
"00074def99"	"00075d3e96"	−0.0498
"00074def99"	"00075d4864"	−0.2928
"00074def99"	"00075d5961"	−0.0405
"00074def99"	"00075d5a63"	−0.1894
"00074def99"	"00075d6150"	−0.0164
"00074def99"	"00075d67d0"	0.0254

"00074def99"	"00075d67e2"	0.0102
"00074def99"	"00075d73fc"	0.2771
"00074def99"	"00075d7729"	0.3211
"00074def99"	"00075d778c"	-0.1015
"00074def99"	"00075d7b9e"	0.0255
"00074def99"	"00075d7c8f"	0.2176
"00074def99"	"00075d804d"	-0.3119
"00074def99"	"00075d819f"	-0.026
"00074def99"	"00075d8601"	-0.1325
"00074def99"	"00075d8c6a"	0.007
"00074def99"	"00075dfedc"	-0.0073
"00074def99"	"00075e05f2"	-0.1919
"00074def99"	"00075e0837"	-0.0907
"00074def99"	"00075e092e"	-0.0225
"00074def99"	"00075e0965"	-0.5032
"00074def99"	"00075e0bc8"	7e-04
"00074def99"	"00075e0fbb"	-0.1083
"00074ecdad"	"00074ecf28"	-0.0209
"00074ecdad"	"00074ed1e1"	-0.0252
"00074ecdad"	"00074ed83b"	-0.0403
"00074ecdad"	"00074ee5e3"	-0.5075
"00074ecdad"	"00074ee6e0"	-0.4154
"00074ecdad"	"00074eea3a"	0.0764
"00074ecdad"	"00074eff82"	-0.3807
"00074ecdad"	"00074f0477"	-0.3812
"00074ecdad"	"00074f08c3"	-0.4459
"00074ecdad"	"00074f1859"	0.0776
"00074ecdad"	"00074f2268"	-0.3446
"00074ecdad"	"00074f28be"	-0.1395
"00074ecdad"	"00074f294b"	0.0842
"00074ecdad"	"00074f2ddd"	-0.1354
"00074ecdad"	"00074f2e75"	0.0607
"00074ecdad"	"00074f3088"	-0.3157
"00074ecdad"	"00074f5a1c"	0.2504
"00074ecdad"	"00074f75b7"	-0.248
"00074ecdad"	"00074f8cd9"	-0.0343
"00074ecdad"	"00074f96dc"	-0.4326
"00074ecdad"	"00074fabaa"	-0.2252
"00074ecdad"	"00074facd9"	-0.1916
"00074ecdad"	"00074fae3c"	-0.2485
"00074ecdad"	"00074fb0a8"	-0.1692
"00074ecdad"	"00074fb4e4"	-0.4098
"00074ecdad"	"00074fb7c2"	0.0612
"00074ecdad"	"00074fbd36"	-0.4519
"00074ecdad"	"00074fc27f"	-0.1207
"00074ecdad"	"00074fc31d"	-0.2361
"00074ecdad"	"00074fd569"	-0.0414
"00074ecdad"	"00074fef15"	-0.4328
"00074ecdad"	"00074ff562"	-0.0818
"00074ecdad"	"00075007ca"	0.1476

"00074ecdad"	"0007500b86"	−0.3589
"00074ecdad"	"0007500d05"	−0.1584
"00074ecdad"	"0007500ee4"	−0.2283
"00074ecdad"	"0007500eee"	−0.2804
"00074ecdad"	"00075013dc"	−0.4255
"00074ecdad"	"000757b515"	−0.3578
"00074ecdad"	"000757bc5a"	−0.2389
"00074ecdad"	"000757c320"	−0.3682
"00074ecdad"	"000757c9aa"	−0.3824
"00074ecdad"	"000757ccbe"	0.0251
"00074ecdad"	"000757cfa9"	−0.3879
"00074ecdad"	"000757d390"	−0.0228
"00074ecdad"	"000757d393"	−0.0302
"00074ecdad"	"000757d598"	−0.2061
"00074ecdad"	"000757d5a2"	−0.1073
"00074ecdad"	"000757d790"	−0.0944
"00074ecdad"	"000757e30c"	−0.2272
"00074ecdad"	"000757e4b0"	−0.0745
"00074ecdad"	"000757e7a0"	0.0761
"00074ecdad"	"000757e8b3"	−0.252
"00074ecdad"	"000757f627"	−0.2578
"00074ecdad"	"000757f925"	−0.1506
"00074ecdad"	"000757fa08"	0.2168
"00074ecdad"	"000757fe52"	−0.2465
"00074ecdad"	"000758024a"	−0.1018
"00074ecdad"	"00075804bb"	−0.1494
"00074ecdad"	"00075a0c04"	−0.1574
"00074ecdad"	"00075a3110"	−0.1539
"00074ecdad"	"00075a341a"	−0.1395
"00074ecdad"	"00075a3dcf"	0.2692
"00074ecdad"	"00075a3e22"	−0.5171
"00074ecdad"	"00075a48d8"	−0.0988
"00074ecdad"	"00075a5cfb"	−0.2345
"00074ecdad"	"00075a6151"	−0.1916
"00074ecdad"	"00075a6708"	−0.1415
"00074ecdad"	"00075a7319"	−0.1648
"00074ecdad"	"00075a7723"	−0.295
"00074ecdad"	"00075a778b"	−0.1228
"00074ecdad"	"00075a7b8e"	−0.1217
"00074ecdad"	"00075a7c79"	−0.1364
"00074ecdad"	"00075a81b6"	−0.4075
"00074ecdad"	"00075a82ac"	−0.3296
"00074ecdad"	"00075a98e5"	−0.5403
"00074ecdad"	"00075b0d29"	−0.6096
"00074ecdad"	"00075b102a"	−0.0917
"00074ecdad"	"00075b1074"	−0.0223
"00074ecdad"	"00075b135d"	−0.4775
"00074ecdad"	"00075b138b"	−0.1363
"00074ecdad"	"00075b13a0"	−0.3347
"00074ecdad"	"00075b13bd"	−0.5246

"00074ecdad"	"00075b16a9"	0.082
"00074ecdad"	"00075b1a28"	-0.0247
"00074ecdad"	"00075b1a97"	-0.0125
"00074ecdad"	"00075b1c7b"	2e-04
"00074ecdad"	"00075b1d24"	-0.1857
"00074ecdad"	"00075b202b"	0.029
"00074ecdad"	"00075b22cb"	0.0821
"00074ecdad"	"00075b22da"	-0.0752
"00074ecdad"	"00075b2556"	-0.1757
"00074ecdad"	"00075b25de"	-0.3021
"00074ecdad"	"00075b260c"	-0.0424
"00074ecdad"	"00075b26f1"	-0.4088
"00074ecdad"	"00075b2920"	-0.0547
"00074ecdad"	"00075b2a64"	-0.3799
"00074ecdad"	"00075b2a9d"	-0.1633
"00074ecdad"	"00075b2b37"	-0.2099
"00074ecdad"	"00075b2cdd"	0.026
"00074ecdad"	"00075b3038"	-0.1445
"00074ecdad"	"00075b30fe"	0.0544
"00074ecdad"	"00075b3362"	-0.5423
"00074ecdad"	"00075b350a"	-0.3584
"00074ecdad"	"00075b350e"	-0.1998
"00074ecdad"	"00075b3651"	-0.4992
"00074ecdad"	"00075b38ca"	0.0648
"00074ecdad"	"00075b39cc"	-0.1145
"00074ecdad"	"00075b3e1e"	0.2069
"00074ecdad"	"00075b3e57"	-0.1037
"00074ecdad"	"00075b4079"	-0.4856
"00074ecdad"	"00075b4150"	0.0237
"00074ecdad"	"00075b4194"	-0.2868
"00074ecdad"	"00075b42d5"	0.2339
"00074ecdad"	"00075b4424"	0.0336
"00074ecdad"	"00075b4470"	-0.1478
"00074ecdad"	"00075b47ed"	-0.0708
"00074ecdad"	"00075b4850"	-0.1731
"00074ecdad"	"00075b4ca0"	-0.4138
"00074ecdad"	"00075b4d7f"	-0.0982
"00074ecdad"	"00075b520f"	-0.1605
"00074ecdad"	"00075b525f"	-0.2607
"00074ecdad"	"00075b58f8"	-0.2305
"00074ecdad"	"00075b5bcc"	-0.3233
"00074ecdad"	"00075b5bfa"	-0.3568
"00074ecdad"	"00075b6339"	-0.1509
"00074ecdad"	"00075b6658"	-0.4158
"00074ecdad"	"00075b679a"	-0.0221
"00074ecdad"	"00075b6cb7"	-0.4389
"00074ecdad"	"00075b6df8"	-0.0168
"00074ecdad"	"00075b6ff6"	-0.0609
"00074ecdad"	"00075b70ee"	-0.0977
"00074ecdad"	"00075b7157"	-0.3241

"00074ecdad"	"00075b7225"	−0.1967
"00074ecdad"	"00075b7c89"	−0.3289
"00074ecdad"	"00075b9048"	−0.1392
"00074ecdad"	"00075d0801"	−0.3834
"00074ecdad"	"00075d1820"	−0.3717
"00074ecdad"	"00075d1f3d"	−0.1621
"00074ecdad"	"00075d2329"	0.0422
"00074ecdad"	"00075d2b9b"	−0.1964
"00074ecdad"	"00075d3941"	−0.3652
"00074ecdad"	"00075d3e96"	−0.1987
"00074ecdad"	"00075d4864"	0.2742
"00074ecdad"	"00075d5961"	−0.2065
"00074ecdad"	"00075d5a63"	−0.0331
"00074ecdad"	"00075d6150"	0.1369
"00074ecdad"	"00075d67d0"	−0.2151
"00074ecdad"	"00075d67e2"	−0.2862
"00074ecdad"	"00075d73fc"	−0.1007
"00074ecdad"	"00075d7729"	−0.3767
"00074ecdad"	"00075d778c"	−0.2358
"00074ecdad"	"00075d7b9e"	−0.3226
"00074ecdad"	"00075d7c8f"	−0.2123
"00074ecdad"	"00075d804d"	−0.3148
"00074ecdad"	"00075d819f"	−0.2711
"00074ecdad"	"00075d8601"	−0.2035
"00074ecdad"	"00075d8c6a"	−0.3321
"00074ecdad"	"00075dfedc"	−0.1786
"00074ecdad"	"00075e05f2"	−0.0885
"00074ecdad"	"00075e0837"	0.0089
"00074ecdad"	"00075e092e"	−0.142
"00074ecdad"	"00075e0965"	−0.1357
"00074ecdad"	"00075e0bc8"	−0.2096
"00074ecdad"	"00075e0fbb"	−0.2008
"00074ecf28"	"00074ed1e1"	−0.1664
"00074ecf28"	"00074ed83b"	−0.2081
"00074ecf28"	"00074ee5e3"	−0.3727
"00074ecf28"	"00074ee6e0"	−0.2332
"00074ecf28"	"00074eea3a"	−0.3155
"00074ecf28"	"00074eff82"	−0.106
"00074ecf28"	"00074f0477"	−0.2956
"00074ecf28"	"00074f08c3"	−0.2279
"00074ecf28"	"00074f1859"	−0.148
"00074ecf28"	"00074f2268"	−0.3968
"00074ecf28"	"00074f28be"	−0.3581
"00074ecf28"	"00074f294b"	−0.2531
"00074ecf28"	"00074f2ddd"	−0.1421
"00074ecf28"	"00074f2e75"	−0.1922
"00074ecf28"	"00074f3088"	0.1236
"00074ecf28"	"00074f5a1c"	−0.2627
"00074ecf28"	"00074f75b7"	−0.2277
"00074ecf28"	"00074f8cd9"	−0.0544

"00074ecf28"	"00074f96dc"	0.1609
"00074ecf28"	"00074fabaa"	−0.4416
"00074ecf28"	"00074facd9"	−0.2632
"00074ecf28"	"00074fae3c"	0.1465
"00074ecf28"	"00074fb0a8"	−0.2188
"00074ecf28"	"00074fb4e4"	−0.1641
"00074ecf28"	"00074fb7c2"	0.0297
"00074ecf28"	"00074fbd36"	−0.3501
"00074ecf28"	"00074fc27f"	−0.2394
"00074ecf28"	"00074fc31d"	−0.0539
"00074ecf28"	"00074fd569"	0.4053
"00074ecf28"	"00074fef15"	0.0297
"00074ecf28"	"00074ff562"	−0.0563
"00074ecf28"	"00075007ca"	−0.1064
"00074ecf28"	"0007500b86"	−0.3433
"00074ecf28"	"0007500d05"	0.0415
"00074ecf28"	"0007500ee4"	0.129
"00074ecf28"	"0007500eee"	−0.2952
"00074ecf28"	"00075013dc"	−0.2776
"00074ecf28"	"000757b515"	−0.3372
"00074ecf28"	"000757bc5a"	−0.3329
"00074ecf28"	"000757c320"	−0.0128
"00074ecf28"	"000757c9aa"	−0.2477
"00074ecf28"	"000757ccbe"	−0.0859
"00074ecf28"	"000757cfa9"	−0.2139
"00074ecf28"	"000757d390"	−0.2293
"00074ecf28"	"000757d393"	−0.0744
"00074ecf28"	"000757d598"	−0.1312
"00074ecf28"	"000757d5a2"	−0.2296
"00074ecf28"	"000757d790"	−0.211
"00074ecf28"	"000757e30c"	−0.2675
"00074ecf28"	"000757e4b0"	−0.1719
"00074ecf28"	"000757e7a0"	0.0038
"00074ecf28"	"000757e8b3"	−0.2174
"00074ecf28"	"000757f627"	−0.1728
"00074ecf28"	"000757f925"	−0.0993
"00074ecf28"	"000757fa08"	−0.2078
"00074ecf28"	"000757fe52"	−0.0177
"00074ecf28"	"000758024a"	−0.1865
"00074ecf28"	"00075804bb"	−0.3778
"00074ecf28"	"00075a0c04"	0.0259
"00074ecf28"	"00075a3110"	−0.1274
"00074ecf28"	"00075a341a"	0.0637
"00074ecf28"	"00075a3dcf"	−0.0378
"00074ecf28"	"00075a3e22"	−0.2523
"00074ecf28"	"00075a48d8"	−0.0305
"00074ecf28"	"00075a5cfb"	−0.2316
"00074ecf28"	"00075a6151"	−0.5608
"00074ecf28"	"00075a6708"	−0.3295
"00074ecf28"	"00075a7319"	−0.2789

"00074ecf28"	"00075a7723"	-0.2518
"00074ecf28"	"00075a778b"	-0.0755
"00074ecf28"	"00075a7b8e"	-0.1982
"00074ecf28"	"00075a7c79"	-0.4018
"00074ecf28"	"00075a81b6"	-0.2788
"00074ecf28"	"00075a82ac"	-0.0106
"00074ecf28"	"00075a98e5"	-0.0479
"00074ecf28"	"00075b0d29"	-0.143
"00074ecf28"	"00075b102a"	-0.3207
"00074ecf28"	"00075b1074"	-0.2599
"00074ecf28"	"00075b135d"	-0.266
"00074ecf28"	"00075b138b"	-0.186
"00074ecf28"	"00075b13a0"	-0.2187
"00074ecf28"	"00075b13bd"	0.1904
"00074ecf28"	"00075b16a9"	0.0108
"00074ecf28"	"00075b1a28"	0.045
"00074ecf28"	"00075b1a97"	0.0977
"00074ecf28"	"00075b1c7b"	0.0631
"00074ecf28"	"00075b1d24"	-0.1698
"00074ecf28"	"00075b202b"	-0.0459
"00074ecf28"	"00075b22cb"	0.2074
"00074ecf28"	"00075b22da"	-0.1717
"00074ecf28"	"00075b2556"	-0.1643
"00074ecf28"	"00075b25de"	-0.1971
"00074ecf28"	"00075b260c"	-0.3545
"00074ecf28"	"00075b26f1"	-0.0988
"00074ecf28"	"00075b2920"	-0.1826
"00074ecf28"	"00075b2a64"	-0.4565
"00074ecf28"	"00075b2a9d"	-0.2762
"00074ecf28"	"00075b2b37"	0.3712
"00074ecf28"	"00075b2cdd"	0.1547
"00074ecf28"	"00075b3038"	-0.0268
"00074ecf28"	"00075b30fe"	-0.123
"00074ecf28"	"00075b3362"	-0.1543
"00074ecf28"	"00075b350a"	-0.1957
"00074ecf28"	"00075b350e"	-0.1205
"00074ecf28"	"00075b3651"	-0.2307
"00074ecf28"	"00075b38ca"	-0.4217
"00074ecf28"	"00075b39cc"	-0.21
"00074ecf28"	"00075b3e1e"	-0.1088
"00074ecf28"	"00075b3e57"	0.5031
"00074ecf28"	"00075b4079"	-0.1182
"00074ecf28"	"00075b4150"	-0.0574
"00074ecf28"	"00075b4194"	-0.3113
"00074ecf28"	"00075b42d5"	-0.3398
"00074ecf28"	"00075b4424"	0.008
"00074ecf28"	"00075b4470"	-0.3329
"00074ecf28"	"00075b47ed"	-0.1179
"00074ecf28"	"00075b4850"	-0.3405
"00074ecf28"	"00075b4ca0"	-0.3056

"00074ecf28"	"00075b4d7f"	0.0113
"00074ecf28"	"00075b520f"	0.2825
"00074ecf28"	"00075b525f"	−0.0303
"00074ecf28"	"00075b58f8"	−0.2753
"00074ecf28"	"00075b5bcc"	−0.0492
"00074ecf28"	"00075b5bfa"	−0.1991
"00074ecf28"	"00075b6339"	−0.1444
"00074ecf28"	"00075b6658"	0.0049
"00074ecf28"	"00075b679a"	0.1879
"00074ecf28"	"00075b6cb7"	−0.6255
"00074ecf28"	"00075b6df8"	0.112
"00074ecf28"	"00075b6ff6"	−0.0898
"00074ecf28"	"00075b70ee"	−0.3361
"00074ecf28"	"00075b7157"	−0.0029
"00074ecf28"	"00075b7225"	−0.1987
"00074ecf28"	"00075b7c89"	−0.1691
"00074ecf28"	"00075b9048"	−0.1164
"00074ecf28"	"00075d0801"	−0.0174
"00074ecf28"	"00075d1820"	0.0424
"00074ecf28"	"00075d1f3d"	−0.3596
"00074ecf28"	"00075d2329"	−0.0734
"00074ecf28"	"00075d2b9b"	−0.4253
"00074ecf28"	"00075d3941"	−0.0597
"00074ecf28"	"00075d3e96"	−0.0308
"00074ecf28"	"00075d4864"	−0.121
"00074ecf28"	"00075d5961"	0.106
"00074ecf28"	"00075d5a63"	0.0974
"00074ecf28"	"00075d6150"	−0.1022
"00074ecf28"	"00075d67d0"	−0.094
"00074ecf28"	"00075d67e2"	−0.1394
"00074ecf28"	"00075d73fc"	−0.0446
"00074ecf28"	"00075d7729"	0.1938
"00074ecf28"	"00075d778c"	−0.1067
"00074ecf28"	"00075d7b9e"	−0.0025
"00074ecf28"	"00075d7c8f"	−0.1652
"00074ecf28"	"00075d804d"	−0.1088
"00074ecf28"	"00075d819f"	−0.3814
"00074ecf28"	"00075d8601"	0.2028
"00074ecf28"	"00075d8c6a"	−0.1604
"00074ecf28"	"00075dfedc"	−0.1956
"00074ecf28"	"00075e05f2"	−0.4899
"00074ecf28"	"00075e0837"	−0.2596
"00074ecf28"	"00075e092e"	−0.2098
"00074ecf28"	"00075e0965"	−0.3449
"00074ecf28"	"00075e0bc8"	−0.1782
"00074ecf28"	"00075e0fbb"	−0.2467
"00074ed1e1"	"00074ed83b"	−0.0369
"00074ed1e1"	"00074ee5e3"	−0.3674
"00074ed1e1"	"00074ee6e0"	−0.2833
"00074ed1e1"	"00074eea3a"	0.3001

"00074ed1e1"	"00074eff82"	−0.0181
"00074ed1e1"	"00074f0477"	−0.0321
"00074ed1e1"	"00074f08c3"	−0.3024
"00074ed1e1"	"00074f1859"	−0.1187
"00074ed1e1"	"00074f2268"	−0.3199
"00074ed1e1"	"00074f28be"	0.1393
"00074ed1e1"	"00074f294b"	0.1161
"00074ed1e1"	"00074f2ddd"	−0.0381
"00074ed1e1"	"00074f2e75"	−0.0572
"00074ed1e1"	"00074f3088"	−0.2345
"00074ed1e1"	"00074f5a1c"	0.041
"00074ed1e1"	"00074f75b7"	−0.1938
"00074ed1e1"	"00074f8cd9"	0.1328
"00074ed1e1"	"00074f96dc"	−0.4586
"00074ed1e1"	"00074fabaa"	−0.1628
"00074ed1e1"	"00074facd9"	−0.1842
"00074ed1e1"	"00074fae3c"	−0.331
"00074ed1e1"	"00074fb0a8"	−0.0172
"00074ed1e1"	"00074fb4e4"	−0.3169
"00074ed1e1"	"00074fb7c2"	0.2853
"00074ed1e1"	"00074fbd36"	−0.3743
"00074ed1e1"	"00074fc27f"	0.1039
"00074ed1e1"	"00074fc31d"	−0.0426
"00074ed1e1"	"00074fd569"	−0.0352
"00074ed1e1"	"00074fef15"	−0.078
"00074ed1e1"	"00074ff562"	0.1986
"00074ed1e1"	"00075007ca"	−0.174
"00074ed1e1"	"0007500b86"	−0.1156
"00074ed1e1"	"0007500d05"	0.2128
"00074ed1e1"	"0007500ee4"	0.0869
"00074ed1e1"	"0007500eee"	−0.288
"00074ed1e1"	"00075013dc"	−0.0509
"00074ed1e1"	"000757b515"	0.1076
"00074ed1e1"	"000757bc5a"	−0.1514
"00074ed1e1"	"000757c320"	−0.4055
"00074ed1e1"	"000757c9aa"	−0.1942
"00074ed1e1"	"000757ccbe"	−0.0108
"00074ed1e1"	"000757cfa9"	−0.3597
"00074ed1e1"	"000757d390"	−0.2163
"00074ed1e1"	"000757d393"	−0.019
"00074ed1e1"	"000757d598"	−0.0701
"00074ed1e1"	"000757d5a2"	0.0921
"00074ed1e1"	"000757d790"	0.1071
"00074ed1e1"	"000757e30c"	−0.2339
"00074ed1e1"	"000757e4b0"	0.1629
"00074ed1e1"	"000757e7a0"	−0.1254
"00074ed1e1"	"000757e8b3"	−0.0142
"00074ed1e1"	"000757f627"	−0.1025
"00074ed1e1"	"000757f925"	−0.008
"00074ed1e1"	"000757fa08"	−0.1549

"00074ed1e1"	"000757fe52"	0.0065
"00074ed1e1"	"000758024a"	−0.2859
"00074ed1e1"	"00075804bb"	−0.3736
"00074ed1e1"	"00075a0c04"	0.2994
"00074ed1e1"	"00075a3110"	0.0331
"00074ed1e1"	"00075a341a"	0.1214
"00074ed1e1"	"00075a3dcf"	0.1636
"00074ed1e1"	"00075a3e22"	0.1232
"00074ed1e1"	"00075a48d8"	0.0929
"00074ed1e1"	"00075a5cfb"	−0.3321
"00074ed1e1"	"00075a6151"	−0.1083
"00074ed1e1"	"00075a6708"	−0.2273
"00074ed1e1"	"00075a7319"	−0.1786
"00074ed1e1"	"00075a7723"	−0.0057
"00074ed1e1"	"00075a778b"	0.208
"00074ed1e1"	"00075a7b8e"	0.1072
"00074ed1e1"	"00075a7c79"	0.2835
"00074ed1e1"	"00075a81b6"	−0.0098
"00074ed1e1"	"00075a82ac"	0.1375
"00074ed1e1"	"00075a98e5"	−0.0426
"00074ed1e1"	"00075b0d29"	−0.2638
"00074ed1e1"	"00075b102a"	−0.1913
"00074ed1e1"	"00075b1074"	0.1305
"00074ed1e1"	"00075b135d"	−0.0865
"00074ed1e1"	"00075b138b"	0.2591
"00074ed1e1"	"00075b13a0"	−0.1877
"00074ed1e1"	"00075b13bd"	−0.2699
"00074ed1e1"	"00075b16a9"	−0.0758
"00074ed1e1"	"00075b1a28"	−0.1998
"00074ed1e1"	"00075b1a97"	0.0225
"00074ed1e1"	"00075b1c7b"	−0.4245
"00074ed1e1"	"00075b1d24"	−0.1119
"00074ed1e1"	"00075b202b"	−0.0585
"00074ed1e1"	"00075b22cb"	−0.0365
"00074ed1e1"	"00075b22da"	0.1148
"00074ed1e1"	"00075b2556"	−0.0819
"00074ed1e1"	"00075b25de"	−0.088
"00074ed1e1"	"00075b260c"	−0.0685
"00074ed1e1"	"00075b26f1"	−0.1365
"00074ed1e1"	"00075b2920"	−0.0734
"00074ed1e1"	"00075b2a64"	−0.1703
"00074ed1e1"	"00075b2a9d"	−0.0151
"00074ed1e1"	"00075b2b37"	−0.4515
"00074ed1e1"	"00075b2cdd"	0.0831
"00074ed1e1"	"00075b3038"	0.1832
"00074ed1e1"	"00075b30fe"	0.0838
"00074ed1e1"	"00075b3362"	−0.3661
"00074ed1e1"	"00075b350a"	−0.2451
"00074ed1e1"	"00075b350e"	−3e−04
"00074ed1e1"	"00075b3651"	−0.1869

"00074ed1e1"	"00075b38ca"	−0.137
"00074ed1e1"	"00075b39cc"	0.2691
"00074ed1e1"	"00075b3e1e"	0.064
"00074ed1e1"	"00075b3e57"	−0.3077
"00074ed1e1"	"00075b4079"	−0.1686
"00074ed1e1"	"00075b4150"	−0.1412
"00074ed1e1"	"00075b4194"	0.0814
"00074ed1e1"	"00075b42d5"	0.1014
"00074ed1e1"	"00075b4424"	−0.1446
"00074ed1e1"	"00075b4470"	−0.1514
"00074ed1e1"	"00075b47ed"	0.4042
"00074ed1e1"	"00075b4850"	0.0719
"00074ed1e1"	"00075b4ca0"	0.0577
"00074ed1e1"	"00075b4d7f"	0.1611
"00074ed1e1"	"00075b520f"	−0.0345
"00074ed1e1"	"00075b525f"	−0.0783
"00074ed1e1"	"00075b58f8"	−0.3962
"00074ed1e1"	"00075b5bcc"	−0.084
"00074ed1e1"	"00075b5bfa"	−0.218
"00074ed1e1"	"00075b6339"	−0.0569
"00074ed1e1"	"00075b6658"	−0.4066
"00074ed1e1"	"00075b679a"	−0.0121
"00074ed1e1"	"00075b6cb7"	−0.0349
"00074ed1e1"	"00075b6df8"	0.1341
"00074ed1e1"	"00075b6ff6"	0.1074
"00074ed1e1"	"00075b70ee"	0.0365
"00074ed1e1"	"00075b7157"	−0.0225
"00074ed1e1"	"00075b7225"	0.1101
"00074ed1e1"	"00075b7c89"	−0.0253
"00074ed1e1"	"00075b9048"	0.1363
"00074ed1e1"	"00075d0801"	0.0616
"00074ed1e1"	"00075d1820"	−0.167
"00074ed1e1"	"00075d1f3d"	−0.0736
"00074ed1e1"	"00075d2329"	0.3088
"00074ed1e1"	"00075d2b9b"	−0.0474
"00074ed1e1"	"00075d3941"	−0.1853
"00074ed1e1"	"00075d3e96"	−0.0859
"00074ed1e1"	"00075d4864"	−0.1073
"00074ed1e1"	"00075d5961"	0.1265
"00074ed1e1"	"00075d5a63"	−0.0269
"00074ed1e1"	"00075d6150"	−0.0375
"00074ed1e1"	"00075d67d0"	−0.1704
"00074ed1e1"	"00075d67e2"	−0.135
"00074ed1e1"	"00075d73fc"	0.0266
"00074ed1e1"	"00075d7729"	−0.2475
"00074ed1e1"	"00075d778c"	−0.2477
"00074ed1e1"	"00075d7b9e"	−0.2726
"00074ed1e1"	"00075d7c8f"	0.1091
"00074ed1e1"	"00075d804d"	0.0504
"00074ed1e1"	"00075d819f"	−0.1343

"00074ed1e1"	"00075d8601"	-0.2086
"00074ed1e1"	"00075d8c6a"	0.13
"00074ed1e1"	"00075dfedc"	0.036
"00074ed1e1"	"00075e05f2"	-0.0402
"00074ed1e1"	"00075e0837"	0.084
"00074ed1e1"	"00075e092e"	0.1287
"00074ed1e1"	"00075e0965"	-0.3306
"00074ed1e1"	"00075e0bc8"	0.01
"00074ed1e1"	"00075e0fbb"	0.0948
"00074ed83b"	"00074ee5e3"	-0.1658
"00074ed83b"	"00074ee6e0"	-0.1265
"00074ed83b"	"00074eea3a"	0.1574
"00074ed83b"	"00074eff82"	-0.1626
"00074ed83b"	"00074f0477"	-0.1898
"00074ed83b"	"00074f08c3"	0.001
"00074ed83b"	"00074f1859"	0.1302
"00074ed83b"	"00074f2268"	-0.0934
"00074ed83b"	"00074f28be"	0.2309
"00074ed83b"	"00074f294b"	0.1655
"00074ed83b"	"00074f2ddd"	0.116
"00074ed83b"	"00074f2e75"	-0.1447
"00074ed83b"	"00074f3088"	0.0377
"00074ed83b"	"00074f5a1c"	0.052
"00074ed83b"	"00074f75b7"	-0.0362
"00074ed83b"	"00074f8cd9"	-0.0125
"00074ed83b"	"00074f96dc"	-0.1621
"00074ed83b"	"00074fabaa"	0.1105
"00074ed83b"	"00074facd9"	-0.083
"00074ed83b"	"00074fae3c"	0.0415
"00074ed83b"	"00074fb0a8"	0.1178
"00074ed83b"	"00074fb4e4"	0.0361
"00074ed83b"	"00074fb7c2"	0.2792
"00074ed83b"	"00074fbd36"	0.0809
"00074ed83b"	"00074fc27f"	0.3451
"00074ed83b"	"00074fc31d"	0.1889
"00074ed83b"	"00074fd569"	0.1974
"00074ed83b"	"00074fef15"	0.0674
"00074ed83b"	"00074ff562"	-0.228
"00074ed83b"	"00075007ca"	0.145
"00074ed83b"	"0007500b86"	0.1326
"00074ed83b"	"0007500d05"	-0.1003
"00074ed83b"	"0007500ee4"	-0.12
"00074ed83b"	"0007500eee"	-0.1285
"00074ed83b"	"00075013dc"	-0.0889
"00074ed83b"	"000757b515"	0.182
"00074ed83b"	"000757bc5a"	0.0535
"00074ed83b"	"000757c320"	-0.0313
"00074ed83b"	"000757c9aa"	0.3891
"00074ed83b"	"000757ccbe"	0.1764
"00074ed83b"	"000757cfa9"	-0.1817

"00074ed83b"	"000757d390"	−0.0778
"00074ed83b"	"000757d393"	0.0111
"00074ed83b"	"000757d598"	−0.0022
"00074ed83b"	"000757d5a2"	0.1229
"00074ed83b"	"000757d790"	0.1561
"00074ed83b"	"000757e30c"	0.0787
"00074ed83b"	"000757e4b0"	0.3054
"00074ed83b"	"000757e7a0"	0.1231
"00074ed83b"	"000757e8b3"	0.2832
"00074ed83b"	"000757f627"	0.2674
"00074ed83b"	"000757f925"	0.2182
"00074ed83b"	"000757fa08"	−0.1074
"00074ed83b"	"000757fe52"	−0.1534
"00074ed83b"	"000758024a"	0.0985
"00074ed83b"	"00075804bb"	0.0596
"00074ed83b"	"00075a0c04"	0.1193
"00074ed83b"	"00075a3110"	0.0761
"00074ed83b"	"00075a341a"	0.0879
"00074ed83b"	"00075a3dcf"	−0.0339
"00074ed83b"	"00075a3e22"	0.2238
"00074ed83b"	"00075a48d8"	0.0584
"00074ed83b"	"00075a5cfb"	0.1699
"00074ed83b"	"00075a6151"	0.1823
"00074ed83b"	"00075a6708"	−0.0511
"00074ed83b"	"00075a7319"	0.049
"00074ed83b"	"00075a7723"	0.3465
"00074ed83b"	"00075a778b"	−0.1622
"00074ed83b"	"00075a7b8e"	0.015
"00074ed83b"	"00075a7c79"	0.1907
"00074ed83b"	"00075a81b6"	0.0566
"00074ed83b"	"00075a82ac"	0.1049
"00074ed83b"	"00075a98e5"	−0.0628
"00074ed83b"	"00075b0d29"	−0.0659
"00074ed83b"	"00075b102a"	0.1186
"00074ed83b"	"00075b1074"	−0.0261
"00074ed83b"	"00075b135d"	0.1715
"00074ed83b"	"00075b138b"	0.2985
"00074ed83b"	"00075b13a0"	−0.1884
"00074ed83b"	"00075b13bd"	−0.0012
"00074ed83b"	"00075b16a9"	0.0723
"00074ed83b"	"00075b1a28"	−0.0369
"00074ed83b"	"00075b1a97"	0.1248
"00074ed83b"	"00075b1c7b"	−0.1695
"00074ed83b"	"00075b1d24"	0.0093
"00074ed83b"	"00075b202b"	0.0779
"00074ed83b"	"00075b22cb"	−0.1353
"00074ed83b"	"00075b22da"	0.0756
"00074ed83b"	"00075b2556"	0.1307
"00074ed83b"	"00075b25de"	−0.1473
"00074ed83b"	"00075b260c"	0.2353

"00074ed83b"	"00075b26f1"	0.1865
"00074ed83b"	"00075b2920"	0.0551
"00074ed83b"	"00075b2a64"	0.1066
"00074ed83b"	"00075b2a9d"	0.0089
"00074ed83b"	"00075b2b37"	−0.2106
"00074ed83b"	"00075b2cdd"	−0.0849
"00074ed83b"	"00075b3038"	0.1552
"00074ed83b"	"00075b30fe"	0.0195
"00074ed83b"	"00075b3362"	0.0103
"00074ed83b"	"00075b350a"	−0.2223
"00074ed83b"	"00075b350e"	0.0893
"00074ed83b"	"00075b3651"	0.0349
"00074ed83b"	"00075b38ca"	−0.1329
"00074ed83b"	"00075b39cc"	−0.0644
"00074ed83b"	"00075b3e1e"	0.2073
"00074ed83b"	"00075b3e57"	−0.0237
"00074ed83b"	"00075b4079"	−0.2138
"00074ed83b"	"00075b4150"	0.1231
"00074ed83b"	"00075b4194"	0.2703
"00074ed83b"	"00075b42d5"	0.0899
"00074ed83b"	"00075b4424"	0.008
"00074ed83b"	"00075b4470"	0.0535
"00074ed83b"	"00075b47ed"	−0.0777
"00074ed83b"	"00075b4850"	0.213
"00074ed83b"	"00075b4ca0"	0.1036
"00074ed83b"	"00075b4d7f"	0.0622
"00074ed83b"	"00075b520f"	−0.1076
"00074ed83b"	"00075b525f"	0.1432
"00074ed83b"	"00075b58f8"	−0.0693
"00074ed83b"	"00075b5bcc"	−0.0085
"00074ed83b"	"00075b5bfa"	−0.1027
"00074ed83b"	"00075b6339"	0.2451
"00074ed83b"	"00075b6658"	−0.2382
"00074ed83b"	"00075b679a"	0.1252
"00074ed83b"	"00075b6cb7"	0.0646
"00074ed83b"	"00075b6df8"	−0.0094
"00074ed83b"	"00075b6ff6"	0.1291
"00074ed83b"	"00075b70ee"	0.0318
"00074ed83b"	"00075b7157"	−0.0769
"00074ed83b"	"00075b7225"	0.0667
"00074ed83b"	"00075b7c89"	−0.0487
"00074ed83b"	"00075b9048"	0.3548
"00074ed83b"	"00075d0801"	−0.1126
"00074ed83b"	"00075d1820"	0.0849
"00074ed83b"	"00075d1f3d"	0.214
"00074ed83b"	"00075d2329"	−0.0342
"00074ed83b"	"00075d2b9b"	0.1612
"00074ed83b"	"00075d3941"	−0.0227
"00074ed83b"	"00075d3e96"	0.2066
"00074ed83b"	"00075d4864"	−0.158

"00074ed83b"	"00075d5961"	0.0622
"00074ed83b"	"00075d5a63"	0.0367
"00074ed83b"	"00075d6150"	0.0347
"00074ed83b"	"00075d67d0"	0.0223
"00074ed83b"	"00075d67e2"	0.084
"00074ed83b"	"00075d73fc"	0.0421
"00074ed83b"	"00075d7729"	−0.008
"00074ed83b"	"00075d778c"	−0.0544
"00074ed83b"	"00075d7b9e"	8e−04
"00074ed83b"	"00075d7c8f"	0.183
"00074ed83b"	"00075d804d"	0.0846
"00074ed83b"	"00075d819f"	0.1309
"00074ed83b"	"00075d8601"	0.0019
"00074ed83b"	"00075d8c6a"	0.1126
"00074ed83b"	"00075dfedc"	−0.0221
"00074ed83b"	"00075e05f2"	0.1621
"00074ed83b"	"00075e0837"	0.0791
"00074ed83b"	"00075e092e"	0.0483
"00074ed83b"	"00075e0965"	−0.0899
"00074ed83b"	"00075e0bc8"	0.151
"00074ed83b"	"00075e0fbb"	−0.052
"00074ee5e3"	"00074ee6e0"	0.2176
"00074ee5e3"	"00074eea3a"	−0.1645
"00074ee5e3"	"00074eff82"	0.0938
"00074ee5e3"	"00074f0477"	0.0577
"00074ee5e3"	"00074f08c3"	−0.0331
"00074ee5e3"	"00074f1859"	0.1084
"00074ee5e3"	"00074f2268"	−0.4866
"00074ee5e3"	"00074f28be"	−0.1288
"00074ee5e3"	"00074f294b"	−0.3632
"00074ee5e3"	"00074f2ddd"	−0.4506
"00074ee5e3"	"00074f2e75"	−0.7204
"00074ee5e3"	"00074f3088"	0.1001
"00074ee5e3"	"00074f5a1c"	−0.154
"00074ee5e3"	"00074f75b7"	0.4358
"00074ee5e3"	"00074f8cd9"	0.1674
"00074ee5e3"	"00074f96dc"	−0.0898
"00074ee5e3"	"00074fabaa"	0.0111
"00074ee5e3"	"00074facd9"	0.3729
"00074ee5e3"	"00074fae3c"	0.1623
"00074ee5e3"	"00074fb0a8"	−0.0805
"00074ee5e3"	"00074fb4e4"	0.0226
"00074ee5e3"	"00074fb7c2"	−0.2928
"00074ee5e3"	"00074fbd36"	−0.2544
"00074ee5e3"	"00074fc27f"	−0.0411
"00074ee5e3"	"00074fc31d"	−0.1706
"00074ee5e3"	"00074fd569"	−0.0857
"00074ee5e3"	"00074fef15"	−0.1329
"00074ee5e3"	"00074ff562"	−0.3105
"00074ee5e3"	"00075007ca"	−0.2267

"00074ee5e3"	"0007500b86"	0.3074
"00074ee5e3"	"0007500d05"	0.3164
"00074ee5e3"	"0007500ee4"	−0.0714
"00074ee5e3"	"0007500eee"	0.3595
"00074ee5e3"	"00075013dc"	−0.4647
"00074ee5e3"	"000757b515"	0.0146
"00074ee5e3"	"000757bc5a"	0.1067
"00074ee5e3"	"000757c320"	0.1054
"00074ee5e3"	"000757c9aa"	0.1238
"00074ee5e3"	"000757ccbe"	−0.4628
"00074ee5e3"	"000757cfa9"	−0.1794
"00074ee5e3"	"000757d390"	−0.1729
"00074ee5e3"	"000757d393"	0.0465
"00074ee5e3"	"000757d598"	−0.2213
"00074ee5e3"	"000757d5a2"	−0.327
"00074ee5e3"	"000757d790"	0.0366
"00074ee5e3"	"000757e30c"	−0.0747
"00074ee5e3"	"000757e4b0"	0.0718
"00074ee5e3"	"000757e7a0"	−0.1031
"00074ee5e3"	"000757e8b3"	−0.0742
"00074ee5e3"	"000757f627"	0.0172
"00074ee5e3"	"000757f925"	0.0822
"00074ee5e3"	"000757fa08"	−0.5077
"00074ee5e3"	"000757fe52"	0.0473
"00074ee5e3"	"000758024a"	−0.3787
"00074ee5e3"	"00075804bb"	−0.0537
"00074ee5e3"	"00075a0c04"	−0.2503
"00074ee5e3"	"00075a3110"	−0.0552
"00074ee5e3"	"00075a341a"	−0.361
"00074ee5e3"	"00075a3dcf"	−0.1062
"00074ee5e3"	"00075a3e22"	−0.2111
"00074ee5e3"	"00075a48d8"	−0.1534
"00074ee5e3"	"00075a5cfb"	−0.2541
"00074ee5e3"	"00075a6151"	0.0124
"00074ee5e3"	"00075a6708"	0.0086
"00074ee5e3"	"00075a7319"	−0.1868
"00074ee5e3"	"00075a7723"	−0.3457
"00074ee5e3"	"00075a778b"	−0.5124
"00074ee5e3"	"00075a7b8e"	0.027
"00074ee5e3"	"00075a7c79"	−0.0657
"00074ee5e3"	"00075a81b6"	−0.2857
"00074ee5e3"	"00075a82ac"	−0.0411
"00074ee5e3"	"00075a98e5"	−0.2568
"00074ee5e3"	"00075b0d29"	−0.0665
"00074ee5e3"	"00075b102a"	0.0413
"00074ee5e3"	"00075b1074"	−0.4934
"00074ee5e3"	"00075b135d"	−0.4748
"00074ee5e3"	"00075b138b"	0.1073
"00074ee5e3"	"00075b13a0"	−0.4214
"00074ee5e3"	"00075b13bd"	−0.1109

"00074ee5e3"	"00075b16a9"	-0.0118
"00074ee5e3"	"00075b1a28"	-0.0871
"00074ee5e3"	"00075b1a97"	-0.1203
"00074ee5e3"	"00075b1c7b"	-0.238
"00074ee5e3"	"00075b1d24"	-0.011
"00074ee5e3"	"00075b202b"	0.0615
"00074ee5e3"	"00075b22cb"	0.0549
"00074ee5e3"	"00075b22da"	-0.3167
"00074ee5e3"	"00075b2556"	-0.3181
"00074ee5e3"	"00075b25de"	0.4693
"00074ee5e3"	"00075b260c"	-0.1955
"00074ee5e3"	"00075b26f1"	0.0563
"00074ee5e3"	"00075b2920"	-0.0566
"00074ee5e3"	"00075b2a64"	-0.0209
"00074ee5e3"	"00075b2a9d"	-0.0178
"00074ee5e3"	"00075b2b37"	-0.3431
"00074ee5e3"	"00075b2cdd"	-0.0554
"00074ee5e3"	"00075b3038"	0.3645
"00074ee5e3"	"00075b30fe"	-0.264
"00074ee5e3"	"00075b3362"	-0.2248
"00074ee5e3"	"00075b350a"	-0.1811
"00074ee5e3"	"00075b350e"	-0.2413
"00074ee5e3"	"00075b3651"	0.1579
"00074ee5e3"	"00075b38ca"	-0.1493
"00074ee5e3"	"00075b39cc"	0.1462
"00074ee5e3"	"00075b3e1e"	0.0654
"00074ee5e3"	"00075b3e57"	-0.0442
"00074ee5e3"	"00075b4079"	-0.0217
"00074ee5e3"	"00075b4150"	-0.4134
"00074ee5e3"	"00075b4194"	0.0685
"00074ee5e3"	"00075b42d5"	-0.0102
"00074ee5e3"	"00075b4424"	-0.1812
"00074ee5e3"	"00075b4470"	0.0911
"00074ee5e3"	"00075b47ed"	-0.0193
"00074ee5e3"	"00075b4850"	0.1366
"00074ee5e3"	"00075b4ca0"	-0.3766
"00074ee5e3"	"00075b4d7f"	-0.0898
"00074ee5e3"	"00075b520f"	-0.0358
"00074ee5e3"	"00075b525f"	-0.2676
"00074ee5e3"	"00075b58f8"	-0.075
"00074ee5e3"	"00075b5bcc"	0.0561
"00074ee5e3"	"00075b5bfa"	0.3475
"00074ee5e3"	"00075b6339"	-0.1516
"00074ee5e3"	"00075b6658"	-0.0041
"00074ee5e3"	"00075b679a"	-0.0139
"00074ee5e3"	"00075b6cb7"	-0.0475
"00074ee5e3"	"00075b6df8"	-0.1977
"00074ee5e3"	"00075b6ff6"	-0.1051
"00074ee5e3"	"00075b70ee"	-0.2305
"00074ee5e3"	"00075b7157"	0.2286

"00074ee5e3"	"00075b7225"	-0.2015
"00074ee5e3"	"00075b7c89"	-0.2443
"00074ee5e3"	"00075b9048"	-0.0069
"00074ee5e3"	"00075d0801"	0.2328
"00074ee5e3"	"00075d1820"	-0.004
"00074ee5e3"	"00075d1f3d"	-0.0093
"00074ee5e3"	"00075d2329"	-0.0756
"00074ee5e3"	"00075d2b9b"	-0.0348
"00074ee5e3"	"00075d3941"	-0.1815
"00074ee5e3"	"00075d3e96"	-0.0341
"00074ee5e3"	"00075d4864"	-0.2643
"00074ee5e3"	"00075d5961"	-0.2222
"00074ee5e3"	"00075d5a63"	0.1782
"00074ee5e3"	"00075d6150"	-0.1587
"00074ee5e3"	"00075d67d0"	-0.0278
"00074ee5e3"	"00075d67e2"	-0.2369
"00074ee5e3"	"00075d73fc"	-0.0973
"00074ee5e3"	"00075d7729"	-0.4546
"00074ee5e3"	"00075d778c"	-0.2245
"00074ee5e3"	"00075d7b9e"	0.1237
"00074ee5e3"	"00075d7c8f"	-0.2251
"00074ee5e3"	"00075d804d"	0.1375
"00074ee5e3"	"00075d819f"	0.1102
"00074ee5e3"	"00075d8601"	0.1979
"00074ee5e3"	"00075d8c6a"	-0.0919
"00074ee5e3"	"00075dfedc"	0.0883
"00074ee5e3"	"00075e05f2"	0.1356
"00074ee5e3"	"00075e0837"	0.0722
"00074ee5e3"	"00075e092e"	-0.2679
"00074ee5e3"	"00075e0965"	-0.4036
"00074ee5e3"	"00075e0bc8"	0.0859
"00074ee5e3"	"00075e0fbb"	-0.4729
"00074ee6e0"	"00074eea3a"	-0.0774
"00074ee6e0"	"00074eff82"	0.314
"00074ee6e0"	"00074f0477"	-0.1263
"00074ee6e0"	"00074f08c3"	-0.0099
"00074ee6e0"	"00074f1859"	-0.0852
"00074ee6e0"	"00074f2268"	-0.1341
"00074ee6e0"	"00074f28be"	0.0204
"00074ee6e0"	"00074f294b"	-0.2568
"00074ee6e0"	"00074f2ddd"	-0.2127
"00074ee6e0"	"00074f2e75"	-0.3567
"00074ee6e0"	"00074f3088"	-0.0442
"00074ee6e0"	"00074f5a1c"	-0.1774
"00074ee6e0"	"00074f75b7"	-0.0243
"00074ee6e0"	"00074f8cd9"	0.1706
"00074ee6e0"	"00074f96dc"	-0.1914
"00074ee6e0"	"00074fabaa"	-0.0579
"00074ee6e0"	"00074facd9"	0.1812
"00074ee6e0"	"00074fae3c"	0.0964

"00074ee6e0"	"00074fb0a8"	0.2419
"00074ee6e0"	"00074fb4e4"	-0.2475
"00074ee6e0"	"00074fb7c2"	0.1557
"00074ee6e0"	"00074fbd36"	-0.3018
"00074ee6e0"	"00074fc27f"	-0.1486
"00074ee6e0"	"00074fc31d"	0.0738
"00074ee6e0"	"00074fd569"	-0.1053
"00074ee6e0"	"00074fef15"	-0.0035
"00074ee6e0"	"00074ff562"	-0.4381
"00074ee6e0"	"00075007ca"	-0.1645
"00074ee6e0"	"0007500b86"	0.0391
"00074ee6e0"	"0007500d05"	0.1233
"00074ee6e0"	"0007500ee4"	-0.0927
"00074ee6e0"	"0007500eee"	0.0955
"00074ee6e0"	"00075013dc"	-0.1252
"00074ee6e0"	"000757b515"	0.0125
"00074ee6e0"	"000757bc5a"	0.1699
"00074ee6e0"	"000757c320"	-0.1589
"00074ee6e0"	"000757c9aa"	0.1093
"00074ee6e0"	"000757ccbe"	-0.086
"00074ee6e0"	"000757cfa9"	-0.2211
"00074ee6e0"	"000757d390"	-0.0211
"00074ee6e0"	"000757d393"	-0.0435
"00074ee6e0"	"000757d598"	-0.1644
"00074ee6e0"	"000757d5a2"	-0.2271
"00074ee6e0"	"000757d790"	0.0777
"00074ee6e0"	"000757e30c"	0.0178
"00074ee6e0"	"000757e4b0"	-0.0999
"00074ee6e0"	"000757e7a0"	-0.2404
"00074ee6e0"	"000757e8b3"	0.0788
"00074ee6e0"	"000757f627"	-0.0837
"00074ee6e0"	"000757f925"	0.1097
"00074ee6e0"	"000757fa08"	-0.1899
"00074ee6e0"	"000757fe52"	-0.1772
"00074ee6e0"	"000758024a"	-0.236
"00074ee6e0"	"00075804bb"	-0.1638
"00074ee6e0"	"00075a0c04"	-0.0716
"00074ee6e0"	"00075a3110"	0.1156
"00074ee6e0"	"00075a341a"	-0.1977
"00074ee6e0"	"00075a3dcf"	-0.2912
"00074ee6e0"	"00075a3e22"	0.0661
"00074ee6e0"	"00075a48d8"	0.0528
"00074ee6e0"	"00075a5cfb"	-0.1119
"00074ee6e0"	"00075a6151"	-0.1863
"00074ee6e0"	"00075a6708"	-0.1403
"00074ee6e0"	"00075a7319"	0.1052
"00074ee6e0"	"00075a7723"	-0.1112
"00074ee6e0"	"00075a778b"	-0.1861
"00074ee6e0"	"00075a7b8e"	0.2542
"00074ee6e0"	"00075a7c79"	-0.0248

"00074ee6e0"	"00075a81b6"	-0.1082
"00074ee6e0"	"00075a82ac"	-0.0747
"00074ee6e0"	"00075a98e5"	-0.2865
"00074ee6e0"	"00075b0d29"	-0.2395
"00074ee6e0"	"00075b102a"	-0.0359
"00074ee6e0"	"00075b1074"	-0.2362
"00074ee6e0"	"00075b135d"	-0.108
"00074ee6e0"	"00075b138b"	0.0432
"00074ee6e0"	"00075b13a0"	-0.3449
"00074ee6e0"	"00075b13bd"	-0.0277
"00074ee6e0"	"00075b16a9"	-0.0869
"00074ee6e0"	"00075b1a28"	-0.0067
"00074ee6e0"	"00075b1a97"	0.1768
"00074ee6e0"	"00075b1c7b"	-0.3343
"00074ee6e0"	"00075b1d24"	0.4901
"00074ee6e0"	"00075b202b"	0.0066
"00074ee6e0"	"00075b22cb"	-0.1526
"00074ee6e0"	"00075b22da"	-0.268
"00074ee6e0"	"00075b2556"	-0.2149
"00074ee6e0"	"00075b25de"	0.1282
"00074ee6e0"	"00075b260c"	-0.1678
"00074ee6e0"	"00075b26f1"	-0.0632
"00074ee6e0"	"00075b2920"	-0.036
"00074ee6e0"	"00075b2a64"	0.0957
"00074ee6e0"	"00075b2a9d"	0.0291
"00074ee6e0"	"00075b2b37"	-0.1298
"00074ee6e0"	"00075b2cdd"	0.1285
"00074ee6e0"	"00075b3038"	-0.0293
"00074ee6e0"	"00075b30fe"	-0.2569
"00074ee6e0"	"00075b3362"	0.1522
"00074ee6e0"	"00075b350a"	0.0109
"00074ee6e0"	"00075b350e"	-0.2627
"00074ee6e0"	"00075b3651"	0.203
"00074ee6e0"	"00075b38ca"	-0.3388
"00074ee6e0"	"00075b39cc"	0.0183
"00074ee6e0"	"00075b3e1e"	-0.0659
"00074ee6e0"	"00075b3e57"	-0.0382
"00074ee6e0"	"00075b4079"	-0.0725
"00074ee6e0"	"00075b4150"	-0.1477
"00074ee6e0"	"00075b4194"	0.0526
"00074ee6e0"	"00075b42d5"	-0.1864
"00074ee6e0"	"00075b4424"	-0.3395
"00074ee6e0"	"00075b4470"	0.1357
"00074ee6e0"	"00075b47ed"	-0.0391
"00074ee6e0"	"00075b4850"	0.0616
"00074ee6e0"	"00075b4ca0"	-0.259
"00074ee6e0"	"00075b4d7f"	0.1183
"00074ee6e0"	"00075b520f"	0.0234
"00074ee6e0"	"00075b525f"	0.0841
"00074ee6e0"	"00075b58f8"	-0.2478

"00074ee6e0"	"00075b5bcc"	0.1527
"00074ee6e0"	"00075b5bfa"	-0.0067
"00074ee6e0"	"00075b6339"	0.0019
"00074ee6e0"	"00075b6658"	-0.029
"00074ee6e0"	"00075b679a"	0.1049
"00074ee6e0"	"00075b6cb7"	-0.077
"00074ee6e0"	"00075b6df8"	-0.1527
"00074ee6e0"	"00075b6ff6"	0.0471
"00074ee6e0"	"00075b70ee"	-0.3108
"00074ee6e0"	"00075b7157"	0.4582
"00074ee6e0"	"00075b7225"	0.1666
"00074ee6e0"	"00075b7c89"	-0.2353
"00074ee6e0"	"00075b9048"	0.0998
"00074ee6e0"	"00075d0801"	-0.0304
"00074ee6e0"	"00075d1820"	-0.0828
"00074ee6e0"	"00075d1f3d"	-0.0542
"00074ee6e0"	"00075d2329"	-0.2084
"00074ee6e0"	"00075d2b9b"	-0.225
"00074ee6e0"	"00075d3941"	-0.1335
"00074ee6e0"	"00075d3e96"	0.0769
"00074ee6e0"	"00075d4864"	-0.1719
"00074ee6e0"	"00075d5961"	-0.0719
"00074ee6e0"	"00075d5a63"	1e-04
"00074ee6e0"	"00075d6150"	0.1358
"00074ee6e0"	"00075d67d0"	0.0255
"00074ee6e0"	"00075d67e2"	-0.1506
"00074ee6e0"	"00075d73fc"	-0.2189
"00074ee6e0"	"00075d7729"	-0.2582
"00074ee6e0"	"00075d778c"	-0.1031
"00074ee6e0"	"00075d7b9e"	0.4965
"00074ee6e0"	"00075d7c8f"	0.0031
"00074ee6e0"	"00075d804d"	0.0763
"00074ee6e0"	"00075d819f"	0.1958
"00074ee6e0"	"00075d8601"	-0.0182
"00074ee6e0"	"00075d8c6a"	-0.2207
"00074ee6e0"	"00075dfedc"	0.0051
"00074ee6e0"	"00075e05f2"	-0.1389
"00074ee6e0"	"00075e0837"	-0.1393
"00074ee6e0"	"00075e092e"	-0.0671
"00074ee6e0"	"00075e0965"	-0.3506
"00074ee6e0"	"00075e0bc8"	0.0024
"00074ee6e0"	"00075e0fbb"	-0.3251
"00074eea3a"	"00074eff82"	0.0921
"00074eea3a"	"00074f0477"	0.0303
"00074eea3a"	"00074f08c3"	-0.2097
"00074eea3a"	"00074f1859"	0.185
"00074eea3a"	"00074f2268"	0.0984
"00074eea3a"	"00074f28be"	0.1135
"00074eea3a"	"00074f294b"	0.4713
"00074eea3a"	"00074f2ddd"	0.1732

"00074eea3a"	"00074f2e75"	-0.008
"00074eea3a"	"00074f3088"	-0.0713
"00074eea3a"	"00074f5a1c"	0.3252
"00074eea3a"	"00074f75b7"	-0.1366
"00074eea3a"	"00074f8cd9"	-0.0461
"00074eea3a"	"00074f96dc"	-0.1586
"00074eea3a"	"00074fabaa"	0.2357
"00074eea3a"	"00074facd9"	0.1397
"00074eea3a"	"00074fae3c"	-0.0586
"00074eea3a"	"00074fb0a8"	-0.0563
"00074eea3a"	"00074fb4e4"	-0.3052
"00074eea3a"	"00074fb7c2"	0.3999
"00074eea3a"	"00074fbd36"	0.1075
"00074eea3a"	"00074fc27f"	0.1312
"00074eea3a"	"00074fc31d"	-0.0447
"00074eea3a"	"00074fd569"	-0.1015
"00074eea3a"	"00074fef15"	0.172
"00074eea3a"	"00074ff562"	-0.4224
"00074eea3a"	"00075007ca"	-0.1997
"00074eea3a"	"0007500b86"	-0.1487
"00074eea3a"	"0007500d05"	1e-04
"00074eea3a"	"0007500ee4"	0.0434
"00074eea3a"	"0007500eee"	0.0451
"00074eea3a"	"00075013dc"	-0.4263
"00074eea3a"	"000757b515"	0.0681
"00074eea3a"	"000757bc5a"	0.2365
"00074eea3a"	"000757c320"	-0.3085
"00074eea3a"	"000757c9aa"	-0.095
"00074eea3a"	"000757ccbe"	0.0836
"00074eea3a"	"000757cfa9"	-0.2461
"00074eea3a"	"000757d390"	-0.2537
"00074eea3a"	"000757d393"	0.0547
"00074eea3a"	"000757d598"	-0.2624
"00074eea3a"	"000757d5a2"	-0.0355
"00074eea3a"	"000757d790"	0.2834
"00074eea3a"	"000757e30c"	-0.2324
"00074eea3a"	"000757e4b0"	0.41
"00074eea3a"	"000757e7a0"	0.085
"00074eea3a"	"000757e8b3"	0.1995
"00074eea3a"	"000757f627"	-0.0292
"00074eea3a"	"000757f925"	0.1396
"00074eea3a"	"000757fa08"	-0.006
"00074eea3a"	"000757fe52"	0.1241
"00074eea3a"	"000758024a"	-0.4179
"00074eea3a"	"00075804bb"	-0.2372
"00074eea3a"	"00075a0c04"	0.1569
"00074eea3a"	"00075a3110"	0.0514
"00074eea3a"	"00075a341a"	-0.0388
"00074eea3a"	"00075a3dcf"	0.411
"00074eea3a"	"00075a3e22"	0.0095

"00074eea3a"	"00075a48d8"	0.0922
"00074eea3a"	"00075a5cfb"	-0.2037
"00074eea3a"	"00075a6151"	-0.1328
"00074eea3a"	"00075a6708"	-0.1449
"00074eea3a"	"00075a7319"	-0.22
"00074eea3a"	"00075a7723"	0.0316
"00074eea3a"	"00075a778b"	0.1352
"00074eea3a"	"00075a7b8e"	0.2264
"00074eea3a"	"00075a7c79"	0.179
"00074eea3a"	"00075a81b6"	-0.0787
"00074eea3a"	"00075a82ac"	0.3425
"00074eea3a"	"00075a98e5"	-0.13
"00074eea3a"	"00075b0d29"	-0.2296
"00074eea3a"	"00075b102a"	0.0412
"00074eea3a"	"00075b1074"	0.5034
"00074eea3a"	"00075b135d"	-0.0329
"00074eea3a"	"00075b138b"	0.4954
"00074eea3a"	"00075b13a0"	0.0796
"00074eea3a"	"00075b13bd"	0.0317
"00074eea3a"	"00075b16a9"	0.0986
"00074eea3a"	"00075b1a28"	0.0881
"00074eea3a"	"00075b1a97"	0.1822
"00074eea3a"	"00075b1c7b"	-0.1861
"00074eea3a"	"00075b1d24"	0.2105
"00074eea3a"	"00075b202b"	0.0317
"00074eea3a"	"00075b22cb"	0.2655
"00074eea3a"	"00075b22da"	0.4479
"00074eea3a"	"00075b2556"	0.2675
"00074eea3a"	"00075b25de"	-0.0713
"00074eea3a"	"00075b260c"	-0.0221
"00074eea3a"	"00075b26f1"	-0.0614
"00074eea3a"	"00075b2920"	-0.1629
"00074eea3a"	"00075b2a64"	-0.2787
"00074eea3a"	"00075b2a9d"	0.0151
"00074eea3a"	"00075b2b37"	-0.5283
"00074eea3a"	"00075b2cdd"	0.1459
"00074eea3a"	"00075b3038"	-0.0663
"00074eea3a"	"00075b30fe"	0.3788
"00074eea3a"	"00075b3362"	-0.0605
"00074eea3a"	"00075b350a"	0.0036
"00074eea3a"	"00075b350e"	0.1073
"00074eea3a"	"00075b3651"	0.0368
"00074eea3a"	"00075b38ca"	0.2108
"00074eea3a"	"00075b39cc"	0.1597
"00074eea3a"	"00075b3e1e"	0.2302
"00074eea3a"	"00075b3e57"	-0.1341
"00074eea3a"	"00075b4079"	-0.138
"00074eea3a"	"00075b4150"	-0.2832
"00074eea3a"	"00075b4194"	0.1578
"00074eea3a"	"00075b42d5"	0.3595

"00074eea3a"	"00075b4424"	-0.067
"00074eea3a"	"00075b4470"	0.3058
"00074eea3a"	"00075b47ed"	0.1966
"00074eea3a"	"00075b4850"	0.2222
"00074eea3a"	"00075b4ca0"	0.0465
"00074eea3a"	"00075b4d7f"	0.1453
"00074eea3a"	"00075b520f"	-0.21
"00074eea3a"	"00075b525f"	0.1624
"00074eea3a"	"00075b58f8"	-0.1077
"00074eea3a"	"00075b5bcc"	0.0311
"00074eea3a"	"00075b5bfa"	-0.2664
"00074eea3a"	"00075b6339"	0.1046
"00074eea3a"	"00075b6658"	-0.0908
"00074eea3a"	"00075b679a"	-0.0284
"00074eea3a"	"00075b6cb7"	0.0673
"00074eea3a"	"00075b6df8"	-0.0206
"00074eea3a"	"00075b6ff6"	0.0596
"00074eea3a"	"00075b70ee"	-0.0741
"00074eea3a"	"00075b7157"	0.1492
"00074eea3a"	"00075b7225"	0.3604
"00074eea3a"	"00075b7c89"	0.0575
"00074eea3a"	"00075b9048"	0.0014
"00074eea3a"	"00075d0801"	-0.1157
"00074eea3a"	"00075d1820"	0.2017
"00074eea3a"	"00075d1f3d"	0.1692
"00074eea3a"	"00075d2329"	0.2789
"00074eea3a"	"00075d2b9b"	-0.0654
"00074eea3a"	"00075d3941"	-0.1502
"00074eea3a"	"00075d3e96"	0.1463
"00074eea3a"	"00075d4864"	0.2717
"00074eea3a"	"00075d5961"	0.1171
"00074eea3a"	"00075d5a63"	0.0038
"00074eea3a"	"00075d6150"	-0.0779
"00074eea3a"	"00075d67d0"	-0.1311
"00074eea3a"	"00075d67e2"	0.079
"00074eea3a"	"00075d73fc"	0.1785
"00074eea3a"	"00075d7729"	-0.3101
"00074eea3a"	"00075d778c"	0.0187
"00074eea3a"	"00075d7b9e"	0.012
"00074eea3a"	"00075d7c8f"	0.3438
"00074eea3a"	"00075d804d"	0.0474
"00074eea3a"	"00075d819f"	0.0975
"00074eea3a"	"00075d8601"	-0.2129
"00074eea3a"	"00075d8c6a"	-0.0503
"00074eea3a"	"00075dfedc"	0.0916
"00074eea3a"	"00075e05f2"	0.1047
"00074eea3a"	"00075e0837"	0.277
"00074eea3a"	"00075e092e"	0.4276
"00074eea3a"	"00075e0965"	-0.2689
"00074eea3a"	"00075e0bc8"	0.2788

"00074eea3a"	"00075e0fbb"	0.3944
"00074eff82"	"00074f0477"	-0.2869
"00074eff82"	"00074f08c3"	-0.1607
"00074eff82"	"00074f1859"	-0.0962
"00074eff82"	"00074f2268"	-0.2139
"00074eff82"	"00074f28be"	0.0028
"00074eff82"	"00074f294b"	-0.2155
"00074eff82"	"00074f2ddd"	-0.2417
"00074eff82"	"00074f2e75"	-0.3484
"00074eff82"	"00074f3088"	0.0729
"00074eff82"	"00074f5a1c"	0.12
"00074eff82"	"00074f75b7"	0.2494
"00074eff82"	"00074f8cd9"	0.0495
"00074eff82"	"00074f96dc"	-0.0967
"00074eff82"	"00074fabaa"	-0.125
"00074eff82"	"00074facd9"	-0.1131
"00074eff82"	"00074fae3c"	0.1391
"00074eff82"	"00074fb0a8"	-0.0174
"00074eff82"	"00074fb4e4"	-0.2766
"00074eff82"	"00074fb7c2"	-0.0101
"00074eff82"	"00074fbd36"	-0.2568
"00074eff82"	"00074fc27f"	-0.071
"00074eff82"	"00074fc31d"	-0.2138
"00074eff82"	"00074fd569"	-0.0683
"00074eff82"	"00074fef15"	0.4289
"00074eff82"	"00074ff562"	-0.2618
"00074eff82"	"00075007ca"	-0.2303
"00074eff82"	"0007500b86"	0.0374
"00074eff82"	"0007500d05"	0.2299
"00074eff82"	"0007500ee4"	0.0996
"00074eff82"	"0007500eee"	0.0187
"00074eff82"	"00075013dc"	-0.2065
"00074eff82"	"000757b515"	-0.0764
"00074eff82"	"000757bc5a"	-0.0473
"00074eff82"	"000757c320"	-0.2025
"00074eff82"	"000757c9aa"	-0.1154
"00074eff82"	"000757ccbe"	-0.2271
"00074eff82"	"000757cfa9"	-0.2694
"00074eff82"	"000757d390"	-0.2761
"00074eff82"	"000757d393"	-0.0986
"00074eff82"	"000757d598"	-0.302
"00074eff82"	"000757d5a2"	-0.2941
"00074eff82"	"000757d790"	0.1363
"00074eff82"	"000757e30c"	-0.1079
"00074eff82"	"000757e4b0"	0.0607
"00074eff82"	"000757e7a0"	0.0064
"00074eff82"	"000757e8b3"	0.0235
"00074eff82"	"000757f627"	-0.1741
"00074eff82"	"000757f925"	-0.0417
"00074eff82"	"000757fa08"	-0.2108

"00074eff82"	"000757fe52"	-0.0099
"00074eff82"	"000758024a"	-0.4059
"00074eff82"	"00075804bb"	-0.472
"00074eff82"	"00075a0c04"	-0.0333
"00074eff82"	"00075a3110"	-0.0035
"00074eff82"	"00075a341a"	-0.2328
"00074eff82"	"00075a3dcf"	0.1697
"00074eff82"	"00075a3e22"	-0.1654
"00074eff82"	"00075a48d8"	0.1073
"00074eff82"	"00075a5cfb"	-0.2716
"00074eff82"	"00075a6151"	-0.1912
"00074eff82"	"00075a6708"	-0.2583
"00074eff82"	"00075a7319"	-0.302
"00074eff82"	"00075a7723"	-0.1655
"00074eff82"	"00075a778b"	-0.1948
"00074eff82"	"00075a7b8e"	0.0937
"00074eff82"	"00075a7c79"	0.0169
"00074eff82"	"00075a81b6"	0.1799
"00074eff82"	"00075a82ac"	0.0965
"00074eff82"	"00075a98e5"	-0.182
"00074eff82"	"00075b0d29"	-0.1395
"00074eff82"	"00075b102a"	-0.243
"00074eff82"	"00075b1074"	-0.262
"00074eff82"	"00075b135d"	-0.1481
"00074eff82"	"00075b138b"	0.0572
"00074eff82"	"00075b13a0"	-0.3589
"00074eff82"	"00075b13bd"	-0.0528
"00074eff82"	"00075b16a9"	-0.0376
"00074eff82"	"00075b1a28"	-0.1621
"00074eff82"	"00075b1a97"	0.1214
"00074eff82"	"00075b1c7b"	-0.2773
"00074eff82"	"00075b1d24"	0.2659
"00074eff82"	"00075b202b"	-0.2114
"00074eff82"	"00075b22cb"	-0.1427
"00074eff82"	"00075b22da"	-0.195
"00074eff82"	"00075b2556"	-0.2735
"00074eff82"	"00075b25de"	0.1282
"00074eff82"	"00075b260c"	-0.196
"00074eff82"	"00075b26f1"	0.1752
"00074eff82"	"00075b2920"	-0.261
"00074eff82"	"00075b2a64"	-0.2122
"00074eff82"	"00075b2a9d"	-0.3325
"00074eff82"	"00075b2b37"	-0.2369
"00074eff82"	"00075b2cdd"	0.1507
"00074eff82"	"00075b3038"	0.0798
"00074eff82"	"00075b30fe"	-0.0456
"00074eff82"	"00075b3362"	0.1245
"00074eff82"	"00075b350a"	-0.3017
"00074eff82"	"00075b350e"	-0.102
"00074eff82"	"00075b3651"	0.2411

"00074eff82"	"00075b38ca"	0.0277
"00074eff82"	"00075b39cc"	0.0176
"00074eff82"	"00075b3e1e"	−0.1429
"00074eff82"	"00075b3e57"	−0.02
"00074eff82"	"00075b4079"	−0.1871
"00074eff82"	"00075b4150"	−0.3756
"00074eff82"	"00075b4194"	0.0549
"00074eff82"	"00075b42d5"	0.1173
"00074eff82"	"00075b4424"	−0.0333
"00074eff82"	"00075b4470"	−0.0629
"00074eff82"	"00075b47ed"	0.0428
"00074eff82"	"00075b4850"	−0.0066
"00074eff82"	"00075b4ca0"	−0.1259
"00074eff82"	"00075b4d7f"	0.2199
"00074eff82"	"00075b520f"	−0.0184
"00074eff82"	"00075b525f"	0.2615
"00074eff82"	"00075b58f8"	−0.2571
"00074eff82"	"00075b5bcc"	0.2203
"00074eff82"	"00075b5bfa"	0.182
"00074eff82"	"00075b6339"	0.1116
"00074eff82"	"00075b6658"	−0.0243
"00074eff82"	"00075b679a"	0.0074
"00074eff82"	"00075b6cb7"	−0.1924
"00074eff82"	"00075b6df8"	0.1204
"00074eff82"	"00075b6ff6"	0.0243
"00074eff82"	"00075b70ee"	−0.2124
"00074eff82"	"00075b7157"	0.5154
"00074eff82"	"00075b7225"	0.1114
"00074eff82"	"00075b7c89"	−0.2024
"00074eff82"	"00075b9048"	−0.0539
"00074eff82"	"00075d0801"	−0.1673
"00074eff82"	"00075d1820"	0.1899
"00074eff82"	"00075d1f3d"	−0.1158
"00074eff82"	"00075d2329"	0.0869
"00074eff82"	"00075d2b9b"	−0.2031
"00074eff82"	"00075d3941"	−0.1878
"00074eff82"	"00075d3e96"	0.0645
"00074eff82"	"00075d4864"	−0.0478
"00074eff82"	"00075d5961"	−0.0665
"00074eff82"	"00075d5a63"	0.1596
"00074eff82"	"00075d6150"	−0.0229
"00074eff82"	"00075d67d0"	−0.1546
"00074eff82"	"00075d67e2"	−0.0377
"00074eff82"	"00075d73fc"	−0.0936
"00074eff82"	"00075d7729"	0.0611
"00074eff82"	"00075d778c"	0.2589
"00074eff82"	"00075d7b9e"	0.3437
"00074eff82"	"00075d7c8f"	−0.0584
"00074eff82"	"00075d804d"	0.0908
"00074eff82"	"00075d819f"	−0.2342

"00074eff82"	"00075d8601"	0.0593
"00074eff82"	"00075d8c6a"	-0.0018
"00074eff82"	"00075dfedc"	0.1261
"00074eff82"	"00075e05f2"	-0.1162
"00074eff82"	"00075e0837"	-0.0813
"00074eff82"	"00075e092e"	-0.1768
"00074eff82"	"00075e0965"	-0.4764
"00074eff82"	"00075e0bc8"	-0.1552
"00074eff82"	"00075e0fbb"	-0.098
"00074f0477"	"00074f08c3"	-0.415
"00074f0477"	"00074f1859"	-0.0886
"00074f0477"	"00074f2268"	-0.1638
"00074f0477"	"00074f28be"	-0.3416
"00074f0477"	"00074f294b"	-0.1058
"00074f0477"	"00074f2ddd"	-0.3297
"00074f0477"	"00074f2e75"	-0.4457
"00074f0477"	"00074f3088"	-0.0442
"00074f0477"	"00074f5a1c"	-0.1658
"00074f0477"	"00074f75b7"	0.0946
"00074f0477"	"00074f8cd9"	0.0772
"00074f0477"	"00074f96dc"	-0.3919
"00074f0477"	"00074fabaa"	-0.3361
"00074f0477"	"00074facd9"	0.1575
"00074f0477"	"00074fae3c"	-0.2093
"00074f0477"	"00074fb0a8"	-0.3795
"00074f0477"	"00074fb4e4"	-0.5526
"00074f0477"	"00074fb7c2"	-0.0539
"00074f0477"	"00074fbd36"	-0.3223
"00074f0477"	"00074fc27f"	-0.0314
"00074f0477"	"00074fc31d"	-0.3608
"00074f0477"	"00074fd569"	-0.0779
"00074f0477"	"00074fef15"	-0.0803
"00074f0477"	"00074ff562"	-0.2006
"00074f0477"	"00075007ca"	-0.0838
"00074f0477"	"0007500b86"	-0.1373
"00074f0477"	"0007500d05"	0.2269
"00074f0477"	"0007500ee4"	-0.0595
"00074f0477"	"0007500eee"	0.1723
"00074f0477"	"00075013dc"	-0.3774
"00074f0477"	"000757b515"	0.1609
"00074f0477"	"000757bc5a"	-0.1598
"00074f0477"	"000757c320"	-0.4803
"00074f0477"	"000757c9aa"	-0.1703
"00074f0477"	"000757ccbe"	-0.4411
"00074f0477"	"000757cfa9"	-0.3822
"00074f0477"	"000757d390"	-0.3959
"00074f0477"	"000757d393"	-0.0644
"00074f0477"	"000757d598"	-0.1938
"00074f0477"	"000757d5a2"	-0.0522
"00074f0477"	"000757d790"	0.1561

"00074f0477"	"000757e30c"	-0.2762
"00074f0477"	"000757e4b0"	-0.0089
"00074f0477"	"000757e7a0"	0.0216
"00074f0477"	"000757e8b3"	-0.2966
"00074f0477"	"000757f627"	-0.4064
"00074f0477"	"000757f925"	0.0062
"00074f0477"	"000757fa08"	-0.1311
"00074f0477"	"000757fe52"	0.0428
"00074f0477"	"000758024a"	-0.3055
"00074f0477"	"00075804bb"	-0.3009
"00074f0477"	"00075a0c04"	0.0266
"00074f0477"	"00075a3110"	-0.0229
"00074f0477"	"00075a341a"	-0.1348
"00074f0477"	"00075a3dcf"	-0.0091
"00074f0477"	"00075a3e22"	-0.2072
"00074f0477"	"00075a48d8"	-0.3062
"00074f0477"	"00075a5cfb"	-0.3211
"00074f0477"	"00075a6151"	-0.0975
"00074f0477"	"00075a6708"	0.0037
"00074f0477"	"00075a7319"	-0.4253
"00074f0477"	"00075a7723"	-0.2164
"00074f0477"	"00075a778b"	-0.073
"00074f0477"	"00075a7b8e"	-0.0924
"00074f0477"	"00075a7c79"	-0.0792
"00074f0477"	"00075a81b6"	-0.407
"00074f0477"	"00075a82ac"	-0.0167
"00074f0477"	"00075a98e5"	-0.3544
"00074f0477"	"00075b0d29"	-0.3368
"00074f0477"	"00075b102a"	-0.1223
"00074f0477"	"00075b1074"	-0.1533
"00074f0477"	"00075b135d"	-0.4562
"00074f0477"	"00075b138b"	0.0361
"00074f0477"	"00075b13a0"	-0.1094
"00074f0477"	"00075b13bd"	-0.2841
"00074f0477"	"00075b16a9"	-0.136
"00074f0477"	"00075b1a28"	-0.3189
"00074f0477"	"00075b1a97"	-0.2799
"00074f0477"	"00075b1c7b"	-0.5429
"00074f0477"	"00075b1d24"	-0.0584
"00074f0477"	"00075b202b"	0.0783
"00074f0477"	"00075b22cb"	-0.1519
"00074f0477"	"00075b22da"	-0.1637
"00074f0477"	"00075b2556"	-0.1119
"00074f0477"	"00075b25de"	0.2743
"00074f0477"	"00075b260c"	-0.1189
"00074f0477"	"00075b26f1"	-0.1671
"00074f0477"	"00075b2920"	-0.1726
"00074f0477"	"00075b2a64"	0.0073
"00074f0477"	"00075b2a9d"	0.122
"00074f0477"	"00075b2b37"	-0.2241

"00074f0477"	"00075b2cdd"	−0.3033
"00074f0477"	"00075b3038"	0.3908
"00074f0477"	"00075b30fe"	−0.1424
"00074f0477"	"00075b3362"	−0.3828
"00074f0477"	"00075b350a"	−0.1739
"00074f0477"	"00075b350e"	−0.3695
"00074f0477"	"00075b3651"	−0.2348
"00074f0477"	"00075b38ca"	−0.2089
"00074f0477"	"00075b39cc"	−0.1109
"00074f0477"	"00075b3e1e"	−0.0049
"00074f0477"	"00075b3e57"	−0.0952
"00074f0477"	"00075b4079"	−0.3561
"00074f0477"	"00075b4150"	−0.0485
"00074f0477"	"00075b4194"	−0.3623
"00074f0477"	"00075b42d5"	−0.0013
"00074f0477"	"00075b4424"	−0.2191
"00074f0477"	"00075b4470"	−0.1598
"00074f0477"	"00075b47ed"	−0.1476
"00074f0477"	"00075b4850"	0.0272
"00074f0477"	"00075b4ca0"	−0.2218
"00074f0477"	"00075b4d7f"	−0.1385
"00074f0477"	"00075b520f"	−0.1214
"00074f0477"	"00075b525f"	−0.5258
"00074f0477"	"00075b58f8"	−0.1675
"00074f0477"	"00075b5bcc"	−0.0779
"00074f0477"	"00075b5bfa"	0.1769
"00074f0477"	"00075b6339"	−0.1187
"00074f0477"	"00075b6658"	−0.2094
"00074f0477"	"00075b679a"	−0.1782
"00074f0477"	"00075b6cb7"	−0.0875
"00074f0477"	"00075b6df8"	−0.095
"00074f0477"	"00075b6ff6"	−0.023
"00074f0477"	"00075b70ee"	−0.1001
"00074f0477"	"00075b7157"	−0.125
"00074f0477"	"00075b7225"	−0.1603
"00074f0477"	"00075b7c89"	0.0157
"00074f0477"	"00075b9048"	0.0518
"00074f0477"	"00075d0801"	−0.0986
"00074f0477"	"00075d1820"	−0.0244
"00074f0477"	"00075d1f3d"	−0.1341
"00074f0477"	"00075d2329"	0.2442
"00074f0477"	"00075d2b9b"	−0.2097
"00074f0477"	"00075d3941"	−0.0451
"00074f0477"	"00075d3e96"	−0.1325
"00074f0477"	"00075d4864"	−0.3532
"00074f0477"	"00075d5961"	−0.0702
"00074f0477"	"00075d5a63"	0.0331
"00074f0477"	"00075d6150"	−0.4225
"00074f0477"	"00075d67d0"	−0.2552
"00074f0477"	"00075d67e2"	−0.4049

"00074f0477"	"00075d73fc"	−0.2159
"00074f0477"	"00075d7729"	−0.3811
"00074f0477"	"00075d778c"	−0.2677
"00074f0477"	"00075d7b9e"	−0.1629
"00074f0477"	"00075d7c8f"	−0.111
"00074f0477"	"00075d804d"	0.2194
"00074f0477"	"00075d819f"	−0.1079
"00074f0477"	"00075d8601"	−0.2446
"00074f0477"	"00075d8c6a"	−0.2002
"00074f0477"	"00075dfedc"	−0.0053
"00074f0477"	"00075e05f2"	−0.0896
"00074f0477"	"00075e0837"	0.1874
"00074f0477"	"00075e092e"	−0.2057
"00074f0477"	"00075e0965"	−0.2539
"00074f0477"	"00075e0bc8"	0.2473
"00074f0477"	"00075e0fbb"	−0.299
"00074f08c3"	"00074f1859"	−0.0952
"00074f08c3"	"00074f2268"	−0.3112
"00074f08c3"	"00074f28be"	0.2805
"00074f08c3"	"00074f294b"	−0.3061
"00074f08c3"	"00074f2ddd"	−0.5445
"00074f08c3"	"00074f2e75"	−0.662
"00074f08c3"	"00074f3088"	−0.2016
"00074f08c3"	"00074f5a1c"	−0.282
"00074f08c3"	"00074f75b7"	−0.1431
"00074f08c3"	"00074f8cd9"	−0.3096
"00074f08c3"	"00074f96dc"	0.0288
"00074f08c3"	"00074fabaa"	0.0316
"00074f08c3"	"00074facd9"	−0.1256
"00074f08c3"	"00074fae3c"	0.0051
"00074f08c3"	"00074fb0a8"	0.3244
"00074f08c3"	"00074fb4e4"	0.2846
"00074f08c3"	"00074fb7c2"	−0.1436
"00074f08c3"	"00074fbd36"	−0.1741
"00074f08c3"	"00074fc27f"	0.0125
"00074f08c3"	"00074fc31d"	−0.083
"00074f08c3"	"00074fd569"	−0.0484
"00074f08c3"	"00074fef15"	−0.1692
"00074f08c3"	"00074ff562"	−0.3224
"00074f08c3"	"00075007ca"	−0.3099
"00074f08c3"	"0007500b86"	−0.0336
"00074f08c3"	"0007500d05"	−0.3981
"00074f08c3"	"0007500ee4"	−0.4452
"00074f08c3"	"0007500eee"	−0.39
"00074f08c3"	"00075013dc"	−0.0309
"00074f08c3"	"000757b515"	−0.1697
"00074f08c3"	"000757bc5a"	0.1853
"00074f08c3"	"000757c320"	0.3525
"00074f08c3"	"000757c9aa"	0.5582
"00074f08c3"	"000757ccbe"	−0.2017

"00074f08c3"	"000757cfa9"	−0.2351
"00074f08c3"	"000757d390"	0.1933
"00074f08c3"	"000757d393"	−0.3325
"00074f08c3"	"000757d598"	−0.1194
"00074f08c3"	"000757d5a2"	−0.4671
"00074f08c3"	"000757d790"	−0.2706
"00074f08c3"	"000757e30c"	0.073
"00074f08c3"	"000757e4b0"	0.2412
"00074f08c3"	"000757e7a0"	−0.2587
"00074f08c3"	"000757e8b3"	−0.0241
"00074f08c3"	"000757f627"	0.255
"00074f08c3"	"000757f925"	−0.2887
"00074f08c3"	"000757fa08"	−0.5709
"00074f08c3"	"000757fe52"	−0.0991
"00074f08c3"	"000758024a"	0.0759
"00074f08c3"	"00075804bb"	0.0067
"00074f08c3"	"00075a0c04"	−0.2609
"00074f08c3"	"00075a3110"	−0.0626
"00074f08c3"	"00075a341a"	−0.3947
"00074f08c3"	"00075a3dcf"	−0.3332
"00074f08c3"	"00075a3e22"	0.0672
"00074f08c3"	"00075a48d8"	−0.2866
"00074f08c3"	"00075a5cfb"	0.1139
"00074f08c3"	"00075a6151"	−0.112
"00074f08c3"	"00075a6708"	−0.1871
"00074f08c3"	"00075a7319"	0.297
"00074f08c3"	"00075a7723"	−0.1783
"00074f08c3"	"00075a778b"	−0.2322
"00074f08c3"	"00075a7b8e"	0.2817
"00074f08c3"	"00075a7c79"	0.0137
"00074f08c3"	"00075a81b6"	0.0235
"00074f08c3"	"00075a82ac"	−0.1097
"00074f08c3"	"00075a98e5"	−0.131
"00074f08c3"	"00075b0d29"	−0.1064
"00074f08c3"	"00075b102a"	−0.2151
"00074f08c3"	"00075b1074"	−0.4183
"00074f08c3"	"00075b135d"	−0.1575
"00074f08c3"	"00075b138b"	0.2221
"00074f08c3"	"00075b13a0"	−0.2407
"00074f08c3"	"00075b13bd"	0.1608
"00074f08c3"	"00075b16a9"	−0.4164
"00074f08c3"	"00075b1a28"	−0.3504
"00074f08c3"	"00075b1a97"	0.0489
"00074f08c3"	"00075b1c7b"	−0.4988
"00074f08c3"	"00075b1d24"	−0.0067
"00074f08c3"	"00075b202b"	0.1117
"00074f08c3"	"00075b22cb"	−0.3454
"00074f08c3"	"00075b22da"	−0.2962
"00074f08c3"	"00075b2556"	−0.2695
"00074f08c3"	"00075b25de"	−0.0486

"00074f08c3"	"00075b260c"	-0.0653
"00074f08c3"	"00075b26f1"	-0.0818
"00074f08c3"	"00075b2920"	0.1536
"00074f08c3"	"00075b2a64"	0.1732
"00074f08c3"	"00075b2a9d"	-0.0645
"00074f08c3"	"00075b2b37"	-0.469
"00074f08c3"	"00075b2cdd"	-0.2076
"00074f08c3"	"00075b3038"	-0.0286
"00074f08c3"	"00075b30fe"	-0.4336
"00074f08c3"	"00075b3362"	-0.2364
"00074f08c3"	"00075b350a"	0.0355
"00074f08c3"	"00075b350e"	-0.2614
"00074f08c3"	"00075b3651"	-0.1396
"00074f08c3"	"00075b38ca"	-0.2917
"00074f08c3"	"00075b39cc"	-0.049
"00074f08c3"	"00075b3e1e"	-0.1496
"00074f08c3"	"00075b3e57"	-0.2125
"00074f08c3"	"00075b4079"	0.0139
"00074f08c3"	"00075b4150"	-0.1205
"00074f08c3"	"00075b4194"	0.1602
"00074f08c3"	"00075b42d5"	-0.2079
"00074f08c3"	"00075b4424"	-0.2105
"00074f08c3"	"00075b4470"	0.1697
"00074f08c3"	"00075b47ed"	-0.0826
"00074f08c3"	"00075b4850"	0.2687
"00074f08c3"	"00075b4ca0"	-0.0691
"00074f08c3"	"00075b4d7f"	-0.1951
"00074f08c3"	"00075b520f"	0.0201
"00074f08c3"	"00075b525f"	-0.1161
"00074f08c3"	"00075b58f8"	-0.2737
"00074f08c3"	"00075b5bcc"	0.2712
"00074f08c3"	"00075b5bfa"	-0.206
"00074f08c3"	"00075b6339"	0.034
"00074f08c3"	"00075b6658"	-0.2102
"00074f08c3"	"00075b679a"	-0.0616
"00074f08c3"	"00075b6cb7"	0.0093
"00074f08c3"	"00075b6df8"	-0.3437
"00074f08c3"	"00075b6ff6"	-0.0272
"00074f08c3"	"00075b70ee"	-0.2913
"00074f08c3"	"00075b7157"	-0.1849
"00074f08c3"	"00075b7225"	-0.2553
"00074f08c3"	"00075b7c89"	-0.2233
"00074f08c3"	"00075b9048"	0.1137
"00074f08c3"	"00075d0801"	-0.1752
"00074f08c3"	"00075d1820"	-0.1291
"00074f08c3"	"00075d1f3d"	0.1786
"00074f08c3"	"00075d2329"	-0.2346
"00074f08c3"	"00075d2b9b"	0.1884
"00074f08c3"	"00075d3941"	-0.229
"00074f08c3"	"00075d3e96"	-0.1149

"00074f08c3"	"00075d4864"	−0.3175
"00074f08c3"	"00075d5961"	−0.402
"00074f08c3"	"00075d5a63"	0.1733
"00074f08c3"	"00075d6150"	−0.1352
"00074f08c3"	"00075d67d0"	−0.0569
"00074f08c3"	"00075d67e2"	−0.0807
"00074f08c3"	"00075d73fc"	−0.3112
"00074f08c3"	"00075d7729"	−0.0067
"00074f08c3"	"00075d778c"	−0.3421
"00074f08c3"	"00075d7b9e"	−0.2021
"00074f08c3"	"00075d7c8f"	−0.1586
"00074f08c3"	"00075d804d"	0.3147
"00074f08c3"	"00075d819f"	−0.004
"00074f08c3"	"00075d8601"	0.1569
"00074f08c3"	"00075d8c6a"	−0.052
"00074f08c3"	"00075dfedc"	−0.0711
"00074f08c3"	"00075e05f2"	−0.2332
"00074f08c3"	"00075e0837"	−0.0854
"00074f08c3"	"00075e092e"	−0.3694
"00074f08c3"	"00075e0965"	−0.0371
"00074f08c3"	"00075e0bc8"	0.1965
"00074f08c3"	"00075e0fbb"	−0.4667
"00074f1859"	"00074f2268"	−0.0808
"00074f1859"	"00074f28be"	−0.0483
"00074f1859"	"00074f294b"	−0.148
"00074f1859"	"00074f2ddd"	−0.0803
"00074f1859"	"00074f2e75"	−0.0792
"00074f1859"	"00074f3088"	0.0342
"00074f1859"	"00074f5a1c"	0.1397
"00074f1859"	"00074f75b7"	0.0077
"00074f1859"	"00074f8cd9"	0.1776
"00074f1859"	"00074f96dc"	−0.1676
"00074f1859"	"00074fabaa"	0.0996
"00074f1859"	"00074facd9"	0.5496
"00074f1859"	"00074fae3c"	0.0692
"00074f1859"	"00074fb0a8"	−0.1222
"00074f1859"	"00074fb4e4"	−0.2799
"00074f1859"	"00074fb7c2"	0.196
"00074f1859"	"00074fbd36"	−0.0058
"00074f1859"	"00074fc27f"	0.0228
"00074f1859"	"00074fc31d"	−0.1679
"00074f1859"	"00074fd569"	−0.0639
"00074f1859"	"00074fef15"	−0.0675
"00074f1859"	"00074ff562"	−0.2579
"00074f1859"	"00075007ca"	−0.1252
"00074f1859"	"0007500b86"	0.15
"00074f1859"	"0007500d05"	0.0908
"00074f1859"	"0007500ee4"	−0.0589
"00074f1859"	"0007500eee"	0.0923
"00074f1859"	"00075013dc"	−0.4537

"00074f1859"	"000757b515"	-0.2862
"00074f1859"	"000757bc5a"	0.0843
"00074f1859"	"000757c320"	-0.0148
"00074f1859"	"000757c9aa"	-0.1157
"00074f1859"	"000757ccbe"	-0.0098
"00074f1859"	"000757cfa9"	-0.0969
"00074f1859"	"000757d390"	-0.2454
"00074f1859"	"000757d393"	0.163
"00074f1859"	"000757d598"	-0.3406
"00074f1859"	"000757d5a2"	-0.2183
"00074f1859"	"000757d790"	-0.2087
"00074f1859"	"000757e30c"	-0.1517
"00074f1859"	"000757e4b0"	0.1388
"00074f1859"	"000757e7a0"	-0.1411
"00074f1859"	"000757e8b3"	0.1648
"00074f1859"	"000757f627"	0.1423
"00074f1859"	"000757f925"	-8e-04
"00074f1859"	"000757fa08"	0.0619
"00074f1859"	"000757fe52"	0.1306
"00074f1859"	"000758024a"	0.071
"00074f1859"	"00075804bb"	-0.0554
"00074f1859"	"00075a0c04"	0.1484
"00074f1859"	"00075a3110"	-0.0569
"00074f1859"	"00075a341a"	0.1007
"00074f1859"	"00075a3dcf"	0.0734
"00074f1859"	"00075a3e22"	0.1012
"00074f1859"	"00075a48d8"	0.1213
"00074f1859"	"00075a5cfb"	-0.0831
"00074f1859"	"00075a6151"	-0.2084
"00074f1859"	"00075a6708"	-0.2229
"00074f1859"	"00075a7319"	-0.276
"00074f1859"	"00075a7723"	-0.1117
"00074f1859"	"00075a778b"	0.1047
"00074f1859"	"00075a7b8e"	0.121
"00074f1859"	"00075a7c79"	-0.0055
"00074f1859"	"00075a81b6"	-0.0588
"00074f1859"	"00075a82ac"	0.2164
"00074f1859"	"00075a98e5"	0.117
"00074f1859"	"00075b0d29"	0.1301
"00074f1859"	"00075b102a"	0.2394
"00074f1859"	"00075b1074"	-0.0886
"00074f1859"	"00075b135d"	-0.2096
"00074f1859"	"00075b138b"	0.2954
"00074f1859"	"00075b13a0"	-0.2046
"00074f1859"	"00075b13bd"	-0.0319
"00074f1859"	"00075b16a9"	0.1477
"00074f1859"	"00075b1a28"	0.2508
"00074f1859"	"00075b1a97"	0.2431
"00074f1859"	"00075b1c7b"	0.0723
"00074f1859"	"00075b1d24"	-6e-04

"00074f1859"	"00075b202b"	0.1676
"00074f1859"	"00075b22cb"	0.3525
"00074f1859"	"00075b22da"	0.1544
"00074f1859"	"00075b2556"	0.1049
"00074f1859"	"00075b25de"	−0.0553
"00074f1859"	"00075b260c"	−0.1663
"00074f1859"	"00075b26f1"	−0.0887
"00074f1859"	"00075b2920"	−0.0741
"00074f1859"	"00075b2a64"	−0.1559
"00074f1859"	"00075b2a9d"	0.0069
"00074f1859"	"00075b2b37"	−0.3783
"00074f1859"	"00075b2cdd"	0.398
"00074f1859"	"00075b3038"	0.0715
"00074f1859"	"00075b30fe"	0.0775
"00074f1859"	"00075b3362"	−0.4234
"00074f1859"	"00075b350a"	−0.0287
"00074f1859"	"00075b350e"	−0.0103
"00074f1859"	"00075b3651"	−0.1988
"00074f1859"	"00075b38ca"	0.2213
"00074f1859"	"00075b39cc"	0.0103
"00074f1859"	"00075b3e1e"	0.7298
"00074f1859"	"00075b3e57"	0.2044
"00074f1859"	"00075b4079"	−0.2583
"00074f1859"	"00075b4150"	−0.1164
"00074f1859"	"00075b4194"	0.0359
"00074f1859"	"00075b42d5"	0.2862
"00074f1859"	"00075b4424"	0.1023
"00074f1859"	"00075b4470"	0.1184
"00074f1859"	"00075b47ed"	0.0463
"00074f1859"	"00075b4850"	−0.0507
"00074f1859"	"00075b4ca0"	−0.2959
"00074f1859"	"00075b4d7f"	0.116
"00074f1859"	"00075b520f"	−0.1766
"00074f1859"	"00075b525f"	−0.3738
"00074f1859"	"00075b58f8"	−0.0231
"00074f1859"	"00075b5bcc"	−0.1228
"00074f1859"	"00075b5bfa"	−0.3559
"00074f1859"	"00075b6339"	−0.0756
"00074f1859"	"00075b6658"	−0.2415
"00074f1859"	"00075b679a"	−0.0707
"00074f1859"	"00075b6cb7"	−0.0011
"00074f1859"	"00075b6df8"	−0.0545
"00074f1859"	"00075b6ff6"	−0.105
"00074f1859"	"00075b70ee"	−0.2225
"00074f1859"	"00075b7157"	0.0577
"00074f1859"	"00075b7225"	0.0259
"00074f1859"	"00075b7c89"	−0.1
"00074f1859"	"00075b9048"	0.0736
"00074f1859"	"00075d0801"	−0.1825
"00074f1859"	"00075d1820"	−0.0803

"00074f1859"	"00075d1f3d"	-0.0214
"00074f1859"	"00075d2329"	0.2518
"00074f1859"	"00075d2b9b"	0.0293
"00074f1859"	"00075d3941"	0.0115
"00074f1859"	"00075d3e96"	-0.3054
"00074f1859"	"00075d4864"	0.1982
"00074f1859"	"00075d5961"	0.1377
"00074f1859"	"00075d5a63"	0.354
"00074f1859"	"00075d6150"	-0.1397
"00074f1859"	"00075d67d0"	-0.2757
"00074f1859"	"00075d67e2"	0.0903
"00074f1859"	"00075d73fc"	0.245
"00074f1859"	"00075d7729"	-0.2739
"00074f1859"	"00075d778c"	-0.2684
"00074f1859"	"00075d7b9e"	0.1344
"00074f1859"	"00075d7c8f"	0.0855
"00074f1859"	"00075d804d"	0.2196
"00074f1859"	"00075d819f"	0.1503
"00074f1859"	"00075d8601"	0.105
"00074f1859"	"00075d8c6a"	-0.0549
"00074f1859"	"00075dfedc"	0.0597
"00074f1859"	"00075e05f2"	0.0545
"00074f1859"	"00075e0837"	0.0522
"00074f1859"	"00075e092e"	0.0929
"00074f1859"	"00075e0965"	-0.0967
"00074f1859"	"00075e0bc8"	0.13
"00074f1859"	"00075e0fbb"	0.0145
"00074f2268"	"00074f28be"	-0.0463
"00074f2268"	"00074f294b"	0.0558
"00074f2268"	"00074f2ddd"	0.2926
"00074f2268"	"00074f2e75"	-0.0611
"00074f2268"	"00074f3088"	-0.2483
"00074f2268"	"00074f5a1c"	0.0312
"00074f2268"	"00074f75b7"	-0.2931
"00074f2268"	"00074f8cd9"	-0.3803
"00074f2268"	"00074f96dc"	-0.1756
"00074f2268"	"00074fabaa"	-0.1122
"00074f2268"	"00074facd9"	-0.1296
"00074f2268"	"00074fae3c"	-0.3229
"00074f2268"	"00074fb0a8"	-0.4847
"00074f2268"	"00074fb4e4"	-0.804
"00074f2268"	"00074fb7c2"	-0.1843
"00074f2268"	"00074fbd36"	0.3464
"00074f2268"	"00074fc27f"	0.0701
"00074f2268"	"00074fc31d"	-0.4403
"00074f2268"	"00074fd569"	-0.5955
"00074f2268"	"00074fef15"	-0.1021
"00074f2268"	"00074ff562"	-0.333
"00074f2268"	"00075007ca"	-0.1032
"00074f2268"	"0007500b86"	-0.1749

"00074f2268"	"0007500d05"	-0.2789
"00074f2268"	"0007500ee4"	-0.1721
"00074f2268"	"0007500eee"	-0.2343
"00074f2268"	"00075013dc"	-0.1771
"00074f2268"	"000757b515"	0.0311
"00074f2268"	"000757bc5a"	0.0329
"00074f2268"	"000757c320"	-0.2946
"00074f2268"	"000757c9aa"	-0.1578
"00074f2268"	"000757ccbe"	-0.0092
"00074f2268"	"000757cfa9"	-0.1876
"00074f2268"	"000757d390"	-0.4649
"00074f2268"	"000757d393"	-0.2084
"00074f2268"	"000757d598"	-0.1283
"00074f2268"	"000757d5a2"	-0.0724
"00074f2268"	"000757d790"	-0.195
"00074f2268"	"000757e30c"	-0.0351
"00074f2268"	"000757e4b0"	0.0838
"00074f2268"	"000757e7a0"	-0.1469
"00074f2268"	"000757e8b3"	-0.01
"00074f2268"	"000757f627"	-0.2113
"00074f2268"	"000757f925"	-0.1903
"00074f2268"	"000757fa08"	-0.0622
"00074f2268"	"000757fe52"	-0.209
"00074f2268"	"000758024a"	-0.2736
"00074f2268"	"00075804bb"	0.0127
"00074f2268"	"00075a0c04"	-0.1285
"00074f2268"	"00075a3110"	0.1179
"00074f2268"	"00075a341a"	0.1583
"00074f2268"	"00075a3dcf"	-0.1059
"00074f2268"	"00075a3e22"	0.1807
"00074f2268"	"00075a48d8"	-0.0204
"00074f2268"	"00075a5cfb"	0.0376
"00074f2268"	"00075a6151"	-0.1685
"00074f2268"	"00075a6708"	-0.0092
"00074f2268"	"00075a7319"	-0.5338
"00074f2268"	"00075a7723"	-0.257
"00074f2268"	"00075a778b"	-0.1915
"00074f2268"	"00075a7b8e"	-0.3094
"00074f2268"	"00075a7c79"	0.0649
"00074f2268"	"00075a81b6"	0.0032
"00074f2268"	"00075a82ac"	0.0023
"00074f2268"	"00075a98e5"	-0.2117
"00074f2268"	"00075b0d29"	-0.1503
"00074f2268"	"00075b102a"	0.2028
"00074f2268"	"00075b1074"	0.4576
"00074f2268"	"00075b135d"	-0.1109
"00074f2268"	"00075b138b"	0.1163
"00074f2268"	"00075b13a0"	-0.1547
"00074f2268"	"00075b13bd"	0.0464
"00074f2268"	"00075b16a9"	-0.4011

"00074f2268"	"00075b1a28"	−0.0145
"00074f2268"	"00075b1a97"	0.0717
"00074f2268"	"00075b1c7b"	−0.5711
"00074f2268"	"00075b1d24"	0.1593
"00074f2268"	"00075b202b"	−0.0476
"00074f2268"	"00075b22cb"	−0.1594
"00074f2268"	"00075b22da"	0.5371
"00074f2268"	"00075b2556"	0.5738
"00074f2268"	"00075b25de"	−0.3561
"00074f2268"	"00075b260c"	0.1128
"00074f2268"	"00075b26f1"	−0.1286
"00074f2268"	"00075b2920"	−0.1919
"00074f2268"	"00075b2a64"	−0.0704
"00074f2268"	"00075b2a9d"	0.0021
"00074f2268"	"00075b2b37"	−0.0916
"00074f2268"	"00075b2cdd"	−0.2127
"00074f2268"	"00075b3038"	−0.2871
"00074f2268"	"00075b30fe"	0.4558
"00074f2268"	"00075b3362"	−0.1909
"00074f2268"	"00075b350a"	0.4841
"00074f2268"	"00075b350e"	−0.1817
"00074f2268"	"00075b3651"	−0.2421
"00074f2268"	"00075b38ca"	−0.1289
"00074f2268"	"00075b39cc"	−0.2469
"00074f2268"	"00075b3e1e"	−0.1374
"00074f2268"	"00075b3e57"	−0.0378
"00074f2268"	"00075b4079"	−0.1172
"00074f2268"	"00075b4150"	−0.1837
"00074f2268"	"00075b4194"	−0.2102
"00074f2268"	"00075b42d5"	−0.0606
"00074f2268"	"00075b4424"	−0.1308
"00074f2268"	"00075b4470"	0.067
"00074f2268"	"00075b47ed"	−0.3277
"00074f2268"	"00075b4850"	0.087
"00074f2268"	"00075b4ca0"	0.1186
"00074f2268"	"00075b4d7f"	0.0711
"00074f2268"	"00075b520f"	−0.2424
"00074f2268"	"00075b525f"	−0.0358
"00074f2268"	"00075b58f8"	−0.0127
"00074f2268"	"00075b5bcc"	0.0491
"00074f2268"	"00075b5bfa"	−0.4413
"00074f2268"	"00075b6339"	0.0711
"00074f2268"	"00075b6658"	−0.1401
"00074f2268"	"00075b679a"	−0.2215
"00074f2268"	"00075b6cb7"	0.0698
"00074f2268"	"00075b6df8"	−0.1586
"00074f2268"	"00075b6ff6"	0.1491
"00074f2268"	"00075b70ee"	−0.301
"00074f2268"	"00075b7157"	0.0266
"00074f2268"	"00075b7225"	−0.0901

"00074f2268"	"00075b7c89"	−0.0377
"00074f2268"	"00075b9048"	−0.0954
"00074f2268"	"00075d0801"	−0.3058
"00074f2268"	"00075d1820"	−0.3899
"00074f2268"	"00075d1f3d"	0.037
"00074f2268"	"00075d2329"	−0.0847
"00074f2268"	"00075d2b9b"	−0.08
"00074f2268"	"00075d3941"	−0.3091
"00074f2268"	"00075d3e96"	−0.3017
"00074f2268"	"00075d4864"	−0.1344
"00074f2268"	"00075d5961"	−0.1867
"00074f2268"	"00075d5a63"	0.0013
"00074f2268"	"00075d6150"	−0.2919
"00074f2268"	"00075d67d0"	−0.3947
"00074f2268"	"00075d67e2"	−0.0447
"00074f2268"	"00075d73fc"	−0.3661
"00074f2268"	"00075d7729"	−0.0121
"00074f2268"	"00075d778c"	−0.0676
"00074f2268"	"00075d7b9e"	0.0274
"00074f2268"	"00075d7c8f"	0.1058
"00074f2268"	"00075d804d"	0.0778
"00074f2268"	"00075d819f"	0.0485
"00074f2268"	"00075d8601"	−0.4044
"00074f2268"	"00075d8c6a"	−0.2001
"00074f2268"	"00075dfedc"	−0.1321
"00074f2268"	"00075e05f2"	−0.1682
"00074f2268"	"00075e0837"	0.0499
"00074f2268"	"00075e092e"	0.3745
"00074f2268"	"00075e0965"	−0.2756
"00074f2268"	"00075e0bc8"	0.0036
"00074f2268"	"00075e0fbb"	0.3782
"00074f28be"	"00074f294b"	0.0416
"00074f28be"	"00074f2ddd"	−0.0143
"00074f28be"	"00074f2e75"	−0.185
"00074f28be"	"00074f3088"	−0.1165
"00074f28be"	"00074f5a1c"	0.2026
"00074f28be"	"00074f75b7"	0.133
"00074f28be"	"00074f8cd9"	0.2009
"00074f28be"	"00074f96dc"	−0.0898
"00074f28be"	"00074fabaa"	0.3353
"00074f28be"	"00074facd9"	−0.042
"00074f28be"	"00074fae3c"	−0.0934
"00074f28be"	"00074fb0a8"	0.4245
"00074f28be"	"00074fb4e4"	0.172
"00074f28be"	"00074fb7c2"	0.1224
"00074f28be"	"00074fbd36"	−0.0056
"00074f28be"	"00074fc27f"	0.2383
"00074f28be"	"00074fc31d"	0.0377
"00074f28be"	"00074fd569"	−0.1702
"00074f28be"	"00074fef15"	−0.2359

"00074f28be"	"00074ff562"	-0.1757
"00074f28be"	"00075007ca"	-0.0663
"00074f28be"	"0007500b86"	0.2685
"00074f28be"	"0007500d05"	-0.0824
"00074f28be"	"0007500ee4"	-0.1568
"00074f28be"	"0007500eee"	-0.2211
"00074f28be"	"00075013dc"	0.5088
"00074f28be"	"000757b515"	0.1772
"00074f28be"	"000757bc5a"	0.3489
"00074f28be"	"000757c320"	-0.0137
"00074f28be"	"000757c9aa"	0.1882
"00074f28be"	"000757ccbe"	0.1809
"00074f28be"	"000757cfa9"	-0.0572
"00074f28be"	"000757d390"	0.143
"00074f28be"	"000757d393"	-0.0107
"00074f28be"	"000757d598"	0.0392
"00074f28be"	"000757d5a2"	0.0562
"00074f28be"	"000757d790"	-0.1401
"00074f28be"	"000757e30c"	0.119
"00074f28be"	"000757e4b0"	0.3126
"00074f28be"	"000757e7a0"	-0.023
"00074f28be"	"000757e8b3"	0.2171
"00074f28be"	"000757f627"	0.5383
"00074f28be"	"000757f925"	0.0499
"00074f28be"	"000757fa08"	0.0219
"00074f28be"	"000757fe52"	-0.101
"00074f28be"	"000758024a"	0.0205
"00074f28be"	"00075804bb"	0.2658
"00074f28be"	"00075a0c04"	0.1328
"00074f28be"	"00075a3110"	0.2681
"00074f28be"	"00075a341a"	-0.2044
"00074f28be"	"00075a3dcf"	-0.0642
"00074f28be"	"00075a3e22"	0.3482
"00074f28be"	"00075a48d8"	0.0741
"00074f28be"	"00075a5cfb"	0.2722
"00074f28be"	"00075a6151"	0.1569
"00074f28be"	"00075a6708"	0.1169
"00074f28be"	"00075a7319"	0.161
"00074f28be"	"00075a7723"	0.1686
"00074f28be"	"00075a778b"	-0.0901
"00074f28be"	"00075a7b8e"	0.3634
"00074f28be"	"00075a7c79"	0.402
"00074f28be"	"00075a81b6"	-0.0064
"00074f28be"	"00075a82ac"	0.2138
"00074f28be"	"00075a98e5"	-0.0442
"00074f28be"	"00075b0d29"	0.0164
"00074f28be"	"00075b102a"	0.0194
"00074f28be"	"00075b1074"	-0.0894
"00074f28be"	"00075b135d"	0.3159
"00074f28be"	"00075b138b"	0.3441

"00074f28be"	"00075b13a0"	-0.2046
"00074f28be"	"00075b13bd"	0.093
"00074f28be"	"00075b16a9"	-0.151
"00074f28be"	"00075b1a28"	-0.1614
"00074f28be"	"00075b1a97"	0.0879
"00074f28be"	"00075b1c7b"	-0.4992
"00074f28be"	"00075b1d24"	0.0517
"00074f28be"	"00075b202b"	0.1221
"00074f28be"	"00075b22cb"	-0.1742
"00074f28be"	"00075b22da"	-0.0287
"00074f28be"	"00075b2556"	-0.1525
"00074f28be"	"00075b25de"	0.027
"00074f28be"	"00075b260c"	0.2116
"00074f28be"	"00075b26f1"	-0.016
"00074f28be"	"00075b2920"	0.3492
"00074f28be"	"00075b2a64"	0.2556
"00074f28be"	"00075b2a9d"	0.0764
"00074f28be"	"00075b2b37"	-0.4286
"00074f28be"	"00075b2cdd"	-0.1009
"00074f28be"	"00075b3038"	0.1281
"00074f28be"	"00075b30fe"	-0.0975
"00074f28be"	"00075b3362"	-0.2324
"00074f28be"	"00075b350a"	0.1816
"00074f28be"	"00075b350e"	0.141
"00074f28be"	"00075b3651"	-0.08
"00074f28be"	"00075b38ca"	0.0245
"00074f28be"	"00075b39cc"	0.1756
"00074f28be"	"00075b3e1e"	-0.0244
"00074f28be"	"00075b3e57"	-0.253
"00074f28be"	"00075b4079"	0.0549
"00074f28be"	"00075b4150"	0.0134
"00074f28be"	"00075b4194"	0.6557
"00074f28be"	"00075b42d5"	0.2107
"00074f28be"	"00075b4424"	-0.1045
"00074f28be"	"00075b4470"	0.3333
"00074f28be"	"00075b47ed"	0.0962
"00074f28be"	"00075b4850"	0.4163
"00074f28be"	"00075b4ca0"	0.172
"00074f28be"	"00075b4d7f"	0.3574
"00074f28be"	"00075b520f"	0.1429
"00074f28be"	"00075b525f"	-0.1401
"00074f28be"	"00075b58f8"	-0.0265
"00074f28be"	"00075b5bcc"	0.2345
"00074f28be"	"00075b5bfa"	-0.2178
"00074f28be"	"00075b6339"	0.1632
"00074f28be"	"00075b6658"	-0.2751
"00074f28be"	"00075b679a"	0.1295
"00074f28be"	"00075b6cb7"	0.0251
"00074f28be"	"00075b6df8"	-0.0079
"00074f28be"	"00075b6ff6"	0.2932

"00074f28be"	"00075b70ee"	0.1354
"00074f28be"	"00075b7157"	−0.0551
"00074f28be"	"00075b7225"	0.0619
"00074f28be"	"00075b7c89"	0.1207
"00074f28be"	"00075b9048"	0.2975
"00074f28be"	"00075d0801"	0.0463
"00074f28be"	"00075d1820"	−0.3105
"00074f28be"	"00075d1f3d"	0.3752
"00074f28be"	"00075d2329"	−0.059
"00074f28be"	"00075d2b9b"	0.1864
"00074f28be"	"00075d3941"	−0.039
"00074f28be"	"00075d3e96"	−0.0189
"00074f28be"	"00075d4864"	−0.0691
"00074f28be"	"00075d5961"	0.1712
"00074f28be"	"00075d5a63"	0.1139
"00074f28be"	"00075d6150"	0.2655
"00074f28be"	"00075d67d0"	−0.1893
"00074f28be"	"00075d67e2"	−0.0364
"00074f28be"	"00075d73fc"	−0.1577
"00074f28be"	"00075d7729"	−0.0412
"00074f28be"	"00075d778c"	−0.4131
"00074f28be"	"00075d7b9e"	−0.1335
"00074f28be"	"00075d7c8f"	0.2684
"00074f28be"	"00075d804d"	0.2582
"00074f28be"	"00075d819f"	0.0317
"00074f28be"	"00075d8601"	0.072
"00074f28be"	"00075d8c6a"	0.2475
"00074f28be"	"00075dfedc"	0.3365
"00074f28be"	"00075e05f2"	0.052
"00074f28be"	"00075e0837"	−0.0153
"00074f28be"	"00075e092e"	−0.113
"00074f28be"	"00075e0965"	0.0031
"00074f28be"	"00075e0bc8"	0.2156
"00074f28be"	"00075e0fbb"	−0.1648
"00074f294b"	"00074f2ddd"	0.1971
"00074f294b"	"00074f2e75"	0.1105
"00074f294b"	"00074f3088"	−0.0516
"00074f294b"	"00074f5a1c"	0.1409
"00074f294b"	"00074f75b7"	−0.1736
"00074f294b"	"00074f8cd9"	−0.1276
"00074f294b"	"00074f96dc"	−0.4585
"00074f294b"	"00074fabaa"	−0.0646
"00074f294b"	"00074facd9"	−0.1812
"00074f294b"	"00074fae3c"	−0.1688
"00074f294b"	"00074fb0a8"	−0.1129
"00074f294b"	"00074fb4e4"	−0.2417
"00074f294b"	"00074fb7c2"	0.365
"00074f294b"	"00074fbd36"	0.2073
"00074f294b"	"00074fc27f"	0.0927
"00074f294b"	"00074fc31d"	0.1433

"00074f294b"	"00074fd569"	−0.2725
"00074f294b"	"00074fef15"	0.0491
"00074f294b"	"00074ff562"	−0.3841
"00074f294b"	"00075007ca"	−0.2659
"00074f294b"	"0007500b86"	−0.0107
"00074f294b"	"0007500d05"	−0.2917
"00074f294b"	"0007500ee4"	−0.0201
"00074f294b"	"0007500eee"	−0.2681
"00074f294b"	"00075013dc"	−0.3435
"00074f294b"	"000757b515"	0.0614
"00074f294b"	"000757bc5a"	0.0259
"00074f294b"	"000757c320"	−0.2182
"00074f294b"	"000757c9aa"	0.0064
"00074f294b"	"000757ccbe"	0.121
"00074f294b"	"000757cfa9"	−0.1968
"00074f294b"	"000757d390"	−0.202
"00074f294b"	"000757d393"	0.0024
"00074f294b"	"000757d598"	−0.1584
"00074f294b"	"000757d5a2"	−0.0881
"00074f294b"	"000757d790"	0.2013
"00074f294b"	"000757e30c"	−0.0591
"00074f294b"	"000757e4b0"	0.1438
"00074f294b"	"000757e7a0"	0.0341
"00074f294b"	"000757e8b3"	0.0244
"00074f294b"	"000757f627"	0.0442
"00074f294b"	"000757f925"	−0.0162
"00074f294b"	"000757fa08"	0.173
"00074f294b"	"000757fe52"	−0.0658
"00074f294b"	"000758024a"	−0.2163
"00074f294b"	"00075804bb"	−0.0768
"00074f294b"	"00075a0c04"	−0.0903
"00074f294b"	"00075a3110"	−0.15
"00074f294b"	"00075a341a"	0.1353
"00074f294b"	"00075a3dcf"	0.2479
"00074f294b"	"00075a3e22"	−0.1533
"00074f294b"	"00075a48d8"	0.0118
"00074f294b"	"00075a5cfb"	−0.1349
"00074f294b"	"00075a6151"	−0.0765
"00074f294b"	"00075a6708"	0.0988
"00074f294b"	"00075a7319"	−0.2333
"00074f294b"	"00075a7723"	0.4167
"00074f294b"	"00075a778b"	0.1106
"00074f294b"	"00075a7b8e"	−0.0229
"00074f294b"	"00075a7c79"	0.0564
"00074f294b"	"00075a81b6"	−0.221
"00074f294b"	"00075a82ac"	0.0984
"00074f294b"	"00075a98e5"	−0.0469
"00074f294b"	"00075b0d29"	−0.1988
"00074f294b"	"00075b102a"	−0.2193
"00074f294b"	"00075b1074"	0.3903

"00074f294b"	"00075b135d"	−0.094
"00074f294b"	"00075b138b"	0.0808
"00074f294b"	"00075b13a0"	0.0091
"00074f294b"	"00075b13bd"	−0.0519
"00074f294b"	"00075b16a9"	0.0288
"00074f294b"	"00075b1a28"	−0.059
"00074f294b"	"00075b1a97"	0.1373
"00074f294b"	"00075b1c7b"	−0.2089
"00074f294b"	"00075b1d24"	0.2161
"00074f294b"	"00075b202b"	−0.2996
"00074f294b"	"00075b22cb"	0.17
"00074f294b"	"00075b22da"	0.2483
"00074f294b"	"00075b2556"	0.3483
"00074f294b"	"00075b25de"	−0.1564
"00074f294b"	"00075b260c"	−0.1486
"00074f294b"	"00075b26f1"	−0.0845
"00074f294b"	"00075b2920"	−0.1466
"00074f294b"	"00075b2a64"	−0.406
"00074f294b"	"00075b2a9d"	−0.259
"00074f294b"	"00075b2b37"	−0.4081
"00074f294b"	"00075b2cdd"	−0.2312
"00074f294b"	"00075b3038"	−0.0292
"00074f294b"	"00075b30fe"	0.2319
"00074f294b"	"00075b3362"	−0.0147
"00074f294b"	"00075b350a"	−0.0084
"00074f294b"	"00075b350e"	−0.2532
"00074f294b"	"00075b3651"	0.0491
"00074f294b"	"00075b38ca"	−0.0792
"00074f294b"	"00075b39cc"	0.0253
"00074f294b"	"00075b3e1e"	−0.0401
"00074f294b"	"00075b3e57"	−0.101
"00074f294b"	"00075b4079"	−0.228
"00074f294b"	"00075b4150"	−0.0652
"00074f294b"	"00075b4194"	−0.0057
"00074f294b"	"00075b42d5"	0.1993
"00074f294b"	"00075b4424"	−0.0102
"00074f294b"	"00075b4470"	0.1015
"00074f294b"	"00075b47ed"	−0.0326
"00074f294b"	"00075b4850"	0.0937
"00074f294b"	"00075b4ca0"	−0.1129
"00074f294b"	"00075b4d7f"	0.0857
"00074f294b"	"00075b520f"	−0.1888
"00074f294b"	"00075b525f"	0.173
"00074f294b"	"00075b58f8"	−0.2867
"00074f294b"	"00075b5bcc"	−0.0119
"00074f294b"	"00075b5bfa"	−0.3835
"00074f294b"	"00075b6339"	0.1797
"00074f294b"	"00075b6658"	−0.1069
"00074f294b"	"00075b679a"	0.2155
"00074f294b"	"00075b6cb7"	−0.0496

"00074f294b"	"00075b6df8"	0.0633
"00074f294b"	"00075b6ff6"	0.1913
"00074f294b"	"00075b70ee"	−0.1227
"00074f294b"	"00075b7157"	−0.1331
"00074f294b"	"00075b7225"	−0.0524
"00074f294b"	"00075b7c89"	−0.1033
"00074f294b"	"00075b9048"	0.0667
"00074f294b"	"00075d0801"	−0.0804
"00074f294b"	"00075d1820"	−0.0622
"00074f294b"	"00075d1f3d"	−0.0697
"00074f294b"	"00075d2329"	0.2059
"00074f294b"	"00075d2b9b"	−0.2243
"00074f294b"	"00075d3941"	−0.2965
"00074f294b"	"00075d3e96"	0.0086
"00074f294b"	"00075d4864"	0.061
"00074f294b"	"00075d5961"	−0.012
"00074f294b"	"00075d5a63"	−0.0913
"00074f294b"	"00075d6150"	−0.0821
"00074f294b"	"00075d67d0"	−0.0555
"00074f294b"	"00075d67e2"	−0.0235
"00074f294b"	"00075d73fc"	0.0107
"00074f294b"	"00075d7729"	−0.224
"00074f294b"	"00075d778c"	−0.0053
"00074f294b"	"00075d7b9e"	−0.0611
"00074f294b"	"00075d7c8f"	0.1121
"00074f294b"	"00075d804d"	−0.2343
"00074f294b"	"00075d819f"	−0.1178
"00074f294b"	"00075d8601"	−0.4227
"00074f294b"	"00075d8c6a"	−0.0734
"00074f294b"	"00075dfedc"	0.0362
"00074f294b"	"00075e05f2"	−0.0399
"00074f294b"	"00075e0837"	−0.0154
"00074f294b"	"00075e092e"	0.3185
"00074f294b"	"00075e0965"	−0.123
"00074f294b"	"00075e0bc8"	0.0854
"00074f294b"	"00075e0fbb"	0.3096
"00074f2ddd"	"00074f2e75"	−0.0901
"00074f2ddd"	"00074f3088"	−0.0959
"00074f2ddd"	"00074f5a1c"	−0.0345
"00074f2ddd"	"00074f75b7"	−0.229
"00074f2ddd"	"00074f8cd9"	−0.144
"00074f2ddd"	"00074f96dc"	−0.207
"00074f2ddd"	"00074fabaa"	−0.0861
"00074f2ddd"	"00074facd9"	−0.1952
"00074f2ddd"	"00074fae3c"	−0.1727
"00074f2ddd"	"00074fb0a8"	−0.3078
"00074f2ddd"	"00074fb4e4"	−0.4709
"00074f2ddd"	"00074fb7c2"	0.1364
"00074f2ddd"	"00074fbd36"	0.1159
"00074f2ddd"	"00074fc27f"	−0.2334

"00074f2ddd"	"00074fc31d"	-0.1907
"00074f2ddd"	"00074fd569"	-0.2115
"00074f2ddd"	"00074fef15"	-0.0366
"00074f2ddd"	"00074ff562"	-0.4109
"00074f2ddd"	"00075007ca"	-0.1024
"00074f2ddd"	"0007500b86"	-0.0779
"00074f2ddd"	"0007500d05"	-0.3155
"00074f2ddd"	"0007500ee4"	-0.0678
"00074f2ddd"	"0007500eee"	-0.4413
"00074f2ddd"	"00075013dc"	-0.1718
"00074f2ddd"	"000757b515"	0.103
"00074f2ddd"	"000757bc5a"	0.0221
"00074f2ddd"	"000757c320"	-0.1059
"00074f2ddd"	"000757c9aa"	-0.21
"00074f2ddd"	"000757ccbe"	0.079
"00074f2ddd"	"000757cfa9"	0.0652
"00074f2ddd"	"000757d390"	-0.4053
"00074f2ddd"	"000757d393"	0.0405
"00074f2ddd"	"000757d598"	0.064
"00074f2ddd"	"000757d5a2"	0.225
"00074f2ddd"	"000757d790"	5e-04
"00074f2ddd"	"000757e30c"	0.0096
"00074f2ddd"	"000757e4b0"	-0.1725
"00074f2ddd"	"000757e7a0"	-0.0839
"00074f2ddd"	"000757e8b3"	0.1409
"00074f2ddd"	"000757f627"	-0.0072
"00074f2ddd"	"000757f925"	0.0687
"00074f2ddd"	"000757fa08"	0.0294
"00074f2ddd"	"000757fe52"	-0.0346
"00074f2ddd"	"000758024a"	-0.1441
"00074f2ddd"	"00075804bb"	0.0735
"00074f2ddd"	"00075a0c04"	0.0284
"00074f2ddd"	"00075a3110"	0.2281
"00074f2ddd"	"00075a341a"	0.013
"00074f2ddd"	"00075a3dcf"	0.1078
"00074f2ddd"	"00075a3e22"	0.0146
"00074f2ddd"	"00075a48d8"	0.1173
"00074f2ddd"	"00075a5cfb"	-0.0301
"00074f2ddd"	"00075a6151"	-0.105
"00074f2ddd"	"00075a6708"	0.1028
"00074f2ddd"	"00075a7319"	-0.3417
"00074f2ddd"	"00075a7723"	0.0272
"00074f2ddd"	"00075a778b"	-0.0368
"00074f2ddd"	"00075a7b8e"	-0.289
"00074f2ddd"	"00075a7c79"	0.2245
"00074f2ddd"	"00075a81b6"	-0.1508
"00074f2ddd"	"00075a82ac"	-0.108
"00074f2ddd"	"00075a98e5"	-0.2269
"00074f2ddd"	"00075b0d29"	0.0176
"00074f2ddd"	"00075b102a"	0.0786

"00074f2ddd"	"00075b1074"	0.2027
"00074f2ddd"	"00075b135d"	0.243
"00074f2ddd"	"00075b138b"	0.028
"00074f2ddd"	"00075b13a0"	-0.1312
"00074f2ddd"	"00075b13bd"	-0.1036
"00074f2ddd"	"00075b16a9"	-0.0535
"00074f2ddd"	"00075b1a28"	0.2648
"00074f2ddd"	"00075b1a97"	0.1122
"00074f2ddd"	"00075b1c7b"	-0.2312
"00074f2ddd"	"00075b1d24"	0.1575
"00074f2ddd"	"00075b202b"	-0.1785
"00074f2ddd"	"00075b22cb"	0.0437
"00074f2ddd"	"00075b22da"	0.2876
"00074f2ddd"	"00075b2556"	0.2249
"00074f2ddd"	"00075b25de"	-0.1036
"00074f2ddd"	"00075b260c"	0.0812
"00074f2ddd"	"00075b26f1"	-0.0961
"00074f2ddd"	"00075b2920"	-0.1682
"00074f2ddd"	"00075b2a64"	-0.3946
"00074f2ddd"	"00075b2a9d"	0.0013
"00074f2ddd"	"00075b2b37"	-0.2115
"00074f2ddd"	"00075b2cdd"	-0.004
"00074f2ddd"	"00075b3038"	-0.048
"00074f2ddd"	"00075b30fe"	0.3702
"00074f2ddd"	"00075b3362"	-0.1296
"00074f2ddd"	"00075b350a"	0.0265
"00074f2ddd"	"00075b350e"	0.057
"00074f2ddd"	"00075b3651"	-0.1701
"00074f2ddd"	"00075b38ca"	-0.2991
"00074f2ddd"	"00075b39cc"	-0.167
"00074f2ddd"	"00075b3e1e"	-0.0563
"00074f2ddd"	"00075b3e57"	-0.0449
"00074f2ddd"	"00075b4079"	-0.0245
"00074f2ddd"	"00075b4150"	-0.1915
"00074f2ddd"	"00075b4194"	0.0631
"00074f2ddd"	"00075b42d5"	-0.0437
"00074f2ddd"	"00075b4424"	-0.2594
"00074f2ddd"	"00075b4470"	0.0221
"00074f2ddd"	"00075b47ed"	-0.1193
"00074f2ddd"	"00075b4850"	0.0515
"00074f2ddd"	"00075b4ca0"	0.2027
"00074f2ddd"	"00075b4d7f"	0.039
"00074f2ddd"	"00075b520f"	-0.1388
"00074f2ddd"	"00075b525f"	0.1495
"00074f2ddd"	"00075b58f8"	-0.0556
"00074f2ddd"	"00075b5bcc"	-0.0193
"00074f2ddd"	"00075b5bfa"	-0.4117
"00074f2ddd"	"00075b6339"	0.1572
"00074f2ddd"	"00075b6658"	-0.1784
"00074f2ddd"	"00075b679a"	0.2527

"00074f2ddd"	"00075b6cb7"	-0.0591
"00074f2ddd"	"00075b6df8"	-0.0443
"00074f2ddd"	"00075b6ff6"	0.0828
"00074f2ddd"	"00075b70ee"	0.0291
"00074f2ddd"	"00075b7157"	0.0212
"00074f2ddd"	"00075b7225"	0.1817
"00074f2ddd"	"00075b7c89"	0.2241
"00074f2ddd"	"00075b9048"	0.0406
"00074f2ddd"	"00075d0801"	-0.0422
"00074f2ddd"	"00075d1820"	-0.0828
"00074f2ddd"	"00075d1f3d"	-0.1378
"00074f2ddd"	"00075d2329"	-0.024
"00074f2ddd"	"00075d2b9b"	-0.2129
"00074f2ddd"	"00075d3941"	-0.1853
"00074f2ddd"	"00075d3e96"	-0.0884
"00074f2ddd"	"00075d4864"	-0.057
"00074f2ddd"	"00075d5961"	-0.0039
"00074f2ddd"	"00075d5a63"	-0.0186
"00074f2ddd"	"00075d6150"	-0.044
"00074f2ddd"	"00075d67d0"	-0.3563
"00074f2ddd"	"00075d67e2"	-0.1227
"00074f2ddd"	"00075d73fc"	0.1379
"00074f2ddd"	"00075d7729"	-0.0872
"00074f2ddd"	"00075d778c"	-0.0676
"00074f2ddd"	"00075d7b9e"	0.0141
"00074f2ddd"	"00075d7c8f"	0.233
"00074f2ddd"	"00075d804d"	-0.1853
"00074f2ddd"	"00075d819f"	0.0557
"00074f2ddd"	"00075d8601"	-0.1692
"00074f2ddd"	"00075d8c6a"	-0.1915
"00074f2ddd"	"00075dfedc"	0.0802
"00074f2ddd"	"00075e05f2"	-0.1489
"00074f2ddd"	"00075e0837"	-0.0524
"00074f2ddd"	"00075e092e"	0.4997
"00074f2ddd"	"00075e0965"	-0.31
"00074f2ddd"	"00075e0bc8"	-0.0091
"00074f2ddd"	"00075e0fbb"	0.2286
"00074f2e75"	"00074f3088"	-0.0397
"00074f2e75"	"00074f5a1c"	-0.0526
"00074f2e75"	"00074f75b7"	-0.1931
"00074f2e75"	"00074f8cd9"	-0.0189
"00074f2e75"	"00074f96dc"	-0.3131
"00074f2e75"	"00074fabaa"	-0.2331
"00074f2e75"	"00074facd9"	-0.3383
"00074f2e75"	"00074fae3c"	-0.314
"00074f2e75"	"00074fb0a8"	-0.4206
"00074f2e75"	"00074fb4e4"	-0.7219
"00074f2e75"	"00074fb7c2"	0.343
"00074f2e75"	"00074fbd36"	-0.1416
"00074f2e75"	"00074fc27f"	-0.1516

"00074f2e75"	"00074fc31d"	-0.1032
"00074f2e75"	"00074fd569"	-0.1101
"00074f2e75"	"00074fef15"	-0.2948
"00074f2e75"	"00074ff562"	-0.3376
"00074f2e75"	"00075007ca"	-0.2946
"00074f2e75"	"0007500b86"	-0.259
"00074f2e75"	"0007500d05"	-0.0947
"00074f2e75"	"0007500ee4"	-0.2775
"00074f2e75"	"0007500eee"	-0.308
"00074f2e75"	"00075013dc"	-0.4175
"00074f2e75"	"000757b515"	-0.1728
"00074f2e75"	"000757bc5a"	-0.2983
"00074f2e75"	"000757c320"	-0.6379
"00074f2e75"	"000757c9aa"	-0.4086
"00074f2e75"	"000757ccbe"	0.2689
"00074f2e75"	"000757cfa9"	-0.4901
"00074f2e75"	"000757d390"	-0.5675
"00074f2e75"	"000757d393"	-0.0331
"00074f2e75"	"000757d598"	-0.3371
"00074f2e75"	"000757d5a2"	-0.2575
"00074f2e75"	"000757d790"	-0.1346
"00074f2e75"	"000757e30c"	-0.2664
"00074f2e75"	"000757e4b0"	-0.22
"00074f2e75"	"000757e7a0"	-0.2738
"00074f2e75"	"000757e8b3"	0.0795
"00074f2e75"	"000757f627"	-0.4482
"00074f2e75"	"000757f925"	0.0022
"00074f2e75"	"000757fa08"	0.4359
"00074f2e75"	"000757fe52"	-0.045
"00074f2e75"	"000758024a"	-0.1789
"00074f2e75"	"00075804bb"	-0.2918
"00074f2e75"	"00075a0c04"	0.1775
"00074f2e75"	"00075a3110"	-0.0561
"00074f2e75"	"00075a341a"	0.4301
"00074f2e75"	"00075a3dcf"	-0.023
"00074f2e75"	"00075a3e22"	0.0639
"00074f2e75"	"00075a48d8"	0.5108
"00074f2e75"	"00075a5cfb"	-0.1637
"00074f2e75"	"00075a6151"	-0.1832
"00074f2e75"	"00075a6708"	-0.1974
"00074f2e75"	"00075a7319"	-0.6826
"00074f2e75"	"00075a7723"	0.0418
"00074f2e75"	"00075a778b"	0.1211
"00074f2e75"	"00075a7b8e"	-0.1606
"00074f2e75"	"00075a7c79"	-0.1651
"00074f2e75"	"00075a81b6"	0.0501
"00074f2e75"	"00075a82ac"	-0.0121
"00074f2e75"	"00075a98e5"	-0.1907
"00074f2e75"	"00075b0d29"	-0.161
"00074f2e75"	"00075b102a"	-0.06

"00074f2e75"	"00075b1074"	0.1826
"00074f2e75"	"00075b135d"	-0.137
"00074f2e75"	"00075b138b"	-0.2721
"00074f2e75"	"00075b13a0"	-0.1262
"00074f2e75"	"00075b13bd"	-0.277
"00074f2e75"	"00075b16a9"	0.0051
"00074f2e75"	"00075b1a28"	-0.1502
"00074f2e75"	"00075b1a97"	-0.2025
"00074f2e75"	"00075b1c7b"	-0.1185
"00074f2e75"	"00075b1d24"	-0.1387
"00074f2e75"	"00075b202b"	-0.2521
"00074f2e75"	"00075b22cb"	-0.0252
"00074f2e75"	"00075b22da"	0.0265
"00074f2e75"	"00075b2556"	-0.0498
"00074f2e75"	"00075b25de"	-0.4144
"00074f2e75"	"00075b260c"	-0.1645
"00074f2e75"	"00075b26f1"	-0.1239
"00074f2e75"	"00075b2920"	-0.3042
"00074f2e75"	"00075b2a64"	-0.3925
"00074f2e75"	"00075b2a9d"	-0.3977
"00074f2e75"	"00075b2b37"	-0.4623
"00074f2e75"	"00075b2cdd"	-0.1317
"00074f2e75"	"00075b3038"	-0.2054
"00074f2e75"	"00075b30fe"	-0.1735
"00074f2e75"	"00075b3362"	-0.1222
"00074f2e75"	"00075b350a"	-0.1495
"00074f2e75"	"00075b350e"	-0.2318
"00074f2e75"	"00075b3651"	-0.2021
"00074f2e75"	"00075b38ca"	-0.099
"00074f2e75"	"00075b39cc"	-0.3257
"00074f2e75"	"00075b3e1e"	0.0468
"00074f2e75"	"00075b3e57"	0.0663
"00074f2e75"	"00075b4079"	-0.2521
"00074f2e75"	"00075b4150"	-0.0809
"00074f2e75"	"00075b4194"	-0.3726
"00074f2e75"	"00075b42d5"	-0.1137
"00074f2e75"	"00075b4424"	0.0511
"00074f2e75"	"00075b4470"	-0.2133
"00074f2e75"	"00075b47ed"	-0.1218
"00074f2e75"	"00075b4850"	-0.3635
"00074f2e75"	"00075b4ca0"	-0.2208
"00074f2e75"	"00075b4d7f"	0.4611
"00074f2e75"	"00075b520f"	-0.3393
"00074f2e75"	"00075b525f"	-0.1493
"00074f2e75"	"00075b58f8"	-0.43
"00074f2e75"	"00075b5bcc"	-0.1752
"00074f2e75"	"00075b5bfa"	-0.4694
"00074f2e75"	"00075b6339"	-0.0198
"00074f2e75"	"00075b6658"	-0.3204
"00074f2e75"	"00075b679a"	-0.0963

"00074f2e75"	"00075b6cb7"	-0.1878
"00074f2e75"	"00075b6df8"	0.4718
"00074f2e75"	"00075b6ff6"	-0.1405
"00074f2e75"	"00075b70ee"	-0.2507
"00074f2e75"	"00075b7157"	-0.1742
"00074f2e75"	"00075b7225"	-0.1203
"00074f2e75"	"00075b7c89"	-0.0064
"00074f2e75"	"00075b9048"	-0.0183
"00074f2e75"	"00075d0801"	-0.2026
"00074f2e75"	"00075d1820"	-0.3765
"00074f2e75"	"00075d1f3d"	-0.3068
"00074f2e75"	"00075d2329"	-0.1438
"00074f2e75"	"00075d2b9b"	-0.1908
"00074f2e75"	"00075d3941"	-0.2464
"00074f2e75"	"00075d3e96"	-0.1643
"00074f2e75"	"00075d4864"	-0.2167
"00074f2e75"	"00075d5961"	-0.0416
"00074f2e75"	"00075d5a63"	-0.1089
"00074f2e75"	"00075d6150"	-0.2964
"00074f2e75"	"00075d67d0"	-0.0847
"00074f2e75"	"00075d67e2"	-0.0723
"00074f2e75"	"00075d73fc"	0.1195
"00074f2e75"	"00075d7729"	-0.0473
"00074f2e75"	"00075d778c"	-0.1768
"00074f2e75"	"00075d7b9e"	-0.1282
"00074f2e75"	"00075d7c8f"	-0.0605
"00074f2e75"	"00075d804d"	-0.5066
"00074f2e75"	"00075d819f"	-0.2795
"00074f2e75"	"00075d8601"	-0.3981
"00074f2e75"	"00075d8c6a"	0.0915
"00074f2e75"	"00075dfedc"	-0.0334
"00074f2e75"	"00075e05f2"	0.0278
"00074f2e75"	"00075e0837"	-0.142
"00074f2e75"	"00075e092e"	-2e-04
"00074f2e75"	"00075e0965"	-0.2392
"00074f2e75"	"00075e0bc8"	-0.22
"00074f2e75"	"00075e0fbb"	0.0274
"00074f3088"	"00074f5a1c"	-0.0021
"00074f3088"	"00074f75b7"	0.0417
"00074f3088"	"00074f8cd9"	0.0412
"00074f3088"	"00074f96dc"	0.2469
"00074f3088"	"00074fabaa"	-0.0962
"00074f3088"	"00074facd9"	0.0903
"00074f3088"	"00074fae3c"	0.2983
"00074f3088"	"00074fb0a8"	0.0182
"00074f3088"	"00074fb4e4"	0.1053
"00074f3088"	"00074fb7c2"	0.1234
"00074f3088"	"00074fbd36"	-0.0401
"00074f3088"	"00074fc27f"	-0.2423
"00074f3088"	"00074fc31d"	0.1085

"00074f3088"	"00074fd569"	0.1934
"00074f3088"	"00074fef15"	0.1093
"00074f3088"	"00074ff562"	-0.2084
"00074f3088"	"00075007ca"	-0.2278
"00074f3088"	"0007500b86"	0.1542
"00074f3088"	"0007500d05"	0.1528
"00074f3088"	"0007500ee4"	0.174
"00074f3088"	"0007500eee"	-0.1189
"00074f3088"	"00075013dc"	-0.0482
"00074f3088"	"000757b515"	-0.0195
"00074f3088"	"000757bc5a"	0.008
"00074f3088"	"000757c320"	-0.1411
"00074f3088"	"000757c9aa"	-0.0648
"00074f3088"	"000757ccbe"	0.1932
"00074f3088"	"000757cfa9"	-0.1187
"00074f3088"	"000757d390"	-0.093
"00074f3088"	"000757d393"	0.0247
"00074f3088"	"000757d598"	-0.2014
"00074f3088"	"000757d5a2"	-0.1318
"00074f3088"	"000757d790"	-0.0934
"00074f3088"	"000757e30c"	-0.1061
"00074f3088"	"000757e4b0"	0.0048
"00074f3088"	"000757e7a0"	0.0656
"00074f3088"	"000757e8b3"	0.0938
"00074f3088"	"000757f627"	0.0669
"00074f3088"	"000757f925"	0.1328
"00074f3088"	"000757fa08"	-0.1314
"00074f3088"	"000757fe52"	0.0996
"00074f3088"	"000758024a"	-0.3833
"00074f3088"	"00075804bb"	-0.086
"00074f3088"	"00075a0c04"	-0.0459
"00074f3088"	"00075a3110"	0.0115
"00074f3088"	"00075a341a"	0.0073
"00074f3088"	"00075a3dcf"	0.0028
"00074f3088"	"00075a3e22"	-0.0627
"00074f3088"	"00075a48d8"	0.072
"00074f3088"	"00075a5cfb"	-0.2112
"00074f3088"	"00075a6151"	-0.2413
"00074f3088"	"00075a6708"	-0.0931
"00074f3088"	"00075a7319"	-0.0939
"00074f3088"	"00075a7723"	-0.0856
"00074f3088"	"00075a778b"	-0.2121
"00074f3088"	"00075a7b8e"	-0.053
"00074f3088"	"00075a7c79"	-0.0574
"00074f3088"	"00075a81b6"	-0.0139
"00074f3088"	"00075a82ac"	0.2833
"00074f3088"	"00075a98e5"	0.12
"00074f3088"	"00075b0d29"	0.1458
"00074f3088"	"00075b102a"	-0.0819
"00074f3088"	"00075b1074"	-0.0835

"00074f3088"	"00075b135d"	-0.0759
"00074f3088"	"00075b138b"	-0.1439
"00074f3088"	"00075b13a0"	-0.1974
"00074f3088"	"00075b13bd"	0.303
"00074f3088"	"00075b16a9"	0.1033
"00074f3088"	"00075b1a28"	-0.1794
"00074f3088"	"00075b1a97"	0.0089
"00074f3088"	"00075b1c7b"	-0.1717
"00074f3088"	"00075b1d24"	-0.0797
"00074f3088"	"00075b202b"	-0.1496
"00074f3088"	"00075b22cb"	-0.0938
"00074f3088"	"00075b22da"	-0.2508
"00074f3088"	"00075b2556"	-0.0785
"00074f3088"	"00075b25de"	0.1541
"00074f3088"	"00075b260c"	-0.2766
"00074f3088"	"00075b26f1"	-0.0985
"00074f3088"	"00075b2920"	-0.0178
"00074f3088"	"00075b2a64"	-0.2271
"00074f3088"	"00075b2a9d"	-0.1612
"00074f3088"	"00075b2b37"	-0.1243
"00074f3088"	"00075b2cdd"	0.0511
"00074f3088"	"00075b3038"	0.1665
"00074f3088"	"00075b30fe"	-0.0719
"00074f3088"	"00075b3362"	-0.0831
"00074f3088"	"00075b350a"	0.0233
"00074f3088"	"00075b350e"	-0.1047
"00074f3088"	"00075b3651"	0.0894
"00074f3088"	"00075b38ca"	-0.01
"00074f3088"	"00075b39cc"	0.0787
"00074f3088"	"00075b3e1e"	0.0215
"00074f3088"	"00075b3e57"	0.3309
"00074f3088"	"00075b4079"	0.1149
"00074f3088"	"00075b4150"	-0.2486
"00074f3088"	"00075b4194"	0.0406
"00074f3088"	"00075b42d5"	0.0308
"00074f3088"	"00075b4424"	0.1243
"00074f3088"	"00075b4470"	0.008
"00074f3088"	"00075b47ed"	0.0208
"00074f3088"	"00075b4850"	-0.0478
"00074f3088"	"00075b4ca0"	-0.0991
"00074f3088"	"00075b4d7f"	0.1724
"00074f3088"	"00075b520f"	-0.0348
"00074f3088"	"00075b525f"	0.0275
"00074f3088"	"00075b58f8"	-0.1516
"00074f3088"	"00075b5bcc"	-0.0351
"00074f3088"	"00075b5bfa"	-0.0231
"00074f3088"	"00075b6339"	-0.0242
"00074f3088"	"00075b6658"	-0.0433
"00074f3088"	"00075b679a"	0.0247
"00074f3088"	"00075b6cb7"	-0.1985

"00074f3088"	"00075b6df8"	0.1112
"00074f3088"	"00075b6ff6"	−0.0317
"00074f3088"	"00075b70ee"	−0.1308
"00074f3088"	"00075b7157"	0.2554
"00074f3088"	"00075b7225"	−0.1739
"00074f3088"	"00075b7c89"	0.1031
"00074f3088"	"00075b9048"	0.0384
"00074f3088"	"00075d0801"	0.199
"00074f3088"	"00075d1820"	0.1209
"00074f3088"	"00075d1f3d"	−0.1292
"00074f3088"	"00075d2329"	0.058
"00074f3088"	"00075d2b9b"	−0.2326
"00074f3088"	"00075d3941"	−0.0691
"00074f3088"	"00075d3e96"	0.2187
"00074f3088"	"00075d4864"	−0.166
"00074f3088"	"00075d5961"	−0.0176
"00074f3088"	"00075d5a63"	0.1612
"00074f3088"	"00075d6150"	−0.0015
"00074f3088"	"00075d67d0"	−0.1035
"00074f3088"	"00075d67e2"	−0.0775
"00074f3088"	"00075d73fc"	0.0835
"00074f3088"	"00075d7729"	0.0154
"00074f3088"	"00075d778c"	−0.1939
"00074f3088"	"00075d7b9e"	0.1279
"00074f3088"	"00075d7c8f"	0.0036
"00074f3088"	"00075d804d"	−0.0069
"00074f3088"	"00075d819f"	0.0763
"00074f3088"	"00075d8601"	0.2217
"00074f3088"	"00075d8c6a"	0.0686
"00074f3088"	"00075dfedc"	0.002
"00074f3088"	"00075e05f2"	−0.1403
"00074f3088"	"00075e0837"	0.0151
"00074f3088"	"00075e092e"	−0.1178
"00074f3088"	"00075e0965"	−0.3535
"00074f3088"	"00075e0bc8"	−0.0775
"00074f3088"	"00075e0fbb"	−0.2113
"00074f5a1c"	"00074f75b7"	0.2678
"00074f5a1c"	"00074f8cd9"	0.0611
"00074f5a1c"	"00074f96dc"	−0.2301
"00074f5a1c"	"00074fabaa"	0.2313
"00074f5a1c"	"00074facd9"	−0.0339
"00074f5a1c"	"00074fae3c"	−0.0411
"00074f5a1c"	"00074fb0a8"	−0.1039
"00074f5a1c"	"00074fb4e4"	−0.2191
"00074f5a1c"	"00074fb7c2"	0.1207
"00074f5a1c"	"00074fbd36"	0.1251
"00074f5a1c"	"00074fc27f"	0.1158
"00074f5a1c"	"00074fc31d"	−0.0983
"00074f5a1c"	"00074fd569"	−0.1697
"00074f5a1c"	"00074fef15"	−0.0632

"00074f5a1c"	"00074ff562"	0.1024
"00074f5a1c"	"00075007ca"	-0.0489
"00074f5a1c"	"0007500b86"	0.1382
"00074f5a1c"	"0007500d05"	0.0135
"00074f5a1c"	"0007500ee4"	-0.1641
"00074f5a1c"	"0007500eee"	-0.0366
"00074f5a1c"	"00075013dc"	-0.0678
"00074f5a1c"	"000757b515"	-0.0517
"00074f5a1c"	"000757bc5a"	-0.0463
"00074f5a1c"	"000757c320"	-0.2933
"00074f5a1c"	"000757c9aa"	-0.1416
"00074f5a1c"	"000757ccbe"	-0.0386
"00074f5a1c"	"000757cfa9"	-0.1821
"00074f5a1c"	"000757d390"	-0.2526
"00074f5a1c"	"000757d393"	0.1395
"00074f5a1c"	"000757d598"	-0.2348
"00074f5a1c"	"000757d5a2"	-0.1633
"00074f5a1c"	"000757d790"	0.0105
"00074f5a1c"	"000757e30c"	-0.2397
"00074f5a1c"	"000757e4b0"	0.179
"00074f5a1c"	"000757e7a0"	-0.1336
"00074f5a1c"	"000757e8b3"	-0.1066
"00074f5a1c"	"000757f627"	-0.0734
"00074f5a1c"	"000757f925"	-0.0686
"00074f5a1c"	"000757fa08"	0.1965
"00074f5a1c"	"000757fe52"	-0.0436
"00074f5a1c"	"000758024a"	-0.182
"00074f5a1c"	"00075804bb"	-0.0139
"00074f5a1c"	"00075a0c04"	-0.0442
"00074f5a1c"	"00075a3110"	-0.0169
"00074f5a1c"	"00075a341a"	-0.108
"00074f5a1c"	"00075a3dcf"	0.4161
"00074f5a1c"	"00075a3e22"	-0.1008
"00074f5a1c"	"00075a48d8"	-0.199
"00074f5a1c"	"00075a5cfb"	-0.0301
"00074f5a1c"	"00075a6151"	-0.0499
"00074f5a1c"	"00075a6708"	-0.1509
"00074f5a1c"	"00075a7319"	-0.3502
"00074f5a1c"	"00075a7723"	0.0858
"00074f5a1c"	"00075a778b"	-0.1023
"00074f5a1c"	"00075a7b8e"	-0.0546
"00074f5a1c"	"00075a7c79"	-0.069
"00074f5a1c"	"00075a81b6"	-0.3142
"00074f5a1c"	"00075a82ac"	0.15
"00074f5a1c"	"00075a98e5"	-0.1117
"00074f5a1c"	"00075b0d29"	-0.0439
"00074f5a1c"	"00075b102a"	-0.054
"00074f5a1c"	"00075b1074"	0.0373
"00074f5a1c"	"00075b135d"	-0.1418
"00074f5a1c"	"00075b138b"	0.0423

"00074f5a1c"	"00075b13a0"	-0.1306
"00074f5a1c"	"00075b13bd"	-3e-04
"00074f5a1c"	"00075b16a9"	-0.1761
"00074f5a1c"	"00075b1a28"	0.068
"00074f5a1c"	"00075b1a97"	0.1215
"00074f5a1c"	"00075b1c7b"	-0.4858
"00074f5a1c"	"00075b1d24"	-0.0861
"00074f5a1c"	"00075b202b"	-0.0426
"00074f5a1c"	"00075b22cb"	-0.0698
"00074f5a1c"	"00075b22da"	0.0091
"00074f5a1c"	"00075b2556"	-0.0813
"00074f5a1c"	"00075b25de"	0.0343
"00074f5a1c"	"00075b260c"	0.1276
"00074f5a1c"	"00075b26f1"	-0.0687
"00074f5a1c"	"00075b2920"	-0.0634
"00074f5a1c"	"00075b2a64"	-0.0927
"00074f5a1c"	"00075b2a9d"	-0.1822
"00074f5a1c"	"00075b2b37"	-0.428
"00074f5a1c"	"00075b2cdd"	0.1019
"00074f5a1c"	"00075b3038"	0.1338
"00074f5a1c"	"00075b30fe"	0.127
"00074f5a1c"	"00075b3362"	-0.4125
"00074f5a1c"	"00075b350a"	0.0361
"00074f5a1c"	"00075b350e"	0.3045
"00074f5a1c"	"00075b3651"	-0.1895
"00074f5a1c"	"00075b38ca"	0.6022
"00074f5a1c"	"00075b39cc"	-0.0534
"00074f5a1c"	"00075b3e1e"	0.2439
"00074f5a1c"	"00075b3e57"	-0.154
"00074f5a1c"	"00075b4079"	-0.2031
"00074f5a1c"	"00075b4150"	-0.0269
"00074f5a1c"	"00075b4194"	0.1178
"00074f5a1c"	"00075b42d5"	0.6098
"00074f5a1c"	"00075b4424"	0.0655
"00074f5a1c"	"00075b4470"	0.0448
"00074f5a1c"	"00075b47ed"	-0.0079
"00074f5a1c"	"00075b4850"	-0.0352
"00074f5a1c"	"00075b4ca0"	-0.0527
"00074f5a1c"	"00075b4d7f"	0.0525
"00074f5a1c"	"00075b520f"	0.0075
"00074f5a1c"	"00075b525f"	-0.1154
"00074f5a1c"	"00075b58f8"	-0.1364
"00074f5a1c"	"00075b5bcc"	0.045
"00074f5a1c"	"00075b5bfa"	-0.0607
"00074f5a1c"	"00075b6339"	0.0643
"00074f5a1c"	"00075b6658"	-0.1823
"00074f5a1c"	"00075b679a"	-0.0805
"00074f5a1c"	"00075b6cb7"	-0.255
"00074f5a1c"	"00075b6df8"	0.0563
"00074f5a1c"	"00075b6ff6"	0.0674

"00074f5a1c"	"00075b70ee"	−0.1528
"00074f5a1c"	"00075b7157"	0.1186
"00074f5a1c"	"00075b7225"	0.2363
"00074f5a1c"	"00075b7c89"	−0.057
"00074f5a1c"	"00075b9048"	0.1825
"00074f5a1c"	"00075d0801"	−0.0656
"00074f5a1c"	"00075d1820"	−0.3145
"00074f5a1c"	"00075d1f3d"	0.0972
"00074f5a1c"	"00075d2329"	0.3123
"00074f5a1c"	"00075d2b9b"	0.2078
"00074f5a1c"	"00075d3941"	−0.1417
"00074f5a1c"	"00075d3e96"	−0.1793
"00074f5a1c"	"00075d4864"	0.4288
"00074f5a1c"	"00075d5961"	0.045
"00074f5a1c"	"00075d5a63"	0.0531
"00074f5a1c"	"00075d6150"	0.1555
"00074f5a1c"	"00075d67d0"	−0.2659
"00074f5a1c"	"00075d67e2"	−0.1524
"00074f5a1c"	"00075d73fc"	−0.0596
"00074f5a1c"	"00075d7729"	−0.0833
"00074f5a1c"	"00075d778c"	−0.1132
"00074f5a1c"	"00075d7b9e"	−0.1328
"00074f5a1c"	"00075d7c8f"	−0.0925
"00074f5a1c"	"00075d804d"	−0.1221
"00074f5a1c"	"00075d819f"	−0.1005
"00074f5a1c"	"00075d8601"	0.0169
"00074f5a1c"	"00075d8c6a"	0.1476
"00074f5a1c"	"00075dfedc"	0.0397
"00074f5a1c"	"00075e05f2"	0.231
"00074f5a1c"	"00075e0837"	0.0785
"00074f5a1c"	"00075e092e"	−0.0219
"00074f5a1c"	"00075e0965"	−0.0865
"00074f5a1c"	"00075e0bc8"	0.0788
"00074f5a1c"	"00075e0fbb"	0.0274
"00074f75b7"	"00074f8cd9"	0.3351
"00074f75b7"	"00074f96dc"	−0.0884
"00074f75b7"	"00074fabaa"	0.0125
"00074f75b7"	"00074facd9"	0.1233
"00074f75b7"	"00074fae3c"	−0.0269
"00074f75b7"	"00074fb0a8"	−0.0173
"00074f75b7"	"00074fb4e4"	−0.0963
"00074f75b7"	"00074fb7c2"	−0.0302
"00074f75b7"	"00074fbd36"	−0.2234
"00074f75b7"	"00074fc27f"	0.1935
"00074f75b7"	"00074fc31d"	−0.0998
"00074f75b7"	"00074fd569"	0.0904
"00074f75b7"	"00074fef15"	0.012
"00074f75b7"	"00074ff562"	−0.1895
"00074f75b7"	"00075007ca"	0.049
"00074f75b7"	"0007500b86"	0.3067

"00074f75b7"	"0007500d05"	0.4159
"00074f75b7"	"0007500ee4"	0.0057
"00074f75b7"	"0007500eee"	0.2685
"00074f75b7"	"00075013dc"	−0.279
"00074f75b7"	"000757b515"	0.1211
"00074f75b7"	"000757bc5a"	0.0586
"00074f75b7"	"000757c320"	−0.1149
"00074f75b7"	"000757c9aa"	−0.0114
"00074f75b7"	"000757ccbe"	−0.2691
"00074f75b7"	"000757cfa9"	−0.3114
"00074f75b7"	"000757d390"	−0.1145
"00074f75b7"	"000757d393"	0.0365
"00074f75b7"	"000757d598"	−0.1477
"00074f75b7"	"000757d5a2"	−0.2173
"00074f75b7"	"000757d790"	0.0219
"00074f75b7"	"000757e30c"	−0.0576
"00074f75b7"	"000757e4b0"	0.0462
"00074f75b7"	"000757e7a0"	0.1043
"00074f75b7"	"000757e8b3"	−0.0509
"00074f75b7"	"000757f627"	−0.1473
"00074f75b7"	"000757f925"	0.0108
"00074f75b7"	"000757fa08"	−0.022
"00074f75b7"	"000757fe52"	0.2018
"00074f75b7"	"000758024a"	−0.1353
"00074f75b7"	"00075804bb"	−0.1514
"00074f75b7"	"00075a0c04"	0.1005
"00074f75b7"	"00075a3110"	−0.0376
"00074f75b7"	"00075a341a"	−0.12
"00074f75b7"	"00075a3dcf"	0.1797
"00074f75b7"	"00075a3e22"	−0.0991
"00074f75b7"	"00075a48d8"	−0.0497
"00074f75b7"	"00075a5cfb"	0.0016
"00074f75b7"	"00075a6151"	0.03
"00074f75b7"	"00075a6708"	0.0465
"00074f75b7"	"00075a7319"	−0.2615
"00074f75b7"	"00075a7723"	0.0331
"00074f75b7"	"00075a778b"	−0.2698
"00074f75b7"	"00075a7b8e"	0.1534
"00074f75b7"	"00075a7c79"	0.1492
"00074f75b7"	"00075a81b6"	−0.1905
"00074f75b7"	"00075a82ac"	0.1977
"00074f75b7"	"00075a98e5"	−0.2144
"00074f75b7"	"00075b0d29"	−0.1021
"00074f75b7"	"00075b102a"	−0.06
"00074f75b7"	"00075b1074"	−0.3906
"00074f75b7"	"00075b135d"	−0.2795
"00074f75b7"	"00075b138b"	0.1209
"00074f75b7"	"00075b13a0"	−0.2264
"00074f75b7"	"00075b13bd"	−0.113
"00074f75b7"	"00075b16a9"	0.0851

"00074f75b7"	"00075b1a28"	−0.0847
"00074f75b7"	"00075b1a97"	0.0318
"00074f75b7"	"00075b1c7b"	−0.1831
"00074f75b7"	"00075b1d24"	0.1233
"00074f75b7"	"00075b202b"	0.1691
"00074f75b7"	"00075b22cb"	0.0504
"00074f75b7"	"00075b22da"	−0.2746
"00074f75b7"	"00075b2556"	−0.2754
"00074f75b7"	"00075b25de"	0.5332
"00074f75b7"	"00075b260c"	0.0158
"00074f75b7"	"00075b26f1"	0.1107
"00074f75b7"	"00075b2920"	−1e−04
"00074f75b7"	"00075b2a64"	0.0579
"00074f75b7"	"00075b2a9d"	−0.2612
"00074f75b7"	"00075b2b37"	−0.4244
"00074f75b7"	"00075b2cdd"	0.0122
"00074f75b7"	"00075b3038"	0.5412
"00074f75b7"	"00075b30fe"	−0.1494
"00074f75b7"	"00075b3362"	−0.2162
"00074f75b7"	"00075b350a"	−0.1117
"00074f75b7"	"00075b350e"	0.1141
"00074f75b7"	"00075b3651"	0.1571
"00074f75b7"	"00075b38ca"	0.3047
"00074f75b7"	"00075b39cc"	−0.0419
"00074f75b7"	"00075b3e1e"	0.0323
"00074f75b7"	"00075b3e57"	0.0915
"00074f75b7"	"00075b4079"	−0.0309
"00074f75b7"	"00075b4150"	−0.0203
"00074f75b7"	"00075b4194"	0.168
"00074f75b7"	"00075b42d5"	0.311
"00074f75b7"	"00075b4424"	0.0673
"00074f75b7"	"00075b4470"	0.043
"00074f75b7"	"00075b47ed"	−0.0362
"00074f75b7"	"00075b4850"	0.0654
"00074f75b7"	"00075b4ca0"	−0.2079
"00074f75b7"	"00075b4d7f"	0.2673
"00074f75b7"	"00075b520f"	−0.0362
"00074f75b7"	"00075b525f"	−0.1781
"00074f75b7"	"00075b58f8"	−0.0288
"00074f75b7"	"00075b5bcc"	0.3384
"00074f75b7"	"00075b5bfa"	0.4934
"00074f75b7"	"00075b6339"	0.1068
"00074f75b7"	"00075b6658"	−0.0371
"00074f75b7"	"00075b679a"	0.188
"00074f75b7"	"00075b6cb7"	−0.1403
"00074f75b7"	"00075b6df8"	0.1891
"00074f75b7"	"00075b6ff6"	0.2078
"00074f75b7"	"00075b70ee"	−0.0842
"00074f75b7"	"00075b7157"	0.2287
"00074f75b7"	"00075b7225"	0.0906

"00074f75b7"	"00075b7c89"	0.01
"00074f75b7"	"00075b9048"	0.2624
"00074f75b7"	"00075d0801"	0.1057
"00074f75b7"	"00075d1820"	−0.0646
"00074f75b7"	"00075d1f3d"	0.0563
"00074f75b7"	"00075d2329"	0.1654
"00074f75b7"	"00075d2b9b"	0.0325
"00074f75b7"	"00075d3941"	−0.0593
"00074f75b7"	"00075d3e96"	0.1411
"00074f75b7"	"00075d4864"	−0.2117
"00074f75b7"	"00075d5961"	0.129
"00074f75b7"	"00075d5a63"	0.1546
"00074f75b7"	"00075d6150"	0.1054
"00074f75b7"	"00075d67d0"	−0.0857
"00074f75b7"	"00075d67e2"	−0.251
"00074f75b7"	"00075d73fc"	0.0964
"00074f75b7"	"00075d7729"	−0.07
"00074f75b7"	"00075d778c"	0.0242
"00074f75b7"	"00075d7b9e"	0.1273
"00074f75b7"	"00075d7c8f"	−0.0902
"00074f75b7"	"00075d804d"	0.0474
"00074f75b7"	"00075d819f"	−0.2336
"00074f75b7"	"00075d8601"	0.1237
"00074f75b7"	"00075d8c6a"	0.1826
"00074f75b7"	"00075dfedc"	0.3428
"00074f75b7"	"00075e05f2"	0.3437
"00074f75b7"	"00075e0837"	0.0573
"00074f75b7"	"00075e092e"	−0.2279
"00074f75b7"	"00075e0965"	−0.1346
"00074f75b7"	"00075e0bc8"	0.1605
"00074f75b7"	"00075e0fbb"	−0.2093
"00074f8cd9"	"00074f96dc"	−0.0671
"00074f8cd9"	"00074fabaa"	−0.0385
"00074f8cd9"	"00074facd9"	0.3436
"00074f8cd9"	"00074fae3c"	0.1273
"00074f8cd9"	"00074fb0a8"	0.017
"00074f8cd9"	"00074fb4e4"	−0.1757
"00074f8cd9"	"00074fb7c2"	0.1904
"00074f8cd9"	"00074fbd36"	−0.3169
"00074f8cd9"	"00074fc27f"	−0.0767
"00074f8cd9"	"00074fc31d"	−0.0639
"00074f8cd9"	"00074fd569"	0.0994
"00074f8cd9"	"00074fef15"	−0.1528
"00074f8cd9"	"00074ff562"	0.0094
"00074f8cd9"	"00075007ca"	0.0533
"00074f8cd9"	"0007500b86"	0.4663
"00074f8cd9"	"0007500d05"	0.6087
"00074f8cd9"	"0007500ee4"	0.0607
"00074f8cd9"	"0007500eee"	0.2487
"00074f8cd9"	"00075013dc"	−0.1283

"00074f8cd9"	"000757b515"	-0.1459
"00074f8cd9"	"000757bc5a"	0.1942
"00074f8cd9"	"000757c320"	-0.1477
"00074f8cd9"	"000757c9aa"	-0.2077
"00074f8cd9"	"000757ccbe"	0.0244
"00074f8cd9"	"000757cfa9"	-0.1597
"00074f8cd9"	"000757d390"	-0.151
"00074f8cd9"	"000757d393"	0.2241
"00074f8cd9"	"000757d598"	-0.3809
"00074f8cd9"	"000757d5a2"	-0.3135
"00074f8cd9"	"000757d790"	0.0923
"00074f8cd9"	"000757e30c"	-0.1073
"00074f8cd9"	"000757e4b0"	-0.1144
"00074f8cd9"	"000757e7a0"	-0.103
"00074f8cd9"	"000757e8b3"	-0.0218
"00074f8cd9"	"000757f627"	0.1645
"00074f8cd9"	"000757f925"	0.3256
"00074f8cd9"	"000757fa08"	0.2133
"00074f8cd9"	"000757fe52"	0.0873
"00074f8cd9"	"000758024a"	0.0793
"00074f8cd9"	"00075804bb"	-0.0342
"00074f8cd9"	"00075a0c04"	0.3235
"00074f8cd9"	"00075a3110"	0.0348
"00074f8cd9"	"00075a341a"	0.0139
"00074f8cd9"	"00075a3dcf"	0.09
"00074f8cd9"	"00075a3e22"	0.1648
"00074f8cd9"	"00075a48d8"	0.23
"00074f8cd9"	"00075a5cfb"	0.0258
"00074f8cd9"	"00075a6151"	-0.0793
"00074f8cd9"	"00075a6708"	0.0665
"00074f8cd9"	"00075a7319"	-0.1594
"00074f8cd9"	"00075a7723"	0.2715
"00074f8cd9"	"00075a778b"	0.1114
"00074f8cd9"	"00075a7b8e"	0.3751
"00074f8cd9"	"00075a7c79"	0.0083
"00074f8cd9"	"00075a81b6"	-0.0339
"00074f8cd9"	"00075a82ac"	0.147
"00074f8cd9"	"00075a98e5"	0.2208
"00074f8cd9"	"00075b0d29"	0.117
"00074f8cd9"	"00075b102a"	0.0709
"00074f8cd9"	"00075b1074"	-0.2022
"00074f8cd9"	"00075b135d"	-0.0948
"00074f8cd9"	"00075b138b"	0.1865
"00074f8cd9"	"00075b13a0"	-0.1393
"00074f8cd9"	"00075b13bd"	-0.0641
"00074f8cd9"	"00075b16a9"	0.2294
"00074f8cd9"	"00075b1a28"	0.1494
"00074f8cd9"	"00075b1a97"	0.0689
"00074f8cd9"	"00075b1c7b"	-0.1542
"00074f8cd9"	"00075b1d24"	0.084

"00074f8cd9"	"00075b202b"	0.0769
"00074f8cd9"	"00075b22cb"	0.0818
"00074f8cd9"	"00075b22da"	−0.125
"00074f8cd9"	"00075b2556"	−0.2907
"00074f8cd9"	"00075b25de"	0.2661
"00074f8cd9"	"00075b260c"	−0.0782
"00074f8cd9"	"00075b26f1"	−0.0492
"00074f8cd9"	"00075b2920"	0.0299
"00074f8cd9"	"00075b2a64"	−0.0089
"00074f8cd9"	"00075b2a9d"	0.0356
"00074f8cd9"	"00075b2b37"	−0.1298
"00074f8cd9"	"00075b2cdd"	0.0961
"00074f8cd9"	"00075b3038"	0.3247
"00074f8cd9"	"00075b30fe"	−0.3018
"00074f8cd9"	"00075b3362"	−0.2939
"00074f8cd9"	"00075b350a"	−0.0199
"00074f8cd9"	"00075b350e"	0.0029
"00074f8cd9"	"00075b3651"	−0.1008
"00074f8cd9"	"00075b38ca"	0.1175
"00074f8cd9"	"00075b39cc"	0.0388
"00074f8cd9"	"00075b3e1e"	0.4648
"00074f8cd9"	"00075b3e57"	0.1262
"00074f8cd9"	"00075b4079"	0.0651
"00074f8cd9"	"00075b4150"	0.1014
"00074f8cd9"	"00075b4194"	0.2232
"00074f8cd9"	"00075b42d5"	0.3069
"00074f8cd9"	"00075b4424"	−0.2316
"00074f8cd9"	"00075b4470"	0.1786
"00074f8cd9"	"00075b47ed"	0.1935
"00074f8cd9"	"00075b4850"	0.0359
"00074f8cd9"	"00075b4ca0"	−0.3981
"00074f8cd9"	"00075b4d7f"	0.4255
"00074f8cd9"	"00075b520f"	0.1254
"00074f8cd9"	"00075b525f"	−0.3302
"00074f8cd9"	"00075b58f8"	−0.027
"00074f8cd9"	"00075b5bcc"	0.1758
"00074f8cd9"	"00075b5bfa"	−0.0681
"00074f8cd9"	"00075b6339"	0.0992
"00074f8cd9"	"00075b6658"	−0.3376
"00074f8cd9"	"00075b679a"	0.19
"00074f8cd9"	"00075b6cb7"	−0.1852
"00074f8cd9"	"00075b6df8"	0.3475
"00074f8cd9"	"00075b6ff6"	0.0197
"00074f8cd9"	"00075b70ee"	−0.0184
"00074f8cd9"	"00075b7157"	0.097
"00074f8cd9"	"00075b7225"	0.103
"00074f8cd9"	"00075b7c89"	0.0502
"00074f8cd9"	"00075b9048"	0.1527
"00074f8cd9"	"00075d0801"	0.2195
"00074f8cd9"	"00075d1820"	−0.0254

"00074f8cd9"	"00075d1f3d"	-0.0139
"00074f8cd9"	"00075d2329"	0.2403
"00074f8cd9"	"00075d2b9b"	-0.2005
"00074f8cd9"	"00075d3941"	-0.0432
"00074f8cd9"	"00075d3e96"	-0.0539
"00074f8cd9"	"00075d4864"	-0.0801
"00074f8cd9"	"00075d5961"	0.2892
"00074f8cd9"	"00075d5a63"	0.1023
"00074f8cd9"	"00075d6150"	-0.009
"00074f8cd9"	"00075d67d0"	-0.2621
"00074f8cd9"	"00075d67e2"	0.0463
"00074f8cd9"	"00075d73fc"	0.2132
"00074f8cd9"	"00075d7729"	-0.3048
"00074f8cd9"	"00075d778c"	-0.2761
"00074f8cd9"	"00075d7b9e"	0.0292
"00074f8cd9"	"00075d7c8f"	0.1104
"00074f8cd9"	"00075d804d"	0.1363
"00074f8cd9"	"00075d819f"	-0.1398
"00074f8cd9"	"00075d8601"	0.2316
"00074f8cd9"	"00075d8c6a"	0.3802
"00074f8cd9"	"00075dfedc"	0.2146
"00074f8cd9"	"00075e05f2"	0.0606
"00074f8cd9"	"00075e0837"	0.0245
"00074f8cd9"	"00075e092e"	0.0128
"00074f8cd9"	"00075e0965"	-0.0458
"00074f8cd9"	"00075e0bc8"	0.0601
"00074f8cd9"	"00075e0fbb"	-0.2215
"00074f96dc"	"00074fabaa"	-0.052
"00074f96dc"	"00074facd9"	-0.2041
"00074f96dc"	"00074fae3c"	0.2667
"00074f96dc"	"00074fb0a8"	-0.2019
"00074f96dc"	"00074fb4e4"	-0.1745
"00074f96dc"	"00074fb7c2"	-0.178
"00074f96dc"	"00074fbd36"	-0.1935
"00074f96dc"	"00074fc27f"	-0.4987
"00074f96dc"	"00074fc31d"	-0.3231
"00074f96dc"	"00074fd569"	0.2584
"00074f96dc"	"00074fef15"	-0.0158
"00074f96dc"	"00074ff562"	-0.4497
"00074f96dc"	"00075007ca"	-0.377
"00074f96dc"	"0007500b86"	-0.0526
"00074f96dc"	"0007500d05"	-0.1413
"00074f96dc"	"0007500ee4"	-0.0907
"00074f96dc"	"0007500eee"	-0.2655
"00074f96dc"	"00075013dc"	-0.1983
"00074f96dc"	"000757b515"	-0.4926
"00074f96dc"	"000757bc5a"	0.0688
"00074f96dc"	"000757c320"	-0.2569
"00074f96dc"	"000757c9aa"	-0.218
"00074f96dc"	"000757ccbe"	-0.1597

"00074f96dc"	"000757cfa9"	-0.3691
"00074f96dc"	"000757d390"	-0.4005
"00074f96dc"	"000757d393"	-0.284
"00074f96dc"	"000757d598"	-0.5306
"00074f96dc"	"000757d5a2"	-0.5011
"00074f96dc"	"000757d790"	-0.4797
"00074f96dc"	"000757e30c"	-0.3555
"00074f96dc"	"000757e4b0"	-0.0033
"00074f96dc"	"000757e7a0"	0.0023
"00074f96dc"	"000757e8b3"	-0.0205
"00074f96dc"	"000757f627"	-0.1443
"00074f96dc"	"000757f925"	-0.1716
"00074f96dc"	"000757fa08"	-0.3145
"00074f96dc"	"000757fe52"	-0.0426
"00074f96dc"	"000758024a"	-0.3351
"00074f96dc"	"00075804bb"	-0.3757
"00074f96dc"	"00075a0c04"	4e-04
"00074f96dc"	"00075a3110"	-0.2294
"00074f96dc"	"00075a341a"	-0.0966
"00074f96dc"	"00075a3dcf"	-0.1575
"00074f96dc"	"00075a3e22"	-0.0882
"00074f96dc"	"00075a48d8"	-0.0092
"00074f96dc"	"00075a5cfb"	-0.1711
"00074f96dc"	"00075a6151"	-0.5122
"00074f96dc"	"00075a6708"	-0.3404
"00074f96dc"	"00075a7319"	-0.3641
"00074f96dc"	"00075a7723"	-0.3198
"00074f96dc"	"00075a778b"	-0.2044
"00074f96dc"	"00075a7b8e"	-0.2529
"00074f96dc"	"00075a7c79"	-0.1472
"00074f96dc"	"00075a81b6"	-0.1135
"00074f96dc"	"00075a82ac"	-0.0945
"00074f96dc"	"00075a98e5"	0.0428
"00074f96dc"	"00075b0d29"	0.0092
"00074f96dc"	"00075b102a"	-0.1251
"00074f96dc"	"00075b1074"	-0.0706
"00074f96dc"	"00075b135d"	-0.1076
"00074f96dc"	"00075b138b"	-0.0178
"00074f96dc"	"00075b13a0"	-0.3683
"00074f96dc"	"00075b13bd"	0.5601
"00074f96dc"	"00075b16a9"	-0.235
"00074f96dc"	"00075b1a28"	0.1109
"00074f96dc"	"00075b1a97"	0.1342
"00074f96dc"	"00075b1c7b"	-0.2008
"00074f96dc"	"00075b1d24"	-0.0276
"00074f96dc"	"00075b202b"	-0.0125
"00074f96dc"	"00075b22cb"	0.0671
"00074f96dc"	"00075b22da"	-0.219
"00074f96dc"	"00075b2556"	-0.3635
"00074f96dc"	"00075b25de"	-0.1569

"00074f96dc"	"00075b260c"	−0.4843
"00074f96dc"	"00075b26f1"	0.0552
"00074f96dc"	"00075b2920"	−0.3497
"00074f96dc"	"00075b2a64"	−0.2938
"00074f96dc"	"00075b2a9d"	−0.3063
"00074f96dc"	"00075b2b37"	−0.243
"00074f96dc"	"00075b2cdd"	0.0571
"00074f96dc"	"00075b3038"	−0.1949
"00074f96dc"	"00075b30fe"	−0.344
"00074f96dc"	"00075b3362"	0.0057
"00074f96dc"	"00075b350a"	−0.019
"00074f96dc"	"00075b350e"	−0.064
"00074f96dc"	"00075b3651"	0.1431
"00074f96dc"	"00075b38ca"	−0.2021
"00074f96dc"	"00075b39cc"	−0.1103
"00074f96dc"	"00075b3e1e"	−0.1336
"00074f96dc"	"00075b3e57"	0.3522
"00074f96dc"	"00075b4079"	0.0261
"00074f96dc"	"00075b4150"	−0.4162
"00074f96dc"	"00075b4194"	−0.045
"00074f96dc"	"00075b42d5"	−0.1422
"00074f96dc"	"00075b4424"	0.0615
"00074f96dc"	"00075b4470"	0.0532
"00074f96dc"	"00075b47ed"	−0.0777
"00074f96dc"	"00075b4850"	−0.0316
"00074f96dc"	"00075b4ca0"	−0.2049
"00074f96dc"	"00075b4d7f"	0.1702
"00074f96dc"	"00075b520f"	0.0769
"00074f96dc"	"00075b525f"	−0.0465
"00074f96dc"	"00075b58f8"	−0.2928
"00074f96dc"	"00075b5bcc"	0.0605
"00074f96dc"	"00075b5bfa"	−0.5342
"00074f96dc"	"00075b6339"	0.0011
"00074f96dc"	"00075b6658"	0.2776
"00074f96dc"	"00075b679a"	−0.1058
"00074f96dc"	"00075b6cb7"	−0.3594
"00074f96dc"	"00075b6df8"	−0.1489
"00074f96dc"	"00075b6ff6"	−0.2882
"00074f96dc"	"00075b70ee"	−0.2808
"00074f96dc"	"00075b7157"	−0.1177
"00074f96dc"	"00075b7225"	−0.1498
"00074f96dc"	"00075b7c89"	−0.0665
"00074f96dc"	"00075b9048"	−0.3708
"00074f96dc"	"00075d0801"	−0.1843
"00074f96dc"	"00075d1820"	0.1233
"00074f96dc"	"00075d1f3d"	−0.1522
"00074f96dc"	"00075d2329"	−0.2431
"00074f96dc"	"00075d2b9b"	−0.2395
"00074f96dc"	"00075d3941"	0.1324
"00074f96dc"	"00075d3e96"	0.0028

"00074f96dc"	"00075d4864"	−0.2992
"00074f96dc"	"00075d5961"	−0.1628
"00074f96dc"	"00075d5a63"	0.2021
"00074f96dc"	"00075d6150"	−0.0772
"00074f96dc"	"00075d67d0"	−0.2509
"00074f96dc"	"00075d67e2"	0.0036
"00074f96dc"	"00075d73fc"	−0.0718
"00074f96dc"	"00075d7729"	−0.2053
"00074f96dc"	"00075d778c"	−0.1547
"00074f96dc"	"00075d7b9e"	−0.1647
"00074f96dc"	"00075d7c8f"	0.0111
"00074f96dc"	"00075d804d"	−0.0749
"00074f96dc"	"00075d819f"	−0.2886
"00074f96dc"	"00075d8601"	0.2982
"00074f96dc"	"00075d8c6a"	−0.062
"00074f96dc"	"00075dfedc"	0.0775
"00074f96dc"	"00075e05f2"	−0.1839
"00074f96dc"	"00075e0837"	−0.1607
"00074f96dc"	"00075e092e"	−0.2328
"00074f96dc"	"00075e0965"	−0.5533
"00074f96dc"	"00075e0bc8"	−0.1774
"00074f96dc"	"00075e0fbb"	−0.53
"00074fabaa"	"00074facd9"	−0.0411
"00074fabaa"	"00074fae3c"	−0.1693
"00074fabaa"	"00074fb0a8"	0.1772
"00074fabaa"	"00074fb4e4"	−0.0409
"00074fabaa"	"00074fb7c2"	0.2687
"00074fabaa"	"00074fbd36"	−0.0151
"00074fabaa"	"00074fc27f"	0.0854
"00074fabaa"	"00074fc31d"	−0.2202
"00074fabaa"	"00074fd569"	−0.0747
"00074fabaa"	"00074fef15"	−0.3356
"00074fabaa"	"00074ff562"	−0.1653
"00074fabaa"	"00075007ca"	−0.2102
"00074fabaa"	"0007500b86"	0.3131
"00074fabaa"	"0007500d05"	0.0509
"00074fabaa"	"0007500ee4"	−0.2214
"00074fabaa"	"0007500eee"	−0.1522
"00074fabaa"	"00075013dc"	0.1507
"00074fabaa"	"000757b515"	−0.0637
"00074fabaa"	"000757bc5a"	0.3029
"00074fabaa"	"000757c320"	0.0587
"00074fabaa"	"000757c9aa"	−0.0418
"00074fabaa"	"000757ccbe"	−0.0244
"00074fabaa"	"000757cfa9"	−0.1819
"00074fabaa"	"000757d390"	−0.0748
"00074fabaa"	"000757d393"	0.086
"00074fabaa"	"000757d598"	−0.1572
"00074fabaa"	"000757d5a2"	−0.4011
"00074fabaa"	"000757d790"	−0.0367

"00074fabaa"	"000757e30c"	−0.044
"00074fabaa"	"000757e4b0"	0.2951
"00074fabaa"	"000757e7a0"	−0.3131
"00074fabaa"	"000757e8b3"	0.1806
"00074fabaa"	"000757f627"	0.055
"00074fabaa"	"000757f925"	0.099
"00074fabaa"	"000757fa08"	−0.1647
"00074fabaa"	"000757fe52"	0.0832
"00074fabaa"	"000758024a"	−0.1477
"00074fabaa"	"00075804bb"	0.1896
"00074fabaa"	"00075a0c04"	−0.0712
"00074fabaa"	"00075a3110"	0.1654
"00074fabaa"	"00075a341a"	−0.2095
"00074fabaa"	"00075a3dcf"	−0.0593
"00074fabaa"	"00075a3e22"	0.1274
"00074fabaa"	"00075a48d8"	−0.0351
"00074fabaa"	"00075a5cfb"	0.2029
"00074fabaa"	"00075a6151"	−0.1432
"00074fabaa"	"00075a6708"	−0.3141
"00074fabaa"	"00075a7319"	−0.2001
"00074fabaa"	"00075a7723"	0.0137
"00074fabaa"	"00075a778b"	−0.0159
"00074fabaa"	"00075a7b8e"	0.0796
"00074fabaa"	"00075a7c79"	0.1875
"00074fabaa"	"00075a81b6"	−0.3682
"00074fabaa"	"00075a82ac"	0.2196
"00074fabaa"	"00075a98e5"	−0.0419
"00074fabaa"	"00075b0d29"	0.0062
"00074fabaa"	"00075b102a"	0.1653
"00074fabaa"	"00075b1074"	−0.0649
"00074fabaa"	"00075b135d"	0.0089
"00074fabaa"	"00075b138b"	0.3881
"00074fabaa"	"00075b13a0"	−0.2318
"00074fabaa"	"00075b13bd"	−0.1458
"00074fabaa"	"00075b16a9"	−0.2448
"00074fabaa"	"00075b1a28"	−0.0934
"00074fabaa"	"00075b1a97"	−0.1022
"00074fabaa"	"00075b1c7b"	−0.5391
"00074fabaa"	"00075b1d24"	−0.1823
"00074fabaa"	"00075b202b"	0.2244
"00074fabaa"	"00075b22cb"	−0.0555
"00074fabaa"	"00075b22da"	−0.0993
"00074fabaa"	"00075b2556"	0.0265
"00074fabaa"	"00075b25de"	0.0155
"00074fabaa"	"00075b260c"	0.1289
"00074fabaa"	"00075b26f1"	−0.2842
"00074fabaa"	"00075b2920"	0.1606
"00074fabaa"	"00075b2a64"	0.2715
"00074fabaa"	"00075b2a9d"	0.0396
"00074fabaa"	"00075b2b37"	−0.5547

"00074fabaa"	"00075b2cdd"	0.1285
"00074fabaa"	"00075b3038"	0.0687
"00074fabaa"	"00075b30fe"	−0.0242
"00074fabaa"	"00075b3362"	−0.1641
"00074fabaa"	"00075b350a"	0.1173
"00074fabaa"	"00075b350e"	0.389
"00074fabaa"	"00075b3651"	−0.1146
"00074fabaa"	"00075b38ca"	0.1563
"00074fabaa"	"00075b39cc"	−0.0745
"00074fabaa"	"00075b3e1e"	0.1671
"00074fabaa"	"00075b3e57"	−0.1666
"00074fabaa"	"00075b4079"	−0.0596
"00074fabaa"	"00075b4150"	−0.071
"00074fabaa"	"00075b4194"	0.4366
"00074fabaa"	"00075b42d5"	0.1695
"00074fabaa"	"00075b4424"	−0.0368
"00074fabaa"	"00075b4470"	0.337
"00074fabaa"	"00075b47ed"	0.0363
"00074fabaa"	"00075b4850"	0.2729
"00074fabaa"	"00075b4ca0"	−0.042
"00074fabaa"	"00075b4d7f"	0.075
"00074fabaa"	"00075b520f"	−0.3557
"00074fabaa"	"00075b525f"	−0.128
"00074fabaa"	"00075b58f8"	−0.0271
"00074fabaa"	"00075b5bcc"	0.0561
"00074fabaa"	"00075b5bfa"	−0.2355
"00074fabaa"	"00075b6339"	−0.0762
"00074fabaa"	"00075b6658"	−0.3098
"00074fabaa"	"00075b679a"	−0.2038
"00074fabaa"	"00075b6cb7"	0.0546
"00074fabaa"	"00075b6df8"	−0.3012
"00074fabaa"	"00075b6ff6"	0.0324
"00074fabaa"	"00075b70ee"	−0.1649
"00074fabaa"	"00075b7157"	−0.0368
"00074fabaa"	"00075b7225"	−0.1411
"00074fabaa"	"00075b7c89"	0.0049
"00074fabaa"	"00075b9048"	0.2009
"00074fabaa"	"00075d0801"	0.1632
"00074fabaa"	"00075d1820"	−0.3396
"00074fabaa"	"00075d1f3d"	0.3639
"00074fabaa"	"00075d2329"	−0.0526
"00074fabaa"	"00075d2b9b"	0.6015
"00074fabaa"	"00075d3941"	−0.0028
"00074fabaa"	"00075d3e96"	−0.1451
"00074fabaa"	"00075d4864"	0.0533
"00074fabaa"	"00075d5961"	−0.1141
"00074fabaa"	"00075d5a63"	0.2596
"00074fabaa"	"00075d6150"	−0.2136
"00074fabaa"	"00075d67d0"	−0.3002
"00074fabaa"	"00075d67e2"	0.0265

"00074fabaa"	"00075d73fc"	−0.1664
"00074fabaa"	"00075d7729"	−0.4341
"00074fabaa"	"00075d778c"	−0.4813
"00074fabaa"	"00075d7b9e"	−0.0834
"00074fabaa"	"00075d7c8f"	−0.0077
"00074fabaa"	"00075d804d"	0.0259
"00074fabaa"	"00075d819f"	0.2438
"00074fabaa"	"00075d8601"	−0.1064
"00074fabaa"	"00075d8c6a"	−0.1244
"00074fabaa"	"00075dfedc"	0.0911
"00074fabaa"	"00075e05f2"	0.1474
"00074fabaa"	"00075e0837"	0.0835
"00074fabaa"	"00075e092e"	−0.1357
"00074fabaa"	"00075e0965"	−0.0197
"00074fabaa"	"00075e0bc8"	0.129
"00074fabaa"	"00075e0fbb"	−0.101
"00074facd9"	"00074fae3c"	0.0848
"00074facd9"	"00074fb0a8"	−0.0464
"00074facd9"	"00074fb4e4"	−0.1376
"00074facd9"	"00074fb7c2"	−0.044
"00074facd9"	"00074fbd36"	−0.1654
"00074facd9"	"00074fc27f"	−0.0866
"00074facd9"	"00074fc31d"	−0.0784
"00074facd9"	"00074fd569"	−0.1715
"00074facd9"	"00074fef15"	−0.1861
"00074facd9"	"00074ff562"	−0.2991
"00074facd9"	"00075007ca"	−0.194
"00074facd9"	"0007500b86"	0.1733
"00074facd9"	"0007500d05"	0.2088
"00074facd9"	"0007500ee4"	0.1395
"00074facd9"	"0007500eee"	0.3085
"00074facd9"	"00075013dc"	−0.3967
"00074facd9"	"000757b515"	−0.0557
"00074facd9"	"000757bc5a"	0.301
"00074facd9"	"000757c320"	−0.1864
"00074facd9"	"000757c9aa"	0.0327
"00074facd9"	"000757ccbe"	−0.2632
"00074facd9"	"000757cfa9"	−0.1254
"00074facd9"	"000757d390"	−0.0011
"00074facd9"	"000757d393"	0.1162
"00074facd9"	"000757d598"	−0.3402
"00074facd9"	"000757d5a2"	−0.1044
"00074facd9"	"000757d790"	−0.2355
"00074facd9"	"000757e30c"	0.0051
"00074facd9"	"000757e4b0"	0.1644
"00074facd9"	"000757e7a0"	0.0521
"00074facd9"	"000757e8b3"	−0.1136
"00074facd9"	"000757f627"	0.1675
"00074facd9"	"000757f925"	−0.0731
"00074facd9"	"000757fa08"	−0.2586

"00074facd9"	"000757fe52"	-0.1189
"00074facd9"	"000758024a"	-0.0746
"00074facd9"	"00075804bb"	-0.0309
"00074facd9"	"00075a0c04"	0.0775
"00074facd9"	"00075a3110"	-0.1011
"00074facd9"	"00075a341a"	-0.0954
"00074facd9"	"00075a3dcf"	-0.0204
"00074facd9"	"00075a3e22"	0.0172
"00074facd9"	"00075a48d8"	-0.073
"00074facd9"	"00075a5cfb"	-0.1485
"00074facd9"	"00075a6151"	-0.2887
"00074facd9"	"00075a6708"	0.0789
"00074facd9"	"00075a7319"	1e-04
"00074facd9"	"00075a7723"	-0.2506
"00074facd9"	"00075a778b"	-0.1557
"00074facd9"	"00075a7b8e"	0.4165
"00074facd9"	"00075a7c79"	-0.0847
"00074facd9"	"00075a81b6"	-0.1849
"00074facd9"	"00075a82ac"	0.1853
"00074facd9"	"00075a98e5"	-0.1661
"00074facd9"	"00075b0d29"	-0.0255
"00074facd9"	"00075b102a"	0.1437
"00074facd9"	"00075b1074"	-0.171
"00074facd9"	"00075b135d"	-0.3362
"00074facd9"	"00075b138b"	0.2955
"00074facd9"	"00075b13a0"	-0.288
"00074facd9"	"00075b13bd"	0.0074
"00074facd9"	"00075b16a9"	0.1784
"00074facd9"	"00075b1a28"	0.1166
"00074facd9"	"00075b1a97"	0.0913
"00074facd9"	"00075b1c7b"	-0.0807
"00074facd9"	"00075b1d24"	0.1598
"00074facd9"	"00075b202b"	0.2223
"00074facd9"	"00075b22cb"	0.1873
"00074facd9"	"00075b22da"	-0.0233
"00074facd9"	"00075b2556"	-0.0661
"00074facd9"	"00075b25de"	0.189
"00074facd9"	"00075b260c"	-0.2497
"00074facd9"	"00075b26f1"	-0.2474
"00074facd9"	"00075b2920"	0.0131
"00074facd9"	"00075b2a64"	-0.0147
"00074facd9"	"00075b2a9d"	0.1907
"00074facd9"	"00075b2b37"	-0.2152
"00074facd9"	"00075b2cdd"	0.1427
"00074facd9"	"00075b3038"	0.0923
"00074facd9"	"00075b30fe"	-0.1478
"00074facd9"	"00075b3362"	-0.3989
"00074facd9"	"00075b350a"	0.1151
"00074facd9"	"00075b350e"	-0.2793
"00074facd9"	"00075b3651"	-0.0918

"00074facd9"	"00075b38ca"	0.1094
"00074facd9"	"00075b39cc"	-0.0866
"00074facd9"	"00075b3e1e"	0.5238
"00074facd9"	"00075b3e57"	0.1048
"00074facd9"	"00075b4079"	-0.114
"00074facd9"	"00075b4150"	-0.2329
"00074facd9"	"00075b4194"	0.0784
"00074facd9"	"00075b42d5"	0.1728
"00074facd9"	"00075b4424"	-0.1334
"00074facd9"	"00075b4470"	0.2669
"00074facd9"	"00075b47ed"	-0.0384
"00074facd9"	"00075b4850"	0.2506
"00074facd9"	"00075b4ca0"	-0.3127
"00074facd9"	"00075b4d7f"	0.0643
"00074facd9"	"00075b520f"	-0.0421
"00074facd9"	"00075b525f"	-0.4659
"00074facd9"	"00075b58f8"	0.1374
"00074facd9"	"00075b5bcc"	-0.0265
"00074facd9"	"00075b5bfa"	-0.2964
"00074facd9"	"00075b6339"	-0.1968
"00074facd9"	"00075b6658"	-0.2768
"00074facd9"	"00075b679a"	-0.0132
"00074facd9"	"00075b6cb7"	0.1073
"00074facd9"	"00075b6df8"	-0.0708
"00074facd9"	"00075b6ff6"	-0.1134
"00074facd9"	"00075b70ee"	-0.2364
"00074facd9"	"00075b7157"	0.2236
"00074facd9"	"00075b7225"	-0.037
"00074facd9"	"00075b7c89"	-0.2085
"00074facd9"	"00075b9048"	0.0907
"00074facd9"	"00075d0801"	0.0903
"00074facd9"	"00075d1820"	-0.0055
"00074facd9"	"00075d1f3d"	0.1368
"00074facd9"	"00075d2329"	0.1213
"00074facd9"	"00075d2b9b"	-0.2082
"00074facd9"	"00075d3941"	-0.1427
"00074facd9"	"00075d3e96"	-0.1364
"00074facd9"	"00075d4864"	-0.1099
"00074facd9"	"00075d5961"	0.0656
"00074facd9"	"00075d5a63"	0.1314
"00074facd9"	"00075d6150"	-0.1574
"00074facd9"	"00075d67d0"	-0.1398
"00074facd9"	"00075d67e2"	-0.2016
"00074facd9"	"00075d73fc"	0.1198
"00074facd9"	"00075d7729"	-0.3014
"00074facd9"	"00075d778c"	-0.3426
"00074facd9"	"00075d7b9e"	0.1513
"00074facd9"	"00075d7c8f"	-0.0238
"00074facd9"	"00075d804d"	0.3108
"00074facd9"	"00075d819f"	0.2569

"00074facd9"	"00075d8601"	0.0565
"00074facd9"	"00075d8c6a"	−0.1453
"00074facd9"	"00075dfedc"	−0.0606
"00074facd9"	"00075e05f2"	−0.0938
"00074facd9"	"00075e0837"	0.1898
"00074facd9"	"00075e092e"	0.2115
"00074facd9"	"00075e0965"	−0.1944
"00074facd9"	"00075e0bc8"	0.3304
"00074facd9"	"00075e0fbb"	−0.1518
"00074fae3c"	"00074fb0a8"	−0.1616
"00074fae3c"	"00074fb4e4"	0.0454
"00074fae3c"	"00074fb7c2"	0.005
"00074fae3c"	"00074fbd36"	−0.193
"00074fae3c"	"00074fc27f"	−0.4078
"00074fae3c"	"00074fc31d"	0.0352
"00074fae3c"	"00074fd569"	0.1186
"00074fae3c"	"00074fef15"	0.0299
"00074fae3c"	"00074ff562"	−0.1916
"00074fae3c"	"00075007ca"	0.0232
"00074fae3c"	"0007500b86"	−0.1681
"00074fae3c"	"0007500d05"	−0.0386
"00074fae3c"	"0007500ee4"	0.0723
"00074fae3c"	"0007500eee"	0.0213
"00074fae3c"	"00075013dc"	−0.2899
"00074fae3c"	"000757b515"	−0.2743
"00074fae3c"	"000757bc5a"	0.0318
"00074fae3c"	"000757c320"	0.1583
"00074fae3c"	"000757c9aa"	0.0516
"00074fae3c"	"000757ccbe"	−0.3322
"00074fae3c"	"000757cfa9"	−0.1458
"00074fae3c"	"000757d390"	−0.2262
"00074fae3c"	"000757d393"	−0.1171
"00074fae3c"	"000757d598"	−0.4529
"00074fae3c"	"000757d5a2"	−0.2599
"00074fae3c"	"000757d790"	−0.1361
"00074fae3c"	"000757e30c"	−0.1951
"00074fae3c"	"000757e4b0"	−0.0119
"00074fae3c"	"000757e7a0"	0.0521
"00074fae3c"	"000757e8b3"	−0.1027
"00074fae3c"	"000757f627"	0.1394
"00074fae3c"	"000757f925"	−0.0374
"00074fae3c"	"000757fa08"	−0.2485
"00074fae3c"	"000757fe52"	−0.2889
"00074fae3c"	"000758024a"	−0.3163
"00074fae3c"	"00075804bb"	−0.151
"00074fae3c"	"00075a0c04"	−0.0634
"00074fae3c"	"00075a3110"	−0.3274
"00074fae3c"	"00075a341a"	−0.2163
"00074fae3c"	"00075a3dcf"	−0.0624
"00074fae3c"	"00075a3e22"	−0.191

"00074fae3c"	"00075a48d8"	−0.0231
"00074fae3c"	"00075a5cfb"	−0.2533
"00074fae3c"	"00075a6151"	−0.0727
"00074fae3c"	"00075a6708"	−0.1694
"00074fae3c"	"00075a7319"	−0.0646
"00074fae3c"	"00075a7723"	0.0173
"00074fae3c"	"00075a778b"	−0.2397
"00074fae3c"	"00075a7b8e"	0.2413
"00074fae3c"	"00075a7c79"	−0.3585
"00074fae3c"	"00075a81b6"	0.0768
"00074fae3c"	"00075a82ac"	−0.0271
"00074fae3c"	"00075a98e5"	0.0202
"00074fae3c"	"00075b0d29"	0.035
"00074fae3c"	"00075b102a"	−0.01
"00074fae3c"	"00075b1074"	−0.1034
"00074fae3c"	"00075b135d"	−0.1063
"00074fae3c"	"00075b138b"	−0.0241
"00074fae3c"	"00075b13a0"	−0.6076
"00074fae3c"	"00075b13bd"	0.2392
"00074fae3c"	"00075b16a9"	0.0065
"00074fae3c"	"00075b1a28"	0.3205
"00074fae3c"	"00075b1a97"	0.179
"00074fae3c"	"00075b1c7b"	0.0429
"00074fae3c"	"00075b1d24"	−0.0773
"00074fae3c"	"00075b202b"	0.0087
"00074fae3c"	"00075b22cb"	0.1788
"00074fae3c"	"00075b22da"	−0.2104
"00074fae3c"	"00075b2556"	−0.1429
"00074fae3c"	"00075b25de"	0.0257
"00074fae3c"	"00075b260c"	−0.2817
"00074fae3c"	"00075b26f1"	0.5344
"00074fae3c"	"00075b2920"	−0.1448
"00074fae3c"	"00075b2a64"	−0.4698
"00074fae3c"	"00075b2a9d"	0.0354
"00074fae3c"	"00075b2b37"	0.0917
"00074fae3c"	"00075b2cdd"	0.2914
"00074fae3c"	"00075b3038"	−0.0303
"00074fae3c"	"00075b30fe"	−0.3023
"00074fae3c"	"00075b3362"	−0.1103
"00074fae3c"	"00075b350a"	−0.1249
"00074fae3c"	"00075b350e"	−0.2008
"00074fae3c"	"00075b3651"	0.0344
"00074fae3c"	"00075b38ca"	−0.0557
"00074fae3c"	"00075b39cc"	0.0959
"00074fae3c"	"00075b3e1e"	0.1382
"00074fae3c"	"00075b3e57"	0.1795
"00074fae3c"	"00075b4079"	−0.1235
"00074fae3c"	"00075b4150"	−0.3505
"00074fae3c"	"00075b4194"	−0.0042
"00074fae3c"	"00075b42d5"	−0.1131

"00074fae3c"	"00075b4424"	0.0067
"00074fae3c"	"00075b4470"	0.0318
"00074fae3c"	"00075b47ed"	0.0641
"00074fae3c"	"00075b4850"	−0.0952
"00074fae3c"	"00075b4ca0"	−0.1568
"00074fae3c"	"00075b4d7f"	−0.1315
"00074fae3c"	"00075b520f"	0.1531
"00074fae3c"	"00075b525f"	−0.0204
"00074fae3c"	"00075b58f8"	−0.2509
"00074fae3c"	"00075b5bcc"	0.045
"00074fae3c"	"00075b5bfa"	−0.1841
"00074fae3c"	"00075b6339"	−0.0612
"00074fae3c"	"00075b6658"	0.0346
"00074fae3c"	"00075b679a"	0.0591
"00074fae3c"	"00075b6cb7"	−0.2381
"00074fae3c"	"00075b6df8"	−0.1415
"00074fae3c"	"00075b6ff6"	−0.4853
"00074fae3c"	"00075b70ee"	−0.2908
"00074fae3c"	"00075b7157"	−0.0341
"00074fae3c"	"00075b7225"	−0.196
"00074fae3c"	"00075b7c89"	−0.2277
"00074fae3c"	"00075b9048"	−0.3318
"00074fae3c"	"00075d0801"	−0.0998
"00074fae3c"	"00075d1820"	0.268
"00074fae3c"	"00075d1f3d"	−0.2075
"00074fae3c"	"00075d2329"	0.1052
"00074fae3c"	"00075d2b9b"	−0.3201
"00074fae3c"	"00075d3941"	0.2945
"00074fae3c"	"00075d3e96"	0.3576
"00074fae3c"	"00075d4864"	−0.0752
"00074fae3c"	"00075d5961"	0.0315
"00074fae3c"	"00075d5a63"	0.0323
"00074fae3c"	"00075d6150"	0.1922
"00074fae3c"	"00075d67d0"	−0.1901
"00074fae3c"	"00075d67e2"	−0.2145
"00074fae3c"	"00075d73fc"	0.0133
"00074fae3c"	"00075d7729"	−0.0693
"00074fae3c"	"00075d778c"	−0.1946
"00074fae3c"	"00075d7b9e"	0.1915
"00074fae3c"	"00075d7c8f"	−0.1395
"00074fae3c"	"00075d804d"	−0.0129
"00074fae3c"	"00075d819f"	−0.0429
"00074fae3c"	"00075d8601"	0.5596
"00074fae3c"	"00075d8c6a"	−0.0847
"00074fae3c"	"00075dfedc"	0.0408
"00074fae3c"	"00075e05f2"	−0.1538
"00074fae3c"	"00075e0837"	0.0886
"00074fae3c"	"00075e092e"	−0.1194
"00074fae3c"	"00075e0965"	−0.442
"00074fae3c"	"00075e0bc8"	−0.1368

"00074fae3c"	"00075e0fbb"	-0.4042
"00074fb0a8"	"00074fb4e4"	0.5692
"00074fb0a8"	"00074fb7c2"	0.1327
"00074fb0a8"	"00074fbd36"	-0.1575
"00074fb0a8"	"00074fc27f"	-0.0154
"00074fb0a8"	"00074fc31d"	0.3646
"00074fb0a8"	"00074fd569"	0.0413
"00074fb0a8"	"00074fef15"	-0.3147
"00074fb0a8"	"00074ff562"	-0.3527
"00074fb0a8"	"00075007ca"	-0.0237
"00074fb0a8"	"0007500b86"	0.1342
"00074fb0a8"	"0007500d05"	-0.0414
"00074fb0a8"	"0007500ee4"	0.1015
"00074fb0a8"	"0007500eee"	-0.2772
"00074fb0a8"	"00075013dc"	0.2334
"00074fb0a8"	"000757b515"	0.1661
"00074fb0a8"	"000757bc5a"	0.2515
"00074fb0a8"	"000757c320"	0.0758
"00074fb0a8"	"000757c9aa"	0.2485
"00074fb0a8"	"000757ccbe"	0.2058
"00074fb0a8"	"000757cfa9"	-0.2229
"00074fb0a8"	"000757d390"	0.5627
"00074fb0a8"	"000757d393"	0.0291
"00074fb0a8"	"000757d598"	-0.1138
"00074fb0a8"	"000757d5a2"	-0.228
"00074fb0a8"	"000757d790"	0.08
"00074fb0a8"	"000757e30c"	0.1231
"00074fb0a8"	"000757e4b0"	0.1537
"00074fb0a8"	"000757e7a0"	0.0758
"00074fb0a8"	"000757e8b3"	0.0764
"00074fb0a8"	"000757f627"	0.3053
"00074fb0a8"	"000757f925"	0.1956
"00074fb0a8"	"000757fa08"	-0.3628
"00074fb0a8"	"000757fe52"	-0.2203
"00074fb0a8"	"000758024a"	0.0223
"00074fb0a8"	"00075804bb"	-0.0397
"00074fb0a8"	"00075a0c04"	-0.0773
"00074fb0a8"	"00075a3110"	-0.0487
"00074fb0a8"	"00075a341a"	-0.3905
"00074fb0a8"	"00075a3dcf"	-0.0498
"00074fb0a8"	"00075a3e22"	0.1709
"00074fb0a8"	"00075a48d8"	-0.1708
"00074fb0a8"	"00075a5cfb"	0.0703
"00074fb0a8"	"00075a6151"	0.0693
"00074fb0a8"	"00075a6708"	-0.1979
"00074fb0a8"	"00075a7319"	0.5915
"00074fb0a8"	"00075a7723"	-0.054
"00074fb0a8"	"00075a778b"	-0.1486
"00074fb0a8"	"00075a7b8e"	0.5401
"00074fb0a8"	"00075a7c79"	0.1088

"00074fb0a8"	"00075a81b6"	−0.3401
"00074fb0a8"	"00075a82ac"	0.1395
"00074fb0a8"	"00075a98e5"	−0.1341
"00074fb0a8"	"00075b0d29"	−0.2109
"00074fb0a8"	"00075b102a"	−0.1126
"00074fb0a8"	"00075b1074"	−0.2908
"00074fb0a8"	"00075b135d"	0.1624
"00074fb0a8"	"00075b138b"	0.0355
"00074fb0a8"	"00075b13a0"	−0.2555
"00074fb0a8"	"00075b13bd"	−0.0204
"00074fb0a8"	"00075b16a9"	0.0845
"00074fb0a8"	"00075b1a28"	−0.2362
"00074fb0a8"	"00075b1a97"	0.0054
"00074fb0a8"	"00075b1c7b"	−0.3536
"00074fb0a8"	"00075b1d24"	0.0287
"00074fb0a8"	"00075b202b"	0.1411
"00074fb0a8"	"00075b22cb"	−0.2021
"00074fb0a8"	"00075b22da"	−0.3855
"00074fb0a8"	"00075b2556"	−0.3014
"00074fb0a8"	"00075b25de"	0.1823
"00074fb0a8"	"00075b260c"	−0.2376
"00074fb0a8"	"00075b26f1"	−0.3115
"00074fb0a8"	"00075b2920"	0.2578
"00074fb0a8"	"00075b2a64"	0.0803
"00074fb0a8"	"00075b2a9d"	0.1298
"00074fb0a8"	"00075b2b37"	−0.2419
"00074fb0a8"	"00075b2cdd"	0.0278
"00074fb0a8"	"00075b3038"	0.044
"00074fb0a8"	"00075b30fe"	−0.2498
"00074fb0a8"	"00075b3362"	−0.1544
"00074fb0a8"	"00075b350a"	−0.2205
"00074fb0a8"	"00075b350e"	0.2114
"00074fb0a8"	"00075b3651"	−0.0995
"00074fb0a8"	"00075b38ca"	−0.1164
"00074fb0a8"	"00075b39cc"	0.1859
"00074fb0a8"	"00075b3e1e"	−0.0685
"00074fb0a8"	"00075b3e57"	−0.2319
"00074fb0a8"	"00075b4079"	0.0088
"00074fb0a8"	"00075b4150"	0.0632
"00074fb0a8"	"00075b4194"	0.3933
"00074fb0a8"	"00075b42d5"	−0.0833
"00074fb0a8"	"00075b4424"	−0.2165
"00074fb0a8"	"00075b4470"	0.2359
"00074fb0a8"	"00075b47ed"	0.2871
"00074fb0a8"	"00075b4850"	0.2935
"00074fb0a8"	"00075b4ca0"	0.0395
"00074fb0a8"	"00075b4d7f"	0.0899
"00074fb0a8"	"00075b520f"	0.1316
"00074fb0a8"	"00075b525f"	−0.1607
"00074fb0a8"	"00075b58f8"	−0.0046

"00074fb0a8"	"00075b5bcc"	0.0288
"00074fb0a8"	"00075b5bfa"	−0.2193
"00074fb0a8"	"00075b6339"	−0.0045
"00074fb0a8"	"00075b6658"	−0.4021
"00074fb0a8"	"00075b679a"	−0.0319
"00074fb0a8"	"00075b6cb7"	−0.1715
"00074fb0a8"	"00075b6df8"	−0.2975
"00074fb0a8"	"00075b6ff6"	−0.0626
"00074fb0a8"	"00075b70ee"	−0.2175
"00074fb0a8"	"00075b7157"	0.1806
"00074fb0a8"	"00075b7225"	−0.0156
"00074fb0a8"	"00075b7c89"	0.017
"00074fb0a8"	"00075b9048"	0.1189
"00074fb0a8"	"00075d0801"	0.2595
"00074fb0a8"	"00075d1820"	0.0338
"00074fb0a8"	"00075d1f3d"	0.0478
"00074fb0a8"	"00075d2329"	−0.0398
"00074fb0a8"	"00075d2b9b"	−0.0056
"00074fb0a8"	"00075d3941"	−0.2204
"00074fb0a8"	"00075d3e96"	0.1385
"00074fb0a8"	"00075d4864"	−0.0583
"00074fb0a8"	"00075d5961"	0.0169
"00074fb0a8"	"00075d5a63"	−0.137
"00074fb0a8"	"00075d6150"	0.2219
"00074fb0a8"	"00075d67d0"	−0.2054
"00074fb0a8"	"00075d67e2"	−0.2388
"00074fb0a8"	"00075d73fc"	−0.2539
"00074fb0a8"	"00075d7729"	−0.2966
"00074fb0a8"	"00075d778c"	−0.4759
"00074fb0a8"	"00075d7b9e"	0.0101
"00074fb0a8"	"00075d7c8f"	−0.0148
"00074fb0a8"	"00075d804d"	0.1409
"00074fb0a8"	"00075d819f"	0.0834
"00074fb0a8"	"00075d8601"	0.0224
"00074fb0a8"	"00075d8c6a"	−0.0107
"00074fb0a8"	"00075dfedc"	0.0758
"00074fb0a8"	"00075e05f2"	−0.0389
"00074fb0a8"	"00075e0837"	−0.0966
"00074fb0a8"	"00075e092e"	−0.2798
"00074fb0a8"	"00075e0965"	−0.1554
"00074fb0a8"	"00075e0bc8"	0.0896
"00074fb0a8"	"00075e0fbb"	−0.274
"00074fb4e4"	"00074fb7c2"	−0.2404
"00074fb4e4"	"00074fbd36"	−0.2418
"00074fb4e4"	"00074fc27f"	−0.2798
"00074fb4e4"	"00074fc31d"	0.2561
"00074fb4e4"	"00074fd569"	0.1216
"00074fb4e4"	"00074fef15"	−0.165
"00074fb4e4"	"00074ff562"	−0.6035
"00074fb4e4"	"00075007ca"	−0.1966

"00074fb4e4"	"0007500b86"	−0.0349
"00074fb4e4"	"0007500d05"	−0.2681
"00074fb4e4"	"0007500ee4"	0.007
"00074fb4e4"	"0007500eee"	−0.3441
"00074fb4e4"	"00075013dc"	0.0522
"00074fb4e4"	"000757b515"	−0.0989
"00074fb4e4"	"000757bc5a"	−0.0724
"00074fb4e4"	"000757c320"	0.3158
"00074fb4e4"	"000757c9aa"	0.2146
"00074fb4e4"	"000757ccbe"	−0.2529
"00074fb4e4"	"000757cfa9"	−0.2382
"00074fb4e4"	"000757d390"	0.5329
"00074fb4e4"	"000757d393"	−0.0731
"00074fb4e4"	"000757d598"	−0.0949
"00074fb4e4"	"000757d5a2"	−0.2774
"00074fb4e4"	"000757d790"	−0.1455
"00074fb4e4"	"000757e30c"	−0.075
"00074fb4e4"	"000757e4b0"	−0.0639
"00074fb4e4"	"000757e7a0"	−0.0217
"00074fb4e4"	"000757e8b3"	−0.2794
"00074fb4e4"	"000757f627"	0.4184
"00074fb4e4"	"000757f925"	−0.0167
"00074fb4e4"	"000757fa08"	−0.6291
"00074fb4e4"	"000757fe52"	−0.3458
"00074fb4e4"	"000758024a"	−0.2874
"00074fb4e4"	"00075804bb"	−0.072
"00074fb4e4"	"00075a0c04"	−0.4587
"00074fb4e4"	"00075a3110"	−0.4351
"00074fb4e4"	"00075a341a"	−0.4482
"00074fb4e4"	"00075a3dcf"	−0.3119
"00074fb4e4"	"00075a3e22"	−0.2681
"00074fb4e4"	"00075a48d8"	−0.4976
"00074fb4e4"	"00075a5cfb"	−0.1918
"00074fb4e4"	"00075a6151"	−0.0317
"00074fb4e4"	"00075a6708"	−0.1708
"00074fb4e4"	"00075a7319"	0.5677
"00074fb4e4"	"00075a7723"	−0.1543
"00074fb4e4"	"00075a778b"	−0.4458
"00074fb4e4"	"00075a7b8e"	0.04
"00074fb4e4"	"00075a7c79"	−0.1995
"00074fb4e4"	"00075a81b6"	−0.5066
"00074fb4e4"	"00075a82ac"	0.0271
"00074fb4e4"	"00075a98e5"	−0.1096
"00074fb4e4"	"00075b0d29"	−0.1513
"00074fb4e4"	"00075b102a"	−0.2863
"00074fb4e4"	"00075b1074"	−0.5869
"00074fb4e4"	"00075b135d"	−0.2127
"00074fb4e4"	"00075b138b"	−0.24
"00074fb4e4"	"00075b13a0"	−0.4609
"00074fb4e4"	"00075b13bd"	−0.0042

"00074fb4e4"	"00075b16a9"	-0.0938
"00074fb4e4"	"00075b1a28"	-0.2218
"00074fb4e4"	"00075b1a97"	-0.1291
"00074fb4e4"	"00075b1c7b"	-0.2761
"00074fb4e4"	"00075b1d24"	-0.2724
"00074fb4e4"	"00075b202b"	-0.2773
"00074fb4e4"	"00075b22cb"	-0.2324
"00074fb4e4"	"00075b22da"	-0.535
"00074fb4e4"	"00075b2556"	-0.3367
"00074fb4e4"	"00075b25de"	0.1286
"00074fb4e4"	"00075b260c"	-0.2681
"00074fb4e4"	"00075b26f1"	-0.326
"00074fb4e4"	"00075b2920"	0.2107
"00074fb4e4"	"00075b2a64"	-0.2962
"00074fb4e4"	"00075b2a9d"	-0.2183
"00074fb4e4"	"00075b2b37"	-0.3245
"00074fb4e4"	"00075b2cdd"	-0.2883
"00074fb4e4"	"00075b3038"	-0.0453
"00074fb4e4"	"00075b30fe"	-0.3892
"00074fb4e4"	"00075b3362"	-0.2309
"00074fb4e4"	"00075b350a"	-0.2836
"00074fb4e4"	"00075b350e"	0.0415
"00074fb4e4"	"00075b3651"	-0.0898
"00074fb4e4"	"00075b38ca"	-0.2534
"00074fb4e4"	"00075b39cc"	-0.0445
"00074fb4e4"	"00075b3e1e"	-0.1508
"00074fb4e4"	"00075b3e57"	-0.1758
"00074fb4e4"	"00075b4079"	-0.0124
"00074fb4e4"	"00075b4150"	-0.2372
"00074fb4e4"	"00075b4194"	0.0765
"00074fb4e4"	"00075b42d5"	-0.3057
"00074fb4e4"	"00075b4424"	-0.2214
"00074fb4e4"	"00075b4470"	-0.0724
"00074fb4e4"	"00075b47ed"	-0.0137
"00074fb4e4"	"00075b4850"	0.0256
"00074fb4e4"	"00075b4ca0"	-0.3049
"00074fb4e4"	"00075b4d7f"	-0.275
"00074fb4e4"	"00075b520f"	-0.0025
"00074fb4e4"	"00075b525f"	-0.2051
"00074fb4e4"	"00075b58f8"	-0.2492
"00074fb4e4"	"00075b5bcc"	-0.0496
"00074fb4e4"	"00075b5bfa"	-0.2924
"00074fb4e4"	"00075b6339"	-0.2724
"00074fb4e4"	"00075b6658"	-0.3433
"00074fb4e4"	"00075b679a"	0.0101
"00074fb4e4"	"00075b6cb7"	-0.398
"00074fb4e4"	"00075b6df8"	-0.3382
"00074fb4e4"	"00075b6ff6"	-0.3401
"00074fb4e4"	"00075b70ee"	-0.2452
"00074fb4e4"	"00075b7157"	-0.1802

"00074fb4e4"	"00075b7225"	−0.2703
"00074fb4e4"	"00075b7c89"	−0.2082
"00074fb4e4"	"00075b9048"	−0.0263
"00074fb4e4"	"00075d0801"	0.0705
"00074fb4e4"	"00075d1820"	0.0929
"00074fb4e4"	"00075d1f3d"	−0.2459
"00074fb4e4"	"00075d2329"	−0.3534
"00074fb4e4"	"00075d2b9b"	−0.1918
"00074fb4e4"	"00075d3941"	−0.3938
"00074fb4e4"	"00075d3e96"	0.1246
"00074fb4e4"	"00075d4864"	−0.2913
"00074fb4e4"	"00075d5961"	−0.1351
"00074fb4e4"	"00075d5a63"	−0.2458
"00074fb4e4"	"00075d6150"	0.0138
"00074fb4e4"	"00075d67d0"	−0.2906
"00074fb4e4"	"00075d67e2"	−0.3915
"00074fb4e4"	"00075d73fc"	−0.2374
"00074fb4e4"	"00075d7729"	−0.3217
"00074fb4e4"	"00075d778c"	−0.4585
"00074fb4e4"	"00075d7b9e"	−0.2256
"00074fb4e4"	"00075d7c8f"	−0.3282
"00074fb4e4"	"00075d804d"	−0.1354
"00074fb4e4"	"00075d819f"	−0.1834
"00074fb4e4"	"00075d8601"	0.2219
"00074fb4e4"	"00075d8c6a"	−0.0473
"00074fb4e4"	"00075dfedc"	−0.0894
"00074fb4e4"	"00075e05f2"	−0.2483
"00074fb4e4"	"00075e0837"	−0.2819
"00074fb4e4"	"00075e092e"	−0.4463
"00074fb4e4"	"00075e0965"	−0.3393
"00074fb4e4"	"00075e0bc8"	−0.2054
"00074fb4e4"	"00075e0fbb"	−0.5804
"00074fb7c2"	"00074fbd36"	0.0086
"00074fb7c2"	"00074fc27f"	0.0164
"00074fb7c2"	"00074fc31d"	0.4031
"00074fb7c2"	"00074fd569"	0.326
"00074fb7c2"	"00074fef15"	0.0965
"00074fb7c2"	"00074ff562"	−0.1207
"00074fb7c2"	"00075007ca"	−0.1037
"00074fb7c2"	"0007500b86"	0.0894
"00074fb7c2"	"0007500d05"	0.1114
"00074fb7c2"	"0007500ee4"	0.1069
"00074fb7c2"	"0007500eee"	−0.0496
"00074fb7c2"	"00075013dc"	−0.0992
"00074fb7c2"	"000757b515"	0.1671
"00074fb7c2"	"000757bc5a"	0.2365
"00074fb7c2"	"000757c320"	−0.1942
"00074fb7c2"	"000757c9aa"	0.0115
"00074fb7c2"	"000757ccbe"	0.2725
"00074fb7c2"	"000757cfa9"	0.0471

"00074fb7c2"	"000757d390"	−0.1574
"00074fb7c2"	"000757d393"	0.2488
"00074fb7c2"	"000757d598"	−0.101
"00074fb7c2"	"000757d5a2"	−0.053
"00074fb7c2"	"000757d790"	0.2733
"00074fb7c2"	"000757e30c"	−0.0801
"00074fb7c2"	"000757e4b0"	0.3516
"00074fb7c2"	"000757e7a0"	−0.0631
"00074fb7c2"	"000757e8b3"	0.2719
"00074fb7c2"	"000757f627"	−0.0347
"00074fb7c2"	"000757f925"	0.1691
"00074fb7c2"	"000757fa08"	0.3829
"00074fb7c2"	"000757fe52"	0.1143
"00074fb7c2"	"000758024a"	−0.0481
"00074fb7c2"	"00075804bb"	−0.0871
"00074fb7c2"	"00075a0c04"	0.5329
"00074fb7c2"	"00075a3110"	0.291
"00074fb7c2"	"00075a341a"	0.3803
"00074fb7c2"	"00075a3dcf"	0.1745
"00074fb7c2"	"00075a3e22"	0.398
"00074fb7c2"	"00075a48d8"	0.2752
"00074fb7c2"	"00075a5cfb"	−0.1538
"00074fb7c2"	"00075a6151"	−0.178
"00074fb7c2"	"00075a6708"	−0.0765
"00074fb7c2"	"00075a7319"	−0.1558
"00074fb7c2"	"00075a7723"	0.4358
"00074fb7c2"	"00075a778b"	0.4551
"00074fb7c2"	"00075a7b8e"	0.2009
"00074fb7c2"	"00075a7c79"	0.2608
"00074fb7c2"	"00075a81b6"	0.1114
"00074fb7c2"	"00075a82ac"	0.4495
"00074fb7c2"	"00075a98e5"	0.1587
"00074fb7c2"	"00075b0d29"	0.1746
"00074fb7c2"	"00075b102a"	0.136
"00074fb7c2"	"00075b1074"	0.2159
"00074fb7c2"	"00075b135d"	0.3491
"00074fb7c2"	"00075b138b"	0.3576
"00074fb7c2"	"00075b13a0"	−0.0673
"00074fb7c2"	"00075b13bd"	0.1054
"00074fb7c2"	"00075b16a9"	0.1108
"00074fb7c2"	"00075b1a28"	0.0207
"00074fb7c2"	"00075b1a97"	0.2132
"00074fb7c2"	"00075b1c7b"	−0.0765
"00074fb7c2"	"00075b1d24"	0.1712
"00074fb7c2"	"00075b202b"	0.15
"00074fb7c2"	"00075b22cb"	0.1768
"00074fb7c2"	"00075b22da"	0.0542
"00074fb7c2"	"00075b2556"	0.1621
"00074fb7c2"	"00075b25de"	0.1018
"00074fb7c2"	"00075b260c"	−0.0312

"00074fb7c2"	"00075b26f1"	0.0896
"00074fb7c2"	"00075b2920"	-0.0073
"00074fb7c2"	"00075b2a64"	-0.0871
"00074fb7c2"	"00075b2a9d"	0.0255
"00074fb7c2"	"00075b2b37"	-0.2654
"00074fb7c2"	"00075b2cdd"	0.3024
"00074fb7c2"	"00075b3038"	0.2396
"00074fb7c2"	"00075b30fe"	0.1508
"00074fb7c2"	"00075b3362"	0.0885
"00074fb7c2"	"00075b350a"	-0.0875
"00074fb7c2"	"00075b350e"	0.1787
"00074fb7c2"	"00075b3651"	0.0468
"00074fb7c2"	"00075b38ca"	0.0697
"00074fb7c2"	"00075b39cc"	0.0542
"00074fb7c2"	"00075b3e1e"	0.2513
"00074fb7c2"	"00075b3e57"	0.1025
"00074fb7c2"	"00075b4079"	-0.1377
"00074fb7c2"	"00075b4150"	0.0594
"00074fb7c2"	"00075b4194"	0.1054
"00074fb7c2"	"00075b42d5"	0.2519
"00074fb7c2"	"00075b4424"	0.1557
"00074fb7c2"	"00075b4470"	0.3059
"00074fb7c2"	"00075b47ed"	0.215
"00074fb7c2"	"00075b4850"	0.1881
"00074fb7c2"	"00075b4ca0"	0.1203
"00074fb7c2"	"00075b4d7f"	0.4242
"00074fb7c2"	"00075b520f"	0.0076
"00074fb7c2"	"00075b525f"	0.1272
"00074fb7c2"	"00075b58f8"	-0.1897
"00074fb7c2"	"00075b5bcc"	0.0136
"00074fb7c2"	"00075b5bfa"	-0.1869
"00074fb7c2"	"00075b6339"	0.0965
"00074fb7c2"	"00075b6658"	0.0863
"00074fb7c2"	"00075b679a"	0.0812
"00074fb7c2"	"00075b6cb7"	0.0529
"00074fb7c2"	"00075b6df8"	0.365
"00074fb7c2"	"00075b6ff6"	0.0891
"00074fb7c2"	"00075b70ee"	0.1095
"00074fb7c2"	"00075b7157"	0.0601
"00074fb7c2"	"00075b7225"	0.2241
"00074fb7c2"	"00075b7c89"	0.2806
"00074fb7c2"	"00075b9048"	0.3049
"00074fb7c2"	"00075d0801"	0.1493
"00074fb7c2"	"00075d1820"	0.0533
"00074fb7c2"	"00075d1f3d"	0.0967
"00074fb7c2"	"00075d2329"	0.257
"00074fb7c2"	"00075d2b9b"	0.1114
"00074fb7c2"	"00075d3941"	0.2
"00074fb7c2"	"00075d3e96"	0.204
"00074fb7c2"	"00075d4864"	0.1176

"00074fb7c2"	"00075d5961"	0.3108
"00074fb7c2"	"00075d5a63"	0.1443
"00074fb7c2"	"00075d6150"	0.0537
"00074fb7c2"	"00075d67d0"	-0.0443
"00074fb7c2"	"00075d67e2"	0.2141
"00074fb7c2"	"00075d73fc"	0.299
"00074fb7c2"	"00075d7729"	0.0025
"00074fb7c2"	"00075d778c"	-0.033
"00074fb7c2"	"00075d7b9e"	0.3271
"00074fb7c2"	"00075d7c8f"	0.2541
"00074fb7c2"	"00075d804d"	-0.054
"00074fb7c2"	"00075d819f"	0.2222
"00074fb7c2"	"00075d8601"	0.0303
"00074fb7c2"	"00075d8c6a"	0.1919
"00074fb7c2"	"00075dfedc"	0.2063
"00074fb7c2"	"00075e05f2"	0.0062
"00074fb7c2"	"00075e0837"	0.1169
"00074fb7c2"	"00075e092e"	0.1493
"00074fb7c2"	"00075e0965"	-0.1113
"00074fb7c2"	"00075e0bc8"	0.2539
"00074fb7c2"	"00075e0fbb"	0.0819
"00074fbd36"	"00074fc27f"	0.0706
"00074fbd36"	"00074fc31d"	0.0979
"00074fbd36"	"00074fd569"	-0.4244
"00074fbd36"	"00074fef15"	0.0513
"00074fbd36"	"00074ff562"	-0.3793
"00074fbd36"	"00075007ca"	-0.4274
"00074fbd36"	"0007500b86"	0.1545
"00074fbd36"	"0007500d05"	-0.5055
"00074fbd36"	"0007500ee4"	-0.1883
"00074fbd36"	"0007500eee"	-0.2257
"00074fbd36"	"00075013dc"	-0.241
"00074fbd36"	"000757b515"	-0.0071
"00074fbd36"	"000757bc5a"	0.0707
"00074fbd36"	"000757c320"	-0.0429
"00074fbd36"	"000757c9aa"	7e-04
"00074fbd36"	"000757ccbe"	-0.0056
"00074fbd36"	"000757cfa9"	0.2876
"00074fbd36"	"000757d390"	-0.4265
"00074fbd36"	"000757d393"	-0.0128
"00074fbd36"	"000757d598"	-0.1625
"00074fbd36"	"000757d5a2"	-0.2503
"00074fbd36"	"000757d790"	-0.1667
"00074fbd36"	"000757e30c"	-0.113
"00074fbd36"	"000757e4b0"	0.0304
"00074fbd36"	"000757e7a0"	-0.2563
"00074fbd36"	"000757e8b3"	0.1461
"00074fbd36"	"000757f627"	0.1165
"00074fbd36"	"000757f925"	-0.0743
"00074fbd36"	"000757fa08"	-0.1679

"00074fbd36"	"000757fe52"	-0.0952
"00074fbd36"	"000758024a"	-0.0628
"00074fbd36"	"00075804bb"	0.0643
"00074fbd36"	"00075a0c04"	-0.3331
"00074fbd36"	"00075a3110"	-0.1292
"00074fbd36"	"00075a341a"	0.0175
"00074fbd36"	"00075a3dcf"	-0.0016
"00074fbd36"	"00075a3e22"	-0.0108
"00074fbd36"	"00075a48d8"	-0.2232
"00074fbd36"	"00075a5cfb"	-0.1007
"00074fbd36"	"00075a6151"	0.0732
"00074fbd36"	"00075a6708"	0.0372
"00074fbd36"	"00075a7319"	-0.4884
"00074fbd36"	"00075a7723"	-0.1104
"00074fbd36"	"00075a778b"	-0.0981
"00074fbd36"	"00075a7b8e"	-0.2571
"00074fbd36"	"00075a7c79"	-0.0983
"00074fbd36"	"00075a81b6"	-0.1687
"00074fbd36"	"00075a82ac"	0.0594
"00074fbd36"	"00075a98e5"	0.0458
"00074fbd36"	"00075b0d29"	0.4781
"00074fbd36"	"00075b102a"	-0.0058
"00074fbd36"	"00075b1074"	-0.1935
"00074fbd36"	"00075b135d"	0.2128
"00074fbd36"	"00075b138b"	-0.0594
"00074fbd36"	"00075b13a0"	-0.0733
"00074fbd36"	"00075b13bd"	0.1974
"00074fbd36"	"00075b16a9"	-0.1294
"00074fbd36"	"00075b1a28"	0.0684
"00074fbd36"	"00075b1a97"	0.0326
"00074fbd36"	"00075b1c7b"	-0.4725
"00074fbd36"	"00075b1d24"	-0.0459
"00074fbd36"	"00075b202b"	-0.2662
"00074fbd36"	"00075b22cb"	-0.0705
"00074fbd36"	"00075b22da"	-0.0091
"00074fbd36"	"00075b2556"	0.1454
"00074fbd36"	"00075b25de"	-0.1007
"00074fbd36"	"00075b260c"	-0.1332
"00074fbd36"	"00075b26f1"	-0.2158
"00074fbd36"	"00075b2920"	-0.2204
"00074fbd36"	"00075b2a64"	-0.1679
"00074fbd36"	"00075b2a9d"	-0.261
"00074fbd36"	"00075b2b37"	-0.3649
"00074fbd36"	"00075b2cdd"	-0.3773
"00074fbd36"	"00075b3038"	-0.2211
"00074fbd36"	"00075b30fe"	-0.0475
"00074fbd36"	"00075b3362"	-0.2104
"00074fbd36"	"00075b350a"	0.0646
"00074fbd36"	"00075b350e"	0.0102
"00074fbd36"	"00075b3651"	-0.1321

"00074fbd36"	"00075b38ca"	−0.1274
"00074fbd36"	"00075b39cc"	−0.1611
"00074fbd36"	"00075b3e1e"	−0.0544
"00074fbd36"	"00075b3e57"	−0.1937
"00074fbd36"	"00075b4079"	−0.0905
"00074fbd36"	"00075b4150"	−0.0112
"00074fbd36"	"00075b4194"	−0.1614
"00074fbd36"	"00075b42d5"	−0.0742
"00074fbd36"	"00075b4424"	−0.0559
"00074fbd36"	"00075b4470"	0.1401
"00074fbd36"	"00075b47ed"	−0.3092
"00074fbd36"	"00075b4850"	0.0096
"00074fbd36"	"00075b4ca0"	0.0306
"00074fbd36"	"00075b4d7f"	0.1084
"00074fbd36"	"00075b520f"	0.1665
"00074fbd36"	"00075b525f"	−0.2044
"00074fbd36"	"00075b58f8"	0.0916
"00074fbd36"	"00075b5bcc"	−0.0582
"00074fbd36"	"00075b5bfa"	−0.4922
"00074fbd36"	"00075b6339"	0.0853
"00074fbd36"	"00075b6658"	−0.0335
"00074fbd36"	"00075b679a"	−0.0163
"00074fbd36"	"00075b6cb7"	0.0097
"00074fbd36"	"00075b6df8"	−0.1619
"00074fbd36"	"00075b6ff6"	−0.1403
"00074fbd36"	"00075b70ee"	−0.1268
"00074fbd36"	"00075b7157"	−0.1305
"00074fbd36"	"00075b7225"	−0.0624
"00074fbd36"	"00075b7c89"	0.1421
"00074fbd36"	"00075b9048"	−0.0954
"00074fbd36"	"00075d0801"	−0.0506
"00074fbd36"	"00075d1820"	−0.0441
"00074fbd36"	"00075d1f3d"	−0.2432
"00074fbd36"	"00075d2329"	−0.0768
"00074fbd36"	"00075d2b9b"	−0.0847
"00074fbd36"	"00075d3941"	−0.3631
"00074fbd36"	"00075d3e96"	−0.3193
"00074fbd36"	"00075d4864"	0.1152
"00074fbd36"	"00075d5961"	0.0488
"00074fbd36"	"00075d5a63"	−0.2468
"00074fbd36"	"00075d6150"	−0.2981
"00074fbd36"	"00075d67d0"	−0.5415
"00074fbd36"	"00075d67e2"	0.1093
"00074fbd36"	"00075d73fc"	−0.0114
"00074fbd36"	"00075d7729"	−0.0671
"00074fbd36"	"00075d778c"	−0.367
"00074fbd36"	"00075d7b9e"	−0.1585
"00074fbd36"	"00075d7c8f"	−0.055
"00074fbd36"	"00075d804d"	−0.277
"00074fbd36"	"00075d819f"	−0.1423

"00074fbd36"	"00075d8601"	−0.1559
"00074fbd36"	"00075d8c6a"	−0.0728
"00074fbd36"	"00075dfedc"	0.112
"00074fbd36"	"00075e05f2"	−0.1309
"00074fbd36"	"00075e0837"	−0.2396
"00074fbd36"	"00075e092e"	−0.1818
"00074fbd36"	"00075e0965"	−0.2398
"00074fbd36"	"00075e0bc8"	0.0744
"00074fbd36"	"00075e0fbb"	−0.1092
"00074fc27f"	"00074fc31d"	−0.0947
"00074fc27f"	"00074fd569"	−0.112
"00074fc27f"	"00074fef15"	−0.0921
"00074fc27f"	"00074ff562"	−0.2767
"00074fc27f"	"00075007ca"	0.1281
"00074fc27f"	"0007500b86"	0.1576
"00074fc27f"	"0007500d05"	0.0874
"00074fc27f"	"0007500ee4"	−0.0565
"00074fc27f"	"0007500eee"	−0.0361
"00074fc27f"	"00075013dc"	−0.1675
"00074fc27f"	"000757b515"	0.3157
"00074fc27f"	"000757bc5a"	0.0184
"00074fc27f"	"000757c320"	0.0423
"00074fc27f"	"000757c9aa"	0.2628
"00074fc27f"	"000757ccbe"	0.0981
"00074fc27f"	"000757cfa9"	−0.1001
"00074fc27f"	"000757d390"	−0.1529
"00074fc27f"	"000757d393"	0.0935
"00074fc27f"	"000757d598"	0.3457
"00074fc27f"	"000757d5a2"	0.2005
"00074fc27f"	"000757d790"	0.1544
"00074fc27f"	"000757e30c"	0.1458
"00074fc27f"	"000757e4b0"	0.2565
"00074fc27f"	"000757e7a0"	0.023
"00074fc27f"	"000757e8b3"	0.2318
"00074fc27f"	"000757f627"	0.0316
"00074fc27f"	"000757f925"	0.1829
"00074fc27f"	"000757fa08"	−0.2177
"00074fc27f"	"000757fe52"	0.2144
"00074fc27f"	"000758024a"	0.126
"00074fc27f"	"00075804bb"	0.0927
"00074fc27f"	"00075a0c04"	−0.0847
"00074fc27f"	"00075a3110"	0.2038
"00074fc27f"	"00075a341a"	0.0925
"00074fc27f"	"00075a3dcf"	−0.0995
"00074fc27f"	"00075a3e22"	0.1641
"00074fc27f"	"00075a48d8"	−0.0484
"00074fc27f"	"00075a5cfb"	0.3737
"00074fc27f"	"00075a6151"	0.1486
"00074fc27f"	"00075a6708"	0.0536
"00074fc27f"	"00075a7319"	−0.2216

"00074fc27f"	"00075a7723"	-0.0173
"00074fc27f"	"00075a778b"	-0.0319
"00074fc27f"	"00075a7b8e"	0.0635
"00074fc27f"	"00075a7c79"	0.2614
"00074fc27f"	"00075a81b6"	-0.1517
"00074fc27f"	"00075a82ac"	0.1826
"00074fc27f"	"00075a98e5"	-0.1657
"00074fc27f"	"00075b0d29"	-0.1264
"00074fc27f"	"00075b102a"	0.0247
"00074fc27f"	"00075b1074"	-0.1535
"00074fc27f"	"00075b135d"	-0.2128
"00074fc27f"	"00075b138b"	0.3675
"00074fc27f"	"00075b13a0"	0.0417
"00074fc27f"	"00075b13bd"	-0.2064
"00074fc27f"	"00075b16a9"	0.0504
"00074fc27f"	"00075b1a28"	-0.2215
"00074fc27f"	"00075b1a97"	-0.0279
"00074fc27f"	"00075b1c7b"	-0.2081
"00074fc27f"	"00075b1d24"	-0.0635
"00074fc27f"	"00075b202b"	0.1783
"00074fc27f"	"00075b22cb"	-0.0523
"00074fc27f"	"00075b22da"	0.1327
"00074fc27f"	"00075b2556"	0.0971
"00074fc27f"	"00075b25de"	-0.036
"00074fc27f"	"00075b260c"	0.4111
"00074fc27f"	"00075b26f1"	-0.141
"00074fc27f"	"00075b2920"	0.3058
"00074fc27f"	"00075b2a64"	0.4374
"00074fc27f"	"00075b2a9d"	-0.1211
"00074fc27f"	"00075b2b37"	-0.2097
"00074fc27f"	"00075b2cdd"	-0.2082
"00074fc27f"	"00075b3038"	0.1016
"00074fc27f"	"00075b30fe"	0.0667
"00074fc27f"	"00075b3362"	-0.3054
"00074fc27f"	"00075b350a"	-0.0298
"00074fc27f"	"00075b350e"	-0.0423
"00074fc27f"	"00075b3651"	-0.1673
"00074fc27f"	"00075b38ca"	-0.1698
"00074fc27f"	"00075b39cc"	-0.1422
"00074fc27f"	"00075b3e1e"	0.0082
"00074fc27f"	"00075b3e57"	0.0536
"00074fc27f"	"00075b4079"	-0.1338
"00074fc27f"	"00075b4150"	0.3611
"00074fc27f"	"00075b4194"	0.0949
"00074fc27f"	"00075b42d5"	0.0404
"00074fc27f"	"00075b4424"	0.1746
"00074fc27f"	"00075b4470"	0.0028
"00074fc27f"	"00075b47ed"	-0.3653
"00074fc27f"	"00075b4850"	0.1853
"00074fc27f"	"00075b4ca0"	-0.0258

"00074fc27f"	"00075b4d7f"	0.1478
"00074fc27f"	"00075b520f"	-0.1343
"00074fc27f"	"00075b525f"	-0.1407
"00074fc27f"	"00075b58f8"	0.1789
"00074fc27f"	"00075b5bcc"	0.099
"00074fc27f"	"00075b5bfa"	0.03
"00074fc27f"	"00075b6339"	0.2552
"00074fc27f"	"00075b6658"	-0.2854
"00074fc27f"	"00075b679a"	0.045
"00074fc27f"	"00075b6cb7"	0.2113
"00074fc27f"	"00075b6df8"	-0.0317
"00074fc27f"	"00075b6ff6"	0.5466
"00074fc27f"	"00075b70ee"	-0.0446
"00074fc27f"	"00075b7157"	0.0017
"00074fc27f"	"00075b7225"	0.0024
"00074fc27f"	"00075b7c89"	-0.0231
"00074fc27f"	"00075b9048"	0.5029
"00074fc27f"	"00075d0801"	-0.1578
"00074fc27f"	"00075d1820"	-0.3063
"00074fc27f"	"00075d1f3d"	0.2375
"00074fc27f"	"00075d2329"	-0.1358
"00074fc27f"	"00075d2b9b"	0.2628
"00074fc27f"	"00075d3941"	-0.3627
"00074fc27f"	"00075d3e96"	-0.0261
"00074fc27f"	"00075d4864"	-0.1765
"00074fc27f"	"00075d5961"	0.115
"00074fc27f"	"00075d5a63"	0.055
"00074fc27f"	"00075d6150"	-0.2054
"00074fc27f"	"00075d67d0"	-0.1071
"00074fc27f"	"00075d67e2"	0.1228
"00074fc27f"	"00075d73fc"	-0.1694
"00074fc27f"	"00075d7729"	-0.0877
"00074fc27f"	"00075d778c"	-0.2323
"00074fc27f"	"00075d7b9e"	-0.1611
"00074fc27f"	"00075d7c8f"	0.091
"00074fc27f"	"00075d804d"	0.3019
"00074fc27f"	"00075d819f"	-0.0922
"00074fc27f"	"00075d8601"	-0.2495
"00074fc27f"	"00075d8c6a"	-0.0107
"00074fc27f"	"00075dfedc"	0.06
"00074fc27f"	"00075e05f2"	0.3887
"00074fc27f"	"00075e0837"	0.068
"00074fc27f"	"00075e092e"	-0.0889
"00074fc27f"	"00075e0965"	0.1913
"00074fc27f"	"00075e0bc8"	0.3512
"00074fc27f"	"00075e0fbb"	0.0744
"00074fc31d"	"00074fd569"	0.1033
"00074fc31d"	"00074fef15"	0.0564
"00074fc31d"	"00074ff562"	-0.4328
"00074fc31d"	"00075007ca"	-0.1814

"00074fc31d"	"0007500b86"	-0.016
"00074fc31d"	"0007500d05"	-0.2784
"00074fc31d"	"0007500ee4"	0.2977
"00074fc31d"	"0007500eee"	-0.2063
"00074fc31d"	"00075013dc"	-0.1236
"00074fc31d"	"000757b515"	0.1161
"00074fc31d"	"000757bc5a"	0.1035
"00074fc31d"	"000757c320"	-0.1187
"00074fc31d"	"000757c9aa"	0.0955
"00074fc31d"	"000757ccbe"	0.1405
"00074fc31d"	"000757cfa9"	0.0732
"00074fc31d"	"000757d390"	0.0412
"00074fc31d"	"000757d393"	0.123
"00074fc31d"	"000757d598"	-0.2233
"00074fc31d"	"000757d5a2"	-0.1099
"00074fc31d"	"000757d790"	-0.0231
"00074fc31d"	"000757e30c"	-0.1056
"00074fc31d"	"000757e4b0"	0.1166
"00074fc31d"	"000757e7a0"	0.0296
"00074fc31d"	"000757e8b3"	7e-04
"00074fc31d"	"000757f627"	0.0248
"00074fc31d"	"000757f925"	0.0749
"00074fc31d"	"000757fa08"	-0.2363
"00074fc31d"	"000757fe52"	-0.3151
"00074fc31d"	"000758024a"	-0.1713
"00074fc31d"	"00075804bb"	-0.1899
"00074fc31d"	"00075a0c04"	-0.0564
"00074fc31d"	"00075a3110"	-0.2633
"00074fc31d"	"00075a341a"	0.0102
"00074fc31d"	"00075a3dcf"	-0.1418
"00074fc31d"	"00075a3e22"	-0.0676
"00074fc31d"	"00075a48d8"	-0.2012
"00074fc31d"	"00075a5cfb"	-0.3448
"00074fc31d"	"00075a6151"	-0.0493
"00074fc31d"	"00075a6708"	0.0708
"00074fc31d"	"00075a7319"	0.1441
"00074fc31d"	"00075a7723"	0.2603
"00074fc31d"	"00075a778b"	-0.1215
"00074fc31d"	"00075a7b8e"	0.2195
"00074fc31d"	"00075a7c79"	-0.0141
"00074fc31d"	"00075a81b6"	-0.1866
"00074fc31d"	"00075a82ac"	0.2685
"00074fc31d"	"00075a98e5"	0.0263
"00074fc31d"	"00075b0d29"	0.0522
"00074fc31d"	"00075b102a"	-0.1119
"00074fc31d"	"00075b1074"	-0.184
"00074fc31d"	"00075b135d"	0.2344
"00074fc31d"	"00075b138b"	0.0104
"00074fc31d"	"00075b13a0"	-0.427
"00074fc31d"	"00075b13bd"	0.0522

"00074fc31d"	"00075b16a9"	0.0438
"00074fc31d"	"00075b1a28"	0.1277
"00074fc31d"	"00075b1a97"	0.0638
"00074fc31d"	"00075b1c7b"	−0.0268
"00074fc31d"	"00075b1d24"	−0.0224
"00074fc31d"	"00075b202b"	−0.1545
"00074fc31d"	"00075b22cb"	0.0128
"00074fc31d"	"00075b22da"	−0.2537
"00074fc31d"	"00075b2556"	−0.0336
"00074fc31d"	"00075b25de"	−0.0475
"00074fc31d"	"00075b260c"	−0.3567
"00074fc31d"	"00075b26f1"	−0.0613
"00074fc31d"	"00075b2920"	−0.0643
"00074fc31d"	"00075b2a64"	−0.2536
"00074fc31d"	"00075b2a9d"	−0.0408
"00074fc31d"	"00075b2b37"	−0.1464
"00074fc31d"	"00075b2cdd"	−0.0238
"00074fc31d"	"00075b3038"	−0.0083
"00074fc31d"	"00075b30fe"	−0.1814
"00074fc31d"	"00075b3362"	−0.0692
"00074fc31d"	"00075b350a"	−0.1681
"00074fc31d"	"00075b350e"	0.0918
"00074fc31d"	"00075b3651"	0.031
"00074fc31d"	"00075b38ca"	−0.1606
"00074fc31d"	"00075b39cc"	−0.0485
"00074fc31d"	"00075b3e1e"	−0.0805
"00074fc31d"	"00075b3e57"	−0.1539
"00074fc31d"	"00075b4079"	−0.0875
"00074fc31d"	"00075b4150"	−0.1325
"00074fc31d"	"00075b4194"	−0.103
"00074fc31d"	"00075b42d5"	−0.1356
"00074fc31d"	"00075b4424"	−0.0035
"00074fc31d"	"00075b4470"	0.1035
"00074fc31d"	"00075b47ed"	−0.0297
"00074fc31d"	"00075b4850"	0.1488
"00074fc31d"	"00075b4ca0"	−0.0447
"00074fc31d"	"00075b4d7f"	0.0043
"00074fc31d"	"00075b520f"	0.3331
"00074fc31d"	"00075b525f"	−0.1094
"00074fc31d"	"00075b58f8"	−0.1142
"00074fc31d"	"00075b5bcc"	−0.0659
"00074fc31d"	"00075b5bfa"	−0.4072
"00074fc31d"	"00075b6339"	−0.1117
"00074fc31d"	"00075b6658"	−0.0517
"00074fc31d"	"00075b679a"	0.1755
"00074fc31d"	"00075b6cb7"	−0.0126
"00074fc31d"	"00075b6df8"	−0.0055
"00074fc31d"	"00075b6ff6"	−0.2167
"00074fc31d"	"00075b70ee"	−0.1635
"00074fc31d"	"00075b7157"	−0.0195

"00074fc31d"	"00075b7225"	0.1386
"00074fc31d"	"00075b7c89"	0.023
"00074fc31d"	"00075b9048"	0.111
"00074fc31d"	"00075d0801"	0.1169
"00074fc31d"	"00075d1820"	0.0661
"00074fc31d"	"00075d1f3d"	-0.0232
"00074fc31d"	"00075d2329"	-0.1229
"00074fc31d"	"00075d2b9b"	-0.3255
"00074fc31d"	"00075d3941"	-0.0289
"00074fc31d"	"00075d3e96"	0.2379
"00074fc31d"	"00075d4864"	-0.1014
"00074fc31d"	"00075d5961"	0.1673
"00074fc31d"	"00075d5a63"	-0.4192
"00074fc31d"	"00075d6150"	0.2405
"00074fc31d"	"00075d67d0"	-0.1302
"00074fc31d"	"00075d67e2"	-8e-04
"00074fc31d"	"00075d73fc"	0.1397
"00074fc31d"	"00075d7729"	-0.082
"00074fc31d"	"00075d778c"	-0.3491
"00074fc31d"	"00075d7b9e"	0.102
"00074fc31d"	"00075d7c8f"	0.0135
"00074fc31d"	"00075d804d"	-0.3044
"00074fc31d"	"00075d819f"	0.0945
"00074fc31d"	"00075d8601"	-0.0561
"00074fc31d"	"00075d8c6a"	-0.0083
"00074fc31d"	"00075dfedc"	-0.0438
"00074fc31d"	"00075e05f2"	-0.099
"00074fc31d"	"00075e0837"	-0.1576
"00074fc31d"	"00075e092e"	-0.054
"00074fc31d"	"00075e0965"	-0.2926
"00074fc31d"	"00075e0bc8"	0.0557
"00074fc31d"	"00075e0fbb"	-0.2542
"00074fd569"	"00074fef15"	0.0689
"00074fd569"	"00074ff562"	-0.2626
"00074fd569"	"00075007ca"	-0.233
"00074fd569"	"0007500b86"	-0.1006
"00074fd569"	"0007500d05"	0.178
"00074fd569"	"0007500ee4"	0.1636
"00074fd569"	"0007500eee"	-0.2314
"00074fd569"	"00075013dc"	-0.2798
"00074fd569"	"000757b515"	-0.0809
"00074fd569"	"000757bc5a"	-0.1251
"00074fd569"	"000757c320"	-0.1977
"00074fd569"	"000757c9aa"	-0.0146
"00074fd569"	"000757ccbe"	0.006
"00074fd569"	"000757cfa9"	-0.4138
"00074fd569"	"000757d390"	-0.0924
"00074fd569"	"000757d393"	-0.0713
"00074fd569"	"000757d598"	-0.0446
"00074fd569"	"000757d5a2"	-0.1035

"00074fd569"	"000757d790"	-0.0448
"00074fd569"	"000757e30c"	-0.3211
"00074fd569"	"000757e4b0"	0.1352
"00074fd569"	"000757e7a0"	0.2006
"00074fd569"	"000757e8b3"	0.0174
"00074fd569"	"000757f627"	-0.2262
"00074fd569"	"000757f925"	0.0127
"00074fd569"	"000757fa08"	-0.1705
"00074fd569"	"000757fe52"	0.0886
"00074fd569"	"000758024a"	-0.2402
"00074fd569"	"00075804bb"	-0.4409
"00074fd569"	"00075a0c04"	0.2252
"00074fd569"	"00075a3110"	0.0119
"00074fd569"	"00075a341a"	0.2078
"00074fd569"	"00075a3dcf"	0.0149
"00074fd569"	"00075a3e22"	0.0237
"00074fd569"	"00075a48d8"	0.0609
"00074fd569"	"00075a5cfb"	-0.106
"00074fd569"	"00075a6151"	-0.3931
"00074fd569"	"00075a6708"	-0.2864
"00074fd569"	"00075a7319"	-0.1094
"00074fd569"	"00075a7723"	-0.0703
"00074fd569"	"00075a778b"	-0.013
"00074fd569"	"00075a7b8e"	-0.2033
"00074fd569"	"00075a7c79"	-0.0169
"00074fd569"	"00075a81b6"	-0.2757
"00074fd569"	"00075a82ac"	0.1867
"00074fd569"	"00075a98e5"	-0.1233
"00074fd569"	"00075b0d29"	-0.1226
"00074fd569"	"00075b102a"	-0.1439
"00074fd569"	"00075b1074"	-0.2979
"00074fd569"	"00075b135d"	-0.0563
"00074fd569"	"00075b138b"	0.0448
"00074fd569"	"00075b13a0"	-0.2565
"00074fd569"	"00075b13bd"	0.1499
"00074fd569"	"00075b16a9"	0.1347
"00074fd569"	"00075b1a28"	0.0143
"00074fd569"	"00075b1a97"	0.2286
"00074fd569"	"00075b1c7b"	0.0723
"00074fd569"	"00075b1d24"	-0.0594
"00074fd569"	"00075b202b"	0.0106
"00074fd569"	"00075b22cb"	0.1979
"00074fd569"	"00075b22da"	-0.2845
"00074fd569"	"00075b2556"	-0.2597
"00074fd569"	"00075b25de"	0.102
"00074fd569"	"00075b260c"	-0.1984
"00074fd569"	"00075b26f1"	0.0834
"00074fd569"	"00075b2920"	-0.1172
"00074fd569"	"00075b2a64"	-0.1956
"00074fd569"	"00075b2a9d"	-0.2338

"00074fd569"	"00075b2b37"	−0.0197
"00074fd569"	"00075b2cdd"	0.1792
"00074fd569"	"00075b3038"	0.3305
"00074fd569"	"00075b30fe"	−0.1597
"00074fd569"	"00075b3362"	0.0312
"00074fd569"	"00075b350a"	−0.3826
"00074fd569"	"00075b350e"	0.1621
"00074fd569"	"00075b3651"	0.0041
"00074fd569"	"00075b38ca"	−0.1407
"00074fd569"	"00075b39cc"	−0.0799
"00074fd569"	"00075b3e1e"	0.0494
"00074fd569"	"00075b3e57"	0.423
"00074fd569"	"00075b4079"	−0.1447
"00074fd569"	"00075b4150"	0.0112
"00074fd569"	"00075b4194"	−0.022
"00074fd569"	"00075b42d5"	−0.0895
"00074fd569"	"00075b4424"	0.1552
"00074fd569"	"00075b4470"	−0.1251
"00074fd569"	"00075b47ed"	0.1415
"00074fd569"	"00075b4850"	−0.0827
"00074fd569"	"00075b4ca0"	−0.1516
"00074fd569"	"00075b4d7f"	0.1171
"00074fd569"	"00075b520f"	0.0418
"00074fd569"	"00075b525f"	−0.0993
"00074fd569"	"00075b58f8"	−0.2964
"00074fd569"	"00075b5bcc"	0.1241
"00074fd569"	"00075b5bfa"	0.081
"00074fd569"	"00075b6339"	−0.0788
"00074fd569"	"00075b6658"	−0.0022
"00074fd569"	"00075b679a"	0.183
"00074fd569"	"00075b6cb7"	−0.4307
"00074fd569"	"00075b6df8"	0.2795
"00074fd569"	"00075b6ff6"	−0.0152
"00074fd569"	"00075b70ee"	−0.1055
"00074fd569"	"00075b7157"	0.039
"00074fd569"	"00075b7225"	0.0879
"00074fd569"	"00075b7c89"	0.0939
"00074fd569"	"00075b9048"	0.1834
"00074fd569"	"00075d0801"	−0.0671
"00074fd569"	"00075d1820"	0.3636
"00074fd569"	"00075d1f3d"	−0.1263
"00074fd569"	"00075d2329"	−0.0104
"00074fd569"	"00075d2b9b"	−0.1636
"00074fd569"	"00075d3941"	0.1652
"00074fd569"	"00075d3e96"	0.4537
"00074fd569"	"00075d4864"	−0.3345
"00074fd569"	"00075d5961"	−0.0527
"00074fd569"	"00075d5a63"	0.1647
"00074fd569"	"00075d6150"	−0.0275
"00074fd569"	"00075d67d0"	−0.1126

"00074fd569"	"00075d67e2"	−0.056
"00074fd569"	"00075d73fc"	0.3014
"00074fd569"	"00075d7729"	−0.0274
"00074fd569"	"00075d778c"	−0.2035
"00074fd569"	"00075d7b9e"	−0.0771
"00074fd569"	"00075d7c8f"	0.0247
"00074fd569"	"00075d804d"	−0.0826
"00074fd569"	"00075d819f"	−0.1997
"00074fd569"	"00075d8601"	0.3418
"00074fd569"	"00075d8c6a"	0.113
"00074fd569"	"00075dfedc"	0.0853
"00074fd569"	"00075e05f2"	−0.2013
"00074fd569"	"00075e0837"	−0.2495
"00074fd569"	"00075e092e"	−0.2625
"00074fd569"	"00075e0965"	−0.317
"00074fd569"	"00075e0bc8"	−0.0472
"00074fd569"	"00075e0fbb"	−0.4411
"00074fef15"	"00074ff562"	−0.5882
"00074fef15"	"00075007ca"	−0.4497
"00074fef15"	"0007500b86"	0.0129
"00074fef15"	"0007500d05"	−0.0606
"00074fef15"	"0007500ee4"	0.0047
"00074fef15"	"0007500eee"	−0.0449
"00074fef15"	"00075013dc"	−0.374
"00074fef15"	"000757b515"	−0.1438
"00074fef15"	"000757bc5a"	−0.1449
"00074fef15"	"000757c320"	−0.1892
"00074fef15"	"000757c9aa"	−0.0563
"00074fef15"	"000757ccbe"	−0.1188
"00074fef15"	"000757cfa9"	−0.1204
"00074fef15"	"000757d390"	−0.3647
"00074fef15"	"000757d393"	0.0238
"00074fef15"	"000757d598"	−0.3323
"00074fef15"	"000757d5a2"	−0.3213
"00074fef15"	"000757d790"	0.1645
"00074fef15"	"000757e30c"	−0.5445
"00074fef15"	"000757e4b0"	0.1074
"00074fef15"	"000757e7a0"	−0.1443
"00074fef15"	"000757e8b3"	−0.036
"00074fef15"	"000757f627"	−0.0568
"00074fef15"	"000757f925"	−0.1339
"00074fef15"	"000757fa08"	−0.1796
"00074fef15"	"000757fe52"	0.0025
"00074fef15"	"000758024a"	−0.5183
"00074fef15"	"00075804bb"	−0.7068
"00074fef15"	"00075a0c04"	0.1047
"00074fef15"	"00075a3110"	−0.0252
"00074fef15"	"00075a341a"	0.1748
"00074fef15"	"00075a3dcf"	0.0524
"00074fef15"	"00075a3e22"	−0.0239

"00074fef15"	"00075a48d8"	-0.0429
"00074fef15"	"00075a5cfb"	-0.5751
"00074fef15"	"00075a6151"	-0.46
"00074fef15"	"00075a6708"	-0.2444
"00074fef15"	"00075a7319"	-0.345
"00074fef15"	"00075a7723"	-0.1643
"00074fef15"	"00075a778b"	-0.112
"00074fef15"	"00075a7b8e"	-0.2106
"00074fef15"	"00075a7c79"	0.0354
"00074fef15"	"00075a81b6"	0.012
"00074fef15"	"00075a82ac"	0.2277
"00074fef15"	"00075a98e5"	0.0987
"00074fef15"	"00075b0d29"	0.0536
"00074fef15"	"00075b102a"	-0.2295
"00074fef15"	"00075b1074"	-0.1026
"00074fef15"	"00075b135d"	-0.1274
"00074fef15"	"00075b138b"	0.1134
"00074fef15"	"00075b13a0"	-0.0843
"00074fef15"	"00075b13bd"	0.2233
"00074fef15"	"00075b16a9"	-0.1879
"00074fef15"	"00075b1a28"	-0.0086
"00074fef15"	"00075b1a97"	0.4297
"00074fef15"	"00075b1c7b"	-0.2959
"00074fef15"	"00075b1d24"	0.2273
"00074fef15"	"00075b202b"	-0.3098
"00074fef15"	"00075b22cb"	0.0586
"00074fef15"	"00075b22da"	0.0291
"00074fef15"	"00075b2556"	-0.0769
"00074fef15"	"00075b25de"	-0.0923
"00074fef15"	"00075b260c"	-0.2654
"00074fef15"	"00075b26f1"	0.0442
"00074fef15"	"00075b2920"	-0.5078
"00074fef15"	"00075b2a64"	-0.5177
"00074fef15"	"00075b2a9d"	-0.3961
"00074fef15"	"00075b2b37"	-0.2976
"00074fef15"	"00075b2cdd"	-0.0644
"00074fef15"	"00075b3038"	-0.006
"00074fef15"	"00075b30fe"	0.0425
"00074fef15"	"00075b3362"	0.1431
"00074fef15"	"00075b350a"	-0.2333
"00074fef15"	"00075b350e"	-0.0534
"00074fef15"	"00075b3651"	0.2557
"00074fef15"	"00075b38ca"	-0.076
"00074fef15"	"00075b39cc"	-0.2345
"00074fef15"	"00075b3e1e"	-0.0403
"00074fef15"	"00075b3e57"	0.1506
"00074fef15"	"00075b4079"	-0.296
"00074fef15"	"00075b4150"	-0.5186
"00074fef15"	"00075b4194"	-0.1708
"00074fef15"	"00075b42d5"	0.0832

"00074fef15"	"00075b4424"	−0.084
"00074fef15"	"00075b4470"	−0.1449
"00074fef15"	"00075b47ed"	−0.1838
"00074fef15"	"00075b4850"	−0.0293
"00074fef15"	"00075b4ca0"	−0.2658
"00074fef15"	"00075b4d7f"	0.1278
"00074fef15"	"00075b520f"	−0.1113
"00074fef15"	"00075b525f"	0.2728
"00074fef15"	"00075b58f8"	−0.5727
"00074fef15"	"00075b5bcc"	0.193
"00074fef15"	"00075b5bfa"	−0.0828
"00074fef15"	"00075b6339"	0.0142
"00074fef15"	"00075b6658"	0.1749
"00074fef15"	"00075b679a"	0.2342
"00074fef15"	"00075b6cb7"	−0.2658
"00074fef15"	"00075b6df8"	0.1346
"00074fef15"	"00075b6ff6"	0.0214
"00074fef15"	"00075b70ee"	−0.3412
"00074fef15"	"00075b7157"	0.0933
"00074fef15"	"00075b7225"	0.3069
"00074fef15"	"00075b7c89"	−0.128
"00074fef15"	"00075b9048"	−0.098
"00074fef15"	"00075d0801"	−0.3572
"00074fef15"	"00075d1820"	0.3708
"00074fef15"	"00075d1f3d"	−0.2521
"00074fef15"	"00075d2329"	−0.0914
"00074fef15"	"00075d2b9b"	−0.3938
"00074fef15"	"00075d3941"	−0.2251
"00074fef15"	"00075d3e96"	−0.0861
"00074fef15"	"00075d4864"	−0.2163
"00074fef15"	"00075d5961"	0.0984
"00074fef15"	"00075d5a63"	−0.0439
"00074fef15"	"00075d6150"	−0.0486
"00074fef15"	"00075d67d0"	−0.1136
"00074fef15"	"00075d67e2"	0.0369
"00074fef15"	"00075d73fc"	0.1772
"00074fef15"	"00075d7729"	0.1898
"00074fef15"	"00075d778c"	0.3383
"00074fef15"	"00075d7b9e"	0.2799
"00074fef15"	"00075d7c8f"	0.1145
"00074fef15"	"00075d804d"	−0.0163
"00074fef15"	"00075d819f"	−0.2325
"00074fef15"	"00075d8601"	0.0144
"00074fef15"	"00075d8c6a"	−0.0299
"00074fef15"	"00075dfedc"	−0.0101
"00074fef15"	"00075e05f2"	−0.238
"00074fef15"	"00075e0837"	−0.2388
"00074fef15"	"00075e092e"	−0.0048
"00074fef15"	"00075e0965"	−0.7832
"00074fef15"	"00075e0bc8"	−0.0137

"00074fef15"	"00075e0fbb"	−0.0848
"00074ff562"	"00075007ca"	−0.0174
"00074ff562"	"0007500b86"	−0.098
"00074ff562"	"0007500d05"	0.2891
"00074ff562"	"0007500ee4"	−0.1033
"00074ff562"	"0007500eee"	−0.2924
"00074ff562"	"00075013dc"	−0.0346
"00074ff562"	"000757b515"	−0.2407
"00074ff562"	"000757bc5a"	−0.3891
"00074ff562"	"000757c320"	−0.3893
"00074ff562"	"000757c9aa"	−0.2664
"00074ff562"	"000757ccbe"	−0.3366
"00074ff562"	"000757cfa9"	−0.3358
"00074ff562"	"000757d390"	−0.4371
"00074ff562"	"000757d393"	−0.311
"00074ff562"	"000757d598"	−0.4748
"00074ff562"	"000757d5a2"	−0.465
"00074ff562"	"000757d790"	−0.2812
"00074ff562"	"000757e30c"	−0.3434
"00074ff562"	"000757e4b0"	−0.1187
"00074ff562"	"000757e7a0"	−0.29
"00074ff562"	"000757e8b3"	−0.2175
"00074ff562"	"000757f627"	−0.4405
"00074ff562"	"000757f925"	−0.297
"00074ff562"	"000757fa08"	−0.1632
"00074ff562"	"000757fe52"	−0.2195
"00074ff562"	"000758024a"	−0.2979
"00074ff562"	"00075804bb"	−0.3516
"00074ff562"	"00075a0c04"	0.0487
"00074ff562"	"00075a3110"	−0.1168
"00074ff562"	"00075a341a"	−0.116
"00074ff562"	"00075a3dcf"	−0.0473
"00074ff562"	"00075a3e22"	−0.1875
"00074ff562"	"00075a48d8"	−0.2543
"00074ff562"	"00075a5cfb"	−0.2432
"00074ff562"	"00075a6151"	−0.1033
"00074ff562"	"00075a6708"	−0.4178
"00074ff562"	"00075a7319"	−0.5115
"00074ff562"	"00075a7723"	−0.2185
"00074ff562"	"00075a778b"	−0.2657
"00074ff562"	"00075a7b8e"	−0.2602
"00074ff562"	"00075a7c79"	−0.1826
"00074ff562"	"00075a81b6"	−0.287
"00074ff562"	"00075a82ac"	−0.2097
"00074ff562"	"00075a98e5"	−0.255
"00074ff562"	"00075b0d29"	−0.2228
"00074ff562"	"00075b102a"	−0.3368
"00074ff562"	"00075b1074"	−0.5231
"00074ff562"	"00075b135d"	−0.4722
"00074ff562"	"00075b138b"	−0.2232

"00074ff562"	"00075b13a0"	−0.4352
"00074ff562"	"00075b13bd"	−0.3328
"00074ff562"	"00075b16a9"	−0.4038
"00074ff562"	"00075b1a28"	−0.4891
"00074ff562"	"00075b1a97"	−0.2708
"00074ff562"	"00075b1c7b"	−0.8171
"00074ff562"	"00075b1d24"	−0.4535
"00074ff562"	"00075b202b"	0.1225
"00074ff562"	"00075b22cb"	−0.3512
"00074ff562"	"00075b22da"	−0.4044
"00074ff562"	"00075b2556"	−0.3043
"00074ff562"	"00075b25de"	−0.1244
"00074ff562"	"00075b260c"	−0.117
"00074ff562"	"00075b26f1"	−0.1573
"00074ff562"	"00075b2920"	−0.1633
"00074ff562"	"00075b2a64"	−0.2887
"00074ff562"	"00075b2a9d"	−0.1153
"00074ff562"	"00075b2b37"	−0.1144
"00074ff562"	"00075b2cdd"	0.032
"00074ff562"	"00075b3038"	0.0401
"00074ff562"	"00075b30fe"	−0.3932
"00074ff562"	"00075b3362"	−0.5777
"00074ff562"	"00075b350a"	−0.1738
"00074ff562"	"00075b350e"	−0.2198
"00074ff562"	"00075b3651"	−0.3937
"00074ff562"	"00075b38ca"	−0.1673
"00074ff562"	"00075b39cc"	0.0226
"00074ff562"	"00075b3e1e"	−0.0773
"00074ff562"	"00075b3e57"	−0.386
"00074ff562"	"00075b4079"	−0.3052
"00074ff562"	"00075b4150"	−0.0891
"00074ff562"	"00075b4194"	−0.1598
"00074ff562"	"00075b42d5"	−0.0601
"00074ff562"	"00075b4424"	−0.0723
"00074ff562"	"00075b4470"	−0.3891
"00074ff562"	"00075b47ed"	0.1388
"00074ff562"	"00075b4850"	−0.2334
"00074ff562"	"00075b4ca0"	−0.2299
"00074ff562"	"00075b4d7f"	−0.1629
"00074ff562"	"00075b520f"	0.0527
"00074ff562"	"00075b525f"	−0.2069
"00074ff562"	"00075b58f8"	−0.3875
"00074ff562"	"00075b5bcc"	−0.2965
"00074ff562"	"00075b5bfa"	−0.0725
"00074ff562"	"00075b6339"	−0.2873
"00074ff562"	"00075b6658"	−0.2291
"00074ff562"	"00075b679a"	−0.4047
"00074ff562"	"00075b6cb7"	−0.585
"00074ff562"	"00075b6df8"	−0.0921
"00074ff562"	"00075b6ff6"	−0.3062

"00074ff562"	"00075b70ee"	-0.403
"00074ff562"	"00075b7157"	-0.1913
"00074ff562"	"00075b7225"	-0.4355
"00074ff562"	"00075b7c89"	-0.3202
"00074ff562"	"00075b9048"	-0.0865
"00074ff562"	"00075d0801"	0.2761
"00074ff562"	"00075d1820"	-0.5751
"00074ff562"	"00075d1f3d"	-0.2046
"00074ff562"	"00075d2329"	0.271
"00074ff562"	"00075d2b9b"	-0.01
"00074ff562"	"00075d3941"	0.0651
"00074ff562"	"00075d3e96"	-0.3657
"00074ff562"	"00075d4864"	-0.0424
"00074ff562"	"00075d5961"	-0.3558
"00074ff562"	"00075d5a63"	0.0352
"00074ff562"	"00075d6150"	-0.1222
"00074ff562"	"00075d67d0"	-0.488
"00074ff562"	"00075d67e2"	-0.5586
"00074ff562"	"00075d73fc"	-0.3788
"00074ff562"	"00075d7729"	-0.1192
"00074ff562"	"00075d778c"	-0.2607
"00074ff562"	"00075d7b9e"	-0.1407
"00074ff562"	"00075d7c8f"	-0.501
"00074ff562"	"00075d804d"	-0.1974
"00074ff562"	"00075d819f"	-0.3876
"00074ff562"	"00075d8601"	-3e-04
"00074ff562"	"00075d8c6a"	0.0375
"00074ff562"	"00075dfedc"	-0.0657
"00074ff562"	"00075e05f2"	-0.3302
"00074ff562"	"00075e0837"	0.0251
"00074ff562"	"00075e092e"	-0.5367
"00074ff562"	"00075e0965"	-0.2114
"00074ff562"	"00075e0bc8"	-0.1332
"00074ff562"	"00075e0fbb"	-0.4947
"00075007ca"	"0007500b86"	-0.1729
"00075007ca"	"0007500d05"	0.0633
"00075007ca"	"0007500ee4"	-0.0588
"00075007ca"	"0007500eee"	0.0362
"00075007ca"	"00075013dc"	-0.0937
"00075007ca"	"000757b515"	0.1481
"00075007ca"	"000757bc5a"	-0.2137
"00075007ca"	"000757c320"	-0.0912
"00075007ca"	"000757c9aa"	0.0877
"00075007ca"	"000757ccbe"	-0.3083
"00075007ca"	"000757cfa9"	-0.1627
"00075007ca"	"000757d390"	-0.0117
"00075007ca"	"000757d393"	-0.0957
"00075007ca"	"000757d598"	0.0933
"00075007ca"	"000757d5a2"	0.1929
"00075007ca"	"000757d790"	0.1135

"00075007ca"	"000757e30c"	0.179
"00075007ca"	"000757e4b0"	−0.0609
"00075007ca"	"000757e7a0"	0.0999
"00075007ca"	"000757e8b3"	−0.1359
"00075007ca"	"000757f627"	−0.0625
"00075007ca"	"000757f925"	0.1463
"00075007ca"	"000757fa08"	−0.0634
"00075007ca"	"000757fe52"	−0.3628
"00075007ca"	"000758024a"	0.1602
"00075007ca"	"00075804bb"	0.1264
"00075007ca"	"00075a0c04"	−0.1416
"00075007ca"	"00075a3110"	−0.1063
"00075007ca"	"00075a341a"	−0.3918
"00075007ca"	"00075a3dcf"	−0.1716
"00075007ca"	"00075a3e22"	−0.2607
"00075007ca"	"00075a48d8"	−0.4011
"00075007ca"	"00075a5cfb"	0.1163
"00075007ca"	"00075a6151"	0.1801
"00075007ca"	"00075a6708"	−0.1311
"00075007ca"	"00075a7319"	−0.0399
"00075007ca"	"00075a7723"	−0.2048
"00075007ca"	"00075a778b"	−0.4107
"00075007ca"	"00075a7b8e"	0.1272
"00075007ca"	"00075a7c79"	−0.1061
"00075007ca"	"00075a81b6"	−0.1679
"00075007ca"	"00075a82ac"	−0.2765
"00075007ca"	"00075a98e5"	−0.4425
"00075007ca"	"00075b0d29"	−0.3467
"00075007ca"	"00075b102a"	0.3618
"00075007ca"	"00075b1074"	−0.1223
"00075007ca"	"00075b135d"	−0.3144
"00075007ca"	"00075b138b"	0.0689
"00075007ca"	"00075b13a0"	−0.7522
"00075007ca"	"00075b13bd"	−0.4408
"00075007ca"	"00075b16a9"	0.0558
"00075007ca"	"00075b1a28"	−0.1513
"00075007ca"	"00075b1a97"	−0.1204
"00075007ca"	"00075b1c7b"	−0.2161
"00075007ca"	"00075b1d24"	−0.0648
"00075007ca"	"00075b202b"	0.4736
"00075007ca"	"00075b22cb"	−0.2519
"00075007ca"	"00075b22da"	−0.1392
"00075007ca"	"00075b2556"	−0.143
"00075007ca"	"00075b25de"	−0.02
"00075007ca"	"00075b260c"	0.5059
"00075007ca"	"00075b26f1"	−0.1276
"00075007ca"	"00075b2920"	0.2684
"00075007ca"	"00075b2a64"	0.0287
"00075007ca"	"00075b2a9d"	0.5245
"00075007ca"	"00075b2b37"	0.1141

"00075007ca"	"00075b2cdd"	0.0307
"00075007ca"	"00075b3038"	0.0015
"00075007ca"	"00075b30fe"	−0.1097
"00075007ca"	"00075b3362"	−0.4732
"00075007ca"	"00075b350a"	−0.0575
"00075007ca"	"00075b350e"	0.022
"00075007ca"	"00075b3651"	−0.3008
"00075007ca"	"00075b38ca"	−0.236
"00075007ca"	"00075b39cc"	−0.0964
"00075007ca"	"00075b3e1e"	0.0576
"00075007ca"	"00075b3e57"	−0.1574
"00075007ca"	"00075b4079"	−0.0428
"00075007ca"	"00075b4150"	0.2627
"00075007ca"	"00075b4194"	−0.0889
"00075007ca"	"00075b42d5"	−0.1328
"00075007ca"	"00075b4424"	−0.202
"00075007ca"	"00075b4470"	−0.1381
"00075007ca"	"00075b47ed"	−0.2096
"00075007ca"	"00075b4850"	−0.1073
"00075007ca"	"00075b4ca0"	6e−04
"00075007ca"	"00075b4d7f"	−0.2259
"00075007ca"	"00075b520f"	−0.1619
"00075007ca"	"00075b525f"	−0.1515
"00075007ca"	"00075b58f8"	0.0485
"00075007ca"	"00075b5bcc"	0.0229
"00075007ca"	"00075b5bfa"	−0.1057
"00075007ca"	"00075b6339"	0.0766
"00075007ca"	"00075b6658"	−0.4187
"00075007ca"	"00075b679a"	−0.0424
"00075007ca"	"00075b6cb7"	−0.1124
"00075007ca"	"00075b6df8"	−0.3001
"00075007ca"	"00075b6ff6"	−0.0882
"00075007ca"	"00075b70ee"	−0.1472
"00075007ca"	"00075b7157"	−0.2477
"00075007ca"	"00075b7225"	−0.3127
"00075007ca"	"00075b7c89"	−0.3333
"00075007ca"	"00075b9048"	0.034
"00075007ca"	"00075d0801"	0.0019
"00075007ca"	"00075d1820"	−0.1727
"00075007ca"	"00075d1f3d"	0.056
"00075007ca"	"00075d2329"	−0.0579
"00075007ca"	"00075d2b9b"	0.1586
"00075007ca"	"00075d3941"	−0.3157
"00075007ca"	"00075d3e96"	0.0251
"00075007ca"	"00075d4864"	−0.2658
"00075007ca"	"00075d5961"	−0.0672
"00075007ca"	"00075d5a63"	−0.0501
"00075007ca"	"00075d6150"	0.0067
"00075007ca"	"00075d67d0"	−0.024
"00075007ca"	"00075d67e2"	−0.4294

"00075007ca"	"00075d73fc"	-0.411
"00075007ca"	"00075d7729"	-0.1007
"00075007ca"	"00075d778c"	-0.2133
"00075007ca"	"00075d7b9e"	-0.0413
"00075007ca"	"00075d7c8f"	-0.1233
"00075007ca"	"00075d804d"	0.0877
"00075007ca"	"00075d819f"	0.0421
"00075007ca"	"00075d8601"	0.0186
"00075007ca"	"00075d8c6a"	-0.2194
"00075007ca"	"00075dfedc"	-0.1079
"00075007ca"	"00075e05f2"	0.0558
"00075007ca"	"00075e0837"	0.5226
"00075007ca"	"00075e092e"	-0.2277
"00075007ca"	"00075e0965"	-0.0535
"00075007ca"	"00075e0bc8"	0.009
"00075007ca"	"00075e0fbb"	-0.2237
"0007500b86"	"0007500d05"	0.3157
"0007500b86"	"0007500ee4"	0.0317
"0007500b86"	"0007500eee"	0.0977
"0007500b86"	"00075013dc"	0.116
"0007500b86"	"000757b515"	-0.0917
"0007500b86"	"000757bc5a"	0.3932
"0007500b86"	"000757c320"	0.1026
"0007500b86"	"000757c9aa"	0.0762
"0007500b86"	"000757ccbe"	0.1142
"0007500b86"	"000757cfa9"	0.1051
"0007500b86"	"000757d390"	-0.2657
"0007500b86"	"000757d393"	0.3338
"0007500b86"	"000757d598"	-0.1905
"0007500b86"	"000757d5a2"	-0.4415
"0007500b86"	"000757d790"	0.0458
"0007500b86"	"000757e30c"	0.0289
"0007500b86"	"000757e4b0"	0.1601
"0007500b86"	"000757e7a0"	-0.197
"0007500b86"	"000757e8b3"	0.3234
"0007500b86"	"000757f627"	0.3021
"0007500b86"	"000757f925"	0.2865
"0007500b86"	"000757fa08"	-0.1404
"0007500b86"	"000757fe52"	0.2207
"0007500b86"	"000758024a"	0.0427
"0007500b86"	"00075804bb"	0.1703
"0007500b86"	"00075a0c04"	-0.0867
"0007500b86"	"00075a3110"	0.0432
"0007500b86"	"00075a341a"	-0.0214
"0007500b86"	"00075a3dcf"	0.0497
"0007500b86"	"00075a3e22"	0.2049
"0007500b86"	"00075a48d8"	0.0546
"0007500b86"	"00075a5cfb"	0.231
"0007500b86"	"00075a6151"	-0.0113
"0007500b86"	"00075a6708"	-0.1173

"0007500b86"	"00075a7319"	-0.266
"0007500b86"	"00075a7723"	0.1698
"0007500b86"	"00075a778b"	0.1026
"0007500b86"	"00075a7b8e"	0.0075
"0007500b86"	"00075a7c79"	0.1686
"0007500b86"	"00075a81b6"	-0.0702
"0007500b86"	"00075a82ac"	0.2458
"0007500b86"	"00075a98e5"	0.3911
"0007500b86"	"00075b0d29"	0.3474
"0007500b86"	"00075b102a"	0.1095
"0007500b86"	"00075b1074"	-0.4231
"0007500b86"	"00075b135d"	-0.0836
"0007500b86"	"00075b138b"	0.2506
"0007500b86"	"00075b13a0"	-0.0283
"0007500b86"	"00075b13bd"	0.0401
"0007500b86"	"00075b16a9"	0.0173
"0007500b86"	"00075b1a28"	-0.0198
"0007500b86"	"00075b1a97"	0.1011
"0007500b86"	"00075b1c7b"	-0.3401
"0007500b86"	"00075b1d24"	-0.0354
"0007500b86"	"00075b202b"	-0.0254
"0007500b86"	"00075b22cb"	0.0095
"0007500b86"	"00075b22da"	-0.1765
"0007500b86"	"00075b2556"	-0.2463
"0007500b86"	"00075b25de"	0.3392
"0007500b86"	"00075b260c"	-0.0302
"0007500b86"	"00075b26f1"	-0.0945
"0007500b86"	"00075b2920"	0.1447
"0007500b86"	"00075b2a64"	0.2052
"0007500b86"	"00075b2a9d"	-0.069
"0007500b86"	"00075b2b37"	-0.3312
"0007500b86"	"00075b2cdd"	-0.0214
"0007500b86"	"00075b3038"	0.2986
"0007500b86"	"00075b30fe"	-0.2103
"0007500b86"	"00075b3362"	-0.2178
"0007500b86"	"00075b350a"	0.0831
"0007500b86"	"00075b350e"	0.1697
"0007500b86"	"00075b3651"	0.0238
"0007500b86"	"00075b38ca"	0.0489
"0007500b86"	"00075b39cc"	0.041
"0007500b86"	"00075b3e1e"	0.2407
"0007500b86"	"00075b3e57"	0.1089
"0007500b86"	"00075b4079"	0.1097
"0007500b86"	"00075b4150"	0.007
"0007500b86"	"00075b4194"	0.4478
"0007500b86"	"00075b42d5"	0.3297
"0007500b86"	"00075b4424"	-0.0161
"0007500b86"	"00075b4470"	0.359
"0007500b86"	"00075b47ed"	0.0687
"0007500b86"	"00075b4850"	0.3132

"0007500b86"	"00075b4ca0"	-0.2577
"0007500b86"	"00075b4d7f"	0.2779
"0007500b86"	"00075b520f"	0.0422
"0007500b86"	"00075b525f"	-0.1462
"0007500b86"	"00075b58f8"	0.1155
"0007500b86"	"00075b5bcc"	0.1011
"0007500b86"	"00075b5bfa"	-0.1129
"0007500b86"	"00075b6339"	0.1906
"0007500b86"	"00075b6658"	-0.1386
"0007500b86"	"00075b679a"	0.151
"0007500b86"	"00075b6cb7"	0.1356
"0007500b86"	"00075b6df8"	-0.0212
"0007500b86"	"00075b6ff6"	0.1691
"0007500b86"	"00075b70ee"	-0.1718
"0007500b86"	"00075b7157"	0.0692
"0007500b86"	"00075b7225"	-0.0662
"0007500b86"	"00075b7c89"	-0.0376
"0007500b86"	"00075b9048"	0.1749
"0007500b86"	"00075d0801"	0.3714
"0007500b86"	"00075d1820"	-0.2518
"0007500b86"	"00075d1f3d"	0.1079
"0007500b86"	"00075d2329"	0.1003
"0007500b86"	"00075d2b9b"	0.1889
"0007500b86"	"00075d3941"	-0.0142
"0007500b86"	"00075d3e96"	-0.2176
"0007500b86"	"00075d4864"	-0.0086
"0007500b86"	"00075d5961"	0.0037
"0007500b86"	"00075d5a63"	0.2171
"0007500b86"	"00075d6150"	-0.0753
"0007500b86"	"00075d67d0"	-0.309
"0007500b86"	"00075d67e2"	0.2465
"0007500b86"	"00075d73fc"	0.0897
"0007500b86"	"00075d7729"	-0.3645
"0007500b86"	"00075d778c"	-0.2194
"0007500b86"	"00075d7b9e"	0.0425
"0007500b86"	"00075d7c8f"	-0.0077
"0007500b86"	"00075d804d"	0.2703
"0007500b86"	"00075d819f"	0.0054
"0007500b86"	"00075d8601"	0.0592
"0007500b86"	"00075d8c6a"	0.172
"0007500b86"	"00075dfedc"	0.1823
"0007500b86"	"00075e05f2"	0.375
"0007500b86"	"00075e0837"	-0.0566
"0007500b86"	"00075e092e"	-0.1875
"0007500b86"	"00075e0965"	-0.0615
"0007500b86"	"00075e0bc8"	0.251
"0007500b86"	"00075e0fbb"	-0.3122
"0007500d05"	"0007500ee4"	0.2287
"0007500d05"	"0007500eee"	0.4199
"0007500d05"	"00075013dc"	-0.3275

"0007500d05"	"000757b515"	-0.0191
"0007500d05"	"000757bc5a"	0.1028
"0007500d05"	"000757c320"	-0.2002
"0007500d05"	"000757c9aa"	-0.2592
"0007500d05"	"000757ccbe"	-0.0594
"0007500d05"	"000757cfa9"	-0.456
"0007500d05"	"000757d390"	-0.1386
"0007500d05"	"000757d393"	0.169
"0007500d05"	"000757d598"	-0.2534
"0007500d05"	"000757d5a2"	-0.3907
"0007500d05"	"000757d790"	0.3146
"0007500d05"	"000757e30c"	-0.1215
"0007500d05"	"000757e4b0"	-0.0908
"0007500d05"	"000757e7a0"	0.0037
"0007500d05"	"000757e8b3"	-0.0033
"0007500d05"	"000757f627"	-0.153
"0007500d05"	"000757f925"	0.2923
"0007500d05"	"000757fa08"	0.0119
"0007500d05"	"000757fe52"	0.2863
"0007500d05"	"000758024a"	-0.2204
"0007500d05"	"00075804bb"	-0.2537
"0007500d05"	"00075a0c04"	0.2668
"0007500d05"	"00075a3110"	0.1583
"0007500d05"	"00075a341a"	0.1282
"0007500d05"	"00075a3dcf"	0.1006
"0007500d05"	"00075a3e22"	0.1224
"0007500d05"	"00075a48d8"	0.2181
"0007500d05"	"00075a5cfb"	0.0093
"0007500d05"	"00075a6151"	-0.0485
"0007500d05"	"00075a6708"	-0.2274
"0007500d05"	"00075a7319"	-0.2424
"0007500d05"	"00075a7723"	-0.0205
"0007500d05"	"00075a778b"	0.0446
"0007500d05"	"00075a7b8e"	0.2333
"0007500d05"	"00075a7c79"	0.1014
"0007500d05"	"00075a81b6"	-0.1479
"0007500d05"	"00075a82ac"	0.3193
"0007500d05"	"00075a98e5"	0.0299
"0007500d05"	"00075b0d29"	-0.1615
"0007500d05"	"00075b102a"	0.0542
"0007500d05"	"00075b1074"	-0.2073
"0007500d05"	"00075b135d"	-0.4216
"0007500d05"	"00075b138b"	0.1648
"0007500d05"	"00075b13a0"	-0.0626
"0007500d05"	"00075b13bd"	-0.0578
"0007500d05"	"00075b16a9"	0.2461
"0007500d05"	"00075b1a28"	-0.1081
"0007500d05"	"00075b1a97"	0.0477
"0007500d05"	"00075b1c7b"	-0.0827
"0007500d05"	"00075b1d24"	-0.0685

"0007500d05"	"00075b202b"	0.2308
"0007500d05"	"00075b22cb"	0.2094
"0007500d05"	"00075b22da"	−0.1131
"0007500d05"	"00075b2556"	−0.0783
"0007500d05"	"00075b25de"	0.374
"0007500d05"	"00075b260c"	−0.0196
"0007500d05"	"00075b26f1"	−0.0477
"0007500d05"	"00075b2920"	0.0657
"0007500d05"	"00075b2a64"	−0.0447
"0007500d05"	"00075b2a9d"	−0.1063
"0007500d05"	"00075b2b37"	−0.0215
"0007500d05"	"00075b2cdd"	0.2563
"0007500d05"	"00075b3038"	0.3797
"0007500d05"	"00075b30fe"	−0.1333
"0007500d05"	"00075b3362"	−0.1782
"0007500d05"	"00075b350a"	−0.1025
"0007500d05"	"00075b350e"	−0.0571
"0007500d05"	"00075b3651"	0.0031
"0007500d05"	"00075b38ca"	0.0989
"0007500d05"	"00075b39cc"	0.0984
"0007500d05"	"00075b3e1e"	0.2659
"0007500d05"	"00075b3e57"	0.3101
"0007500d05"	"00075b4079"	−0.0909
"0007500d05"	"00075b4150"	0.0328
"0007500d05"	"00075b4194"	0.0964
"0007500d05"	"00075b42d5"	0.1344
"0007500d05"	"00075b4424"	−0.0347
"0007500d05"	"00075b4470"	0.0872
"0007500d05"	"00075b47ed"	0.2556
"0007500d05"	"00075b4850"	−0.0349
"0007500d05"	"00075b4ca0"	−0.4429
"0007500d05"	"00075b4d7f"	0.3435
"0007500d05"	"00075b520f"	−0.0838
"0007500d05"	"00075b525f"	−0.187
"0007500d05"	"00075b58f8"	0.0287
"0007500d05"	"00075b5bcc"	0.1304
"0007500d05"	"00075b5bfa"	0.3946
"0007500d05"	"00075b6339"	−0.0434
"0007500d05"	"00075b6658"	−0.2181
"0007500d05"	"00075b679a"	−0.0093
"0007500d05"	"00075b6cb7"	−0.2669
"0007500d05"	"00075b6df8"	0.3632
"0007500d05"	"00075b6ff6"	0.0319
"0007500d05"	"00075b70ee"	−0.2241
"0007500d05"	"00075b7157"	0.4282
"0007500d05"	"00075b7225"	0.0199
"0007500d05"	"00075b7c89"	−0.1038
"0007500d05"	"00075b9048"	0.18
"0007500d05"	"00075d0801"	0.2837
"0007500d05"	"00075d1820"	−0.0104

"0007500d05"	"00075d1f3d"	-0.0512
"0007500d05"	"00075d2329"	0.3414
"0007500d05"	"00075d2b9b"	-0.1173
"0007500d05"	"00075d3941"	0.0797
"0007500d05"	"00075d3e96"	0.1178
"0007500d05"	"00075d4864"	-0.0967
"0007500d05"	"00075d5961"	0.1759
"0007500d05"	"00075d5a63"	0.2096
"0007500d05"	"00075d6150"	-0.0829
"0007500d05"	"00075d67d0"	-0.3482
"0007500d05"	"00075d67e2"	-0.0673
"0007500d05"	"00075d73fc"	0.0776
"0007500d05"	"00075d7729"	-0.2039
"0007500d05"	"00075d778c"	-0.035
"0007500d05"	"00075d7b9e"	0.2594
"0007500d05"	"00075d7c8f"	-0.1045
"0007500d05"	"00075d804d"	0.166
"0007500d05"	"00075d819f"	-0.1684
"0007500d05"	"00075d8601"	0.2727
"0007500d05"	"00075d8c6a"	0.3217
"0007500d05"	"00075dfedc"	0.1282
"0007500d05"	"00075e05f2"	0.115
"0007500d05"	"00075e0837"	0.1556
"0007500d05"	"00075e092e"	-0.1208
"0007500d05"	"00075e0965"	-0.0757
"0007500d05"	"00075e0bc8"	0.1088
"0007500d05"	"00075e0fbb"	-0.1225
"0007500ee4"	"0007500eee"	-0.2904
"0007500ee4"	"00075013dc"	-0.259
"0007500ee4"	"000757b515"	0.0979
"0007500ee4"	"000757bc5a"	-0.0725
"0007500ee4"	"000757c320"	-0.2602
"0007500ee4"	"000757c9aa"	-0.1424
"0007500ee4"	"000757ccbe"	-0.0777
"0007500ee4"	"000757cfa9"	-0.0443
"0007500ee4"	"000757d390"	0.0116
"0007500ee4"	"000757d393"	0.1419
"0007500ee4"	"000757d598"	-0.0816
"0007500ee4"	"000757d5a2"	0.1414
"0007500ee4"	"000757d790"	0.0056
"0007500ee4"	"000757e30c"	-0.001
"0007500ee4"	"000757e4b0"	0.0419
"0007500ee4"	"000757e7a0"	0.5137
"0007500ee4"	"000757e8b3"	-0.076
"0007500ee4"	"000757f627"	-0.0755
"0007500ee4"	"000757f925"	0.1528
"0007500ee4"	"000757fa08"	-0.2695
"0007500ee4"	"000757fe52"	-0.0626
"0007500ee4"	"000758024a"	-0.2786
"0007500ee4"	"00075804bb"	-0.3566

"0007500ee4"	"00075a0c04"	-0.026
"0007500ee4"	"00075a3110"	-0.1407
"0007500ee4"	"00075a341a"	0.1168
"0007500ee4"	"00075a3dcf"	0.0893
"0007500ee4"	"00075a3e22"	-0.2078
"0007500ee4"	"00075a48d8"	-0.198
"0007500ee4"	"00075a5cfb"	-0.1573
"0007500ee4"	"00075a6151"	-0.2271
"0007500ee4"	"00075a6708"	0.1011
"0007500ee4"	"00075a7319"	0.0869
"0007500ee4"	"00075a7723"	-0.0955
"0007500ee4"	"00075a778b"	0.0472
"0007500ee4"	"00075a7b8e"	0.1121
"0007500ee4"	"00075a7c79"	-0.0283
"0007500ee4"	"00075a81b6"	-0.286
"0007500ee4"	"00075a82ac"	0.3329
"0007500ee4"	"00075a98e5"	-0.0239
"0007500ee4"	"00075b0d29"	-0.0703
"0007500ee4"	"00075b102a"	-0.193
"0007500ee4"	"00075b1074"	-0.1941
"0007500ee4"	"00075b135d"	-0.1052
"0007500ee4"	"00075b138b"	-0.0309
"0007500ee4"	"00075b13a0"	-0.3128
"0007500ee4"	"00075b13bd"	-0.0071
"0007500ee4"	"00075b16a9"	0.2005
"0007500ee4"	"00075b1a28"	0.0122
"0007500ee4"	"00075b1a97"	0.0881
"0007500ee4"	"00075b1c7b"	0.0506
"0007500ee4"	"00075b1d24"	0.0431
"0007500ee4"	"00075b202b"	-0.0739
"0007500ee4"	"00075b22cb"	0.189
"0007500ee4"	"00075b22da"	-0.0626
"0007500ee4"	"00075b2556"	-0.0077
"0007500ee4"	"00075b25de"	0.1833
"0007500ee4"	"00075b260c"	-0.3036
"0007500ee4"	"00075b26f1"	0.0058
"0007500ee4"	"00075b2920"	-0.0283
"0007500ee4"	"00075b2a64"	-0.2753
"0007500ee4"	"00075b2a9d"	0.0306
"0007500ee4"	"00075b2b37"	0.3168
"0007500ee4"	"00075b2cdd"	0.2479
"0007500ee4"	"00075b3038"	-0.0363
"0007500ee4"	"00075b30fe"	-0.0873
"0007500ee4"	"00075b3362"	0.0501
"0007500ee4"	"00075b350a"	-0.0994
"0007500ee4"	"00075b350e"	0.0012
"0007500ee4"	"00075b3651"	0.1135
"0007500ee4"	"00075b38ca"	-0.058
"0007500ee4"	"00075b39cc"	0.1628
"0007500ee4"	"00075b3e1e"	-0.0495

"0007500ee4"	"00075b3e57"	0.2349
"0007500ee4"	"00075b4079"	-0.1177
"0007500ee4"	"00075b4150"	-0.0538
"0007500ee4"	"00075b4194"	0.0828
"0007500ee4"	"00075b42d5"	-0.0586
"0007500ee4"	"00075b4424"	0.2115
"0007500ee4"	"00075b4470"	-0.0881
"0007500ee4"	"00075b47ed"	0.2263
"0007500ee4"	"00075b4850"	-0.0318
"0007500ee4"	"00075b4ca0"	-0.0809
"0007500ee4"	"00075b4d7f"	0.014
"0007500ee4"	"00075b520f"	0.2554
"0007500ee4"	"00075b525f"	0.0908
"0007500ee4"	"00075b58f8"	0.1481
"0007500ee4"	"00075b5bcc"	-0.1571
"0007500ee4"	"00075b5bfa"	-0.2141
"0007500ee4"	"00075b6339"	-0.0794
"0007500ee4"	"00075b6658"	0.1219
"0007500ee4"	"00075b679a"	-0.0334
"0007500ee4"	"00075b6cb7"	-0.1634
"0007500ee4"	"00075b6df8"	0.0208
"0007500ee4"	"00075b6ff6"	-0.1487
"0007500ee4"	"00075b70ee"	-0.1192
"0007500ee4"	"00075b7157"	0.2362
"0007500ee4"	"00075b7225"	-0.1262
"0007500ee4"	"00075b7c89"	0.0179
"0007500ee4"	"00075b9048"	-0.0765
"0007500ee4"	"00075d0801"	0.2642
"0007500ee4"	"00075d1820"	0.3289
"0007500ee4"	"00075d1f3d"	-0.2783
"0007500ee4"	"00075d2329"	0.112
"0007500ee4"	"00075d2b9b"	-0.4561
"0007500ee4"	"00075d3941"	-0.079
"0007500ee4"	"00075d3e96"	0.3265
"0007500ee4"	"00075d4864"	-0.0933
"0007500ee4"	"00075d5961"	0.1404
"0007500ee4"	"00075d5a63"	-0.0327
"0007500ee4"	"00075d6150"	0.0338
"0007500ee4"	"00075d67d0"	-0.2977
"0007500ee4"	"00075d67e2"	-0.1781
"0007500ee4"	"00075d73fc"	-0.0236
"0007500ee4"	"00075d7729"	-0.0932
"0007500ee4"	"00075d778c"	0.0365
"0007500ee4"	"00075d7b9e"	0.2957
"0007500ee4"	"00075d7c8f"	-0.0899
"0007500ee4"	"00075d804d"	0.1124
"0007500ee4"	"00075d819f"	-0.0849
"0007500ee4"	"00075d8601"	0.1273
"0007500ee4"	"00075d8c6a"	-0.0152
"0007500ee4"	"00075dfedc"	0.0207

"0007500ee4"	"00075e05f2"	-0.2133
"0007500ee4"	"00075e0837"	-0.0943
"0007500ee4"	"00075e092e"	0.0739
"0007500ee4"	"00075e0965"	-0.2763
"0007500ee4"	"00075e0bc8"	-0.0325
"0007500ee4"	"00075e0fbb"	-0.1308
"0007500eee"	"00075013dc"	-0.4798
"0007500eee"	"000757b515"	-0.0274
"0007500eee"	"000757bc5a"	0.1286
"0007500eee"	"000757c320"	-0.1627
"0007500eee"	"000757c9aa"	-0.0392
"0007500eee"	"000757ccbe"	-0.4327
"0007500eee"	"000757cfa9"	-0.4482
"0007500eee"	"000757d390"	-0.4942
"0007500eee"	"000757d393"	0.0895
"0007500eee"	"000757d598"	-0.4996
"0007500eee"	"000757d5a2"	-0.4512
"0007500eee"	"000757d790"	0.006
"0007500eee"	"000757e30c"	-0.2302
"0007500eee"	"000757e4b0"	0.1214
"0007500eee"	"000757e7a0"	-0.1804
"0007500eee"	"000757e8b3"	-0.1168
"0007500eee"	"000757f627"	-0.1104
"0007500eee"	"000757f925"	0.0511
"0007500eee"	"000757fa08"	-0.1025
"0007500eee"	"000757fe52"	-0.1444
"0007500eee"	"000758024a"	-0.407
"0007500eee"	"00075804bb"	-0.0896
"0007500eee"	"00075a0c04"	-0.0376
"0007500eee"	"00075a3110"	-0.2826
"0007500eee"	"00075a341a"	-0.3776
"0007500eee"	"00075a3dcf"	0.0571
"0007500eee"	"00075a3e22"	-0.18
"0007500eee"	"00075a48d8"	-0.1071
"0007500eee"	"00075a5cfb"	-0.3628
"0007500eee"	"00075a6151"	-0.0421
"0007500eee"	"00075a6708"	-0.2803
"0007500eee"	"00075a7319"	-0.5076
"0007500eee"	"00075a7723"	-0.1059
"0007500eee"	"00075a778b"	-0.4752
"0007500eee"	"00075a7b8e"	0.1079
"0007500eee"	"00075a7c79"	-0.1912
"0007500eee"	"00075a81b6"	-0.1175
"0007500eee"	"00075a82ac"	-0.0025
"0007500eee"	"00075a98e5"	-0.2642
"0007500eee"	"00075b0d29"	-0.3351
"0007500eee"	"00075b102a"	0.2522
"0007500eee"	"00075b1074"	-0.2898
"0007500eee"	"00075b135d"	-0.5118
"0007500eee"	"00075b138b"	0.1457

"0007500eee"	"00075b13a0"	−0.3187
"0007500eee"	"00075b13bd"	−0.2054
"0007500eee"	"00075b16a9"	0.0846
"0007500eee"	"00075b1a28"	−0.1246
"0007500eee"	"00075b1a97"	−0.1976
"0007500eee"	"00075b1c7b"	−0.2529
"0007500eee"	"00075b1d24"	0.0557
"0007500eee"	"00075b202b"	0.1066
"0007500eee"	"00075b22cb"	0.0022
"0007500eee"	"00075b22da"	−0.24
"0007500eee"	"00075b2556"	−0.1722
"0007500eee"	"00075b25de"	0.2214
"0007500eee"	"00075b260c"	−0.1472
"0007500eee"	"00075b26f1"	0.0298
"0007500eee"	"00075b2920"	−0.1109
"0007500eee"	"00075b2a64"	−0.1419
"0007500eee"	"00075b2a9d"	0.0643
"0007500eee"	"00075b2b37"	−0.203
"0007500eee"	"00075b2cdd"	−0.3293
"0007500eee"	"00075b3038"	0.1161
"0007500eee"	"00075b30fe"	−0.0968
"0007500eee"	"00075b3362"	−0.2082
"0007500eee"	"00075b350a"	−0.2105
"0007500eee"	"00075b350e"	−0.2695
"0007500eee"	"00075b3651"	0.0346
"0007500eee"	"00075b38ca"	−0.0297
"0007500eee"	"00075b39cc"	−0.0414
"0007500eee"	"00075b3e1e"	0.2351
"0007500eee"	"00075b3e57"	−0.0458
"0007500eee"	"00075b4079"	−0.1852
"0007500eee"	"00075b4150"	−0.2967
"0007500eee"	"00075b4194"	−0.1688
"0007500eee"	"00075b42d5"	0.0556
"0007500eee"	"00075b4424"	−0.3675
"0007500eee"	"00075b4470"	0.1979
"0007500eee"	"00075b47ed"	−0.098
"0007500eee"	"00075b4850"	0.1003
"0007500eee"	"00075b4ca0"	−0.4406
"0007500eee"	"00075b4d7f"	0.1091
"0007500eee"	"00075b520f"	−0.0181
"0007500eee"	"00075b525f"	−0.4412
"0007500eee"	"00075b58f8"	−0.0707
"0007500eee"	"00075b5bcc"	0.086
"0007500eee"	"00075b5bfa"	0.0781
"0007500eee"	"00075b6339"	−0.1615
"0007500eee"	"00075b6658"	−0.059
"0007500eee"	"00075b679a"	−0.0706
"0007500eee"	"00075b6cb7"	0.079
"0007500eee"	"00075b6df8"	−0.1294
"0007500eee"	"00075b6ff6"	−0.2016

"0007500eee"	"00075b70ee"	−0.362
"0007500eee"	"00075b7157"	0.0335
"0007500eee"	"00075b7225"	−0.0804
"0007500eee"	"00075b7c89"	−0.3806
"0007500eee"	"00075b9048"	−0.1254
"0007500eee"	"00075d0801"	0.0864
"0007500eee"	"00075d1820"	−0.0376
"0007500eee"	"00075d1f3d"	−0.0522
"0007500eee"	"00075d2329"	0.1043
"0007500eee"	"00075d2b9b"	−0.0967
"0007500eee"	"00075d3941"	−0.0557
"0007500eee"	"00075d3e96"	−0.0279
"0007500eee"	"00075d4864"	−0.0437
"0007500eee"	"00075d5961"	0.0637
"0007500eee"	"00075d5a63"	0.1371
"0007500eee"	"00075d6150"	−0.2053
"0007500eee"	"00075d67d0"	−0.0578
"0007500eee"	"00075d67e2"	−0.247
"0007500eee"	"00075d73fc"	−0.1227
"0007500eee"	"00075d7729"	−0.4129
"0007500eee"	"00075d778c"	−0.1166
"0007500eee"	"00075d7b9e"	0.1244
"0007500eee"	"00075d7c8f"	−0.2314
"0007500eee"	"00075d804d"	0.1327
"0007500eee"	"00075d819f"	0.1157
"0007500eee"	"00075d8601"	−0.0057
"0007500eee"	"00075d8c6a"	−0.2884
"0007500eee"	"00075dfedc"	−0.0272
"0007500eee"	"00075e05f2"	0.0792
"0007500eee"	"00075e0837"	0.2995
"0007500eee"	"00075e092e"	−0.2617
"0007500eee"	"00075e0965"	−0.3841
"0007500eee"	"00075e0bc8"	0.0875
"0007500eee"	"00075e0fbb"	−0.2784
"00075013dc"	"000757b515"	−0.1065
"00075013dc"	"000757bc5a"	0.044
"00075013dc"	"000757c320"	−0.2013
"00075013dc"	"000757c9aa"	−0.0422
"00075013dc"	"000757ccbe"	0.0664
"00075013dc"	"000757cfa9"	−0.1864
"00075013dc"	"000757d390"	−0.0693
"00075013dc"	"000757d393"	−0.2009
"00075013dc"	"000757d598"	0.0113
"00075013dc"	"000757d5a2"	−0.067
"00075013dc"	"000757d790"	−0.2456
"00075013dc"	"000757e30c"	−0.0099
"00075013dc"	"000757e4b0"	0.0805
"00075013dc"	"000757e7a0"	−0.2907
"00075013dc"	"000757e8b3"	−0.0708
"00075013dc"	"000757f627"	0.1778

"00075013dc"	"000757f925"	−0.0986
"00075013dc"	"000757fa08"	−0.2786
"00075013dc"	"000757fe52"	−0.3208
"00075013dc"	"000758024a"	−0.2044
"00075013dc"	"00075804bb"	0.126
"00075013dc"	"00075a0c04"	−0.2815
"00075013dc"	"00075a3110"	0.0182
"00075013dc"	"00075a341a"	−0.3701
"00075013dc"	"00075a3dcf"	−0.4687
"00075013dc"	"00075a3e22"	−0.0225
"00075013dc"	"00075a48d8"	−0.3389
"00075013dc"	"00075a5cfb"	0.0938
"00075013dc"	"00075a6151"	−0.0877
"00075013dc"	"00075a6708"	−0.158
"00075013dc"	"00075a7319"	−0.0295
"00075013dc"	"00075a7723"	−0.1622
"00075013dc"	"00075a778b"	−0.2249
"00075013dc"	"00075a7b8e"	−0.0698
"00075013dc"	"00075a7c79"	0.0843
"00075013dc"	"00075a81b6"	−0.2367
"00075013dc"	"00075a82ac"	−0.1049
"00075013dc"	"00075a98e5"	−0.1011
"00075013dc"	"00075b0d29"	−0.1384
"00075013dc"	"00075b102a"	−0.1662
"00075013dc"	"00075b1074"	−0.4325
"00075013dc"	"00075b135d"	−0.071
"00075013dc"	"00075b138b"	−0.0021
"00075013dc"	"00075b13a0"	−0.3711
"00075013dc"	"00075b13bd"	−0.2682
"00075013dc"	"00075b16a9"	−0.6914
"00075013dc"	"00075b1a28"	−0.4259
"00075013dc"	"00075b1a97"	−0.2725
"00075013dc"	"00075b1c7b"	−0.7856
"00075013dc"	"00075b1d24"	−0.2413
"00075013dc"	"00075b202b"	0.0054
"00075013dc"	"00075b22cb"	−0.6243
"00075013dc"	"00075b22da"	−0.4273
"00075013dc"	"00075b2556"	−0.4059
"00075013dc"	"00075b25de"	−0.148
"00075013dc"	"00075b260c"	0.0828
"00075013dc"	"00075b26f1"	−0.3404
"00075013dc"	"00075b2920"	0.2949
"00075013dc"	"00075b2a64"	0.0216
"00075013dc"	"00075b2a9d"	0.091
"00075013dc"	"00075b2b37"	−0.4296
"00075013dc"	"00075b2cdd"	−0.3746
"00075013dc"	"00075b3038"	−0.1043
"00075013dc"	"00075b30fe"	−0.3472
"00075013dc"	"00075b3362"	−0.3839
"00075013dc"	"00075b350a"	0.1093

"00075013dc"	"00075b350e"	0.0454
"00075013dc"	"00075b3651"	−0.3234
"00075013dc"	"00075b38ca"	−0.4326
"00075013dc"	"00075b39cc"	−0.202
"00075013dc"	"00075b3e1e"	−0.2903
"00075013dc"	"00075b3e57"	−0.3297
"00075013dc"	"00075b4079"	−0.1156
"00075013dc"	"00075b4150"	−0.0564
"00075013dc"	"00075b4194"	0.2525
"00075013dc"	"00075b42d5"	−0.1645
"00075013dc"	"00075b4424"	−0.0816
"00075013dc"	"00075b4470"	0.0283
"00075013dc"	"00075b47ed"	−0.1774
"00075013dc"	"00075b4850"	0.2772
"00075013dc"	"00075b4ca0"	−0.0845
"00075013dc"	"00075b4d7f"	−0.0884
"00075013dc"	"00075b520f"	0.0287
"00075013dc"	"00075b525f"	−0.2981
"00075013dc"	"00075b58f8"	−0.3032
"00075013dc"	"00075b5bcc"	−0.0877
"00075013dc"	"00075b5bfa"	−0.5199
"00075013dc"	"00075b6339"	−0.1383
"00075013dc"	"00075b6658"	−0.5041
"00075013dc"	"00075b679a"	−0.0224
"00075013dc"	"00075b6cb7"	−0.1794
"00075013dc"	"00075b6df8"	−0.4957
"00075013dc"	"00075b6ff6"	0.0628
"00075013dc"	"00075b70ee"	−0.1617
"00075013dc"	"00075b7157"	−0.4469
"00075013dc"	"00075b7225"	−0.2389
"00075013dc"	"00075b7c89"	−0.0987
"00075013dc"	"00075b9048"	0.1046
"00075013dc"	"00075d0801"	0.0065
"00075013dc"	"00075d1820"	−0.6367
"00075013dc"	"00075d1f3d"	0.2422
"00075013dc"	"00075d2329"	−0.321
"00075013dc"	"00075d2b9b"	0.1901
"00075013dc"	"00075d3941"	−0.0516
"00075013dc"	"00075d3e96"	−0.2136
"00075013dc"	"00075d4864"	−0.3248
"00075013dc"	"00075d5961"	−0.2885
"00075013dc"	"00075d5a63"	0.0647
"00075013dc"	"00075d6150"	0.0235
"00075013dc"	"00075d67d0"	−0.3674
"00075013dc"	"00075d67e2"	−0.3043
"00075013dc"	"00075d73fc"	−0.4665
"00075013dc"	"00075d7729"	−0.2481
"00075013dc"	"00075d778c"	−0.5852
"00075013dc"	"00075d7b9e"	−0.2836
"00075013dc"	"00075d7c8f"	−0.2282

"00075013dc"	"00075d804d"	0.0292
"00075013dc"	"00075d819f"	−0.0599
"00075013dc"	"00075d8601"	−0.2916
"00075013dc"	"00075d8c6a"	−0.3121
"00075013dc"	"00075dfedc"	−0.0917
"00075013dc"	"00075e05f2"	−0.1005
"00075013dc"	"00075e0837"	0.0453
"00075013dc"	"00075e092e"	−0.4269
"00075013dc"	"00075e0965"	−0.2923
"00075013dc"	"00075e0bc8"	−0.0064
"00075013dc"	"00075e0fbb"	−0.6072
"000757b515"	"000757bc5a"	0.0238
"000757b515"	"000757c320"	−0.1463
"000757b515"	"000757c9aa"	0.3578
"000757b515"	"000757ccbe"	−0.0049
"000757b515"	"000757cfa9"	−0.0855
"000757b515"	"000757d390"	0.0275
"000757b515"	"000757d393"	0.1265
"000757b515"	"000757d598"	0.2696
"000757b515"	"000757d5a2"	0.3915
"000757b515"	"000757d790"	0.2083
"000757b515"	"000757e30c"	0.1854
"000757b515"	"000757e4b0"	0.3029
"000757b515"	"000757e7a0"	0.2361
"000757b515"	"000757e8b3"	0.1372
"000757b515"	"000757f627"	−0.0112
"000757b515"	"000757f925"	0.3072
"000757b515"	"000757fa08"	−0.1415
"000757b515"	"000757fe52"	−0.1014
"000757b515"	"000758024a"	−0.0851
"000757b515"	"00075804bb"	−0.0212
"000757b515"	"00075a0c04"	0.0928
"000757b515"	"00075a3110"	0.3892
"000757b515"	"00075a341a"	−0.0204
"000757b515"	"00075a3dcf"	0.0408
"000757b515"	"00075a3e22"	0.2084
"000757b515"	"00075a48d8"	−0.0791
"000757b515"	"00075a5cfb"	−0.219
"000757b515"	"00075a6151"	0.331
"000757b515"	"00075a6708"	0.3016
"000757b515"	"00075a7319"	0.0075
"000757b515"	"00075a7723"	0.0996
"000757b515"	"00075a778b"	−0.2547
"000757b515"	"00075a7b8e"	0.2563
"000757b515"	"00075a7c79"	0.4593
"000757b515"	"00075a81b6"	0.0161
"000757b515"	"00075a82ac"	0.2144
"000757b515"	"00075a98e5"	−0.3681
"000757b515"	"00075b0d29"	−0.0924
"000757b515"	"00075b102a"	0.1729

"000757b515"	"00075b1074"	0.0665
"000757b515"	"00075b135d"	0.057
"000757b515"	"00075b138b"	0.1758
"000757b515"	"00075b13a0"	-0.2516
"000757b515"	"00075b13bd"	-0.1015
"000757b515"	"00075b16a9"	0.0331
"000757b515"	"00075b1a28"	-0.3715
"000757b515"	"00075b1a97"	-0.2726
"000757b515"	"00075b1c7b"	-0.5655
"000757b515"	"00075b1d24"	0.0931
"000757b515"	"00075b202b"	0.1881
"000757b515"	"00075b22cb"	-0.3159
"000757b515"	"00075b22da"	0.0802
"000757b515"	"00075b2556"	0.144
"000757b515"	"00075b25de"	0.357
"000757b515"	"00075b260c"	0.3495
"000757b515"	"00075b26f1"	-0.0924
"000757b515"	"00075b2920"	0.2401
"000757b515"	"00075b2a64"	0.1628
"000757b515"	"00075b2a9d"	0.3321
"000757b515"	"00075b2b37"	-0.2548
"000757b515"	"00075b2cdd"	-0.2105
"000757b515"	"00075b3038"	0.3259
"000757b515"	"00075b30fe"	0.2483
"000757b515"	"00075b3362"	-0.1427
"000757b515"	"00075b350a"	-0.0947
"000757b515"	"00075b350e"	-0.1305
"000757b515"	"00075b3651"	0.034
"000757b515"	"00075b38ca"	-0.3549
"000757b515"	"00075b39cc"	0.2033
"000757b515"	"00075b3e1e"	-0.2834
"000757b515"	"00075b3e57"	-0.1368
"000757b515"	"00075b4079"	-0.0485
"000757b515"	"00075b4150"	0.0806
"000757b515"	"00075b4194"	0.1995
"000757b515"	"00075b42d5"	-0.1313
"000757b515"	"00075b4424"	-0.137
"000757b515"	"00075b4470"	0.0238
"000757b515"	"00075b47ed"	0.0531
"000757b515"	"00075b4850"	0.1693
"000757b515"	"00075b4ca0"	0.5397
"000757b515"	"00075b4d7f"	0.1413
"000757b515"	"00075b520f"	-0.0146
"000757b515"	"00075b525f"	0.0402
"000757b515"	"00075b58f8"	-0.0526
"000757b515"	"00075b5bcc"	0.1023
"000757b515"	"00075b5bfa"	0.1452
"000757b515"	"00075b6339"	0.3323
"000757b515"	"00075b6658"	-0.113
"000757b515"	"00075b679a"	0.0329

"000757b515"	"00075b6cb7"	0.1444
"000757b515"	"00075b6df8"	0.0471
"000757b515"	"00075b6ff6"	0.3496
"000757b515"	"00075b70ee"	0.0425
"000757b515"	"00075b7157"	0.2013
"000757b515"	"00075b7225"	-0.0203
"000757b515"	"00075b7c89"	0.4053
"000757b515"	"00075b9048"	0.3325
"000757b515"	"00075d0801"	0.2109
"000757b515"	"00075d1820"	-0.0692
"000757b515"	"00075d1f3d"	0.1491
"000757b515"	"00075d2329"	0.0518
"000757b515"	"00075d2b9b"	0.1147
"000757b515"	"00075d3941"	-0.2376
"000757b515"	"00075d3e96"	0.194
"000757b515"	"00075d4864"	-0.2311
"000757b515"	"00075d5961"	-0.0359
"000757b515"	"00075d5a63"	0.0975
"000757b515"	"00075d6150"	-0.2514
"000757b515"	"00075d67d0"	0.1966
"000757b515"	"00075d67e2"	-0.1504
"000757b515"	"00075d73fc"	-0.2363
"000757b515"	"00075d7729"	0.1299
"000757b515"	"00075d778c"	-0.1042
"000757b515"	"00075d7b9e"	0.083
"000757b515"	"00075d7c8f"	0.3051
"000757b515"	"00075d804d"	0.1664
"000757b515"	"00075d819f"	0.2412
"000757b515"	"00075d8601"	-0.1421
"000757b515"	"00075d8c6a"	0.0654
"000757b515"	"00075dfedc"	0.1892
"000757b515"	"00075e05f2"	0.0526
"000757b515"	"00075e0837"	0.2185
"000757b515"	"00075e092e"	0.019
"000757b515"	"00075e0965"	-0.2445
"000757b515"	"00075e0bc8"	0.3317
"000757b515"	"00075e0fbb"	-0.0075
"000757bc5a"	"000757c320"	0.0609
"000757bc5a"	"000757c9aa"	0.0762
"000757bc5a"	"000757ccbe"	0.0196
"000757bc5a"	"000757cfa9"	0.0609
"000757bc5a"	"000757d390"	0.0182
"000757bc5a"	"000757d393"	0.0774
"000757bc5a"	"000757d598"	-0.1799
"000757bc5a"	"000757d5a2"	-0.2207
"000757bc5a"	"000757d790"	-0.0103
"000757bc5a"	"000757e30c"	0.3233
"000757bc5a"	"000757e4b0"	0.4569
"000757bc5a"	"000757e7a0"	-0.0295
"000757bc5a"	"000757e8b3"	0.2745

"000757bc5a"	"000757f627"	0.2545
"000757bc5a"	"000757f925"	0.045
"000757bc5a"	"000757fa08"	−0.0674
"000757bc5a"	"000757fe52"	−0.0478
"000757bc5a"	"000758024a"	0.0737
"000757bc5a"	"00075804bb"	0.3221
"000757bc5a"	"00075a0c04"	0.0486
"000757bc5a"	"00075a3110"	0.0779
"000757bc5a"	"00075a341a"	−0.2294
"000757bc5a"	"00075a3dcf"	0.0113
"000757bc5a"	"00075a3e22"	0.3188
"000757bc5a"	"00075a48d8"	−0.051
"000757bc5a"	"00075a5cfb"	0.2249
"000757bc5a"	"00075a6151"	−0.0773
"000757bc5a"	"00075a6708"	0.1386
"000757bc5a"	"00075a7319"	−0.0332
"000757bc5a"	"00075a7723"	0.1051
"000757bc5a"	"00075a778b"	0.149
"000757bc5a"	"00075a7b8e"	0.3479
"000757bc5a"	"00075a7c79"	0.3375
"000757bc5a"	"00075a81b6"	−0.0538
"000757bc5a"	"00075a82ac"	0.3517
"000757bc5a"	"00075a98e5"	0.1297
"000757bc5a"	"00075b0d29"	0.1073
"000757bc5a"	"00075b102a"	0.2676
"000757bc5a"	"00075b1074"	0.1299
"000757bc5a"	"00075b135d"	0.0409
"000757bc5a"	"00075b138b"	0.4952
"000757bc5a"	"00075b13a0"	−0.1782
"000757bc5a"	"00075b13bd"	0.1501
"000757bc5a"	"00075b16a9"	−0.0267
"000757bc5a"	"00075b1a28"	0.079
"000757bc5a"	"00075b1a97"	0.0579
"000757bc5a"	"00075b1c7b"	−0.1903
"000757bc5a"	"00075b1d24"	0.1333
"000757bc5a"	"00075b202b"	0.3545
"000757bc5a"	"00075b22cb"	0.0759
"000757bc5a"	"00075b22da"	0.0599
"000757bc5a"	"00075b2556"	0.0709
"000757bc5a"	"00075b25de"	0.2288
"000757bc5a"	"00075b260c"	−0.1555
"000757bc5a"	"00075b26f1"	−0.0678
"000757bc5a"	"00075b2920"	0.3356
"000757bc5a"	"00075b2a64"	0.1327
"000757bc5a"	"00075b2a9d"	0.1078
"000757bc5a"	"00075b2b37"	−0.2645
"000757bc5a"	"00075b2cdd"	0.0525
"000757bc5a"	"00075b3038"	0.0807
"000757bc5a"	"00075b30fe"	0.057
"000757bc5a"	"00075b3362"	−0.1759

"000757bc5a"	"00075b350a"	0.1995
"000757bc5a"	"00075b350e"	0.0132
"000757bc5a"	"00075b3651"	−0.0861
"000757bc5a"	"00075b38ca"	0.0472
"000757bc5a"	"00075b39cc"	−0.0043
"000757bc5a"	"00075b3e1e"	0.0704
"000757bc5a"	"00075b3e57"	0.0509
"000757bc5a"	"00075b4079"	−0.0016
"000757bc5a"	"00075b4150"	0.0022
"000757bc5a"	"00075b4194"	0.3299
"000757bc5a"	"00075b42d5"	0.2117
"000757bc5a"	"00075b4424"	−0.0685
"000757bc5a"	"00075b4470"	0.9659
"000757bc5a"	"00075b47ed"	0.0241
"000757bc5a"	"00075b4850"	0.7618
"000757bc5a"	"00075b4ca0"	−0.0293
"000757bc5a"	"00075b4d7f"	0.2588
"000757bc5a"	"00075b520f"	−0.0568
"000757bc5a"	"00075b525f"	−0.2108
"000757bc5a"	"00075b58f8"	0.3129
"000757bc5a"	"00075b5bcc"	0.1015
"000757bc5a"	"00075b5bfa"	−0.2676
"000757bc5a"	"00075b6339"	0.0963
"000757bc5a"	"00075b6658"	−0.163
"000757bc5a"	"00075b679a"	0.088
"000757bc5a"	"00075b6cb7"	0.3759
"000757bc5a"	"00075b6df8"	−0.1317
"000757bc5a"	"00075b6ff6"	0.1147
"000757bc5a"	"00075b70ee"	0.0871
"000757bc5a"	"00075b7157"	0.0997
"000757bc5a"	"00075b7225"	0.0715
"000757bc5a"	"00075b7c89"	0.1653
"000757bc5a"	"00075b9048"	0.0845
"000757bc5a"	"00075d0801"	0.1594
"000757bc5a"	"00075d1820"	−0.1505
"000757bc5a"	"00075d1f3d"	0.3831
"000757bc5a"	"00075d2329"	0.0493
"000757bc5a"	"00075d2b9b"	0.0193
"000757bc5a"	"00075d3941"	0.202
"000757bc5a"	"00075d3e96"	0.2063
"000757bc5a"	"00075d4864"	0.1005
"000757bc5a"	"00075d5961"	0.0252
"000757bc5a"	"00075d5a63"	0.1696
"000757bc5a"	"00075d6150"	−0.1362
"000757bc5a"	"00075d67d0"	−0.3531
"000757bc5a"	"00075d67e2"	0.1137
"000757bc5a"	"00075d73fc"	0.1645
"000757bc5a"	"00075d7729"	−0.2895
"000757bc5a"	"00075d778c"	−0.4042
"000757bc5a"	"00075d7b9e"	0.0244

"000757bc5a"	"00075d7c8f"	0.096
"000757bc5a"	"00075d804d"	0.3433
"000757bc5a"	"00075d819f"	0.2573
"000757bc5a"	"00075d8601"	-0.0226
"000757bc5a"	"00075d8c6a"	-0.0459
"000757bc5a"	"00075dfedc"	0.0511
"000757bc5a"	"00075e05f2"	0.064
"000757bc5a"	"00075e0837"	0.2558
"000757bc5a"	"00075e092e"	0.15
"000757bc5a"	"00075e0965"	0.0104
"000757bc5a"	"00075e0bc8"	0.5051
"000757bc5a"	"00075e0fbb"	0.0017
"000757c320"	"000757c9aa"	0.3004
"000757c320"	"000757ccbe"	-0.1477
"000757c320"	"000757cfa9"	0.0727
"000757c320"	"000757d390"	0.0569
"000757c320"	"000757d393"	0.1706
"000757c320"	"000757d598"	0.053
"000757c320"	"000757d5a2"	-0.2417
"000757c320"	"000757d790"	0.0616
"000757c320"	"000757e30c"	0.074
"000757c320"	"000757e4b0"	-0.1735
"000757c320"	"000757e7a0"	-0.3338
"000757c320"	"000757e8b3"	0.0577
"000757c320"	"000757f627"	0.311
"000757c320"	"000757f925"	0.2119
"000757c320"	"000757fa08"	-0.6413
"000757c320"	"000757fe52"	-0.0749
"000757c320"	"000758024a"	-0.1056
"000757c320"	"00075804bb"	0.2863
"000757c320"	"00075a0c04"	-0.3728
"000757c320"	"00075a3110"	-0.1294
"000757c320"	"00075a341a"	-0.2788
"000757c320"	"00075a3dcf"	-0.2698
"000757c320"	"00075a3e22"	-0.0175
"000757c320"	"00075a48d8"	-0.1937
"000757c320"	"00075a5cfb"	-0.118
"000757c320"	"00075a6151"	0.0586
"000757c320"	"00075a6708"	-0.155
"000757c320"	"00075a7319"	0.1742
"000757c320"	"00075a7723"	-0.0566
"000757c320"	"00075a778b"	-0.2145
"000757c320"	"00075a7b8e"	-0.1671
"000757c320"	"00075a7c79"	-0.0961
"000757c320"	"00075a81b6"	-0.1754
"000757c320"	"00075a82ac"	-0.1587
"000757c320"	"00075a98e5"	0.1388
"000757c320"	"00075b0d29"	0.0201
"000757c320"	"00075b102a"	0.0608
"000757c320"	"00075b1074"	-0.5017

"000757c320"	"00075b135d"	-0.3278
"000757c320"	"00075b138b"	0.0602
"000757c320"	"00075b13a0"	-0.2443
"000757c320"	"00075b13bd"	-0.0821
"000757c320"	"00075b16a9"	0.0175
"000757c320"	"00075b1a28"	-0.0332
"000757c320"	"00075b1a97"	-0.0138
"000757c320"	"00075b1c7b"	-0.1003
"000757c320"	"00075b1d24"	-0.2041
"000757c320"	"00075b202b"	-0.1339
"000757c320"	"00075b22cb"	-0.0081
"000757c320"	"00075b22da"	-0.2661
"000757c320"	"00075b2556"	-0.0797
"000757c320"	"00075b25de"	0.0703
"000757c320"	"00075b260c"	0.0561
"000757c320"	"00075b26f1"	-0.0755
"000757c320"	"00075b2920"	0.1991
"000757c320"	"00075b2a64"	-0.0557
"000757c320"	"00075b2a9d"	-0.1987
"000757c320"	"00075b2b37"	-0.1961
"000757c320"	"00075b2cdd"	-0.1482
"000757c320"	"00075b3038"	0.0331
"000757c320"	"00075b30fe"	-0.2457
"000757c320"	"00075b3362"	-0.2417
"000757c320"	"00075b350a"	-0.0052
"000757c320"	"00075b350e"	-0.1611
"000757c320"	"00075b3651"	-0.1962
"000757c320"	"00075b38ca"	-0.3383
"000757c320"	"00075b39cc"	3e-04
"000757c320"	"00075b3e1e"	0.0917
"000757c320"	"00075b3e57"	0.0832
"000757c320"	"00075b4079"	0.128
"000757c320"	"00075b4150"	-0.1527
"000757c320"	"00075b4194"	0.0716
"000757c320"	"00075b42d5"	-0.3013
"000757c320"	"00075b4424"	-0.1887
"000757c320"	"00075b4470"	0.0609
"000757c320"	"00075b47ed"	-0.185
"000757c320"	"00075b4850"	0.0888
"000757c320"	"00075b4ca0"	-0.2992
"000757c320"	"00075b4d7f"	-0.2916
"000757c320"	"00075b520f"	-0.1299
"000757c320"	"00075b525f"	-0.0489
"000757c320"	"00075b58f8"	-0.2302
"000757c320"	"00075b5bcc"	0.141
"000757c320"	"00075b5bfa"	-0.2337
"000757c320"	"00075b6339"	0.1054
"000757c320"	"00075b6658"	-0.1965
"000757c320"	"00075b679a"	0.0226
"000757c320"	"00075b6cb7"	-0.0373

"000757c320"	"00075b6df8"	-0.3728
"000757c320"	"00075b6ff6"	-0.0756
"000757c320"	"00075b70ee"	-0.2824
"000757c320"	"00075b7157"	-0.1614
"000757c320"	"00075b7225"	-0.2936
"000757c320"	"00075b7c89"	-0.1147
"000757c320"	"00075b9048"	-0.0289
"000757c320"	"00075d0801"	-0.0414
"000757c320"	"00075d1820"	-0.1306
"000757c320"	"00075d1f3d"	-0.1743
"000757c320"	"00075d2329"	-0.2705
"000757c320"	"00075d2b9b"	0.0589
"000757c320"	"00075d3941"	-0.1955
"000757c320"	"00075d3e96"	-0.2165
"000757c320"	"00075d4864"	-0.1538
"000757c320"	"00075d5961"	-0.1618
"000757c320"	"00075d5a63"	0.0193
"000757c320"	"00075d6150"	-0.168
"000757c320"	"00075d67d0"	-0.1922
"000757c320"	"00075d67e2"	0.0618
"000757c320"	"00075d73fc"	-0.2192
"000757c320"	"00075d7729"	-0.3784
"000757c320"	"00075d778c"	-0.2648
"000757c320"	"00075d7b9e"	-0.0147
"000757c320"	"00075d7c8f"	-0.1608
"000757c320"	"00075d804d"	0.1722
"000757c320"	"00075d819f"	0.0921
"000757c320"	"00075d8601"	0.276
"000757c320"	"00075d8c6a"	-0.2215
"000757c320"	"00075dfedc"	0.1091
"000757c320"	"00075e05f2"	0.0162
"000757c320"	"00075e0837"	-0.0727
"000757c320"	"00075e092e"	-0.2875
"000757c320"	"00075e0965"	-0.2401
"000757c320"	"00075e0bc8"	-0.1399
"000757c320"	"00075e0fbb"	-0.3641
"000757c9aa"	"000757ccbe"	0.0065
"000757c9aa"	"000757cfa9"	-0.1198
"000757c9aa"	"000757d390"	-0.0481
"000757c9aa"	"000757d393"	0.0237
"000757c9aa"	"000757d598"	0.2288
"000757c9aa"	"000757d5a2"	0.0734
"000757c9aa"	"000757d790"	-0.0022
"000757c9aa"	"000757e30c"	0.2188
"000757c9aa"	"000757e4b0"	0.326
"000757c9aa"	"000757e7a0"	-0.0148
"000757c9aa"	"000757e8b3"	0.2755
"000757c9aa"	"000757f627"	0.1966
"000757c9aa"	"000757f925"	0.2176
"000757c9aa"	"000757fa08"	-0.4551

"000757c9aa"	"000757fe52"	-0.1787
"000757c9aa"	"000758024a"	0.0796
"000757c9aa"	"00075804bb"	0.0801
"000757c9aa"	"00075a0c04"	-0.0979
"000757c9aa"	"00075a3110"	0.0093
"000757c9aa"	"00075a341a"	-0.2643
"000757c9aa"	"00075a3dcf"	-0.2079
"000757c9aa"	"00075a3e22"	0.133
"000757c9aa"	"00075a48d8"	-0.1015
"000757c9aa"	"00075a5cfb"	0.0768
"000757c9aa"	"00075a6151"	0.2764
"000757c9aa"	"00075a6708"	0.0359
"000757c9aa"	"00075a7319"	0.1487
"000757c9aa"	"00075a7723"	0.0437
"000757c9aa"	"00075a778b"	-0.3504
"000757c9aa"	"00075a7b8e"	0.3491
"000757c9aa"	"00075a7c79"	0.0145
"000757c9aa"	"00075a81b6"	0.1106
"000757c9aa"	"00075a82ac"	-0.2246
"000757c9aa"	"00075a98e5"	-0.2246
"000757c9aa"	"00075b0d29"	-0.1138
"000757c9aa"	"00075b102a"	0.0289
"000757c9aa"	"00075b1074"	-0.3295
"000757c9aa"	"00075b135d"	-0.1091
"000757c9aa"	"00075b138b"	0.2138
"000757c9aa"	"00075b13a0"	-0.1512
"000757c9aa"	"00075b13bd"	-0.0479
"000757c9aa"	"00075b16a9"	-0.1053
"000757c9aa"	"00075b1a28"	-0.4613
"000757c9aa"	"00075b1a97"	-0.1551
"000757c9aa"	"00075b1c7b"	-0.4156
"000757c9aa"	"00075b1d24"	0.0596
"000757c9aa"	"00075b202b"	0.1748
"000757c9aa"	"00075b22cb"	-0.3343
"000757c9aa"	"00075b22da"	-0.1955
"000757c9aa"	"00075b2556"	-0.1746
"000757c9aa"	"00075b25de"	0.0857
"000757c9aa"	"00075b260c"	0.2125
"000757c9aa"	"00075b26f1"	0.1662
"000757c9aa"	"00075b2920"	0.2317
"000757c9aa"	"00075b2a64"	0.2627
"000757c9aa"	"00075b2a9d"	0.2572
"000757c9aa"	"00075b2b37"	-0.2544
"000757c9aa"	"00075b2cdd"	-0.2558
"000757c9aa"	"00075b3038"	0.0957
"000757c9aa"	"00075b30fe"	-0.3331
"000757c9aa"	"00075b3362"	0.0201
"000757c9aa"	"00075b350a"	0.0594
"000757c9aa"	"00075b350e"	-0.2331
"000757c9aa"	"00075b3651"	-0.0655

"000757c9aa"	"00075b38ca"	-0.4251
"000757c9aa"	"00075b39cc"	0.122
"000757c9aa"	"00075b3e1e"	0.0162
"000757c9aa"	"00075b3e57"	-0.067
"000757c9aa"	"00075b4079"	0.1544
"000757c9aa"	"00075b4150"	0.081
"000757c9aa"	"00075b4194"	0.3807
"000757c9aa"	"00075b42d5"	-0.3186
"000757c9aa"	"00075b4424"	-0.1472
"000757c9aa"	"00075b4470"	0.0762
"000757c9aa"	"00075b47ed"	-0.0066
"000757c9aa"	"00075b4850"	0.2897
"000757c9aa"	"00075b4ca0"	0.1811
"000757c9aa"	"00075b4d7f"	-0.1909
"000757c9aa"	"00075b520f"	0.0416
"000757c9aa"	"00075b525f"	0.0948
"000757c9aa"	"00075b58f8"	-0.2289
"000757c9aa"	"00075b5bcc"	0.2096
"000757c9aa"	"00075b5bfa"	-0.1087
"000757c9aa"	"00075b6339"	0.3912
"000757c9aa"	"00075b6658"	-0.1017
"000757c9aa"	"00075b679a"	0.0041
"000757c9aa"	"00075b6cb7"	0.1931
"000757c9aa"	"00075b6df8"	-0.2714
"000757c9aa"	"00075b6ff6"	0.1164
"000757c9aa"	"00075b70ee"	-0.14
"000757c9aa"	"00075b7157"	-0.0249
"000757c9aa"	"00075b7225"	-0.0273
"000757c9aa"	"00075b7c89"	-0.1688
"000757c9aa"	"00075b9048"	0.2409
"000757c9aa"	"00075d0801"	0.1025
"000757c9aa"	"00075d1820"	0.0185
"000757c9aa"	"00075d1f3d"	0.1492
"000757c9aa"	"00075d2329"	-0.1282
"000757c9aa"	"00075d2b9b"	0.2505
"000757c9aa"	"00075d3941"	-0.2035
"000757c9aa"	"00075d3e96"	0.1242
"000757c9aa"	"00075d4864"	-0.3565
"000757c9aa"	"00075d5961"	-0.2849
"000757c9aa"	"00075d5a63"	0.1868
"000757c9aa"	"00075d6150"	-0.124
"000757c9aa"	"00075d67d0"	0.334
"000757c9aa"	"00075d67e2"	-0.0328
"000757c9aa"	"00075d73fc"	-0.3208
"000757c9aa"	"00075d7729"	-0.0155
"000757c9aa"	"00075d778c"	-0.0169
"000757c9aa"	"00075d7b9e"	0.0309
"000757c9aa"	"00075d7c8f"	0.0225
"000757c9aa"	"00075d804d"	0.2871
"000757c9aa"	"00075d819f"	0.2629

"000757c9aa"	"00075d8601"	-0.01
"000757c9aa"	"00075d8c6a"	-0.045
"000757c9aa"	"00075dfedc"	0.0718
"000757c9aa"	"00075e05f2"	0.1138
"000757c9aa"	"00075e0837"	0.1691
"000757c9aa"	"00075e092e"	-0.1458
"000757c9aa"	"00075e0965"	-0.1616
"000757c9aa"	"00075e0bc8"	0.3442
"000757c9aa"	"00075e0fbb"	-0.3928
"000757ccbe"	"000757cfa9"	-0.2017
"000757ccbe"	"000757d390"	-0.3114
"000757ccbe"	"000757d393"	0.2507
"000757ccbe"	"000757d598"	-0.136
"000757ccbe"	"000757d5a2"	-0.1432
"000757ccbe"	"000757d790"	0.1107
"000757ccbe"	"000757e30c"	-0.2125
"000757ccbe"	"000757e4b0"	-0.0302
"000757ccbe"	"000757e7a0"	-0.2102
"000757ccbe"	"000757e8b3"	0.3135
"000757ccbe"	"000757f627"	0.0121
"000757ccbe"	"000757f925"	0.3914
"000757ccbe"	"000757fa08"	0.0778
"000757ccbe"	"000757fe52"	0.017
"000757ccbe"	"000758024a"	-0.1328
"000757ccbe"	"00075804bb"	-0.2325
"000757ccbe"	"00075a0c04"	0.0734
"000757ccbe"	"00075a3110"	0.3027
"000757ccbe"	"00075a341a"	0.2179
"000757ccbe"	"00075a3dcf"	0.0497
"000757ccbe"	"00075a3e22"	0.3119
"000757ccbe"	"00075a48d8"	0.4217
"000757ccbe"	"00075a5cfb"	-0.1656
"000757ccbe"	"00075a6151"	-0.0794
"000757ccbe"	"00075a6708"	0.0193
"000757ccbe"	"00075a7319"	-0.2213
"000757ccbe"	"00075a7723"	0.1461
"000757ccbe"	"00075a778b"	0.0644
"000757ccbe"	"00075a7b8e"	-0.0067
"000757ccbe"	"00075a7c79"	0.0748
"000757ccbe"	"00075a81b6"	0.0291
"000757ccbe"	"00075a82ac"	0.1501
"000757ccbe"	"00075a98e5"	0.2065
"000757ccbe"	"00075b0d29"	-0.0709
"000757ccbe"	"00075b102a"	-0.1022
"000757ccbe"	"00075b1074"	0.0924
"000757ccbe"	"00075b135d"	0.0869
"000757ccbe"	"00075b138b"	0.0048
"000757ccbe"	"00075b13a0"	0.1811
"000757ccbe"	"00075b13bd"	0.0141
"000757ccbe"	"00075b16a9"	-0.0678

"000757ccbe"	"00075b1a28"	-0.155
"000757ccbe"	"00075b1a97"	-0.0304
"000757ccbe"	"00075b1c7b"	-0.2267
"000757ccbe"	"00075b1d24"	8e-04
"000757ccbe"	"00075b202b"	-0.0717
"000757ccbe"	"00075b22cb"	-0.1168
"000757ccbe"	"00075b22da"	-3e-04
"000757ccbe"	"00075b2556"	-0.0513
"000757ccbe"	"00075b25de"	-0.3146
"000757ccbe"	"00075b260c"	-0.1227
"000757ccbe"	"00075b26f1"	-0.2346
"000757ccbe"	"00075b2920"	-0.0135
"000757ccbe"	"00075b2a64"	-0.1911
"000757ccbe"	"00075b2a9d"	-0.199
"000757ccbe"	"00075b2b37"	-0.3594
"000757ccbe"	"00075b2cdd"	-0.0933
"000757ccbe"	"00075b3038"	-0.1515
"000757ccbe"	"00075b30fe"	-0.0273
"000757ccbe"	"00075b3362"	-0.0316
"000757ccbe"	"00075b350a"	0.0664
"000757ccbe"	"00075b350e"	-0.0055
"000757ccbe"	"00075b3651"	-0.2323
"000757ccbe"	"00075b38ca"	-0.225
"000757ccbe"	"00075b39cc"	0.0054
"000757ccbe"	"00075b3e1e"	0.0163
"000757ccbe"	"00075b3e57"	0.1822
"000757ccbe"	"00075b4079"	-0.007
"000757ccbe"	"00075b4150"	-0.0306
"000757ccbe"	"00075b4194"	0.2035
"000757ccbe"	"00075b42d5"	-0.0607
"000757ccbe"	"00075b4424"	-0.02
"000757ccbe"	"00075b4470"	0.004
"000757ccbe"	"00075b47ed"	0.071
"000757ccbe"	"00075b4850"	0.0287
"000757ccbe"	"00075b4ca0"	-0.0251
"000757ccbe"	"00075b4d7f"	0.25
"000757ccbe"	"00075b520f"	-0.1003
"000757ccbe"	"00075b525f"	-0.1007
"000757ccbe"	"00075b58f8"	-0.2414
"000757ccbe"	"00075b5bcc"	-0.1702
"000757ccbe"	"00075b5bfa"	-0.4337
"000757ccbe"	"00075b6339"	0.2468
"000757ccbe"	"00075b6658"	-0.2021
"000757ccbe"	"00075b679a"	-0.0847
"000757ccbe"	"00075b6cb7"	-0.1053
"000757ccbe"	"00075b6df8"	0.0985
"000757ccbe"	"00075b6ff6"	0.1161
"000757ccbe"	"00075b70ee"	-0.2667
"000757ccbe"	"00075b7157"	-0.024
"000757ccbe"	"00075b7225"	0.0604

"000757ccbe"	"00075b7c89"	0.257
"000757ccbe"	"00075b9048"	0.1443
"000757ccbe"	"00075d0801"	-0.0361
"000757ccbe"	"00075d1820"	-0.331
"000757ccbe"	"00075d1f3d"	0.0058
"000757ccbe"	"00075d2329"	-0.1249
"000757ccbe"	"00075d2b9b"	-0.1777
"000757ccbe"	"00075d3941"	-0.1947
"000757ccbe"	"00075d3e96"	-0.0955
"000757ccbe"	"00075d4864"	-0.0752
"000757ccbe"	"00075d5961"	0.028
"000757ccbe"	"00075d5a63"	0.0539
"000757ccbe"	"00075d6150"	-0.0495
"000757ccbe"	"00075d67d0"	-0.0707
"000757ccbe"	"00075d67e2"	0.2809
"000757ccbe"	"00075d73fc"	0.0493
"000757ccbe"	"00075d7729"	-0.2053
"000757ccbe"	"00075d778c"	-0.1735
"000757ccbe"	"00075d7b9e"	-0.0515
"000757ccbe"	"00075d7c8f"	0.2564
"000757ccbe"	"00075d804d"	-0.0803
"000757ccbe"	"00075d819f"	-0.0225
"000757ccbe"	"00075d8601"	-0.2635
"000757ccbe"	"00075d8c6a"	0.122
"000757ccbe"	"00075dfedc"	-0.0604
"000757ccbe"	"00075e05f2"	0.1296
"000757ccbe"	"00075e0837"	-0.2035
"000757ccbe"	"00075e092e"	0.1525
"000757ccbe"	"00075e0965"	-0.1851
"000757ccbe"	"00075e0bc8"	0.0265
"000757ccbe"	"00075e0fbb"	-0.0378
"000757cfa9"	"000757d390"	-0.2561
"000757cfa9"	"000757d393"	0.2999
"000757cfa9"	"000757d598"	-0.0778
"000757cfa9"	"000757d5a2"	-3e-04
"000757cfa9"	"000757d790"	-0.2057
"000757cfa9"	"000757e30c"	-0.039
"000757cfa9"	"000757e4b0"	-0.075
"000757cfa9"	"000757e7a0"	-0.1743
"000757cfa9"	"000757e8b3"	0.213
"000757cfa9"	"000757f627"	-0.0191
"000757cfa9"	"000757f925"	-0.1072
"000757cfa9"	"000757fa08"	-0.1138
"000757cfa9"	"000757fe52"	-0.0597
"000757cfa9"	"000758024a"	0.126
"000757cfa9"	"00075804bb"	0.1236
"000757cfa9"	"00075a0c04"	-0.0322
"000757cfa9"	"00075a3110"	0.0017
"000757cfa9"	"00075a341a"	-0.0759
"000757cfa9"	"00075a3dcf"	-0.2657

"000757cfa9"	"00075a3e22"	0.0033
"000757cfa9"	"00075a48d8"	−0.3599
"000757cfa9"	"00075a5cfb"	−0.098
"000757cfa9"	"00075a6151"	−0.2198
"000757cfa9"	"00075a6708"	−0.1087
"000757cfa9"	"00075a7319"	−0.1715
"000757cfa9"	"00075a7723"	−0.1605
"000757cfa9"	"00075a778b"	−0.0135
"000757cfa9"	"00075a7b8e"	−0.3219
"000757cfa9"	"00075a7c79"	−0.0421
"000757cfa9"	"00075a81b6"	−0.3077
"000757cfa9"	"00075a82ac"	−0.1404
"000757cfa9"	"00075a98e5"	−0.0154
"000757cfa9"	"00075b0d29"	0.6286
"000757cfa9"	"00075b102a"	−0.0961
"000757cfa9"	"00075b1074"	−0.4362
"000757cfa9"	"00075b135d"	0.2222
"000757cfa9"	"00075b138b"	−0.0049
"000757cfa9"	"00075b13a0"	−0.4326
"000757cfa9"	"00075b13bd"	−0.122
"000757cfa9"	"00075b16a9"	0.0158
"000757cfa9"	"00075b1a28"	−0.0608
"000757cfa9"	"00075b1a97"	−0.0952
"000757cfa9"	"00075b1c7b"	−0.1359
"000757cfa9"	"00075b1d24"	−0.1777
"000757cfa9"	"00075b202b"	−0.1206
"000757cfa9"	"00075b22cb"	−0.1914
"000757cfa9"	"00075b22da"	−0.24
"000757cfa9"	"00075b2556"	−0.1398
"000757cfa9"	"00075b25de"	−0.0583
"000757cfa9"	"00075b260c"	−0.0731
"000757cfa9"	"00075b26f1"	−0.4391
"000757cfa9"	"00075b2920"	−0.0345
"000757cfa9"	"00075b2a64"	−0.1396
"000757cfa9"	"00075b2a9d"	−0.0978
"000757cfa9"	"00075b2b37"	−0.0262
"000757cfa9"	"00075b2cdd"	−0.1498
"000757cfa9"	"00075b3038"	−0.2854
"000757cfa9"	"00075b30fe"	−0.3836
"000757cfa9"	"00075b3362"	−0.3629
"000757cfa9"	"00075b350a"	0.011
"000757cfa9"	"00075b350e"	−0.1494
"000757cfa9"	"00075b3651"	−0.4923
"000757cfa9"	"00075b38ca"	−0.1404
"000757cfa9"	"00075b39cc"	−0.1664
"000757cfa9"	"00075b3e1e"	−0.0542
"000757cfa9"	"00075b3e57"	−0.2215
"000757cfa9"	"00075b4079"	−0.0787
"000757cfa9"	"00075b4150"	−0.071
"000757cfa9"	"00075b4194"	−0.1443

"000757cfa9"	"00075b42d5"	-0.1214
"000757cfa9"	"00075b4424"	-0.1056
"000757cfa9"	"00075b4470"	0.0609
"000757cfa9"	"00075b47ed"	-0.3982
"000757cfa9"	"00075b4850"	-3e-04
"000757cfa9"	"00075b4ca0"	-0.1961
"000757cfa9"	"00075b4d7f"	-0.0136
"000757cfa9"	"00075b520f"	0.0233
"000757cfa9"	"00075b525f"	-0.2311
"000757cfa9"	"00075b58f8"	0.1448
"000757cfa9"	"00075b5bcc"	-0.2546
"000757cfa9"	"00075b5bfa"	-0.7155
"000757cfa9"	"00075b6339"	0.2264
"000757cfa9"	"00075b6658"	-0.0364
"000757cfa9"	"00075b679a"	-0.0029
"000757cfa9"	"00075b6cb7"	-0.2178
"000757cfa9"	"00075b6df8"	-0.2213
"000757cfa9"	"00075b6ff6"	-0.1914
"000757cfa9"	"00075b70ee"	0.0224
"000757cfa9"	"00075b7157"	-0.255
"000757cfa9"	"00075b7225"	-0.1733
"000757cfa9"	"00075b7c89"	0.1036
"000757cfa9"	"00075b9048"	-0.193
"000757cfa9"	"00075d0801"	0.0556
"000757cfa9"	"00075d1820"	-0.2719
"000757cfa9"	"00075d1f3d"	-0.1311
"000757cfa9"	"00075d2329"	-0.286
"000757cfa9"	"00075d2b9b"	-0.1833
"000757cfa9"	"00075d3941"	-0.3987
"000757cfa9"	"00075d3e96"	-0.4741
"000757cfa9"	"00075d4864"	-0.0426
"000757cfa9"	"00075d5961"	0.2119
"000757cfa9"	"00075d5a63"	-0.149
"000757cfa9"	"00075d6150"	-0.287
"000757cfa9"	"00075d67d0"	-0.4935
"000757cfa9"	"00075d67e2"	0.0084
"000757cfa9"	"00075d73fc"	-0.1101
"000757cfa9"	"00075d7729"	-0.1289
"000757cfa9"	"00075d778c"	-0.3459
"000757cfa9"	"00075d7b9e"	-0.0035
"000757cfa9"	"00075d7c8f"	0.1237
"000757cfa9"	"00075d804d"	-0.16
"000757cfa9"	"00075d819f"	-0.1887
"000757cfa9"	"00075d8601"	0.0354
"000757cfa9"	"00075d8c6a"	-0.0959
"000757cfa9"	"00075dfedc"	0.1378
"000757cfa9"	"00075e05f2"	-0.3453
"000757cfa9"	"00075e0837"	-0.2576
"000757cfa9"	"00075e092e"	-0.345
"000757cfa9"	"00075e0965"	-0.1333

"000757cfa9"	"00075e0bc8"	0.1695
"000757cfa9"	"00075e0fbb"	-0.26
"000757d390"	"000757d393"	-0.1637
"000757d390"	"000757d598"	0.0427
"000757d390"	"000757d5a2"	-0.169
"000757d390"	"000757d790"	-0.0322
"000757d390"	"000757e30c"	0.111
"000757d390"	"000757e4b0"	-0.1774
"000757d390"	"000757e7a0"	0.0532
"000757d390"	"000757e8b3"	-0.3803
"000757d390"	"000757f627"	0.1693
"000757d390"	"000757f925"	-0.2164
"000757d390"	"000757fa08"	-0.4216
"000757d390"	"000757fe52"	-0.1933
"000757d390"	"000758024a"	-0.0884
"000757d390"	"00075804bb"	0.009
"000757d390"	"00075a0c04"	-0.3121
"000757d390"	"00075a3110"	-0.1748
"000757d390"	"00075a341a"	-0.3318
"000757d390"	"00075a3dcf"	-0.2501
"000757d390"	"00075a3e22"	-0.1601
"000757d390"	"00075a48d8"	-0.4862
"000757d390"	"00075a5cfb"	-0.0202
"000757d390"	"00075a6151"	-0.0549
"000757d390"	"00075a6708"	-0.0055
"000757d390"	"00075a7319"	0.7621
"000757d390"	"00075a7723"	-0.29
"000757d390"	"00075a778b"	-0.1963
"000757d390"	"00075a7b8e"	0.2719
"000757d390"	"00075a7c79"	0.0669
"000757d390"	"00075a81b6"	-0.4208
"000757d390"	"00075a82ac"	-0.0096
"000757d390"	"00075a98e5"	-0.4409
"000757d390"	"00075b0d29"	-0.3831
"000757d390"	"00075b102a"	-0.2521
"000757d390"	"00075b1074"	-0.3513
"000757d390"	"00075b135d"	-0.249
"000757d390"	"00075b138b"	-0.1989
"000757d390"	"00075b13a0"	-0.3736
"000757d390"	"00075b13bd"	-0.1866
"000757d390"	"00075b16a9"	0.0239
"000757d390"	"00075b1a28"	-0.2405
"000757d390"	"00075b1a97"	-0.0392
"000757d390"	"00075b1c7b"	-0.316
"000757d390"	"00075b1d24"	-0.086
"000757d390"	"00075b202b"	-0.0093
"000757d390"	"00075b22cb"	-0.2493
"000757d390"	"00075b22da"	-0.2852
"000757d390"	"00075b2556"	-0.1555
"000757d390"	"00075b25de"	0.0976

"000757d390"	"00075b260c"	-0.075
"000757d390"	"00075b26f1"	-0.681
"000757d390"	"00075b2920"	0.2719
"000757d390"	"00075b2a64"	-0.0822
"000757d390"	"00075b2a9d"	-0.186
"000757d390"	"00075b2b37"	-0.1614
"000757d390"	"00075b2cdd"	-0.0749
"000757d390"	"00075b3038"	0.0052
"000757d390"	"00075b30fe"	-0.1859
"000757d390"	"00075b3362"	-0.3011
"000757d390"	"00075b350a"	-0.1706
"000757d390"	"00075b350e"	-0.0581
"000757d390"	"00075b3651"	-0.224
"000757d390"	"00075b38ca"	-0.1429
"000757d390"	"00075b39cc"	-0.1085
"000757d390"	"00075b3e1e"	-0.1768
"000757d390"	"00075b3e57"	-0.2267
"000757d390"	"00075b4079"	-0.1025
"000757d390"	"00075b4150"	-0.0209
"000757d390"	"00075b4194"	-0.0949
"000757d390"	"00075b42d5"	-0.1098
"000757d390"	"00075b4424"	-0.301
"000757d390"	"00075b4470"	0.0026
"000757d390"	"00075b47ed"	-0.0251
"000757d390"	"00075b4850"	0.0321
"000757d390"	"00075b4ca0"	-0.242
"000757d390"	"00075b4d7f"	-0.1406
"000757d390"	"00075b520f"	-0.1998
"000757d390"	"00075b525f"	-0.1285
"000757d390"	"00075b58f8"	0.0086
"000757d390"	"00075b5bcc"	0.0799
"000757d390"	"00075b5bfa"	-0.1266
"000757d390"	"00075b6339"	-0.2472
"000757d390"	"00075b6658"	-0.5393
"000757d390"	"00075b679a"	0.0598
"000757d390"	"00075b6cb7"	-0.3813
"000757d390"	"00075b6df8"	-0.231
"000757d390"	"00075b6ff6"	-0.0476
"000757d390"	"00075b70ee"	-0.0632
"000757d390"	"00075b7157"	0.0665
"000757d390"	"00075b7225"	-0.2352
"000757d390"	"00075b7c89"	-0.1377
"000757d390"	"00075b9048"	0.1198
"000757d390"	"00075d0801"	0.0305
"000757d390"	"00075d1820"	-0.0649
"000757d390"	"00075d1f3d"	-0.1136
"000757d390"	"00075d2329"	-0.2731
"000757d390"	"00075d2b9b"	-0.2061
"000757d390"	"00075d3941"	-0.5614
"000757d390"	"00075d3e96"	0.0481

"000757d390"	"00075d4864"	-0.1804
"000757d390"	"00075d5961"	-0.1963
"000757d390"	"00075d5a63"	-0.275
"000757d390"	"00075d6150"	0.118
"000757d390"	"00075d67d0"	-0.2849
"000757d390"	"00075d67e2"	-0.4655
"000757d390"	"00075d73fc"	-0.3675
"000757d390"	"00075d7729"	-0.1921
"000757d390"	"00075d778c"	-0.4173
"000757d390"	"00075d7b9e"	-0.0428
"000757d390"	"00075d7c8f"	-0.2854
"000757d390"	"00075d804d"	-0.0115
"000757d390"	"00075d819f"	-0.1966
"000757d390"	"00075d8601"	-0.0015
"000757d390"	"00075d8c6a"	-0.194
"000757d390"	"00075dfedc"	-0.1764
"000757d390"	"00075e05f2"	-0.3937
"000757d390"	"00075e0837"	-0.2199
"000757d390"	"00075e092e"	-0.2444
"000757d390"	"00075e0965"	-0.1219
"000757d390"	"00075e0bc8"	-0.1742
"000757d390"	"00075e0fbb"	-0.2848
"000757d393"	"000757d598"	-0.0509
"000757d393"	"000757d5a2"	-0.0629
"000757d393"	"000757d790"	0.3519
"000757d393"	"000757e30c"	-0.1013
"000757d393"	"000757e4b0"	0.0547
"000757d393"	"000757e7a0"	-0.1034
"000757d393"	"000757e8b3"	0.2573
"000757d393"	"000757f627"	0.1322
"000757d393"	"000757f925"	0.5541
"000757d393"	"000757fa08"	0.0328
"000757d393"	"000757fe52"	0.11
"000757d393"	"000758024a"	-0.0333
"000757d393"	"00075804bb"	0.0832
"000757d393"	"00075a0c04"	0.168
"000757d393"	"00075a3110"	0.1412
"000757d393"	"00075a341a"	0.1292
"000757d393"	"00075a3dcf"	0.2122
"000757d393"	"00075a3e22"	0.229
"000757d393"	"00075a48d8"	0.2375
"000757d393"	"00075a5cfb"	-0.1744
"000757d393"	"00075a6151"	0.1491
"000757d393"	"00075a6708"	-0.0261
"000757d393"	"00075a7319"	-0.1148
"000757d393"	"00075a7723"	0.3282
"000757d393"	"00075a778b"	0.1586
"000757d393"	"00075a7b8e"	-0.1556
"000757d393"	"00075a7c79"	0.0828
"000757d393"	"00075a81b6"	-0.233

"000757d393"	"00075a82ac"	0.2555
"000757d393"	"00075a98e5"	0.3954
"000757d393"	"00075b0d29"	0.1988
"000757d393"	"00075b102a"	0.2367
"000757d393"	"00075b1074"	−0.1838
"000757d393"	"00075b135d"	−0.0108
"000757d393"	"00075b138b"	0.2385
"000757d393"	"00075b13a0"	0.0863
"000757d393"	"00075b13bd"	−0.0967
"000757d393"	"00075b16a9"	0.2461
"000757d393"	"00075b1a28"	0.0423
"000757d393"	"00075b1a97"	0.0257
"000757d393"	"00075b1c7b"	0.0243
"000757d393"	"00075b1d24"	−0.1182
"000757d393"	"00075b202b"	0.0363
"000757d393"	"00075b22cb"	0.1064
"000757d393"	"00075b22da"	−0.0244
"000757d393"	"00075b2556"	0.062
"000757d393"	"00075b25de"	0.2936
"000757d393"	"00075b260c"	−0.011
"000757d393"	"00075b26f1"	−0.1146
"000757d393"	"00075b2920"	0.0691
"000757d393"	"00075b2a64"	−0.0691
"000757d393"	"00075b2a9d"	0.0809
"000757d393"	"00075b2b37"	−0.1193
"000757d393"	"00075b2cdd"	0.0981
"000757d393"	"00075b3038"	0.1801
"000757d393"	"00075b30fe"	−0.0214
"000757d393"	"00075b3362"	−0.0789
"000757d393"	"00075b350a"	−0.1281
"000757d393"	"00075b350e"	0.0741
"000757d393"	"00075b3651"	−0.1653
"000757d393"	"00075b38ca"	0.0832
"000757d393"	"00075b39cc"	0.0502
"000757d393"	"00075b3e1e"	0.3334
"000757d393"	"00075b3e57"	0.2091
"000757d393"	"00075b4079"	−0.1404
"000757d393"	"00075b4150"	0.0642
"000757d393"	"00075b4194"	0.1336
"000757d393"	"00075b42d5"	0.1112
"000757d393"	"00075b4424"	−0.1368
"000757d393"	"00075b4470"	0.0774
"000757d393"	"00075b47ed"	−0.0196
"000757d393"	"00075b4850"	−0.0124
"000757d393"	"00075b4ca0"	−0.2472
"000757d393"	"00075b4d7f"	0.261
"000757d393"	"00075b520f"	0.0337
"000757d393"	"00075b525f"	−0.1813
"000757d393"	"00075b58f8"	0.0448
"000757d393"	"00075b5bcc"	−0.1549

"000757d393"	"00075b5bfa"	−0.2205
"000757d393"	"00075b6339"	0.231
"000757d393"	"00075b6658"	−0.0318
"000757d393"	"00075b679a"	0.0359
"000757d393"	"00075b6cb7"	−0.0729
"000757d393"	"00075b6df8"	0.1306
"000757d393"	"00075b6ff6"	−0.0784
"000757d393"	"00075b70ee"	−0.12
"000757d393"	"00075b7157"	0.0814
"000757d393"	"00075b7225"	0.0218
"000757d393"	"00075b7c89"	0.2325
"000757d393"	"00075b9048"	0.1519
"000757d393"	"00075d0801"	0.3122
"000757d393"	"00075d1820"	−0.2486
"000757d393"	"00075d1f3d"	−0.0629
"000757d393"	"00075d2329"	0.0628
"000757d393"	"00075d2b9b"	−0.0499
"000757d393"	"00075d3941"	−0.1557
"000757d393"	"00075d3e96"	−0.1842
"000757d393"	"00075d4864"	0.1836
"000757d393"	"00075d5961"	0.4397
"000757d393"	"00075d5a63"	−0.0123
"000757d393"	"00075d6150"	−0.1211
"000757d393"	"00075d67d0"	−0.0547
"000757d393"	"00075d67e2"	0.3184
"000757d393"	"00075d73fc"	0.0889
"000757d393"	"00075d7729"	−0.3602
"000757d393"	"00075d778c"	−0.0667
"000757d393"	"00075d7b9e"	0.1218
"000757d393"	"00075d7c8f"	0.1864
"000757d393"	"00075d804d"	0.1866
"000757d393"	"00075d819f"	0.1541
"000757d393"	"00075d8601"	0.0969
"000757d393"	"00075d8c6a"	0.1426
"000757d393"	"00075dfedc"	−4e−04
"000757d393"	"00075e05f2"	0.2123
"000757d393"	"00075e0837"	9e−04
"000757d393"	"00075e092e"	0.1064
"000757d393"	"00075e0965"	−0.1092
"000757d393"	"00075e0bc8"	0.1671
"000757d393"	"00075e0fbb"	0.0089
"000757d598"	"000757d5a2"	0.5704
"000757d598"	"000757d790"	−0.0277
"000757d598"	"000757e30c"	0.3139
"000757d598"	"000757e4b0"	−0.072
"000757d598"	"000757e7a0"	−0.033
"000757d598"	"000757e8b3"	0.1121
"000757d598"	"000757f627"	−0.1819
"000757d598"	"000757f925"	0.1029
"000757d598"	"000757fa08"	−0.3926

"000757d598"	"000757fe52"	−0.0297
"000757d598"	"000758024a"	0.0744
"000757d598"	"00075804bb"	0.2791
"000757d598"	"00075a0c04"	−0.2667
"000757d598"	"00075a3110"	0.1705
"000757d598"	"00075a341a"	−0.2117
"000757d598"	"00075a3dcf"	−0.4316
"000757d598"	"00075a3e22"	−0.1743
"000757d598"	"00075a48d8"	−0.2657
"000757d598"	"00075a5cfb"	0.2655
"000757d598"	"00075a6151"	0.0622
"000757d598"	"00075a6708"	0.0807
"000757d598"	"00075a7319"	−0.075
"000757d598"	"00075a7723"	−0.3055
"000757d598"	"00075a778b"	−0.2409
"000757d598"	"00075a7b8e"	−0.1272
"000757d598"	"00075a7c79"	0.1285
"000757d598"	"00075a81b6"	−0.3388
"000757d598"	"00075a82ac"	−0.2984
"000757d598"	"00075a98e5"	−0.5931
"000757d598"	"00075b0d29"	−0.2523
"000757d598"	"00075b102a"	0.0675
"000757d598"	"00075b1074"	−0.3578
"000757d598"	"00075b135d"	−0.1411
"000757d598"	"00075b138b"	−0.0777
"000757d598"	"00075b13a0"	−0.3193
"000757d598"	"00075b13bd"	−0.4746
"000757d598"	"00075b16a9"	−0.0346
"000757d598"	"00075b1a28"	−0.3553
"000757d598"	"00075b1a97"	−0.1978
"000757d598"	"00075b1c7b"	−0.1846
"000757d598"	"00075b1d24"	−0.1633
"000757d598"	"00075b202b"	0.0476
"000757d598"	"00075b22cb"	−0.2656
"000757d598"	"00075b22da"	−0.1241
"000757d598"	"00075b2556"	−0.1047
"000757d598"	"00075b25de"	0.0597
"000757d598"	"00075b260c"	0.3218
"000757d598"	"00075b26f1"	−0.3626
"000757d598"	"00075b2920"	0.4711
"000757d598"	"00075b2a64"	0.247
"000757d598"	"00075b2a9d"	0.0281
"000757d598"	"00075b2b37"	−0.1977
"000757d598"	"00075b2cdd"	−0.2421
"000757d598"	"00075b3038"	0.0725
"000757d598"	"00075b30fe"	−0.086
"000757d598"	"00075b3362"	−0.341
"000757d598"	"00075b350a"	−0.087
"000757d598"	"00075b350e"	−0.1227
"000757d598"	"00075b3651"	−0.3864

"000757d598"	"00075b38ca"	−0.5689
"000757d598"	"00075b39cc"	−0.2183
"000757d598"	"00075b3e1e"	−0.2723
"000757d598"	"00075b3e57"	−0.0604
"000757d598"	"00075b4079"	−0.0225
"000757d598"	"00075b4150"	0.3238
"000757d598"	"00075b4194"	−0.0438
"000757d598"	"00075b42d5"	−0.3732
"000757d598"	"00075b4424"	−0.0314
"000757d598"	"00075b4470"	−0.1799
"000757d598"	"00075b47ed"	−0.4522
"000757d598"	"00075b4850"	0.0381
"000757d598"	"00075b4ca0"	0.0468
"000757d598"	"00075b4d7f"	−0.1715
"000757d598"	"00075b520f"	−0.3046
"000757d598"	"00075b525f"	−0.0975
"000757d598"	"00075b58f8"	−0.065
"000757d598"	"00075b5bcc"	0.0557
"000757d598"	"00075b5bfa"	−0.081
"000757d598"	"00075b6339"	0.157
"000757d598"	"00075b6658"	−0.4692
"000757d598"	"00075b679a"	0.1272
"000757d598"	"00075b6cb7"	−0.0011
"000757d598"	"00075b6df8"	−0.2368
"000757d598"	"00075b6ff6"	0.3647
"000757d598"	"00075b70ee"	0.4484
"000757d598"	"00075b7157"	−0.1331
"000757d598"	"00075b7225"	−0.0867
"000757d598"	"00075b7c89"	−0.0525
"000757d598"	"00075b9048"	0.311
"000757d598"	"00075d0801"	−0.1793
"000757d598"	"00075d1820"	−0.2807
"000757d598"	"00075d1f3d"	−0.089
"000757d598"	"00075d2329"	−0.5115
"000757d598"	"00075d2b9b"	0.0872
"000757d598"	"00075d3941"	−0.6043
"000757d598"	"00075d3e96"	0.0137
"000757d598"	"00075d4864"	−0.4336
"000757d598"	"00075d5961"	−0.3231
"000757d598"	"00075d5a63"	−0.082
"000757d598"	"00075d6150"	−0.1209
"000757d598"	"00075d67d0"	−0.1768
"000757d598"	"00075d67e2"	−0.1397
"000757d598"	"00075d73fc"	−0.3261
"000757d598"	"00075d7729"	−0.1048
"000757d598"	"00075d778c"	−0.376
"000757d598"	"00075d7b9e"	−0.2911
"000757d598"	"00075d7c8f"	−0.1019
"000757d598"	"00075d804d"	0.0224
"000757d598"	"00075d819f"	−0.0942

"000757d598"	"00075d8601"	−0.3174
"000757d598"	"00075d8c6a"	−0.1968
"000757d598"	"00075dfedc"	0.0648
"000757d598"	"00075e05f2"	−0.0446
"000757d598"	"00075e0837"	−0.0653
"000757d598"	"00075e092e"	−0.2651
"000757d598"	"00075e0965"	−0.0387
"000757d598"	"00075e0bc8"	0.0726
"000757d598"	"00075e0fbb"	−0.3471
"000757d5a2"	"000757d790"	−0.1151
"000757d5a2"	"000757e30c"	0.1721
"000757d5a2"	"000757e4b0"	0.0596
"000757d5a2"	"000757e7a0"	0.2693
"000757d5a2"	"000757e8b3"	−0.0227
"000757d5a2"	"000757f627"	−0.109
"000757d5a2"	"000757f925"	0.0773
"000757d5a2"	"000757fa08"	−0.2769
"000757d5a2"	"000757fe52"	−0.2952
"000757d5a2"	"000758024a"	−0.1157
"000757d5a2"	"00075804bb"	0.1617
"000757d5a2"	"00075a0c04"	−0.0016
"000757d5a2"	"00075a3110"	0.0922
"000757d5a2"	"00075a341a"	−0.0937
"000757d5a2"	"00075a3dcf"	−0.135
"000757d5a2"	"00075a3e22"	−0.0971
"000757d5a2"	"00075a48d8"	−0.2002
"000757d5a2"	"00075a5cfb"	−0.0327
"000757d5a2"	"00075a6151"	−0.0283
"000757d5a2"	"00075a6708"	0.1524
"000757d5a2"	"00075a7319"	−0.0462
"000757d5a2"	"00075a7723"	−0.204
"000757d5a2"	"00075a778b"	−0.1958
"000757d5a2"	"00075a7b8e"	−0.1567
"000757d5a2"	"00075a7c79"	0.1387
"000757d5a2"	"00075a81b6"	−0.2244
"000757d5a2"	"00075a82ac"	−0.2123
"000757d5a2"	"00075a98e5"	−0.5511
"000757d5a2"	"00075b0d29"	−0.2598
"000757d5a2"	"00075b102a"	9e−04
"000757d5a2"	"00075b1074"	−0.0994
"000757d5a2"	"00075b135d"	−0.052
"000757d5a2"	"00075b138b"	−0.001
"000757d5a2"	"00075b13a0"	−0.5435
"000757d5a2"	"00075b13bd"	−0.3972
"000757d5a2"	"00075b16a9"	0.0697
"000757d5a2"	"00075b1a28"	−0.2368
"000757d5a2"	"00075b1a97"	−0.1378
"000757d5a2"	"00075b1c7b"	−0.1353
"000757d5a2"	"00075b1d24"	−0.1248
"000757d5a2"	"00075b202b"	−0.0174

"000757d5a2"	"00075b22cb"	−0.2075
"000757d5a2"	"00075b22da"	−0.0375
"000757d5a2"	"00075b2556"	−0.0423
"000757d5a2"	"00075b25de"	−0.1207
"000757d5a2"	"00075b260c"	0.2226
"000757d5a2"	"00075b26f1"	−0.1155
"000757d5a2"	"00075b2920"	0.2195
"000757d5a2"	"00075b2a64"	0.0647
"000757d5a2"	"00075b2a9d"	0.1814
"000757d5a2"	"00075b2b37"	−0.1892
"000757d5a2"	"00075b2cdd"	−0.1845
"000757d5a2"	"00075b3038"	−0.0531
"000757d5a2"	"00075b30fe"	−0.0258
"000757d5a2"	"00075b3362"	−0.4134
"000757d5a2"	"00075b350a"	−0.1482
"000757d5a2"	"00075b350e"	−0.2475
"000757d5a2"	"00075b3651"	−0.2355
"000757d5a2"	"00075b38ca"	−0.419
"000757d5a2"	"00075b39cc"	−0.0091
"000757d5a2"	"00075b3e1e"	−0.2163
"000757d5a2"	"00075b3e57"	−0.112
"000757d5a2"	"00075b4079"	−0.2725
"000757d5a2"	"00075b4150"	0.084
"000757d5a2"	"00075b4194"	0.0068
"000757d5a2"	"00075b42d5"	−0.2995
"000757d5a2"	"00075b4424"	−0.0628
"000757d5a2"	"00075b4470"	−0.2207
"000757d5a2"	"00075b47ed"	−0.2682
"000757d5a2"	"00075b4850"	0.0259
"000757d5a2"	"00075b4ca0"	0.1919
"000757d5a2"	"00075b4d7f"	−0.1577
"000757d5a2"	"00075b520f"	−0.2416
"000757d5a2"	"00075b525f"	−0.206
"000757d5a2"	"00075b58f8"	−0.0612
"000757d5a2"	"00075b5bcc"	−0.198
"000757d5a2"	"00075b5bfa"	−0.3355
"000757d5a2"	"00075b6339"	0.0715
"000757d5a2"	"00075b6658"	−0.3815
"000757d5a2"	"00075b679a"	0.0348
"000757d5a2"	"00075b6cb7"	−0.0295
"000757d5a2"	"00075b6df8"	−0.1914
"000757d5a2"	"00075b6ff6"	0.1956
"000757d5a2"	"00075b70ee"	0.353
"000757d5a2"	"00075b7157"	−0.1091
"000757d5a2"	"00075b7225"	−0.0891
"000757d5a2"	"00075b7c89"	0.1367
"000757d5a2"	"00075b9048"	0.2493
"000757d5a2"	"00075d0801"	−0.3052
"000757d5a2"	"00075d1820"	−0.1488
"000757d5a2"	"00075d1f3d"	−0.0729

"000757d5a2"	"00075d2329"	–0.2123
"000757d5a2"	"00075d2b9b"	–0.1873
"000757d5a2"	"00075d3941"	–0.3864
"000757d5a2"	"00075d3e96"	0.1059
"000757d5a2"	"00075d4864"	–0.4286
"000757d5a2"	"00075d5961"	–0.084
"000757d5a2"	"00075d5a63"	–0.1271
"000757d5a2"	"00075d6150"	–0.0169
"000757d5a2"	"00075d67d0"	–0.0963
"000757d5a2"	"00075d67e2"	–0.3392
"000757d5a2"	"00075d73fc"	–0.1782
"000757d5a2"	"00075d7729"	–0.1103
"000757d5a2"	"00075d778c"	–0.3513
"000757d5a2"	"00075d7b9e"	–0.162
"000757d5a2"	"00075d7c8f"	0.0801
"000757d5a2"	"00075d804d"	0.0396
"000757d5a2"	"00075d819f"	0.061
"000757d5a2"	"00075d8601"	–0.2564
"000757d5a2"	"00075d8c6a"	–0.3087
"000757d5a2"	"00075dfedc"	–0.0072
"000757d5a2"	"00075e05f2"	–0.1358
"000757d5a2"	"00075e0837"	0.0962
"000757d5a2"	"00075e092e"	0.0358
"000757d5a2"	"00075e0965"	–0.3137
"000757d5a2"	"00075e0bc8"	0.0401
"000757d5a2"	"00075e0fbb"	–0.1722
"000757d790"	"000757e30c"	0.0068
"000757d790"	"000757e4b0"	–0.0335
"000757d790"	"000757e7a0"	–0.047
"000757d790"	"000757e8b3"	0.0214
"000757d790"	"000757f627"	–0.0364
"000757d790"	"000757f925"	0.4049
"000757d790"	"000757fa08"	–0.2319
"000757d790"	"000757fe52"	0.0709
"000757d790"	"000758024a"	–0.34
"000757d790"	"00075804bb"	–0.1418
"000757d790"	"00075a0c04"	–0.0237
"000757d790"	"00075a3110"	0.2022
"000757d790"	"00075a341a"	–0.0193
"000757d790"	"00075a3dcf"	0.2776
"000757d790"	"00075a3e22"	0.0687
"000757d790"	"00075a48d8"	0.0804
"000757d790"	"00075a5cfb"	–0.1182
"000757d790"	"00075a6151"	0.3436
"000757d790"	"00075a6708"	0.0366
"000757d790"	"00075a7319"	–0.0152
"000757d790"	"00075a7723"	0.0197
"000757d790"	"00075a778b"	0.1569
"000757d790"	"00075a7b8e"	–0.1183
"000757d790"	"00075a7c79"	0.2135

"000757d790"	"00075a81b6"	−0.1036
"000757d790"	"00075a82ac"	0.1651
"000757d790"	"00075a98e5"	0.0488
"000757d790"	"00075b0d29"	−0.3049
"000757d790"	"00075b102a"	0.0131
"000757d790"	"00075b1074"	−0.0194
"000757d790"	"00075b135d"	−0.1668
"000757d790"	"00075b138b"	0.1105
"000757d790"	"00075b13a0"	0.106
"000757d790"	"00075b13bd"	−0.3292
"000757d790"	"00075b16a9"	0.0511
"000757d790"	"00075b1a28"	−0.2333
"000757d790"	"00075b1a97"	−0.0284
"000757d790"	"00075b1c7b"	−0.0926
"000757d790"	"00075b1d24"	−0.033
"000757d790"	"00075b202b"	0.0403
"000757d790"	"00075b22cb"	−0.0686
"000757d790"	"00075b22da"	0.0315
"000757d790"	"00075b2556"	−0.0068
"000757d790"	"00075b25de"	0.1954
"000757d790"	"00075b260c"	0.1689
"000757d790"	"00075b26f1"	0.0142
"000757d790"	"00075b2920"	0.0466
"000757d790"	"00075b2a64"	−0.1527
"000757d790"	"00075b2a9d"	0.0074
"000757d790"	"00075b2b37"	−0.1962
"000757d790"	"00075b2cdd"	0.063
"000757d790"	"00075b3038"	0.2431
"000757d790"	"00075b30fe"	0.0628
"000757d790"	"00075b3362"	0.0832
"000757d790"	"00075b350a"	−0.3963
"000757d790"	"00075b350e"	0.0443
"000757d790"	"00075b3651"	0.0807
"000757d790"	"00075b38ca"	−0.1536
"000757d790"	"00075b39cc"	0.0455
"000757d790"	"00075b3e1e"	0.054
"000757d790"	"00075b3e57"	−0.0235
"000757d790"	"00075b4079"	−0.2832
"000757d790"	"00075b4150"	0.0193
"000757d790"	"00075b4194"	−0.0485
"000757d790"	"00075b42d5"	0.0094
"000757d790"	"00075b4424"	−0.127
"000757d790"	"00075b4470"	−0.0103
"000757d790"	"00075b47ed"	0.0343
"000757d790"	"00075b4850"	0.0699
"000757d790"	"00075b4ca0"	−0.0843
"000757d790"	"00075b4d7f"	−0.0504
"000757d790"	"00075b520f"	−0.2019
"000757d790"	"00075b525f"	0.2065
"000757d790"	"00075b58f8"	−0.1265

"000757d790"	"00075b5bcc"	−0.0893
"000757d790"	"00075b5bfa"	0.2858
"000757d790"	"00075b6339"	0.1671
"000757d790"	"00075b6658"	−0.1561
"000757d790"	"00075b679a"	−0.0598
"000757d790"	"00075b6cb7"	−0.1347
"000757d790"	"00075b6df8"	0.0219
"000757d790"	"00075b6ff6"	−0.0086
"000757d790"	"00075b70ee"	−0.2191
"000757d790"	"00075b7157"	0.2032
"000757d790"	"00075b7225"	0.0392
"000757d790"	"00075b7c89"	0.102
"000757d790"	"00075b9048"	0.2871
"000757d790"	"00075d0801"	0.0337
"000757d790"	"00075d1820"	0.0301
"000757d790"	"00075d1f3d"	−0.0919
"000757d790"	"00075d2329"	0.1925
"000757d790"	"00075d2b9b"	−0.0678
"000757d790"	"00075d3941"	−0.3212
"000757d790"	"00075d3e96"	0.0417
"000757d790"	"00075d4864"	−0.0876
"000757d790"	"00075d5961"	0.0934
"000757d790"	"00075d5a63"	−0.169
"000757d790"	"00075d6150"	−0.1782
"000757d790"	"00075d67d0"	−0.0368
"000757d790"	"00075d67e2"	0.066
"000757d790"	"00075d73fc"	−0.1366
"000757d790"	"00075d7729"	−0.3009
"000757d790"	"00075d778c"	0.1551
"000757d790"	"00075d7b9e"	0.1766
"000757d790"	"00075d7c8f"	0.0866
"000757d790"	"00075d804d"	0.143
"000757d790"	"00075d819f"	0.029
"000757d790"	"00075d8601"	−0.166
"000757d790"	"00075d8c6a"	−0.0406
"000757d790"	"00075dfedc"	−0.0645
"000757d790"	"00075e05f2"	0.3223
"000757d790"	"00075e0837"	0.2127
"000757d790"	"00075e092e"	0.1217
"000757d790"	"00075e0965"	−0.2975
"000757d790"	"00075e0bc8"	0.1351
"000757d790"	"00075e0fbb"	0.0453
"000757e30c"	"000757e4b0"	0.0265
"000757e30c"	"000757e7a0"	0.2457
"000757e30c"	"000757e8b3"	0.0344
"000757e30c"	"000757f627"	0.061
"000757e30c"	"000757f925"	0.0578
"000757e30c"	"000757fa08"	−0.2078
"000757e30c"	"000757fe52"	−0.21
"000757e30c"	"000758024a"	0.4307

"000757e30c"	"00075804bb"	0.4755
"000757e30c"	"00075a0c04"	−0.2941
"000757e30c"	"00075a3110"	0.0573
"000757e30c"	"00075a341a"	−0.2962
"000757e30c"	"00075a3dcf"	−0.295
"000757e30c"	"00075a3e22"	−0.083
"000757e30c"	"00075a48d8"	−0.1558
"000757e30c"	"00075a5cfb"	0.5053
"000757e30c"	"00075a6151"	0.1988
"000757e30c"	"00075a6708"	0.2792
"000757e30c"	"00075a7319"	0.0633
"000757e30c"	"00075a7723"	−0.0242
"000757e30c"	"00075a778b"	−0.0271
"000757e30c"	"00075a7b8e"	0.2847
"000757e30c"	"00075a7c79"	0.1192
"000757e30c"	"00075a81b6"	−0.1923
"000757e30c"	"00075a82ac"	−0.1453
"000757e30c"	"00075a98e5"	−0.381
"000757e30c"	"00075b0d29"	−0.1299
"000757e30c"	"00075b102a"	0.1763
"000757e30c"	"00075b1074"	−0.1294
"000757e30c"	"00075b135d"	−0.1468
"000757e30c"	"00075b138b"	0.0302
"000757e30c"	"00075b13a0"	−0.4032
"000757e30c"	"00075b13bd"	−0.3727
"000757e30c"	"00075b16a9"	0.1307
"000757e30c"	"00075b1a28"	−0.189
"000757e30c"	"00075b1a97"	−0.2988
"000757e30c"	"00075b1c7b"	0.0325
"000757e30c"	"00075b1d24"	0.0783
"000757e30c"	"00075b202b"	0.203
"000757e30c"	"00075b22cb"	−0.259
"000757e30c"	"00075b22da"	−0.1053
"000757e30c"	"00075b2556"	0.0144
"000757e30c"	"00075b25de"	0.0514
"000757e30c"	"00075b260c"	0.0466
"000757e30c"	"00075b26f1"	−0.1442
"000757e30c"	"00075b2920"	0.6026
"000757e30c"	"00075b2a64"	0.3256
"000757e30c"	"00075b2a9d"	0.1435
"000757e30c"	"00075b2b37"	−0.0726
"000757e30c"	"00075b2cdd"	−0.0177
"000757e30c"	"00075b3038"	−0.0153
"000757e30c"	"00075b30fe"	−0.114
"000757e30c"	"00075b3362"	−0.1745
"000757e30c"	"00075b350a"	−0.0728
"000757e30c"	"00075b350e"	−0.3164
"000757e30c"	"00075b3651"	−0.098
"000757e30c"	"00075b38ca"	−0.3366
"000757e30c"	"00075b39cc"	−0.1442

"000757e30c"	"00075b3e1e"	-0.1567
"000757e30c"	"00075b3e57"	-0.0539
"000757e30c"	"00075b4079"	-0.1351
"000757e30c"	"00075b4150"	0.3843
"000757e30c"	"00075b4194"	0.0569
"000757e30c"	"00075b42d5"	-0.0821
"000757e30c"	"00075b4424"	0.0557
"000757e30c"	"00075b4470"	0.2892
"000757e30c"	"00075b47ed"	-0.307
"000757e30c"	"00075b4850"	0.3299
"000757e30c"	"00075b4ca0"	-0.0333
"000757e30c"	"00075b4d7f"	-0.0485
"000757e30c"	"00075b520f"	-0.1972
"000757e30c"	"00075b525f"	-0.0241
"000757e30c"	"00075b58f8"	0.3729
"000757e30c"	"00075b5bcc"	-0.119
"000757e30c"	"00075b5bfa"	-0.2405
"000757e30c"	"00075b6339"	0.138
"000757e30c"	"00075b6658"	-0.4732
"000757e30c"	"00075b679a"	-0.1031
"000757e30c"	"00075b6cb7"	0.2491
"000757e30c"	"00075b6df8"	-0.2842
"000757e30c"	"00075b6ff6"	0.1464
"000757e30c"	"00075b70ee"	0.2437
"000757e30c"	"00075b7157"	0.0764
"000757e30c"	"00075b7225"	-0.2933
"000757e30c"	"00075b7c89"	-0.1904
"000757e30c"	"00075b9048"	0.255
"000757e30c"	"00075d0801"	0.1179
"000757e30c"	"00075d1820"	-0.1928
"000757e30c"	"00075d1f3d"	0.0975
"000757e30c"	"00075d2329"	-0.2142
"000757e30c"	"00075d2b9b"	0.0678
"000757e30c"	"00075d3941"	-0.5335
"000757e30c"	"00075d3e96"	0.0661
"000757e30c"	"00075d4864"	-0.1967
"000757e30c"	"00075d5961"	-0.1996
"000757e30c"	"00075d5a63"	0.0977
"000757e30c"	"00075d6150"	-0.4091
"000757e30c"	"00075d67d0"	-0.1949
"000757e30c"	"00075d67e2"	-0.2579
"000757e30c"	"00075d73fc"	-0.3898
"000757e30c"	"00075d7729"	-0.2366
"000757e30c"	"00075d778c"	-0.2486
"000757e30c"	"00075d7b9e"	-0.1236
"000757e30c"	"00075d7c8f"	-0.0908
"000757e30c"	"00075d804d"	0.1234
"000757e30c"	"00075d819f"	0.128
"000757e30c"	"00075d8601"	-0.3662
"000757e30c"	"00075d8c6a"	-0.1407

"000757e30c"	"00075dfedc"	-0.093
"000757e30c"	"00075e05f2"	0.0542
"000757e30c"	"00075e0837"	0.2395
"000757e30c"	"00075e092e"	-0.0815
"000757e30c"	"00075e0965"	0.2969
"000757e30c"	"00075e0bc8"	0.1328
"000757e30c"	"00075e0fbb"	-0.0743
"000757e4b0"	"000757e7a0"	0.1945
"000757e4b0"	"000757e8b3"	0.2052
"000757e4b0"	"000757f627"	0.0881
"000757e4b0"	"000757f925"	-0.0262
"000757e4b0"	"000757fa08"	-0.1146
"000757e4b0"	"000757fe52"	-0.1849
"000757e4b0"	"000758024a"	-0.1949
"000757e4b0"	"00075804bb"	0.0948
"000757e4b0"	"00075a0c04"	0.2273
"000757e4b0"	"00075a3110"	0.2175
"000757e4b0"	"00075a341a"	-0.0626
"000757e4b0"	"00075a3dcf"	0.1049
"000757e4b0"	"00075a3e22"	0.2319
"000757e4b0"	"00075a48d8"	-0.1234
"000757e4b0"	"00075a5cfb"	-0.0084
"000757e4b0"	"00075a6151"	-0.1711
"000757e4b0"	"00075a6708"	-0.0315
"000757e4b0"	"00075a7319"	-0.1546
"000757e4b0"	"00075a7723"	-0.0069
"000757e4b0"	"00075a778b"	-0.0951
"000757e4b0"	"00075a7b8e"	0.1199
"000757e4b0"	"00075a7c79"	0.2769
"000757e4b0"	"00075a81b6"	0.0249
"000757e4b0"	"00075a82ac"	0.2856
"000757e4b0"	"00075a98e5"	-0.1626
"000757e4b0"	"00075b0d29"	-0.1052
"000757e4b0"	"00075b102a"	0.1898
"000757e4b0"	"00075b1074"	0.2333
"000757e4b0"	"00075b135d"	0.0344
"000757e4b0"	"00075b138b"	0.6258
"000757e4b0"	"00075b13a0"	-0.3573
"000757e4b0"	"00075b13bd"	0.069
"000757e4b0"	"00075b16a9"	-0.2016
"000757e4b0"	"00075b1a28"	-0.2173
"000757e4b0"	"00075b1a97"	0.0876
"000757e4b0"	"00075b1c7b"	-0.4213
"000757e4b0"	"00075b1d24"	0.0265
"000757e4b0"	"00075b202b"	0.3657
"000757e4b0"	"00075b22cb"	-0.0702
"000757e4b0"	"00075b22da"	0.2858
"000757e4b0"	"00075b2556"	0.1463
"000757e4b0"	"00075b25de"	0.1538
"000757e4b0"	"00075b260c"	0.0752

"000757e4b0"	"00075b26f1"	0.1824
"000757e4b0"	"00075b2920"	0.0613
"000757e4b0"	"00075b2a64"	0.1093
"000757e4b0"	"00075b2a9d"	0.3368
"000757e4b0"	"00075b2b37"	-0.2457
"000757e4b0"	"00075b2cdd"	-0.0832
"000757e4b0"	"00075b3038"	0.3024
"000757e4b0"	"00075b30fe"	0.3052
"000757e4b0"	"00075b3362"	-0.1773
"000757e4b0"	"00075b350a"	0.0849
"000757e4b0"	"00075b350e"	0.1126
"000757e4b0"	"00075b3651"	-9e-04
"000757e4b0"	"00075b38ca"	0.1219
"000757e4b0"	"00075b39cc"	0.0692
"000757e4b0"	"00075b3e1e"	0.1469
"000757e4b0"	"00075b3e57"	-0.1281
"000757e4b0"	"00075b4079"	-0.1627
"000757e4b0"	"00075b4150"	-0.1631
"000757e4b0"	"00075b4194"	0.3464
"000757e4b0"	"00075b42d5"	0.2932
"000757e4b0"	"00075b4424"	0.153
"000757e4b0"	"00075b4470"	0.5263
"000757e4b0"	"00075b47ed"	0.1255
"000757e4b0"	"00075b4850"	0.6307
"000757e4b0"	"00075b4ca0"	0.2738
"000757e4b0"	"00075b4d7f"	0.1754
"000757e4b0"	"00075b520f"	0.121
"000757e4b0"	"00075b525f"	0.0483
"000757e4b0"	"00075b58f8"	-0.0219
"000757e4b0"	"00075b5bcc"	0.1437
"000757e4b0"	"00075b5bfa"	-0.0597
"000757e4b0"	"00075b6339"	0.09
"000757e4b0"	"00075b6658"	-0.0706
"000757e4b0"	"00075b679a"	0.0179
"000757e4b0"	"00075b6cb7"	0.1843
"000757e4b0"	"00075b6df8"	-0.0395
"000757e4b0"	"00075b6ff6"	0.2651
"000757e4b0"	"00075b70ee"	0.0065
"000757e4b0"	"00075b7157"	0.0226
"000757e4b0"	"00075b7225"	0.1114
"000757e4b0"	"00075b7c89"	0.0291
"000757e4b0"	"00075b9048"	0.1662
"000757e4b0"	"00075d0801"	0.0714
"000757e4b0"	"00075d1820"	-0.0466
"000757e4b0"	"00075d1f3d"	0.5195
"000757e4b0"	"00075d2329"	0.1889
"000757e4b0"	"00075d2b9b"	0.3533
"000757e4b0"	"00075d3941"	0.1665
"000757e4b0"	"00075d3e96"	0.26
"000757e4b0"	"00075d4864"	0.0998

"000757e4b0"	"00075d5961"	−0.0413
"000757e4b0"	"00075d5a63"	0.327
"000757e4b0"	"00075d6150"	−0.0612
"000757e4b0"	"00075d67d0"	0.0555
"000757e4b0"	"00075d67e2"	0.0507
"000757e4b0"	"00075d73fc"	−0.0137
"000757e4b0"	"00075d7729"	0.0364
"000757e4b0"	"00075d778c"	−0.1395
"000757e4b0"	"00075d7b9e"	−0.0229
"000757e4b0"	"00075d7c8f"	0.1953
"000757e4b0"	"00075d804d"	0.3678
"000757e4b0"	"00075d819f"	0.3687
"000757e4b0"	"00075d8601"	−0.0415
"000757e4b0"	"00075d8c6a"	0.0109
"000757e4b0"	"00075dfedc"	0.0247
"000757e4b0"	"00075e05f2"	0.0116
"000757e4b0"	"00075e0837"	0.4201
"000757e4b0"	"00075e092e"	0.0952
"000757e4b0"	"00075e0965"	−0.2041
"000757e4b0"	"00075e0bc8"	0.5106
"000757e4b0"	"00075e0fbb"	0.1272
"000757e7a0"	"000757e8b3"	−0.1082
"000757e7a0"	"000757f627"	−0.1804
"000757e7a0"	"000757f925"	−0.0279
"000757e7a0"	"000757fa08"	−0.1326
"000757e7a0"	"000757fe52"	−0.0865
"000757e7a0"	"000758024a"	−0.1342
"000757e7a0"	"00075804bb"	−0.17
"000757e7a0"	"00075a0c04"	−0.0897
"000757e7a0"	"00075a3110"	−0.0927
"000757e7a0"	"00075a341a"	−0.0334
"000757e7a0"	"00075a3dcf"	0.1437
"000757e7a0"	"00075a3e22"	−0.3646
"000757e7a0"	"00075a48d8"	−0.221
"000757e7a0"	"00075a5cfb"	0.0106
"000757e7a0"	"00075a6151"	−0.0563
"000757e7a0"	"00075a6708"	0.0879
"000757e7a0"	"00075a7319"	0.1618
"000757e7a0"	"00075a7723"	−0.0907
"000757e7a0"	"00075a778b"	−0.2013
"000757e7a0"	"00075a7b8e"	0.1366
"000757e7a0"	"00075a7c79"	0.1008
"000757e7a0"	"00075a81b6"	−0.235
"000757e7a0"	"00075a82ac"	0.0775
"000757e7a0"	"00075a98e5"	−0.4583
"000757e7a0"	"00075b0d29"	−0.29
"000757e7a0"	"00075b102a"	−0.0803
"000757e7a0"	"00075b1074"	−0.0928
"000757e7a0"	"00075b135d"	−0.1628
"000757e7a0"	"00075b138b"	−0.0033

"000757e7a0"	"00075b13a0"	-0.4026
"000757e7a0"	"00075b13bd"	-0.0549
"000757e7a0"	"00075b16a9"	0.297
"000757e7a0"	"00075b1a28"	-0.047
"000757e7a0"	"00075b1a97"	-0.0221
"000757e7a0"	"00075b1c7b"	0.219
"000757e7a0"	"00075b1d24"	0.1626
"000757e7a0"	"00075b202b"	-0.0114
"000757e7a0"	"00075b22cb"	0.036
"000757e7a0"	"00075b22da"	-0.0753
"000757e7a0"	"00075b2556"	-0.0475
"000757e7a0"	"00075b25de"	0.1585
"000757e7a0"	"00075b260c"	-0.1826
"000757e7a0"	"00075b26f1"	0.0438
"000757e7a0"	"00075b2920"	0.1042
"000757e7a0"	"00075b2a64"	-0.1259
"000757e7a0"	"00075b2a9d"	0.0262
"000757e7a0"	"00075b2b37"	0.1807
"000757e7a0"	"00075b2cdd"	-0.0051
"000757e7a0"	"00075b3038"	0.0448
"000757e7a0"	"00075b30fe"	0.0112
"000757e7a0"	"00075b3362"	-0.0856
"000757e7a0"	"00075b350a"	-0.2715
"000757e7a0"	"00075b350e"	-0.2172
"000757e7a0"	"00075b3651"	0.0807
"000757e7a0"	"00075b38ca"	-0.055
"000757e7a0"	"00075b39cc"	0.1448
"000757e7a0"	"00075b3e1e"	-0.1675
"000757e7a0"	"00075b3e57"	0.1697
"000757e7a0"	"00075b4079"	-0.2217
"000757e7a0"	"00075b4150"	0.0352
"000757e7a0"	"00075b4194"	0.002
"000757e7a0"	"00075b42d5"	0.1136
"000757e7a0"	"00075b4424"	0.2983
"000757e7a0"	"00075b4470"	-0.0451
"000757e7a0"	"00075b47ed"	0.0451
"000757e7a0"	"00075b4850"	0.1123
"000757e7a0"	"00075b4ca0"	-0.0438
"000757e7a0"	"00075b4d7f"	0.0398
"000757e7a0"	"00075b520f"	0.0101
"000757e7a0"	"00075b525f"	-0.0111
"000757e7a0"	"00075b58f8"	0.2342
"000757e7a0"	"00075b5bcc"	-0.0994
"000757e7a0"	"00075b5bfa"	-0.1323
"000757e7a0"	"00075b6339"	0.0709
"000757e7a0"	"00075b6658"	0.041
"000757e7a0"	"00075b679a"	0.0072
"000757e7a0"	"00075b6cb7"	-0.0162
"000757e7a0"	"00075b6df8"	-0.0101
"000757e7a0"	"00075b6ff6"	0.079

"000757e7a0"	"00075b70ee"	0.103
"000757e7a0"	"00075b7157"	0.0252
"000757e7a0"	"00075b7225"	-0.1437
"000757e7a0"	"00075b7c89"	-0.0773
"000757e7a0"	"00075b9048"	-0.0871
"000757e7a0"	"00075d0801"	-0.0156
"000757e7a0"	"00075d1820"	0.3373
"000757e7a0"	"00075d1f3d"	-0.0264
"000757e7a0"	"00075d2329"	0.0769
"000757e7a0"	"00075d2b9b"	-0.4001
"000757e7a0"	"00075d3941"	-0.2388
"000757e7a0"	"00075d3e96"	0.4327
"000757e7a0"	"00075d4864"	-0.0814
"000757e7a0"	"00075d5961"	-0.0855
"000757e7a0"	"00075d5a63"	0.0832
"000757e7a0"	"00075d6150"	-0.0121
"000757e7a0"	"00075d67d0"	-0.1312
"000757e7a0"	"00075d67e2"	-0.3087
"000757e7a0"	"00075d73fc"	-0.1045
"000757e7a0"	"00075d7729"	-0.0931
"000757e7a0"	"00075d778c"	0.0847
"000757e7a0"	"00075d7b9e"	-0.0272
"000757e7a0"	"00075d7c8f"	0.012
"000757e7a0"	"00075d804d"	0.086
"000757e7a0"	"00075d819f"	-0.1484
"000757e7a0"	"00075d8601"	0.074
"000757e7a0"	"00075d8c6a"	-0.0868
"000757e7a0"	"00075dfedc"	0.1232
"000757e7a0"	"00075e05f2"	-0.0866
"000757e7a0"	"00075e0837"	0.1008
"000757e7a0"	"00075e092e"	-0.1039
"000757e7a0"	"00075e0965"	-0.0636
"000757e7a0"	"00075e0bc8"	0.078
"000757e7a0"	"00075e0fbb"	-0.1507
"000757e8b3"	"000757f627"	0.0833
"000757e8b3"	"000757f925"	0.3033
"000757e8b3"	"000757fa08"	-0.0737
"000757e8b3"	"000757fe52"	0.3544
"000757e8b3"	"000758024a"	0.0313
"000757e8b3"	"00075804bb"	0.1453
"000757e8b3"	"00075a0c04"	0.2218
"000757e8b3"	"00075a3110"	0.1138
"000757e8b3"	"00075a341a"	0.1112
"000757e8b3"	"00075a3dcf"	-0.0092
"000757e8b3"	"00075a3e22"	0.3879
"000757e8b3"	"00075a48d8"	0.3819
"000757e8b3"	"00075a5cfb"	0.2194
"000757e8b3"	"00075a6151"	0.2219
"000757e8b3"	"00075a6708"	-0.254
"000757e8b3"	"00075a7319"	-0.313

"000757e8b3"	"00075a7723"	0.124
"000757e8b3"	"00075a778b"	-0.0301
"000757e8b3"	"00075a7b8e"	-0.1239
"000757e8b3"	"00075a7c79"	0.3689
"000757e8b3"	"00075a81b6"	0.2779
"000757e8b3"	"00075a82ac"	0.1351
"000757e8b3"	"00075a98e5"	0.1191
"000757e8b3"	"00075b0d29"	0.2409
"000757e8b3"	"00075b102a"	0.2508
"000757e8b3"	"00075b1074"	-0.1266
"000757e8b3"	"00075b135d"	0.2354
"000757e8b3"	"00075b138b"	0.3613
"000757e8b3"	"00075b13a0"	-0.0241
"000757e8b3"	"00075b13bd"	0.0085
"000757e8b3"	"00075b16a9"	0.2828
"000757e8b3"	"00075b1a28"	-0.0729
"000757e8b3"	"00075b1a97"	0.0699
"000757e8b3"	"00075b1c7b"	-0.0202
"000757e8b3"	"00075b1d24"	0.2032
"000757e8b3"	"00075b202b"	0.0583
"000757e8b3"	"00075b22cb"	0.1732
"000757e8b3"	"00075b22da"	0.0779
"000757e8b3"	"00075b2556"	0.051
"000757e8b3"	"00075b25de"	0.048
"000757e8b3"	"00075b260c"	0.1567
"000757e8b3"	"00075b26f1"	0.0426
"000757e8b3"	"00075b2920"	0.1028
"000757e8b3"	"00075b2a64"	0.028
"000757e8b3"	"00075b2a9d"	0.0218
"000757e8b3"	"00075b2b37"	-0.2848
"000757e8b3"	"00075b2cdd"	0.0985
"000757e8b3"	"00075b3038"	0.0334
"000757e8b3"	"00075b30fe"	-0.1067
"000757e8b3"	"00075b3362"	0.0775
"000757e8b3"	"00075b350a"	0.0707
"000757e8b3"	"00075b350e"	-5e-04
"000757e8b3"	"00075b3651"	-0.0898
"000757e8b3"	"00075b38ca"	-0.2857
"000757e8b3"	"00075b39cc"	0.1977
"000757e8b3"	"00075b3e1e"	0.1747
"000757e8b3"	"00075b3e57"	0.0449
"000757e8b3"	"00075b4079"	0.1032
"000757e8b3"	"00075b4150"	0.0028
"000757e8b3"	"00075b4194"	0.3028
"000757e8b3"	"00075b42d5"	-0.0797
"000757e8b3"	"00075b4424"	-0.0514
"000757e8b3"	"00075b4470"	0.2745
"000757e8b3"	"00075b47ed"	0.0717
"000757e8b3"	"00075b4850"	0.2514
"000757e8b3"	"00075b4ca0"	0.1812

"000757e8b3"	"00075b4d7f"	0.4112
"000757e8b3"	"00075b520f"	-0.1044
"000757e8b3"	"00075b525f"	0.173
"000757e8b3"	"00075b58f8"	-0.0643
"000757e8b3"	"00075b5bcc"	0.1068
"000757e8b3"	"00075b5bfa"	-0.2697
"000757e8b3"	"00075b6339"	0.5741
"000757e8b3"	"00075b6658"	-0.0114
"000757e8b3"	"00075b679a"	0.2475
"000757e8b3"	"00075b6cb7"	0.1888
"000757e8b3"	"00075b6df8"	0.0282
"000757e8b3"	"00075b6ff6"	0.2307
"000757e8b3"	"00075b70ee"	-0.0375
"000757e8b3"	"00075b7157"	0.1811
"000757e8b3"	"00075b7225"	0.1723
"000757e8b3"	"00075b7c89"	0.2158
"000757e8b3"	"00075b9048"	0.118
"000757e8b3"	"00075d0801"	0.207
"000757e8b3"	"00075d1820"	-0.1247
"000757e8b3"	"00075d1f3d"	0.1531
"000757e8b3"	"00075d2329"	-0.1189
"000757e8b3"	"00075d2b9b"	0.1469
"000757e8b3"	"00075d3941"	0.1399
"000757e8b3"	"00075d3e96"	0.0592
"000757e8b3"	"00075d4864"	-0.0697
"000757e8b3"	"00075d5961"	-0.057
"000757e8b3"	"00075d5a63"	0.2071
"000757e8b3"	"00075d6150"	-0.1533
"000757e8b3"	"00075d67d0"	-0.0183
"000757e8b3"	"00075d67e2"	0.461
"000757e8b3"	"00075d73fc"	0.3029
"000757e8b3"	"00075d7729"	-0.0845
"000757e8b3"	"00075d778c"	-0.008
"000757e8b3"	"00075d7b9e"	0.1678
"000757e8b3"	"00075d7c8f"	0.5077
"000757e8b3"	"00075d804d"	0.1858
"000757e8b3"	"00075d819f"	0.156
"000757e8b3"	"00075d8601"	0.0104
"000757e8b3"	"00075d8c6a"	0.1289
"000757e8b3"	"00075dfedc"	0.4417
"000757e8b3"	"00075e05f2"	0.2861
"000757e8b3"	"00075e0837"	0.1143
"000757e8b3"	"00075e092e"	-0.1038
"000757e8b3"	"00075e0965"	-0.1346
"000757e8b3"	"00075e0bc8"	0.2052
"000757e8b3"	"00075e0fbb"	-0.0956
"000757f627"	"000757f925"	0.1184
"000757f627"	"000757fa08"	-0.33
"000757f627"	"000757fe52"	-0.2007
"000757f627"	"000758024a"	0.0912

"000757f627"	"00075804bb"	0.2159
"000757f627"	"00075a0c04"	−0.1007
"000757f627"	"00075a3110"	−0.0663
"000757f627"	"00075a341a"	−0.1894
"000757f627"	"00075a3dcf"	−0.053
"000757f627"	"00075a3e22"	0.2684
"000757f627"	"00075a48d8"	0.0027
"000757f627"	"00075a5cfb"	0.089
"000757f627"	"00075a6151"	0.1966
"000757f627"	"00075a6708"	0.1188
"000757f627"	"00075a7319"	0.2318
"000757f627"	"00075a7723"	0.0224
"000757f627"	"00075a778b"	−0.0081
"000757f627"	"00075a7b8e"	0.3053
"000757f627"	"00075a7c79"	0.0994
"000757f627"	"00075a81b6"	0.1075
"000757f627"	"00075a82ac"	0.2291
"000757f627"	"00075a98e5"	0.3698
"000757f627"	"00075b0d29"	0.2239
"000757f627"	"00075b102a"	0.0597
"000757f627"	"00075b1074"	−0.3458
"000757f627"	"00075b135d"	0.0435
"000757f627"	"00075b138b"	0.2913
"000757f627"	"00075b13a0"	−0.0973
"000757f627"	"00075b13bd"	0.2384
"000757f627"	"00075b16a9"	−0.0026
"000757f627"	"00075b1a28"	−0.0406
"000757f627"	"00075b1a97"	0.2407
"000757f627"	"00075b1c7b"	−0.2913
"000757f627"	"00075b1d24"	−0.0461
"000757f627"	"00075b202b"	−0.0266
"000757f627"	"00075b22cb"	0.0364
"000757f627"	"00075b22da"	−0.1212
"000757f627"	"00075b2556"	−0.1402
"000757f627"	"00075b25de"	0.0316
"000757f627"	"00075b260c"	0.0495
"000757f627"	"00075b26f1"	−0.0277
"000757f627"	"00075b2920"	0.1743
"000757f627"	"00075b2a64"	−0.0952
"000757f627"	"00075b2a9d"	0.0904
"000757f627"	"00075b2b37"	−0.2392
"000757f627"	"00075b2cdd"	−0.0745
"000757f627"	"00075b3038"	−0.065
"000757f627"	"00075b30fe"	−0.1803
"000757f627"	"00075b3362"	−0.3144
"000757f627"	"00075b350a"	−0.0191
"000757f627"	"00075b350e"	−0.0524
"000757f627"	"00075b3651"	−0.0803
"000757f627"	"00075b38ca"	−0.3041
"000757f627"	"00075b39cc"	0.0224

"000757f627"	"00075b3e1e"	0.1675
"000757f627"	"00075b3e57"	−0.0426
"000757f627"	"00075b4079"	−0.114
"000757f627"	"00075b4150"	−0.11
"000757f627"	"00075b4194"	0.3877
"000757f627"	"00075b42d5"	−0.0069
"000757f627"	"00075b4424"	−0.346
"000757f627"	"00075b4470"	0.239
"000757f627"	"00075b47ed"	−0.0578
"000757f627"	"00075b4850"	0.2552
"000757f627"	"00075b4ca0"	−0.0095
"000757f627"	"00075b4d7f"	0.1495
"000757f627"	"00075b520f"	0.1736
"000757f627"	"00075b525f"	−0.1449
"000757f627"	"00075b58f8"	−0.1496
"000757f627"	"00075b5bcc"	−0.0439
"000757f627"	"00075b5bfa"	−0.377
"000757f627"	"00075b6339"	0.0628
"000757f627"	"00075b6658"	−0.3032
"000757f627"	"00075b679a"	0.1813
"000757f627"	"00075b6cb7"	−0.0737
"000757f627"	"00075b6df8"	−0.1931
"000757f627"	"00075b6ff6"	−0.0318
"000757f627"	"00075b70ee"	−0.1348
"000757f627"	"00075b7157"	−0.0942
"000757f627"	"00075b7225"	−0.2281
"000757f627"	"00075b7c89"	−0.0249
"000757f627"	"00075b9048"	0.1536
"000757f627"	"00075d0801"	0.0083
"000757f627"	"00075d1820"	−0.0483
"000757f627"	"00075d1f3d"	0.0566
"000757f627"	"00075d2329"	−0.1405
"000757f627"	"00075d2b9b"	−0.0473
"000757f627"	"00075d3941"	−0.3274
"000757f627"	"00075d3e96"	−0.2444
"000757f627"	"00075d4864"	−0.1048
"000757f627"	"00075d5961"	0.2035
"000757f627"	"00075d5a63"	−0.0551
"000757f627"	"00075d6150"	0.022
"000757f627"	"00075d67d0"	−0.1626
"000757f627"	"00075d67e2"	0.1618
"000757f627"	"00075d73fc"	−0.0397
"000757f627"	"00075d7729"	−0.0859
"000757f627"	"00075d778c"	−0.4894
"000757f627"	"00075d7b9e"	0.0089
"000757f627"	"00075d7c8f"	0.0548
"000757f627"	"00075d804d"	0.284
"000757f627"	"00075d819f"	0.1116
"000757f627"	"00075d8601"	0.2176
"000757f627"	"00075d8c6a"	−0.0617

"000757f627"	"00075dfedc"	0.0011
"000757f627"	"00075e05f2"	0.0383
"000757f627"	"00075e0837"	−0.0177
"000757f627"	"00075e092e"	−0.0116
"000757f627"	"00075e0965"	−0.331
"000757f627"	"00075e0bc8"	0.1424
"000757f627"	"00075e0fbb"	−0.2936
"000757f925"	"000757fa08"	−0.0612
"000757f925"	"000757fe52"	0.0065
"000757f925"	"000758024a"	−0.0178
"000757f925"	"00075804bb"	0.1132
"000757f925"	"00075a0c04"	0.1058
"000757f925"	"00075a3110"	0.2261
"000757f925"	"00075a341a"	0.0636
"000757f925"	"00075a3dcf"	−0.0354
"000757f925"	"00075a3e22"	0.183
"000757f925"	"00075a48d8"	0.2885
"000757f925"	"00075a5cfb"	−0.0207
"000757f925"	"00075a6151"	0.2076
"000757f925"	"00075a6708"	0.0169
"000757f925"	"00075a7319"	−0.14
"000757f925"	"00075a7723"	0.3099
"000757f925"	"00075a778b"	0.0775
"000757f925"	"00075a7b8e"	0.022
"000757f925"	"00075a7c79"	0.0373
"000757f925"	"00075a81b6"	−0.1059
"000757f925"	"00075a82ac"	0.1147
"000757f925"	"00075a98e5"	0.2097
"000757f925"	"00075b0d29"	−0.0234
"000757f925"	"00075b102a"	0.2824
"000757f925"	"00075b1074"	−0.0309
"000757f925"	"00075b135d"	−0.1538
"000757f925"	"00075b138b"	0.1677
"000757f925"	"00075b13a0"	−0.0266
"000757f925"	"00075b13bd"	−0.1367
"000757f925"	"00075b16a9"	0.3527
"000757f925"	"00075b1a28"	−0.0624
"000757f925"	"00075b1a97"	−0.1154
"000757f925"	"00075b1c7b"	−0.2286
"000757f925"	"00075b1d24"	−0.0011
"000757f925"	"00075b202b"	0.0582
"000757f925"	"00075b22cb"	0.0011
"000757f925"	"00075b22da"	0.016
"000757f925"	"00075b2556"	−0.0074
"000757f925"	"00075b25de"	0.2134
"000757f925"	"00075b260c"	0.1466
"000757f925"	"00075b26f1"	0.0244
"000757f925"	"00075b2920"	0.2245
"000757f925"	"00075b2a64"	0.0902
"000757f925"	"00075b2a9d"	0.2163

"000757f925"	"00075b2b37"	−0.2025
"000757f925"	"00075b2cdd"	−0.0785
"000757f925"	"00075b3038"	0.0961
"000757f925"	"00075b30fe"	−0.0819
"000757f925"	"00075b3362"	0.0868
"000757f925"	"00075b350a"	−0.0679
"000757f925"	"00075b350e"	−0.0178
"000757f925"	"00075b3651"	0.0583
"000757f925"	"00075b38ca"	−0.2306
"000757f925"	"00075b39cc"	0.097
"000757f925"	"00075b3e1e"	0.2615
"000757f925"	"00075b3e57"	0.2032
"000757f925"	"00075b4079"	0.1665
"000757f925"	"00075b4150"	0.1873
"000757f925"	"00075b4194"	0.1892
"000757f925"	"00075b42d5"	−0.1484
"000757f925"	"00075b4424"	−0.0777
"000757f925"	"00075b4470"	0.045
"000757f925"	"00075b47ed"	0.1119
"000757f925"	"00075b4850"	0.0403
"000757f925"	"00075b4ca0"	−0.107
"000757f925"	"00075b4d7f"	0.1911
"000757f925"	"00075b520f"	−0.1072
"000757f925"	"00075b525f"	−0.0044
"000757f925"	"00075b58f8"	−0.0289
"000757f925"	"00075b5bcc"	−0.0075
"000757f925"	"00075b5bfa"	−0.0975
"000757f925"	"00075b6339"	0.3169
"000757f925"	"00075b6658"	−0.1527
"000757f925"	"00075b679a"	−0.0092
"000757f925"	"00075b6cb7"	0.0129
"000757f925"	"00075b6df8"	0.0379
"000757f925"	"00075b6ff6"	0.0936
"000757f925"	"00075b70ee"	−0.0518
"000757f925"	"00075b7157"	0.1372
"000757f925"	"00075b7225"	0.022
"000757f925"	"00075b7c89"	0.3239
"000757f925"	"00075b9048"	0.0928
"000757f925"	"00075d0801"	0.2466
"000757f925"	"00075d1820"	0.0854
"000757f925"	"00075d1f3d"	−0.0574
"000757f925"	"00075d2329"	−0.0968
"000757f925"	"00075d2b9b"	0.0182
"000757f925"	"00075d3941"	−0.079
"000757f925"	"00075d3e96"	0.1389
"000757f925"	"00075d4864"	−0.0613
"000757f925"	"00075d5961"	0.1296
"000757f925"	"00075d5a63"	0.0039
"000757f925"	"00075d6150"	−0.1655
"000757f925"	"00075d67d0"	0.0348

"000757f925"	"00075d67e2"	0.2062
"000757f925"	"00075d73fc"	-0.0841
"000757f925"	"00075d7729"	-0.3715
"000757f925"	"00075d778c"	-0.0337
"000757f925"	"00075d7b9e"	0.1112
"000757f925"	"00075d7c8f"	0.1248
"000757f925"	"00075d804d"	0.1058
"000757f925"	"00075d819f"	0.1247
"000757f925"	"00075d8601"	0.0313
"000757f925"	"00075d8c6a"	0.1669
"000757f925"	"00075dfedc"	0.1313
"000757f925"	"00075e05f2"	0.2517
"000757f925"	"00075e0837"	0.1259
"000757f925"	"00075e092e"	0.0249
"000757f925"	"00075e0965"	-0.1017
"000757f925"	"00075e0bc8"	-0.0016
"000757f925"	"00075e0fbb"	-0.1333
"000757fa08"	"000757fe52"	0.0045
"000757fa08"	"000758024a"	0.0977
"000757fa08"	"00075804bb"	-0.1696
"000757fa08"	"00075a0c04"	0.2001
"000757fa08"	"00075a3110"	0.1545
"000757fa08"	"00075a341a"	0.1138
"000757fa08"	"00075a3dcf"	0.056
"000757fa08"	"00075a3e22"	-0.0901
"000757fa08"	"00075a48d8"	0.1718
"000757fa08"	"00075a5cfb"	-0.0952
"000757fa08"	"00075a6151"	-0.2663
"000757fa08"	"00075a6708"	-0.046
"000757fa08"	"00075a7319"	-0.6023
"000757fa08"	"00075a7723"	0.2718
"000757fa08"	"00075a778b"	0.1245
"000757fa08"	"00075a7b8e"	-0.0761
"000757fa08"	"00075a7c79"	-0.1798
"000757fa08"	"00075a81b6"	-0.2684
"000757fa08"	"00075a82ac"	0.0019
"000757fa08"	"00075a98e5"	-0.2853
"000757fa08"	"00075b0d29"	0.0432
"000757fa08"	"00075b102a"	-0.0321
"000757fa08"	"00075b1074"	0.1732
"000757fa08"	"00075b135d"	-0.048
"000757fa08"	"00075b138b"	-0.1588
"000757fa08"	"00075b13a0"	-0.0592
"000757fa08"	"00075b13bd"	-0.2448
"000757fa08"	"00075b16a9"	-0.1005
"000757fa08"	"00075b1a28"	-0.0604
"000757fa08"	"00075b1a97"	-0.1944
"000757fa08"	"00075b1c7b"	-0.3414
"000757fa08"	"00075b1d24"	0.1101
"000757fa08"	"00075b202b"	-0.0553

"000757fa08"	"00075b22cb"	−0.1335
"000757fa08"	"00075b22da"	0.065
"000757fa08"	"00075b2556"	0.0762
"000757fa08"	"00075b25de"	−0.1146
"000757fa08"	"00075b260c"	−0.0807
"000757fa08"	"00075b26f1"	−0.2988
"000757fa08"	"00075b2920"	−0.1686
"000757fa08"	"00075b2a64"	−0.368
"000757fa08"	"00075b2a9d"	−0.1852
"000757fa08"	"00075b2b37"	−0.2269
"000757fa08"	"00075b2cdd"	−0.1555
"000757fa08"	"00075b3038"	−0.1283
"000757fa08"	"00075b30fe"	0.0812
"000757fa08"	"00075b3362"	−0.1819
"000757fa08"	"00075b350a"	−0.1422
"000757fa08"	"00075b350e"	−0.1166
"000757fa08"	"00075b3651"	−0.3851
"000757fa08"	"00075b38ca"	0.1
"000757fa08"	"00075b39cc"	−0.1977
"000757fa08"	"00075b3e1e"	0.0068
"000757fa08"	"00075b3e57"	−0.0376
"000757fa08"	"00075b4079"	−0.3778
"000757fa08"	"00075b4150"	0.154
"000757fa08"	"00075b4194"	−0.2251
"000757fa08"	"00075b42d5"	0.2066
"000757fa08"	"00075b4424"	−0.0739
"000757fa08"	"00075b4470"	0.0207
"000757fa08"	"00075b47ed"	−0.0657
"000757fa08"	"00075b4850"	−0.271
"000757fa08"	"00075b4ca0"	−0.3969
"000757fa08"	"00075b4d7f"	0.4725
"000757fa08"	"00075b520f"	−0.2247
"000757fa08"	"00075b525f"	−0.3067
"000757fa08"	"00075b58f8"	−0.0643
"000757fa08"	"00075b5bcc"	−0.1369
"000757fa08"	"00075b5bfa"	−0.3311
"000757fa08"	"00075b6339"	0.1386
"000757fa08"	"00075b6658"	−0.164
"000757fa08"	"00075b679a"	0.0397
"000757fa08"	"00075b6cb7"	−0.3972
"000757fa08"	"00075b6df8"	0.3789
"000757fa08"	"00075b6ff6"	0.0328
"000757fa08"	"00075b70ee"	0.0555
"000757fa08"	"00075b7157"	−0.1963
"000757fa08"	"00075b7225"	0.0451
"000757fa08"	"00075b7c89"	0.0485
"000757fa08"	"00075b9048"	−0.1012
"000757fa08"	"00075d0801"	−0.0703
"000757fa08"	"00075d1820"	−0.334
"000757fa08"	"00075d1f3d"	−0.2241

"000757fa08"	"00075d2329"	0.1067
"000757fa08"	"00075d2b9b"	−0.2421
"000757fa08"	"00075d3941"	−0.1842
"000757fa08"	"00075d3e96"	−0.1789
"000757fa08"	"00075d4864"	0.2524
"000757fa08"	"00075d5961"	0.1618
"000757fa08"	"00075d5a63"	0.2624
"000757fa08"	"00075d6150"	−0.1593
"000757fa08"	"00075d67d0"	−0.3168
"000757fa08"	"00075d67e2"	−0.2284
"000757fa08"	"00075d73fc"	0.0428
"000757fa08"	"00075d7729"	−0.0279
"000757fa08"	"00075d778c"	−0.0816
"000757fa08"	"00075d7b9e"	0.017
"000757fa08"	"00075d7c8f"	0.0841
"000757fa08"	"00075d804d"	−0.275
"000757fa08"	"00075d819f"	−0.3586
"000757fa08"	"00075d8601"	−0.2542
"000757fa08"	"00075d8c6a"	0.2355
"000757fa08"	"00075dfedc"	0.0448
"000757fa08"	"00075e05f2"	−0.3149
"000757fa08"	"00075e0837"	−0.219
"000757fa08"	"00075e092e"	−0.1199
"000757fa08"	"00075e0965"	0.1751
"000757fa08"	"00075e0bc8"	−0.0187
"000757fa08"	"00075e0fbb"	0.0484
"000757fe52"	"000758024a"	−0.0755
"000757fe52"	"00075804bb"	−0.1955
"000757fe52"	"00075a0c04"	−0.032
"000757fe52"	"00075a3110"	0.0996
"000757fe52"	"00075a341a"	0.1416
"000757fe52"	"00075a3dcf"	0.2052
"000757fe52"	"00075a3e22"	−0.048
"000757fe52"	"00075a48d8"	0.1775
"000757fe52"	"00075a5cfb"	0.2299
"000757fe52"	"00075a6151"	0.0633
"000757fe52"	"00075a6708"	−0.2229
"000757fe52"	"00075a7319"	−0.3611
"000757fe52"	"00075a7723"	−0.079
"000757fe52"	"00075a778b"	0.2542
"000757fe52"	"00075a7b8e"	−0.079
"000757fe52"	"00075a7c79"	0.2481
"000757fe52"	"00075a81b6"	−0.0441
"000757fe52"	"00075a82ac"	0.1984
"000757fe52"	"00075a98e5"	−0.0079
"000757fe52"	"00075b0d29"	0.1012
"000757fe52"	"00075b102a"	−0.1647
"000757fe52"	"00075b1074"	−0.272
"000757fe52"	"00075b135d"	−0.1717
"000757fe52"	"00075b138b"	0.1618

"000757fe52"	"00075b13a0"	0.5615
"000757fe52"	"00075b13bd"	-0.0217
"000757fe52"	"00075b16a9"	0.1199
"000757fe52"	"00075b1a28"	-0.0218
"000757fe52"	"00075b1a97"	-0.0041
"000757fe52"	"00075b1c7b"	0.0413
"000757fe52"	"00075b1d24"	0.0204
"000757fe52"	"00075b202b"	-0.1259
"000757fe52"	"00075b22cb"	0.2816
"000757fe52"	"00075b22da"	0.051
"000757fe52"	"00075b2556"	-0.0055
"000757fe52"	"00075b25de"	0.1857
"000757fe52"	"00075b260c"	0.0767
"000757fe52"	"00075b26f1"	-0.2882
"000757fe52"	"00075b2920"	-0.0574
"000757fe52"	"00075b2a64"	-0.0836
"000757fe52"	"00075b2a9d"	-0.3746
"000757fe52"	"00075b2b37"	-0.3458
"000757fe52"	"00075b2cdd"	0.0455
"000757fe52"	"00075b3038"	0.1422
"000757fe52"	"00075b30fe"	-0.0771
"000757fe52"	"00075b3362"	-0.1632
"000757fe52"	"00075b350a"	-0.0066
"000757fe52"	"00075b350e"	0.0926
"000757fe52"	"00075b3651"	-0.2759
"000757fe52"	"00075b38ca"	-0.1535
"000757fe52"	"00075b39cc"	-0.002
"000757fe52"	"00075b3e1e"	-0.0189
"000757fe52"	"00075b3e57"	0.2474
"000757fe52"	"00075b4079"	0.0322
"000757fe52"	"00075b4150"	0.1018
"000757fe52"	"00075b4194"	-0.0738
"000757fe52"	"00075b42d5"	0.0767
"000757fe52"	"00075b4424"	-0.0213
"000757fe52"	"00075b4470"	-0.064
"000757fe52"	"00075b47ed"	-0.0136
"000757fe52"	"00075b4850"	-0.1807
"000757fe52"	"00075b4ca0"	-0.2395
"000757fe52"	"00075b4d7f"	0.3382
"000757fe52"	"00075b520f"	-0.1023
"000757fe52"	"00075b525f"	-0.0374
"000757fe52"	"00075b58f8"	0.0187
"000757fe52"	"00075b5bcc"	0.1455
"000757fe52"	"00075b5bfa"	0.008
"000757fe52"	"00075b6339"	0.327
"000757fe52"	"00075b6658"	-0.0678
"000757fe52"	"00075b679a"	0.2336
"000757fe52"	"00075b6cb7"	-0.1161
"000757fe52"	"00075b6df8"	0.1581
"000757fe52"	"00075b6ff6"	0.3393

"000757fe52"	"00075b70ee"	-0.0955
"000757fe52"	"00075b7157"	0.1445
"000757fe52"	"00075b7225"	0.1939
"000757fe52"	"00075b7c89"	0.1745
"000757fe52"	"00075b9048"	0.189
"000757fe52"	"00075d0801"	0.1331
"000757fe52"	"00075d1820"	0.004
"000757fe52"	"00075d1f3d"	-0.0938
"000757fe52"	"00075d2329"	0.1126
"000757fe52"	"00075d2b9b"	0.0154
"000757fe52"	"00075d3941"	-0.1634
"000757fe52"	"00075d3e96"	-0.1456
"000757fe52"	"00075d4864"	0.0175
"000757fe52"	"00075d5961"	5e-04
"000757fe52"	"00075d5a63"	0.3376
"000757fe52"	"00075d6150"	-0.2338
"000757fe52"	"00075d67d0"	-0.2657
"000757fe52"	"00075d67e2"	0.2104
"000757fe52"	"00075d73fc"	0.2559
"000757fe52"	"00075d7729"	-0.209
"000757fe52"	"00075d778c"	0.021
"000757fe52"	"00075d7b9e"	0.0554
"000757fe52"	"00075d7c8f"	0.1661
"000757fe52"	"00075d804d"	0.1464
"000757fe52"	"00075d819f"	-0.3099
"000757fe52"	"00075d8601"	-0.0086
"000757fe52"	"00075d8c6a"	0.1004
"000757fe52"	"00075dfedc"	0.3076
"000757fe52"	"00075e05f2"	0.2764
"000757fe52"	"00075e0837"	-0.1573
"000757fe52"	"00075e092e"	-0.1424
"000757fe52"	"00075e0965"	0.128
"000757fe52"	"00075e0bc8"	0.1726
"000757fe52"	"00075e0fbb"	-0.0887
"000758024a"	"00075804bb"	0.1957
"000758024a"	"00075a0c04"	-0.1119
"000758024a"	"00075a3110"	-0.0923
"000758024a"	"00075a341a"	-0.2196
"000758024a"	"00075a3dcf"	-0.4371
"000758024a"	"00075a3e22"	-0.0424
"000758024a"	"00075a48d8"	-0.1369
"000758024a"	"00075a5cfb"	0.4006
"000758024a"	"00075a6151"	-0.02
"000758024a"	"00075a6708"	4e-04
"000758024a"	"00075a7319"	-0.1651
"000758024a"	"00075a7723"	0.067
"000758024a"	"00075a778b"	0.2025
"000758024a"	"00075a7b8e"	0.2351
"000758024a"	"00075a7c79"	-0.1314
"000758024a"	"00075a81b6"	-0.2559

"000758024a"	"00075a82ac"	-0.3232
"000758024a"	"00075a98e5"	-0.1911
"000758024a"	"00075b0d29"	0.2367
"000758024a"	"00075b102a"	0.0989
"000758024a"	"00075b1074"	-0.4176
"000758024a"	"00075b135d"	-0.0038
"000758024a"	"00075b138b"	0.0687
"000758024a"	"00075b13a0"	-0.3056
"000758024a"	"00075b13bd"	-0.3436
"000758024a"	"00075b16a9"	0.0128
"000758024a"	"00075b1a28"	-0.0586
"000758024a"	"00075b1a97"	-0.2408
"000758024a"	"00075b1c7b"	-0.1214
"000758024a"	"00075b1d24"	-0.065
"000758024a"	"00075b202b"	0.2979
"000758024a"	"00075b22cb"	-0.2308
"000758024a"	"00075b22da"	-0.301
"000758024a"	"00075b2556"	-0.2171
"000758024a"	"00075b25de"	-0.1513
"000758024a"	"00075b260c"	0.0429
"000758024a"	"00075b26f1"	-0.4315
"000758024a"	"00075b2920"	0.328
"000758024a"	"00075b2a64"	0.3137
"000758024a"	"00075b2a9d"	0.0205
"000758024a"	"00075b2b37"	-0.2153
"000758024a"	"00075b2cdd"	-0.1469
"000758024a"	"00075b3038"	-0.1557
"000758024a"	"00075b30fe"	-0.4221
"000758024a"	"00075b3362"	-0.4641
"000758024a"	"00075b350a"	-0.1253
"000758024a"	"00075b350e"	-0.2266
"000758024a"	"00075b3651"	-0.4767
"000758024a"	"00075b38ca"	-0.3688
"000758024a"	"00075b39cc"	-0.4144
"000758024a"	"00075b3e1e"	0.02
"000758024a"	"00075b3e57"	-0.0867
"000758024a"	"00075b4079"	-0.2424
"000758024a"	"00075b4150"	0.6816
"000758024a"	"00075b4194"	-0.1063
"000758024a"	"00075b42d5"	-0.1111
"000758024a"	"00075b4424"	-0.0516
"000758024a"	"00075b4470"	0.0396
"000758024a"	"00075b47ed"	-0.4881
"000758024a"	"00075b4850"	-0.0483
"000758024a"	"00075b4ca0"	-0.216
"000758024a"	"00075b4d7f"	0.0798
"000758024a"	"00075b520f"	-0.1276
"000758024a"	"00075b525f"	-0.3437
"000758024a"	"00075b58f8"	0.3007
"000758024a"	"00075b5bcc"	-0.1119

"000758024a"	"00075b5bfa"	-0.5622
"000758024a"	"00075b6339"	0.2082
"000758024a"	"00075b6658"	-0.6046
"000758024a"	"00075b679a"	-0.0135
"000758024a"	"00075b6cb7"	-0.0543
"000758024a"	"00075b6df8"	-0.2417
"000758024a"	"00075b6ff6"	0.0119
"000758024a"	"00075b70ee"	0.1553
"000758024a"	"00075b7157"	-0.2263
"000758024a"	"00075b7225"	-0.2068
"000758024a"	"00075b7c89"	-0.171
"000758024a"	"00075b9048"	0.1004
"000758024a"	"00075d0801"	-0.1348
"000758024a"	"00075d1820"	-0.2871
"000758024a"	"00075d1f3d"	1e-04
"000758024a"	"00075d2329"	-0.3293
"000758024a"	"00075d2b9b"	0.0995
"000758024a"	"00075d3941"	-0.4206
"000758024a"	"00075d3e96"	-0.3622
"000758024a"	"00075d4864"	-0.1607
"000758024a"	"00075d5961"	-0.0302
"000758024a"	"00075d5a63"	0.1459
"000758024a"	"00075d6150"	-0.4562
"000758024a"	"00075d67d0"	-0.2915
"000758024a"	"00075d67e2"	-0.0389
"000758024a"	"00075d73fc"	-0.1963
"000758024a"	"00075d7729"	-0.0816
"000758024a"	"00075d778c"	-0.4845
"000758024a"	"00075d7b9e"	-0.271
"000758024a"	"00075d7c8f"	-0.1413
"000758024a"	"00075d804d"	0.0164
"000758024a"	"00075d819f"	-0.2619
"000758024a"	"00075d8601"	-0.2495
"000758024a"	"00075d8c6a"	-0.007
"000758024a"	"00075dfedc"	-0.077
"000758024a"	"00075e05f2"	-0.0578
"000758024a"	"00075e0837"	-0.1143
"000758024a"	"00075e092e"	-0.3353
"000758024a"	"00075e0965"	0.4743
"000758024a"	"00075e0bc8"	0.2235
"000758024a"	"00075e0fbb"	-0.3538
"00075804bb"	"00075a0c04"	-0.3608
"00075804bb"	"00075a3110"	-0.0798
"00075804bb"	"00075a341a"	-0.4237
"00075804bb"	"00075a3dcf"	-0.3715
"00075804bb"	"00075a3e22"	-0.0618
"00075804bb"	"00075a48d8"	-0.2859
"00075804bb"	"00075a5cfb"	0.4753
"00075804bb"	"00075a6151"	0.2041
"00075804bb"	"00075a6708"	0.0787

"00075804bb"	"00075a7319"	−0.0516
"00075804bb"	"00075a7723"	0.1056
"00075804bb"	"00075a778b"	−0.232
"00075804bb"	"00075a7b8e"	−0.0969
"00075804bb"	"00075a7c79"	0.0431
"00075804bb"	"00075a81b6"	−0.2564
"00075804bb"	"00075a82ac"	−0.2289
"00075804bb"	"00075a98e5"	−0.2042
"00075804bb"	"00075b0d29"	−0.0484
"00075804bb"	"00075b102a"	0.3211
"00075804bb"	"00075b1074"	−0.2054
"00075804bb"	"00075b135d"	−0.0461
"00075804bb"	"00075b138b"	0.033
"00075804bb"	"00075b13a0"	−0.4746
"00075804bb"	"00075b13bd"	−0.358
"00075804bb"	"00075b16a9"	0.0193
"00075804bb"	"00075b1a28"	−0.0534
"00075804bb"	"00075b1a97"	−0.2943
"00075804bb"	"00075b1c7b"	−0.1948
"00075804bb"	"00075b1d24"	−0.2295
"00075804bb"	"00075b202b"	0.102
"00075804bb"	"00075b22cb"	−0.3238
"00075804bb"	"00075b22da"	−0.0707
"00075804bb"	"00075b2556"	0.0717
"00075804bb"	"00075b25de"	0.0516
"00075804bb"	"00075b260c"	0.2148
"00075804bb"	"00075b26f1"	−0.2295
"00075804bb"	"00075b2920"	0.5856
"00075804bb"	"00075b2a64"	0.3655
"00075804bb"	"00075b2a9d"	0.2259
"00075804bb"	"00075b2b37"	−0.1196
"00075804bb"	"00075b2cdd"	−0.345
"00075804bb"	"00075b3038"	0.0514
"00075804bb"	"00075b30fe"	−0.0997
"00075804bb"	"00075b3362"	−0.4929
"00075804bb"	"00075b350a"	0.1827
"00075804bb"	"00075b350e"	−0.1853
"00075804bb"	"00075b3651"	−0.4008
"00075804bb"	"00075b38ca"	−0.1276
"00075804bb"	"00075b39cc"	−0.1923
"00075804bb"	"00075b3e1e"	0.0981
"00075804bb"	"00075b3e57"	−0.2563
"00075804bb"	"00075b4079"	0.0781
"00075804bb"	"00075b4150"	0.2002
"00075804bb"	"00075b4194"	0.0759
"00075804bb"	"00075b42d5"	−0.0242
"00075804bb"	"00075b4424"	−0.1792
"00075804bb"	"00075b4470"	0.3915
"00075804bb"	"00075b47ed"	−0.4539
"00075804bb"	"00075b4850"	0.3481

"00075804bb"	"00075b4ca0"	−0.1247
"00075804bb"	"00075b4d7f"	−0.1164
"00075804bb"	"00075b520f"	−0.0918
"00075804bb"	"00075b525f"	−0.2626
"00075804bb"	"00075b58f8"	0.237
"00075804bb"	"00075b5bcc"	0.0462
"00075804bb"	"00075b5bfa"	−0.3232
"00075804bb"	"00075b6339"	0.1533
"00075804bb"	"00075b6658"	−0.5738
"00075804bb"	"00075b679a"	−0.005
"00075804bb"	"00075b6cb7"	0.1483
"00075804bb"	"00075b6df8"	−0.4169
"00075804bb"	"00075b6ff6"	0.1468
"00075804bb"	"00075b70ee"	0.2982
"00075804bb"	"00075b7157"	−0.16
"00075804bb"	"00075b7225"	−0.3132
"00075804bb"	"00075b7c89"	−0.147
"00075804bb"	"00075b9048"	0.1219
"00075804bb"	"00075d0801"	0.0999
"00075804bb"	"00075d1820"	−0.5425
"00075804bb"	"00075d1f3d"	0.2104
"00075804bb"	"00075d2329"	−0.2951
"00075804bb"	"00075d2b9b"	0.2496
"00075804bb"	"00075d3941"	−0.2331
"00075804bb"	"00075d3e96"	−0.0967
"00075804bb"	"00075d4864"	−0.0113
"00075804bb"	"00075d5961"	−0.1237
"00075804bb"	"00075d5a63"	0.0453
"00075804bb"	"00075d6150"	−0.1511
"00075804bb"	"00075d67d0"	−0.2309
"00075804bb"	"00075d67e2"	−0.111
"00075804bb"	"00075d73fc"	−0.3546
"00075804bb"	"00075d7729"	−0.3898
"00075804bb"	"00075d778c"	−0.5944
"00075804bb"	"00075d7b9e"	−0.2533
"00075804bb"	"00075d7c8f"	−0.0812
"00075804bb"	"00075d804d"	0.2336
"00075804bb"	"00075d819f"	0.2713
"00075804bb"	"00075d8601"	−0.0996
"00075804bb"	"00075d8c6a"	−0.1898
"00075804bb"	"00075dfedc"	−0.0133
"00075804bb"	"00075e05f2"	0.0089
"00075804bb"	"00075e0837"	0.1729
"00075804bb"	"00075e092e"	−0.2372
"00075804bb"	"00075e0965"	0.3015
"00075804bb"	"00075e0bc8"	0.0052
"00075804bb"	"00075e0fbb"	−0.2233
"00075a0c04"	"00075a3110"	0.2932
"00075a0c04"	"00075a341a"	0.3579
"00075a0c04"	"00075a3dcf"	0.0095

"00075a0c04"	"00075a3e22"	0.6191
"00075a0c04"	"00075a48d8"	0.4272
"00075a0c04"	"00075a5cfb"	−0.2689
"00075a0c04"	"00075a6151"	−0.3727
"00075a0c04"	"00075a6708"	−0.2082
"00075a0c04"	"00075a7319"	−0.3203
"00075a0c04"	"00075a7723"	0.1802
"00075a0c04"	"00075a778b"	0.1681
"00075a0c04"	"00075a7b8e"	0.0196
"00075a0c04"	"00075a7c79"	0.2481
"00075a0c04"	"00075a81b6"	0.1612
"00075a0c04"	"00075a82ac"	0.2745
"00075a0c04"	"00075a98e5"	0.0449
"00075a0c04"	"00075b0d29"	0.0682
"00075a0c04"	"00075b102a"	0.0745
"00075a0c04"	"00075b1074"	0.152
"00075a0c04"	"00075b135d"	0.2107
"00075a0c04"	"00075b138b"	0.344
"00075a0c04"	"00075b13a0"	−0.1948
"00075a0c04"	"00075b13bd"	0.126
"00075a0c04"	"00075b16a9"	0.1571
"00075a0c04"	"00075b1a28"	−0.2031
"00075a0c04"	"00075b1a97"	0.1682
"00075a0c04"	"00075b1c7b"	−0.3447
"00075a0c04"	"00075b1d24"	0.0477
"00075a0c04"	"00075b202b"	0.233
"00075a0c04"	"00075b22cb"	0.009
"00075a0c04"	"00075b22da"	0.0773
"00075a0c04"	"00075b2556"	0.0127
"00075a0c04"	"00075b25de"	−0.0066
"00075a0c04"	"00075b260c"	−0.0061
"00075a0c04"	"00075b26f1"	0.1263
"00075a0c04"	"00075b2920"	−0.2648
"00075a0c04"	"00075b2a64"	−0.0628
"00075a0c04"	"00075b2a9d"	−0.025
"00075a0c04"	"00075b2b37"	−0.3021
"00075a0c04"	"00075b2cdd"	0.22
"00075a0c04"	"00075b3038"	0.2054
"00075a0c04"	"00075b30fe"	−0.0948
"00075a0c04"	"00075b3362"	−0.1227
"00075a0c04"	"00075b350a"	−0.0153
"00075a0c04"	"00075b350e"	−0.0344
"00075a0c04"	"00075b3651"	−0.0869
"00075a0c04"	"00075b38ca"	0.0815
"00075a0c04"	"00075b39cc"	0.0766
"00075a0c04"	"00075b3e1e"	0.3201
"00075a0c04"	"00075b3e57"	0.1296
"00075a0c04"	"00075b4079"	−0.1566
"00075a0c04"	"00075b4150"	−0.1722
"00075a0c04"	"00075b4194"	0.1066

"00075a0c04"	"00075b42d5"	0.0916
"00075a0c04"	"00075b4424"	-0.0587
"00075a0c04"	"00075b4470"	0.0486
"00075a0c04"	"00075b47ed"	0.2378
"00075a0c04"	"00075b4850"	0.0733
"00075a0c04"	"00075b4ca0"	0.0411
"00075a0c04"	"00075b4d7f"	0.5194
"00075a0c04"	"00075b520f"	-0.084
"00075a0c04"	"00075b525f"	-0.0377
"00075a0c04"	"00075b58f8"	-0.3939
"00075a0c04"	"00075b5bcc"	0.092
"00075a0c04"	"00075b5bfa"	-0.2463
"00075a0c04"	"00075b6339"	0.2132
"00075a0c04"	"00075b6658"	0.0751
"00075a0c04"	"00075b679a"	-0.0034
"00075a0c04"	"00075b6cb7"	-0.2479
"00075a0c04"	"00075b6df8"	0.5048
"00075a0c04"	"00075b6ff6"	0.0784
"00075a0c04"	"00075b70ee"	2e-04
"00075a0c04"	"00075b7157"	0.069
"00075a0c04"	"00075b7225"	0.24
"00075a0c04"	"00075b7c89"	0.298
"00075a0c04"	"00075b9048"	0.1655
"00075a0c04"	"00075d0801"	-0.0636
"00075a0c04"	"00075d1820"	-0.0306
"00075a0c04"	"00075d1f3d"	0.0382
"00075a0c04"	"00075d2329"	0.1769
"00075a0c04"	"00075d2b9b"	-0.0495
"00075a0c04"	"00075d3941"	0.2507
"00075a0c04"	"00075d3e96"	-0.1328
"00075a0c04"	"00075d4864"	-0.277
"00075a0c04"	"00075d5961"	0.326
"00075a0c04"	"00075d5a63"	0.1446
"00075a0c04"	"00075d6150"	-0.0587
"00075a0c04"	"00075d67d0"	-0.0836
"00075a0c04"	"00075d67e2"	0.0639
"00075a0c04"	"00075d73fc"	0.1962
"00075a0c04"	"00075d7729"	0.1772
"00075a0c04"	"00075d778c"	0.009
"00075a0c04"	"00075d7b9e"	0.1119
"00075a0c04"	"00075d7c8f"	0.4317
"00075a0c04"	"00075d804d"	0.0673
"00075a0c04"	"00075d819f"	-0.051
"00075a0c04"	"00075d8601"	0.1398
"00075a0c04"	"00075d8c6a"	0.3847
"00075a0c04"	"00075dfedc"	0.3526
"00075a0c04"	"00075e05f2"	-0.2049
"00075a0c04"	"00075e0837"	0.0369
"00075a0c04"	"00075e092e"	0.0633
"00075a0c04"	"00075e0965"	-0.4431

"00075a0c04"	"00075e0bc8"	0.1911
"00075a0c04"	"00075e0fbb"	0.0231
"00075a3110"	"00075a341a"	0.1593
"00075a3110"	"00075a3dcf"	−0.0886
"00075a3110"	"00075a3e22"	0.4039
"00075a3110"	"00075a48d8"	0.2308
"00075a3110"	"00075a5cfb"	−0.011
"00075a3110"	"00075a6151"	−0.1616
"00075a3110"	"00075a6708"	0.2479
"00075a3110"	"00075a7319"	−0.1888
"00075a3110"	"00075a7723"	−0.1671
"00075a3110"	"00075a778b"	0.0292
"00075a3110"	"00075a7b8e"	0.0481
"00075a3110"	"00075a7c79"	0.4066
"00075a3110"	"00075a81b6"	−0.0631
"00075a3110"	"00075a82ac"	0.1169
"00075a3110"	"00075a98e5"	−0.2781
"00075a3110"	"00075b0d29"	0.0579
"00075a3110"	"00075b102a"	0.1157
"00075a3110"	"00075b1074"	0.0711
"00075a3110"	"00075b135d"	0.113
"00075a3110"	"00075b138b"	0.2593
"00075a3110"	"00075b13a0"	0.0209
"00075a3110"	"00075b13bd"	0
"00075a3110"	"00075b16a9"	−0.1902
"00075a3110"	"00075b1a28"	−0.2087
"00075a3110"	"00075b1a97"	0.0286
"00075a3110"	"00075b1c7b"	−0.4922
"00075a3110"	"00075b1d24"	0.0155
"00075a3110"	"00075b202b"	0.2603
"00075a3110"	"00075b22cb"	−0.21
"00075a3110"	"00075b22da"	0.1916
"00075a3110"	"00075b2556"	0.0119
"00075a3110"	"00075b25de"	0.0933
"00075a3110"	"00075b260c"	0.3444
"00075a3110"	"00075b26f1"	−0.1659
"00075a3110"	"00075b2920"	0.1041
"00075a3110"	"00075b2a64"	0.1194
"00075a3110"	"00075b2a9d"	0.0739
"00075a3110"	"00075b2b37"	−0.2754
"00075a3110"	"00075b2cdd"	0.0748
"00075a3110"	"00075b3038"	0.2234
"00075a3110"	"00075b30fe"	0.18
"00075a3110"	"00075b3362"	−0.199
"00075a3110"	"00075b350a"	−0.0083
"00075a3110"	"00075b350e"	−0.0501
"00075a3110"	"00075b3651"	−0.1421
"00075a3110"	"00075b38ca"	−0.2249
"00075a3110"	"00075b39cc"	−0.0748
"00075a3110"	"00075b3e1e"	−0.0761

"00075a3110"	"00075b3e57"	0.0946
"00075a3110"	"00075b4079"	-0.1582
"00075a3110"	"00075b4150"	0.0235
"00075a3110"	"00075b4194"	0.271
"00075a3110"	"00075b42d5"	0.1012
"00075a3110"	"00075b4424"	-0.0845
"00075a3110"	"00075b4470"	0.0623
"00075a3110"	"00075b47ed"	-0.0619
"00075a3110"	"00075b4850"	0.1401
"00075a3110"	"00075b4ca0"	0.0908
"00075a3110"	"00075b4d7f"	0.3028
"00075a3110"	"00075b520f"	-0.1781
"00075a3110"	"00075b525f"	0.0463
"00075a3110"	"00075b58f8"	-0.11
"00075a3110"	"00075b5bcc"	0.0012
"00075a3110"	"00075b5bfa"	0.1489
"00075a3110"	"00075b6339"	0.0979
"00075a3110"	"00075b6658"	-0.1691
"00075a3110"	"00075b679a"	-0.0237
"00075a3110"	"00075b6cb7"	-0.1052
"00075a3110"	"00075b6df8"	0.2225
"00075a3110"	"00075b6ff6"	0.3834
"00075a3110"	"00075b70ee"	0.0264
"00075a3110"	"00075b7157"	0.1186
"00075a3110"	"00075b7225"	0.1337
"00075a3110"	"00075b7c89"	0.3009
"00075a3110"	"00075b9048"	0.4681
"00075a3110"	"00075d0801"	0.0097
"00075a3110"	"00075d1820"	-0.2522
"00075a3110"	"00075d1f3d"	0.202
"00075a3110"	"00075d2329"	-0.0872
"00075a3110"	"00075d2b9b"	0.1243
"00075a3110"	"00075d3941"	-0.1758
"00075a3110"	"00075d3e96"	-0.1142
"00075a3110"	"00075d4864"	-0.1742
"00075a3110"	"00075d5961"	-0.0201
"00075a3110"	"00075d5a63"	0.2596
"00075a3110"	"00075d6150"	-0.1759
"00075a3110"	"00075d67d0"	-0.1154
"00075a3110"	"00075d67e2"	-0.0427
"00075a3110"	"00075d73fc"	-0.087
"00075a3110"	"00075d7729"	0.1296
"00075a3110"	"00075d778c"	-0.158
"00075a3110"	"00075d7b9e"	0.0942
"00075a3110"	"00075d7c8f"	0.3245
"00075a3110"	"00075d804d"	0.114
"00075a3110"	"00075d819f"	0.1571
"00075a3110"	"00075d8601"	-0.0754
"00075a3110"	"00075d8c6a"	0.1616
"00075a3110"	"00075dfedc"	0.0851

"00075a3110"	"00075e05f2"	-0.077
"00075a3110"	"00075e0837"	0.0613
"00075a3110"	"00075e092e"	0.077
"00075a3110"	"00075e0965"	-0.1228
"00075a3110"	"00075e0bc8"	0.3072
"00075a3110"	"00075e0fbb"	0.0383
"00075a341a"	"00075a3dcf"	7e-04
"00075a341a"	"00075a3e22"	0.3837
"00075a341a"	"00075a48d8"	0.3985
"00075a341a"	"00075a5cfb"	-0.1868
"00075a341a"	"00075a6151"	-0.338
"00075a341a"	"00075a6708"	-0.1843
"00075a341a"	"00075a7319"	-0.3893
"00075a341a"	"00075a7723"	0.1309
"00075a341a"	"00075a778b"	0.3459
"00075a341a"	"00075a7b8e"	-0.3634
"00075a341a"	"00075a7c79"	0.0385
"00075a341a"	"00075a81b6"	0.0865
"00075a341a"	"00075a82ac"	0.3893
"00075a341a"	"00075a98e5"	0.1499
"00075a341a"	"00075b0d29"	0.1935
"00075a341a"	"00075b102a"	-0.1931
"00075a341a"	"00075b1074"	0.0658
"00075a341a"	"00075b135d"	-0.1794
"00075a341a"	"00075b138b"	-0.0461
"00075a341a"	"00075b13a0"	0.0356
"00075a341a"	"00075b13bd"	0.1651
"00075a341a"	"00075b16a9"	0.1311
"00075a341a"	"00075b1a28"	0.1443
"00075a341a"	"00075b1a97"	0.2917
"00075a341a"	"00075b1c7b"	-0.0544
"00075a341a"	"00075b1d24"	0.0794
"00075a341a"	"00075b202b"	-0.2381
"00075a341a"	"00075b22cb"	0.2635
"00075a341a"	"00075b22da"	0.2619
"00075a341a"	"00075b2556"	0.3574
"00075a341a"	"00075b25de"	-0.1455
"00075a341a"	"00075b260c"	-0.0932
"00075a341a"	"00075b26f1"	0.0202
"00075a341a"	"00075b2920"	-0.2292
"00075a341a"	"00075b2a64"	-0.2455
"00075a341a"	"00075b2a9d"	-0.4929
"00075a341a"	"00075b2b37"	-0.0852
"00075a341a"	"00075b2cdd"	0.1381
"00075a341a"	"00075b3038"	-0.0347
"00075a341a"	"00075b30fe"	0.0296
"00075a341a"	"00075b3362"	0.1051
"00075a341a"	"00075b350a"	-0.0204
"00075a341a"	"00075b350e"	-0.252
"00075a341a"	"00075b3651"	0.0302

"00075a341a"	"00075b38ca"	−0.0899
"00075a341a"	"00075b39cc"	−0.2388
"00075a341a"	"00075b3e1e"	0.1324
"00075a341a"	"00075b3e57"	0.446
"00075a341a"	"00075b4079"	−0.2348
"00075a341a"	"00075b4150"	−0.0891
"00075a341a"	"00075b4194"	−0.3461
"00075a341a"	"00075b42d5"	0.0045
"00075a341a"	"00075b4424"	0.379
"00075a341a"	"00075b4470"	−0.2455
"00075a341a"	"00075b47ed"	−0.0448
"00075a341a"	"00075b4850"	−0.2418
"00075a341a"	"00075b4ca0"	−0.2425
"00075a341a"	"00075b4d7f"	0.3695
"00075a341a"	"00075b520f"	−0.1418
"00075a341a"	"00075b525f"	0.0889
"00075a341a"	"00075b58f8"	−0.406
"00075a341a"	"00075b5bcc"	−0.0704
"00075a341a"	"00075b5bfa"	−0.2698
"00075a341a"	"00075b6339"	0.0694
"00075a341a"	"00075b6658"	0.1609
"00075a341a"	"00075b679a"	0.0329
"00075a341a"	"00075b6cb7"	−0.307
"00075a341a"	"00075b6df8"	0.5689
"00075a341a"	"00075b6ff6"	0.1655
"00075a341a"	"00075b70ee"	−0.264
"00075a341a"	"00075b7157"	0.0688
"00075a341a"	"00075b7225"	−0.0351
"00075a341a"	"00075b7c89"	0.0931
"00075a341a"	"00075b9048"	0.1419
"00075a341a"	"00075d0801"	−0.121
"00075a341a"	"00075d1820"	−0.0935
"00075a341a"	"00075d1f3d"	−0.332
"00075a341a"	"00075d2329"	−0.0893
"00075a341a"	"00075d2b9b"	−0.248
"00075a341a"	"00075d3941"	−0.0476
"00075a341a"	"00075d3e96"	−0.0814
"00075a341a"	"00075d4864"	−0.1177
"00075a341a"	"00075d5961"	0.1024
"00075a341a"	"00075d5a63"	0.0353
"00075a341a"	"00075d6150"	−0.1977
"00075a341a"	"00075d67d0"	−0.1774
"00075a341a"	"00075d67e2"	0.1327
"00075a341a"	"00075d73fc"	0.2357
"00075a341a"	"00075d7729"	0.1165
"00075a341a"	"00075d778c"	0.1529
"00075a341a"	"00075d7b9e"	0.2419
"00075a341a"	"00075d7c8f"	0.1713
"00075a341a"	"00075d804d"	−0.2314
"00075a341a"	"00075d819f"	−0.3789

"00075a341a"	"00075d8601"	−0.0051
"00075a341a"	"00075d8c6a"	0.256
"00075a341a"	"00075dfedc"	0.0492
"00075a341a"	"00075e05f2"	−0.0508
"00075a341a"	"00075e0837"	−0.3877
"00075a341a"	"00075e092e"	0.1218
"00075a341a"	"00075e0965"	−0.2666
"00075a341a"	"00075e0bc8"	−0.1058
"00075a341a"	"00075e0fbb"	0.0292
"00075a3dcf"	"00075a3e22"	−0.1514
"00075a3dcf"	"00075a48d8"	0.1463
"00075a3dcf"	"00075a5cfb"	−0.3979
"00075a3dcf"	"00075a6151"	0.0757
"00075a3dcf"	"00075a6708"	−0.0616
"00075a3dcf"	"00075a7319"	−0.2353
"00075a3dcf"	"00075a7723"	−0.0108
"00075a3dcf"	"00075a778b"	−0.0256
"00075a3dcf"	"00075a7b8e"	0.0131
"00075a3dcf"	"00075a7c79"	0.0276
"00075a3dcf"	"00075a81b6"	−0.0062
"00075a3dcf"	"00075a82ac"	0.2135
"00075a3dcf"	"00075a98e5"	−0.0533
"00075a3dcf"	"00075b0d29"	−0.1824
"00075a3dcf"	"00075b102a"	−0.1808
"00075a3dcf"	"00075b1074"	0.0815
"00075a3dcf"	"00075b135d"	−0.1668
"00075a3dcf"	"00075b138b"	0.0774
"00075a3dcf"	"00075b13a0"	0.1391
"00075a3dcf"	"00075b13bd"	0.0399
"00075a3dcf"	"00075b16a9"	0.131
"00075a3dcf"	"00075b1a28"	−0.0798
"00075a3dcf"	"00075b1a97"	0.0538
"00075a3dcf"	"00075b1c7b"	−0.1538
"00075a3dcf"	"00075b1d24"	0.0813
"00075a3dcf"	"00075b202b"	−0.1246
"00075a3dcf"	"00075b22cb"	0.1237
"00075a3dcf"	"00075b22da"	0.1155
"00075a3dcf"	"00075b2556"	−0.0195
"00075a3dcf"	"00075b25de"	0.2357
"00075a3dcf"	"00075b260c"	−0.1075
"00075a3dcf"	"00075b26f1"	−0.031
"00075a3dcf"	"00075b2920"	−0.3653
"00075a3dcf"	"00075b2a64"	−0.4197
"00075a3dcf"	"00075b2a9d"	−0.1598
"00075a3dcf"	"00075b2b37"	−0.2873
"00075a3dcf"	"00075b2cdd"	0.1033
"00075a3dcf"	"00075b3038"	0.2368
"00075a3dcf"	"00075b30fe"	0.1814
"00075a3dcf"	"00075b3362"	−0.0764
"00075a3dcf"	"00075b350a"	−0.1989

"00075a3dcf"	"00075b350e"	0.1392
"00075a3dcf"	"00075b3651"	-0.0222
"00075a3dcf"	"00075b38ca"	0.3113
"00075a3dcf"	"00075b39cc"	0.3907
"00075a3dcf"	"00075b3e1e"	0.0486
"00075a3dcf"	"00075b3e57"	0.1008
"00075a3dcf"	"00075b4079"	-0.2468
"00075a3dcf"	"00075b4150"	-0.2607
"00075a3dcf"	"00075b4194"	0.1641
"00075a3dcf"	"00075b42d5"	0.3569
"00075a3dcf"	"00075b4424"	-0.1389
"00075a3dcf"	"00075b4470"	0.0833
"00075a3dcf"	"00075b47ed"	0.5002
"00075a3dcf"	"00075b4850"	-0.0533
"00075a3dcf"	"00075b4ca0"	0.0356
"00075a3dcf"	"00075b4d7f"	0.1557
"00075a3dcf"	"00075b520f"	0.1277
"00075a3dcf"	"00075b525f"	-0.073
"00075a3dcf"	"00075b58f8"	-0.1334
"00075a3dcf"	"00075b5bcc"	-0.0458
"00075a3dcf"	"00075b5bfa"	0.028
"00075a3dcf"	"00075b6339"	0.1634
"00075a3dcf"	"00075b6658"	0.0999
"00075a3dcf"	"00075b679a"	0.0651
"00075a3dcf"	"00075b6cb7"	-0.2051
"00075a3dcf"	"00075b6df8"	0.2893
"00075a3dcf"	"00075b6ff6"	0.0256
"00075a3dcf"	"00075b70ee"	-0.2422
"00075a3dcf"	"00075b7157"	0.2165
"00075a3dcf"	"00075b7225"	0.0175
"00075a3dcf"	"00075b7c89"	0.1212
"00075a3dcf"	"00075b9048"	-8e-04
"00075a3dcf"	"00075d0801"	0.1355
"00075a3dcf"	"00075d1820"	0.0356
"00075a3dcf"	"00075d1f3d"	-0.1517
"00075a3dcf"	"00075d2329"	0.7233
"00075a3dcf"	"00075d2b9b"	-0.1521
"00075a3dcf"	"00075d3941"	-0.2053
"00075a3dcf"	"00075d3e96"	-0.0939
"00075a3dcf"	"00075d4864"	0.453
"00075a3dcf"	"00075d5961"	0.1222
"00075a3dcf"	"00075d5a63"	0.2063
"00075a3dcf"	"00075d6150"	-0.0838
"00075a3dcf"	"00075d67d0"	-0.0565
"00075a3dcf"	"00075d67e2"	-0.0415
"00075a3dcf"	"00075d73fc"	0.2417
"00075a3dcf"	"00075d7729"	-0.2181
"00075a3dcf"	"00075d778c"	0.0436
"00075a3dcf"	"00075d7b9e"	-0.106
"00075a3dcf"	"00075d7c8f"	0.1815

"00075a3dcf"	"00075d804d"	0.0228
"00075a3dcf"	"00075d819f"	-0.0891
"00075a3dcf"	"00075d8601"	0.0381
"00075a3dcf"	"00075d8c6a"	0.0242
"00075a3dcf"	"00075dfedc"	0.0402
"00075a3dcf"	"00075e05f2"	0.0993
"00075a3dcf"	"00075e0837"	0.1116
"00075a3dcf"	"00075e092e"	0.1821
"00075a3dcf"	"00075e0965"	-0.3923
"00075a3dcf"	"00075e0bc8"	0.1804
"00075a3dcf"	"00075e0fbb"	0.1958
"00075a3e22"	"00075a48d8"	0.4148
"00075a3e22"	"00075a5cfb"	0.0245
"00075a3e22"	"00075a6151"	-0.0815
"00075a3e22"	"00075a6708"	-0.0982
"00075a3e22"	"00075a7319"	-0.1355
"00075a3e22"	"00075a7723"	0.1828
"00075a3e22"	"00075a778b"	0.1562
"00075a3e22"	"00075a7b8e"	0.0527
"00075a3e22"	"00075a7c79"	0.3568
"00075a3e22"	"00075a81b6"	0.2492
"00075a3e22"	"00075a82ac"	0.3997
"00075a3e22"	"00075a98e5"	0.3637
"00075a3e22"	"00075b0d29"	0.2237
"00075a3e22"	"00075b102a"	0.2032
"00075a3e22"	"00075b1074"	0.1116
"00075a3e22"	"00075b135d"	0.2993
"00075a3e22"	"00075b138b"	0.3747
"00075a3e22"	"00075b13a0"	-0.0047
"00075a3e22"	"00075b13bd"	0.2937
"00075a3e22"	"00075b16a9"	0.007
"00075a3e22"	"00075b1a28"	-0.1293
"00075a3e22"	"00075b1a97"	0.1724
"00075a3e22"	"00075b1c7b"	-0.434
"00075a3e22"	"00075b1d24"	-0.0401
"00075a3e22"	"00075b202b"	0.1595
"00075a3e22"	"00075b22cb"	-0.0746
"00075a3e22"	"00075b22da"	0.1227
"00075a3e22"	"00075b2556"	0.1052
"00075a3e22"	"00075b25de"	-0.0811
"00075a3e22"	"00075b260c"	0.1613
"00075a3e22"	"00075b26f1"	0.0185
"00075a3e22"	"00075b2920"	-0.008
"00075a3e22"	"00075b2a64"	0.189
"00075a3e22"	"00075b2a9d"	-0.0235
"00075a3e22"	"00075b2b37"	-0.3535
"00075a3e22"	"00075b2cdd"	0.0976
"00075a3e22"	"00075b3038"	0.1261
"00075a3e22"	"00075b30fe"	-0.0696
"00075a3e22"	"00075b3362"	-0.1856

"00075a3e22"	"00075b350a"	0.1715
"00075a3e22"	"00075b350e"	0.0299
"00075a3e22"	"00075b3651"	−0.221
"00075a3e22"	"00075b38ca"	−0.1262
"00075a3e22"	"00075b39cc"	0.023
"00075a3e22"	"00075b3e1e"	0.1773
"00075a3e22"	"00075b3e57"	0.1581
"00075a3e22"	"00075b4079"	−0.055
"00075a3e22"	"00075b4150"	−0.0836
"00075a3e22"	"00075b4194"	0.3215
"00075a3e22"	"00075b42d5"	0.0025
"00075a3e22"	"00075b4424"	−0.1942
"00075a3e22"	"00075b4470"	0.3026
"00075a3e22"	"00075b47ed"	0.0732
"00075a3e22"	"00075b4850"	0.2308
"00075a3e22"	"00075b4ca0"	0.1759
"00075a3e22"	"00075b4d7f"	0.4205
"00075a3e22"	"00075b520f"	−0.1432
"00075a3e22"	"00075b525f"	−0.0945
"00075a3e22"	"00075b58f8"	−0.3073
"00075a3e22"	"00075b5bcc"	0.0693
"00075a3e22"	"00075b5bfa"	−0.3174
"00075a3e22"	"00075b6339"	0.2587
"00075a3e22"	"00075b6658"	−0.1899
"00075a3e22"	"00075b679a"	−0.0987
"00075a3e22"	"00075b6cb7"	−0.031
"00075a3e22"	"00075b6df8"	0.259
"00075a3e22"	"00075b6ff6"	0.1308
"00075a3e22"	"00075b70ee"	−0.2036
"00075a3e22"	"00075b7157"	0.1579
"00075a3e22"	"00075b7225"	0.2004
"00075a3e22"	"00075b7c89"	0.3382
"00075a3e22"	"00075b9048"	0.3094
"00075a3e22"	"00075d0801"	−0.0108
"00075a3e22"	"00075d1820"	−0.2503
"00075a3e22"	"00075d1f3d"	0.1368
"00075a3e22"	"00075d2329"	−0.0579
"00075a3e22"	"00075d2b9b"	0.0703
"00075a3e22"	"00075d3941"	0.0898
"00075a3e22"	"00075d3e96"	−0.2039
"00075a3e22"	"00075d4864"	−0.3142
"00075a3e22"	"00075d5961"	0.1451
"00075a3e22"	"00075d5a63"	0.0614
"00075a3e22"	"00075d6150"	−0.2445
"00075a3e22"	"00075d67d0"	−0.1193
"00075a3e22"	"00075d67e2"	0.3104
"00075a3e22"	"00075d73fc"	0.0302
"00075a3e22"	"00075d7729"	0.0174
"00075a3e22"	"00075d778c"	−0.2942
"00075a3e22"	"00075d7b9e"	0.0625

"00075a3e22"	"00075d7c8f"	0.4855
"00075a3e22"	"00075d804d"	0.2987
"00075a3e22"	"00075d819f"	0.1381
"00075a3e22"	"00075d8601"	0.0752
"00075a3e22"	"00075d8c6a"	0.3609
"00075a3e22"	"00075dfedc"	0.1282
"00075a3e22"	"00075e05f2"	0.0082
"00075a3e22"	"00075e0837"	-0.1432
"00075a3e22"	"00075e092e"	0.078
"00075a3e22"	"00075e0965"	-0.3985
"00075a3e22"	"00075e0bc8"	0.1778
"00075a3e22"	"00075e0fbb"	-0.0168
"00075a48d8"	"00075a5cfb"	-0.103
"00075a48d8"	"00075a6151"	-0.0226
"00075a48d8"	"00075a6708"	-0.1854
"00075a48d8"	"00075a7319"	-0.4248
"00075a48d8"	"00075a7723"	0.1712
"00075a48d8"	"00075a778b"	0.0888
"00075a48d8"	"00075a7b8e"	-0.0298
"00075a48d8"	"00075a7c79"	0.0837
"00075a48d8"	"00075a81b6"	0.3712
"00075a48d8"	"00075a82ac"	0.1014
"00075a48d8"	"00075a98e5"	0.1281
"00075a48d8"	"00075b0d29"	-0.0129
"00075a48d8"	"00075b102a"	-0.0222
"00075a48d8"	"00075b1074"	0.1544
"00075a48d8"	"00075b135d"	0.0844
"00075a48d8"	"00075b138b"	0.1421
"00075a48d8"	"00075b13a0"	0.1005
"00075a48d8"	"00075b13bd"	0.03
"00075a48d8"	"00075b16a9"	0.1799
"00075a48d8"	"00075b1a28"	-0.074
"00075a48d8"	"00075b1a97"	0.0337
"00075a48d8"	"00075b1c7b"	-0.0253
"00075a48d8"	"00075b1d24"	0.1142
"00075a48d8"	"00075b202b"	-0.1121
"00075a48d8"	"00075b22cb"	0.1006
"00075a48d8"	"00075b22da"	0.0921
"00075a48d8"	"00075b2556"	0.0332
"00075a48d8"	"00075b25de"	-0.1625
"00075a48d8"	"00075b260c"	-0.0823
"00075a48d8"	"00075b26f1"	0.1462
"00075a48d8"	"00075b2920"	-0.2414
"00075a48d8"	"00075b2a64"	-0.1901
"00075a48d8"	"00075b2a9d"	-0.2698
"00075a48d8"	"00075b2b37"	-0.3767
"00075a48d8"	"00075b2cdd"	0.1134
"00075a48d8"	"00075b3038"	0.046
"00075a48d8"	"00075b30fe"	-0.1217
"00075a48d8"	"00075b3362"	0.025

"00075a48d8"	"00075b350a"	0.0078
"00075a48d8"	"00075b350e"	−0.2787
"00075a48d8"	"00075b3651"	−0.0254
"00075a48d8"	"00075b38ca"	−0.2226
"00075a48d8"	"00075b39cc"	0.0572
"00075a48d8"	"00075b3e1e"	0.167
"00075a48d8"	"00075b3e57"	0.286
"00075a48d8"	"00075b4079"	−0.0377
"00075a48d8"	"00075b4150"	−0.2183
"00075a48d8"	"00075b4194"	0.0877
"00075a48d8"	"00075b42d5"	−0.1357
"00075a48d8"	"00075b4424"	−0.1347
"00075a48d8"	"00075b4470"	−0.0671
"00075a48d8"	"00075b47ed"	0.165
"00075a48d8"	"00075b4850"	−0.1392
"00075a48d8"	"00075b4ca0"	−0.1437
"00075a48d8"	"00075b4d7f"	0.6095
"00075a48d8"	"00075b520f"	−0.2023
"00075a48d8"	"00075b525f"	0.0051
"00075a48d8"	"00075b58f8"	−0.4023
"00075a48d8"	"00075b5bcc"	0.0247
"00075a48d8"	"00075b5bfa"	−0.1591
"00075a48d8"	"00075b6339"	0.2581
"00075a48d8"	"00075b6658"	−0.0984
"00075a48d8"	"00075b679a"	0.061
"00075a48d8"	"00075b6cb7"	−0.1205
"00075a48d8"	"00075b6df8"	0.6046
"00075a48d8"	"00075b6ff6"	0.0815
"00075a48d8"	"00075b70ee"	−0.226
"00075a48d8"	"00075b7157"	0.2492
"00075a48d8"	"00075b7225"	−0.0392
"00075a48d8"	"00075b7c89"	0.0979
"00075a48d8"	"00075b9048"	0.1153
"00075a48d8"	"00075d0801"	−0.057
"00075a48d8"	"00075d1820"	−0.1621
"00075a48d8"	"00075d1f3d"	−0.135
"00075a48d8"	"00075d2329"	0.01
"00075a48d8"	"00075d2b9b"	−0.169
"00075a48d8"	"00075d3941"	−0.0954
"00075a48d8"	"00075d3e96"	−0.1591
"00075a48d8"	"00075d4864"	−0.2136
"00075a48d8"	"00075d5961"	0.0815
"00075a48d8"	"00075d5a63"	0.2201
"00075a48d8"	"00075d6150"	−0.2672
"00075a48d8"	"00075d67d0"	0.0841
"00075a48d8"	"00075d67e2"	0.2918
"00075a48d8"	"00075d73fc"	0.168
"00075a48d8"	"00075d7729"	−0.0522
"00075a48d8"	"00075d778c"	−0.0077
"00075a48d8"	"00075d7b9e"	0.0721

"00075a48d8"	"00075d7c8f"	0.3304
"00075a48d8"	"00075d804d"	−0.0153
"00075a48d8"	"00075d819f"	−0.1114
"00075a48d8"	"00075d8601"	0.0185
"00075a48d8"	"00075d8c6a"	0.3245
"00075a48d8"	"00075dfedc"	0.143
"00075a48d8"	"00075e05f2"	0.1389
"00075a48d8"	"00075e0837"	−0.1617
"00075a48d8"	"00075e092e"	0.2081
"00075a48d8"	"00075e0965"	−0.3087
"00075a48d8"	"00075e0bc8"	−0.0292
"00075a48d8"	"00075e0fbb"	0.097
"00075a5cfb"	"00075a6151"	0.0938
"00075a5cfb"	"00075a6708"	−0.0824
"00075a5cfb"	"00075a7319"	−0.1752
"00075a5cfb"	"00075a7723"	−0.0423
"00075a5cfb"	"00075a778b"	0.0994
"00075a5cfb"	"00075a7b8e"	−0.0637
"00075a5cfb"	"00075a7c79"	0.1008
"00075a5cfb"	"00075a81b6"	−0.2375
"00075a5cfb"	"00075a82ac"	−0.1041
"00075a5cfb"	"00075a98e5"	−0.2478
"00075a5cfb"	"00075b0d29"	−0.0204
"00075a5cfb"	"00075b102a"	0.0592
"00075a5cfb"	"00075b1074"	−0.2556
"00075a5cfb"	"00075b135d"	−0.0179
"00075a5cfb"	"00075b138b"	0.1451
"00075a5cfb"	"00075b13a0"	−0.128
"00075a5cfb"	"00075b13bd"	−0.3073
"00075a5cfb"	"00075b16a9"	−0.0151
"00075a5cfb"	"00075b1a28"	−0.056
"00075a5cfb"	"00075b1a97"	−0.1162
"00075a5cfb"	"00075b1c7b"	−0.1473
"00075a5cfb"	"00075b1d24"	−0.0905
"00075a5cfb"	"00075b202b"	0.173
"00075a5cfb"	"00075b22cb"	−0.1589
"00075a5cfb"	"00075b22da"	0.0257
"00075a5cfb"	"00075b2556"	−0.0826
"00075a5cfb"	"00075b25de"	−0.181
"00075a5cfb"	"00075b260c"	0.2623
"00075a5cfb"	"00075b26f1"	−0.2161
"00075a5cfb"	"00075b2920"	0.4976
"00075a5cfb"	"00075b2a64"	0.4505
"00075a5cfb"	"00075b2a9d"	0.0354
"00075a5cfb"	"00075b2b37"	0.0055
"00075a5cfb"	"00075b2cdd"	−0.1359
"00075a5cfb"	"00075b3038"	−0.1249
"00075a5cfb"	"00075b30fe"	−0.2814
"00075a5cfb"	"00075b3362"	−0.278
"00075a5cfb"	"00075b350a"	0.1168

"00075a5cfb"	"00075b350e"	0.0534
"00075a5cfb"	"00075b3651"	-0.4279
"00075a5cfb"	"00075b38ca"	-0.1953
"00075a5cfb"	"00075b39cc"	-0.4688
"00075a5cfb"	"00075b3e1e"	-0.0271
"00075a5cfb"	"00075b3e57"	0.0283
"00075a5cfb"	"00075b4079"	0.0197
"00075a5cfb"	"00075b4150"	0.542
"00075a5cfb"	"00075b4194"	0.1055
"00075a5cfb"	"00075b42d5"	-0.0885
"00075a5cfb"	"00075b4424"	0.0572
"00075a5cfb"	"00075b4470"	0.1883
"00075a5cfb"	"00075b47ed"	-0.4451
"00075a5cfb"	"00075b4850"	0.2223
"00075a5cfb"	"00075b4ca0"	-0.1173
"00075a5cfb"	"00075b4d7f"	0.1233
"00075a5cfb"	"00075b520f"	-0.2328
"00075a5cfb"	"00075b525f"	-0.1862
"00075a5cfb"	"00075b58f8"	0.3972
"00075a5cfb"	"00075b5bcc"	0.1235
"00075a5cfb"	"00075b5bfa"	-0.3089
"00075a5cfb"	"00075b6339"	0.1583
"00075a5cfb"	"00075b6658"	-0.6058
"00075a5cfb"	"00075b679a"	0.0354
"00075a5cfb"	"00075b6cb7"	0.0501
"00075a5cfb"	"00075b6df8"	-0.2094
"00075a5cfb"	"00075b6ff6"	0.3256
"00075a5cfb"	"00075b70ee"	0.0469
"00075a5cfb"	"00075b7157"	-0.0658
"00075a5cfb"	"00075b7225"	-0.0423
"00075a5cfb"	"00075b7c89"	-0.1904
"00075a5cfb"	"00075b9048"	0.3629
"00075a5cfb"	"00075d0801"	-0.0463
"00075a5cfb"	"00075d1820"	-0.2074
"00075a5cfb"	"00075d1f3d"	0.298
"00075a5cfb"	"00075d2329"	-0.2734
"00075a5cfb"	"00075d2b9b"	0.262
"00075a5cfb"	"00075d3941"	-0.3142
"00075a5cfb"	"00075d3e96"	0.0686
"00075a5cfb"	"00075d4864"	-0.1967
"00075a5cfb"	"00075d5961"	-0.1931
"00075a5cfb"	"00075d5a63"	0.2089
"00075a5cfb"	"00075d6150"	-0.2366
"00075a5cfb"	"00075d67d0"	-0.4763
"00075a5cfb"	"00075d67e2"	-0.0131
"00075a5cfb"	"00075d73fc"	-0.2262
"00075a5cfb"	"00075d7729"	-0.1568
"00075a5cfb"	"00075d778c"	-0.4847
"00075a5cfb"	"00075d7b9e"	-0.3203
"00075a5cfb"	"00075d7c8f"	-0.0748

"00075a5cfb"	"00075d804d"	0.2341
"00075a5cfb"	"00075d819f"	−0.1923
"00075a5cfb"	"00075d8601"	−0.253
"00075a5cfb"	"00075d8c6a"	−0.091
"00075a5cfb"	"00075dfedc"	−0.0245
"00075a5cfb"	"00075e05f2"	0.1432
"00075a5cfb"	"00075e0837"	−0.025
"00075a5cfb"	"00075e092e"	−0.1805
"00075a5cfb"	"00075e0965"	0.5977
"00075a5cfb"	"00075e0bc8"	0.1796
"00075a5cfb"	"00075e0fbb"	−0.1975
"00075a6151"	"00075a6708"	0.2152
"00075a6151"	"00075a7319"	−0.1247
"00075a6151"	"00075a7723"	0.093
"00075a6151"	"00075a778b"	−0.2586
"00075a6151"	"00075a7b8e"	0.0795
"00075a6151"	"00075a7c79"	0.1888
"00075a6151"	"00075a81b6"	0.0757
"00075a6151"	"00075a82ac"	−0.0677
"00075a6151"	"00075a98e5"	−0.1121
"00075a6151"	"00075b0d29"	−0.1161
"00075a6151"	"00075b102a"	0.1353
"00075a6151"	"00075b1074"	−0.3988
"00075a6151"	"00075b135d"	−0.0492
"00075a6151"	"00075b138b"	−0.0382
"00075a6151"	"00075b13a0"	−0.0122
"00075a6151"	"00075b13bd"	−0.3533
"00075a6151"	"00075b16a9"	0.0178
"00075a6151"	"00075b1a28"	−0.2626
"00075a6151"	"00075b1a97"	−0.3694
"00075a6151"	"00075b1c7b"	−0.1887
"00075a6151"	"00075b1d24"	−0.1918
"00075a6151"	"00075b202b"	−0.0385
"00075a6151"	"00075b22cb"	−0.1932
"00075a6151"	"00075b22da"	−0.2167
"00075a6151"	"00075b2556"	−0.0976
"00075a6151"	"00075b25de"	0.1138
"00075a6151"	"00075b260c"	0.2343
"00075a6151"	"00075b26f1"	−0.0066
"00075a6151"	"00075b2920"	0.1785
"00075a6151"	"00075b2a64"	−0.0759
"00075a6151"	"00075b2a9d"	0.1877
"00075a6151"	"00075b2b37"	−0.3635
"00075a6151"	"00075b2cdd"	−0.1792
"00075a6151"	"00075b3038"	0.1209
"00075a6151"	"00075b30fe"	−0.2473
"00075a6151"	"00075b3362"	−0.3565
"00075a6151"	"00075b350a"	−0.2912
"00075a6151"	"00075b350e"	−0.1284
"00075a6151"	"00075b3651"	−0.2609

"00075a6151"	"00075b38ca"	−0.4146
"00075a6151"	"00075b39cc"	0.2026
"00075a6151"	"00075b3e1e"	−0.1142
"00075a6151"	"00075b3e57"	−0.387
"00075a6151"	"00075b4079"	−0.1485
"00075a6151"	"00075b4150"	0.1656
"00075a6151"	"00075b4194"	0.0596
"00075a6151"	"00075b42d5"	−0.2608
"00075a6151"	"00075b4424"	−0.3964
"00075a6151"	"00075b4470"	−0.0773
"00075a6151"	"00075b47ed"	−0.012
"00075a6151"	"00075b4850"	−0.0202
"00075a6151"	"00075b4ca0"	0.1568
"00075a6151"	"00075b4d7f"	−0.0616
"00075a6151"	"00075b520f"	−0.0118
"00075a6151"	"00075b525f"	−0.219
"00075a6151"	"00075b58f8"	0.0138
"00075a6151"	"00075b5bcc"	−0.1314
"00075a6151"	"00075b5bfa"	0.0942
"00075a6151"	"00075b6339"	0.3619
"00075a6151"	"00075b6658"	−0.4055
"00075a6151"	"00075b679a"	−0.0531
"00075a6151"	"00075b6cb7"	0.064
"00075a6151"	"00075b6df8"	−0.1995
"00075a6151"	"00075b6ff6"	−0.0988
"00075a6151"	"00075b70ee"	6e−04
"00075a6151"	"00075b7157"	−0.0654
"00075a6151"	"00075b7225"	−0.2458
"00075a6151"	"00075b7c89"	0.1119
"00075a6151"	"00075b9048"	0.2678
"00075a6151"	"00075d0801"	0.1442
"00075a6151"	"00075d1820"	−0.2188
"00075a6151"	"00075d1f3d"	−0.1131
"00075a6151"	"00075d2329"	0.06
"00075a6151"	"00075d2b9b"	0.0055
"00075a6151"	"00075d3941"	−0.3026
"00075a6151"	"00075d3e96"	−0.1035
"00075a6151"	"00075d4864"	−0.1593
"00075a6151"	"00075d5961"	−0.1746
"00075a6151"	"00075d5a63"	−0.1351
"00075a6151"	"00075d6150"	−0.2114
"00075a6151"	"00075d67d0"	−0.0199
"00075a6151"	"00075d67e2"	0.0425
"00075a6151"	"00075d73fc"	−0.2824
"00075a6151"	"00075d7729"	−0.3478
"00075a6151"	"00075d778c"	−0.2467
"00075a6151"	"00075d7b9e"	−0.1189
"00075a6151"	"00075d7c8f"	−0.0348
"00075a6151"	"00075d804d"	0.1183
"00075a6151"	"00075d819f"	−0.0229

"00075a6151"	"00075d8601"	-0.106
"00075a6151"	"00075d8c6a"	0.0358
"00075a6151"	"00075dfedc"	0.1237
"00075a6151"	"00075e05f2"	0.3888
"00075a6151"	"00075e0837"	0.1584
"00075a6151"	"00075e092e"	-0.2675
"00075a6151"	"00075e0965"	-0.0851
"00075a6151"	"00075e0bc8"	1e-04
"00075a6151"	"00075e0fbb"	-0.3016
"00075a6708"	"00075a7319"	-0.1212
"00075a6708"	"00075a7723"	0.0172
"00075a6708"	"00075a778b"	-0.0986
"00075a6708"	"00075a7b8e"	0.2205
"00075a6708"	"00075a7c79"	0.221
"00075a6708"	"00075a81b6"	-0.2505
"00075a6708"	"00075a82ac"	0.0452
"00075a6708"	"00075a98e5"	-0.1725
"00075a6708"	"00075b0d29"	-0.0704
"00075a6708"	"00075b102a"	-0.0112
"00075a6708"	"00075b1074"	-0.0986
"00075a6708"	"00075b135d"	-0.164
"00075a6708"	"00075b138b"	0.0365
"00075a6708"	"00075b13a0"	-0.1858
"00075a6708"	"00075b13bd"	-0.1294
"00075a6708"	"00075b16a9"	-0.1855
"00075a6708"	"00075b1a28"	-0.0735
"00075a6708"	"00075b1a97"	-0.1506
"00075a6708"	"00075b1c7b"	-0.3427
"00075a6708"	"00075b1d24"	0.1001
"00075a6708"	"00075b202b"	0.1152
"00075a6708"	"00075b22cb"	-0.1786
"00075a6708"	"00075b22da"	-0.0295
"00075a6708"	"00075b2556"	-0.0458
"00075a6708"	"00075b25de"	0.1341
"00075a6708"	"00075b260c"	-0.0082
"00075a6708"	"00075b26f1"	-0.1846
"00075a6708"	"00075b2920"	0.1369
"00075a6708"	"00075b2a64"	-0.0687
"00075a6708"	"00075b2a9d"	0.0223
"00075a6708"	"00075b2b37"	-0.3336
"00075a6708"	"00075b2cdd"	-0.2325
"00075a6708"	"00075b3038"	0.0749
"00075a6708"	"00075b30fe"	-0.0475
"00075a6708"	"00075b3362"	-0.309
"00075a6708"	"00075b350a"	0.0553
"00075a6708"	"00075b350e"	-0.2494
"00075a6708"	"00075b3651"	-0.0682
"00075a6708"	"00075b38ca"	-0.314
"00075a6708"	"00075b39cc"	-0.1425
"00075a6708"	"00075b3e1e"	-0.2122

"00075a6708"	"00075b3e57"	-0.0871
"00075a6708"	"00075b4079"	-0.1729
"00075a6708"	"00075b4150"	0.099
"00075a6708"	"00075b4194"	-0.0304
"00075a6708"	"00075b42d5"	-0.0629
"00075a6708"	"00075b4424"	-0.1428
"00075a6708"	"00075b4470"	0.123
"00075a6708"	"00075b47ed"	-0.2441
"00075a6708"	"00075b4850"	0.2427
"00075a6708"	"00075b4ca0"	0.0364
"00075a6708"	"00075b4d7f"	-0.0439
"00075a6708"	"00075b520f"	0.0295
"00075a6708"	"00075b525f"	-0.2535
"00075a6708"	"00075b58f8"	7e-04
"00075a6708"	"00075b5bcc"	-0.0412
"00075a6708"	"00075b5bfa"	-0.1674
"00075a6708"	"00075b6339"	0.0461
"00075a6708"	"00075b6658"	-0.1495
"00075a6708"	"00075b679a"	0.1242
"00075a6708"	"00075b6cb7"	0.0717
"00075a6708"	"00075b6df8"	0.0327
"00075a6708"	"00075b6ff6"	0.0686
"00075a6708"	"00075b70ee"	0.1599
"00075a6708"	"00075b7157"	-0.162
"00075a6708"	"00075b7225"	-0.1633
"00075a6708"	"00075b7c89"	0.3587
"00075a6708"	"00075b9048"	0.3693
"00075a6708"	"00075d0801"	-0.0206
"00075a6708"	"00075d1820"	-0.1841
"00075a6708"	"00075d1f3d"	0.0961
"00075a6708"	"00075d2329"	-0.0868
"00075a6708"	"00075d2b9b"	-0.2722
"00075a6708"	"00075d3941"	-0.3806
"00075a6708"	"00075d3e96"	-0.0696
"00075a6708"	"00075d4864"	-0.2743
"00075a6708"	"00075d5961"	-0.0895
"00075a6708"	"00075d5a63"	-0.083
"00075a6708"	"00075d6150"	-0.2046
"00075a6708"	"00075d67d0"	-0.1298
"00075a6708"	"00075d67e2"	-0.1737
"00075a6708"	"00075d73fc"	-0.0225
"00075a6708"	"00075d7729"	-0.1178
"00075a6708"	"00075d778c"	-0.3622
"00075a6708"	"00075d7b9e"	-0.1426
"00075a6708"	"00075d7c8f"	-0.0743
"00075a6708"	"00075d804d"	0.0339
"00075a6708"	"00075d819f"	0.0192
"00075a6708"	"00075d8601"	-0.2971
"00075a6708"	"00075d8c6a"	-0.0877
"00075a6708"	"00075dfedc"	-0.1064

"00075a6708"	"00075e05f2"	−0.176
"00075a6708"	"00075e0837"	0.0692
"00075a6708"	"00075e092e"	0.1729
"00075a6708"	"00075e0965"	−0.1234
"00075a6708"	"00075e0bc8"	0.206
"00075a6708"	"00075e0fbb"	−0.2123
"00075a7319"	"00075a7723"	−0.2447
"00075a7319"	"00075a778b"	−0.3442
"00075a7319"	"00075a7b8e"	0.3101
"00075a7319"	"00075a7c79"	−0.0497
"00075a7319"	"00075a81b6"	−0.3438
"00075a7319"	"00075a82ac"	−0.1945
"00075a7319"	"00075a98e5"	−0.3456
"00075a7319"	"00075b0d29"	−0.3525
"00075a7319"	"00075b102a"	−0.2883
"00075a7319"	"00075b1074"	−0.3869
"00075a7319"	"00075b135d"	−0.1488
"00075a7319"	"00075b138b"	−0.1652
"00075a7319"	"00075b13a0"	−0.4384
"00075a7319"	"00075b13bd"	−0.1036
"00075a7319"	"00075b16a9"	−0.055
"00075a7319"	"00075b1a28"	−0.2837
"00075a7319"	"00075b1a97"	−0.0333
"00075a7319"	"00075b1c7b"	−0.221
"00075a7319"	"00075b1d24"	−0.0626
"00075a7319"	"00075b202b"	−0.1081
"00075a7319"	"00075b22cb"	−0.3606
"00075a7319"	"00075b22da"	−0.3431
"00075a7319"	"00075b2556"	−0.2542
"00075a7319"	"00075b25de"	0.0501
"00075a7319"	"00075b260c"	−0.1174
"00075a7319"	"00075b26f1"	−0.4718
"00075a7319"	"00075b2920"	0.1635
"00075a7319"	"00075b2a64"	−0.1212
"00075a7319"	"00075b2a9d"	−0.0745
"00075a7319"	"00075b2b37"	−0.1547
"00075a7319"	"00075b2cdd"	−0.1164
"00075a7319"	"00075b3038"	−0.0773
"00075a7319"	"00075b30fe"	−0.3418
"00075a7319"	"00075b3362"	−0.2307
"00075a7319"	"00075b350a"	−0.1815
"00075a7319"	"00075b350e"	−0.2218
"00075a7319"	"00075b3651"	−0.0859
"00075a7319"	"00075b38ca"	−0.2406
"00075a7319"	"00075b39cc"	0.1145
"00075a7319"	"00075b3e1e"	−0.1755
"00075a7319"	"00075b3e57"	−0.2288
"00075a7319"	"00075b4079"	−0.1147
"00075a7319"	"00075b4150"	−0.189
"00075a7319"	"00075b4194"	0.0554

"00075a7319"	"00075b42d5"	-0.1657
"00075a7319"	"00075b4424"	-0.3678
"00075a7319"	"00075b4470"	-0.0489
"00075a7319"	"00075b47ed"	0.1054
"00075a7319"	"00075b4850"	0.0564
"00075a7319"	"00075b4ca0"	-0.24
"00075a7319"	"00075b4d7f"	-0.236
"00075a7319"	"00075b520f"	-0.1092
"00075a7319"	"00075b525f"	-0.0984
"00075a7319"	"00075b58f8"	-0.1147
"00075a7319"	"00075b5bcc"	-0.1004
"00075a7319"	"00075b5bfa"	-0.2835
"00075a7319"	"00075b6339"	-0.1979
"00075a7319"	"00075b6658"	-0.4563
"00075a7319"	"00075b679a"	-0.0062
"00075a7319"	"00075b6cb7"	-0.2841
"00075a7319"	"00075b6df8"	-0.3474
"00075a7319"	"00075b6ff6"	-0.209
"00075a7319"	"00075b70ee"	-0.2283
"00075a7319"	"00075b7157"	0.0167
"00075a7319"	"00075b7225"	-0.3044
"00075a7319"	"00075b7c89"	-0.2739
"00075a7319"	"00075b9048"	0.0134
"00075a7319"	"00075d0801"	0.0118
"00075a7319"	"00075d1820"	0.0028
"00075a7319"	"00075d1f3d"	-0.1472
"00075a7319"	"00075d2329"	-0.2588
"00075a7319"	"00075d2b9b"	-0.3165
"00075a7319"	"00075d3941"	-0.5086
"00075a7319"	"00075d3e96"	0.0718
"00075a7319"	"00075d4864"	-0.2804
"00075a7319"	"00075d5961"	-0.1902
"00075a7319"	"00075d5a63"	-0.3048
"00075a7319"	"00075d6150"	0.1107
"00075a7319"	"00075d67d0"	-0.1873
"00075a7319"	"00075d67e2"	-0.4428
"00075a7319"	"00075d73fc"	-0.4109
"00075a7319"	"00075d7729"	-0.2921
"00075a7319"	"00075d778c"	-0.3809
"00075a7319"	"00075d7b9e"	0.0139
"00075a7319"	"00075d7c8f"	-0.161
"00075a7319"	"00075d804d"	0.0918
"00075a7319"	"00075d819f"	0.0045
"00075a7319"	"00075d8601"	0.1233
"00075a7319"	"00075d8c6a"	-0.203
"00075a7319"	"00075dfedc"	-0.2202
"00075a7319"	"00075e05f2"	-0.2755
"00075a7319"	"00075e0837"	-0.1163
"00075a7319"	"00075e092e"	-0.1669
"00075a7319"	"00075e0965"	-0.2449

"00075a7319"	"00075e0bc8"	-0.0919
"00075a7319"	"00075e0fbb"	-0.2787
"00075a7723"	"00075a778b"	0.0851
"00075a7723"	"00075a7b8e"	0.0386
"00075a7723"	"00075a7c79"	0.0051
"00075a7723"	"00075a81b6"	-0.0776
"00075a7723"	"00075a82ac"	0.2172
"00075a7723"	"00075a98e5"	0.2657
"00075a7723"	"00075b0d29"	0.0177
"00075a7723"	"00075b102a"	-0.0109
"00075a7723"	"00075b1074"	0.046
"00075a7723"	"00075b135d"	0.1235
"00075a7723"	"00075b138b"	0.0899
"00075a7723"	"00075b13a0"	-0.1132
"00075a7723"	"00075b13bd"	-0.0204
"00075a7723"	"00075b16a9"	0.0763
"00075a7723"	"00075b1a28"	-0.0442
"00075a7723"	"00075b1a97"	-0.0737
"00075a7723"	"00075b1c7b"	-0.2061
"00075a7723"	"00075b1d24"	0.1305
"00075a7723"	"00075b202b"	-0.2305
"00075a7723"	"00075b22cb"	-0.1627
"00075a7723"	"00075b22da"	-0.0228
"00075a7723"	"00075b2556"	0.1974
"00075a7723"	"00075b25de"	0.0112
"00075a7723"	"00075b260c"	-0.1526
"00075a7723"	"00075b26f1"	0.1265
"00075a7723"	"00075b2920"	0.0059
"00075a7723"	"00075b2a64"	-0.0965
"00075a7723"	"00075b2a9d"	-0.1662
"00075a7723"	"00075b2b37"	-0.3478
"00075a7723"	"00075b2cdd"	-0.2152
"00075a7723"	"00075b3038"	0.1119
"00075a7723"	"00075b30fe"	-0.0744
"00075a7723"	"00075b3362"	0.0683
"00075a7723"	"00075b350a"	-0.098
"00075a7723"	"00075b350e"	-0.0797
"00075a7723"	"00075b3651"	-0.0359
"00075a7723"	"00075b38ca"	-0.0031
"00075a7723"	"00075b39cc"	-0.1605
"00075a7723"	"00075b3e1e"	0.0712
"00075a7723"	"00075b3e57"	-0.0813
"00075a7723"	"00075b4079"	-0.0589
"00075a7723"	"00075b4150"	0.0993
"00075a7723"	"00075b4194"	0.0868
"00075a7723"	"00075b42d5"	0.1126
"00075a7723"	"00075b4424"	-0.1283
"00075a7723"	"00075b4470"	0.1051
"00075a7723"	"00075b47ed"	-0.0528
"00075a7723"	"00075b4850"	0.0244

"00075a7723"	"00075b4ca0"	-0.135
"00075a7723"	"00075b4d7f"	0.2639
"00075a7723"	"00075b520f"	-0.1587
"00075a7723"	"00075b525f"	-0.0314
"00075a7723"	"00075b58f8"	-0.1917
"00075a7723"	"00075b5bcc"	0.1621
"00075a7723"	"00075b5bfa"	-0.293
"00075a7723"	"00075b6339"	0.2862
"00075a7723"	"00075b6658"	-0.1381
"00075a7723"	"00075b679a"	-0.0057
"00075a7723"	"00075b6cb7"	-0.0021
"00075a7723"	"00075b6df8"	0.2559
"00075a7723"	"00075b6ff6"	0.1059
"00075a7723"	"00075b70ee"	0.0104
"00075a7723"	"00075b7157"	-0.0423
"00075a7723"	"00075b7225"	-0.0303
"00075a7723"	"00075b7c89"	0.114
"00075a7723"	"00075b9048"	0.1093
"00075a7723"	"00075d0801"	0.1083
"00075a7723"	"00075d1820"	-0.2088
"00075a7723"	"00075d1f3d"	0.004
"00075a7723"	"00075d2329"	0.0792
"00075a7723"	"00075d2b9b"	-0.0733
"00075a7723"	"00075d3941"	-0.022
"00075a7723"	"00075d3e96"	0.0799
"00075a7723"	"00075d4864"	0.0824
"00075a7723"	"00075d5961"	0.2584
"00075a7723"	"00075d5a63"	-0.0592
"00075a7723"	"00075d6150"	-0.1212
"00075a7723"	"00075d67d0"	-0.0777
"00075a7723"	"00075d67e2"	0.1374
"00075a7723"	"00075d73fc"	0.025
"00075a7723"	"00075d7729"	-0.2027
"00075a7723"	"00075d778c"	-0.0156
"00075a7723"	"00075d7b9e"	0.0665
"00075a7723"	"00075d7c8f"	0.119
"00075a7723"	"00075d804d"	-0.0785
"00075a7723"	"00075d819f"	-0.077
"00075a7723"	"00075d8601"	-0.0327
"00075a7723"	"00075d8c6a"	0.3498
"00075a7723"	"00075dfedc"	0.0139
"00075a7723"	"00075e05f2"	-0.0528
"00075a7723"	"00075e0837"	-0.1848
"00075a7723"	"00075e092e"	0.1105
"00075a7723"	"00075e0965"	0.1882
"00075a7723"	"00075e0bc8"	-0.0718
"00075a7723"	"00075e0fbb"	0.035
"00075a778b"	"00075a7b8e"	0.0574
"00075a778b"	"00075a7c79"	0.0457
"00075a778b"	"00075a81b6"	-0.0482

"00075a778b"	"00075a82ac"	0.2918
"00075a778b"	"00075a98e5"	0.3653
"00075a778b"	"00075b0d29"	0.2122
"00075a778b"	"00075b102a"	−0.2152
"00075a778b"	"00075b1074"	−0.0917
"00075a778b"	"00075b135d"	−0.0766
"00075a778b"	"00075b138b"	0.1865
"00075a778b"	"00075b13a0"	0.2781
"00075a778b"	"00075b13bd"	−0.1438
"00075a778b"	"00075b16a9"	0.0462
"00075a778b"	"00075b1a28"	0.0869
"00075a778b"	"00075b1a97"	0.0968
"00075a778b"	"00075b1c7b"	−0.0904
"00075a778b"	"00075b1d24"	−0.1889
"00075a778b"	"00075b202b"	−0.0836
"00075a778b"	"00075b22cb"	0.1691
"00075a778b"	"00075b22da"	−0.0748
"00075a778b"	"00075b2556"	−0.052
"00075a778b"	"00075b25de"	−0.1489
"00075a778b"	"00075b260c"	−0.1693
"00075a778b"	"00075b26f1"	−0.2187
"00075a778b"	"00075b2920"	−0.0994
"00075a778b"	"00075b2a64"	−0.0884
"00075a778b"	"00075b2a9d"	−0.2744
"00075a778b"	"00075b2b37"	−0.3832
"00075a778b"	"00075b2cdd"	0.1816
"00075a778b"	"00075b3038"	−0.0685
"00075a778b"	"00075b30fe"	−0.157
"00075a778b"	"00075b3362"	−0.333
"00075a778b"	"00075b350a"	−0.2018
"00075a778b"	"00075b350e"	−0.1182
"00075a778b"	"00075b3651"	−0.3107
"00075a778b"	"00075b38ca"	−0.1421
"00075a778b"	"00075b39cc"	−0.2171
"00075a778b"	"00075b3e1e"	0.0536
"00075a778b"	"00075b3e57"	0.2618
"00075a778b"	"00075b4079"	−0.4911
"00075a778b"	"00075b4150"	0.2684
"00075a778b"	"00075b4194"	−0.2246
"00075a778b"	"00075b42d5"	0.0961
"00075a778b"	"00075b4424"	0.2096
"00075a778b"	"00075b4470"	0.1136
"00075a778b"	"00075b47ed"	−0.0511
"00075a778b"	"00075b4850"	−0.055
"00075a778b"	"00075b4ca0"	−0.2853
"00075a778b"	"00075b4d7f"	0.2089
"00075a778b"	"00075b520f"	−0.137
"00075a778b"	"00075b525f"	−0.1971
"00075a778b"	"00075b58f8"	−0.0398
"00075a778b"	"00075b5bcc"	−0.3076

"00075a778b"	"00075b5bfa"	-0.5422
"00075a778b"	"00075b6339"	0.1235
"00075a778b"	"00075b6658"	-0.3254
"00075a778b"	"00075b679a"	-0.0516
"00075a778b"	"00075b6cb7"	-0.27
"00075a778b"	"00075b6df8"	0.0738
"00075a778b"	"00075b6ff6"	0.0472
"00075a778b"	"00075b70ee"	0.0566
"00075a778b"	"00075b7157"	-0.234
"00075a778b"	"00075b7225"	0.0125
"00075a778b"	"00075b7c89"	0.1592
"00075a778b"	"00075b9048"	0.144
"00075a778b"	"00075d0801"	-0.2469
"00075a778b"	"00075d1820"	-0.03
"00075a778b"	"00075d1f3d"	-0.2517
"00075a778b"	"00075d2329"	0.1004
"00075a778b"	"00075d2b9b"	-0.1827
"00075a778b"	"00075d3941"	-0.0568
"00075a778b"	"00075d3e96"	-0.2986
"00075a778b"	"00075d4864"	-0.0279
"00075a778b"	"00075d5961"	0.1537
"00075a778b"	"00075d5a63"	0.0566
"00075a778b"	"00075d6150"	-0.3248
"00075a778b"	"00075d67d0"	-0.3629
"00075a778b"	"00075d67e2"	0.2905
"00075a778b"	"00075d73fc"	0.0833
"00075a778b"	"00075d7729"	-0.2829
"00075a778b"	"00075d778c"	-0.2935
"00075a778b"	"00075d7b9e"	-0.058
"00075a778b"	"00075d7c8f"	-0.0819
"00075a778b"	"00075d804d"	0.0802
"00075a778b"	"00075d819f"	-0.3674
"00075a778b"	"00075d8601"	-0.1797
"00075a778b"	"00075d8c6a"	-0.0057
"00075a778b"	"00075dfedc"	-0.0298
"00075a778b"	"00075e05f2"	0.025
"00075a778b"	"00075e0837"	-0.2502
"00075a778b"	"00075e092e"	-0.0318
"00075a778b"	"00075e0965"	-0.006
"00075a778b"	"00075e0bc8"	0.0637
"00075a778b"	"00075e0fbb"	-0.1622
"00075a7b8e"	"00075a7c79"	0.0675
"00075a7b8e"	"00075a81b6"	0.1692
"00075a7b8e"	"00075a82ac"	0.1732
"00075a7b8e"	"00075a98e5"	-0.1919
"00075a7b8e"	"00075b0d29"	-0.0777
"00075a7b8e"	"00075b102a"	0.1004
"00075a7b8e"	"00075b1074"	-0.0924
"00075a7b8e"	"00075b135d"	-0.0337
"00075a7b8e"	"00075b138b"	0.3304

"00075a7b8e"	"00075b13a0"	−0.0596
"00075a7b8e"	"00075b13bd"	0.0263
"00075a7b8e"	"00075b16a9"	0.0319
"00075a7b8e"	"00075b1a28"	−0.0694
"00075a7b8e"	"00075b1a97"	−0.0591
"00075a7b8e"	"00075b1c7b"	−0.1737
"00075a7b8e"	"00075b1d24"	0.1171
"00075a7b8e"	"00075b202b"	0.3901
"00075a7b8e"	"00075b22cb"	0.1291
"00075a7b8e"	"00075b22da"	−0.0822
"00075a7b8e"	"00075b2556"	−0.2131
"00075a7b8e"	"00075b25de"	0.0455
"00075a7b8e"	"00075b260c"	−0.0938
"00075a7b8e"	"00075b26f1"	0.0262
"00075a7b8e"	"00075b2920"	0.3492
"00075a7b8e"	"00075b2a64"	0.1597
"00075a7b8e"	"00075b2a9d"	0.3228
"00075a7b8e"	"00075b2b37"	−0.3672
"00075a7b8e"	"00075b2cdd"	0.2159
"00075a7b8e"	"00075b3038"	−0.0135
"00075a7b8e"	"00075b30fe"	−0.2191
"00075a7b8e"	"00075b3362"	−0.3147
"00075a7b8e"	"00075b350a"	0.0375
"00075a7b8e"	"00075b350e"	−0.0908
"00075a7b8e"	"00075b3651"	−0.0142
"00075a7b8e"	"00075b38ca"	−0.0893
"00075a7b8e"	"00075b39cc"	0.1483
"00075a7b8e"	"00075b3e1e"	0.0471
"00075a7b8e"	"00075b3e57"	−0.0767
"00075a7b8e"	"00075b4079"	−0.0188
"00075a7b8e"	"00075b4150"	0.1103
"00075a7b8e"	"00075b4194"	0.4474
"00075a7b8e"	"00075b42d5"	0.1378
"00075a7b8e"	"00075b4424"	−0.2357
"00075a7b8e"	"00075b4470"	0.2977
"00075a7b8e"	"00075b47ed"	0.1949
"00075a7b8e"	"00075b4850"	0.2121
"00075a7b8e"	"00075b4ca0"	0.1008
"00075a7b8e"	"00075b4d7f"	0.08
"00075a7b8e"	"00075b520f"	0.1061
"00075a7b8e"	"00075b525f"	−0.1814
"00075a7b8e"	"00075b58f8"	0.0365
"00075a7b8e"	"00075b5bcc"	0.2545
"00075a7b8e"	"00075b5bfa"	−0.2412
"00075a7b8e"	"00075b6339"	−0.0044
"00075a7b8e"	"00075b6658"	−0.435
"00075a7b8e"	"00075b679a"	−0.0179
"00075a7b8e"	"00075b6cb7"	0.2078
"00075a7b8e"	"00075b6df8"	−0.0838
"00075a7b8e"	"00075b6ff6"	0.033

"00075a7b8e"	"00075b70ee"	0.0019
"00075a7b8e"	"00075b7157"	0.1792
"00075a7b8e"	"00075b7225"	0.0717
"00075a7b8e"	"00075b7c89"	-0.1518
"00075a7b8e"	"00075b9048"	0.1218
"00075a7b8e"	"00075d0801"	0.0916
"00075a7b8e"	"00075d1820"	0.1654
"00075a7b8e"	"00075d1f3d"	0.1862
"00075a7b8e"	"00075d2329"	0.0942
"00075a7b8e"	"00075d2b9b"	-0.0077
"00075a7b8e"	"00075d3941"	-0.1782
"00075a7b8e"	"00075d3e96"	0.1675
"00075a7b8e"	"00075d4864"	-0.0331
"00075a7b8e"	"00075d5961"	0.1086
"00075a7b8e"	"00075d5a63"	0.0201
"00075a7b8e"	"00075d6150"	0.053
"00075a7b8e"	"00075d67d0"	-0.1123
"00075a7b8e"	"00075d67e2"	-0.2406
"00075a7b8e"	"00075d73fc"	-0.0981
"00075a7b8e"	"00075d7729"	-0.1229
"00075a7b8e"	"00075d778c"	-0.3338
"00075a7b8e"	"00075d7b9e"	0.149
"00075a7b8e"	"00075d7c8f"	-0.0094
"00075a7b8e"	"00075d804d"	0.1941
"00075a7b8e"	"00075d819f"	0.0799
"00075a7b8e"	"00075d8601"	-0.0105
"00075a7b8e"	"00075d8c6a"	0.0212
"00075a7b8e"	"00075dfedc"	0.0569
"00075a7b8e"	"00075e05f2"	0.0467
"00075a7b8e"	"00075e0837"	0.3137
"00075a7b8e"	"00075e092e"	0.0833
"00075a7b8e"	"00075e0965"	-0.002
"00075a7b8e"	"00075e0bc8"	0.3056
"00075a7b8e"	"00075e0fbb"	-0.1427
"00075a7c79"	"00075a81b6"	0.0705
"00075a7c79"	"00075a82ac"	0.3209
"00075a7c79"	"00075a98e5"	-0.0093
"00075a7c79"	"00075b0d29"	-0.0375
"00075a7c79"	"00075b102a"	0.2078
"00075a7c79"	"00075b1074"	0.0679
"00075a7c79"	"00075b135d"	0.2056
"00075a7c79"	"00075b138b"	0.4812
"00075a7c79"	"00075b13a0"	-0.0719
"00075a7c79"	"00075b13bd"	-0.1146
"00075a7c79"	"00075b16a9"	-0.0631
"00075a7c79"	"00075b1a28"	-0.1481
"00075a7c79"	"00075b1a97"	0.1335
"00075a7c79"	"00075b1c7b"	-0.3863
"00075a7c79"	"00075b1d24"	0.1124
"00075a7c79"	"00075b202b"	0.212

"00075a7c79"	"00075b22cb"	-0.061
"00075a7c79"	"00075b22da"	0.1529
"00075a7c79"	"00075b2556"	0.0731
"00075a7c79"	"00075b25de"	0.1404
"00075a7c79"	"00075b260c"	0.249
"00075a7c79"	"00075b26f1"	-0.0633
"00075a7c79"	"00075b2920"	0.245
"00075a7c79"	"00075b2a64"	0.1353
"00075a7c79"	"00075b2a9d"	0.0674
"00075a7c79"	"00075b2b37"	-0.4418
"00075a7c79"	"00075b2cdd"	0.0664
"00075a7c79"	"00075b3038"	0.3616
"00075a7c79"	"00075b30fe"	0.2477
"00075a7c79"	"00075b3362"	-0.1777
"00075a7c79"	"00075b350a"	0.0792
"00075a7c79"	"00075b350e"	0.0385
"00075a7c79"	"00075b3651"	0.0107
"00075a7c79"	"00075b38ca"	-0.1329
"00075a7c79"	"00075b39cc"	0.1801
"00075a7c79"	"00075b3e1e"	-0.0224
"00075a7c79"	"00075b3e57"	-0.077
"00075a7c79"	"00075b4079"	-0.0669
"00075a7c79"	"00075b4150"	-0.0708
"00075a7c79"	"00075b4194"	0.3288
"00075a7c79"	"00075b42d5"	0.2004
"00075a7c79"	"00075b4424"	-0.0546
"00075a7c79"	"00075b4470"	0.3213
"00075a7c79"	"00075b47ed"	0.0402
"00075a7c79"	"00075b4850"	0.4301
"00075a7c79"	"00075b4ca0"	0.3137
"00075a7c79"	"00075b4d7f"	0.337
"00075a7c79"	"00075b520f"	-0.2367
"00075a7c79"	"00075b525f"	0.1249
"00075a7c79"	"00075b58f8"	-0.1155
"00075a7c79"	"00075b5bcc"	0.2787
"00075a7c79"	"00075b5bfa"	0.044
"00075a7c79"	"00075b6339"	0.3122
"00075a7c79"	"00075b6658"	-0.1138
"00075a7c79"	"00075b679a"	0.2149
"00075a7c79"	"00075b6cb7"	0.2812
"00075a7c79"	"00075b6df8"	0.0472
"00075a7c79"	"00075b6ff6"	0.5043
"00075a7c79"	"00075b70ee"	0.064
"00075a7c79"	"00075b7157"	0.1069
"00075a7c79"	"00075b7225"	0.1352
"00075a7c79"	"00075b7c89"	0.4321
"00075a7c79"	"00075b9048"	0.4776
"00075a7c79"	"00075d0801"	-0.0925
"00075a7c79"	"00075d1820"	-0.2094
"00075a7c79"	"00075d1f3d"	0.3393

"00075a7c79"	"00075d2329"	0.0939
"00075a7c79"	"00075d2b9b"	0.2123
"00075a7c79"	"00075d3941"	0.0616
"00075a7c79"	"00075d3e96"	0.0662
"00075a7c79"	"00075d4864"	−0.2576
"00075a7c79"	"00075d5961"	0.0508
"00075a7c79"	"00075d5a63"	0.3327
"00075a7c79"	"00075d6150"	0.0326
"00075a7c79"	"00075d67d0"	−0.0397
"00075a7c79"	"00075d67e2"	0.1944
"00075a7c79"	"00075d73fc"	0.1254
"00075a7c79"	"00075d7729"	0.0015
"00075a7c79"	"00075d778c"	−0.1074
"00075a7c79"	"00075d7b9e"	−0.0024
"00075a7c79"	"00075d7c8f"	0.4289
"00075a7c79"	"00075d804d"	0.356
"00075a7c79"	"00075d819f"	0.2032
"00075a7c79"	"00075d8601"	−0.1409
"00075a7c79"	"00075d8c6a"	0.0039
"00075a7c79"	"00075dfedc"	0.3961
"00075a7c79"	"00075e05f2"	0.1444
"00075a7c79"	"00075e0837"	0.1605
"00075a7c79"	"00075e092e"	0.1132
"00075a7c79"	"00075e0965"	−0.228
"00075a7c79"	"00075e0bc8"	0.2959
"00075a7c79"	"00075e0fbb"	0.0836
"00075a81b6"	"00075a82ac"	−0.0435
"00075a81b6"	"00075a98e5"	0.0774
"00075a81b6"	"00075b0d29"	0.0515
"00075a81b6"	"00075b102a"	−0.057
"00075a81b6"	"00075b1074"	−0.1269
"00075a81b6"	"00075b135d"	−0.012
"00075a81b6"	"00075b138b"	0.1569
"00075a81b6"	"00075b13a0"	−0.1985
"00075a81b6"	"00075b13bd"	0.0623
"00075a81b6"	"00075b16a9"	−0.0697
"00075a81b6"	"00075b1a28"	−0.2819
"00075a81b6"	"00075b1a97"	0.024
"00075a81b6"	"00075b1c7b"	−0.2613
"00075a81b6"	"00075b1d24"	−0.0259
"00075a81b6"	"00075b202b"	−0.1502
"00075a81b6"	"00075b22cb"	−0.1823
"00075a81b6"	"00075b22da"	−0.0222
"00075a81b6"	"00075b2556"	−0.1143
"00075a81b6"	"00075b25de"	−0.267
"00075a81b6"	"00075b260c"	0.0711
"00075a81b6"	"00075b26f1"	0.1626
"00075a81b6"	"00075b2920"	−0.2645
"00075a81b6"	"00075b2a64"	−0.1978
"00075a81b6"	"00075b2a9d"	−0.0933

"00075a81b6"	"00075b2b37"	−0.4718
"00075a81b6"	"00075b2cdd"	−0.0722
"00075a81b6"	"00075b3038"	−0.1292
"00075a81b6"	"00075b30fe"	−0.1791
"00075a81b6"	"00075b3362"	−0.287
"00075a81b6"	"00075b350a"	−0.0836
"00075a81b6"	"00075b350e"	−0.4645
"00075a81b6"	"00075b3651"	−0.149
"00075a81b6"	"00075b38ca"	−0.4514
"00075a81b6"	"00075b39cc"	0.0293
"00075a81b6"	"00075b3e1e"	−0.0367
"00075a81b6"	"00075b3e57"	−0.0411
"00075a81b6"	"00075b4079"	−0.1237
"00075a81b6"	"00075b4150"	−0.3587
"00075a81b6"	"00075b4194"	−0.0658
"00075a81b6"	"00075b42d5"	−0.2025
"00075a81b6"	"00075b4424"	−0.2577
"00075a81b6"	"00075b4470"	−0.0699
"00075a81b6"	"00075b47ed"	0.0575
"00075a81b6"	"00075b4850"	−0.0041
"00075a81b6"	"00075b4ca0"	0.2597
"00075a81b6"	"00075b4d7f"	0.2408
"00075a81b6"	"00075b520f"	−0.0628
"00075a81b6"	"00075b525f"	0.0908
"00075a81b6"	"00075b58f8"	−0.4498
"00075a81b6"	"00075b5bcc"	0.0219
"00075a81b6"	"00075b5bfa"	−0.267
"00075a81b6"	"00075b6339"	0.375
"00075a81b6"	"00075b6658"	−0.2195
"00075a81b6"	"00075b679a"	5e−04
"00075a81b6"	"00075b6cb7"	−0.0439
"00075a81b6"	"00075b6df8"	0.1353
"00075a81b6"	"00075b6ff6"	−0.0422
"00075a81b6"	"00075b70ee"	−0.1562
"00075a81b6"	"00075b7157"	0.0339
"00075a81b6"	"00075b7225"	−0.1992
"00075a81b6"	"00075b7c89"	−0.1248
"00075a81b6"	"00075b9048"	−0.0595
"00075a81b6"	"00075d0801"	−0.2336
"00075a81b6"	"00075d1820"	−0.1434
"00075a81b6"	"00075d1f3d"	−0.0558
"00075a81b6"	"00075d2329"	−0.0111
"00075a81b6"	"00075d2b9b"	−0.1996
"00075a81b6"	"00075d3941"	−0.1614
"00075a81b6"	"00075d3e96"	−0.2282
"00075a81b6"	"00075d4864"	−0.3577
"00075a81b6"	"00075d5961"	−0.3271
"00075a81b6"	"00075d5a63"	0.1174
"00075a81b6"	"00075d6150"	−0.2157
"00075a81b6"	"00075d67d0"	0.1152

"00075a81b6"	"00075d67e2"	0.361
"00075a81b6"	"00075d73fc"	-0.067
"00075a81b6"	"00075d7729"	0.2378
"00075a81b6"	"00075d778c"	-0.0166
"00075a81b6"	"00075d7b9e"	0.1913
"00075a81b6"	"00075d7c8f"	0.136
"00075a81b6"	"00075d804d"	0.1531
"00075a81b6"	"00075d819f"	-0.1134
"00075a81b6"	"00075d8601"	0.0058
"00075a81b6"	"00075d8c6a"	-0.0158
"00075a81b6"	"00075dfedc"	0.0605
"00075a81b6"	"00075e05f2"	0.1209
"00075a81b6"	"00075e0837"	0.06
"00075a81b6"	"00075e092e"	-0.0721
"00075a81b6"	"00075e0965"	-0.5805
"00075a81b6"	"00075e0bc8"	-0.0541
"00075a81b6"	"00075e0fbb"	-0.2415
"00075a82ac"	"00075a98e5"	0.3958
"00075a82ac"	"00075b0d29"	0.1844
"00075a82ac"	"00075b102a"	0.0803
"00075a82ac"	"00075b1074"	0.0559
"00075a82ac"	"00075b135d"	-0.0371
"00075a82ac"	"00075b138b"	0.4061
"00075a82ac"	"00075b13a0"	0.026
"00075a82ac"	"00075b13bd"	0.3801
"00075a82ac"	"00075b16a9"	0.1412
"00075a82ac"	"00075b1a28"	0.0852
"00075a82ac"	"00075b1a97"	0.3193
"00075a82ac"	"00075b1c7b"	-0.0637
"00075a82ac"	"00075b1d24"	0.0978
"00075a82ac"	"00075b202b"	0.0322
"00075a82ac"	"00075b22cb"	0.3349
"00075a82ac"	"00075b22da"	0.0855
"00075a82ac"	"00075b2556"	0.2224
"00075a82ac"	"00075b25de"	0.1668
"00075a82ac"	"00075b260c"	-0.1028
"00075a82ac"	"00075b26f1"	-0.0447
"00075a82ac"	"00075b2920"	0.0265
"00075a82ac"	"00075b2a64"	-0.1562
"00075a82ac"	"00075b2a9d"	-0.1616
"00075a82ac"	"00075b2b37"	-0.2672
"00075a82ac"	"00075b2cdd"	0.3054
"00075a82ac"	"00075b3038"	0.1873
"00075a82ac"	"00075b30fe"	0.2011
"00075a82ac"	"00075b3362"	-0.1385
"00075a82ac"	"00075b350a"	-0.023
"00075a82ac"	"00075b350e"	0.212
"00075a82ac"	"00075b3651"	0.0663
"00075a82ac"	"00075b38ca"	0.1497
"00075a82ac"	"00075b39cc"	-0.006

"00075a82ac"	"00075b3e1e"	0.0986
"00075a82ac"	"00075b3e57"	0.2775
"00075a82ac"	"00075b4079"	-0.2072
"00075a82ac"	"00075b4150"	-0.1976
"00075a82ac"	"00075b4194"	0.1198
"00075a82ac"	"00075b42d5"	0.2493
"00075a82ac"	"00075b4424"	0.2157
"00075a82ac"	"00075b4470"	0.3361
"00075a82ac"	"00075b47ed"	0.1057
"00075a82ac"	"00075b4850"	0.2593
"00075a82ac"	"00075b4ca0"	0.0061
"00075a82ac"	"00075b4d7f"	0.3501
"00075a82ac"	"00075b520f"	-0.038
"00075a82ac"	"00075b525f"	-0.0854
"00075a82ac"	"00075b58f8"	-0.0308
"00075a82ac"	"00075b5bcc"	0.038
"00075a82ac"	"00075b5bfa"	-0.0314
"00075a82ac"	"00075b6339"	-0.0257
"00075a82ac"	"00075b6658"	0.0383
"00075a82ac"	"00075b679a"	-0.0128
"00075a82ac"	"00075b6cb7"	0.0642
"00075a82ac"	"00075b6df8"	0.3007
"00075a82ac"	"00075b6ff6"	0.1528
"00075a82ac"	"00075b70ee"	-0.1655
"00075a82ac"	"00075b7157"	0.2617
"00075a82ac"	"00075b7225"	0.1173
"00075a82ac"	"00075b7c89"	0.3494
"00075a82ac"	"00075b9048"	0.3287
"00075a82ac"	"00075d0801"	0.0848
"00075a82ac"	"00075d1820"	-0.0119
"00075a82ac"	"00075d1f3d"	0.1575
"00075a82ac"	"00075d2329"	0.1919
"00075a82ac"	"00075d2b9b"	-0.1099
"00075a82ac"	"00075d3941"	0.0632
"00075a82ac"	"00075d3e96"	0.1447
"00075a82ac"	"00075d4864"	-0.0275
"00075a82ac"	"00075d5961"	0.4032
"00075a82ac"	"00075d5a63"	0.01
"00075a82ac"	"00075d6150"	-0.0514
"00075a82ac"	"00075d67d0"	-0.1945
"00075a82ac"	"00075d67e2"	0.1504
"00075a82ac"	"00075d73fc"	0.3271
"00075a82ac"	"00075d7729"	-0.0299
"00075a82ac"	"00075d778c"	-0.2029
"00075a82ac"	"00075d7b9e"	0.1784
"00075a82ac"	"00075d7c8f"	0.1918
"00075a82ac"	"00075d804d"	0.1297
"00075a82ac"	"00075d819f"	0.0373
"00075a82ac"	"00075d8601"	0.164
"00075a82ac"	"00075d8c6a"	0.3044

"00075a82ac"	"00075dfedc"	0.1278
"00075a82ac"	"00075e05f2"	0.0022
"00075a82ac"	"00075e0837"	−0.0123
"00075a82ac"	"00075e092e"	0.0608
"00075a82ac"	"00075e0965"	−0.2374
"00075a82ac"	"00075e0bc8"	0.2502
"00075a82ac"	"00075e0fbb"	0.071
"00075a98e5"	"00075b0d29"	0.3811
"00075a98e5"	"00075b102a"	−0.0849
"00075a98e5"	"00075b1074"	−0.2879
"00075a98e5"	"00075b135d"	−0.0433
"00075a98e5"	"00075b138b"	0.1911
"00075a98e5"	"00075b13a0"	0.024
"00075a98e5"	"00075b13bd"	0.241
"00075a98e5"	"00075b16a9"	−0.1237
"00075a98e5"	"00075b1a28"	0.0152
"00075a98e5"	"00075b1a97"	0.1871
"00075a98e5"	"00075b1c7b"	−0.2528
"00075a98e5"	"00075b1d24"	−0.2603
"00075a98e5"	"00075b202b"	−0.2223
"00075a98e5"	"00075b22cb"	0.0763
"00075a98e5"	"00075b22da"	−0.2817
"00075a98e5"	"00075b2556"	−0.2147
"00075a98e5"	"00075b25de"	−0.1635
"00075a98e5"	"00075b260c"	−0.3448
"00075a98e5"	"00075b26f1"	−0.0887
"00075a98e5"	"00075b2920"	−0.2133
"00075a98e5"	"00075b2a64"	−0.2691
"00075a98e5"	"00075b2a9d"	−0.2672
"00075a98e5"	"00075b2b37"	−0.4337
"00075a98e5"	"00075b2cdd"	0.0378
"00075a98e5"	"00075b3038"	−0.0325
"00075a98e5"	"00075b30fe"	−0.3537
"00075a98e5"	"00075b3362"	−0.3254
"00075a98e5"	"00075b350a"	0.0363
"00075a98e5"	"00075b350e"	−0.049
"00075a98e5"	"00075b3651"	−0.1156
"00075a98e5"	"00075b38ca"	−0.1393
"00075a98e5"	"00075b39cc"	−0.0251
"00075a98e5"	"00075b3e1e"	0.1908
"00075a98e5"	"00075b3e57"	0.2526
"00075a98e5"	"00075b4079"	−0.1286
"00075a98e5"	"00075b4150"	−0.2243
"00075a98e5"	"00075b4194"	0.0425
"00075a98e5"	"00075b42d5"	0.0153
"00075a98e5"	"00075b4424"	−0.1287
"00075a98e5"	"00075b4470"	0.1136
"00075a98e5"	"00075b47ed"	−0.0591
"00075a98e5"	"00075b4850"	−0.1122
"00075a98e5"	"00075b4ca0"	−0.3

"00075a98e5"	"00075b4d7f"	0.1277
"00075a98e5"	"00075b520f"	-0.0298
"00075a98e5"	"00075b525f"	-0.2587
"00075a98e5"	"00075b58f8"	-0.3938
"00075a98e5"	"00075b5bcc"	-0.133
"00075a98e5"	"00075b5bfa"	-0.5941
"00075a98e5"	"00075b6339"	0.1001
"00075a98e5"	"00075b6658"	-0.1334
"00075a98e5"	"00075b679a"	-0.0804
"00075a98e5"	"00075b6cb7"	-0.2135
"00075a98e5"	"00075b6df8"	-0.0149
"00075a98e5"	"00075b6ff6"	-0.2356
"00075a98e5"	"00075b70ee"	-0.4243
"00075a98e5"	"00075b7157"	-0.169
"00075a98e5"	"00075b7225"	-0.1953
"00075a98e5"	"00075b7c89"	0.1466
"00075a98e5"	"00075b9048"	-0.002
"00075a98e5"	"00075d0801"	-0.0915
"00075a98e5"	"00075d1820"	-0.1658
"00075a98e5"	"00075d1f3d"	-0.2579
"00075a98e5"	"00075d2329"	0.0694
"00075a98e5"	"00075d2b9b"	-0.2404
"00075a98e5"	"00075d3941"	0.0251
"00075a98e5"	"00075d3e96"	-0.4132
"00075a98e5"	"00075d4864"	-0.1226
"00075a98e5"	"00075d5961"	0.1842
"00075a98e5"	"00075d5a63"	-0.0395
"00075a98e5"	"00075d6150"	-0.3471
"00075a98e5"	"00075d67d0"	-0.2773
"00075a98e5"	"00075d67e2"	0.5803
"00075a98e5"	"00075d73fc"	0.0855
"00075a98e5"	"00075d7729"	-0.3713
"00075a98e5"	"00075d778c"	-0.4059
"00075a98e5"	"00075d7b9e"	-0.0833
"00075a98e5"	"00075d7c8f"	0.0384
"00075a98e5"	"00075d804d"	0.2028
"00075a98e5"	"00075d819f"	-0.1618
"00075a98e5"	"00075d8601"	0.1308
"00075a98e5"	"00075d8c6a"	0.0853
"00075a98e5"	"00075dfedc"	-0.1046
"00075a98e5"	"00075e05f2"	0.0344
"00075a98e5"	"00075e0837"	-0.3234
"00075a98e5"	"00075e092e"	-0.1189
"00075a98e5"	"00075e0965"	-0.4985
"00075a98e5"	"00075e0bc8"	-0.1356
"00075a98e5"	"00075e0fbb"	-0.3197
"00075b0d29"	"00075b102a"	-0.0325
"00075b0d29"	"00075b1074"	-0.4659
"00075b0d29"	"00075b135d"	0.3365
"00075b0d29"	"00075b138b"	0.0384

"00075b0d29"	"00075b13a0"	-0.2056
"00075b0d29"	"00075b13bd"	0.3006
"00075b0d29"	"00075b16a9"	0.0139
"00075b0d29"	"00075b1a28"	0.1717
"00075b0d29"	"00075b1a97"	0.0809
"00075b0d29"	"00075b1c7b"	-0.2018
"00075b0d29"	"00075b1d24"	-0.1858
"00075b0d29"	"00075b202b"	-0.0795
"00075b0d29"	"00075b22cb"	0.0641
"00075b0d29"	"00075b22da"	-0.2993
"00075b0d29"	"00075b2556"	-0.1865
"00075b0d29"	"00075b25de"	0.0048
"00075b0d29"	"00075b260c"	-0.1059
"00075b0d29"	"00075b26f1"	-0.2275
"00075b0d29"	"00075b2920"	-0.1176
"00075b0d29"	"00075b2a64"	-0.1087
"00075b0d29"	"00075b2a9d"	-0.1992
"00075b0d29"	"00075b2b37"	-0.3151
"00075b0d29"	"00075b2cdd"	0.0293
"00075b0d29"	"00075b3038"	-0.1107
"00075b0d29"	"00075b30fe"	-0.3994
"00075b0d29"	"00075b3362"	-0.3761
"00075b0d29"	"00075b350a"	-0.0485
"00075b0d29"	"00075b350e"	-0.0317
"00075b0d29"	"00075b3651"	-0.2164
"00075b0d29"	"00075b38ca"	-0.1562
"00075b0d29"	"00075b39cc"	-0.1424
"00075b0d29"	"00075b3e1e"	0.0391
"00075b0d29"	"00075b3e57"	0.0549
"00075b0d29"	"00075b4079"	-0.1009
"00075b0d29"	"00075b4150"	0.0225
"00075b0d29"	"00075b4194"	-0.0494
"00075b0d29"	"00075b42d5"	-0.0175
"00075b0d29"	"00075b4424"	0.0193
"00075b0d29"	"00075b4470"	0.0905
"00075b0d29"	"00075b47ed"	-0.238
"00075b0d29"	"00075b4850"	-0.0626
"00075b0d29"	"00075b4ca0"	-0.0105
"00075b0d29"	"00075b4d7f"	0.2697
"00075b0d29"	"00075b520f"	0.1479
"00075b0d29"	"00075b525f"	-0.2869
"00075b0d29"	"00075b58f8"	0.0528
"00075b0d29"	"00075b5bcc"	-0.1739
"00075b0d29"	"00075b5bfa"	-0.5449
"00075b0d29"	"00075b6339"	0.2003
"00075b0d29"	"00075b6658"	-0.0296
"00075b0d29"	"00075b679a"	0.0416
"00075b0d29"	"00075b6cb7"	-0.1525
"00075b0d29"	"00075b6df8"	0.1035
"00075b0d29"	"00075b6ff6"	-0.2289

"00075b0d29"	"00075b70ee"	-0.0083
"00075b0d29"	"00075b7157"	-0.0747
"00075b0d29"	"00075b7225"	-0.0815
"00075b0d29"	"00075b7c89"	0.2673
"00075b0d29"	"00075b9048"	0.0494
"00075b0d29"	"00075d0801"	0.1167
"00075b0d29"	"00075d1820"	-0.06
"00075b0d29"	"00075d1f3d"	-0.194
"00075b0d29"	"00075d2329"	-0.1625
"00075b0d29"	"00075d2b9b"	-0.169
"00075b0d29"	"00075d3941"	-0.1419
"00075b0d29"	"00075d3e96"	-0.3663
"00075b0d29"	"00075d4864"	-0.1169
"00075b0d29"	"00075d5961"	0.1667
"00075b0d29"	"00075d5a63"	0.026
"00075b0d29"	"00075d6150"	-0.3739
"00075b0d29"	"00075d67d0"	-0.533
"00075b0d29"	"00075d67e2"	0.2639
"00075b0d29"	"00075d73fc"	0.2361
"00075b0d29"	"00075d7729"	0.0818
"00075b0d29"	"00075d778c"	-0.4456
"00075b0d29"	"00075d7b9e"	0.0029
"00075b0d29"	"00075d7c8f"	0.1
"00075b0d29"	"00075d804d"	-0.1635
"00075b0d29"	"00075d819f"	-0.2325
"00075b0d29"	"00075d8601"	0.2862
"00075b0d29"	"00075d8c6a"	0.2635
"00075b0d29"	"00075dfedc"	0.2377
"00075b0d29"	"00075e05f2"	-0.1209
"00075b0d29"	"00075e0837"	-0.2918
"00075b0d29"	"00075e092e"	-0.3369
"00075b0d29"	"00075e0965"	-0.1201
"00075b0d29"	"00075e0bc8"	0.1845
"00075b0d29"	"00075e0fbb"	-0.4111
"00075b102a"	"00075b1074"	0.1212
"00075b102a"	"00075b135d"	-0.0827
"00075b102a"	"00075b138b"	0.392
"00075b102a"	"00075b13a0"	-0.4869
"00075b102a"	"00075b13bd"	-0.039
"00075b102a"	"00075b16a9"	0.1981
"00075b102a"	"00075b1a28"	0.0313
"00075b102a"	"00075b1a97"	0.0719
"00075b102a"	"00075b1c7b"	-0.0389
"00075b102a"	"00075b1d24"	-0.0282
"00075b102a"	"00075b202b"	0.3001
"00075b102a"	"00075b22cb"	0.128
"00075b102a"	"00075b22da"	0.2361
"00075b102a"	"00075b2556"	0.2556
"00075b102a"	"00075b25de"	0.055
"00075b102a"	"00075b260c"	0.3128

"00075b102a"	"00075b26f1"	-5e-04
"00075b102a"	"00075b2920"	0.1899
"00075b102a"	"00075b2a64"	0.0927
"00075b102a"	"00075b2a9d"	0.4668
"00075b102a"	"00075b2b37"	-0.2465
"00075b102a"	"00075b2cdd"	0.1902
"00075b102a"	"00075b3038"	0.0705
"00075b102a"	"00075b30fe"	0.1806
"00075b102a"	"00075b3362"	-0.2658
"00075b102a"	"00075b350a"	-0.0438
"00075b102a"	"00075b350e"	0.116
"00075b102a"	"00075b3651"	-0.1514
"00075b102a"	"00075b38ca"	-0.1409
"00075b102a"	"00075b39cc"	-0.0763
"00075b102a"	"00075b3e1e"	0.2974
"00075b102a"	"00075b3e57"	0.0046
"00075b102a"	"00075b4079"	-0.2038
"00075b102a"	"00075b4150"	-0.0224
"00075b102a"	"00075b4194"	0.0175
"00075b102a"	"00075b42d5"	0.1189
"00075b102a"	"00075b4424"	-0.2182
"00075b102a"	"00075b4470"	0.3526
"00075b102a"	"00075b47ed"	-0.2063
"00075b102a"	"00075b4850"	0.2577
"00075b102a"	"00075b4ca0"	-0.0113
"00075b102a"	"00075b4d7f"	0.0791
"00075b102a"	"00075b520f"	-0.2292
"00075b102a"	"00075b525f"	-0.2583
"00075b102a"	"00075b58f8"	0.1125
"00075b102a"	"00075b5bcc"	-0.0028
"00075b102a"	"00075b5bfa"	-0.2817
"00075b102a"	"00075b6339"	0.0691
"00075b102a"	"00075b6658"	-0.2575
"00075b102a"	"00075b679a"	-0.0293
"00075b102a"	"00075b6cb7"	0.5034
"00075b102a"	"00075b6df8"	-0.1917
"00075b102a"	"00075b6ff6"	-0.0846
"00075b102a"	"00075b70ee"	0.0127
"00075b102a"	"00075b7157"	-0.092
"00075b102a"	"00075b7225"	-0.0221
"00075b102a"	"00075b7c89"	0.0377
"00075b102a"	"00075b9048"	0.0167
"00075b102a"	"00075d0801"	-0.0158
"00075b102a"	"00075d1820"	-0.1606
"00075b102a"	"00075d1f3d"	0.2759
"00075b102a"	"00075d2329"	-0.1857
"00075b102a"	"00075d2b9b"	0.2414
"00075b102a"	"00075d3941"	-0.1062
"00075b102a"	"00075d3e96"	-0.0196
"00075b102a"	"00075d4864"	-0.2082

"00075b102a"	"00075d5961"	−0.0491
"00075b102a"	"00075d5a63"	0.044
"00075b102a"	"00075d6150"	−0.0812
"00075b102a"	"00075d67d0"	−0.2056
"00075b102a"	"00075d67e2"	0.0335
"00075b102a"	"00075d73fc"	0.062
"00075b102a"	"00075d7729"	−0.3049
"00075b102a"	"00075d778c"	−0.3864
"00075b102a"	"00075d7b9e"	−0.0781
"00075b102a"	"00075d7c8f"	0.1279
"00075b102a"	"00075d804d"	0.1165
"00075b102a"	"00075d819f"	0.5742
"00075b102a"	"00075d8601"	0.0207
"00075b102a"	"00075d8c6a"	−0.0249
"00075b102a"	"00075dfedc"	0.1215
"00075b102a"	"00075e05f2"	0.1899
"00075b102a"	"00075e0837"	0.5077
"00075b102a"	"00075e092e"	−0.0111
"00075b102a"	"00075e0965"	−0.2143
"00075b102a"	"00075e0bc8"	0.1168
"00075b102a"	"00075e0fbb"	−0.1392
"00075b1074"	"00075b135d"	−0.0864
"00075b1074"	"00075b138b"	0.1161
"00075b1074"	"00075b13a0"	−0.2634
"00075b1074"	"00075b13bd"	0.0225
"00075b1074"	"00075b16a9"	−0.25
"00075b1074"	"00075b1a28"	0.0273
"00075b1074"	"00075b1a97"	−0.0599
"00075b1074"	"00075b1c7b"	−0.4617
"00075b1074"	"00075b1d24"	0.0198
"00075b1074"	"00075b202b"	−0.0721
"00075b1074"	"00075b22cb"	−0.0115
"00075b1074"	"00075b22da"	0.6748
"00075b1074"	"00075b2556"	0.4899
"00075b1074"	"00075b25de"	−0.4162
"00075b1074"	"00075b260c"	−0.1377
"00075b1074"	"00075b26f1"	−0.0823
"00075b1074"	"00075b2920"	−0.2515
"00075b1074"	"00075b2a64"	−0.3268
"00075b1074"	"00075b2a9d"	−0.0053
"00075b1074"	"00075b2b37"	−0.3114
"00075b1074"	"00075b2cdd"	−0.0191
"00075b1074"	"00075b3038"	−0.1817
"00075b1074"	"00075b30fe"	0.5799
"00075b1074"	"00075b3362"	−0.0848
"00075b1074"	"00075b350a"	0.2001
"00075b1074"	"00075b350e"	−0.1384
"00075b1074"	"00075b3651"	−0.1826
"00075b1074"	"00075b38ca"	0.0274
"00075b1074"	"00075b39cc"	−0.0935

"00075b1074"	"00075b3e1e"	-0.0319
"00075b1074"	"00075b3e57"	-0.0689
"00075b1074"	"00075b4079"	-0.0424
"00075b1074"	"00075b4150"	-0.4001
"00075b1074"	"00075b4194"	-0.1857
"00075b1074"	"00075b42d5"	0.1416
"00075b1074"	"00075b4424"	-0.2286
"00075b1074"	"00075b4470"	0.2149
"00075b1074"	"00075b47ed"	0.0152
"00075b1074"	"00075b4850"	0.0708
"00075b1074"	"00075b4ca0"	0.0781
"00075b1074"	"00075b4d7f"	0.0848
"00075b1074"	"00075b520f"	-0.455
"00075b1074"	"00075b525f"	0.1032
"00075b1074"	"00075b58f8"	-0.204
"00075b1074"	"00075b5bcc"	0.0045
"00075b1074"	"00075b5bfa"	-0.4957
"00075b1074"	"00075b6339"	-0.0451
"00075b1074"	"00075b6658"	-0.2012
"00075b1074"	"00075b679a"	-0.1733
"00075b1074"	"00075b6cb7"	0.0247
"00075b1074"	"00075b6df8"	0.0075
"00075b1074"	"00075b6ff6"	0.0438
"00075b1074"	"00075b70ee"	-0.2019
"00075b1074"	"00075b7157"	-0.0244
"00075b1074"	"00075b7225"	-0.0189
"00075b1074"	"00075b7c89"	-0.0016
"00075b1074"	"00075b9048"	-0.261
"00075b1074"	"00075d0801"	-0.2468
"00075b1074"	"00075d1820"	-0.1736
"00075b1074"	"00075d1f3d"	0.0264
"00075b1074"	"00075d2329"	0.0497
"00075b1074"	"00075d2b9b"	-0.156
"00075b1074"	"00075d3941"	-0.2222
"00075b1074"	"00075d3e96"	-0.0112
"00075b1074"	"00075d4864"	0.0024
"00075b1074"	"00075d5961"	-0.0983
"00075b1074"	"00075d5a63"	-0.0089
"00075b1074"	"00075d6150"	-0.2906
"00075b1074"	"00075d67d0"	-0.082
"00075b1074"	"00075d67e2"	-0.2326
"00075b1074"	"00075d73fc"	-0.2402
"00075b1074"	"00075d7729"	-0.1601
"00075b1074"	"00075d778c"	-0.0793
"00075b1074"	"00075d7b9e"	-0.1577
"00075b1074"	"00075d7c8f"	0.268
"00075b1074"	"00075d804d"	-0.1006
"00075b1074"	"00075d819f"	0.0304
"00075b1074"	"00075d8601"	-0.4321
"00075b1074"	"00075d8c6a"	-0.1102

"00075b1074"	"00075dfedc"	-0.2052
"00075b1074"	"00075e05f2"	-0.2345
"00075b1074"	"00075e0837"	0.1053
"00075b1074"	"00075e092e"	0.5737
"00075b1074"	"00075e0965"	-0.351
"00075b1074"	"00075e0bc8"	-0.0858
"00075b1074"	"00075e0fbb"	0.6331
"00075b135d"	"00075b138b"	0.0664
"00075b135d"	"00075b13a0"	-0.4018
"00075b135d"	"00075b13bd"	0.0923
"00075b135d"	"00075b16a9"	-0.1654
"00075b135d"	"00075b1a28"	-0.0064
"00075b135d"	"00075b1a97"	-0.0416
"00075b135d"	"00075b1c7b"	-0.3225
"00075b135d"	"00075b1d24"	-0.1294
"00075b135d"	"00075b202b"	-0.1272
"00075b135d"	"00075b22cb"	-0.2786
"00075b135d"	"00075b22da"	-0.1553
"00075b135d"	"00075b2556"	-0.1607
"00075b135d"	"00075b25de"	-0.1867
"00075b135d"	"00075b260c"	-0.1594
"00075b135d"	"00075b26f1"	-0.1113
"00075b135d"	"00075b2920"	-0.2043
"00075b135d"	"00075b2a64"	-0.103
"00075b135d"	"00075b2a9d"	-0.0495
"00075b135d"	"00075b2b37"	-0.2952
"00075b135d"	"00075b2cdd"	-0.0402
"00075b135d"	"00075b3038"	-0.0537
"00075b135d"	"00075b30fe"	-0.2005
"00075b135d"	"00075b3362"	-0.2486
"00075b135d"	"00075b350a"	-0.1957
"00075b135d"	"00075b350e"	0.15
"00075b135d"	"00075b3651"	-0.3419
"00075b135d"	"00075b38ca"	-0.2107
"00075b135d"	"00075b39cc"	-0.0781
"00075b135d"	"00075b3e1e"	-0.1729
"00075b135d"	"00075b3e57"	-0.3285
"00075b135d"	"00075b4079"	-0.1269
"00075b135d"	"00075b4150"	-0.14
"00075b135d"	"00075b4194"	0.1645
"00075b135d"	"00075b42d5"	-0.181
"00075b135d"	"00075b4424"	-0.3901
"00075b135d"	"00075b4470"	0.0409
"00075b135d"	"00075b47ed"	-0.1152
"00075b135d"	"00075b4850"	0.001
"00075b135d"	"00075b4ca0"	0.4159
"00075b135d"	"00075b4d7f"	0.3075
"00075b135d"	"00075b520f"	0.1187
"00075b135d"	"00075b525f"	-0.242
"00075b135d"	"00075b58f8"	0.0122

"00075b135d"	"00075b5bcc"	0.0038
"00075b135d"	"00075b5bfa"	−0.5131
"00075b135d"	"00075b6339"	0.1243
"00075b135d"	"00075b6658"	−0.2327
"00075b135d"	"00075b679a"	−0.0338
"00075b135d"	"00075b6cb7"	−0.1621
"00075b135d"	"00075b6df8"	0.0449
"00075b135d"	"00075b6ff6"	−0.2477
"00075b135d"	"00075b70ee"	0.0743
"00075b135d"	"00075b7157"	−0.0036
"00075b135d"	"00075b7225"	0.0629
"00075b135d"	"00075b7c89"	0.2541
"00075b135d"	"00075b9048"	0.0759
"00075b135d"	"00075d0801"	−0.0353
"00075b135d"	"00075d1820"	−0.0602
"00075b135d"	"00075d1f3d"	−0.0582
"00075b135d"	"00075d2329"	−0.1027
"00075b135d"	"00075d2b9b"	−0.1184
"00075b135d"	"00075d3941"	−0.111
"00075b135d"	"00075d3e96"	−0.0796
"00075b135d"	"00075d4864"	−0.2464
"00075b135d"	"00075d5961"	0.2269
"00075b135d"	"00075d5a63"	−0.2132
"00075b135d"	"00075d6150"	0.0024
"00075b135d"	"00075d67d0"	−0.4906
"00075b135d"	"00075d67e2"	0.1309
"00075b135d"	"00075d73fc"	0.0077
"00075b135d"	"00075d7729"	0.1178
"00075b135d"	"00075d778c"	−0.5143
"00075b135d"	"00075d7b9e"	−0.1132
"00075b135d"	"00075d7c8f"	0.3133
"00075b135d"	"00075d804d"	−0.1145
"00075b135d"	"00075d819f"	−0.0192
"00075b135d"	"00075d8601"	−0.015
"00075b135d"	"00075d8c6a"	0.1785
"00075b135d"	"00075dfedc"	0.1804
"00075b135d"	"00075e05f2"	−0.3131
"00075b135d"	"00075e0837"	−0.331
"00075b135d"	"00075e092e"	−0.0919
"00075b135d"	"00075e0965"	−0.243
"00075b135d"	"00075e0bc8"	0.0245
"00075b135d"	"00075e0fbb"	−0.1904
"00075b138b"	"00075b13a0"	−0.1281
"00075b138b"	"00075b13bd"	0.0938
"00075b138b"	"00075b16a9"	0.0224
"00075b138b"	"00075b1a28"	−0.0236
"00075b138b"	"00075b1a97"	0.2842
"00075b138b"	"00075b1c7b"	−0.188
"00075b138b"	"00075b1d24"	0.1797
"00075b138b"	"00075b202b"	0.455

"00075b138b"	"00075b22cb"	0.2252
"00075b138b"	"00075b22da"	0.2708
"00075b138b"	"00075b2556"	0.1406
"00075b138b"	"00075b25de"	0.0385
"00075b138b"	"00075b260c"	0.3197
"00075b138b"	"00075b26f1"	0.0777
"00075b138b"	"00075b2920"	0.0796
"00075b138b"	"00075b2a64"	0.2335
"00075b138b"	"00075b2a9d"	0.3117
"00075b138b"	"00075b2b37"	-0.4175
"00075b138b"	"00075b2cdd"	0.1928
"00075b138b"	"00075b3038"	0.2663
"00075b138b"	"00075b30fe"	0.17
"00075b138b"	"00075b3362"	-0.2196
"00075b138b"	"00075b350a"	0.1944
"00075b138b"	"00075b350e"	0.1698
"00075b138b"	"00075b3651"	-0.0292
"00075b138b"	"00075b38ca"	-0.0143
"00075b138b"	"00075b39cc"	0.0205
"00075b138b"	"00075b3e1e"	0.2903
"00075b138b"	"00075b3e57"	-0.0024
"00075b138b"	"00075b4079"	-0.0607
"00075b138b"	"00075b4150"	-0.0341
"00075b138b"	"00075b4194"	0.4128
"00075b138b"	"00075b42d5"	0.279
"00075b138b"	"00075b4424"	-0.0594
"00075b138b"	"00075b4470"	0.4796
"00075b138b"	"00075b47ed"	0.0278
"00075b138b"	"00075b4850"	0.5392
"00075b138b"	"00075b4ca0"	0.1104
"00075b138b"	"00075b4d7f"	0.2906
"00075b138b"	"00075b520f"	-0.0484
"00075b138b"	"00075b525f"	0.029
"00075b138b"	"00075b58f8"	-0.005
"00075b138b"	"00075b5bcc"	0.2362
"00075b138b"	"00075b5bfa"	-0.1093
"00075b138b"	"00075b6339"	0.23
"00075b138b"	"00075b6658"	-0.1547
"00075b138b"	"00075b679a"	0.1635
"00075b138b"	"00075b6cb7"	0.3603
"00075b138b"	"00075b6df8"	-0.0171
"00075b138b"	"00075b6ff6"	0.33
"00075b138b"	"00075b70ee"	0.0321
"00075b138b"	"00075b7157"	0.0289
"00075b138b"	"00075b7225"	0.2113
"00075b138b"	"00075b7c89"	0.118
"00075b138b"	"00075b9048"	0.3471
"00075b138b"	"00075d0801"	-0.0469
"00075b138b"	"00075d1820"	-0.0172
"00075b138b"	"00075d1f3d"	0.681

"00075b138b"	"00075d2329"	0.1006
"00075b138b"	"00075d2b9b"	0.3652
"00075b138b"	"00075d3941"	0.064
"00075b138b"	"00075d3e96"	−0.0078
"00075b138b"	"00075d4864"	−0.1358
"00075b138b"	"00075d5961"	0.1835
"00075b138b"	"00075d5a63"	0.3135
"00075b138b"	"00075d6150"	−0.1
"00075b138b"	"00075d67d0"	0.102
"00075b138b"	"00075d67e2"	0.39
"00075b138b"	"00075d73fc"	0.1998
"00075b138b"	"00075d7729"	−0.1385
"00075b138b"	"00075d778c"	−0.2139
"00075b138b"	"00075d7b9e"	0.0585
"00075b138b"	"00075d7c8f"	0.4196
"00075b138b"	"00075d804d"	0.4547
"00075b138b"	"00075d819f"	0.4138
"00075b138b"	"00075d8601"	0.0144
"00075b138b"	"00075d8c6a"	−0.0102
"00075b138b"	"00075dfedc"	0.1703
"00075b138b"	"00075e05f2"	0.2496
"00075b138b"	"00075e0837"	0.4503
"00075b138b"	"00075e092e"	0.2391
"00075b138b"	"00075e0965"	−0.1388
"00075b138b"	"00075e0bc8"	0.5311
"00075b138b"	"00075e0fbb"	0.1573
"00075b13a0"	"00075b13bd"	−0.0658
"00075b13a0"	"00075b16a9"	−0.2471
"00075b13a0"	"00075b1a28"	−0.4092
"00075b13a0"	"00075b1a97"	−0.31
"00075b13a0"	"00075b1c7b"	−0.4187
"00075b13a0"	"00075b1d24"	−0.2147
"00075b13a0"	"00075b202b"	−0.5089
"00075b13a0"	"00075b22cb"	−0.0705
"00075b13a0"	"00075b22da"	−0.0341
"00075b13a0"	"00075b2556"	−0.1706
"00075b13a0"	"00075b25de"	−0.2081
"00075b13a0"	"00075b260c"	−0.2346
"00075b13a0"	"00075b26f1"	−0.563
"00075b13a0"	"00075b2920"	−0.4121
"00075b13a0"	"00075b2a64"	−0.4264
"00075b13a0"	"00075b2a9d"	−0.5895
"00075b13a0"	"00075b2b37"	−0.5302
"00075b13a0"	"00075b2cdd"	−0.3511
"00075b13a0"	"00075b3038"	−0.2368
"00075b13a0"	"00075b30fe"	−0.1784
"00075b13a0"	"00075b3362"	−0.2983
"00075b13a0"	"00075b350a"	−0.2654
"00075b13a0"	"00075b350e"	−0.2987
"00075b13a0"	"00075b3651"	−0.5807

"00075b13a0"	"00075b38ca"	-0.4706
"00075b13a0"	"00075b39cc"	-0.2625
"00075b13a0"	"00075b3e1e"	-0.2554
"00075b13a0"	"00075b3e57"	0.0776
"00075b13a0"	"00075b4079"	-0.3137
"00075b13a0"	"00075b4150"	-0.1672
"00075b13a0"	"00075b4194"	-0.2211
"00075b13a0"	"00075b42d5"	-0.1243
"00075b13a0"	"00075b4424"	-0.3898
"00075b13a0"	"00075b4470"	-0.195
"00075b13a0"	"00075b47ed"	-0.1995
"00075b13a0"	"00075b4850"	-0.2569
"00075b13a0"	"00075b4ca0"	-0.451
"00075b13a0"	"00075b4d7f"	0.0837
"00075b13a0"	"00075b520f"	-0.2186
"00075b13a0"	"00075b525f"	-0.2843
"00075b13a0"	"00075b58f8"	-0.2913
"00075b13a0"	"00075b5bcc"	-0.3386
"00075b13a0"	"00075b5bfa"	-0.3489
"00075b13a0"	"00075b6339"	0.1119
"00075b13a0"	"00075b6658"	-0.2892
"00075b13a0"	"00075b679a"	-0.1543
"00075b13a0"	"00075b6cb7"	-0.3091
"00075b13a0"	"00075b6df8"	-0.0344
"00075b13a0"	"00075b6ff6"	0.1271
"00075b13a0"	"00075b70ee"	-0.4151
"00075b13a0"	"00075b7157"	-0.2055
"00075b13a0"	"00075b7225"	-0.0169
"00075b13a0"	"00075b7c89"	0.076
"00075b13a0"	"00075b9048"	0.0873
"00075b13a0"	"00075d0801"	-0.1725
"00075b13a0"	"00075d1820"	-0.2043
"00075b13a0"	"00075d1f3d"	-0.4522
"00075b13a0"	"00075d2329"	-0.0984
"00075b13a0"	"00075d2b9b"	-0.3738
"00075b13a0"	"00075d3941"	-0.637
"00075b13a0"	"00075d3e96"	-0.5714
"00075b13a0"	"00075d4864"	-0.1415
"00075b13a0"	"00075d5961"	-0.1806
"00075b13a0"	"00075d5a63"	-0.102
"00075b13a0"	"00075d6150"	-0.5052
"00075b13a0"	"00075d67d0"	-0.219
"00075b13a0"	"00075d67e2"	0.0872
"00075b13a0"	"00075d73fc"	-0.1564
"00075b13a0"	"00075d7729"	-0.4323
"00075b13a0"	"00075d778c"	-0.0642
"00075b13a0"	"00075d7b9e"	-0.2129
"00075b13a0"	"00075d7c8f"	-0.0831
"00075b13a0"	"00075d804d"	0.0104
"00075b13a0"	"00075d819f"	-0.4591

"00075b13a0"	"00075d8601"	−0.4063
"00075b13a0"	"00075d8c6a"	−0.1187
"00075b13a0"	"00075dfedc"	−0.1588
"00075b13a0"	"00075e05f2"	0.0902
"00075b13a0"	"00075e0837"	−0.3803
"00075b13a0"	"00075e092e"	−0.056
"00075b13a0"	"00075e0965"	−0.2562
"00075b13a0"	"00075e0bc8"	0.0417
"00075b13a0"	"00075e0fbb"	−0.1635
"00075b13bd"	"00075b16a9"	−0.07
"00075b13bd"	"00075b1a28"	0.1367
"00075b13bd"	"00075b1a97"	0.4372
"00075b13bd"	"00075b1c7b"	−0.2724
"00075b13bd"	"00075b1d24"	0.0668
"00075b13bd"	"00075b202b"	−0.1674
"00075b13bd"	"00075b22cb"	0.2332
"00075b13bd"	"00075b22da"	−0.0124
"00075b13bd"	"00075b2556"	−0.0108
"00075b13bd"	"00075b25de"	−0.01
"00075b13bd"	"00075b260c"	−0.3226
"00075b13bd"	"00075b26f1"	0.0849
"00075b13bd"	"00075b2920"	−0.3703
"00075b13bd"	"00075b2a64"	−0.2831
"00075b13bd"	"00075b2a9d"	−0.3242
"00075b13bd"	"00075b2b37"	−0.1566
"00075b13bd"	"00075b2cdd"	0.1543
"00075b13bd"	"00075b3038"	−0.0513
"00075b13bd"	"00075b30fe"	−0.0401
"00075b13bd"	"00075b3362"	−0.1016
"00075b13bd"	"00075b350a"	0.0133
"00075b13bd"	"00075b350e"	−0.1167
"00075b13bd"	"00075b3651"	0.1463
"00075b13bd"	"00075b38ca"	−0.1378
"00075b13bd"	"00075b39cc"	−0.0173
"00075b13bd"	"00075b3e1e"	−0.1143
"00075b13bd"	"00075b3e57"	0.3638
"00075b13bd"	"00075b4079"	−0.0289
"00075b13bd"	"00075b4150"	−0.4347
"00075b13bd"	"00075b4194"	−0.0062
"00075b13bd"	"00075b42d5"	0.0554
"00075b13bd"	"00075b4424"	−0.127
"00075b13bd"	"00075b4470"	0.1345
"00075b13bd"	"00075b47ed"	−0.0368
"00075b13bd"	"00075b4850"	0.0454
"00075b13bd"	"00075b4ca0"	0.0321
"00075b13bd"	"00075b4d7f"	0.2852
"00075b13bd"	"00075b520f"	0.2044
"00075b13bd"	"00075b525f"	−0.0725
"00075b13bd"	"00075b58f8"	−0.2255
"00075b13bd"	"00075b5bcc"	0.0919

"00075b13bd"	"00075b5bfa"	-0.3326
"00075b13bd"	"00075b6339"	-0.0428
"00075b13bd"	"00075b6658"	0.2681
"00075b13bd"	"00075b679a"	0.014
"00075b13bd"	"00075b6cb7"	-0.1664
"00075b13bd"	"00075b6df8"	0.1642
"00075b13bd"	"00075b6ff6"	-0.0988
"00075b13bd"	"00075b70ee"	-0.3481
"00075b13bd"	"00075b7157"	0.179
"00075b13bd"	"00075b7225"	0.0225
"00075b13bd"	"00075b7c89"	0.0734
"00075b13bd"	"00075b9048"	-0.0979
"00075b13bd"	"00075d0801"	-0.0843
"00075b13bd"	"00075d1820"	0.1913
"00075b13bd"	"00075d1f3d"	-0.096
"00075b13bd"	"00075d2329"	-0.0355
"00075b13bd"	"00075d2b9b"	-0.4144
"00075b13bd"	"00075d3941"	0.0793
"00075b13bd"	"00075d3e96"	0.0542
"00075b13bd"	"00075d4864"	-0.128
"00075b13bd"	"00075d5961"	0.0765
"00075b13bd"	"00075d5a63"	-0.0731
"00075b13bd"	"00075d6150"	-0.0136
"00075b13bd"	"00075d67d0"	-0.2673
"00075b13bd"	"00075d67e2"	0.0885
"00075b13bd"	"00075d73fc"	0.1277
"00075b13bd"	"00075d7729"	0.1401
"00075b13bd"	"00075d778c"	-0.2832
"00075b13bd"	"00075d7b9e"	0.0685
"00075b13bd"	"00075d7c8f"	0.1181
"00075b13bd"	"00075d804d"	0.0517
"00075b13bd"	"00075d819f"	-0.0915
"00075b13bd"	"00075d8601"	0.5076
"00075b13bd"	"00075d8c6a"	0.2423
"00075b13bd"	"00075dfedc"	0.0727
"00075b13bd"	"00075e05f2"	-0.2976
"00075b13bd"	"00075e0837"	-0.2242
"00075b13bd"	"00075e092e"	-0.0256
"00075b13bd"	"00075e0965"	-0.4898
"00075b13bd"	"00075e0bc8"	0.0113
"00075b13bd"	"00075e0fbb"	-0.2566
"00075b16a9"	"00075b1a28"	0.1502
"00075b16a9"	"00075b1a97"	0.188
"00075b16a9"	"00075b1c7b"	0.4213
"00075b16a9"	"00075b1d24"	0.1706
"00075b16a9"	"00075b202b"	-0.1378
"00075b16a9"	"00075b22cb"	0.453
"00075b16a9"	"00075b22da"	-0.1514
"00075b16a9"	"00075b2556"	0.0052
"00075b16a9"	"00075b25de"	0.1511

"00075b16a9"	"00075b260c"	-0.0646
"00075b16a9"	"00075b26f1"	-0.1214
"00075b16a9"	"00075b2920"	0.0607
"00075b16a9"	"00075b2a64"	-0.0803
"00075b16a9"	"00075b2a9d"	-0.1686
"00075b16a9"	"00075b2b37"	-0.0886
"00075b16a9"	"00075b2cdd"	0.1777
"00075b16a9"	"00075b3038"	0.027
"00075b16a9"	"00075b30fe"	-0.1776
"00075b16a9"	"00075b3362"	-0.0948
"00075b16a9"	"00075b350a"	-0.3532
"00075b16a9"	"00075b350e"	-0.2199
"00075b16a9"	"00075b3651"	-0.0486
"00075b16a9"	"00075b38ca"	-0.0688
"00075b16a9"	"00075b39cc"	0.1163
"00075b16a9"	"00075b3e1e"	0.2897
"00075b16a9"	"00075b3e57"	0.2145
"00075b16a9"	"00075b4079"	-0.1605
"00075b16a9"	"00075b4150"	0.0598
"00075b16a9"	"00075b4194"	-0.1283
"00075b16a9"	"00075b42d5"	0.02
"00075b16a9"	"00075b4424"	-0.0404
"00075b16a9"	"00075b4470"	-0.0267
"00075b16a9"	"00075b47ed"	0.0795
"00075b16a9"	"00075b4850"	-0.0864
"00075b16a9"	"00075b4ca0"	-0.2695
"00075b16a9"	"00075b4d7f"	0.2629
"00075b16a9"	"00075b520f"	-0.0723
"00075b16a9"	"00075b525f"	-0.1846
"00075b16a9"	"00075b58f8"	0.0953
"00075b16a9"	"00075b5bcc"	-0.0702
"00075b16a9"	"00075b5bfa"	-0.2605
"00075b16a9"	"00075b6339"	0.2264
"00075b16a9"	"00075b6658"	-0.1971
"00075b16a9"	"00075b679a"	0.2233
"00075b16a9"	"00075b6cb7"	-0.0976
"00075b16a9"	"00075b6df8"	0.2082
"00075b16a9"	"00075b6ff6"	-0.026
"00075b16a9"	"00075b70ee"	0.0653
"00075b16a9"	"00075b7157"	0.2448
"00075b16a9"	"00075b7225"	0.0811
"00075b16a9"	"00075b7c89"	0.0409
"00075b16a9"	"00075b9048"	-0.0041
"00075b16a9"	"00075d0801"	0.0911
"00075b16a9"	"00075d1820"	0.313
"00075b16a9"	"00075d1f3d"	-0.2253
"00075b16a9"	"00075d2329"	0.0047
"00075b16a9"	"00075d2b9b"	-0.3801
"00075b16a9"	"00075d3941"	-0.2178
"00075b16a9"	"00075d3e96"	0.1712

"00075b16a9"	"00075d4864"	−0.1039
"00075b16a9"	"00075d5961"	0.1322
"00075b16a9"	"00075d5a63"	−0.1663
"00075b16a9"	"00075d6150"	−0.0522
"00075b16a9"	"00075d67d0"	−0.284
"00075b16a9"	"00075d67e2"	0.0465
"00075b16a9"	"00075d73fc"	0.3468
"00075b16a9"	"00075d7729"	−0.3575
"00075b16a9"	"00075d778c"	−0.1347
"00075b16a9"	"00075d7b9e"	0.0013
"00075b16a9"	"00075d7c8f"	0.1662
"00075b16a9"	"00075d804d"	−0.1457
"00075b16a9"	"00075d819f"	−0.1307
"00075b16a9"	"00075d8601"	0.2028
"00075b16a9"	"00075d8c6a"	0.0808
"00075b16a9"	"00075dfedc"	0.3189
"00075b16a9"	"00075e05f2"	−0.004
"00075b16a9"	"00075e0837"	−0.0394
"00075b16a9"	"00075e092e"	−0.1962
"00075b16a9"	"00075e0965"	−0.1238
"00075b16a9"	"00075e0bc8"	−0.2169
"00075b16a9"	"00075e0fbb"	−0.196
"00075b1a28"	"00075b1a97"	0.3752
"00075b1a28"	"00075b1c7b"	0.2607
"00075b1a28"	"00075b1d24"	0.1694
"00075b1a28"	"00075b202b"	−0.1343
"00075b1a28"	"00075b22cb"	0.5655
"00075b1a28"	"00075b22da"	0.0964
"00075b1a28"	"00075b2556"	0.1297
"00075b1a28"	"00075b25de"	−0.0742
"00075b1a28"	"00075b260c"	−0.2783
"00075b1a28"	"00075b26f1"	0.0015
"00075b1a28"	"00075b2920"	−0.0914
"00075b1a28"	"00075b2a64"	−0.4
"00075b1a28"	"00075b2a9d"	−0.1461
"00075b1a28"	"00075b2b37"	−0.0317
"00075b1a28"	"00075b2cdd"	0.2812
"00075b1a28"	"00075b3038"	−0.1704
"00075b1a28"	"00075b30fe"	0.1073
"00075b1a28"	"00075b3362"	−0.2743
"00075b1a28"	"00075b350a"	−0.1796
"00075b1a28"	"00075b350e"	0.0727
"00075b1a28"	"00075b3651"	−0.0589
"00075b1a28"	"00075b38ca"	−0.036
"00075b1a28"	"00075b39cc"	−0.2856
"00075b1a28"	"00075b3e1e"	0.222
"00075b1a28"	"00075b3e57"	0.1747
"00075b1a28"	"00075b4079"	−0.2022
"00075b1a28"	"00075b4150"	−0.0893
"00075b1a28"	"00075b4194"	−0.2857

"00075b1a28"	"00075b42d5"	0.1182
"00075b1a28"	"00075b4424"	0.0864
"00075b1a28"	"00075b4470"	0.0634
"00075b1a28"	"00075b47ed"	−0.2412
"00075b1a28"	"00075b4850"	−0.1257
"00075b1a28"	"00075b4ca0"	−0.258
"00075b1a28"	"00075b4d7f"	0.0348
"00075b1a28"	"00075b520f"	0.1739
"00075b1a28"	"00075b525f"	−0.1705
"00075b1a28"	"00075b58f8"	0.0215
"00075b1a28"	"00075b5bcc"	−0.0097
"00075b1a28"	"00075b5bfa"	−0.531
"00075b1a28"	"00075b6339"	−0.1747
"00075b1a28"	"00075b6658"	−0.1124
"00075b1a28"	"00075b679a"	0.3123
"00075b1a28"	"00075b6cb7"	−0.2369
"00075b1a28"	"00075b6df8"	−0.07
"00075b1a28"	"00075b6ff6"	−0.2777
"00075b1a28"	"00075b70ee"	−0.1989
"00075b1a28"	"00075b7157"	0.0325
"00075b1a28"	"00075b7225"	0.2192
"00075b1a28"	"00075b7c89"	−0.1504
"00075b1a28"	"00075b9048"	−0.1111
"00075b1a28"	"00075d0801"	−0.1325
"00075b1a28"	"00075d1820"	0.1041
"00075b1a28"	"00075d1f3d"	−0.2494
"00075b1a28"	"00075d2329"	−0.1436
"00075b1a28"	"00075d2b9b"	−0.4198
"00075b1a28"	"00075d3941"	0.1607
"00075b1a28"	"00075d3e96"	0.1505
"00075b1a28"	"00075d4864"	0.2793
"00075b1a28"	"00075d5961"	0.0968
"00075b1a28"	"00075d5a63"	−0.1217
"00075b1a28"	"00075d6150"	0.0642
"00075b1a28"	"00075d67d0"	−0.55
"00075b1a28"	"00075d67e2"	−0.0647
"00075b1a28"	"00075d73fc"	0.4048
"00075b1a28"	"00075d7729"	−0.3347
"00075b1a28"	"00075d778c"	−0.4094
"00075b1a28"	"00075d7b9e"	−0.0238
"00075b1a28"	"00075d7c8f"	−0.0146
"00075b1a28"	"00075d804d"	−0.2546
"00075b1a28"	"00075d819f"	−0.1856
"00075b1a28"	"00075d8601"	0.1944
"00075b1a28"	"00075d8c6a"	−0.1325
"00075b1a28"	"00075dfedc"	−0.0321
"00075b1a28"	"00075e05f2"	−0.0478
"00075b1a28"	"00075e0837"	−0.2462
"00075b1a28"	"00075e092e"	0.1897
"00075b1a28"	"00075e0965"	−0.1559

"00075b1a28"	"00075e0bc8"	-0.2352
"00075b1a28"	"00075e0fbb"	-0.1809
"00075b1a97"	"00075b1c7b"	0.0737
"00075b1a97"	"00075b1d24"	0.4027
"00075b1a97"	"00075b202b"	-0.0096
"00075b1a97"	"00075b22cb"	0.485
"00075b1a97"	"00075b22da"	0.0878
"00075b1a97"	"00075b2556"	3e-04
"00075b1a97"	"00075b25de"	-0.039
"00075b1a97"	"00075b260c"	-0.1112
"00075b1a97"	"00075b26f1"	0.1317
"00075b1a97"	"00075b2920"	-0.2317
"00075b1a97"	"00075b2a64"	-0.2737
"00075b1a97"	"00075b2a9d"	-0.1569
"00075b1a97"	"00075b2b37"	-0.1266
"00075b1a97"	"00075b2cdd"	0.4361
"00075b1a97"	"00075b3038"	0.0433
"00075b1a97"	"00075b30fe"	0.0581
"00075b1a97"	"00075b3362"	-0.0367
"00075b1a97"	"00075b350a"	0.0082
"00075b1a97"	"00075b350e"	0.1303
"00075b1a97"	"00075b3651"	0.2797
"00075b1a97"	"00075b38ca"	0.0995
"00075b1a97"	"00075b39cc"	-0.0551
"00075b1a97"	"00075b3e1e"	0.2692
"00075b1a97"	"00075b3e57"	0.2464
"00075b1a97"	"00075b4079"	-0.1418
"00075b1a97"	"00075b4150"	-0.2528
"00075b1a97"	"00075b4194"	0.0509
"00075b1a97"	"00075b42d5"	0.3076
"00075b1a97"	"00075b4424"	0.0693
"00075b1a97"	"00075b4470"	0.0423
"00075b1a97"	"00075b47ed"	0.0113
"00075b1a97"	"00075b4850"	0.0451
"00075b1a97"	"00075b4ca0"	-0.1349
"00075b1a97"	"00075b4d7f"	0.2276
"00075b1a97"	"00075b520f"	0.0481
"00075b1a97"	"00075b525f"	0.2127
"00075b1a97"	"00075b58f8"	-0.3873
"00075b1a97"	"00075b5bcc"	0.2723
"00075b1a97"	"00075b5bfa"	-0.2015
"00075b1a97"	"00075b6339"	0.0323
"00075b1a97"	"00075b6658"	0.1178
"00075b1a97"	"00075b679a"	0.3491
"00075b1a97"	"00075b6cb7"	-0.1389
"00075b1a97"	"00075b6df8"	0.1401
"00075b1a97"	"00075b6ff6"	0.1883
"00075b1a97"	"00075b70ee"	-0.2218
"00075b1a97"	"00075b7157"	0.168
"00075b1a97"	"00075b7225"	0.3212

"00075b1a97"	"00075b7c89"	−0.0503
"00075b1a97"	"00075b9048"	0.1424
"00075b1a97"	"00075d0801"	−0.3213
"00075b1a97"	"00075d1820"	0.2603
"00075b1a97"	"00075d1f3d"	−0.0108
"00075b1a97"	"00075d2329"	0.0208
"00075b1a97"	"00075d2b9b"	−0.2769
"00075b1a97"	"00075d3941"	0.0447
"00075b1a97"	"00075d3e96"	0.1086
"00075b1a97"	"00075d4864"	−0.0203
"00075b1a97"	"00075d5961"	0.1645
"00075b1a97"	"00075d5a63"	−0.0415
"00075b1a97"	"00075d6150"	0.3517
"00075b1a97"	"00075d67d0"	−0.2529
"00075b1a97"	"00075d67e2"	0.1592
"00075b1a97"	"00075d73fc"	0.3649
"00075b1a97"	"00075d7729"	0.0497
"00075b1a97"	"00075d778c"	−0.123
"00075b1a97"	"00075d7b9e"	0.2047
"00075b1a97"	"00075d7c8f"	0.2238
"00075b1a97"	"00075d804d"	0.0223
"00075b1a97"	"00075d819f"	−0.0577
"00075b1a97"	"00075d8601"	0.3519
"00075b1a97"	"00075d8c6a"	−0.0178
"00075b1a97"	"00075dfedc"	0.1616
"00075b1a97"	"00075e05f2"	−0.1187
"00075b1a97"	"00075e0837"	−0.1443
"00075b1a97"	"00075e092e"	0.1456
"00075b1a97"	"00075e0965"	−0.4705
"00075b1a97"	"00075e0bc8"	−0.044
"00075b1a97"	"00075e0fbb"	−0.0968
"00075b1c7b"	"00075b1d24"	−0.1582
"00075b1c7b"	"00075b202b"	−0.3745
"00075b1c7b"	"00075b22cb"	0.4852
"00075b1c7b"	"00075b22da"	−0.3505
"00075b1c7b"	"00075b2556"	−0.1767
"00075b1c7b"	"00075b25de"	−0.2815
"00075b1c7b"	"00075b260c"	−0.434
"00075b1c7b"	"00075b26f1"	−0.1501
"00075b1c7b"	"00075b2920"	−0.0441
"00075b1c7b"	"00075b2a64"	−0.5643
"00075b1c7b"	"00075b2a9d"	−0.4591
"00075b1c7b"	"00075b2b37"	−0.0834
"00075b1c7b"	"00075b2cdd"	0.2061
"00075b1c7b"	"00075b3038"	−0.3515
"00075b1c7b"	"00075b30fe"	−0.3642
"00075b1c7b"	"00075b3362"	−0.2706
"00075b1c7b"	"00075b350a"	−0.5777
"00075b1c7b"	"00075b350e"	−0.3645
"00075b1c7b"	"00075b3651"	−0.3018

"00075b1c7b"	"00075b38ca"	−0.3428
"00075b1c7b"	"00075b39cc"	−0.3153
"00075b1c7b"	"00075b3e1e"	−0.0059
"00075b1c7b"	"00075b3e57"	0.2553
"00075b1c7b"	"00075b4079"	−0.4766
"00075b1c7b"	"00075b4150"	−0.1187
"00075b1c7b"	"00075b4194"	−0.4638
"00075b1c7b"	"00075b42d5"	−0.2262
"00075b1c7b"	"00075b4424"	0.0917
"00075b1c7b"	"00075b4470"	−0.2069
"00075b1c7b"	"00075b47ed"	−0.447
"00075b1c7b"	"00075b4850"	−0.3207
"00075b1c7b"	"00075b4ca0"	−0.6441
"00075b1c7b"	"00075b4d7f"	−0.1113
"00075b1c7b"	"00075b520f"	−0.2935
"00075b1c7b"	"00075b525f"	−0.3429
"00075b1c7b"	"00075b58f8"	0.0086
"00075b1c7b"	"00075b5bcc"	−0.4362
"00075b1c7b"	"00075b5bfa"	−0.5229
"00075b1c7b"	"00075b6339"	−0.1323
"00075b1c7b"	"00075b6658"	−0.3112
"00075b1c7b"	"00075b679a"	−0.0358
"00075b1c7b"	"00075b6cb7"	−0.1032
"00075b1c7b"	"00075b6df8"	−0.1221
"00075b1c7b"	"00075b6ff6"	−0.3969
"00075b1c7b"	"00075b70ee"	−0.0962
"00075b1c7b"	"00075b7157"	−0.1116
"00075b1c7b"	"00075b7225"	−0.1701
"00075b1c7b"	"00075b7c89"	−0.4636
"00075b1c7b"	"00075b9048"	−0.2448
"00075b1c7b"	"00075d0801"	−0.3576
"00075b1c7b"	"00075d1820"	0.0104
"00075b1c7b"	"00075d1f3d"	−0.4148
"00075b1c7b"	"00075d2329"	−0.3883
"00075b1c7b"	"00075d2b9b"	−0.7242
"00075b1c7b"	"00075d3941"	−0.3943
"00075b1c7b"	"00075d3e96"	0.1096
"00075b1c7b"	"00075d4864"	−0.3176
"00075b1c7b"	"00075d5961"	0.0091
"00075b1c7b"	"00075d5a63"	−0.3128
"00075b1c7b"	"00075d6150"	−0.1666
"00075b1c7b"	"00075d67d0"	−0.4119
"00075b1c7b"	"00075d67e2"	−0.0462
"00075b1c7b"	"00075d73fc"	0.1852
"00075b1c7b"	"00075d7729"	−0.5912
"00075b1c7b"	"00075d778c"	−0.2702
"00075b1c7b"	"00075d7b9e"	−0.101
"00075b1c7b"	"00075d7c8f"	−0.1606
"00075b1c7b"	"00075d804d"	−0.3136
"00075b1c7b"	"00075d819f"	−0.3417

"00075b1c7b"	"00075d8601"	0.0654
"00075b1c7b"	"00075d8c6a"	−0.2547
"00075b1c7b"	"00075dfedc"	−0.1315
"00075b1c7b"	"00075e05f2"	0.0203
"00075b1c7b"	"00075e0837"	−0.27
"00075b1c7b"	"00075e092e"	−0.23
"00075b1c7b"	"00075e0965"	−0.1459
"00075b1c7b"	"00075e0bc8"	−0.367
"00075b1c7b"	"00075e0fbb"	−0.3704
"00075b1d24"	"00075b202b"	0.0283
"00075b1d24"	"00075b22cb"	0.0969
"00075b1d24"	"00075b22da"	0.0819
"00075b1d24"	"00075b2556"	0.0987
"00075b1d24"	"00075b25de"	0.1827
"00075b1d24"	"00075b260c"	−0.157
"00075b1d24"	"00075b26f1"	−0.0423
"00075b1d24"	"00075b2920"	−0.0432
"00075b1d24"	"00075b2a64"	−0.1445
"00075b1d24"	"00075b2a9d"	−0.0835
"00075b1d24"	"00075b2b37"	−0.12
"00075b1d24"	"00075b2cdd"	0.0259
"00075b1d24"	"00075b3038"	−0.0641
"00075b1d24"	"00075b30fe"	0.0827
"00075b1d24"	"00075b3362"	0.401
"00075b1d24"	"00075b350a"	0.1785
"00075b1d24"	"00075b350e"	−0.1092
"00075b1d24"	"00075b3651"	0.3387
"00075b1d24"	"00075b38ca"	−0.1621
"00075b1d24"	"00075b39cc"	0.0283
"00075b1d24"	"00075b3e1e"	0.0065
"00075b1d24"	"00075b3e57"	−0.0039
"00075b1d24"	"00075b4079"	0.0832
"00075b1d24"	"00075b4150"	−0.0706
"00075b1d24"	"00075b4194"	0.0425
"00075b1d24"	"00075b42d5"	0.0982
"00075b1d24"	"00075b4424"	−0.1006
"00075b1d24"	"00075b4470"	0.1177
"00075b1d24"	"00075b47ed"	0.0379
"00075b1d24"	"00075b4850"	0.1205
"00075b1d24"	"00075b4ca0"	−0.113
"00075b1d24"	"00075b4d7f"	0.2761
"00075b1d24"	"00075b520f"	0.0011
"00075b1d24"	"00075b525f"	0.3527
"00075b1d24"	"00075b58f8"	−0.2477
"00075b1d24"	"00075b5bcc"	0.4315
"00075b1d24"	"00075b5bfa"	−0.175
"00075b1d24"	"00075b6339"	0.2654
"00075b1d24"	"00075b6658"	0.2635
"00075b1d24"	"00075b679a"	0.3531
"00075b1d24"	"00075b6cb7"	−0.0096

"00075b1d24"	"00075b6df8"	0.0789
"00075b1d24"	"00075b6ff6"	0.2742
"00075b1d24"	"00075b70ee"	-0.1775
"00075b1d24"	"00075b7157"	0.34
"00075b1d24"	"00075b7225"	0.3257
"00075b1d24"	"00075b7c89"	-0.0627
"00075b1d24"	"00075b9048"	-0.0043
"00075b1d24"	"00075d0801"	0.0472
"00075b1d24"	"00075d1820"	0.1392
"00075b1d24"	"00075d1f3d"	0.0244
"00075b1d24"	"00075d2329"	-0.0452
"00075b1d24"	"00075d2b9b"	-0.2216
"00075b1d24"	"00075d3941"	-0.28
"00075b1d24"	"00075d3e96"	0.1023
"00075b1d24"	"00075d4864"	0.052
"00075b1d24"	"00075d5961"	-0.027
"00075b1d24"	"00075d5a63"	0.1687
"00075b1d24"	"00075d6150"	0.0335
"00075b1d24"	"00075d67d0"	0.031
"00075b1d24"	"00075d67e2"	-0.0889
"00075b1d24"	"00075d73fc"	0.1964
"00075b1d24"	"00075d7729"	-0.0671
"00075b1d24"	"00075d778c"	0.393
"00075b1d24"	"00075d7b9e"	0.4002
"00075b1d24"	"00075d7c8f"	0.352
"00075b1d24"	"00075d804d"	-0.0516
"00075b1d24"	"00075d819f"	-0.0084
"00075b1d24"	"00075d8601"	-0.1795
"00075b1d24"	"00075d8c6a"	-0.1042
"00075b1d24"	"00075dfedc"	0.1737
"00075b1d24"	"00075e05f2"	-0.1703
"00075b1d24"	"00075e0837"	-0.0558
"00075b1d24"	"00075e092e"	0.1889
"00075b1d24"	"00075e0965"	-0.1869
"00075b1d24"	"00075e0bc8"	0.062
"00075b1d24"	"00075e0fbb"	0.0724
"00075b202b"	"00075b22cb"	-0.0901
"00075b202b"	"00075b22da"	-0.1281
"00075b202b"	"00075b2556"	-0.1319
"00075b202b"	"00075b25de"	0.1655
"00075b202b"	"00075b260c"	0.1961
"00075b202b"	"00075b26f1"	-0.0586
"00075b202b"	"00075b2920"	0.3775
"00075b202b"	"00075b2a64"	0.2886
"00075b202b"	"00075b2a9d"	0.5137
"00075b202b"	"00075b2b37"	-0.0728
"00075b202b"	"00075b2cdd"	0.2167
"00075b202b"	"00075b3038"	0.2142
"00075b202b"	"00075b30fe"	-0.1104
"00075b202b"	"00075b3362"	-0.2836

"00075b202b"	"00075b350a"	0.0972
"00075b202b"	"00075b350e"	0.0743
"00075b202b"	"00075b3651"	−0.0956
"00075b202b"	"00075b38ca"	−0.0664
"00075b202b"	"00075b39cc"	−0.0927
"00075b202b"	"00075b3e1e"	0.2102
"00075b202b"	"00075b3e57"	0.015
"00075b202b"	"00075b4079"	−0.1047
"00075b202b"	"00075b4150"	0.3485
"00075b202b"	"00075b4194"	0.2131
"00075b202b"	"00075b42d5"	0.048
"00075b202b"	"00075b4424"	0.08
"00075b202b"	"00075b4470"	0.3389
"00075b202b"	"00075b47ed"	−0.0779
"00075b202b"	"00075b4850"	0.3361
"00075b202b"	"00075b4ca0"	0.1379
"00075b202b"	"00075b4d7f"	0.0527
"00075b202b"	"00075b520f"	0.0077
"00075b202b"	"00075b525f"	−0.154
"00075b202b"	"00075b58f8"	0.127
"00075b202b"	"00075b5bcc"	0.1135
"00075b202b"	"00075b5bfa"	−0.0263
"00075b202b"	"00075b6339"	0.0479
"00075b202b"	"00075b6658"	−0.1737
"00075b202b"	"00075b679a"	−0.0328
"00075b202b"	"00075b6cb7"	−0.02
"00075b202b"	"00075b6df8"	−0.2216
"00075b202b"	"00075b6ff6"	0.0627
"00075b202b"	"00075b70ee"	−0.0075
"00075b202b"	"00075b7157"	−0.0427
"00075b202b"	"00075b7225"	−0.0847
"00075b202b"	"00075b7c89"	0.0269
"00075b202b"	"00075b9048"	0.2326
"00075b202b"	"00075d0801"	0.1189
"00075b202b"	"00075d1820"	−0.1884
"00075b202b"	"00075d1f3d"	0.4532
"00075b202b"	"00075d2329"	0.0814
"00075b202b"	"00075d2b9b"	0.3198
"00075b202b"	"00075d3941"	0.1709
"00075b202b"	"00075d3e96"	0.0857
"00075b202b"	"00075d4864"	−0.0947
"00075b202b"	"00075d5961"	0.043
"00075b202b"	"00075d5a63"	0.3584
"00075b202b"	"00075d6150"	−0.1665
"00075b202b"	"00075d67d0"	−0.0932
"00075b202b"	"00075d67e2"	−0.1426
"00075b202b"	"00075d73fc"	−0.1278
"00075b202b"	"00075d7729"	−0.0277
"00075b202b"	"00075d778c"	−0.3786
"00075b202b"	"00075d7b9e"	−0.05

"00075b202b"	"00075d7c8f"	0.0251
"00075b202b"	"00075d804d"	0.2614
"00075b202b"	"00075d819f"	0.1983
"00075b202b"	"00075d8601"	−0.0151
"00075b202b"	"00075d8c6a"	−0.1872
"00075b202b"	"00075dfedc"	0.0044
"00075b202b"	"00075e05f2"	0.0451
"00075b202b"	"00075e0837"	0.5458
"00075b202b"	"00075e092e"	−0.0697
"00075b202b"	"00075e0965"	0.0501
"00075b202b"	"00075e0bc8"	0.568
"00075b202b"	"00075e0fbb"	−0.1817
"00075b22cb"	"00075b22da"	0.1568
"00075b22cb"	"00075b2556"	0.1256
"00075b22cb"	"00075b25de"	−0.0864
"00075b22cb"	"00075b260c"	−0.2705
"00075b22cb"	"00075b26f1"	0.0413
"00075b22cb"	"00075b2920"	−0.1927
"00075b22cb"	"00075b2a64"	−0.4438
"00075b22cb"	"00075b2a9d"	−0.2446
"00075b22cb"	"00075b2b37"	−0.2243
"00075b22cb"	"00075b2cdd"	0.4925
"00075b22cb"	"00075b3038"	−0.0582
"00075b22cb"	"00075b30fe"	0.095
"00075b22cb"	"00075b3362"	−0.1818
"00075b22cb"	"00075b350a"	−0.2317
"00075b22cb"	"00075b350e"	−0.0309
"00075b22cb"	"00075b3651"	0.0658
"00075b22cb"	"00075b38ca"	−0.1328
"00075b22cb"	"00075b39cc"	−0.0106
"00075b22cb"	"00075b3e1e"	0.2349
"00075b22cb"	"00075b3e57"	0.3878
"00075b22cb"	"00075b4079"	−0.2624
"00075b22cb"	"00075b4150"	−0.1986
"00075b22cb"	"00075b4194"	−0.1415
"00075b22cb"	"00075b42d5"	0.1216
"00075b22cb"	"00075b4424"	0.1376
"00075b22cb"	"00075b4470"	0.0603
"00075b22cb"	"00075b47ed"	−0.0091
"00075b22cb"	"00075b4850"	−0.0663
"00075b22cb"	"00075b4ca0"	−0.2515
"00075b22cb"	"00075b4d7f"	0.192
"00075b22cb"	"00075b520f"	0.0055
"00075b22cb"	"00075b525f"	−0.089
"00075b22cb"	"00075b58f8"	−0.1551
"00075b22cb"	"00075b5bcc"	−0.07
"00075b22cb"	"00075b5bfa"	−0.3397
"00075b22cb"	"00075b6339"	−0.0816
"00075b22cb"	"00075b6658"	0.0542
"00075b22cb"	"00075b679a"	0.2565

"00075b22cb"	"00075b6cb7"	-0.1211
"00075b22cb"	"00075b6df8"	0.1236
"00075b22cb"	"00075b6ff6"	-0.117
"00075b22cb"	"00075b70ee"	-0.2622
"00075b22cb"	"00075b7157"	0.1161
"00075b22cb"	"00075b7225"	0.2031
"00075b22cb"	"00075b7c89"	-0.0839
"00075b22cb"	"00075b9048"	-0.0409
"00075b22cb"	"00075d0801"	-0.1062
"00075b22cb"	"00075d1820"	0.2656
"00075b22cb"	"00075d1f3d"	-0.1819
"00075b22cb"	"00075d2329"	-0.0559
"00075b22cb"	"00075d2b9b"	-0.4123
"00075b22cb"	"00075d3941"	0.0852
"00075b22cb"	"00075d3e96"	0.1288
"00075b22cb"	"00075d4864"	0.0258
"00075b22cb"	"00075d5961"	0.0886
"00075b22cb"	"00075d5a63"	-0.0372
"00075b22cb"	"00075d6150"	0.0356
"00075b22cb"	"00075d67d0"	-0.3793
"00075b22cb"	"00075d67e2"	0.0997
"00075b22cb"	"00075d73fc"	0.4963
"00075b22cb"	"00075d7729"	-0.4006
"00075b22cb"	"00075d778c"	-0.2343
"00075b22cb"	"00075d7b9e"	0.0821
"00075b22cb"	"00075d7c8f"	0.1082
"00075b22cb"	"00075d804d"	-0.1385
"00075b22cb"	"00075d819f"	-0.1576
"00075b22cb"	"00075d8601"	0.1943
"00075b22cb"	"00075d8c6a"	-0.119
"00075b22cb"	"00075dfedc"	0.2279
"00075b22cb"	"00075e05f2"	0.1199
"00075b22cb"	"00075e0837"	-0.0975
"00075b22cb"	"00075e092e"	0.1461
"00075b22cb"	"00075e0965"	-0.3554
"00075b22cb"	"00075e0bc8"	-0.1238
"00075b22cb"	"00075e0fbb"	-0.1274
"00075b22da"	"00075b2556"	0.6705
"00075b22da"	"00075b25de"	-0.2306
"00075b22da"	"00075b260c"	0.1002
"00075b22da"	"00075b26f1"	-0.0063
"00075b22da"	"00075b2920"	-0.2141
"00075b22da"	"00075b2a64"	-0.3709
"00075b22da"	"00075b2a9d"	0.0508
"00075b22da"	"00075b2b37"	-0.1444
"00075b22da"	"00075b2cdd"	-0.0567
"00075b22da"	"00075b3038"	-0.0432
"00075b22da"	"00075b30fe"	0.6327
"00075b22da"	"00075b3362"	-0.006
"00075b22da"	"00075b350a"	0.1011

"00075b22da"	"00075b350e"	-0.0718
"00075b22da"	"00075b3651"	-0.2192
"00075b22da"	"00075b38ca"	-0.048
"00075b22da"	"00075b39cc"	-0.2212
"00075b22da"	"00075b3e1e"	0.1327
"00075b22da"	"00075b3e57"	-0.0156
"00075b22da"	"00075b4079"	-0.1442
"00075b22da"	"00075b4150"	-0.3053
"00075b22da"	"00075b4194"	-0.1358
"00075b22da"	"00075b42d5"	0.1742
"00075b22da"	"00075b4424"	-0.1818
"00075b22da"	"00075b4470"	0.1293
"00075b22da"	"00075b47ed"	-0.0537
"00075b22da"	"00075b4850"	0.1109
"00075b22da"	"00075b4ca0"	0.0357
"00075b22da"	"00075b4d7f"	0.1617
"00075b22da"	"00075b520f"	-0.2321
"00075b22da"	"00075b525f"	0.1499
"00075b22da"	"00075b58f8"	-0.0213
"00075b22da"	"00075b5bcc"	0.095
"00075b22da"	"00075b5bfa"	-0.2768
"00075b22da"	"00075b6339"	0.0577
"00075b22da"	"00075b6658"	-0.1824
"00075b22da"	"00075b679a"	0.0519
"00075b22da"	"00075b6cb7"	0.0422
"00075b22da"	"00075b6df8"	0.0938
"00075b22da"	"00075b6ff6"	0.2663
"00075b22da"	"00075b70ee"	-0.1962
"00075b22da"	"00075b7157"	0.064
"00075b22da"	"00075b7225"	0.1416
"00075b22da"	"00075b7c89"	-0.0288
"00075b22da"	"00075b9048"	-0.1029
"00075b22da"	"00075d0801"	-0.1714
"00075b22da"	"00075d1820"	-0.1351
"00075b22da"	"00075d1f3d"	0.0789
"00075b22da"	"00075d2329"	0.0045
"00075b22da"	"00075d2b9b"	-0.099
"00075b22da"	"00075d3941"	-0.3003
"00075b22da"	"00075d3e96"	-0.0256
"00075b22da"	"00075d4864"	0.0981
"00075b22da"	"00075d5961"	-0.0515
"00075b22da"	"00075d5a63"	0.0883
"00075b22da"	"00075d6150"	-0.1728
"00075b22da"	"00075d67d0"	-0.2676
"00075b22da"	"00075d67e2"	-0.0056
"00075b22da"	"00075d73fc"	-8e-04
"00075b22da"	"00075d7729"	0.0038
"00075b22da"	"00075d778c"	0.0843
"00075b22da"	"00075d7b9e"	0.0064
"00075b22da"	"00075d7c8f"	0.3022

"00075b22da"	"00075d804d"	0.1214
"00075b22da"	"00075d819f"	0.0769
"00075b22da"	"00075d8601"	-0.3221
"00075b22da"	"00075d8c6a"	0.0397
"00075b22da"	"00075dfedc"	-0.1097
"00075b22da"	"00075e05f2"	-0.1198
"00075b22da"	"00075e0837"	0.1189
"00075b22da"	"00075e092e"	0.6202
"00075b22da"	"00075e0965"	-0.1518
"00075b22da"	"00075e0bc8"	0.0236
"00075b22da"	"00075e0fbb"	0.607
"00075b2556"	"00075b25de"	-0.1346
"00075b2556"	"00075b260c"	0.1
"00075b2556"	"00075b26f1"	-0.1061
"00075b2556"	"00075b2920"	-0.0644
"00075b2556"	"00075b2a64"	-0.243
"00075b2556"	"00075b2a9d"	-0.0894
"00075b2556"	"00075b2b37"	-0.1112
"00075b2556"	"00075b2cdd"	-0.0686
"00075b2556"	"00075b3038"	-0.0624
"00075b2556"	"00075b30fe"	0.5964
"00075b2556"	"00075b3362"	0.0815
"00075b2556"	"00075b350a"	0.2194
"00075b2556"	"00075b350e"	-0.2222
"00075b2556"	"00075b3651"	-0.1427
"00075b2556"	"00075b38ca"	-0.0764
"00075b2556"	"00075b39cc"	-0.1927
"00075b2556"	"00075b3e1e"	-0.0019
"00075b2556"	"00075b3e57"	0.0119
"00075b2556"	"00075b4079"	-0.3043
"00075b2556"	"00075b4150"	-0.1788
"00075b2556"	"00075b4194"	-0.3324
"00075b2556"	"00075b42d5"	-0.0129
"00075b2556"	"00075b4424"	-0.0578
"00075b2556"	"00075b4470"	0.1403
"00075b2556"	"00075b47ed"	-0.1788
"00075b2556"	"00075b4850"	0.0658
"00075b2556"	"00075b4ca0"	-0.1224
"00075b2556"	"00075b4d7f"	0.0653
"00075b2556"	"00075b520f"	-0.2907
"00075b2556"	"00075b525f"	0.0949
"00075b2556"	"00075b58f8"	0.0815
"00075b2556"	"00075b5bcc"	-0.0351
"00075b2556"	"00075b5bfa"	-0.2589
"00075b2556"	"00075b6339"	-0.0481
"00075b2556"	"00075b6658"	-0.1355
"00075b2556"	"00075b679a"	0.026
"00075b2556"	"00075b6cb7"	-0.0111
"00075b2556"	"00075b6df8"	0.0285
"00075b2556"	"00075b6ff6"	0.1243

"00075b2556"	"00075b70ee"	-0.0755
"00075b2556"	"00075b7157"	0.0876
"00075b2556"	"00075b7225"	-0.1263
"00075b2556"	"00075b7c89"	-0.0716
"00075b2556"	"00075b9048"	-0.0553
"00075b2556"	"00075d0801"	-0.0961
"00075b2556"	"00075d1820"	-0.1963
"00075b2556"	"00075d1f3d"	0.0014
"00075b2556"	"00075d2329"	-0.054
"00075b2556"	"00075d2b9b"	-0.0941
"00075b2556"	"00075d3941"	-0.2086
"00075b2556"	"00075d3e96"	0.0196
"00075b2556"	"00075d4864"	0.0727
"00075b2556"	"00075d5961"	0.0272
"00075b2556"	"00075d5a63"	-0.0029
"00075b2556"	"00075d6150"	-0.2082
"00075b2556"	"00075d67d0"	-0.3238
"00075b2556"	"00075d67e2"	-0.0899
"00075b2556"	"00075d73fc"	-0.0388
"00075b2556"	"00075d7729"	-0.0764
"00075b2556"	"00075d778c"	0.0444
"00075b2556"	"00075d7b9e"	0.1348
"00075b2556"	"00075d7c8f"	0.1177
"00075b2556"	"00075d804d"	-0.0963
"00075b2556"	"00075d819f"	0.1493
"00075b2556"	"00075d8601"	-0.2192
"00075b2556"	"00075d8c6a"	0.024
"00075b2556"	"00075dfedc"	-0.112
"00075b2556"	"00075e05f2"	-0.2108
"00075b2556"	"00075e0837"	0.0816
"00075b2556"	"00075e092e"	0.4272
"00075b2556"	"00075e0965"	-0.0214
"00075b2556"	"00075e0bc8"	-0.0609
"00075b2556"	"00075e0fbb"	0.5077
"00075b25de"	"00075b260c"	-0.0657
"00075b25de"	"00075b26f1"	-0.031
"00075b25de"	"00075b2920"	0.1907
"00075b25de"	"00075b2a64"	-0.0347
"00075b25de"	"00075b2a9d"	0.0425
"00075b25de"	"00075b2b37"	-0.1092
"00075b25de"	"00075b2cdd"	-0.05
"00075b25de"	"00075b3038"	0.5985
"00075b25de"	"00075b30fe"	-0.0148
"00075b25de"	"00075b3362"	-0.0655
"00075b25de"	"00075b350a"	-0.1845
"00075b25de"	"00075b350e"	0.0242
"00075b25de"	"00075b3651"	0.1291
"00075b25de"	"00075b38ca"	0.049
"00075b25de"	"00075b39cc"	0.1864
"00075b25de"	"00075b3e1e"	0.0295

"00075b25de"	"00075b3e57"	0.0398
"00075b25de"	"00075b4079"	0.0546
"00075b25de"	"00075b4150"	0.0567
"00075b25de"	"00075b4194"	0.1603
"00075b25de"	"00075b42d5"	0.1264
"00075b25de"	"00075b4424"	-0.0378
"00075b25de"	"00075b4470"	0.2288
"00075b25de"	"00075b47ed"	0.1906
"00075b25de"	"00075b4850"	0.1713
"00075b25de"	"00075b4ca0"	-0.0669
"00075b25de"	"00075b4d7f"	0.212
"00075b25de"	"00075b520f"	0.1858
"00075b25de"	"00075b525f"	-0.0888
"00075b25de"	"00075b58f8"	0.0639
"00075b25de"	"00075b5bcc"	0.2206
"00075b25de"	"00075b5bfa"	0.4729
"00075b25de"	"00075b6339"	0.0527
"00075b25de"	"00075b6658"	0.1544
"00075b25de"	"00075b679a"	0.2274
"00075b25de"	"00075b6cb7"	-0.0893
"00075b25de"	"00075b6df8"	0.1117
"00075b25de"	"00075b6ff6"	0.1147
"00075b25de"	"00075b70ee"	0.1089
"00075b25de"	"00075b7157"	0.2456
"00075b25de"	"00075b7225"	4e-04
"00075b25de"	"00075b7c89"	0.1193
"00075b25de"	"00075b9048"	0.102
"00075b25de"	"00075d0801"	0.4497
"00075b25de"	"00075d1820"	0.0488
"00075b25de"	"00075d1f3d"	-0.1245
"00075b25de"	"00075d2329"	0.2476
"00075b25de"	"00075d2b9b"	-0.0176
"00075b25de"	"00075d3941"	-0.0145
"00075b25de"	"00075d3e96"	0.2443
"00075b25de"	"00075d4864"	0.158
"00075b25de"	"00075d5961"	0.1282
"00075b25de"	"00075d5a63"	0.1337
"00075b25de"	"00075d6150"	-0.0089
"00075b25de"	"00075d67d0"	-0.1731
"00075b25de"	"00075d67e2"	-0.1891
"00075b25de"	"00075d73fc"	0.1068
"00075b25de"	"00075d7729"	-0.1899
"00075b25de"	"00075d778c"	-0.0559
"00075b25de"	"00075d7b9e"	0.1109
"00075b25de"	"00075d7c8f"	-0.1321
"00075b25de"	"00075d804d"	0.2007
"00075b25de"	"00075d819f"	0.0084
"00075b25de"	"00075d8601"	0.3198
"00075b25de"	"00075d8c6a"	0.144
"00075b25de"	"00075dfedc"	0.2476

"00075b25de"	"00075e05f2"	0.0574
"00075b25de"	"00075e0837"	0.1494
"00075b25de"	"00075e092e"	-0.224
"00075b25de"	"00075e0965"	-0.1053
"00075b25de"	"00075e0bc8"	0.3068
"00075b25de"	"00075e0fbb"	-0.2536
"00075b260c"	"00075b26f1"	-0.2001
"00075b260c"	"00075b2920"	0.2282
"00075b260c"	"00075b2a64"	0.1978
"00075b260c"	"00075b2a9d"	0.2251
"00075b260c"	"00075b2b37"	-0.3697
"00075b260c"	"00075b2cdd"	-0.1621
"00075b260c"	"00075b3038"	0.0793
"00075b260c"	"00075b30fe"	0.0256
"00075b260c"	"00075b3362"	-0.412
"00075b260c"	"00075b350a"	0.0644
"00075b260c"	"00075b350e"	0.04
"00075b260c"	"00075b3651"	-0.3033
"00075b260c"	"00075b38ca"	-0.2631
"00075b260c"	"00075b39cc"	-0.0964
"00075b260c"	"00075b3e1e"	-7e-04
"00075b260c"	"00075b3e57"	-0.152
"00075b260c"	"00075b4079"	-0.2219
"00075b260c"	"00075b4150"	0.1761
"00075b260c"	"00075b4194"	0.1195
"00075b260c"	"00075b42d5"	-0.0932
"00075b260c"	"00075b4424"	-0.2111
"00075b260c"	"00075b4470"	-0.0861
"00075b260c"	"00075b47ed"	-0.2143
"00075b260c"	"00075b4850"	0.0592
"00075b260c"	"00075b4ca0"	0.0799
"00075b260c"	"00075b4d7f"	-0.0743
"00075b260c"	"00075b520f"	-0.271
"00075b260c"	"00075b525f"	-0.0588
"00075b260c"	"00075b58f8"	-0.07
"00075b260c"	"00075b5bcc"	0.0576
"00075b260c"	"00075b5bfa"	0.013
"00075b260c"	"00075b6339"	0.2659
"00075b260c"	"00075b6658"	-0.5485
"00075b260c"	"00075b679a"	0.1773
"00075b260c"	"00075b6cb7"	-0.0828
"00075b260c"	"00075b6df8"	-0.1328
"00075b260c"	"00075b6ff6"	0.2452
"00075b260c"	"00075b70ee"	-0.0752
"00075b260c"	"00075b7157"	-0.1388
"00075b260c"	"00075b7225"	-0.0349
"00075b260c"	"00075b7c89"	0.0144
"00075b260c"	"00075b9048"	0.4137
"00075b260c"	"00075d0801"	-0.1215
"00075b260c"	"00075d1820"	-0.3254

"00075b260c"	"00075d1f3d"	0.3683
"00075b260c"	"00075d2329"	-0.1183
"00075b260c"	"00075d2b9b"	0.4397
"00075b260c"	"00075d3941"	-0.2753
"00075b260c"	"00075d3e96"	-0.0926
"00075b260c"	"00075d4864"	-0.3762
"00075b260c"	"00075d5961"	-0.1803
"00075b260c"	"00075d5a63"	0.1529
"00075b260c"	"00075d6150"	-0.123
"00075b260c"	"00075d67d0"	-3e-04
"00075b260c"	"00075d67e2"	-0.0658
"00075b260c"	"00075d73fc"	-0.2079
"00075b260c"	"00075d7729"	-0.0198
"00075b260c"	"00075d778c"	-0.2655
"00075b260c"	"00075d7b9e"	-0.1105
"00075b260c"	"00075d7c8f"	0.1553
"00075b260c"	"00075d804d"	0.1241
"00075b260c"	"00075d819f"	0.1389
"00075b260c"	"00075d8601"	-0.0892
"00075b260c"	"00075d8c6a"	-0.0422
"00075b260c"	"00075dfedc"	0.1104
"00075b260c"	"00075e05f2"	0.1621
"00075b260c"	"00075e0837"	0.3842
"00075b260c"	"00075e092e"	-0.1632
"00075b260c"	"00075e0965"	-0.0735
"00075b260c"	"00075e0bc8"	0.1556
"00075b260c"	"00075e0fbb"	-0.1516
"00075b26f1"	"00075b2920"	-0.2216
"00075b26f1"	"00075b2a64"	-0.2971
"00075b26f1"	"00075b2a9d"	-0.0952
"00075b26f1"	"00075b2b37"	-0.1426
"00075b26f1"	"00075b2cdd"	0.0216
"00075b26f1"	"00075b3038"	0.0995
"00075b26f1"	"00075b30fe"	-0.2203
"00075b26f1"	"00075b3362"	0.1352
"00075b26f1"	"00075b350a"	-0.2763
"00075b26f1"	"00075b350e"	-0.2331
"00075b26f1"	"00075b3651"	0.336
"00075b26f1"	"00075b38ca"	-0.1504
"00075b26f1"	"00075b39cc"	0.0796
"00075b26f1"	"00075b3e1e"	-0.0026
"00075b26f1"	"00075b3e57"	0.0964
"00075b26f1"	"00075b4079"	-0.2427
"00075b26f1"	"00075b4150"	-0.3343
"00075b26f1"	"00075b4194"	0.1207
"00075b26f1"	"00075b42d5"	-0.1693
"00075b26f1"	"00075b4424"	0.0588
"00075b26f1"	"00075b4470"	-0.0678
"00075b26f1"	"00075b47ed"	0.0339
"00075b26f1"	"00075b4850"	0.0159

"00075b26f1"	"00075b4ca0"	0.0491
"00075b26f1"	"00075b4d7f"	−0.0289
"00075b26f1"	"00075b520f"	0.0799
"00075b26f1"	"00075b525f"	0.1385
"00075b26f1"	"00075b58f8"	−0.4403
"00075b26f1"	"00075b5bcc"	0.1343
"00075b26f1"	"00075b5bfa"	0.0559
"00075b26f1"	"00075b6339"	0.0379
"00075b26f1"	"00075b6658"	0.2734
"00075b26f1"	"00075b679a"	−0.1302
"00075b26f1"	"00075b6cb7"	−0.0259
"00075b26f1"	"00075b6df8"	0.0384
"00075b26f1"	"00075b6ff6"	−0.2422
"00075b26f1"	"00075b70ee"	−0.3123
"00075b26f1"	"00075b7157"	−0.0165
"00075b26f1"	"00075b7225"	−0.1037
"00075b26f1"	"00075b7c89"	−0.2224
"00075b26f1"	"00075b9048"	−0.1382
"00075b26f1"	"00075d0801"	−0.2676
"00075b26f1"	"00075d1820"	0.0957
"00075b26f1"	"00075d1f3d"	−0.1544
"00075b26f1"	"00075d2329"	0.0819
"00075b26f1"	"00075d2b9b"	−0.1162
"00075b26f1"	"00075d3941"	0.3996
"00075b26f1"	"00075d3e96"	0.3974
"00075b26f1"	"00075d4864"	−0.2668
"00075b26f1"	"00075d5961"	0.0319
"00075b26f1"	"00075d5a63"	0.0278
"00075b26f1"	"00075d6150"	0.1036
"00075b26f1"	"00075d67d0"	−0.175
"00075b26f1"	"00075d67e2"	−0.1957
"00075b26f1"	"00075d73fc"	−0.0593
"00075b26f1"	"00075d7729"	−0.0392
"00075b26f1"	"00075d778c"	0.0902
"00075b26f1"	"00075d7b9e"	0.1329
"00075b26f1"	"00075d7c8f"	−0.1221
"00075b26f1"	"00075d804d"	0.0211
"00075b26f1"	"00075d819f"	−0.0525
"00075b26f1"	"00075d8601"	0.2603
"00075b26f1"	"00075d8c6a"	0.0184
"00075b26f1"	"00075dfedc"	0.1223
"00075b26f1"	"00075e05f2"	0.0569
"00075b26f1"	"00075e0837"	0.1292
"00075b26f1"	"00075e092e"	−0.0369
"00075b26f1"	"00075e0965"	−0.468
"00075b26f1"	"00075e0bc8"	−0.0584
"00075b26f1"	"00075e0fbb"	−0.2594
"00075b2920"	"00075b2a64"	0.417
"00075b2920"	"00075b2a9d"	0.2098
"00075b2920"	"00075b2b37"	−0.0104

"00075b2920"	"00075b2cdd"	-0.1499
"00075b2920"	"00075b3038"	0.0611
"00075b2920"	"00075b30fe"	-0.1193
"00075b2920"	"00075b3362"	-0.2334
"00075b2920"	"00075b350a"	0.0847
"00075b2920"	"00075b350e"	-0.1367
"00075b2920"	"00075b3651"	-0.2419
"00075b2920"	"00075b38ca"	-0.2381
"00075b2920"	"00075b39cc"	-0.1248
"00075b2920"	"00075b3e1e"	0.0249
"00075b2920"	"00075b3e57"	0.0143
"00075b2920"	"00075b4079"	0.0656
"00075b2920"	"00075b4150"	0.5296
"00075b2920"	"00075b4194"	0.2112
"00075b2920"	"00075b42d5"	-0.0515
"00075b2920"	"00075b4424"	0.191
"00075b2920"	"00075b4470"	0.32
"00075b2920"	"00075b47ed"	-0.2995
"00075b2920"	"00075b4850"	0.3321
"00075b2920"	"00075b4ca0"	-0.0374
"00075b2920"	"00075b4d7f"	-0.045
"00075b2920"	"00075b520f"	-0.1772
"00075b2920"	"00075b525f"	-0.1706
"00075b2920"	"00075b58f8"	0.2459
"00075b2920"	"00075b5bcc"	0.1223
"00075b2920"	"00075b5bfa"	-0.1705
"00075b2920"	"00075b6339"	0.1082
"00075b2920"	"00075b6658"	-0.4543
"00075b2920"	"00075b679a"	0.0018
"00075b2920"	"00075b6cb7"	0.1626
"00075b2920"	"00075b6df8"	-0.3016
"00075b2920"	"00075b6ff6"	0.253
"00075b2920"	"00075b70ee"	0.2058
"00075b2920"	"00075b7157"	-0.0378
"00075b2920"	"00075b7225"	-0.1502
"00075b2920"	"00075b7c89"	-0.0652
"00075b2920"	"00075b9048"	0.264
"00075b2920"	"00075d0801"	0.1179
"00075b2920"	"00075d1820"	-0.3156
"00075b2920"	"00075d1f3d"	0.2315
"00075b2920"	"00075d2329"	-0.2745
"00075b2920"	"00075d2b9b"	0.2004
"00075b2920"	"00075d3941"	-0.2052
"00075b2920"	"00075d3e96"	0.2598
"00075b2920"	"00075d4864"	-0.0375
"00075b2920"	"00075d5961"	-0.0741
"00075b2920"	"00075d5a63"	0.2369
"00075b2920"	"00075d6150"	-0.1444
"00075b2920"	"00075d67d0"	-0.3181
"00075b2920"	"00075d67e2"	-0.1546

"00075b2920"	"00075d73fc"	-0.2886
"00075b2920"	"00075d7729"	-0.2195
"00075b2920"	"00075d778c"	-0.3878
"00075b2920"	"00075d7b9e"	-0.2156
"00075b2920"	"00075d7c8f"	-0.1219
"00075b2920"	"00075d804d"	0.2033
"00075b2920"	"00075d819f"	0.0042
"00075b2920"	"00075d8601"	-0.1293
"00075b2920"	"00075d8c6a"	-0.0656
"00075b2920"	"00075dfedc"	0.0362
"00075b2920"	"00075e05f2"	0.0953
"00075b2920"	"00075e0837"	0.1752
"00075b2920"	"00075e092e"	-0.2609
"00075b2920"	"00075e0965"	0.4358
"00075b2920"	"00075e0bc8"	0.2351
"00075b2920"	"00075e0fbb"	-0.3067
"00075b2a64"	"00075b2a9d"	0.0817
"00075b2a64"	"00075b2b37"	-0.3544
"00075b2a64"	"00075b2cdd"	-0.2556
"00075b2a64"	"00075b3038"	0.1278
"00075b2a64"	"00075b30fe"	-0.3377
"00075b2a64"	"00075b3362"	-0.3904
"00075b2a64"	"00075b350a"	0.1502
"00075b2a64"	"00075b350e"	-0.1358
"00075b2a64"	"00075b3651"	-0.2178
"00075b2a64"	"00075b38ca"	-0.1831
"00075b2a64"	"00075b39cc"	-0.1655
"00075b2a64"	"00075b3e1e"	-0.0647
"00075b2a64"	"00075b3e57"	-0.1472
"00075b2a64"	"00075b4079"	0.0745
"00075b2a64"	"00075b4150"	0.3696
"00075b2a64"	"00075b4194"	0.1569
"00075b2a64"	"00075b42d5"	-0.0979
"00075b2a64"	"00075b4424"	-0.1143
"00075b2a64"	"00075b4470"	0.0986
"00075b2a64"	"00075b47ed"	-0.3585
"00075b2a64"	"00075b4850"	0.303
"00075b2a64"	"00075b4ca0"	0.0031
"00075b2a64"	"00075b4d7f"	0.0024
"00075b2a64"	"00075b520f"	-0.2192
"00075b2a64"	"00075b525f"	-0.3481
"00075b2a64"	"00075b58f8"	0.1126
"00075b2a64"	"00075b5bcc"	0.122
"00075b2a64"	"00075b5bfa"	-0.1937
"00075b2a64"	"00075b6339"	0.1106
"00075b2a64"	"00075b6658"	-0.463
"00075b2a64"	"00075b679a"	-0.2696
"00075b2a64"	"00075b6cb7"	0.1906
"00075b2a64"	"00075b6df8"	-0.3123
"00075b2a64"	"00075b6ff6"	0.2459

"00075b2a64"	"00075b70ee"	0.212
"00075b2a64"	"00075b7157"	-0.0139
"00075b2a64"	"00075b7225"	-0.2534
"00075b2a64"	"00075b7c89"	-0.1689
"00075b2a64"	"00075b9048"	0.3291
"00075b2a64"	"00075d0801"	-0.0276
"00075b2a64"	"00075d1820"	-0.4095
"00075b2a64"	"00075d1f3d"	0.3581
"00075b2a64"	"00075d2329"	-0.2732
"00075b2a64"	"00075d2b9b"	0.4229
"00075b2a64"	"00075d3941"	-0.3704
"00075b2a64"	"00075d3e96"	-0.2876
"00075b2a64"	"00075d4864"	-0.4173
"00075b2a64"	"00075d5961"	-0.1489
"00075b2a64"	"00075d5a63"	0.1493
"00075b2a64"	"00075d6150"	-0.3225
"00075b2a64"	"00075d67d0"	-0.1041
"00075b2a64"	"00075d67e2"	-0.0617
"00075b2a64"	"00075d73fc"	-0.5549
"00075b2a64"	"00075d7729"	-0.2855
"00075b2a64"	"00075d778c"	-0.526
"00075b2a64"	"00075d7b9e"	-0.2738
"00075b2a64"	"00075d7c8f"	-0.0727
"00075b2a64"	"00075d804d"	0.2956
"00075b2a64"	"00075d819f"	0.0562
"00075b2a64"	"00075d8601"	-0.2841
"00075b2a64"	"00075d8c6a"	-0.127
"00075b2a64"	"00075dfedc"	0.0144
"00075b2a64"	"00075e05f2"	0.0917
"00075b2a64"	"00075e0837"	-0.0086
"00075b2a64"	"00075e092e"	-0.4167
"00075b2a64"	"00075e0965"	0.1864
"00075b2a64"	"00075e0bc8"	0.1886
"00075b2a64"	"00075e0fbb"	-0.3651
"00075b2a9d"	"00075b2b37"	-0.0148
"00075b2a9d"	"00075b2cdd"	0.045
"00075b2a9d"	"00075b3038"	0.0068
"00075b2a9d"	"00075b30fe"	-0.0528
"00075b2a9d"	"00075b3362"	-0.4375
"00075b2a9d"	"00075b350a"	0.038
"00075b2a9d"	"00075b350e"	-0.0025
"00075b2a9d"	"00075b3651"	-0.3422
"00075b2a9d"	"00075b38ca"	-0.34
"00075b2a9d"	"00075b39cc"	0.0799
"00075b2a9d"	"00075b3e1e"	0.1642
"00075b2a9d"	"00075b3e57"	-0.3465
"00075b2a9d"	"00075b4079"	-0.1155
"00075b2a9d"	"00075b4150"	-0.0043
"00075b2a9d"	"00075b4194"	0.1855
"00075b2a9d"	"00075b42d5"	-0.1367

"00075b2a9d"	"00075b4424"	−0.3985
"00075b2a9d"	"00075b4470"	0.1771
"00075b2a9d"	"00075b47ed"	0.0635
"00075b2a9d"	"00075b4850"	0.2477
"00075b2a9d"	"00075b4ca0"	0.2272
"00075b2a9d"	"00075b4d7f"	−0.2593
"00075b2a9d"	"00075b520f"	0.1148
"00075b2a9d"	"00075b525f"	−0.251
"00075b2a9d"	"00075b58f8"	0.0412
"00075b2a9d"	"00075b5bcc"	−0.0458
"00075b2a9d"	"00075b5bfa"	−0.3329
"00075b2a9d"	"00075b6339"	0.0844
"00075b2a9d"	"00075b6658"	−0.4173
"00075b2a9d"	"00075b679a"	−0.0788
"00075b2a9d"	"00075b6cb7"	0.19
"00075b2a9d"	"00075b6df8"	−0.5132
"00075b2a9d"	"00075b6ff6"	−0.1365
"00075b2a9d"	"00075b70ee"	−0.1381
"00075b2a9d"	"00075b7157"	−0.1736
"00075b2a9d"	"00075b7225"	−0.1955
"00075b2a9d"	"00075b7c89"	−0.044
"00075b2a9d"	"00075b9048"	0.0164
"00075b2a9d"	"00075d0801"	0.2085
"00075b2a9d"	"00075d1820"	−0.1668
"00075b2a9d"	"00075d1f3d"	0.2861
"00075b2a9d"	"00075d2329"	0.0277
"00075b2a9d"	"00075d2b9b"	0.1932
"00075b2a9d"	"00075d3941"	−0.0409
"00075b2a9d"	"00075d3e96"	−0.0207
"00075b2a9d"	"00075d4864"	−0.2284
"00075b2a9d"	"00075d5961"	−0.1522
"00075b2a9d"	"00075d5a63"	0.1393
"00075b2a9d"	"00075d6150"	−0.105
"00075b2a9d"	"00075d67d0"	0.093
"00075b2a9d"	"00075d67e2"	−0.2668
"00075b2a9d"	"00075d73fc"	−0.3265
"00075b2a9d"	"00075d7729"	−0.2023
"00075b2a9d"	"00075d778c"	−0.4727
"00075b2a9d"	"00075d7b9e"	−0.0898
"00075b2a9d"	"00075d7c8f"	0.1051
"00075b2a9d"	"00075d804d"	0.3518
"00075b2a9d"	"00075d819f"	0.5242
"00075b2a9d"	"00075d8601"	−0.1255
"00075b2a9d"	"00075d8c6a"	−0.3166
"00075b2a9d"	"00075dfedc"	−0.0868
"00075b2a9d"	"00075e05f2"	−0.0615
"00075b2a9d"	"00075e0837"	0.5313
"00075b2a9d"	"00075e092e"	−0.021
"00075b2a9d"	"00075e0965"	−0.2121
"00075b2a9d"	"00075e0bc8"	0.3224

"00075b2a9d"	"00075e0fbb"	-0.2522
"00075b2b37"	"00075b2cdd"	-0.1593
"00075b2b37"	"00075b3038"	-0.2615
"00075b2b37"	"00075b30fe"	-0.2751
"00075b2b37"	"00075b3362"	-0.0424
"00075b2b37"	"00075b350a"	-0.1756
"00075b2b37"	"00075b350e"	-0.3153
"00075b2b37"	"00075b3651"	-0.458
"00075b2b37"	"00075b38ca"	-0.3488
"00075b2b37"	"00075b39cc"	-0.316
"00075b2b37"	"00075b3e1e"	-0.1969
"00075b2b37"	"00075b3e57"	0.126
"00075b2b37"	"00075b4079"	-0.1157
"00075b2b37"	"00075b4150"	0.0112
"00075b2b37"	"00075b4194"	-0.3927
"00075b2b37"	"00075b42d5"	-0.4335
"00075b2b37"	"00075b4424"	-0.1335
"00075b2b37"	"00075b4470"	-0.2645
"00075b2b37"	"00075b47ed"	-0.3777
"00075b2b37"	"00075b4850"	-0.3187
"00075b2b37"	"00075b4ca0"	-0.3777
"00075b2b37"	"00075b4d7f"	-0.1687
"00075b2b37"	"00075b520f"	0.291
"00075b2b37"	"00075b525f"	-0.1132
"00075b2b37"	"00075b58f8"	0.0666
"00075b2b37"	"00075b5bcc"	-0.1176
"00075b2b37"	"00075b5bfa"	-0.2402
"00075b2b37"	"00075b6339"	-0.1399
"00075b2b37"	"00075b6658"	0.0162
"00075b2b37"	"00075b679a"	-0.2199
"00075b2b37"	"00075b6cb7"	-0.5044
"00075b2b37"	"00075b6df8"	-0.1485
"00075b2b37"	"00075b6ff6"	-0.2029
"00075b2b37"	"00075b70ee"	-0.3959
"00075b2b37"	"00075b7157"	-0.0433
"00075b2b37"	"00075b7225"	-0.399
"00075b2b37"	"00075b7c89"	-0.4638
"00075b2b37"	"00075b9048"	-0.3914
"00075b2b37"	"00075d0801"	0.0251
"00075b2b37"	"00075d1820"	0.0434
"00075b2b37"	"00075d1f3d"	-0.5223
"00075b2b37"	"00075d2329"	-0.244
"00075b2b37"	"00075d2b9b"	-0.5653
"00075b2b37"	"00075d3941"	-0.2768
"00075b2b37"	"00075d3e96"	0.0395
"00075b2b37"	"00075d4864"	-0.0678
"00075b2b37"	"00075d5961"	0.0875
"00075b2b37"	"00075d5a63"	-0.141
"00075b2b37"	"00075d6150"	-0.1672
"00075b2b37"	"00075d67d0"	-0.4402

"00075b2b37"	"00075d67e2"	-0.4164
"00075b2b37"	"00075d73fc"	-0.4887
"00075b2b37"	"00075d7729"	0.0176
"00075b2b37"	"00075d778c"	-0.1139
"00075b2b37"	"00075d7b9e"	0.0844
"00075b2b37"	"00075d7c8f"	-0.2649
"00075b2b37"	"00075d804d"	0.0086
"00075b2b37"	"00075d819f"	-0.3064
"00075b2b37"	"00075d8601"	0.137
"00075b2b37"	"00075d8c6a"	-0.2948
"00075b2b37"	"00075dfedc"	-0.2965
"00075b2b37"	"00075e05f2"	-0.5946
"00075b2b37"	"00075e0837"	-0.3093
"00075b2b37"	"00075e092e"	-0.2818
"00075b2b37"	"00075e0965"	-0.1229
"00075b2b37"	"00075e0bc8"	-0.1643
"00075b2b37"	"00075e0fbb"	-0.3326
"00075b2cdd"	"00075b3038"	0.071
"00075b2cdd"	"00075b30fe"	-0.0294
"00075b2cdd"	"00075b3362"	-0.2393
"00075b2cdd"	"00075b350a"	-0.2168
"00075b2cdd"	"00075b350e"	0.1214
"00075b2cdd"	"00075b3651"	0.0109
"00075b2cdd"	"00075b38ca"	0.0265
"00075b2cdd"	"00075b39cc"	0.0583
"00075b2cdd"	"00075b3e1e"	0.2702
"00075b2cdd"	"00075b3e57"	0.2273
"00075b2cdd"	"00075b4079"	-0.2822
"00075b2cdd"	"00075b4150"	-0.1965
"00075b2cdd"	"00075b4194"	0.0608
"00075b2cdd"	"00075b42d5"	0.186
"00075b2cdd"	"00075b4424"	0.1049
"00075b2cdd"	"00075b4470"	0.0184
"00075b2cdd"	"00075b47ed"	0.1337
"00075b2cdd"	"00075b4850"	-0.116
"00075b2cdd"	"00075b4ca0"	-0.0278
"00075b2cdd"	"00075b4d7f"	0.0578
"00075b2cdd"	"00075b520f"	-0.1018
"00075b2cdd"	"00075b525f"	0.0649
"00075b2cdd"	"00075b58f8"	-0.1062
"00075b2cdd"	"00075b5bcc"	-0.1439
"00075b2cdd"	"00075b5bfa"	-0.1801
"00075b2cdd"	"00075b6339"	-0.1397
"00075b2cdd"	"00075b6658"	-0.0469
"00075b2cdd"	"00075b679a"	-0.0906
"00075b2cdd"	"00075b6cb7"	-0.1466
"00075b2cdd"	"00075b6df8"	0.0577
"00075b2cdd"	"00075b6ff6"	-0.191
"00075b2cdd"	"00075b70ee"	-0.2548
"00075b2cdd"	"00075b7157"	0.2997

"00075b2cdd"	"00075b7225"	0.0348
"00075b2cdd"	"00075b7c89"	-0.0896
"00075b2cdd"	"00075b9048"	0.1045
"00075b2cdd"	"00075d0801"	-0.0218
"00075b2cdd"	"00075d1820"	7e-04
"00075b2cdd"	"00075d1f3d"	-0.1149
"00075b2cdd"	"00075d2329"	0.1666
"00075b2cdd"	"00075d2b9b"	-0.1709
"00075b2cdd"	"00075d3941"	0.0644
"00075b2cdd"	"00075d3e96"	-0.0233
"00075b2cdd"	"00075d4864"	-0.0334
"00075b2cdd"	"00075d5961"	0.0723
"00075b2cdd"	"00075d5a63"	0.003
"00075b2cdd"	"00075d6150"	0.0766
"00075b2cdd"	"00075d67d0"	-0.2819
"00075b2cdd"	"00075d67e2"	-0.0306
"00075b2cdd"	"00075d73fc"	0.2025
"00075b2cdd"	"00075d7729"	-0.1694
"00075b2cdd"	"00075d778c"	-0.1748
"00075b2cdd"	"00075d7b9e"	0.2452
"00075b2cdd"	"00075d7c8f"	0.1017
"00075b2cdd"	"00075d804d"	0.0085
"00075b2cdd"	"00075d819f"	0.0898
"00075b2cdd"	"00075d8601"	0.3053
"00075b2cdd"	"00075d8c6a"	0.0082
"00075b2cdd"	"00075dfedc"	0.0953
"00075b2cdd"	"00075e05f2"	0.0073
"00075b2cdd"	"00075e0837"	0.0589
"00075b2cdd"	"00075e092e"	0.1211
"00075b2cdd"	"00075e0965"	-0.3945
"00075b2cdd"	"00075e0bc8"	-0.0924
"00075b2cdd"	"00075e0fbb"	-0.1734
"00075b3038"	"00075b30fe"	0.1468
"00075b3038"	"00075b3362"	-0.3356
"00075b3038"	"00075b350a"	-0.121
"00075b3038"	"00075b350e"	0.1531
"00075b3038"	"00075b3651"	0.0817
"00075b3038"	"00075b38ca"	0.1206
"00075b3038"	"00075b39cc"	0.1924
"00075b3038"	"00075b3e1e"	0.1249
"00075b3038"	"00075b3e57"	0.0479
"00075b3038"	"00075b4079"	0.0045
"00075b3038"	"00075b4150"	0.0075
"00075b3038"	"00075b4194"	0.2385
"00075b3038"	"00075b42d5"	0.3305
"00075b3038"	"00075b4424"	-0.0827
"00075b3038"	"00075b4470"	0.0807
"00075b3038"	"00075b47ed"	0.2123
"00075b3038"	"00075b4850"	0.1946
"00075b3038"	"00075b4ca0"	0.0884

"00075b3038"	"00075b4d7f"	0.2201
"00075b3038"	"00075b520f"	0.1016
"00075b3038"	"00075b525f"	-0.1008
"00075b3038"	"00075b58f8"	-0.154
"00075b3038"	"00075b5bcc"	0.3076
"00075b3038"	"00075b5bfa"	0.6691
"00075b3038"	"00075b6339"	0.0658
"00075b3038"	"00075b6658"	-0.1546
"00075b3038"	"00075b679a"	0.2208
"00075b3038"	"00075b6cb7"	-0.0702
"00075b3038"	"00075b6df8"	0.3149
"00075b3038"	"00075b6ff6"	0.2711
"00075b3038"	"00075b70ee"	0.1226
"00075b3038"	"00075b7157"	0.2193
"00075b3038"	"00075b7225"	0.0336
"00075b3038"	"00075b7c89"	0.1661
"00075b3038"	"00075b9048"	0.4605
"00075b3038"	"00075d0801"	0.291
"00075b3038"	"00075d1820"	-0.0774
"00075b3038"	"00075d1f3d"	0.1462
"00075b3038"	"00075d2329"	0.4027
"00075b3038"	"00075d2b9b"	0.1666
"00075b3038"	"00075d3941"	0.152
"00075b3038"	"00075d3e96"	0.1574
"00075b3038"	"00075d4864"	-0.1414
"00075b3038"	"00075d5961"	0.043
"00075b3038"	"00075d5a63"	0.2089
"00075b3038"	"00075d6150"	-0.0046
"00075b3038"	"00075d67d0"	0.0047
"00075b3038"	"00075d67e2"	-0.0388
"00075b3038"	"00075d73fc"	0.1137
"00075b3038"	"00075d7729"	-0.0816
"00075b3038"	"00075d778c"	-0.2083
"00075b3038"	"00075d7b9e"	-0.0213
"00075b3038"	"00075d7c8f"	0.0589
"00075b3038"	"00075d804d"	0.3046
"00075b3038"	"00075d819f"	0.1095
"00075b3038"	"00075d8601"	0.1904
"00075b3038"	"00075d8c6a"	0.2976
"00075b3038"	"00075dfedc"	0.22
"00075b3038"	"00075e05f2"	0.184
"00075b3038"	"00075e0837"	0.1923
"00075b3038"	"00075e092e"	-0.0539
"00075b3038"	"00075e0965"	-0.172
"00075b3038"	"00075e0bc8"	0.2508
"00075b3038"	"00075e0fbb"	-0.0521
"00075b30fe"	"00075b3362"	-0.2702
"00075b30fe"	"00075b350a"	-0.0303
"00075b30fe"	"00075b350e"	0.0029
"00075b30fe"	"00075b3651"	-0.0949

"00075b30fe"	"00075b38ca"	-0.0306
"00075b30fe"	"00075b39cc"	-0.1084
"00075b30fe"	"00075b3e1e"	-0.0899
"00075b30fe"	"00075b3e57"	-0.0431
"00075b30fe"	"00075b4079"	-0.1804
"00075b30fe"	"00075b4150"	-0.3444
"00075b30fe"	"00075b4194"	-0.1688
"00075b30fe"	"00075b42d5"	0.2825
"00075b30fe"	"00075b4424"	-0.1423
"00075b30fe"	"00075b4470"	0.1264
"00075b30fe"	"00075b47ed"	-0.1233
"00075b30fe"	"00075b4850"	0.1041
"00075b30fe"	"00075b4ca0"	0.0969
"00075b30fe"	"00075b4d7f"	0.0676
"00075b30fe"	"00075b520f"	-0.2728
"00075b30fe"	"00075b525f"	0.1548
"00075b30fe"	"00075b58f8"	0.0095
"00075b30fe"	"00075b5bcc"	0.0333
"00075b30fe"	"00075b5bfa"	-0.0797
"00075b30fe"	"00075b6339"	-0.1277
"00075b30fe"	"00075b6658"	-0.1654
"00075b30fe"	"00075b679a"	0.0645
"00075b30fe"	"00075b6cb7"	-0.078
"00075b30fe"	"00075b6df8"	-0.0357
"00075b30fe"	"00075b6ff6"	0.2787
"00075b30fe"	"00075b70ee"	-0.0677
"00075b30fe"	"00075b7157"	0.1176
"00075b30fe"	"00075b7225"	0.0661
"00075b30fe"	"00075b7c89"	-0.038
"00075b30fe"	"00075b9048"	-0.0573
"00075b30fe"	"00075d0801"	-0.1401
"00075b30fe"	"00075d1820"	-0.1678
"00075b30fe"	"00075d1f3d"	0.0473
"00075b30fe"	"00075d2329"	0.0809
"00075b30fe"	"00075d2b9b"	-0.1176
"00075b30fe"	"00075d3941"	-0.2943
"00075b30fe"	"00075d3e96"	0.0342
"00075b30fe"	"00075d4864"	0.2677
"00075b30fe"	"00075d5961"	-0.113
"00075b30fe"	"00075d5a63"	0.098
"00075b30fe"	"00075d6150"	-0.1366
"00075b30fe"	"00075d67d0"	-0.2057
"00075b30fe"	"00075d67e2"	-0.1484
"00075b30fe"	"00075d73fc"	-0.1376
"00075b30fe"	"00075d7729"	-0.084
"00075b30fe"	"00075d778c"	0.0083
"00075b30fe"	"00075d7b9e"	0.0322
"00075b30fe"	"00075d7c8f"	0.115
"00075b30fe"	"00075d804d"	0.0822
"00075b30fe"	"00075d819f"	0.0576

"00075b30fe"	"00075d8601"	-0.2878
"00075b30fe"	"00075d8c6a"	-0.1688
"00075b30fe"	"00075dfedc"	-0.141
"00075b30fe"	"00075e05f2"	-0.1698
"00075b30fe"	"00075e0837"	0.0799
"00075b30fe"	"00075e092e"	0.4792
"00075b30fe"	"00075e0965"	-0.3167
"00075b30fe"	"00075e0bc8"	-0.059
"00075b30fe"	"00075e0fbb"	0.5627
"00075b3362"	"00075b350a"	-0.2097
"00075b3362"	"00075b350e"	-0.2714
"00075b3362"	"00075b3651"	0.3803
"00075b3362"	"00075b38ca"	-0.2736
"00075b3362"	"00075b39cc"	-0.1719
"00075b3362"	"00075b3e1e"	-0.253
"00075b3362"	"00075b3e57"	0.0646
"00075b3362"	"00075b4079"	-0.1262
"00075b3362"	"00075b4150"	-0.263
"00075b3362"	"00075b4194"	-0.109
"00075b3362"	"00075b42d5"	-0.4008
"00075b3362"	"00075b4424"	-0.0477
"00075b3362"	"00075b4470"	-0.1759
"00075b3362"	"00075b47ed"	-0.0529
"00075b3362"	"00075b4850"	-0.2068
"00075b3362"	"00075b4ca0"	-0.3354
"00075b3362"	"00075b4d7f"	0.0978
"00075b3362"	"00075b520f"	-0.128
"00075b3362"	"00075b525f"	0.3733
"00075b3362"	"00075b58f8"	-0.4488
"00075b3362"	"00075b5bcc"	0.159
"00075b3362"	"00075b5bfa"	-0.2887
"00075b3362"	"00075b6339"	0.092
"00075b3362"	"00075b6658"	0.4907
"00075b3362"	"00075b679a"	-0.0117
"00075b3362"	"00075b6cb7"	-0.3298
"00075b3362"	"00075b6df8"	-0.0208
"00075b3362"	"00075b6ff6"	-0.2211
"00075b3362"	"00075b70ee"	-0.5169
"00075b3362"	"00075b7157"	0.1057
"00075b3362"	"00075b7225"	-0.1191
"00075b3362"	"00075b7c89"	-0.217
"00075b3362"	"00075b9048"	-0.3013
"00075b3362"	"00075d0801"	-0.0137
"00075b3362"	"00075d1820"	0.0695
"00075b3362"	"00075d1f3d"	-0.3178
"00075b3362"	"00075d2329"	-0.3235
"00075b3362"	"00075d2b9b"	-0.255
"00075b3362"	"00075d3941"	-0.1668
"00075b3362"	"00075d3e96"	0.1731
"00075b3362"	"00075d4864"	-0.2331

"00075b3362"	"00075d5961"	−0.0215
"00075b3362"	"00075d5a63"	0.0499
"00075b3362"	"00075d6150"	−0.1516
"00075b3362"	"00075d67d0"	−0.1942
"00075b3362"	"00075d67e2"	−0.2091
"00075b3362"	"00075d73fc"	−0.1331
"00075b3362"	"00075d7729"	−0.1284
"00075b3362"	"00075d778c"	0.4905
"00075b3362"	"00075d7b9e"	0.3113
"00075b3362"	"00075d7c8f"	−0.0184
"00075b3362"	"00075d804d"	−0.369
"00075b3362"	"00075d819f"	−0.2138
"00075b3362"	"00075d8601"	−0.2738
"00075b3362"	"00075d8c6a"	−0.2226
"00075b3362"	"00075dfedc"	−0.0518
"00075b3362"	"00075e05f2"	−0.3627
"00075b3362"	"00075e0837"	−0.3636
"00075b3362"	"00075e092e"	−0.0057
"00075b3362"	"00075e0965"	−0.3757
"00075b3362"	"00075e0bc8"	−0.2399
"00075b3362"	"00075e0fbb"	−0.1058
"00075b350a"	"00075b350e"	0.0038
"00075b350a"	"00075b3651"	−0.2482
"00075b350a"	"00075b38ca"	0.0707
"00075b350a"	"00075b39cc"	−0.0526
"00075b350a"	"00075b3e1e"	0.0951
"00075b350a"	"00075b3e57"	0.003
"00075b350a"	"00075b4079"	0.482
"00075b350a"	"00075b4150"	−0.1256
"00075b350a"	"00075b4194"	0.1517
"00075b350a"	"00075b42d5"	−0.025
"00075b350a"	"00075b4424"	−0.2042
"00075b350a"	"00075b4470"	0.2337
"00075b350a"	"00075b47ed"	−0.0433
"00075b350a"	"00075b4850"	0.1935
"00075b350a"	"00075b4ca0"	−0.0896
"00075b350a"	"00075b4d7f"	0.0477
"00075b350a"	"00075b520f"	−0.176
"00075b350a"	"00075b525f"	0.0149
"00075b350a"	"00075b58f8"	−0.2254
"00075b350a"	"00075b5bcc"	0.3135
"00075b350a"	"00075b5bfa"	−0.5007
"00075b350a"	"00075b6339"	0.1234
"00075b350a"	"00075b6658"	−0.169
"00075b350a"	"00075b679a"	−0.0461
"00075b350a"	"00075b6cb7"	−0.0017
"00075b350a"	"00075b6df8"	−0.1119
"00075b350a"	"00075b6ff6"	0.2216
"00075b350a"	"00075b70ee"	−0.1409
"00075b350a"	"00075b7157"	−0.0294

"00075b350a"	"00075b7225"	−0.1197
"00075b350a"	"00075b7c89"	0.0489
"00075b350a"	"00075b9048"	−0.0289
"00075b350a"	"00075d0801"	0.0364
"00075b350a"	"00075d1820"	−0.3651
"00075b350a"	"00075d1f3d"	0.233
"00075b350a"	"00075d2329"	−0.0952
"00075b350a"	"00075d2b9b"	0.1559
"00075b350a"	"00075d3941"	−0.1412
"00075b350a"	"00075d3e96"	−0.3423
"00075b350a"	"00075d4864"	−0.2051
"00075b350a"	"00075d5961"	−0.1543
"00075b350a"	"00075d5a63"	0.2589
"00075b350a"	"00075d6150"	−0.2644
"00075b350a"	"00075d67d0"	−0.0645
"00075b350a"	"00075d67e2"	−0.0183
"00075b350a"	"00075d73fc"	−0.3189
"00075b350a"	"00075d7729"	−0.1616
"00075b350a"	"00075d778c"	−0.2261
"00075b350a"	"00075d7b9e"	0.06
"00075b350a"	"00075d7c8f"	0.1124
"00075b350a"	"00075d804d"	0.0571
"00075b350a"	"00075d819f"	0.0635
"00075b350a"	"00075d8601"	−0.1991
"00075b350a"	"00075d8c6a"	−0.1095
"00075b350a"	"00075dfedc"	0.0261
"00075b350a"	"00075e05f2"	−0.2118
"00075b350a"	"00075e0837"	0.0508
"00075b350a"	"00075e092e"	0.237
"00075b350a"	"00075e0965"	−0.1591
"00075b350a"	"00075e0bc8"	0.1542
"00075b350a"	"00075e0fbb"	0.2108
"00075b350e"	"00075b3651"	−0.2717
"00075b350e"	"00075b38ca"	0.2413
"00075b350e"	"00075b39cc"	−0.0873
"00075b350e"	"00075b3e1e"	0.0802
"00075b350e"	"00075b3e57"	−0.1552
"00075b350e"	"00075b4079"	0.0574
"00075b350e"	"00075b4150"	−0.0062
"00075b350e"	"00075b4194"	0.296
"00075b350e"	"00075b42d5"	0.2109
"00075b350e"	"00075b4424"	−0.1792
"00075b350e"	"00075b4470"	0.0826
"00075b350e"	"00075b47ed"	0.0839
"00075b350e"	"00075b4850"	−0.0231
"00075b350e"	"00075b4ca0"	0.0858
"00075b350e"	"00075b4d7f"	0.026
"00075b350e"	"00075b520f"	−0.041
"00075b350e"	"00075b525f"	−0.1148
"00075b350e"	"00075b58f8"	−0.134

"00075b350e"	"00075b5bcc"	0.2004
"00075b350e"	"00075b5bfa"	-0.2071
"00075b350e"	"00075b6339"	-0.0689
"00075b350e"	"00075b6658"	-0.2631
"00075b350e"	"00075b679a"	-0.02
"00075b350e"	"00075b6cb7"	-0.1701
"00075b350e"	"00075b6df8"	-0.0904
"00075b350e"	"00075b6ff6"	-0.0955
"00075b350e"	"00075b70ee"	-0.1709
"00075b350e"	"00075b7157"	-0.0406
"00075b350e"	"00075b7225"	0.4324
"00075b350e"	"00075b7c89"	0.1737
"00075b350e"	"00075b9048"	0.1386
"00075b350e"	"00075d0801"	0.1422
"00075b350e"	"00075d1820"	-0.0074
"00075b350e"	"00075d1f3d"	0.0719
"00075b350e"	"00075d2329"	0.0471
"00075b350e"	"00075d2b9b"	0.2782
"00075b350e"	"00075d3941"	-0.1212
"00075b350e"	"00075d3e96"	-0.0522
"00075b350e"	"00075d4864"	0.0866
"00075b350e"	"00075d5961"	0.1122
"00075b350e"	"00075d5a63"	-0.0612
"00075b350e"	"00075d6150"	0.0898
"00075b350e"	"00075d67d0"	-0.5092
"00075b350e"	"00075d67e2"	-0.0385
"00075b350e"	"00075d73fc"	0.0887
"00075b350e"	"00075d7729"	-0.3027
"00075b350e"	"00075d778c"	-0.3898
"00075b350e"	"00075d7b9e"	-0.2065
"00075b350e"	"00075d7c8f"	0.0447
"00075b350e"	"00075d804d"	-0.1442
"00075b350e"	"00075d819f"	-0.0059
"00075b350e"	"00075d8601"	-0.0825
"00075b350e"	"00075d8c6a"	0.0183
"00075b350e"	"00075dfedc"	0.0317
"00075b350e"	"00075e05f2"	0.0776
"00075b350e"	"00075e0837"	-0.0676
"00075b350e"	"00075e092e"	-0.038
"00075b350e"	"00075e0965"	-0.3085
"00075b350e"	"00075e0bc8"	0.0786
"00075b350e"	"00075e0fbb"	-0.0563
"00075b3651"	"00075b38ca"	-0.1567
"00075b3651"	"00075b39cc"	0.1491
"00075b3651"	"00075b3e1e"	-0.1683
"00075b3651"	"00075b3e57"	0.059
"00075b3651"	"00075b4079"	-0.2359
"00075b3651"	"00075b4150"	-0.4038
"00075b3651"	"00075b4194"	-0.0462
"00075b3651"	"00075b42d5"	0.0539

"00075b3651"	"00075b4424"	0.0749
"00075b3651"	"00075b4470"	-0.1018
"00075b3651"	"00075b47ed"	0.0115
"00075b3651"	"00075b4850"	-0.006
"00075b3651"	"00075b4ca0"	-0.1699
"00075b3651"	"00075b4d7f"	0.1129
"00075b3651"	"00075b520f"	-0.0342
"00075b3651"	"00075b525f"	0.3525
"00075b3651"	"00075b58f8"	-0.4479
"00075b3651"	"00075b5bcc"	0.1376
"00075b3651"	"00075b5bfa"	-0.0078
"00075b3651"	"00075b6339"	-0.0978
"00075b3651"	"00075b6658"	0.4197
"00075b3651"	"00075b679a"	0.081
"00075b3651"	"00075b6cb7"	-0.1978
"00075b3651"	"00075b6df8"	-0.0166
"00075b3651"	"00075b6ff6"	-0.1508
"00075b3651"	"00075b70ee"	-0.4107
"00075b3651"	"00075b7157"	0.213
"00075b3651"	"00075b7225"	-0.1722
"00075b3651"	"00075b7c89"	-0.1931
"00075b3651"	"00075b9048"	0.004
"00075b3651"	"00075d0801"	-0.0454
"00075b3651"	"00075d1820"	0.2003
"00075b3651"	"00075d1f3d"	-0.1783
"00075b3651"	"00075d2329"	-0.1632
"00075b3651"	"00075d2b9b"	-0.2351
"00075b3651"	"00075d3941"	-0.0252
"00075b3651"	"00075d3e96"	0.2262
"00075b3651"	"00075d4864"	-0.3595
"00075b3651"	"00075d5961"	-0.0822
"00075b3651"	"00075d5a63"	-0.0805
"00075b3651"	"00075d6150"	0.042
"00075b3651"	"00075d67d0"	-0.0739
"00075b3651"	"00075d67e2"	-0.2558
"00075b3651"	"00075d73fc"	-0.0627
"00075b3651"	"00075d7729"	-0.1615
"00075b3651"	"00075d778c"	0.193
"00075b3651"	"00075d7b9e"	0.3181
"00075b3651"	"00075d7c8f"	-0.1359
"00075b3651"	"00075d804d"	-0.1869
"00075b3651"	"00075d819f"	-0.078
"00075b3651"	"00075d8601"	0.0243
"00075b3651"	"00075d8c6a"	-0.0866
"00075b3651"	"00075dfedc"	0.0836
"00075b3651"	"00075e05f2"	-0.0149
"00075b3651"	"00075e0837"	0.005
"00075b3651"	"00075e092e"	-0.019
"00075b3651"	"00075e0965"	-0.5951
"00075b3651"	"00075e0bc8"	-0.1634

"00075b3651"	"00075e0fbb"	-0.1836
"00075b38ca"	"00075b39cc"	-0.0812
"00075b38ca"	"00075b3e1e"	0.3871
"00075b38ca"	"00075b3e57"	-0.1312
"00075b38ca"	"00075b4079"	-0.1269
"00075b38ca"	"00075b4150"	-0.3305
"00075b38ca"	"00075b4194"	0.0106
"00075b38ca"	"00075b42d5"	0.6503
"00075b38ca"	"00075b4424"	9e-04
"00075b38ca"	"00075b4470"	0.1322
"00075b38ca"	"00075b47ed"	0.1101
"00075b38ca"	"00075b4850"	-0.055
"00075b38ca"	"00075b4ca0"	-0.427
"00075b38ca"	"00075b4d7f"	0.0363
"00075b38ca"	"00075b520f"	-0.3016
"00075b38ca"	"00075b525f"	-0.2361
"00075b38ca"	"00075b58f8"	-0.0777
"00075b38ca"	"00075b5bcc"	0.1004
"00075b38ca"	"00075b5bfa"	-0.1377
"00075b38ca"	"00075b6339"	-0.1616
"00075b38ca"	"00075b6658"	-0.1874
"00075b38ca"	"00075b679a"	-0.3038
"00075b38ca"	"00075b6cb7"	-0.2507
"00075b38ca"	"00075b6df8"	0.0876
"00075b38ca"	"00075b6ff6"	-0.1663
"00075b38ca"	"00075b70ee"	-0.1407
"00075b38ca"	"00075b7157"	0.0596
"00075b38ca"	"00075b7225"	0.0125
"00075b38ca"	"00075b7c89"	-0.0973
"00075b38ca"	"00075b9048"	-0.1464
"00075b38ca"	"00075d0801"	-0.1082
"00075b38ca"	"00075d1820"	-0.1494
"00075b38ca"	"00075d1f3d"	0.008
"00075b38ca"	"00075d2329"	0.285
"00075b38ca"	"00075d2b9b"	-0.0014
"00075b38ca"	"00075d3941"	-0.1068
"00075b38ca"	"00075d3e96"	-0.1267
"00075b38ca"	"00075d4864"	0.2704
"00075b38ca"	"00075d5961"	0.2126
"00075b38ca"	"00075d5a63"	0.0035
"00075b38ca"	"00075d6150"	0.0119
"00075b38ca"	"00075d67d0"	-0.4946
"00075b38ca"	"00075d67e2"	-0.2911
"00075b38ca"	"00075d73fc"	-0.0622
"00075b38ca"	"00075d7729"	-0.3109
"00075b38ca"	"00075d778c"	-0.1626
"00075b38ca"	"00075d7b9e"	-0.1178
"00075b38ca"	"00075d7c8f"	-0.1393
"00075b38ca"	"00075d804d"	-0.1767
"00075b38ca"	"00075d819f"	-0.238

"00075b38ca"	"00075d8601"	0.0491
"00075b38ca"	"00075d8c6a"	0.1387
"00075b38ca"	"00075dfedc"	-0.0469
"00075b38ca"	"00075e05f2"	-0.1158
"00075b38ca"	"00075e0837"	-0.0669
"00075b38ca"	"00075e092e"	-0.0275
"00075b38ca"	"00075e0965"	-0.1275
"00075b38ca"	"00075e0bc8"	-0.0685
"00075b38ca"	"00075e0fbb"	0.2108
"00075b39cc"	"00075b3e1e"	-0.0401
"00075b39cc"	"00075b3e57"	-0.0916
"00075b39cc"	"00075b4079"	-0.0936
"00075b39cc"	"00075b4150"	-0.3542
"00075b39cc"	"00075b4194"	0.3368
"00075b39cc"	"00075b42d5"	0.0275
"00075b39cc"	"00075b4424"	-0.2968
"00075b39cc"	"00075b4470"	0.065
"00075b39cc"	"00075b47ed"	0.7287
"00075b39cc"	"00075b4850"	0.0059
"00075b39cc"	"00075b4ca0"	0.1009
"00075b39cc"	"00075b4d7f"	0.0387
"00075b39cc"	"00075b520f"	0.124
"00075b39cc"	"00075b525f"	-0.0587
"00075b39cc"	"00075b58f8"	-0.2268
"00075b39cc"	"00075b5bcc"	-0.1404
"00075b39cc"	"00075b5bfa"	-0.1072
"00075b39cc"	"00075b6339"	0.1614
"00075b39cc"	"00075b6658"	0.0398
"00075b39cc"	"00075b679a"	0.005
"00075b39cc"	"00075b6cb7"	-0.0868
"00075b39cc"	"00075b6df8"	-0.1076
"00075b39cc"	"00075b6ff6"	-0.1802
"00075b39cc"	"00075b70ee"	-0.1178
"00075b39cc"	"00075b7157"	0.0856
"00075b39cc"	"00075b7225"	-0.1951
"00075b39cc"	"00075b7c89"	0.0846
"00075b39cc"	"00075b9048"	-0.1022
"00075b39cc"	"00075d0801"	0.3075
"00075b39cc"	"00075d1820"	0.0294
"00075b39cc"	"00075d1f3d"	-0.2505
"00075b39cc"	"00075d2329"	0.4139
"00075b39cc"	"00075d2b9b"	-0.116
"00075b39cc"	"00075d3941"	-0.07
"00075b39cc"	"00075d3e96"	-0.0444
"00075b39cc"	"00075d4864"	-0.0158
"00075b39cc"	"00075d5961"	-0.0334
"00075b39cc"	"00075d5a63"	0.1618
"00075b39cc"	"00075d6150"	-0.0098
"00075b39cc"	"00075d67d0"	0.0772
"00075b39cc"	"00075d67e2"	-0.1409

"00075b39cc"	"00075d73fc"	-0.1366
"00075b39cc"	"00075d7729"	-0.4151
"00075b39cc"	"00075d778c"	-0.17
"00075b39cc"	"00075d7b9e"	-0.0272
"00075b39cc"	"00075d7c8f"	0.1764
"00075b39cc"	"00075d804d"	0.1225
"00075b39cc"	"00075d819f"	0.1246
"00075b39cc"	"00075d8601"	0.2048
"00075b39cc"	"00075d8c6a"	0.1049
"00075b39cc"	"00075dfedc"	0.3175
"00075b39cc"	"00075e05f2"	-0.0304
"00075b39cc"	"00075e0837"	0.141
"00075b39cc"	"00075e092e"	-0.1606
"00075b39cc"	"00075e0965"	-0.5986
"00075b39cc"	"00075e0bc8"	-0.0097
"00075b39cc"	"00075e0fbb"	-0.0926
"00075b3e1e"	"00075b3e57"	0.1247
"00075b3e1e"	"00075b4079"	0.0656
"00075b3e1e"	"00075b4150"	-0.0513
"00075b3e1e"	"00075b4194"	0.0789
"00075b3e1e"	"00075b42d5"	0.3342
"00075b3e1e"	"00075b4424"	0.0312
"00075b3e1e"	"00075b4470"	0.1398
"00075b3e1e"	"00075b47ed"	0.1141
"00075b3e1e"	"00075b4850"	0.0541
"00075b3e1e"	"00075b4ca0"	-0.331
"00075b3e1e"	"00075b4d7f"	0.1059
"00075b3e1e"	"00075b520f"	-0.0806
"00075b3e1e"	"00075b525f"	-0.1811
"00075b3e1e"	"00075b58f8"	-0.1482
"00075b3e1e"	"00075b5bcc"	0.1392
"00075b3e1e"	"00075b5bfa"	-0.3196
"00075b3e1e"	"00075b6339"	0.0833
"00075b3e1e"	"00075b6658"	-0.2545
"00075b3e1e"	"00075b679a"	-0.0155
"00075b3e1e"	"00075b6cb7"	0.0102
"00075b3e1e"	"00075b6df8"	0.0702
"00075b3e1e"	"00075b6ff6"	-0.081
"00075b3e1e"	"00075b70ee"	-0.198
"00075b3e1e"	"00075b7157"	0.0256
"00075b3e1e"	"00075b7225"	0.1274
"00075b3e1e"	"00075b7c89"	-0.0629
"00075b3e1e"	"00075b9048"	0.0963
"00075b3e1e"	"00075d0801"	-0.0039
"00075b3e1e"	"00075d1820"	0.0069
"00075b3e1e"	"00075d1f3d"	0.0548
"00075b3e1e"	"00075d2329"	0.2216
"00075b3e1e"	"00075d2b9b"	0.163
"00075b3e1e"	"00075d3941"	0.0919
"00075b3e1e"	"00075d3e96"	-0.1366

"00075b3e1e"	"00075d4864"	0.143
"00075b3e1e"	"00075d5961"	0.2229
"00075b3e1e"	"00075d5a63"	0.2526
"00075b3e1e"	"00075d6150"	−0.0501
"00075b3e1e"	"00075d67d0"	−0.1426
"00075b3e1e"	"00075d67e2"	0.139
"00075b3e1e"	"00075d73fc"	0.2022
"00075b3e1e"	"00075d7729"	−0.2998
"00075b3e1e"	"00075d778c"	−0.152
"00075b3e1e"	"00075d7b9e"	0.0741
"00075b3e1e"	"00075d7c8f"	0.1783
"00075b3e1e"	"00075d804d"	0.2092
"00075b3e1e"	"00075d819f"	0.1654
"00075b3e1e"	"00075d8601"	0.2081
"00075b3e1e"	"00075d8c6a"	0.0331
"00075b3e1e"	"00075dfedc"	0.1577
"00075b3e1e"	"00075e05f2"	0.0977
"00075b3e1e"	"00075e0837"	0.1974
"00075b3e1e"	"00075e092e"	0.1341
"00075b3e1e"	"00075e0965"	−0.1249
"00075b3e1e"	"00075e0bc8"	0.056
"00075b3e1e"	"00075e0fbb"	−0.0283
"00075b3e57"	"00075b4079"	−0.0961
"00075b3e57"	"00075b4150"	0.0786
"00075b3e57"	"00075b4194"	−0.0595
"00075b3e57"	"00075b42d5"	−0.0841
"00075b3e57"	"00075b4424"	0.3193
"00075b3e57"	"00075b4470"	0.0168
"00075b3e57"	"00075b47ed"	−0.0383
"00075b3e57"	"00075b4850"	−0.1441
"00075b3e57"	"00075b4ca0"	−0.3261
"00075b3e57"	"00075b4d7f"	0.2103
"00075b3e57"	"00075b520f"	−0.0923
"00075b3e57"	"00075b525f"	−0.0822
"00075b3e57"	"00075b58f8"	0.0113
"00075b3e57"	"00075b5bcc"	3e−04
"00075b3e57"	"00075b5bfa"	−0.1399
"00075b3e57"	"00075b6339"	0.0697
"00075b3e57"	"00075b6658"	0.1663
"00075b3e57"	"00075b679a"	0.1701
"00075b3e57"	"00075b6cb7"	−0.1713
"00075b3e57"	"00075b6df8"	0.2434
"00075b3e57"	"00075b6ff6"	0.1241
"00075b3e57"	"00075b70ee"	−0.2682
"00075b3e57"	"00075b7157"	0.1472
"00075b3e57"	"00075b7225"	−0.1136
"00075b3e57"	"00075b7c89"	0.0558
"00075b3e57"	"00075b9048"	0.0785
"00075b3e57"	"00075d0801"	−0.0961
"00075b3e57"	"00075d1820"	0.1373

"00075b3e57"	"00075d1f3d"	-0.2537
"00075b3e57"	"00075d2329"	0.0629
"00075b3e57"	"00075d2b9b"	-0.2722
"00075b3e57"	"00075d3941"	0.0985
"00075b3e57"	"00075d3e96"	0.1438
"00075b3e57"	"00075d4864"	-0.1228
"00075b3e57"	"00075d5961"	0.1326
"00075b3e57"	"00075d5a63"	0.3878
"00075b3e57"	"00075d6150"	-0.2032
"00075b3e57"	"00075d67d0"	-0.0344
"00075b3e57"	"00075d67e2"	0.1644
"00075b3e57"	"00075d73fc"	0.1676
"00075b3e57"	"00075d7729"	0.0117
"00075b3e57"	"00075d778c"	0.0192
"00075b3e57"	"00075d7b9e"	0.1566
"00075b3e57"	"00075d7c8f"	-0.0169
"00075b3e57"	"00075d804d"	0.1462
"00075b3e57"	"00075d819f"	-0.1868
"00075b3e57"	"00075d8601"	0.3015
"00075b3e57"	"00075d8c6a"	-0.0232
"00075b3e57"	"00075dfedc"	-0.0285
"00075b3e57"	"00075e05f2"	0.0108
"00075b3e57"	"00075e0837"	-0.1512
"00075b3e57"	"00075e092e"	0.0235
"00075b3e57"	"00075e0965"	-0.2207
"00075b3e57"	"00075e0bc8"	0.0219
"00075b3e57"	"00075e0fbb"	-0.168
"00075b4079"	"00075b4150"	-0.184
"00075b4079"	"00075b4194"	0.1161
"00075b4079"	"00075b42d5"	-0.1929
"00075b4079"	"00075b4424"	-0.343
"00075b4079"	"00075b4470"	-0.0016
"00075b4079"	"00075b47ed"	-0.0545
"00075b4079"	"00075b4850"	0.0044
"00075b4079"	"00075b4ca0"	-0.0562
"00075b4079"	"00075b4d7f"	0.0546
"00075b4079"	"00075b520f"	-0.1574
"00075b4079"	"00075b525f"	0.1424
"00075b4079"	"00075b58f8"	-0.3138
"00075b4079"	"00075b5bcc"	0.557
"00075b4079"	"00075b5bfa"	-0.3085
"00075b4079"	"00075b6339"	0.1413
"00075b4079"	"00075b6658"	-0.1263
"00075b4079"	"00075b679a"	-0.1514
"00075b4079"	"00075b6cb7"	-0.0749
"00075b4079"	"00075b6df8"	-0.0527
"00075b4079"	"00075b6ff6"	0.0991
"00075b4079"	"00075b70ee"	-0.1096
"00075b4079"	"00075b7157"	0.0867
"00075b4079"	"00075b7225"	-0.0781

"00075b4079"	"00075b7c89"	−0.0378
"00075b4079"	"00075b9048"	−0.2244
"00075b4079"	"00075d0801"	0.3233
"00075b4079"	"00075d1820"	−0.0661
"00075b4079"	"00075d1f3d"	−0.0152
"00075b4079"	"00075d2329"	−0.2563
"00075b4079"	"00075d2b9b"	0.0028
"00075b4079"	"00075d3941"	−0.3846
"00075b4079"	"00075d3e96"	−0.0874
"00075b4079"	"00075d4864"	−0.2078
"00075b4079"	"00075d5961"	−0.1967
"00075b4079"	"00075d5a63"	−0.0083
"00075b4079"	"00075d6150"	−0.2197
"00075b4079"	"00075d67d0"	−0.0249
"00075b4079"	"00075d67e2"	−0.1367
"00075b4079"	"00075d73fc"	−0.3487
"00075b4079"	"00075d7729"	−0.1708
"00075b4079"	"00075d778c"	−0.0809
"00075b4079"	"00075d7b9e"	−0.0036
"00075b4079"	"00075d7c8f"	0.0294
"00075b4079"	"00075d804d"	−0.0418
"00075b4079"	"00075d819f"	−0.2253
"00075b4079"	"00075d8601"	0.0034
"00075b4079"	"00075d8c6a"	−0.0023
"00075b4079"	"00075dfedc"	0.1204
"00075b4079"	"00075e05f2"	−0.1823
"00075b4079"	"00075e0837"	−0.15
"00075b4079"	"00075e092e"	0.0278
"00075b4079"	"00075e0965"	−0.1888
"00075b4079"	"00075e0bc8"	−0.0951
"00075b4079"	"00075e0fbb"	−0.0583
"00075b4150"	"00075b4194"	−0.0883
"00075b4150"	"00075b42d5"	−0.1662
"00075b4150"	"00075b4424"	0.1787
"00075b4150"	"00075b4470"	−0.0134
"00075b4150"	"00075b47ed"	−0.379
"00075b4150"	"00075b4850"	−0.1144
"00075b4150"	"00075b4ca0"	−0.1592
"00075b4150"	"00075b4d7f"	0.105
"00075b4150"	"00075b520f"	−0.0621
"00075b4150"	"00075b525f"	−0.2956
"00075b4150"	"00075b58f8"	0.3347
"00075b4150"	"00075b5bcc"	−0.0071
"00075b4150"	"00075b5bfa"	−0.2227
"00075b4150"	"00075b6339"	0.1745
"00075b4150"	"00075b6658"	−0.5149
"00075b4150"	"00075b679a"	0.0306
"00075b4150"	"00075b6cb7"	−0.1748
"00075b4150"	"00075b6df8"	−0.1094
"00075b4150"	"00075b6ff6"	0.1892

"00075b4150"	"00075b70ee"	0.2319
"00075b4150"	"00075b7157"	-0.1513
"00075b4150"	"00075b7225"	-0.0292
"00075b4150"	"00075b7c89"	-0.0166
"00075b4150"	"00075b9048"	0.2907
"00075b4150"	"00075d0801"	-0.0178
"00075b4150"	"00075d1820"	-0.1475
"00075b4150"	"00075d1f3d"	-0.0412
"00075b4150"	"00075d2329"	-0.1028
"00075b4150"	"00075d2b9b"	0.0799
"00075b4150"	"00075d3941"	-0.2758
"00075b4150"	"00075d3e96"	0.0473
"00075b4150"	"00075d4864"	-0.0077
"00075b4150"	"00075d5961"	0.024
"00075b4150"	"00075d5a63"	0.1175
"00075b4150"	"00075d6150"	-0.3268
"00075b4150"	"00075d67d0"	-0.4077
"00075b4150"	"00075d67e2"	-0.0779
"00075b4150"	"00075d73fc"	-0.2011
"00075b4150"	"00075d7729"	-0.1536
"00075b4150"	"00075d778c"	-0.4255
"00075b4150"	"00075d7b9e"	-0.2707
"00075b4150"	"00075d7c8f"	-0.2211
"00075b4150"	"00075d804d"	-0.038
"00075b4150"	"00075d819f"	-0.3172
"00075b4150"	"00075d8601"	-0.3159
"00075b4150"	"00075d8c6a"	-0.06
"00075b4150"	"00075dfedc"	-0.0421
"00075b4150"	"00075e05f2"	0.0685
"00075b4150"	"00075e0837"	-0.0663
"00075b4150"	"00075e092e"	-0.3749
"00075b4150"	"00075e0965"	0.5813
"00075b4150"	"00075e0bc8"	0.2346
"00075b4150"	"00075e0fbb"	-0.3712
"00075b4194"	"00075b42d5"	0.1702
"00075b4194"	"00075b4424"	-0.2671
"00075b4194"	"00075b4470"	0.3143
"00075b4194"	"00075b47ed"	0.3525
"00075b4194"	"00075b4850"	0.3297
"00075b4194"	"00075b4ca0"	0.1902
"00075b4194"	"00075b4d7f"	0.1561
"00075b4194"	"00075b520f"	-0.0659
"00075b4194"	"00075b525f"	0.0306
"00075b4194"	"00075b58f8"	-0.1529
"00075b4194"	"00075b5bcc"	0.2001
"00075b4194"	"00075b5bfa"	-0.0973
"00075b4194"	"00075b6339"	0.1804
"00075b4194"	"00075b6658"	-0.2973
"00075b4194"	"00075b679a"	-0.0039
"00075b4194"	"00075b6cb7"	0.0646

"00075b4194"	"00075b6df8"	−0.1436
"00075b4194"	"00075b6ff6"	0.1832
"00075b4194"	"00075b70ee"	−0.0497
"00075b4194"	"00075b7157"	0.1454
"00075b4194"	"00075b7225"	0.1083
"00075b4194"	"00075b7c89"	0.0634
"00075b4194"	"00075b9048"	0.1876
"00075b4194"	"00075d0801"	0.3031
"00075b4194"	"00075d1820"	−0.1099
"00075b4194"	"00075d1f3d"	0.3194
"00075b4194"	"00075d2329"	0.1598
"00075b4194"	"00075d2b9b"	0.2404
"00075b4194"	"00075d3941"	−0.0223
"00075b4194"	"00075d3e96"	0.0805
"00075b4194"	"00075d4864"	−0.1173
"00075b4194"	"00075d5961"	0.0575
"00075b4194"	"00075d5a63"	0.2872
"00075b4194"	"00075d6150"	0.0642
"00075b4194"	"00075d67d0"	−0.0567
"00075b4194"	"00075d67e2"	−0.0076
"00075b4194"	"00075d73fc"	−0.0737
"00075b4194"	"00075d7729"	−0.2592
"00075b4194"	"00075d778c"	−0.2626
"00075b4194"	"00075d7b9e"	−0.0082
"00075b4194"	"00075d7c8f"	0.289
"00075b4194"	"00075d804d"	0.2847
"00075b4194"	"00075d819f"	0.2702
"00075b4194"	"00075d8601"	0.0641
"00075b4194"	"00075d8c6a"	0.1284
"00075b4194"	"00075dfedc"	0.2121
"00075b4194"	"00075e05f2"	0.1031
"00075b4194"	"00075e0837"	0.1032
"00075b4194"	"00075e092e"	0.0395
"00075b4194"	"00075e0965"	−0.2297
"00075b4194"	"00075e0bc8"	0.2662
"00075b4194"	"00075e0fbb"	−0.102
"00075b42d5"	"00075b4424"	0.054
"00075b42d5"	"00075b4470"	0.2458
"00075b42d5"	"00075b47ed"	0.0382
"00075b42d5"	"00075b4850"	0.2012
"00075b42d5"	"00075b4ca0"	−0.2319
"00075b42d5"	"00075b4d7f"	0.2266
"00075b42d5"	"00075b520f"	−0.1379
"00075b42d5"	"00075b525f"	0.0252
"00075b42d5"	"00075b58f8"	−0.0443
"00075b42d5"	"00075b5bcc"	0.0306
"00075b42d5"	"00075b5bfa"	−0.0909
"00075b42d5"	"00075b6339"	0.0023
"00075b42d5"	"00075b6658"	−0.1391
"00075b42d5"	"00075b679a"	−0.0283

"00075b42d5"	"00075b6cb7"	0.0195
"00075b42d5"	"00075b6df8"	0.0983
"00075b42d5"	"00075b6ff6"	0.1963
"00075b42d5"	"00075b70ee"	0.0305
"00075b42d5"	"00075b7157"	0.0435
"00075b42d5"	"00075b7225"	0.2126
"00075b42d5"	"00075b7c89"	-0.0607
"00075b42d5"	"00075b9048"	0.0857
"00075b42d5"	"00075d0801"	0.0117
"00075b42d5"	"00075d1820"	-0.0983
"00075b42d5"	"00075d1f3d"	0.1983
"00075b42d5"	"00075d2329"	0.2708
"00075b42d5"	"00075d2b9b"	0.0119
"00075b42d5"	"00075d3941"	-0.1373
"00075b42d5"	"00075d3e96"	-0.108
"00075b42d5"	"00075d4864"	0.3929
"00075b42d5"	"00075d5961"	0.0579
"00075b42d5"	"00075d5a63"	0.1146
"00075b42d5"	"00075d6150"	0.0406
"00075b42d5"	"00075d67d0"	-0.3292
"00075b42d5"	"00075d67e2"	-0.017
"00075b42d5"	"00075d73fc"	0.0585
"00075b42d5"	"00075d7729"	-0.3636
"00075b42d5"	"00075d778c"	-0.0832
"00075b42d5"	"00075d7b9e"	-0.0557
"00075b42d5"	"00075d7c8f"	0.1006
"00075b42d5"	"00075d804d"	0.0963
"00075b42d5"	"00075d819f"	-0.0643
"00075b42d5"	"00075d8601"	-0.0506
"00075b42d5"	"00075d8c6a"	0.1923
"00075b42d5"	"00075dfedc"	0.0881
"00075b42d5"	"00075e05f2"	0.2195
"00075b42d5"	"00075e0837"	0.1494
"00075b42d5"	"00075e092e"	0.1231
"00075b42d5"	"00075e0965"	-0.066
"00075b42d5"	"00075e0bc8"	0.1305
"00075b42d5"	"00075e0fbb"	0.1788
"00075b4424"	"00075b4470"	-0.0841
"00075b4424"	"00075b47ed"	-0.2808
"00075b4424"	"00075b4850"	-0.0022
"00075b4424"	"00075b4ca0"	-0.2675
"00075b4424"	"00075b4d7f"	-0.0173
"00075b4424"	"00075b520f"	-0.0567
"00075b4424"	"00075b525f"	-0.0367
"00075b4424"	"00075b58f8"	-0.0594
"00075b4424"	"00075b5bcc"	-0.152
"00075b4424"	"00075b5bfa"	-0.2507
"00075b4424"	"00075b6339"	-0.0588
"00075b4424"	"00075b6658"	0.123
"00075b4424"	"00075b679a"	-0.1425

"00075b4424"	"00075b6cb7"	-0.1689
"00075b4424"	"00075b6df8"	-0.0153
"00075b4424"	"00075b6ff6"	0.0719
"00075b4424"	"00075b70ee"	-0.1007
"00075b4424"	"00075b7157"	-0.1432
"00075b4424"	"00075b7225"	-0.1494
"00075b4424"	"00075b7c89"	-0.1123
"00075b4424"	"00075b9048"	0.0393
"00075b4424"	"00075d0801"	-0.2584
"00075b4424"	"00075d1820"	-0.0834
"00075b4424"	"00075d1f3d"	-0.1134
"00075b4424"	"00075d2329"	-0.0851
"00075b4424"	"00075d2b9b"	-0.0439
"00075b4424"	"00075d3941"	0.1779
"00075b4424"	"00075d3e96"	0.1726
"00075b4424"	"00075d4864"	0.0093
"00075b4424"	"00075d5961"	-0.1037
"00075b4424"	"00075d5a63"	0.1671
"00075b4424"	"00075d6150"	-0.0442
"00075b4424"	"00075d67d0"	-0.3966
"00075b4424"	"00075d67e2"	-0.1401
"00075b4424"	"00075d73fc"	-0.0018
"00075b4424"	"00075d7729"	-0.0367
"00075b4424"	"00075d778c"	-0.0162
"00075b4424"	"00075d7b9e"	-0.0285
"00075b4424"	"00075d7c8f"	-0.3127
"00075b4424"	"00075d804d"	-0.1957
"00075b4424"	"00075d819f"	-0.3904
"00075b4424"	"00075d8601"	-0.0443
"00075b4424"	"00075d8c6a"	-0.1006
"00075b4424"	"00075dfedc"	0.11
"00075b4424"	"00075e05f2"	0.0737
"00075b4424"	"00075e0837"	-0.0773
"00075b4424"	"00075e092e"	-0.3381
"00075b4424"	"00075e0965"	0.0329
"00075b4424"	"00075e0bc8"	0.0458
"00075b4424"	"00075e0fbb"	-0.3552
"00075b4470"	"00075b47ed"	0.0935
"00075b4470"	"00075b4850"	0.7462
"00075b4470"	"00075b4ca0"	-0.0293
"00075b4470"	"00075b4d7f"	0.242
"00075b4470"	"00075b520f"	-0.0568
"00075b4470"	"00075b525f"	-0.2108
"00075b4470"	"00075b58f8"	0.2788
"00075b4470"	"00075b5bcc"	0.1015
"00075b4470"	"00075b5bfa"	-0.2676
"00075b4470"	"00075b6339"	0.0963
"00075b4470"	"00075b6658"	-0.163
"00075b4470"	"00075b679a"	0.088
"00075b4470"	"00075b6cb7"	0.41

"00075b4470"	"00075b6df8"	-0.1317
"00075b4470"	"00075b6ff6"	0.0992
"00075b4470"	"00075b70ee"	0.0871
"00075b4470"	"00075b7157"	0.0841
"00075b4470"	"00075b7225"	0.0715
"00075b4470"	"00075b7c89"	0.1653
"00075b4470"	"00075b9048"	0.069
"00075b4470"	"00075d0801"	0.1594
"00075b4470"	"00075d1820"	-0.1505
"00075b4470"	"00075d1f3d"	0.3675
"00075b4470"	"00075d2329"	0.1187
"00075b4470"	"00075d2b9b"	0.1043
"00075b4470"	"00075d3941"	0.202
"00075b4470"	"00075d3e96"	0.2063
"00075b4470"	"00075d4864"	0.1761
"00075b4470"	"00075d5961"	0.0252
"00075b4470"	"00075d5a63"	0.2066
"00075b4470"	"00075d6150"	-0.1362
"00075b4470"	"00075d67d0"	-0.2776
"00075b4470"	"00075d67e2"	0.0981
"00075b4470"	"00075d73fc"	0.1645
"00075b4470"	"00075d7729"	-0.2895
"00075b4470"	"00075d778c"	-0.4042
"00075b4470"	"00075d7b9e"	0.0087
"00075b4470"	"00075d7c8f"	0.096
"00075b4470"	"00075d804d"	0.3277
"00075b4470"	"00075d819f"	0.3423
"00075b4470"	"00075d8601"	-0.0226
"00075b4470"	"00075d8c6a"	-0.0459
"00075b4470"	"00075dfedc"	0.0511
"00075b4470"	"00075e05f2"	0.0299
"00075b4470"	"00075e0837"	0.3252
"00075b4470"	"00075e092e"	0.1344
"00075b4470"	"00075e0965"	-0.0052
"00075b4470"	"00075e0bc8"	0.4895
"00075b4470"	"00075e0fbb"	0.0711
"00075b47ed"	"00075b4850"	0.0031
"00075b47ed"	"00075b4ca0"	0.054
"00075b47ed"	"00075b4d7f"	0.1128
"00075b47ed"	"00075b520f"	0.1029
"00075b47ed"	"00075b525f"	-0.0665
"00075b47ed"	"00075b58f8"	-0.3471
"00075b47ed"	"00075b5bcc"	-0.0371
"00075b47ed"	"00075b5bfa"	-0.0911
"00075b47ed"	"00075b6339"	0.0494
"00075b47ed"	"00075b6658"	-0.1035
"00075b47ed"	"00075b679a"	0.0546
"00075b47ed"	"00075b6cb7"	-0.2578
"00075b47ed"	"00075b6df8"	0.1256
"00075b47ed"	"00075b6ff6"	-0.1891

"00075b47ed"	"00075b70ee"	−0.2864
"00075b47ed"	"00075b7157"	0.1211
"00075b47ed"	"00075b7225"	−0.0481
"00075b47ed"	"00075b7c89"	0.089
"00075b47ed"	"00075b9048"	−0.1314
"00075b47ed"	"00075d0801"	0.3605
"00075b47ed"	"00075d1820"	0.1158
"00075b47ed"	"00075d1f3d"	−0.222
"00075b47ed"	"00075d2329"	0.5748
"00075b47ed"	"00075d2b9b"	−0.0789
"00075b47ed"	"00075d3941"	0.0937
"00075b47ed"	"00075d3e96"	0.0484
"00075b47ed"	"00075d4864"	0.0725
"00075b47ed"	"00075d5961"	−0.0945
"00075b47ed"	"00075d5a63"	0.2335
"00075b47ed"	"00075d6150"	−0.0312
"00075b47ed"	"00075d67d0"	−0.0267
"00075b47ed"	"00075d67e2"	−0.2129
"00075b47ed"	"00075d73fc"	0.05
"00075b47ed"	"00075d7729"	−0.2863
"00075b47ed"	"00075d778c"	−0.1195
"00075b47ed"	"00075d7b9e"	−0.0237
"00075b47ed"	"00075d7c8f"	0.1045
"00075b47ed"	"00075d804d"	−0.0046
"00075b47ed"	"00075d819f"	0.0083
"00075b47ed"	"00075d8601"	0.173
"00075b47ed"	"00075d8c6a"	0.1973
"00075b47ed"	"00075dfedc"	0.1579
"00075b47ed"	"00075e05f2"	−0.1638
"00075b47ed"	"00075e0837"	0.0476
"00075b47ed"	"00075e092e"	−0.0039
"00075b47ed"	"00075e0965"	−0.5512
"00075b47ed"	"00075e0bc8"	−0.0194
"00075b47ed"	"00075e0fbb"	−0.0063
"00075b4850"	"00075b4ca0"	0.1564
"00075b4850"	"00075b4d7f"	0.093
"00075b4850"	"00075b520f"	−0.0046
"00075b4850"	"00075b525f"	−0.0766
"00075b4850"	"00075b58f8"	0.1429
"00075b4850"	"00075b5bcc"	0.1665
"00075b4850"	"00075b5bfa"	−0.1678
"00075b4850"	"00075b6339"	0.1534
"00075b4850"	"00075b6658"	−0.1523
"00075b4850"	"00075b679a"	0.1174
"00075b4850"	"00075b6cb7"	0.5283
"00075b4850"	"00075b6df8"	−0.1832
"00075b4850"	"00075b6ff6"	0.287
"00075b4850"	"00075b70ee"	0.1502
"00075b4850"	"00075b7157"	−0.0028
"00075b4850"	"00075b7225"	0.0471

"00075b4850"	"00075b7c89"	0.0852
"00075b4850"	"00075b9048"	0.2721
"00075b4850"	"00075d0801"	0.1269
"00075b4850"	"00075d1820"	−0.1876
"00075b4850"	"00075d1f3d"	0.6062
"00075b4850"	"00075d2329"	−0.0379
"00075b4850"	"00075d2b9b"	0.2036
"00075b4850"	"00075d3941"	0.1488
"00075b4850"	"00075d3e96"	0.1856
"00075b4850"	"00075d4864"	−0.1235
"00075b4850"	"00075d5961"	−0.1416
"00075b4850"	"00075d5a63"	0.1853
"00075b4850"	"00075d6150"	−0.0034
"00075b4850"	"00075d67d0"	−0.0933
"00075b4850"	"00075d67e2"	−0.0063
"00075b4850"	"00075d73fc"	0.0541
"00075b4850"	"00075d7729"	−0.1475
"00075b4850"	"00075d778c"	−0.2934
"00075b4850"	"00075d7b9e"	−0.0612
"00075b4850"	"00075d7c8f"	0.1178
"00075b4850"	"00075d804d"	0.4285
"00075b4850"	"00075d819f"	0.3594
"00075b4850"	"00075d8601"	−0.1074
"00075b4850"	"00075d8c6a"	−0.1171
"00075b4850"	"00075dfedc"	0.1385
"00075b4850"	"00075e05f2"	0.1196
"00075b4850"	"00075e0837"	0.447
"00075b4850"	"00075e092e"	0.1159
"00075b4850"	"00075e0965"	−0.0891
"00075b4850"	"00075e0bc8"	0.5743
"00075b4850"	"00075e0fbb"	−0.0532
"00075b4ca0"	"00075b4d7f"	0.0078
"00075b4ca0"	"00075b520f"	0.0761
"00075b4ca0"	"00075b525f"	0.0318
"00075b4ca0"	"00075b58f8"	−0.2313
"00075b4ca0"	"00075b5bcc"	0.0825
"00075b4ca0"	"00075b5bfa"	−0.2788
"00075b4ca0"	"00075b6339"	0.2552
"00075b4ca0"	"00075b6658"	−0.1488
"00075b4ca0"	"00075b679a"	−0.1277
"00075b4ca0"	"00075b6cb7"	0.0017
"00075b4ca0"	"00075b6df8"	−0.1694
"00075b4ca0"	"00075b6ff6"	−0.006
"00075b4ca0"	"00075b70ee"	−0.0894
"00075b4ca0"	"00075b7157"	0.0495
"00075b4ca0"	"00075b7225"	−0.0303
"00075b4ca0"	"00075b7c89"	0.3135
"00075b4ca0"	"00075b9048"	0.1652
"00075b4ca0"	"00075d0801"	−0.0013
"00075b4ca0"	"00075d1820"	−0.1197

"00075b4ca0"	"00075d1f3d"	0.1162
"00075b4ca0"	"00075d2329"	0.04
"00075b4ca0"	"00075d2b9b"	0.112
"00075b4ca0"	"00075d3941"	-0.0779
"00075b4ca0"	"00075d3e96"	-0.014
"00075b4ca0"	"00075d4864"	-0.2651
"00075b4ca0"	"00075d5961"	-0.2178
"00075b4ca0"	"00075d5a63"	-7e-04
"00075b4ca0"	"00075d6150"	-0.1796
"00075b4ca0"	"00075d67d0"	-0.1258
"00075b4ca0"	"00075d67e2"	-0.0487
"00075b4ca0"	"00075d73fc"	-0.1965
"00075b4ca0"	"00075d7729"	0.2283
"00075b4ca0"	"00075d778c"	-0.3145
"00075b4ca0"	"00075d7b9e"	-0.1486
"00075b4ca0"	"00075d7c8f"	0.2283
"00075b4ca0"	"00075d804d"	0.0562
"00075b4ca0"	"00075d819f"	0.1077
"00075b4ca0"	"00075d8601"	-0.1961
"00075b4ca0"	"00075d8c6a"	-0.1381
"00075b4ca0"	"00075dfedc"	0.1151
"00075b4ca0"	"00075e05f2"	-0.0325
"00075b4ca0"	"00075e0837"	0.1285
"00075b4ca0"	"00075e092e"	0.0239
"00075b4ca0"	"00075e0965"	-0.483
"00075b4ca0"	"00075e0bc8"	0.1891
"00075b4ca0"	"00075e0fbb"	-0.1494
"00075b4d7f"	"00075b520f"	0.1444
"00075b4d7f"	"00075b525f"	0.036
"00075b4d7f"	"00075b58f8"	0.0361
"00075b4d7f"	"00075b5bcc"	0.2531
"00075b4d7f"	"00075b5bfa"	-0.0506
"00075b4d7f"	"00075b6339"	0.3183
"00075b4d7f"	"00075b6658"	0.1334
"00075b4d7f"	"00075b679a"	0.3308
"00075b4d7f"	"00075b6cb7"	-0.0985
"00075b4d7f"	"00075b6df8"	0.6424
"00075b4d7f"	"00075b6ff6"	0.3076
"00075b4d7f"	"00075b70ee"	0.1021
"00075b4d7f"	"00075b7157"	0.3067
"00075b4d7f"	"00075b7225"	0.2722
"00075b4d7f"	"00075b7c89"	0.3396
"00075b4d7f"	"00075b9048"	0.2173
"00075b4d7f"	"00075d0801"	0.1853
"00075b4d7f"	"00075d1820"	-0.0187
"00075b4d7f"	"00075d1f3d"	0.0542
"00075b4d7f"	"00075d2329"	0.0556
"00075b4d7f"	"00075d2b9b"	-0.1264
"00075b4d7f"	"00075d3941"	-0.0394
"00075b4d7f"	"00075d3e96"	0.0375

"00075b4d7f"	"00075d4864"	0.0807
"00075b4d7f"	"00075d5961"	0.3728
"00075b4d7f"	"00075d5a63"	0.2284
"00075b4d7f"	"00075d6150"	0.0172
"00075b4d7f"	"00075d67d0"	−0.1788
"00075b4d7f"	"00075d67e2"	0.341
"00075b4d7f"	"00075d73fc"	0.3147
"00075b4d7f"	"00075d7729"	0.2172
"00075b4d7f"	"00075d778c"	0.04
"00075b4d7f"	"00075d7b9e"	0.2203
"00075b4d7f"	"00075d7c8f"	0.3846
"00075b4d7f"	"00075d804d"	0.0816
"00075b4d7f"	"00075d819f"	−0.164
"00075b4d7f"	"00075d8601"	0.1271
"00075b4d7f"	"00075d8c6a"	0.4368
"00075b4d7f"	"00075dfedc"	0.4523
"00075b4d7f"	"00075e05f2"	0.1096
"00075b4d7f"	"00075e0837"	−0.1048
"00075b4d7f"	"00075e092e"	0.0627
"00075b4d7f"	"00075e0965"	−0.0312
"00075b4d7f"	"00075e0bc8"	0.2086
"00075b4d7f"	"00075e0fbb"	0.18
"00075b520f"	"00075b525f"	−0.2123
"00075b520f"	"00075b58f8"	−0.0827
"00075b520f"	"00075b5bcc"	−0.0109
"00075b520f"	"00075b5bfa"	−0.2262
"00075b520f"	"00075b6339"	0.0395
"00075b520f"	"00075b6658"	0.1171
"00075b520f"	"00075b679a"	0.2468
"00075b520f"	"00075b6cb7"	−0.4325
"00075b520f"	"00075b6df8"	−0.024
"00075b520f"	"00075b6ff6"	−0.1717
"00075b520f"	"00075b70ee"	−0.2013
"00075b520f"	"00075b7157"	0.0549
"00075b520f"	"00075b7225"	0.02
"00075b520f"	"00075b7c89"	−0.0259
"00075b520f"	"00075b9048"	0.0117
"00075b520f"	"00075d0801"	0.2501
"00075b520f"	"00075d1820"	0.0973
"00075b520f"	"00075d1f3d"	−0.1851
"00075b520f"	"00075d2329"	0.1093
"00075b520f"	"00075d2b9b"	−0.3086
"00075b520f"	"00075d3941"	0.1215
"00075b520f"	"00075d3e96"	−0.0428
"00075b520f"	"00075d4864"	0.098
"00075b520f"	"00075d5961"	0.1552
"00075b520f"	"00075d5a63"	−0.1067
"00075b520f"	"00075d6150"	0.1948
"00075b520f"	"00075d67d0"	−0.2351
"00075b520f"	"00075d67e2"	−0.0684

"00075b520f"	"00075d73fc"	0.0084
"00075b520f"	"00075d7729"	0.0403
"00075b520f"	"00075d778c"	−0.3188
"00075b520f"	"00075d7b9e"	−0.009
"00075b520f"	"00075d7c8f"	−0.085
"00075b520f"	"00075d804d"	0.0603
"00075b520f"	"00075d819f"	−0.0772
"00075b520f"	"00075d8601"	0.2644
"00075b520f"	"00075d8c6a"	−0.0278
"00075b520f"	"00075dfedc"	0.061
"00075b520f"	"00075e05f2"	−0.1587
"00075b520f"	"00075e0837"	−0.0885
"00075b520f"	"00075e092e"	−0.2537
"00075b520f"	"00075e0965"	−0.3101
"00075b520f"	"00075e0bc8"	0.0918
"00075b520f"	"00075e0fbb"	−0.4894
"00075b525f"	"00075b58f8"	−0.6081
"00075b525f"	"00075b5bcc"	0.2307
"00075b525f"	"00075b5bfa"	−0.1077
"00075b525f"	"00075b6339"	0.2534
"00075b525f"	"00075b6658"	0.1777
"00075b525f"	"00075b679a"	0.04
"00075b525f"	"00075b6cb7"	−0.3468
"00075b525f"	"00075b6df8"	−0.0438
"00075b525f"	"00075b6ff6"	0.0946
"00075b525f"	"00075b70ee"	−0.201
"00075b525f"	"00075b7157"	0.2039
"00075b525f"	"00075b7225"	0.057
"00075b525f"	"00075b7c89"	−0.2576
"00075b525f"	"00075b9048"	−0.1604
"00075b525f"	"00075d0801"	0.069
"00075b525f"	"00075d1820"	0.1207
"00075b525f"	"00075d1f3d"	−0.123
"00075b525f"	"00075d2329"	−0.2848
"00075b525f"	"00075d2b9b"	−0.0889
"00075b525f"	"00075d3941"	−0.3188
"00075b525f"	"00075d3e96"	0.1001
"00075b525f"	"00075d4864"	−0.0507
"00075b525f"	"00075d5961"	−0.3163
"00075b525f"	"00075d5a63"	−0.02
"00075b525f"	"00075d6150"	−0.0558
"00075b525f"	"00075d67d0"	0.1162
"00075b525f"	"00075d67e2"	−0.0486
"00075b525f"	"00075d73fc"	−0.3168
"00075b525f"	"00075d7729"	0.0516
"00075b525f"	"00075d778c"	0.6112
"00075b525f"	"00075d7b9e"	0.5153
"00075b525f"	"00075d7c8f"	0.1202
"00075b525f"	"00075d804d"	−0.1448
"00075b525f"	"00075d819f"	−0.1363

"00075b525f"	"00075d8601"	−0.1619
"00075b525f"	"00075d8c6a"	−0.0743
"00075b525f"	"00075dfedc"	0.0691
"00075b525f"	"00075e05f2"	−0.0648
"00075b525f"	"00075e0837"	−0.0183
"00075b525f"	"00075e092e"	0.2022
"00075b525f"	"00075e0965"	−0.4627
"00075b525f"	"00075e0bc8"	−0.1986
"00075b525f"	"00075e0fbb"	0.1963
"00075b58f8"	"00075b5bcc"	−0.3193
"00075b58f8"	"00075b5bfa"	−0.3238
"00075b58f8"	"00075b6339"	−0.0961
"00075b58f8"	"00075b6658"	−0.455
"00075b58f8"	"00075b679a"	−0.1442
"00075b58f8"	"00075b6cb7"	0.0382
"00075b58f8"	"00075b6df8"	−0.3211
"00075b58f8"	"00075b6ff6"	−0.1341
"00075b58f8"	"00075b70ee"	0.041
"00075b58f8"	"00075b7157"	−0.018
"00075b58f8"	"00075b7225"	−0.3542
"00075b58f8"	"00075b7c89"	−0.1837
"00075b58f8"	"00075b9048"	−0.0357
"00075b58f8"	"00075d0801"	0.1072
"00075b58f8"	"00075d1820"	−0.2018
"00075b58f8"	"00075d1f3d"	0.0043
"00075b58f8"	"00075d2329"	−0.0451
"00075b58f8"	"00075d2b9b"	−0.1972
"00075b58f8"	"00075d3941"	−0.3739
"00075b58f8"	"00075d3e96"	−0.0074
"00075b58f8"	"00075d4864"	−0.0477
"00075b58f8"	"00075d5961"	0.0737
"00075b58f8"	"00075d5a63"	−0.0456
"00075b58f8"	"00075d6150"	−0.3289
"00075b58f8"	"00075d67d0"	−0.6936
"00075b58f8"	"00075d67e2"	−0.2344
"00075b58f8"	"00075d73fc"	−0.2387
"00075b58f8"	"00075d7729"	−0.2272
"00075b58f8"	"00075d778c"	−0.5352
"00075b58f8"	"00075d7b9e"	−0.2159
"00075b58f8"	"00075d7c8f"	−0.2691
"00075b58f8"	"00075d804d"	0.1661
"00075b58f8"	"00075d819f"	−0.1203
"00075b58f8"	"00075d8601"	−0.1522
"00075b58f8"	"00075d8c6a"	−0.121
"00075b58f8"	"00075dfedc"	−0.2098
"00075b58f8"	"00075e05f2"	−0.0626
"00075b58f8"	"00075e0837"	0.0257
"00075b58f8"	"00075e092e"	−0.1208
"00075b58f8"	"00075e0965"	0.4589
"00075b58f8"	"00075e0bc8"	0.242

"00075b58f8"	"00075e0fbb"	-0.0827
"00075b5bcc"	"00075b5bfa"	0.1472
"00075b5bcc"	"00075b6339"	0.2915
"00075b5bcc"	"00075b6658"	0.0412
"00075b5bcc"	"00075b679a"	0.2759
"00075b5bcc"	"00075b6cb7"	0.024
"00075b5bcc"	"00075b6df8"	0.1854
"00075b5bcc"	"00075b6ff6"	0.4353
"00075b5bcc"	"00075b70ee"	-0.0649
"00075b5bcc"	"00075b7157"	0.3107
"00075b5bcc"	"00075b7225"	0.2437
"00075b5bcc"	"00075b7c89"	0.0775
"00075b5bcc"	"00075b9048"	0.1854
"00075b5bcc"	"00075d0801"	0.0378
"00075b5bcc"	"00075d1820"	0.1361
"00075b5bcc"	"00075d1f3d"	0.2338
"00075b5bcc"	"00075d2329"	3e-04
"00075b5bcc"	"00075d2b9b"	0.2027
"00075b5bcc"	"00075d3941"	-0.0589
"00075b5bcc"	"00075d3e96"	0.2395
"00075b5bcc"	"00075d4864"	-0.1203
"00075b5bcc"	"00075d5961"	0.0017
"00075b5bcc"	"00075d5a63"	0.2352
"00075b5bcc"	"00075d6150"	0.1354
"00075b5bcc"	"00075d67d0"	-0.0419
"00075b5bcc"	"00075d67e2"	-0.0386
"00075b5bcc"	"00075d73fc"	0.0268
"00075b5bcc"	"00075d7729"	0.2166
"00075b5bcc"	"00075d778c"	0.0977
"00075b5bcc"	"00075d7b9e"	0.1708
"00075b5bcc"	"00075d7c8f"	0.1725
"00075b5bcc"	"00075d804d"	0.1996
"00075b5bcc"	"00075d819f"	-0.0851
"00075b5bcc"	"00075d8601"	0.1268
"00075b5bcc"	"00075d8c6a"	0.1079
"00075b5bcc"	"00075dfedc"	0.3375
"00075b5bcc"	"00075e05f2"	-0.1721
"00075b5bcc"	"00075e0837"	-0.0033
"00075b5bcc"	"00075e092e"	0.0794
"00075b5bcc"	"00075e0965"	-0.0764
"00075b5bcc"	"00075e0bc8"	0.0509
"00075b5bcc"	"00075e0fbb"	4e-04
"00075b5bfa"	"00075b6339"	-0.2043
"00075b5bfa"	"00075b6658"	-0.2477
"00075b5bfa"	"00075b679a"	-0.1312
"00075b5bfa"	"00075b6cb7"	-0.4112
"00075b5bfa"	"00075b6df8"	0.1504
"00075b5bfa"	"00075b6ff6"	0.0802
"00075b5bfa"	"00075b70ee"	-0.1969
"00075b5bfa"	"00075b7157"	0.2938

"00075b5bfa"	"00075b7225"	-0.1978
"00075b5bfa"	"00075b7c89"	-0.2629
"00075b5bfa"	"00075b9048"	0.163
"00075b5bfa"	"00075d0801"	-0.0716
"00075b5bfa"	"00075d1820"	-0.1746
"00075b5bfa"	"00075d1f3d"	-0.1846
"00075b5bfa"	"00075d2329"	0.0923
"00075b5bfa"	"00075d2b9b"	-0.139
"00075b5bfa"	"00075d3941"	-0.1999
"00075b5bfa"	"00075d3e96"	0.1496
"00075b5bfa"	"00075d4864"	-0.3357
"00075b5bfa"	"00075d5961"	-0.2527
"00075b5bfa"	"00075d5a63"	-0.067
"00075b5bfa"	"00075d6150"	-0.0699
"00075b5bfa"	"00075d67d0"	-0.2207
"00075b5bfa"	"00075d67e2"	-0.4065
"00075b5bfa"	"00075d73fc"	-0.2551
"00075b5bfa"	"00075d7729"	-7e-04
"00075b5bfa"	"00075d778c"	-0.0604
"00075b5bfa"	"00075d7b9e"	0.1076
"00075b5bfa"	"00075d7c8f"	-0.3809
"00075b5bfa"	"00075d804d"	0.0166
"00075b5bfa"	"00075d819f"	-0.2256
"00075b5bfa"	"00075d8601"	0.0481
"00075b5bfa"	"00075d8c6a"	0.0737
"00075b5bfa"	"00075dfedc"	-0.1127
"00075b5bfa"	"00075e05f2"	-0.0136
"00075b5bfa"	"00075e0837"	-0.0125
"00075b5bfa"	"00075e092e"	-0.4005
"00075b5bfa"	"00075e0965"	-0.3172
"00075b5bfa"	"00075e0bc8"	-0.0382
"00075b5bfa"	"00075e0fbb"	-0.3409
"00075b6339"	"00075b6658"	-0.0169
"00075b6339"	"00075b679a"	0.2507
"00075b6339"	"00075b6cb7"	0.0338
"00075b6339"	"00075b6df8"	0.0529
"00075b6339"	"00075b6ff6"	0.3986
"00075b6339"	"00075b70ee"	0.1133
"00075b6339"	"00075b7157"	0.138
"00075b6339"	"00075b7225"	0.187
"00075b6339"	"00075b7c89"	0.3753
"00075b6339"	"00075b9048"	0.1823
"00075b6339"	"00075d0801"	0.0735
"00075b6339"	"00075d1820"	0.0173
"00075b6339"	"00075d1f3d"	0.1125
"00075b6339"	"00075d2329"	0.035
"00075b6339"	"00075d2b9b"	0.0985
"00075b6339"	"00075d3941"	-0.1049
"00075b6339"	"00075d3e96"	-0.0256
"00075b6339"	"00075d4864"	-0.1003

"00075b6339"	"00075d5961"	0.1087
"00075b6339"	"00075d5a63"	0.3234
"00075b6339"	"00075d6150"	-0.1371
"00075b6339"	"00075d67d0"	0.2148
"00075b6339"	"00075d67e2"	0.4438
"00075b6339"	"00075d73fc"	-0.018
"00075b6339"	"00075d7729"	0.0303
"00075b6339"	"00075d778c"	0.2434
"00075b6339"	"00075d7b9e"	0.1676
"00075b6339"	"00075d7c8f"	0.5397
"00075b6339"	"00075d804d"	0.2871
"00075b6339"	"00075d819f"	-0.0883
"00075b6339"	"00075d8601"	0.0142
"00075b6339"	"00075d8c6a"	0.1953
"00075b6339"	"00075dfedc"	0.5174
"00075b6339"	"00075e05f2"	0.2262
"00075b6339"	"00075e0837"	0.0708
"00075b6339"	"00075e092e"	-0.0692
"00075b6339"	"00075e0965"	-0.0303
"00075b6339"	"00075e0bc8"	0.1904
"00075b6339"	"00075e0fbb"	-0.0555
"00075b6658"	"00075b679a"	-0.133
"00075b6658"	"00075b6cb7"	-0.3204
"00075b6658"	"00075b6df8"	0.0297
"00075b6658"	"00075b6ff6"	-0.2487
"00075b6658"	"00075b70ee"	-0.4543
"00075b6658"	"00075b7157"	-0.0585
"00075b6658"	"00075b7225"	-0.2078
"00075b6658"	"00075b7c89"	-0.0388
"00075b6658"	"00075b9048"	-0.3352
"00075b6658"	"00075d0801"	0
"00075b6658"	"00075d1820"	0.0345
"00075b6658"	"00075d1f3d"	-0.4015
"00075b6658"	"00075d2329"	-0.1671
"00075b6658"	"00075d2b9b"	-0.3748
"00075b6658"	"00075d3941"	-1e-04
"00075b6658"	"00075d3e96"	-0.0565
"00075b6658"	"00075d4864"	-0.033
"00075b6658"	"00075d5961"	0.0603
"00075b6658"	"00075d5a63"	0.0144
"00075b6658"	"00075d6150"	0
"00075b6658"	"00075d67d0"	-0.237
"00075b6658"	"00075d67e2"	-0.1646
"00075b6658"	"00075d73fc"	-0.056
"00075b6658"	"00075d7729"	0.0019
"00075b6658"	"00075d778c"	0.4006
"00075b6658"	"00075d7b9e"	0.2874
"00075b6658"	"00075d7c8f"	-0.0979
"00075b6658"	"00075d804d"	-0.246
"00075b6658"	"00075d819f"	-0.2605

"00075b6658"	"00075d8601"	0.1174
"00075b6658"	"00075d8c6a"	−0.1508
"00075b6658"	"00075dfedc"	0.152
"00075b6658"	"00075e05f2"	−0.2955
"00075b6658"	"00075e0837"	−0.2515
"00075b6658"	"00075e092e"	−0.1593
"00075b6658"	"00075e0965"	−0.6443
"00075b6658"	"00075e0bc8"	−0.0435
"00075b6658"	"00075e0fbb"	−0.3919
"00075b679a"	"00075b6cb7"	−0.2339
"00075b679a"	"00075b6df8"	0.1447
"00075b679a"	"00075b6ff6"	0.3315
"00075b679a"	"00075b70ee"	−0.021
"00075b679a"	"00075b7157"	0.0236
"00075b679a"	"00075b7225"	0.2287
"00075b679a"	"00075b7c89"	0.0446
"00075b679a"	"00075b9048"	0.2274
"00075b679a"	"00075d0801"	0.0249
"00075b679a"	"00075d1820"	0.0871
"00075b679a"	"00075d1f3d"	0.0764
"00075b679a"	"00075d2329"	−0.02
"00075b679a"	"00075d2b9b"	−0.1835
"00075b679a"	"00075d3941"	0.0288
"00075b679a"	"00075d3e96"	0.1709
"00075b679a"	"00075d4864"	−0.129
"00075b679a"	"00075d5961"	0.0423
"00075b679a"	"00075d5a63"	0.1556
"00075b679a"	"00075d6150"	0.2177
"00075b679a"	"00075d67d0"	−0.0058
"00075b679a"	"00075d67e2"	0.0664
"00075b679a"	"00075d73fc"	0.5054
"00075b679a"	"00075d7729"	0.1062
"00075b679a"	"00075d778c"	−0.1818
"00075b679a"	"00075d7b9e"	0.0107
"00075b679a"	"00075d7c8f"	0.1791
"00075b679a"	"00075d804d"	−0.0449
"00075b679a"	"00075d819f"	−0.0781
"00075b679a"	"00075d8601"	0.0619
"00075b679a"	"00075d8c6a"	−0.1159
"00075b679a"	"00075dfedc"	0.22
"00075b679a"	"00075e05f2"	−0.0529
"00075b679a"	"00075e0837"	−0.0155
"00075b679a"	"00075e092e"	0.0078
"00075b679a"	"00075e0965"	−0.2377
"00075b679a"	"00075e0bc8"	0.1055
"00075b679a"	"00075e0fbb"	−0.1437
"00075b6cb7"	"00075b6df8"	−0.3914
"00075b6cb7"	"00075b6ff6"	0.0807
"00075b6cb7"	"00075b70ee"	−0.0845
"00075b6cb7"	"00075b7157"	−0.2154

"00075b6cb7"	"00075b7225"	−0.227
"00075b6cb7"	"00075b7c89"	−0.1383
"00075b6cb7"	"00075b9048"	0.0312
"00075b6cb7"	"00075d0801"	−0.1506
"00075b6cb7"	"00075d1820"	−0.3393
"00075b6cb7"	"00075d1f3d"	0.259
"00075b6cb7"	"00075d2329"	−0.1667
"00075b6cb7"	"00075d2b9b"	0.1597
"00075b6cb7"	"00075d3941"	−0.2582
"00075b6cb7"	"00075d3e96"	−0.1033
"00075b6cb7"	"00075d4864"	−0.3914
"00075b6cb7"	"00075d5961"	−0.2064
"00075b6cb7"	"00075d5a63"	−0.003
"00075b6cb7"	"00075d6150"	−0.3196
"00075b6cb7"	"00075d67d0"	−0.1265
"00075b6cb7"	"00075d67e2"	−0.0794
"00075b6cb7"	"00075d73fc"	−0.0808
"00075b6cb7"	"00075d7729"	−0.3947
"00075b6cb7"	"00075d778c"	−0.4264
"00075b6cb7"	"00075d7b9e"	−0.267
"00075b6cb7"	"00075d7c8f"	−0.0616
"00075b6cb7"	"00075d804d"	0.168
"00075b6cb7"	"00075d819f"	0.4508
"00075b6cb7"	"00075d8601"	−0.3759
"00075b6cb7"	"00075d8c6a"	−0.2797
"00075b6cb7"	"00075dfedc"	−0.0577
"00075b6cb7"	"00075e05f2"	0.2351
"00075b6cb7"	"00075e0837"	0.361
"00075b6cb7"	"00075e092e"	−0.0025
"00075b6cb7"	"00075e0965"	−0.1503
"00075b6cb7"	"00075e0bc8"	0.3484
"00075b6cb7"	"00075e0fbb"	−0.1251
"00075b6df8"	"00075b6ff6"	0.1319
"00075b6df8"	"00075b70ee"	−0.0284
"00075b6df8"	"00075b7157"	0.324
"00075b6df8"	"00075b7225"	0.139
"00075b6df8"	"00075b7c89"	0.2295
"00075b6df8"	"00075b9048"	0.2424
"00075b6df8"	"00075d0801"	0.0322
"00075b6df8"	"00075d1820"	0.0148
"00075b6df8"	"00075d1f3d"	−0.2272
"00075b6df8"	"00075d2329"	0.1826
"00075b6df8"	"00075d2b9b"	−0.3569
"00075b6df8"	"00075d3941"	−0.149
"00075b6df8"	"00075d3e96"	−0.0028
"00075b6df8"	"00075d4864"	−0.0686
"00075b6df8"	"00075d5961"	0.2014
"00075b6df8"	"00075d5a63"	−0.0257
"00075b6df8"	"00075d6150"	−0.137
"00075b6df8"	"00075d67d0"	−0.2514

"00075b6df8"	"00075d67e2"	0.0606
"00075b6df8"	"00075d73fc"	0.3714
"00075b6df8"	"00075d7729"	0.2663
"00075b6df8"	"00075d778c"	0.0881
"00075b6df8"	"00075d7b9e"	0.1088
"00075b6df8"	"00075d7c8f"	0.1818
"00075b6df8"	"00075d804d"	-0.2312
"00075b6df8"	"00075d819f"	-0.3606
"00075b6df8"	"00075d8601"	0.1516
"00075b6df8"	"00075d8c6a"	0.6097
"00075b6df8"	"00075dfedc"	0.1483
"00075b6df8"	"00075e05f2"	-0.186
"00075b6df8"	"00075e0837"	-0.2596
"00075b6df8"	"00075e092e"	0.0711
"00075b6df8"	"00075e0965"	-0.1976
"00075b6df8"	"00075e0bc8"	-0.0227
"00075b6df8"	"00075e0fbb"	0.0984
"00075b6ff6"	"00075b70ee"	0.1344
"00075b6ff6"	"00075b7157"	0.1195
"00075b6ff6"	"00075b7225"	0.1315
"00075b6ff6"	"00075b7c89"	0.141
"00075b6ff6"	"00075b9048"	0.3812
"00075b6ff6"	"00075d0801"	-0.1082
"00075b6ff6"	"00075d1820"	-0.2826
"00075b6ff6"	"00075d1f3d"	0.3103
"00075b6ff6"	"00075d2329"	-0.0333
"00075b6ff6"	"00075d2b9b"	0.1478
"00075b6ff6"	"00075d3941"	-0.2957
"00075b6ff6"	"00075d3e96"	-0.0299
"00075b6ff6"	"00075d4864"	-0.1084
"00075b6ff6"	"00075d5961"	-0.0671
"00075b6ff6"	"00075d5a63"	0.297
"00075b6ff6"	"00075d6150"	-0.0299
"00075b6ff6"	"00075d67d0"	0.0347
"00075b6ff6"	"00075d67e2"	0.0624
"00075b6ff6"	"00075d73fc"	-0.0489
"00075b6ff6"	"00075d7729"	0.0936
"00075b6ff6"	"00075d778c"	-0.0607
"00075b6ff6"	"00075d7b9e"	0.0027
"00075b6ff6"	"00075d7c8f"	0.2917
"00075b6ff6"	"00075d804d"	0.2715
"00075b6ff6"	"00075d819f"	-0.0767
"00075b6ff6"	"00075d8601"	-0.2803
"00075b6ff6"	"00075d8c6a"	-0.0476
"00075b6ff6"	"00075dfedc"	0.2428
"00075b6ff6"	"00075e05f2"	0.0718
"00075b6ff6"	"00075e0837"	0.0212
"00075b6ff6"	"00075e092e"	0.0592
"00075b6ff6"	"00075e0965"	0.1018
"00075b6ff6"	"00075e0bc8"	0.2923

"00075b6ff6"	"00075e0fbb"	0.1804
"00075b70ee"	"00075b7157"	-0.2399
"00075b70ee"	"00075b7225"	-0.0136
"00075b70ee"	"00075b7c89"	0.005
"00075b70ee"	"00075b9048"	0.0196
"00075b70ee"	"00075d0801"	-0.1292
"00075b70ee"	"00075d1820"	-0.1505
"00075b70ee"	"00075d1f3d"	-0.0336
"00075b70ee"	"00075d2329"	-0.2607
"00075b70ee"	"00075d2b9b"	-0.1965
"00075b70ee"	"00075d3941"	-0.4322
"00075b70ee"	"00075d3e96"	-0.0453
"00075b70ee"	"00075d4864"	-0.142
"00075b70ee"	"00075d5961"	-0.0422
"00075b70ee"	"00075d5a63"	-0.0939
"00075b70ee"	"00075d6150"	-0.0921
"00075b70ee"	"00075d67d0"	-0.3712
"00075b70ee"	"00075d67e2"	-0.0793
"00075b70ee"	"00075d73fc"	-0.1409
"00075b70ee"	"00075d7729"	-0.238
"00075b70ee"	"00075d778c"	-0.4167
"00075b70ee"	"00075d7b9e"	-0.3347
"00075b70ee"	"00075d7c8f"	-0.1044
"00075b70ee"	"00075d804d"	-0.1254
"00075b70ee"	"00075d819f"	-0.2688
"00075b70ee"	"00075d8601"	-0.1595
"00075b70ee"	"00075d8c6a"	0.1058
"00075b70ee"	"00075dfedc"	0.1271
"00075b70ee"	"00075e05f2"	-0.2118
"00075b70ee"	"00075e0837"	-0.0803
"00075b70ee"	"00075e092e"	-0.2472
"00075b70ee"	"00075e0965"	0.06
"00075b70ee"	"00075e0bc8"	0.0259
"00075b70ee"	"00075e0fbb"	-0.2268
"00075b7157"	"00075b7225"	0.2718
"00075b7157"	"00075b7c89"	-0.0149
"00075b7157"	"00075b9048"	0.1697
"00075b7157"	"00075d0801"	0.217
"00075b7157"	"00075d1820"	0.1757
"00075b7157"	"00075d1f3d"	-0.0479
"00075b7157"	"00075d2329"	0.1284
"00075b7157"	"00075d2b9b"	-0.2271
"00075b7157"	"00075d3941"	-0.1732
"00075b7157"	"00075d3e96"	0.1763
"00075b7157"	"00075d4864"	0.0656
"00075b7157"	"00075d5961"	0.0384
"00075b7157"	"00075d5a63"	0.0921
"00075b7157"	"00075d6150"	0.006
"00075b7157"	"00075d67d0"	-0.1717
"00075b7157"	"00075d67e2"	-0.0184

"00075b7157"	"00075d73fc"	0.0163
"00075b7157"	"00075d7729"	0.1157
"00075b7157"	"00075d778c"	0.1887
"00075b7157"	"00075d7b9e"	0.5359
"00075b7157"	"00075d7c8f"	0.1519
"00075b7157"	"00075d804d"	0.1628
"00075b7157"	"00075d819f"	0.085
"00075b7157"	"00075d8601"	0.1674
"00075b7157"	"00075d8c6a"	0.193
"00075b7157"	"00075dfedc"	0.0884
"00075b7157"	"00075e05f2"	−0.0724
"00075b7157"	"00075e0837"	−0.1207
"00075b7157"	"00075e092e"	0.1839
"00075b7157"	"00075e0965"	−0.222
"00075b7157"	"00075e0bc8"	−0.0473
"00075b7157"	"00075e0fbb"	0.1173
"00075b7225"	"00075b7c89"	0.1385
"00075b7225"	"00075b9048"	0.1145
"00075b7225"	"00075d0801"	−0.1232
"00075b7225"	"00075d1820"	0.262
"00075b7225"	"00075d1f3d"	0.0364
"00075b7225"	"00075d2329"	−0.0883
"00075b7225"	"00075d2b9b"	−0.247
"00075b7225"	"00075d3941"	−0.0586
"00075b7225"	"00075d3e96"	0.1291
"00075b7225"	"00075d4864"	0.0879
"00075b7225"	"00075d5961"	0.0347
"00075b7225"	"00075d5a63"	−0.0972
"00075b7225"	"00075d6150"	0.1906
"00075b7225"	"00075d67d0"	−0.4178
"00075b7225"	"00075d67e2"	0.0881
"00075b7225"	"00075d73fc"	0.3551
"00075b7225"	"00075d7729"	−0.189
"00075b7225"	"00075d778c"	−0.0135
"00075b7225"	"00075d7b9e"	0.0861
"00075b7225"	"00075d7c8f"	0.3237
"00075b7225"	"00075d804d"	−0.1459
"00075b7225"	"00075d819f"	−0.0798
"00075b7225"	"00075d8601"	−0.1213
"00075b7225"	"00075d8c6a"	0.1171
"00075b7225"	"00075dfedc"	0.2265
"00075b7225"	"00075e05f2"	0.0187
"00075b7225"	"00075e0837"	−0.1507
"00075b7225"	"00075e092e"	0.1523
"00075b7225"	"00075e0965"	−0.32
"00075b7225"	"00075e0bc8"	0.0751
"00075b7225"	"00075e0fbb"	−0.0527
"00075b7c89"	"00075b9048"	0.2642
"00075b7c89"	"00075d0801"	−0.0277
"00075b7c89"	"00075d1820"	−0.0202

"00075b7c89"	"00075d1f3d"	-0.0572
"00075b7c89"	"00075d2329"	0.1021
"00075b7c89"	"00075d2b9b"	-0.0382
"00075b7c89"	"00075d3941"	0.0811
"00075b7c89"	"00075d3e96"	-0.1078
"00075b7c89"	"00075d4864"	-0.1638
"00075b7c89"	"00075d5961"	0.1844
"00075b7c89"	"00075d5a63"	0.0732
"00075b7c89"	"00075d6150"	-0.2723
"00075b7c89"	"00075d67d0"	-0.3079
"00075b7c89"	"00075d67e2"	0.1594
"00075b7c89"	"00075d73fc"	0.209
"00075b7c89"	"00075d7729"	-0.0845
"00075b7c89"	"00075d778c"	-0.3617
"00075b7c89"	"00075d7b9e"	-0.1208
"00075b7c89"	"00075d7c8f"	0.3774
"00075b7c89"	"00075d804d"	0.0145
"00075b7c89"	"00075d819f"	-0.0456
"00075b7c89"	"00075d8601"	-0.0186
"00075b7c89"	"00075d8c6a"	0.1469
"00075b7c89"	"00075dfedc"	0.3559
"00075b7c89"	"00075e05f2"	-0.1182
"00075b7c89"	"00075e0837"	-0.1379
"00075b7c89"	"00075e092e"	0.011
"00075b7c89"	"00075e0965"	-0.3912
"00075b7c89"	"00075e0bc8"	0.1612
"00075b7c89"	"00075e0fbb"	-0.084
"00075b9048"	"00075d0801"	0.0035
"00075b9048"	"00075d1820"	-0.22
"00075b9048"	"00075d1f3d"	0.3259
"00075b9048"	"00075d2329"	0.0795
"00075b9048"	"00075d2b9b"	0.2928
"00075b9048"	"00075d3941"	-0.1458
"00075b9048"	"00075d3e96"	-0.0098
"00075b9048"	"00075d4864"	-0.3292
"00075b9048"	"00075d5961"	0.0948
"00075b9048"	"00075d5a63"	0.1617
"00075b9048"	"00075d6150"	0.0586
"00075b9048"	"00075d67d0"	0.0108
"00075b9048"	"00075d67e2"	0.0993
"00075b9048"	"00075d73fc"	0.157
"00075b9048"	"00075d7729"	0.1172
"00075b9048"	"00075d778c"	-0.3494
"00075b9048"	"00075d7b9e"	-0.0116
"00075b9048"	"00075d7c8f"	0.1144
"00075b9048"	"00075d804d"	0.2597
"00075b9048"	"00075d819f"	0.2272
"00075b9048"	"00075d8601"	-0.1063
"00075b9048"	"00075d8c6a"	0.1307
"00075b9048"	"00075dfedc"	0.116

"00075b9048"	"00075e05f2"	0.2526
"00075b9048"	"00075e0837"	0.1568
"00075b9048"	"00075e092e"	0.046
"00075b9048"	"00075e0965"	0.0396
"00075b9048"	"00075e0bc8"	0.3386
"00075b9048"	"00075e0fbb"	-0.1736
"00075d0801"	"00075d1820"	-0.1483
"00075d0801"	"00075d1f3d"	-0.0364
"00075d0801"	"00075d2329"	0.1447
"00075d0801"	"00075d2b9b"	0.057
"00075d0801"	"00075d3941"	-0.1613
"00075d0801"	"00075d3e96"	0.0268
"00075d0801"	"00075d4864"	0.1979
"00075d0801"	"00075d5961"	-0.0505
"00075d0801"	"00075d5a63"	0.1386
"00075d0801"	"00075d6150"	-0.2089
"00075d0801"	"00075d67d0"	-0.0158
"00075d0801"	"00075d67e2"	-0.2178
"00075d0801"	"00075d73fc"	-0.1145
"00075d0801"	"00075d7729"	-0.2439
"00075d0801"	"00075d778c"	-0.1025
"00075d0801"	"00075d7b9e"	0.1038
"00075d0801"	"00075d7c8f"	0.0024
"00075d0801"	"00075d804d"	0.0475
"00075d0801"	"00075d819f"	0.1398
"00075d0801"	"00075d8601"	0.0173
"00075d0801"	"00075d8c6a"	0.1721
"00075d0801"	"00075dfedc"	0.0579
"00075d0801"	"00075e05f2"	0.0101
"00075d0801"	"00075e0837"	0.126
"00075d0801"	"00075e092e"	-0.0654
"00075d0801"	"00075e0965"	-0.0477
"00075d0801"	"00075e0bc8"	0.1696
"00075d0801"	"00075e0fbb"	-0.1318
"00075d1820"	"00075d1f3d"	-0.3871
"00075d1820"	"00075d2329"	-9e-04
"00075d1820"	"00075d2b9b"	-0.4927
"00075d1820"	"00075d3941"	-0.1505
"00075d1820"	"00075d3e96"	0.3738
"00075d1820"	"00075d4864"	-0.213
"00075d1820"	"00075d5961"	-0.0661
"00075d1820"	"00075d5a63"	-0.1037
"00075d1820"	"00075d6150"	-0.1237
"00075d1820"	"00075d67d0"	-0.3663
"00075d1820"	"00075d67e2"	-0.1707
"00075d1820"	"00075d73fc"	0.1737
"00075d1820"	"00075d7729"	-0.1141
"00075d1820"	"00075d778c"	0.0214
"00075d1820"	"00075d7b9e"	0.1104
"00075d1820"	"00075d7c8f"	-0.0197

"00075d1820"	"00075d804d"	-0.1324
"00075d1820"	"00075d819f"	-0.2894
"00075d1820"	"00075d8601"	0.229
"00075d1820"	"00075d8c6a"	-0.0794
"00075d1820"	"00075dfedc"	0.0937
"00075d1820"	"00075e05f2"	-0.2935
"00075d1820"	"00075e0837"	-0.1904
"00075d1820"	"00075e092e"	-0.0853
"00075d1820"	"00075e0965"	-0.4864
"00075d1820"	"00075e0bc8"	-0.1418
"00075d1820"	"00075e0fbb"	-0.2481
"00075d1f3d"	"00075d2329"	-0.1142
"00075d1f3d"	"00075d2b9b"	0.4242
"00075d1f3d"	"00075d3941"	-0.0497
"00075d1f3d"	"00075d3e96"	0.0147
"00075d1f3d"	"00075d4864"	-0.237
"00075d1f3d"	"00075d5961"	-0.1348
"00075d1f3d"	"00075d5a63"	0.2834
"00075d1f3d"	"00075d6150"	-0.0899
"00075d1f3d"	"00075d67d0"	0.0496
"00075d1f3d"	"00075d67e2"	0.0235
"00075d1f3d"	"00075d73fc"	-0.0826
"00075d1f3d"	"00075d7729"	-0.0934
"00075d1f3d"	"00075d778c"	-0.4361
"00075d1f3d"	"00075d7b9e"	-0.1741
"00075d1f3d"	"00075d7c8f"	0.2023
"00075d1f3d"	"00075d804d"	0.2303
"00075d1f3d"	"00075d819f"	0.3255
"00075d1f3d"	"00075d8601"	-0.2025
"00075d1f3d"	"00075d8c6a"	-0.1788
"00075d1f3d"	"00075dfedc"	-3e-04
"00075d1f3d"	"00075e05f2"	0.0432
"00075d1f3d"	"00075e0837"	0.4275
"00075d1f3d"	"00075e092e"	0.0348
"00075d1f3d"	"00075e0965"	0.0517
"00075d1f3d"	"00075e0bc8"	0.4359
"00075d1f3d"	"00075e0fbb"	-0.0314
"00075d2329"	"00075d2b9b"	-0.0159
"00075d2329"	"00075d3941"	0.1238
"00075d2329"	"00075d3e96"	-0.0315
"00075d2329"	"00075d4864"	0.2589
"00075d2329"	"00075d5961"	0.1616
"00075d2329"	"00075d5a63"	0.3337
"00075d2329"	"00075d6150"	-0.1347
"00075d2329"	"00075d67d0"	-0.1138
"00075d2329"	"00075d67e2"	-0.2138
"00075d2329"	"00075d73fc"	0.1091
"00075d2329"	"00075d7729"	-0.1383
"00075d2329"	"00075d778c"	-0.1911
"00075d2329"	"00075d7b9e"	-0.1194

"00075d2329"	"00075d7c8f"	0.0514
"00075d2329"	"00075d804d"	0.2185
"00075d2329"	"00075d819f"	−0.0022
"00075d2329"	"00075d8601"	0.1367
"00075d2329"	"00075d8c6a"	0.038
"00075d2329"	"00075dfedc"	0.1068
"00075d2329"	"00075e05f2"	−0.1111
"00075d2329"	"00075e0837"	0.2187
"00075d2329"	"00075e092e"	0.0631
"00075d2329"	"00075e0965"	−0.2066
"00075d2329"	"00075e0bc8"	0.2111
"00075d2329"	"00075e0fbb"	0.1419
"00075d2b9b"	"00075d3941"	−0.1359
"00075d2b9b"	"00075d3e96"	−0.2952
"00075d2b9b"	"00075d4864"	−0.1326
"00075d2b9b"	"00075d5961"	−0.2434
"00075d2b9b"	"00075d5a63"	0.4458
"00075d2b9b"	"00075d6150"	−0.3068
"00075d2b9b"	"00075d67d0"	0.0782
"00075d2b9b"	"00075d67e2"	−0.0972
"00075d2b9b"	"00075d73fc"	−0.3538
"00075d2b9b"	"00075d7729"	−0.1862
"00075d2b9b"	"00075d778c"	−0.3198
"00075d2b9b"	"00075d7b9e"	−0.2312
"00075d2b9b"	"00075d7c8f"	−0.0583
"00075d2b9b"	"00075d804d"	0.1193
"00075d2b9b"	"00075d819f"	0.251
"00075d2b9b"	"00075d8601"	−0.2728
"00075d2b9b"	"00075d8c6a"	−0.1909
"00075d2b9b"	"00075dfedc"	0.0259
"00075d2b9b"	"00075e05f2"	0.1826
"00075d2b9b"	"00075e0837"	0.3244
"00075d2b9b"	"00075e092e"	−0.3029
"00075d2b9b"	"00075e0965"	−0.0322
"00075d2b9b"	"00075e0bc8"	0.189
"00075d2b9b"	"00075e0fbb"	−0.1575
"00075d3941"	"00075d3e96"	0.3004
"00075d3941"	"00075d4864"	−0.1977
"00075d3941"	"00075d5961"	−0.1288
"00075d3941"	"00075d5a63"	0.0899
"00075d3941"	"00075d6150"	0.0527
"00075d3941"	"00075d67d0"	−0.5163
"00075d3941"	"00075d67e2"	−0.1805
"00075d3941"	"00075d73fc"	0.1647
"00075d3941"	"00075d7729"	−0.2605
"00075d3941"	"00075d778c"	−0.5092
"00075d3941"	"00075d7b9e"	−0.0274
"00075d3941"	"00075d7c8f"	−0.1781
"00075d3941"	"00075d804d"	−0.0135
"00075d3941"	"00075d819f"	−0.0412

"00075d3941"	"00075d8601"	0.285
"00075d3941"	"00075d8c6a"	-0.1112
"00075d3941"	"00075dfedc"	0.1716
"00075d3941"	"00075e05f2"	-0.1574
"00075d3941"	"00075e0837"	0.0566
"00075d3941"	"00075e092e"	-0.3096
"00075d3941"	"00075e0965"	-0.4066
"00075d3941"	"00075e0bc8"	0.0293
"00075d3941"	"00075e0fbb"	-0.5139
"00075d3e96"	"00075d4864"	-0.124
"00075d3e96"	"00075d5961"	-0.0741
"00075d3e96"	"00075d5a63"	-0.0628
"00075d3e96"	"00075d6150"	0.2628
"00075d3e96"	"00075d67d0"	-0.3028
"00075d3e96"	"00075d67e2"	-0.3357
"00075d3e96"	"00075d73fc"	0.1476
"00075d3e96"	"00075d7729"	-0.0785
"00075d3e96"	"00075d778c"	-0.0996
"00075d3e96"	"00075d7b9e"	0.1315
"00075d3e96"	"00075d7c8f"	-0.1084
"00075d3e96"	"00075d804d"	-0.0476
"00075d3e96"	"00075d819f"	-0.0658
"00075d3e96"	"00075d8601"	0.2173
"00075d3e96"	"00075d8c6a"	-0.0351
"00075d3e96"	"00075dfedc"	0.048
"00075d3e96"	"00075e05f2"	-0.1033
"00075d3e96"	"00075e0837"	0.1244
"00075d3e96"	"00075e092e"	-0.031
"00075d3e96"	"00075e0965"	-0.0571
"00075d3e96"	"00075e0bc8"	-0.0165
"00075d3e96"	"00075e0fbb"	-0.1912
"00075d4864"	"00075d5961"	0.0053
"00075d4864"	"00075d5a63"	0.0997
"00075d4864"	"00075d6150"	-0.0265
"00075d4864"	"00075d67d0"	-0.4354
"00075d4864"	"00075d67e2"	-0.1253
"00075d4864"	"00075d73fc"	-0.0393
"00075d4864"	"00075d7729"	-0.3111
"00075d4864"	"00075d778c"	-0.0645
"00075d4864"	"00075d7b9e"	0.0148
"00075d4864"	"00075d7c8f"	-0.1843
"00075d4864"	"00075d804d"	-0.1321
"00075d4864"	"00075d819f"	-0.2264
"00075d4864"	"00075d8601"	-0.0757
"00075d4864"	"00075d8c6a"	-0.151
"00075d4864"	"00075dfedc"	-0.1602
"00075d4864"	"00075e05f2"	-0.1008
"00075d4864"	"00075e0837"	-0.142
"00075d4864"	"00075e092e"	-0.022
"00075d4864"	"00075e0965"	0.0676

"00075d4864"	"00075e0bc8"	−0.0463
"00075d4864"	"00075e0fbb"	0.1108
"00075d5961"	"00075d5a63"	−0.1257
"00075d5961"	"00075d6150"	0.0963
"00075d5961"	"00075d67d0"	−0.3572
"00075d5961"	"00075d67e2"	0.011
"00075d5961"	"00075d73fc"	0.1055
"00075d5961"	"00075d7729"	−0.0767
"00075d5961"	"00075d778c"	−0.2088
"00075d5961"	"00075d7b9e"	0.07
"00075d5961"	"00075d7c8f"	0.1937
"00075d5961"	"00075d804d"	0.0138
"00075d5961"	"00075d819f"	−0.1735
"00075d5961"	"00075d8601"	0.1879
"00075d5961"	"00075d8c6a"	0.1528
"00075d5961"	"00075dfedc"	0.1568
"00075d5961"	"00075e05f2"	−0.1747
"00075d5961"	"00075e0837"	−0.1394
"00075d5961"	"00075e092e"	0.0356
"00075d5961"	"00075e0965"	−0.0746
"00075d5961"	"00075e0bc8"	0.1819
"00075d5961"	"00075e0fbb"	0.1076
"00075d5a63"	"00075d6150"	−0.2453
"00075d5a63"	"00075d67d0"	0.1669
"00075d5a63"	"00075d67e2"	0.0996
"00075d5a63"	"00075d73fc"	−0.0141
"00075d5a63"	"00075d7729"	0.1165
"00075d5a63"	"00075d778c"	0.1021
"00075d5a63"	"00075d7b9e"	0.1478
"00075d5a63"	"00075d7c8f"	0.1532
"00075d5a63"	"00075d804d"	0.3752
"00075d5a63"	"00075d819f"	0.0945
"00075d5a63"	"00075d8601"	0.0159
"00075d5a63"	"00075d8c6a"	−0.0932
"00075d5a63"	"00075dfedc"	0.2273
"00075d5a63"	"00075e05f2"	0.022
"00075d5a63"	"00075e0837"	0.1954
"00075d5a63"	"00075e092e"	−0.0169
"00075d5a63"	"00075e0965"	0.1012
"00075d5a63"	"00075e0bc8"	0.3657
"00075d5a63"	"00075e0fbb"	0.0109
"00075d6150"	"00075d67d0"	−0.3456
"00075d6150"	"00075d67e2"	−0.3316
"00075d6150"	"00075d73fc"	−0.0044
"00075d6150"	"00075d7729"	−0.1028
"00075d6150"	"00075d778c"	−0.2087
"00075d6150"	"00075d7b9e"	0.1254
"00075d6150"	"00075d7c8f"	−0.2388
"00075d6150"	"00075d804d"	−0.1562
"00075d6150"	"00075d819f"	−0.0801

"00075d6150"	"00075d8601"	0.2161
"00075d6150"	"00075d8c6a"	-0.1235
"00075d6150"	"00075dfedc"	0.1981
"00075d6150"	"00075e05f2"	-0.1463
"00075d6150"	"00075e0837"	-0.166
"00075d6150"	"00075e092e"	-0.1355
"00075d6150"	"00075e0965"	-0.4538
"00075d6150"	"00075e0bc8"	-0.2375
"00075d6150"	"00075e0fbb"	-0.5054
"00075d67d0"	"00075d67e2"	-0.0959
"00075d67d0"	"00075d73fc"	-0.4516
"00075d67d0"	"00075d7729"	-0.1211
"00075d67d0"	"00075d778c"	0.0071
"00075d67d0"	"00075d7b9e"	0.0561
"00075d67d0"	"00075d7c8f"	0.0768
"00075d67d0"	"00075d804d"	0.0919
"00075d67d0"	"00075d819f"	0.1225
"00075d67d0"	"00075d8601"	-0.3038
"00075d67d0"	"00075d8c6a"	-0.3875
"00075d67d0"	"00075dfedc"	-0.1999
"00075d67d0"	"00075e05f2"	-0.033
"00075d67d0"	"00075e0837"	0.1248
"00075d67d0"	"00075e092e"	-0.1218
"00075d67d0"	"00075e0965"	-0.6428
"00075d67d0"	"00075e0bc8"	-0.039
"00075d67d0"	"00075e0fbb"	-0.2232
"00075d67e2"	"00075d73fc"	0.1124
"00075d67e2"	"00075d7729"	-0.2031
"00075d67e2"	"00075d778c"	-0.2067
"00075d67e2"	"00075d7b9e"	-0.0071
"00075d67e2"	"00075d7c8f"	0.3348
"00075d67e2"	"00075d804d"	0.193
"00075d67e2"	"00075d819f"	-0.1057
"00075d67e2"	"00075d8601"	-0.0569
"00075d67e2"	"00075d8c6a"	0.0368
"00075d67e2"	"00075dfedc"	0.0814
"00075d67e2"	"00075e05f2"	0.2168
"00075d67e2"	"00075e0837"	-0.2581
"00075d67e2"	"00075e092e"	-0.0577
"00075d67e2"	"00075e0965"	-0.3214
"00075d67e2"	"00075e0bc8"	0.0059
"00075d67e2"	"00075e0fbb"	-0.1909
"00075d73fc"	"00075d7729"	-0.1126
"00075d73fc"	"00075d778c"	-0.3368
"00075d73fc"	"00075d7b9e"	-0.1394
"00075d73fc"	"00075d7c8f"	0.1743
"00075d73fc"	"00075d804d"	-0.2602
"00075d73fc"	"00075d819f"	-0.0432
"00075d73fc"	"00075d8601"	0.1242
"00075d73fc"	"00075d8c6a"	0.1239

"00075d73fc"	"00075dfedc"	0.188
"00075d73fc"	"00075e05f2"	-0.05
"00075d73fc"	"00075e0837"	-0.0497
"00075d73fc"	"00075e092e"	0.0123
"00075d73fc"	"00075e0965"	-0.3217
"00075d73fc"	"00075e0bc8"	0.1259
"00075d73fc"	"00075e0fbb"	-0.2205
"00075d7729"	"00075d778c"	-0.0146
"00075d7729"	"00075d7b9e"	0.138
"00075d7729"	"00075d7c8f"	-0.0891
"00075d7729"	"00075d804d"	-0.06
"00075d7729"	"00075d819f"	-0.3184
"00075d7729"	"00075d8601"	0.025
"00075d7729"	"00075d8c6a"	0.0309
"00075d7729"	"00075dfedc"	-0.0579
"00075d7729"	"00075e05f2"	-0.55
"00075d7729"	"00075e0837"	-0.2089
"00075d7729"	"00075e092e"	-0.1981
"00075d7729"	"00075e0965"	-0.2844
"00075d7729"	"00075e0bc8"	0.0916
"00075d7729"	"00075e0fbb"	-0.2202
"00075d778c"	"00075d7b9e"	0.52
"00075d778c"	"00075d7c8f"	-0.0443
"00075d778c"	"00075d804d"	-0.168
"00075d778c"	"00075d819f"	-0.4058
"00075d778c"	"00075d8601"	-0.27
"00075d778c"	"00075d8c6a"	-0.0739
"00075d778c"	"00075dfedc"	-0.0269
"00075d778c"	"00075e05f2"	-0.1391
"00075d778c"	"00075e0837"	-0.2014
"00075d778c"	"00075e092e"	0.0762
"00075d778c"	"00075e0965"	-0.4655
"00075d778c"	"00075e0bc8"	-0.268
"00075d778c"	"00075e0fbb"	9e-04
"00075d7b9e"	"00075d7c8f"	0.0772
"00075d7b9e"	"00075d804d"	0.1291
"00075d7b9e"	"00075d819f"	0.1043
"00075d7b9e"	"00075d8601"	0.1848
"00075d7b9e"	"00075d8c6a"	-0.0783
"00075d7b9e"	"00075dfedc"	0.1552
"00075d7b9e"	"00075e05f2"	-0.051
"00075d7b9e"	"00075e0837"	-0.0523
"00075d7b9e"	"00075e092e"	0.176
"00075d7b9e"	"00075e0965"	-0.36
"00075d7b9e"	"00075e0bc8"	-0.0558
"00075d7b9e"	"00075e0fbb"	-0.0209
"00075d7c8f"	"00075d804d"	0.2065
"00075d7c8f"	"00075d819f"	0.1564
"00075d7c8f"	"00075d8601"	-0.0479
"00075d7c8f"	"00075d8c6a"	0.1889

"00075d7c8f"	"00075dfedc"	0.4321
"00075d7c8f"	"00075e05f2"	-0.0529
"00075d7c8f"	"00075e0837"	0.0371
"00075d7c8f"	"00075e092e"	0.2198
"00075d7c8f"	"00075e0965"	-0.315
"00075d7c8f"	"00075e0bc8"	0.1221
"00075d7c8f"	"00075e0fbb"	0.2661
"00075d804d"	"00075d819f"	0.2346
"00075d804d"	"00075d8601"	0.1394
"00075d804d"	"00075d8c6a"	-0.0871
"00075d804d"	"00075dfedc"	0.0555
"00075d804d"	"00075e05f2"	0.1016
"00075d804d"	"00075e0837"	0.266
"00075d804d"	"00075e092e"	0.0491
"00075d804d"	"00075e0965"	-0.1022
"00075d804d"	"00075e0bc8"	0.3835
"00075d804d"	"00075e0fbb"	-0.0792
"00075d819f"	"00075d8601"	-0.1504
"00075d819f"	"00075d8c6a"	-0.3456
"00075d819f"	"00075dfedc"	-0.1364
"00075d819f"	"00075e05f2"	0.0466
"00075d819f"	"00075e0837"	0.4937
"00075d819f"	"00075e092e"	0.1423
"00075d819f"	"00075e0965"	-0.4123
"00075d819f"	"00075e0bc8"	0.2232
"00075d819f"	"00075e0fbb"	-0.0685
"00075d8601"	"00075d8c6a"	0.3061
"00075d8601"	"00075dfedc"	0.2717
"00075d8601"	"00075e05f2"	-0.2386
"00075d8601"	"00075e0837"	-0.0735
"00075d8601"	"00075e092e"	-0.366
"00075d8601"	"00075e0965"	-0.3336
"00075d8601"	"00075e0bc8"	-0.083
"00075d8601"	"00075e0fbb"	-0.518
"00075d8c6a"	"00075dfedc"	0.2321
"00075d8c6a"	"00075e05f2"	-0.1139
"00075d8c6a"	"00075e0837"	-0.2573
"00075d8c6a"	"00075e092e"	-0.1742
"00075d8c6a"	"00075e0965"	0.0211
"00075d8c6a"	"00075e0bc8"	-0.0181
"00075d8c6a"	"00075e0fbb"	-0.048
"00075dfedc"	"00075e05f2"	0.103
"00075dfedc"	"00075e0837"	0.0211
"00075dfedc"	"00075e092e"	-0.3098
"00075dfedc"	"00075e0965"	-0.2265
"00075dfedc"	"00075e0bc8"	0.1048
"00075dfedc"	"00075e0fbb"	-0.238
"00075e05f2"	"00075e0837"	0.2671
"00075e05f2"	"00075e092e"	-0.0359
"00075e05f2"	"00075e0965"	-0.1924

"00075e05f2"	"00075e0bc8"	0.1773
"00075e05f2"	"00075e0fbb"	-0.2499
"00075e0837"	"00075e092e"	0.0642
"00075e0837"	"00075e0965"	-0.1969
"00075e0837"	"00075e0bc8"	0.4237
"00075e0837"	"00075e0fbb"	0.0456
"00075e092e"	"00075e0965"	-0.3644
"00075e092e"	"00075e0bc8"	0.0902
"00075e092e"	"00075e0fbb"	0.6077
"00075e0965"	"00075e0bc8"	0.131
"00075e0965"	"00075e0fbb"	-0.1687
"00075e0bc8"	"00075e0fbb"	0.0109
